# Harnessing Multistep Chalcogen Bonding Activation in the α-Stereoselective Synthesis of Iminoglycosides

**DOI:** 10.1021/jacs.4c00262

**Published:** 2024-04-02

**Authors:** Caiming Wang, Anna Krupp, Carsten Strohmann, Bastian Grabe, Charles C. J. Loh

**Affiliations:** † Abteilung Chemische Biologie, Max Planck Institut für Molekulare Physiologie, Otto-Hahn-Straße 11, 44227 Dortmund, Germany; ‡ Fakultät für Chemie und Chemische Biologie, Technische Universität Dortmund, Otto-Hahn-Straße 4a, 44227 Dortmund, Germany; § Anorganische Chemie, Technische Universität Dortmund, Otto-Hahn-Straße 6, 44227 Dortmund, Germany

**Keywords:** carbohydrates, catalysis, group 16 compounds, noncovalent interactions, selectivity

## Abstract

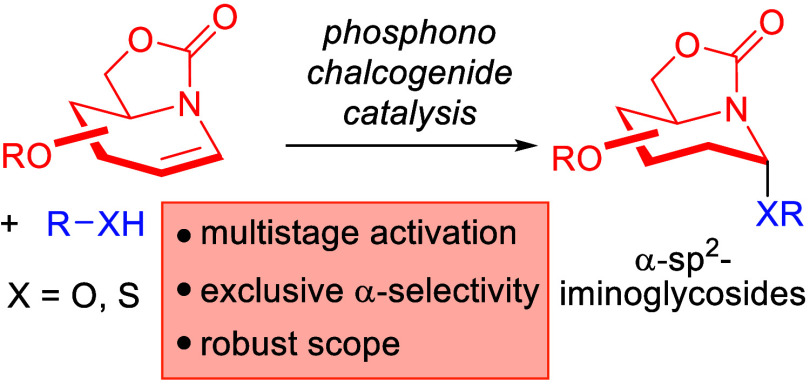

The use of noncovalent interactions (NCIs) has received significant attention as a pivotal synthetic handle. Recently, the exploitation of unconventional NCIs has gained considerable traction in challenging reaction manifolds such as glycosylation due to their capacity to facilitate entry into difficult-to-access sugars and glycomimetics. While investigations involving oxacyclic pyrano- or furanoside scaffolds are relatively common, methods that allow the selective synthesis of biologically important iminosugars are comparatively rare. Here, we report the capacity of a phosphonochalcogenide (PCH) to catalyze the stereoselective α-iminoglycosylation of iminoglycals with a wide array of glycosyl acceptors with remarkable protecting group tolerance. Mechanistic studies have illuminated the counterintuitive role of the catalyst in serially activating both the glycosyl donor and acceptor in the up/downstream stages of the reaction through chalcogen bonding (ChB). The dynamic interaction of chalcogens with substrates opens up new mechanistic opportunities based on iterative ChB catalyst engagement and disengagement in multiple elementary steps.

## 1 Introduction

The important role of noncovalent interactions (NCIs) in synthesis is blossoming as a fundamental catalytic concept.^1^ Exploiting NCIs often facilitates unprecedented entry into difficult-to-access molecular structures with high stereoselectivity. Particularly within the challenging field of stereoselective carbohydrate synthesis,^2^ NCIs have recently garnered significant momentum^3^ due to their multifaceted role in accessing previously elusive glycosidic chemical spaces.

The use of understudied unconventional NCIs that operate through sigma-hole activation^4^ has entered the foray of stereoselective glycosylation in recent years due to their unique nonprotic manifold and higher directionality. It is also important as a matter of accurate definition that besides the contribution from electrostatics wherein the sigma hole terminology is derived, charge transfer, orbital delocalization, dispersion and polarization components also contribute to these NCIs.^4^ These characteristics often enable glycosylation to proceed under milder but still more robust conditions. Halogen bonding (XB) catalysis, in particular,^4a^ has achieved considerable success for various glycosylation manifolds. After an initial proof-of-concept work by Codée and Huber using an XB promoter in Königs–Knorr glycosylation,^5^ Takemoto’s group demonstrated the use of an XB cocatalyst in *N*-glycofunctionalization.^6^ Our research group has also contributed to the exclusive XB-catalyzed stereoselective strain-release glycosylation^7^ and 2-deoxyglycosylation of glycals.^8^ Lately, Niu and co-workers also demonstrated the elegant use of XB assistance in conjunction with radical activation to access challenging 1,2-*cis*-glycosides in a highly stereoselective fashion.^9^

**Figure 1:**
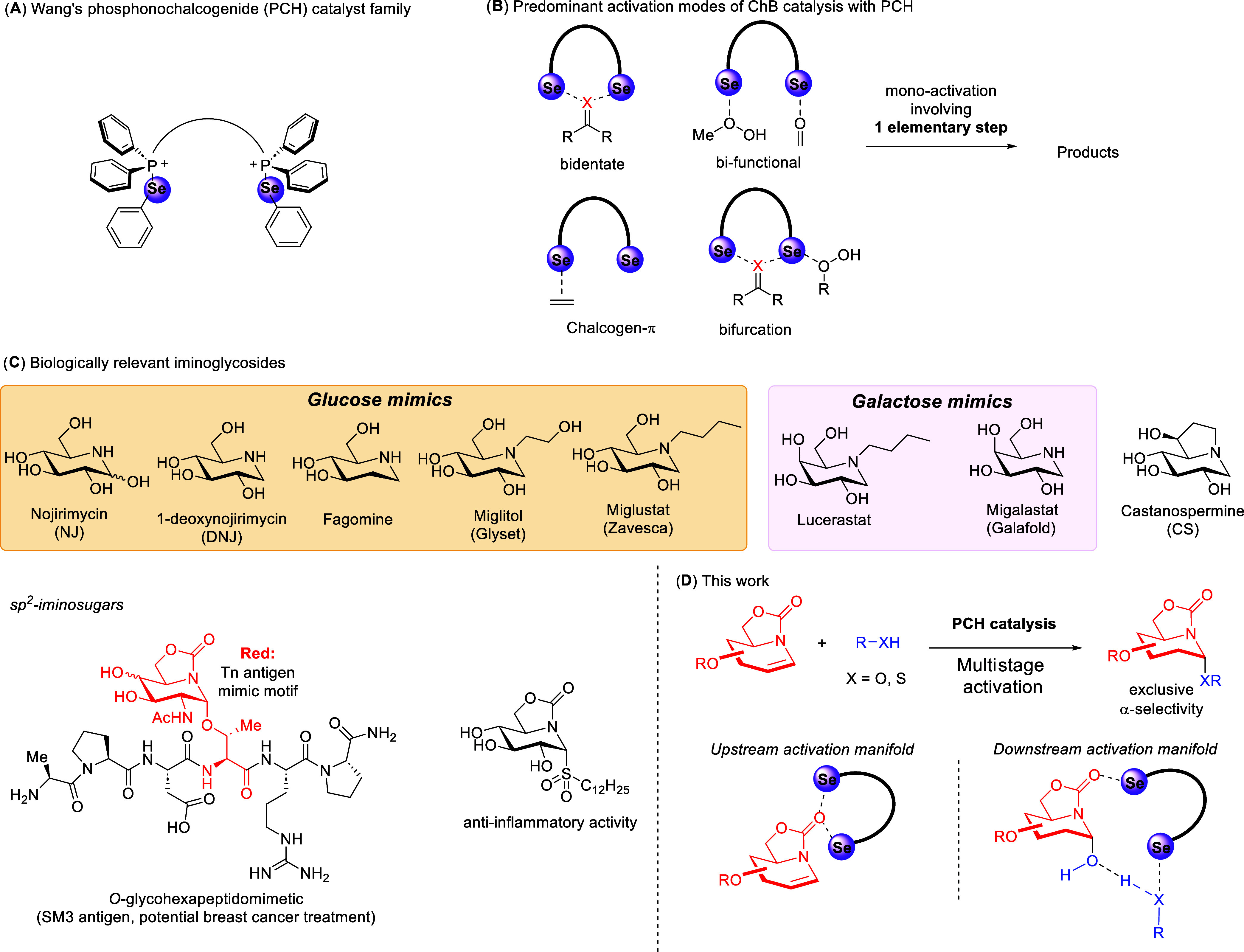
Chalcogen bonding, phosphonochalcogenide catalysis, and development of an α-selective iminoglycosylation. (A) The phosphonochalcogenide catalyst family. (B) Predominant activation modes of ChB catalysis. (C) Biologically relevant iminoglycosides. (D) This work.

In light of these advances, other classes of sigma-hole based NCIs are comparatively underexploited. A good example is chalcogen bonding (ChB) catalysis, which has made substantial strides in recent years,^4b,4d^ in particular through the use of modular phosphonochalcogenides (PCH) pioneered by Wang and co-workers (Figure 1A).^10^ Using PCH catalysts, the core scaffold electronics could be fine-tuned using easily commercially available bis-phosphine precursors. While a wide variety of activation modes ranging from bidentate,^11^ bifunctional,^12^ chalcogen-π,^13^ bifurcation,^14^ and even synergistic ChB, π–π, and CH-π^15^ were reported by Wang, Huber, and our group, all of the currently known ChB-catalyzed reactions are predominantly based on a single activation event within a single elementary step (Figure 1B). Hence, the potential to harness the temporal catalyst–substrate engaging/disengaging nature of NCIs in the ChB activation manifold in numerous activation events within a multielementary step manifold could offer untapped opportunities for synthesis. To the best of our knowledge, this enzymatic-like phenomenon remains unknown in ChB catalysis.

Related instances were only known in prior work using XB-based manifolds such as our work in XB-catalyzed glycosylation,^7,8^ and also proposed based on computational studies in molecular iodine-catalyzed strategies.^17^

By virtue of our enriching journey in uncovering unusual sigma-hole based catalytic manifolds in carbohydrate chemistry, particularly demonstrated in our recent reports on the stereoselective access of 7-membered ring sugars,^14^ and β-indolyl glycosides,^15^ we are curious if further exploration of new mechanistic avenues using ChB catalysis in carbohydrate synthesis could address difficult carbohydrate classes that are considerably understudied.

We were thus specially drawn toward iminoglycosides or aza-sugars, as this is a glycomimetic containing a well-recognized privileged piperidine core scaffold^18^ that is relevant in a wide range of diseases (Figure 1C).^19^ This includes lysosomal storage disorders (Gaucher disease),^20^ influenza infections,^21^ cystic fibrosis,^22^ and more recently, in coronavirus SARS-CoV-2 replication.^23^ The iminoglycoside scaffold is also present in natural products such as in nojirimycin (NJ) and deoxynojirimycin,^24^ which is a β-glucosidase inhibitor. The naturally occurring 2-deoxy derivative, fagomine, was further reported to possess antihyperglycemic effects.^25^ The iminoglycoside scaffold is also known to be of interest in drug development. Particularly, iminosugar frameworks based on the glucose mimics such as Miglitol (Glyset) and Miglustat (Zavesca) had been approved for diabetic treatment and therapy in Gaucher and Niemann-Pick C diseases;^24^ the galactose mimics such as Lucerastat and Migalastat (Galafold) had also found useful in chaperone-based therapy against Fabry disease.^24^ Of particular recent interest is the emergence of α-sp^2^-iminosugars developed by Ortiz Mellet and co-workers as a useful glycomimetic, which possesses a broad biological activity profile. This includes potential anticancer and anti-inflammatory activities, toll-like receptor agonist activity, and use as a pharmacological chaperone for late-onset Tay-Sachs disease.^24,26^ Prior studies demonstrated that the sp^2^-iminoglycosyl donors are also chemical mimics that imitate the glycosyl profile of normal monosaccharide donors.^27^

Despite the wide-ranging uses of iminosugars, the synthetic development of stereoselective iminoglycosylations is very limited. Catalytic iminoglycosylation is undoubtedly scarce compared to conventional pyrano- and furanosylations. This is particularly conspicuous in the overwhelming examples of *O*-2-deoxyglycosylations^28^ compared to iminoglycal congeners. Of important synthetic interest is the documented difficulties in activating iminoglycal donors from prior literature.^29^ High loadings of common Lewis acids were reported to be ineffective, and the sole use of strong Brønsted acids such as phosphoric acid, well established to be versatile in many reaction classes under low loadings,^30^ was also reported to be insufficient. As a consequence, high Brønsted acid catalyst loading also required amplification by a cocatalyst before iminoglycal activation can occur. Under elevated acidic conditions, side reactions can occur that influence substrate tolerance. For example, migration of the isopropylidene protecting group was noted and unsatisfactory yields were observed when sterically more hindered secondary alcohols on saccharide substrates were employed under such conditions. Considering the well-known lability of the 2-deoxy glycosidic linkage to acid hydrolysis^8,31^ and the above-mentioned side reactions, strongly protic conditions are less desirable in glycosylation procedures, particularly if traces of water are not tediously eliminated from the reaction vessel.

Herein, we report an α-selective chalcogen bond-catalyzed iminoglycosylation of iminoglycals over a wide range of glycosyl donors and acceptors (Figure 1D). Our method also features remarkable protecting group tolerance of the glycosyl donor, which spans from arming to disarming protecting groups. Further, glycosyl donors containing both glucosyl- and galactosyl-mimetic scaffolds, which are well represented in biologically relevant molecules, can be well assimilated in our method. This protocol can also be conducted under mild and ambient conditions without the vigorous exclusion of moisture, which is often required in other glycosylation procedures. Mechanistic studies offered evidence that the employed PCH catalyst uniquely engages substrates in up- and downstream elementary steps of a stepwise mechanism by activating both the iminoglycal and glycosyl acceptor at different stages of the reaction. This suggests a good basis that PCH catalysis is endowed with favorable NCI-based catalytic characteristics that are contemporaneously compatible with multiple functional groups commonly found in glycosyl substrates.

## 2 Results and Discussion

### 2.1 Establishment of α-Selective Iminoglycosylation

We began our preliminary investigation by studying a panel of commonly utilized noncovalent catalysts in a model reaction between 2,3-disiloxoiminoglycal **1a** and *n*-octanol **2a** as a representative glycosyl acceptor (Table 1). While the known versatile halogen bonding-based catalysts **A**(32) and **B**(33) have been previously successfully used by us in catalyzing glycosylations,^7,8^ we were somewhat surprised that they were ineffective in activating the more challenging iminoglycals. Attempting Schreiner’s hydrogen bonding thiourea catalyst **C**(34) was also ineffective, an observation that was also echoed in previous studies.^29^ Next, we studied the newer generations of ChB-based phosphonochalcogenide (PCH) catalysts.^10^ To our delight, PCH catalysts resulted in an obvious improvement in the catalytic robustness. When 1,2-*bis*-(diphenylphosphino)ethane (dppe)-derived PCH catalyst **D** was employed, we noted that a very good yield of 88% of the iminoglycoside **3a** was obtained with exclusive α-selectivity. A tetrachlorogalate congener of dppe scaffold **E** also performed similarly. Fine-tuning of the central diphosphino core is possible. When Xantphos-derived PCH derivatives **F–H** were screened, we generally observed consistently excellent selectivity with marginal fluctuations in the reaction yield. Generally, we observed that switching the counteranion from triflate to sterically encumbered and noncoordinating ones such as tetrachlorogallate or tetrakis(3,5-*bis*(trifluoromethyl)phenyl)borate (BAr^F^) retained the effectiveness of the PCH catalysts. Similar reactivity profiles toward variations of counteranions suggest that triflate interference is unlikely to influence the PCH-based catalytic manifold here. By deepening the chalcogen’s sigma holes through the use of derivatives **I-J** recently developed by Wang,^13b^ we were able to elevate the efficiency of the reaction and arrive at the optimized conditions using catalyst **J** at 2 mol % catalyst loadings with exclusive α-selectivity.

**Table 1:**
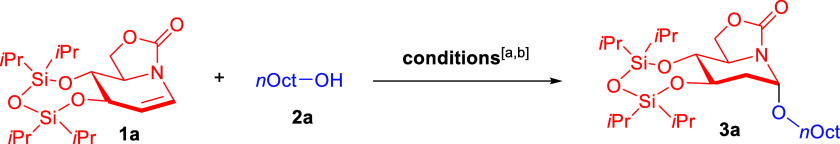

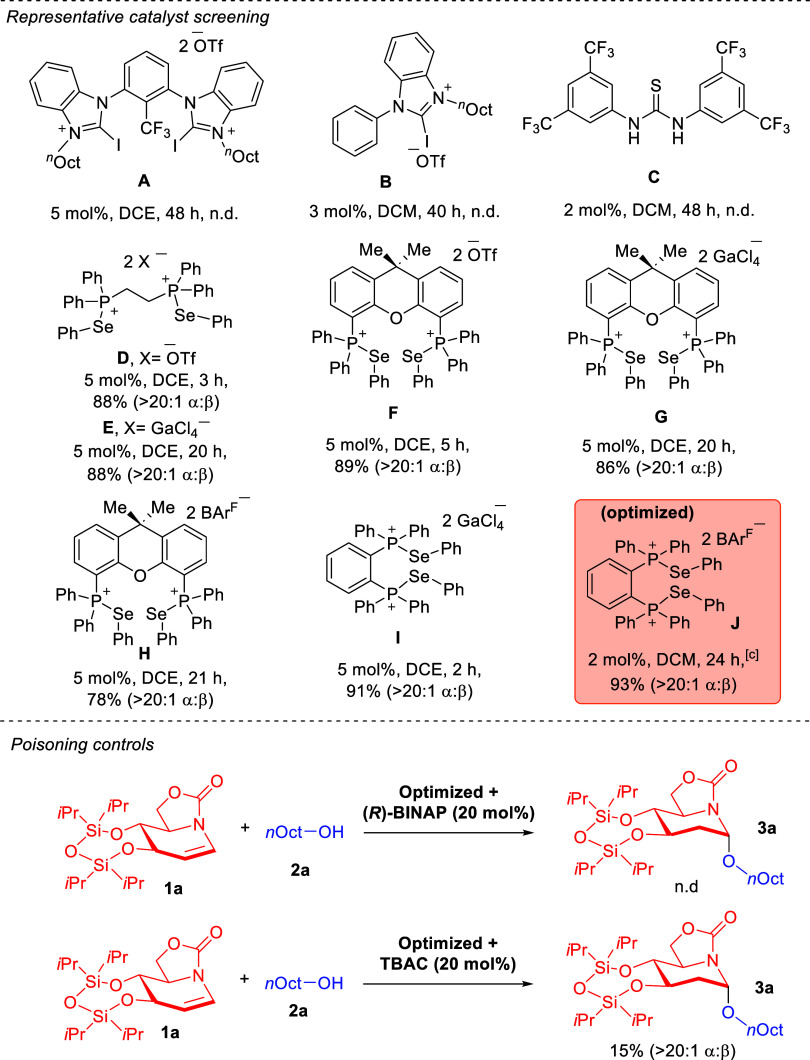
Selected Optimization of Iminoglycosylation and Diagnostic ChB Poisoning Controls

a,b Conditions: [a] **1a** (0.05 mmol), **2a** (0.075 mmol), catalyst, 50 °C, solvent (0.2 mL), time, argon. [b] The yield and α:β ratio of **3a** were determined by crude ^1^H NMR spectral analysis using 1,3,5-trimethoxybenzene as an internal standard. [c] Conducted at rt. n.d.: not detected.

We also performed the necessary verification controls by using literature known ChB catalytic poisons^11a,35^ that possess very high binding affinity with chalcogens.^36^ First, by adding 20 mol % (*R*)-BINAP as a phosphine poison at optimized conditions, we were able to terminate the catalysis, and no product **3a** was detected (Table 1). Second, the addition of halide poison using 20 mol % tetrabutyl ammonium chloride (TBAC) under optimized conditions had a substantial inhibitory effect on ChB catalysis and resulted in a 15% NMR yield of **3a**. These diagnostic poisoning experiments affirm that the catalytic mode of action involves sigma-hole based manifolds.

### 2.2 Determining the Substrate Scope

Upon arriving at the optimized conditions, we expanded the synthetic use of our strategy by evaluating the substrate scope (Tables 2 and 3). In general, this methodology is amenable to a robust range of glucosyl (Table 2) and galactosyl (Table 3) iminoglycal scaffolds and a sizable variety of *O*- and *S*-glycosyl acceptors with varying stereochemical environments. Exclusive α-selectivity was also consistent throughout the substrate scope.

**Table 2:**
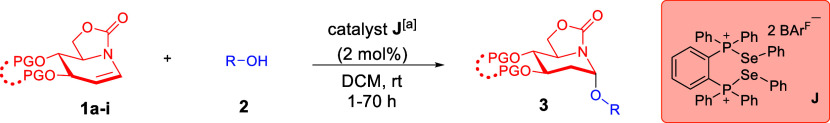

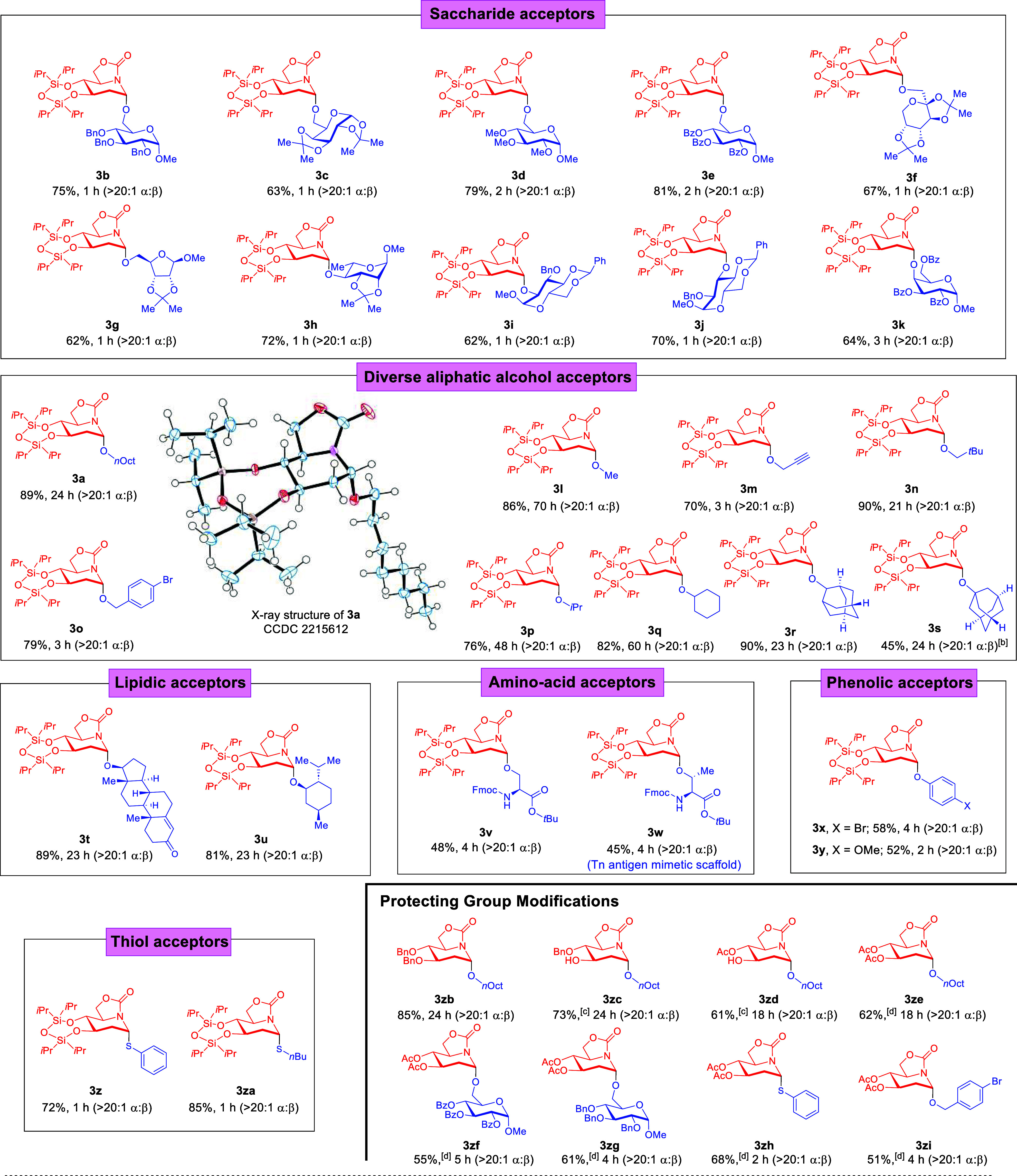
Substrate Scope of Glucosyl Iminoglycal Donors

a Conditions: [a] **1a**–**i** (0.2 mmol), **2** (0.3 mmol), rt, CH_2_Cl_2_ (0.8 mL), time, argon. Isolated yields after silica gel chromatography and α/β ratios are shown in parentheses. The α:β ratios were determined by crude ^1^H NMR spectral analysis. [b] 40 °C. [c] The TBS-protecting group at the 3-OH position was cleaved under catalytic conditions. [d] Catalyst **D** (2 mol %) was used instead, 40 °C. Me = methyl, Et = ethyl, *i*Pr = isopropyl, TBS = *tert*-butyldimethylsilyl, Fmoc = fluorenylmethyloxycarbonyl, and rt = room temperature. For the X-ray structure, thermal ellipsoids are shown at 50% probability, and the monomeric component of the tetrameric unit cell for **3a** is displayed.

**Table 3:**
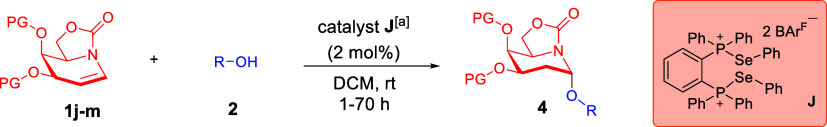

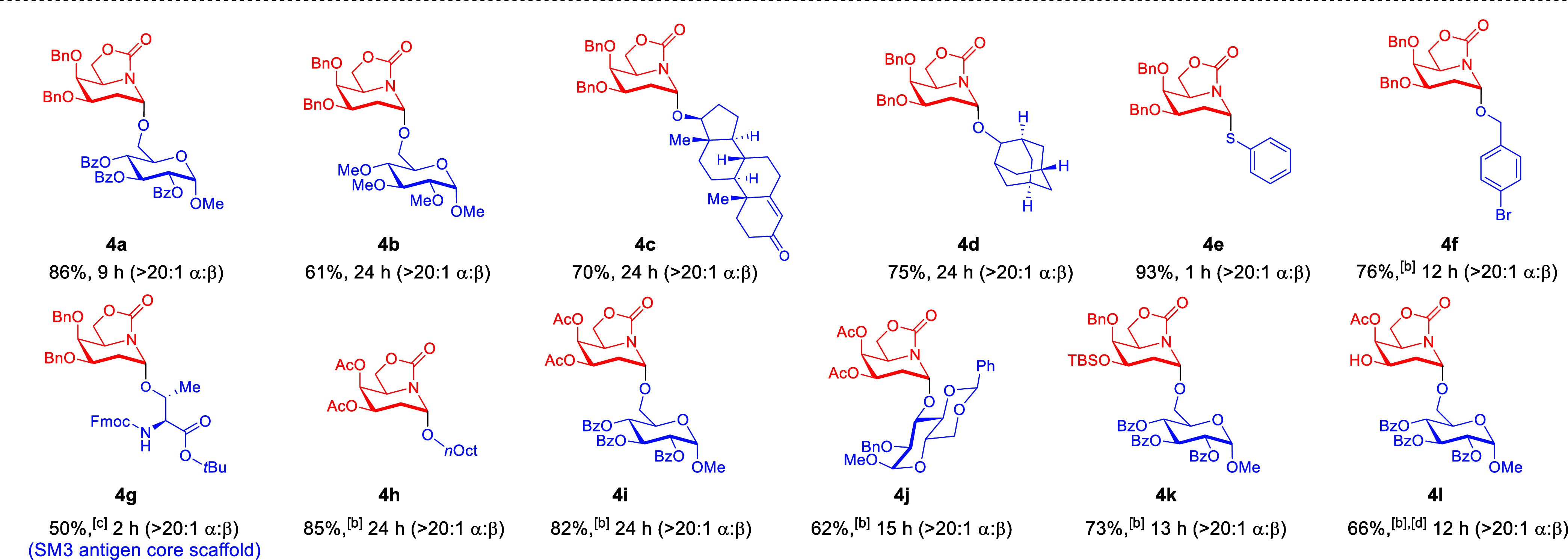
Substrate Scope of Galactosyl Iminoglycal Donors

a Conditions: [a] **1j**–**m** (0.2 mmol), **2** (0.3 mmol), rt, CH_2_Cl_2_ (0.8 mL), time, argon. Isolated yields after silica gel chromatography and α:β ratios are shown in parentheses, respectively. The α:β ratios were determined by crude ^1^H NMR spectral analysis. [b] 40 °C instead. [c] **1j** (0.1 mmol), **2** (0.15 mmol), CH_2_Cl_2_ (0.4 mL) instead. [d] The TBS-protecting group at the 3-OH position was cleaved under catalytic conditions. Me = methyl, Et = ethyl, *i*Pr = isopropyl, TBS = *tert*-butyldimethylsilyl, Fmoc = fluorenylmethyloxycarbonyl, and rt = room temperature.

When we investigated the applicability of saccharide acceptors in our 2-deoxyiminoglycosylation, we noted that diverse architectures of naturally occurring sugars with various protecting groups were easily assimilated using this strategy. These included d-glucopyranoses (**3b**, **3d**, **3e**, **3i**, and **3j)**, d-galactopyranoses (**3c**, **3k**), d-fructopyranoses (**3f**), d-ribofuranoses (**3g**), and l-rhamnopyranose (**3h**). Further, a broad selection of primary and secondary hydroxyl groups situated at different positions of the pyrano/furanoside rings was also accommodated within our protocol.

Next, we proceeded to evaluate easily available aliphatic alcohols as potential nucleophiles. To our delight, our strategy also features steric robustness as a broad selection of primary alcohols (**3a**–**3o**) ranging from straight chain, propargyl to benzylic alcohols; secondary alcohols (**3p**–**3r**) encompassing isopropanol to sterically larger 2-adamantanol; and sterically challenging tertiary alcohol acceptors (**3s**) such as 1-adamantanol can even be smoothly utilized. Additionally, biologically relevant lipidic chiral alcohols, such as testosterone (**3t**) and menthol (**3u**), can also be employed as glycosyl acceptors. As appending amino-acid acceptors would facilitate entry into 2-deoxyiminoglycoside derivatives of the biologically relevant Tn antigen mimetic scaffold,^26a^ we were delighted that our method tolerated *N*-terminal Fmoc and *C*-terminal Boc (*tert*-butyloxycarbonyl) protected l-serine (**3v**) and l-threonine (**3w**) derivatives. This is compatible with orthogonal protecting group schemes commonly employed in solid-phase peptide synthesis. Further, phenolic (**3x**–**3y**) acceptors and even thiol-based acceptors (**3z-3za**) can also be accommodated in our method without any depletion of anomeric selectivity.

Considering the importance of protecting group tolerance in chemical glycosylations, we proceeded to study the flexibility of protecting group permutations in our iminoglycosylation. “Arming” protecting groups^37^ such as benzyl (**3zb**) and TBS groups (**3zc-3zd**) were well accommodated in our strategy. Importantly, more challenging iminoglucal substrates bearing electron-withdrawing “disarming” protecting groups^37^ such as acetyl can also be employed using our ChB-catalyzed strategy as fully protected schemes in **3ze-3zi**, or in hybrid schemes such as **3zd** in conjunction with a TBS group with good yields and no detrimental influences on the exclusive α-selectivity. The acetyl-protected iminoglucal donor was also observed to tolerate a good selection of glycosyl acceptors ranging from saccharides, simple alcohols, and benzyl alcohol to thiol-based ones.

As both the glucosyl and galactosyl versions of the iminosugar were well represented in biologically relevant molecules (vide supra, Figure 1c), we further evaluated the substrate scope of iminogalactal donors **1j**–**m** (Table 3).

Gratifyingly, the ChB-catalyzed method is also amenable to accessing the galactosyl iminosugars with the preservation of robustness. A broad spectrum of nucleophiles, including saccharide-based acceptors (**4a**, **4b**, **4i**, **4j**, **4k**, **4l**), lipidic acceptors (**4c**), sterically hindered 2-adamantanol (**4d**), thiol-based acceptor (**4e**), benzyl alcohol acceptor (**4f**), and linear aliphatic alcohol (**4h**) were employed to generate the target iminogalactosides with consistent exclusive α-selectivity and good to excellent yields. Importantly, protected l-threonine could also be smoothly utilized to gain entry into the 2-deoxy derivative of the SM3 antigen core scaffold **4g**.^26a^ A brief survey of protecting groups was also fruitful,^37^ as arming groups such as benzyl and TBS, disarming acetyl groups, and even donors containing hybrid protecting schemes were all tolerated within our ChB-catalyzed method. It is also important to emphasize that our method did not involve Schlenk or rigorous water-exclusion techniques throughout the scope, which improves its overall practicability in synthetic handling.

### 2.3 Mechanistic Studies

**Figure 2:**
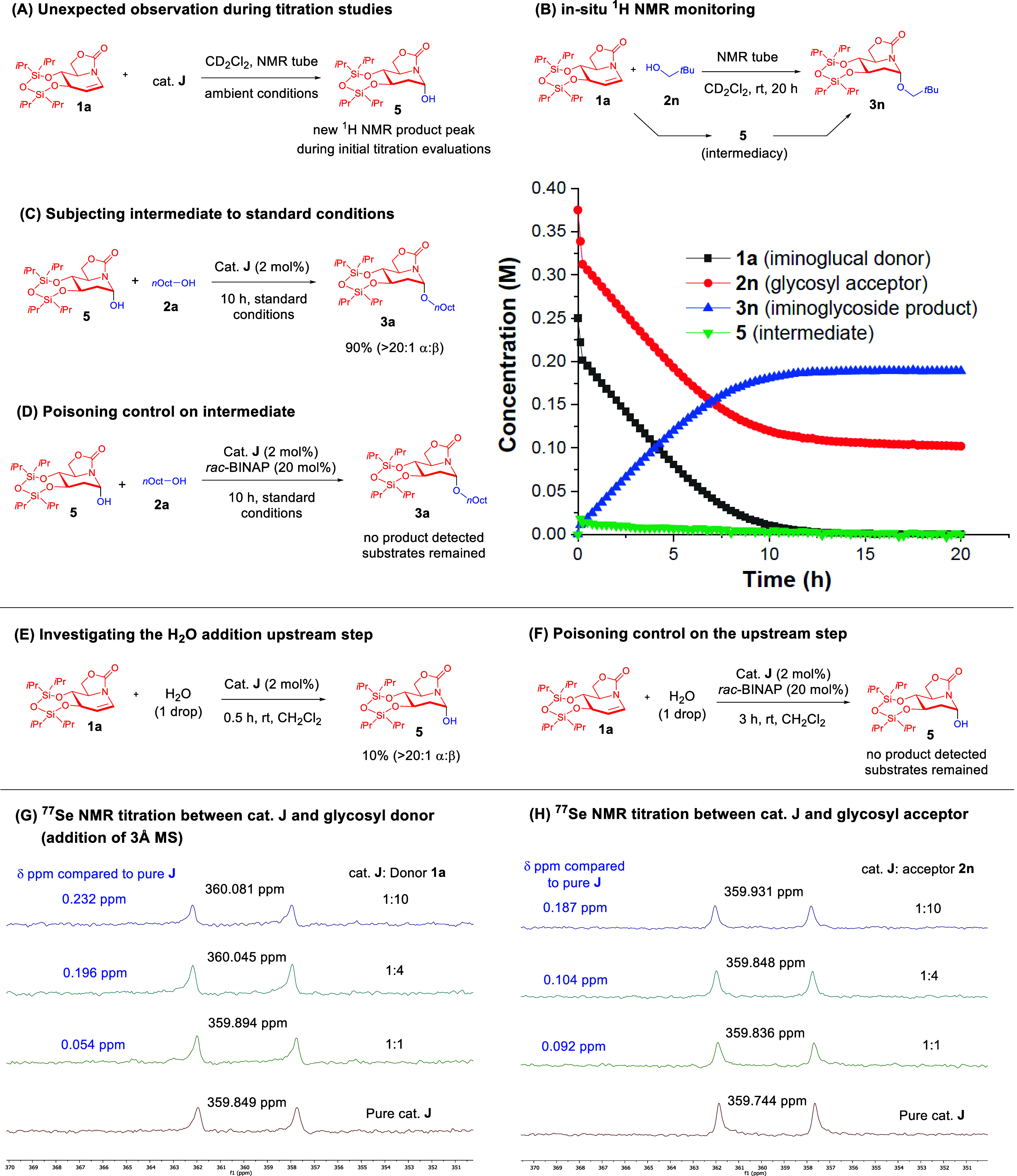
Mechanistic study. (A) Unexpected observation of a water addition product. (B) In situ ^1^H NMR kinetic profile of the reaction. (C) Control experiment, in which intermediate **5** is subjected to the reaction conditions. (D) Ascertaining ChB activation under standard conditions with intermediate **5** by employing a phosphine poison. (E) Investigation of water addition in the upstream step. (F) Phosphine poisoning control in the upstream step. (G) Anhydrous ^77^Se NMR titration between cat. **J** and donor **1a**. (H) ^77^Se NMR titration between cat. **J** and glycosyl acceptor **2n**.

In our early attempts at NMR titrations of the PCH catalyst against iminoglucal donor **1a** to understand participating NCIs that could contribute to catalysis, we surprisingly observed that a new product peak appeared in the NMR tube (Figure 2A, see SI Supporting Figure S2). By meticulously isolating and fully characterizing the unexpected product, the molecule could be unambiguously assigned to water addition product **5** onto the glycal. This water source was likely due to the presence of trace water under ambient conditions, where water was not deliberately excluded from the reaction flask. This was somewhat unexpected, as most reported 2-deoxyglycosylations were proposed to mechanistically proceed through the direct addition of alcohols across the C1–C2 olefin,^28^ often through an initial C2 protonation.

We followed this lead by conducting an in situ ^1^H NMR monitoring experiment (Figure 2B) to ascertain if **5** could be a relevant intermediate within the reaction’s mechanistic manifold at standard conditions. We managed to detect **5** as a gradually diminishing species from the first NMR detection data point, which is consistent with the temporal kinetic profile of intermediate **5** participating in the mechanism.

To further investigate if **5** could be a relevant intermediate en route to the iminoglycoside product, we subjected purified **5** to standard catalytic conditions using catalyst **J** in the presence of *n*-octanol as a glycosyl acceptor (Figure 2C, see SI Supporting Figure S8). To our delight, iminoglycosylation proceeded effectively to yield the target α-iminoglycoside **3a** with excellent yield and anomeric selectivity. This further confirmed that our reaction mechanism likely involved the intermediacy of **5** in two stepwise elementary steps. A clearer insight was separately obtained through a sequential ^1^H NMR monitoring experiment (see SI Section 7.2.2 Supporting Figures S6 and S7), whereby we first mixed donor **1a** and catalyst **J** without the addition of the glycosyl acceptor. After monitoring for 0.5 h in the first reaction phase, we sequentially added acceptor **2n** into the NMR tube and continued monitoring until the end of the reaction. We observed in the first phase a mass conserved formation of **5** and an equivalent depletion of **1a**, which then levels out, likely due to the reversibility and attainment of dynamic equilibrium of this step; after adding **2n** at the 0.5 h time point, we note then the gradual depletion of **5** and the subsequent appearance of the product, which plateaued at around 7 h. This sequential experiment further confirmed that **5** was an intermediate in the stepwise formation of the iminoglycoside product.

To determine the catalytic influence during the conversion of intermediate **5** to **3a**, additional controls were essential. First, we set up a negative control where no catalyst was added at the standard reaction conditions (see SI Supporting Figure S9), and noted that the substrate remained unreacted and no product **3a** was generated. Second, we performed a phosphine poisoning experiment^11,35^ at standard conditions by using *rac*-BINAP as an additive (Figure 2D and see SI Supporting Figure S10) to understand if the downstream elementary step involves sigma-hole based activation. Similarly, this poisoning control terminated the reaction, and the substrate remained unreacted.

As the establishment of the intermediacy of **5** supported the postulate of an upstream reaction of H_2_O with glycal, we then proceeded to study if this initial elementary step is ChB-catalyzed. First, by applying our standard conditions in the presence of one drop of water but at the same time in the absence of the glycosyl acceptor (Figure 2E), we observed that 10% of **5** could be obtained within 0.5 h. Second, when we added *rac*-BINAP (20 mol %) as a ChB poison under analogous conditions as in the former case (Figure 2F, see SI Supporting Figure S13), we observed that no traces of **5** were formed, and the substrates remained unreacted. This indicated that the upstream water addition step is catalyzed by **J** through a sigma-hole based activation process.

Taking into account that the NMR titration between catalyst **J** and the iminoglycal required a nonreacting system for an accurate supramolecular study, we conducted a series of NMR titrations in the presence of activated powdered 3 Å molecular sieves in the NMR tube to suppress the water addition step (Figure 2G, see Supporting Figure S14). First, we noted that the presence of molecular sieves suppressed the formation of intermediate **5** in the NMR tube during NMR titration between cat. **J** and donor **1a**, and a downfield shift in the ^77^Se NMR resonance of approximately 0.232 ppm was observed when the donor concentration was increased. Importantly, parallel ^13^C NMR measurements (see Supporting Figures S16) of these titration points reflect downfield shifts of the carbonyl carbon (∼0.155 ppm downfield compared to pure **1a**). These shifts support the postulate that the selenium’s sigma holes plausibly engage in the activation of the carbamate carbonyl oxygen. Thus, the participation of bidentate ChB is likely operative.

**Figure 3:**
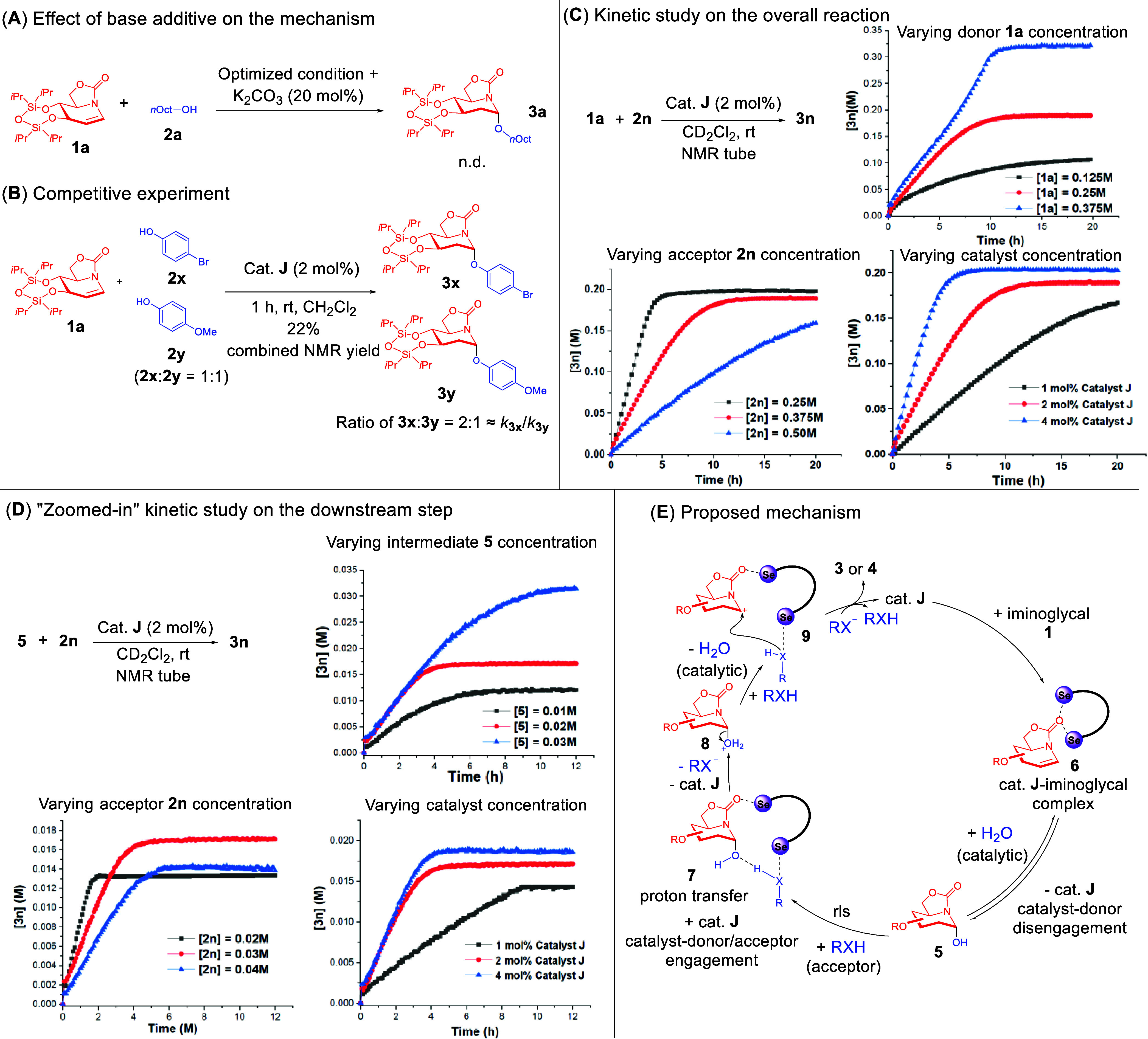
Kinetic study and the proposed mechanism. (A) Addition of a base additive terminates the reaction. n.d.: not detected. (B) Competitive experiments indicated the crucial influence of glycosyl acceptor’s p*K*_a_ on the reaction rate. (C) ^1^H NMR kinetic study of the overall iminoglycosylation. (D) ^1^H NMR “zoomed-in” kinetic study using isolated intermediate **5** as the glycosyl donor to understand the downstream elementary step. (E) Proposed hypothesis for the multistage ChB-catalyzed mechanism.

Next, we conducted ^77^Se NMR titration between catalyst **J** and a representative glycosyl acceptor **2n** (Figure 2H, see Supporting Figure S19). We observed that there was also a downfield shift of the ^77^Se resonance (0.187 ppm downfield compared to pure **J**) as the acceptor concentration increased, which supports the postulate that ChB activation between the catalyst and the glycosyl acceptor is operative within the catalytic manifold. Concurrent measurement of this titration set using ^1^H NMR also revealed a downfield shift of the hydroxyl proton as the catalyst concentration increased (with reference to the hydroxyl resonance of pure **2n**, see SI Supporting Figure S20). These observations are in line with a selenium-hydroxyl activation in the presence of the catalyst.^14,15^ Subsequently, ^77^Se and ^13^C NMR titrations between catalyst **J** and intermediate **5** (see SI Supporting Figures S21 and S23) revealed a ^77^Se NMR downfield shift (0.028 ppm downfield with reference to pure **J**) when the concentration of **5** was increased (see SI Supporting Figure S21), and a concomitant downfield shift of the carbamate carbonyl ^13^C resonance (1.70 ppm downfield with reference to pure **5**, see SI Supporting Figure S23). This suggests that catalyst **J** may engage both intermediate **5** and the acceptor’s hydroxyl oxygen through bifunctional activation in the downstream mechanistic step. As our control experiments, binary titration data, and the identification of the intermediacy of **5** suggested a stepwise mechanism where ChB activation imparted by catalyst **J** is likely involved in both elementary steps; therefore, we conducted further kinetic studies to establish the rate-limiting step of this mechanism. Taking into consideration the complete termination of the reaction in an early control experiment when 20 mol% of K_2_CO_3_ was introduced as an additive (Figure 3A), we surmised that proton transfer is essential in our catalytic mechanism elementary steps since the base could disturb the proton-shuttling process.

Subsequently, we designed a competitive experiment where an equal molar of *p*-bromophenol acceptor **2x** (p*K*_a_ ∼ 9.37) and *p*-methoxyphenol acceptor **2y** (p*K*_a_ ∼ 10.4) with distinctive differences in their p*K*_a_ were allowed to react parallelly in the same pot with the same glycosyl donor **1a** (Figure 3B).^38^ Since the ratio of both iminoglycosides (**3x**:**3y**) formed in this experiment can be used to estimate the ratio of the rate constants of both competing reactions, this experiment could illuminate whether the acceptor’s acidity mechanistically influences the rate-limiting step. Intriguingly, we determined that an approximate decrease of 1 in p*K*_a_ led to a 2-fold increase in the reaction rate. An exact replicate of this competitive experiment confirmed this result (Supporting Figure S25). Since the acceptor’s p*K*_a_ is correlated with the OH bond-breaking process, this competitive experiment further reinforces the hypothesis that proton transfer from the glycosyl acceptor is ingrained in the rate-limiting step (rls).

Next, we investigated the kinetic behavior of the overall iminoglycosylation by modifying the concentrations of glycosyl donor **1a**, acceptor **2n**, and catalyst **J** (Figure 3C). When we changed the concentration of the glycosyl donor **1a**, an overall increase in the reaction rate was observed in the kinetic profile, as evidenced by the leftward shift of the kinetic curve. This supports the hypothesis that the reaction has a positive order with respect to **1a**.

Following this, we varied the concentration of the acceptor **2n** and noticed an inverse correlation between the increasing concentration and decreased reaction rates. Such a negative order profile has been observed previously in our laboratories when saccharides with free alcohols were employed,^8,39^ which could be attributed to the formation of hydrogen bonding aggregates.^40^ The establishment of aggregates at higher acceptor concentrations would stabilize such supramolecular clusters and compete for productive ChB–hydroxyl interactions, which culminates in the decrease of the reaction rate. Last but not least, when the concentration of catalyst **J** was varied, we noted a positive order with respect to the catalyst. This is in line with the hypothesis that the catalyst is directly involved in the rls.

Since we are able to isolate intermediate **5**, we decided to perform a similar NMR kinetic study by replacing the glycosyl donor with **5** (Figure 3D). This endeavor would provide us with a “zoomed-in” understanding of the kinetic profile of the hypothesized downstream elementary step and compare this kinetic profile with that of the overall multistep reaction (Figure 3C). Intriguingly, the kinetic behavior of the downstream step correlates closely with that of the overall reaction and generally displayed positive orders with respect to both **5** and the catalyst, and a negative order with respect to the glycosyl acceptor. A similar kinetic profile suggests that the iminoglycosylation of intermediate **5** with the glycosyl acceptor constitutes the rate-limiting step of the reaction. Further, we surmise that the upstream elementary step between iminoglycal **1a** and water is a reversible reaction since our sequential ^1^H NMR monitoring experiment in the absence of a glycosyl acceptor (see the first 0.5 h phase of Supporting Figures S6 and S7) gave a rather low yield (∼10%) of **5** without proceeding to completion.

In light of the suite of NMR titration and kinetic investigations, we propose the following working hypothesis that forms the basis of the multistep ChB activation mechanism (Figure 3E). The reaction commences with the activation of phosphonochalcogenide catalyst **J** on the iminoglycal donor to form donor–catalyst complex **6**. Based on ^77^Se, ^13^C NMR titration, and DFT modeling (vide infra), we postulate that noncovalent activation involves bidentate activation of the carbonyl oxygen in the upstream step.

Next, one molecule of water present as trace in the reaction reacts with **6** reversibly to form water addition intermediate **5**, which we isolated and characterized. It is imperative to emphasize the catalytic nature of water as this molecule of water is re-expelled from the catalytic cycle further downstream, thus obviating the need for deliberate water addition or tedious water-exclusion techniques in the reaction. The observation of complete reaction termination when 3 Å molecular sieves were added at standard conditions further augments the importance of catalytic water in this manifold. This step occurs simultaneously with the disengagement of catalyst **J** from the substrate.

Subsequently, **J** re-engages a molecule of glycosyl acceptor either on the hydroxyl oxygen or on the thiol’s sulfur and weakens the X–H bond in the process. By virtue of our NMR titration between intermediate **5** and the catalyst, we surmise that this step proceeds through a bifunctional mode **7**, whereby one selenium of **J** is anchored onto the carbamate carbonyl oxygen, while the second selenium concurrently activates the acceptor’s hydroxyl group. The acidic hydrogen on the X–H bond is then transferred onto the anomeric hydroxyl oxygen on **5**, with the activation complex of this proton-shuttling elementary step denoted as **7**.

This elementary step is likely the rate-limiting step (rls) of the mechanism, as our competitive experiment (Figure 3B) ascertained that a decrease in p*K*_a_ resulted in rate acceleration, consistent with the protic nature of this elementary step. Additionally, the correlation between the kinetic profiles of the overall reaction (Figure 3C) and the “zoomed-in” kinetic profile from intermediate **5** (Figure 3D), as well as the reversibility between **6** and **5**, suggested that the rls would involve **5**, the glycosyl acceptor, and the catalyst in the elementary step.

The proton transfer then culminates in the formation of **8**, where the presence of a good water-leaving group prepares the glycosyl donor for the final glycosylation step. We propose that catalyst **J** disengages and the oxyanion/thiolate intermediate temporarily exits the catalytic cycle.

Further downstream, upon the departure of catalytic water from **8** to form the iminoglycosyl carbocation in **9**, we postulate that the catalyst would re-establish a bidentate activation mode similar to **7** to activate a new molecule of the glycosyl acceptor. This would subsequently facilitate an O/S nucleophilic attack on the anomeric center. A final proton transfer from the oxygen in the newly formed glycosidic linkage to neutralize the previously exited oxyanion/thiolate species forms α-iminoglycosides **3**–**4** and recycles catalyst **J** back into a fresh new catalytic round.

**Figure 4:**
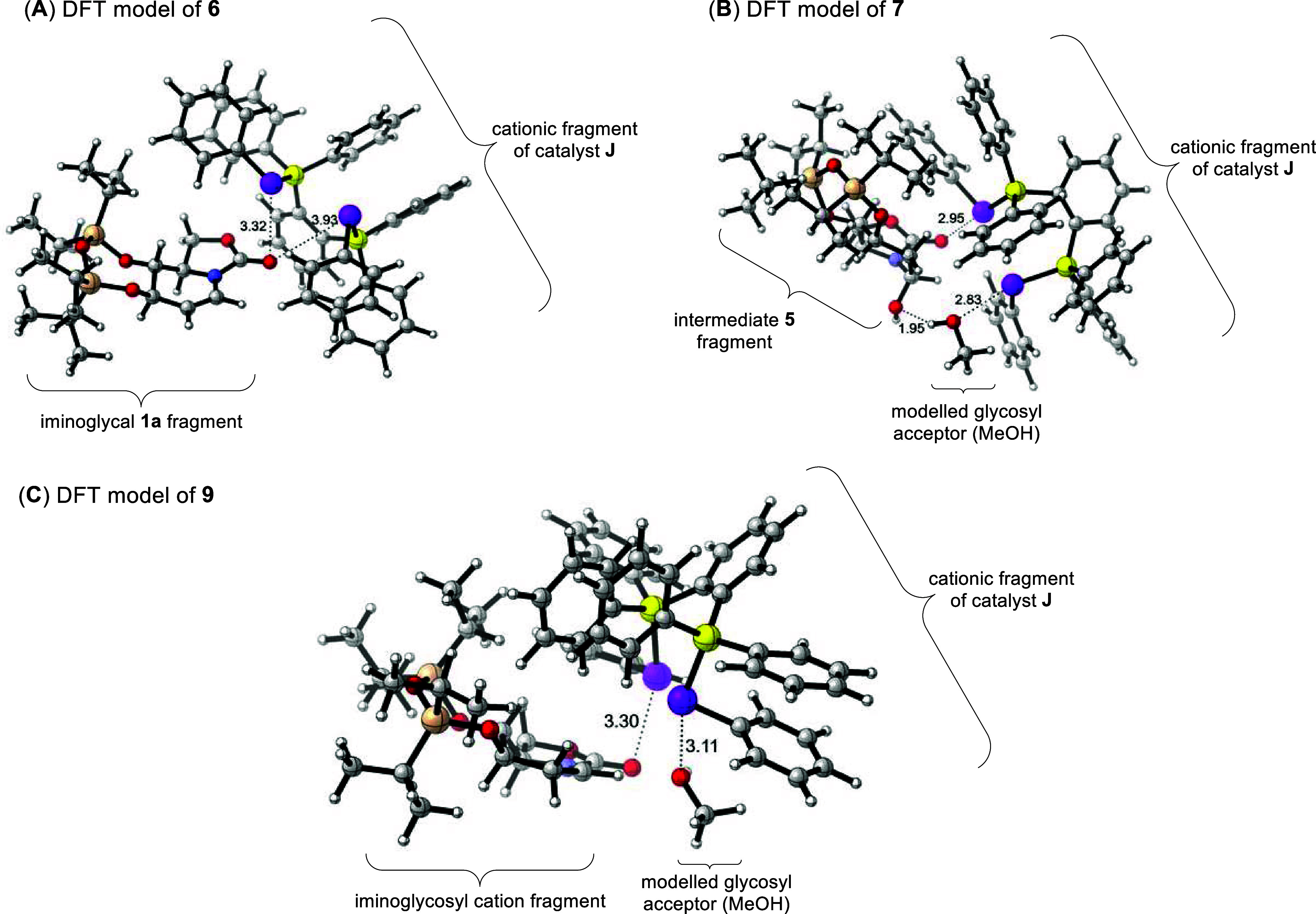
DFT-computed geometries and relevant ChB modes of the participating catalytic species in the postulated mechanism. (A) DFT model of upstream complex **6** in the bidentate carbonyl activation mode. (B) DFT model of downstream complex **7** in the bifunctional ChB mode and a hydrogen bond established between the glycosyl acceptor and intermediate **5**. (C) DFT model of downstream bidentate ChB activation mode **9** (red atoms = oxygen, gray atoms = carbon, yellow atoms = phosphorus, purple atoms = selenium, light orange atoms = silicon, white atoms = hydrogen). The relevant NCIs are displayed using dotted lines, and the relevant distances (in Å) are shown.

To gain deeper insight into the theoretical plausibility of our postulated ChB catalytic modes (Figure 4) at various stages of the proposed mechanism, we modeled the proposed species **6**, **7**, **8**, and **9** using ORCA^41^ at the M06-2X-D3(0)/def2-SVP/CPCM(CH_2_Cl_2_) level of theory,^42^ as the Minnesota functionals are known to be suitable for describing chalcogen bonding interactions.^43^ Upon obtaining the DFT-optimized geometries, we performed NCI analysis^44^ on **6**, **7**, and **9** (putative ChB modes) using the wave function analyzer Multiwfn^45^ to unravel relevant regions of NCIs through colored isosurfaces. Delightfully, our DFT investigations yielded well-converged geometric minima for these postulated species. Further, NCI analysis (see **SI**, Supporting Figures S48–S50) also supports the following: (1) bidentate engagement of the catalyst with the carbamate carbonyl oxygen in **6** (Figure 4A); (2) bifunctional activation mode in **7** where the catalyst seleniums are engaged in two ChBs with both the carbamate oxygen and the oxygen of the glycosyl acceptor (Figure 4B), concurrently with a hydrogen bond between the glycosyl acceptor and intermediate **5**; and (3) analogous bidentate downstream activation mode in **9** on the iminoglycosyl cation (Figure 4C).

## 3 Conclusions

In conclusion, we demonstrate a phosphonochalcogenide-catalyzed α-selective iminoglycosylation that involves multiple ChB activation in the upstream and downstream mechanistic steps. This mechanistic manifold is distinctive in comparison to prior ChB catalytic modes, which are primarily based on monoelementary step activation manifolds. Further, the use of the PCH catalyst obviates the requirement for moisture exclusion and even utilizes trace water catalytically within the mechanistic manifold, thus improving the overall usability of the reaction under mild conditions. Importantly, our demonstration addresses the general scarcity of robust catalytic iminoglycosylations and offers a practical solution to access biologically relevant sp^2^-iminoglycosidic scaffolds. Through detailed NMR titrations and kinetics, we propose a multielementary-step mechanism that involves a proton transfer process as the rate-limiting step. We are optimistic that this strategy showcases the vastly underexploited potential of sigma-hole based activation in expanding the frontiers of stereoselective carbohydrate and glycomimetic syntheses.

## Supporting Information Available

Detailed experimental procedures, spectra data, crystallographic data of **3a** and analytical data (PDF)
