# Supplementary material for: Harnessing Multistep Chalcogen Bonding Activation in the α-Stereoselective Synthesis of Iminoglycosides
Source: J Am Chem Soc. 2024 Apr 2;146(15):10608–20. doi: 10.1021/jacs.4c00262 (PMC11027159; doi:10.1021/jacs.4c00262)
Supplement: Supplementary file 1 — ja4c00262_si_001.pdf [file ja4c00262_si_001.pdf]

Supplementary information

Harnessing multi-step chalcogen bonding activation in the α-stereoselective synthesis of iminoglycosides

Caiming Wang,[†,‡] Anna Krupp,[#] Carsten Strohmann,[#] Bastian Grabe[‡] and Charles C. J. Loh *[,†,‡]

[†]Abteilung Chemische Biologie, Max Planck Institut für Molekulare Physiologie, Otto-Hahn-Straße 11, 44227 Dortmund, Germany.

[‡]Fakultät für Chemie und Chemische Biologie, Technische Universität Dortmund, Otto-Hahn-Straße 4a, 44227 Dortmund, Germany.

[#]Anorganische Chemie, Technische Universität Dortmund, Otto-Hahn-Straße 6, 44227 Dortmund, Germany

Contents

1. General Information 3
2. Preparation of catalysts 3
3. Synthesis of glycosyl iminoglycal donors 8
3.1. Synthesize glucosyl iminoglycal donors 8
3.2. Galactosyl iminoglycal donors synthesis 10
3.3. Data of steps to synthesize iminoglycal donors 12
4. Supplementary methods 26
4.1. Optimization of reaction conditions 26
4.2. General procedure for harnessing multi-step chalcogen bonding activation in the α-stereoselective synthesis of iminoglycosides. 27
5. Characterization data for products and intermediate **5** 27
6. X-Ray crystallography data of **3a** 52
7. Mechanistic study 84
7.1. Unexpected observation of intermediate **5** between donor **1a** and catalyst **J** 84
7.2.1. *In-situ* ^{1}H NMR monitoring experiment in NMR tube 84
7.2.2. *Sequential In-situ* reaction monitoring in NMR tube 88
7.3. Subjecting intermediate to standard conditions 92
7.4. Control experiments on intermediate 93
7.4.1. Without catalyst **J** between intermediate **5** and **2a** 93
7.4.2. Poisoning control on intermediate **5** 94
7.5. Investigating the H_2O addition upstream step 95
7.6. Poisoning control on the upstream step 97
7.7. NMR data for titration experiments 97
7.7.1. NMR titration between catalyst **J** and donor **1a** (with 3Å molecular sieves) 97
7.7.2. NMR titration between catalyst **J** and acceptor **2n** 100
7.7.3. NMR titration between catalyst **J** and intermediate **5** 102
7.8. Effect of base additive (K_2CO_3) on the mechanism 104
7.9. Competitive experiment between different phenols 104
7.10. Kinetic experiment on the overall reaction 106
7.10.1. Donor **1a** concentration dependence 106
7.10.2. Acceptor **2n** concentration dependence 109
7.10.3. Catalyst **J** concentration dependence 111
7.11. Kinetic experiment on the downstream step 114
7.11.1. NMR monitoring of the downstream step under standard conditions 114
7.11.2.1. Intermediate **5** concentration dependence 118
7.11.2.2. Acceptor **2n** concentration dependence 122
7.11.2.3. Catalyst **J** concentration dependence 125
7.12. Kinetic analysis 129

7.12.1. The order of catalyst **J**, donor **1a**, acceptor **2n** of the overall reaction 129
7.12.1.1. Burés method to determine order with respect to catalyst **J**.. 129
7.12.1.2. Initial rate method to determine order with respect to donor **1a** 131
7.12.2 The order of catalyst **J**, intermediate **5**, acceptor **2n** of the downstream step.................... 133
7.12.2.1 Burés method to determine order with respect to catalyst **J**.. 133
7.12.2.2. Initial rate method to determine order with respect to intermediate **5**............................ 136
7.12.2.3. Initial rate method to determine order with respect to acceptor **2n** of the downstream step ………………………………………………………………………………………………..138
7.13. Experiments to test stability of catalyst **J** in the presence of deprotonated alcohol 139
7.14. Computational details... 141
8. References 154
9. NMR spectra 156

1. General Information

Unless otherwise stated, all reactions were set up under inert atmosphere (argon) utilizing glassware that were oven dried and cooled under argon purging. Silica Gel Flash Column Chromatography was performed on *Silica gel Merck 60* (*particle size 40-63 µm.* Starting materials were purchased directly from commercial suppliers (Sigma Aldrich, Acros, Alfa Aesar, VWR, TCI) and used without further purifications unless otherwise stated. All solvents were dried according to standard procedures or brought from commercial suppliers. Reactions were monitored using thin-layer chromatography (TLC) on *Merck silica gel aluminium plates with F254 indicator*. Visualization of the developed plates was performed under UV light (254 nm) or $KMnO_4$ stain.

NMR characterization data (^1H NMR, ^{13}C NMR and 2D spectra) were collected at 300 K on a *Bruker DRX400 (400 MHz)*, *Bruker DRX500 (500 MHz)*, *INOVA500 (500 MHz), DRX600 (600 MHz)* and *Bruker DRX700 (700 MHz)* using $CDCl_3$ and CD_2Cl_2 as solvent. Data for ^1H NMR are reported as follows: chemical shift (δ ppm), multiplicity (s = singlet, d = doublet, t = triplet, q = quartet, m = multiplet, br = broad), coupling constant (Hz), referenced to the solvent resonance as internal standard ($CDCl_3$: δ = 7.26 ppm for ^1H, δ = 77.16 ppm for ^{13}C; CD_2Cl_2: δ = 5.32 ppm for ^1H, δ = 54.00 ppm for ^{13}C; CD_3OD: δ = 3.31 ppm for ^1H, δ = 49.00 ppm for ^{13}C).

High resolution mass spectra were recorded on an *LTQ Orbitrap* mass spectrometer coupled to an *Accela HPLC*-System (HPLC column: *Hypersyl GOLD*, 50 mm x 1 mm, particle size 1.9 µm, ionization method: electron spray ionization) and Bruker ultrafleXtreme MALDI-TOF-TOF (3 decimal accuracy). Optical rotations were measured in a *Schmidt + Haensch Polartronic HH8* polarimeter equipped with a sodium lamp source (589 nm), and are reported as follows: $[\alpha]_D^{T\,°C}$ (*c* = g/100 mL, solvent).

The anomeric selectivity was determined by ^1H-NMR of the crude reaction mixture *via* integration of characteristic signals in the ^1H NMR spectra, Chemical yields refer to isolated substances after flash column chromatography. NMR yields were determined using 1,3,5-trimethoxybenzene or 1,1,2,2-tetrachloroethane as internal standard.

2. Preparation of catalysts

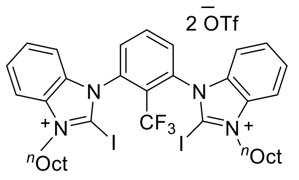

A

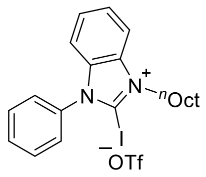

B

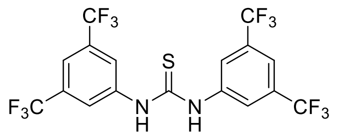

C

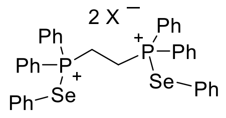

D, X= OTf^-
E, X= $GaCl_4^-$

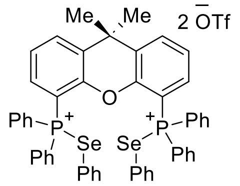

F

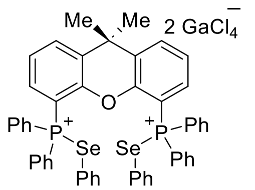

G

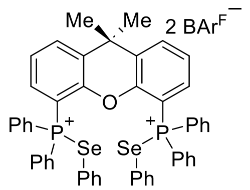

H

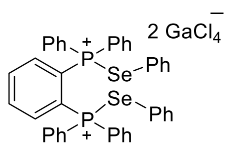

I

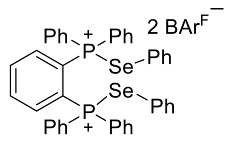

J

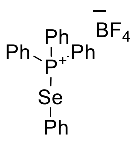

K

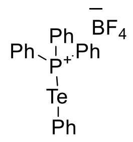

L

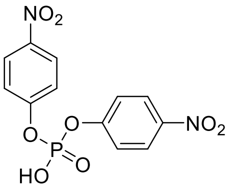

M

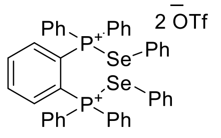

N

Catalysts **A** and **B** were prepared according to the literature procedure.[10] **C** and **M** were Commercial available, **D-J, N** were prepared according to the literature procedure.[11-12] **K** and **L** were prepared using the following procedure:

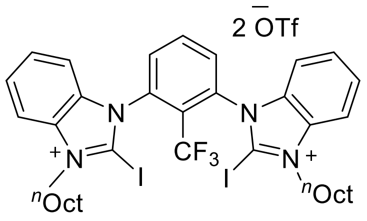

A

^{1}H NMR (600 MHz, CD_2Cl_2) δ 8.33 (t, *J* = 8.4 Hz, 1H), 8.21 – 8.17 (m, 2H), 7.98 – 7.93 (m, 2H), 7.82 – 7.78 (m, 2H), 7.75 – 7.68 (m, 4H), 4.66 – 4.50 (m, 4H), 2.01 (p, *J* = 7.8 Hz, 4H), 1.50 – 1.35 (m, 8H), 1.33 – 1.23 (m, 12H), 0.87 (t, *J* = 7.2 Hz, 6H). **^{13}C NMR** (150 MHz, CD_2Cl_2) δ 138.49, 136.52, 136.05, 135.08, 133.13, 129.34, 128.46, 127.81 (d, *J* = 31.1 Hz), 121.57 (d, *J* = 275.6 Hz), 120.86 (d, *J* = 318.3 Hz), 116.25, 115.19, 113.16, 51.85, 32.17, 29.58, 29.54, 29.50, 27.08, 23.11, 14.35. **^{19}F NMR** (565 MHz, CD_2Cl_2) δ -55.85, -78.76. The analytical data are in accordance with the reported literature.[10]

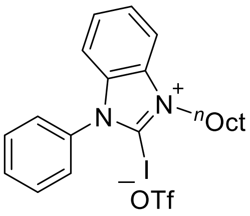

B

^{1}H NMR (600 MHz, CD_2Cl_2) δ 7.85 – 7.73 (m, 4H), 7.70 – 7.66 (m, 1H), 7.61 – 7.57 (m, 1H), 7.55 – 7.50 (m, 2H), 7.42 – 7.36 (m, 1H), 4.61 (t, *J* = 7.8 Hz, 2H), 2.06 – 1.99 (m, 2H), 1.56 – 1.50 (m, 2H), 1.42 (dq, *J* = 9.2, 6.7 Hz, 2H), 1.37 – 1.26 (m, 6H), 0.89 (t, *J* = 7.2 Hz, 3H). **^{13}C NMR** (150 MHz, CD_2Cl_2) δ 135.64, 134.64, 133.70, 132.61, 131.46, 128.38, 128.13, 128.08, 113.77 (d, *J* = 113.77 Hz), 112.85, 51.64, 32.24, 29.73, 29.62, 29.59, 27.28, 23.15, 14.38. **^{19}F NMR** (565 MHz, CD_2Cl_2) δ -78.80. The analytical data are in accordance with the reported literature.[10]

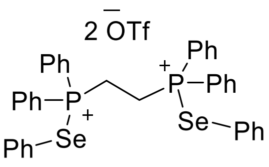

D

TMSOTf (2.0 mmol, 444.52 mg, 0.362 ml) was added to a red solution of PhSeCl (2.0 mmol) in dry CH_2Cl_2 (5.0 mL) at 0 °C. The reaction mixture was allowed to warm to room temperature and stirred for 40 minutes to give a dark orange solution. Then 1,2-bis(diphenylphosphanyl)ethane (1.0 mmol, 398.43 mg) in anhydrous CH_2Cl_2 (5.0 mL) was added over 10 minutes at 0 °C. The reaction mixture was allowed to warm to room temperature and stand for 1 h to generate suspended solid. The suspension was filtered and washed by anhydrous diethyl ether to obtain a white solid (800 mg, 80 %). The analytical data are in accordance with the reported literature.[11]

^{1}H NMR (600 MHz, CD_2Cl_2) δ 7.83 – 7.79 (m, 4H), 7.76 – 7.71 (m, 6H), 7.68 – 7.60 (m, 10H), 7.36 – 7.31 (m, 2H), 7.09 – 7.03 (m, 8H), 3.30 (d, *J* = 3.7 Hz, 4H). **^{13}C NMR** (150 MHz, CD_2Cl_2) δ 137.99, 136.47, 134.41, 134.37, 134.34, 132.04, 132.01, 131.18, 131.14, 131.09, 129.76, 128.33, 121.29 (q, *J* = 318.8 Hz), 118.66. **^{31}P NMR** (243 MHz, CD_2Cl_2) δ 42.91. The analytical data are in accordance with the reported literature.[11]

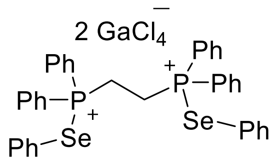

E

^{1}H NMR (600 MHz, CD_2Cl_2) δ 7.93 – 7.85 (m, 4H), 7.76 – 7.69 (m, 8H), 7.65 – 7.56 (m, 8H), 7.43 – 7.37 (m, 2H), 7.15 – 7.07 (m, 4H), 7.05 – 6.98 (m, 4H), 3.09 (d, *J* = 3.6 Hz, 4H). **^{13}C NMR** (150 MHz, CD_2Cl_2) δ 137.89, 137.24, 134.11, 134.07, 134.03, 132.57, 131.75, 131.71, 131.66, 131.53, 117.81, 116.46 (td, *J* = 70.5, 70.6 Hz), 20.65(t, *J* = 20.9 Hz). **^{31}P NMR** (243 MHz, CD_2Cl_2) δ 41.37. The analytical data are in accordance with the reported literature.[11]

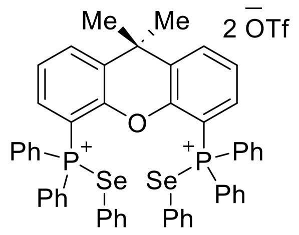

F

^{1}H NMR (400 MHz, CD_2Cl_2) δ 8.24 – 8.09 (m, 2H), 7.63 – 7.36 (m, 26H), 7.22 – 7.16 (m, 8H), 1.96 (s, 6H). **^{13}C NMR** (100 MHz, CD_2Cl_2) δ 153.01, 138.25 (d, *J* = 3.7 Hz), 137.13, 136.90, 136.87, 136.46 (d, *J* = 3.5 Hz), 134.86 (d, *J* = 10.7 Hz), 133.84, 132.38 (d, *J* = 3.5 Hz), 131.30, 131.24, 131.21, 131.16, 126.59, 126.46, 121.32, 121.23, 120.36, 119.58, 35.43, 34.24. The analytical data are in accordance with the reported literature.[12]

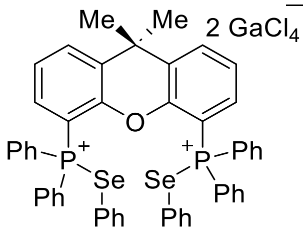

G

^{1}H NMR (400 MHz, CD_2Cl_2) δ 8.17 (d, *J* = 7.9, 2H), 7.65 – 7.59 (m, 4H), 7.57 – 7.51 (m, 8H), 7.50 – 7.38 (m, 13H), 7.22 (t, *J* = 7.6 Hz, 4H), 7.16 – 7.09 (m, 5H), 1.98 (s, 6H). **^{13}C NMR** (100 MHz, CD_2Cl_2) δ 153.09 (d, *J* = 2.3 Hz), 138.15 (d, *J* = 3.8 Hz), 137.21(d, *J* = 8.5 Hz), 137.03(d, *J* = 2.6 Hz), 136.54 (d, *J* = 3.3 Hz), 134.79 (d, *J* = 10.2 Hz), 133.83 (d, *J* = 6.7 Hz), 132.52 (d, *J* = 3.3 Hz), 131.25 (d, *J* = 3.5 Hz), 126.65 (d, *J* = 13.2 Hz), 120.34, 119.56, 34.39. The analytical data are in accordance with the reported literature.[12]

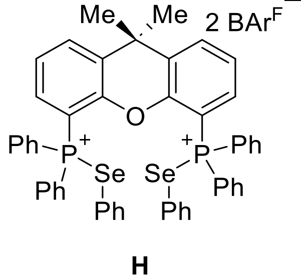

H

^{1}H NMR (600 MHz, CD_2Cl_2) δ 8.12 – 8.07 (m, 2H), 7.75 – 7.69 (m, 16H), 7.62 – 7.58 (m, 4H), 7.56 – 7.52 (m, 8H), 7.49 – 7.43 (m, 8H), 7.41 – 7.38 (m, 4H), 7.35 – 7.28 (m, 8H), 7.19 – 7.13 (m, 4H), 7.13 – 7.06 (m, 2H), 7.01 – 6.96 (m, 4H), 1.87 (s, 6H). **^{13}C NMR** (150 MHz, CD_2Cl_2) δ 162.79, 162.46, 162.13, 161.80, 152.98, 152.96, 137.70, 137.68, 137.30, 137.25, 136.98, 136.97, 136.88, 136.85, 135.35, 134.53, 134.46, 133.86, 133.81, 132.94, 132.91, 131.45, 131.43, 131.25, 131.16, 129.75, 129.71,

129.56, 129.54, 129.52, 129.50, 129.35, 129.33, 129.31, 129.29, 129.11, 127.84, 126.84, 126.75, 126.03, 124.23, 122.42, 120.79, 120.73, 119.93, 119.41, 118.10, 118.07, 118.04, 118.01, 117.99, 35.39, 33.96. **^{19}F NMR** (565 MHz, CD_2Cl_2) δ -62.80. The analytical data are in accordance with the reported literature.[12]

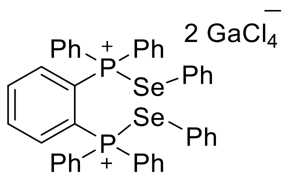

I

^{1}H NMR (400 MHz, CD_2Cl_2) δ 8.23 – 8.15 (m, 4H), 7.83 – 7.77 (m, 4H), 7.65 – 7.59 (m, 8H), 7.50 – 7.44 (m, 8H), 7.41 – 7.36 (m, 2H), 7.13 – 7.08 (m, 4H), 6.74 – 6.66 (m, 4H). **^{13}C NMR** (100 MHz, CD_2Cl_2) δ 143.08, 142.98, 137.92, 137.90, 137.88, 137.40, 137.32, 136.95, 134.77, 134.71, 134.66, 132.94, 131.99, 131.54, 131.48, 131.40, 119.68, 118.91.

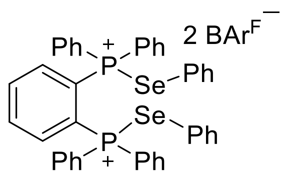

J

The sodium tetrakis[3,5-bis(trifluoromethyl) phenyl]borate (1.0 mmol, 886.2 mg) was added to a solution of catalyst **N** (0.5 mmol, 528.0 mg) in dry CH_2Cl_2 (6.0 mL) under argon and the reaction mixture was stirred at room temperature for 4 h. Then the reaction mixture was filtered and the filtrate was concentrated to give a saturated solution (~4 mL CH_2Cl_2) under reduced pressure and then 10.0 mL *n*-hexane was slowly added. The two-phase solution was then placed for 12 h at 0 °C under argon and the desirable product precipitated out as a white solid. Then the precipitated white solid was collected by filtration and recrystallized from dichloromethane and n-hexane to give pure catalyst **J** as a white solid in 62% yield.

^{1}H NMR (600 MHz, CD_2Cl_2) δ 8.09 – 7.98 (m, 4H), 7.80 – 7.76 (m, 4H), 7.73 – 7.71 (m, 16H), 7.57 – 7.52 (m, 16H), 7.41 – 7.37 (m, 2H), 7.34 – 7.28 (m, 8H), 7.07 (t, *J* = 7.8 Hz, 4H), 6.68 – 6.59 (m, 4H). **^{13}C NMR** (150 MHz, CD_2Cl_2) δ 162.81, 162.48, 162.15, 161.82, 142.68, 142.62, 142.55, 137.58, 137.55, 137.52, 136.84, 136.81, 136.78, 136.75, 136.72, 135.36, 134.40, 134.37, 134.34, 134.31, 134.28, 133.52, 133.50, 131.69, 131.68, 131.64, 131.62, 131.57, 131.53, 131.51, 129.79, 129.77, 129.75, 129.73, 129.58, 129.56, 129.54, 129.52, 129.37, 129.35, 129.33, 129.31, 129.16, 129.14, 129.12, 129.11, 127.85, 126.09, 126.05, 125.61, 125.56, 124.24, 122.44, 121.75, 121.72, 121.70, 119.16, 118.65, 118.12, 118.09, 118.06, 118.04, 118.01. **^{31}P NMR** (243 MHz, CD_2Cl_2) δ 40.23. **^{77}Se NMR** (115 MHz, CD_2Cl_2) δ 363.50 (d, $J_{\text{se-p}}$ = 425.96 Hz). The analytical data are in accordance with the reported literature.[12]

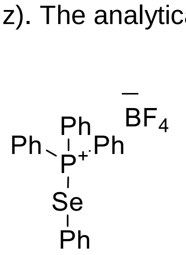

K

$[Ph_3C][BF_4]$(1.0 mmol, 330.15 mg) was added to a solution of 1,2-diphenyldiselane (1.0 mmol, 312.13 mg) in dry CH_2Cl_2 (10.0 mL) and the reaction mixture was stirred for 10 minutes to give a dark green solution. Then Ph_3P (1.0 mmol, 262.29 mg) was added to the above reaction mixture and the reaction was run for 24 hours to generate a pale yellow solution. The solution was reduced to 2.0 mL under vacuum, and 5.0 mL of n-hexane was added with rigorous stirring to give a white precipitate.The

precipitate was filtered off and washed by anhydrous diethyl ether to afford pure catalyst as a light yellow solid in 91% yield (460 mg).

^{1}H NMR (600 MHz, CD_2Cl_2) δ 7.89 – 7.84 (m, 3H), 7.71 – 7.66 (m, 6H), 7.58 (ddd, *J* = 14.1, 8.3, 1.3 Hz, 6H), 7.52 – 7.47 (m, 1H), 7.31 – 7.24 (m, 4H). **^{13}C NMR** (150 MHz, CD_2Cl_2) δ 138.31, 136.50, 136.48, 134.45, 134.38, 132.50, 131.32, 131.12, 131.03, 119.51, 119.08, 118.56. **^{19}F NMR** (565 MHz, CD_2Cl_2) δ -153.00. **^{31}P NMR** (243 MHz, CD_2Cl_2) δ 37.68.

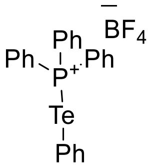

L

[Ph_3C][BF_4] (0.5 mmol, 165.06 mg) was added to a solution of 1,2-diphenylditellurid (0.5 mmol, 204.7 mg) in dry CH_2Cl_2 (5.0 mL) and the reaction mixture was stirred for 10 minutes to give a dark green solution. Then Ph_3P (0.5 mmol, 131.1 mg) was added to the above reaction mixture and the reaction was run for 24 hours to generate a pale yellow solution. The solution was reduced to 2.0 mL under vacuum, and 5.0 mL of n-pantene was added with rigorous stirring to give a yellow precipitate (twice). The precipitate was filtered off and washed by n-pantene to afford pure catalyst **L** as a yellow solid in 54% yield (150 mg).

^{1}H NMR (600 MHz, $CDCl_3$) δ 7.76 – 7.72 (m, 3H), 7.64 – 7.60 (m, 6H), 7.56 – 7.48 (m, 8H), 7.44 – 7.39 (m, 1H), 7.18 – 7.13 (m, 2H). **^{13}C NMR** (150 MHz, $CDCl_3$) δ 142.18, 135.44, 135.42, 134.10, 134.03, 133.22, 133.14, 131.71, 130.86, 130.71, 130.62, 129.44, 129.35, 120.10, 119.65. **^{31}P NMR** (243 MHz, $CDCl_3$) δ 10.06.

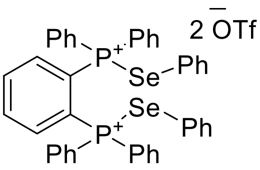

N

TMSOTf (3.0 mmol, 666.7 mg, 2.0 equiv) was added to a red solution of Phenylselenylchlorid (3.0 mmol, 574.5 mg, 2.0 equiv) in dry Et_2O (7.0 mL) at 0 °C under argon. The reaction mixture was allowed to warm to room temperature and stirred for 1 h to give a dark orange solution. Then 1,2-Bis(diphenylphosphino)benzene (1.5 mmol, 669.7 mg, 1.0 equiv) in anhydrous CH_2Cl_2 (4.0 mL) was added over 30 minutes at 0 °C. The reaction mixture was allowed to warm to room temperature and stand for 1 h. After the reaction, a small amount of ether was added to the system, and the white solid suspension was filtered, then dissolve the white solid with 3 ml DCM, add diether until white solid Precipitate, then filtered to get the white solid, washed by ether to get the final product as white solid in 47% yield (750 mg).

^{1}H NMR (500 MHz, CD_2Cl_2) δ 8.23 – 8.11 (m, 4H), 7.79 – 7.73 (m, 4H), 7.62 – 7.51 (m, 16H), 7.39 – 7.35 (m, 2H), 7.12 – 7.07 (m, 4H), 6.85 – 6.73 (m, 4H). **^{13}C NMR** (150 MHz, CD_2Cl_2) δ 143.13, 143.06, 143.00, 138.05, 137.00, 136.70, 135.45, 134.99, 134.95, 134.92, 132.94, 132.90, 132.86, 132.68, 132.01, 131.41, 131.25, 131.17, 130.26, 130.22, 130.17, 129.76, 128.33, 122.61, 119.83, 119.32. **^{19}F NMR** (565 MHz, CD_2Cl_2) δ -78.81.

3. Synthesis of glycosyl iminoglycal donors

3.1. Synthesize glucosyl iminoglycal donors

Method 1

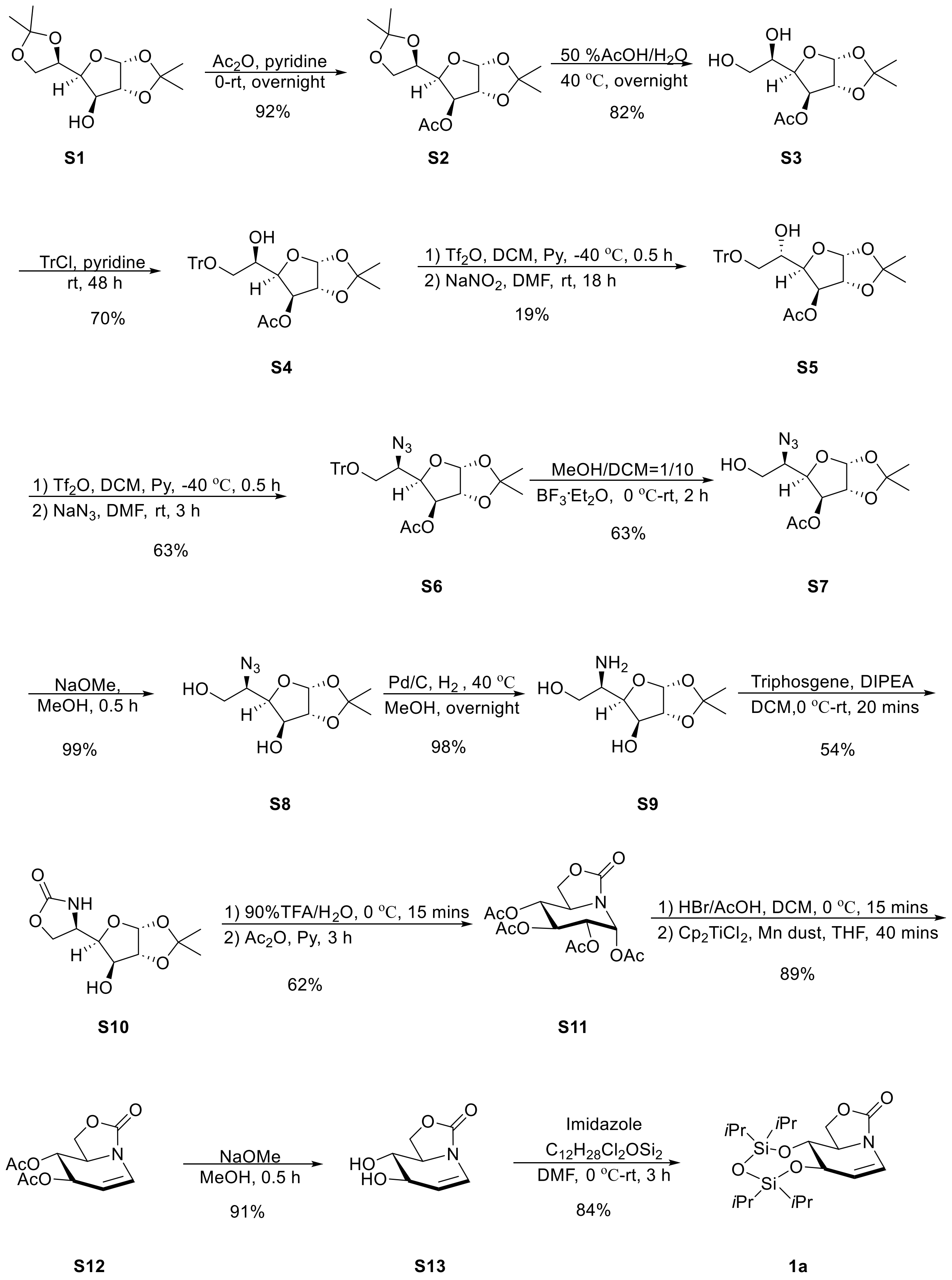

Method 2

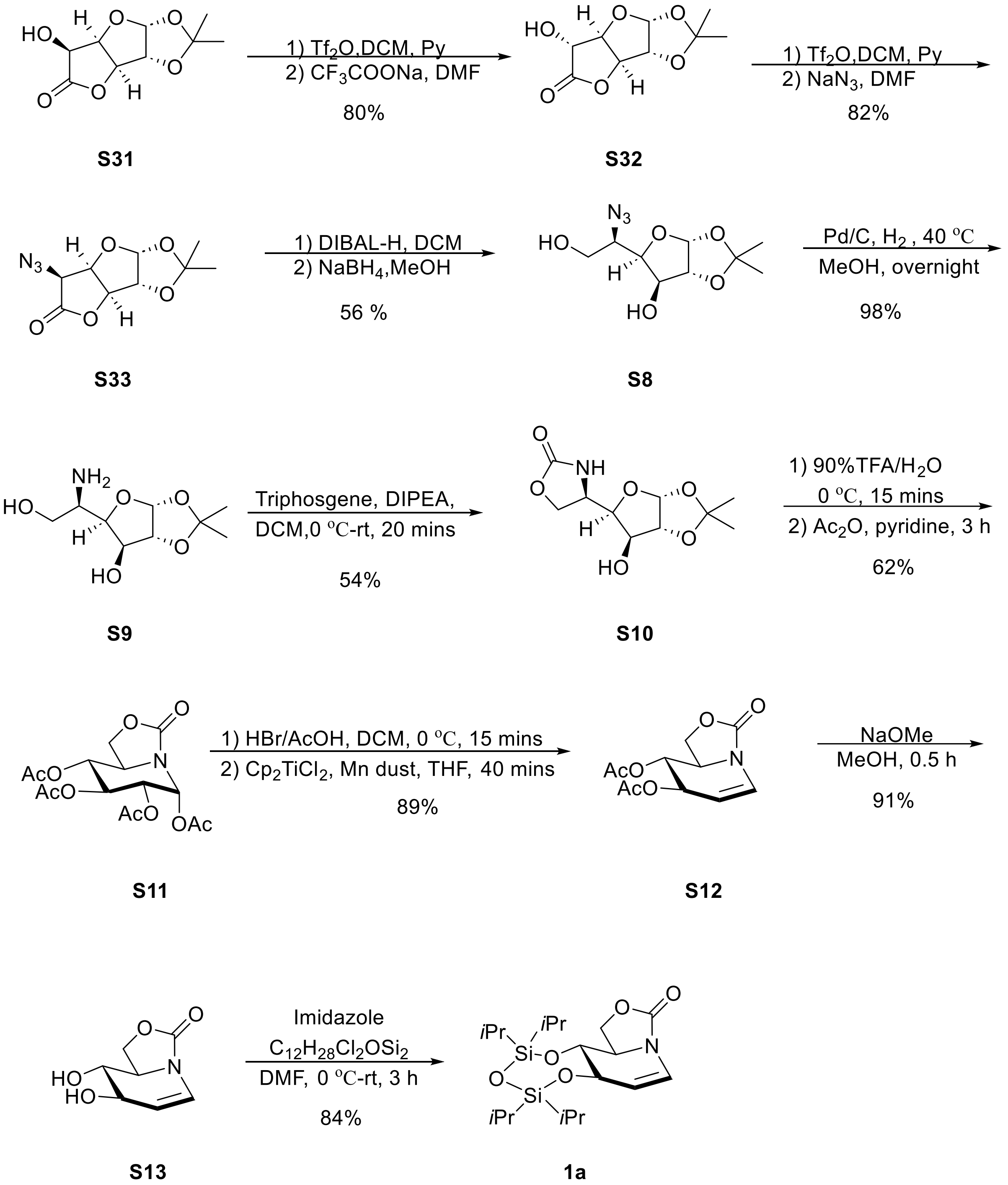

S31
1) Tf2O,DCM, Py
2) CF3COONa, DMF
80%
S32
1) Tf2O,DCM, Py
2) NaN3, DMF
82%
S33
1) DIBAL-H, DCM
2) NaBH4,MeOH
56 %
S8
Pd/C, H2 , 40 °C
MeOH, overnight
98%
S9
Triphosgene, DIPEA,
DCM,0 °C-rt, 20 mins
54%
S10
1) 90%TFA/H2O
0 °C, 15 mins
2) Ac2O, pyridine, 3 h
62%
S11
1) HBr/AcOH, DCM, 0 °C, 15 mins
2) Cp2TiCl2, Mn dust, THF, 40 mins
89%
S12
NaOMe
MeOH, 0.5 h
91%
S13
Imidazole
C12H28Cl2OSi2
DMF, 0 °C-rt, 3 h
84%
1a

Synthesis of glucosyl iminoglycal donors bearing different protecting groups

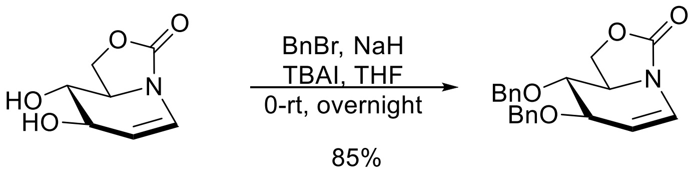

S13 **1b**

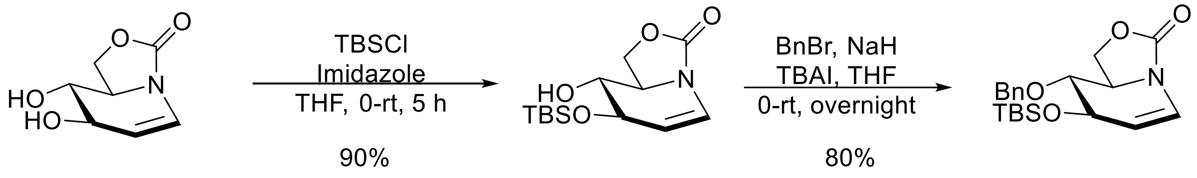

S13 **S14** **1c**

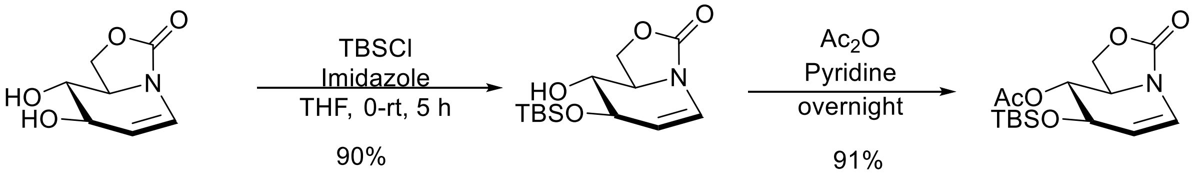

S13 **S14** **1d**

3.2. Galactosyl iminoglycal donors synthesis

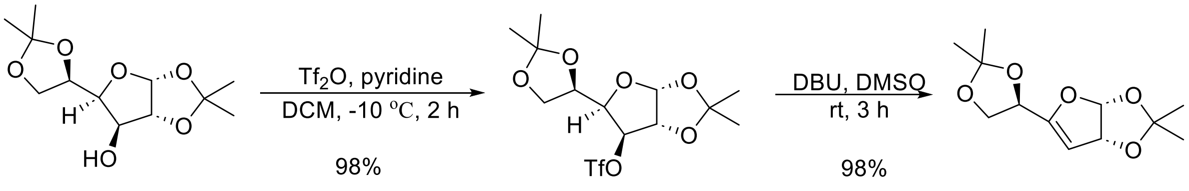

S1 **S15** **S16**

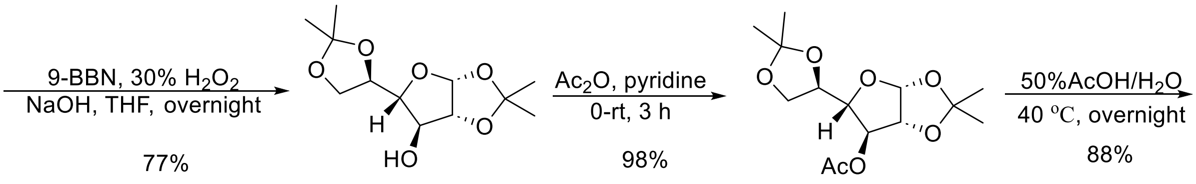

S17 **S18**

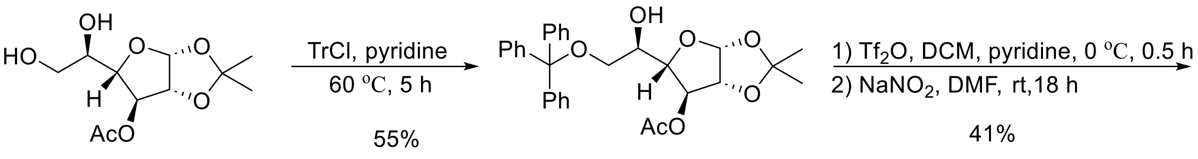

S19 **S20**

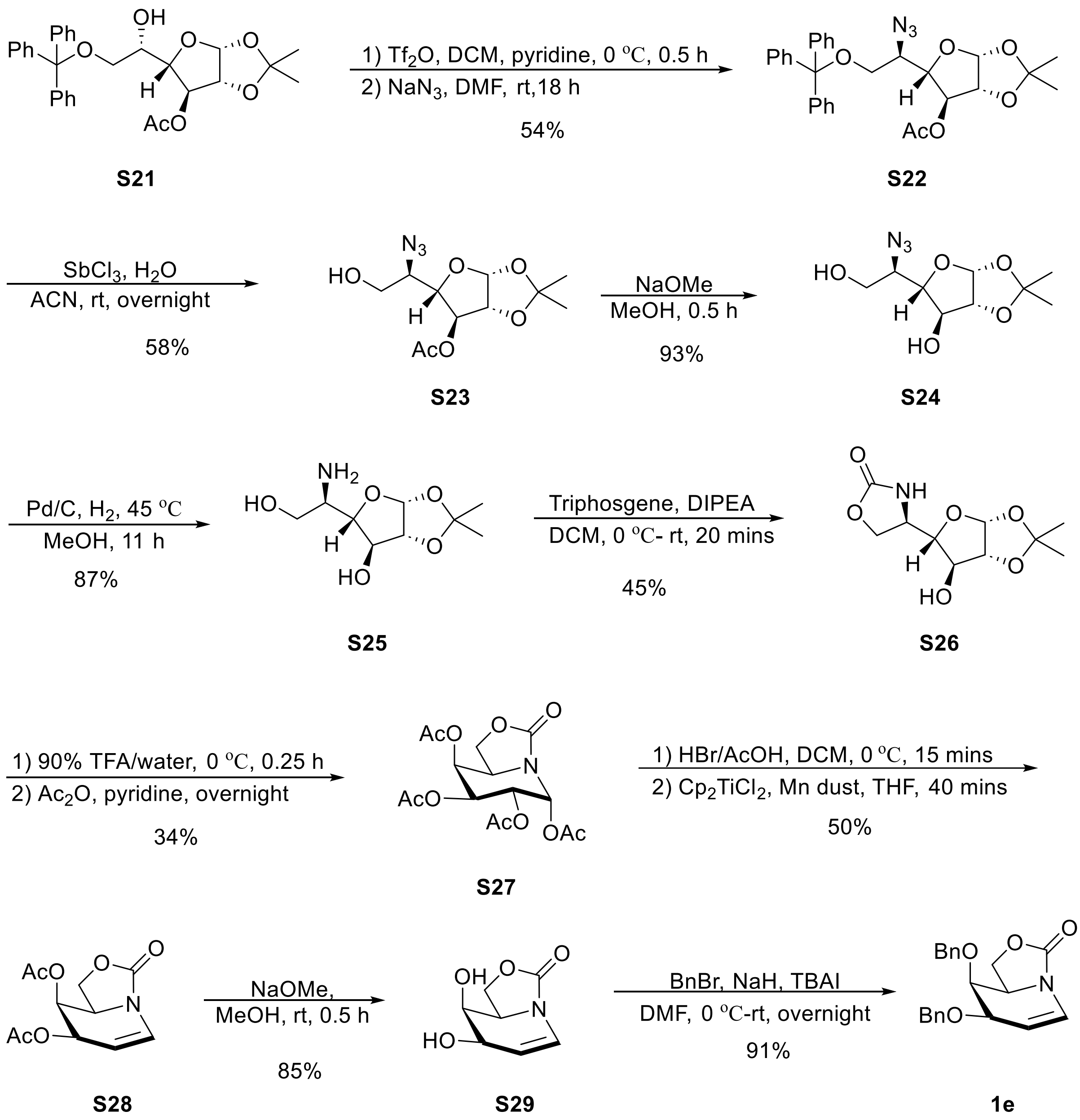

Synthesis of galactosyl iminoglycal donors bearing different protecting groups

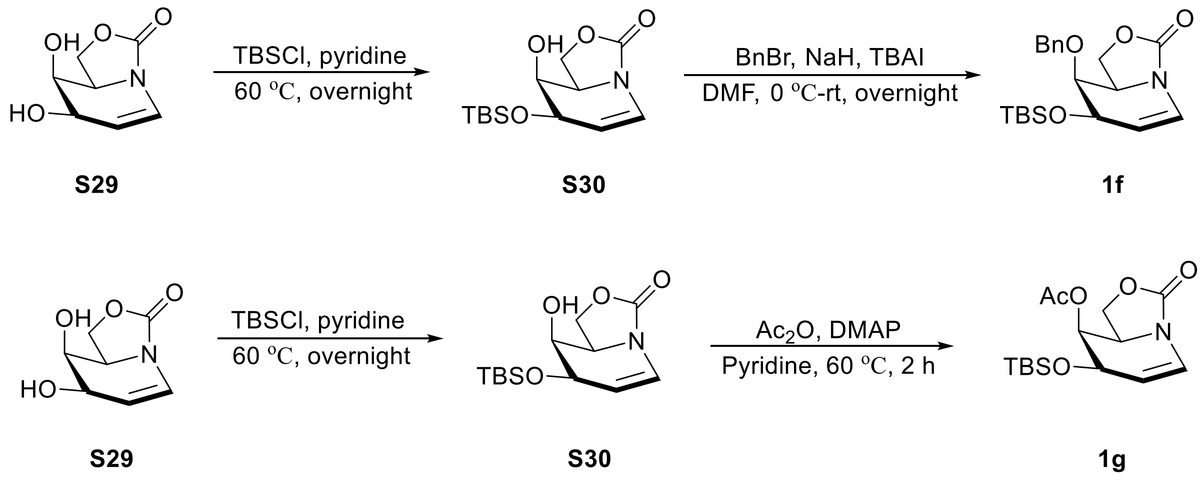

3.3. Data of steps to synthesize iminoglycal donors

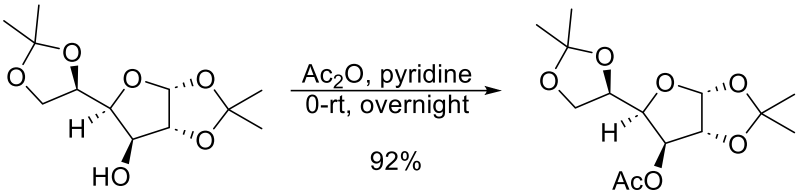

S1 **S2**

Compound **S2** was prepared according to the literature procedure.[1] Dissolve **S1** (150 g, 0.577 mol, 1 equiv) in 800 mL of absolute pyridine at room temperature, add Ac_2O (65.5 mL, 0.692 mol, 1.2 equiv) dropwise to the mixture at 0 ℃, then the reaction stirred at rt for overnight. After it finished through TLC, add 500 mL water to the mixture at 0 ℃, evaporate the pyridine, extract the mixture 3 times with 150 ml of petroleum ether, wash the extract twice with 200 mL of 5% aqueous sodium hydroxide solution and three times with 100 mL of water, dry the extract over anhydrous sodium sulfate, remove the solvent in vacuo. Purify the crude product by flash silica gel chromatography with ethyl acetate: petroleum ether (1:4) as eluent, get the product **S2** 160.0 g (92% yield) as white solid.

^{1}H NMR (600 MHz, $CDCl_3$) δ 5.86 (d, *J* = 4.2 Hz, 1H), 5.24 (d, *J* = 2.4 Hz, 1H), 4.49 (d, *J* = 3.6 Hz, 1H), 4.25 – 4.17 (m, 2H), 4.06 (dd, *J* = 8.4, 5.4 Hz, 1H), 4.02 – 3.99 (m, 1H), 2.09 (s, 3H), 1.50 (s, 3H), 1.40 (s, 3H), 1.30 (d, *J* = 10.2 Hz, 6H). **^{13}C NMR** (150 MHz, $CDCl_3$) δ 169.72, 112.39, 109.45, 105.15, 83.45, 79.80, 76.25, 72.55, 67.29, 26.97, 26.83, 26.31, 25.39, 21.00 . **ESI-HRMS**: Calculated for $C_{14}H_{22}O_7Na$ $(M+Na)^+$: 325.1258, Found: 325.1254. $[\alpha]_D^{20}$ = +23.5 (c = 1.0, $CHCl_3$).

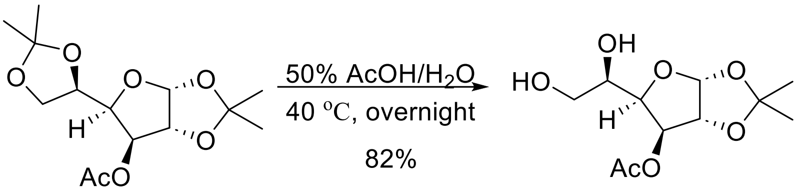

S2 **S3**

Compound **S3** was prepared according to the literature procedure.[1] The protected carbohydrate derivative **S2** (160 g, 0.530 mol, 1.0 equiv) was dissolved in 50% AcOH/H_2O (300 mL) and stirred overnight at 40 ℃. Upon the completion of the reaction, the solvent was removed under pressure and the obtained product was dried under high vacuum. This result in white solid product **S3** 120.0 g (82% yield).

^{1}H NMR (600 MHz, $CDCl_3$) δ 5.96 (d, *J* = 3.6 Hz, 1H), 4.54 (d, *J* = 3.6 Hz, 1H), 4.43 (d, *J* = 9.0 Hz, 1H), 4.37 (t, *J* = 3.0 Hz, 1H), 4.26 – 4.22 (m, 2H), 4.08 (dd, *J* = 6.0, 3.0 Hz, 1H), 3.10 (d, *J* = 3.6 Hz, 1H), 2.96 (d, *J* = 4.2 Hz, 1H), 2.12 (s, 3H), 1.49 (s, 3H), 1.32 (s, 3H). **^{13}C NMR** (150 MHz, $CDCl_3$) δ 171.75, 112.02, 105.11, 85.32, 79.37, 75.84, 69.56, 66.24, 26.96, 26.32, 20.97. **ESI-HRMS**: Calculated for $C_{11}H_{18}O_7Na$ $(M+Na)^+$: 285.0945, Found: 285.0939. $[\alpha]_D^{20}$ = +21.2 (c = 1.0, $CHCl_3$).

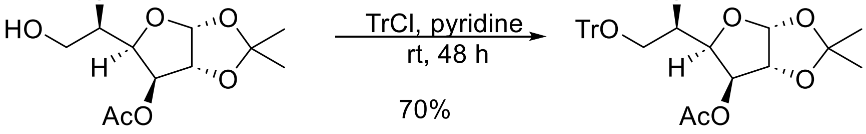

S3 **S4**

Compound **S4** was prepared according to the literature procedure.[1] Trityl chloride (165 g, 594 mmol, 1.3 equiv.) was added to a solution of **S3** (120 g, 458 mmol, 1.0 equiv) in pyridine (400 mL) and the solution was stirred at room temperature for 48 h. The solution was filtered through diatomaceous earth to collect the filtrate. After evaporation to eliminate the pyridne by adding cyclohexane. Then the resulting solution was dissolved in EA and washed with saturated copper sulfate solution twice to eliminate the pyridine, then dried in anhydrous Na_2SO_4, and the solvents evaporated under reduced

pressure. The residue was purified by column chromatography eluting with ethyl acetate/petroleum ether (1:5) to get the product **S4** 160 g (70% yield) as colorless oil.

^{1}H NMR (600 MHz, $CDCl_3$) δ 7.47 – 7.43 (m, 5H), 7.32 – 7.28 (m, 5H), 7.26 – 7.22 (m, 5H), 5.86 (d, *J* = 3.6 Hz, 1H), 5.34 (d, *J* = 3.0 Hz, 1H), 4.51 (d, *J* = 3.6 Hz, 1H), 4.37 (dd, *J* = 9.0, 2.4 Hz, 1H), 3.86 – 3.78 (m, 1H), 3.43 – 3.35 (m, 2H), 2.51 (d, *J* = 5.4 Hz, 1H), 2.09 (s, 3H), 1.51 (s, 3H), 1.31 (s, 3H). **^{13}C NMR** (150 MHz, $CDCl_3$) δ 170.25, 143.90, 128.81, 128.05, 127.25, 112.35, 105.06, 86.91, 83.30, 78.76, 76.42, 67.89, 64.94, 26.80, 26.44, 21.02. **ESI-HRMS**: Calculated for $C_{30}H_{32}O_7Na$ $(M+Na)^+$: 527.2040, Found: 527.2045. $[\alpha]_D^{20}$ = -24.6 (c = 2.0, $CHCl_3$).

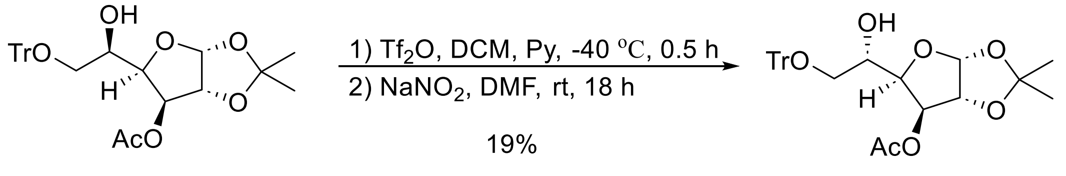

S4 **S5**

Compound **S5** was prepared according to the literature procedure.[1] Pyridine (88.7 mL,1.10 mol, 1.5 equiv) and trifluoromethanesulfonic anhydride (135.8 mL, 807.4 mmol, 1.1 equiv) were added under nitrogen to a solution of **S4** (370 g, 734 mmol, 1.0 equiv) in CH_2Cl_2 (800 mL) at -40 °C. The reaction mixture was allowed to reach room temperature and after stirring for 30 mins, the mixture was diluted with CH_2Cl_2 (400 mL), washed with iced saturated aqueous $NaHCO_3$ (400 mL), dried (anhydrous Na_2SO_4) and concentrated. The resulting triflate ester was dissolved in DMF (400 mL), $NaNO_2$ (505.7 g, 7.34 mol, 10.0 equiv) was added and the reaction mixture was stirred at room temperature for 18 h. The resulting residue was dissolved in CH_2Cl_2 (500 mL) and washed with water (2×300 mL). The organic extract was dried (anhydrous Na_2SO_4) and concentrated. The resulting residue was purified by column chromatography using 1:8-1:5 PE/EA as eluent to give **S5** 70 g (19% yield) as colorless oil.

^{1}H NMR (500 MHz, $CDCl_3$) δ 7.46 – 7.40 (m, 6H), 7.34 – 7.27 (m, 6H), 7.25 – 7.21 (m, 3H), 5.93 (d, *J* = 4.0 Hz, 1H), 5.02 (d, *J* = 3.0 Hz, 1H), 4.50 (d, *J* = 3.5 Hz, 1H), 4.46 (dd, *J* = 6.0, 3.0 Hz, 1H), 4.10 – 4.01 (m, 1H), 3.26 (dd, *J* = 9.5, 4.5 Hz, 1H), 3.06 (dd, *J* = 9.5, 5.5 Hz, 1H), 2.39 (d, *J* = 5.0 Hz, 1H), 1.93 (s, 3H), 1.52 (s, 3H), 1.31 (s, 3H). **^{13}C NMR** (125 MHz, $CDCl_3$) δ 169.82, 143.75, 128.72, 128.05, 127.26, 112.35, 104.49, 86.83, 83.85, 79.18, 76.85, 69.35, 64.53, 26.79, 26.47, 20.90. **ESI-HRMS**: Calculated for $C_{30}H_{32}O_7Na$ $(M+Na)^+$: 527.2040, Found: 527.2044. $[\alpha]_D^{20}$ = -19.0 (c = 1.0, $CHCl_3$).

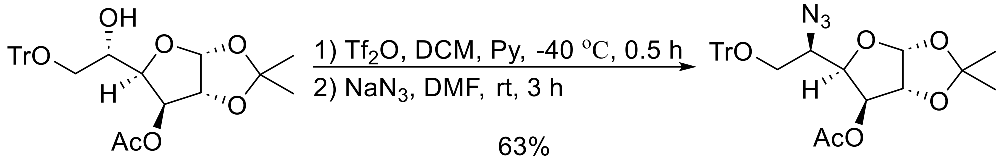

S5 **S6**

Compound **S6** was prepared according to the literature procedure.[1] Pyridine (16.7 mL, 208.3 mmol, 1.5 equiv) and trifluoromethanesulfonic anhydride (25.7 mL, 152.8 mmol, 1.1 equiv) were added under nitrogen to a solution of **S5** (70.0 g, 138.9 mmol, 1.0 equiv) in CH_2Cl_2 (150 mL) at -40 °C. The reaction mixture was allowed to reach room temperature, after the reaction finished (detected through TLC), saturated aqueous $NaHCO_3$ (80 mL) was added until no bulbs formed, the solution was extracted with CH_2Cl_2, washed with saturated sodium chloride solution, and dried (anhydrous Na_2SO_4) and concentrated. The resulting triflate ester was dissolved in DMF (100 mL), NaN_3 (90.3 g, 1.39 mol, 10.0 equiv) was added and the reaction mixture was stirred at room temperature for 3 h. The resulting residue was dissolved in ethyl acetate (100 mL) and washed with saturated sodium chloride solution (2×80 mL). The organic extract was dried (anhydrous Na_2SO_4) and concentrated. The resulting residue was purified by column chromatography (PE/EA=10/1) to give **S6** 46.0 g (63% yield) as colorless oil.

^{1}H NMR (500 MHz, $CDCl_3$) δ 7.50 – 7.45 (m, 6H), 7.34 – 7.29 (m, 6H), 7.26 – 7.22 (m, 3H), 5.82 (d, *J* = 3.5 Hz, 1H), 5.24 (d, *J* = 3.0 Hz, 1H), 4.48 (d, *J* = 4.0 Hz, 1H), 4.19 (dd, *J* = 9.5, 3.0 Hz, 1H), 3.66 (ddd, *J* = 9.5, 7.5, 2.5 Hz, 1H), 3.54 (dd, *J* = 10.0, 2.5 Hz, 1H), 3.37 (dd, *J* = 10.0, 7.5 Hz, 1H), 2.12 (s, 3H), 1.46 (s, 3H), 1.28 (s, 3H). **^{13}C NMR** (125 MHz, $CDCl_3$) δ 169.72, 143.72, 128.84, 128.02, 127.24, 112.47, 105.11, 87.29, 83.03, 77.22, 76.34, 64.36, 60.02, 26.74, 26.32, 21.03. **ESI-HRMS**: Calculated for $C_{30}H_{31}N_3O_6Na$ $(M+Na)^+$: 552.2105, Found:552.2110. $[\alpha]_D^{20}$ = -19.8 (c = 1.1, $CHCl_3$).

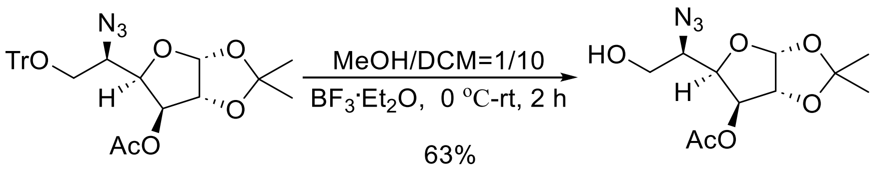

S6 **S7**

Compound **S7** was prepared according to the literature procedure.[1] $BF_3·Et_2O$ complex (25.0 mL) and MeOH (10 mL) were added to a solution of the tritylated azido derivative **S6** (46 g, 91.2 mmol, 1.0 equiv) in CH_2Cl_2 (100 mL) at 0 °C under argon. The reaction mixture was allowed to reach room temperature and stirred for 2 h, diluted with CH_2Cl_2 (100 mL), then washed with saturated aqueous $NaHCO_3$ (2×50 mL), dried (anhydrous Na_2SO_4) and concentrated. The resulting residue was purified by column chromatography (1:5 - 1:2 EtOAc/petroleum ether) to give **S7** 14.0 g (63% yield) as an amorphous white solid.

^{1}H NMR (500 MHz, $CDCl_3$) δ 5.89 (d, *J* = 3.5 Hz, 1H), 5.26 (d, *J* = 3.0 Hz, 1H), 4.53 (d, *J* = 4.0 Hz, 1H), 4.21 (dd, *J* = 9.5, 3.0 Hz, 1H), 3.99 (ddd, *J* = 11.0, 5.5, 3.5 Hz, 1H), 3.82 – 3.71 (m, 2H), 2.20 – 3.15 (m, 1H), 2.13 (s, 3H), 1.50 (s, 3H), 1.30 (s, 3H). **^{13}C NMR** (125 MHz, $CDCl_3$) δ 169.72, 112.66, 105.08, 83.10, 77.78, 76.39, 63.56, 61.26, 26.77, 26.27, 21.03. **ESI-HRMS**: Calculated for $C_{11}H_{17}N_3O_6Na$ $(M+Na)^+$: 310.1010, Found: 310.1005. $[α]_D^{20}$ = -29.7 (c = 1.5, $CHCl_3$).

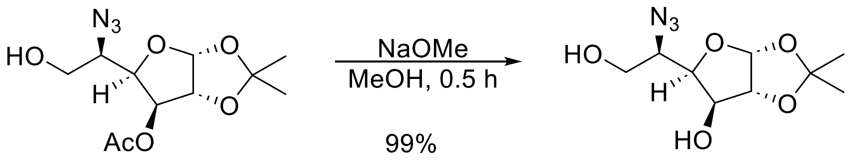

S7 **S8**

Compound **S8** was prepared according to the literature procedure.[1] NaOMe (2.89 g, 53.6 mmol, 1.1 equiv) was added to a solution of **S7** (14 g, 48.7 mmol, 1.0 equiv) in MeOH (50 mL) at room temperature, and stirred for 30 mins. Filter to remove solids, evaporate the solvent in vacuo, Purify the residue by flash column chromatography (PE/EA, 2:1) to obtain **S8** 12.0 g (99% yield) as yellow oil.

^{1}H NMR (500 MHz, $CDCl_3$) δ 5.93 (d, *J* = 4.0 Hz, 1H), 4.53 (d, *J* = 4.0 Hz, 1H), 4.34 – 4.30 (m, 1H), 4.14 – 4.10 (m, 1H), 3.97 (dd, *J* = 11.5, 3.5 Hz, 1H), 3.89 – 3.84 (m, 1H), 3.83 – 3.77 (m, 1H), 2.86 (s, 1H), 2.64 (s, 1H), 1.49 (s, 3H), 1.31 (s, 3H). **^{13}C NMR** (125 MHz, $CDCl_3$) δ 112.23, 104.96, 85.09, 79.29, 74.94, 63.09, 60.91, 26.80, 26.24. **ESI-HRMS**: Calculated for $C_9H_{15}N_3O_5Na$ $(M+Na)^+$: 268.0904, Found: 268.0897. $[α]_D^{20}$ = -13.1 (c = 2.2, $CHCl_3$).

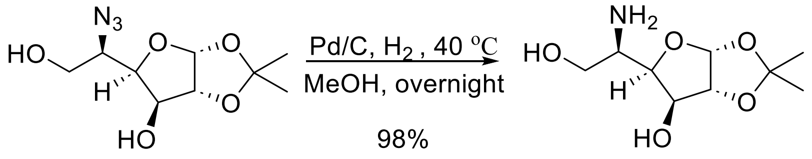

S8 **S9**

Compound **S9** was prepared according to the literature procedure.[1] A solution of azido sugar **S8** (12.0 g, 48.9 mmol, 1.0 equiv) and 10% Pd/C (2.64 g) in MeOH (80 mL) was hydrogenated under an atmospheric pressure of hydrogen using a balloon. The mixture was stirred at 40 °C overnight, filtered through diatomite and concentrated to give **S9** 10.5 g (98% yield) as a hygroscopic solid that was used in the next step without further purification.

^{1}H NMR (600 MHz, CD_3OD) δ 5.88 (d, *J* = 3.7 Hz, 1H), 4.48 (d, *J* = 3.7 Hz, 1H), 4.19 (d, *J* = 2.8 Hz, 1H), 3.98 (dd, *J* = 8.3, 2.8 Hz, 1H), 3.78 (dd, *J* = 11.0, 3.6 Hz, 1H), 3.57 (dd, *J* = 11.0, 6.8 Hz, 1H), 3.18 (ddd, *J* = 8.2, 6.8, 3.6 Hz, 1H), 1.44 (s, 3H), 1.29 (s, 3H). **^{13}C NMR** (150 MHz, CD_3OD) δ 112.63, 106.27, 86.74, 81.65, 75.81, 64.42, 52.73, 27.00, 26.38. **ESI-HRMS**: Calculated for $C_9H_{18}NO_5$ $(M+H)^+$: 220.1180, Found: 220.1172. $[α]_D^{20}$ = -17.2 (c = 1.3, $CHCl_3$).

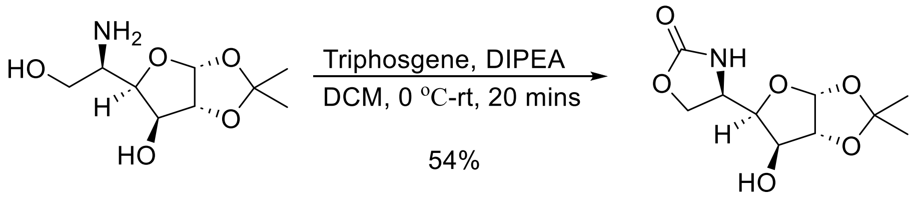

S9 **S10**

Compound **S10** was prepared according to the literature procedure.[2] Diisopropylethylamine (60.3 mL, 478.9 mmol, 10.0 equiv) was added to a stirred solution of **S9** (10.5 g, 47.89 mmol, 1.0 equiv) in CH_2Cl_2 (90 mL) at 0 °C. Then triphosgene (21.3 g, 54.74 mmol, 1.5 equiv) was dissolved in CH_2Cl_2 (20 mL) and added to the solution in dropwise. The reaction mixture was allowed to reach room temperature and stirred for 15 mins. The solvent was removed under reduced pressure and the residue was purified by column chromatography EtOAc/MeOH (40:1) to give **S10** 6.3 g (53% yield) as yellow solid.

^{1}H NMR (600 MHz, CD_3OD) δ 5.96 – 5.88 (m, 1H), 4.53 – 4.43 (m, 3H), 4.20 – 4.10 (m, 3H), 1.46 (s, 3H), 1.30 (s, 3H). **^{13}C NMR** (150 MHz, CD_3OD) δ 162.22, 112.98, 106.65, 86.91, 82.96, 75.51, 68.58, 52.72, 27.14, 26.41. **ESI-HRMS**: Calculated for $C_{10}H_{16}NO_6$ $(M+H)^+$: 246.0972, Found: 246.0965. $[\alpha]_D^{20}$ = -26.5 (c = 1.2, MeOH).

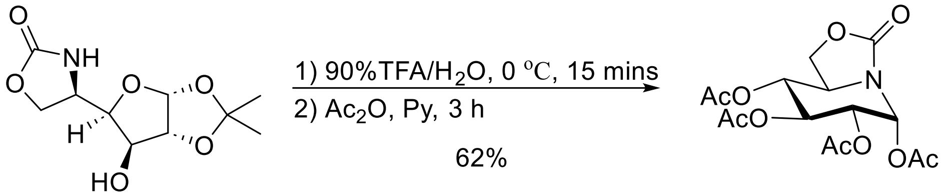

S10 **S11**

Compound **S11** was prepared according to the literature procedure.[2] Compound **S10** (6.3 g, 25.69 mmol, 1.0 equiv) was deacetylated by treatment with 90% TFA/water (60 mL). The reaction mixture was concentrated and the residue was evaporated several times with water to eliminate traces of acid. The resulting residue was subjected to conventional acetylation with Ac_2O/pyridine (1:1, 20 mL), after the reaction finished, quenched with water and wash the solution with saturated copper sulfate solution to eliminate the pyridine. Then the peracetylated mixture was purified by column chromatography using 1:1 EA/PE as eluent. This result in **S11** 5.9 g (62% yield) as white solid.

^{1}H NMR (500 MHz, $CDCl_3$) δ 6.72 (d, J = 4.0 Hz, 1H), 5.50 (t, J = 10.0 Hz, 1H), 5.09 (dd, J = 10.5, 4.0 Hz, 1H), 4.96 (t, J = 10.0 Hz, 1H), 4.45 (dd, J = 9.5, 8.0 Hz, 1H), 4.28 (t, J = 9.0 Hz, 1H), 4.05 (dt, J = 9.5, 8.0 Hz, 1H), 2.14 (s, 3H), 2.08 (s, 3H), 2.05 (s, 3H), 2.01 (s, 3H). **^{13}C NMR** (125 MHz, $CDCl_3$) δ 170.12, 169.97, 169.47, 168.73, 154.31, 77.36, 72.39, 69.16, 69.01, 67.05, 52.84, 20.78, 20.72, 20.66, 20.49. **ESI-HRMS**: Calculated for $C_{15}H_{20}NO_{10}$ $(M+H)^+$: 396.0901, Found: 396.0899. $[\alpha]_D^{20}$ = +52.6 (c = 1.0, $CHCl_3$).

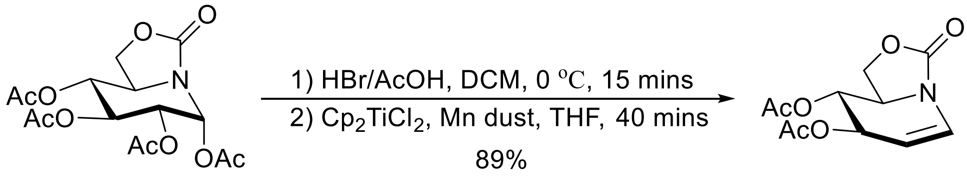

S11 **S12**

Compound **S12** was prepared according to the literature procedure.[2] To a solution of **S11** (5.7 g, 15.27 mmol, 1.0 equiv) in anhydrous DCM (50 mL), HBr/AcOH (33%, 9.56 mL) were dropwise added at 0 °C and the reaction mixture was stirred for 15 mins, detected by TLC(PE/EA=1/1), diluted with DCM (30 mL) and washed with saturated aqueous $NaHCO_3$ (20 mL). The organic layer was dried (anhydrous Na_2SO_4) and concentrated under reduced pressure to yield the corresponding 1-bromo derivative as a white solid. This product was used without further purification in the next step. A mixture of Cp_2TiCl_2 (3.74g, 18.4 mmol, 1.2 equiv) and Mn dust (2.227g, 40.35 mmol, 2.6 equiv) in deoxygenated THF (40 mL) was stirred at rt until the red solution turned green. Then the glycosyl bromo derivative in deoxygenated THF (20 mL) was added and the reaction mixture was stirred for 40 mins. Diluted with EtOAc (80 mL), quenched with 1 M HCl (2 x 10 mL), washed with brine, and dried (anhydrous Na_2SO_4).

The resulting crude was purified by column chromatography (PE/EA=1/1) to yield the corresponding product **S12** 3.5 g (89% yield) as red oil.

^{1}H NMR (500 MHz, $CDCl_3$) δ 6.64 (dd, *J* = 8.0, 2.0 Hz, 1H), 5.60 (dt, *J* = 8.0, 2.0 Hz, 1H), 5.15 (dd, *J* = 10.5, 8.0 Hz, 1H), 4.91 (dd, *J* = 8.0, 2.5 Hz, 1H), 4.47 (t, *J* = 9.0 Hz, 1H), 4.23 (t, *J* = 9.0 Hz, 1H), 4.14 (dd, *J* = 10.5, 8.0 Hz, 1H), 2.05 (s, 3H), 2.03 (s, 3H). **^{13}C NMR** (125 MHz, $CDCl_3$) δ 170.62, 170.19, 153.03, 123.47, 105.80, 71.03, 70.11, 67.03, 53.97, 20.97, 20.73. **ESI-HRMS**: Calculated for $C_{19}H_{32}NO_7$ $(M+H)^+$: 386.2173, Found: 386.2181. $[\alpha]_D^{20}$ = -1.6 (c = 3.5, $CHCl_3$).

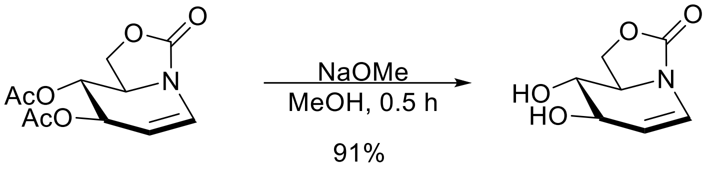

S12 **S13**

Compound **S13** was prepared according to the literature procedure.[3] NaOMe (1.3 g, 24.29 mmol, 2.0 equiv) was added to a solution of **S12** (3.1g, 12.15 mmol, 1.0 equiv) in MeOH (20 ml) at room temperature and stirred for 30 mins. Filter out the solid with MeOH to wash solid, collected the liquid and evaporate the solvent in vacuo, purify the residue by flash column chromatography (DCM/MeOH: 10:1) to obtain **S13** 1.9 g (91% yield) as yellow oil.

^{1}H NMR (500 MHz, CD_3OD) δ 6.50 (dd, *J* = 7.9, 1.9 Hz, 1H), 4.99 (dd, *J* = 7.9, 2.1 Hz, 1H), 4.65 (t, *J* = 8.5 Hz, 1H), 4.29 – 4.19 (m, 2H), 4.07 – 3.94 (m, 1H), 3.55 (dd, *J* = 10.4, 7.8 Hz, 1H). **^{13}C NMR** (125 MHz, CD_3OD) δ 155.95, 122.03, 112.83, 74.47, 72.06, 69.12, 56.86. **ESI-HRMS**: Calculated for $C_7H_{10}NO_4$ $(M+H)^+$: 172.0604, Found:172.0604. $[\alpha]_D^{20}$ = +138.3 (c = 0.3, MeOH).

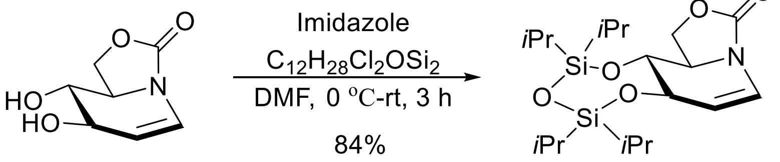

S13 **1a**

Compound **1a** was prepared according to the literature procedure.[4] **S13** (1.5 g, 8.76 mmol, 1.0 equiv) and imidazole (1.193 g, 17.53 mmol, 2.0 equiv) were dissolved in anhydrous DMF (30 mL) under an argon atmosphere and the solution was cooled to 0 ℃, and 1,3-dichloro-1,1,3,3-tetraisopropyldisiloxane (4.2 ml, 13.15 mmol, 1.5 equiv) was added dropwise at this temperature, then increase the solution to room temperature and stirred for 3 h. Afterwards, the solution was poured into ice water, and extracted with EA (20 mL) twice, the organic layer was washed with brine (15 mL), dried over anhydrous Na_2SO_4, filtered and concentrated in vacuo. Following by column chromatography (20:1 n-pentane/EA), the title compound **1a** was obtained 3.05 g (84% yield) as white solid.

^{1}H NMR (500 MHz, $CDCl_3$) δ 6.55 (dd, *J* = 8.0, 2.0 Hz, 1H), 4.94 (dd, *J* = 8.0, 2.0 Hz, 1H), 4.61 (t, *J* = 9.0 Hz, 1H), 4.50 (dt, *J* = 7.0, 2.0 Hz, 1H), 4.17 (t, *J* = 9.0 Hz, 1H), 4.05 – 3.93 (m, 1H), 3.85 (dd, *J* = 10.0, 7.0 Hz, 1H), 1.12 – 0.96 (m, 28H). **^{13}C NMR** (125 MHz, $CDCl_3$) δ 153.64, 121.52, 111.04, 77.36, 76.34, 73.65, 67.51, 55.65, 17.69, 17.47, 17.37, 17.35, 17.30, 17.25, 17.24, 13.08, 12.90, 12.46, 12.27. **ESI-HRMS**: Calculated for $C_{19}H_{36}NO_5Si_2$ $(M+H)^+$: 414.2127, Found:414.2124. $[\alpha]_D^{20}$ = +46.9 (c = 0.9, $CHCl_3$).

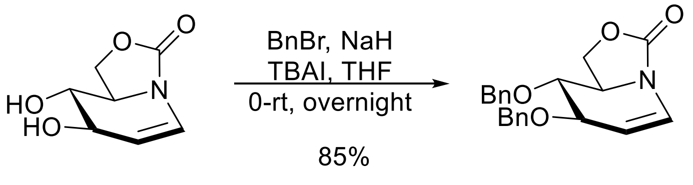

S13 **1b**

Compound **1b** was prepared according to the literature procedure.[3] Substrate **S13** (171.15 mg, 1 mmol, 1.0 equiv) was dissolved in anhydrous THF (10 ml), cooled to 0 ℃, NaH (60%, 159.98 mg, 4 mmol, 4.0 equiv) was added, stirred for 1 h at rt, then added BnBr (0.475 ml, 4 mmol, 4.0 equiv) and TBAI (184.684 mg, 0.5 mmol, 0.5 equiv) to the solution, stirred for overnight, the residue dissolved in DCM, washed

with water, extracted with DCM, dry over anhydrous Na_2SO_4, concentrated in vacuo, then purified through flash chromatography (PE/EA=3/1) to obtain **1b** 300 mg (85% yield) as white solid.

^{1}H NMR (600 MHz, $CDCl_3$) δ 7.26 – 7.19 (m, 7H), 7.17 – 7.14 (m, 3H), 6.46 (dd, *J* = 7.8, 1.8 Hz, 1H), 4.98 (dd, *J* = 7.8, 1.8 Hz, 1H), 4.80 (d, *J* = 12.0 Hz, 1H), 4.64 – 4.48 (m, 3H), 4.38 – 4.33 (m, 1H), 4.28 (dt, *J* = 7.2, 1.8 Hz, 1H), 3.88 – 3.81 (m, 1H), 3.66 (t, *J* = 9.0 Hz, 1H), 3.54 (dd, *J* = 10.2, 7.2 Hz, 1H). **^{13}C NMR** (150 MHz, $CDCl_3$) δ 153.49, 137.85, 137.80, 128.77, 128.70, 128.43, 128.38, 128.11, 128.02, 122.32, 107.37, 79.11, 77.64, 74.09, 71.53, 67.76, 54.56. **ESI-HRMS**: Calculated for $C_{21}H_{22}NO_4$ $(M+H)^+$: 352.1543, Found:352.1542. $[\alpha]_D^{20}$ = +65.2 (c = 1.0, $CHCl_3$).

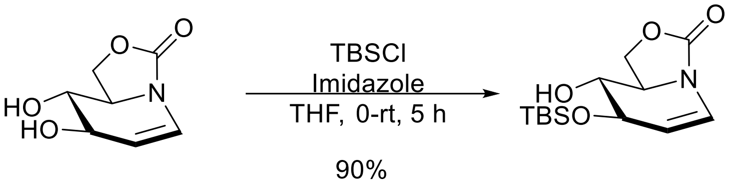

S13 **S14**

Compound **S14** was prepared under the following procedure. **S13** (100 mg, 0.58 mmol, 1.0 equiv) and imidazole (87.5 mg, 1.29 mmol, 2.2 equiv) were dissolved in anhydrous THF (10 mL) under an argon atmosphere and the solution was cooled to 0℃, then added TBSCl (193 mg, 1.29 mmol, 2.2 equiv) to the solution at this temperature and stirred for 5 h. Afterwards, the solution was quenched with water, and extracted with EA (20 mL) twice, the organic layer was washed with brine (15 mL), dried over anhydrous Na_2SO_4, filtered and concentrated in vacuo. then purified through flash chromatography (3:1 PE/EA), the title compound **S14** (150 mg, 90% yield) was obtained as white solid.

^{1}H NMR (700 MHz, $CDCl_3$) δ 6.51 (dd, *J* = 8.4, 2.1 Hz, 1H), 4.86 (dd, *J* = 8.4, 2.1 Hz, 1H), 4.63 (t, *J* = 8.4 Hz, 1H), 4.36 (dt, *J* = 7.0, 2.1 Hz, 1H), 4.21 (t, *J* = 9.1 Hz, 1H), 4.06 – 3.97 (m, 1H), 3.67 (ddd, *J* = 10.5, 7.7, 2.8 Hz, 1H), 0.92 (s, 9H), 0.14 (d, *J* = 3.5 Hz, 6H). **^{13}C NMR** (175 MHz, $CDCl_3$) δ 153.79, 121.69, 110.44, 74.01, 72.54, 67.59, 54.74, 25.88, 18.16, -4.17, -4.38. **ESI-HRMS**: Calculated for $C_{13}H_{24}NO_4Si$ $(M+H)^+$: 286.1469, Found: 286.1464. $[\alpha]_D^{20}$ = +28.1 (c = 0.2, $CHCl_3$).

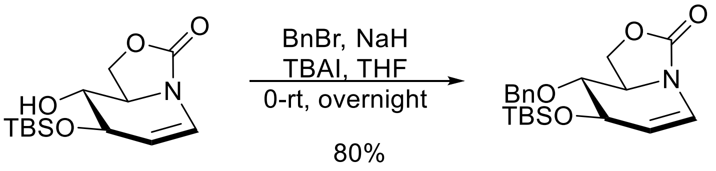

S14 **1c**

Compound **1c** was prepared under the following procedure. Substrate **S14** (85.6 mg, 0.3 mmol, 1.0 equiv) dissolved in anhydrous THF (5 ml), cooled to 0℃, NaH (60%, 23.98 mg, 0.6 mmol, 2.0 equiv) was added, stirred for 1 h at rt, then added BnBr (102.62 mg, 0.6 mmol, 2.0 equiv) and TBAI (55.4 mg, 0.15 mmol, 0.5 equiv) to the solution, the solution were stirred overnight. The residue dissolved in DCM, washed with water, extracted with DCM, dried over anhydrous Na_2SO_4, concentrated in vacuo, and purified through flash chromatography (PE/EA=5/1) to obtain **1c** 90.6 mg (80%, yield) as white solid.

^{1}H NMR (600 MHz, $CDCl_3$) δ 7.40 – 7.32 (m, 3H), 7.31 – 7.28 (m, 2H), 6.49 (dd, *J* = 7.8, 1.8 Hz, 1H), 4.92 (d, *J* = 12.0 Hz, 1H), 4.85 (dd, *J* = 7.8, 1.8 Hz, 1H), 4.62 (d, *J* = 12.0 Hz, 1H), 4.56 (dt, *J* = 7.2, 1.8 Hz, 1H), 4.36 (t, *J* = 9.0 Hz, 1H), 3.93 (td, *J* = 10.2, 8.4 Hz, 1H), 3.58 (t, *J* = 9.0 Hz, 1H), 3.53 (dd, *J* = 10.2, 7.2 Hz, 1H), 0.96 (s, 9H), 0.17 (d, *J* = 5.4 Hz, 6H). **^{13}C NMR** (150 MHz, $CDCl_3$) δ 153.55, 137.90, 128.84, 128.55, 128.47, 121.22, 111.31, 79.56, 74.67, 72.66, 67.63, 54.59, 25.96, 18.12, -4.24, -4.43. **ESI-HRMS**: Calculated for $C_{20}H_{30}NO_4Si$ $(M+H)^+$: 376.1939, Found: 376.1938. $[\alpha]_D^{20}$ = +76.1 (c = 1.1, $CHCl_3$).

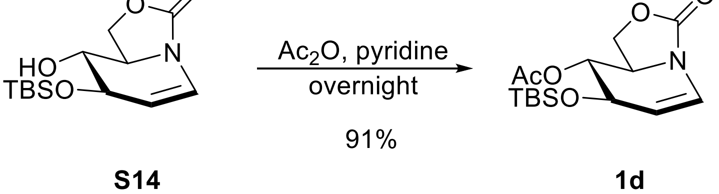

S14 **1d**

Compound **1d** was prepared under the following procedure. Dissolve (85.6 mg, 0.3 mmol, 1.0 equiv) in 5.0 mL of absolute pyridine at room temperature, then add Ac_2O (0.56 mL, 0.6 mmol, 2.0 equiv)

dropwise to the mixture at 0 ℃, then the reaction stirred at room temperature overnight. After it finished through TLC, add 10 mL water to the mixture, evaporate the pyridine, extract the mixture with 20 mL of EA, wash the extract twice with 20 mL of saturated copper(II) sulphate solution and three times with 10 mL of water, dry the extract over anhydrous sodium sulfate, remove the solvent in vacuo, the product was purified through flash chromatography (n-pentane/EA=3/1) to get the title compound **1d** 88.3 mg (91%, yield) as white solid.

^{1}H NMR (600 MHz, $CDCl_3$) δ 6.57 (dd, *J* = 7.8, 1.8 Hz, 1H), 5.01 (dd, *J* = 10.8, 7.8 Hz, 1H), 4.88 (dd, *J* = 7.8, 1.8 Hz, 1H), 4.53 (dt, *J* = 7.2, 2.4 Hz, 1H), 4.43 (dd, *J* = 9.0, 7.8 Hz, 1H), 4.29 (t, *J* = 9.0 Hz, 1H), 4.03 (td, *J* = 10.2, 7.8 Hz, 1H), 2.11 (s, 3H), 0.88 (s, 9H), 0.09 (d, *J* = 21.0 Hz, 6H). **^{13}C NMR** (150 MHz, $CDCl_3$) δ 170.29, 153.42, 121.43, 110.48, 74.28, 69.16, 67.32, 54.51, 25.66, 20.99, 18.04, -4.43, -4.69. **ESI-HRMS**: Calculated for $C_{15}H_{26}NO_5Si$ $(M+H)^+$: 328.1575, Found: 328.1573. $[\alpha]_D^{20}$ = -46.0 (c = 0.5, $CHCl_3$).

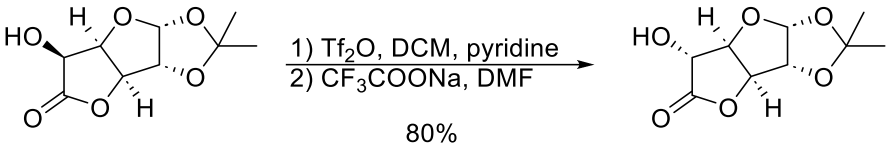

S31 **S32**

Compound **S32** was prepared according to the literature procedure.[5] Triflic anhydride (46.6 mL, 277.54 mmol, 1.2 equiv) was added to a solution of the acetonide **S31** (50.0 g, 231.3 mmol, 1.0 equiv) in DCM (300 mL) and pyridine (37.26 mL, 462.6 mmol, 2.0 equiv) at -30 ℃and stirred for 1 h at room temperature (the solution turned to dark red). The reaction mixture was washed with 2 M HCl (3 x 250 mL) and the organic fraction was dried over anhydrous sodium sulfate, filtered and concentrated. The crude triflate was dissolved in DMF (250 mL) and sodium trifluoroacetate (62.9 g, 462.6 mmol, 2.0 equiv) was added dropwise. The reaction mixture was stirred for overnight at RT and diluted with saturated aqueous $NaHCO_3$ (125 mL), extracted with EA (3 x 250 mL), and the organic phase was dried over anhydrous sodium sulfate, filtered and concentrated. The crude product was purified by recrystallization using EA/PE = 1/3 as solvent to get the compound **S32** 40.0 g (80% yield, over two steps) as white solid.

^{1}H NMR (600 MHz, $CDCl_3$) δ 5.93 (d, *J* = 3.6 Hz, 1H), 5.05 (d, *J* = 3.6 Hz, 1H), 4.83 (d, *J* = 3.6 Hz, 1H), 4.79 (d, *J* = 3.0 Hz, 1H), 4.35 – 4.30 (m, 1H), 3.04 – 2.92 (m, 1H), 1.52 (s, 3H), 1.35 (s, 3H). **^{13}C NMR** (150 MHz, $CDCl_3$) δ 174.75, 113.33, 106.27, 85.20, 82.51, 82.15, 71.84, 27.15, 26.70. **ESI-HRMS**: Calculated for $C_9H_{13}O_6$$(M+H)^+$: 217.0707, Found: 217.0706. $[\alpha]_D^{20}$ = +110.2 (c = 1.0, $CHCl_3$).

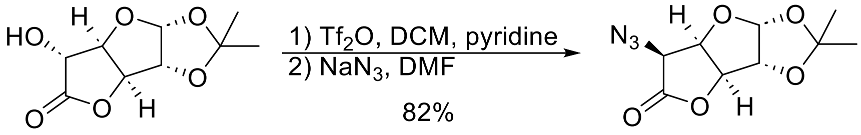

S32 **S33**

Compound **S33** was prepared according to the literature procedure.[5] Triflic anhydride (37.4 mL, 222.03 mmol, 1.2 equiv) was added to a solution of **S32** (40 g, 185.02 mmol, 1.0 equiv) in DCM (300 mL) and pyridine (17.95 mL, 222.03 mmol, 1.2 equiv) at -30 ℃ and the reaction mixture was then stirred at that temperature for a further 1 h. The reaction mixture was diluted with DCM (200 mL) and washed with 2 M HCl (3 x 100 mL), dried over anhydrous sodium sulfate, filtered and concentrated. The crude triflate was dissolved in DMF (200 mL), cooled to -20 ℃, and sodium azide (42.1 g, 647.6 mmol, 3.5 equiv) was added. The reaction mixture was stirred at that temperature for 1 h. The reaction mixture was diluted with 5% aq. NaCl (50 mL) and extracted with EtOAc (3 x 20 mL). The combined organic fractions were dried, filtered and concentrated. The crude product was purified by column chromatography using 1:5 EA-PE as eluent to get the product **S33** 37.0 g (82%, over two steps) as white solid.

^{1}H NMR (600 MHz, $CDCl_3$) δ 6.02 (d, *J* = 3.6 Hz, 1H), 5.02 (dd, *J* = 4.2, 3.0 Hz, 1H), 4.87 – 4.83 (m, 2H), 4.08 (d, *J* = 4.2 Hz, 1H), 1.53 (s, 3H), 1.36 (d, *J* = 0.8 Hz, 3H). **^{13}C NMR** (150 MHz, $CDCl_3$) δ 169.81, 113.80, 106.93, 82.64, 82.58, 79.22, 60.64, 27.05, 26.63. **ESI-HRMS**: Calculated for $C_9H_{12}N_3O_5$$(M+H)^+$: 242.0771, Found: 242.0776. $[\alpha]_D^{20}$ = +21.6 (c = 1.0, $CHCl_3$).

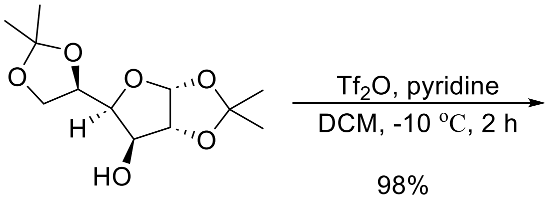

S1 **S15**

Compound **S15** was prepared according to the literature procedure.[6] Add anhydrous pyridine (92.8 mL, 1.155 mol, 1.5 equiv) to a cooled and stirred solution (-10 °C) of 1,2:5,6-di-O-isopropylidene-D-glucofuranose **S1** (200.0 g, 0.77 mol) in dry CH_2Cl_2 (1000 mL) kept under argon atmosphere. Add Tf_2O (142.0 mL, 0.085 mol, 1.1 equiv) dropwise to the mixture. Stir the mixture at -10 °C for 2 hours. Pour the crude mixture into ice and saturated aqueous solution of $NaHCO_3$ (500 mL). Extract the aqueous layer with dichloromethane (500 mL). Dry the combined organic layers over anhydrous sodium sulfate and concentrate under reduced pressure to afford crude product, which was purified through flash chromatography with PE/EA=20/1 to get product **S15** 290.0 g (98% yield) as white solid.

^{1}H NMR (600 MHz, $CDCl_3$) δ 5.98 (d, *J* = 3.6 Hz, 1H), 5.25 (d, *J* = 2.4 Hz, 1H), 4.76 (d, *J* = 3.6 Hz, 1H), 4.23 – 4.17 (m, 2H), 4.15 (dd, *J* = 8.4, 6.0 Hz, 1H), 3.97 (dd, *J* = 8.4, 4.2 Hz, 1H), 1.51 (s, 3H), 1.42 (s, 3H), 1.33 (d, *J* = 5.4 Hz, 6H). **^{13}C NMR** (150 MHz, $CDCl_3$) δ 121.71, 119.59, 117.47, 115.35, 113.25, 109.99, 105.14, 88.30, 83.37, 80.02, 71.83, 67.73, 26.92, 26.68, 26.35, 24.97. **ESI-HRMS**: Calculated for $C_{13}H_{20}F_3O_8S$ $(M+H)^+$: 393.0825, Found: 393.0833. $[\alpha]_D^{20}$ = -33.3 (c = 2.0, $CHCl_3$).

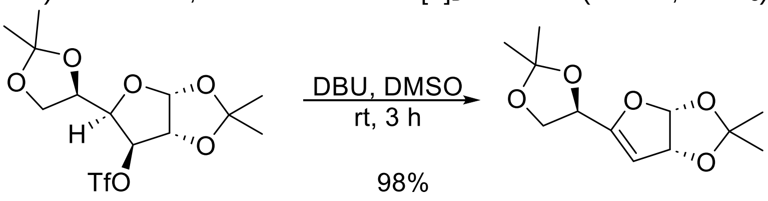

S15 **S16**

Compound **S16** was prepared according to the literature procedure.[7] Add DBU (220 ml, 1.478 mol, 2.0 equiv) to **S15** (290.0 g, 739.0 mmol, 1.0 equiv) in 1000 ml DMSO, the reaction stirred for 3 hours at room temperature. The solution was extracted with EA, and washed with water several times to eliminate DMSO to get the crude product. The residue was purified by flash chromatography eluting with EtOAc/petroleum ether (1:20) to obtain the product **S16** 190.0 g (98 yield) as white solid.

^{1}H NMR (600 MHz, $CDCl_3$) δ 6.08 (d, *J* = 5.4 Hz, 1H), 5.32 – 5.28 (m, 1H), 5.24 (dd, *J* = 2.4, 1.2 Hz, 1H), 4.61 – 4.56 (m, 1H), 4.14 (dd, *J* = 8.4, 6.6 Hz, 1H), 3.97 (dd, *J* = 8.4, 5.4 Hz, 1H), 1.47 (s, 6H), 1.44 (s, 3H), 1.39 (s, 3H). **^{13}C NMR** (150 MHz, $CDCl_3$) δ 160.19, 112.47, 110.49, 106.74, 99.11, 83.55, 71.44, 67.13, 28.40, 28.07, 26.38, 25.67. **ESI-HRMS**: Calculated for $C_{12}H_{19}O_5$ $(M+H)^+$: 243.1227, Found: 243.1220. $[\alpha]_D^{20}$ = +24.8 (c = 2.6, $CHCl_3$).

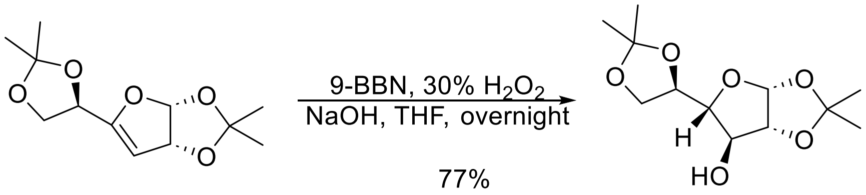

S16 **S17**

Compound **S17** was prepared according to the literature procedure.[8] A solution of the acetal **S16** (190 g, 785 mmol, 1.0 equiv) in 1000 mL of dry THF at 0 °C was treated with 9-BBN (1.884 L, 0.5 M in THF, 1.2 equiv) dropwise. The solution was stirred at rt for 2 hours under argon until the acetal consumed, then decease the solution to 0 °C with ice bar and treated sequentially with 30% H_2O_2 solution (250 mL), add 6 N NaOH (190 mL) to the solution dropwise (give off a lot of heat) and stirred overnight prior to dilution with EA and washing with water and brine. The organic phase was dried, filtered and concentrated. The crude oil was purified by flash chromatography on silica gel with the fluent PE/EA=1/1 to get the compound **S17** 158.0 g (77% yield) as white solid.

^{1}H NMR (600 MHz, $CDCl_3$) δ 5.86 (d, *J* = 4.2 Hz, 1H), 4.54 (dd, *J* = 3.6, 1.2 Hz, 1H), 4.38 – 4.33 (m, 1H), 4.14 – 4.09 (m, 1H), 4.08 – 4.04 (m, 1H), 3.88 – 3.82 (m, 2H), 2.44 – 2.30 (m, 1H), 1.54 (s, 3H),

1.44 (s, 3H), 1.36 (d, *J* = 15.0 Hz, 6H). **^{13}C NMR** (150 MHz, $CDCl_3$) δ 113.77, 110.04, 105.08, 87.75, 86.07, 76.30, 75.51, 65.80, 27.55, 26.86, 26.68, 25.41. **ESI-HRMS**: Calculated for $C_{12}H_{20}O_6Na$ $(M+Na)^+$: 283.1152, Found: 283.1147. $[\alpha]_D^{20}$ = -24.7 (c = 3.4, $CHCl_3$).

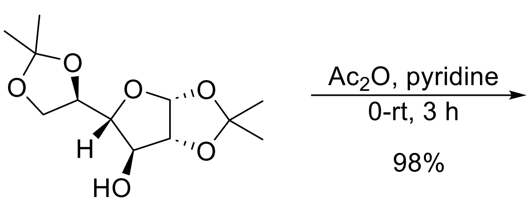

S17 **S18**

Compound **S18** was prepared according to the literature procedure.[8] Dissolve the sugar **S17** (158.0 g, 608.0 mmol, 1.0 equiv) in 350 mL of absolute pyridine at room temperature, add Ac_2O (68.9 mL, 729.6 mmol, 1.2 eq) dropwise to the mixture at 0 °C, then the reaction stirred at rt for 3 hours. After it finished through TLC, add 400 mL water to the mixture, evaporate the pyridine, extract the mixture with 200 mL of EA, wash the extract twice with 300 mL of saturated copper (II) sulphate solution and three times with 100 mL of water, dry the extract over anhydrous sodium sulfate, remove the solvent in vacuo. Purify the crude product by flash silica gel chromatography with ethyl acetate/ petroleum ether (1/8) as eluent to get the product **S18** 180.0 g (98% yield) as white solid.

^{1}H NMR (600 MHz, $CDCl_3$) δ 5.89 (d, *J* = 3.6 Hz, 1H), 4.87 (d, *J* = 2.4 Hz, 1H), 4.53 (d, *J* = 3.6 Hz, 1H), 4.39 – 4.31 (m, 1H), 4.04 (dd, *J* = 8.4, 6.6 Hz, 1H), 3.96 (dd, *J* = 8.4, 2.4 Hz, 1H), 3.81 (dd, *J* = 8.4, 6.6 Hz, 1H), 2.05 (s, 3H), 1.53 (s, 3H), 1.39 (s, 3H), 1.32 (s, 3H), 1.28 (s, 3H). **^{13}C NMR** (150 MHz, $CDCl_3$) δ 169.83, 113.31, 109.95, 105.68, 86.28, 84.60, 75.58, 65.92, 26.93, 26.71, 26.20, 25.37, 20.82. **ESI-**HRMS: Calculated for $C_{14}H_{22}O_7Na$ $(M+Na)^+$: 325.1258, Found: 325.1254. $[\alpha]_D^{20}$ = -16.5 (c = 4.0, CHCl3).

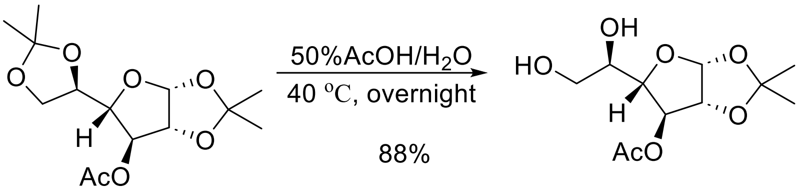

S18 **S19**

Compound **S19** was prepared according to the literature procedure.[1] The protected carbohydrate derivative **S18** (180.0 g, 596.0 mmol, 1.0 equiv) was dissolved in 50% AcOH (300 mL) and stirred overnight at 40 °C. Upon the completion of the reaction, add DCM to extract the product, then purified the residue by flash silica gel chromatography with the eluent PE/EA=1/1 to get the compound **S19** 137.0 g (88% yield) as colorless oil.

^{1}H NMR (600 MHz, $CDCl_3$) δ 5.91 (d, *J* = 4.2 Hz, 1H), 5.04 (d, *J* = 1.8 Hz, 1H), 4.61 (d, *J* = 3.6 Hz, 1H), 4.05 (dd, *J* = 7.8, 1.2 Hz, 1H), 3.90 – 3.84 (m, 1H), 3.73 (d, *J* = 11.4 Hz, 1H), 3.64 (d, *J* = 11.4 Hz, 1H), 3.13 (s, 1H), 2.82 (s, 1H), 2.05 (s, 3H), 1.50 (s, 3H), 1.27 (s, 3H). **^{13}C NMR** (150 MHz, $CDCl_3$) δ 170.35, 112.93, 105.60, 86.43, 84.66, 77.88, 70.63, 63.36, 26.52, 25.85, 20.82. **ESI-HRMS**: Calculated for $C_{11}H_{18}O_7Na$ $(M+Na)^+$: 285.0945, Found: 285.0939. $[\alpha]_D^{20}$ = +27.0 (c = 1.5, $CHCl_3$).

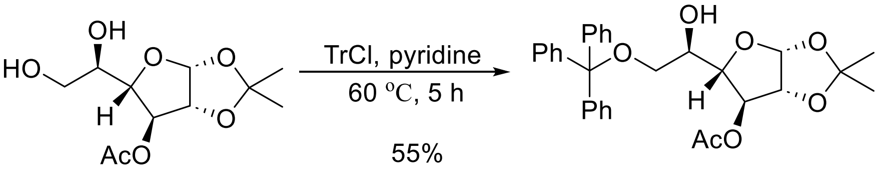

S19 **S20**

Compound **S20** was prepared according to the literature procedure.[9] **S19** (137.0 g, 522.9 mmol, 1.0 equiv), trityl chloride (174.4 g, 627.5 mmol, 1.2 equiv), and DMAP (1.405 g, 11.5 mmol, 2.2 %) were dissolved under argon in pyridine (200 mL) at rt. The reaction mixture was stirred at 60 °C for 5 h and concentrated. The crude product was purified by flash silica gel chromatography with the eluent PE/EA=5/1 to get the compound **S20** 175.0 g (55% yield) as white solid.

^{1}H NMR (600 MHz, $CDCl_3$) δ 7.50 – 7.44 (m, 6H), 7.35 – 7.30 (m, 6H), 7.29 – 7.24 (m, 3H), 5.94 (d, *J* = 4.2 Hz, 1H), 5.16 (d, *J* = 2.4 Hz, 1H), 4.63 (d, *J* = 3.6 Hz, 1H), 4.27 (dd, *J* = 6.6, 2.4 Hz, 1H), 4.07 – 3.99 (m, 1H), 3.36 – 3.25 (m, 2H), 2.72 (s, 1H), 2.07 (s, 3H), 1.57 (s, 3H), 1.34 (s, 3H). **^{13}C NMR** (150

MHz, $CDCl_3$) δ 169.87, 143.92, 128.77, 127.92, 127.13, 113.04, 105.54, 87.00, 86.34, 85.14, 78.20, 69.88, 65.06, 26.74, 26.11, 20.89. **ESI-HRMS**: Calculated for $C_{30}H_{32}O_7Na$ $(M+Na)^+$: 527.2040, Found: 527.2045. $[\alpha]_D^{20}$ = -7.4 (c = 2.8, $CHCl_3$).

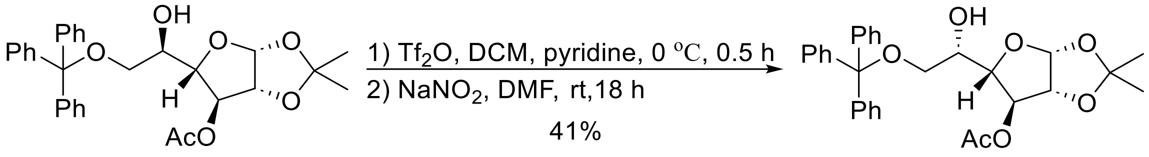

S20 **S21**

Compound **S21** was prepared according to the literature procedure.[1] Pyridine (55.9 mL, 694.4 mmol, 2.0 equiv) and trifluoromethanesulfonic anhydride (87.6 mL, 520.8 mmol, 1.5 equiv) were added under nitrogen to a solution of sugar **S20** (175 g, 347.2 mmol, 1.0 equiv) in CH_2Cl_2 (400 mL) under 0 °C(ice bath). The reaction mixture was allowed to reach room temperature and after stirring for 30 mins, the mixture was diluted with CH_2Cl_2 (200 mL), washed with iced saturated aqueous $NaHCO_3$ (100 mL), dried (anhydrous Na_2SO_4) and concentrated. The resulting triflate ester was dissolved in DMF (200 mL), $NaNO_2$ (119.6 g, 1.736 mol, 5.0 equiv) was added in three times and the reaction mixture was stirred at room temperature for 18 h. The resulting residue was dissolved in CH_2Cl_2 (200 mL) and washed with water (2×80 mL). The organic extract was dried (anhydrous Na_2SO_4) and concentrated. The resulting residue was purified by column chromatography using 1:8-1:5 PE/EA as eluent to give **S21** 72.0 g (41% yield) as yellow solid.

^{1}H NMR (500 MHz, $CDCl_3$) δ 7.48 – 7.44 (m, 6H), 7.35 – 7.30 (m, 9H), 7.29 – 7.24 (m, 3H), 5.91 (d, *J* = 4.0 Hz, 1H), 5.42 (d, *J* = 1.0 Hz, 1H), 4.59 (d, *J* = 3.5 Hz, 1H), 4.22 – 4.18 (m, 1H), 4.10 – 4.03 (m, 1H), 3.46 – 3.31 (m, 2H), 2.10 (s, 3H), 1.51 (s, 3H), 1.31 (s, 3H). **^{13}C NMR** (125 MHz, $CDCl_3$) δ 170.08, 143.87, 128.77, 128.01, 127.24, 112.65, 106.03, 86.92, 85.89, 84.87, 77.94, 70.41, 64.16, 26.80, 25.88, 21.09. **ESI-HRMS**: Calculated for $C_{30}H_{32}O_7Na$ $(M+Na)^+$: 527.2040, Found:527.2044, Found: 527.2045. $[\alpha]_D^{20}$ = -11.5 (c = 1.0, $CHCl_3$).

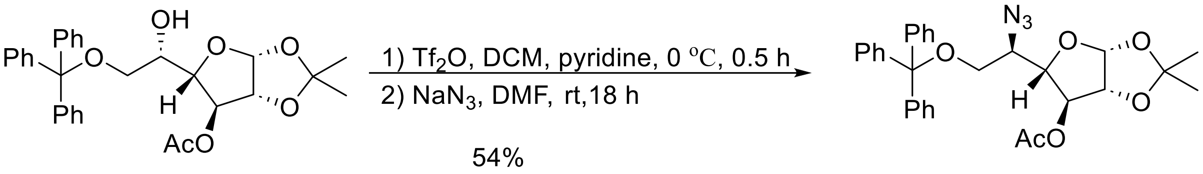

S21 **S22**

Compound **S22** was prepared according to the literature procedure.[1] Pyridine (22.9 mL, 285.2 mmol, 2.0 equiv) and trifluoromethanesulfonic anhydride (36.0 mL, 213.9 mmol, 1.5 equiv) were added under nitrogen to a solution of sugar **S21** (72.0 g, 142.6 mmol, 1.0 equiv) in CH_2Cl_2 (150 mL) under 0 °C. The reaction mixture was allowed to reach room temperature and after stirring for 30 mins, the mixture was diluted with CH_2Cl_2 (100 mL), washed with iced saturated aqueous $NaHCO_3$ (40 mL), dried (anhydrous Na_2SO_4) and concentrated. The resulting triflate ester was dissolved in DMF (150 mL), NaN_3 (46.3 g, 713.0 mmol, 5.0 equiv) was added and the reaction mixture was stirred at room temperature for 18 h. The resulting residue was dissolved in CH_2Cl_2 (200 mL) and washed with water (2×100 mL). The organic extract was dried (anhydrous Na_2SO_4) and concentrated. The resulting residue was purified by column chromatography using 1:10-1:5 PE/EA as eluent to give **S22** 41.0 g (54% yield) as colorless oil.

^{1}H NMR (600 MHz, $CDCl_3$) δ 7.49 – 7.44 (m, 6H), 7.35 – 7.30 (m, 6H), 7.29 – 7.24 (m, 3H), 5.88 (d, *J* = 4.2 Hz, 1H), 5.00 (d, *J* = 2.4 Hz, 1H), 4.56 (d, *J* = 4.2 Hz, 1H), 4.09 (dd, *J* = 7.8, 3.0 Hz, 1H), 3.86 – 3.78 (m, 1H), 3.40 (dd, *J* = 10.2, 3.6 Hz, 1H), 3.30 (dd, *J* = 10.2, 6.6 Hz, 1H), 2.03 (s, 3H), 1.58 (s, 3H), 1.34 (s, 3H). **^{13}C NMR** (150 MHz, $CDCl_3$) δ 169.71, 143.61, 128.71, 128.04, 128.03, 127.30, 113.59, 105.43, 87.44, 84.89, 84.20, 77.37, 63.97, 62.56, 27.04, 26.27, 20.82. **ESI-HRMS**: Calculated for $C_{30}H_{31}N_3O_6Na$ $(M+Na)^+$: 552.2105, Found:552.2110. $[\alpha]_D^{20}$ = -17.1 (c = 1.1, $CHCl_3$).

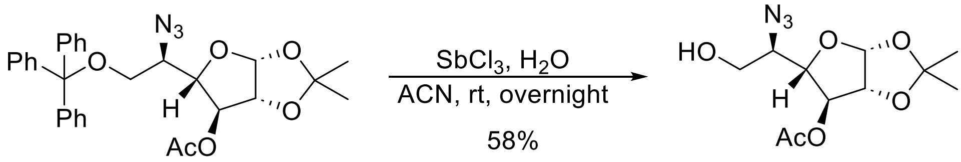

S22 **S23**

Compound **S23** was prepared under the following procedure. Antimony trichloride (1.76 g, 7.74 mmol, 10%) was added to a stirred solution of substrates **S22** (41.0 g, 77.4 mmol, 1.0 equiv) in acetonitrile (150 mL), followed by the addition of water (2.8 mL, 154.8 mmol, 2.0 equiv) at room temperature. After complete conversion, a saturated solution of sodium bicarbonate (80 mL) was added, and the reaction mixture was concentrated under reduced pressure. The residue was extracted with DCM (60 mL). The organic layer was dried (anhydrous Na_2SO_4) and concentrated under reduced pressure to give a crude product, which was isolated through column chromatography (PE/EA = 1/1) to get the compound **S23** 12.8 g (58% yield) as colorless oil.

^{1}H NMR (600 MHz, $CDCl_3$) δ 5.95 (d, *J* = 4.2 Hz, 1H), 5.09 (d, *J* = 1.2 Hz, 1H), 4.65 (d, *J* = 3.6 Hz, 1H), 4.10 (d, *J* = 9.0 Hz, 1H), 3.82 – 3.75 (m, 2H), 3.71 (dd, *J* = 12.0, 4.8 Hz, 1H), 2.75 (s, 1H), 2.07 (s, 3H), 1.55 (s, 3H), 1.29 (s, 3H). **^{13}C NMR** (150 MHz, $CDCl_3$) δ 170.69, 113.12, 105.74, 86.03, 84.29, 77.63, 63.50, 62.51, 26.63, 25.73, 20.80. **ESI-HRMS**: Calculated for $C_{11}H_{17}N_3O_6Na$ $(M+Na)^+$: 310.1010, Found: 310.1005. $[\alpha]_D^{20}$ = -20.1 (c = 2.8, $CHCl_3$).

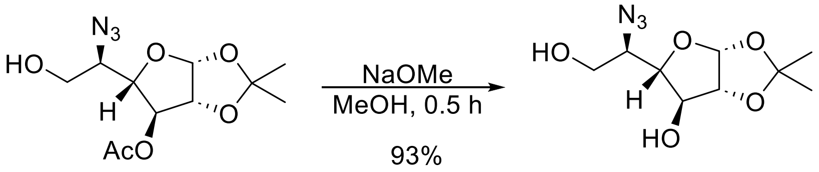

S23 **S24**

Compound **S24** was prepared according to the literature procedure.[1] NaOMe (2.648g, 49.0 mmol, 1.1 equiv) was added to a solution of **S23** (12.8g, 44.56 mmol, 1.0 equiv) in MeOH (50 mL) at room temperature and stirred for 30 mins. Filter to remove solids, evaporate the solvent in vacuo, purify the residue by flash column chromatography (PE/EA = 2:1) to obtain **S24** 10.2g (93% yield) as yellow solid.

^{1}H NMR (600 MHz, $CDCl_3$) δ 5.91 (d, *J* = 3.6 Hz, 1H), 4.59 (dd, *J* = 4.2, 1.2 Hz, 1H), 4.28 (d, *J* = 3.6 Hz, 1H), 4.01 (dd, *J* = 7.8, 3.6 Hz, 1H), 3.84 – 3.73 (m, 3H), 3.11 (s, 1H), 2.62 (s, 1H), 1.56 (s, 3H), 1.35 (s, 3H). **^{13}C NMR** (150 MHz, $CDCl_3$) δ 113.64, 105.30, 87.32, 86.58, 76.44, 63.57, 62.71, 27.27, 26.46. **ESI-HRMS**: Calculated for $C_9H_{15}N_3O_5Na$ $(M+Na)^+$: 268.0904, Found:268.0897. $[\alpha]_D^{20}$ = -62.6 (c = 1.4, $CHCl_3$).

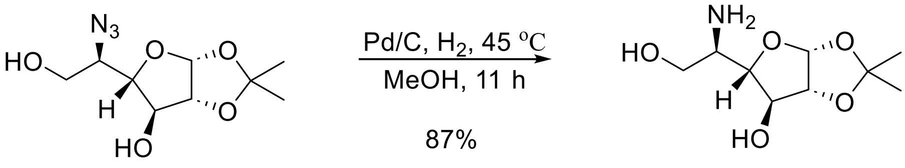

S24 **S25**

Compound **S25** was prepared according to the literature procedure.[1] A solution of azido sugar **S24** (10.2 g, 41.59 mmol, 1.0 equiv) and 10% Pd/C (1.02 g) in MeOH (15 mL) was hydrogenated under an atmospheric pressure of hydrogen using a balloon. The mixture was stirred at 45 °C for 11 h, filtered through celite and concentrated to give **S25** 7.9 g (87% yield) as oil which was used in the next step without further purification.

^{1}H NMR (600 MHz, $CDCl_3$) δ 5.88 (d, *J* = 3.6 Hz, 1H), 4.55 (d, *J* = 4.2 Hz, 1H), 4.20 (d, *J* = 3.0 Hz, 1H), 3.83 (dd, *J* = 8.4, 3.0 Hz, 1H), 3.67 (dd, *J* = 11.4, 4.2 Hz, 1H), 3.60 (dd, *J* = 11.4, 4.8 Hz, 1H), 3.10 (dt, *J* = 9.0, 4.8 Hz, 1H), 1.49 (s, 3H), 1.31 (s, 3H). **^{13}C NMR** (150 MHz, $CDCl_3$) δ 112.82, 105.31, 88.40, 87.48, 76.06, 63.49, 53.75, 27.21, 26.32. **ESI-HRMS**: Calculated for $C_9H_{18}NO_5$ $(M+H)^+$: 220.1180, Found:220.1172. $[\alpha]_D^{20}$ = -8.0 (c = 0.7, $CHCl_3$).

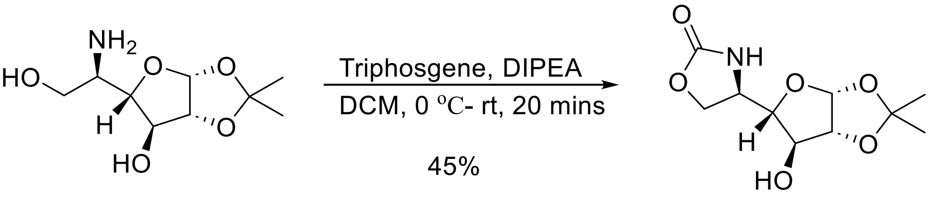

S25 **S26**

Compound **S26** was prepared according to the literature procedure.[2] Diisopropylethylamine (62.8ml, 360.3 mmol, 10.0 equiv) and triphosgene (16.0g, 54.05 mmol, 1.5 equiv) were added to a stirred

solution of **S25** (7.9 g, 36.03 mmol, 1.0 equiv) in CH_2Cl_2 (50 mL) at 0 °C. The reaction mixture was allowed to reach room temperature and stirred for 20 mins. The solvent was removed under reduced pressure and the residue was purified by column chromatography (60:1 EtOAc/MeOH) to give **S26** 4.0 g (45% yield) as yellow solid.

^{1}H NMR (600 MHz, $CDCl_3$) δ 6.24 (d, *J* = 25.8 Hz, 1H), 5.94 (d, *J* = 3.6 Hz, 1H), 4.57 (d, *J* = 4.2 Hz, 1H), 4.47 (t, *J* = 9.0 Hz, 1H), 4.26 (dd, *J* = 9.0, 5.4 Hz, 1H), 4.17 – 4.09 (m, 2H), 4.06 (s, 1H), 4.00 (dd, *J* = 9.0, 1.8 Hz, 1H), 1.51 (s, 3H), 1.31 (s, 3H). **^{13}C NMR** (150 MHz, $CDCl_3$) δ 159.80, 113.06, 105.80, 89.14, 86.83, 77.37, 77.16, 76.95, 75.11, 66.69, 53.89, 27.00, 25.95. **ESI-HRMS**: Calculated for $C_{10}H_{16}NO_6$ $(M+H)^+$: 246.0972, Found:246.0965. $[\alpha]_D^{20}$ = -27.5 (c = 0.2, MeOH).

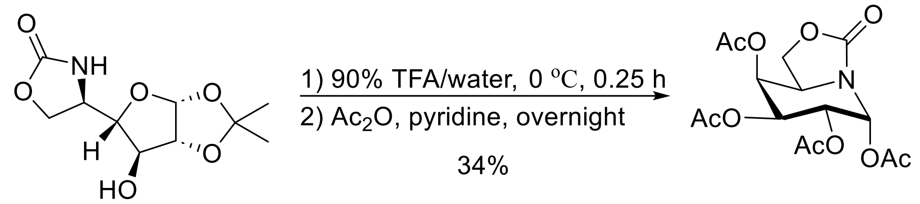

S26 **S27**

Compound **S27** was prepared according to the literature procedure.[2] Compound **S26** (4.0 g, 16.31 mmol, 1.0 equiv) was deacetylated by treatment with 90% TFA/water (20 mL). The reaction mixture was concentrated and the residue evaporated several times with water to eliminate trace of acid. The resulting residue was subjected to conventional acetylation with Ac_2O/pyridine (1:1, 60 mL) and the peracetylated mixture was purified by column chromatography using 1:1 EtOAc/petroleum ether as eluent to get **S27** 2.05 g (34% yield) as white solid.

^{1}H NMR (600 MHz, $CDCl_3$) δ 6.73 (d, *J* = 7.8 Hz, 1H), 5.46 (s, 1H), 5.34 – 5.21 (m, 2H), 4.39 (t, *J* = 9.0 Hz, 1H), 4.34 – 4.29 (m, 1H), 4.03 – 3.98 (m, 1H), 2.18 – 2.13 (m, 3H), 2.12 – 2.09 (m, 3H), 2.00 – 1.96 (m, 6H). **^{13}C NMR** (150 MHz, $CDCl_3$) δ 170.29, 170.07, 169.48, 168.72, 154.39, 72.99, 68.08, 67.51, 65.80, 63.03, 51.46, 20.68, 20.63, 20.62, 20.47. **ESI-HRMS**: Calculated for $C_{15}H_{20}NO_{10}$ $(M+H)^+$: 396.0901, Found:396.0899. $[\alpha]_D^{20}$ = +75.6 (c = 1.3, $CHCl_3$).

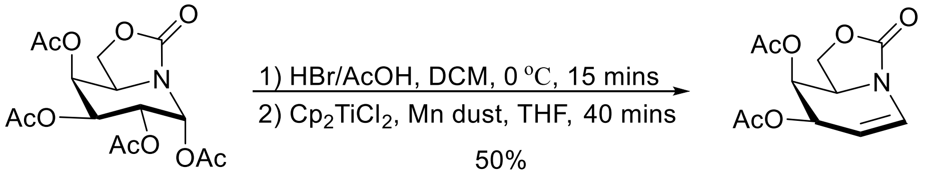

S27 **S28**

Compound **S28** was prepared according to the literature procedure.[2] To a solution of **S27** (2.05 g, 5.49 mmol, 1.0 equiv) in anhydrous DCM (20 mL), HBr/AcOH (33%, 0.7 mL) were added dropwise at 0 °C and the reaction mixture was stirred for 15 mins, detected by TLC, diluted with DCM (30 mL) and washed with saturated aqueous $NaHCO_3$ (20 mL). The organic layer was dried (anhydrous Na_2SO_4) and concentrated under reduced pressure to yield the corresponding 1-bromo derivative as a white solid. This product was used without further purification in the next step. A mixture of Cp_2TiCl_2 (1.639 g, 6.588 mmol, 1.2 equiv) and Mn dust (0.8 g, 14.5 mmol, 2.6 equiv) in deoxygenated THF (20 mL) was stirred at rt until the red solution turned green. Then the 1-bromo derivative (2.0 g) in deoxygenated THF (10 mL) was added and the reaction mixture was stirred for 40 mins. The solvent was removed under reduced pressure, diluted with EtOAc (30 mL), quenched with 1 M HCl (2 x 10 mL), washed with brine, and dried (anhydrous Na_2SO_4). The resulting crude was purified by column chromatography (PE/EA=1/1) to yield the corresponding product **S28** 0.7 g (50% yield) as yellow solid.

^{1}H NMR (600 MHz, $CDCl_3$) δ 6.73 (d, *J* = 7.8 Hz, 1H), 5.65 (d, *J* = 1.2 Hz, 1H), 5.46 (t, *J* = 1.8 Hz, 1H), 4.85 (dt, *J* = 7.8, 1.8 Hz, 1H), 4.49 (t, *J* = 9.0 Hz, 1H), 4.41 (t, *J* = 8.7 Hz, 2H), 4.04 (t, *J* = 8.4 Hz, 1H), 2.15 (s, 3H), 2.04 (s, 3H). **^{13}C NMR** (150 MHz, $CDCl_3$) δ 170.63, 170.39, 153.67, 124.06, 104.99, 66.76, 63.42, 61.49, 53.70, 20.85, 20.79. **ESI-HRMS**: Calculated for $C_{11}H_{14}NO_6$ $(M+H)^+$: 256.0816, Found:256.0809. $[\alpha]_D^{20}$ = +60.8 (c = 1.1, $CHCl_3$).

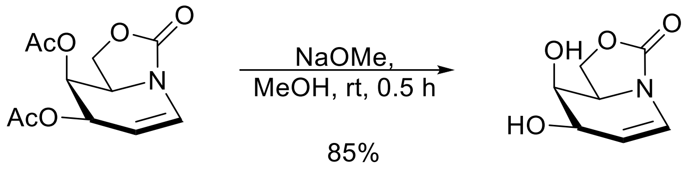

S28 S29

Compound **S29** was prepared according to the literature procedure.[3] NaOMe (254 mg, 4.7 mmol, 2.0 equiv) was added to a solution of **S28** (0.6g, 2.35 mmol, 1.0 equiv) in MeOH (20 mL) at room temperature and stirred for 30 mins. Filter out the solid with MeOH to wash solid, collected the liquid and evaporate the solvent in vacuo, purify the residue by flash column chromatography (DCM/MeOH, 10:1) to obtain **S29** 0.34 g (85% yield) as colorless oil.

^{1}H NMR (500 MHz, CD_3OD) δ 6.50 (dd, *J* = 8.0, 2.0 Hz, 1H), 4.89 (dt, *J* = 8.1, 1.8 Hz, 1H), 4.53 – 4.48 (m, 1H), 4.47 – 4.45 (m, 1H), 4.40 – 4.34 (m, 1H), 4.29 (t, *J* = 9.0 Hz, 1H), 3.84 – 3.80 (m, 1H). **^{13}C NMR** (125 MHz, CD_3OD) δ 156.76, 122.57, 111.44, 67.49, 65.49, 64.60, 56.82. **ESI-HRMS**: Calculated for $C_7H_{10}NO_4$ $(M+H)^+$: 172.0604, Found:172.0604. $[\alpha]_D^{20}$ = +31.8 (c = 0.8, MeOH).

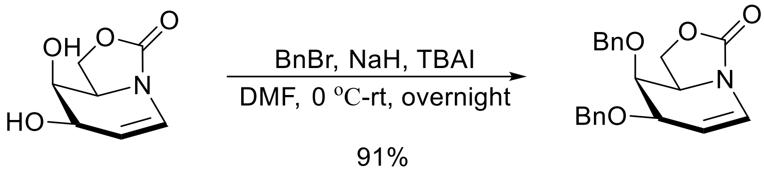

S29 1e

Compound **1e** was prepared according to the literature procedure.[3] To a solution of compound **S29** (0.34 g, 2.0 mmol, 1.0 equiv) in anhydrous DMF (10 mL) stirred at 0 °C was added sodium hydride (320 mg in 60% mineral oil, 8.0 mmol, 4.0 equiv) over a 15 min period. The reaction mixture was stirred for 30 mins and warmed up to room temperature. Then, benzyl bromide (0.95 mL, 8.0 mmol, 4.0 equiv) and tetra-n-butylammonium iodide (TBAI, 147.7 mg, 1.0 mmol, 0.5 equiv) were sequentially added in dropwise. The mixture was stirred at room temperature overnight under a nitrogen atmosphere. After completion of the reaction as monitored by TLC, the resultant was diluted with EtOAc and washed twice with brine. The combined organic layer was dried over anhydrous Na_2SO_4. The filtrate was condensed under reduced pressure and purified by silica gel flash chromatography (EtOAc:PE = 1:1) to get the desired product **1e** 525 mg (91% yield) as an amorphous white solid.

^{1}H NMR (600 MHz, $CDCl_3$) δ 7.40 – 7.36 (m, 4H), 7.35 – 7.29 (m, 6H), 6.63 (dd, *J* = 8.4, 2.4 Hz, 1H), 5.10 – 5.05 (m, 2H), 4.73 (dd, *J* = 24.4, 12.0 Hz, 2H), 4.65 (d, *J* = 12.0 Hz, 1H), 4.38 (s, 1H), 4.23 (t, *J* = 7.8 Hz, 1H), 4.16 – 4.07 (m, 2H), 3.84 – 3.80 (m, 1H). **^{13}C NMR** (150 MHz, $CDCl_3$) δ 154.19, 137.99, 137.90, 128.73, 128.66, 128.35, 128.11, 128.08, 127.63, 122.80, 106.36, 75.30, 74.38, 71.45, 67.59, 63.69, 55.34. **ESI-HRMS**: Calculated for $C_{21}H_{22}NO_4$ $(M+H)^+$: 352.1543, Found: 352.1542. $[\alpha]_D^{20}$ = +25.4 (c = 0.7, $CHCl_3$).

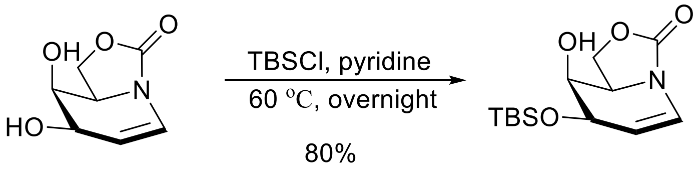

S29 S30

Compound **S30** was prepared under the following procedure. D-galactcal **S29** (8.558 mg, 0.05 mmol, 1.0 equiv) was dissolved in anhydrous pyridine (2.0 mL) under an argon atmosphere and TBSCl (30.144 mg, 0.1 mmol, 2.0 equiv) was added at this temperature. Afterwards, the solution was poured into ice water, and extracted with EA (5 mL) twice, the organic layer was washed with brine (5 mL), dried over anhydrous Na_2SO_4, filtered and concentrated in vacuo and purified by flash chromatography (20:1 n-pentane/EA), the title compound **S30** 11.4 g (80% yield) was obtained as a white solid.

^{1}H NMR (600 MHz, $CDCl_3$) δ 6.61 (dd, *J* = 8.4, 1.8 Hz, 1H), 4.71 (dt, *J* = 7.8, 1.8 Hz, 1H), 4.58 – 4.55 (m, 1H), 4.51 (dd, *J* = 9.6, 8.4 Hz, 1H), 4.44 (t, *J* = 8.4 Hz, 1H), 4.14 (t, *J* = 9.0 Hz, 1H), 3.81 – 3.77 (m,

1H), 2.76 (d, J = 1.2 Hz, 1H), 0.92 (s, 9H), 0.14 (d, J = 9.0 Hz, 6H). **^{13}C NMR** (150 MHz, $CDCl_3$) δ 154.27, 122.58, 108.06, 67.00, 63.76, 63.52, 54.78, 25.84, 25.84, 18.23, -4.54, -4.77. **ESI-HRMS**: Calculated for $C_{13}H_{24}NO_4Si$ $(M+H)^+$: 285.1396, Found: 285.1394. $[\alpha]_D^{20}$ = +18.5 (c = 1.0, $CHCl_3$).

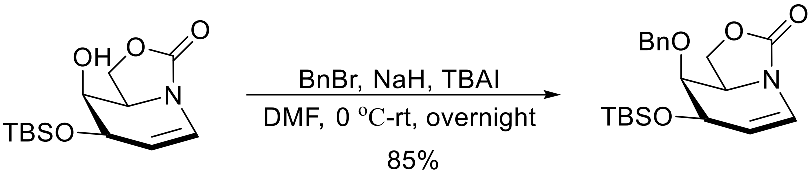

S30 **1f**

Compound **1f** was prepared under the following procedure. To a solution of compound **S30** (14.271 mg, 0.05 mmol, 1.0 equiv) in anhydrous DMF (2.0 mL) stirred at 0 °C was added sodium hydride (3.0 mg in 60% mineral oil, 0.08 mmol, 1.5 equiv) over a 15 min period. The reaction mixture was stirred for 30 mins and warmed up to room temperature. Then, benzyl bromide (10.26 mg, 0.06 mmol, 1.2 equiv) and tetra-n-butylammonium iodide (TBAI, 1.847 mg, 0.01 mmol, 20%) were sequentially added in dropwise. The mixture was stirred at room temperature overnight under a nitrogen atmosphere. After completion of the reaction as monitored by TLC, the resultant was diluted with EtOAc and washed twice with brine. The combined organic layer was dried over anhydrous Na_2SO_4. The filtrate was condensed under reduced pressure and purified by silica gel flash column chromatography (EA:PE = 1:3, v/v). The desired products **1f** 15.69 mg (85% yield) as an amorphous white solid.

^{1}H NMR (600 MHz, $CDCl_3$) δ 7.37 – 7.28 (m, 5H), 6.57 (dd, J = 8.4, 2.4 Hz, 1H), 5.12 (d, J = 12.0 Hz, 1H), 4.84 (dt, J = 7.8, 1.8 Hz, 1H), 4.74 – 4.62 (m, 2H), 4.24 – 4.20 (m, 1H), 4.17 – 4.12 (m, 1H), 4.11 – 4.08 (m, 1H), 3.66 – 3.63 (m, 1H), 0.96 (s, 9H), 0.16 (d, J = 1.8 Hz, 6H). **^{13}C NMR** (150 MHz, $CDCl_3$) δ 154.22, 138.21, 128.66, 128.22, 128.07, 122.00, 109.83, 74.83, 70.24, 69.60, 63.70, 55.29, 26.00, 18.31, -4.52, -4.60. **ESI-HRMS**: Calculated for $C_{20}H_{30}NO_4Si$ $(M+H)^+$: 376.1939, Found: 376.1938. $[\alpha]_D^{20}$ = +4.0 (c = 0.3, $CHCl_3$).

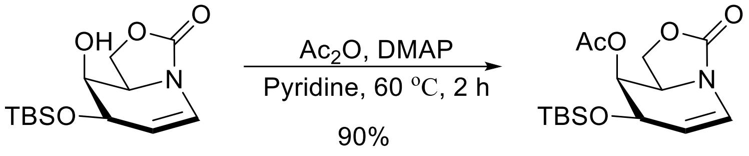

S30 **1g**

Compound **1g** was prepared under the following procedure. To a solution of compound S30 (14.271 mg, 0.05 mmol, 1.0 equiv) in pyridine (2.0 mL) stirred at rt, was added DMAP (1.22 mg, 0.01 mmol, 20%), Ac2O (5.6 ul, 0.06 mmol, 1.2 equiv) was added to the solution dropwise, the mixture was stirred at 60 °C for 2 hours. After it finished through TLC detection, add 5 mL water to the mixture, evaporate the pyridine, extract the mixture with 5 mL of EA, wash the extract twice with 5 mL of saturated copper(II) sulphate solution and three times with 5 mL of water, dry the extract over anhydrous sodium sulfate, remove the solvent in vacuo, the product was purified through flash chromatography (n-pentane/EA=3/1) to get the title compound **1g** 14.7 mg (90% yield) as white solid.

^{1}H NMR (600 MHz, $CDCl_3$) δ 6.60 (dd, J = 8.4, 1.8 Hz, 1H), 5.29 (d, J = 4.2 Hz, 1H), 4.83 (d, J = 7.8 Hz, 1H), 4.66 – 4.62 (m, 1H), 4.45 (t, J = 9.0 Hz, 1H), 4.34 (d, J = 9.0 Hz, 1H), 4.00 (t, J = 9.0 Hz, 1H), 2.14 (s, 3H), 0.87 (s, 9H), 0.09 (d, J = 24.6 Hz, 6H). **^{13}C NMR** (150 MHz, $CDCl_3$) δ 170.97, 153.80, 122.00, 110.05, 65.94, 64.26, 63.57, 54.02, 25.76, 20.96, 18.20, -4.93, -4.97. **ESI-HRMS**: Calculated for $C_{15}H_{26}NO_5Si$ $(M+H)^+$: 328.1575, Found: 328.1573. $[\alpha]_D^{20}$ = +10.5 (c = 0.4, $CHCl_3$).

4. Supplementary methods

4.1. Optimization of reaction conditions

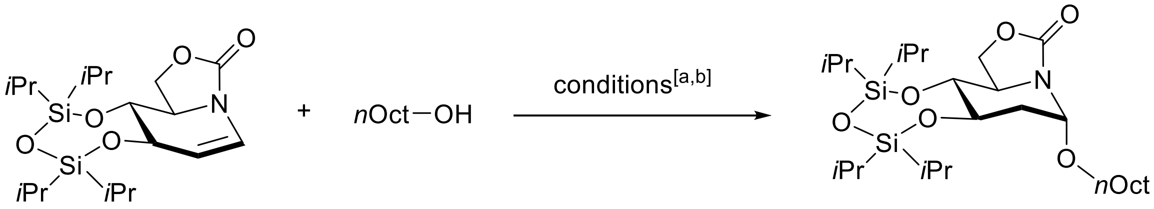

1a **2a** **3a**

Entry	Cat (mol%)	Solvent	Additive (mol%)	Time (h)	Yield (3a)	α : β
1	**A** (5 %)	DCE	-	48	n.d.	-
2[c]	**B** (3 %)	DCM	-	40	n.d.	-
3	**C** (2 %)	DCM	-	48	n.d.	-
4	**D** (5 %)	DCE	-	3	88%	>20/1
5	**E** (5 %)	DCE	-	20	88%	>20/1
6	**F** (5 %)	DCE	-	5	89%	>20/1
7	**G** (5 %)	DCE	-	20	86%	>20/1
8	**H** (5 %)	DCE	-	21	78%	>20/1
9	**I** (5 %)	DCE	-	2	91%	>20/1
10[c]	**J** (2 %)	DCM	-	24	93%	>20/1
11	**K** (5 %)	DCE	-	45	89%	>20/1
12	**L** (5 %)	DCE	-	45	83%	>20/1
13	**M** (0.5 %)	DCE	-	3	6%	>20/1
14	**N** (5 %)	DCE	-	2	91%	>20/1
15[c]	**N** (2 %)	DCM	-	36	84%	>20/1
16[c]	TfOH (2 %)	DCM	-	8	75%	>20/1
17[c]	**J** (2 %)	Toluene	-	24	45%	>20/1
18[c]	**J** (2 %)	THF	-	24	83%	>20/1
19[c]	**J** (0.5 %)	DCM	-	72	87%	>20/1
20[c]	**J** (2 %)	DCM	K_2CO_3 (20%)	24	n.d.	-
21[c]	**J** (2 %)	DCM	(*R*)-BINAP (20%)	24	n.d.	-
22[c]	**J** (2 %)	DCM	TBAC (20%)	24	15%	>20/1
23[c, d]	**J** (2 %)	DCM	3Å molecular sieves (15 mg)	24	n.d.	-

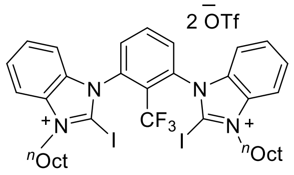
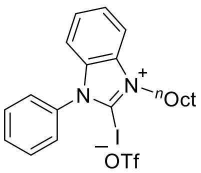
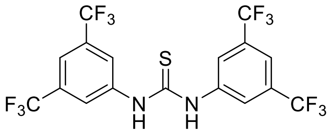
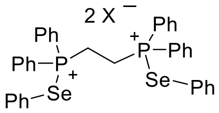

A **B** **C** **D**, X= OTf⁻ **E**, X= GaCl₄⁻

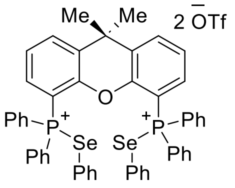
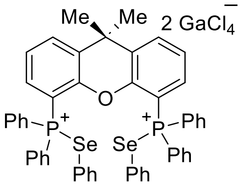
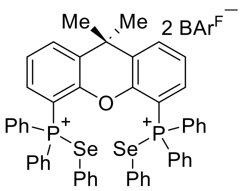
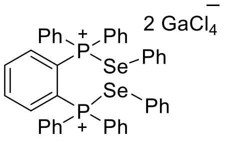

F **G** **H** **I**

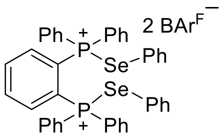
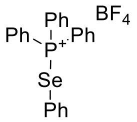
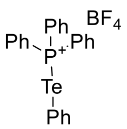
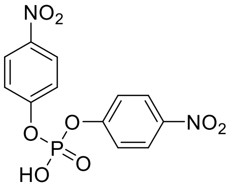
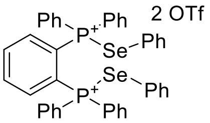

J **K** **L** **M** **N**

Conditions: [a] **1a** (0.05 mmol), **2a** (0.075 mmol), catalyst, 50 ℃, solvent (0.2 mL), time, argon. [b] Yield and α:β selectivity of **3a** were determined by crude ^{1}H NMR spectra analysis using 1,3,5-trimethoxybenzene as an internal standard. n.d.: not detected. [c] Conducted at rt. TBAC = tetrabutylammonium chloride. TfOH = trifluoromethanesulfonic acid.

4.2. General procedure for harnessing multi-step chalcogen bonding activation in the α-stereoselective synthesis of iminoglycosides.

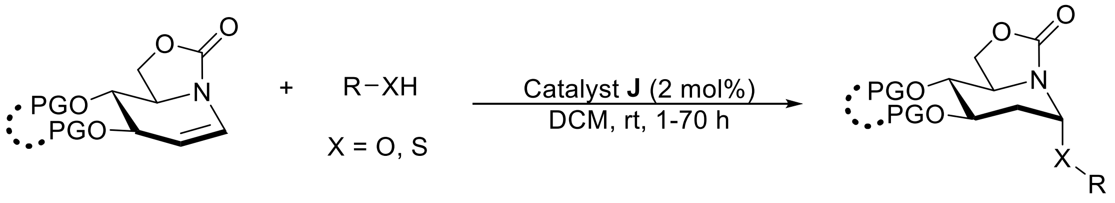

1a-1g **2** **3a-4k**

General procedure: To an oven dried vial was charged donor iminoglycal **1** (0.2 mmol, 1.0 equiv), catalyst **J** (0.004 mmol, 2 mol%), dry CH_2Cl_2 (0.8 mL) and then acceptor alcohol **2** (0.3 mmol, 1.5 equiv). The vial was sealed and the mixture was stirred at room temperature for 1-70 h. The reaction mixture was filtered over a short silica plug and flushed with 20 mL of ethyl acetate twice. The filtrate was then evaporated and the determination of the anomeric selectivity (α/β) and yield were by ^{1}H-NMR analysis of this concentrated crude mixture with 1,3,5-trimethoxybenzene as the internal standard. The crude mixture is subsequently dry loaded onto silica gel and subjected to flash column chromatography for purification.

5. Characterization data for products and intermediate 5

(5a*R*,7*R*,11a*R*,11b*R*)-7-hydroxy-2,2,4,4-tetraisopropylhexahydro-9*H*-oxazolo[3,4-*a*][1,3,5,2,4]trioxadisilepino[6,7-*c*]pyridin-9-one (5)

The title product compound is prepared according to the following procedure: To an oven dried vial was charged iminoglycal **1a** (0.2 mmol, 1.0 equiv), 2 mol% catalyst **J**, dry CH_2Cl_2 (0.8 mL), the vial was sealed and the mixture was stirred at room temperature for 0.5 h. The reaction mixture was filtered over a short silica plug and flushed with 20 mL of ethyl acetate twice, the filtrate was then evaporated and

isolated by flash column chromatography (3:1 Pentane: Ethyl Acetate) giving **5** as a white solid (8.6 mg, 10% yield, α/β ratio > 20:1).

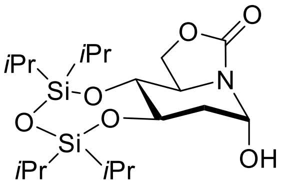

5

^{1}H NMR (600 MHz, $CDCl_3$) δ 5.37 (dd, *J* = 3.9, 1.6 Hz, 1H), 4.47 (t, *J* = 8.5 Hz, 1H), 4.25 (dd, *J* = 9.0, 4.3 Hz, 1H), 4.00 (dd, *J* = 5.7, 2.8 Hz, 1H), 3.62 (ddd, *J* = 9.4, 8.0, 4.3 Hz, 1H), 3.47 (t, *J* = 8.9 Hz, 1H), 2.13 (ddd, *J* = 13.8, 4.6, 1.7 Hz, 1H), 1.74 (ddd, *J* = 13.8, 11.4, 3.9 Hz, 1H), 1.10 – 0.98 (m, 28H).**^{13}C NMR** (150 MHz, $CDCl_3$) δ 155.98, 77.88, 77.23, 71.26, 66.17, 54.98, 37.80, 17.69, 17.59, 17.48, 17.41, 17.35, 17.31, 13.07, 12.96, 12.39, 12.23. **ESI-HRMS**: $C_{19}H_{38}NO_6Si_2$ $(M+H)^+$: 432.2232, Found: 432.2233. $[\alpha]_D^{20}$ = +36.1 (c = 1.0, $CHCl_3$).

(5a*R*,7*R*,11a*R*,11b*R*)-2,2,4,4-tetraisopropyl-7-(octyloxy)hexahydro-9*H*-oxazolo[3,4-*a*][1,3,5,2,4]trioxadisilepino[6,7-*c*]pyridin-9-one (3a)

The title product compound is prepared according to above general procedure with iminoglycal **1a** (0.2 mmol, 1.0 equiv), 2 mol% catalyst **J**, dry CH_2Cl_2 (0.8 mL) and acceptor at room temperature for 24 h and isolated by flash column chromatography (25:1 Pentane: Ethyl Acetate) giving **3a** as a white solid (97 mg, 89% yield, α/β ratio > 20:1).

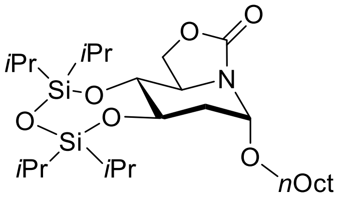

3a

^{1}H NMR (700 MHz, $CDCl_3$) δ 5.12 (d, *J* = 2.8 Hz, 1H), 4.48 (t, *J* = 9.1 Hz, 1H), 4.20 (dd, *J* = 9.1, 5.6 Hz, 1H), 4.03 (ddd, *J* = 11.2, 8.4, 4.9 Hz, 1H), 3.71 – 3.65 (m, 1H), 3.49 – 3.44 (m, 2H), 3.43 – 3.38 (m, 1H), 2.19 (ddd, *J* = 13.3, 4.9, 1.4 Hz, 1H), 1.67 (ddd, *J* = 14.0, 11.2, 4.2 Hz, 1H), 1.59 – 1.52 (m, 2H), 1.35 – 1.23 (m, 10H), 1.12 – 0.93 (m, 28H), 0.88 (t, *J* = 7.0 Hz, 3H). **^{13}C NMR** (175 MHz, $CDCl_3$) δ 156.54, 79.63, 78.95, 71.47, 68.13, 66.69, 54.45, 38.12, 31.94, 29.48, 29.46, 29.41, 26.28, 22.81, 17.73, 17.63, 17.53, 17.44, 17.37, 17.33, 14.23, 13.09, 12.96, 12.41, 12.33. **ESI-HRMS**: Calculated for $C_{27}H_{54}NO_6Si_2$ $(M+H)^+$: 544.3484, Found: 544.3488. $[\alpha]_D^{20}$ = +54.8 (c = 1.0, $CHCl_3$).

(5a*R*,7*R*,11a*R*,11b*R*)-2,2,4,4-tetraisopropyl-7-(((2*R*,3*R*,4*S*,5*R*,6*S*)-3,4,5-tris(benzyloxy)-6-methoxytetrahydro-2*H*-pyran-2-yl)methoxy)hexahydro-9*H*-oxazolo[3,4-*a*][1,3,5,2,4]trioxadisilepino[6,7-*c*]pyridin-9-one (3b)

The title product compound is prepared according to above general procedure with iminoglycal **1a** (0.2 mmol, 1.0 equiv), 2 mol% catalyst **J**, dry CH_2Cl_2 (0.8 mL) and acceptor at room temperature for 1 h and isolated by flash column chromatography (5:1 Pentane: Ethyl Acetate) giving **3b** as a colorless oil (132 mg, 75% yield, α/β ratio > 20:1).

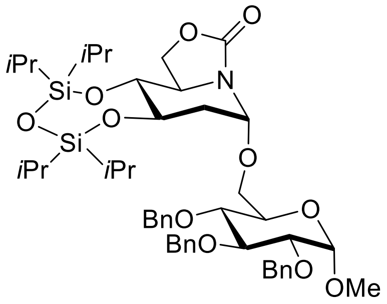

3b

^{1}H NMR (700 MHz, $CDCl_3$) δ 7.39 – 7.25 (m, 15H), 5.18 (d, *J* = 2.8 Hz, 1H), 4.98 (dd, *J* = 27.3, 11.2 Hz, 2H), 4.79 (dd, *J* = 12.6, 7.0 Hz, 2H), 4.68 (d, *J* = 11.9 Hz, 1H), 4.60 (d, *J* = 3.5 Hz, 1H), 4.56 (d, *J* = 11.2 Hz, 1H), 4.19 (t, *J* = 8.4 Hz, 1H), 4.09 (dd, *J* = 8.4, 5.6 Hz, 1H), 4.03 – 3.95 (m, 2H), 3.79– 3.75 (m, 1H), 3.69 – 3.63 (m, 2H), 3.60 – 3.56 (m, 1H), 3.52 (dd, *J* = 9.1, 3.5 Hz, 1H), 3.47 – 3.40 (m, 2H), 3.36 (s, 3H), 2.24 (dd, *J* = 11.9, 4.2 Hz, 1H), 1.70 – 1.60 (m, 1H), 1.11 – 0.93 (m, 28H). **^{13}C NMR** (175 MHz, $CDCl_3$) δ 156.50, 138.79, 138.43, 138.26, 128.62, 128.57, 128.24, 128.10, 128.09, 127.81, 127.80, 127.32, 98.10, 82.27, 80.20, 78.76, 78.15, 75.87, 75.01, 73.51, 71.50, 69.87, 66.69, 66.65, 55.12, 54.31, 37.89, 17.70, 17.68, 17.49, 17.39, 17.37, 17.36, 17.34, 17.30, 13.04, 12.96, 12.39, 12.23. **ESI-HRMS**: Calculated for $C_{47}H_{67}NO_{11}Si_2Na$ $(M+Na)^+$: 900.4145, Found: 900.4158. $[\alpha]_D^{20}$ = +40.4 (c = 1.4, $CHCl_3$).

(5a*R*,7*R*,11a*R*,11b*R*)-2,2,4,4-tetraisopropyl-7-(((3a*R*,5*R*,5a*S*,8a*S*,8b*R*)-2,2,7,7-tetramethyltetrahydro-5*H*-bis([1,3]dioxolo)[4,5-b:4',5'-d]pyran-5-yl)methoxy)hexahydro-9*H*-oxazolo[3,4-*a*][1,3,5,2,4]trioxadisilepino[6,7-*c*]pyridin-9-one (3c)

The title product compound is prepared according to above general procedure with iminoglycal **1a** (0.2 mmol, 1.0 equiv), 2 mol% catalyst **J**, dry CH_2Cl_2 (0.8 mL) and acceptor at room temperature for 1 h and isolated by flash column chromatography (5:1 Pentane: Ethyl Acetate) giving **3c** as a white solid (85 mg, 63% yield, α/β ratio > 20:1).

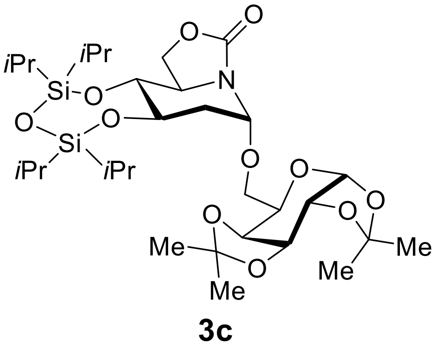

3c

^{1}H NMR (500 MHz, $CDCl_3$) δ 5.51 (d, *J* = 5.0 Hz, 1H), 5.18 (d, *J* = 2.5 Hz, 1H), 4.59 (dd, *J* = 8.0, 2.5 Hz, 1H), 4.49 (t, *J* = 8.5 Hz, 1H), 4.30 (dd, *J* = 5.0, 2.5 Hz, 1H), 4.22 – 4.15 (m, 2H), 4.07 – 4.00 (m, 1H), 3.99 – 3.93 (m, 1H), 3.92 – 3.82 (m, 1H), 3.72 – 3.66 (m, 2H), 3.46 (t, *J* = 9.0 Hz, 1H), 2.22 (ddd, *J* = 13.5, 4.5, 1.5 Hz, 1H), 1.73 – 1.61 (m, 1H), 1.53 (s, 3H), 1.43 (s, 3H), 1.31 (d, *J* = 4.5 Hz, 6H), 1.10 – 0.98 (m, 28H). **^{13}C NMR** (125 MHz, $CDCl_3$) δ 156.65, 109.70, 108.79, 96.48, 79.42, 78.96, 71.58, 71.49, 70.88, 70.65, 67.39, 67.02, 66.80, 54.04, 38.04, 26.11, 26.08, 25.15, 24.66, 17.72, 17.68, 17.52, 17.43, 17.40, 17.35, 17.34, 17.32, 13.08, 12.89, 12.38, 12.27. **ESI-HRMS**: Calculated for $C_{31}H_{56}NO_{11}Si_2$ $(M+H)^+$: 674.3386, Found: 674.3396. $[\alpha]_D^{20}$ = +9.3 (c = 1.3, $CHCl_3$).

(5a*R*,7*R*,11a*R*,11b*R*)-2,2,4,4-tetraisopropyl-7-(((2*R*,3*R*,4*S*,5*R*,6*S*)-3,4,5,6-tetramethoxytetrahydro-2*H*-pyran-2-yl)methoxy)hexahydro-9*H*-oxazolo[3,4-*a*][1,3,5,2,4]trioxadisilepino[6,7-*c*]pyridin-9-one (3d)

The title product compound is prepared according to above general procedure with iminoglycal **1a** (0.2 mmol, 1.0 equiv), 2 mol% catalyst **J**, dry CH_2Cl_2 (0.8 mL) and acceptor at room temperature for 2 h and isolated by flash column chromatography (5:1 Pentane: Ethyl Acetate) giving **3d** as a colorless oil (102 mg, 79% yield, α/β ratio > 20:1).

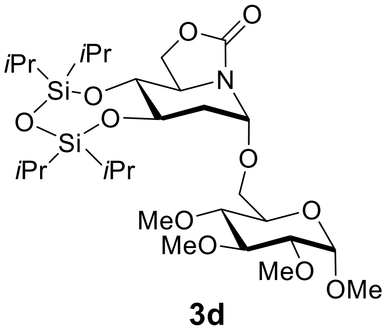

3d

^{1}H NMR (500 MHz, $CDCl_3$) δ 5.22 (d, *J* = 2.5 Hz, 1H), 4.79 (d, *J* = 3.5 Hz, 1H), 4.50 (t, *J* = 9.0 Hz, 1H), 4.20 (dd, *J* = 9.0, 6.0 Hz, 1H), 4.08 – 3.99 (m, 1H), 3.76 (td, *J* = 8.5, 6.0 Hz, 1H), 3.70 – 3.65 (m, 1H), 3.64 – 3.58 (m, 5H), 3.54 – 3.45 (m, 8H), 3.37 (s, 3H), 3.17 (dd, *J* = 9.5, 3.5 Hz, 1H), 3.02 (t, *J* = 9.5 Hz, 1H), 2.25 (ddd, *J* = 13.0, 5.0, 1.5 Hz, 1H), 1.68 (ddd, *J* = 14.0, 11.5, 4.0 Hz, 1H), 1.11 – 0.94 (m, 28H). **^{13}C NMR** (125 MHz, $CDCl_3$) δ 156.54, 97.34, 83.78, 81.99, 80.07, 79.75, 78.82, 71.44, 69.81, 66.85, 66.72, 61.04, 60.60, 59.13, 55.01, 54.36, 37.90, 17.70, 17.60, 17.48, 17.39, 17.33, 17.32, 17.31, 17.29, 13.05, 12.94, 12.35, 12.16. **ESI-HRMS**: Calculated for $C_{29}H_{56}NO_{11}Si_2$ $(M+H)^+$: 650.3386, Found: 674.3399. $[\alpha]_D^{20}$ = +90.8 (c = 1.0, $CHCl_3$).

(2*S*,3*R*,4*S*,5*R*,6*R*)-2-methoxy-6-((((5a*R*,7*R*,11a*R*,11b*R*)-2,2,4,4-tetraisopropyl-9-oxohexahydro-9*H*-oxazolo[3,4-*a*][1,3,5,2,4]trioxadisilepino[6,7-*c*]pyridin-7-yl)oxy)methyl)tetrahydro-2*H*-pyran-3,4,5-triyl tribenzoate (3e)

The title product compound is prepared according to above general procedure with iminoglycal **1a** (0.2 mmol, 1.0 equiv), 2 mol% catalyst **J**, dry CH_2Cl_2 (0.8 mL) and acceptor at room temperature for 2 h and isolated by flash column chromatography (3:1 Pentane: Ethyl Acetate) giving **3e** as a white solid (149 mg, 81% yield, α/β ratio > 20:1).

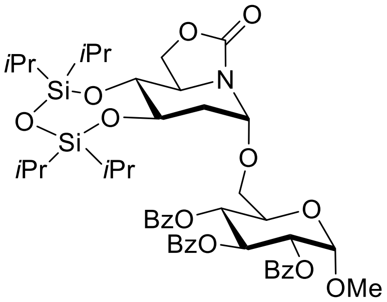

3e

^{1}H NMR (500 MHz, $CDCl_3$) δ 7.98 (dd, *J* = 8.5, 1.5 Hz, 2H), 7.94 (dd, *J* = 8.5, 1.0 Hz, 2H), 7.87 (dd, *J* = 8.5, 1.0 Hz, 2H), 7.55 – 7.48 (m, 2H), 7.46 – 7.35 (m, 5H), 7.30 (t, *J* = 7.5 Hz, 2H), 6.11 (t, *J* = 9.5 Hz, 1H), 5.63 (t, *J* = 10.0 Hz, 1H), 5.26 – 5.21 (m, 2H), 5.18 (d, *J* = 2.5 Hz, 1H), 4.25 – 4.04 (m, 4H), 3.66 (d, *J* = 3.5 Hz, 2H), 3.62 (td, *J* = 8.0, 5.5 Hz, 1H), 3.46 (s, 3H), 3.42 (t, *J* = 9.0 Hz, 1H), 2.31 – 2.25 (m, 1H), 1.68 (ddd, *J* = 14.5, 11.5, 4.0 Hz, 1H), 1.22 – 0.93 (m, 28H). **^{13}C NMR** (125 MHz, $CDCl_3$) δ 166.04, 165.91, 165.04, 156.57, 133.58, 133.54, 133.21, 130.09, 129.93, 129.85, 129.43, 129.17, 129.11, 128.65, 128.57, 128.38, 97.15, 80.03, 78.72, 72.32, 71.09, 70.75, 68.94, 68.31, 66.58, 65.79, 55.60, 54.15, 37.84, 17.75, 17.70, 17.52, 17.44, 17.42, 17.41, 17.36, 13.06, 12.92, 12.39, 12.28. **ESI-HRMS**: Calculated for $C_{47}H_{62}NO_{14}Si_2$ $(M+H)^+$: 920.3703, Found: 920.3724. $[\alpha]_D^{20}$ = +55.3 (c = 2.0, $CHCl_3$).

(5a*R*,7*R*,11a*R*,11b*R*)-2,2,4,4-tetraisopropyl-7-(((3a*S*,5a*R*,8a*R*,8b*S*)-2,2,7,7-tetramethyltetrahydro-3a*H*-bis([1,3]dioxolo)[4,5-b:4',5'-d]pyran-3a-yl)methoxy)hexahydro-9*H*-oxazolo[3,4-*a*][1,3,5,2,4]trioxadisilepino[6,7-*c*]pyridin-9-one (3f)

The title product compound is prepared according to above general procedure with iminoglycal **1a** (0.2 mmol, 1.0 equiv), 2 mol% catalyst **J**, dry CH_2Cl_2 (0.8 mL) and acceptor at room temperature for 1 h and isolated by flash column chromatography (3:1 Pentane: Ethyl Acetate) giving **3f** as a colorless oil (90 mg, 67% yield, α/β ratio > 20:1).

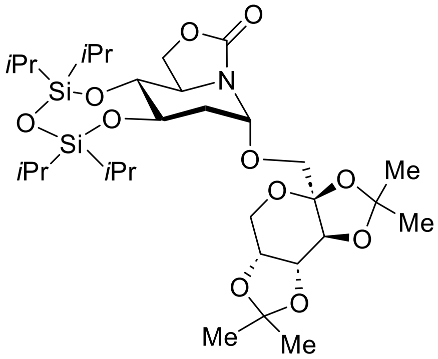

3f

^{1}H NMR (500 MHz, $CDCl_3$) δ 5.19 (d, *J* = 2.5 Hz, 1H), 4.62 (dd, *J* = 8.0, 3.0 Hz, 1H), 4.48 (t, *J* = 8.5 Hz, 1H), 4.41 (d, *J* = 2.5 Hz, 1H), 4.27 – 4.17 (m, 2H), 4.02 (ddd, *J* = 11.5, 8.0, 4.5 Hz, 1H), 3.90 (dd, *J* = 12.5, 1.5 Hz, 1H), 3.79 – 3.70 (m, 2H), 3.67 (d, *J* = 10.5 Hz, 1H), 3.55 – 3.41 (m, 1H), 2.25 (ddd, *J* = 13.5, 5.0, 1.5 Hz, 1H), 1.76 – 1.67 (m, 1H), 1.54 (s, 3H), 1.48 (s, 3H), 1.40 (s, 3H), 1.35 (s, 3H), 1.09 – 0.94 (m, 28H). **^{13}C NMR** (125 MHz, $CDCl_3$) δ 156.33, 109.35, 108.63, 102.13, 80.04, 78.58, 71.42, 71.11, 70.20, 69.91, 68.42, 66.59, 61.23, 54.07, 37.71, 26.67, 26.06, 25.81, 24.23, 17.72, 17.56, 17.52, 17.45, 17.34, 17.29, 17.27, 13.02, 12.93, 12.24, 12.10. **ESI-HRMS**: Calculated for $C_{31}H_{56}NO_{11}Si_2$ $(M+H)^+$: 674.3386, Found: 674.3396. $[\alpha]_D^{20}$ = +23.1 (c = 1.8, $CHCl_3$).

(5a*R*,7*R*,11a*R*,11b*R*)-2,2,4,4-tetraisopropyl-7-(((3a*R*,4*R*,6*R*,6a*R*)-6-methoxy-2,2-dimethyltetrahydrofuro[3,4-*d*][1,3]dioxol-4-yl)methoxy)hexahydro-9*H*-oxazolo[3,4-*a*][1,3,5,2,4]trioxadisilepino[6,7-*c*]pyridin-9-one (3g)

The title product compound is prepared according to above general procedure with iminoglycal **1a** (0.2 mmol, 1.0 equiv), 2 mol% catalyst **J**, dry CH_2Cl_2 (0.8 mL) and acceptor at room temperature for 1 h and isolated by flash column chromatography (3:1 Pentane: Ethyl Acetate) giving **3g** as a colorless oil (77 mg, 62% yield, α/β ratio > 20:1).

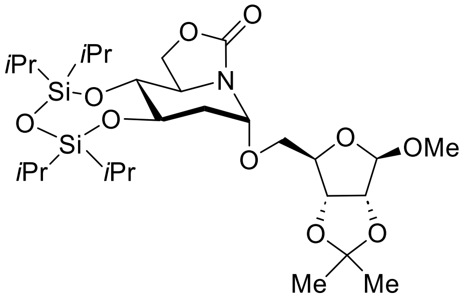

3g

^{1}H NMR (600 MHz, $CDCl_3$) δ 5.15 (d, *J* = 2.4Hz, 1H), 4.95 (s, 1H), 4.69 – 4.61 (m, 1H), 4.57 (d, *J* = 6.0 Hz, 1H), 4.48 (t, *J* = 8.4 Hz, 1H), 4.31 (ddd, *J* = 7.2, 6.0, 1.2 Hz, 1H), 4.22 (dd, *J* = 9.0, 5.6 Hz, 1H), 4.07 – 4.01 (m, 1H), 3.75 – 3.68 (m, 1H), 3.55 (dd, *J* = 10.2, 6.6 Hz, 1H), 3.46 (ddd, *J* = 9.6, 7.2, 6.6 Hz, 2H), 3.31 (s, 3H), 2.25 (ddd, *J* = 13.8, 4.8, 1.8 Hz, 1H), 1.68 (ddd, *J* = 13.8, 10.8, 3.6 Hz, 1H), 1.49 (s, 3H), 1.32(s, 3H), 1.11 – 0.98 (m, 28H). **^{13}C NMR** (150 MHz, $CDCl_3$) δ 156.43, 112.55, 109.63, 85.33, 85.01, 82.20, 80.30, 78.74, 71.34, 69.19, 66.70, 55.05, 54.39, 37.88, 26.60, 25.10, 17.73, 17.63, 17.53, 17.42, 17.37, 17.36, 17.35, 17.33, 13.07, 12.94, 12.40, 12.29. **ESI-HRMS**: Calculated for $C_{28}H_{52}NO_{10}Si_2$ $(M+H)^+$: 618.3124, Found: 618.3132. $[\alpha]_D^{20}$ = +12.1 (c = 0.7, $CHCl_3$).

(5a*R*,7*R*,11a*R*,11b*R*)-2,2,4,4-tetraisopropyl-7-(((3a*R*,4*R*,6*S*,7*S*,7a*R*)-4-methoxy-2,2,6-trimethyltetrahydro-4*H*-[1,3]dioxolo[4,5-*c*]pyran-7-yl)oxy)hexahydro-9*H*-oxazolo[3,4-*a*][1,3,5,2,4]trioxadisilepino[6,7-*c*]pyridin-9-one (3h)

The title product compound is prepared according to above general procedure with iminoglycal **1a** (0.2 mmol, 1.0 equiv), 2 mol% catalyst **J**, dry CH_2Cl_2 (0.8 mL) and acceptor at room temperature for 1 h and isolated by flash column chromatography (10:1 Pentane: Ethyl Acetate) giving **3h** as a white solid (88 mg, 72% yield, α/β ratio > 20:1).

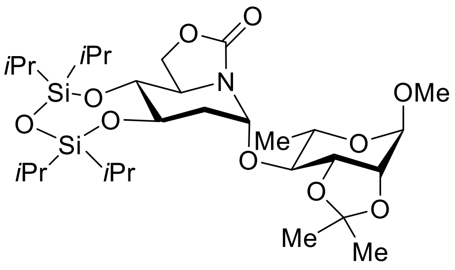

3h

^{1}H NMR (600 MHz, $CDCl_3$) δ 5.22 (dd, *J* = 4.2, 1.8 Hz, 1H), 4.84 (s, 1H), 4.43 (t, *J* = 8.4 Hz, 1H), 4.22 (dd, *J* = 9.0, 5.4 Hz, 1H), 4.10 (d, *J* = 5.4 Hz, 1H), 4.04 – 3.98 (m, 2H), 3.94 – 3.87 (m, 1H), 3.66 – 3.59 (m, 1H), 3.46 (t, *J* = 9.0 Hz, 1H), 3.36 (s, 3H), 3.23 (dd, *J* = 10.2, 7.2 Hz, 1H), 2.19 (ddd, *J* = 13.8, 5.4, 1.8 Hz, 1H), 1.67 (ddd, *J* = 13.8, 11.4, 4.2 Hz, 1H), 1.48 (s, 3H), 1.37 – 1.29 (m, 6H), 1.13 – 0.89 (m, 28H). **^{13}C NMR** (150 MHz, $CDCl_3$) δ 156.25, 109.44, 98.06, 80.49, 80.34, 78.97, 77.32, 75.99, 71.60, 66.53, 64.74, 54.99, 53.89, 38.11, 27.74, 26.48, 17.76, 17.69, 17.58, 17.47, 17.43, 17.40, 17.33, 13.03, 12.96, 12.44, 12.41. **ESI-HRMS**: Calculated for $C_{29}H_{54}NO_{10}Si_2$ $(M+H)^+$: 632.3281, Found: 632.3288. $[\alpha]_D^{20}$ = +27.8 (c = 1.3, $CHCl_3$).

(5a*R*,7*R*,11a*R*,11b*R*)-7-(((2*S*,6*S*,7*R*,8*S*)-8-(benzyloxy)-6-methoxy-2-phenylhexahydropyrano[3,2-*d*][1,3]dioxin-7-yl)oxy)-2,2,4,4-tetraisopropylhexahydro-9*H*-oxazolo[3,4-*a*][1,3,5,2,4]trioxadisilepino[6,7-c]pyridin-9-one (3i)

The title product compound is prepared according to above general procedure with iminoglycal **1a** (0.2 mmol, 1.0 equiv), 2 mol% catalyst **J**, dry CH_2Cl_2 (0.8 mL) and acceptor at room temperature for 1 h and isolated by flash column chromatography (5:1 Pentane: Ethyl Acetate) giving **3i** as a white solid (97 mg, 62% yield, α/β ratio > 20:1).

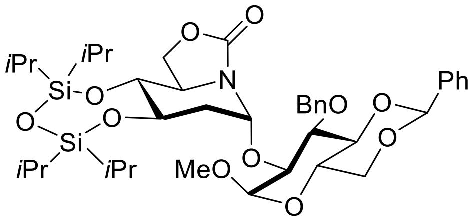

3i

^{1}H NMR (500 MHz, $CDCl_3$) δ 7.49 – 7.44 (m, 2H), 7.40 – 7.30 (m, 5H), 7.28 – 7.24 (m, 3H), 5.57 (s, 1H), 5.26 (d, *J* = 2.5 Hz, 1H), 5.02 (d, *J* = 3.5 Hz, 1H), 4.89 (d, *J* = 11.0 Hz, 1H), 4.70 (d, *J* = 10.5 Hz, 1H), 4.29 (dd, *J* = 10.0, 4.5 Hz, 1H), 4.15 – 4.07 (m, 1H), 4.06 – 4.01 (m, 2H), 3.92 (t, *J* = 9.5 Hz, 1H), 3.85 – 3.79 (m, 1H), 3.78 – 3.70 (m, 2H), 3.67 – 3.59 (m, 2H), 3.46 (s, 3H), 3.44 – 3.38 (m, 1H), 2.34 (ddd, *J* = 14.0, 5.0, 1.5 Hz, 1H), 1.72 (ddd, *J* = 13.5, 11.5, 4.0 Hz, 1H), 1.11 – 0.98 (m, 28H). **^{13}C NMR** (125 MHz, $CDCl_3$) δ 156.80, 138.43, 137.46, 129.07, 128.50, 128.37, 128.16, 127.88, 126.14, 101.42, 97.48, 82.24, 78.65, 77.36, 77.07, 75.63, 75.40, 71.58, 69.15, 66.56, 62.38, 55.47, 53.88, 37.86, 17.71, 17.70, 17.54, 17.46, 17.42, 17.38, 17.31, 13.00, 12.94, 12.34, 12.32. **ESI-HRMS**: Calculated for $C_{40}H_{60}NO_{11}Si_2$ $(M+H)^+$: 786.3699, Found: 786.3714. $[\alpha]_D^{20}$ = +46.7 (c = 1.1, $CHCl_3$).

(5a*R*,7*R*,11a*R*,11b*R*)-7-(((2*S*,6*S*,7*R*,8*S*)-7-(benzyloxy)-6-methoxy-2-phenylhexahydropyrano[3,2-*d*][1,3]dioxin-8-yl)oxy)-2,2,4,4-tetraisopropylhexahydro-9*H*-oxazolo[3,4-*a*][1,3,5,2,4]trioxadisilepino[6,7-*c*]pyridin-9-one (3j)

The title product compound is prepared according to above general procedure with iminoglycal **1a** (0.2 mmol, 1.0 equiv), 2 mol% catalyst **J**, dry CH_2Cl_2 (0.8 mL) and acceptor at room temperature for 1 h and isolated by flash column chromatography (3:1 Pentane: Ethyl Acetate) giving **3j** as a white solid (110 mg, 70% yield, α/β ratio > 20:1).

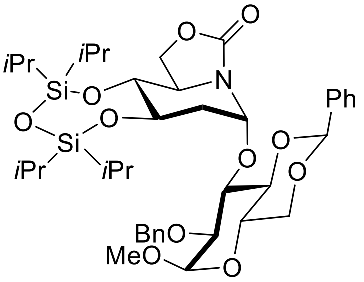

3j

^{1}H NMR (600 MHz, $CDCl_3$) δ 7.54 (d, *J* = 6.6 Hz, 1H), 7.41 – 7.30 (m, 8H), 5.60 (dd, *J* = 4.2, 1.8 Hz, 1H), 5.53 (s, 1H), 4.71 (d, *J* = 12.0 Hz, 1H), 4.64 – 4.57 (m, 2H), 4.23 (dd, *J* = 10.2, 4.8 Hz, 1H), 4.14 – 4.06 (m, 2H), 4.04 – 4.01 (m, 2H), 3.84 – 3.78 (m, 2H), 3.69 (t, *J* = 10.2 Hz, 1H), 3.57 (t, *J* = 9.6 Hz, 1H), 3.44 (dd, *J* = 9.6, 3.6 Hz, 1H), 3.41 – 3.37 (m, 4H), 2.22 (ddd, *J* = 13.8, 4.8, 1.8 Hz, 1H), 1.62 (ddd, *J* = 14.4, 12.0, 4.2 Hz, 1H), 1.15 – 0.95 (m, 28H). **^{13}C NMR** (150 MHz, $CDCl_3$) δ 156.39, 137.83, 137.33, 129.14, 128.74, 128.74, 128.44, 128.42, 128.34, 126.38, 101.68, 98.83, 82.53, 80.68, 78.49, 77.83, 74.06, 73.46, 71.63, 69.18, 66.16, 62.11, 55.38, 54.21, 38.05, 17.84, 17.69, 17.51, 17.49, 17.42, 17.38, 17.37, 17.32, 13.08, 13.02, 12.40, 12.23. **ESI-HRMS**: Calculated for $C_{40}H_{60}NO_{11}Si_2$ $(M+H)^+$: 786.3699, Found: 786.3716. $[\alpha]_D^{20}$ = +44.1 (c = 1.2, $CHCl_3$).

(2*S*,3*R*,4*S*,5*S*,6*R*)-6-((benzoyloxy)methyl)-2-methoxy-5-(((5a*R*,7*R*,11a*R*,11b*R*)-2,2,4,4-tetraisopropyl-9-oxohexahydro-9*H*-oxazolo[3,4-*a*][1,3,5,2,4]trioxadisilepino[6,7-c]pyridin-7-yl)oxy)tetrahydro-2*H*-pyran-3,4-diyl dibenzoate (3k)

The title product compound is prepared according to above general procedure with iminoglycal **1a** (0.2 mmol, 1.0 equiv), 2 mol% catalyst **J**, dry CH_2Cl_2 (0.8 mL) and acceptor at room temperature for 3 h and isolated by flash column chromatography (3:1 Pentane: Ethyl Acetate) giving **3k** as a white solid (118 mg, 64% yield, α/β ratio > 20:1).

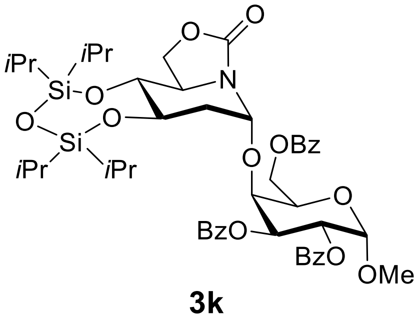

3k

^{1}H NMR (600 MHz, $CDCl_3$) δ 8.19 – 8.14 (m, 2H), 8.01 – 7.96 (m, 4H), 7.60 – 7.45 (m, 5H), 7.40 – 7.32 (m, 4H), 5.71 – 5.58 (m, 2H). 5.27 (dd, *J* = 4.8, 1.5 Hz, 1H), 5.19 (d, *J* = 3.6 Hz, 1H), 4.81 – 4.73 (m, 1H), 4.61 (d, *J* = 3.6 Hz, 1H), 4.41 – 4.34 (m, 2H), 4.19 – 4.09 (m, 1H), 3.69 – 3.58 (m, 2H), 3.44 (s, 3H), 3.27 (t, *J* = 9.0 Hz, 1H), 2.80 (t, *J* = 7.8 Hz,1H), 2.38 (ddd, *J* = 13.8, 4.8, 1.2 Hz, 1H), 1.68 (ddd, *J* = 13.2, 11.4, 4.2 Hz, 1H), 1.18 – 0.93 (m, 28H). **^{13}C NMR** (150 MHz, $CDCl_3$) δ 166.30, 166.14, 165.72, 156.31, 133.58, 133.51, 133.44, 130.11, 129.91, 129.54, 129.53, 129.45, 128.71, 128.68, 128.57, 97.64, 81.14, 78.96, 74.61, 71.09, 70.14, 68.78, 67.88, 66.40, 62.24, 55.71, 54.35, 37.90, 17.72, 17.63, 17.46,

17.44, 17.43, 17.41, 17.40, 17.32, 13.02, 12.95, 12.45, 12.42. **ESI-HRMS**: Calculated for $C_{47}H_{62}NO_{14}Si_2$ $(M+H)^+$: 920.3703, Found: 920.3724. $[\alpha]_D^{20}$ = +102.5 (c = 1.4, $CHCl_3$).

(5a*R*,7*R*,11a*R*,11b*R*)-2,2,4,4-tetraisopropyl-7-methoxyhexahydro-9*H*-oxazolo[3,4-*a*][1,3,5,2,4]trioxadisilepino[6,7-*c*]pyridin-9-one (3l)

The title product compound is prepared according to above general procedure with iminoglycal **1a** (0.2 mmol, 1.0 equiv), 2 mol% catalyst **J**, dry CH_2Cl_2 (0.8 mL) and acceptor at room temperature for 70 h and isolated by flash column chromatography (20:1 Pentane: Ethyl Acetate) giving **3l** as a white solid (76 mg, 86% yield, α/β ratio > 20:1).

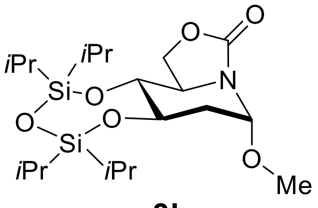

3l

^{1}H NMR (700 MHz, $CDCl_3$) δ 5.04 (dd, *J* = 4.2, 1.4 Hz, 1H), 4.50 (t, *J* = 8.4 Hz, 1H), 4.22 (dd, *J* = 9.1, 5.6 Hz, 1H), 4.01 (ddd, *J* = 11.2, 8.4, 4.9 Hz, 1H), 3.70 (ddd, *J* = 9.1, 8.4, 5.6 Hz, 1H), 3.50 – 3.44 (m, 1H), 3.31 (s, 3H), 2.20 (ddd, *J* = 14.0, 4.9, 1.4 Hz, 1H), 1.68 (ddd, *J* = 14.0, 11.2, 4.2 Hz, 1H), 1.11 – 0.94 (m, 28H). **^{13}C NMR** (175 MHz, $CDCl_3$) δ 156.65, 81.16, 78.89, 71.35, 66.72, 55.59, 54.25, 37.92, 17.73, 17.64, 17.53, 17.44, 17.35, 17.33, 13.08, 12.92, 12.35, 12.26. **ESI-HRMS**: Calculated for $C_{20}H_{40}NO_6Si_2$ $(M+H)^+$: 446.2389, Found: 446.2395. $[\alpha]_D^{20}$ = +47.9 (c = 1.0, $CHCl_3$).

(5a*R*,7*R*,11a*R*,11b*R*)-2,2,4,4-tetraisopropyl-7-(prop-2-yn-1-yloxy)hexahydro-9*H*-oxazolo[3,4-*a*][1,3,5,2,4]trioxadisilepino[6,7-c]pyridin-9-one (3m)

The title product compound is prepared according to above general procedure with iminoglycal **1a** (0.2 mmol, 1.0 equiv), 2 mol% catalyst **J**, dry CH_2Cl_2 (0.8 mL) and acceptor at room temperature for 3 h and isolated by flash column chromatography (20:1 Pentane: Ethyl Acetate) giving **3m** as a white solid (66 mg, 70% yield, α/β ratio > 20:1).

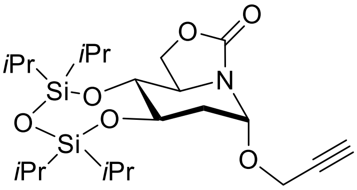

3m

^{1}H NMR (600 MHz, $CDCl_3$) δ 5.31 (dd, *J* = 4.2, 1.8 Hz, 1H), 4.48 (t, *J* = 8.4 Hz, 1H), 4.25 – 4.13 (m, 3H), 4.03 (ddd, *J* = 10.8, 8.4, 4.8 Hz, 1H), 3.77 (ddd, *J* = 9.0, 8.4, 5.4 Hz, 1H), 3.48 (t, *J* = 9.0 Hz, 1H), 2.45 (t, *J* = 2.4 Hz, 1H), 2.24 (ddd, *J* = 13.8, 4.8, 1.8 Hz, 1H), 1.72 (ddd, *J* = 13.8, 11.4, 4.2 Hz, 1H), 1.11 – 0.94 (m, 28H). **^{13}C NMR** (150 MHz, $CDCl_3$) δ 156.44, 79.76, 79.45, 78.80, 74.52, 71.29, 66.70, 55.81, 54.40, 37.90, 17.73, 17.64, 17.53, 17.43, 17.35, 17.33, 13.05, 12.92, 12.34, 12.27. **ESI-HRMS**: Calculated for $C_{22}H_{40}NO_6Si_2$ $(M+H)^+$: 470.2389, Found: 470.2388. $[\alpha]_D^{20}$ = +71.3 (c = 1.0, $CHCl_3$).

(5a*R*,7*R*,11a*R*,11b*R*)-2,2,4,4-tetraisopropyl-7-(neopentyloxy)hexahydro-9*H*-oxazolo[3,4-*a*][1,3,5,2,4]trioxadisilepino[6,7-*c*]pyridin-9-one (3n)

The title product compound is prepared according to above general procedure with iminoglycal **1a** (0.2 mmol, 1.0 equiv), 2 mol% catalyst **J**, dry CH_2Cl_2 (0.8 mL) and acceptor at room temperature for 21 h and isolated by flash column chromatography (20:1 Pentane: Ethyl Acetate) giving **3n** as a white solid (90 mg, 90% yield, α/β ratio > 20:1).

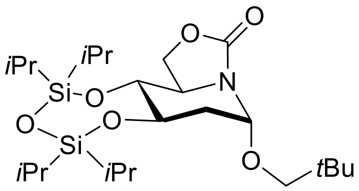

3n

^{1}H NMR (600 MHz, $CDCl_3$) δ 5.09 (dd, *J* = 3.6, 1.8 Hz, 1H), 4.48 (t, *J* = 9.0 Hz, 1H), 4.20 (dd, *J* = 9.0, 6.0 Hz, 1H), 4.06 (ddd, *J* = 11.4, 8.4, 4.8 Hz, 1H), 3.66 (td, *J* = 9.0, 5.4 Hz, 1H), 3.47 (t, *J* = 9.0 Hz, 1H), 3.17 – 3.03 (m, 2H), 2.22 (ddd, *J* = 13.8, 4.8, 1.8 Hz, 1H), 1.67 (ddd, *J* = 13.8, 11.4, 3.6 Hz, 1H), 1.13 – 0.95 (m, 28H), 0.90 (s, 9H). **^{13}C NMR** (150 MHz, $CDCl_3$) δ 156.55, 80.06, 78.93, 78.13, 71.44, 66.74, 54.65, 38.06, 31.89, 26.73, 17.71, 17.52, 17.50, 17.41, 17.37, 17.31, 17.29, 13.11, 12.94, 12.46, 12.38. **ESI-HRMS**: Calculated for $C_{24}H_{48}NO_6Si_2$ $(M+H)^+$: 502.3015, Found:502.3028. $[\alpha]_D^{20}$ = +46.5 (c = 1.0, $CHCl_3$).

(5a*R*,7*R*,11a*R*,11b*R*)-7-((4-bromobenzyl)oxy)-2,2,4,4-tetraisopropylhexahydro-9*H*-oxazolo[3,4-*a*][1,3,5,2,4]trioxadisilepino[6,7-*c*]pyridin-9-one (3o)

The title product compound is prepared according to above general procedure with iminoglycal **1a** (0.2 mmol, 1.0 equiv), 2 mol% catalyst **J**, dry CH_2Cl_2 (0.8 mL) and acceptor at room temperature for 3 h and isolated by flash column chromatography (3:1 Pentane: Ethyl Acetate) giving **3o** as a colorless oil (95 mg, 79% yield, α/β ratio > 20:1).

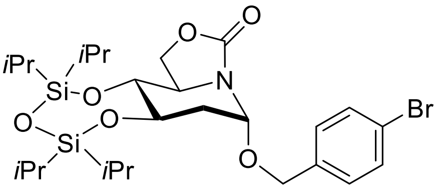

3o

^{1}H NMR (600 MHz, $CDCl_3$) δ 7.48 (d, *J* = 8.4 Hz, 1H), 7.22 (d, *J* = 8.4 Hz, 2H), 5.22 (dd, *J* = 4.2, 1.8 Hz, 1H), 4.56 – 4.36 (m, 3H), 4.20 (dd, *J* = 9.0, 5.4 Hz, 1H), 4.11 – 4.00 (m, 1H), 3.70 – 3.62 (m, 1H), 3.47 (t, *J* = 9.0 Hz, 1H), 2.23 (ddd, *J* = 13.8, 4.8, 1.8 Hz, 1H), 1.70 (ddd, *J* = 13.8, 10.8, 3.6 Hz, 1H), 1.12 – 0.93 (m, 28H). **^{13}C NMR** (150 MHz, $CDCl_3$) δ 156.48, 136.63, 131.72, 131.71, 129.50, 121.91, 79.36, 78.86, 71.39, 69.15, 66.73, 54.41, 38.04, 17.72, 17.65, 17.52, 17.42, 17.36, 17.32, 13.06, 12.94, 12.38, 12.30. **ESI-HRMS**: Calculated for $C_{26}H_{43}BrNO_6Si_2$ $(M+H)^+$: 600.1807, Found: 600.1813. $[\alpha]_D^{20}$ = +33.2 (c = 1.1, $CHCl_3$).

(5a*R*,7*R*,11a*R*,11b*R*)-7-isopropoxy-2,2,4,4-tetraisopropylhexahydro-9*H*-oxazolo[3,4-*a*][1,3,5,2,4]trioxadisilepino[6,7-*c*]pyridin-9-one (3p)

The title product compound is prepared according to above general procedure with iminoglycal **1a** (0.2 mmol, 1.0 equiv), 2 mol% catalyst **J**, dry CH_2Cl_2 (0.8 mL) and acceptor at room temperature for 48 h and isolated by flash column chromatography (20:1 Pentane: Ethyl Acetate) giving **3p** as a white solid (72 mg, 76% yield, α/β ratio > 20:1).

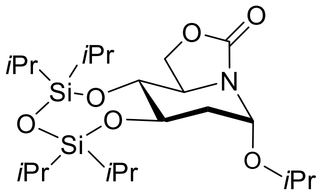

3p

^{1}H NMR (700 MHz, $CDCl_3$) δ 5.23 (dd, *J* = 4.2, 1.4 Hz, 1H), 4.46 (t, *J* = 8.4 Hz, 1H), 4.21 (dd, *J* = 9.1, 5.6 Hz, 1H), 4.04 (ddd, *J* = 11.2, 8.4, 4.9 Hz, 1H), 3.74 (p, *J* = 6.3 Hz, 1H), 3.69 (ddd, *J* = 9.8, 8.4, 5.6 Hz, 1H), 3.46 (t, *J* = 9.1 Hz, 1H), 2.13 (ddd, *J* = 13.3, 4.9, 1.4 Hz, 1H), 1.67 (ddd, *J* = 15.4, 11.2, 4.2 Hz, 1H), 1.18 (d, *J* = 6.3 Hz, 3H), 1.15 (d, *J* = 6.3 Hz, 3H), 1.10 – 0.95 (m, 28H). **^{13}C NMR** (175 MHz, $CDCl_3$) δ 156.44, 78.95, 71.48, 69.00, 66.59, 54.45, 38.53, 23.39, 21.37, 17.74, 17.63, 17.55, 17.44, 17.38, 17.34, 13.08, 12.96, 12.43, 12.36. **ESI-HRMS**: Calculated for $C_{22}H_{44}NO_6Si_2$ $(M+H)^+$: 474.2701, Found:474.2708. $[\alpha]_D^{20}$ = +56.9 (c = 1.3, $CHCl_3$).

(5a*R*,7*R*,11a*R*,11b*R*)-7-(cyclohexyloxy)-2,2,4,4-tetraisopropylhexahydro-9*H*-oxazolo[3,4-*a*][1,3,5,2,4]trioxadisilepino[6,7-*c*]pyridin-9-one (3q)

The title product compound is prepared according to above general procedure with iminoglycal **1a** (0.2 mmol, 1.0 equiv), 2 mol% catalyst **J**, dry CH_2Cl_2 (0.8 mL) and acceptor at room temperature for 60 h and isolated by flash column chromatography (20:1 Pentane: Ethyl Acetate) giving **3q** as a white solid (84 mg, 82% yield, α/β ratio > 20:1).

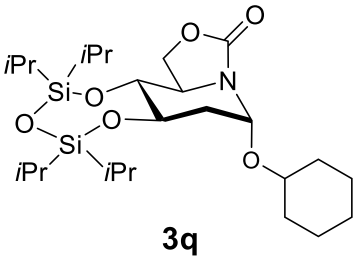

3q

^{1}H NMR (600 MHz, $CDCl_3$) δ 5.27 (d, *J* = 2.4 Hz, 1H), 4.46 (t, *J* = 8.4 Hz, 1H), 4.20 (dd, *J* = 9.0, 5.4 Hz, 1H), 4.06 (ddd, *J* = 11.4, 8.4, 4.8 Hz, 1H), 3.70 (td, *J* = 9.0, 5.4 Hz, 1H), 3.49 – 3.37 (m, 2H), 2.14 (ddd, *J* = 13.2, 4.8, 1.2 Hz, 1H), 1.93 (d, *J* = 10.2 Hz, 1H), 1.77 – 1.63 (m, 4H), 1.56 – 1.44 (m, 1H), 1.39 – 1.19 (m, 5H), 1.12 – 0.91 (m, 28H). **^{13}C NMR** (150 MHz, $CDCl_3$) δ 156.39, 78.91, 77.18, 71.47, 66.57, 54.53, 38.58, 33.44, 31.33, 25.79, 24.16, 23.89, 22.48, 17.72, 17.61, 17.53, 17.43, 17.38, 17.36, 17.33, 13.07, 12.96, 12.42, 12.34. **ESI-HRMS**: Calculated for $C_{25}H_{48}NO_6Si_2$ $(M+H)^+$: 514.3015, Found: 514.3017. $[\alpha]_D^{20}$ = +55.9 (c = 1.4, $CHCl_3$).

(5a*R*,7*R*,11a*R*,11b*R*)-7-(((1*R*,3*S*,5*R*,7*S*)-adamantan-2-yl)oxy)-2,2,4,4-tetraisopropylhexahydro-9*H*-oxazolo[3,4-*a*][1,3,5,2,4]trioxadisilepino[6,7-*c*]pyridin-9-one (3r)

The title product compound is prepared according to above general procedure with iminoglycal **1a** (0.2 mmol, 1.0 equiv), 2 mol% catalyst **J**, dry CH_2Cl_2 (0.8 mL) and acceptor at room temperature for 23 h and isolated by flash column chromatography (20:1 Pentane: Ethyl Acetate) giving **3r** as a colorless oil (102 mg, 90% yield, α/β ratio > 20:1).

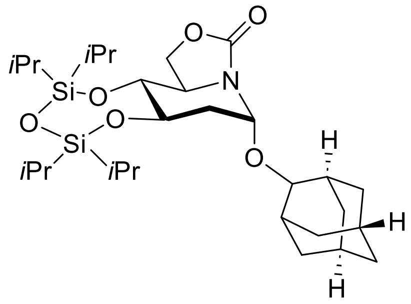

3r

^{1}H NMR (600 MHz, $CDCl_3$) δ 5.28 (dd, *J* = 3.6, 1.8 Hz, 1H), 4.46 (t, *J* = 8.4 Hz, 1H), 4.20 (dd, *J* = 9.0, 5.4 Hz, 1H), 4.16 – 4.08 (m, 1H), 3.71 (ddd, *J* = 9.0, 8.4, 5.4 Hz, 1H), 3.60 (t, *J* = 3.6 Hz, 1H), 3.47 (t, *J* = 9.0 Hz, 1H), 2.19 (ddd, *J* = 13.8, 4.8, 1.8 Hz, 1H), 2.10 (d, *J* = 3.6 Hz, 1H), 2.03 – 1.94 (m, 2H), 1.86 – 1.75 (m, 5H), 1.72 – 1.61 (m, 5H), 1.52 – 1.46 (m, 2H), 1.12 – 0.91 (m, 28H). **^{13}C NMR** (150 MHz, $CDCl_3$) δ 156.39, 79.07, 78.91, 77.22, 71.49, 66.61, 54.76, 38.72, 37.62, 36.75, 36.32, 33.64, 31.91, 31.58, 30.86, 27.56, 27.32, 17.71, 17.53, 17.51, 17.43, 17.39, 17.33, 17.30, 13.10, 12.97, 12.45, 12.36. **ESI-HRMS**: Calculated for $C_{29}H_{52}NO_6Si_2$ $(M+H)^+$: 566.3328, Found:566.3333. $[\alpha]_D^{20}$ = +44.9 (c = 3.8, $CHCl_3$).

(5a*R*,7*R*,11a*R*,11b*R*)-7-(((3*S*,5*S*,7*S*)-adamantan-1-yl)oxy)-2,2,4,4-tetraisopropylhexahydro-9*H*-oxazolo[3,4-*a*][1,3,5,2,4]trioxadisilepino[6,7-*c*]pyridin-9-one (3s)

The title product compound is prepared according to above general procedure with iminoglycal **1a** (0.2 mmol, 1.0 equiv), 2 mol% catalyst **J**, dry CH_2Cl_2 (0.8 mL) and acceptor at room temperature for 24 h and isolated by flash column chromatography (3:1 Pentane: Ethyl Acetate) giving **3s** as a white solid (51 mg, 45% yield, α/β ratio > 20:1).

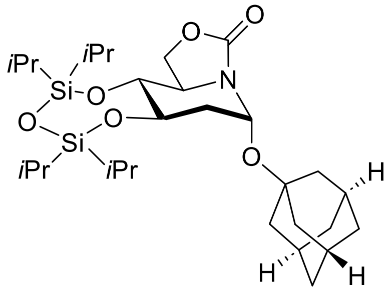

3s

^{1}H NMR (600 MHz, $CDCl_3$) δ 5.47 (dd, *J* = 3.6, 1.8 Hz, 1H), 4.40 (t, *J* = 8.4 Hz, 1H), 4.21 (dd, *J* = 9.0, 4.8 Hz, 1H), 4.10 (ddd, *J* = 10.8, 8.4, 4.2 Hz, 1H), 3.78 (td, *J* = 8.4, 4.2 Hz, 1H), 3.43 (t, *J* = 9.0 Hz, 1H), 2.13 (s, 3H), 2.00 (ddd, *J* = 13.8, 4.8, 1.8 Hz, 1H), 1.77 (q, *J* = 11.4 Hz, 6H), 1.67 – 1.57 (m, 7H), 1.12 – 0.94 (m, 28H). **^{13}C NMR** (150 MHz, $CDCl_3$) δ 155.13, 78.76, 74.40, 72.65, 71.46, 66.20, 54.64, 42.22, 40.18, 36.36, 30.69, 17.74, 17.58, 17.56, 17.45, 17.41, 17.36, 13.09, 12.99, 12.46, 12.34. **ESI-HRMS**: Calculated for $C_{29}H_{51}NO_6Si_2Na$ $(M+Na)^+$: 588.3147, Found: 588.3144. $[\alpha]_D^{20}$ = +21.5 (c = 1.0, $CHCl_3$).

(5a*R*,7*R*,11a*R*,11b*R*)-7-(((8*R*,9*S*,10*R*,13*S*,14*S*,17*S*)-10,13-dimethyl-3-oxo-2,3,6,7,8,9,10,11,12,13,14,15,16,17-tetradecahydro-1*H*-cyclopenta[a]phenanthren-17-yl)oxy)-2,2,4,4-tetraisopropylhexahydro-9*H*-oxazolo[3,4-*a*][1,3,5,2,4]trioxadisilepino[6,7-*c*]pyridin-9-one (3t)

The title product compound is prepared according to above general procedure with iminoglycal **1a** (0.2 mmol, 1.0 equiv), 2 mol% catalyst **J**, dry CH_2Cl_2 (0.8 mL) and acceptor at room temperature for 23 h and isolated by flash column chromatography (3:1 Pentane: Ethyl Acetate) giving **3t** as a white solid (125 mg, 89% yield, α/β ratio > 20:1).

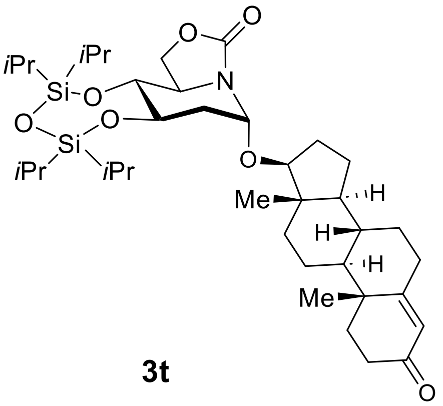

3t

^{1}H NMR (600 MHz, CD_2Cl_2) δ 5.67 – 5.62 (m, 1H), 5.10 (dd, *J* = 3.6, 1.8 Hz, 1H), 4.44 (t, *J* = 8.4 Hz, 1H), 4.17 (dd, *J* = 9.0, 6.0 Hz, 1H), 4.09 – 4.02 (m, 1H), 3.79 (d, *J* = 10.2 Hz, 1H), 3.64 (td, *J* = 9.0, 6.0 Hz, 1H), 3.47 (t, *J* = 9.0 Hz, 1H), 3.38 (t, *J* = 7.8 Hz, 1H), 2.42 – 2.33 (m, 2H), 2.28 – 2.20 (m, 2H), 2.17 – 2.07 (m, 2H), 2.03 – 1.97 (m, 1H), 1.85 – 1.79 (m, 1H), 1.78 – 1.72 (m, 1H), 1.70 – 1.60 (m, 3H), 1.59 – 1.55 (m, 2H), 1.52 – 1.39 (m, 2H), 1.38 – 1.28 (m, 1H), 1.17 (s, 3H), 1.12 – 0.98 (m, 30H), 0.78 (s, 3H). **^{13}C NMR** (150 MHz, CD_2Cl_2) δ 199.41, 171.59, 156.72, 124.20, 84.94, 79.38, 78.41, 71.90, 67.12, 54.94, 54.56, 54.40, 50.91, 42.87, 39.18, 38.70, 37.36, 36.34, 35.90, 34.51, 33.29, 32.11, 27.48, 23.92, 21.18, 17.91, 17.77, 17.71, 17.60, 17.57, 17.57, 17.54, 17.50, 13.54, 13.40, 12.90, 12.86, 11.91. **ESI-HRMS**: Calculated for $C_{38}H_{64}NO_7Si_2$ $(M+H)^+$: 702.4216, Found: 702.4228. $[\alpha]_D^{20}$ = +90.3 (c = 1.8, $CHCl_3$).

(5a*R*,7*R*,11a*R*,11b*R*)-2,2,4,4-tetraisopropyl-7-(((1*R*,2*S*,5*R*)-2-isopropyl-5-methylcyclohexyl)oxy)hexahydro-9*H*-oxazolo[3,4-*a*][1,3,5,2,4]trioxadisilepino[6,7-*c*]pyridin-9-one (3u)

The title product compound is prepared according to above general procedure with iminoglycal **1a** (0.2 mmol, 1.0 equiv), 2 mol% catalyst **J**, dry CH_2Cl_2 (0.8 mL) and acceptor at room temperature for 23 h and isolated by flash column chromatography (3:1 Pentane: Ethyl Acetate) giving **3u** as a colorless oil (92 mg, 81% yield, α/β ratio > 20:1).

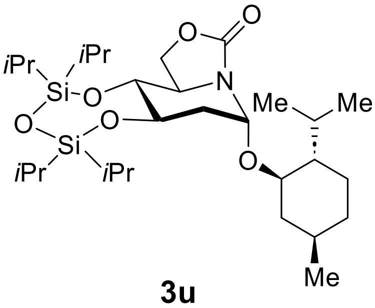

3u

^{1}H NMR (600 MHz, $CDCl_3$) δ 5.17 (dd, *J* = 4.2, 1.8 Hz, 1H), 4.46 (t, *J* = 8.4 Hz, 1H), 4.19 (dd, *J* = 9.0, 6.0 Hz, 1H), 4.04 (ddd, *J* = 10.8, 8.4, 4.2 Hz, 1H), 3.77 (ddd, *J* = 9.6, 8.4, 5.4 Hz, 1H), 3.45 (t, *J* = 9.0 Hz, 1H), 3.32 (td, *J* = 10.8, 4.2 Hz, 1H), 2.19 (ddd, *J* = 13.2, 4.8, 1.8 Hz, 1H), 2.08 (td, *J* = 7.2, 3.0 Hz, 1H), 1.93 – 1.86 (m, 1H), 1.67 – 1.56 (m, 3H), 1.41 – 1.34 (m, 1H), 1.22 – 1.16 (m, 1H), 1.12 – 0.92 (m, 31H), 0.90 (dd, *J* = 13.8, 7.2 Hz, 6H), 0.82 (d, *J* = 6.6 Hz, 3H). **^{13}C NMR** (150 MHz, $CDCl_3$) δ 155.95, 80.64, 80.12, 78.91, 71.50, 66.52, 54.68, 48.88, 42.64, 38.56, 34.45, 31.64, 26.03, 23.38, 22.46, 21.25, 17.71, 17.52, 17.44, 17.36, 17.33, 17.31, 16.55, 13.04, 12.93, 12.39, 12.30. **ESI-HRMS**: Calculated for $C_{29}H_{56}NO_6Si_2$ $(M+H)^+$: 570.3641, Found: 570.3644. $[\alpha]_D^{20}$ = +10.1 (c = 1.2, $CHCl_3$).

***tert*-butyl *N*-(((9*H*-fluoren-9-yl)methoxy)carbonyl)-*O*-((5a*R*,7*R*,11a*R*,11b*R*)-2,2,4,4-tetraisopropyl-9-oxohexahydro-9*H*-oxazolo[3,4-*a*][1,3,5,2,4]trioxadisilepino[6,7-*c*]pyridin-7-yl)-*L*-serinate (3v)**

The title product compound is prepared according to above general procedure with iminoglycal **1a** (0.2 mmol, 1.0 equiv), 2 mol% catalyst **J**, dry CH_2Cl_2 (0.8 mL) and acceptor at room temperature for 4 h and

isolated by flash column chromatography (3:1 Pentane: Ethyl Acetate) giving **3v** as a colorless oil (77.2 mg, 48% yield, α/β ratio > 20:1).

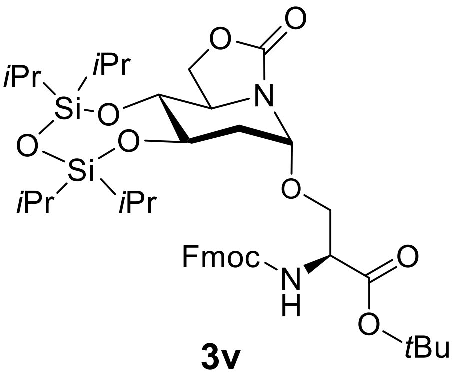

3v

^{1}H NMR (600 MHz, $CDCl_3$) δ 7.83 – 7.73 (m, 2H), 7.65 – 7.54 (m, 2H), 7.43 – 7.38 (m, 2H), 7.34 – 7.29 (m, 2H), 5.55 (d, J = 8.0 Hz, 1H), 5.18 – 5.11 (m, 1H), 4.51 – 4.39 (m, 3H), 4.34 (dd, J = 10.7, 7.3 Hz, 1H), 4.26 – 4.17 (m, 2H), 3.99 (ddd, J = 12.3, 8.3, 4.7 Hz, 1H), 3.88 (dd, J = 10.0, 3.5 Hz, 1H), 3.80 (dd, J = 10.0, 3.3 Hz, 1H), 3.67 (td, J = 8.8, 5.5 Hz, 1H), 3.45 (t, J = 8.9 Hz, 1H), 2.19 – 2.10 (m, 1H), 1.68 (ddd, J = 13.9, 11.3, 4.1 Hz, 1H), 1.49 (s, 9H), 1.12 – 0.95 (m, 28H). **^{13}C NMR** (150 MHz, $CDCl_3$) δ 169.09, 156.40, 155.92, 144.04, 143.87, 141.45, 127.89, 127.24, 127.20, 125.24, 125.19, 120.17, 120.15, 82.83, 80.62, 78.61, 71.22, 68.91, 67.36, 66.69, 54.85, 54.43, 47.27, 37.82, 28.14, 17.72, 17.59, 17.52, 17.42, 17.37, 17.31, 13.05, 12.93, 12.36, 12.32. **ESI-HRMS**: Calculated for $C_{41}H_{60}N_2O_{10}Si_2Na$ $(M+H)^+$: 819.3679, Found: 819.3679. $[\alpha]_D^{20}$ = +25.3 (c = 1.0, $CHCl_3$).

tert-butyl _N_-(((9_H_-fluoren-9-yl)methoxy)carbonyl)-_O_-((5a_R_,7_R_,11a_R_,11b_R_)-2,2,4,4-tetraisopropyl-9-oxohexahydro-9_H_-oxazolo[3,4-_a_][1,3,5,2,4]trioxadisilepino[6,7-_c_]pyridin-7-yl)-_L_-threoninate (3w)

The title product compound is prepared according to above general procedure with iminoglycal **1a** (0.2 mmol, 1.0 equiv), 2 mol% catalyst **J**, dry CH_2Cl_2 (0.8 mL) and acceptor at room temperature for 4 h and isolated by flash column chromatography (3:1 Pentane: Ethyl Acetate) giving **3w** as a colorless oil (73 mg, 45% yield, α/β ratio > 20:1).

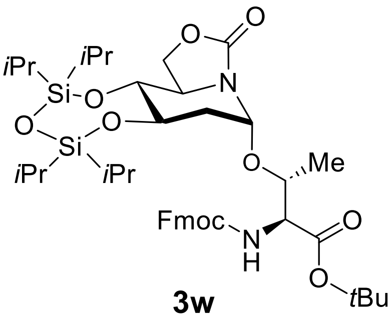

3w

^{1}H NMR (600 MHz, $CDCl_3$) δ 7.77 (dd, J = 7.6, 2.3 Hz, 2H), 7.62 (t, J = 7.0 Hz, 2H), 7.43 – 7.38 (m, 2H), 7.36 – 7.29 (m, 2H), 5.36 (d, J = 9.7 Hz, 1H), 5.21 (dd, J = 4.3, 1.6 Hz, 1H), 4.51 – 4.36 (m, 3H), 4.35 – 4.30 (m, 1H), 4.29 – 4.19 (m, 3H), 4.01 (ddd, J = 11.3, 8.4, 4.7 Hz, 1H), 3.70 (td, J = 8.8, 5.5 Hz, 1H), 3.46 (t, J = 8.9 Hz, 1H), 2.14 (ddd, J = 13.9, 4.8, 1.6 Hz, 1H), 1.63 (ddd, J = 13.8, 11.3, 4.2 Hz, 1H), 1.52 (s, 9H), 1.21 (d, J = 6.4 Hz, 3H), 1.13 – 0.98 (m, 28H). **^{13}C NMR** (150 MHz, $CDCl_3$) δ 169.57, 156.73, 156.12, 144.12, 143.89, 141.46, 127.88, 127.22, 127.18, 125.27, 120.15, 120.13, 82.88, 81.08, 78.67, 75.86, 71.27, 67.37, 66.65, 59.24, 54.56, 47.36, 38.17, 28.16, 18.87, 17.72, 17.66, 17.53, 17.40, 17.38, 17.32, 13.04, 13.01, 12.38, 12.33. **ESI-HRMS**: Calculated for $C_{41}H_{62}N_2O_{10}Si_2Na$ $(M+H)^+$: 833.3835, Found: 833.3835. $[\alpha]_D^{20}$ = +35.6 (c = 1.0, $CHCl_3$).

(5a*R*,7*R*,11a*R*,11b*R*)-7-(4-bromophenoxy)-2,2,4,4-tetraisopropylhexahydro-9*H*-oxazolo[3,4-*a*][1,3,5,2,4]trioxadisilepino[6,7-*c*]pyridin-9-one (3x)

The title product compound is prepared according to above general procedure with iminoglycal **1a** (0.2 mmol, 1.0 equiv), 2 mol% catalyst **J**, dry CH_2Cl_2 (0.8 mL) and acceptor at room temperature for 4 h and isolated by flash column chromatography (3:1 Pentane: Ethyl Acetate) giving **3x** as a colorless oil (68 mg, 58% yield, α/β ratio > 20:1).

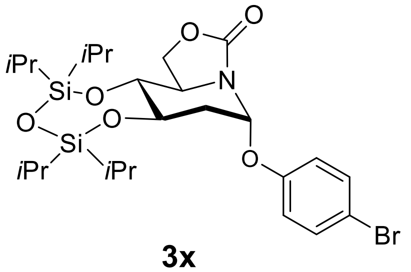

3x

^{1}**H NMR** (600 MHz, $CDCl_3$) δ 7.39 (d, *J* = 9.0 Hz, 2H), 6.94 (d, *J* = 9.0 Hz, 2H), 5.89 (dd, *J* = 4.2, 1.8 Hz, 1H), 4.38 (dd, *J* = 9.0, 8.4 Hz, 1H), 4.26 (dd, *J* = 9.0, 4.2 Hz, 1H), 4.19 (ddd, *J* = 11.4, 8.4, 4.8 Hz, 1H), 3.66 (ddd, *J* = 9.6, 8.4, 4.2 Hz, 1H), 3.53 (t, *J* = 9.0 Hz, 1H), 2.40 (ddd, *J* = 14.4, 4.8, 1.8 Hz, 1H), 1.84 (ddd, *J* = 14.4, 11.4, 4.2 Hz, 1H), 1.16 – 0.95 (m, 28H). ^{13}**C NMR** (150 MHz, $CDCl_3$) δ 155.79, 154.68, 132.71, 117.91, 114.86, 78.18, 78.03, 71.13, 66.31, 54.53, 37.65, 17.74, 17.66, 17.54, 17.42, 17.37, 17.35, 17.32, 13.07, 12.98, 12.37, 12.27. **ESI-HRMS**: Calculated for $C_{25}H_{41}BrNO_6Si_2$ $(M+H)^+$: 586.1650, Found: 586.1655. $[\alpha]_D^{20}$ = +73.2 (c = 1.1, $CHCl_3$).

(5a*R*,7*R*,11a*R*,11b*R*)-2,2,4,4-tetraisopropyl-7-(4-methoxyphenoxy)hexahydro-9*H*-oxazolo[3,4-*a*][1,3,5,2,4]trioxadisilepino[6,7-*c*]pyridin-9-one (3y)

The title product compound is prepared according to above general procedure with iminoglycal **1a** (0.2 mmol, 1.0 equiv), 2 mol% catalyst **J**, dry CH_2Cl_2 (0.8 mL) and acceptor at room temperature for 2 h and isolated by flash column chromatography (20:1 Pentane: Ethyl Acetate) giving **3y** as a colorless oil (56 mg, 52% yield, α/β ratio > 20:1).

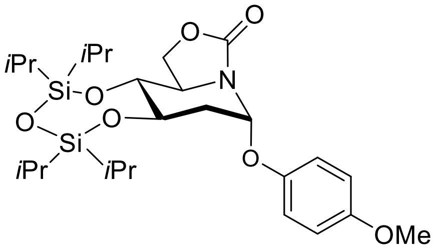

3y

^{1}**H NMR** (600 MHz, $CDCl_3$) δ 7.46 (d, *J* = 3.0 Hz, 1H), 7.25 (d, *J* = 4.2 Hz, 1H), 6.81 (d, *J* = 9.0 Hz, 1H), 6.70 (dd, *J* = 9.0, 3.0 Hz, 1H), 5.28 (d, *J* = 9.0 Hz, 1H), 4.57 (d, *J* = 3.0 Hz, 1H), 4.50 (t, *J* = 9.0 Hz, 1H), 4.37 (dd, *J* = 9.0, 4.8 Hz, 1H), 4.05 (ddd, *J* = 10.2, 9.0, 5.4 Hz, 1H), 3.83 – 3.78 (m, 1H), 3.73 (s, 3H), 2.61 (ddd, *J* = 15.6, 3.6, 1.2 Hz, 1H), 2.35 (ddd, *J* = 15.6, 9.0, 3.6 Hz, 1H), 1.10 – 1.05 (m, 14H), 1.02 – 0.97 (m, 14H). ^{13}**C NMR** (150 MHz, $CDCl_3$) δ 159.08, 153.63, 148.58, 128.06, 117.93, 116.45, 113.09, 75.89, 68.79, 66.97, 56.09, 50.72, 46.32, 33.38, 17.76, 17.65, 17.47, 17.38, 17.28, 17.02, 14.50, 13.43, 13.07, 12.81. **ESI-HRMS**: Calculated for $C_{26}H_{44}NO_7Si_2$ $(M+H)^+$: 538.2651, Found: 538.2656. $[\alpha]_D^{20}$ = +21.4 (c = 0.2, $CHCl_3$).

(5a*R*,7*R*,11a*R*,11b*R*)-2,2,4,4-tetraisopropyl-7-(phenylthio)hexahydro-9*H*-oxazolo[3,4-*a*][1,3,5,2,4]trioxadisilepino[6,7-*c*]pyridin-9-one (3z)

The title product compound is prepared according to above general procedure with iminoglycal **1a** (0.2 mmol, 1.0 equiv), 2 mol% catalyst **J**, dry CH_2Cl_2 (0.8 mL) and acceptor at room temperature for 1 h and

isolated by flash column chromatography (20:1 Pentane: Ethyl Acetate) giving **3z** as a colorless oil (75 mg, 72% yield, α/β ratio > 20:1).

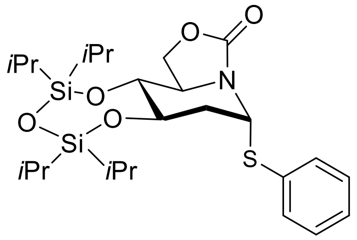

3z

¹H NMR (600 MHz, $CDCl_3$) δ 7.49 – 7.44 (m, 2H), 7.35 – 7.27 (m, 3H), 5.58 (dd, *J* = 6.0, 1.2 Hz, 1H), 4.39 (t, *J* = 9.0 Hz, 1H), 4.20 (dd, *J* = 9.0, 4.8 Hz, 1H), 4.13 (ddd, *J* = 11.4, 8.4, 4.2 Hz, 1H), 3.93 (td, *J* = 9.0, 4.8 Hz, 1H), 3.47 (t, *J* = 9.0 Hz, 1H), 2.25 (ddd, *J* = 14.4, 4.8, 1.2 Hz, 1H), 2.00 (ddd, *J* = 13.8, 10.8, 5.4 Hz, 1H), 1.16 – 0.94 (m, 28H). **¹³C NMR** (150 MHz, $CDCl_3$) δ 155.59, 132.62, 132.36, 129.40, 128.31, 78.78, 72.01, 66.13, 57.98, 54.28, 37.49, 17.71, 17.67, 17.51, 17.41, 17.37, 17.36, 17.30, 13.06, 12.96, 12.35, 12.30. **ESI-HRMS**: Calculated for $C_{25}H_{42}NO_5SSi_2$ $(M+H)^+$: 524.2317, Found:524.2318. $[\alpha]_D^{20}$ = +120.1 (c = 1.0, $CHCl_3$).

(5a*R*,7*R*,11a*R*,11b*R*)-7-(butylthio)-2,2,4,4-tetraisopropylhexahydro-9*H*-oxazolo[3,4-*a*][1,3,5,2,4]trioxadisilepino[6,7-*c*]pyridin-9-one (3za)

The title product compound is prepared according to above general procedure with iminoglycal **1a** (0.2 mmol, 1.0 equiv), 2 mol% catalyst **J**, dry CH_2Cl_2 (0.8 mL) and acceptor at room temperature for 1 h and isolated by flash column chromatography (20:1 Pentane: Ethyl Acetate) giving **3za** as a colorless oil (85 mg, 85% yield, α/β ratio > 20:1).

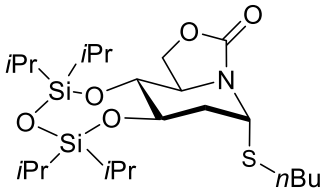

3za

¹H NMR (600 MHz, $CDCl_3$) δ 5.33 – 5.23 (m, 1H), 4.49 (t, *J* = 8.4 Hz, 1H), 4.23 (dd, *J* = 9.0, 5.4 Hz, 1H), 4.00 (ddd, *J* = 11.4, 8.4, 4.8 Hz, 1H), 3.92 (td, *J* = 9.0, 5.4 Hz, 1H), 3.44 (t, *J* = 9.0 Hz, 1H), 2.63 (ddd, *J* = 12.6, 8.4, 6.0 Hz, 1H), 2.49 (ddd, *J* = 13.2, 8.4, 6.6 Hz, 1H), 2.13 (ddd, *J* = 13.8, 4.8, 1.2 Hz, 1H), 1.96 (ddd, *J* = 14.4, 11.4, 6.0 Hz, 1H), 1.65 – 1.54 (m, 2H), 1.44 – 1.35 (m, 2H), 1.12 – 0.93 (m, 28H), 0.91 (t, *J* = 7.2 Hz, 3H). **¹³C NMR** (150 MHz, $CDCl_3$) δ 156.22, 79.15, 72.12, 66.48, 55.01, 53.92, 38.03, 31.62, 31.06, 22.09, 17.72, 17.61, 17.51, 17.40, 17.36, 17.33, 17.30, 13.75, 13.06, 12.93, 12.35, 12.28. **ESI-HRMS**: Calculated for $C_{23}H_{46}NO_5SSi_2$ $(M+H)^+$: 504.2630, Found: 504.2631. $[\alpha]_D^{20}$ = +81.8 (c = 1.4, $CHCl_3$).

(5*R*,7*R*,8*R*,8a*R*)-7,8-bis(benzyloxy)-5-(octyloxy)hexahydro-3*H*-oxazolo[3,4-*a*]pyridin-3-one (3zb)

The title product compound is prepared according to above general procedure with iminoglycal **1b** (0.2 mmol, 1.0 equiv), 2 mol% catalyst **J**, dry CH_2Cl_2 (0.8 mL) and acceptor at room temperature for 24 h and isolated by flash column chromatography (3:1 Pentane: Ethyl Acetate) giving **3zb** as a white solid (82 mg, 85% yield, α/β ratio > 20:1).

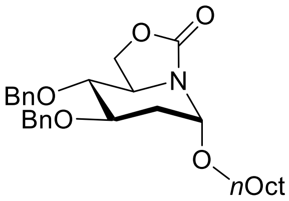

3zb

^{1}H NMR (700 MHz, $CDCl_3$) δ 7.37 – 7.34 (m, 6H), 7.33 – 7.30 (m, 2H), 7.29 – 7.27 (m, 2H), 5.13 (dd, *J* = 4.2, 2.1 Hz, 1H), 4.97 (d, *J* = 11.9 Hz, 1H), 4.71 (d, *J* = 11.9 Hz, 1H), 4.65 (dd, *J* = 11.9, 7.7 Hz, 2H), 4.36 (t, *J* = 8.4 Hz, 1H), 3.98 (ddd, *J* = 11.9, 9.1, 4.9 Hz, 1H), 3.82 (dd, *J* = 9.1, 6.3 Hz, 1H), 3.75 (ddd, *J* = 9.8, 8.4, 6.3 Hz, 1H), 3.46 – 3.37 (m, 2H), 3.32 (t, *J* = 9.1 Hz, 1H), 2.38 (ddd, *J* = 13.3, 4.9, 2.1 Hz, 1H), 1.62 (ddd, *J* = 13.3, 11.2, 3.5 Hz, 1H), 1.56 – 1.51 (m, 2H), 1.33 – 1.24 (m, 10H), 0.89 (t, *J* = 7.0 Hz, 3H). **^{13}C NMR** (175 MHz, $CDCl_3$) δ 156.47, 138.31, 138.11, 128.72, 128.60, 128.33, 128.29, 127.91, 127.84, 81.60, 79.51, 77.75, 74.92, 72.20, 68.13, 66.94, 53.20, 35.13, 31.96, 29.51, 29.49, 29.36, 26.28, 22.79, 14.24. **ESI-HRMS**: Calculated for $C_{29}H_{40}NO_5$ $(M+H)^+$: 504.2720, Found: 504.2724. $[\alpha]_D^{20}$ = +64.5 (c = 2.7, $CHCl_3$).

(5*R*,7*R*,8*R*,8a*R*)-8-(benzyloxy)-7-hydroxy-5-(octyloxy)hexahydro-3*H*-oxazolo[3,4-*a*]pyridin-3-one (3zc)

The title product compound is prepared according to above general procedure with iminoglycal **1c** (0.2 mmol, 1.0 equiv), 2 mol% catalyst **J**, dry CH_2Cl_2 (0.8 mL) and acceptor at room temperature for 24 h and isolated by flash column chromatography (2:1 Pentane: Ethyl Acetate) giving **3zc** as a white solid (57 mg, 73% yield, α/β ratio > 20:1).

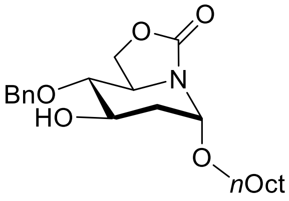

3zc

^{1}H NMR (600 MHz, $CDCl_3$) δ 7.40 – 7.36 (m, 2H), 7.36 – 7.31 (m, 3H), 5.13 (dd, *J* = 3.6, 1.8 Hz, 1H), 4.83 (d, *J* = 11.4 Hz, 1H), 4.69 (d, *J* = 12.0 Hz, 1H), 4.38 (t, *J* = 8.4 Hz, 1H), 4.17 – 4.08 (m, 1H), 3.91 (dd, *J* = 9.0, 6.6 Hz, 1H), 3.76 (ddd, *J* = 9.0, 8.4, 6.6 Hz, 1H), 3.48 – 3.36 (m, 2H), 3.21 (t, *J* = 9.0 Hz, 1H), 2.26 – 2.17 (m, 2H), 1.67 (ddd, *J* = 13.2, 12.0, 3.6 Hz, 1H), 1.55 – 1.46 (m, 2H), 1.33 – 1.19 (m, 10H), 0.88 (t, *J* = 7.2 Hz, 3H). **^{13}C NMR** (150 MHz, $CDCl_3$) δ 156.43, 137.79, 128.94, 128.57, 128.13, 83.87, 79.41, 74.76, 69.36, 68.24, 66.95, 53.32, 37.38, 31.96, 29.55, 29.49, 29.37, 26.28, 22.79, 14.24. **ESI-HRMS**: Calculated for $C_{22}H_{34}NO_5$ $(M+H)^+$: 392.2431, Found: 392.2436. $[\alpha]_D^{20}$ = +60.9 (c = 1.1, $CHCl_3$).

(5*R*,7*R*,8*R*,8a*R*)-7-hydroxy-5-(octyloxy)-3-oxohexahydro-3*H*-oxazolo[3,4-*a*]pyridin-8-yl acetate (3zd)

The title product compound is prepared according to above general procedure with iminoglycal **1d** (0.2 mmol, 1.0 equiv), 2 mol% catalyst **J**, dry CH_2Cl_2 (0.8 mL) and acceptor at room temperature for 18 h and isolated by flash column chromatography (2:1 Pentane: Ethyl Acetate) giving **3zd** as a colorless oil (42 mg, 61% yield, α/β ratio > 20:1).

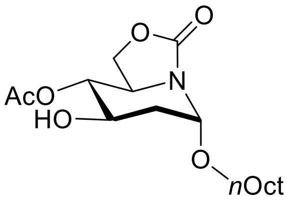

3zd

^{1}H NMR (600 MHz, $CDCl_3$) δ 5.18 (dd, *J* = 3.6, 1.8 Hz, 1H), 4.65 (t, *J* = 9.6 Hz, 1H), 4.41 (t, *J* = 9.0 Hz, 1H), 4.24 (dd, *J* = 9.0, 7.2 Hz, 1H), 4.17 – 4.08 (m, 1H), 3.85 (ddd, *J* = 9.6, 7.8, 6.6 Hz, 1H), 3.50 – 3.40 (m, 2H), 2.31 (ddd, *J* = 13.2, 4.8, 1.8 Hz, 1H), 2.15 (s, 3H), 2.13 – 2.09 (m, 1H), 1.73 (ddd, *J* = 13.2, 11.4, 3.6 Hz, 1H), 1.57 – 1.51 (m, 2H), 1.34 – 1.23 (m, 10H), 0.88 (t, *J* = 7.2 Hz, 3H). **^{13}C NMR** (150 MHz, $CDCl_3$) δ 171.33, 156.31, 79.36, 77.32, 68.44, 67.30, 66.71, 52.67, 37.64, 31.95, 29.53, 29.48, 29.37, 26.26, 22.79, 21.03, 14.24. **ESI-HRMS**: Calculated for $C_{17}H_{30}NO_6$ $(M+H)^+$: 344.2068, Found:344.2066. $[\alpha]_D^{20}$ = +14.0 (c = 0.7, $CHCl_3$).

(5*R*,7*R*,8*R*,8a*R*)-5-(octyloxy)-3-oxohexahydro-3*H*-oxazolo[3,4-*a*]pyridine-7,8-diyl diacetate (3ze)

The title product compound is prepared according to above general procedure with iminoglycal **S12** (0.2 mmol, 1.0 equiv), 2 mol% catalyst **D**, dry CH_2Cl_2 (0.8 mL) and acceptor at 40 °C for 18 h and isolated by flash column chromatography (3:1 Pentane: Ethyl Acetate) giving **3ze** as a white solid (48 mg, 62% yield, α/β ratio > 20:1).

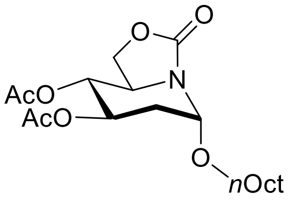

3ze

^{1}H NMR (700 MHz, $CDCl_3$) δ 5.40 – 5.32 (m, 1H), 5.18 (dd, *J* = 4.2, 2.1 Hz, 1H), 4.86 (t, *J* = 9.8 Hz, 1H), 4.41 (t, *J* = 8.4 Hz, 1H), 4.23 (dd, *J* = 9.1, 6.3 Hz, 1H), 3.92 (ddd, *J* = 9.8, 8.4, 6.3 Hz, 1H), 3.52 – 3.38 (m, 2H), 2.31 (ddd, *J* = 13.3, 4.9, 2.1 Hz, 1H), 2.07 (s, 3H), 2.03 (s, 3H), 1.77 (ddd, *J* = 13.3, 11.9, 4.2 Hz, 1H), 1.58 – 1.52 (m, 2H), 1.34 – 1.22 (m, 10H), 0.88 (t, *J* = 7.0 Hz, 3H). **^{13}C NMR** (175 MHz, $CDCl_3$) δ 170.43, 170.04, 156.14, 79.06, 73.67, 68.53, 68.50, 66.52, 52.54, 34.87, 31.95, 29.47, 29.35, 26.24, 22.78, 21.03, 20.81, 14.23. **ESI-HRMS**: Calculated for $C_{19}H_{32}NO_7$ $(M+H)^+$: 386.2173, Found: 386.2181. $[\alpha]_D^{20}$ = +28.3 (c = 1.1, $CHCl_3$).

(2*R*,3*R*,4*S*,5*R*,6*S*)-2-((((5*R*,7*R*,8*R*,8a*R*)-7,8-diacetoxy-3-oxohexahydro-3*H*-oxazolo[3,4-*a*]pyridin-5-yl)oxy)methyl)-6-methoxytetrahydro-2*H*-pyran-3,4,5-triyl tribenzoate (3zf)

The title product compound is prepared according to above general procedure with iminoglycal **S12** (0.2 mmol, 1.0 equiv), 2 mol% catalyst **D**, dry CH_2Cl_2 (0.8 mL) and acceptor at 40 °C for 5 h and isolated by flash column chromatography (3:1 Pentane: Ethyl Acetate) giving **3zf** as a white solid (83 mg, 55% yield, α/β ratio > 20:1).

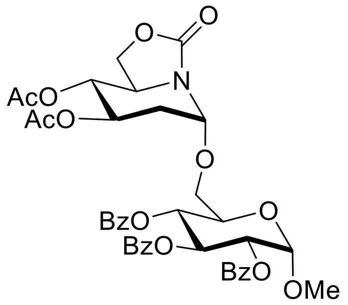

3zf

^{1}H NMR (600 MHz, $CDCl_3$) δ 7.99 – 7.86 (m, 6H), 7.55 – 7.48 (m, 2H), 7.45 – 7.35 (m, 5H), 7.32 – 7.28 (m, 2H), 6.14 (t, *J* = 9.6 Hz, 1H), 5.63 (t, *J* = 9.6 Hz, 1H), 5.39 (ddd, *J* = 11.4, 9.0, 4.8 Hz, 1H), 5.31 – 5.25 (m, 2H), 5.22 (dd, *J* = 3.6, 1.8 Hz, 2H), 4.83 (t, *J* = 9.6 Hz, 1H), 4.22 – 4.10 (m, 3H), 4.03 – 3.97 (m, 1H), 3.76 – 3.68 (m, 2H), 3.48 (s, 3H), 2.37 (ddd, *J* = 13.8, 4.8, 2.4 Hz, 1H), 2.10 (s, 3H), 2.03 (s, 3H), 1.83 (ddd, *J* = 13.2, 11.4, 4.2 Hz, 1H). **^{13}C NMR** (150 MHz, $CDCl_3$) δ 170.50, 169.88, 165.94, 165.42, 156.28, 133.68, 133.49, 133.22, 130.08, 129.90, 129.87, 129.37, 129.20, 129.00, 128.68, 128.55, 128.42, 97.19, 79.46, 73.51, 72.20, 70.66, 69.18, 68.33, 68.27, 66.55, 66.35, 55.81, 52.30, 34.44, 21.03, 20.87. **ESI-HRMS**: Calculated for $C_{39}H_{39}NO_{15}Na$ (M+Na)+: 784.2212, Found: 784.2223. $[\alpha]_D^{20}$ = +56.9 (c = 1.1, $CHCl_3$).

(5*R*,7*R*,8*R*,8a*R*)-3-oxo-5-(((2*R*,3*R*,4*S*,5*R*,6*S*)-3,4,5-tris(benzyloxy)-6-methoxytetrahydro-2*H*-pyran-2-yl)methoxy)hexahydro-3*H*-oxazolo[3,4-*a*]pyridine-7,8-diyl diacetate (3zg)

The title product compound is prepared according to above general procedure with iminoglycal **S12** (0.2 mmol, 1.0 equiv), 2 mol% catalyst **D**, dry CH_2Cl_2 (0.8 mL) and acceptor at 40 ^oC for 4 h and isolated by flash column chromatography (3:1 Pentane: Ethyl Acetate) giving **3zg** as a colorless oil (88 mg, 61% yield, α/β ratio > 20:1).

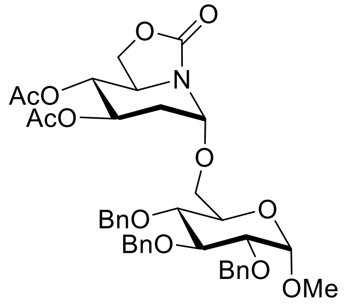

3zg

^{1}H NMR (600 MHz, $CDCl_3$) δ 7.39 – 7.36 (m, 2H), 7.36 – 7.30 (m, 9H), 7.30 – 7.26 (m, 4H), 5.29 – 5.25 (m, 1H), 5.24 (dd, *J* = 3.6, 1.8 Hz, 1H), 5.00 (dd, *J* = 11.4, 2.4 Hz, 2H), 4.82 – 4.77 (m, 3H), 4.67 (d, *J* = 12.6 Hz, 1H), 4.60 (d, *J* = 3.6 Hz, 1H), 4.13 – 3.97 (m, 3H), 3.81 – 3.75 (m, 1H), 3.74 – 3.63 (m, 3H), 3.58 – 3.46 (m, 2H), 3.39 (s, 3H), 3.34 (d, *J* = 18.0 Hz, 1H), 2.35 (ddd, *J* = 13.2, 4.8, 1.8 Hz, 1H), 2.04 (s, 3H), 2.02 (s, 3H), 1.75 (ddd, *J* = 13.2, 12.0, 4.2 Hz, 1H). **^{13}C NMR** (150 MHz, $CDCl_3$) δ 170.33, 169.88, 156.08, 138.75, 138.58, 138.27, 128.62, 128.59, 128.55, 128.25, 128.13, 128.06, 127.77, 127.74, 127.23, 98.20, 82.24, 80.25, 79.63, 77.86, 75.88, 74.87, 73.58, 73.44, 69.66, 68.27, 66.97, 66.47, 55.35, 52.35, 34.59, 21.00, 20.77. **ESI-HRMS**: Calculated for $C_{39}H_{45}NO_{12}Na$ $(M+Na)^+$: 742.2834, Found: 742.2844. $[\alpha]_D^{20}$ = +47.5 (c = 1.0, $CHCl_3$).

(5*R*,7*R*,8*R*,8a*R*)-3-oxo-5-(phenylthio)hexahydro-3*H*-oxazolo[3,4-*a*]pyridine-7,8-diyl diacetate (3zh)

The title product compound is prepared according to above general procedure with iminoglycal **S12** (0.2 mmol, 1.0 equiv), 2 mol% catalyst **D**, dry CH_2Cl_2 (0.8 mL) and acceptor at 40 ^oC for 2 h and isolated

by flash column chromatography (3:1 Pentane: Ethyl Acetate) giving **3zh** as a colorless oil (50 mg, 68% yield, α/β ratio > 20:1).

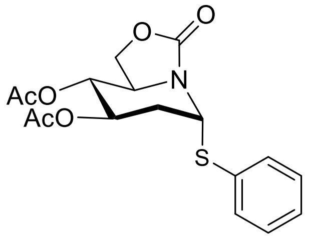

3zh

^{1}H NMR (700 MHz, $CDCl_3$) δ 7.52 – 7.46 (m, 2H), 7.38 – 7.29 (m, 3H), 5.64 – 5.56 (m, 1H), 5.44 (ddd, *J* = 11.9, 9.8, 4.9 Hz, 1H), 4.89 (t, *J* = 9.8 Hz, 1H), 4.33 (t, *J* = 8.4 Hz, 1H), 4.20 (dd, *J* = 9.1, 5.6 Hz, 1H), 4.15 (ddd, *J* = 9.8, 8.4, 5.6 Hz, 1H), 2.36 (ddd, *J* = 13.3, 4.9, 1.4 Hz, 1H), 2.09 (s, 3H), 2.08 – 2.02 (m, 4H). **^{13}C NMR** (175 MHz, $CDCl_3$) δ 170.38, 169.99, 155.16, 133.35, 131.49, 129.50, 128.79, 73.38, 68.91, 66.02, 57.45, 52.48, 34.12, 21.00, 20.83. **ESI-HRMS**: Calculated for $C_{17}H_{19}NO_6SNa$ $(M+Na)^+$: 388.0825, Found: 388.0826. $[\alpha]_D^{20}$ = +91.2 (c = 0.6, $CHCl_3$).

(5*R*,7*R*,8*R*,8a*R*)-5-((4-bromobenzyl)oxy)-3-oxohexahydro-3*H*-oxazolo[3,4-*a*]pyridine-7,8-diyl diacetate (3zi)

The title product compound is prepared according to above general procedure with iminoglycal **S12** (0.2 mmol, 1.0 equiv), 2 mol% catalyst **D**, dry CH_2Cl_2 (0.8 mL) and acceptor at 40 °C for 4 h and isolated by flash column chromatography (3:1 Pentane: Ethyl Acetate) giving **3zi** as a colorless oil (45 mg, 51% yield, α/β ratio > 20:1).

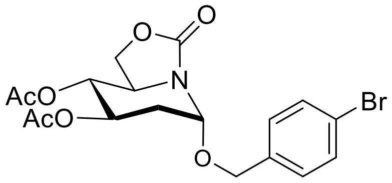

3zi

^{1}H NMR (700 MHz, $CDCl_3$) δ 7.51 – 7.45 (m, 2H), 7.23 (d, *J* = 8.4 Hz, 2H), 5.36 (ddd, *J* = 11.9, 9.8, 4.9 Hz, 1H), 5.32 – 5.27 (m, 1H), 4.87 (t, *J* = 9.8 Hz, 1H), 4.51 (s, 2H), 4.32(t, *J* = 8.4 Hz, 1H), 4.21 (dd, *J* = 9.1, 6.3 Hz, 1H), 3.83 (ddd, *J* = 9.8, 8.4, 6.3 Hz, 1H), 2.35 (ddd, *J* = 13.3, 4.9, 2.1 Hz, 1H), 2.07 (s, 3H), 2.03 (s, 3H), 1.80 (ddd, *J* = 13.3, 11.9, 4.2 Hz, 1H). **^{13}C NMR** (175 MHz, $CDCl_3$) δ 170.40, 170.03, 156.05, 136.24, 131.78, 129.63, 122.11, 78.78, 73.48, 69.55, 68.33, 66.58, 52.59, 34.79, 21.02, 20.81. **ESI-HRMS**: Calculated for $C_{18}H_{21}BrNO_7$ $(M+H)^+$: 442.0496, Found: 442.0501. $[\alpha]_D^{20}$ = +29.1 (c = 1.3, $CHCl_3$).

(2*R*,3*R*,4*S*,5*R*,6*S*)-2-((((5*R*,7*R*,8*S*,8a*R*)-7,8-bis(benzyloxy)-3-oxohexahydro-3*H*-oxazolo[3,4-*a*]pyridin-5-yl)oxy)methyl)-6-methoxytetrahydro-2*H*-pyran-3,4,5-triyl tribenzoate (4a)

The title product compound is prepared according to above general procedure with iminoglycal **1e** (0.2 mmol, 1.0 equiv), 2 mol% catalyst **J**, dry CH_2Cl_2 (0.8 mL) and acceptor at room temperature for 9 h and isolated by flash column chromatography (2:1 Pentane: Ethyl Acetate) giving **4a** as a white solid (147 mg, 86% yield, α/β ratio > 20:1).

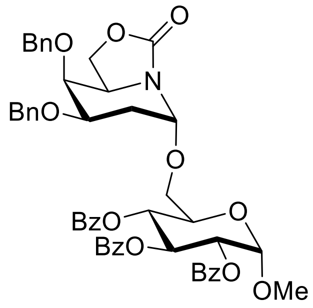

4a

^{1}**H NMR** (600 MHz, $CDCl_3$) δ 8.01 – 7.97 (m, 2H), 7.91 – 7.86 (m, 4H), 7.56 – 7.49 (m, 2H), 7.46 – 7.36 (m, 9H), 7.35 – 7.26 (m, 8H), 6.10 (t, *J* = 10.2 Hz, 1H), 5.66 (t, *J* = 10.2 Hz, 1H), 5.32 (dd, *J* = 10.2, 3.6 Hz, 1H), 5.27 – 5.23 (m, 2H), 5.01 (d, *J* = 12.0 Hz, 1H), 4.76 – 4.66 (m, 2H), 4.62 (d, *J* = 12.0 Hz, 1H), 4.21 – 4.16 (m, 1H), 4.07 – 3.99 (m, 2H), 3.86 – 3.81 (m, 1H), 3.75 – 3.65 (m, 4H), 3.45 (s, 3H), 2.30 – 2.18 (m, 2H). ^{13}**C NMR** (150 MHz, $CDCl_3$) δ 166.03, 165.91, 165.52, 157.23, 138.33, 133.80, 133.54, 133.21, 130.09, 129.80, 129.71, 129.46, 129.18, 129.02, 128.82, 128.64, 128.57, 128.54, 128.42, 127.91, 127.86, 127.81, 127.71, 97.37, 80.62, 75.19, 73.83, 72.84, 72.01, 71.25, 70.93, 68.87, 68.19, 65.36, 63.63, 55.79, 53.38, 29.76. **ESI-HRMS**: Calculated for $C_{49}H_{48}NO_{13}$ $(M+H)^+$: 858.3120, Found: 858.3138. $[\alpha]_D^{20}$ = +37.9 (c = 1.2, $CHCl_3$).

(5R,7R,8S,8aR)-7,8-bis(benzyloxy)-5-(((2R,3R,4S,5R,6S)-3,4,5,6-tetramethoxytetrahydro-2H-pyran-2-yl)methoxy)hexahydro-3H-oxazolo[3,4-a]pyridin-3-one (4b)

The title product compound is prepared according to above general procedure with iminoglycal **1e** (0.2 mmol, 1.0 equiv), 2 mol% catalyst **J**, dry CH_2Cl_2 (0.8 mL) and acceptor at room temperature for 24 h and isolated by flash column chromatography (1:3 Pentane: Ethyl Acetate) giving **4b** as a white solid (71 mg, 61% yield, α/β ratio > 20:1).

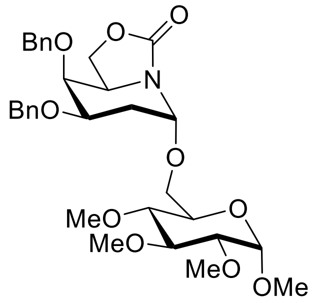

4b

^{1}**H NMR** (600 MHz, $CDCl_3$) δ 7.40 – 7.28 (m, 10H), 5.30 (d, *J* = 2.4 Hz, 1H), 5.03 (d, *J* = 12.0 Hz, 1H), 4.77 (d, *J* = 3.6 Hz, 1H), 4.66 (dd, *J* = 12.0, 3.6 Hz, 2H), 4.58 (d, *J* = 12.0 Hz, 1H), 4.26 – 4.16 (m, 2H), 3.95 – 3.85 (m, 2H), 3.72 (s, 1H), 3.64 – 3.61 (m, 5H), 3.53 – 3.50 (m, 4H), 3.45 (s, 3H), 3.41 (s, 1H), 3.33 (s, 3H), 3.20 – 3.15 (m, 1H), 3.05 (t, *J* = 9.0 Hz, 1H), 2.28 – 2.12 (m, 2H). ^{13}**C NMR** (150 MHz, $CDCl_3$) δ 157.11, 138.23, 138.10, 128.68, 128.60, 127.98, 127.96, 127.90, 127.58, 97.41, 83.43, 82.06, 80.61, 79.44, 74.96, 73.92, 72.72, 70.90, 69.55, 66.57, 63.61, 61.01, 60.11, 59.11, 55.15, 53.64, 29.78. **ESI-HRMS**: Calculated for $C_{31}H_{42}NO_{10}$ $(M+H)^+$: 588.2803, Found: 588.2815. $[\alpha]_D^{20}$ = +104.3 (c = 0.4, $CHCl_3$).

(5*R*,7*R*,8S,8a*R*)-7,8-bis(benzyloxy)-5-(((8*R*,9*S*,10*R*,13*S*,14*S*,17*S*)-10,13-dimethyl-3-oxo-2,3,6,7,8,9,10,11,12,13,14,15,16,17-tetradecahydro-1*H*-cyclopenta[*a*]phenanthren-17-yl)oxy)hexahydro-3*H*-oxazolo[3,4-*a*]pyridin-3-one (4c)

The title product compound is prepared according to above general procedure with iminoglycal **1e** (0.2 mmol, 1.0 equiv), 2 mol% catalyst **J**, dry CH_2Cl_2 (0.8 mL) and acceptor at room temperature for 24 h

and isolated by flash column chromatography (3:1 Pentane: Ethyl Acetate) giving **4c** as a white solid (89 mg, 70% yield, α/β ratio > 20:1).

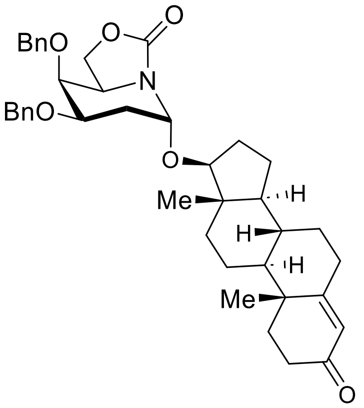

4c

^{1}H NMR (600 MHz, $CDCl_3$) δ 7.39 – 7.27 (m, 10H), 5.76 (s, 1H), 5.27 – 5.19 (m, 1H), 5.03 (d, *J* = 12.0 Hz, 1H), 4.71 – 4.57 (m, 3H), 4.25 – 4.17 (m, 2H), 3.94 – 3.83 (m, 2H), 3.72 (s, 1H), 3.37 (t, *J* = 8.4 Hz, 1H), 2.48 – 2.34 (m, 3H), 2.32 – 1.97 (m, 5H), 1.89 – 1.79 (m, 1H), 1.75 – 1.27 (m, 7H), 1.19 (d, *J* = 9.6 Hz, 3H), 1.10 – 0.88 (m, 4H), 0.78 (d, *J* = 24.0 Hz, 3H). **^{13}C NMR** (150 MHz, $CDCl_3$) δ 200.58, 172.63, 157.29, 138.25, 128.65, 128.60, 127.95, 127.94, 127.90, 127.69, 127.53, 123.88, 84.64, 78.58, 75.13, 73.90, 72.88, 70.98, 63.66, 53.98, 53.68, 50.40, 42.41, 38.84, 36.81, 35.71, 35.50, 33.88, 33.00, 31.55, 30.15, 27.17, 23.49, 20.69, 17.55, 11.85. **ESI-HRMS**: Calculated for $C_{40}H_{50}NO_6$ $(M+H)^+$: 640.3633, Found: 640.3647. $[\alpha]_D^{20}$ = +2.0 (c = 0.1, $CHCl_3$).

(5*R*,7*R*,8*S*,8a*R*)-5-(((1*R*,3*S*,5*R*,7*S*)-adamantan-2-yl)oxy)-7,8-bis(benzyloxy)hexahydro-3*H*-oxazolo[3,4-*a*]pyridin-3-one (4d)

The title product compound is prepared according to above general procedure with iminoglycal **1e** (0.2 mmol, 1.0 equiv), 2 mol% catalyst **J**, dry CH_2Cl_2 (0.8 mL) and acceptor at room temperature for 24 h and isolated by flash column chromatography (1:1 Pentane: Ethyl Acetate) giving **4d** as a white solid (75 mg, 75% yield, α/β ratio > 20:1).

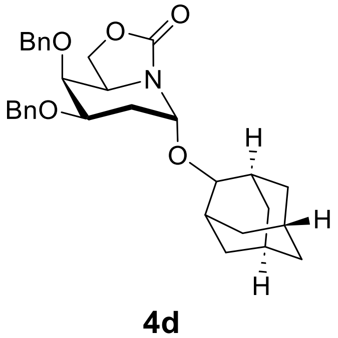

4d

^{1}H NMR (600 MHz, $CDCl_3$) δ 7.39 – 7.36 (m, 4H), 7.34 – 7.28 (m, 6H), 5.37 (d, *J* = 2.4 Hz, 1H), 5.04 (d, *J* = 12.0 Hz, 1H), 4.71 – 4.59 (m, 3H), 4.27 – 4.15 (m, 2H), 4.01 (ddd, *J* = 12.0, 4.2, 1.8 Hz, 1H), 3.97 – 3.88 (m, 1H), 3.73 (s, 1H), 3.58 (s, 1H), 2.22 (td, *J* = 12.0, 3.6 Hz, 1H), 2.13 – 2.06 (m, 2H), 2.01 – 1.94 (m, 2H), 1.85 – 1.61 (m, 9H), 1.52 – 1.42 (m, 2H). **^{13}C NMR** (150 MHz, $CDCl_3$) δ 156.92, 138.41, 138.27, 128.63, 128.57, 127.90, 127.82, 127.70, 78.83, 77.71, 75.32, 73.90, 73.24, 70.96, 63.56, 53.83, 37.64, 36.76, 36.34, 33.72, 31.96, 31.65, 30.95, 30.54, 27.59, 27.33. **ESI-HRMS**: Calculated for $C_{31}H_{37}NO_5Na$ $(M+Na)^+$: 526.2564, Found: 526.2569. $[\alpha]_D^{20}$ = +32.7 (c = 0.6, $CHCl_3$).

(5R,7R,8S,8aR)-7,8-bis(benzyloxy)-5-(phenylthio)hexahydro-3H-oxazolo[3,4-a]pyridin-3-one (4e)

The title product compound is prepared according to above general procedure with iminoglycal **1e** (0.2 mmol, 1.0 equiv), 2 mol% catalyst **J**, dry CH_2Cl_2 (0.8 mL) and acceptor at room temperature for 1 h and isolated by flash column chromatography (1:1 Pentane: Ethyl Acetate) giving **4e** as a white solid (86 mg, 93% yield, α/β ratio > 20:1).

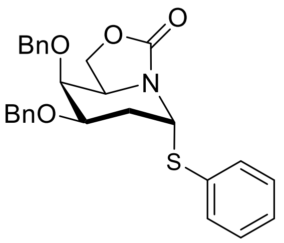

4e

^{1}H NMR (600 MHz, $CDCl_3$) δ 7.44 – 7.36 (m, 6H), 7.35 – 7.27 (m, 9H), 5.73 (d, *J* = 5.4 Hz, 1H), 5.02 (d, *J* = 12.0 Hz, 1H), 4.67 (dd, *J* = 12.0, 8.4 Hz, 2H), 4.61 (d, *J* = 12.0 Hz, 1H), 4.15 – 4.06 (m, 3H), 4.02 (ddd, *J* = 12.0, 4.2, 1.8 Hz, 1H), 3.76 (s, 1H), 2.50 (td, *J* = 12.6, 5.4 Hz, 1H), 2.10 (dd, *J* = 13.2, 4.2 Hz, 1H). **^{13}C NMR** (150 MHz, $CDCl_3$) δ 156.17, 138.16, 138.02, 132.55, 132.48, 129.33, 128.73, 128.61, 128.13, 128.05, 128.02, 127.93, 127.61, 75.90, 74.00, 73.16, 71.17, 63.38, 58.14, 53.43, 29.28. **ESI-HRMS**: Calculated for $C_{27}H_{27}NO_4SNa$ $(M+Na)^+$: 484.1553, Found: 484.1557. $[\alpha]_D^{20}$ = +93.3 (c = 1.0, $CHCl_3$).

(5*R*,7*R*,8*S*,8a*R*)-7,8-bis(benzyloxy)-5-((4-bromobenzyl)oxy)hexahydro-3*H*-oxazolo[3,4-*a*]pyridin-3-one (4f)

The title product compound is prepared according to above general procedure with iminoglycal **1e** (0.2 mmol, 1.0 equiv), 2 mol% catalyst **J**, dry CH_2Cl_2 (0.8 mL) and acceptor at 40 °C for 12 h and isolated by flash column chromatography (3:1 Pentane: Ethyl Acetate) giving **4f** as a white solid (82 mg, 76% yield, α/β ratio > 20:1).

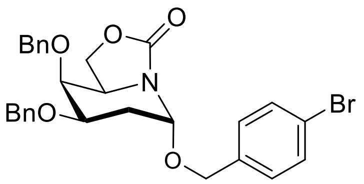

4f

^{1}H NMR (600 MHz, $CDCl_3$) δ 7.48 – 7.45 (m, 2H), 7.39 – 7.27 (m, 10H), 7.20 – 7.17 (m, 2H), 5.34 (d, *J* = 2.4 Hz, 1H), 5.02 (d, *J* = 12.0 Hz, 1H), 4.69 – 4.57 (m, 3H), 4.51 – 4.44 (m, 2H), 4.16 (dd, *J* = 8.4, 5.4 Hz, 1H), 4.11 (t, *J* = 8.4 Hz, 1H), 3.94 (ddd, *J* = 12.0, 4.2, 1.8 Hz, 1H), 3.79 (ddd, *J* = 9.0, 5.4, 2.4 Hz, 1H), 3.73 (s, 1H), 2.24 (td, *J* = 16.2, 12.0, 4.2 Hz, 1H), 2.14 (dd, *J* = 12.6, 4.2 Hz, 1H). **^{13}C NMR** (150 MHz, $CDCl_3$) δ 157.06, 138.20, 138.18, 137.01, 131.68, 129.50, 128.67, 128.60, 127.97, 127.96, 127.92, 127.54, 121.81, 80.15, 75.31, 73.91, 72.69, 71.11, 69.16, 63.61, 53.67, 29.91. **ESI-HRMS**: Calculated for $C_{28}H_{28}BrNO_5Na$ $(M+Na)^+$: 560.1043, Found: 560.1050. $[\alpha]_D^{20}$ = +4.9 (c = 1.7, $CHCl_3$).

***tert*-butyl *N*-(((9*H*-fluoren-9-yl)methoxy)carbonyl)-*O*-((5*R*,7*R*,8*S*,8a*R*)-7,8-bis(benzyloxy)-3-oxohexahydro-3*H*-oxazolo[3,4-*a*]pyridin-5-yl)-*L*-threoninate (4g)**

The title product compound is prepared according to above general procedure with iminoglycal **1e** (0.1 mmol, 1.0 equiv), 2 mol% catalyst **J**, dry CH_2Cl_2 (0.4 mL) and acceptor at 40 °C for 4 h and isolated by flash column chromatography (1:1 Pentane: Ethyl Acetate) giving **4g** as a white solid (37.4 mg, 50% yield, α/β ratio > 20:1).

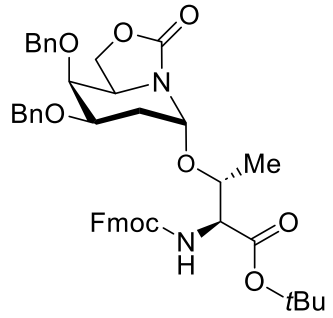

4g

^{1}H NMR (600 MHz, $CDCl_3$) δ 7.81 – 7.73 (m, 2H), 7.68 – 7.62 (m, 2H), 7.41 – 7.37 (m, 5H), 7.34 – 7.28 (m, 9H), 5.37 (d, J = 9.7 Hz, 1H), 5.33 – 5.28 (m, 1H), 5.02 (d, J = 11.9 Hz, 1H), 4.65 (dt, J = 22.6, 11.5 Hz, 3H), 4.50 – 4.40 (m, 2H), 4.32 – 4.19 (m, 5H), 3.93 – 3.86 (m, 2H), 3.75 (d, J = 2.1 Hz, 1H), 2.15 (td, J = 12.5, 4.3 Hz, 1H), 2.07 – 2.00 (m, 1H), 1.53 (s, 9H), 1.15 (d, J = 6.4 Hz, 3H). **^{13}C NMR** (150 MHz, $CDCl_3$) δ 169.62, 156.79, 156.70, 144.10, 143.92, 141.46, 138.17, 138.05, 128.68, 128.60, 128.03, 127.92, 127.90, 127.88, 127.86, 127.63, 127.22, 127.21, 125.26, 125.24, 120.15, 120.13, 82.90, 81.59, 75.44, 74.99, 73.98, 72.74, 71.06, 67.26, 63.64, 59.43, 53.71, 47.40, 29.93, 28.17, 18.86. **ESI-HRMS**: Calculated for $C_{44}H_{48}N_2O_9Na$ $(M+Na)^+$: 771.3252, Found: 771.3260. $[\alpha]_D^{20}$ = +21.6 (c = 1.0, $CHCl_3$).

(5*R*,7*R*,8*S*,8a*R*)-5-(octyloxy)-3-oxohexahydro-3*H*-oxazolo[3,4-*a*]pyridine-7,8-diyl diacetate (4h)

The title product compound is prepared according to above general procedure with iminoglycal **S28** (0.2 mmol, 1.0 equiv), 2 mol% catalyst **J**, dry CH_2Cl_2 (0.8 mL) and acceptor at 40 °C for 24 h and isolated by flash column chromatography (2:1 Pentane: Ethyl Acetate) giving **4h** as a white solid (65 mg, 85% yield, α/β ratio > 20:1).

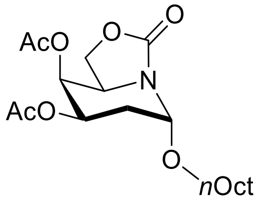

4h

^{1}H NMR (600 MHz, $CDCl_3$) δ 5.35 (s, 1H), 5.29 – 5.23 (m, 2H), 4.38 (t, *J* = 9.0 Hz, 1H), 4.17 (ddd, *J* = 9.0, 5.4, 1.8 Hz, 1H), 4.03 (dd, *J* = 9.0, 5.4 Hz, 1H), 3.49 (dt, *J* = 9.6, 7.2 Hz, 1H), 3.43 (dt, *J* = 9.6, 6.6 Hz, 1H), 2.15 (s, 3H), 2.08 (td, *J* = 13.2, 4.2 Hz, 1H), 2.01 – 1.95 (m, 4H), 1.58 – 1.51 (m, 2H), 1.34 – 1.22 (m, 10H), 0.88 (t, *J* = 7.2 Hz, 3H). **^{13}C NMR** (150 MHz, $CDCl_3$) δ 170.59, 170.20, 156.53, 79.68, 68.36, 67.10, 66.94, 63.19, 51.66, 31.96, 29.71, 29.49, 29.37, 26.29, 22.78, 20.97, 20.85, 14.23. **ESI-HRMS**: Calculated for $C_{19}H_{32}NO_7$ $(M+H)^+$: 386.2173, Found: 386.2174. $[\alpha]_D^{20}$ = +39.8 (c = 2.2, $CHCl_3$).

(2*R*,3*R*,4*S*,5*R*,6*S*)-2-((((5*R*,7*R*,8*S*,8a*R*)-7,8-diacetoxy-3-oxohexahydro-3*H*-oxazolo[3,4-*a*]pyridin-5-yl)oxy)methyl)-6-methoxytetrahydro-2*H*-pyran-3,4,5-triyl tribenzoate (4i)

The title product compound is prepared according to above general procedure with iminoglycal **S28** (0.2 mmol, 1.0 equiv), 2 mol% catalyst **J**, dry CH_2Cl_2 (0.8 mL) and acceptor at 40 °C for 24 h and isolated by flash column chromatography (1:1 Pentane: Ethyl Acetate) giving **4i** as a white solid (125 mg, 82% yield, α/β ratio > 20:1).

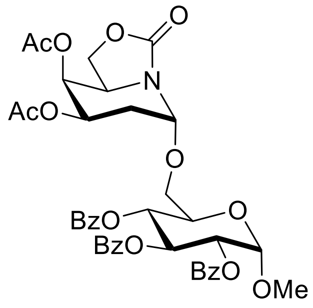

4i

^{1}H NMR (600 MHz, $CDCl_3$) δ 8.01 – 7.86 (m, 6H), 7.56 – 7.48 (m, 2H), 7.46 – 7.36 (m, 5H), 7.33 – 7.28 (m, 2H), 6.13 (t, *J* = 9.6 Hz, 1H), 5.66 (t, *J* = 9.6 Hz, 1H), 5.39 – 5.37 (m, 1H), 5.33 (ddd, *J* = 10.8, 6.0, 2.4 Hz, 1H), 5.30 – 5.24 (m, 3H), 4.27 (ddd, *J* = 9.0, 4.8, 1.8 Hz, 1H), 4.18 (dt, *J* = 10.2, 3.0 Hz, 1H), 4.09 (t, *J* = 9.6 Hz, 1H), 3.92 (dd, *J* = 9.0, 4.8 Hz, 1H), 3.76 – 3.68 (m, 2H), 3.48 (s, 3H), 2.13 (s, 3H), 2.12 – 2.07 (m, 2H), 2.02 (s, 3H). **^{13}C NMR** (150 MHz, $CDCl_3$) δ 170.56, 170.07, 165.95, 165.93, 165.49, 156.70, 133.76, 133.50, 133.25, 130.09, 129.88, 129.86, 129.34, 129.20, 128.95, 128.79, 128.55, 128.44, 97.32, 80.17, 72.14, 70.62, 69.08, 68.24, 67.12, 66.83, 66.02, 63.39, 55.89, 51.51, 29.37, 20.99, 20.83. **ESI-HRMS**: Calculated for $C_{39}H_{39}NO_{15}Na$ $(M+Na)^+$: 784.2212, Found: 784.2223. $[\alpha]_D^{20}$ = +79.0 (c = 0.7, $CHCl_3$).

(5*R*,7*R*,8*S*,8a*R*)-5-(((2*S*,6*S*,7*R*,8*S*)-7-(benzyloxy)-6-methoxy-2-phenylhexahydropyrano[3,2-*d*][1,3]dioxin-8-yl)oxy)-3-oxohexahydro-3*H*-oxazolo[3,4-*a*]pyridine-7,8-diyl diacetate (4j)

The title product compound is prepared according to above general procedure with iminoglycal **S28** (0.2 mmol, 1.0 equiv), 2 mol% catalyst **J**, dry CH_2Cl_2 (0.8 mL) and acceptor at 40 °C for 15 h and isolated by flash column chromatography (2:1 Pentane: Ethyl Acetate) giving **4j** as a white solid (78 mg, 62% yield, α/β ratio > 20:1).

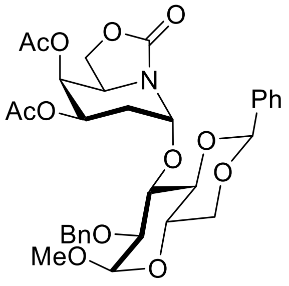

4j

^{1}H NMR (600 MHz, $CDCl_3$) δ 7.53 – 7.48 (m, 2H), 7.42 – 7.31 (m, 8H), 5.63 (t, *J* = 3.0 Hz, 1H), 5.52 (s, 1H), 5.26 (td, *J* = 9.0, 2.4 Hz, 1H), 5.08 (t, *J* = 2.4 Hz, 1H), 4.78 (d, *J* = 3.6 Hz, 1H), 4.66 (d, *J* = 10.8 Hz, 1H), 4.55 (d, *J* = 10.8 Hz, 1H), 4.25 (dd, *J* = 10.2, 4.8 Hz, 1H), 4.08 – 4.00 (m, 2H), 3.82 (td, *J* = 10.2, 4.8 Hz, 1H), 3.76 – 3.65 (m, 3H), 3.56 (t, *J* = 9.0 Hz, 1H), 3.50 (dd, *J* = 9.0, 3.6 Hz, 1H), 3.41 (s, 3H), 2.07 (s, 3H), 2.01 – 1.96 (m, 4H). **^{13}C NMR** (150 MHz, $CDCl_3$) δ 170.64, 170.20, 156.37, 137.75, 137.34, 129.19, 128.86, 128.64, 128.51, 128.45, 126.37, 101.77, 98.30, 82.27, 80.69, 78.66, 74.01, 72.93, 69.17, 67.43, 66.84, 62.92, 62.13, 55.38, 51.35, 29.70, 21.01, 20.82. **ESI-HRMS**: Calculated for $C_{32}H_{38}NO_{12}$ $(M+H)^+$: 628.2389, Found: 628.2399. $[\alpha]_D^{20}$ = +100.9 (c = 1.1, $CHCl_3$).

(2*R*,3*R*,4*S*,5*R*,6*S*)-2-((((5*R*,7*R*,8*S*,8a*R*)-8-(benzyloxy)-7-((tert-butyldimethylsilyl)oxy)-3-oxohexahydro-3*H*-oxazolo[3,4-*a*]pyridin-5-yl)oxy)methyl)-6-methoxytetrahydro-2*H*-pyran-3,4,5-triyl tribenzoate (4k)

The title product compound is prepared according to above general procedure with iminoglycal **1f** (0.2 mmol, 1.0 equiv), 2 mol% catalyst **J**, dry CH_2Cl_2 (0.8 mL) and acceptor at 40 °C for 13 h and isolated

by flash column chromatography (3:1 Pentane: Ethyl Acetate) giving **4k** as a white solid (128 mg, 73% yield, α/β ratio > 20:1).

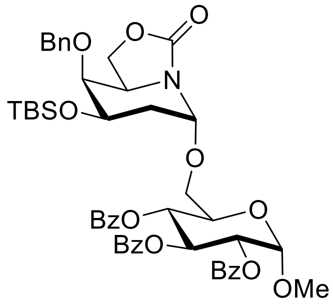

4k

^{1}H NMR (600 MHz, $CDCl_3$) δ 8.01 – 7.95 (m, 2H), 7.93 – 7.84 (m, 4H), 7.52 (q, *J* = 7.2 Hz, 2H), 7.47 – 7.36 (m, 5H), 7.35 – 7.27 (m, 7H), 6.10 (t, *J* = 9.6 Hz, 1H), 5.67 (t, *J* = 9.6 Hz, 1H), 5.30 – 5.18 (m, 3H), 5.07 (d, *J* = 12.0 Hz, 1H), 4.60 (d, *J* = 12.0 Hz, 1H), 4.35 (dd, *J* = 12.0, 2.4 Hz, 1H), 4.20 (d, *J* = 10.2 Hz, 1H), 4.04 (dd, *J* = 7.2, 4.8 Hz, 1H), 3.84 – 3.75 (m, 2H), 3.70 – 3.60 (m, 2H), 3.51 (s, 1H), 3.47 (s, 3H), 2.24 (td, *J* = 13.2, 4.2 Hz, 1H), 1.97 (dd, *J* = 13.2, 4.2 Hz, 1H), 0.99 (s, 9H), 0.22 (d, *J* = 1.2 Hz, 6H). **^{13}C NMR** (150 MHz, $CDCl_3$) δ 166.03, 165.87, 165.33, 157.17, 138.60, 133.73, 133.53, 133.19, 130.09, 129.83, 129.75, 129.48, 129.21, 129.10, 128.78, 128.57, 128.54, 128.39, 127.75, 127.67, 97.24, 80.62, 75.75, 74.29, 72.26, 70.92, 68.86, 68.31, 65.23, 63.55, 55.70, 53.35, 32.99, 26.05, 18.34, -4.55. **ESI-HRMS**: Calculated for $C_{48}H_{56}NO_{13}Si$ $(M+H)^+$: 882.3515, Found: 882.3531. $[\alpha]_D^{20}$ = +42.3 (c = 1.0, $CHCl_3$).

(2*R*,3*R*,4*S*,5*R*,6*S*)-2-((((5*R*,7*R*,8*S*,8a*R*)-8-acetoxy-7-hydroxy-3-oxohexahydro-3*H*-oxazolo[3,4-*a*]pyridin-5-yl)oxy)methyl)-6-methoxytetrahydro-2*H*-pyran-3,4,5-triyl tribenzoate (4l)

The title product compound is prepared according to above general procedure with iminoglycal **1g** (0.2 mmol, 1.0 equiv), 2 mol% catalyst **J**, dry CH_2Cl_2 (0.8 mL) and acceptor at 40 °C for 12 h and isolated by flash column chromatography (1:1 Pentane: Ethyl Acetate) giving **4l** as a white solid (95 mg, 66% yield, α/β ratio > 20:1).

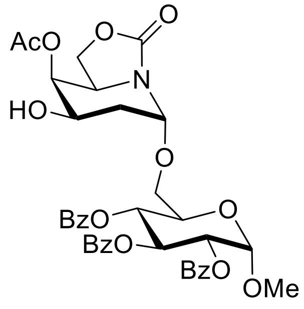

4l

^{1}H NMR (600 MHz, $CDCl_3$) δ 7.99 – 7.85 (m, 6H), 7.56 – 7.49 (m, 2H), 7.45 – 7.35 (m, 5H), 7.30 (t, *J* = 7.8 Hz, 2H), 6.12 (t, *J* = 9.6 Hz, 1H), 5.66 (t, *J* = 9.6 Hz, 1H), 5.31 – 5.22 (m, 3H), 4.33 (dd, *J* = 7.8, 4.8 Hz, 1H), 4.19 (dt, *J* = 10.2, 3.0 Hz, 1H), 4.10 – 4.01 (m, 2H), 3.96 (s, 1H), 3.71 (d, *J* = 3.0 Hz, 2H), 3.47 (s, 3H), 2.18 – 2.09 (m, 4H), 2.08 – 2.00 (m, 2H). **^{13}C NMR** (150 MHz, $CDCl_3$) δ 169.81, 165.95, 165.47, 157.10, 133.72, 133.48, 133.21, 130.09, 129.87, 129.84, 129.40, 129.23, 129.04, 128.76, 128.55, 128.42, 97.28, 80.01, 72.18, 70.78, 69.29, 69.06, 68.24, 65.95, 65.86, 63.28, 55.83, 52.33, 28.50, 21.28. **ESI-HRMS**: $C_{37}H_{38}NO_{14}$ $(M+H)^+$: 720.2287, Found: 720.2301. $[\alpha]_D^{20}$ = +26.1 (c = 1.0, $CHCl_3$).

6. X-Ray crystallography data of 3a

The crystal structure of compounds **3a** was determined using the *Bruker D8 Venture four-circle diffractometer* equipped with a *PHOTON II* CPAD detector by *Bruker AXS GmbH*. The X-ray radiation was generated by the *IμS* microfocus source Mo (λ = 0.71073 Å) from *Incoatec GmbH* equipped with HELIOS mirror optics and a single-hole collimator by *Bruker AXS GmbH*. The selected single crystal of **3a** was covered with an inert oil (perfluoropolyalkyl ether) and mounted on the *MicroMount* from *MiTeGen*. The APEX 4 Suite (v.2021.10-0) software integrated with SAINT (integration) and SADABS (adsorption correction) programs by *Bruker AXS GmbH* was used for data collection. The processing and finalization of the crystal structure were performed using the Olex2 program.[15] The crystal structures were solved by the ShelXT[16] structure solution program using the Intrinsic Phasing option, which were further refined by the ShelXL[17] refinement package using Least Squares minimization. The non-hydrogen atoms were anisotropically refined. The C-bound H atoms were placed in geometrically calculated positions, and a fixed isotropic displacement parameter was assigned to each atom according to the riding-model: C–H = 0.95–1.00 Å with U_{iso}(H) = 1.5U_{eq}(CH_3) and 1.2U_{eq}(CH_2, CH) for other hydrogen atoms. The crystallographic data for the structures of **3a** has been deposited under the CCDC number 2215612 (B2720) in the Cambridge Crystallographic Data Centre. A copy of these data can be obtained for free by applying to CCDC, 12 Union Road, Cambridge CB2 IEZ, UK, fax: 144-(0)1223-336033 or e-mail: deposit@ccdc.cam.ac.uk.

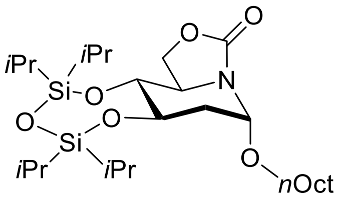

3a

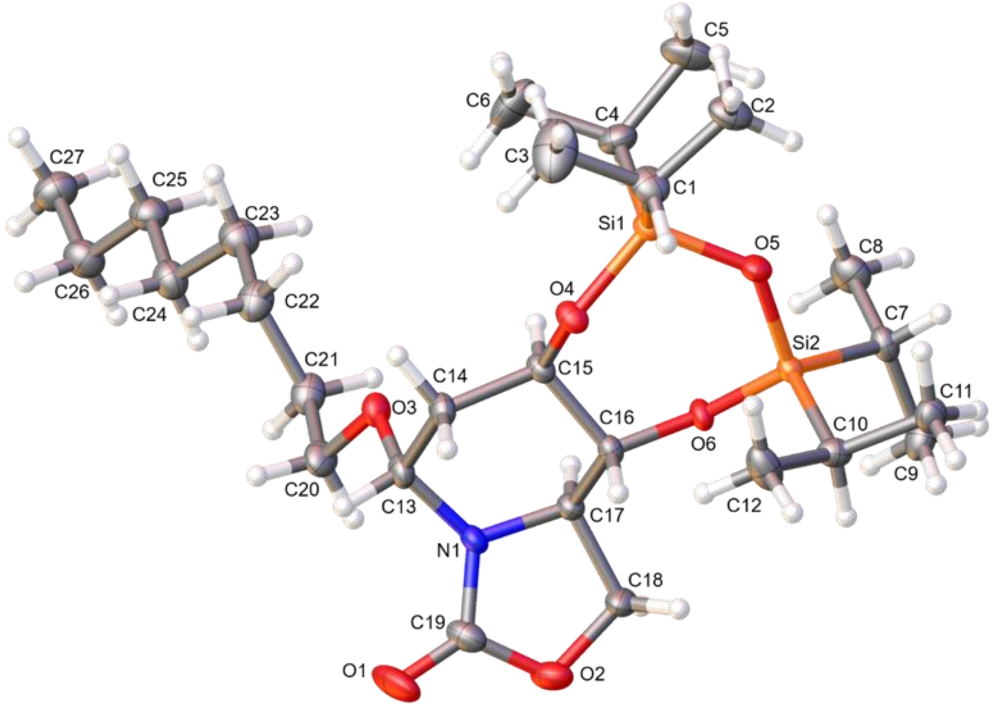

Supplementary Figure S1. Molecular structure in the crystal of compound **3a**. The displacement ellipsoids are drawn at the 50% probability level. The asymmetric unit contains four of the shown molecules, which has been omitted for clarity. In addition, for reason of clarity, the disorder of an *n*-octyl and an *i*-propyl group was not shown.

Table S1. Crystallographic data of compound **3a**.

Compound	**3a**
Empirical formula	$C_{27}H_{53}NO_6Si_2$
Formula weight	543.88
Temperature/K	100.00
Crystal system	monoclinic
Space group	$P2_1$
a/Å	23.3220(12)
b/Å	7.6496(4)
c/Å	36.0472(18)
α/°	90
β/°	98.683(2)
γ/°	90
Volume/Å^3	6357.3(6)
Z	8
ρ_{calc}g/cm^3	1.137
μ/mm^{-1}	0.148
F(000)	2384.0
Crystal size/mm^3	0.605 × 0.252 × 0.115
Radiation	MoKα (λ = 0.71073)
2Θ range for data collection/°	3.612 to 61.108
Index ranges	–33 ≤ h ≤ 33, –10 ≤ k ≤ 10, –48 ≤ l ≤ 51
Reflections collected	157288
Independent reflections	38800 [R_{int} = 0.0417, R_{sigma} = 0.0413]
Data/restraints/parameters	38800/1/1372
Goodness-of-fit on F^2	1.088
Final *R* indexes [I>=2σ (I)]	R_1 = 0.0545, wR_2 = 0.1361
Final *R* indexes [all data]	R_1 = 0.0649, wR_2 = 0.1432
Largest diff. peak/hole / e Å^{-3}	0.92/–0.39
Flack parameter	–0.040(19)

Table S2. Fractional Atomic Coordinates (×10^4) and Equivalent Isotropic Displacement Parameters (Å^2×10^3) for mo_B2720_0m. U_{eq} is defined as 1/3 of the trace of the orthogonalised U_{IJ} tensor.

**Atom**	***x***	***y***	***z***	**U(eq)**
Si1	11142.5(3)	10447.5(11)	8544.0(2)	19.67(15)
Si2	11989.0(3)	9134.6(10)	9186.1(2)	16.81(14)
O1	9562.1(13)	7021(4)	10081.9(8)	45.8(7)
O2	10519.0(11)	6462(3)	10165.1(6)	31.0(5)
O3	9534.1(9)	6019(3)	9008.7(6)	22.6(4)
O4	10658.6(9)	10248(3)	8831.5(6)	21.3(4)
O5	11789.1(9)	10089(3)	8781.5(6)	22.2(4)

O6	11466.6(8)	7844(3)	9285.0(6)	19.1(4)
N1	10075.9(10)	6840(4)	9584.9(7)	20.5(5)
C1	11112.4(15)	12813(4)	8403.0(10)	30.0(7)
C2	11610.2(16)	13306(5)	8189.9(10)	32.7(7)
C3	10535(2)	13419(6)	8204.9(16)	54.6(13)
C4	11011.9(15)	8741(5)	8170.6(9)	27.8(6)
C5	11462(2)	8785(6)	7896.6(11)	43.0(9)
C6	10390.4(19)	8704(6)	7957.5(12)	48.4(11)
C7	12601.6(12)	7599(4)	9150.3(9)	22.2(6)
C8	12439.6(16)	6233(5)	8843.8(10)	31.6(7)
C9	12809.4(14)	6736(5)	9532.4(10)	30.2(7)
C10	12199.0(13)	10851(4)	9553.0(8)	22.4(6)
C11	12777.2(13)	11692(4)	9488.7(9)	26.2(6)
C12	11743.4(15)	12275(5)	9569.0(12)	33.9(8)
C13	9643.0(12)	7381(4)	9273.4(9)	22.0(6)
C14	9874.3(12)	8948(4)	9079.7(9)	21.2(5)
C15	10487.9(12)	8668(4)	8993.0(8)	18.0(5)
C16	10897.9(11)	8236(4)	9355.0(8)	17.8(5)
C17	10674.5(11)	6593(4)	9530.3(8)	18.4(5)
C18	10964.3(13)	6196(5)	9931.7(8)	25.5(6)
C19	10004.4(15)	6804(5)	9950.7(9)	28.0(6)
C20	9213.3(14)	4609(4)	9143.0(10)	28.1(7)
C21	9186.9(14)	3089(4)	8873.7(10)	29.2(7)
C22	8882.4(15)	3499(4)	8480.1(10)	30.2(7)
C23	8833.2(16)	1940(5)	8213.4(10)	33.6(7)
C24	8435.3(14)	483(5)	8312.4(10)	29.9(7)
C25	8355.0(15)	-1015(5)	8030.7(10)	30.4(7)

C26	7978.9(16)	-2479(5)	8144.5(11)	32.7(7)
C27	7854.7(17)	-3917(5)	7853.5(11)	36.4(8)
Si3A	6844.4(4)	-56.0(12)	6465.7(2)	24.51(17)
Si4	7373.0(3)	1149.4(11)	5810.9(2)	19.42(15)
O7	4519.8(11)	3049(4)	4900.4(8)	44.2(7)
O8	5435.4(10)	3686(3)	4820.7(6)	29.8(5)
O9	4983.5(9)	4017(3)	5977.6(6)	22.6(4)
O10	6233.0(9)	-10(3)	6171.1(6)	23.3(4)
O11	7381.2(10)	300(3)	6226.8(6)	26.2(5)
O12	6801.3(8)	2425(3)	5708.9(6)	21.0(4)
N2	5261.7(10)	3247(3)	5398.8(7)	20.3(5)
C28	6841.8(15)	1743(5)	6820.5(9)	30.2(7)
C29	7365.5(19)	1667(7)	7134.8(11)	46.1(10)
C30	6273.8(18)	1829(6)	6991.6(10)	37.3(8)
C31A	6956(3)	-2409(8)	6619.0(18)	23.8(11)
C31B	6854(5)	-2177(15)	6737(3)	23.8(11)
C32A	6547(3)	-2920(8)	6893(2)	39.4(16)
C32B	6428(5)	-3484(14)	6534(4)	52(4)
C33	7552.0(18)	-2831(5)	6785.9(11)	39.0(9)
C34	7396.7(14)	-626(4)	5455.7(9)	26.5(6)
C35	6946.9(15)	-2089(5)	5465.1(13)	41.5(9)
C36	8005.5(14)	-1445(4)	5510.1(10)	28.2(6)
C37	8003.8(13)	2667(4)	5824.3(9)	25.2(6)
C38	8019.9(15)	3481(5)	5437.1(10)	31.0(7)
C39	7996.8(16)	4081(5)	6124.8(11)	33.8(7)
C40	4979.2(12)	2664(4)	5710.4(8)	20.7(5)
C41	5313.1(12)	1136(4)	5905.8(8)	21.0(5)
C42	5962.0(12)	1518(4)	5997.9(8)	19.3(5)
C43	6202.4(11)	1962(4)	5638.0(8)	19.1(5)
C44	5883.1(12)	3569(4)	5456.7(8)	18.8(5)

C45	5987.0(13)	3961(5)	5054.0(8)	25.4(6)
C46	5022.7(14)	3300(4)	5034.1(9)	25.3(6)
C47	4591.1(15)	5404(5)	5852.7(10)	29.0(7)
C48	4710.0(16)	6927(5)	6116.9(10)	31.1(7)
C49	4646.4(17)	6512(4)	6519.3(10)	31.6(7)
C50	4744.0(16)	8091(5)	6783.3(10)	33.0(7)
C51	4271.7(15)	9479(5)	6712.5(10)	29.3(7)
C52	4367.3(14)	11022(4)	6979.3(9)	27.7(6)
C53	3889.7(16)	12392(5)	6908.6(11)	33.0(7)
C54	3961(2)	13861(5)	7198.4(11)	40.8(9)
Si5	15972.8(4)	8123.6(11)	8461.7(2)	21.25(16)
Si6	16875.0(3)	6824.2(11)	9074.7(2)	18.65(15)
O13	14556.4(15)	4904(5)	10101.6(9)	52.6(9)
O14	15510.4(12)	4273(4)	10139.6(7)	36.3(6)
O15	14411.9(9)	4001(3)	9020.7(6)	24.2(4)
O16	15535.3(9)	8090(3)	8782.0(6)	22.9(4)
O17	16637.5(9)	7744(3)	8669.6(6)	22.9(4)
O18	16354.2(9)	5634(3)	9209.5(6)	21.8(4)
N3	15012.5(11)	4786(4)	9578.3(7)	24.9(5)
C55	15759.0(14)	6342(5)	8112.5(9)	28.9(7)
C56	15104.2(16)	6283(6)	7969.2(11)	37.2(8)
C57	16102.7(18)	6388(6)	7780.3(10)	41.1(9)
C58	15963.4(17)	10417(5)	8284.7(11)	36.1(8)
C59	16464(2)	10848(5)	8075.1(11)	41.3(9)
C60A	15393(2)	10982(7)	8059.2(19)	55.9(18)
C60B	15553(12)	11610(30)	8422(7)	41.3(9)
C61	17447.2(13)	5202(4)	9010.5(9)	24.1(6)

C62	17707.5(14)	4353(5)	9386.5(10)	29.8(7)
C63	17206.3(16)	3806(5)	8721.8(10)	33.3(7)
C64	17150.7(14)	8543(4)	9424.3(9)	26.1(6)
C65	17728.0(15)	9284(5)	9341.9(12)	37.8(8)
C66	16719.3(16)	10033(5)	9449.7(11)	33.3(7)
C67	14555.2(13)	5348(4)	9284.0(9)	24.1(6)
C68	14770.5(13)	6896(4)	9077.0(9)	23.6(6)
C69	15365.8(12)	6536(4)	8962.9(8)	19.7(5)
C70	15806.0(12)	6085(4)	9305.7(8)	19.7(5)
C71	15590.3(12)	4468(4)	9492.2(8)	20.9(5)
C72	15919.7(14)	4034(5)	9884.0(9)	30.0(7)
C73	14978.0(17)	4670(4)	9949.0(9)	29.7(7)
C74	14161.9(15)	2510(5)	9174.2(10)	29.3(7)
C75	13984.3(14)	1197(5)	8862.8(9)	27.1(6)
C76	13544.0(15)	1938(5)	8548.2(10)	32.7(7)
C77	13311.5(14)	613(5)	8243.9(10)	31.0(7)
C78	13774.3(15)	-178(5)	8043.4(10)	31.1(7)
C79	13539.7(16)	-1210(5)	7686.8(10)	33.2(7)
C80	13115.3(15)	-2665(5)	7741.6(10)	31.5(7)
C81	13361.8(17)	-4041(5)	8024.8(11)	36.5(8)
Si7	11631.6(4)	1798.7(12)	6535.8(2)	22.02(16)
Si8	12278.2(3)	3167.5(11)	5944.3(2)	20.44(15)
O19	9554.8(13)	5098(4)	4879.3(8)	48.9(8)
O20	10494.8(12)	5751(3)	4868.6(6)	33.9(6)
O21	9829.2(9)	6069(3)	5970.9(6)	24.7(4)
O22	11049.8(9)	1967(3)	6216.4(6)	23.6(4)
O23	12210.8(9)	2156(3)	6336.6(6)	24.3(4)
O24	11707.9(9)	4423(3)	5820.4(6)	22.0(4)

N4	10208.2(10)	5358(4)	5421.0(7)	23.5(5)
C1A	9474(5)	9569(13)	6841(2)	89(3)
C1B	9137(3)	11214(8)	6794.1(19)	48.6(14)
C1C	9340(4)	12400(11)	7137(2)	51.9(18)
C82	11617.8(13)	3518(5)	6902.6(8)	26.0(6)
C83	11048.5(15)	3569(6)	7068.8(10)	35.7(8)
C84	12147.4(15)	3449(5)	7215.9(9)	32.8(7)
C85	11637.6(18)	-544(5)	6678.8(14)	48.8(12)
C86A	11096(2)	-1222(7)	6785.2(16)	35.0(14)
C86B	11256(5)	-1729(18)	6510(4)	36(4)
C87	12170.3(16)	-1080(5)	6951.3(10)	34.9(8)
C88	12372.7(15)	1495(4)	5580.0(9)	29.0(7)
C89	12992.9(17)	770(5)	5638.9(13)	42.0(9)
C90	11938.7(16)	-11(5)	5561.5(11)	35.8(8)
C91	12893.1(13)	4754(4)	6021.7(9)	25.9(6)
C92	12815.1(16)	6074(5)	6330.9(11)	36.2(8)
C93	12968.6(15)	5693(5)	5654.8(11)	32.8(7)
C94	9867.9(12)	4747(4)	5704.4(9)	22.9(6)
C95	10167.8(12)	3191(4)	5908.9(9)	22.9(6)
C96	10812.4(12)	3531(4)	6040.0(8)	20.0(5)
C97	11113.7(11)	4012(4)	5707.3(8)	19.5(5)
C98	10826.4(12)	5650(4)	5521.7(8)	21.0(5)
C99	10997.0(14)	6117(5)	5140.6(9)	28.9(7)
C100	10033.9(16)	5373(4)	5047.3(9)	29.3(7)
C101	9470.9(15)	7506(5)	5822.8(11)	33.6(7)
C102	9494.8(16)	8896(5)	6121.6(12)	37.8(8)
C103	9286(3)	8295(7)	6477.7(17)	70.6(17)
C104	9147(14)	9800(40)	6703(6)	89(3)

C105	9284(8)	10320(20)	7022(5)	48.6(14)
C106	9520(10)	11890(30)	7251(6)	51.9(18)
C107	9057(3)	13989(10)	7113.1(17)	77.8(19)
C108	9303(3)	15285(8)	7402.1(16)	65.4(14)

Table S3. Anisotropic Displacement Parameters (Å^2×10^3) for mo_B2720_0m. The Anisotropic displacement factor exponent takes the form: $-2\pi^2[h^2a^{*2}U_{11}+2hka^*b^*U_{12}+…]$.

**Atom**	**U_{11}**	**U_{22}**	**U_{33}**	**U_{23}**	**U_{13}**	**U_{12}**
Si1	20.9(3)	21.1(4)	17.9(4)	4.6(3)	5.7(3)	0.4(3)
Si2	14.9(3)	18.8(3)	17.5(3)	0.6(3)	4.6(3)	-1.2(3)
O1	44.8(15)	61.7(19)	37.6(15)	13.5(14)	27.7(12)	22.1(14)
O2	37.5(13)	37.9(14)	19.0(10)	2.4(9)	8.6(9)	7.2(10)
O3	18.1(9)	23.4(10)	26.2(11)	3.0(8)	3.0(8)	-3.5(8)
O4	22.2(9)	18.2(10)	25.0(10)	5.4(8)	8.5(8)	1.6(8)
O5	21.1(9)	28.5(11)	17.8(10)	5.1(8)	6.0(7)	-1.5(8)
O6	12.2(8)	22.5(10)	23.2(10)	4.0(8)	5.1(7)	0.2(7)
N1	16.3(10)	27.1(12)	19.8(11)	3.7(10)	7.6(8)	0.3(10)
C1	33.5(16)	24.2(15)	33.6(17)	8.6(13)	9.2(13)	-0.2(13)
C2	42.5(19)	29.2(16)	29.1(16)	2.9(13)	14.5(14)	-8.1(15)
C3	45(2)	32(2)	83(4)	23(2)	-4(2)	1.2(18)
C4	32.6(16)	31.2(16)	18.6(14)	0.9(12)	0.6(11)	1.8(13)
C5	65(3)	40(2)	29.0(18)	-5.3(15)	22.6(18)	-0.9(19)
C6	47(2)	49(2)	42(2)	-9.6(18)	-18.4(18)	3.6(19)
C7	17.6(12)	21.5(13)	28.6(15)	-1.3(11)	7.4(10)	-0.8(10)
C8	34.3(17)	28.1(16)	34.4(18)	-5.4(14)	11.3(14)	3.0(14)
C9	22.4(14)	28.2(15)	37.5(18)	3.4(14)	-3.7(12)	2.1(13)
C10	26.8(14)	22.0(14)	19.0(13)	-0.1(10)	5.1(10)	-1.7(11)
C11	22.6(13)	27.7(15)	28.0(15)	-4.0(12)	2.9(11)	-3.7(12)
C12	28.1(15)	29.9(17)	47(2)	-12.6(15)	15.8(14)	-4.7(13)

C13	14.7(11)	25.0(14)	27.0(15)	3.5(11)	5.3(10)	1.9(10)
C14	15.4(11)	22.3(13)	26.0(14)	4.3(11)	3.5(10)	2.1(10)
C15	17.7(12)	17.3(12)	20.3(13)	2.9(10)	6.8(9)	1.2(10)
C16	14.4(11)	19.4(12)	20.4(12)	-0.2(10)	5.2(9)	-0.1(10)
C17	16.8(11)	21.8(13)	17.1(12)	2.7(10)	3.9(9)	-0.6(10)
C18	22.7(13)	34.8(16)	19.2(13)	6.1(12)	3.7(10)	-4.7(12)
C19	33.0(15)	27.7(15)	26.2(15)	4.2(12)	13.7(12)	5.1(13)
C20	24.5(14)	29.6(16)	30.1(16)	7.8(13)	4.1(12)	-7.2(12)
C21	26.4(14)	23.5(15)	36.6(17)	7.3(13)	0.6(12)	-1.5(12)
C22	29.8(16)	23.1(15)	36.4(18)	5.7(13)	0.2(13)	1.3(12)
C23	35.2(17)	31.6(17)	34.9(18)	4.5(14)	7.5(14)	-0.9(15)
C24	26.2(14)	29.4(16)	34.8(17)	1.7(14)	7.2(12)	1.5(13)
C25	33.5(16)	32.0(17)	26.2(15)	3.4(13)	5.4(12)	1.1(14)
C26	35.7(17)	30.4(17)	33.9(18)	-1.9(14)	11.1(14)	1.8(14)
C27	41.9(19)	34.2(18)	32.0(18)	-2.2(15)	1.7(15)	-1.4(16)
Si3A	25.9(4)	23.1(4)	22.2(4)	8.2(3)	-3.7(3)	-0.6(3)
Si4	16.4(3)	18.7(3)	21.5(4)	0.3(3)	-2.8(3)	2.8(3)
O7	30.9(12)	62.6(19)	33.0(13)	13.2(13)	-15.2(10)	-17.9(13)
O8	30.9(11)	38.5(13)	18.1(10)	4.0(9)	-2.9(8)	-6.8(10)
O9	21.1(9)	23.7(10)	23.3(10)	1.3(8)	4.5(8)	3.4(8)
O10	25.1(10)	17.6(9)	25.1(11)	6.4(8)	-3.2(8)	-0.7(8)
O11	23.8(10)	29.9(12)	23.2(11)	4.5(9)	-2.3(8)	5.2(9)
O12	14.5(9)	21.3(10)	26.1(11)	3.3(8)	-0.3(7)	1.4(8)
N2	14.9(10)	26.4(12)	18.6(11)	0.3(9)	-0.7(8)	1.2(9)
C28	36.0(17)	33.2(16)	19.3(14)	4.1(12)	-2.3(12)	-2.1(14)
C29	51(2)	58(3)	25.5(17)	-3.2(17)	-9.6(16)	-4(2)
C30	47(2)	40.8(19)	25.3(16)	4.2(15)	10.4(14)	3.8(17)
C31A	31(3)	24(2)	18(3)	8(2)	9(2)	7.7(19)
C31B	31(3)	24(2)	18(3)	8(2)	9(2)	7.7(19)

C32A	41(3)	34(3)	49(4)	18(3)	23(3)	11(3)
C32B	47(6)	28(5)	76(9)	26(5)	-1(6)	-3(5)
C33	54(2)	31.3(18)	29.8(18)	7.3(14)	0.2(16)	14.3(17)
C34	25.9(14)	24.8(15)	26.0(15)	-4.4(12)	-5.4(11)	5.5(12)
C35	23.6(15)	29.7(18)	68(3)	-16.2(18)	-3.0(16)	0.5(14)
C36	27.8(15)	24.4(15)	32.4(17)	-4.4(12)	4.4(12)	4.4(12)
C37	16.3(12)	24.2(14)	33.4(16)	-1.4(12)	-1.2(11)	1.4(11)
C38	24.8(15)	30.4(17)	38.9(18)	1.8(14)	8.3(13)	1.5(13)
C39	34.2(17)	29.1(16)	35.6(18)	-6.5(14)	-3.2(14)	-6.2(14)
C40	15.9(11)	22.4(13)	23.5(14)	2.8(10)	2.3(10)	-1.0(10)
C41	20.0(12)	19.6(12)	23.4(14)	2.3(11)	3.2(10)	-3.8(11)
C42	19.1(12)	15.9(12)	22.0(13)	2.3(10)	0.0(10)	-0.5(10)
C43	15.7(11)	19.1(12)	21.1(13)	1.1(10)	-1.7(9)	0.5(10)
C44	16.4(11)	22.5(13)	17.4(12)	2.7(10)	2.2(9)	1.8(10)
C45	19.9(13)	35.4(16)	20.9(13)	8.3(12)	3.5(10)	5.8(12)
C46	26.6(14)	24.3(15)	22.2(14)	2.4(11)	-5.2(11)	-1.7(12)
C47	29.0(15)	29.5(16)	29.3(16)	4.7(13)	6.6(12)	11.6(13)
C48	35.8(17)	23.4(15)	35.8(18)	6.9(13)	11.3(14)	5.1(13)
C49	40.3(18)	24.4(16)	31.4(17)	3.6(12)	9.9(14)	3.1(14)
C50	36.5(17)	31.1(17)	30.8(17)	-1.1(14)	2.8(13)	6.2(15)
C51	27.7(15)	28.3(16)	31.3(17)	-4.1(13)	3.0(12)	0.1(13)
C52	27.5(14)	27.1(15)	27.3(15)	0.3(12)	0.9(12)	-1.7(13)
C53	32.9(17)	30.7(17)	33.8(18)	-5.2(14)	-0.4(13)	-0.2(14)
C54	56(2)	31.5(18)	34.4(19)	-5.2(15)	5.8(17)	3.5(17)
Si5	22.3(4)	24.5(4)	16.5(3)	3.1(3)	1.4(3)	0.6(3)
Si6	17.1(3)	23.1(4)	15.7(3)	1.6(3)	2.4(3)	-2.9(3)
O13	66(2)	61.2(19)	39.1(16)	16.4(14)	34.9(15)	28.0(16)
O14	51.4(15)	40.7(14)	17.2(11)	2.4(10)	6.0(10)	8.5(12)

O15	21.3(10)	27.2(11)	23.6(10)	0.8(9)	1.2(8)	-6.2(9)
O16	24.4(10)	22.0(10)	22.6(10)	3.8(8)	4.2(8)	1.5(8)
O17	21.7(9)	29.6(11)	16.9(9)	3.1(8)	1.4(7)	-4.1(8)
O18	17.9(9)	25.5(10)	22.8(10)	3.8(8)	5.3(7)	-0.8(8)
N3	20.0(11)	36.4(14)	19.0(12)	0.3(10)	5.6(9)	-5.8(11)
C55	29.0(15)	35.0(17)	20.7(14)	-2.8(12)	-2.8(11)	3.1(13)
C56	31.2(16)	44(2)	31.7(18)	-3.1(15)	-10.9(14)	-2.3(15)
C57	44(2)	56(3)	23.4(16)	-9.1(16)	5.6(14)	4.2(18)
C58	39.0(18)	30.9(17)	39(2)	13.6(15)	9.1(15)	1.3(15)
C59	58(2)	38(2)	30.5(18)	7.5(15)	12.8(17)	-6.4(18)
C60A	32(2)	44(3)	82(4)	28(3)	-24(2)	-4(2)
C60B	58(2)	38(2)	30.5(18)	7.5(15)	12.8(17)	-6.4(18)
C61	20.0(12)	28.8(15)	24.3(14)	4.3(12)	5.9(10)	-0.7(11)
C62	23.7(14)	33.5(17)	32.7(17)	9.9(13)	5.1(12)	-0.9(13)
C63	33.3(17)	36.4(18)	31.1(17)	-5.7(14)	7.5(13)	1.9(14)
C64	30.2(15)	27.6(15)	19.3(13)	0.1(11)	-0.4(11)	-6.5(12)
C65	26.4(16)	34.4(18)	50(2)	2.5(16)	-2.6(15)	-7.9(14)
C66	36.4(17)	31.7(17)	34.0(18)	-8.7(14)	11.8(14)	-9.3(14)
C67	19.4(12)	29.0(15)	24.6(14)	-1.3(12)	5.8(10)	-0.9(12)
C68	19.3(12)	24.4(14)	27.7(15)	-0.9(12)	5.5(10)	1.9(11)
C69	19.1(12)	20.0(13)	20.4(13)	0.5(10)	4.9(10)	-0.2(10)
C70	17.9(12)	22.9(13)	18.7(12)	-0.1(10)	4.3(9)	-2.2(11)
C71	18.3(12)	26.4(14)	17.5(13)	2.5(10)	0.8(10)	-1.9(11)
C72	26.0(14)	42.0(19)	20.2(14)	7.7(13)	-2.0(11)	-10.2(14)
C73	43.1(18)	25.0(15)	23.5(15)	2.4(12)	13.5(13)	3.8(14)
C74	27.9(15)	31.7(16)	28.2(16)	3.2(13)	4.3(12)	-9.4(13)
C75	23.3(14)	27.7(15)	28.9(16)	2.8(13)	-0.6(11)	-1.9(12)

C76	30.6(16)	28.4(16)	36.0(18)	-2.9(14)	-4.7(13)	4.6(14)
C77	25.1(15)	28.9(16)	37.0(18)	-2.2(14)	-2.4(13)	1.3(13)
C78	27.2(15)	34.9(17)	29.8(17)	3.1(14)	-0.1(12)	-4.6(14)
C79	33.7(17)	37.3(18)	27.4(16)	2.0(14)	1.2(13)	-3.0(15)
C80	29.3(15)	32.2(17)	31.2(17)	-1.9(13)	-1.3(13)	2.1(13)
C81	40.5(19)	31.9(18)	35.8(19)	2.1(14)	2.0(15)	3.2(15)
Si7	20.8(4)	24.4(4)	18.6(4)	5.1(3)	-4.1(3)	0.4(3)
Si8	17.2(3)	23.9(4)	19.0(4)	0.4(3)	-1.1(3)	5.5(3)
O19	45.4(15)	58.0(19)	34.0(14)	15.2(13)	-23.7(12)	-24.9(14)
O20	45.4(14)	37.9(14)	16.4(10)	1.6(9)	-1.7(9)	-8.9(11)
O21	21.8(10)	25.7(10)	26.8(11)	3.2(9)	4.2(8)	5.7(9)
O22	22.1(10)	21.8(10)	23.9(10)	5.6(8)	-6.1(8)	0.0(8)
O23	22.3(10)	31.3(12)	17.9(10)	3.4(8)	-1.5(8)	7.5(9)
O24	15.0(9)	26.0(11)	23.5(10)	3.1(8)	-2.0(7)	1.7(8)
N4	15.8(10)	31.4(13)	21.0(12)	2.8(10)	-4.3(9)	4.5(10)
C1A	143(9)	99(6)	30(4)	19(4)	25(4)	75(6)
C1B	62(4)	37(3)	49(3)	0(2)	18(3)	2(3)
C1C	56(5)	59(5)	41(4)	-11(3)	12(3)	-5(4)
C82	24.9(14)	35.0(17)	17.8(13)	1.1(11)	2.4(11)	-4.4(12)
C83	29.8(16)	53(2)	25.8(16)	1.1(15)	8.2(13)	-1.7(16)
C84	31.7(16)	44(2)	20.8(15)	-2.0(13)	-3.2(12)	-6.4(15)
C85	40(2)	35(2)	61(3)	21.3(19)	-24.4(19)	-5.6(17)
C86A	33(3)	30(3)	42(3)	12(2)	5(2)	-3(2)
C86B	31(6)	38(7)	37(7)	7(5)	-7(5)	0(5)
C87	37.6(18)	30.7(17)	31.4(17)	6.4(14)	-11.1(14)	4.8(15)
C88	34.1(16)	27.5(16)	24.9(15)	-0.9(12)	3.0(12)	9.1(13)
C89	37.2(19)	36(2)	57(3)	-6.1(17)	20.3(18)	8.3(16)
C90	33.2(17)	33.4(17)	37.9(19)	-12.4(15)	-4.1(14)	8.0(15)

C91	19.2(13)	26.9(15)	29.9(16)	0.8(12)	-1.8(11)	3.0(11)
C92	28.1(16)	37.4(18)	41(2)	-8.3(16)	-2.8(14)	-3.5(15)
C93	27.9(16)	31.1(17)	38.9(19)	8.1(14)	3.5(13)	3.3(13)
C94	15.0(11)	26.2(14)	26.4(14)	1.7(11)	-0.9(10)	2.3(11)
C95	17.8(12)	22.3(13)	26.4(14)	-0.2(11)	-3.1(10)	-0.8(11)
C96	19.2(12)	22.0(13)	17.2(12)	2.6(10)	-3.1(9)	1.9(10)
C97	15.5(11)	22.5(13)	18.8(12)	0.6(10)	-2.6(9)	4.3(10)
C98	16.1(11)	26.3(14)	19.5(13)	3.3(11)	-0.2(9)	6.1(10)
C99	25.8(14)	39.2(17)	22.2(14)	7.3(13)	5.0(11)	10.2(14)
C100	36.4(16)	25.3(15)	22.5(15)	4.2(12)	-7.8(12)	-4.9(13)
C101	25.6(15)	31.5(17)	44(2)	3.9(15)	5.7(14)	9.0(13)
C102	27.5(16)	27.2(17)	59(2)	-3.9(16)	6.4(16)	3.4(14)
C103	102(5)	48(3)	74(4)	-22(3)	51(3)	-2(3)
C104	143(9)	99(6)	30(4)	19(4)	25(4)	75(6)
C105	62(4)	37(3)	49(3)	0(2)	18(3)	2(3)
C106	56(5)	59(5)	41(4)	-11(3)	12(3)	-5(4)
C107	80(4)	94(5)	54(3)	-6(3)	-7(3)	-36(4)
C108	71(3)	64(3)	63(3)	-8(3)	15(3)	-17(3

Table S4. Bond Lengths for mo_B2720_0m.

Atom	Atom	Length/Å	Atom	Atom	Length/Å
Si1	O4	1.651(2)	Si5	C55	1.871(3)
Si1	O5	1.641(2)	Si5	C58	1.866(4)
Si1	C1	1.878(3)	Si6	O17	1.640(2)
Si1	C4	1.867(3)	Si6	O18	1.649(2)
Si2	O5	1.635(2)	Si6	C61	1.862(3)
Si2	O6	1.649(2)	Si6	C64	1.867(3)
Si2	C7	1.869(3)	O13	C73	1.210(4)
Si2	C10	1.876(3)	O14	C72	1.435(4)
O1	C19	1.209(4)	O14	C73	1.360(5)

O2	C18	1.446(4)	O15	C67	1.406(4)
O2	C19	1.351(4)	O15	C74	1.430(4)
O3	C13	1.409(4)	O16	C69	1.440(4)
O3	C20	1.438(4)	O18	C70	1.417(3)
O4	C15	1.424(3)	N3	C67	1.451(4)
O6	C16	1.419(3)	N3	C71	1.448(4)
N1	C13	1.452(4)	N3	C73	1.354(4)
N1	C17	1.451(3)	C55	C56	1.537(5)
N1	C19	1.354(4)	C55	C57	1.539(5)
C1	C2	1.533(5)	C58	C59	1.520(5)
C1	C3	1.499(5)	C58	C60A	1.513(6)
C4	C5	1.546(5)	C58	C60B	1.46(3)
C4	C6	1.535(5)	C61	C62	1.542(4)
C7	C8	1.526(5)	C61	C63	1.537(5)
C7	C9	1.538(5)	C64	C65	1.531(5)
C10	C11	1.543(4)	C64	C66	1.532(5)
C10	C12	1.529(5)	C67	C68	1.524(5)
C13	C14	1.526(4)	C68	C69	1.531(4)
C14	C15	1.525(4)	C69	C70	1.522(4)
C15	C16	1.533(4)	C70	C71	1.528(4)
C16	C17	1.533(4)	C71	C72	1.539(4)
C17	C18	1.532(4)	C74	C75	1.516(5)
C20	C21	1.510(5)	C75	C76	1.520(4)
C21	C22	1.520(5)	C76	C77	1.531(5)
C22	C23	1.525(5)	C77	C78	1.513(5)
C23	C24	1.527(5)	C78	C79	1.537(5)
C24	C25	1.523(5)	C79	C80	1.522(5)

C25	C26	1.517(5)	C80	C81	1.518(5)
C26	C27	1.518(5)	Si7	O22	1.647(2)
Si3A	O10	1.645(2)	Si7	O23	1.646(2)
Si3A	O11	1.646(3)	Si7	C82	1.869(3)
Si3A	C28	1.880(4)	Si7	C85	1.864(4)
Si3A	C31A	1.889(6)	Si8	O23	1.640(2)
Si3A	C31B	1.893(11)	Si8	O24	1.647(2)
Si4	O11	1.632(2)	Si8	C88	1.870(3)
Si4	O12	1.648(2)	Si8	C91	1.867(3)
Si4	C34	1.873(3)	O19	C100	1.207(4)
Si4	C37	1.869(3)	O20	C99	1.437(4)
O7	C46	1.214(4)	O20	C100	1.365(5)
O8	C45	1.442(4)	O21	C94	1.407(4)
O8	C46	1.352(4)	O21	C101	1.434(4)
O9	C40	1.413(4)	O22	C96	1.427(3)
O9	C47	1.429(4)	O24	C97	1.419(3)
O10	C42	1.427(3)	N4	C94	1.462(4)
O12	C43	1.426(3)	N4	C98	1.450(4)
N2	C40	1.455(4)	N4	C100	1.347(4)
N2	C44	1.454(3)	C1A	C1B	1.480(10)
N2	C46	1.349(4)	C1A	C103	1.638(11)
C28	C29	1.536(5)	C1B	C1C	1.550(10)
C28	C30	1.545(5)	C1C	C107	1.380(11)
C31A	C32A	1.525(9)	C82	C83	1.537(5)
C31A	C33	1.465(7)	C82	C84	1.544(4)
C31B	C32B	1.518(17)	C85	C86A	1.469(7)
C31B	C33	1.687(12)	C85	C86B	1.348(14)
C34	C35	1.538(5)	C85	C87	1.519(5)
C34	C36	1.537(4)	C88	C89	1.534(5)
C37	C38	1.534(5)	C88	C90	1.528(5)

C37	C39	1.532(5)	C91	C92	1.535(5)
C40	C41	1.517(4)	C91	C93	1.538(5)
C41	C42	1.528(4)	C94	C95	1.515(4)
C42	C43	1.527(4)	C95	C96	1.529(4)
C43	C44	1.532(4)	C96	C97	1.524(4)
C44	C45	1.537(4)	C97	C98	1.527(4)
C47	C48	1.504(5)	C98	C99	1.529(4)
C48	C49	1.514(5)	C101	C102	1.508(5)
C49	C50	1.533(5)	C102	C103	1.512(7)
C50	C51	1.523(5)	C103	C104	1.47(2)
C51	C52	1.518(5)	C104	C105	1.21(3)
C52	C53	1.523(5)	C105	C106	1.51(3)
C53	C54	1.526(5)	C106	C107	1.96(2)
Si5	O16	1.652(2)	C107	C108	1.489(8)
Si5	O17	1.642(2)			

Table S5. Bond Angles for mo_B2720_0m.

Atom	Atom	Atom	Angle/˚	Atom	Atom	Atom	Angle/˚
O4	Si1	C1	104.95(14)	O17	Si5	O16	108.56(11)
O4	Si1	C4	109.60(14)	O17	Si5	C55	108.39(14)
O5	Si1	O4	108.56(11)	O17	Si5	C58	106.48(15)
O5	Si1	C1	107.22(14)	C58	Si5	C55	117.82(18)
O5	Si1	C4	107.24(14)	O17	Si6	O18	109.54(11)
C4	Si1	C1	118.91(16)	O17	Si6	C61	109.10(13)
O5	Si2	O6	109.87(11)	O17	Si6	C64	109.53(14)
O5	Si2	C7	109.70(13)	O18	Si6	C61	104.04(13)
O5	Si2	C10	108.99(13)	O18	Si6	C64	112.58(13)
O6	Si2	C7	103.66(12)	C61	Si6	C64	111.90(15)

O6	Si2	C10	112.94(13)	C73	O14	C72	110.4(2)
C7	Si2	C10	111.58(14)	C67	O15	C74	113.1(2)
C19	O2	C18	110.4(2)	C69	O16	Si5	124.77(19)
C13	O3	C20	112.1(2)	Si6	O17	Si5	130.58(14)
C15	O4	Si1	126.53(18)	C70	O18	Si6	132.1(2)
Si2	O5	Si1	131.00(13)	C71	N3	C67	119.9(2)
C16	O6	Si2	130.77(19)	C73	N3	C67	126.5(3)
C17	N1	C13	120.2(2)	C73	N3	C71	113.4(3)
C19	N1	C13	125.8(3)	C56	C55	Si5	113.4(3)
C19	N1	C17	113.1(2)	C56	C55	C57	110.3(3)
C2	C1	Si1	111.9(2)	C57	C55	Si5	112.9(3)
C3	C1	Si1	114.9(3)	C59	C58	Si5	113.8(3)
C3	C1	C2	112.3(3)	C60A	C58	Si5	114.6(3)
C5	C4	Si1	113.1(3)	C60A	C58	C59	110.5(4)
C6	C4	Si1	114.4(3)	C60B	C58	Si5	116.4(10)
C6	C4	C5	111.2(3)	C60B	C58	C59	128.7(10)
C8	C7	Si2	111.8(2)	C62	C61	Si6	111.5(2)
C8	C7	C9	111.4(3)	C63	C61	Si6	110.6(2)
C9	C7	Si2	110.4(2)	C63	C61	C62	110.9(3)
C11	C10	Si2	109.1(2)	C65	C64	Si6	110.7(2)
C12	C10	Si2	114.6(2)	C65	C64	C66	109.9(3)
C12	C10	C11	109.7(3)	C66	C64	Si6	113.7(2)
O3	C13	N1	110.2(2)	O15	C67	N3	110.5(3)
O3	C13	C14	108.1(2)	O15	C67	C68	107.5(2)
N1	C13	C14	109.0(2)	N3	C67	C68	109.1(2)
C15	C14	C13	113.0(2)	C67	C68	C69	111.8(2)
O4	C15	C14	107.1(2)	O16	C69	C68	107.2(2)

O4	C15	C16	110.7(2)	O16	C69	C70	110.9(2)
C14	C15	C16	109.9(2)	C70	C69	C68	110.5(2)
O6	C16	C15	112.0(2)	O18	C70	C69	112.3(2)
O6	C16	C17	106.6(2)	O18	C70	C71	106.3(2)
C15	C16	C17	108.7(2)	C69	C70	C71	108.3(2)
N1	C17	C16	110.0(2)	N3	C71	C70	109.7(2)
N1	C17	C18	101.2(2)	N3	C71	C72	101.0(2)
C18	C17	C16	115.0(2)	C70	C71	C72	115.2(3)
O2	C18	C17	105.6(2)	O14	C72	C71	105.9(3)
O1	C19	O2	122.6(3)	O13	C73	O14	123.1(3)
O1	C19	N1	128.0(3)	O13	C73	N3	127.8(4)
O2	C19	N1	109.4(3)	N3	C73	O14	109.1(3)
O3	C20	C21	109.5(3)	O15	C74	C75	108.9(3)
C20	C21	C22	114.1(3)	C74	C75	C76	112.6(3)
C21	C22	C23	114.0(3)	C75	C76	C77	114.5(3)
C22	C23	C24	114.5(3)	C78	C77	C76	113.9(3)
C25	C24	C23	114.3(3)	C77	C78	C79	114.5(3)
C26	C25	C24	113.1(3)	C80	C79	C78	115.1(3)
C25	C26	C27	114.0(3)	C81	C80	C79	114.0(3)
O10	Si3A	O11	108.21(12)	O22	Si7	C82	109.83(13)
O10	Si3A	C28	109.41(14)	O22	Si7	C85	103.86(15)
O10	Si3A	C31A	106.1(2)	O23	Si7	O22	109.02(12)
O10	Si3A	C31B	107.1(4)	O23	Si7	C82	107.14(13)
O11	Si3A	C28	108.41(15)	O23	Si7	C85	107.94(19)
O11	Si3A	C31A	103.2(2)	C85	Si7	C82	118.8(2)
O11	Si3A	C31B	117.5(3)	O23	Si8	O24	109.29(12)
C28	Si3A	C31A	120.9(2)	O23	Si8	C88	108.63(14)
C28	Si3A	C31B	106.1(4)	O23	Si8	C91	110.43(13)
O11	Si4	O12	109.74(12)	O24	Si8	C88	112.62(13)
O11	Si4	C34	109.98(14)	O24	Si8	C91	103.42(13)

O11	Si4	C37	108.89(14)	C91	Si8	C88	112.36(16)
O12	Si4	C34	112.53(13)	C100	O20	C99	109.8(2)
O12	Si4	C37	104.19(13)	C94	O21	C101	113.0(3)
C37	Si4	C34	111.33(15)	C96	O22	Si7	126.91(19)
C46	O8	C45	110.4(2)	Si8	O23	Si7	130.27(13)
C40	O9	C47	113.0(2)	C97	O24	Si8	131.5(2)
C42	O10	Si3A	125.68(18)	C98	N4	C94	119.9(2)
Si4	O11	Si3A	129.97(14)	C100	N4	C94	126.0(3)
C43	O12	Si4	129.07(19)	C100	N4	C98	112.8(3)
C44	N2	C40	119.9(2)	C1B	C1A	C103	109.9(7)
C46	N2	C40	126.4(3)	C1A	C1B	C1C	108.3(7)
C46	N2	C44	113.0(2)	C107	C1C	C1B	112.3(6)
C29	C28	Si3A	113.0(3)	C83	C82	Si7	113.7(2)
C29	C28	C30	110.0(3)	C83	C82	C84	111.0(3)
C30	C28	Si3A	113.2(3)	C84	C82	Si7	113.3(2)
C32A	C31A	Si3A	111.2(4)	C86A	C85	Si7	116.1(3)
C33	C31A	Si3A	114.2(4)	C86A	C85	C87	112.8(4)
C33	C31A	C32A	109.0(5)	C86B	C85	Si7	123.0(6)
C32B	C31B	Si3A	111.0(8)	C86B	C85	C87	122.0(6)
C32B	C31B	C33	113.9(9)	C87	C85	Si7	114.2(3)
C33	C31B	Si3A	104.1(6)	C89	C88	Si8	111.0(3)
C35	C34	Si4	115.0(3)	C90	C88	Si8	113.5(2)
C36	C34	Si4	109.3(2)	C90	C88	C89	109.8(3)
C36	C34	C35	108.7(3)	C92	C91	Si8	111.3(2)
C38	C37	Si4	110.9(2)	C92	C91	C93	111.0(3)
C39	C37	Si4	111.4(2)	C93	C91	Si8	111.1(2)
C39	C37	C38	111.1(3)	O21	C94	N4	110.2(3)

O9	C40	N2	110.1(2)	O21	C94	C95	107.8(2)
O9	C40	C41	107.3(2)	N4	C94	C95	109.4(2)
N2	C40	C41	109.5(2)	C94	C95	C96	112.2(3)
C40	C41	C42	112.1(2)	O22	C96	C95	107.0(2)
O10	C42	C41	107.3(2)	O22	C96	C97	111.1(2)
O10	C42	C43	111.0(2)	C97	C96	C95	110.3(2)
C43	C42	C41	109.7(2)	O24	C97	C96	111.9(2)
O12	C43	C42	112.2(2)	O24	C97	C98	106.8(2)
O12	C43	C44	106.1(2)	C96	C97	C98	108.6(2)
C42	C43	C44	108.9(2)	N4	C98	C97	109.7(2)
N2	C44	C43	109.6(2)	N4	C98	C99	101.2(2)
N2	C44	C45	101.1(2)	C97	C98	C99	115.4(3)
C43	C44	C45	115.1(2)	O20	C99	C98	105.8(3)
O8	C45	C44	105.6(2)	O19	C100	O20	122.4(3)
O7	C46	O8	122.5(3)	O19	C100	N4	128.1(4)
O7	C46	N2	127.9(3)	N4	C100	O20	109.5(3)
N2	C46	O8	109.6(3)	O21	C101	C102	108.5(3)
O9	C47	C48	109.1(3)	C101	C102	C103	114.2(4)
C47	C48	C49	114.1(3)	C102	C103	C1A	114.6(5)
C48	C49	C50	113.8(3)	C104	C103	C102	110.8(11)
C51	C50	C49	114.2(3)	C105	C104	C103	137(2)
C52	C51	C50	113.8(3)	C104	C105	C106	143(2)
C51	C52	C53	113.3(3)	C105	C106	C107	111.9(14)
C52	C53	C54	113.0(3)	C1C	C107	C108	114.3(6)
O16	Si5	C55	109.19(15)	C108	C107	C106	103.3(8)
O16	Si5	C58	106.09(15)				

Table S6. Torsion Angles for mo_B2720_0m.

A	B	C	D	Angle/˚	A	B	C	D	Angle/˚
Si1	O4	C15	C14	-154.3(2)	Si5	O16	C69	C68	-152.6(2)
Si1	O4	C15	C16	85.9(3)	Si5	O16	C69	C70	86.6(3)
Si2	O6	C16	C15	75.9(3)	Si6	O18	C70	C69	74.6(3)
Si2	O6	C16	C17	-165.38(19)	Si6	O18	C70	C71	-167.1(2)
O3	C13	C14	C15	-71.0(3)	O15	C67	C68	C69	-70.8(3)
O3	C20	C21	C22	-61.4(4)	O15	C74	C75	C76	-59.7(4)
O4	Si1	O5	Si2	17.9(2)	O16	Si5	O17	Si6	20.4(2)
O4	Si1	C1	C2	169.9(2)	O16	Si5	C55	C56	47.7(3)
O4	Si1	C1	C3	-60.6(4)	O16	Si5	C55	C57	174.1(2)
O4	Si1	C4	C5	-177.7(2)	O16	Si5	C58	C59	163.0(3)
O4	Si1	C4	C6	53.6(3)	O16	Si5	C58	C60A	-68.5(4)
O4	C15	C16	O6	-66.0(3)	O16	Si5	C58	C60B	-6.1(12)
O4	C15	C16	C17	176.5(2)	O16	C69	C70	O18	-65.0(3)
O5	Si1	O4	C15	-70.6(2)	O16	C69	C70	C71	178.0(2)
O5	Si1	C1	C2	54.6(3)	O17	Si5	O16	C69	-72.9(2)
O5	Si1	C1	C3	-175.9(3)	O17	Si5	C55	C56	165.8(2)
O5	Si1	C4	C5	-60.1(3)	O17	Si5	C55	C57	-67.8(3)
O5	Si1	C4	C6	171.3(3)	O17	Si5	C58	C59	47.5(3)
O5	Si2	O6	C16	-64.5(3)	O17	Si5	C58	C60A	176.0(4)
O5	Si2	C7	C8	-57.3(3)	O17	Si5	C58	C60B	-121.7(12)
O5	Si2	C7	C9	178.0(2)	O17	Si6	O18	C70	-64.0(3)
O5	Si2	C10	C11	-71.2(2)	O17	Si6	C61	C62	177.4(2)
O5	Si2	C10	C12	52.3(3)	O17	Si6	C61	C63	-58.7(3)
O6	Si2	O5	Si1	21.4(2)	O17	Si6	C64	C65	-72.8(3)
O6	Si2	C7	C8	60.0(2)	O17	Si6	C64	C66	51.5(3)
O6	Si2	C7	C9	-64.7(2)	O18	Si6	O17	Si5	20.1(2)
O6	Si2	C10	C11	166.43(19)	O18	Si6	C61	C62	-65.8(2)
O6	Si2	C10	C12	-70.1(3)	O18	Si6	C61	C63	58.1(2)
O6	C16	C17	N1	-175.7(2)	O18	Si6	C64	C65	165.1(2)
O6	C16	C17	C18	70.8(3)	O18	Si6	C64	C66	-70.6(3)
N1	C13	C14	C15	48.8(3)	O18	C70	C71	N3	-176.4(2)
N1	C17	C18	O2	-6.4(3)	O18	C70	C71	C72	70.4(3)
C1	Si1	O4	C15	175.0(2)	N3	C67	C68	C69	49.1(3)
C1	Si1	O5	Si2	130.8(2)	N3	C71	C72	O14	-3.9(3)
C1	Si1	C4	C5	61.6(3)	C55	Si5	O16	C69	45.1(3)
C1	Si1	C4	C6	-67.0(3)	C55	Si5	O17	Si6	-98.1(2)
C4	Si1	O4	C15	46.2(3)	C55	Si5	C58	C59	-74.4(3)
C4	Si1	O5	Si2	-100.4(2)	C55	Si5	C58	C60A	54.2(4)
C4	Si1	C1	C2	-67.1(3)	C55	Si5	C58	C60B	116.5(12)
C4	Si1	C1	C3	62.4(4)	C58	Si5	O16	C69	173.0(2)

C7	Si2	O5	Si1	134.69(19)	C58	Si5	O17	Si6	134.3(2)
C7	Si2	O6	C16	178.3(2)	C58	Si5	C55	C56	-73.3(3)
C7	Si2	C10	C11	50.1(2)	C58	Si5	C55	C57	53.1(3)
C7	Si2	C10	C12	173.6(2)	C61	Si6	O17	Si5	133.38(19)
C10	Si2	O5	Si1	-102.9(2)	C61	Si6	O18	C70	179.5(2)
C10	Si2	O6	C16	57.4(3)	C61	Si6	C64	C65	48.3(3)
C10	Si2	C7	C8	-178.2(2)	C61	Si6	C64	C66	172.7(2)
C10	Si2	C7	C9	57.2(3)	C64	Si6	O17	Si5	-103.8(2)
C13	O3	C20	C21	-171.7(2)	C64	Si6	O18	C70	58.1(3)
C13	N1	C17	C16	53.3(4)	C64	Si6	C61	C62	56.0(3)
C13	N1	C17	C18	175.4(3)	C64	Si6	C61	C63	179.9(2)
C13	N1	C19	O1	8.7(6)	C67	O15	C74	C75	176.0(3)
C13	N1	C19	O2	-171.7(3)	C67	N3	C71	C70	54.4(4)
C13	C14	C15	O4	-177.1(2)	C67	N3	C71	C72	176.5(3)
C13	C14	C15	C16	-56.8(3)	C67	N3	C73	O13	5.0(6)
C14	C15	C16	O6	175.9(2)	C67	N3	C73	O14	-173.6(3)
C14	C15	C16	C17	58.4(3)	C67	C68	C69	O16	-178.2(2)
C15	C16	C17	N1	-54.9(3)	C67	C68	C69	C70	-57.2(3)
C15	C16	C17	C18	-168.3(2)	C68	C69	C70	O18	176.3(2)
C16	C17	C18	O2	112.2(3)	C68	C69	C70	C71	59.3(3)
C17	N1	C13	O3	69.6(3)	C69	C70	C71	N3	-55.6(3)
C17	N1	C13	C14	-48.9(4)	C69	C70	C71	C72	-168.7(3)
C17	N1	C19	O1	177.7(4)	C70	C71	C72	O14	114.3(3)
C17	N1	C19	O2	-2.8(4)	C71	N3	C67	O15	67.7(4)
C18	O2	C19	O1	177.7(4)	C71	N3	C67	C68	-50.3(4)
C18	O2	C19	N1	-1.8(4)	C71	N3	C73	O13	178.4(4)
C19	O2	C18	C17	5.3(4)	C71	N3	C73	O14	-0.2(4)
C19	N1	C13	O3	-122.2(3)	C72	O14	C73	O13	178.7(4)
C19	N1	C13	C14	119.3(3)	C72	O14	C73	N3	-2.6(4)
C19	N1	C17	C16	-116.3(3)	C73	O14	C72	C71	4.1(4)
C19	N1	C17	C18	5.7(3)	C73	N3	C67	O15	-119.2(3)
C20	O3	C13	N1	72.0(3)	C73	N3	C67	C68	122.8(3)
C20	O3	C13	C14	-168.9(2)	C73	N3	C71	C70	-119.5(3)
C20	C21	C22	C23	-177.5(3)	C73	N3	C71	C72	2.6(4)
C21	C22	C23	C24	67.2(4)	C74	O15	C67	N3	65.4(3)
C22	C23	C24	C25	176.0(3)	C74	O15	C67	C68	-175.6(2)
C23	C24	C25	C26	177.5(3)	C74	C75	C76	C77	-174.9(3)
C24	C25	C26	C27	175.5(3)	C75	C76	C77	C78	-60.4(4)
Si3A	O10	C42	C41	-153.9(2)	C76	C77	C78	C79	-168.0(3)
Si3A	O10	C42	C43	86.2(3)	C77	C78	C79	C80	-55.2(4)
Si4	O12	C43	C42	76.2(3)	C78	C79	C80	C81	-58.8(4)
Si4	O12	C43	C44	-165.0(2)	Si7	O22	C96	C95	-155.4(2)
O9	C40	C41	C42	-69.9(3)	Si7	O22	C96	C97	84.2(3)
O9	C47	C48	C49	-59.2(4)	Si8	O24	C97	C96	77.3(3)
O10	Si3A	O11	Si4	18.3(2)	Si8	O24	C97	C98	-163.9(2)

O10	Si3A	C28	C29	173.3(3)	O21	C94	C95	C96	-71.0(3)
O10	Si3A	C28	C30	47.5(3)	O21	C101	C102	C103	-59.8(5)
O10	Si3A	C31A	C32A	-74.3(5)	O22	Si7	O23	Si8	20.8(2)
O10	Si3A	C31A	C33	161.8(4)	O22	Si7	C82	C83	51.6(3)
O10	Si3A	C31B	C32B	19.1(9)	O22	Si7	C82	C84	179.6(2)
O10	Si3A	C31B	C33	142.0(5)	O22	Si7	C85	C86A	-51.2(4)
O10	C42	C43	O12	-64.8(3)	O22	Si7	C85	C86B	5.4(9)
O10	C42	C43	C44	178.0(2)	O22	Si7	C85	C87	174.9(3)
O11	Si3A	O10	C42	-73.1(3)	O22	C96	C97	O24	-64.5(3)
O11	Si3A	C28	C29	-68.9(3)	O22	C96	C97	C98	177.8(2)
O11	Si3A	C28	C30	165.3(2)	O23	Si7	O22	C96	-71.4(3)
O11	Si3A	C31A	C32A	172.0(4)	O23	Si7	C82	C83	169.9(2)
O11	Si3A	C31A	C33	48.2(5)	O23	Si7	C82	C84	-62.1(3)
O11	Si3A	C31B	C32B	-102.8(8)	O23	Si7	C85	C86A	-166.9(4)
O11	Si3A	C31B	C33	20.1(7)	O23	Si7	C85	C86B	-110.3(9)
O11	Si4	O12	C43	-68.2(3)	O23	Si7	C85	C87	59.2(4)
O11	Si4	C34	C35	50.6(3)	O23	Si8	O24	C97	-65.6(3)
O11	Si4	C34	C36	-71.9(3)	O23	Si8	C88	C89	-77.4(3)
O11	Si4	C37	C38	179.2(2)	O23	Si8	C88	C90	46.8(3)
O11	Si4	C37	C39	-56.5(3)	O23	Si8	C91	C92	-56.6(3)
O12	Si4	O11	Si3A	24.6(2)	O23	Si8	C91	C93	179.1(2)
O12	Si4	C34	C35	-72.1(3)	O24	Si8	O23	Si7	19.0(2)
O12	Si4	C34	C36	165.4(2)	O24	Si8	C88	C89	161.4(2)
O12	Si4	C37	C38	-63.8(2)	O24	Si8	C88	C90	-74.4(3)
O12	Si4	C37	C39	60.5(3)	O24	Si8	C91	C92	60.2(3)
O12	C43	C44	N2	-176.1(2)	O24	Si8	C91	C93	-64.1(2)
O12	C43	C44	C45	70.8(3)	O24	C97	C98	N4	-176.3(2)
N2	C40	C41	C42	49.7(3)	O24	C97	C98	C99	70.2(3)
N2	C44	C45	O8	-4.2(3)	N4	C94	C95	C96	48.8(3)
C28	Si3A	O10	C42	44.9(3)	N4	C98	C99	O20	-9.5(3)
C28	Si3A	O11	Si4	-100.3(2)	C1A	C1B	C1C	C107	178.6(7)
C28	Si3A	C31A	C32A	50.8(6)	C1B	C1A	C103	C102	72.1(9)
C28	Si3A	C31A	C33	-73.1(5)	C1B	C1C	C107	C108	-170.4(6)
C28	Si3A	C31B	C32B	135.8(8)	C82	Si7	O22	C96	45.7(3)
C28	Si3A	C31B	C33	-101.2(5)	C82	Si7	O23	Si8	-98.0(2)
C31A	Si3A	O10	C42	176.8(3)	C82	Si7	C85	C86A	71.0(4)
C31A	Si3A	O11	Si4	130.4(3)	C82	Si7	C85	C86B	127.7(9)
C31A	Si3A	C28	C29	49.7(4)	C82	Si7	C85	C87	-62.9(4)
C31A	Si3A	C28	C30	-76.0(3)	C85	Si7	O22	C96	173.7(3)
C31B	Si3A	O10	C42	159.4(4)	C85	Si7	O23	Si8	133.0(2)
C31B	Si3A	O11	Si4	139.6(4)	C85	Si7	C82	C83	-67.6(3)
C31B	Si3A	C28	C29	58.1(5)	C85	Si7	C82	C84	60.4(3)
C31B	Si3A	C28	C30	-67.7(4)	C88	Si8	O23	Si7	-104.2(2)
C34	Si4	O11	Si3A	-99.7(2)	C88	Si8	O24	C97	55.2(3)
C34	Si4	O12	C43	54.6(3)	C88	Si8	C91	C92	-178.1(2)
C34	Si4	C37	C38	57.8(3)	C88	Si8	C91	C93	57.6(3)

C34	Si4	C37	C39	-177.9(2)	C91	Si8	O23	Si7	132.13(19)
C37	Si4	O11	Si3A	138.0(2)	C91	Si8	O24	C97	176.8(3)
C37	Si4	O12	C43	175.4(2)	C91	Si8	C88	C89	45.1(3)
C37	Si4	C34	C35	171.4(2)	C91	Si8	C88	C90	169.3(2)
C37	Si4	C34	C36	48.8(3)	C94	O21	C101	C102	-176.0(3)
C40	O9	C47	C48	-168.7(2)	C94	N4	C98	C97	53.3(4)
C40	N2	C44	C43	52.7(3)	C94	N4	C98	C99	175.7(3)
C40	N2	C44	C45	174.6(3)	C94	N4	C100	O19	9.6(6)
C40	N2	C46	O7	8.1(6)	C94	N4	C100	O20	-170.0(3)
C40	N2	C46	O8	-171.7(3)	C94	C95	C96	O22	-178.2(2)
C40	C41	C42	O10	-178.6(2)	C94	C95	C96	C97	-57.3(3)
C40	C41	C42	C43	-57.9(3)	C95	C96	C97	O24	177.1(2)
C41	C42	C43	O12	176.8(2)	C95	C96	C97	C98	59.4(3)
C41	C42	C43	C44	59.7(3)	C96	C97	C98	N4	-55.4(3)
C42	C43	C44	N2	-55.1(3)	C96	C97	C98	C99	-168.9(3)
C42	C43	C44	C45	-168.2(2)	C97	C98	C99	O20	108.9(3)
C43	C44	C45	O8	113.7(3)	C98	N4	C94	O21	69.1(3)
C44	N2	C40	O9	68.5(3)	C98	N4	C94	C95	-49.2(4)
C44	N2	C40	C41	-49.2(4)	C98	N4	C100	O19	176.6(4)
C44	N2	C46	O7	178.4(4)	C98	N4	C100	O20	-3.0(4)
C44	N2	C46	O8	-1.4(4)	C99	O20	C100	O19	176.6(4)
C45	O8	C46	O7	178.5(3)	C99	O20	C100	N4	-3.8(4)
C45	O8	C46	N2	-1.7(4)	C100	O20	C99	C98	8.6(4)
C46	O8	C45	C44	3.8(3)	C100	N4	C94	O21	-124.7(3)
C46	N2	C40	O9	-121.7(3)	C100	N4	C94	C95	117.0(3)
C46	N2	C40	C41	120.5(3)	C100	N4	C98	C97	-114.6(3)
C46	N2	C44	C43	-118.3(3)	C100	N4	C98	C99	7.8(4)
C46	N2	C44	C45	3.5(3)	C101	O21	C94	N4	70.2(3)
C47	O9	C40	N2	72.3(3)	C101	O21	C94	C95	-170.5(3)
C47	O9	C40	C41	-168.5(2)	C101	C102	C103	C1A	163.7(5)
C47	C48	C49	C50	-177.6(3)	C101	C102	C103	C104	-162.2(14)
C48	C49	C50	C51	69.1(4)	C102	C103	C104	C105	-126(4)
C49	C50	C51	C52	179.1(3)	C103	C1A	C1B	C1C	-178.4(6)
C50	C51	C52	C53	-179.4(3)	C103	C104	C105	C106	127(4)
C51	C52	C53	C54	175.0(3)	C104	C105	C106	C107	54(4)

Table S7. Hydrogen Atom Coordinates (Å×10^4) and Isotropic Displacement Parameters (Å^2×10^3) for mo_B2720_0m.

**Atom**	***x***	***y***	***z***	**U(eq)**
H1	11182.25	13488.53	8643.03	36
H2A	11981.44	13004.55	8342.15	49

H2B	11598.4	14565.05	8139.16	49
H2C	11569.13	12663.55	7952.13	49
H3A	10439.49	12786.74	7967.14	82
H3B	10551.87	14675.75	8154.85	82
H3C	10236.64	13190.91	8363.15	82
H4	11069.16	7593.46	8303.95	33
H5A	11410.74	7754.74	7733.25	64
H5B	11854.17	8781.12	8039.66	64
H5C	11406.13	9846.18	7743.12	64
H6A	10309.86	9799.84	7818.73	73
H6B	10116.23	8566.76	8136.4	73
H6C	10348.37	7720.08	7781.23	73
H7	12931.2	8302.09	9081.24	27
H8A	12132.11	5478.36	8911.63	47
H8B	12781.01	5523.25	8817.63	47
H8C	12301.8	6820.75	8605.45	47
H9A	12872.96	7637.54	9727.4	45
H9B	13173.27	6109.79	9522.01	45
H9C	12514.53	5910.71	9591.11	45
H10	12265.59	10256.26	9802.78	27
H11A	13076.47	10786.26	9499.1	39
H11B	12895.28	12565.5	9684.15	39
H11C	12727.19	12257.38	9241.98	39
H12A	11673.89	12892.83	9328.22	51
H12B	11883.14	13104.02	9769.59	51
H12C	11381.16	11740.09	9619.32	51
H13	9275.79	7708.18	9367.8	26
H14A	9612.17	9186.77	8842.51	25
H14B	9871.31	9988.34	9242.66	25
H15	10489.22	7688.02	8809.57	22

H16	10910.65	9236.48	9534.51	21
H17	10706.43	5557.64	9365.21	22
H18A	11107	4975.84	9952.05	31
H18B	11295.5	6994.28	10007.21	31
H20A	8815.47	5005.01	9164.51	34
H20B	9404.2	4239.06	9394.79	34
H21A	8983.08	2106.08	8975.86	35
H21B	9587.43	2699.99	8858.1	35
H22A	9096.61	4445.94	8373.38	36
H22B	8487.79	3938.96	8496.65	36
H23A	9225.42	1448.85	8211.35	40
H23B	8687.62	2357.63	7956.48	40
H24A	8596.17	6.98	8561.86	36
H24B	8050.42	989.61	8331.97	36
H25A	8176.55	-553.71	7783.47	37
H25B	8740.39	-1494.7	8002.7	37
H26A	7605.91	-1978.06	8193.12	39
H26B	8173.75	-2998.51	8382.04	39
H27A	7636.71	-3432.35	7622.63	55
H27B	7625.6	-4838.86	7950.12	55
H27C	8221.42	-4407.88	7799.7	55
H28	6871.99	2869.82	6684.01	36
H29A	7331.65	639.91	7292.48	69
H29B	7375.47	2727.41	7288.3	69
H29C	7723.41	1585.93	7024.13	69
H30A	5942.88	1918.04	6789.7	56
H30B	6282.39	2854.42	7155.22	56

H30C	6235.87	767.89	7138.33	56
H31A	6856.93	-3155.66	6390.57	29
H31B	6747.16	-1935.49	6990.89	29
H32A	6682.04	-2395.83	7138.93	59
H32B	6539.7	-4195.02	6917.58	59
H32C	6154.97	-2495.72	6799.33	59
H32D	6465.06	-3494.76	6266.37	77
H32E	6032.18	-3149.43	6563.31	77
H32F	6513	-4651.96	6639.63	77
H33A	7810.99	-2659.56	6598.75	58
H33B	7571.64	-4052.01	6869.21	58
H33C	7672	-2063.85	7001.36	58
H33D	7592.69	-3959.83	6915.81	58
H33E	7798.54	-1961.95	6932.54	58
H33F	7670.19	-2951.48	6537.57	58
H34	7326.72	-79.85	5200.89	32
H35A	6950.65	-2476.62	5724.57	62
H35B	7042.93	-3075.48	5312.32	62
H35C	6560.25	-1646.51	5364.38	62
H36A	8294.27	-536.28	5487.45	42
H36B	8023.99	-2341.42	5317.96	42
H36C	8086.36	-1980.47	5759.53	42
H37	8365.98	1964.06	5892.17	30
H38A	8012.14	2551.72	5249.34	47
H38B	8375.86	4168.89	5443.65	47
H38C	7682.19	4243.24	5371.1	47
H39A	7648.92	4804.46	6062.36	51

H39B	8343.02	4815.07	6134.49	51
H39C	7993.99	3526.2	6369.57	51
H40	4570.94	2303.89	5616.25	25
H41A	5253.58	92.46	5742.17	25
H41B	5159.08	869.97	6140.77	25
H42	6026.83	2524.73	6176.26	23
H43	6145.12	949.71	5460.72	23
H44	5977.01	4620.53	5619.54	23
H45A	6118.69	5182.33	5032.63	30
H45B	6284.47	3164.61	4979.71	30
H47A	4186.26	5001.12	5846.28	35
H47B	4642.13	5763.8	5595.89	35
H48A	4440.53	7889.18	6026.85	37
H48B	5109.56	7349.12	6110.06	37
H49A	4927.79	5586.35	6613.32	38
H49B	4252.15	6044.54	6525.29	38
H50A	4769.03	7674.35	7045.28	40
H50B	5120.76	8634.07	6756.44	40
H51A	4250.92	9911.29	6452.01	35
H51B	3893.77	8932.96	6735.55	35
H52A	4742.97	11578.06	6954.44	33
H52B	4391.65	10590.55	7240.14	33
H53A	3510.08	11817.42	6911.26	40
H53B	3888.51	12901.55	6655.99	40
H54A	3642.53	14700.01	7139.38	61
H54B	4331.58	14455.21	7193.21	61
H54C	3953.47	13369.76	7448.78	61
H55	15859.12	5212.62	8246.59	35
H56A	14892.18	6133.34	8181.95	56

H56B	15018.68	5300.65	7795.15	56
H56C	14984.24	7378.23	7839.04	56
H57A	15968.76	7372.05	7615.63	62
H57B	16041.62	5292.94	7638.85	62
H57C	16516.62	6529.3	7874.9	62
H58	16018.72	11175.37	8513.23	43
H58A	15692.9	10134.58	8049.83	43
H59A	16831.33	10536.41	8230.78	62
H59B	16461.52	12102.18	8019.3	62
H59C	16422.11	10183.93	7840.09	62
H60A	15419.32	12210.86	7986.34	84
H60B	15082.13	10846.02	8211.68	84
H60C	15309.4	10256.84	7833.46	84
H60D	15590.17	11515.83	8695.73	62
H60E	15157.17	11298.74	8309.52	62
H60F	15636.95	12810.54	8352.87	62
H61	17765.83	5836.42	8910.03	29
H62A	17895.95	5251.94	9556.47	45
H62B	17994.06	3470.62	9341.85	45
H62C	17397.75	3795.63	9500.15	45
H63A	16883.68	3191.53	8810.11	50
H63B	17513.24	2967.49	8690.75	50
H63C	17068.32	4367.91	8480.54	50
H64	17226.16	7969.7	9676.2	31
H65A	18016.9	8346	9358.89	57
H65B	17862.08	10197.18	9525.34	57
H65C	17674.62	9783.84	9088.67	57
H66A	16620.17	10585.47	9203.05	50
H66B	16894.76	10902.61	9632.02	50
H66C	16366.9	9564.72	9530.36	50
H67	14204.57	5694.91	9395.47	29
H68A	14795.99	7940.7	9240.72	28
H68B	14488	7149.08	8849.67	28
H69	15334.04	5538.66	8781.59	24
H70	15848.09	7086.87	9485.9	24
H71	15588.25	3431.65	9322.88	25
H72A	16061.59	2812.97	9892.78	36
H72B	16255.77	4825.99	9948.36	36
H74A	13818.75	2868.24	9287.19	35
H74B	14447.56	1975	9372.84	35

H75A	13816.66	154.82	8968.95	33
H75B	14333.17	818.02	8758.41	33
H76A	13725.99	2917.46	8429.33	39
H76B	13213.23	2419.32	8658.02	39
H77A	13112.56	-339.41	8360.09	37
H77B	13019.73	1197.91	8056.65	37
H78A	14017.72	-967.22	8219.29	37
H78B	14026.94	772.63	7975.11	37
H79A	13345.02	-383.19	7497.16	40
H79B	13871.99	-1727.48	7584.35	40
H80A	12767.86	-2140.76	7824.64	38
H80B	12989.3	-3240.78	7496.93	38
H81A	13721.82	-4509.83	7955.08	55
H81B	13079.92	-4990.01	8027.19	55
H81C	13442.6	-3513.13	8274.9	55
H10O	9401.24	8965.92	7072.38	107
H10P	9893.53	9834.57	6865.91	107
H10Q	8717.55	10959.73	6777.86	58
H10R	9201.18	11810.3	6560.14	58
H10S	9269.77	11792.66	7368.66	62
H10T	9762.67	12604.44	7155.87	62
H82	11642.97	4663.79	6772.41	31
H83A	10719.57	3628.23	6865	54
H83B	11046.3	4600.31	7229.9	54
H83C	11016.84	2510.62	7217.61	54
H84A	12145.63	4477.95	7377.79	49
H84B	12504.48	3439.05	7103.29	49
H84C	12128.39	2387.14	7365.57	49
H85	11678.22	-1194.51	6443.48	59
H85A	11403.07	-315.28	6884.11	59
H86A	10767.2	-839.3	6600.87	52
H86B	11109.13	-2501.74	6791.78	52
H86C	11049.67	-775.17	7033.76	52
H86D	11145.86	-2532.2	6699.04	55
H86E	11435.71	-2389.52	6325.1	55
H86F	10910.36	-1124.85	6384.12	55
H87A	12161.25	-2344.5	6994.16	52
H87B	12172.57	-464.53	7189.9	52
H87C	12520.66	-777.88	6845.6	52
H88	12309.02	2094.83	5330.75	35
H89A	13268.94	1735.64	5639.1	63
H89B	13038.76	-43.77	5435.41	63
H89C	13067.18	154.06	5879.77	63
H90A	11987.21	-621.23	5803.29	54
H90B	12007.45	-829.66	5363.71	54
H90C	11543.27	451.34	5505.62	54

H91	13255.9	4077.6	6107.08	31
H92A	12457.03	6739.57	6256.83	54
H92B	13146.31	6876.57	6367.89	54
H92C	12792.01	5448.56	6565.48	54
H93A	13042.55	4827.41	5467.14	49
H93B	13296.87	6503.26	5702.13	49
H93C	12614.51	6344.68	5561.26	49
H94	9470.35	4405.36	5581.62	28
H95A	10124.92	2163.34	5740.13	27
H95B	9977.14	2914.04	6129.27	27
H96	10857.49	4508.86	6226.24	24
H97	11077.3	3027.96	5522.72	23
H98	10897.92	6668.53	5696.46	25
H99A	11103.79	7367.62	5132.88	35
H99B	11330.92	5401.52	5090.96	35
H10G	9066.18	7105.83	5748.59	40
H10H	9612.1	7986.36	5598.09	40
H10I	9255.5	9901.63	6018.01	45
H10J	9899.99	9308.97	6184.86	45
H10K	9439.73	7108.06	6540.25	85
H10L	8857.15	8210.63	6430.14	85
H10M	8935.52	7559.24	6413.13	85
H10N	9589.83	7576.13	6626.95	85
H10A	9237.72	10815.06	6551.07	107
H10B	8718.7	9767.99	6675.07	107
H10C	9560.97	9395.28	7126.17	58
H10D	8927.54	10065.6	7129.94	58
H10E	9925.13	12100.32	7212.47	62
H10F	9521.58	11633.87	7520.72	62
H10U	8644.32	13788.47	7136.71	93
H10V	9069.17	14492.73	6861.47	93
H10W	9100.76	14390.93	6857.89	93
H10X	8640.71	13775.21	7123.57	93
H	8997.35	16093.02	7451.79	98
H10Y	9613.9	15943.11	7310.55	98
HA	9459.63	14672.5	7634.09	98

Table S8. Atomic Occupancy for mo_B2720_0m.

Atom	***Occupancy***	***Atom***	***Occupancy***	***Atom***	***Occupancy***
C31A	0.623(8)	H31A	0.623(8)	C31B	0.377(8)
H31B	0.377(8)	C32A	0.623(8)	H32A	0.623(8)
H32B	0.623(8)	H32C	0.623(8)	C32B	0.377(8)
H32D	0.377(8)	H32E	0.377(8)	H32F	0.377(8)
H33A	0.623(8)	H33B	0.623(8)	H33C	0.623(8)
H33D	0.377(8)	H33E	0.377(8)	H33F	0.377(8)

H58	0.839(9)	H58A	0.161(9)	C60A	0.839(9)
H60A	0.839(9)	H60B	0.839(9)	H60C	0.839(9)
C60B	0.161(9)	H60D	0.161(9)	H60E	0.161(9)
H60F	0.161(9)	C1A	0.723(7)	H10O	0.723(7)
H10P	0.723(7)	C1B	0.723(7)	H10Q	0.723(7)
H10R	0.723(7)	C1C	0.723(7)	H10S	0.723(7)
H10T	0.723(7)	H85	0.709(11)	H85A	0.291(11)
C86A	0.709(11)	H86A	0.709(11)	H86B	0.709(11)
H86C	0.709(11)	C86B	0.291(11)	H86D	0.291(11)
H86E	0.291(11)	H86F	0.291(11)	H10K	0.723(7)
H10L	0.723(7)	H10M	0.277(7)	H10N	0.277(7)
C104	0.277(7)	H10A	0.277(7)	H10B	0.277(7)
C105	0.277(7)	H10C	0.277(7)	H10D	0.277(7)
C106	0.277(7)	H10E	0.277(7)	H10F	0.277(7)
H10U	0.723(7)	H10V	0.723(7)	H10W	0.277(7)
H10X	0.277(7)				

Crystal structure determination of [mo_B2720_0m]

Crystal Data for $C_{27}H_{53}NO_6Si_2$ (*M* =543.88 g/mol): monoclinic, space group $P2_1$ (no. 4), *a* = 23.3220(12) Å, *b* = 7.6496(4) Å, *c* = 36.0472(18) Å, *β* = 98.683(2)°, *V* = 6357.3(6) Å^3, *Z* = 8, *T* = 100.00 K, μ(MoKα) = 0.148 mm^{-1}, *Dcalc* = 1.137 g/cm^3, 157288 reflections measured (3.612° ≤ 2Θ ≤ 61.108°), 38800 unique (R_{int} = 0.0417, R_{sigma} = 0.0413) which were used in all calculations. The final R_1 was 0.0545 (I > 2σ(I)) and wR_2 was 0.1432 (all data).

7. Mechanistic study

7.1. Unexpected observation of intermediate 5 between donor 1a and catalyst J

Procedure: To a dry NMR tube, filled with donor **1a** (2.15 mg, 0.005 mmol), catalyst **J** (12.4 mg, 0.005 mmol), 0.5 ml CD_2Cl_2, then 10 uL 1,1,2,2-tetrachloroethane was added as internal standard, the tube was measured under room temperature.

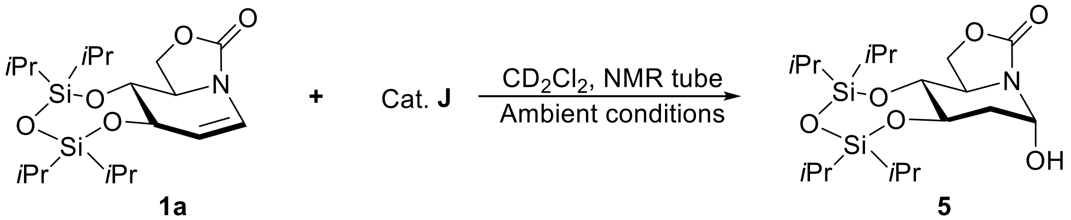

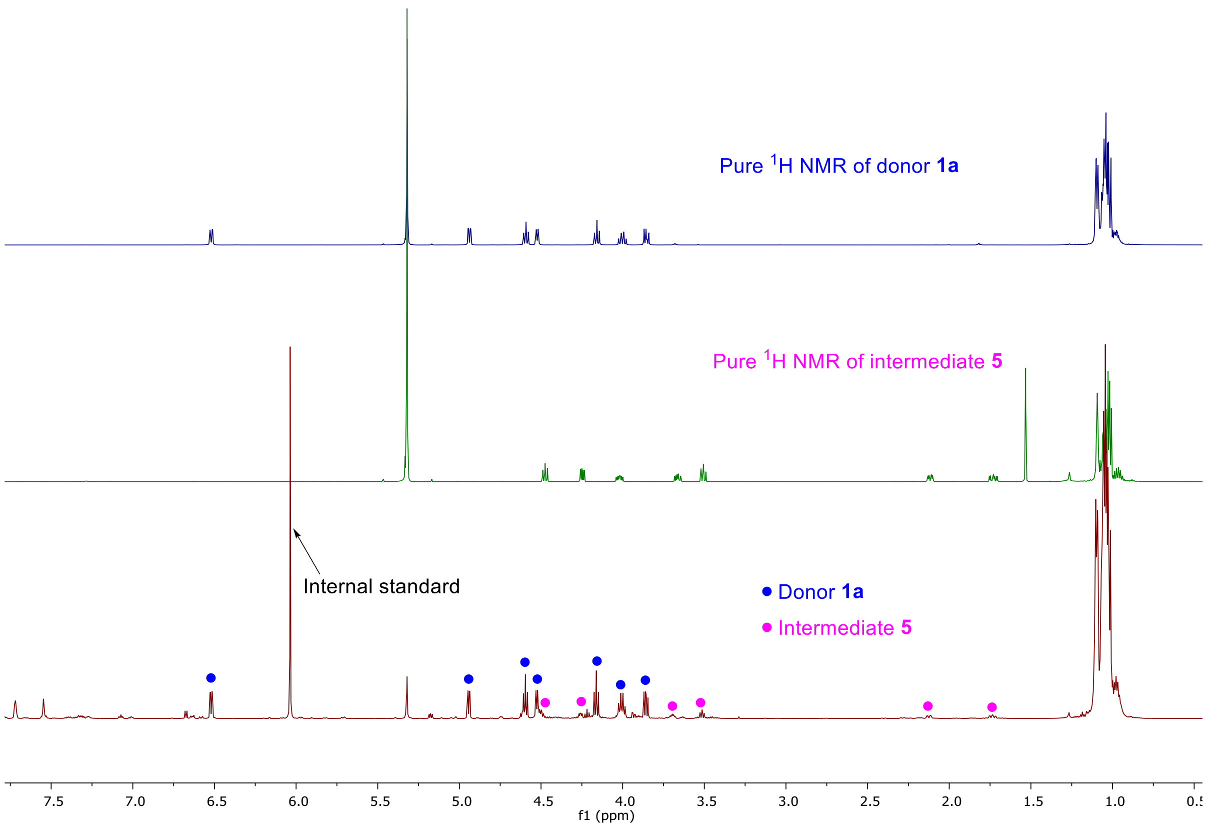

Supplementary Figure S2. Crude ^{1}H NMR spectra for observation of intermediate **5**

7.2.1. *In-situ* ^{1}H NMR monitoring experiment in NMR tube

Procedure: To a dry NMR tube was added donor **1a** (51.7 mg, 0.125 mmol), catalyst **J** (6.2 mg, 2.0 mol%) and 0.5 mL CD_2Cl_2, then acceptor **2n** (16.5 mg, 0.1875 mmol, 1.5 equiv.), 1,1,2,2-tetrachloroethane (10.0 μL) as the internal standard were added. Afterwards, the tube was sealed with cap and measured on the NMR spectrometer at room temperature by recording ^{1}H spectra at different time.

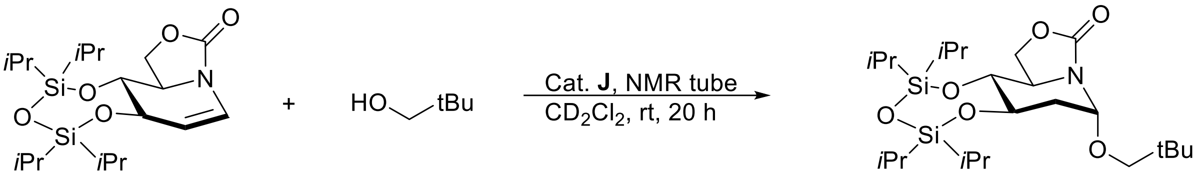

1a **2n** **3n**

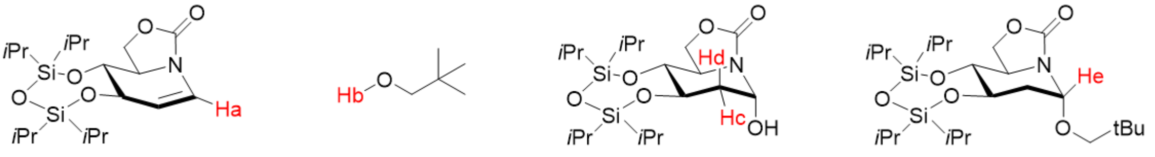

1a **2n** **5** **3n**

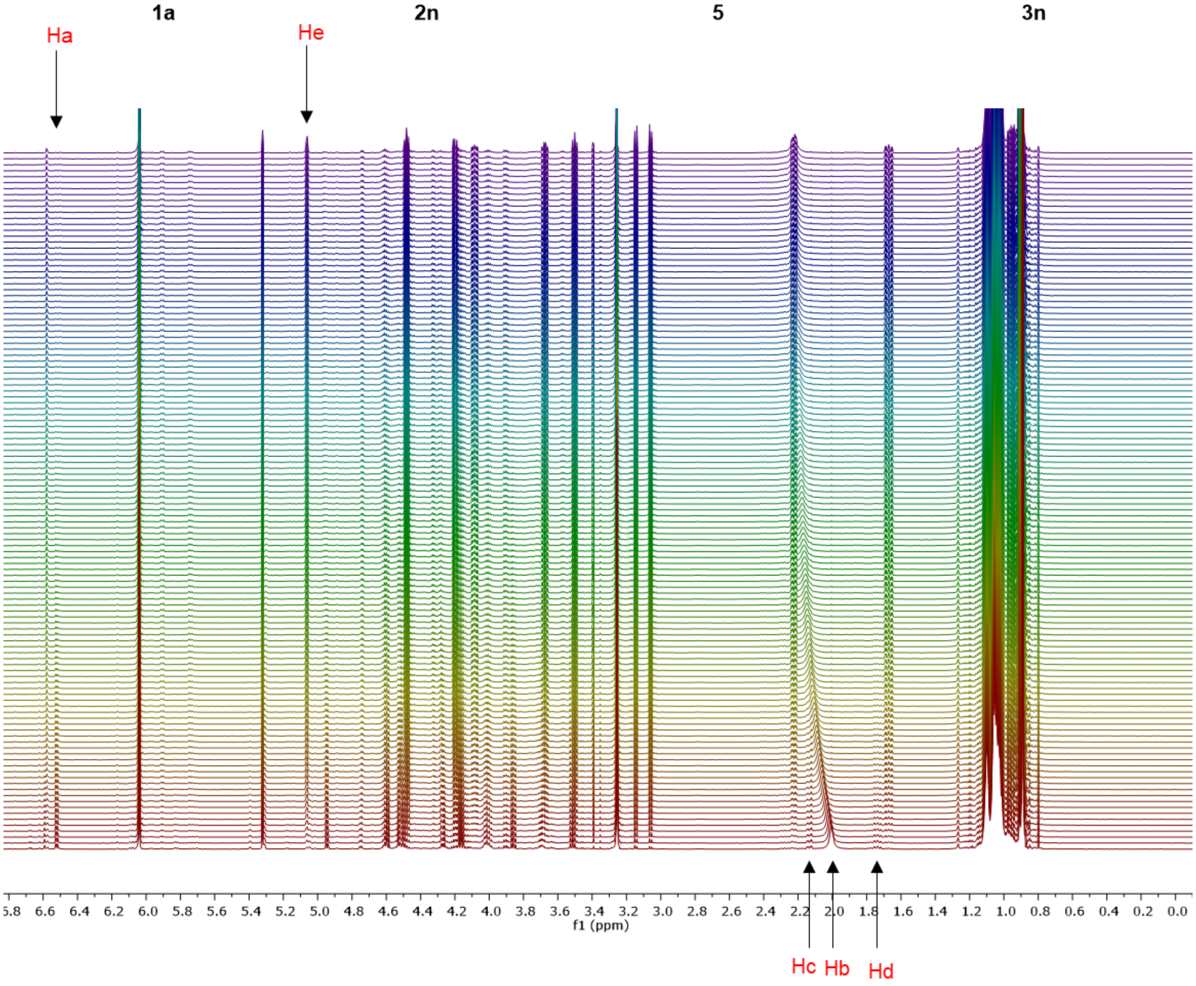

Supplementary Figure S3. Stacked ^{1}H NMR spectra for the monitoring under standard conditions

Table S9. Temporal concentrations for **1a**, **2n**, **3n** and **5** as calculated by ^{1}H NMR integrals

Time (h)	[1a]/M	[2n]/M	[3n]/M	[5]/M
0	0.25	0.375	0	0
0.13	0.222003	0.33871	0.011121	0.018225
0.25	0.201743	0.312202	0.012875	0.013566
0.5	0.195025	0.305612	0.018942	0.014359
0.75	0.188439	0.299252	0.024892	0.012705
1	0.181781	0.292816	0.030892	0.011468

1.25	0.174923	0.286254	0.036825	0.011573
1.5	0.16826	0.279745	0.042749	0.011197
1.75	0.16158	0.273433	0.048561	0.011216
2	0.155092	0.266863	0.054576	0.010097
2.25	0.148544	0.260557	0.060291	0.00993
2.5	0.142001	0.253918	0.066079	0.010142
2.75	0.135475	0.247576	0.071769	0.009096
3	0.129035	0.241245	0.077389	0.008281
3.25	0.122794	0.234904	0.082971	0.008134
3.5	0.116527	0.228676	0.088598	0.007787
3.75	0.110217	0.222442	0.09379	0.007781
4	0.104011	0.216317	0.099255	0.007864
4.25	0.098161	0.210344	0.10464	0.007852
4.5	0.092167	0.204374	0.109637	0.008394
4.75	0.086513	0.198518	0.114855	0.007624
5	0.080751	0.192818	0.119694	0.007083
5.25	0.075391	0.18731	0.124814	0.007521
5.5	0.070037	0.182007	0.12914	0.006983
5.75	0.064845	0.176571	0.13375	0.006068
6	0.059798	0.171489	0.137932	0.005794
6.25	0.055087	0.166573	0.142136	0.00715
6.5	0.050518	0.161839	0.146085	0.006971
6.75	0.046152	0.157486	0.149931	0.004558
7	0.041989	0.153182	0.153426	0.006157
7.25	0.038119	0.14922	0.156957	0.006456
7.5	0.034677	0.145306	0.160326	0.004559
7.75	0.031198	0.141929	0.163198	0.005859
8	0.027948	0.138614	0.166096	0.001736
8.25	0.025185	0.1355	0.168638	0.004832
8.5	0.022087	0.132224	0.170427	0.004682
8.75	0.019942	0.129888	0.172975	0.003571
9	0.017678	0.127506	0.174874	0.004853
9.25	0.015767	0.125269	0.17669	0.003054
9.5	0.01405	0.123287	0.178417	0.004541
9.75	0.012108	0.121173	0.179601	0.003247
10	0.010769	0.119509	0.180936	0.002015
10.25	0.00943	0.117814	0.181977	0.005072
10.5	0.008343	0.116452	0.182906	0.002811
10.75	0.007139	0.115394	0.183753	0.004406
11	0.006429	0.114352	0.184671	0.002785
11.25	0.005789	0.113732	0.185886	0.003197
11.5	0.00485	0.112988	0.186534	0.0035
11.75	0.004237	0.111411	0.186469	0.003964
12	0.003866	0.110924	0.186937	0.002821
12.25	0.003275	0.11084	0.187909	0.003351

12.5	0.003063	0.109398	0.187676	0.002768
12.75	0.002351	0.108781	0.187554	-0.00065
13	0.00236	0.108275	0.188111	0.0023
13.25	0.001998	0.107847	0.187949	0.001043
13.5	0.00182	0.107397	0.188477	0.002836
13.75	0.001567	0.107162	0.188616	0.002122
14	0.001367	0.106775	0.18866	0.002122
14.25	0.001339	0.106443	0.188851	0.00051
14.5	0.001082	0.106252	0.188857	0.002323
14.75	0.001106	0.106062	0.189084	0.001697
15	0.001037	0.10571	0.188997	0.001661
15.25	0.001087	0.105268	0.188966	0.001443
15.5	0.000176	0.105108	0.188801	0.001417
15.75	0.000801	0.104939	0.189309	0.000687
16	0.000695	0.104718	0.18914	0.001828
16.25	0.000645	0.104422	0.189239	0.000876
16.5	0.000605	0.104205	0.18915	0.001832
16.75	0.000607	0.104132	0.189136	-0.00224
17	0.000391	0.103909	0.189005	-0.00103
17.25	0.000445	0.103737	0.189095	0.001354
17.5	0.000482	0.104393	0.18994	0.002241
17.75	0.000437	0.10309	0.188937	0.001126
18	-0.00018	0.103116	0.188855	0.000461
18.25	0.000459	0.103067	0.189054	0.000867
18.5	0.000384	0.102917	0.189065	-0.00017
18.75	0.000197	0.102779	0.188959	0.000845
19	0.000419	0.102653	0.189115	0.001627
19.25	0.000467	0.102474	0.189066	-0.00297
19.5	0.000366	0.102356	0.189088	0.000155
19.75	0.000457	0.102176	0.189181	0.001367
20	0.000266	0.101866	0.188975	-1.1E-05

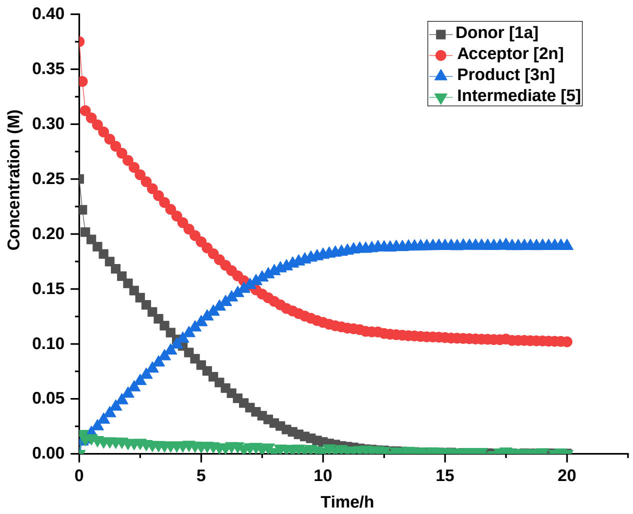

Supplementary Figure S4. Temporal kinetic profile of the standard reaction in NMR tube

7.2.2. *Sequential In-situ* reaction monitoring in NMR tube

Procedure: To a dry NMR tube was added donor **1a** (51.7 mg, 0.125 mmol, 1.0 equiv.), catalyst **J** (6.2 mg, 2.0 mol%), 0.5 mL CD_2Cl_2 and 1,1,2,2-tetrachloroethane (10.0 μL) as the internal standard were added, the tube was sealed with cap and measured on the NMR spectrometer every 5 mins at room temperature for 0.5 h, which showed around 10% intermediate **5** was formed. Afterwards, acceptor **2n** (16.5 mg, 0.1875 mmol, 1.5 equiv.) was added and the tube was sealed with cap again and measured on the NMR spectrometer under the same NMR device at room temperature by recording ^1H spectra every 15 mins for 20 h.

Frist step: Without **2n**

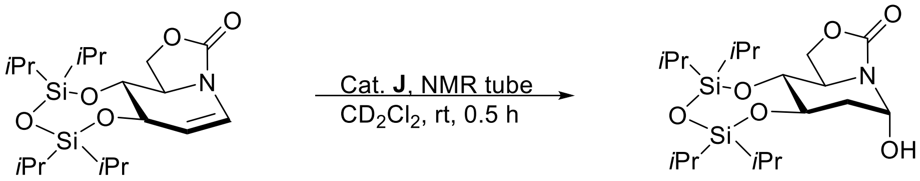

1a **5**

Second step: Add **2n** to the tube after 0.5 h

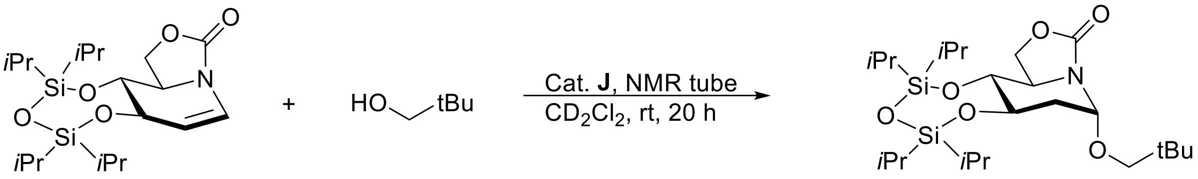

1a **2n** **3n**

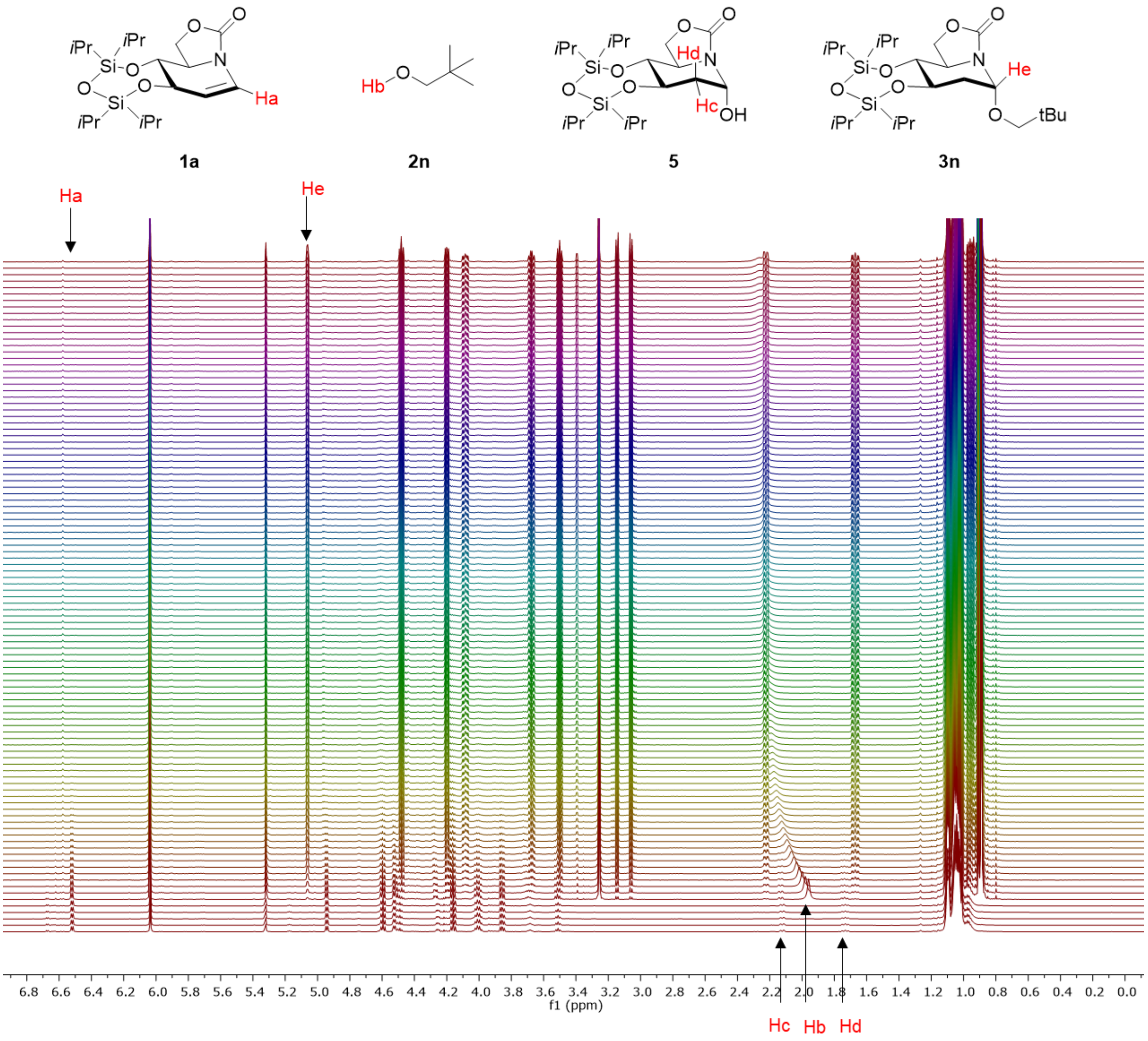

Supplementary Figure S5. Stacked ^{1}H NMR spectra for *sequential In-situ* reaction monitoring

Table S10. Temporal concentrations for **1a**, **2n**, **3n** and **5** as calculated by ^{1}H NMR integrals

Time (h)	[1a]/M	[2n]/M	[3n]/M	[5]/M
0	0.25			0
0.08	0.21793941			0.02756311
0.16	0.21784562			0.02748213
0.24	0.21792134			0.02759416
0.32	0.21789554			0.02750912
0.4	0.21789043	0.375	0	0.02748921
0.65	0.148923126	0.297826833	0.057967913	0.017086638
0.9	0.098300944	0.216415713	0.062368266	0.013724963
1.15	0.086522622	0.205676566	0.079288123	0.011475684
1.4	0.075778986	0.196847064	0.09426899	0.008799367
1.65	0.067009185	0.188856323	0.110055443	0.005757983
1.9	0.058190484	0.181494086	0.121157546	0.006238767
2.15	0.051160494	0.174916176	0.131849906	0.004434955
2.4	0.041493283	0.167094808	0.135440004	0.002869941

2.65	0.037938683	0.162236814	0.147505963	0.00288531
2.9	0.034374891	0.15775962	0.156359187	0.002576887
3.15	0.030067931	0.153783986	0.162712077	0.002754359
3.4	0.025516287	0.150433302	0.167634674	0.000976147
3.65	0.02384046	0.148066681	0.174864599	0.00112224
3.9	0.021195616	0.144531909	0.178012036	0.001587689
4.15	0.018167636	0.142261906	0.18068297	0.001336932
4.4	0.015968486	0.140772809	0.18451465	0.001128069
4.65	0.01561672	0.138867946	0.188628852	0.000579535
4.9	0.014260012	0.137152004	0.1901031	0.000699625
5.15	0.012831807	0.135518154	0.192086487	0.000428046
5.4	0.011912299	0.135023074	0.195102853	0.002760796
5.65	0.010214109	0.133401732	0.195154462	0.00156636
5.9	0.009262432	0.132176394	0.195504445	0.001485242
6.15	0.008063544	0.131418809	0.196250319	0.001296382
6.4	0.00825642	0.131438964	0.199610305	0.001111458
6.65	0.007814833	0.129650021	0.198350946	0.000935575
6.9	0.006940447	0.129711492	0.200183058	0.000345838
7.15	0.006167675	0.1284195	0.198990353	0.002301824
7.4	0.006348001	0.127677089	0.19979283	0.002239964
7.65	0.00609651	0.127533893	0.201135196	0.001763944
7.9	0.005690105	0.127970956	0.202729921	-0.00136187
8.15	0.002987097	0.126285514	0.198969478	-0.007653191
8.4	0.004708935	0.126657776	0.202474965	-0.001827931
8.65	0.004747147	0.126064947	0.202805467	-0.00113438
8.9	0.005542133	0.126015908	0.204049898	0.001576733
9.15	0.005510536	0.124936719	0.202568387	-0.004269308
9.4	0.004157725	0.125139666	0.203717253	-0.001872328
9.65	0.004826394	0.124873718	0.204245275	0.001287336
9.9	0.003934003	0.125515797	0.204930919	0.000394994
10.15	0.003971232	0.124336808	0.204103895	-0.000917184
10.4	0.003459763	0.124680354	0.204478955	-0.000629985
10.65	0.003852789	0.123649045	0.204420465	-0.002102571
10.9	0.004937404	0.122571308	0.20316993	-0.006412447
11.15	0.003844444	0.122290635	0.202215425	-0.006265259
11.4	0.003842534	0.124272807	0.206810046	0.003229138
11.65	0.003334411	0.122806231	0.204262551	-0.001924104
11.9	0.003362805	0.12256627	0.204360483	-0.001428547
12.15	0.002636117	0.122331476	0.203792182	-0.001140523
12.4	0.003844524	0.121404211	0.202956863	-0.005878159
12.65	0.003767973	0.123076023	0.206986133	0.003675357
12.9	0.0033704	0.121779711	0.204976074	-0.002769621
13.15	0.003308693	0.120505297	0.202344798	-0.006711405
13.4	0.003451952	0.119973395	0.202430591	-0.007874925
13.65	0.003485348	0.121391461	0.205177133	0.001046663

13.9	0.00433966	0.120920672	0.205448685	-0.00132465
14.15	0.00433019	0.120597074	0.204999003	0.000717506
14.4	0.004059033	0.120438163	0.205171533	-3.16399E-06
14.65	0.003951339	0.120333903	0.20538759	-0.000198203
14.9	0.003986308	0.119130416	0.202536696	-0.007330043
15.15	0.003864652	0.120807819	0.20665867	0.002855246
15.4	0.004050217	0.119701578	0.20553205	0.000201384
15.65	0.002648577	0.119694537	0.204304978	-0.002453941
15.9	0.003017574	0.119417823	0.204309325	-0.00176387
16.15	0.003279986	0.120219352	0.206690676	0.002948263
16.4	0.003826818	0.118970581	0.204720913	0.000859752
16.65	0.003252355	0.117830213	0.202501372	-0.007536952
16.9	0.003993285	0.118357075	0.204430541	-0.000614619
17.15	0.003722753	0.118281044	0.204542304	-0.001041846
17.4	0.003376266	0.117271928	0.202303405	-0.006756772
17.65	0.003150583	0.117106472	0.202107536	-0.006177869
17.9	0.002930166	0.116811393	0.202164854	-0.007392409
18.15	0.002974256	0.116859272	0.202525626	-0.00642389
18.4	0.004256673	0.116835285	0.202794394	-0.006113652
18.65	0.002284137	0.117584093	0.204087082	-0.001239555
18.9	0.003399968	0.116262791	0.202235378	-0.006901611
19.15	0.004081245	0.116177632	0.202899442	-0.006800301
19.4	0.002580039	0.117249181	0.204766303	-0.000741548
19.65	0.00293384	0.116815056	0.203938771	-0.002980937
19.9	0.00293384	0.116815056	0.203938771	-0.002980937

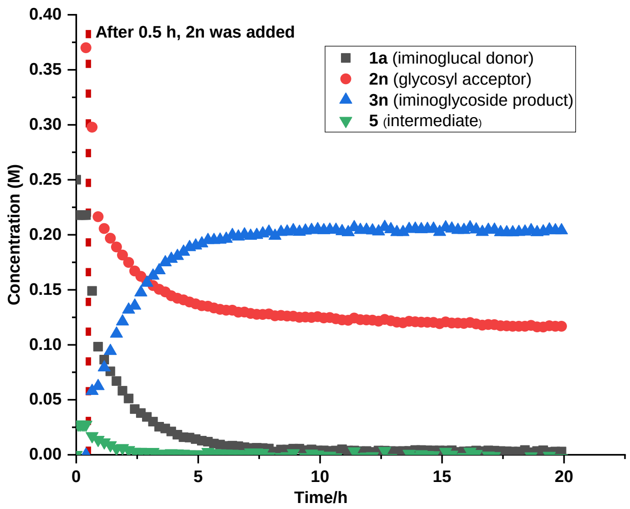

Supplementary Figure S6. Temporal kinetic profile of *sequential In-situ* reaction monitoring

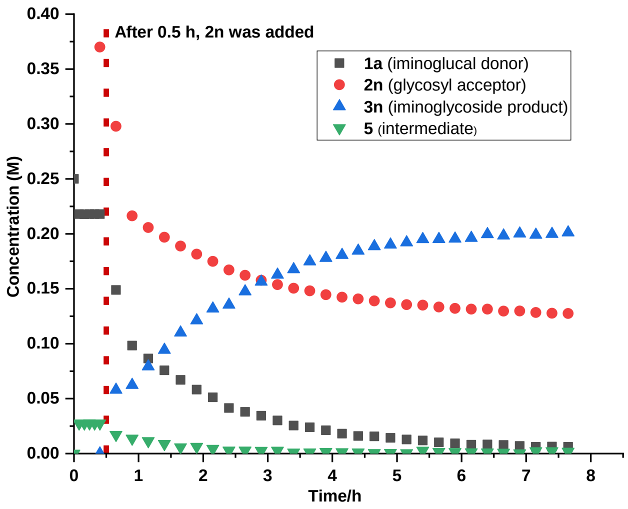

Supplementary Figure S7. Zoomed-in temporal kinetic profile of **supplementary figure S5** for clarity

7.3. Subjecting intermediate to standard conditions

Procedure: To an oven dried dram vial purged with an argon balloon was charged with donor **5** (21.5 mg, 0.05 mmol), acceptor **2a** (9.8 mg, 0.075 mmol, 1.5 equiv.), catalyst **J** (2.5 mg, 2.0 mol%), dry 0.2 ml CH_2Cl_2. The dram vial was sealed and stirred at room temperature for 10 h. The reaction mixture was filtered over a short silica plug and flushed with 10 mL of DCM. The filtrate was then evaporated and the determination of the anomeric selectivity (α/β) is by ^{1}H-NMR analysis of this concentrated crude mixture with 1,3,5-trimethoxybenzene as the internal standard. The crude mixture is subsequently loaded onto silica gel and subjected to flash column chromatography for purification to obtain product **3a** as white solid (24.4 mg, 90% yield).

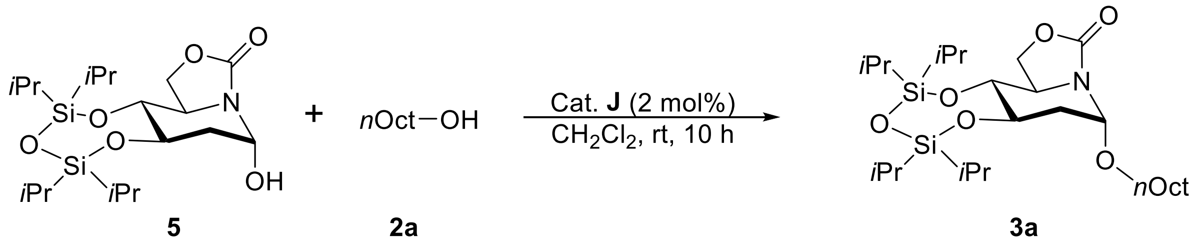

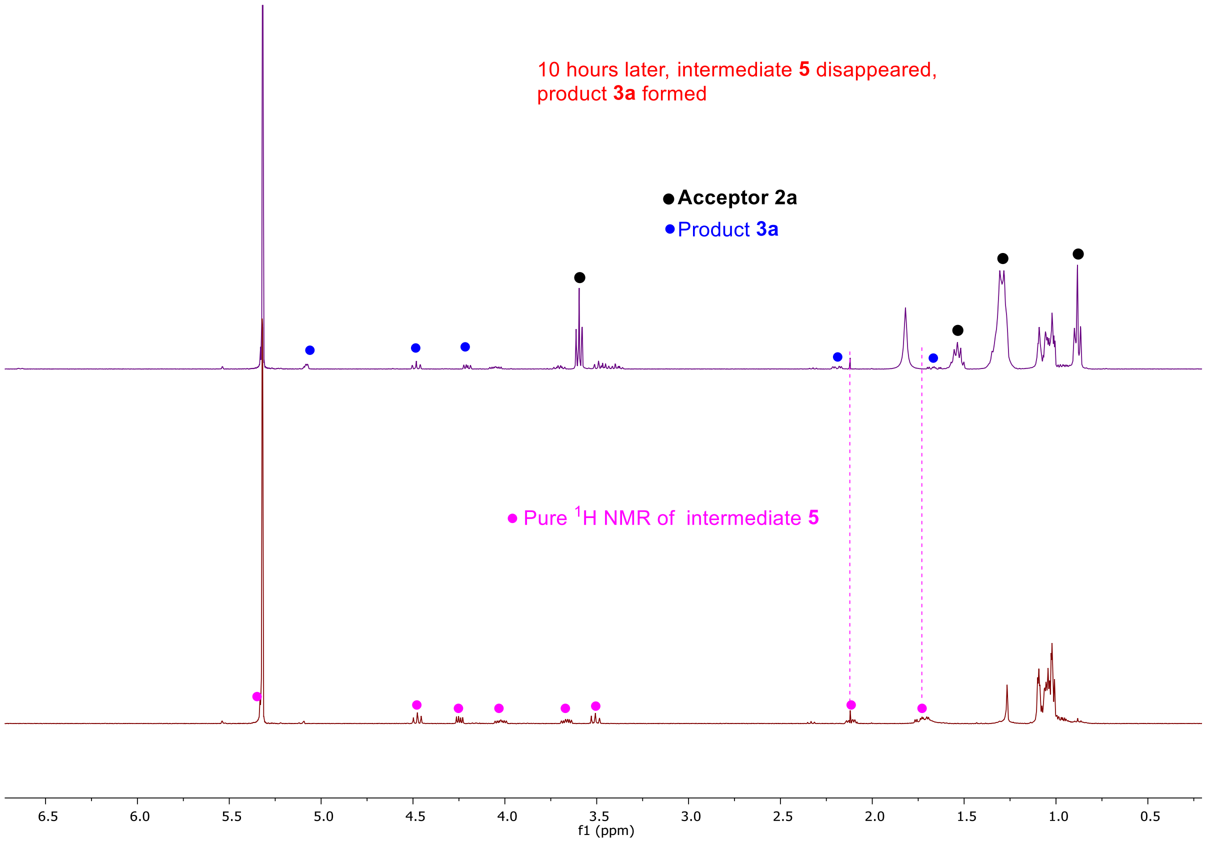

Supplementary Figure S8. ^{1}H NMR Spectra of intermediate **5** react with **2a** under standard condition

7.4. Control experiments on intermediate

7.4.1. Without catalyst J between intermediate 5 and 2a

Procedure: To an oven dried dram vial purged with an argon balloon was charged with donor **5** (8.6 mg, 0.02 mmol), acceptor **2a** (3.9 mg, 0.03 mmol, 1.5 equiv.), dry 0.1 ml CH_2Cl_2. The dram vial was sealed and stirred at room temperature for 10 h. The reaction mixture was filtered over a short silica plug and flushed with 10 mL of DCM. The filtrate was then evaporated and the determination of the anomeric selectivity (α/β) is by ^{1}H-NMR analysis of this concentrated crude mixture with 1,1,2,2-tetrachloroethane as the internal standard.

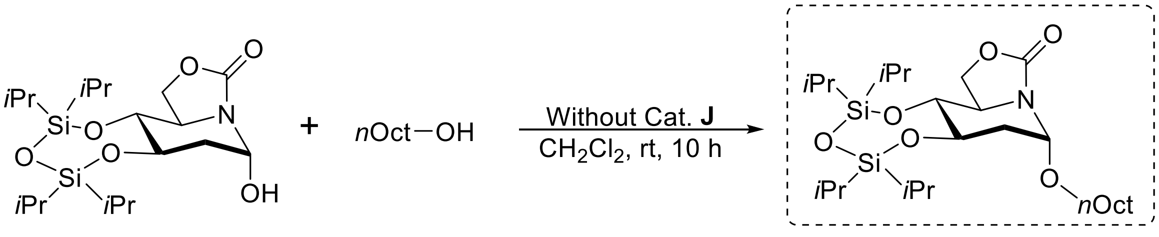

5 **2a** **3a**

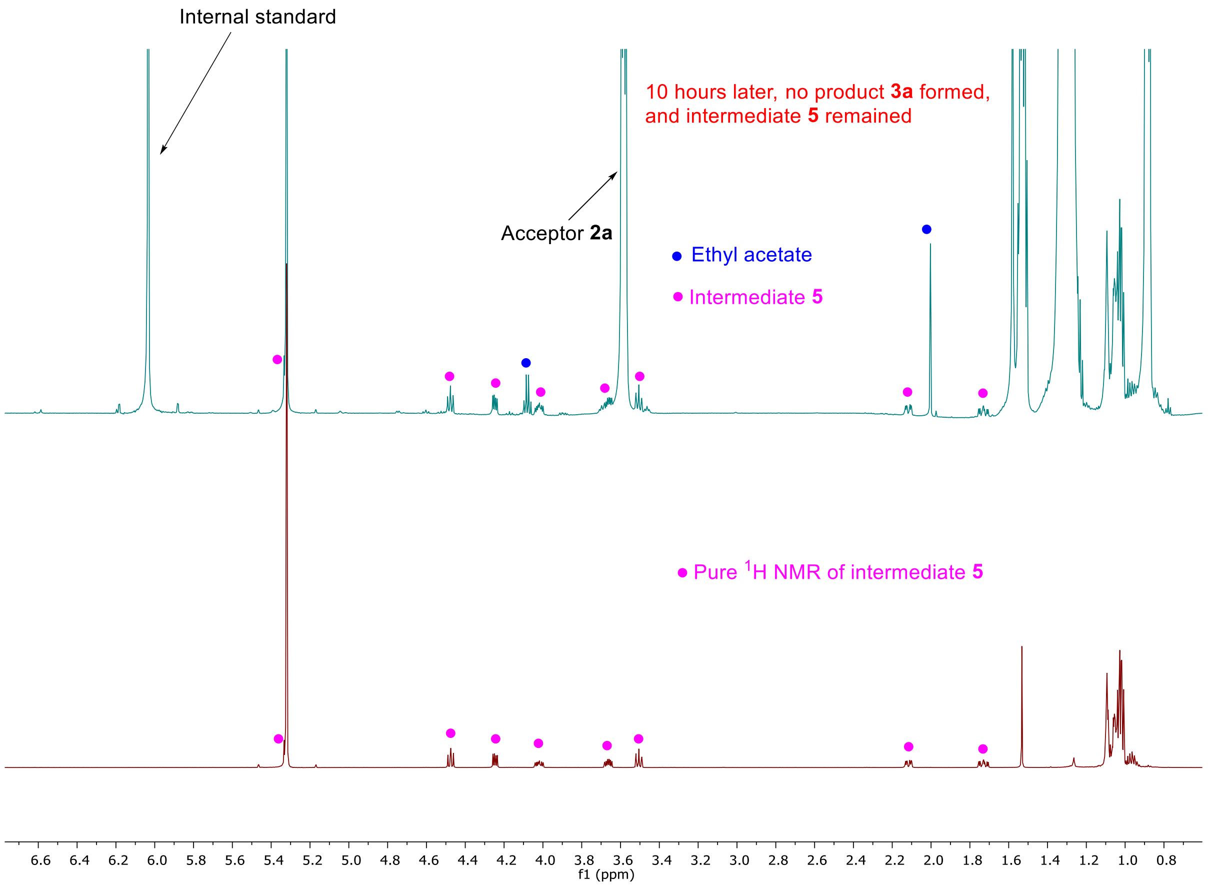

Supplementary Figure S9. Crude ^{1}H NMR spectra of intermediate **5** react with **2a** without catalyst **J**

7.4.2. Poisoning control on intermediate 5

Procedure: To an oven dried dram vial purged with an argon balloon was charged with donor **5** (8.6 mg, 0.02 mmol), acceptor **2a** (3.9 mg, 0.03 mmol, 1.5 equiv.), catalyst **J** (1.0 mg, 2.0 mol%), racemic (2,2′-bis(diphenylphosphino)-1,1′-binaphthyl) (2.5 mg, 0.004 mmol, 20 mol%) and dry 0.2 ml CH_2Cl_2. The dram vial was sealed and stirred at room temperature for 10 h. The reaction mixture was filtered over a short silica plug and flushed with 10 mL of DCM. The filtrate was then evaporated and the determination of the anomeric selectivity (α/β) is by ^{1}H-NMR analysis of this concentrated crude mixture with 1,1,2,2-tetrachloroethane as the internal standard.

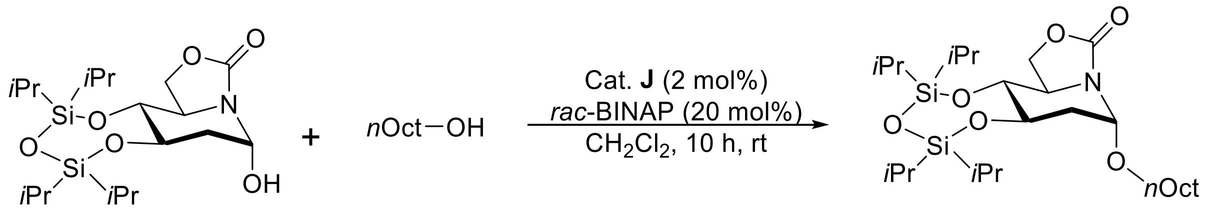

5 **2a** **3a**

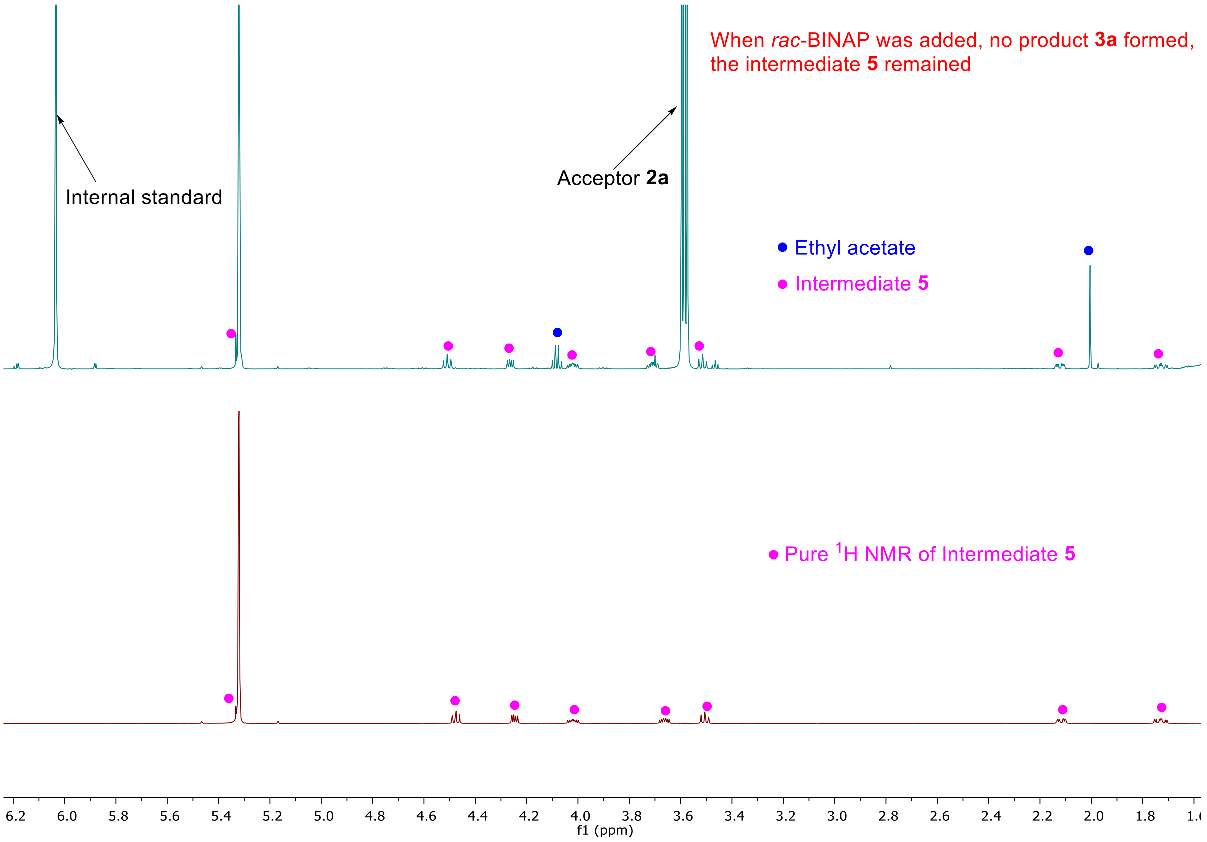

Supplementary Figure S10. Crude ^1H NMR spectra of poisoning experiment with *rac*-BINAP

7.5. Investigating the H_2O addition upstream step

Procedure: To an oven dried dram vial purged with an argon balloon was charged with donor **1a** (20.6 mg, 0.05 mmol), a drop of water, catalyst **J** (2.5 mg, 2.0 mol%), dry 0.2 ml CH_2Cl_2. The dram vial was sealed and stirred at room temperature for 0.5 h. The reaction mixture was filtered over a short silica plug and flushed with 10 mL of DCM. The filtrate was then evaporated and the determination of the anomeric selectivity (α/β) is by ^1H-NMR analysis of this concentrated crude mixture with 1,1,2,2-tetrachloroethane as the internal standard.

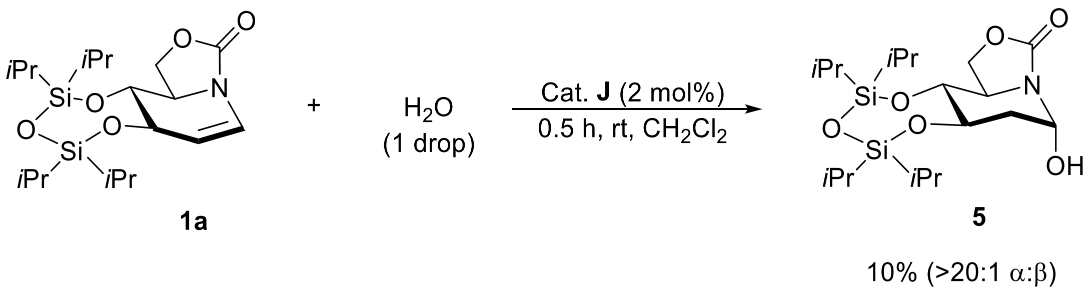

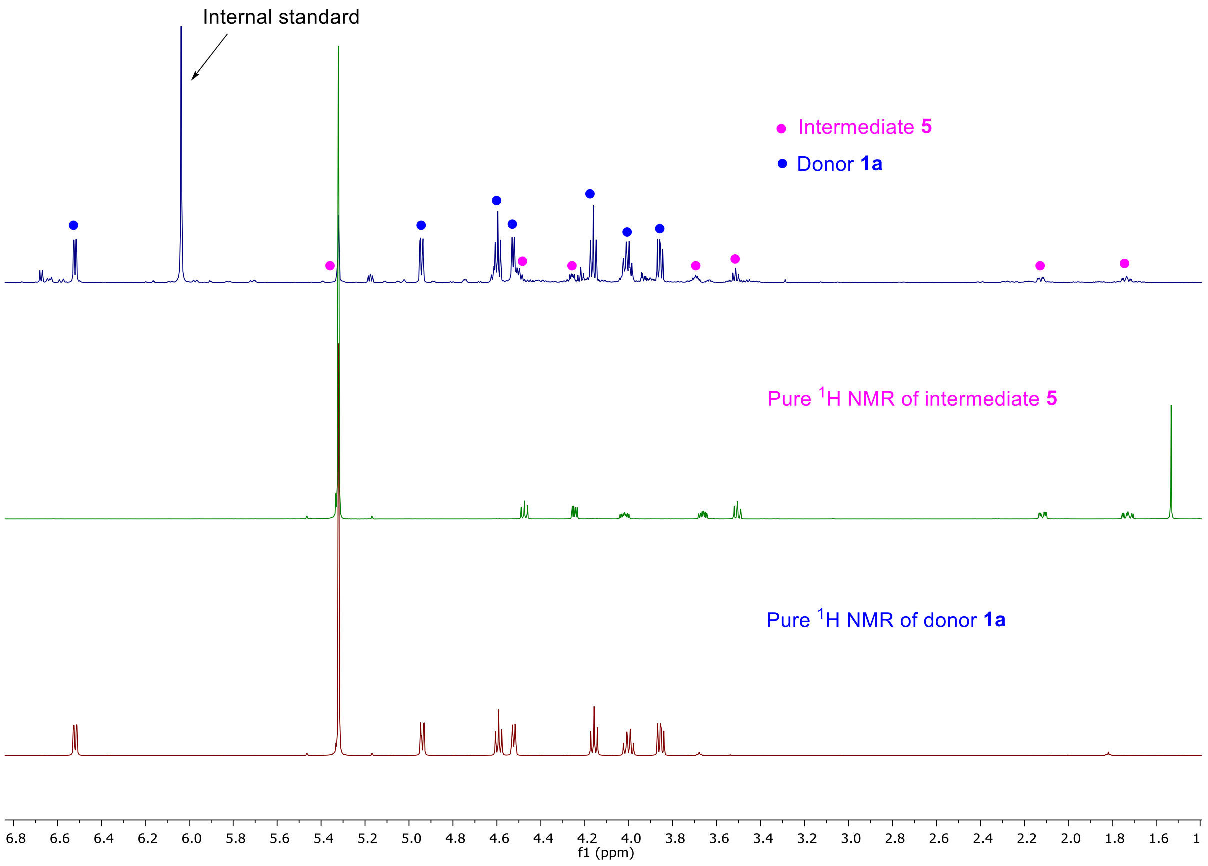

Supplementary Figure S11. Crude ^{1}H NMR spectra of H_2O addition to the upstream step

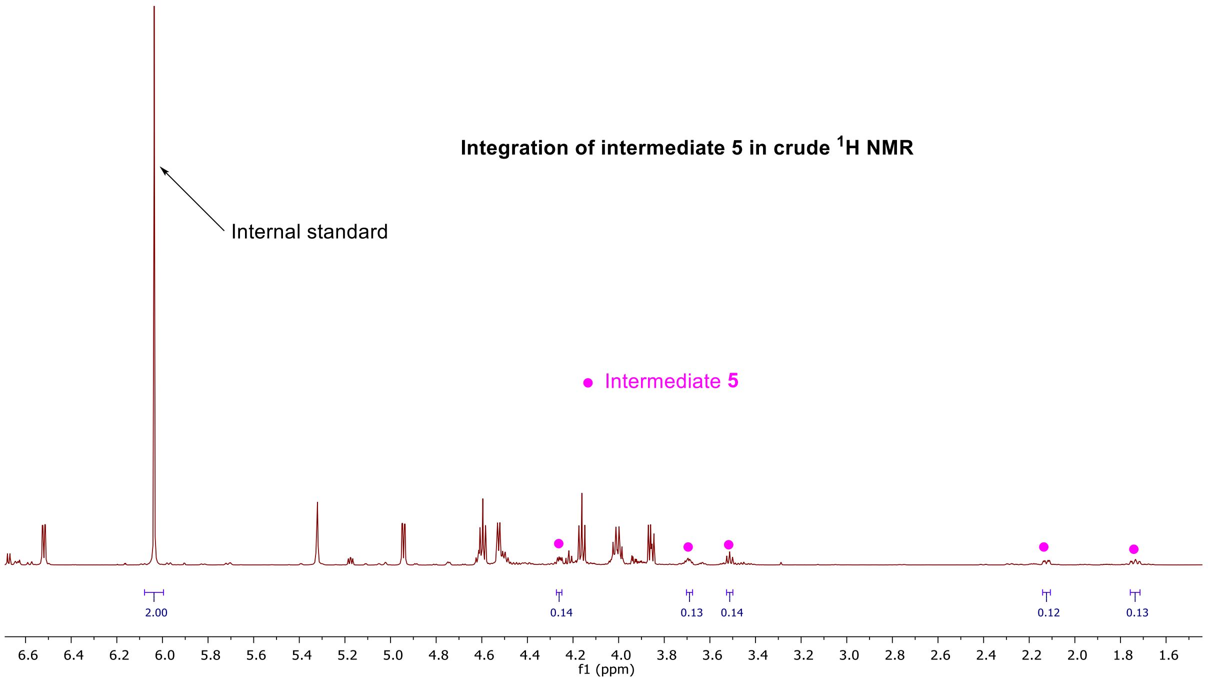

Supplementary Figure S12. Integration of intermediate **5** of the above spectra

7.6. Poisoning control on the upstream step

Procedure: To an oven dried dram vial purged with an argon balloon was charged with donor **1a** (20.6 mg, 0.05 mmol), a drop of water, catalyst **J** (2.5 mg, 2.0 mol%), racemic (2,2′-bis(diphenylphosphino)-1,1′-binaphthyl) (6.2 mg, 0.01 mmol, 20 mol%), dry 0.2 ml CH_2Cl_2. The dram vial was sealed and stirred at room temperature for 3 h. The reaction mixture was filtered over a short silica plug and flushed with 10 mL of DCM. The filtrate was then evaporated and the determination of the anomeric selectivity (α/β) is by ^{1}H-NMR analysis of this concentrated crude mixture with 1,1,2,2-tetrachloroethane as the internal standard.

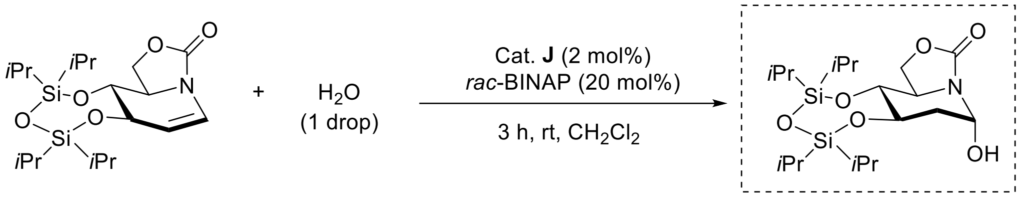

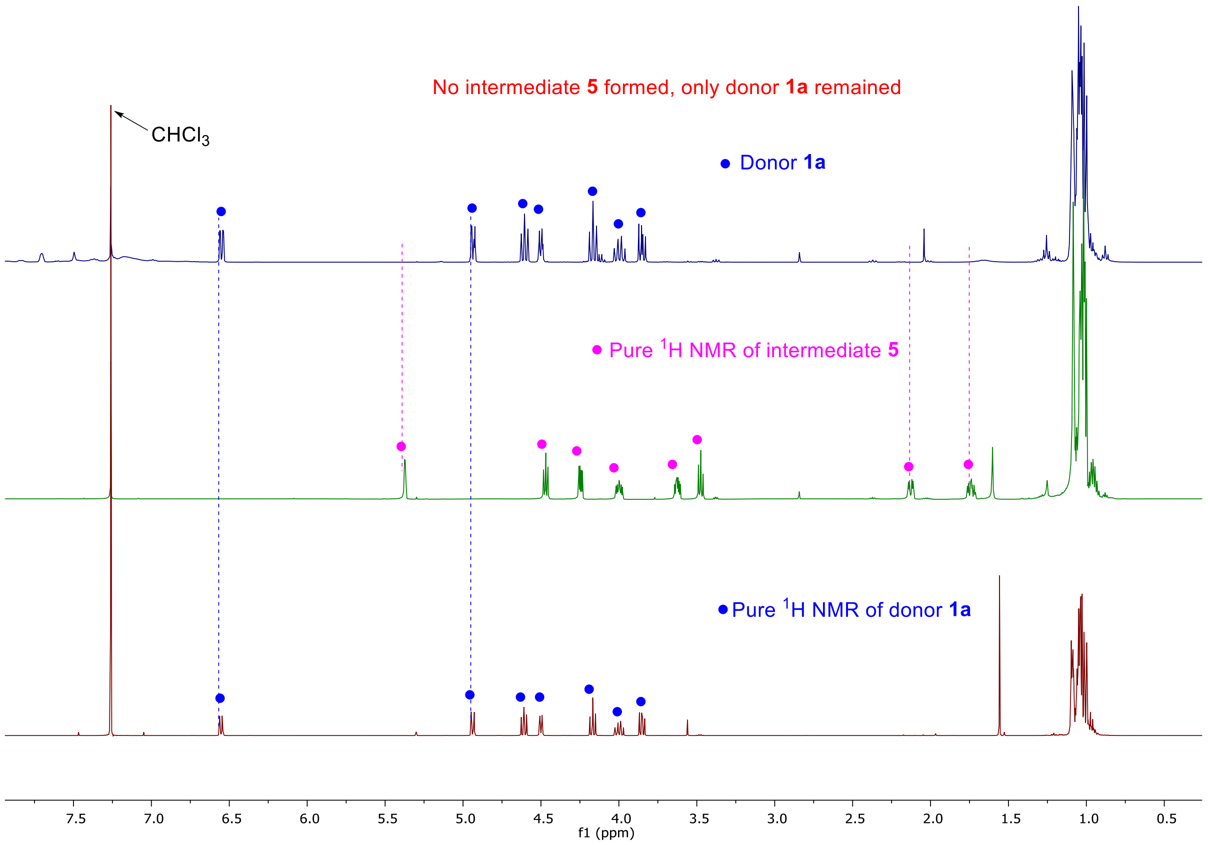

Supplementary Figure S13. Crude ^{1}H NMR spectra of poisoning experiment by *rac*-BINAP on the upstream step

7.7. NMR data for titration experiments

7.7.1. NMR titration between catalyst J and donor 1a (with 3Å molecular sieves)

Procedure: To a dry NMR tube filled with 20mg of activated powdered 3Å molecular sieves, catalyst **J** (24.85 mg, 0.01 mmol) and varying amounts of donor **1a** were added (Note: catalyst **J** to donor **1a** molar ratios are 1:1, 1:2, 1:4, 1:6, 1:8, 1:10). Varying amounts of CD_2Cl_2 were added in order to reach a total volume of 500 μL. The detected ^{77}Se NMR (use 1,1′-Diselanediyldibenzene as external standard) and ^{31}P NMR (use phosphoric acid as external standard) shift of catalyst **J**, ^{1}H NMR and ^{13}C NMR shift of donor **1a** are shown respectively.

^{77}Se NMR shift on catalyst J

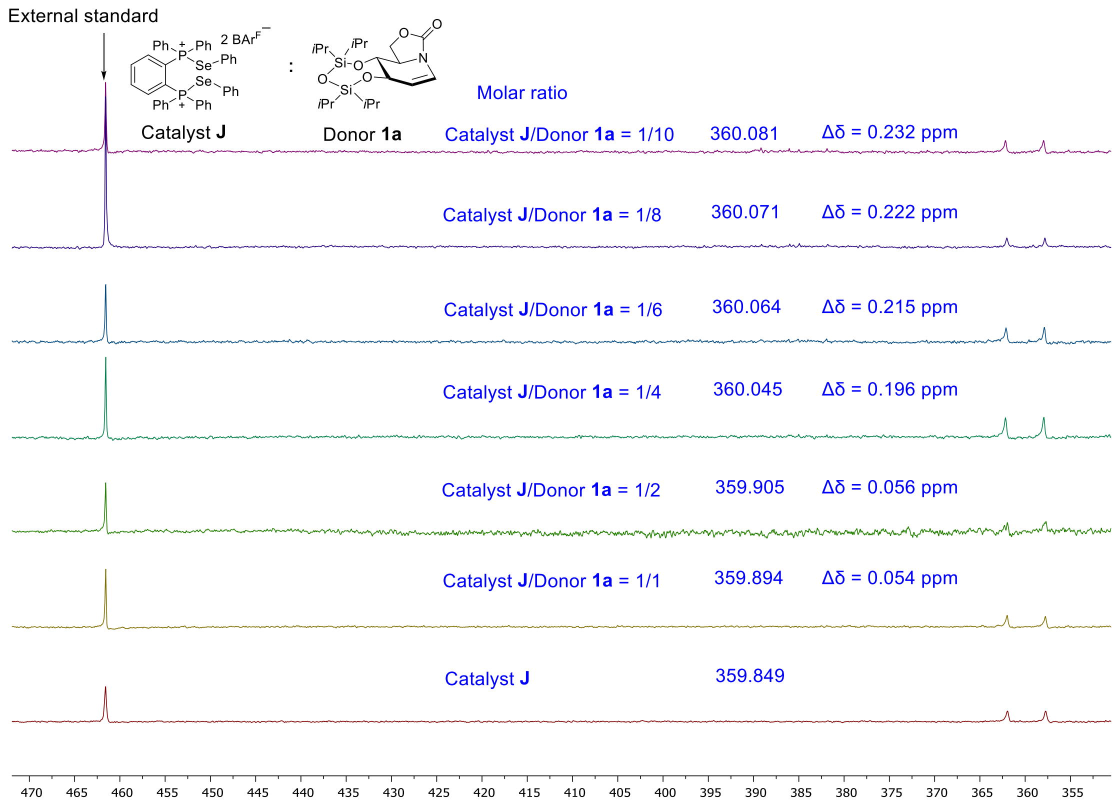

Supplementary Figure S14. ^{77}Se titration for catalyst **J** and donor **1a**

^{31}P NMR shift on catalyst J (insignificant pnictogen bonding from phosphorus)

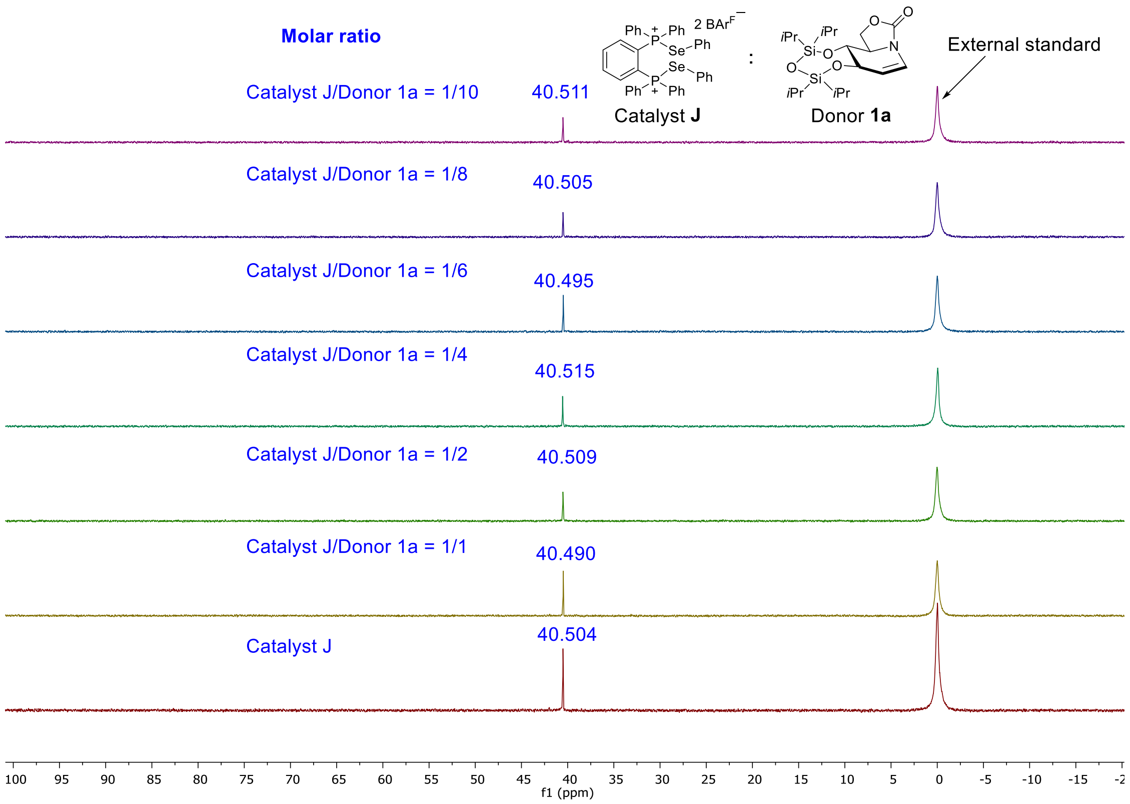

Supplementary Figure S15. ^{31}P titration for catalyst **J** and donor **1a**

^{13}C NMR shift of carbonyl group donor 1a with catalyst J

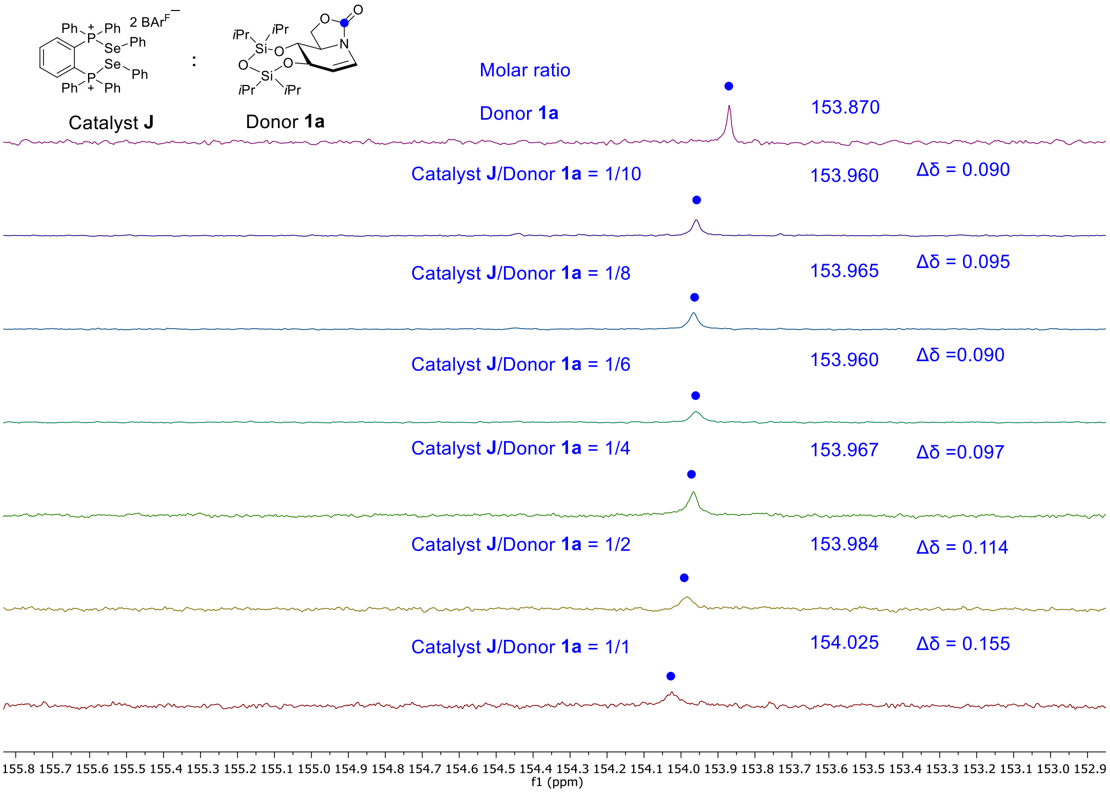

Supplementary Figure S16. ^{13}C titration of carbonyl group for catalyst **J** and donor **1a**

^{13}C NMR shift on the alkene of donor 1a with catalyst J

^{13}C NMR shift on C_2 of the alkene

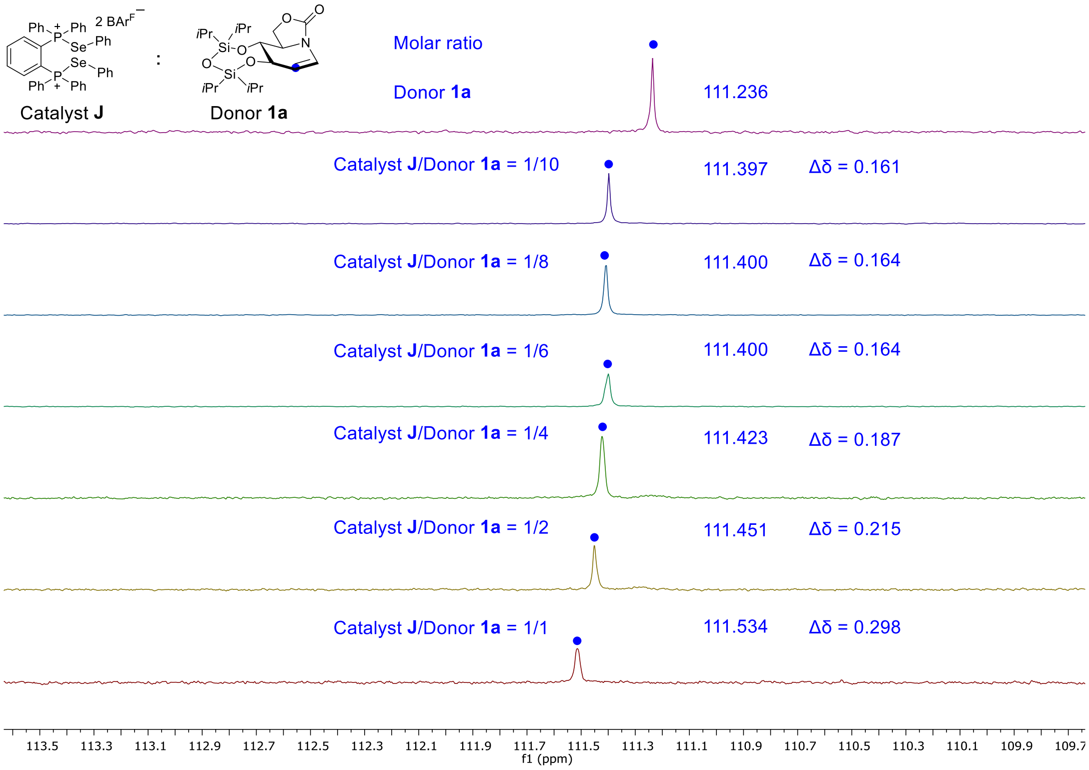

Supplementary Figure S17. ^{13}C titration of C_2 on the alkene for catalyst **J** and donor **1a**

^{13}C NMR shift on C_1 of the alkene

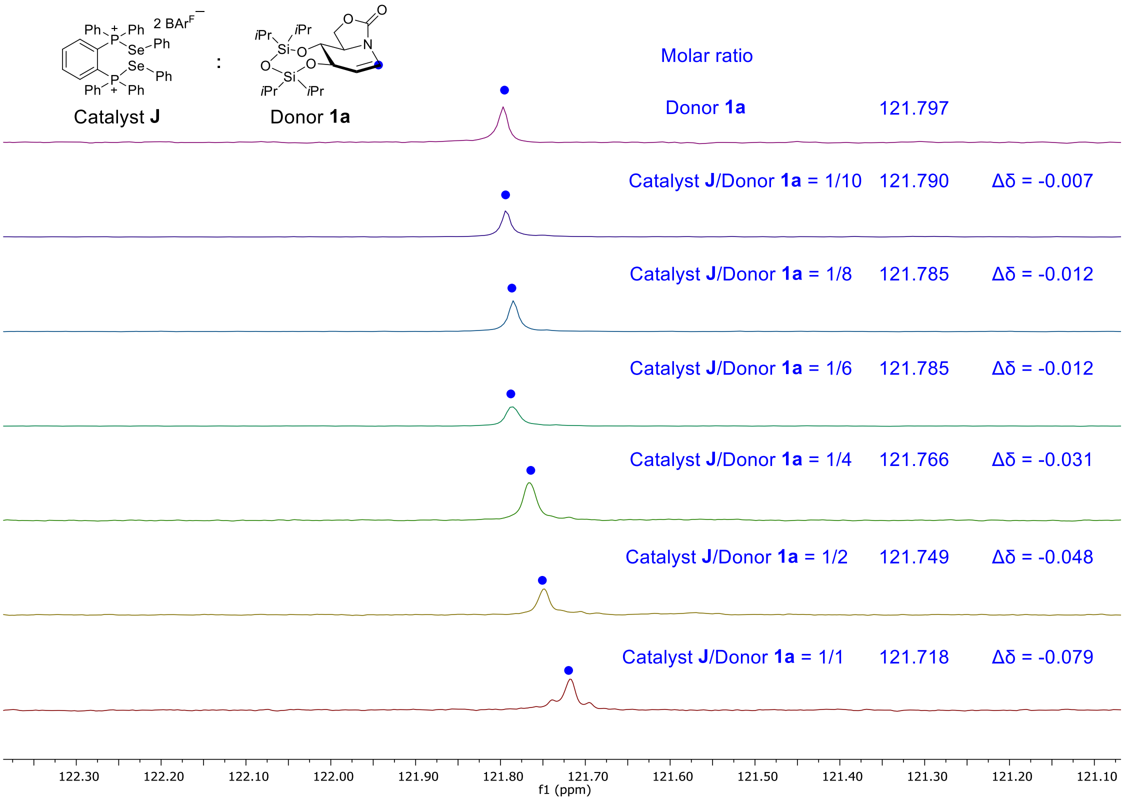

Supplementary Figure S18. ^{13}C titration of C_1 on the alkene for catalyst **J** and donor **1a**

7.7.2. NMR titration between catalyst J and acceptor 2n

Procedure: To a dry NMR tube, filled with catalyst **J** (24.85 mg, 0.01 mmol) and varying amounts of acceptor **2n** were added (Note: catalyst **J** to acceptor **2n** molar ratios are 1:1, 1:2, 1:4, 1:6, 1:8, 1:10). Varying amounts of CD_2Cl_2 were added in order to reach a total volume of 500 μL. The detected ^{77}Se NMR (use 1,1′-Diselanediyldibenzene as external standard) shift of catalyst **J** and ^{1}H NMR shift of acceptor **2n** are shown respectively.

^{77}Se NMR shift on catalyst J

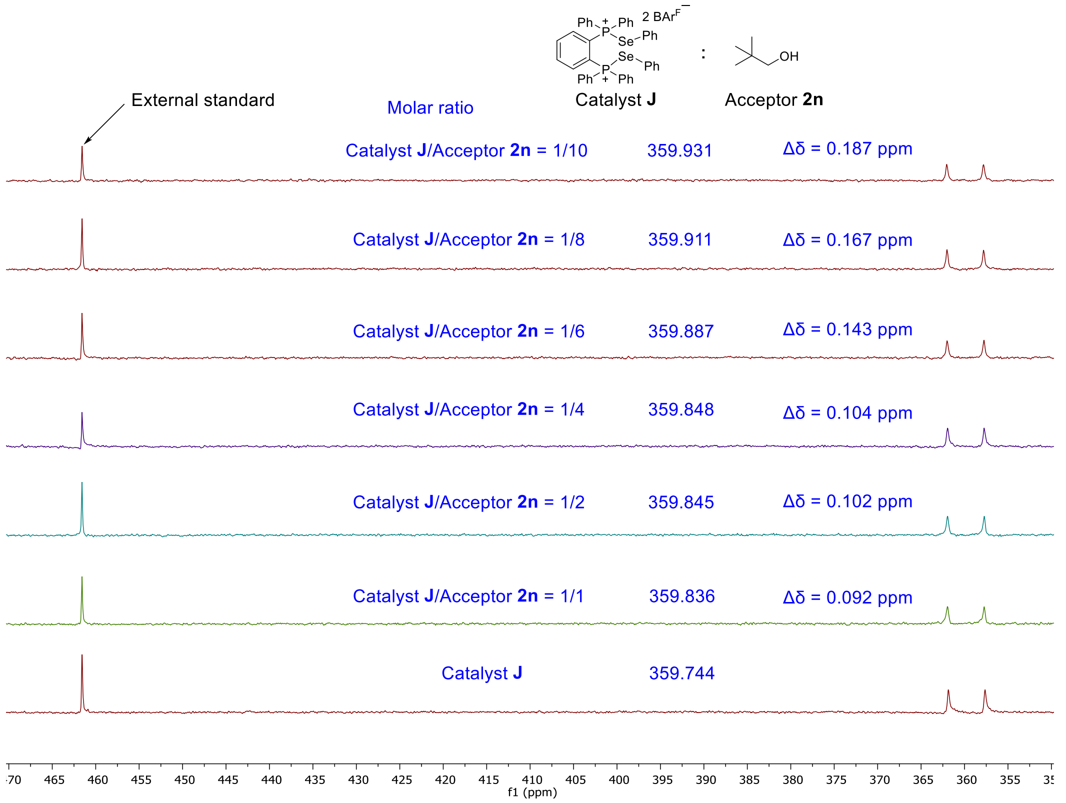

Supplementary Figure S19. ^{77}Se titration for catalyst **J** and acceptor **2n**

^{1}H NMR shift of the hydroxyl proton on acceptor 2n

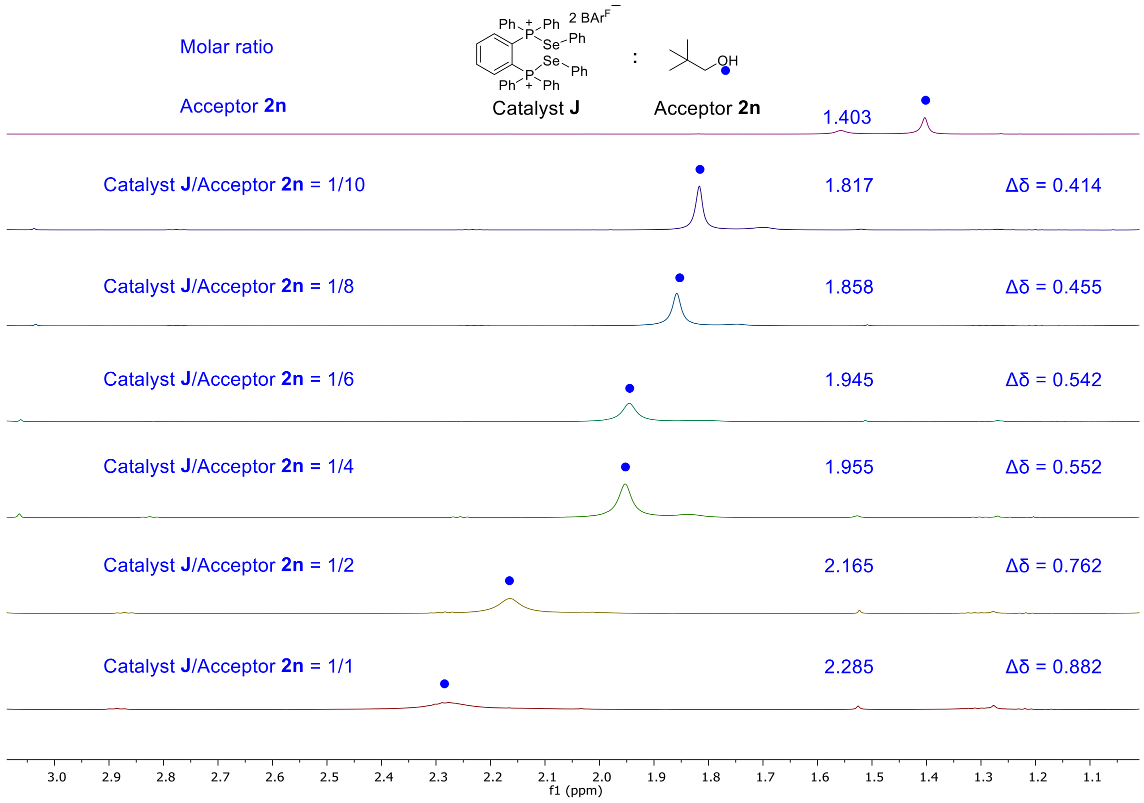

Supplementary Figure S20. ^{1}H NMR shift of the hydroxyl proton on acceptor **2n**

7.7.3. NMR titration between catalyst J and intermediate 5

Procedure: To a dry NMR tube, filled with catalyst **J** (24.85 mg, 0.01 mmol) and varying amounts of intermediate **5** were added (Note: catalyst **J** to intermediate **5** molar ratios are 1:1, 1:3). Varying amounts of CD_2Cl_2 were added in order to reach a total volume of 500 μL. The detected ^{77}Se NMR (use 1,1′-Diselanediyldibenzene as external standard) and ^{31}P NMR (use phosphoric acid as external standard) shift of catalyst **J** are shown respectively.

^{77}Se NMR shift on catalyst J

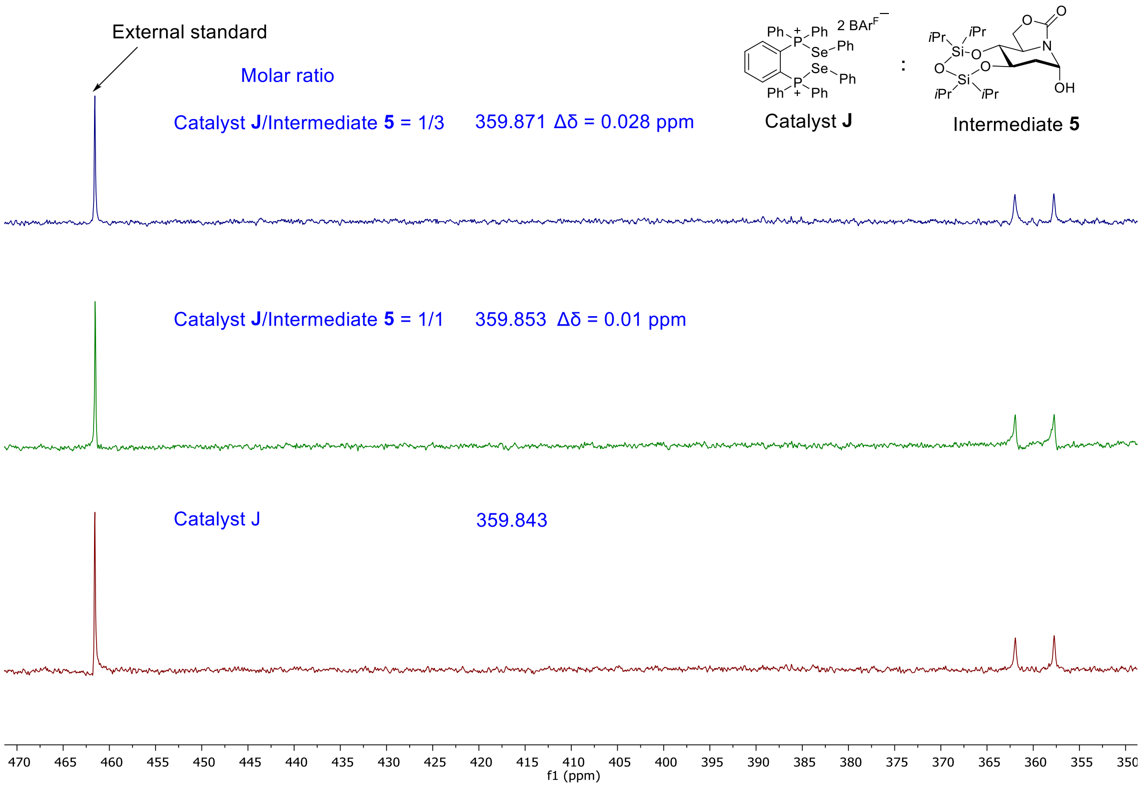

Supplementary Figure S21. ^{77}Se NMR titration for catalyst **J** and intermediate **5**

^{31}P NMR shift on catalyst J

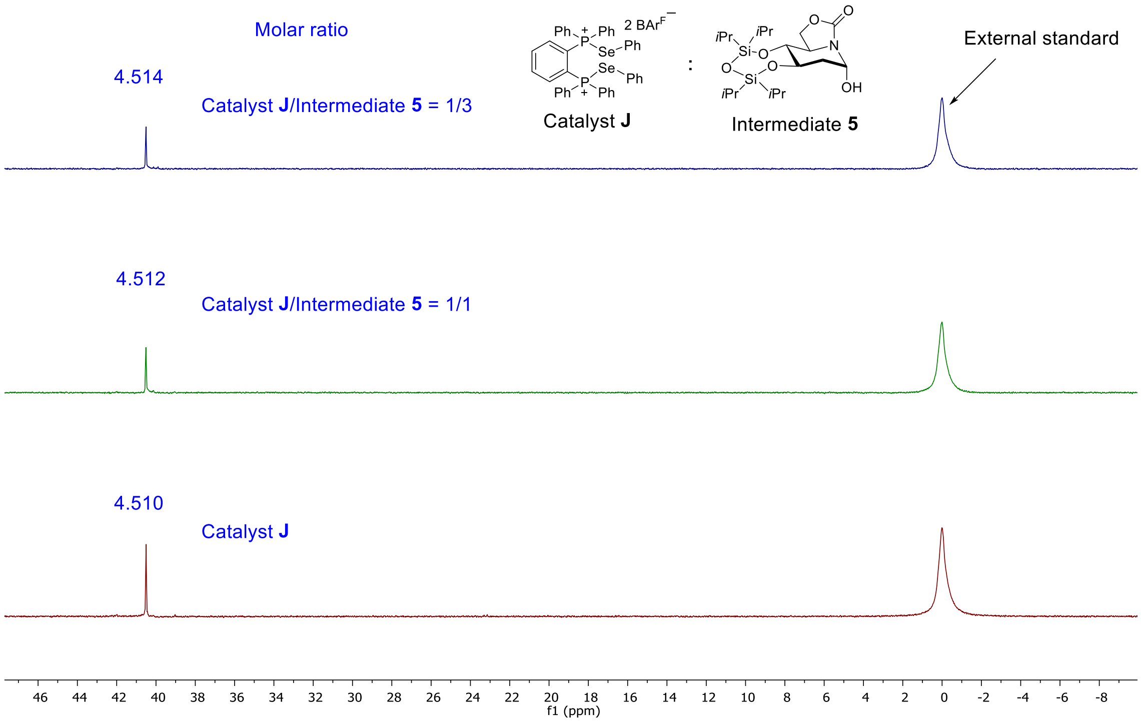

Supplementary Figure S22. ^{31}P NMR titration for catalyst **J** and intermediate **5**

^{13}C NMR shift of carbonyl group intermediate 5 with catalyst J

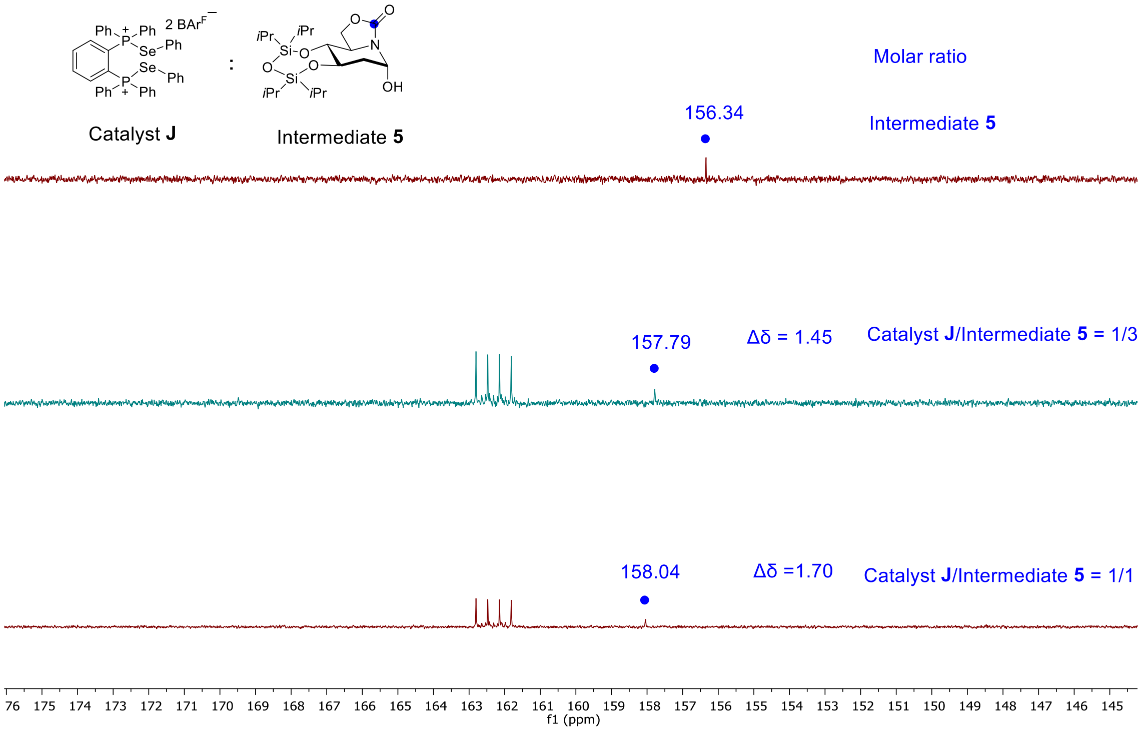

Supplementary Figure S23. ^{13}C NMR titration for catalyst **J** and intermediate **5**

7.8. Effect of base additive(K_2CO_3) on the mechanism

Procedure: To an oven dried dram vial purged with an argon balloon was charged catalyst **J** (2.5 mg, 0.001 mmol, 2 mol%), glucosyl iminoglycal donor **1a** (0.05 mmol, 1.0 equiv.), dry DCM (0.2 mL), glycosyl acceptor **2a** (0.075 mmol, 9.75 mg, 1.5 equiv.), and then K_2CO_3 powder (0.01 mmol, 1.38 mg, 20 mol%) were added. The dram vial was sealed and the mixture was stirred for 24 h at room temperature.

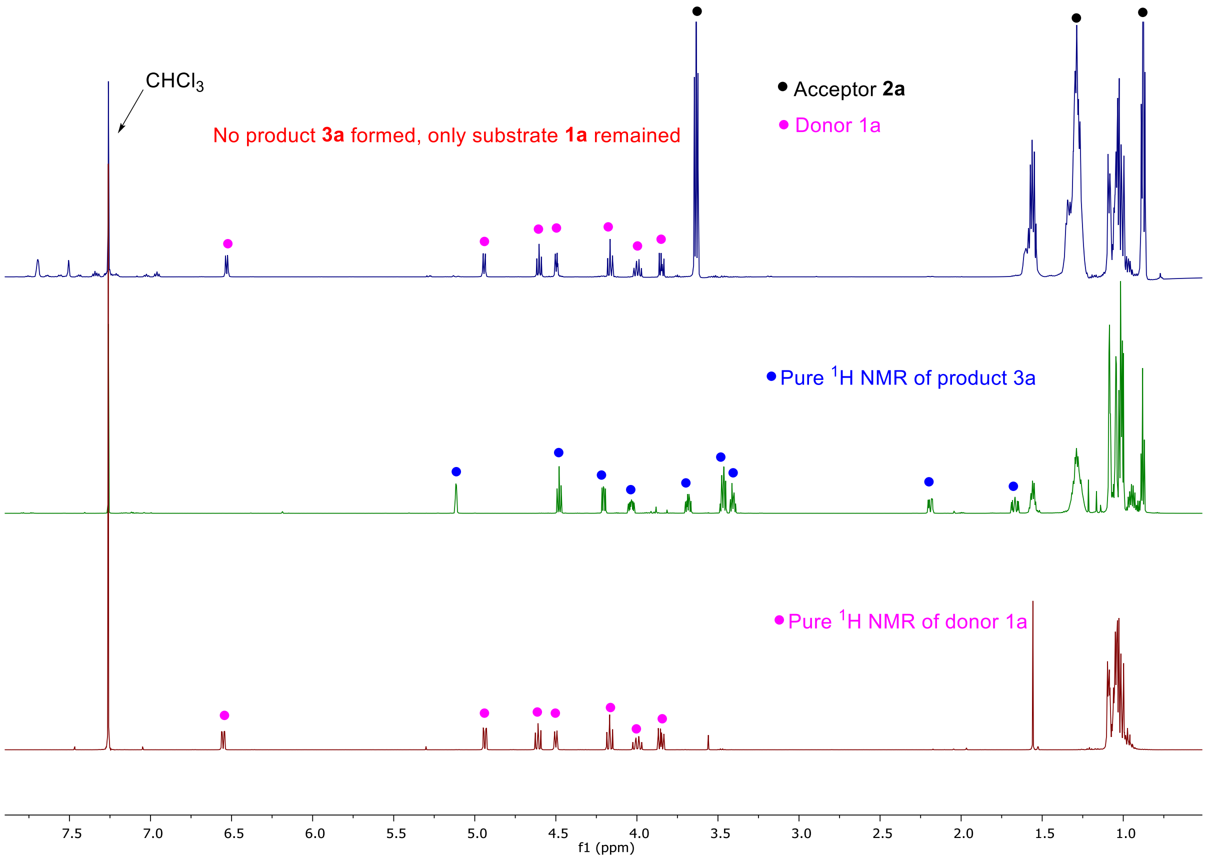

Supplementary Figure S24. Crude ^1H NMR spectra of K_2CO_3 effect on the mechanism

7.9. Competitive experiment between different phenols

Procedure: To an oven dried dram vial purged with an argon balloon was charged catalyst **J** (9.94 mg, 0.004 mmol, 2 mol%), glucosyl iminoglycal donor **1a** (0.2 mmol, 1 equiv.), dry DCM (0.8 mL), and then phenol acceptor **2x** (0.3 mmol, 51.9 mg, 1.5 equiv.) and phenol acceptor **2y** (0.3 mmol, 37.2 mg, 1.5 equiv.) were added. The dram vial was sealed and the mixture was stirred for 1 h at room temperature. Upon completion of the reaction, the reaction mixture was filtered over a short silica plug and flushed with 10 mL of EA. The filtrate was then evaporated and the determination of the anomeric selectivity (α/β) is by ^1H-NMR analysis of this concentrated crude mixture with 1,1,2,2-tetrachloroethane as the internal standard. (This experiment repeated twice)

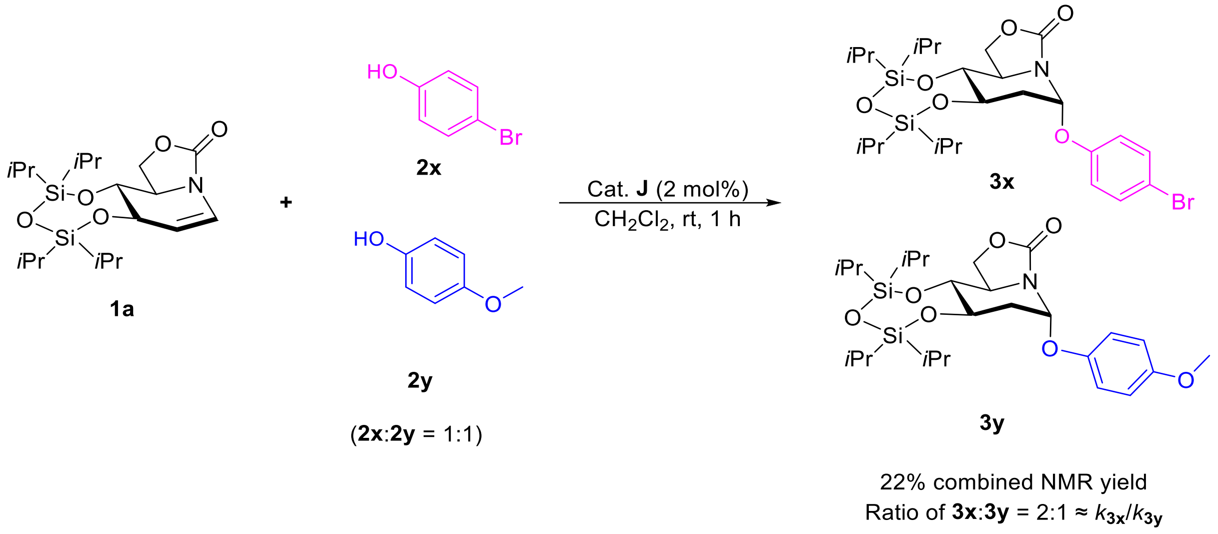

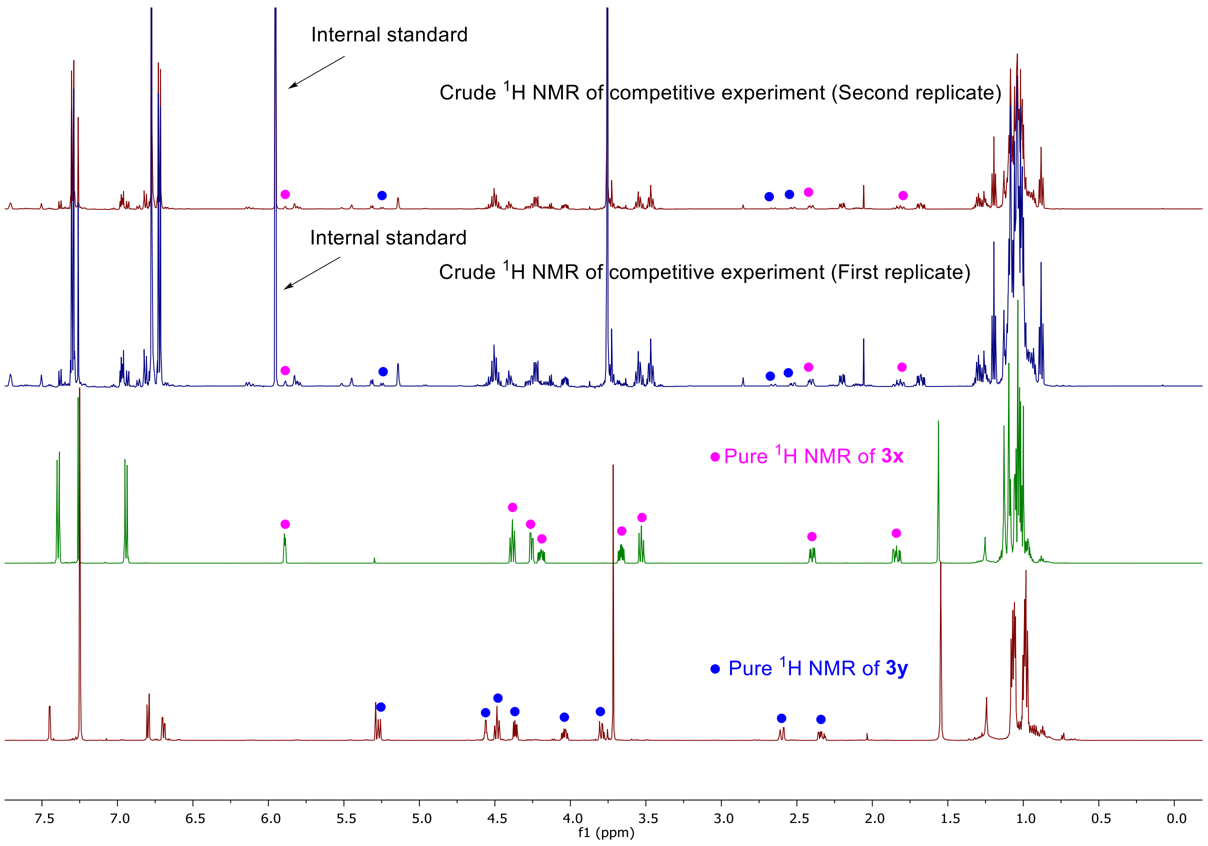

Supplementary Figure S25. Crude ^{1}H NMR spectra of competitive experiment

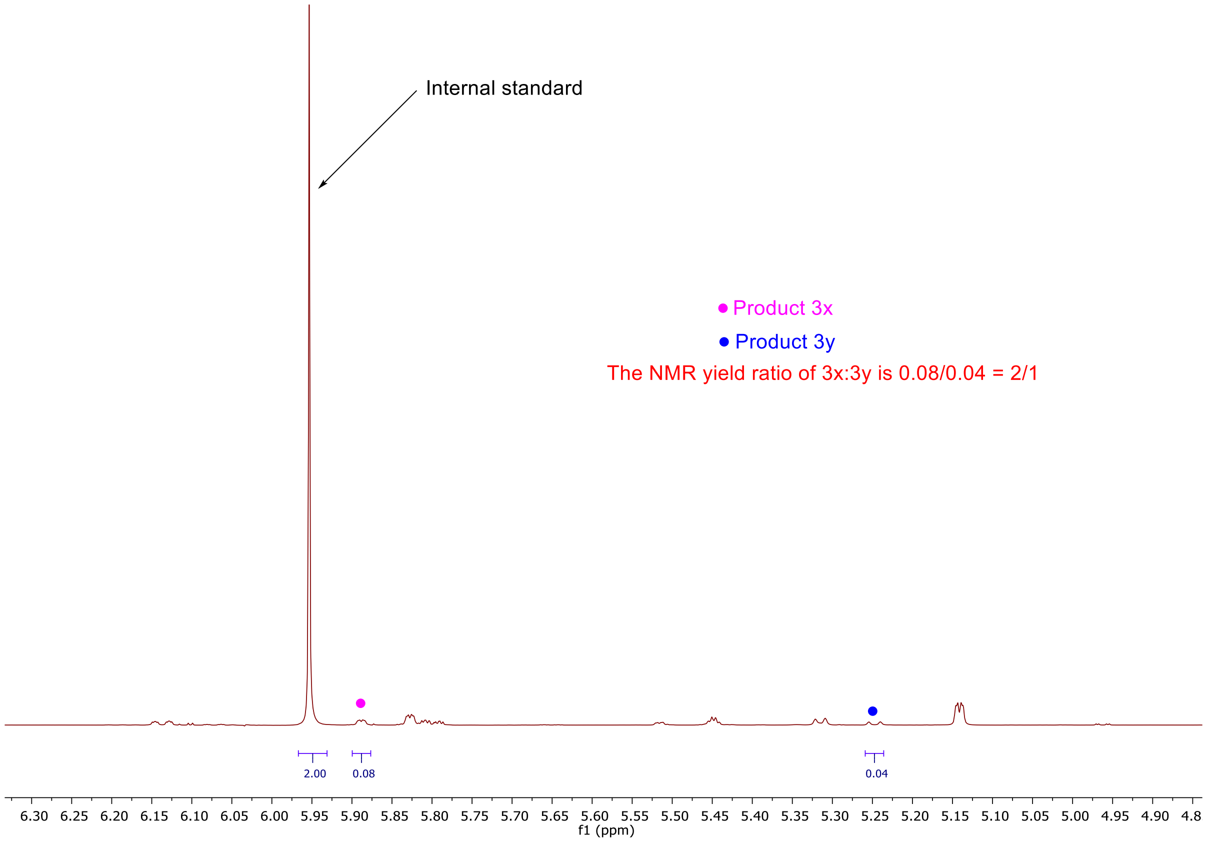

Supplementary Figure S26. Zoomed in spectra between 4.800-6.300 ppm field of the **Supplementary Figure S25**

7.10. Kinetic experiment on the overall reaction

7.10.1. Donor 1a concentration dependence

Procedure: To a dry NMR tube, donor **1a** (25.9 mg, 0.0625 mmol), catalyst **J** (6.2 mg, 0.00125 mmol), acceptor **2n** (16.5 mg, 0.1875 mmol) and 1,1,2,2-tetrachloroethane (10 μL) as the internal standard, CD_2Cl_2 was added in order to reach a total volume of 500 μL. Afterwards, the tube was sealed with cap and measured on the NMR spectrometer at room temperature by recording ^1H spectra at different time.

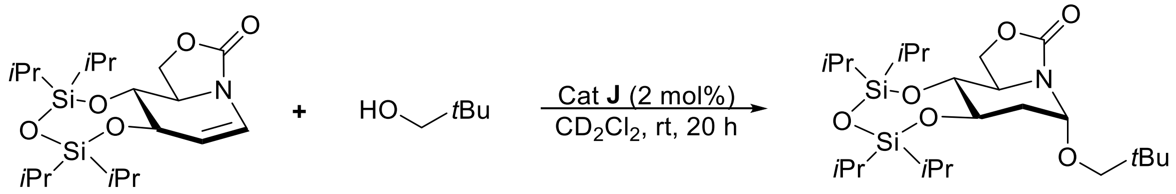

1a **2n** **3n**

0.125 M 0.375 M

Procedure: To a dry NMR tube, donor **1a** (77.5 mg, 0.1875 mmol), catalyst **J** (6.2 mg, 0.00125 mmol), acceptor **2n** (16.5 mg, 0.1875 mmol) and 1,1,2,2-tetrachloroethane (10 μL) as the internal standard, CD_2Cl_2 was added in order to reach a total volume of 500 μL. Afterwards, the tube was sealed with cap and measured on the NMR spectrometer at room temperature by recording ^1H spectra at different time.

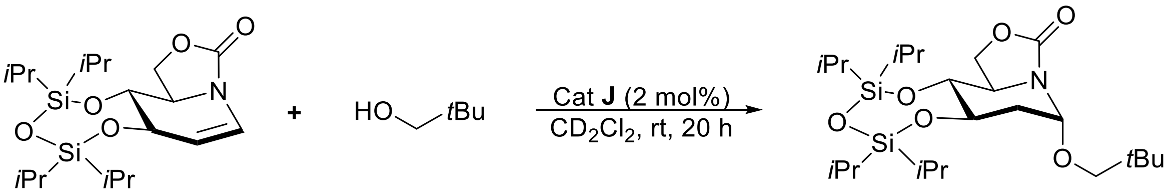

1a 0.375 M

2n 0.375 M

3n

Table S11. Concentration for **3n** calculated by ^{1}H NMR analysis for varying the donor concentration experiment

Time/h	**3n** (**1a** =0.125M)	**3n** (**1a** =0.25M)	**3n** (**1a** =0.375M)
0	0	0	0
0.13	0.002909406	0.007454567	0.021069363
0.25	0.010149164	0.012875205	0.023171831
0.5	0.015840583	0.018941507	0.031556055
0.75	0.02029985	0.024891528	0.040501979
1	0.023920862	0.030891828	0.048623108
1.25	0.027601678	0.036824752	0.055538261
1.5	0.030641474	0.04274904	0.062253341
1.75	0.033412465	0.04856081	0.068730045
2	0.036386294	0.054575775	0.074867064
2.25	0.038634339	0.060291205	0.082091297
2.5	0.041179689	0.06607881	0.087961816
2.75	0.043345663	0.071768692	0.093951009
3	0.045479268	0.077389176	0.099824111
3.25	0.047627703	0.08297125	0.106143558
3.5	0.049816148	0.088598229	0.111976006
3.75	0.051988124	0.093789873	0.117294418
4	0.054054629	0.099255414	0.123817375
4.25	0.055660776	0.104640102	0.129764048
4.5	0.05741323	0.109636738	0.134888006
4.75	0.059748392	0.114854794	0.14268528
5	0.061174263	0.119694249	0.147148419
5.25	0.063073216	0.124814048	0.152772039
5.5	0.064853511	0.129140135	0.15964661
5.75	0.066464179	0.1337502	0.165788557
6	0.068603803	0.137931833	0.172981086
6.25	0.069312719	0.142135686	0.179800528
6.5	0.071066116	0.146085487	0.186410551
6.75	0.072498647	0.149931012	0.193456481
7	0.074011273	0.153426106	0.200676159
7.25	0.075048858	0.156956915	0.207869907
7.5	0.076739577	0.160325716	0.216066931
7.75	0.077458949	0.163198368	0.223172909
8	0.079079808	0.166095627	0.231104333
8.25	0.080196384	0.168638359	0.239988395
8.5	0.081276736	0.170427213	0.24830506
8.75	0.082905335	0.172975252	0.256278386
9	0.083344163	0.174874224	0.267109313
9.25	0.084389511	0.176689624	0.274997077
9.5	0.085762503	0.1784175	0.285504423
9.75	0.086691244	0.179601269	0.294362329
10	0.087521961	0.180935598	0.302128248
10.25	0.088852446	0.181977271	0.304937343
10.5	0.089898659	0.182906249	0.308476897

10.75	0.090041837	0.183753131	0.312776785
11	0.090982948	0.184671471	0.312387867
11.25	0.091992109	0.185885674	0.314704127
11.5	0.092929891	0.186534338	0.3146613
11.75	0.0937166	0.186468876	0.315246297
12	0.094358651	0.186936721	0.317360276
12.25	0.094401619	0.187909208	0.317000495
12.5	0.095712394	0.187675959	0.318080497
12.75	0.0963055	0.187554157	0.318963169
13	0.096868714	0.18811096	0.318335054
13.25	0.097487619	0.187949172	0.318935312
13.5	0.097869935	0.188477014	0.319895678
13.75	0.09806173	0.188615654	0.318464586
14	0.098733714	0.188659756	0.318938582
14.25	0.099076599	0.188851015	0.318162767
14.5	0.099855852	0.18885694	0.320235391
14.75	0.100565198	0.189084114	0.319283425
15	0.100799603	0.188997165	0.321993449
15.25	0.100702247	0.188966027	0.320785123
15.5	0.101427559	0.188801099	0.320611816
15.75	0.10159461	0.189308751	0.319232094
16	0.102240913	0.189140431	0.321529059
16.25	0.102759792	0.189239049	0.31835022
16.5	0.10281612	0.189150414	0.321256931
16.75	0.103405369	0.189135781	0.320007646
17	0.103347228	0.189005221	0.319754898
17.25	0.103925784	0.189095341	0.321222753
17.5	0.103599912	0.189939579	0.320832556
17.75	0.10434133	0.188936583	0.320906597
18	0.104631937	0.188854589	0.319833848
18.25	0.104920119	0.189053772	0.32014916
18.5	0.105471876	0.189065235	0.320768351
18.75	0.105140451	0.188958698	0.320242553
19	0.105555277	0.189115007	0.321511159
19.25	0.105595836	0.189066007	0.319209773
19.5	0.10569332	0.189087778	0.319415482
19.75	0.106008594	0.189181266	0.321717576
20	0.106223531	0.188974569	0.321536953

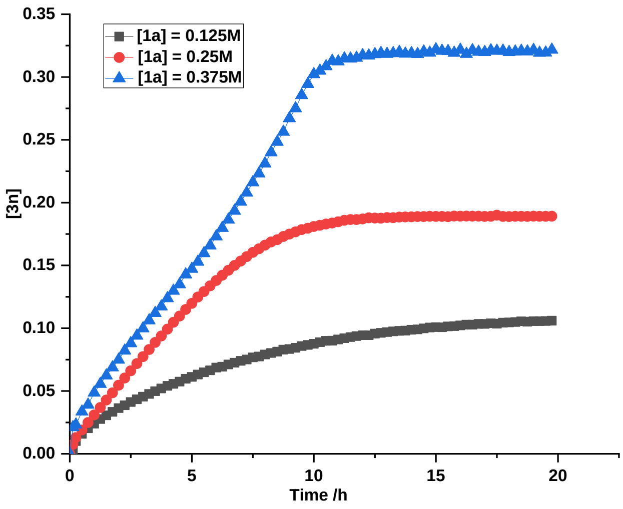

Supplementary Figure S27. Overlapped profile for the donor **1a** concentration dependence experiments

7.10.2. Acceptor 2n concentration dependence

Procedure: To a dry NMR tube, donor **1a** (51.7 mg, 0.125 mmol), catalyst **J** (6.2 mg, 0.00125 mmol), acceptor **2n** (11.0 mg, 0.125 mmol) and 1,1,2,2-tetrachloroethane (10 μL) as the internal standard, CD_2Cl_2 was added in order to reach a total volume of 500 μL. Afterwards, the tube was sealed with cap and measured on the NMR spectrometer at room temperature by recording ^1H spectra at different time.

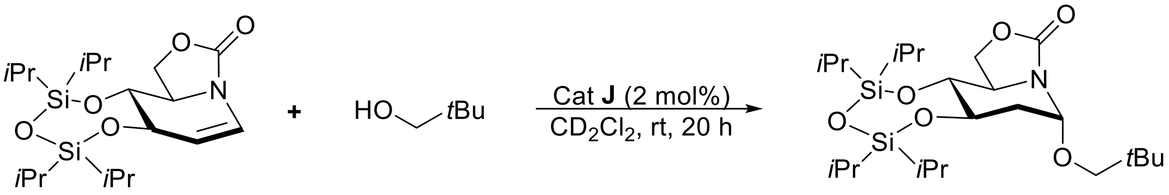

1a 0.25 M

2n 0.25 M

3n

Procedure: To a dry NMR tube, donor **1a** (51.7 mg, 0.125 mmol), catalyst **J** (6.2 mg, 0.00125 mmol), acceptor **2n** (22.0 mg, 0.25 mmol) and 1,1,2,2-tetrachloroethane (10 μL) as the internal standard, CD_2Cl_2 was added in order to reach a total volume of 500 μL. Afterwards, the tube was sealed with cap and measured on the NMR spectrometer at room temperature by recording ^1H spectra at different time.

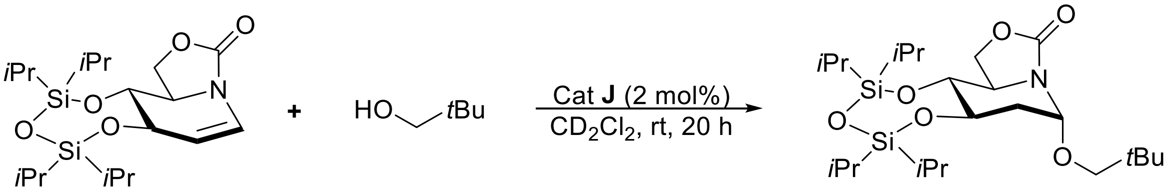

1a 0.25 M

2n 0.50 M

3n

Table S12. Concentration for **3n** calculated by ^{1}H NMR analysis for varying the acceptor concentration experiment

Time/h	**3n** (**2n** =0.25M)	**3n** (**2n** =0.375M)	**3n** (**2n** =0.50M)
0	0	0	0
0.13	0.005749023	0.007454567	0.004107864
0.25	0.013756622	0.012875205	0.006133061
0.5	0.023950153	0.018941507	0.007316883
0.75	0.037261336	0.024891528	0.010403472
1	0.048885162	0.030891828	0.0121223
1.25	0.060822387	0.036824752	0.016148672
1.5	0.0727052	0.04274904	0.017753084
1.75	0.084813301	0.04856081	0.020909103
2	0.096764955	0.054575775	0.023128446
2.25	0.108679639	0.060291205	0.0263176
2.5	0.120916211	0.06607881	0.028025495
2.75	0.132746872	0.071768692	0.030489716
3	0.144457506	0.077389176	0.035353177
3.25	0.155368204	0.08297125	0.036310594
3.5	0.164510968	0.088598229	0.039233068
3.75	0.172174031	0.093789873	0.043004064
4	0.178699763	0.099255414	0.044997943
4.25	0.183727507	0.104640102	0.046718465
4.5	0.187243815	0.109636738	0.048537204
4.75	0.189452204	0.114854794	0.052598284
5	0.191492694	0.119694249	0.05459226
5.25	0.192544174	0.124814048	0.057402283
5.5	0.193417758	0.129140135	0.058689874
5.75	0.194079167	0.1337502	0.060261377
6	0.194625756	0.137931833	0.063017693
6.25	0.195004949	0.142135686	0.065601027
6.5	0.195353815	0.146085487	0.068404087
6.75	0.19565584	0.149931012	0.071000051
7	0.195856431	0.153426106	0.073957817
7.25	0.195581889	0.156956915	0.07572264
7.5	0.196388325	0.160325716	0.077656844
7.75	0.196080617	0.163198368	0.081070126
8	0.196654282	0.166095627	0.082148708
8.25	0.197035766	0.168638359	0.084564412
8.5	0.196602763	0.170427213	0.086112044
8.75	0.196974505	0.172975252	0.087998261
9	0.197374978	0.174874224	0.090426046
9.25	0.196986087	0.176689624	0.092396405
9.5	0.19763486	0.1784175	0.094992156
9.75	0.197201119	0.179601269	0.096600848
10	0.197824025	0.180935598	0.097975877
10.25	0.197440178	0.181977271	0.100932306
10.5	0.197643504	0.182906249	0.102681365
10.75	0.198106576	0.183753131	0.104699998
11	0.197608331	0.184671471	0.106026743
11.25	0.19780252	0.185885674	0.108074997
11.5	0.198280025	0.186534338	0.110931065
11.75	0.197906504	0.186468876	0.112569155
12	0.198033926	0.186936721	0.114734488
12.25	0.198734858	0.187909208	0.116516069
12.5	0.19845967	0.187675959	0.118853891
12.75	0.198157874	0.187554157	0.119085183
13	0.198596049	0.18811096	0.121370422

13.25	0.198841561	0.187949172	0.123485322
13.5	0.198703314	0.188477014	0.124954614
13.75	0.197990117	0.188615654	0.126217879
14	0.198118625	0.188659756	0.128034756
14.25	0.198264322	0.188851015	0.130121711
14.5	0.198382441	0.18885694	0.131987875
14.75	0.198246138	0.189084114	0.133180929
15	0.198430025	0.188997165	0.135233985
15.25	0.198694931	0.188966027	0.135987536
15.5	0.198494132	0.188801099	0.137955054
15.75	0.198680711	0.189308751	0.137420182
16	0.198550232	0.189140431	0.141092221
16.25	0.198304022	0.189239049	0.141418303
16.5	0.197838906	0.189150414	0.142375074
16.75	0.198650007	0.189135781	0.14401866
17	0.197997498	0.189005221	0.145062668
17.25	0.198502158	0.189095341	0.146258126
17.5	0.19776042	0.189939579	0.148446043
17.75	0.197889345	0.188936583	0.148986314
18	0.197718042	0.188854589	0.150146694
18.25	0.197979413	0.189053772	0.15153835
18.5	0.19799414	0.189065235	0.154194279
18.75	0.198402507	0.188958698	0.153979241
19	0.197877727	0.189115007	0.156303399
19.25	0.19759592	0.189066007	0.155785131
19.5	0.19748061	0.189087778	0.157629864
19.75	0.197485605	0.189181266	0.159091825
20	0.197824891	0.188974569	0.159314205

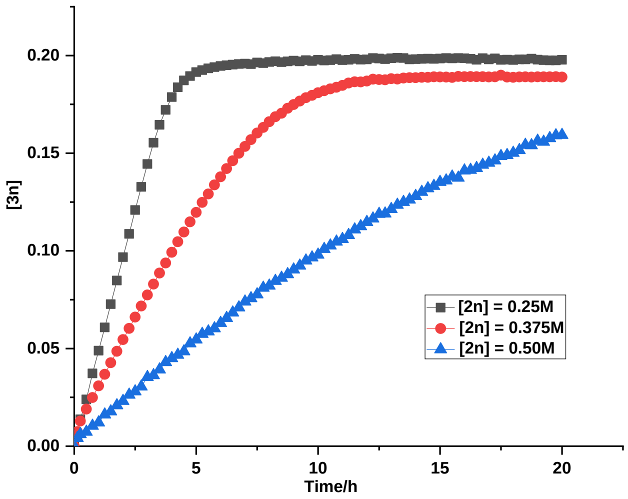

Supplementary Figure S28. Overlapped profile for the acceptor **2n** concentration dependence experiments

7.10.3. Catalyst J concentration dependence

Procedure: To a dry NMR tube, donor **1a** (51.7 mg, 0.125 mmol), catalyst **J** (3.1 mg, 1.0 mol%), acceptor **2n** (16.5 mg, 0.1875 mmol) and 1,1,2,2-tetrachloroethane (10 μL) as the internal standard, CD_2Cl_2 was added in order to reach a total volume of 500 μL. Afterwards, the tube was sealed with cap and measured on the NMR spectrometer at room temperature by recording ^{1}H spectra at different time.

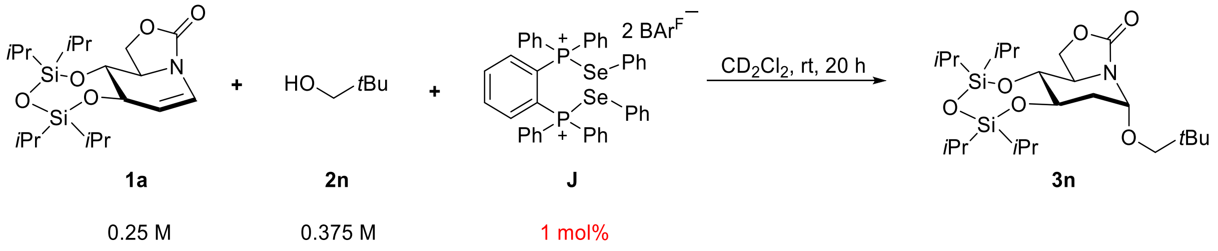

Procedure: To a dry NMR tube, donor **1a** (51.7 mg, 0.125 mmol), catalyst **J** (12.4 mg, 4.0 mol%), acceptor **2n** (16.5 mg, 0.1875 mmol) and 1,1,2,2-tetrachloroethane (10 µL) as the internal standard, CD_2Cl_2 was added in order to reach a total volume of 500 µL. Afterwards, the tube was sealed with cap and measured on the NMR spectrometer at room temperature by recording ^1H spectra at different time.

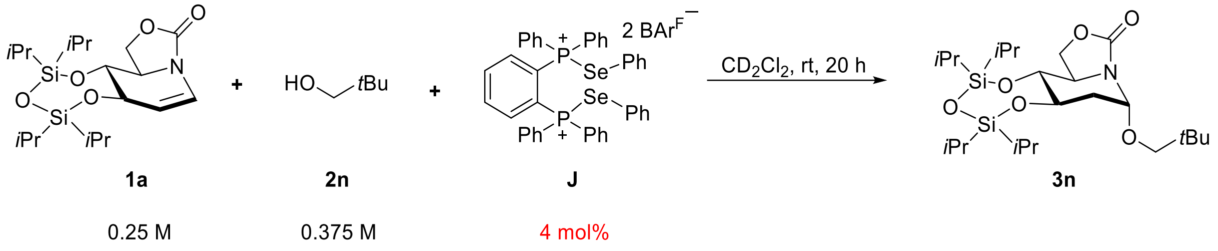

Table S13. Concentration for **3n** calculated by ^1H NMR analysis for varying the catalyst concentration experiment

Time/h	**3n** (**J** = 1.0 mol%)	**3n** (**J** = 2.0 mol%)	**3n** (**J** = 4.0 mol%)
0	0	0	0
0.13	3.04E-04	0.00745	0.00484
0.25	0.00328	0.01288	0.01734
0.5	0.0061	0.01894	0.02969
0.75	0.00861	0.02489	0.04093
1	0.01184	0.03089	0.05256
1.25	0.01495	0.03682	0.06478
1.5	0.01771	0.04275	0.07613
1.75	0.02045	0.04856	0.08771
2	0.02376	0.05458	0.09891
2.25	0.02574	0.06029	0.11031
2.5	0.029	0.06608	0.12062
2.75	0.03198	0.07177	0.13113
3	0.0344	0.07739	0.1405
3.25	0.03659	0.08297	0.14999
3.5	0.03967	0.0886	0.15838
3.75	0.04212	0.09379	0.16598
4	0.04506	0.09926	0.17261
4.25	0.04778	0.10464	0.17852
4.5	0.0505	0.10964	0.18353
4.75	0.05352	0.11485	0.18768
5	0.05582	0.11969	0.19078
5.25	0.0584	0.12481	0.19373
5.5	0.06135	0.12914	0.19577
5.75	0.06362	0.13375	0.19739
6	0.06616	0.13793	0.19872
6.25	0.06896	0.14214	0.19969
6.5	0.07142	0.14609	0.20068
6.75	0.07414	0.14993	0.20131
7	0.07675	0.15343	0.20204
7.25	0.07966	0.15696	0.20186
7.5	0.08145	0.16033	0.20232

7.75	0.08427	0.1632	0.20287
8	0.08652	0.1661	0.20307
8.25	0.08898	0.16864	0.20319
8.5	0.09195	0.17043	0.20304
8.75	0.09384	0.17298	0.20346
9	0.09667	0.17487	0.20339
9.25	0.09905	0.17669	0.20321
9.5	0.10077	0.17842	0.20343
9.75	0.10321	0.1796	0.20323
10	0.10541	0.18094	0.20333
10.25	0.1079	0.18198	0.20359
10.5	0.11029	0.18291	0.20355
10.75	0.11276	0.18375	0.20341
11	0.11452	0.18467	0.20314
11.25	0.1174	0.18589	0.20328
11.5	0.11904	0.18653	0.20374
11.75	0.12119	0.18647	0.20398
12	0.12281	0.18694	0.20367
12.25	0.12503	0.18791	0.20334
12.5	0.1277	0.18768	0.20313
12.75	0.12901	0.18755	0.20348
13	0.131	0.18811	0.20338
13.25	0.13312	0.18795	0.20352
13.5	0.13498	0.18848	0.20317
13.75	0.13625	0.18862	0.20312
14	0.13826	0.18866	0.2035
14.25	0.14001	0.18885	0.20356
14.5	0.14184	0.18886	0.20357
14.75	0.14371	0.18908	0.20382
15	0.14417	0.189	0.20303
15.25	0.14592	0.18897	0.20331
15.5	0.14791	0.1888	0.20349
15.75	0.14958	0.18931	0.20351
16	0.15103	0.18914	0.20336
16.25	0.15214	0.18924	0.20284
16.5	0.15323	0.18915	0.20305
16.75	0.15443	0.18914	0.20315
17	0.15545	0.18901	0.20325
17.25	0.15714	0.1891	0.20342
17.5	0.15854	0.18994	0.20309
17.75	0.15893	0.18894	0.20246
18	0.16027	0.18885	0.20294
18.25	0.16114	0.18905	0.20285
18.5	0.16131	0.18907	0.20299
18.75	0.16332	0.18896	0.20347
19	0.1637	0.18912	0.20332
19.25	0.16516	0.18907	0.20257
19.5	0.16551	0.18909	0.20232
19.75	0.16565	0.18918	0.20288
20	0.16718	0.18897	0.20268

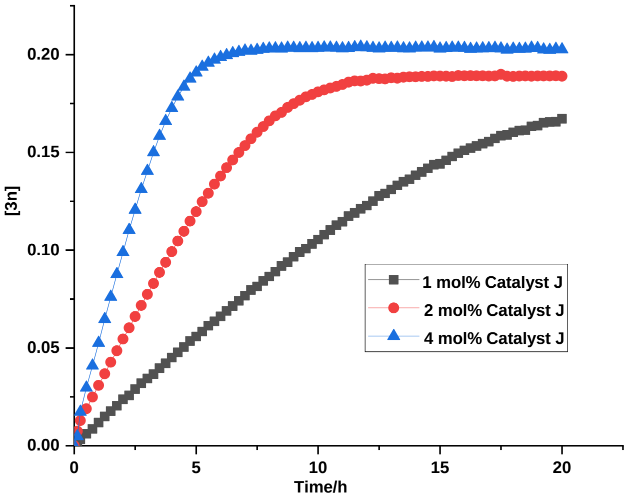

Supplementary Figure S29. Overlapped profile for the catalyst **J** concentration dependence experiments

7.11. Kinetic experiment on the downstream step

7.11.1. NMR monitoring of the downstream step under standard conditions

Procedure: To a dry NMR tube, intermediate **5** (4.31 mg, 0.01 mmol), 100 uL catalyst **J** (0.5 mg, 2.0 mol%, 5 mg in 1 mL CD_2Cl_2 solution), 150 uL acceptor **2n** (1.32 mg, 0.015 mmol, 8.8 mg in 1 mL CD_2Cl_2 solution) and 1,1,2,2-tetrachloroethane (5 μL) as the internal standard, CD_2Cl_2 was added in order to reach a total volume of 500 μL. Afterwards, the tube was sealed with cap and measured on the NMR spectrometer at room temperature by recording ^1H spectra at different time.

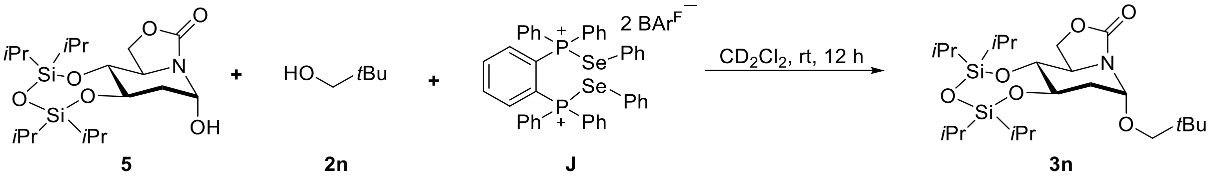

0.02 M | 0.03 M | 2 mol%

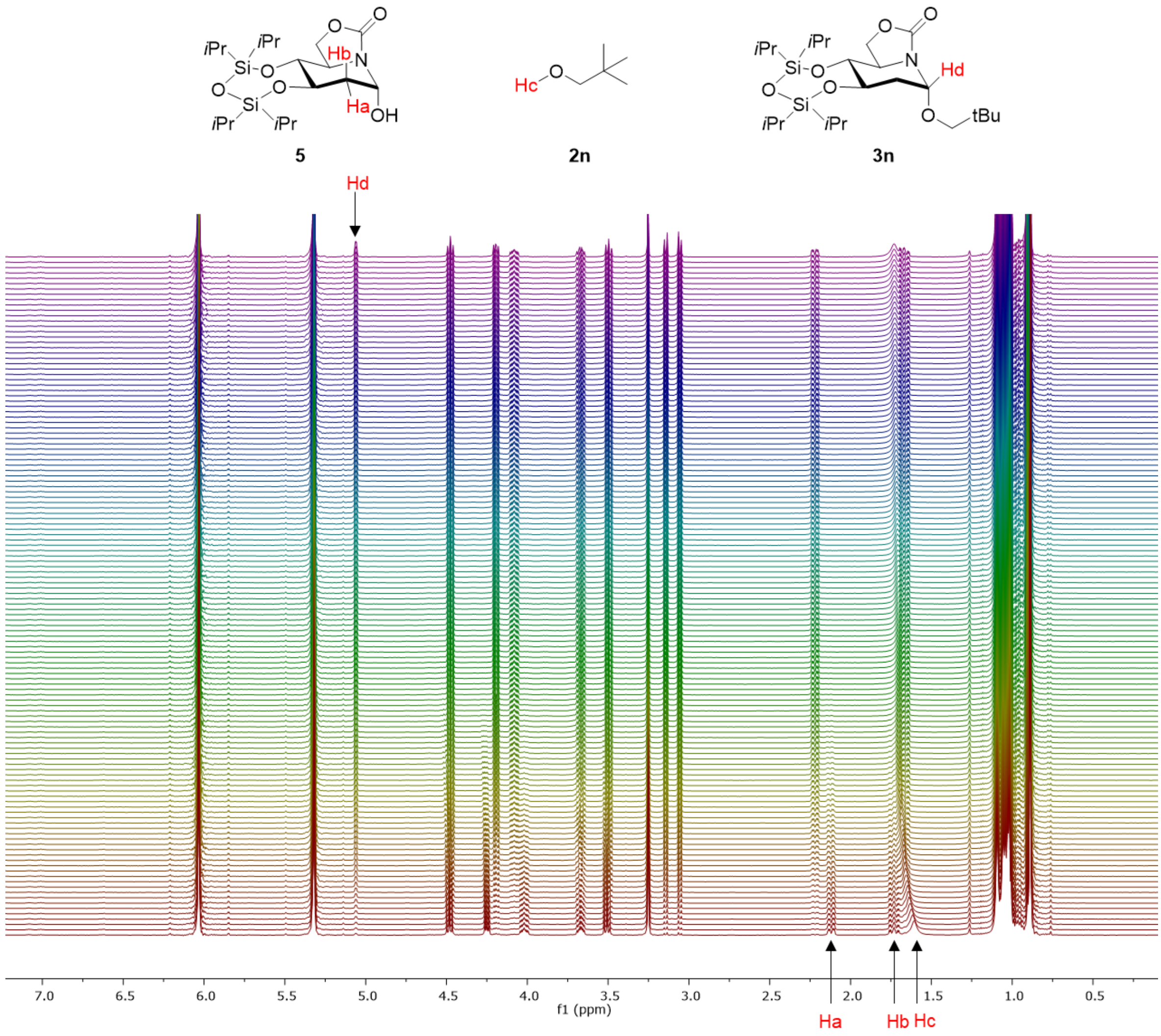

Supplementary Figure S30. Stacked ^{1}H NMR spectra for the monitoring of the downstream step under standard conditions

Table S14. Concentration for **5**, **2n**, **3n** calculated by ^{1}H NMR analysis for the monitoring of the downstream step under standard conditions

Time/h	[**INT 5**]/M	[**Acceptor 2n**]/M	[**3n**]/M
0	0.02	0.03	0
0.083333333	0.015862224	0.029009081	0.002250139
0.166666667	0.014955739	0.027614601	0.002400277
0.25	0.01456833	0.027238127	0.002707422
0.333333333	0.014187169	0.02686771	0.002943161
0.416666667	0.013827234	0.026414959	0.003087869
0.5	0.013478372	0.026101064	0.003532359
0.583333333	0.013076045	0.025710892	0.003797842
0.666666667	0.012687608	0.025230099	0.004009203
0.75	0.012287516	0.024925469	0.004366604
0.833333333	0.011929391	0.024493276	0.004733963
0.916666667	0.011588844	0.024069273	0.005171606
1	0.011165766	0.02360739	0.005659506
1.083333333	0.0107915	0.023240958	0.006053219
1.166666667	0.010404049	0.02278384	0.006572896
1.25	0.009984903	0.022475337	0.007026405
1.333333333	0.009617776	0.022041338	0.007649822

1.416666667	0.009211646	0.021635386	0.008045616
1.5	0.008819664	0.021303233	0.008446858
1.583333333	0.008428917	0.020896904	0.008725427
1.666666667	0.008130727	0.02057929	0.00905207
1.75	0.007749846	0.020123626	0.009543294
1.833333333	0.007393915	0.019744202	0.009828941
1.916666667	0.006966747	0.019410224	0.01020221
2	0.006564496	0.019099871	0.010587932
2.083333333	0.006273556	0.018722255	0.010946142
2.166666667	0.005949303	0.018365086	0.011286493
2.25	0.005599022	0.018033197	0.011562825
2.333333333	0.005265048	0.017685753	0.011943869
2.416666667	0.004940979	0.017375764	0.012313545
2.5	0.004651463	0.017082706	0.012589046
2.583333333	0.004356195	0.016768146	0.012859592
2.666666667	0.004093988	0.01653627	0.013224876
2.75	0.003775204	0.016219104	0.013483244
2.833333333	0.003557167	0.015951085	0.013693776
2.916666667	0.003257125	0.015768235	0.01409764
3	0.003112064	0.015466554	0.014252346
3.083333333	0.002809253	0.015238754	0.014481878
3.166666667	0.00254104	0.015064256	0.014773471
3.25	0.002300924	0.014881976	0.015099843
3.333333333	0.002137092	0.014719233	0.015176988
3.416666667	0.00199633	0.014488888	0.015310585
3.5	0.001827223	0.014307828	0.015468782
3.583333333	0.001729546	0.014109967	0.015642104
3.666666667	0.001542235	0.014019388	0.015765478
3.75	0.001339949	0.013957942	0.01594802
3.833333333	0.001317069	0.013733235	0.015971532
3.916666667	0.001121937	0.01373961	0.016156747
4	0.001081826	0.01359148	0.016265489
4.083333333	0.00105261	0.013535881	0.01636509
4.166666667	0.000891104	0.01348045	0.016428835
4.25	0.000843232	0.013420744	0.016483845
4.333333333	0.000719627	0.013315145	0.016499361
4.416666667	0.000831423	0.013224839	0.016522247
4.5	0.000666556	0.013224202	0.016701461
4.583333333	0.00067634	0.013173734	0.016605256
4.666666667	0.000582588	0.01314629	0.016710188
4.75	0.00058099	0.013131461	0.01671996
4.833333333	0.000576095	0.013111487	0.016780776
4.916666667	0.000527876	0.013042435	0.016792551
5	0.000457018	0.013092588	0.016849661
5.083333333	0.000508152	0.013032857	0.016772025
5.166666667	0.00036375	0.013067867	0.016944123
5.25	0.000426755	0.012997477	0.016820912
5.333333333	0.000497881	0.012969715	0.016797036
5.416666667	0.000448498	0.012985164	0.016884001
5.5	0.000503055	0.012925893	0.016941149
5.583333333	0.000410776	0.012942769	0.016925489
5.666666667	0.000507977	0.012904532	0.016879239
5.75	0.000447727	0.0129025	0.016901507
5.833333333	0.000385356	0.012968686	0.016952203
5.916666667	0.000352712	0.012952106	0.016947636
6	0.000405115	0.012915115	0.016928687
6.083333333	0.000404054	0.012865953	0.016948507
6.166666667	0.000409406	0.012905554	0.016978312

6.25	0.000427426	0.012943477	0.016971856
6.333333333	0.00038356	0.012871981	0.016925596
6.416666667	0.000347691	0.012885307	0.016947605
6.5	0.00039409	0.012906388	0.016993448
6.583333333	0.000425765	0.012877401	0.016907982
6.666666667	0.000448047	0.012837197	0.016899595
6.75	0.000460081	0.012832714	0.016890986
6.833333333	0.000392467	0.01290488	0.016959148
6.916666667	0.000410327	0.012906327	0.017018772
7	0.000398738	0.012873648	0.016923385
7.083333333	0.000350843	0.012898636	0.017023565
7.166666667	0.000368851	0.012923961	0.017111199
7.25	0.000410812	0.012867528	0.016956393
7.333333333	0.000359758	0.012898173	0.017081688
7.416666667	0.000353538	0.012958742	0.017072405
7.5	0.000442703	0.012819123	0.016917833
7.583333333	0.000325928	0.012888361	0.017030209
7.666666667	0.000392471	0.012879068	0.017069772
7.75	0.000408782	0.01284818	0.016991674
7.833333333	0.000323276	0.012920192	0.017048957
7.916666667	0.000373586	0.012848049	0.017074615
8	0.000374383	0.012847972	0.017074235
8.083333333	0.000448286	0.01281947	0.016967664
8.166666667	0.000392311	0.012874306	0.01704508
8.25	0.000343517	0.012835142	0.017094348
8.333333333	0.000398458	0.012878518	0.017095463
8.416666667	0.000417225	0.012837281	0.017017191
8.5	0.000394627	0.012836365	0.017004782
8.583333333	0.000393759	0.012852429	0.01710298
8.666666667	0.000356449	0.012833897	0.017101542
8.75	0.000382477	0.012801053	0.017001028
8.833333333	0.000511008	0.012788177	0.017008351
8.916666667	0.00045202	0.012768629	0.016979521
9	0.00041714	0.012785574	0.017046574
9.083333333	0.000398307	0.012854197	0.017138496
9.166666667	0.000371416	0.012833064	0.017070213
9.25	0.000383132	0.012815436	0.017096934
9.333333333	0.000319452	0.012899006	0.01713887
9.416666667	0.000440834	0.012816366	0.017019152
9.5	0.000425976	0.012788179	0.017096596
9.583333333	0.000380322	0.012838361	0.017140241
9.666666667	0.000383848	0.012801671	0.017139515
9.75	0.000467918	0.012725303	0.017028461
9.833333333	0.000478706	0.012781864	0.017154886
9.916666667	0.000456937	0.012835755	0.017076474
10	0.000516862	0.012756219	0.01701293
10.08333333	0.00043213	0.012753488	0.017069941
10.16666667	0.000433299	0.012805942	0.017130085
10.25	0.00038867	0.01277923	0.017072181
10.33333333	0.000494985	0.012773793	0.017060047
10.41666667	0.000450861	0.012811034	0.017133211
10.5	0.000519756	0.012707637	0.016993155
10.58333333	0.000473437	0.012740034	0.017026993
10.66666667	0.00041339	0.012804079	0.017084216
10.75	0.000398532	0.012818823	0.017164315
10.83333333	0.000428231	0.012742331	0.017088407
10.91666667	0.000511813	0.012698786	0.016979483
11	0.000449442	0.012771063	0.017162896

11.08333333	0.000479662	0.012710537	0.017126893
11.16666667	0.000456659	0.012753382	0.017055771
11.25	0.000427337	0.01273881	0.017121147
11.33333333	0.000525872	0.012679869	0.017073888
11.41666667	0.000544664	0.012694752	0.017113296
11.5	0.000450426	0.012714099	0.017083011
11.58333333	0.000594133	0.012638384	0.017023204
11.66666667	0.00055349	0.012677041	0.017090576
11.75	0.000466769	0.01273823	0.017076713
11.83333333	0.00054984	0.012651918	0.017073141
11.91666667	0.000432884	0.012756517	0.017142872
12	0.000458227	0.012734061	0.017101449

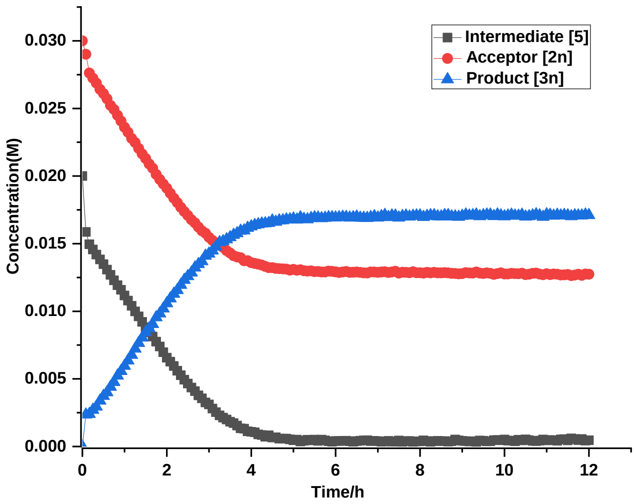

Supplementary Figure S31. Temporal kinetics profile for the monitoring under standard conditions

7.11.2. Concentration dependence studies

7.11.2.1. Intermediate 5 concentration dependence

Procedure: To a dry NMR tube, intermediate **5** (2.15 mg, 0.005 mmol), 100 uL catalyst **J** (0.5 mg, 2.0 mol%, 5 mg in 1 mL CD_2Cl_2 solution), 150 uL acceptor **2n** (1.32 mg, 0.015 mmol, 8.8 mg in 1 mL CD_2Cl_2 solution) and 1,1,2,2-tetrachloroethane (5 μL) as the internal standard, CD_2Cl_2 was added in order to reach a total volume of 500 μL. Afterwards, the tube was sealed with cap and measured on the NMR spectrometer at room temperature by recording ^{1}H spectra at different time.

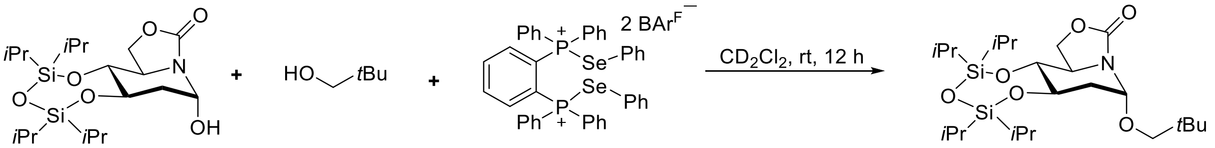

5 **2n** **J** **3n**

0.01 M 0.03 M 2 mol%

Procedure: To a dry NMR tube, intermediate **5** (6.45 mg, 0.015 mmol), 100 uL catalyst **J** (0.5 mg, 2.0 mol%, 5 mg in 1 mL CD_2Cl_2 solution), 150 uL acceptor **2n** (1.32 mg, 0.015 mmol, 8.8 mg in 1 mL CD_2Cl_2 solution) and 1,1,2,2-tetrachloroethane (5 μL) as the internal standard, CD_2Cl_2 was added in order to reach a total volume of 500 μL. Afterwards, the tube was sealed with cap and measured on the NMR spectrometer at room temperature by recording ^{1}H spectra at different time.

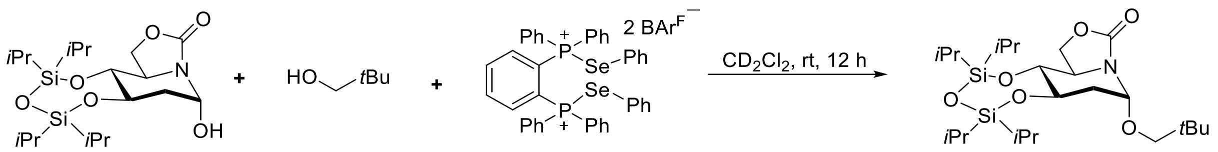

Table S15. Concentration for **3n** calculated by ^{1}H NMR analysis for varying the intermediate concentration experiment

Time/h	**3n** (**5** =0.01M)	**3n** (**5** =0.02M)	**3n** (**5** =0.03M)
0	0	0	0
0.083333333	0.001018559	0.002250139	0.002445645
0.166666667	0.001133386	0.002400277	0.002744085
0.25	0.001361327	0.002707422	0.003140676
0.333333333	0.001409775	0.002943161	0.003489459
0.416666667	0.001657371	0.003087869	0.00378169
0.5	0.001959813	0.003532359	0.004161465
0.583333333	0.00208164	0.003797842	0.004566873
0.666666667	0.002438764	0.004009203	0.005001738
0.75	0.002657091	0.004366604	0.00536088
0.833333333	0.002841954	0.004733963	0.005637585
0.916666667	0.003154602	0.005171606	0.006061223
1	0.003334886	0.005659506	0.006342497
1.083333333	0.00356497	0.006053219	0.00650596
1.166666667	0.003707335	0.006572896	0.006760321
1.25	0.003980393	0.007026405	0.007314329
1.333333333	0.004176297	0.007649822	0.007327426
1.416666667	0.004403842	0.008045616	0.008235678
1.5	0.004679201	0.008446858	0.008373619
1.583333333	0.004945057	0.008725427	0.008793195
1.666666667	0.005079132	0.00905207	0.009318178
1.75	0.005156259	0.009543294	0.009679479
1.833333333	0.005306414	0.009828941	0.009965638
1.916666667	0.005633547	0.01020221	0.010306031
2	0.005630087	0.010587932	0.010657663
2.083333333	0.00607986	0.010946142	0.011320146
2.166666667	0.006393092	0.011286493	0.01146261
2.25	0.006499689	0.011562825	0.011976189
2.333333333	0.006668549	0.011943869	0.012214081
2.416666667	0.006791376	0.012313545	0.012608538
2.5	0.007101646	0.012589046	0.012839328
2.583333333	0.0070823	0.012859592	0.013330337
2.666666667	0.007269776	0.013224876	0.013528915
2.75	0.00744119	0.013483244	0.013884715
2.833333333	0.007575864	0.013693776	0.014376452
2.916666667	0.007514969	0.01409764	0.014492244
3	0.007913145	0.014252346	0.015023635
3.083333333	0.007904412	0.014481878	0.015315648
3.166666667	0.008175984	0.014773471	0.015561329
3.25	0.008353083	0.015099843	0.015879669
3.333333333	0.008486643	0.015176988	0.016321477
3.416666667	0.008474968	0.015310585	0.016602394
3.5	0.008592387	0.015468782	0.016824406
3.583333333	0.008774907	0.015642104	0.017148017
3.666666667	0.008896362	0.015765478	0.017617755
3.75	0.009076068	0.01594802	0.017840733

3.833333333	0.009151265	0.015971532	0.018279496
3.916666667	0.009240418	0.016156747	0.018346837
4	0.009591067	0.016265489	0.018701253
4.083333333	0.009347275	0.01636509	0.019123373
4.166666667	0.009640756	0.016428835	0.019398097
4.25	0.009642731	0.016483845	0.019661915
4.333333333	0.009834102	0.016499361	0.020012031
4.416666667	0.009902493	0.016522247	0.02018868
4.5	0.009934223	0.016701461	0.020635083
4.583333333	0.010147939	0.016605256	0.020697931
4.666666667	0.010162595	0.016710188	0.021479005
4.75	0.010167936	0.01671996	0.021335593
4.833333333	0.010353252	0.016780776	0.021514997
4.916666667	0.010498472	0.016792551	0.021894241
5	0.010448015	0.016849661	0.022620648
5.083333333	0.010596611	0.016772025	0.022244551
5.166666667	0.010658522	0.016944123	0.022316994
5.25	0.010870589	0.016820912	0.022823789
5.333333333	0.011028524	0.016797036	0.022923397
5.416666667	0.010818901	0.016884001	0.023189071
5.5	0.010976796	0.016941149	0.023320765
5.583333333	0.011052014	0.016925489	0.023892454
5.666666667	0.011348478	0.016879239	0.023781154
5.75	0.011247966	0.016901507	0.024538334
5.833333333	0.011121543	0.016952203	0.024128036
5.916666667	0.011316798	0.016947636	0.024493104
6	0.011342117	0.016928687	0.024461672
6.083333333	0.011298645	0.016948507	0.024457297
6.166666667	0.011495984	0.016978312	0.025237001
6.25	0.01139904	0.016971856	0.025042724
6.333333333	0.011622144	0.016925596	0.025280289
6.416666667	0.011469009	0.016947605	0.025661833
6.5	0.011493719	0.016993448	0.025898342
6.583333333	0.011498159	0.016907982	0.026017106
6.666666667	0.01143441	0.016899595	0.026009848
6.75	0.011626853	0.016890986	0.026172538
6.833333333	0.011842182	0.016959148	0.026048654
6.916666667	0.01157513	0.017018772	0.026475339
7	0.011738831	0.016923385	0.026431259
7.083333333	0.011826146	0.017023565	0.027097473
7.166666667	0.011864818	0.017111199	0.02688298
7.25	0.011771961	0.016956393	0.027169938
7.333333333	0.011982375	0.017081688	0.027314479
7.416666667	0.01185099	0.017072405	0.027367546
7.5	0.011831618	0.016917833	0.027765677
7.583333333	0.011775928	0.017030209	0.027911888
7.666666667	0.011659911	0.017069772	0.027915447
7.75	0.011801988	0.016991674	0.028010596
7.833333333	0.012023037	0.017048957	0.028311109
7.916666667	0.012088133	0.017074615	0.028489496
8	0.011974474	0.017074235	0.028467533
8.083333333	0.011694862	0.016967664	0.028675805
8.166666667	0.011803103	0.01704508	0.028781851
8.25	0.011966565	0.017094348	0.028789231
8.333333333	0.012007049	0.017095463	0.029010563
8.416666667	0.011786647	0.017017191	0.029185961
8.5	0.011888207	0.017004782	0.029198392
8.583333333	0.012157391	0.01710298	0.029200782

8.666666667	0.011942335	0.017101542	0.029071177
8.75	0.011963694	0.017001028	0.029410964
8.833333333	0.011927236	0.017008351	0.029588149
8.916666667	0.011765289	0.016979521	0.029701601
9	0.012076118	0.017046574	0.029788813
9.083333333	0.012042156	0.017138496	0.029781257
9.166666667	0.012188771	0.017070213	0.029826892
9.25	0.012033696	0.017096934	0.030111449
9.333333333	0.011946008	0.01713887	0.030252192
9.416666667	0.011786108	0.017019152	0.030359856
9.5	0.012257706	0.017096596	0.030105505
9.583333333	0.012021887	0.017140241	0.03035585
9.666666667	0.011948006	0.017139515	0.030440851
9.75	0.011890653	0.017028461	0.030669549
9.833333333	0.011892945	0.017154886	0.03086399
9.916666667	0.011971231	0.017076474	0.030752184
10	0.011960579	0.01701293	0.030608835
10.08333333	0.012159815	0.017069941	0.030854518
10.16666667	0.011926703	0.017130085	0.030711155
10.25	0.011988333	0.017072181	0.030917265
10.33333333	0.012016388	0.017060047	0.030807634
10.41666667	0.01191057	0.017133211	0.030964283
10.5	0.011965636	0.016993155	0.03087043
10.58333333	0.011952381	0.017026993	0.031134726
10.66666667	0.012200789	0.017084216	0.031103032
10.75	0.012172803	0.017164315	0.031122198
10.83333333	0.01214108	0.017088407	0.03108192
10.91666667	0.011998613	0.016979483	0.031157413
11	0.012037202	0.017162896	0.031244993
11.08333333	0.0121947	0.017126893	0.031248122
11.16666667	0.012129116	0.017055771	0.031260859
11.25	0.011844561	0.017121147	0.03115943
11.33333333	0.011976774	0.017073888	0.031414742
11.41666667	0.012066533	0.017113296	0.031470563
11.5	0.012032365	0.017083011	0.031327453
11.58333333	0.01210186	0.017023204	0.031301431
11.66666667	0.012178324	0.017090576	0.031295062
11.75	0.012135605	0.017076713	0.031334671
11.83333333	0.012125048	0.017073141	0.031333468
11.91666667	0.012046384	0.017142872	0.031620944
12	0.012078365	0.017101449	0.031380164

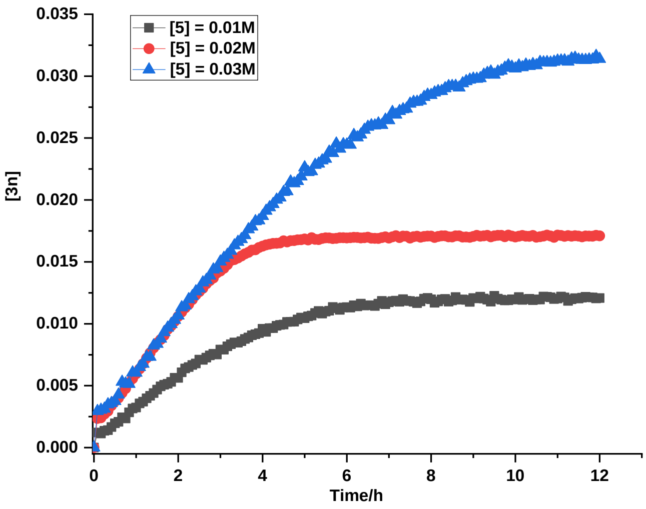

Supplementary Figure S32. Overlapped profile for the intermediate **5** concentration dependence experiments

7.11.2.2. Acceptor 2n concentration dependence

Procedure: To a dry NMR tube, intermediate **5** (4.31 mg, 0.01 mmol), 100 uL catalyst **J** (0.5 mg, 2.0 mol%, 5 mg in 1 mL CD_2Cl_2 solution), 100 uL acceptor **2n** (0.88 mg, 0.01 mmol, 8.8 mg in 1 mL CD_2Cl_2 solution) and 1,1,2,2-tetrachloroethane (5 μL) as the internal standard, CD_2Cl_2 was added in order to reach a total volume of 500 μL. Afterwards, the tube was sealed with cap and measured on the NMR spectrometer at room temperature by recording ^{1}H spectra at different time.

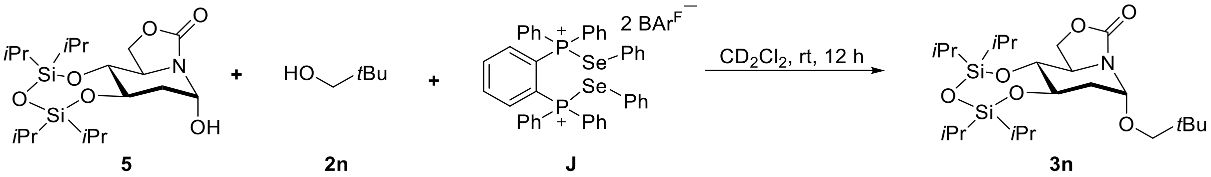

Procedure: To a dry NMR tube, intermediate **5** (4.31 mg, 0.01 mmol), 100 uL catalyst **J** (0.5 mg, 2.0 mol%, 5 mg in 1 mL CD_2Cl_2 solution), 200 uL acceptor **2n** (1.76 mg, 0.02 mmol, 8.8 mg in 1 mL CD_2Cl_2 solution) and 1,1,2,2-tetrachloroethane (5 μL) as the internal standard, CD_2Cl_2 was added in order to reach a total volume of 500 μL. Afterwards, the tube was sealed with cap and measured on the NMR spectrometer at room temperature by recording ^{1}H spectra at different time.

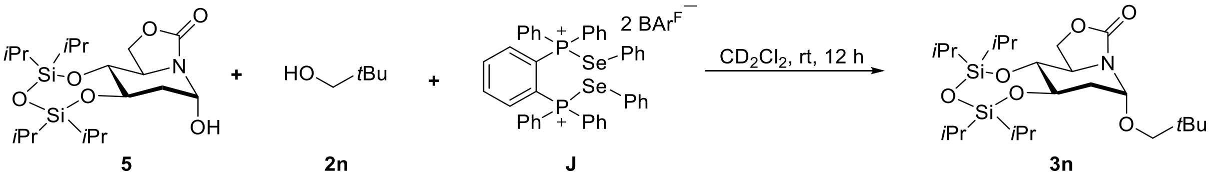

Table S16. Concentration for **3n** calculated by ^{1}H NMR analysis for varying the acceptor concentration experiment

Time/h	**3n** (**2n** =0.02M)	**3n** (**2n** =0.03M)	**3n** (**2n** =0.04M)
0	0	0	0
0.083333333	0.001252007	0.002250139	0.000924891

0.166666667	0.001889968	0.002400277	0.001115415
0.25	0.002627733	0.002707422	0.001411238
0.333333333	0.003405758	0.002943161	0.00163545
0.416666667	0.003994215	0.003387869	0.001905497
0.5	0.004733537	0.003732359	0.002034402
0.583333333	0.005474477	0.003997842	0.00223826
0.666666667	0.005866983	0.004409203	0.002674978
0.75	0.006775113	0.00476666	0.0029193
0.833333333	0.0073818	0.005233963	0.003082433
0.916666667	0.008029887	0.005571606	0.003275703
1	0.008564403	0.005959506	0.003716667
1.083333333	0.00927999	0.006353219	0.003921386
1.166666667	0.009897023	0.006772896	0.004224273
1.25	0.010506217	0.007226405	0.004452735
1.333333333	0.011061636	0.007649822	0.004645836
1.416666667	0.011624209	0.008045616	0.004902049
1.5	0.01205512	0.008446858	0.005380849
1.583333333	0.012539261	0.008725427	0.005630007
1.666666667	0.012817742	0.00905207	0.005809793
1.75	0.012868791	0.009543294	0.006045612
1.833333333	0.012962322	0.009828941	0.006478416
1.916666667	0.013205481	0.01020221	0.006659499
2	0.013239706	0.010587932	0.006988229
2.083333333	0.013316066	0.010946142	0.007224878
2.166666667	0.013262426	0.011286493	0.007446348
2.25	0.013270418	0.011562825	0.007683328
2.333333333	0.013254437	0.011943869	0.007920701
2.416666667	0.013228859	0.012313545	0.008234948
2.5	0.013223457	0.012589046	0.008468021
2.583333333	0.013130611	0.012859592	0.008711187
2.666666667	0.013214	0.013224876	0.008921709
2.75	0.013215	0.013483244	0.009128386
2.833333333	0.013216	0.013693776	0.00934165
2.916666667	0.013217	0.01409764	0.009560342
3	0.013218	0.014252346	0.009612058
3.083333333	0.013219	0.014481878	0.010030172
3.166666667	0.01322	0.014773471	0.010283009
3.25	0.013221	0.015099843	0.010310044
3.333333333	0.013222	0.015176988	0.010667604
3.416666667	0.013223	0.015310585	0.010730345
3.5	0.013224	0.015468782	0.011111139
3.583333333	0.013225	0.015642104	0.011172833
3.666666667	0.013226	0.015765478	0.011556114
3.75	0.013227	0.01594802	0.011668928
3.833333333	0.013228	0.015971532	0.011956653
3.916666667	0.013229	0.016156747	0.011983147
4	0.01323	0.016265489	0.012041128
4.083333333	0.013231	0.01636509	0.012335673
4.166666667	0.013232	0.016428835	0.012610592
4.25	0.013233	0.016483845	0.012679032
4.333333333	0.013234	0.016499361	0.012729532
4.416666667	0.013235	0.016522247	0.012856067
4.5	0.013236	0.016701461	0.012897096
4.583333333	0.013237	0.016605256	0.012964359
4.666666667	0.013238	0.016710188	0.013087684
4.75	0.013239	0.01671996	0.013246528
4.833333333	0.01324	0.016780776	0.013502873
4.916666667	0.013241	0.016792551	0.013312124

5	0.013242	0.016849661	0.013527481
5.083333333	0.013243	0.016772025	0.013507428
5.166666667	0.013244	0.016944123	0.01359324
5.25	0.013245	0.016820912	0.013556902
5.333333333	0.013246	0.016797036	0.01383873
5.416666667	0.013247	0.016884001	0.013926772
5.5	0.013248	0.016941149	0.013930984
5.583333333	0.013249	0.016925489	0.013985518
5.666666667	0.01325	0.016879239	0.014028965
5.75	0.013251	0.016901507	0.014155642
5.833333333	0.013252	0.016952203	0.014076838
5.916666667	0.013253	0.016947636	0.014088985
6	0.013254	0.016928687	0.0140064
6.083333333	0.013255	0.016948507	0.014176955
6.166666667	0.013256	0.016978312	0.014231766
6.25	0.013257	0.016971856	0.014073297
6.333333333	0.013258	0.016925596	0.014069419
6.416666667	0.013259	0.016947605	0.014154298
6.5	0.01326	0.016993448	0.014077799
6.583333333	0.013261	0.016907982	0.014155333
6.666666667	0.013262	0.016899595	0.014107524
6.75	0.013263	0.016890986	0.014104048
6.833333333	0.013264	0.016959148	0.014221869
6.916666667	0.013265	0.017018772	0.014172578
7	0.013266	0.016923385	0.014143029
7.083333333	0.013267	0.017023565	0.014131724
7.166666667	0.013268	0.017111199	0.014172136
7.25	0.013269	0.016956393	0.014265494
7.333333333	0.01327	0.017081688	0.014375901
7.416666667	0.013271	0.017072405	0.014097034
7.5	0.013272	0.016917833	0.014208475
7.583333333	0.013273	0.017030209	0.014054531
7.666666667	0.013274	0.017069772	0.014235771
7.75	0.013275	0.016991674	0.014131538
7.833333333	0.013276	0.017048957	0.014081997
7.916666667	0.013277	0.017074615	0.014069153
8	0.013278	0.017074235	0.014204959
8.083333333	0.013279	0.016967664	0.014263083
8.166666667	0.01328	0.01704508	0.014190256
8.25	0.013281	0.017094348	0.014154041
8.333333333	0.013282	0.017095463	0.014293545
8.416666667	0.013283	0.017017191	0.014188412
8.5	0.013284	0.017004782	0.014135319
8.583333333	0.013285	0.01710298	0.014142062
8.666666667	0.013286	0.017101542	0.014276344
8.75	0.013287	0.017001028	0.014108207
8.833333333	0.013288	0.017008351	0.014098073
8.916666667	0.013289	0.016979521	0.014099845
9	0.01329	0.017046574	0.014029405
9.083333333	0.013291	0.017138496	0.014287226
9.166666667	0.013292	0.017070213	0.014018914
9.25	0.013293	0.017096934	0.014357278
9.333333333	0.013294	0.01713887	0.014003798
9.416666667	0.013295	0.017019152	0.014129729
9.5	0.013296	0.017096596	0.014110809
9.583333333	0.013297	0.017140241	0.014124842
9.666666667	0.013298	0.017139515	0.014090041
9.75	0.013299	0.017028461	0.014018113

9.833333333	0.0133	0.017154886	0.01407576
9.916666667	0.013301	0.017076474	0.014083269
10	0.013302	0.01701293	0.014106545
10.08333333	0.013303	0.017069941	0.014008315
10.16666667	0.013304	0.017130085	0.014081751
10.25	0.013305	0.017072181	0.014111853
10.33333333	0.013306	0.017060047	0.013984845
10.41666667	0.013307	0.017133211	0.014031684
10.5	0.013308	0.016993155	0.01397883
10.58333333	0.013309	0.017026993	0.014183065
10.66666667	0.01331	0.017084216	0.01403771
10.75	0.013311	0.017164315	0.014021125
10.83333333	0.013312	0.017088407	0.013915042
10.91666667	0.013313	0.016979483	0.014201927
11	0.013314	0.017162896	0.014209898
11.08333333	0.013315	0.017126893	0.014168051
11.16666667	0.013316	0.017055771	0.013990742
11.25	0.013317	0.017121147	0.014029849
11.33333333	0.013318	0.017073888	0.01389576
11.41666667	0.013319	0.017113296	0.013910963
11.5	0.01332	0.017083011	0.013933641
11.58333333	0.013321	0.017023204	0.014277404
11.66666667	0.013322	0.017090576	0.014005184
11.75	0.013323	0.017076713	0.013903902
11.83333333	0.013324	0.017073141	0.01412044
11.91666667	0.013325	0.017142872	0.014129621
12	0.013326	0.017101449	0.013875603

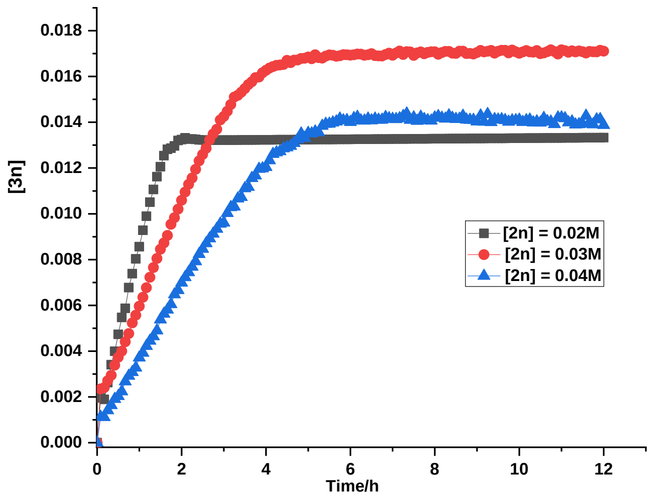

Supplementary Figure S33. Overlapped profile for the acceptor **2n** concentration dependence experiments

7.11.2.3. Catalyst J concentration dependence

Procedure: To a dry NMR tube, intermediate **5** (4.31 mg, 0.01 mmol), 50 uL catalyst **J** (0.25 mg, 1.0 mol%, 5 mg in 1 mL CD_2Cl_2 solution), 150 uL acceptor **2n** (1.32 mg, 0.015 mmol, 8.8 mg in 1 mL CD_2Cl_2 solution) and 1,1,2,2-tetrachloroethane (5 μL) as the internal standard, CD_2Cl_2 was added in order to reach a total volume of 500 μL. Afterwards, the tube was sealed with cap and measured on the NMR spectrometer at room temperature by recording ^{1}H spectra at different time.

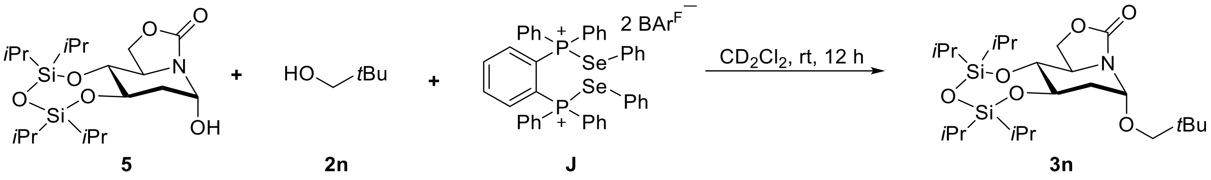

Procedure: To a dry NMR tube, intermediate **5** (4.31 mg, 0.01 mmol), 200 uL catalyst **J** (1.0 mg, 4.0 mol%, 5 mg in 1 mL CD_2Cl_2 solution), 150 uL acceptor **2n** (1.32 mg, 0.015 mmol, 8.8 mg in 1 mL CD_2Cl_2 solution) and 1,1,2,2-tetrachloroethane (5 μL) as the internal standard, CD_2Cl_2 was added in order to reach a total volume of 500 μL. Afterwards, the tube was sealed with cap and measured on the NMR spectrometer at room temperature by recording ^{1}H spectra at different time.

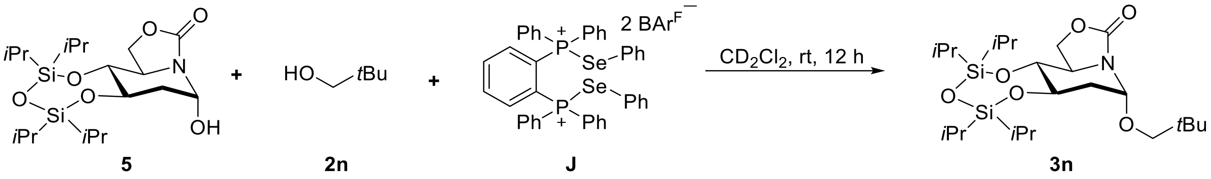

Table S17. Concentration for **3n** calculated by ^{1}H NMR analysis for varying the catalyst concentration experiment

Time/h	**3n** (**J** =1.0 mol%)	**3n** (**J** =2.0 mol%)	**3n** (**J** =4.0 mol%)
0	0	0	0
0.083333333	0.001303396	0.001807902	0.002350139
0.166666667	0.001141359	0.001555842	0.002400277
0.25	0.001356538	0.002004458	0.002707422
0.333333333	0.001501213	0.002410083	0.002943161
0.416666667	0.001674877	0.002749532	0.003387869
0.5	0.002067001	0.003160112	0.003732359
0.583333333	0.002147713	0.003622271	0.003997842
0.666666667	0.002371864	0.004038682	0.004409203
0.75	0.00242771	0.00461082	0.00476666
0.833333333	0.002585764	0.004974012	0.005233963
0.916666667	0.002653771	0.005464259	0.005571606
1	0.002999161	0.005919555	0.005959506
1.083333333	0.003017463	0.006353219	0.006520234
1.166666667	0.003035424	0.006772896	0.006726917
1.25	0.003102649	0.007226405	0.007373659
1.333333333	0.00344962	0.007649822	0.007719528
1.416666667	0.003715008	0.008045616	0.008173315
1.5	0.003612512	0.008446858	0.00871686
1.583333333	0.003909598	0.008725427	0.009203691
1.666666667	0.003991046	0.00905207	0.009675574
1.75	0.00426227	0.009543294	0.010050322
1.833333333	0.004225834	0.009828941	0.010334686
1.916666667	0.004303283	0.01020221	0.010931785
2	0.004487461	0.010587932	0.011193025
2.083333333	0.004584286	0.010946142	0.011751539
2.166666667	0.004977048	0.011286493	0.012323566
2.25	0.00500907	0.011562825	0.012497552
2.333333333	0.005303647	0.011943869	0.012894818
2.416666667	0.005136527	0.012313545	0.013298308
2.5	0.005511159	0.012589046	0.013725874
2.583333333	0.005642238	0.012859592	0.014128029

2.666666667	0.005608459	0.013224876	0.01439336
2.75	0.005709397	0.013483244	0.014697621
2.833333333	0.005930211	0.013693776	0.015072194
2.916666667	0.005973292	0.01409764	0.015480096
3	0.006270684	0.014252346	0.015739519
3.083333333	0.006391275	0.014481878	0.016038024
3.166666667	0.006291027	0.014773471	0.016311658
3.25	0.006381486	0.015099843	0.016476942
3.333333333	0.006598661	0.015176988	0.016857863
3.416666667	0.00679134	0.015310585	0.017071657
3.5	0.006935236	0.015468782	0.017317754
3.583333333	0.007159958	0.015642104	0.017483873
3.666666667	0.007342589	0.015765478	0.017604311
3.75	0.007244659	0.01594802	0.017763921
3.833333333	0.007524521	0.015971532	0.017875384
3.916666667	0.007349194	0.016156747	0.018032751
4	0.007772982	0.016265489	0.018089431
4.083333333	0.007738597	0.01636509	0.018238726
4.166666667	0.007995206	0.016428835	0.018459805
4.25	0.008129173	0.016483845	0.018343419
4.333333333	0.008051917	0.016499361	0.018461418
4.416666667	0.008217736	0.016522247	0.018592097
4.5	0.008185699	0.016701461	0.018449981
4.583333333	0.008621457	0.016605256	0.01855951
4.666666667	0.008582504	0.016710188	0.018666229
4.75	0.00864585	0.01671996	0.018513225
4.833333333	0.008865297	0.016780776	0.018650923
4.916666667	0.00908036	0.016792551	0.018789136
5	0.009258326	0.016849661	0.018763488
5.083333333	0.009284871	0.016772025	0.01856241
5.166666667	0.009427526	0.016944123	0.018740916
5.25	0.009247307	0.016820912	0.018661135
5.333333333	0.009474084	0.016797036	0.018718942
5.416666667	0.009710498	0.016884001	0.018617787
5.5	0.00995853	0.016941149	0.018724067
5.583333333	0.009806011	0.016925489	0.0188222
5.666666667	0.010168296	0.016879239	0.01873457
5.75	0.010126325	0.016901507	0.018829159
5.833333333	0.010510291	0.016952203	0.018746975
5.916666667	0.010341993	0.016947636	0.018803245
6	0.010516166	0.016928687	0.018633879
6.083333333	0.010552799	0.016948507	0.018929508
6.166666667	0.010799132	0.016978312	0.018876821
6.25	0.010825913	0.016971856	0.018715477
6.333333333	0.010900001	0.016925596	0.018829009
6.416666667	0.010915572	0.016947605	0.018621971
6.5	0.011099073	0.016993448	0.018737484
6.583333333	0.011195414	0.016907982	0.018795081
6.666666667	0.011467614	0.016899595	0.018707557
6.75	0.011471732	0.016890986	0.018787145
6.833333333	0.011566463	0.016959148	0.018629535
6.916666667	0.011670704	0.017018772	0.018551556
7	0.011874812	0.016923385	0.0187706
7.083333333	0.011902741	0.017023565	0.018670851
7.166666667	0.012019853	0.017111199	0.018623334
7.25	0.012112865	0.016956393	0.018687658
7.333333333	0.012192892	0.017081688	0.018650773
7.416666667	0.012242374	0.017072405	0.018802718

7.5	0.012377029	0.016917833	0.018591294
7.583333333	0.012598235	0.017030209	0.018527817
7.666666667	0.012430628	0.017069772	0.018501518
7.75	0.012696395	0.016991674	0.018607806
7.833333333	0.012935524	0.017048957	0.018617147
7.916666667	0.012740097	0.017074615	0.018810813
8	0.012832229	0.017074235	0.018696684
8.083333333	0.013271483	0.016967664	0.01891257
8.166666667	0.012958466	0.01704508	0.018638277
8.25	0.013248218	0.017094348	0.018608624
8.333333333	0.013325507	0.017095463	0.018678651
8.416666667	0.013500851	0.017017191	0.018518142
8.5	0.013779755	0.017004782	0.018674764
8.583333333	0.013671749	0.01710298	0.018659752
8.666666667	0.013784402	0.017101542	0.018610757
8.75	0.013796856	0.017001028	0.018706204
8.833333333	0.013788285	0.017008351	0.018944511
8.916666667	0.013991646	0.016979521	0.018774705
9	0.01400472	0.017046574	0.01856988
9.083333333	0.014189505	0.017138496	0.018597872
9.166666667	0.014238821	0.017070213	0.018495216
9.25	0.014245	0.017096934	0.01856475
9.333333333	0.014243	0.01713887	0.018586372
9.416666667	0.014257358	0.017019152	0.018591092
9.5	0.014263537	0.017096596	0.018634777
9.583333333	0.014269715	0.017140241	0.01860652
9.666666667	0.01400472	0.017139515	0.01857478
9.75	0.014189505	0.017028461	0.018566159
9.833333333	0.014238821	0.017154886	0.018650463
9.916666667	0.014245	0.017076474	0.018498935
10	0.014251179	0.01701293	0.018681397
10.08333333	0.014257358	0.017069941	0.018437202
10.16666667	0.014263537	0.017130085	0.018758952
10.25	0.014269715	0.017072181	0.018744347
10.33333333	0.014189505	0.017060047	0.01848702
10.41666667	0.014238821	0.017133211	0.018772457
10.5	0.014245	0.016993155	0.018597872
10.58333333	0.014251179	0.017026993	0.018495216
10.66666667	0.014257358	0.017084216	0.01856475
10.75	0.014263537	0.017164315	0.018586372
10.83333333	0.014269715	0.017088407	0.018591092
10.91666667	0.01400472	0.016979483	0.018634777
11	0.014189505	0.017162896	0.01860652
11.08333333	0.014238821	0.017126893	0.01857478
11.16666667	0.014245	0.017055771	0.018566159
11.25	0.014251179	0.017121147	0.018650463
11.33333333	0.014257358	0.017073888	0.018498935
11.41666667	0.014263537	0.017113296	0.018681397
11.5	0.014269715	0.017083011	0.018437202
11.58333333	0.014189505	0.017023204	0.018758952
11.66666667	0.014238821	0.017090576	0.018744347
11.75	0.014245	0.017076713	0.01848702
11.83333333	0.014251179	0.017073141	0.018772457
11.91666667	0.014257358	0.017142872	0.018495216
12	0.014263537	0.017101449	0.01856475

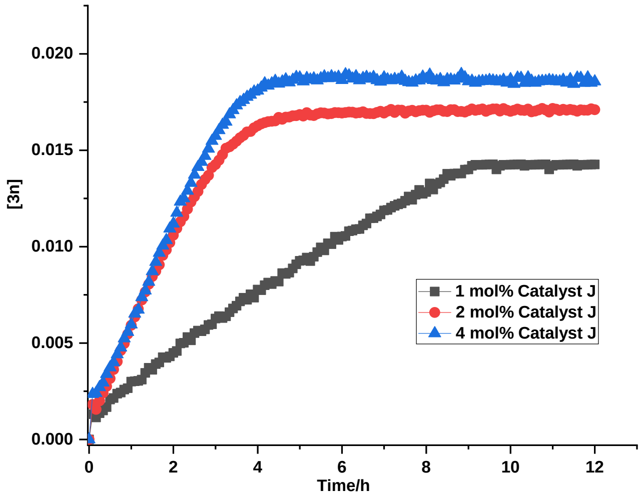

Supplementary Figure S34. Overlapped profile for the catalyst **J** concentration dependence experiments

7.12. Kinetic analysis

7.12.1. The order of catalyst J, donor 1a, acceptor 2n of the overall reaction

7.12.1.1. Burés method to determine order with respect to catalyst J[13,14]

Table S18. Concentration for **3n** & Product formation over time multiplied by [catalyst] to the 1.2 power.

	1.0 mol% catalyst **J**		2.0 mol% catalyst **J**		4.0 mol% catalyst **J**	
Time/min	t[**cat**]$^{1.2}$	**[3n]**	t[**cat**]$^{1.2}$	**[3n]**	t[**cat**]$^{1.2}$	**[3n]**
0	0	0.0003	0.0000	0.00745	0.0000	0.00484
15	0.01131	0.0033	0.0259	0.01288	0.0596	0.01734
30	0.02263	0.0061	0.0519	0.01894	0.1191	0.02969
45	0.03394	0.0086	0.0778	0.02489	0.1787	0.04093
60	0.04526	0.0118	0.1037	0.03089	0.2383	0.05256
75	0.05657	0.0149	0.1297	0.03682	0.2979	0.06478
90	0.06788	0.0177	0.1556	0.04275	0.3574	0.07613
105	0.0792	0.0204	0.1815	0.04856	0.4170	0.08771
120	0.09051	0.0238	0.2074	0.05458	0.4766	0.09891
135	0.10183	0.0257	0.2334	0.06029	0.5362	0.11031
150	0.11314	0.0290	0.2593	0.06608	0.5957	0.12062
165	0.12445	0.0320	0.2852	0.07177	0.6553	0.13113
180	0.13577	0.0344	0.3112	0.07739	0.7149	0.1405
195	0.14708	0.0366	0.3371	0.08297	0.7744	0.14999
210	0.1584	0.0397	0.3630	0.0886	0.8340	0.15838
225	0.16971	0.0421	0.3890	0.09379	0.8936	0.16598
240	0.18103	0.0451	0.4149	0.09926	0.9532	0.17261
255	0.19234	0.0478	0.4408	0.10464	1.0127	0.17852
270	0.20365	0.0505	0.4668	0.10964	1.0723	0.18353
285	0.21497	0.0535	0.4927	0.11485	1.1319	0.18768
300	0.22628	0.0558	0.5186	0.11969	1.1915	0.19078
315	0.2376	0.0584	0.5445	0.12481	1.2510	0.19373
330	0.24891	0.0613	0.5705	0.12914	1.3106	0.19577
345	0.26022	0.0636	0.5964	0.13375	1.3702	0.19739
360	0.27154	0.0662	0.6223	0.13793	1.4297	0.19872
375	0.28285	0.0690	0.6483	0.14214	1.4893	0.19969

390	0.29417	0.0714	0.6742	0.14609	1.5489	0.20068
405	0.30548	0.0741	0.7001	0.14993	1.6085	0.20131
420	0.31679	0.0767	0.7261	0.15343	1.6680	0.20204
435	0.32811	0.0797	0.7520	0.15696	1.7276	0.20186
450	0.33942	0.0815	0.7779	0.16033	1.7872	0.20232
465	0.35074	0.0843	0.8038	0.1632	1.8468	0.20287
480	0.36205	0.0865	0.8298	0.1661	1.9063	0.20307
495	0.37336	0.0890	0.8557	0.16864	1.9659	0.20319
510	0.38468	0.0920	0.8816	0.17043	2.0255	0.20304
525	0.39599	0.0938	0.9076	0.17298	2.0850	0.20346
540	0.40731	0.0967	0.9335	0.17487	2.1446	0.20339
555	0.41862	0.0991	0.9594	0.17669	2.2042	0.20321
570	0.42994	0.1008	0.9854	0.17842	2.2638	0.20343
585	0.44125	0.1032	1.0113	0.1796	2.3233	0.20323
600	0.45256	0.1054	1.0372	0.18094	2.3829	0.20333
615	0.46388	0.1079	1.0632	0.18198	2.4425	0.20359
630	0.47519	0.1103	1.0891	0.18291	2.5021	0.20355
645	0.48651	0.1128	1.1150	0.18375	2.5616	0.20341
660	0.49782	0.1145	1.1409	0.18467	2.6212	0.20314
675	0.50913	0.1174	1.1669	0.18589	2.6808	0.20328
690	0.52045	0.1190	1.1928	0.18653	2.7403	0.20374
705	0.53176	0.1212	1.2187	0.18647	2.7999	0.20398
720	0.54308	0.1228	1.2447	0.18694	2.8595	0.20367
735	0.55439	0.1250	1.2706	0.18791	2.9191	0.20334
750	0.5657	0.1277	1.2965	0.18768	2.9786	0.20313
765	0.57702	0.1290	1.3225	0.18755	3.0382	0.20348
780	0.58833	0.1310	1.3484	0.18811	3.0978	0.20338
795	0.59965	0.1331	1.3743	0.18795	3.1574	0.20352
810	0.61096	0.1350	1.4003	0.18848	3.2169	0.20317
825	0.62227	0.1363	1.4262	0.18862	3.2765	0.20312
840	0.63359	0.1383	1.4521	0.18866	3.3361	0.2035
855	0.6449	0.1400	1.4780	0.18885	3.3956	0.20356
870	0.65622	0.1418	1.5040	0.18886	3.4552	0.20357
885	0.66753	0.1437	1.5299	0.18908	3.5148	0.20382
900	0.67884	0.1442	1.5558	0.189	3.5744	0.20303
915	0.69016	0.1459	1.5818	0.18897	3.6339	0.20331
930	0.70147	0.1479	1.6077	0.1888	3.6935	0.20349
945	0.71279	0.1496	1.6336	0.18931	3.7531	0.20351
960	0.7241	0.1510	1.6596	0.18914	3.8127	0.20336
975	0.73542	0.1521	1.6855	0.18924	3.8722	0.20284
990	0.74673	0.1532	1.7114	0.18915	3.9318	0.20305
1005	0.75804	0.1544	1.7373	0.18914	3.9914	0.20315
1020	0.76936	0.1555	1.7633	0.18901	4.0509	0.20325
1035	0.78067	0.1571	1.7892	0.1891	4.1105	0.20342
1050	0.79199	0.1585	1.8151	0.18994	4.1701	0.20309
1065	0.8033	0.1589	1.8411	0.18894	4.2297	0.20246
1080	0.81461	0.1603	1.8670	0.18885	4.2892	0.20294
1095	0.82593	0.1611	1.8929	0.18905	4.3488	0.20285
1110	0.83724	0.1613	1.9189	0.18907	4.4084	0.20299
1125	0.84856	0.1633	1.9448	0.18896	4.4680	0.20347
1140	0.85987	0.1637	1.9707	0.18912	4.5275	0.20332
1155	0.87118	0.1652	1.9967	0.18907	4.5871	0.20257
1170	0.8825	0.1655	2.0226	0.18909	4.6467	0.20232
1185	0.89381	0.1657	2.0485	0.18918	4.7063	0.20288
1200	0.90513	0.1672	2.0744	0.18897	4.7658	0.20268

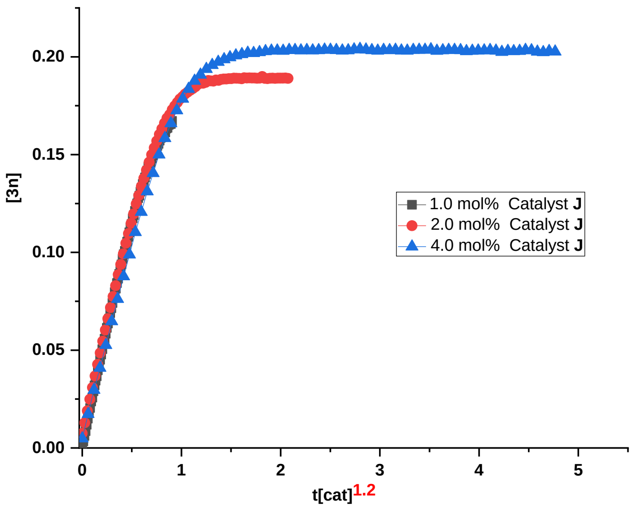

Supplementary Figure S35. Product formation over time multiplied by [**catalyst J**] to the 1.2 power. Graphical overlay represents a 1.2 dependence.

7.12.1.2. Initial rate method to determine order with respect to donor 1a

Table S19. Initial rate of donor **3n** under different concentration of donor **1a**.

Time/mins	[**3n**]$_{initial}$ (Donor **1a** = 0.125M)	[**3n**]$_{initial}$ (Donor **1a** = 0.25M)	[**3n**]$_{initial}$ (Donor **1a** = 0.375M)
15	0.010149164	0.012875205	0.023171831
30	0.015840583	0.018941507	0.031556055
45	0.02029985	0.024891528	0.040501979
60	0.023920862	0.030891828	0.048623108
75	0.027601678	0.036824752	0.055538261
90	0.030641474	0.04274904	0.062253341
105	0.033412465	0.04856081	0.068730045

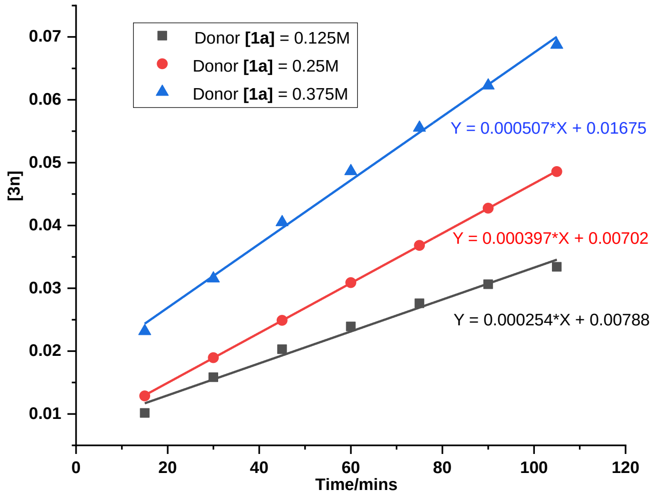

Supplementary Figure S36. Initial rate of product **3n** under different concentration of donor **1a**.

Table S20. Determination of order with respect to donor **1a**.

Entry	Concentration of **1a**	**[1a]**$^{0.5}$	Reaction rate
1	0.125M	0.353553391	0.000254 mmol/min
2	0.25M	0.5	0.000397 mmol/min
3	0.375M	0.612372436	0.000507 mmol/min

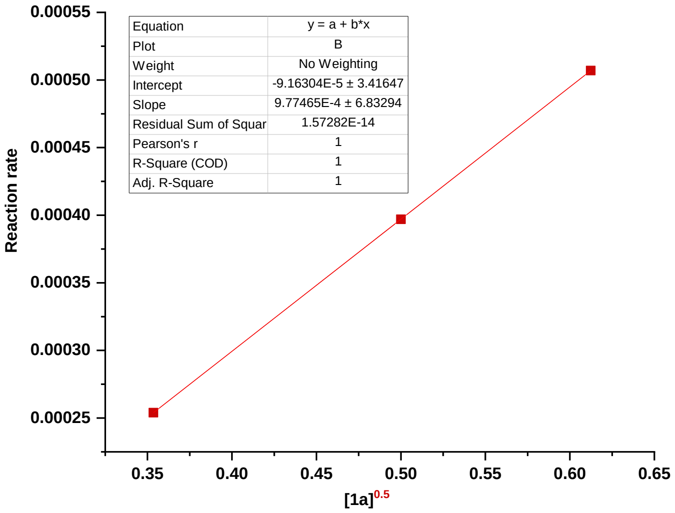

Supplementary Figure S37. Determination of order w.r.t **1a** using initial rate method

7.12.1.3 Initial rate method to determine order with respect to acceptor 2n

Table S21. Initial rate of donor **3n** under different concentration of acceptor **2n**.

Time/mins	**[3n]**$_{initial}$ (Acceptor **2n** = 0.25M)	**[3n]**$_{initial}$ (Acceptor **2n** = 0.375M)	**[3n]**$_{initial}$ (Acceptor **2n** = 0.50M)
15	0.01376	0.01288	0.00613
30	0.02395	0.01894	0.00732
45	0.03726	0.02489	0.0104
60	0.04889	0.03089	0.01212
75	0.06082	0.03682	0.01615
90	0.07271	0.04275	0.01775
105	0.08481	0.04856	0.02091
120	0.09676	0.05458	0.02313
135	0.10868	0.06029	0.02632
150	0.12092	0.06608	0.02803
165	0.13275	0.07177	0.03049
180	0.14446	0.07739	0.03535

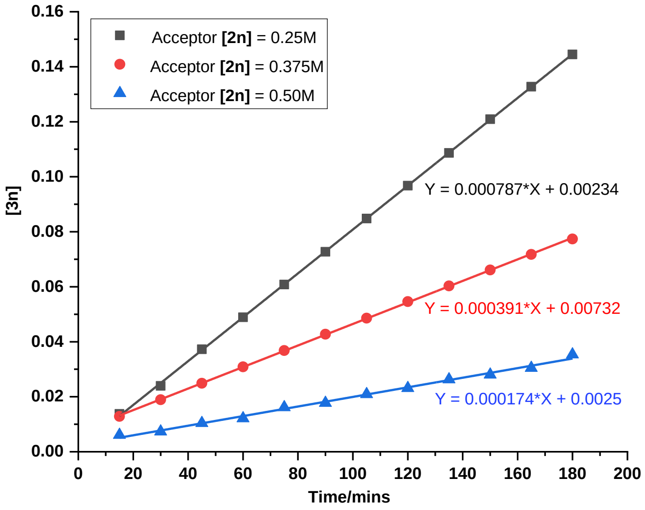

Supplementary Figure S38. Initial rate of product **3n** under different concentration of acceptor **2n**.

Table S22. Determination of order with respect to acceptor **2n**.

Entry	Concentration of **2n**	$[\mathbf{2n}]^{-0.7}$	Reaction rate
1	0.25M	2.639015822	0.000787 mmol/min
2	0.375M	1.98690962	0.000391 mmol/min
3	0.50M	1.624504793	0.000174 mmol/min

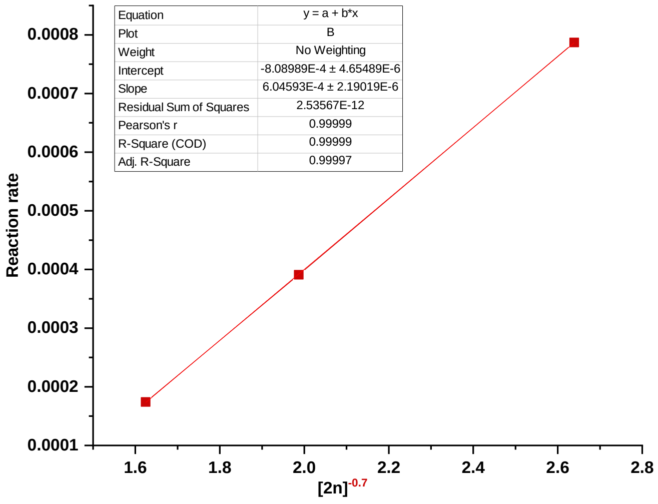

Supplementary Figure S39. Determination of order w.r.t **2n** using initial rate method

7.12.2 The order of catalyst J, intermediate 5, acceptor 2n of the downstream step

7.12.2.1 Burés method to determine order with respect to catalyst J[13,14]

Table S23. Concentration for **3n** & Product formation over time multiplied by [catalyst] to the 1.1 power.

	1.0 mol% catalyst **J**		2.0 mol% catalyst **J**		4.0 mol% catalyst **J**	
Time/min	t**[cat]**$^{1.1}$	**[3n]**	t**[cat]**$^{1.1}$	**[3n]**	t**[cat]**$^{1.1}$	**[3n]**
0	0	0	0	0	0	0
5	0.000426681	0.001303396	0.001117726	0.002250139	0.001428685	0.002350139
10	0.000853361	0.001141359	0.002235452	0.002400277	0.002857369	0.002400277
15	0.001280042	0.001356538	0.003353178	0.002707422	0.004286054	0.002707422
20	0.001706723	0.001501213	0.004470904	0.002943161	0.005714739	0.002943161
25	0.002133404	0.001674877	0.00558863	0.003387869	0.007143423	0.003387869
30	0.002560084	0.002067001	0.006706356	0.003732359	0.008572108	0.003732359
35	0.002986765	0.002147713	0.007824082	0.003997842	0.010000793	0.003997842
40	0.003413446	0.002371864	0.008941808	0.004409203	0.011429477	0.004409203
45	0.003840126	0.00242771	0.010059534	0.00476666	0.012858162	0.00476666
50	0.004266807	0.002585764	0.01117726	0.005233963	0.014286847	0.005233963
55	0.004693488	0.002653771	0.012294986	0.005571606	0.015715531	0.005571606
60	0.005120168	0.002999161	0.013412712	0.005959506	0.017144216	0.005959506
65	0.005546849	0.003017463	0.014530438	0.006353219	0.0185729	0.006520234
70	0.00597353	0.003035424	0.015648164	0.006772896	0.020001585	0.006726917
75	0.006400211	0.003102649	0.01676589	0.007226405	0.02143027	0.007373659
80	0.006826891	0.00344962	0.017883616	0.007649822	0.022858954	0.007719528
85	0.007253572	0.003715008	0.019001343	0.008045616	0.024287639	0.008173315
90	0.007680253	0.003612512	0.020119069	0.008446858	0.025716324	0.00871686
95	0.008106933	0.003909598	0.021236795	0.008725427	0.027145008	0.009203691
100	0.008533614	0.003991046	0.022354521	0.00905207	0.028573693	0.009675574
105	0.008960295	0.00426227	0.023472247	0.009543294	0.030002378	0.010050322
110	0.009386975	0.004225834	0.024589973	0.009828941	0.031431062	0.010334686
115	0.009813656	0.004303283	0.025707699	0.01020221	0.032859747	0.010931785
120	0.010240337	0.004487461	0.026825425	0.010587932	0.034288432	0.011193025
125	0.010667018	0.004584286	0.027943151	0.010946142	0.035717116	0.011751539
130	0.011093698	0.004977048	0.029060877	0.011286493	0.037145801	0.012323566
135	0.011520379	0.00500907	0.030178603	0.011562825	0.038574486	0.012497552
140	0.01194706	0.005303647	0.031296329	0.011943869	0.04000317	0.012894818
145	0.01237374	0.005136527	0.032414055	0.012313545	0.041431855	0.013298308
150	0.012800421	0.005511159	0.033531781	0.012589046	0.04286054	0.013725874
155	0.013227102	0.005642238	0.034649507	0.012859592	0.044289224	0.014128029
160	0.013653782	0.005608459	0.035767233	0.013224876	0.045717909	0.01439336
165	0.014080463	0.005709397	0.036884959	0.013483244	0.047146594	0.014697621
170	0.014507144	0.005930211	0.038002685	0.013693776	0.048575278	0.015072194
175	0.014933825	0.005973292	0.039120411	0.01409764	0.050003963	0.015480096
180	0.015360505	0.006270684	0.040238137	0.014252346	0.051432648	0.015739519
185	0.015787186	0.006391275	0.041355863	0.014481878	0.052861332	0.016038024
190	0.016213867	0.006291027	0.042473589	0.014773471	0.054290017	0.016311658
195	0.016640547	0.006381486	0.043591315	0.015099843	0.055718701	0.016476942
200	0.017067228	0.006598661	0.044709041	0.015176988	0.057147386	0.016857863
205	0.017493909	0.00679134	0.045826767	0.015310585	0.058576071	0.017071657
210	0.017920589	0.006935236	0.046944493	0.015468782	0.060004755	0.017317754
215	0.01834727	0.007159958	0.048062219	0.015642104	0.06143344	0.017483873
220	0.018773951	0.007342589	0.049179945	0.015765478	0.062862125	0.017604311
225	0.019200632	0.007244659	0.050297671	0.01594802	0.064290809	0.017763921
230	0.019627312	0.007524521	0.051415397	0.015971532	0.065719494	0.017875384
235	0.020053993	0.007349194	0.052533123	0.016156747	0.067148179	0.018032751
240	0.020480674	0.007772982	0.053650849	0.016265489	0.068576863	0.018089431
245	0.020907354	0.007738597	0.054768576	0.01636509	0.070005548	0.018238726
250	0.021334035	0.007995206	0.055886302	0.016428835	0.071434233	0.018459805
255	0.021760716	0.008129173	0.057004028	0.016483845	0.072862917	0.018343419
260	0.022187396	0.008051917	0.058121754	0.016499361	0.074291602	0.018461418
265	0.022614077	0.008217736	0.05923948	0.016522247	0.075720287	0.018592097
270	0.023040758	0.008185699	0.060357206	0.016701461	0.077148971	0.018449981
275	0.023467439	0.008621457	0.061474932	0.016605256	0.078577656	0.01855951
280	0.023894119	0.008582504	0.062592658	0.016710188	0.080006341	0.018666229

285	0.0243208	0.00864585	0.063710384	0.01671996	0.081435025	0.018513225
290	0.024747481	0.008865297	0.06482811	0.016780776	0.08286371	0.018650923
295	0.025174161	0.00908036	0.065945836	0.016792551	0.084292395	0.018789136
300	0.025600842	0.009258326	0.067063562	0.016849661	0.085721079	0.018763488
305	0.026027523	0.009284871	0.068181288	0.016772025	0.087149764	0.01856241
310	0.026454203	0.009427526	0.069299014	0.016944123	0.088578449	0.018740916
315	0.026880884	0.009247307	0.07041674	0.016820912	0.090007133	0.018661135
320	0.027307565	0.009474084	0.071534466	0.016797036	0.091435818	0.018718942
325	0.027734246	0.009710498	0.072652192	0.016884001	0.092864502	0.018617787
330	0.028160926	0.00995853	0.073769918	0.016941149	0.094293187	0.018724067
335	0.028587607	0.009806011	0.074887644	0.016925489	0.095721872	0.0188222
340	0.029014288	0.010168296	0.07600537	0.016879239	0.097150556	0.01873457
345	0.029440968	0.010126325	0.077123096	0.016901507	0.098579241	0.018829159
350	0.029867649	0.010510291	0.078240822	0.016952203	0.100007926	0.018746975
355	0.03029433	0.010341993	0.079358548	0.016947636	0.10143661	0.018803245
360	0.03072101	0.010516166	0.080476274	0.016928687	0.102865295	0.018633879
365	0.031147691	0.010552799	0.081594	0.016948507	0.10429398	0.018929508
370	0.031574372	0.010799132	0.082711726	0.016978312	0.105722664	0.018876821
375	0.032001053	0.010825913	0.083829452	0.016971856	0.107151349	0.018715477
380	0.032427733	0.010900001	0.084947178	0.016925596	0.108580034	0.018829009
385	0.032854414	0.010915572	0.086064904	0.016947605	0.110008718	0.018621971
390	0.033281095	0.011099073	0.08718263	0.016993448	0.111437403	0.018737484
395	0.033707775	0.011195414	0.088300356	0.016907982	0.112866088	0.018795081
400	0.034134456	0.011467614	0.089418082	0.016899595	0.114294772	0.018707557
405	0.034561137	0.011471732	0.090535809	0.016890986	0.115723457	0.018787145
410	0.034987817	0.011566463	0.091653535	0.016959148	0.117152142	0.018629535
415	0.035414498	0.011670704	0.092771261	0.017018772	0.118580826	0.018551556
420	0.035841179	0.011874812	0.093888987	0.016923385	0.120009511	0.0187706
425	0.03626786	0.011902741	0.095006713	0.017023565	0.121438196	0.018670851
430	0.03669454	0.012019853	0.096124439	0.017111199	0.12286688	0.018623334
435	0.037121221	0.012112865	0.097242165	0.016956393	0.124295565	0.018687658
440	0.037547902	0.012192892	0.098359891	0.017081688	0.12572425	0.018650773
445	0.037974582	0.012242374	0.099477617	0.017072405	0.127152934	0.018802718
450	0.038401263	0.012377029	0.100595343	0.016917833	0.128581619	0.018591294
455	0.038827944	0.012598235	0.101713069	0.017030209	0.130010303	0.018527817
460	0.039254624	0.012430628	0.102830795	0.017069772	0.131438988	0.018501518
465	0.039681305	0.012696395	0.103948521	0.016991674	0.132867673	0.018607806
470	0.040107986	0.012935524	0.105066247	0.017048957	0.134296357	0.018617147
475	0.040534667	0.012740097	0.106183973	0.017074615	0.135725042	0.018810813
480	0.040961347	0.012832229	0.107301699	0.017074235	0.137153727	0.018696684
485	0.041388028	0.013271483	0.108419425	0.016967664	0.138582411	0.01891257
490	0.041814709	0.012958466	0.109537151	0.01704508	0.140011096	0.018638277
495	0.042241389	0.013248218	0.110654877	0.017094348	0.141439781	0.018608624
500	0.04266807	0.013325507	0.111772603	0.017095463	0.142868465	0.018678651
505	0.043094751	0.013500851	0.112890329	0.017017191	0.14429715	0.018518142
510	0.043521431	0.013779755	0.114008055	0.017004782	0.145725835	0.018674764
515	0.043948112	0.013671749	0.115125781	0.01710298	0.147154519	0.018659752
520	0.044374793	0.013784402	0.116243507	0.017101542	0.148583204	0.018610757
525	0.044801474	0.013796856	0.117361233	0.017001028	0.150011889	0.018706204
530	0.045228154	0.013788285	0.118478959	0.017008351	0.151440573	0.018944511
535	0.045654835	0.013991646	0.119596685	0.016979521	0.152869258	0.018774705
540	0.046081516	0.01400472	0.120714411	0.017046574	0.154297943	0.01856988
545	0.046508196	0.014189505	0.121832137	0.017138496	0.155726627	0.018597872
550	0.046934877	0.014238821	0.122949863	0.017070213	0.157155312	0.018495216
555	0.047361558	0.014245	0.124067589	0.017096934	0.158583997	0.01856475
560	0.047788238	0.014243	0.125185315	0.01713887	0.160012681	0.018586372
565	0.048214919	0.014257358	0.126303042	0.017019152	0.161441366	0.018591092
570	0.0486416	0.014263537	0.127420768	0.017096596	0.162870051	0.018634777
575	0.049068281	0.014269715	0.128538494	0.017140241	0.164298735	0.01860652
580	0.049494961	0.01400472	0.12965622	0.017139515	0.16572742	0.01857478
585	0.049921642	0.014189505	0.130773946	0.017028461	0.167156104	0.018566159
590	0.050348323	0.014238821	0.131891672	0.017154886	0.168584789	0.018650463
595	0.050775003	0.014245	0.133009398	0.017076474	0.170013474	0.018498935
600	0.051201684	0.014251179	0.134127124	0.01701293	0.171442158	0.018681397

605	0.051628365	0.014257358	0.13524485	0.017069941	0.172870843	0.018437202
610	0.052055045	0.014263537	0.136362576	0.017130085	0.174299528	0.018758952
615	0.052481726	0.014269715	0.137480302	0.017072181	0.175728212	0.018744347
620	0.052908407	0.014189505	0.138598028	0.017060047	0.177156897	0.01848702
625	0.053335088	0.014238821	0.139715754	0.017133211	0.178585582	0.018772457
630	0.053761768	0.014245	0.14083348	0.016993155	0.180014266	0.018597872
635	0.054188449	0.014251179	0.141951206	0.017026993	0.181442951	0.018495216
640	0.05461513	0.014257358	0.143068932	0.017084216	0.182871636	0.01856475
645	0.05504181	0.014263537	0.144186658	0.017164315	0.18430032	0.018586372
650	0.055468491	0.014269715	0.145304384	0.017088407	0.185729005	0.018591092
655	0.055895172	0.01400472	0.14642211	0.016979483	0.18715769	0.018634777
660	0.056321852	0.014189505	0.147539836	0.017162896	0.188586374	0.01860652
665	0.056748533	0.014238821	0.148657562	0.017126893	0.190015059	0.01857478
670	0.057175214	0.014245	0.149775288	0.017055771	0.191443744	0.018566159
675	0.057601895	0.014251179	0.150893014	0.017121147	0.192872428	0.018650463
680	0.058028575	0.014257358	0.15201074	0.017073888	0.194301113	0.018498935
685	0.058455256	0.014263537	0.153128466	0.017113296	0.195729798	0.018681397
690	0.058881937	0.014269715	0.154246192	0.017083011	0.197158482	0.018437202
695	0.059308617	0.014189505	0.155363918	0.017023204	0.198587167	0.018758952
700	0.059735298	0.014238821	0.156481644	0.017090576	0.200015852	0.018744347
705	0.060161979	0.014245	0.15759937	0.017076713	0.201444536	0.01848702
710	0.060588659	0.014251179	0.158717096	0.017073141	0.202873221	0.018772457
715	0.06101534	0.014257358	0.159834822	0.017142872	0.204301905	0.018495216
720	0.061442021	0.014263537	0.160952548	0.017101449	0.20573059	0.01856475

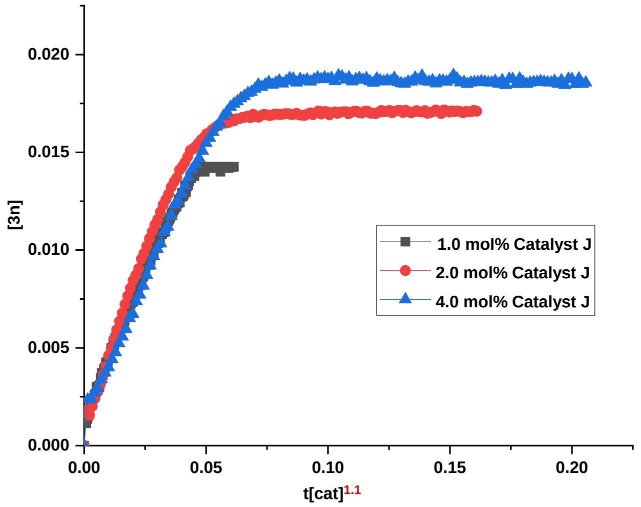

Supplementary Figure S40. Product formation over time multiplied by [**catalyst J**] to the 1.1 power. Graphical overlay represents a 1.1 dependence.

7.12.2.2. Initial rate method to determine order with respect to intermediate 5

Table S24. Initial rate of donor **3n** under different concentration of intermediate **5**.

Time/mins	[**3n**]$_{initial}$ (Intermediate **5** = 0.01M)	[**3n**]$_{initial}$ (Intermediate **5** = 0.02M)	[**3n**]$_{initial}$ (Intermediate **5** = 0.03M)
5	0.001018559	0.002250139	0.002445645
10	0.001133386	0.002400277	0.002744085
15	0.001361327	0.002707422	0.003140676
20	0.001409775	0.002943161	0.003489459
25	0.001657371	0.003387869	0.00378169
30	0.001959813	0.003732359	0.004161465
35	0.00208164	0.003997842	0.004566873
40	0.002438764	0.004409203	0.005001738

45	0.002657091	0.00476666	0.00536088
50	0.002841954	0.005233963	0.005637585
55	0.003154602	0.005571606	0.006061223
60	0.003334886	0.005959506	0.006342497

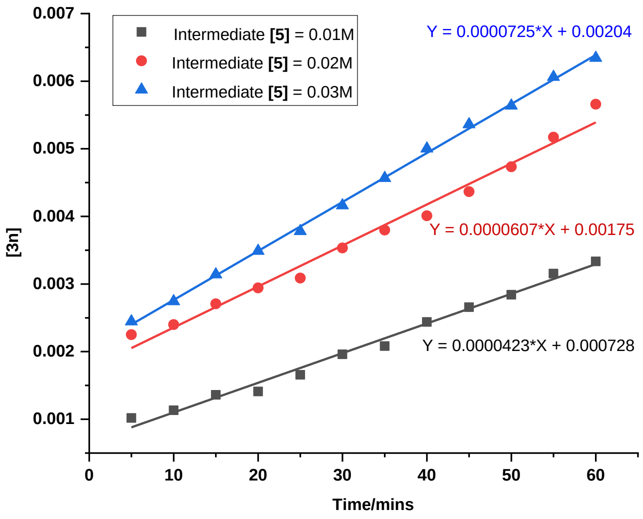

Supplementary Figure S41. Initial rate of product **3n** under different concentration of intermediate **5**.

Table S25. Determination of order with respect to intermediate **5**.

Entry	Concentration of **5**	**[5]**$^{0.2}$	Reaction rate
1	0.01M	0.398107171	0.0000423 mmol/min
2	0.02M	0.457305052	0.0000607 mmol/min
3	0.03M	0.49593442	0.0000725 mmol/min

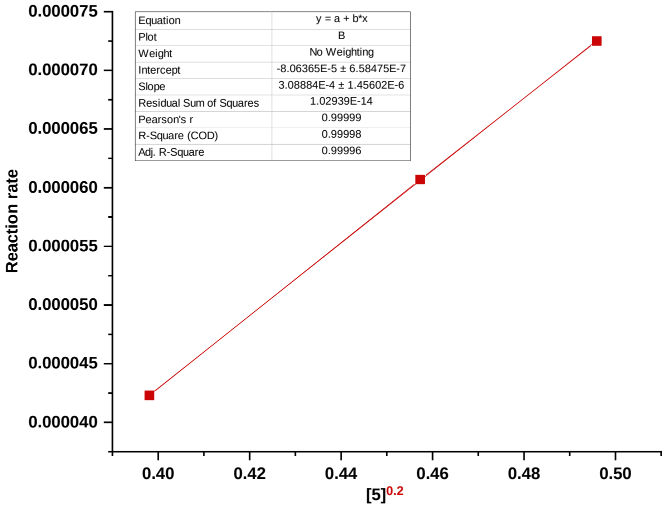

Supplementary Figure S42. Determination of order w.r.t **5** using initial rate method

7.12.2.3. Initial rate method to determine order with respect to acceptor 2n of the downstream step

Table S26. Initial rate of donor **3n** under different concentration of acceptor **2n**.

Time/mins	**[3n]**$_{initial}$ (Acceptor **[2n]** = 0.02M)	**[3n]**$_{initial}$ (Acceptor **[2n]** = 0.03M)	**[3n]**$_{initial}$ (Acceptor **[2n]** = 0.04M)
5	0.001252007	0.002250139	0.000924891
10	0.001889968	0.002400277	0.001115415
15	0.002627733	0.002707422	0.001411238
20	0.003405758	0.002943161	0.00163545
25	0.003994215	0.003387869	0.001905497
30	0.004733537	0.003732359	0.002034402
35	0.005474477	0.003997842	0.00223826
40	0.005866983	0.004409203	0.002674978
45	0.006775113	0.00476666	0.0029193
50	0.0073818	0.005233963	0.003082433
55	0.008029887	0.005571606	0.003275703
60	0.008564403	0.005959506	0.003716667

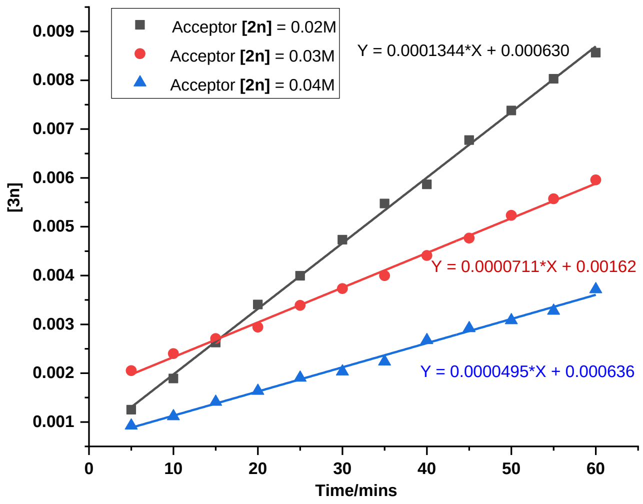

Supplementary Figure S43. Initial rate of product **3n** under different concentration of acceptor **[2n]**.

Table S27. Determination of order with respect to acceptor **2n**.

Entry	Concentration of **[2n]**	**[2n]**$^{-2.0}$	Reaction rate
1	0.02M	2500	0.000134 mmol/min
2	0.03M	1111.111111	0.0000711 mmol/min
3	0.04M	625	0.0000495 mmol/min

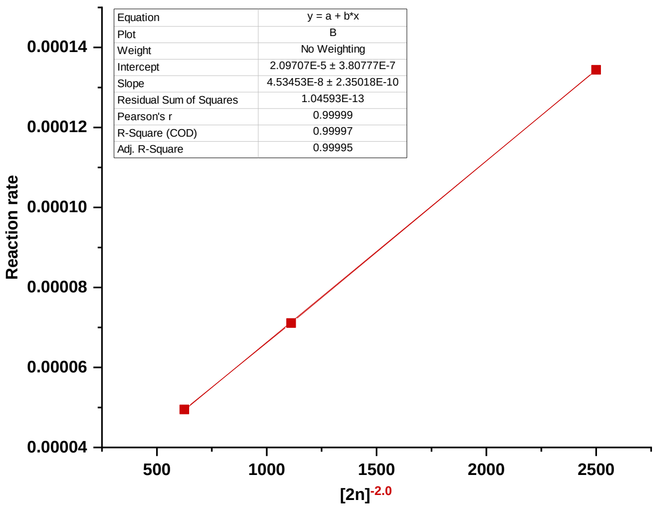

Supplementary Figure S44. Determination of order w.r.t **2n** using initial rate method

7.13. Experiments to test stability of catalyst J in the presence of deprotonated alcohol

Procedure: To a dry NMR tube, glycosyl acceptor **2n** (0.88 mg, 0.01 mmol), base K_2CO_3 (1.38 mg, 0.01 mmol) and 0.5 ml CD_2Cl_2 were added sequently, then catalyst **J** (24.85 mg, 0.00125 mmol) was added later. At the same time, To an another dry NMR tube, the same amount of acceptor **2n** , catalyst **J** and CD_2Cl_2 were added. Afterwards, the two tubes were sealed with cap and measured on the NMR spectrometer at room temperature by recording ^1H, ^{13}C and ^{77}Se spectra at 1 hour and 24 hours respectively.

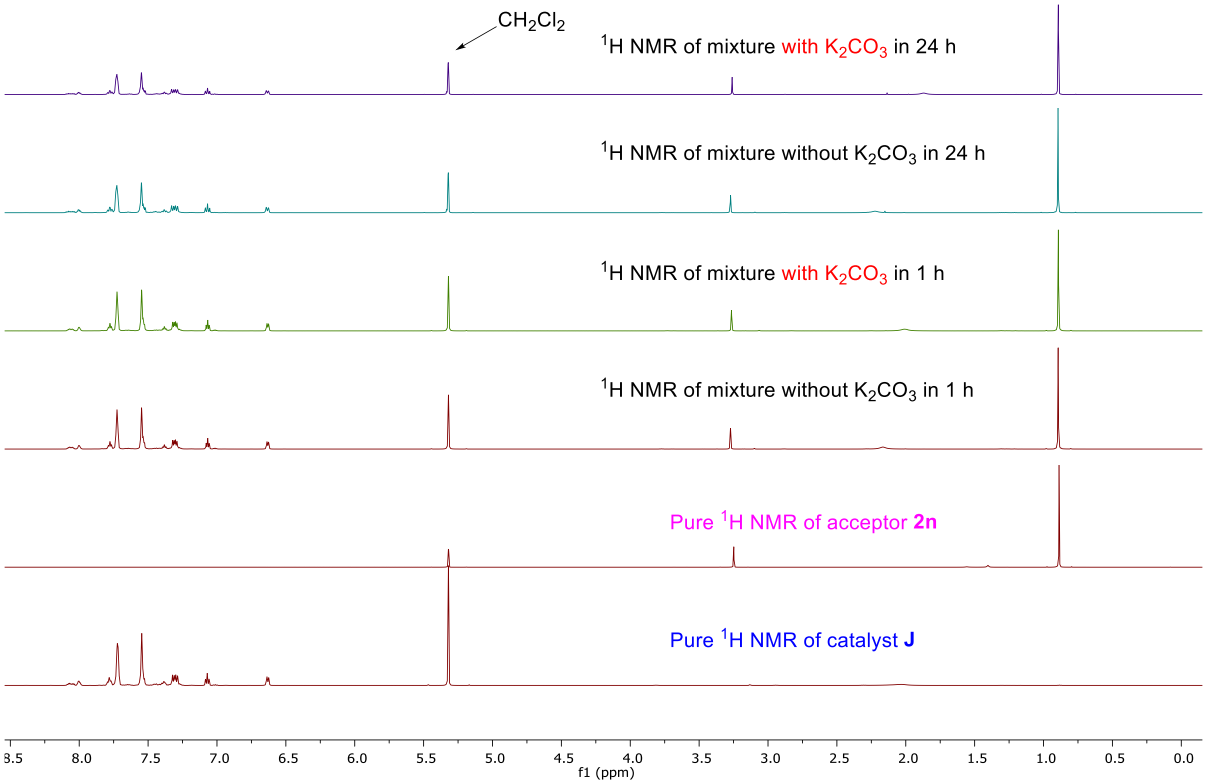

Supplementary Figure S45. ^1H NMR of the comparative experiment to test stability of catalyst

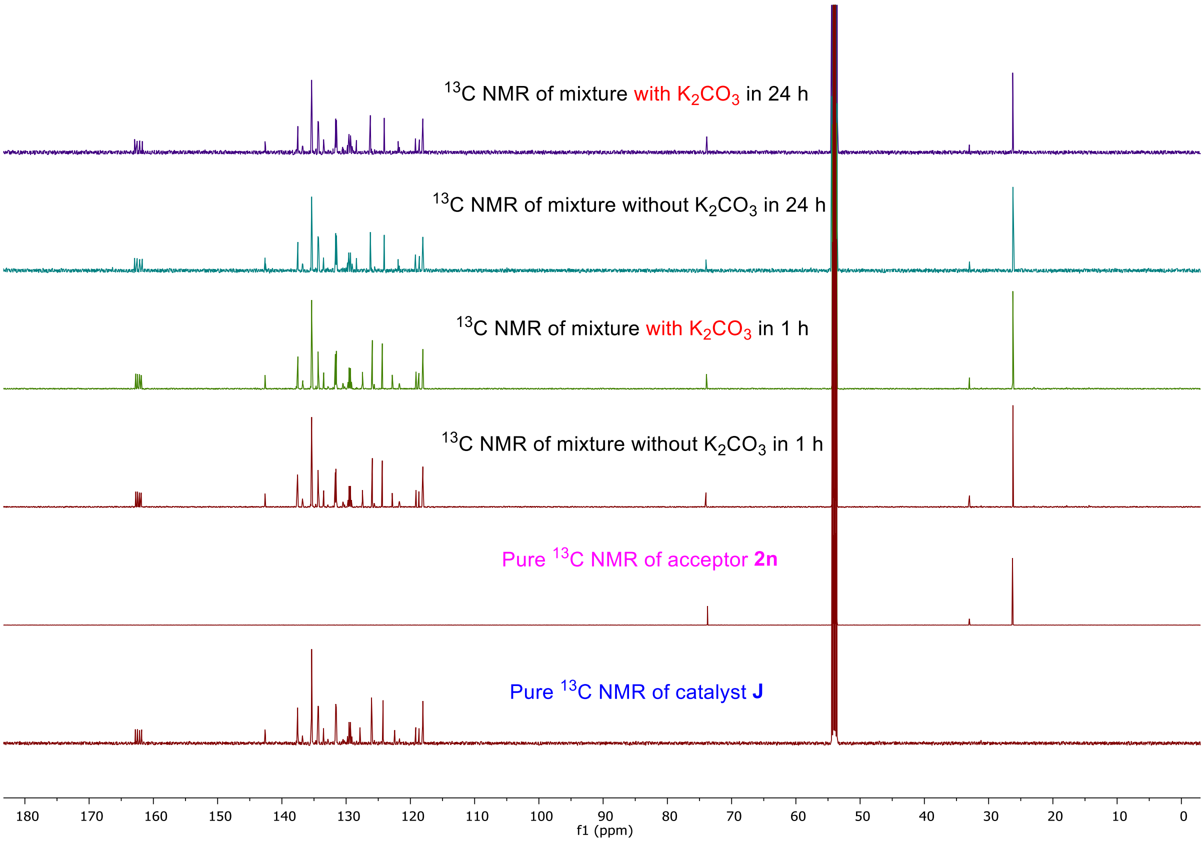

Supplementary Figure S46. ^{13}C NMR of the comparative experiment to test stability of catalyst

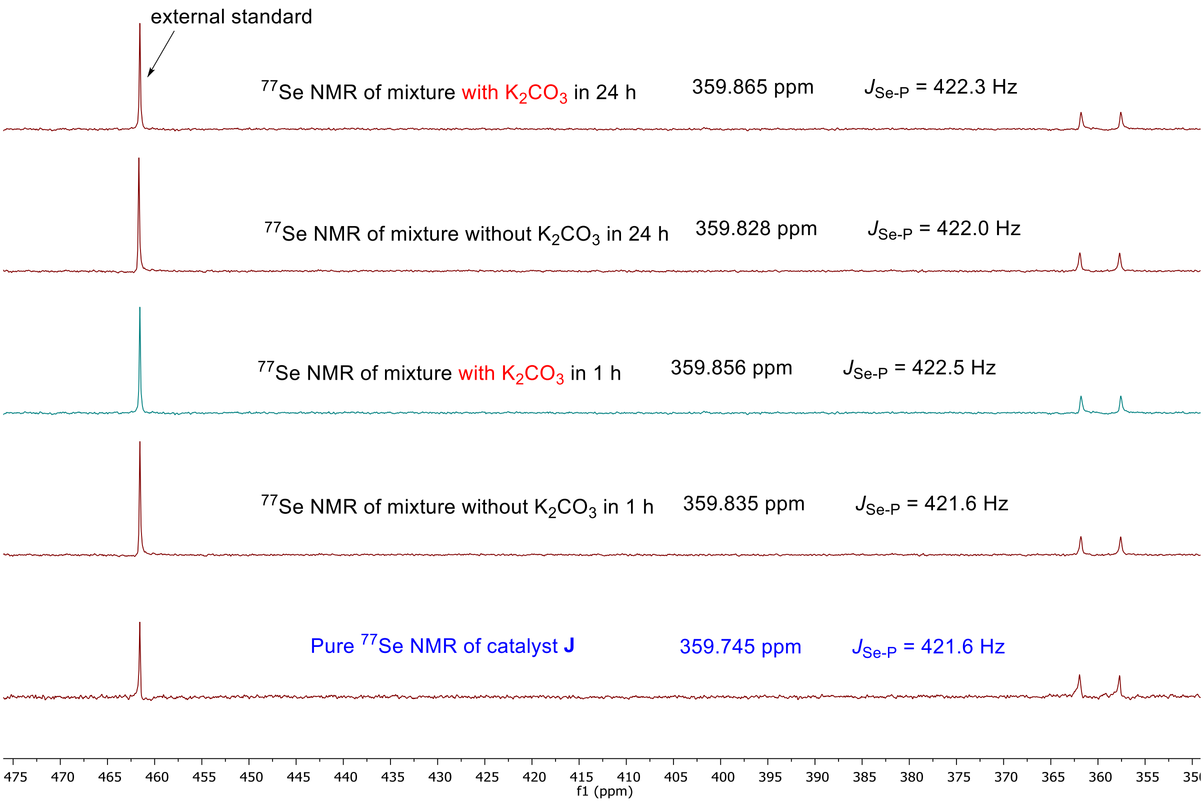

Supplementary Figure S47. ^{77}Se NMR of the comparative experiment to test stability of catalyst

7.14. Computational details

All quantum chemical calculations were performed with the developmental version of the Orca 5.0.4 program package,[18-19] and its interface to the open sourced stand-alone xTB code.[20] All molecular geometries were preliminarily optimized using the Grimme's semi-empirical GFN2-xTB hamilitonian[21] with the analytical linearized Poisson-Boltzmann (ALPB) implicit solvation model for CH_2Cl_2.[22]

Plausible geometries were then further optimized (utilizing TIGHTSCF settings) at a higher DFT level using the meta-hybrid M06-2X functional[23] developed by Trular et al. with tight integration grids (DEFGRID3 setting in Orca), corrected using using the D3(0) dispersion model (the zero-damping variant recovers the long-range contribution, which is deficient in M06-2X),[24-25] and using the def2-SVP basis set.[26] The M06-2X functional has been demonstrated in benchmark studies[27-28] to be well suited in describing chalcogen bonding and non-covalent interactions in general.

The default Gaussian charge scheme with a scaled vdW-type cavity of the conductor-like polarizable continuum (CPCM) solvation model for CH_2Cl_2 as implemented in Orca was applied.[29-30] The resolution-of-identity approximation[31] for Coulomb integrals and COSX numerical integration for HF exchange (RIJCOSX) was applied along with the appropriate corresponding auxiliary basis sets.[32-33] Harmonic frequencies calculations at 298.15 K were computed at the same level of theory and used to verify the nature of the optimized stationary point as minima (no imaginary frequencies, threshold set at $i\omega < 10$ cm^{-1}) based on the modified ideal gas-rigid rotor-harmonic oscillator (RRHO) model.[34] Spectator counteranions were excluded in the computations.

Yang's noncovalent interaction analysis (NCI analysis) based on reduced density gradient (RDG) isosurfaces and colored by the mapped function $sign(\lambda_2)\rho$,[35] were performed to unravel relevant noncovalent interactions between catalyst **J** and the interacting substrates/intermediates, using algorithms implemented by Lu et al. in Multiwfn 3.8[36] on the ORCA computed wavefunctions of the respective geometries.

RDG isosurfaces from the NCI analysis were then rendered using VMD 1.9.4a51.

CYLView[37] rendering of intermediates 6, 7, 8 and 9

The cartesian coordinates (in Å) of the optimized stationary points (minima) for plausible intermediates at the M06-2X-D3(0)/def2-SVP/CPCM(CH_2Cl_2) level of theory are provided. The geometries were rendered using CYLView20. (Red atoms = oxygen, grey atoms = carbon, yellow atoms = phosphorus, purple atoms = selenium, light orange atoms = silicon, white atoms = hydrogen). The relevant NCIs are displayed using dotted lines, and the relevant distances (in in Å) are shown alongside.

Complex 6

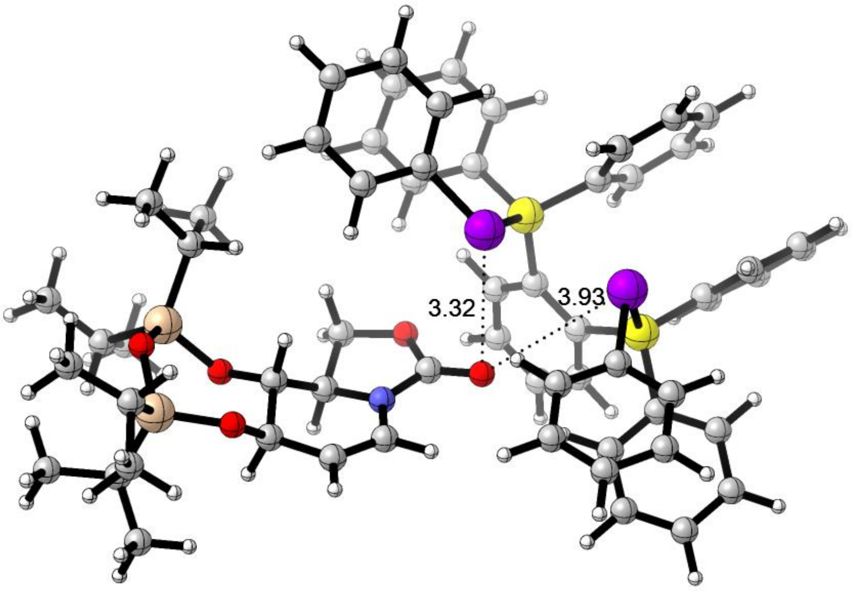

142

C	-4.03697165472150	0.12118678608613	0.68849507942303
C	-4.75090670654686	1.43399511107776	0.32739778194276
C	-2.84168339405592	0.41230322792714	1.57640613499296
H	-3.67234467146060	-0.32388458476322	-0.25850783534217
O	-4.88428542284102	-0.75203782271513	1.38762201865581
C	-3.75913838963784	2.50871827173858	-0.05642206163798
H	-5.31215425081655	1.75021464537110	1.22883518734344
O	-5.63846624021324	1.24429273936313	-0.75164929883732
N	-1.94431832611993	1.33904317686953	0.91646602804336
C	-1.89577823829946	-0.76575774693351	1.81883524558859
H	-3.20435034695476	0.84860399185655	2.52371447415048
C	-2.44992609777977	2.42794400668986	0.20905458164586
Si	-5.78942104887951	-1.98892140984465	0.67564541225455
Si	-7.07127055602234	0.36176305639346	-0.71364102863957
O	-6.68746479582301	-1.26048054867171	-0.52731504981935
C	-7.82135179090080	0.55789697219711	-2.41381533111309
C	-8.07533400338516	0.89798834632243	0.78243352194337
C	-6.89939736330012	-2.60747094517984	2.04995046103896
C	-4.64733419928651	-3.22708077248266	-0.15181523974854
C	-9.43419405324691	0.18669379875405	0.82234283387675
H	-9.33163966999700	-0.90517688063345	0.71877491584861
H	-10.08382466327738	0.54024933608251	0.00561778048060
H	-9.95715032882861	0.38867373796757	1.77038757423526
C	-8.22843714141207	2.41814937067568	0.91303958102072
H	-8.85256598985737	2.82606425601303	0.10420201359666
H	-7.25873584573951	2.93741155420287	0.87646865555806
H	-8.71474668660302	2.67861657902155	1.86643889789549
C	-8.79656401896881	-0.57350337800060	-2.76062328051530

C	-8.45774340814867	1.93685476771818	-2.62335821216336
H	-9.68462078780839	-0.54857825659074	-2.10983174392838
H	-8.32722202437416	-1.56125598257765	-2.64954333300584
H	-9.14813009052279	-0.47577680261459	-3.79976738980723
H	-7.77131494432365	2.75501629140905	-2.35629772786785
H	-9.36772199497824	2.04580221508387	-2.01211727556824
H	-8.75209419487611	2.07392948042971	-3.67557609061583
C	-5.37805447206785	-4.06324968334545	-1.21064914084266
C	-3.90622556621818	-4.11210364114198	0.85739271614181
H	-3.90304345404111	-2.59181193204803	-0.67076470653479
H	-4.68179949414707	-4.74501489454100	-1.72616486895388
H	-5.85766072463811	-3.42704724336185	-1.96773674876027
H	-6.15920380148241	-4.68903552010411	-0.75219771544570
H	-3.40962155783551	-3.52395696857929	1.64538191818454
H	-3.13604387948780	-4.71987569576825	0.35480364887341
H	-4.60184315331804	-4.80930321034693	1.35048119082580
C	-7.72620687217467	-3.82369131534624	1.61400148835921
H	-7.59477047437462	-1.76362573464421	2.21275775775918
C	-6.15593322675731	-2.86397788065585	3.36640590941915
H	-7.08455700350511	-4.70854734841517	1.47588365403186
H	-8.25931009757223	-3.64337557570543	0.66738558288934
H	-8.47623754485914	-4.07902685900543	2.37867008071388
H	-5.45616164068720	-3.70836554494968	3.27361737468781
H	-6.86831598447156	-3.11457759391132	4.16811457361015
H	-5.58130299757383	-1.98227458162182	3.68466640114220
H	-7.47724597578753	0.54338884604931	1.64413139081753
H	-6.94691354084103	0.47615060667717	-3.08518850950008
H	-1.71377452222060	3.17604428422084	-0.08942897908526
H	-4.15094286977978	3.37378169925161	-0.59251905126123
H	-2.00874455783424	-1.53632602667839	1.03891126367167
H	-2.00178242215220	-1.22075523498838	2.80877290065846
O	-0.58296805995791	-0.19196994765518	1.71525491155424
C	-0.63604464583329	1.00579571057978	1.09354072334364
O	0.34045201384185	1.63835063335461	0.77372903730332
H	8.04808550060604	-1.54839960845096	-1.64652856791912
H	6.21539189461680	-1.33434368952351	-3.31666734861290
C	7.01208999937014	-1.47426519477391	-1.31146231495931
H	9.58895283920709	1.30022088032976	1.08688056939392
C	5.98641325398691	-1.35255530754504	-2.25064212004571
C	8.51424155611461	1.44549129734373	0.96646420457526
H	8.06809031294364	1.01546322233267	3.03887883125743
C	6.72215216396329	-1.50104866451312	0.05411462811078
H	7.52456889734417	-1.59947811801544	0.78638581825306
C	4.66066416136351	-1.26243447776038	-1.82945933657527
C	7.66339789098544	1.28487188147197	2.06254577649969
H	8.65692617153989	1.91090964745865	-1.13884613043993
C	7.99423037919083	1.78725179516362	-0.28132069461807
H	3.85422309175862	-1.18081713601601	-2.56253901793420
C	5.40405834866352	-1.38975122956832	0.48792714455319
C	4.37393111853591	-1.26807182298015	-0.45872458483675
C	6.29085356937197	1.46388264023687	1.91498221382273
H	4.20971671910281	-3.79000239991703	0.09458588458843
C	6.62105378485195	1.97206572586796	-0.44287001246973
H	2.28977531361570	-3.32965299920530	-2.46950423090437
H	5.18324845373987	-1.40327666048591	1.55877535426720
H	5.63587030419720	1.32560767331069	2.77704281113914
C	5.77125464753638	1.80801076263745	0.65748407122523
H	3.53788708208418	-6.13211476609644	0.50304072913942
C	3.19374849477209	-4.00860863708470	0.42645302050046
H	5.72408547327523	4.47335719678427	0.64531072309405

H	6.22253998913472	2.24328990108515	-1.42187649828017
Se	1.16599874462376	-0.70356682214971	-1.43059159768658
P	2.67958239775272	-1.28569284452151	0.16691768599949
C	1.21755195099728	-3.47114747705199	-2.32018253890579
C	2.81111194888437	-5.33235001080992	0.64935286163259
H	1.24728418876986	-5.52532758584359	-2.96223788292766
H	5.19944528496248	6.88313679281371	0.90396247282454
C	2.25920064515432	-2.98496448783640	0.61520426043569
C	0.62896017049159	-4.70286378954686	-2.59924390718945
C	4.69099949329784	4.79310175567016	0.79565123691552
C	0.42458115278183	-2.43003123291637	-1.82780209845092
C	4.39384735772157	6.14887608586425	0.93847594113880
H	2.80947738543538	6.67049095483531	-2.75845991817919
C	2.64611420796559	-0.21791166290527	1.65905472759216
P	3.99683742778638	2.12867015019070	0.50457071138868
C	2.30215933747390	5.73764157548305	-2.50765211352279
H	0.32444035546813	6.59531645493307	-2.61672089530560
H	1.76382585983742	-1.84904007612030	2.77082010867307
C	0.90818366059889	5.69295315975954	-2.42778600352891
C	1.50774938694110	-5.62967069231512	1.04279714034402
C	2.11938763029914	-0.82203624585353	2.80430141315853
C	3.12227734457043	1.11612659276108	1.75710486308821
C	3.65044319666871	3.86009874835768	0.85010256620923
C	3.05184448335147	4.58656381733315	-2.27245262890042
H	4.14126501149819	4.61045174869991	-2.33751955523914
C	0.25762172143755	4.49808980652270	-2.11609611850728
C	-0.74716504097577	-4.87729532775888	-2.43158132575755
C	0.94539147229853	-3.28283532375555	1.01317507654004
H	1.21229781271717	-6.66758311954357	1.20419650915030
C	-0.95383801972813	-2.59235553660761	-1.66398929909511
C	2.38915008354326	3.40170640036314	-1.93781007106223
H	-0.83204028693617	4.46416835020060	-2.06685341657090
C	2.03056408207043	-0.14532346812228	4.02186387435703
C	3.07409541287013	6.55972226547073	1.12357371486340
C	0.99571722872078	3.34109339364625	-1.86285243014806
C	3.02980127114289	1.77718770767994	2.98728025117188
C	0.57467057206935	-4.60436072453824	1.22866335668164
H	-1.20646109993620	-5.83993254549459	-2.66132523612439
C	2.48268029676045	1.15957649371900	4.11391789940134
C	-1.53792772369859	-3.81877650785721	-1.98389216930194
Se	3.42558153354298	1.81228624738107	-1.65320190158817
H	1.60891663898107	-0.65588498355362	4.88812114895902
H	2.84751958552774	7.62181160513144	1.23052387849112
H	0.21468588172726	-2.48015257230675	1.13511152438490
H	3.39848172041631	2.79807517792089	3.09073077210345
H	0.49625963771235	2.41194544505303	-1.58401968630788
H	-1.56702113050763	-1.76781566731367	-1.29172637117219
C	2.31955244526734	4.26466913634644	1.03914340579639
C	2.03973637325312	5.62022824893145	1.17732008810073
H	2.42736383232333	1.70968515707635	5.05373984714209
H	-0.44707690051902	-4.83549249175678	1.53344305418974
H	-2.61519656547302	-3.94798453210941	-1.87219424497735
H	1.51533348569596	3.52440217862351	1.06834601814292
H	1.00872395327631	5.94538874727167	1.32360668741985

Complex 7

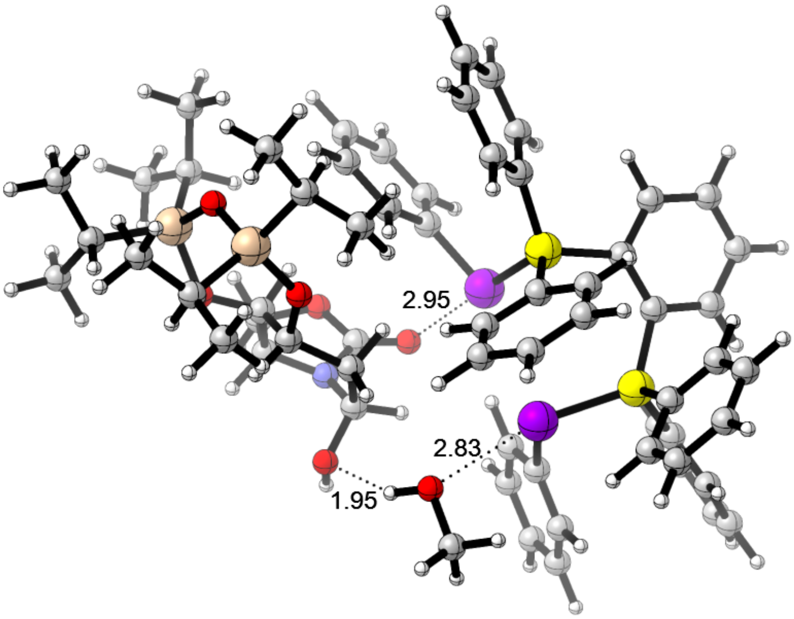

151

H	6.86703103100661	-0.20121478960166	-4.19781251158549
H	4.87752264617870	0.92845691088772	-5.18637401361308
C	5.87581768887105	-0.37647974080166	-3.77645677341527
H	8.78055700076771	-0.19499022668374	0.11430617315256
C	4.76368457900884	0.26013716321831	-4.33141773877580
C	7.72435503725300	0.07756314770288	0.14282775215305
H	7.14609220427900	-1.66367800354758	1.28662734917984
C	5.72787302855653	-1.24069962994687	-2.68926208279168
H	6.59662787014927	-1.74453312166961	-2.26309649026122
C	3.49702511035230	0.04056063063157	-3.79489278058662
C	6.80994933029892	-0.74741847670462	0.79982134566564
H	8.00264881976244	1.89677155056331	-0.99093283498215
C	7.29041600275315	1.24887110765238	-0.47862009112971
H	2.62467881006381	0.53416961100488	-4.22648874646345
C	4.46751223398195	-1.45596941704319	-2.13579795234907
C	3.35540039506381	-0.80040414383493	-2.68543711671253
C	5.45927507128994	-0.40870653995767	0.83472442306344
H	2.86311372666437	-2.85240807198693	-4.10022066887589
C	5.94102595938776	1.59727200627863	-0.45379183081688
H	0.52758262438602	-0.83602990907697	-5.17249437671100
H	4.35501438994312	-2.13304488323506	-1.28485876903609
H	4.75783536161732	-1.07127498681883	1.34405796929058
C	5.02264897757758	0.76492952468948	0.20212942813115
H	1.93748447952869	-4.75404270865485	-5.37466116829342
C	1.89097428689838	-3.26287473672884	-3.82260566730371
H	5.06578221138307	2.83207640027618	1.93471380987086
H	5.61052371468924	2.51242257408388	-0.94804313491347
Se	0.15323265970933	0.39005923032161	-2.41326006726796
P	1.72919580547341	-1.18792353784302	-2.00755169273902
C	-0.50370941655519	-1.02561070081367	-4.86234120616143
C	1.36516580891472	-4.33377455039297	-4.54625503275380
H	-1.01423621741317	-2.03684335023835	-6.69467309739216
H	4.59699233572733	4.58584586899191	3.62478075388651
C	1.15191118231771	-2.71893477988874	-2.76455650247581
C	-1.37057433551323	-1.70704658460594	-5.71569662447869
C	4.03402999554070	3.07333963116375	2.19803719270695
C	-0.97724619592915	-0.58830868689702	-3.62099822033195

C	3.76827623020650	4.06186785376271	3.14721670359813
H	2.62243549786180	6.87123654846281	0.55547162059821
C	1.89072460724866	-1.33448659698071	-0.19114340632155
P	3.27078350129376	1.23403329969606	0.26043363931294
C	2.04533916853241	6.05547702610120	0.11712378358672
H	0.13895630894505	7.01283206193728	0.44770663868357
H	1.14965974274470	-3.36321738886378	-0.28790194168836
C	0.65158651524600	6.13309040185018	0.05529150635843
C	0.11496214500438	-4.85447911214076	-4.21659876784570
C	1.46391427022827	-2.54682237728758	0.36064263896160
C	2.34268534216361	-0.30397610056261	0.67583288707516
C	2.96542834014421	2.40258350634399	1.59697939965446
C	2.70587702027441	4.93487074726621	-0.38402706395666
H	3.79481172374763	4.86774525051202	-0.34128588034602
C	-0.08617701635799	5.09347990417004	-0.51231659604754
C	-2.69688491498597	-1.93127810331166	-5.33843642900380
C	-0.11044197759062	-3.24028286253303	-2.43342936165839
H	-0.29589732761417	-5.68647112043066	-4.79071371878281
C	-2.30602571566259	-0.79295746213604	-3.24093524425533
C	1.95552562958421	3.89023731771290	-0.93167391762522
H	-1.17392176210770	5.15632301659676	-0.57167202145362
C	1.43526979842303	-2.75099607099816	1.74155477946928
C	2.45160298510465	4.37642602819883	3.48023413568341
C	0.56225699771607	3.96086954603822	-1.00754483522712
C	2.29008936289212	-0.52559375893221	2.05925296505130
C	-0.61858560642338	-4.31376228477064	-3.15554092774625
H	-3.37004752562035	-2.46209113024904	-6.01424589798339
C	1.83013568214968	-1.73135514705697	2.59274562457550
C	-3.16701247352345	-1.46265613504019	-4.11079324832858
Se	2.86941490007278	2.37109940308040	-1.66566278596826
H	1.09959311546727	-3.71089049434750	2.13510206802822
H	2.24996496555758	5.15319158251419	4.21957483896396
H	-0.70589399594003	-2.79619567565342	-1.63186958534845
H	2.63902512306200	0.24153781486213	2.75138965846102
H	-0.02269028874316	3.15426381783374	-1.45397834213765
H	-2.66336618073952	-0.43600805461023	-2.27355646957629
C	1.63777327173812	2.71056593235405	1.93195000828213
C	1.38683964501790	3.69923418068536	2.87677458099777
H	1.80559560336735	-1.86324756878667	3.67484121086117
H	-1.59876099130721	-4.71879581909246	-2.90071794237218
H	-4.20593302254549	-1.62619226447552	-3.82100174538692
H	0.80594401731020	2.18622853044193	1.45400091576586
H	0.35754703503212	3.94577410784337	3.14085135811289
H	4.79218916421361	4.45651233499902	-2.85237235363100
C	4.12845254125546	4.97067253180234	-3.56353127928989
H	4.73119843772707	5.27450117285695	-4.43504339687041
H	3.73935196969261	5.87783508851228	-3.07136079836110
O	3.10120743419461	4.07395136292771	-3.91842064128308
H	2.48147052695730	4.51296049706176	-4.52507021815836
O	-1.77234513414474	2.54622644061717	-3.01653942734351
O	-3.12710483805633	2.40961036241230	-4.80203121661176
C	-1.97824114866810	2.74248641814409	-4.19220711435500
C	-3.10413187013514	2.84985523463021	-6.16812415325811
H	-3.67879970251831	3.78198624482305	-6.24737069683348
N	-1.13023343371748	3.29700257222837	-5.11095907952959
H	0.44619036082501	3.41561572582267	-3.79423797189118
H	-3.55609878944267	2.07337667994278	-6.79646517476158
C	0.29314206068320	3.41924969572485	-4.88638355441720
C	-1.61682337144878	3.06009851689964	-6.45998237359432
H	0.80407409768995	1.36738905128528	-4.88609546361821

C	1.01903503987991	2.24152347895912	-5.51332622351155
H	-1.29744247390928	0.93785492653069	-6.49166729213617
C	-0.94779574234573	1.82250832730584	-7.06216161941359
H	2.10337791127063	2.41912764242815	-5.48644152930914
H	-4.49495982400224	1.37434190407608	-8.85973384099943
H	-5.42814947312658	-0.09535172270705	-8.49303785717682
C	0.57984629005046	1.87249413341073	-6.92896000657123
C	-4.54481115136204	0.28716821324049	-9.02779795040501
H	2.86825340793635	-1.17385371139603	-6.15418739450129
H	-3.18693031743433	-0.31100388953008	-7.46857179682354
O	-1.32924058922621	1.74828955773598	-8.41220169578955
O	1.14339336948136	0.60335258469381	-7.17611185732524
C	-3.27582649808254	-0.43970859535865	-8.56400522770353
H	3.48037857758381	-2.77225613509223	-6.65336826649078
C	3.10933538206041	-1.80204975779416	-7.02375585981410
H	0.94937197846987	2.61547481702656	-7.65979664065330
H	-4.71705156801424	0.12656703214333	-10.10368395605852
H	1.08312735330820	-2.44269037536983	-7.31088673830814
H	-4.25416082541807	-2.38129089205711	-8.38636158739012
Si	-1.71208002248421	0.33658325272045	-9.25145868551396
C	1.88790837280811	-1.99825055331380	-7.92679583846975
C	-3.35903033146919	-1.94299861790875	-8.85669105795485
H	3.93885533510759	-1.31834435965122	-7.56349333032019
Si	1.13349307642359	-0.38998392102674	-8.52766819056513
H	-2.27753490688829	2.94167788516910	-10.66162883317616
O	-0.45038993056404	-0.72445067290728	-8.96072326810071
H	3.46308213498780	1.44416027746119	-8.62368796190845
H	-2.47540566006374	-2.48312474296831	-8.48615342573125
H	-3.43246356494276	-2.13271579237256	-9.93919269074287
H	2.45121317675534	-3.95934016801156	-8.69913828706450
C	2.18670117784077	-2.96120350580334	-9.08211872999576
C	-2.56375645850919	2.11298538793227	-11.32523066822409
C	-1.72844719162636	0.85670264266551	-11.04991086530222
C	3.40211764835045	0.92511636163439	-9.59269784209868
H	-3.63771608170294	1.92138101952597	-11.18385778392965
C	1.96106513508840	0.53116806560778	-9.93815942771189
H	4.04854326164667	0.03449402231919	-9.53887527783582
H	1.36142950837917	1.46052178546885	-10.00876759450926
H	1.32659908041400	-3.07740893695626	-9.75951718349307
H	3.04018772179484	-2.60182627843168	-9.67820904864786
H	-2.42415360613336	2.44603268643188	-12.36574592963807
H	3.82432125575187	1.59084849353034	-10.36169977352981
C	-2.12024845524059	-0.30480733230396	-11.97182908032829
H	-1.52475119166436	-1.21016217570819	-11.77403832025217
C	1.87671394726211	-0.19060086261196	-11.28841660450912
H	-3.18215336445111	-0.56893115946603	-11.84317799337088
H	0.85543038839125	-0.53507357611727	-11.51102643521678
H	2.53514743871094	-1.07178525396961	-11.30620698158529
H	-1.97587983016659	-0.03152187140235	-13.02882840106565
H	2.19487808148755	0.47398566342609	-12.10719348068363
H	-1.43606094427878	3.92664872624147	-7.11099695872736
H	-0.67112039443122	1.11117438013146	-11.24741055641439
H	0.29166058919652	5.35537348934808	-5.08932447765432
O	0.77825149103167	4.60499204188169	-5.46341059944000

Intermediate 8

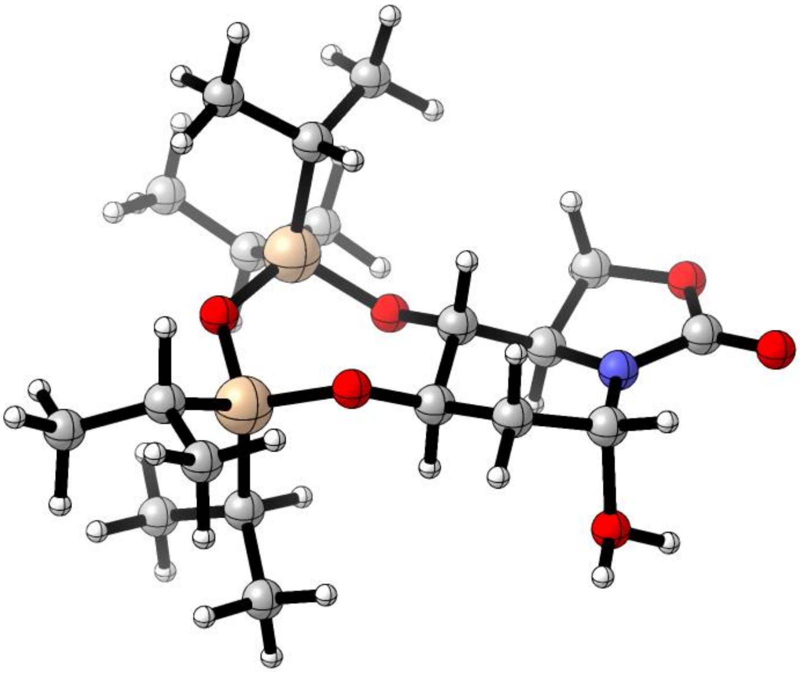

66

H	1.80770281693436	4.00018694251423	-5.43331977569573
O	-1.90684649455654	3.79275239737427	-3.28393427055007
O	-3.39582429632206	3.54219793201534	-4.95400359056694
C	-2.18170906479923	3.49055080355509	-4.40776119929546
C	-3.40269952730197	2.94737346720760	-6.26354742960116
H	-4.03572712241795	3.55468422375267	-6.91667651659383
N	-1.29347398824537	3.02706716802770	-5.37614046239935
H	0.24110365943675	2.68462717972755	-4.06386925054235
H	-3.80876351568401	1.92928347248818	-6.17830214306363
C	0.04097255659010	2.77466581406518	-5.13750226338090
C	-1.92922633978698	2.93054203815945	-6.68532604251811
H	0.31447144337319	0.72381643178186	-5.51775200308157
C	0.61341855805021	1.67060369745912	-5.99328688286333
H	-1.79051255161083	0.78849522454282	-6.86455497950329
C	-1.45274999900753	1.68538564164433	-7.41869574926416
H	1.71203116707559	1.70205539358911	-5.98848687893014
H	-4.49992962257170	0.21778180944793	-7.80410631881661
H	-4.76685765467645	-1.53612401214189	-7.65987347786133
C	0.08251178311145	1.68983172013997	-7.42750948565166
C	-4.41349370856134	-0.74243574437391	-8.33643855330754
H	2.81478820178381	-0.93018355326957	-7.97392977325633
H	-2.33146766043436	-1.10661749802521	-7.91081203833942
O	-1.97992602827936	1.72255965244914	-8.71255069108583
O	0.58844884091727	0.56525506900260	-8.08687414645620
C	-2.97980299230979	-1.01875882438959	-8.80262828857192
H	3.70558655514954	-1.97007383590918	-9.11422063864843
C	3.10805697275409	-1.04907580581922	-9.02648830015117
H	0.40777977604455	2.62152092266412	-7.92841702857769
H	-5.10311684542679	-0.71111530180096	-9.19481761055125
H	1.25687301613081	-1.96393824834118	-9.63231105707373
H	-3.21804564752533	-3.18095388537377	-8.94186018679730
Si	-2.25023268016048	0.42000740433376	-9.76058024493702
C	1.88550826596424	-1.11151593395161	-9.94923483593340
C	-2.87697249393908	-2.34316618258274	-9.57010103255478
H	3.76495558779364	-0.20577749314897	-9.29096184864270

Si 0.74432967149256 0.35666323905773 -9.75761124906850
H -4.09163014382282 2.80330140632476 -10.00031568496679
O -0.76187317994178 -0.06008955141250 -10.35087542200606
H 2.74101528981296 2.45846605766332 -9.00223016022234
H -1.84334070474379 -2.54984400918045 -9.88285032912328
H -3.50634473898211 -2.33196895724345 -10.47305258242344
H 2.82688386915961 -2.28986968086200 -11.52587532887118
C 2.29268456851256 -1.33395660836111 -11.41122595961731
C -4.41487441763003 2.02284645126500 -10.70416891068480
C -3.23671085060175 1.15834651392216 -11.16663444066685
C 2.62812910698599 2.45819070322411 -10.09841344615488
H -5.18547535614388 1.41563314129223 -10.20500533384413
C 1.23444327367951 1.99410981112674 -10.53544719355554
H 3.40613500262680 1.79722916383274 -10.51242212249831
H 0.48607424856201 2.70563453734605 -10.13822203237157
H 1.42350519836338 -1.35114054462365 -12.08671442334846
H 2.97074839232026 -0.53600256976304 -11.75342542696525
H -4.89314010670943 2.51629799505071 -11.56484218478292
H 2.83618058811311 3.47759776859957 -10.45952012210535
C -3.67532507671027 0.08787274968886 -12.17367535353671
H -2.83394630218329 -0.54402238993847 -12.49772470691838
C 1.08489004045753 1.98874591604692 -12.06238818082153
H -4.44303944421440 -0.57032192955541 -11.73646138169442
H 0.08317221775063 1.65291659101852 -12.36892706169695
H 1.82215736073298 1.32250185346835 -12.53498040625273
H -4.11302997237225 0.55188510206192 -13.07111244361179
H 1.24502999000518 2.99900095398187 -12.47067502046914
H -1.67851845618443 3.81262018695895 -7.29822443984795
H -2.50149553719943 1.81796603264854 -11.66403534936022
H 0.53883795301766 4.87387205951388 -5.06428006682920
O 0.83697654835455 4.07655992003289 -5.55322824061926

Complex 9

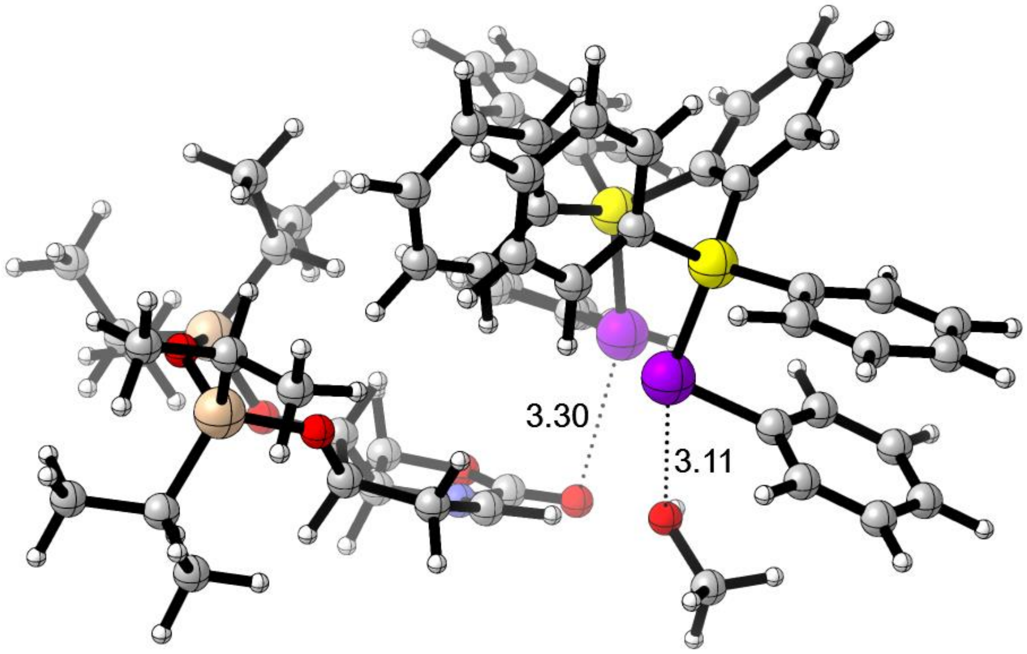

149
O -1.20780693753981 7.84083103355985 -5.25331046219970
O -2.78644552817104 6.53268153197517 -6.18372260937506

C	-1.56924770125156	6.90027677794557	-5.87916654466955
C	-2.86110912433996	5.23221257587296	-6.80951863036572
H	-3.52809080329023	5.31307325310452	-7.67180977722827
N	-0.66894031191541	5.88862853423818	-6.42383705125616
H	1.00983739662290	6.60731300083175	-5.49494638253605
H	-3.26849055269295	4.52956185897424	-6.07245389223633
C	0.58035749009476	5.79614100627589	-6.11638248332944
C	-1.41696048234531	4.89656370049355	-7.19693177436377
H	1.89498457262193	4.27524667027452	-5.62855310774352
C	1.37961710784038	4.63446928883693	-6.53856138295855
H	-0.96464627516635	3.44906715885462	-5.69502184575628
C	-0.88626087080991	3.52444882631344	-6.79869057467930
H	2.19738800280896	5.00817064806096	-7.18070661269617
H	-3.73756972022892	2.37380029040724	-5.19172371803927
H	-3.54216252853236	1.64967695052199	-3.57520467867992
C	0.60115629417009	3.47283892255927	-7.18432868056940
C	-3.47061448489190	1.44807031762379	-4.65685612315888
H	3.44742079114970	1.40209349435169	-5.61205326494733
H	-1.33497792678545	1.69105191882849	-4.66306632879811
O	-1.62658241248619	2.53933182449097	-7.43653458260214
O	1.18600637861377	2.27953802522911	-6.75510500843871
C	-2.07043366643023	0.95479433307341	-5.03733682827387
H	4.29362945936959	-0.16536850563440	-5.53677609890432
C	3.64448648574842	0.46898520409441	-6.16141168149583
H	0.64919917120902	3.56328770665407	-8.28399577821023
H	-4.23524595032904	0.69505284265047	-4.90454310017941
H	1.80296360669733	-0.48434159944138	-5.57423336506110
H	-1.80937628846568	-0.30802607954999	-3.28017028061478
Si	-1.82084310885946	0.93554342053661	-6.89967750592263
C	2.34987971039412	-0.27290782970171	-6.51250149377085
C	-1.72827466613219	-0.38578644566846	-4.37691321621596
H	4.21324365434720	0.72903187486687	-7.06765741964322
Si	1.13317957357266	0.75438188804778	-7.49607340137092
H	-4.13501214316855	2.14620878611240	-8.41358924724184
O	-0.39604917068813	0.13605729989550	-7.23478551611456
H	3.03100572186283	2.41210020987824	-9.09200406919484
H	-0.70317685336734	-0.70296730180299	-4.62060165524840
H	-2.41399303411188	-1.18271430433904	-4.70404616850909
H	3.22569059341536	-2.27012999485176	-6.53604269869236
C	2.63702742921971	-1.61599683902134	-7.19740063921874
C	-4.40383743781209	1.12710163285450	-8.09955133348860
C	-3.16802912061071	0.22831711501794	-7.97844095105700
C	2.79297741445936	1.49813340619972	-9.65909909757066
H	-4.94316449488761	1.19824161824880	-7.14290685803795
C	1.38972451096187	0.97063116295796	-9.33770131448689
H	3.55902392467855	0.74440116833194	-9.41663326309018
H	0.65066375611274	1.74487656294181	-9.61842566966516
H	1.71277589401813	-2.15136923270490	-7.46379347851071
H	3.22079922489953	-1.46862721930409	-8.11967028706506
H	-5.10582259481453	0.71647791904627	-8.84178207125050
H	2.88870539435902	1.73191986617364	-10.73064759387681
C	-3.53479603111514	-1.19604291729873	-7.54115175474829
H	-2.64834384210934	-1.84393729672538	-7.46223099371788
C	1.04927354774650	-0.29231016902427	-10.13916460306889
H	-4.03745308059488	-1.18984386163326	-6.56076108867650
H	0.04216811890042	-0.66440735673839	-9.89992145723257
H	1.76566010149766	-1.10210447439319	-9.93507520558416
H	-4.22619342448340	-1.65857847770655	-8.26210932003219
H	1.08713449274984	-0.08400187968970	-11.21964651558440
H	-1.24062876117640	5.08180757576130	-8.26745978678431

H	-2.68612511609595	0.17023345882816	-8.97181632986243
H	5.05285804192758	1.14279466054232	-3.21334120972193
H	5.32664680577415	-0.39431248807793	-1.27596605251477
C	4.75628466175846	1.46152720167776	-2.21314305296047
C	4.90664450595923	0.60164806199632	-1.12694335233410
H	8.08825643349248	5.81738074028315	-0.04146386125740
H	1.91117593502250	-0.23807752964852	-3.05831811334748
H	4.12167410722698	3.40884250891897	-2.88570733286481
C	4.22375396989987	2.73881831303997	-2.02968624093546
H	6.14297185572108	4.43860097377008	-0.70277132211462
H	3.47731183984146	8.40303252003122	-4.97357831713629
H	5.28650365076673	7.25417801929401	-2.72561044976441
H	6.07268556243117	9.54236641793698	-2.13792504075731
C	4.52096801275731	1.00927320501687	0.15310191177249
C	7.08312961875642	6.13758907087453	0.23507422319081
H	1.31676620537057	1.64691210491970	-4.59861920455391
C	1.51141157136367	0.69506664930437	-2.65552987462866
C	5.98720345533920	5.35858351700211	-0.13562128849632
H	4.63684035188859	0.33601453453251	1.00329257466738
C	1.18668041096248	1.74184307591981	-3.51961883579140
C	4.62009715810226	7.95291765593801	-2.21635746067133
C	2.56007638094338	8.93915911734508	-4.69805070363367
C	5.05688040252989	9.23558283450095	-1.88387451510177
C	3.84068493414715	3.14226209619587	-0.74627906828693
H	2.21915028283257	9.52670201825504	-5.56428599311495
H	1.58778119955451	0.01734142017020	-0.60278437429383
C	1.33288341982858	0.83549561724315	-1.27757699802661
C	3.98727883783067	2.27860643287676	0.35062142590278
C	6.89282468505155	7.31532874236321	0.95732723499297
H	7.75356175401423	7.92142413550761	1.24430337448965
H	2.79616379924793	9.62130694276769	-3.86501717276819
O	1.59832341215719	7.97218347599988	-4.31299654578841
Se	2.72244923943030	5.81474213714781	-2.36944831425908
C	4.69878633857162	5.76900128950034	0.22820337147550
C	0.66876691849793	2.93005925986591	-3.00885548340545
C	3.32085400367735	7.57290161492632	-1.87702659553904
P	3.29783938102537	4.83557953048064	-0.42533998876609
C	4.19714049790184	10.11999587869734	-1.23019985789334
H	3.68783560409556	2.58572933484752	1.35513777573158
C	0.83462084710559	2.02651969136586	-0.75553876424504
H	4.54277532551606	11.12186459770934	-0.97054253153783
H	0.38473697540654	3.74249616076624	-3.68131576514510
C	5.60530532174874	7.72097941256597	1.31894888554303
C	0.50040313894584	3.07457889816006	-1.62855873604245
H	0.75495807488648	8.41883224302663	-4.15077196934316
C	4.50367296889972	6.95441012468691	0.95370593373849
C	2.44508448533283	8.45041857794998	-1.23577653826604
C	2.89362559746761	9.73068503910337	-0.91116887032154
H	0.69827257778601	2.12863563244077	0.32482380076788
H	5.45634883987185	8.64163927509212	1.88467531582292
H	3.52928086061176	4.89636278103433	2.31735260218100
C	1.95919678290204	4.89461834802386	0.82892214043057
H	3.50104586465424	7.28491791103521	1.23105469988136
C	2.45417561391769	4.94123363285410	2.13907223910576
H	1.42872160027286	8.13759144261073	-0.98729048169336
H	-1.76014913507014	1.95777478113107	-1.21367253260809
P	-0.34255566014919	4.50741866619497	-0.93094683080685
H	2.22190367096187	10.42521753193662	-0.40461245651143
Se	-0.55361566490462	6.17681415542119	-2.47755219521203
C	0.54861707697051	4.91431153879985	0.63073247029176

H	-2.37739047808887	4.00266673916125	-3.57964249677048
C	-2.45747832477940	2.70568055568078	-0.83344862788695
C	1.61022724119847	5.05899620239294	3.24465341535926
C	-2.99815937897681	4.88194580283157	-3.37749377614584
C	-2.03956292129042	4.01055512173513	-0.54514463921961
H	2.03768974620556	5.09002673002125	4.24719631652106
C	-0.27501167914713	5.05406762336595	1.75319383743586
H	-4.12067012460075	1.34095286977873	-0.87453290531313
C	-2.43579184244631	6.04499362993968	-2.84051513872987
C	-3.79718280806789	2.35842334273553	-0.65100941038596
C	0.24291119787299	5.14615583633245	3.04679355686487
H	-4.82835233132343	3.93498011941369	-4.01007303270576
C	-4.37078660035126	4.84660183253091	-3.62198922237160
H	-1.35666617332404	5.06170795101523	1.64564960658165
C	-2.96487020923344	4.96787746699737	-0.09458522071053
C	-3.21546963837466	7.17918564708916	-2.59808250031612
H	-0.44052674151276	5.25746422941278	3.88898971981137
C	-4.71302847351961	3.30532093328285	-0.19777770366505
H	-2.65462368678523	5.99808335031816	0.09668543889111
H	-2.76293162178220	8.07917488536813	-2.17771254331143
C	-5.15819923217412	5.97549464199239	-3.38370552531075
C	-4.29589928285294	4.61028853456210	0.08277899744895
C	-4.57931416051183	7.14387196797263	-2.88778518495574
H	-5.76035312968173	3.02964960637820	-0.06507486928297
H	-6.22942407593400	5.94206507394338	-3.58761130303273
H	-5.01236710923827	5.35577980120738	0.43009633075291
H	-5.19244394455717	8.02779709409766	-2.70668415317295

NCI analysis[36] of complex 6

Red atoms = oxygen, cyan atoms = carbon, yellow atoms = phosphorus, purple atoms = selenium, brown atoms = silicon, white atoms = hydrogen *(Blue isosurface = strong attraction; Green isosurface = weak van der Waals interactions; Red isosurface = repulsion, steric effect in ring and cage)*

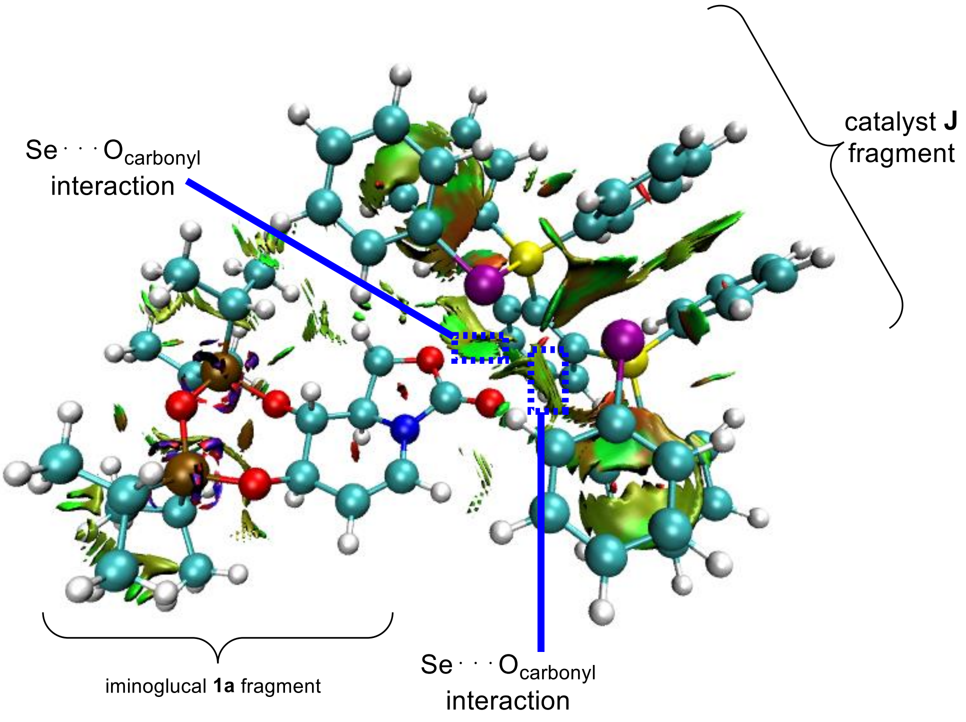

Supplementary Figure S48: NCI analysis of modelled complex **6**

NCI analysis[36] of complex 7

(Iminoglycal 1a, MeOH and the cationic component of catalyst J were modelled)

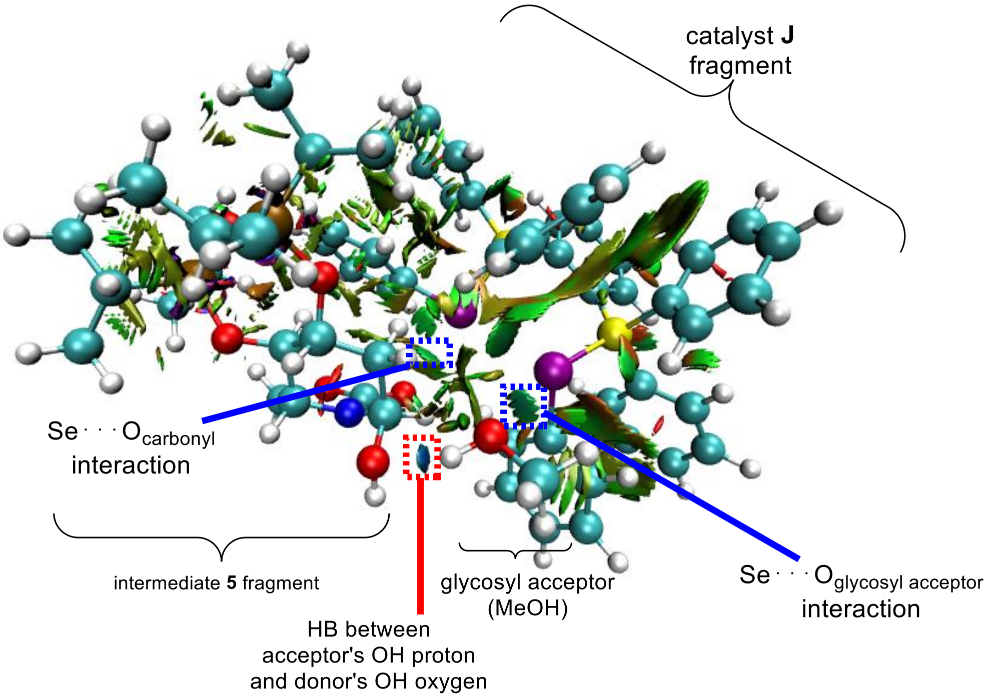

Supplementary Figure S49: NCI analysis of modelled complex **7**

NCI analysis[36] of complex 9 (Perspective viewed from the bottom α-face for clarity)

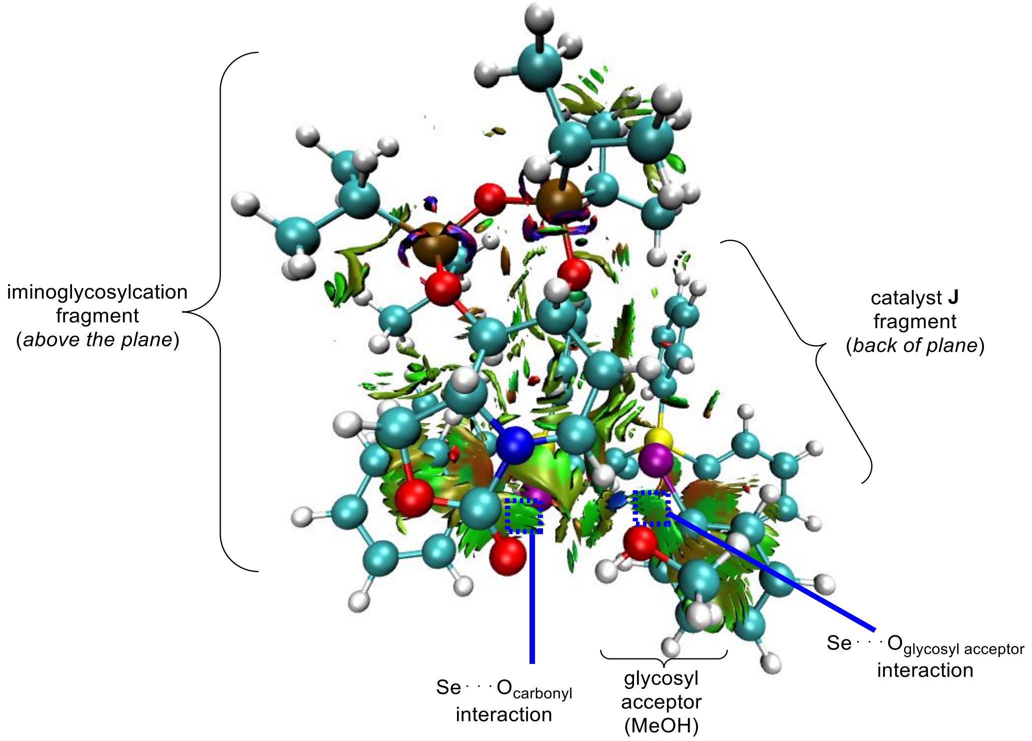

Supplementary Figure S50: NCI analysis of modelled complex **9**

8. References

1) García-Moreno, M. I., Ortiz Mellet, C., & García Fernández, J. M. Synthesis of Calystegine B2, B3, and B4 Analogues: Mapping the Structure-Glycosidase Inhibitory Activity Relationships in the 1-Deoxy-6-oxacalystegine Series. *European Journal of Organic Chemistry*, **2004**(*8*), 1803-1819.

2) Díaz Pérez, P., García-Moreno, M.I., Ortiz Mellet, C. and García Fernández, J.M., 2005. Synthesis and comparative glycosidase inhibitory properties of reducing castanospermine analogues. *Eur. J. Org. Chem*. **2005**, 2903–2913.

3) Herrera-González, I., Sánchez-Fernández, E. M., Sau, A., Nativi, C., García Fernández, J. M., Galán, M. C., & Ortiz Mellet, C. Stereoselective synthesis of iminosugar 2-deoxy (thio) glycosides from bicyclic iminoglycal carbamates promoted by Cerium (IV) ammonium nitrate and cooperative Brønsted acid-type organocatalysis. *The Journal of Organic Chemistry*, **2020**(*85)*, 5038-5047.

4) C. Xu, V. U. Bhaskara Rao, J. Weigen, C. C. J. Loh*, *Nat. Commun.* **2020**, *11*, 4911.

5) Raji Reddy C, Subbarao M, Kolgave DH, Ajaykumar U, Vinaya PP. Access to Diverse Seleno-spirocyclohexadienones via Ag(II)-Catalyzed Selenylative ipso-Annulation with Se and Boronic Acids. *ACS Omega*. **2022** (*42*), 38045-38052.

6) Sato, K. I., Akai, S., Sakuma, M., Kojima, M., & Suzuki, K. J. Practical synthesis of [1-13C]-and [6-13C]-d-galactose. *Tetrahedron letters*, **2003**(*44*), 4903-4907.

7) Hussain, N., Tatina, M. B., & Mukherjee, D. Cross dehydrogenative coupling of sugar enol ethers with terminal alkenes in the synthesis of pseudo-disaccharides, chiral oxadecalins and a conjugated triene. *Organic & Biomolecular Chemistry*, **2018** (*16*), 2666-2677.

8) Watterson, M. P., Pickering, L., Smith, M. D., Hudson, S. J., Marsh, P. R., Mordaunt, J. E., ... & Fleet, G. W. 3-Azidotetrahydrofuran-2-carboxylates: monomers for five-ring templated β-amino acid foldamers?. *Tetrahedron: Asymmetry*, **1999**(*10*), 1855-1859.

9) Mariano, S., Roos, A. K., Mowbray, S. L., & Salmon, L. Competitive inhibitors of type B ribose 5-phosphate isomerases: design, synthesis and kinetic evaluation of new D-allose and D-allulose 6-phosphate derivatives. *Carbohydrate research*, **2009**, *344*(7), 869-880.

10) C. Xu, V. U. Bhaskara Rao, J. Weigen, C. C. J. Loh*, *Nat. Commun.* **2020**, *11*, 4911.

11) Wang, W., Zhu, H., Liu, S., Zhao, Z., Zhang, L., Hao, J., & Wang, Y. Chalcogen–chalcogen bonding catalysis enables assembly of discrete molecules. *Journal of the American Chemical Society*, **2019**(*23*), 9175-9179.

12) Yuan, X., & Wang, Y. A Selenide Catalyst for the Activation of Alkenes through Se··· π Bonding. *Angewandte Chemie International Edition*, **2022**(*27*), e202203671.

13) Burés, J. A Simple Graphical Method to Determine the Order in Catalyst. *Angew. Chem. Int. Ed*. **2016**, *55*, 2028-2031.

14) Nielsen, C. D.-T.; Burés, J. Visual kinetic analysis, *Chem. Sci*., **2019**, *10*, 348-353.

15) Dolomanov, O.V., Bourhis, L.J., Gildea, R.J, Howard, J.A.K. & Puschmann, H. (**2009**), *J. Appl. Cryst.* 42, 339-341.

16) Sheldrick, G.M. (**2015**). *Acta Cryst.* A71, 3-8.

17) Sheldrick, G.M. (**2015**). *Acta Cryst.* C71, 3-8.

18) Neese, F. The ORCA program system. *Wiley Interdiscip. Rev. Comput. Mol. Sci.* **2012**, *2*, 73-78.

19) Nesse, F. Software update: The ORCA program system—Version 5.0. *WIREs Comput. Mol. Sci.* **2022**, *5*, e1606.

20) Bannwarth, C.; Ehlert, S.; Grimme, S. GFN2-xTB — An Accurate and Broadly Parametrized Self-Consistent Tight-Binding Quantum Chemical Method with Multipole Electrostatics and Density-Dependent Dispersion Contributions. *J. Chem. Theory Comput.* **2019**, *15*, 1652-1671.

21) Ehlert, S.; Stahn, M.; Spicher, S.; Grimme, S. Robust and Efficient Implicit Solvation Model for Fast Semiempirical Methods. *J. Chem. Theory Comput.* **2021**, *17*, 4250-4261.

22) Pracht, P.; Bohle, F.; Grimme, S. Automated exploration of the low-energy chemical space with fast quantum chemical methods. *Phys. Chem. Chem. Phys.* **2020**, *22*, 7169-7192.

23) Zhao, Y.; Truhlar, D. G., The M06 suite of density functionals for main group thermochemistry, thermochemical kinetics, noncovalent interactions, excited states, and transition elements: two new functionals and systematic testing of four M06-class functionals and 12 other functionals. *Theor. Chem. Acc.* **2008**, *120*, 215-241.

24) Grimme, S.; Antony, J.; Ehrlich, S.; Krieg, H. A consistent and accurate *ab initio* parametrization of density functional dispersion correction (DFT-D) for the 94 elements H-Pu. *J. Chem. Phys.* **2010**, *132*, 154104.

25) Grimme, S.; Hansen, A.; Brandenburg, J. G.; Bannwarth, C., Dispersion-Corrected Mean-Field Electronic Structure Methods. *Chem. Rev.* **2016**, *116*, 5105-5154.

26) Weigend, F.; Ahlrichs, R., Balanced basis sets of split valence, triple zeta valence and quadruple zeta valence quality for H to Rn: Design and assessment of accuracy. *Phys. Chem. Chem. Phys.* **2005,** *7*, 3297-3305.

27) de Azevedo Santos, L.; Ramalho, T. C.; Hamlin, T. A.; Bickelhaupt, F. M., Chalcogen bonds: Hierarchical ab initio benchmark and density functional theory performance study. *J. Comput. Chem.* **2021,** *42*, 688-698.

28) Bauzá, A.; Alkorta, I.; Frontera, A.; Elguero, J., On the Reliability of Pure and Hybrid DFT Methods for the Evaluation of Halogen, Chalcogen, and Pnicogen Bonds Involving Anionic and Neutral Electron Donors. *J. Chem. Theory Comput.* **2013,** *9*, 5201-5210.

29) Garcia-Ratés, M.; Nesse, F. Effect of the Solute Cavity on the Solvation Energy and its Derivatives within the Framework of the Gaussian Charge Scheme. *J. Comput. Chem.* **2020**, *41*, 922-939.

30) Barone, V.; Cossi, M. Potential energy surfaces for the low-lying ^2A‘ and ^2A’ States of HO_2: Use of the diatomics in molecules model to fit *ab initio* data**.** *J. Phys. Chem. A* **1998**, *102*, 1995.

31) F. Neese, F. Wennmohs, A. Hansen, U. Becker. Efficient, approximate and parallel Hartree–Fock and hybrid DFT calculations. A ‘chain-of-spheres’ algorithm for the Hartree–Fock exchange. *Chem. Phys.* **2009**, *356*, 98-109.

32) K. Eichkorn, O. Treutler, H. Oehm, M. Häser, R. Ahlrichs. Auxiliary basis sets to approximate Coulomb potentials *Chem. Phys.* **1995**, *242*, 652-660.

33) K. Eichkorn, F. Weigend, O. Treutler, R. Ahlrichs. Auxiliary basis sets for main row atoms and transition metals and their use to approximate Coulomb potentials. *Theor. Chem. Acc.* **1997**, *97*, 119-124.

34) Grimme, S. Supramolecular Binding Thermodynamics by Dispersion-Corrected Density Functional Theory. *Chem. Eur. J.* **2012**, *18*, 9955-9964.

35) E. R Johnson, S. Keinan, P. Mori-Sánchez, J. Contreras-García, A. J. Cohen, W. Yang, Revealing Noncovalent Interactions. *J. Am. Chem. Soc.* **132**, 6498-6506 (2010).

36) T. Lu, F. Chen, Multiwfn: A Multifunctional Wavefunction Analyzer, *J. Comput. Chem.* **2012**, *33*, 580-592.

37) CYLview20; Legault, C. Y., Université de Sherbrooke, **2020** (http://www.cylview.org)

9. NMR spectra

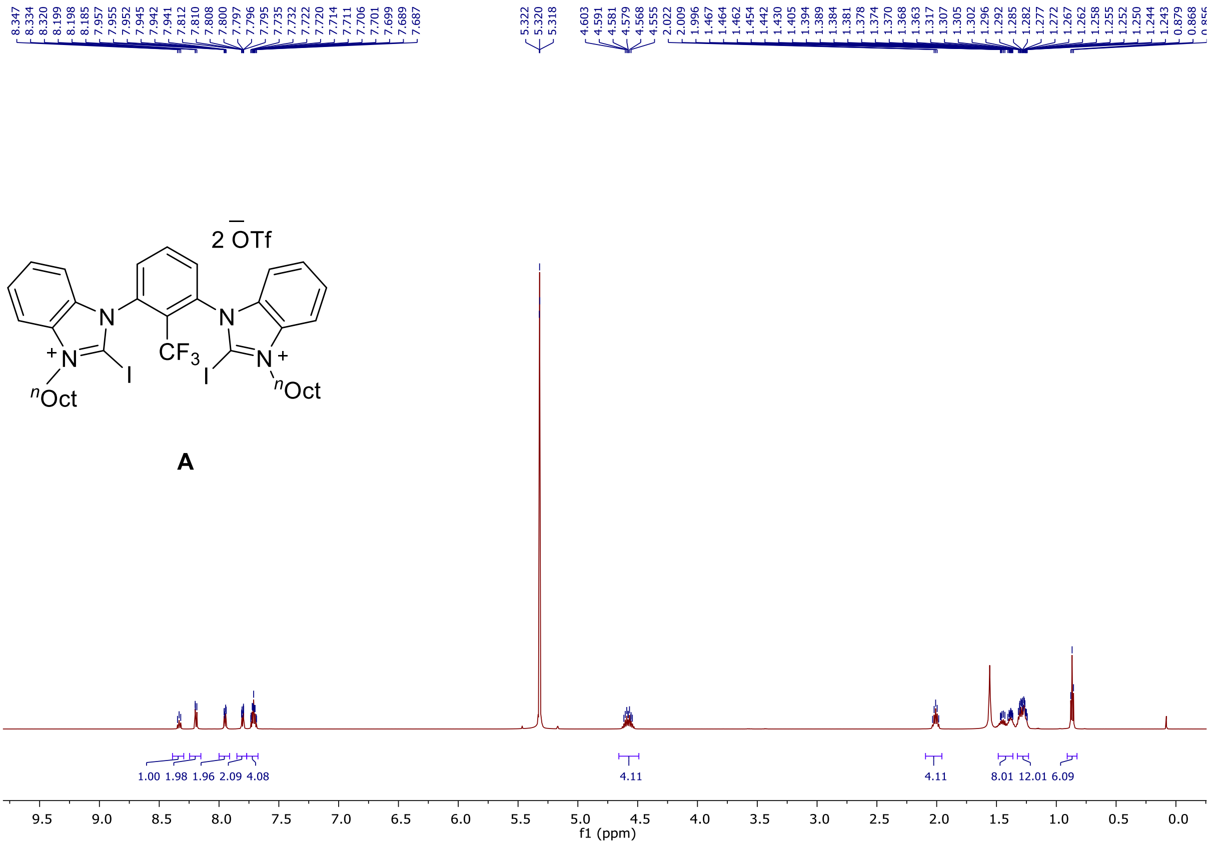

Supplementary Figure S51. ^{1}H NMR spectra for A

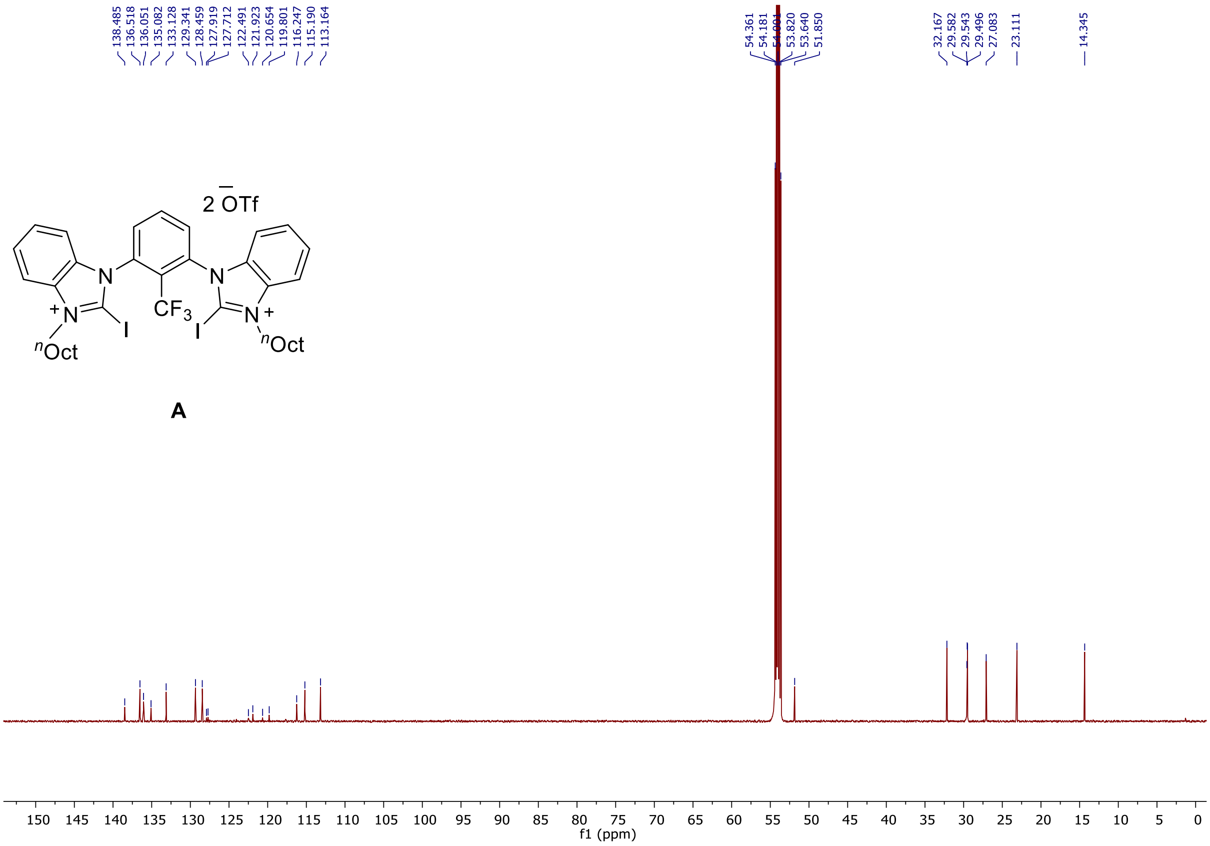

Supplementary Figure S52. ^{13}C NMR spectra for A

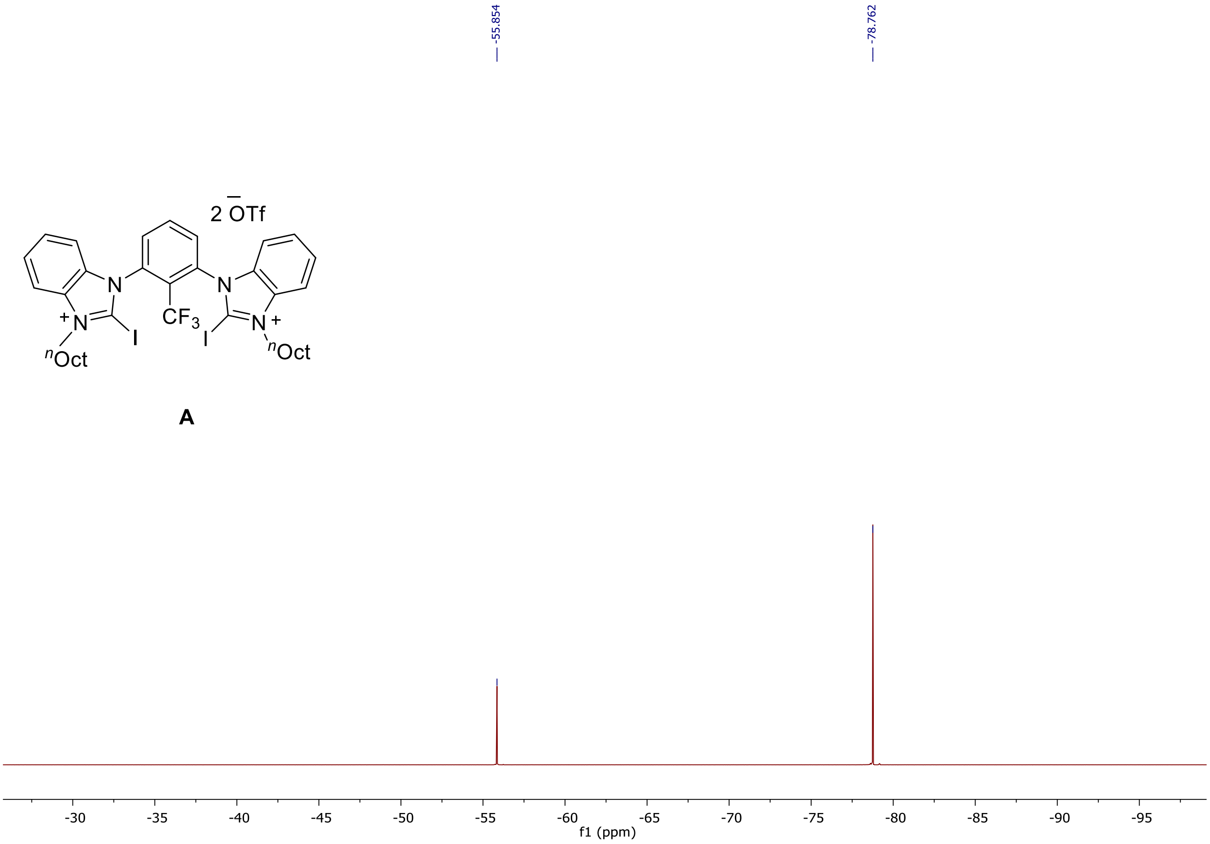

Supplementary Figure S53. ^{19}F NMR spectra for A

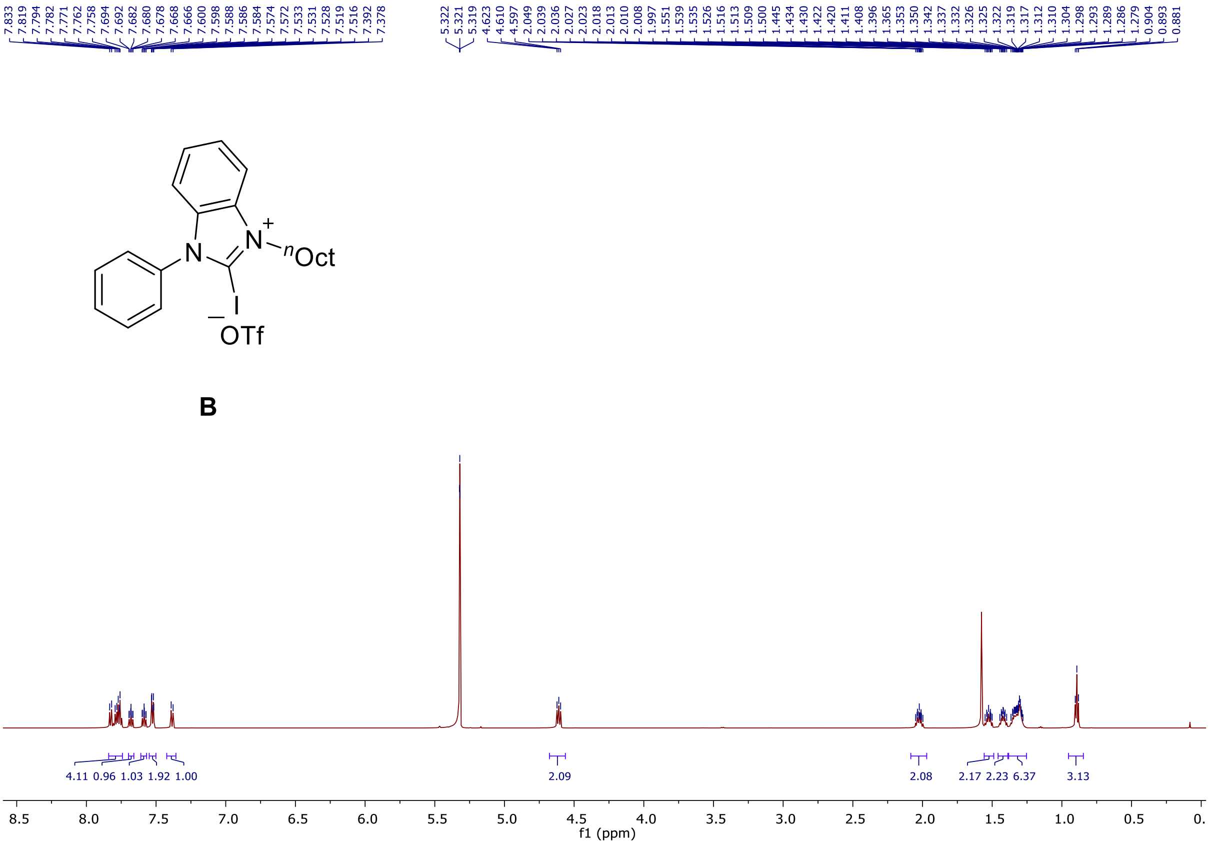

Supplementary Figure S54. ^{1}H NMR spectra for B

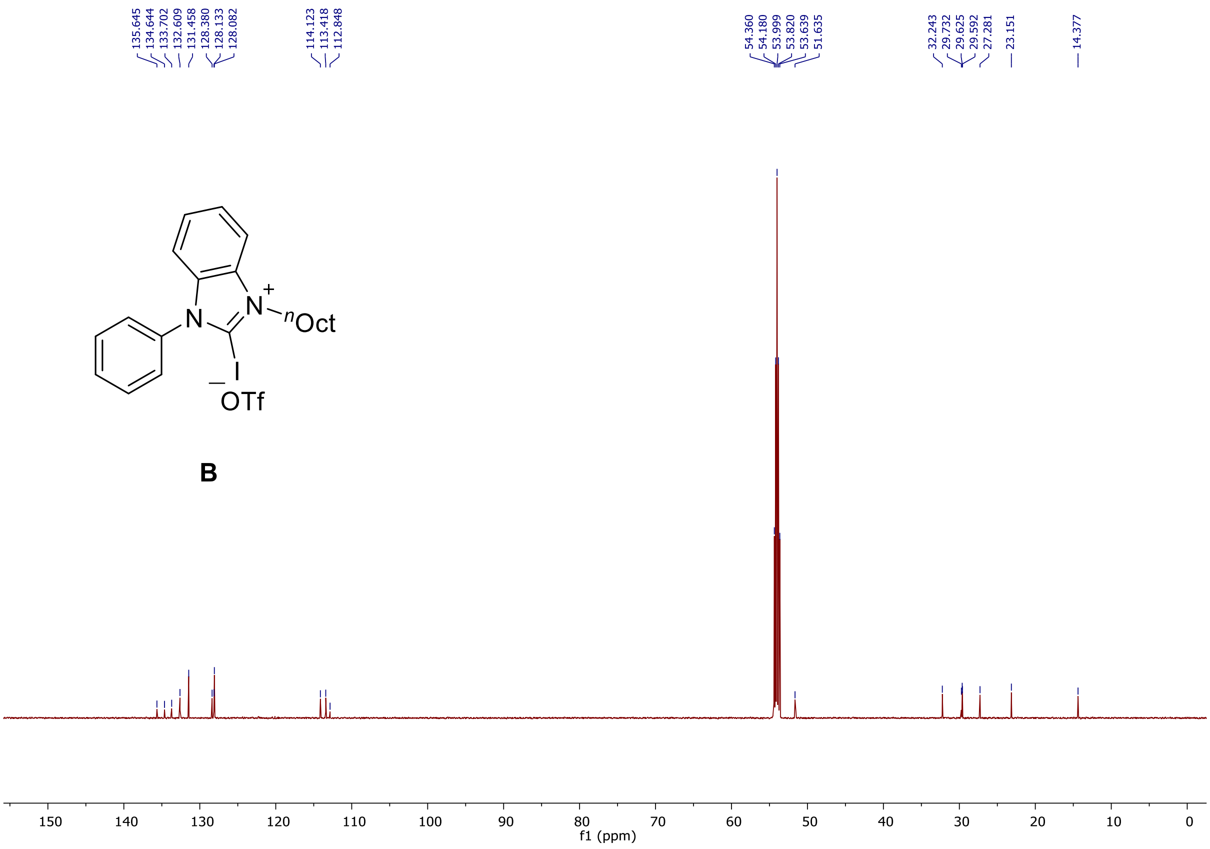

Supplementary Figure S55. ^{13}**C NMR spectra for B**

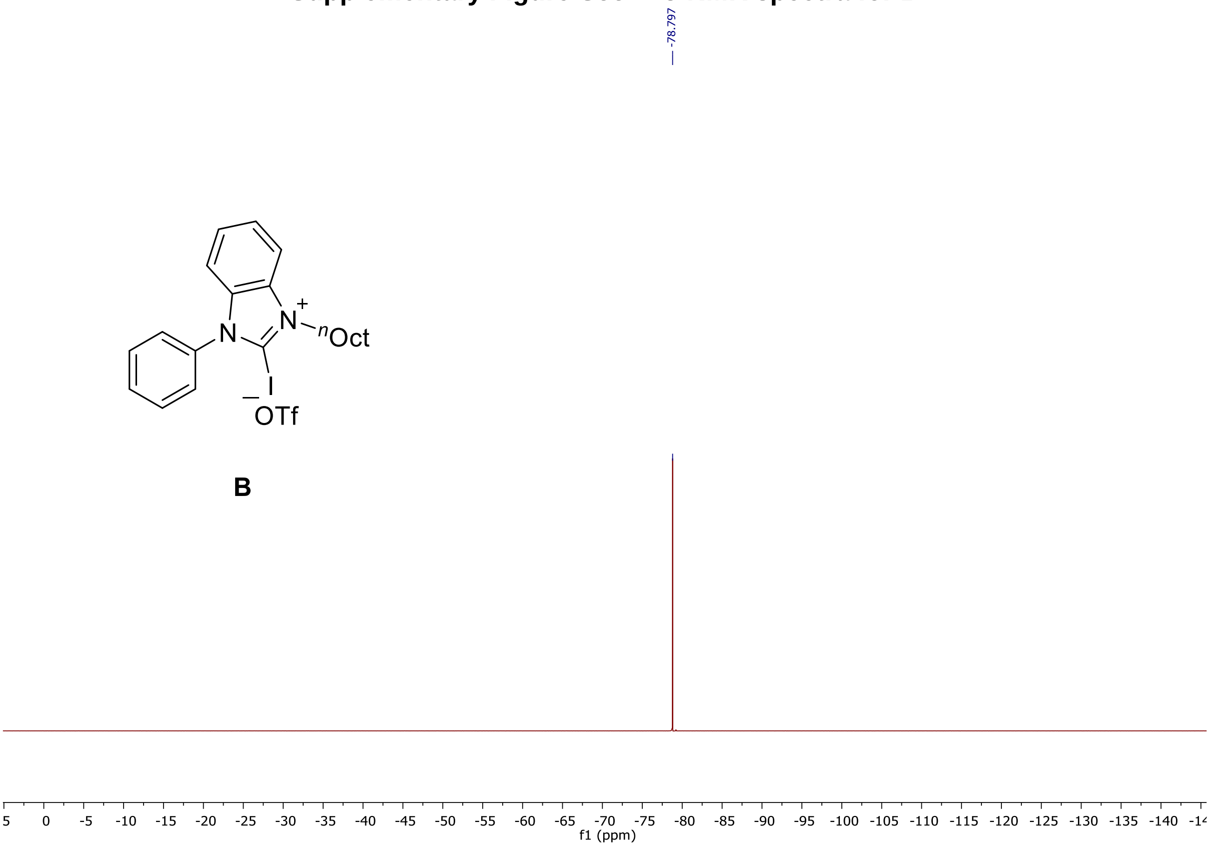

Supplementary Figure S56. ^{19}**F NMR spectra for B**

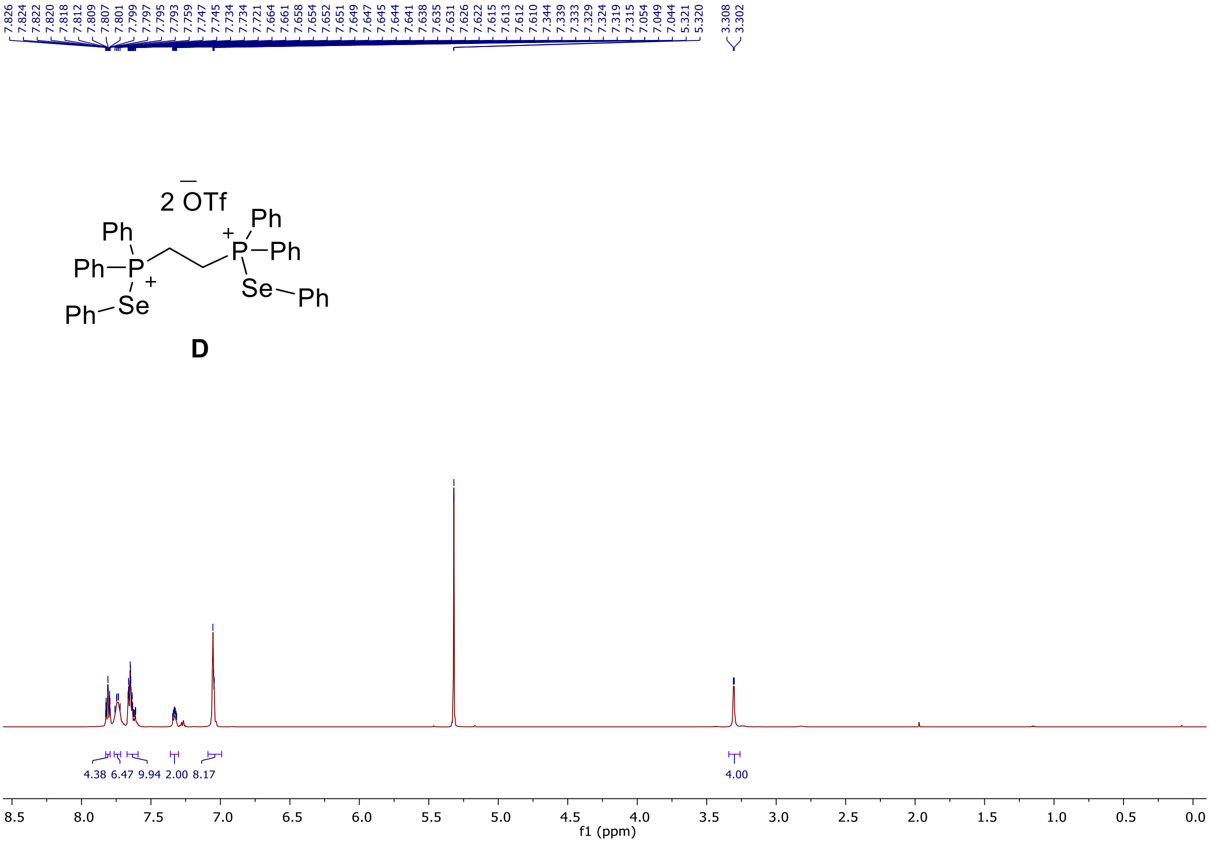

Supplementary Figure S57. ^{1}H NMR spectra for D

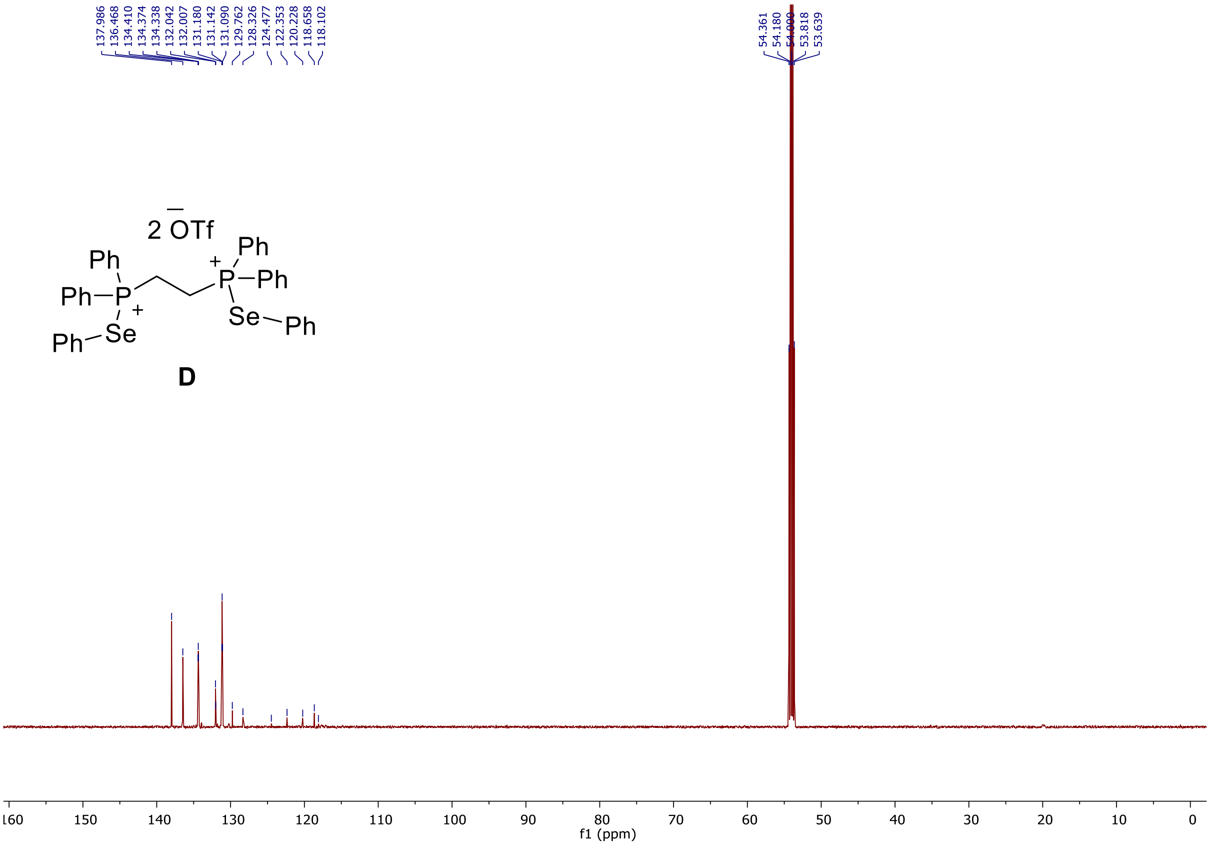

Supplementary Figure S58. ^{13}C NMR spectra for D

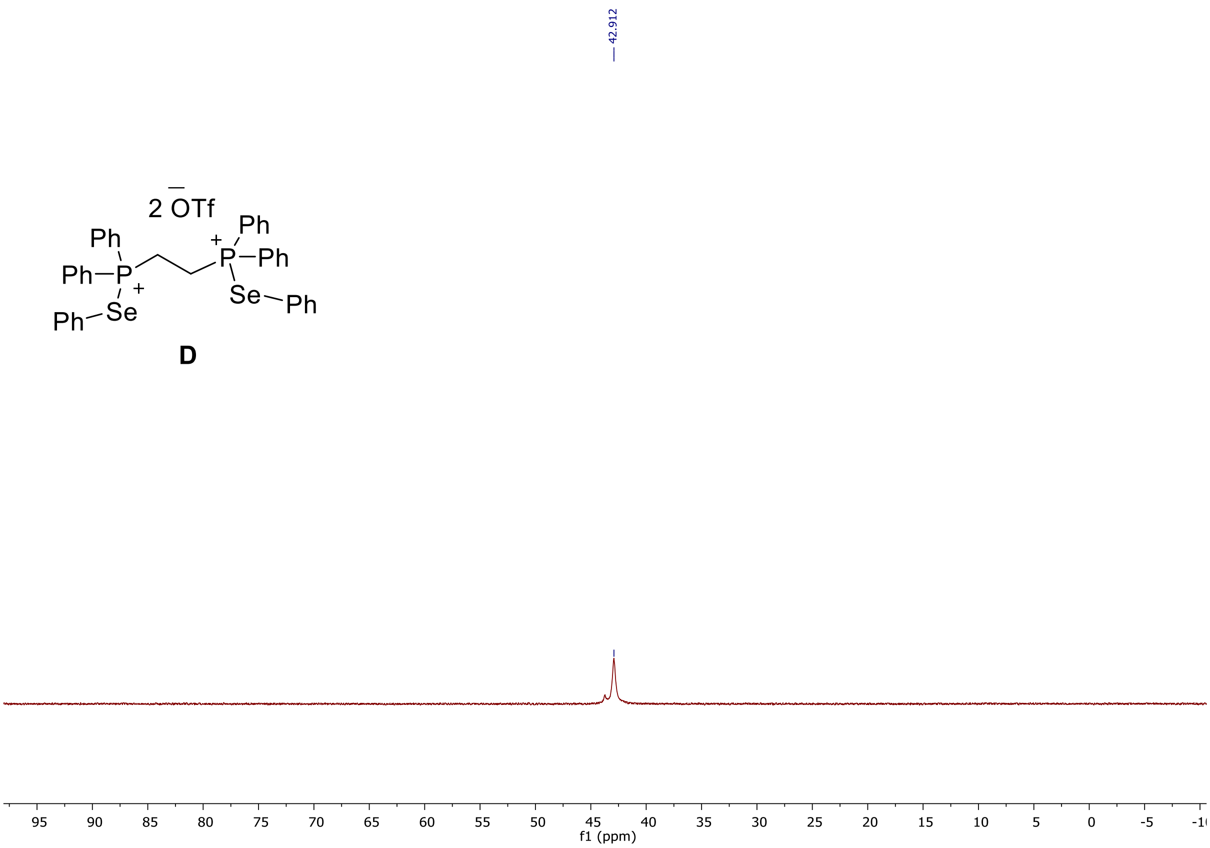

Supplementary Figure S59. ^{31}P NMR spectra for D

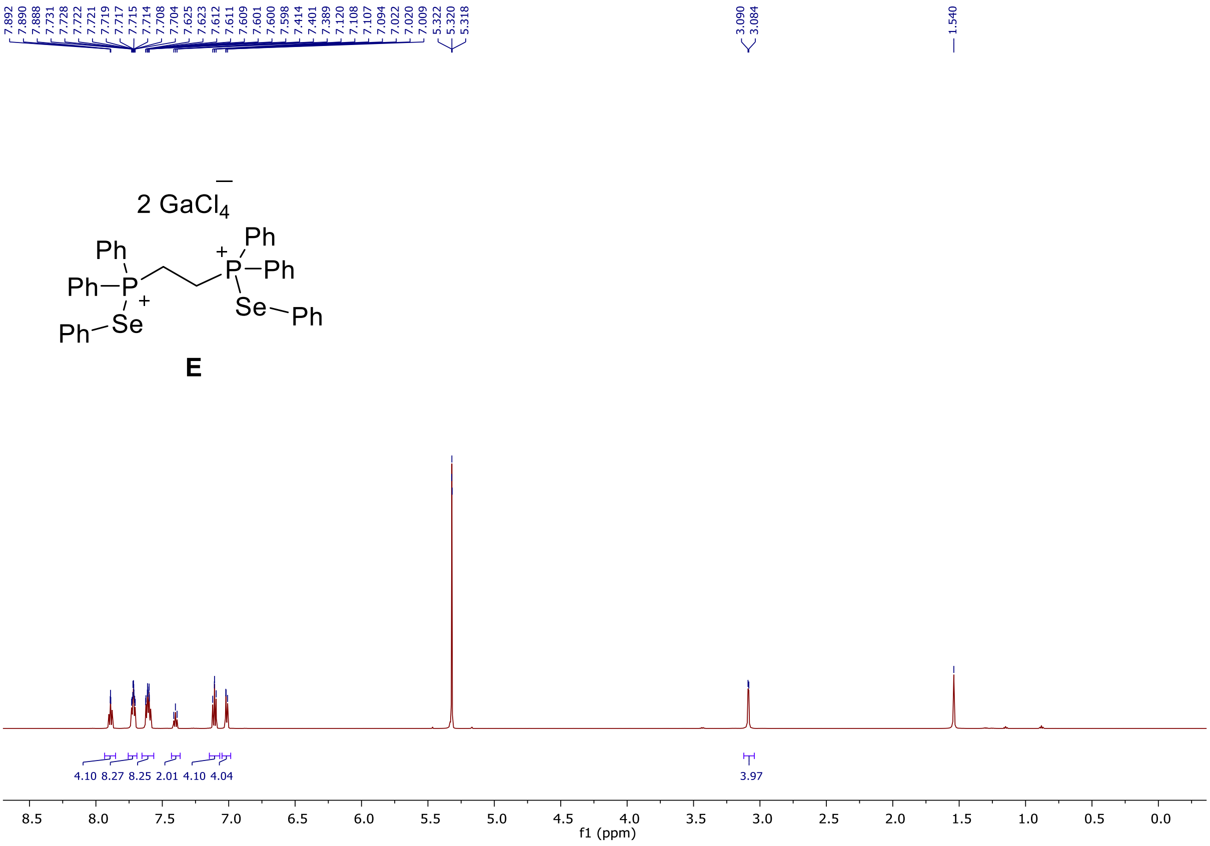

Supplementary Figure S60. ^{1}H NMR spectra for E

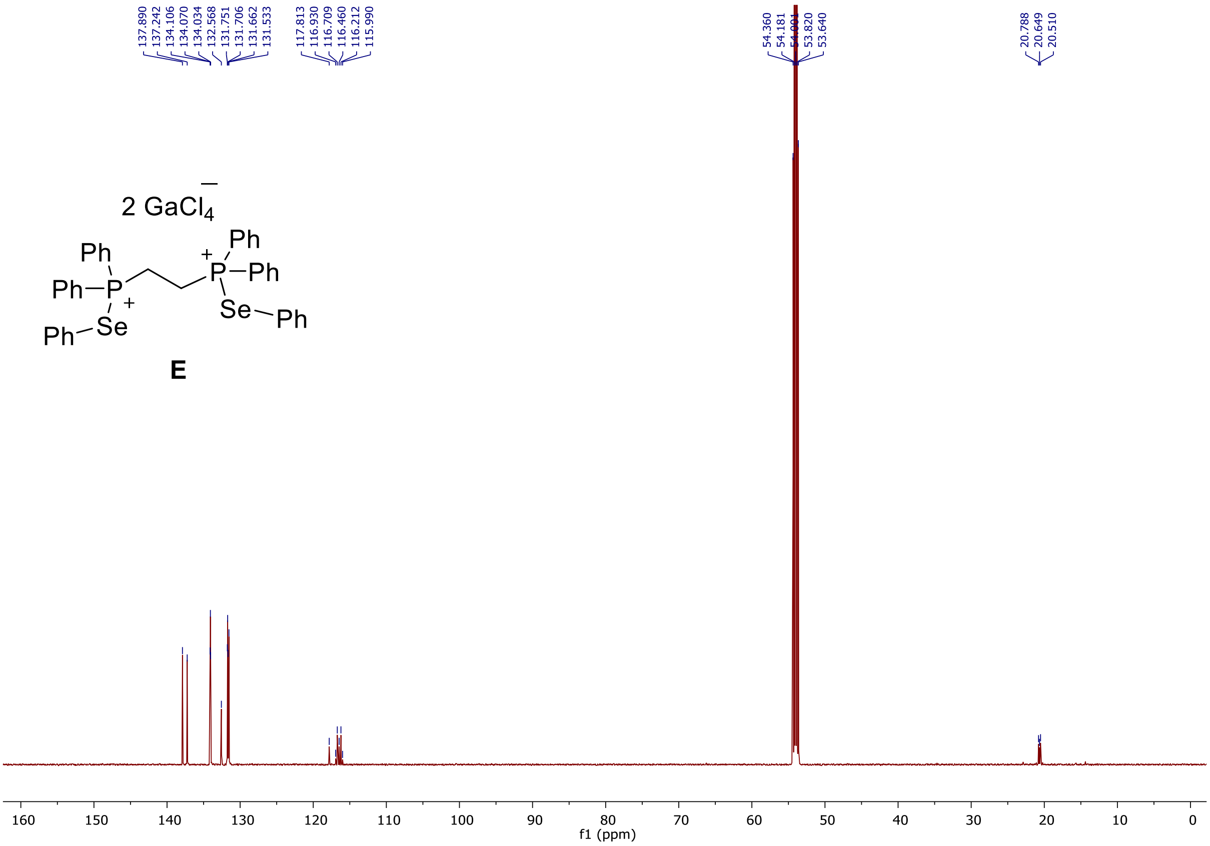

Supplementary Figure S61. ^{13}**C NMR spectra for E**

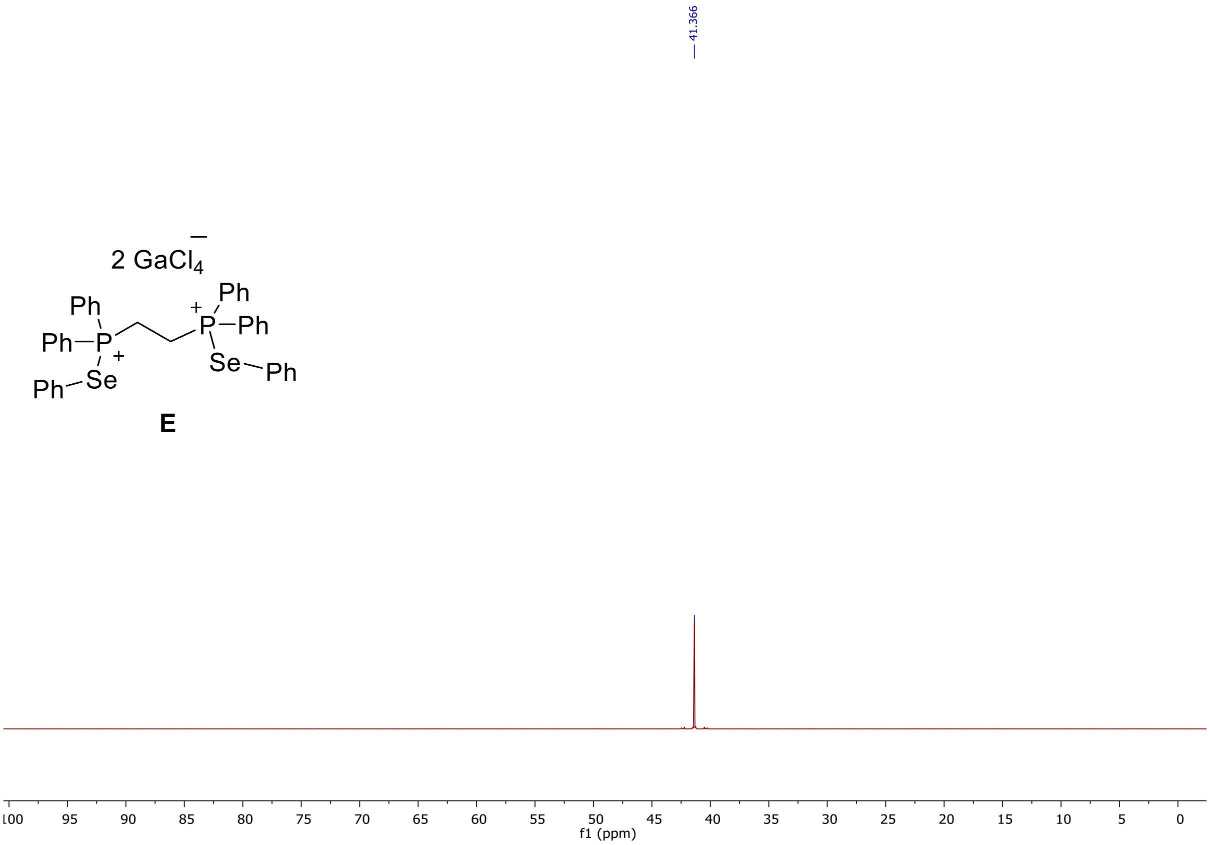

Supplementary Figure S62. ^{31}**P NMR spectra for E**

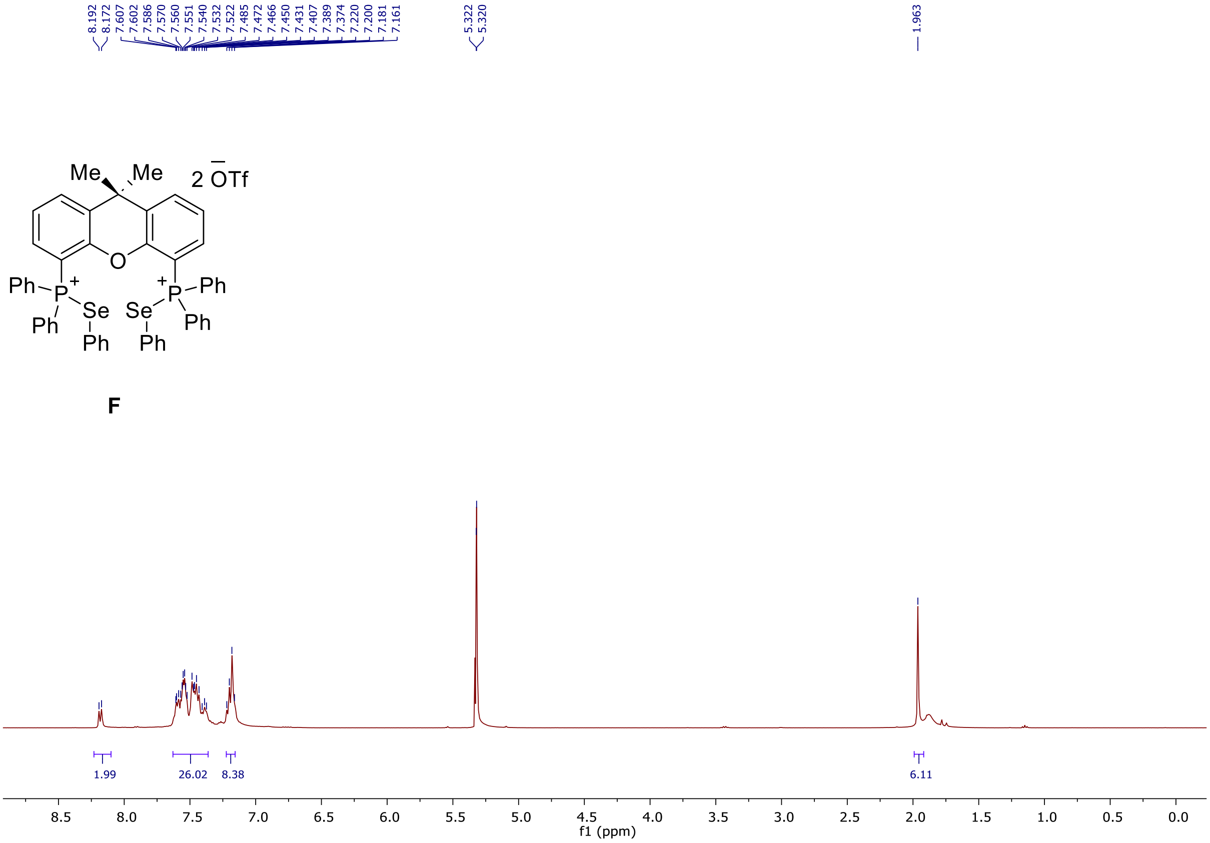

Supplementary Figure S63. ^{1}H NMR spectra for F

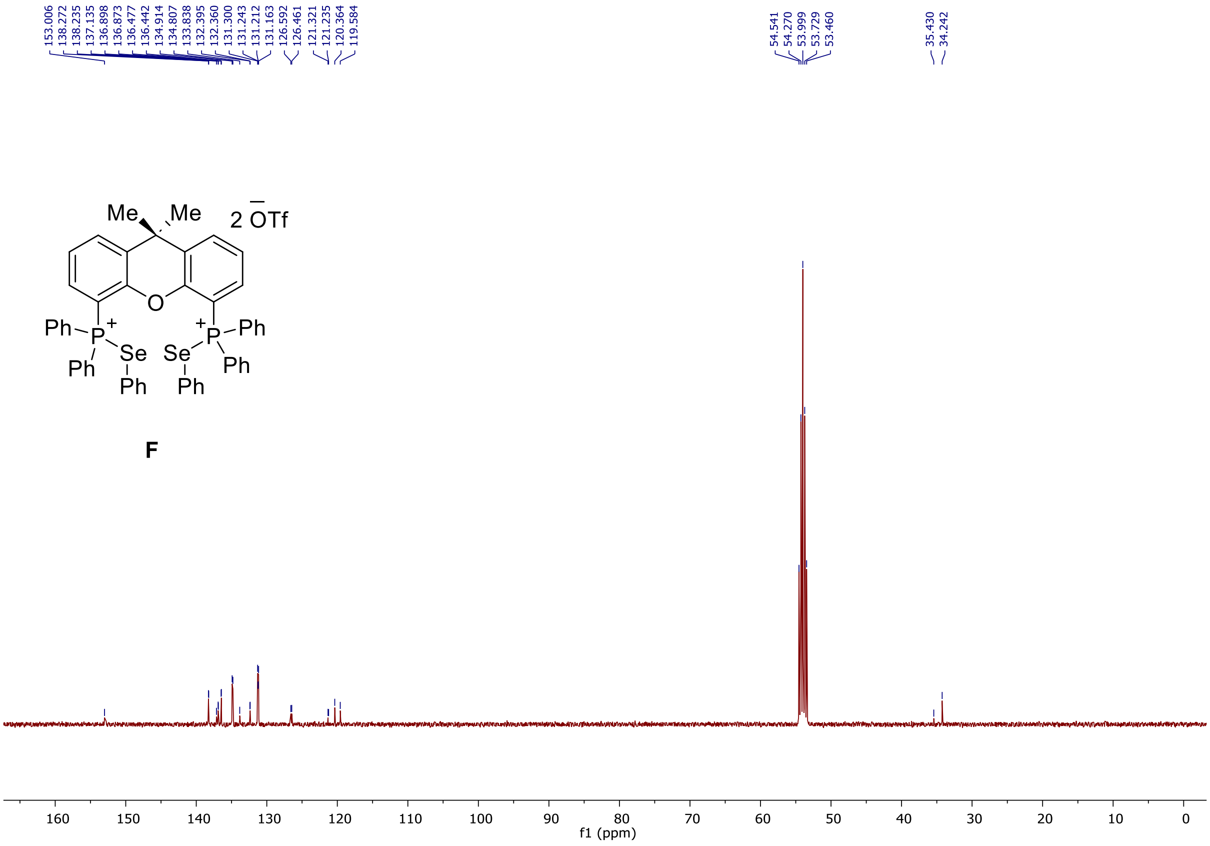

Supplementary Figure S64. ^{13}C NMR spectra for F

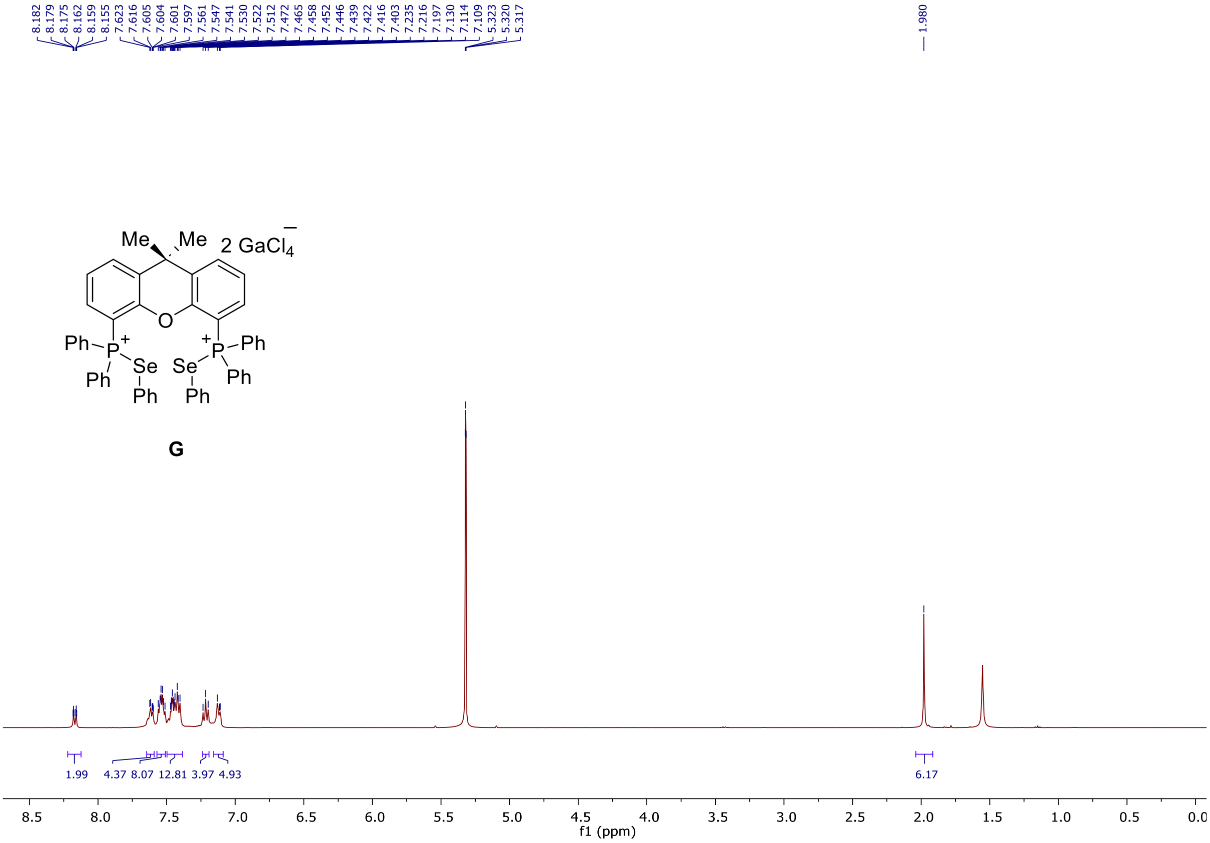

Supplementary Figure S65. ^{1}H NMR spectra for G

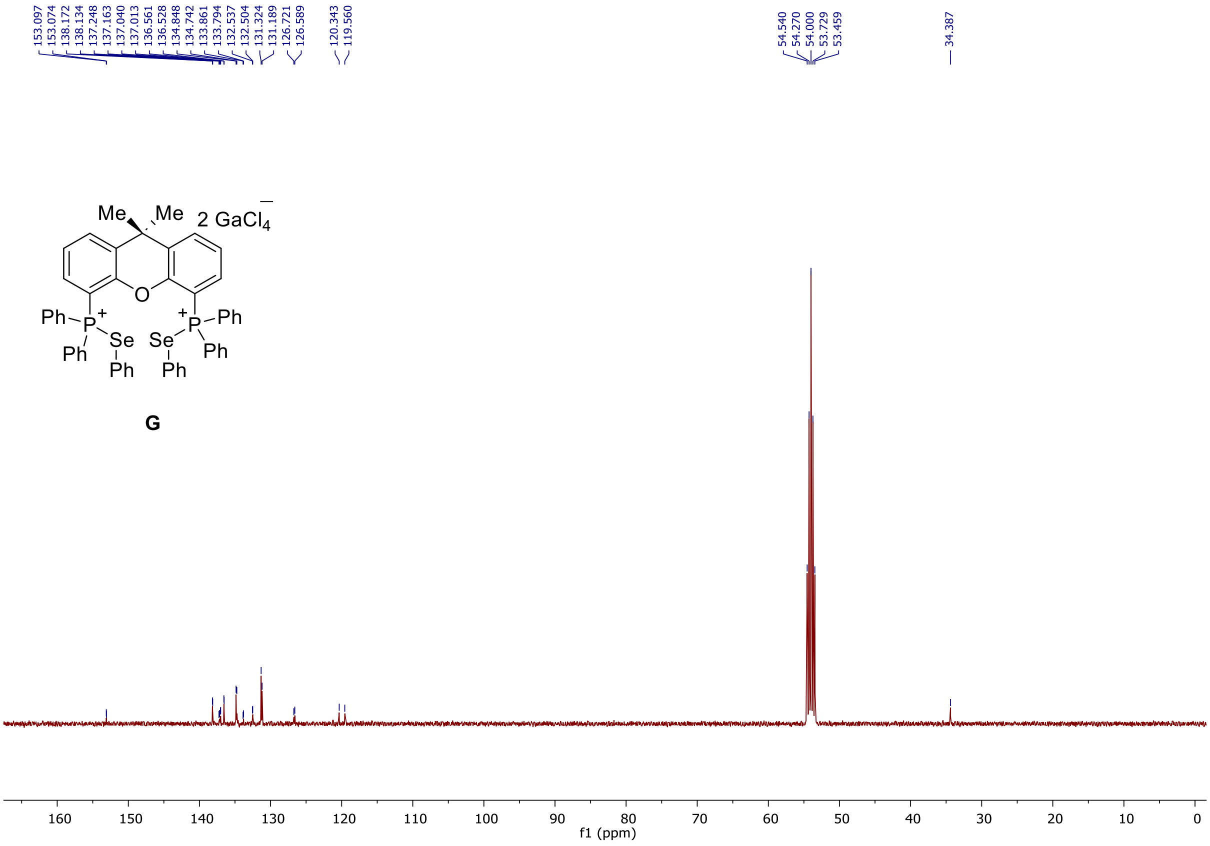

Supplementary Figure S66. ^{13}C NMR spectra for G

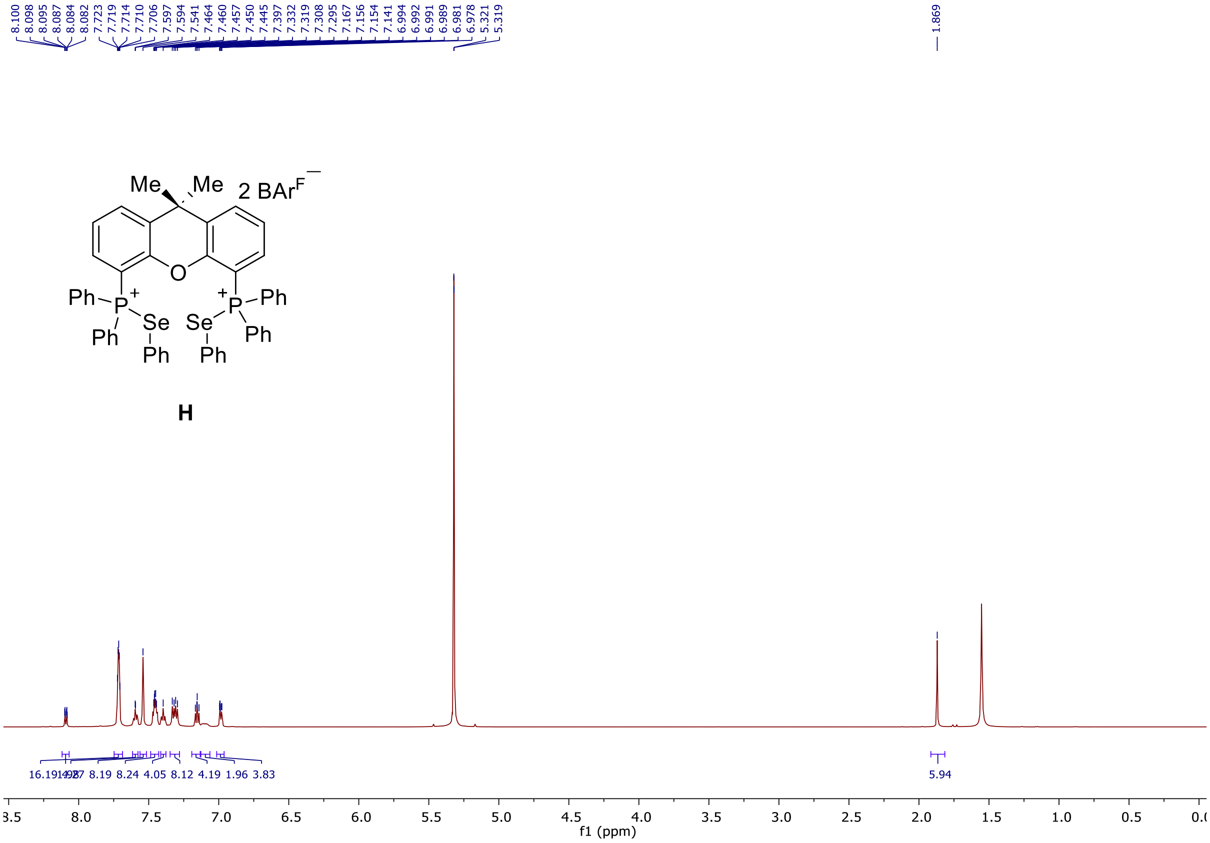

Supplementary Figure S67. ^{1}**H NMR spectra for H**

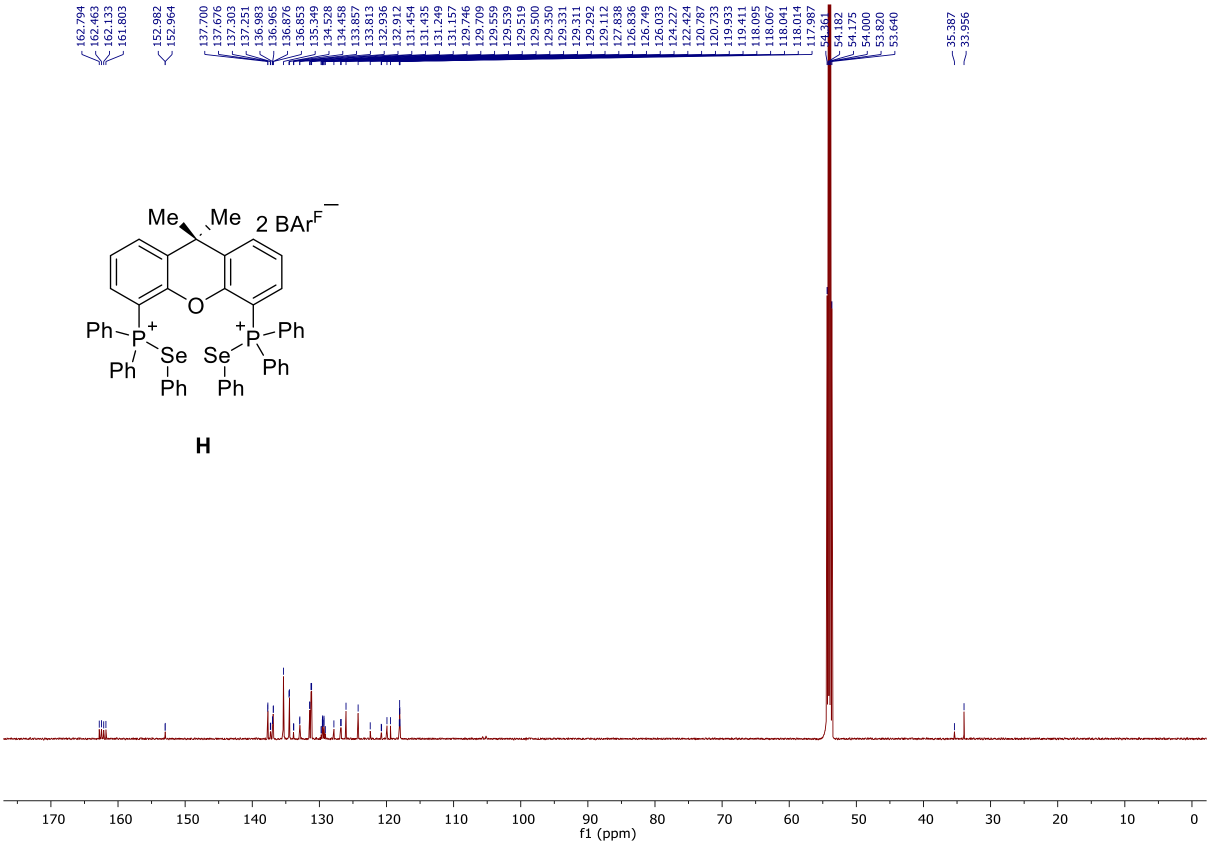

Supplementary Figure S68. ^{13}**C NMR spectra for H**

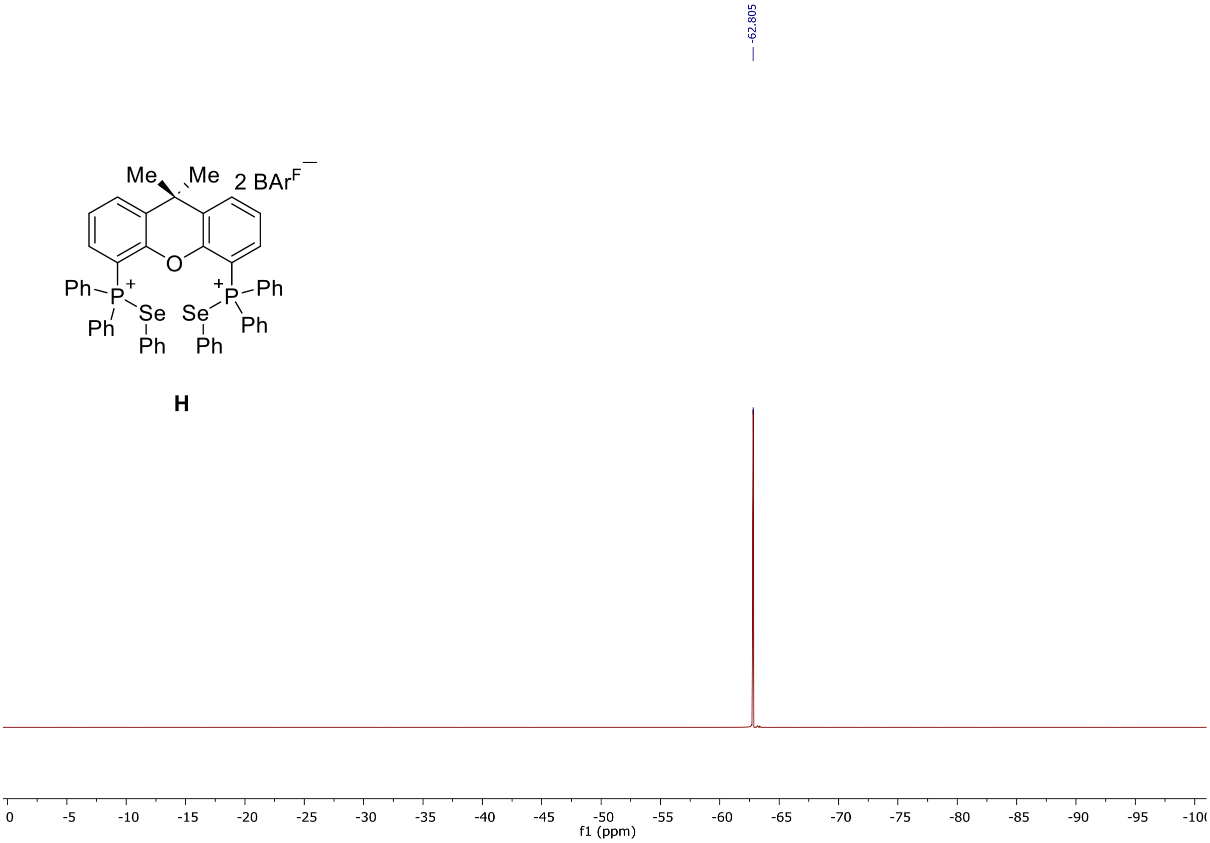

Supplementary Figure S69. ^{19}F NMR spectra for H

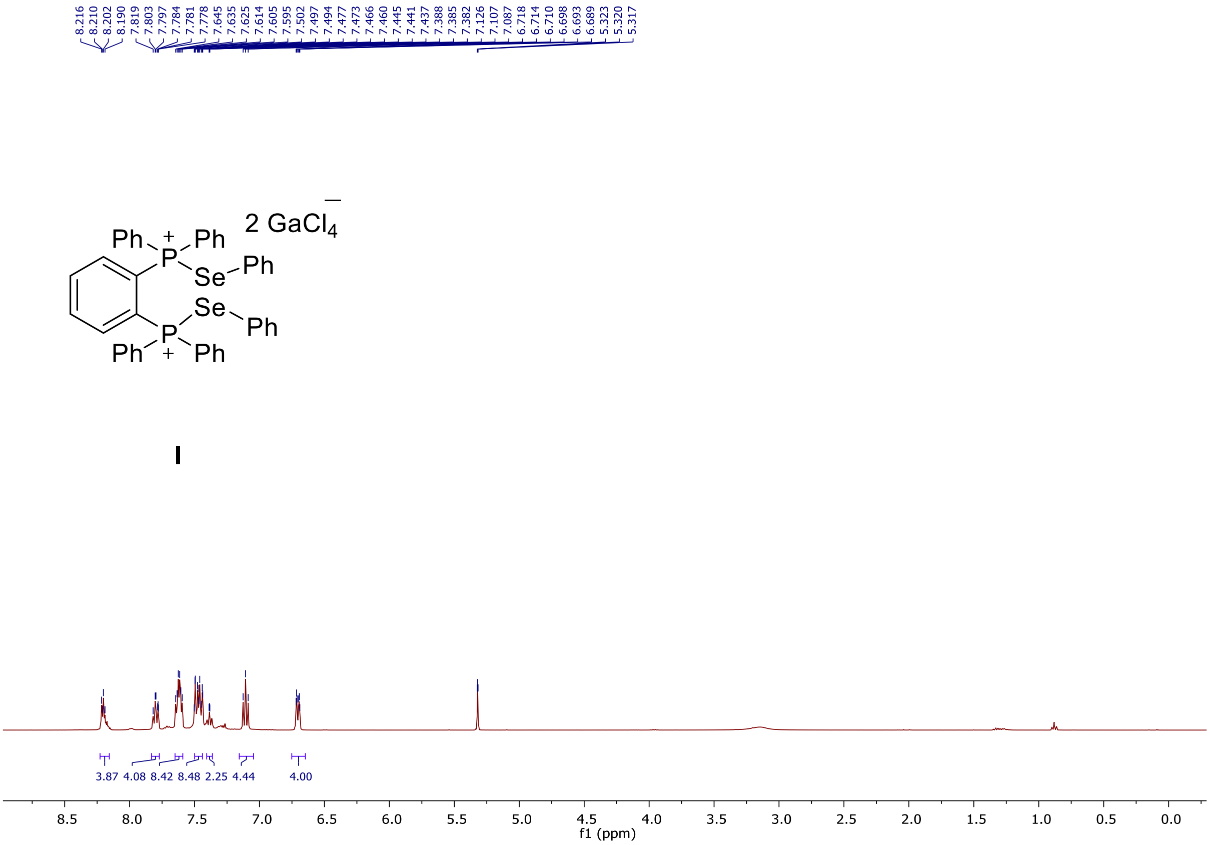

Supplementary Figure S70. ^{1}H NMR spectra for I

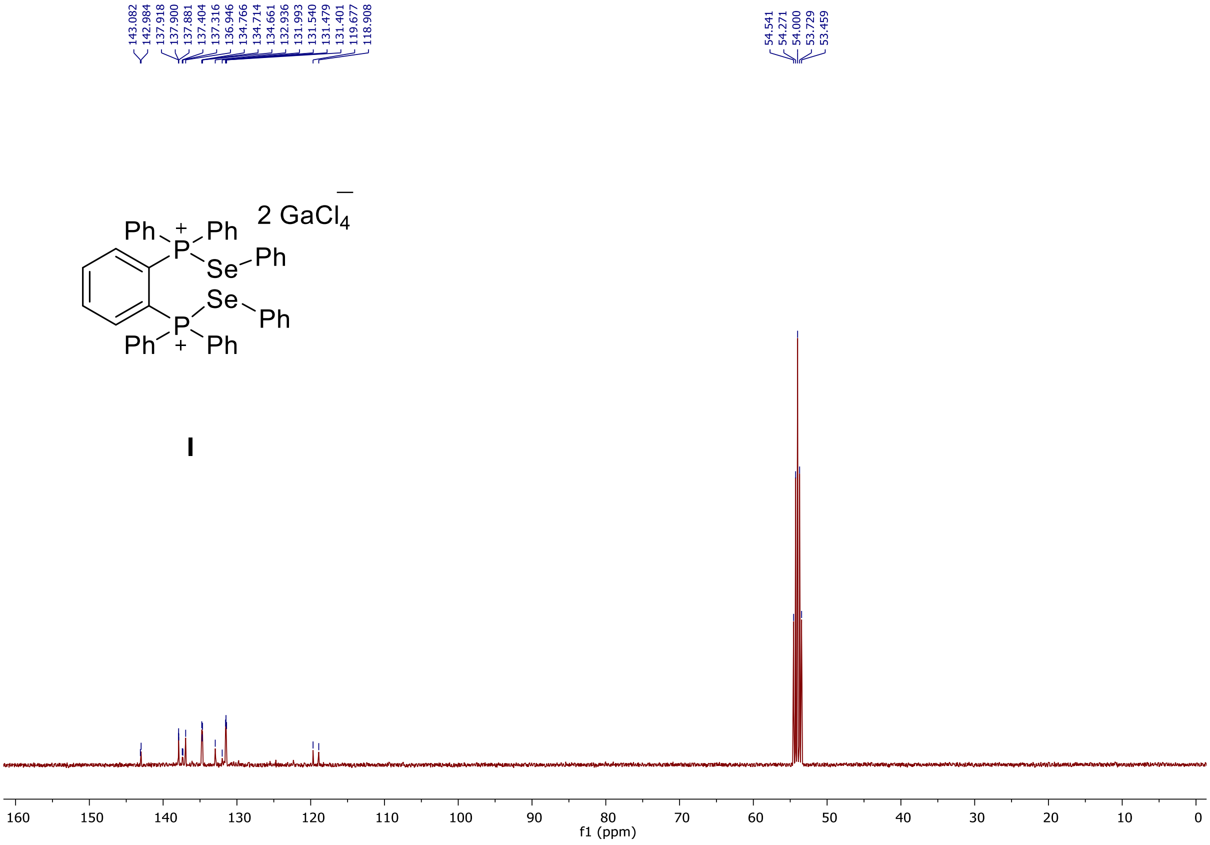

Supplementary Figure S71. ^{13}C NMR spectra for I

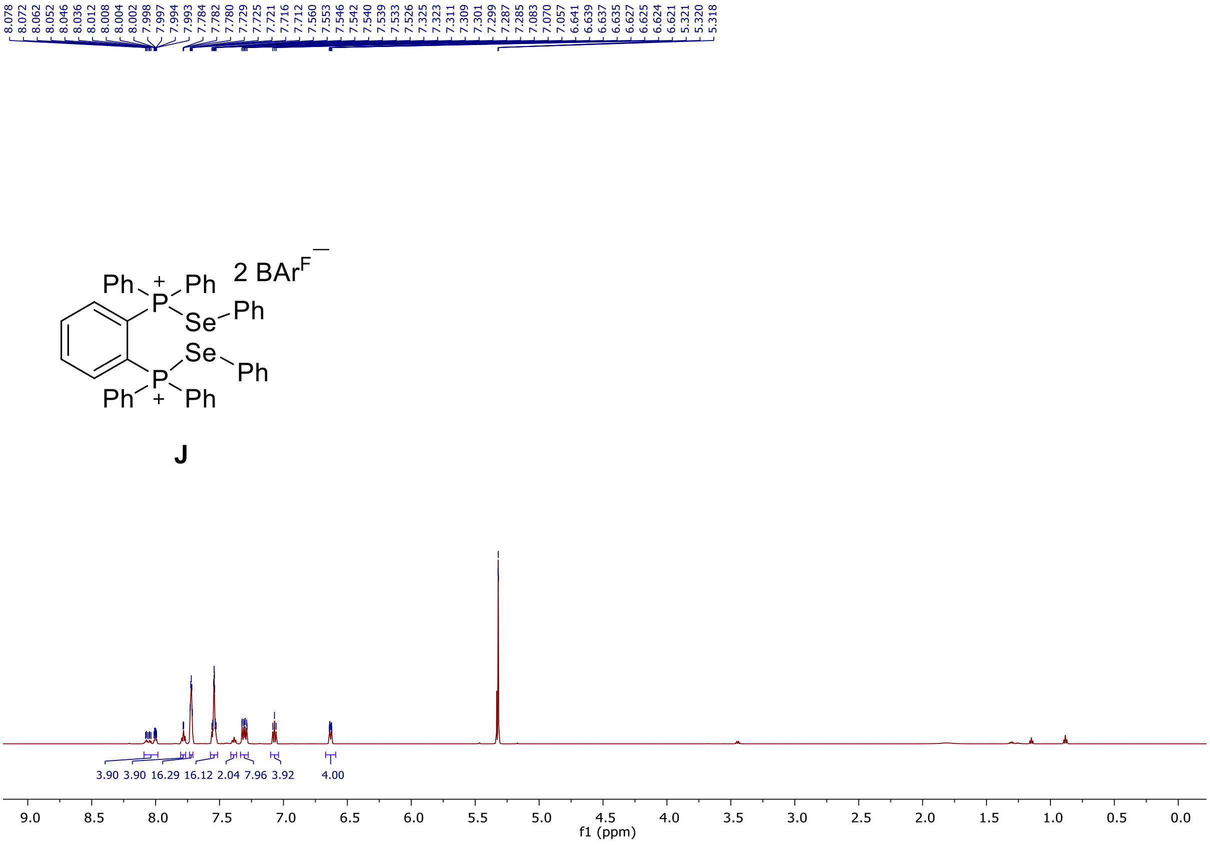

Supplementary Figure S72. ^{1}H NMR spectra for J

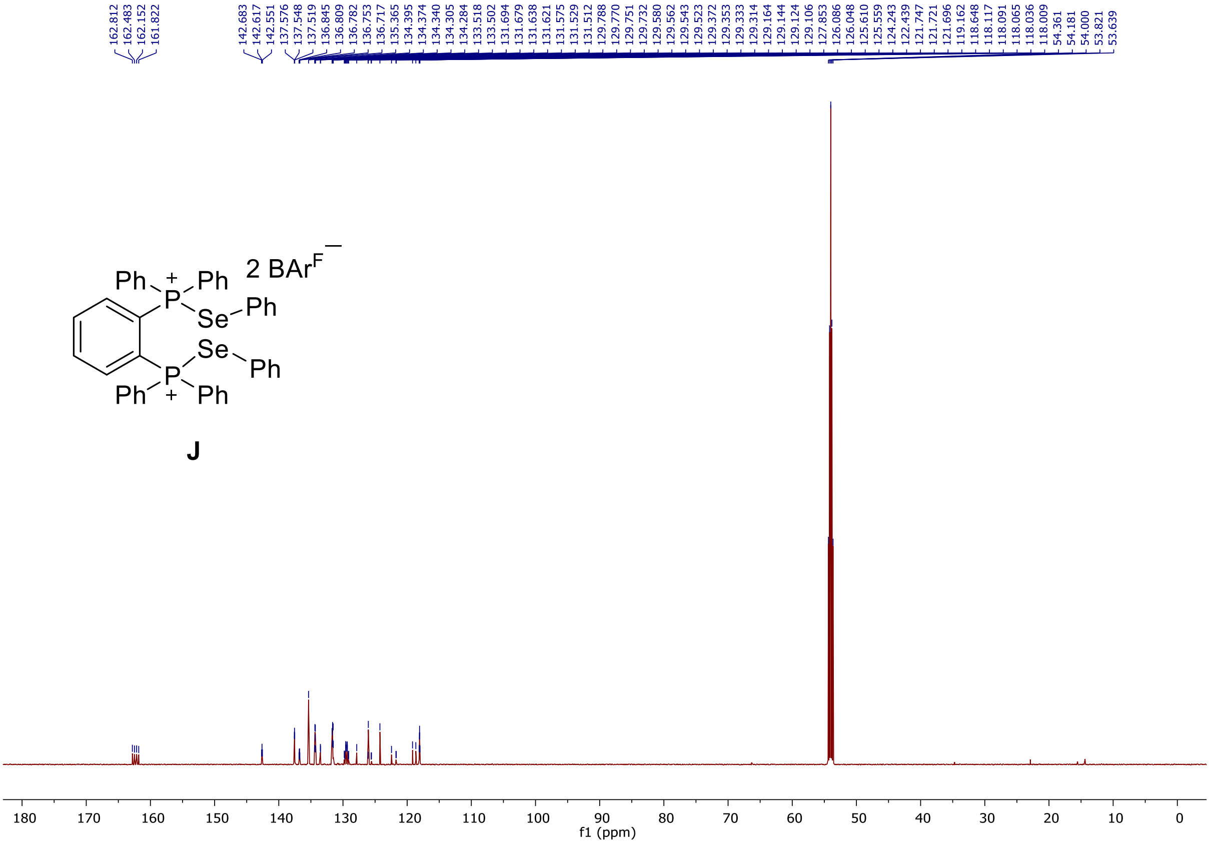

Supplementary Figure S73. ^{13}C NMR spectra for J

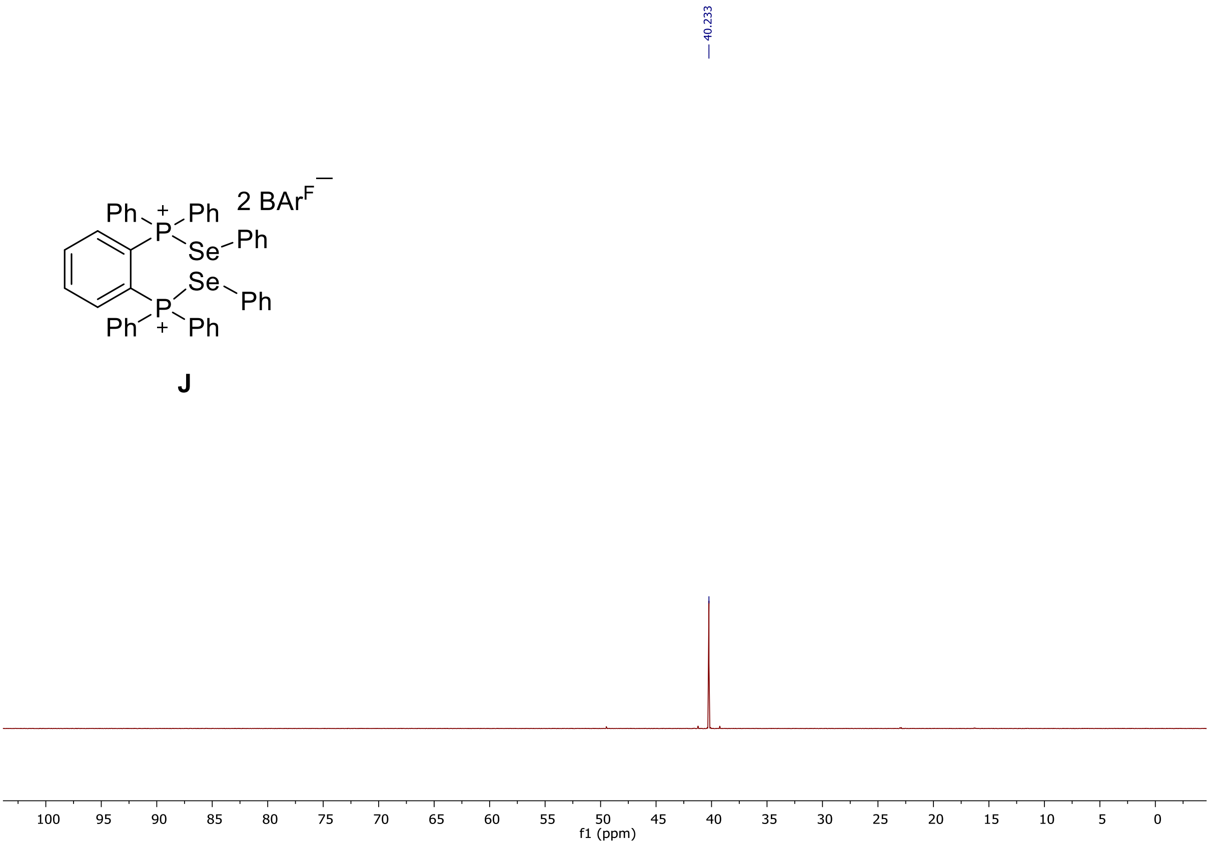

Supplementary Figure S74. ^{31}P NMR spectra for J

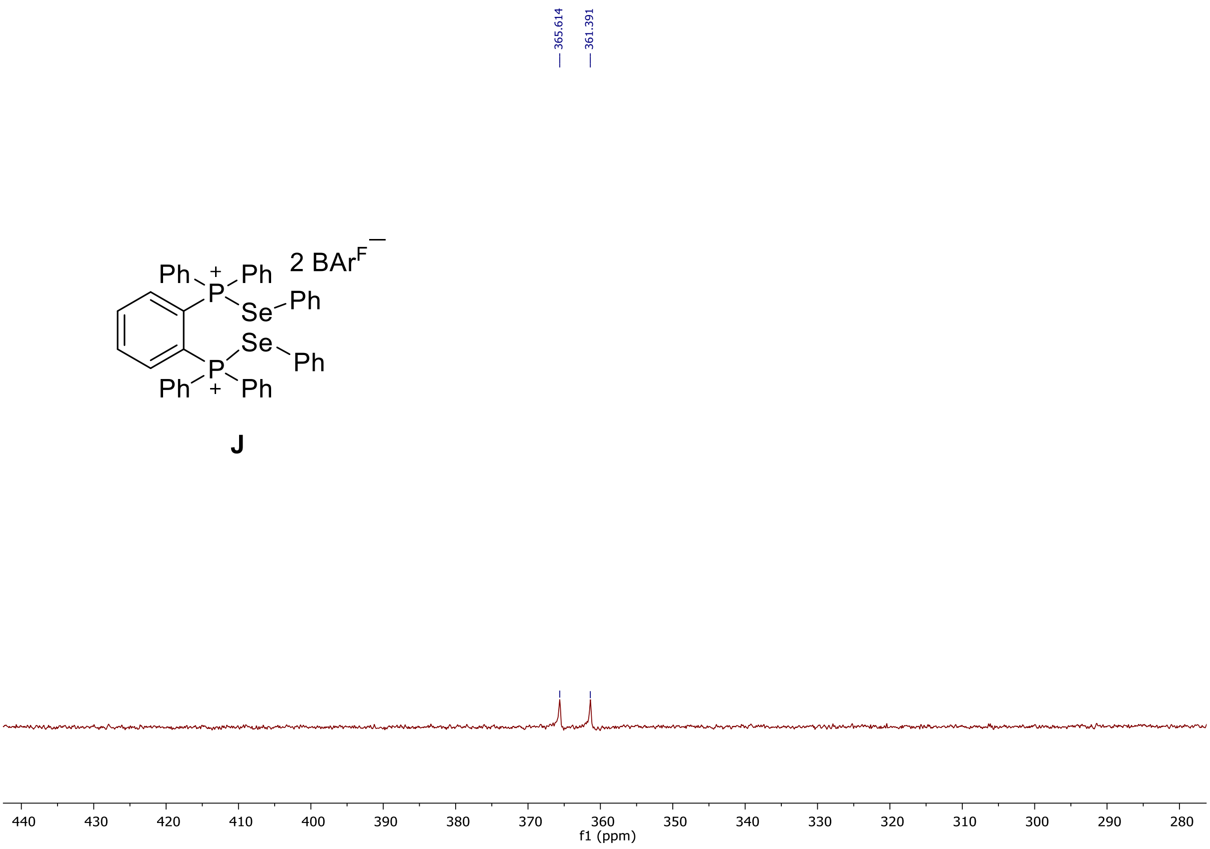

Supplementary Figure S75. ^{77}Se NMR spectra for J

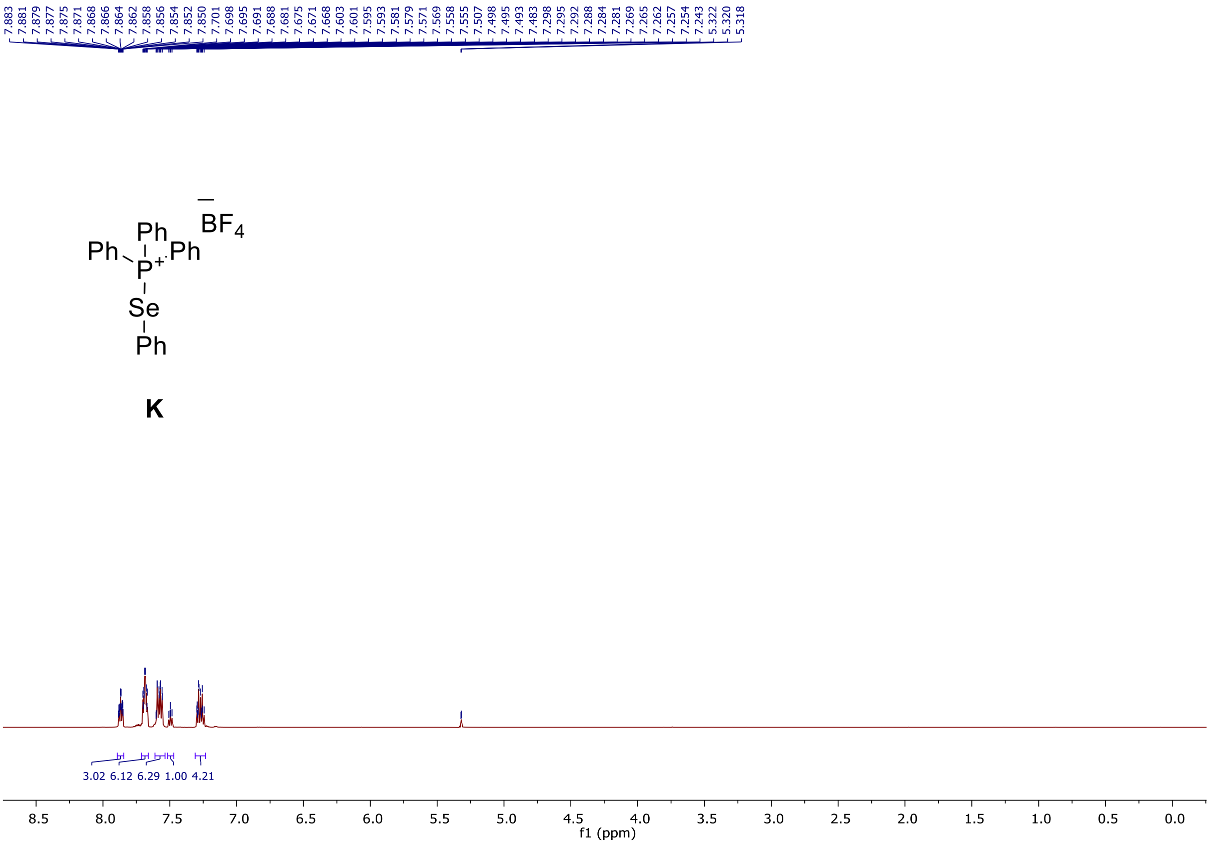

Supplementary Figure S76. ^{1}H NMR spectra for K

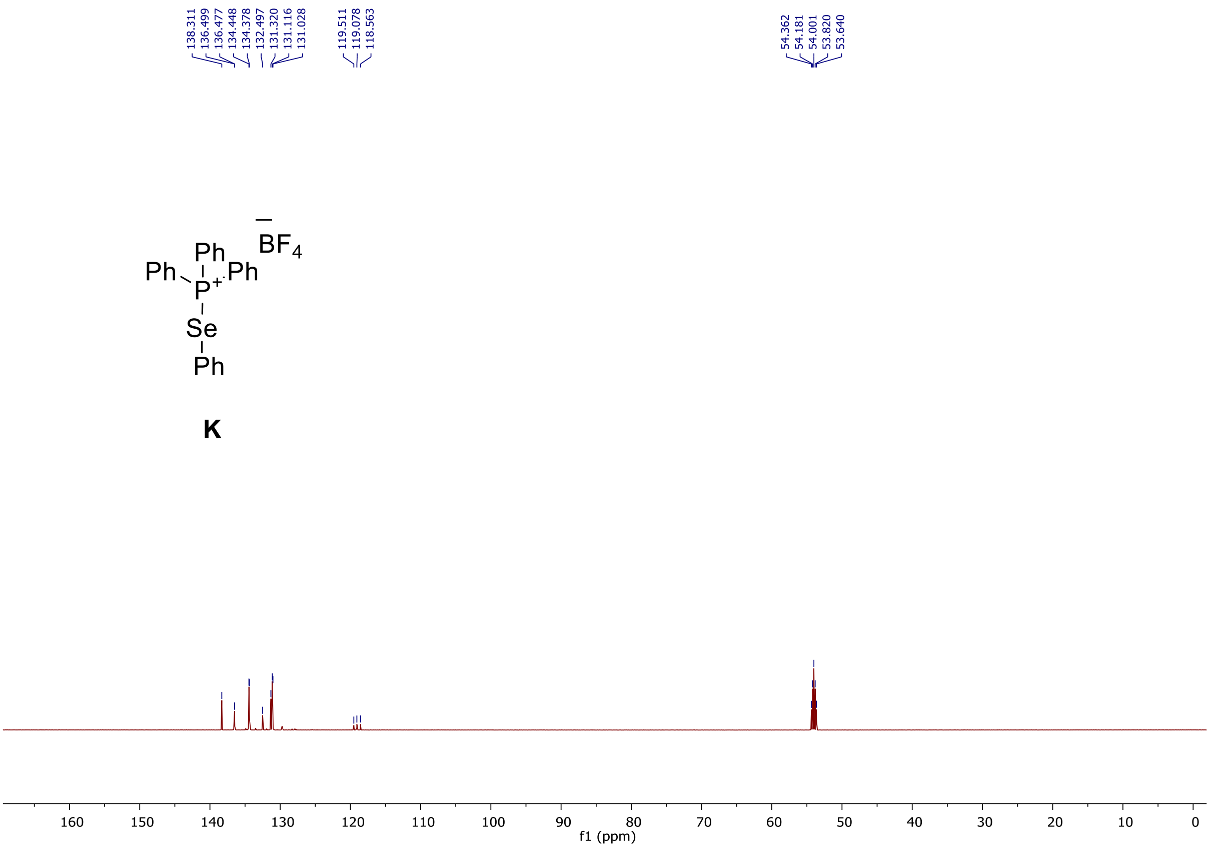

Supplementary Figure S77. ^{13}C NMR spectra for K

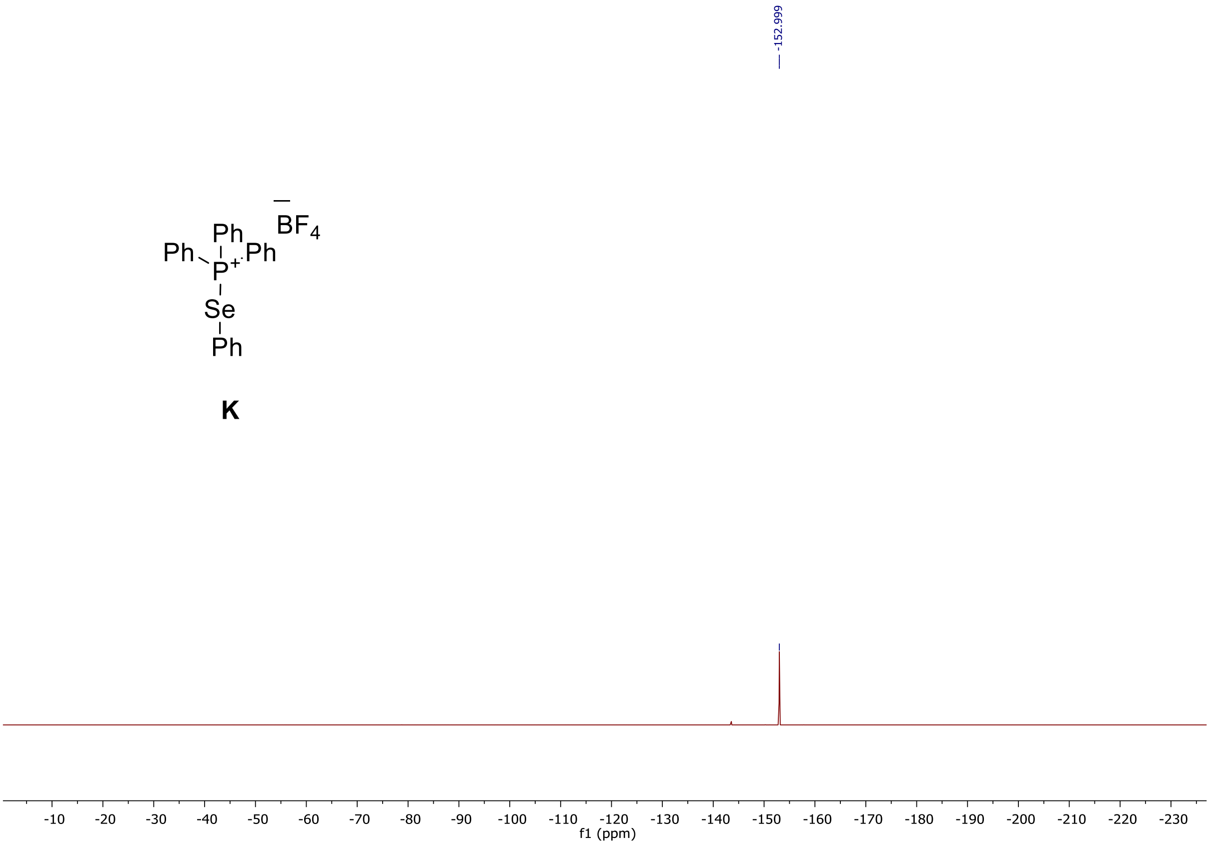

Supplementary Figure S78. ^{19}F NMR spectra for K

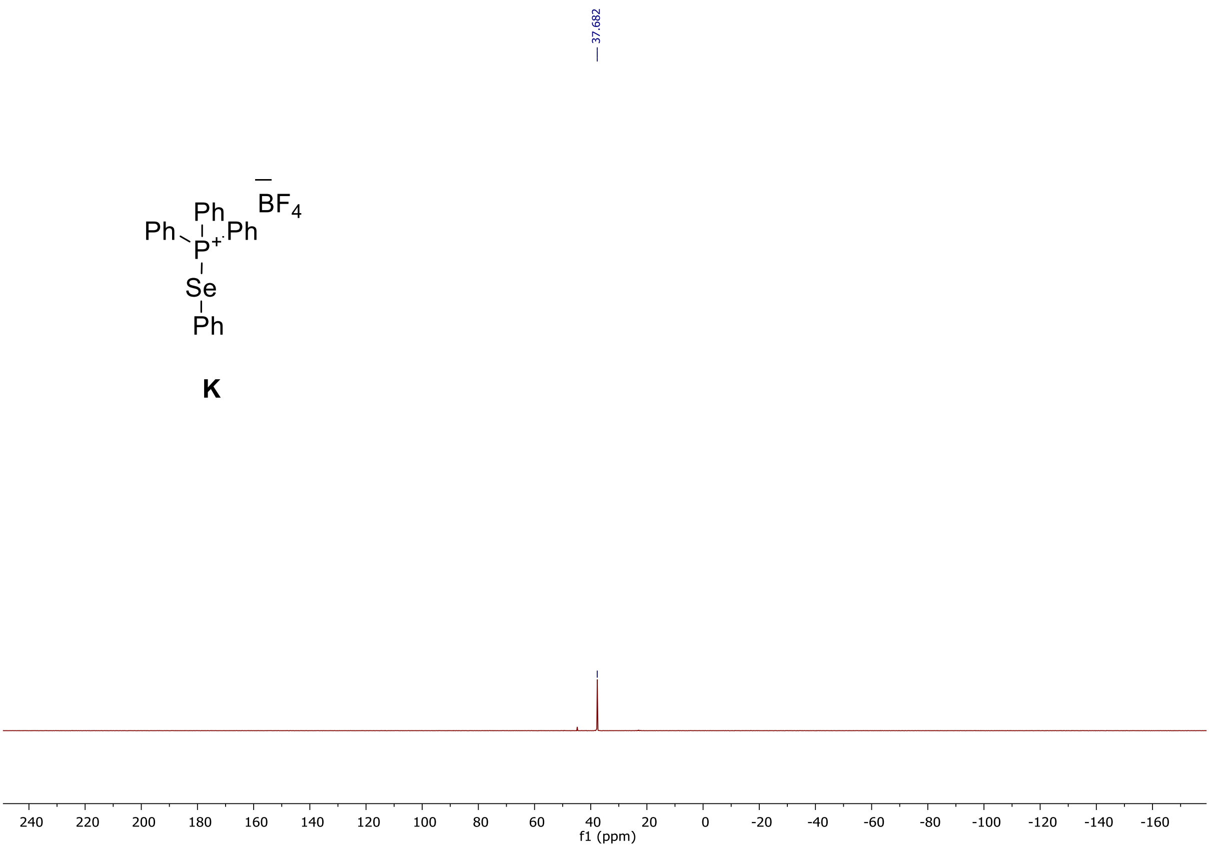

Supplementary Figure S79. ^{31}P NMR spectra for K

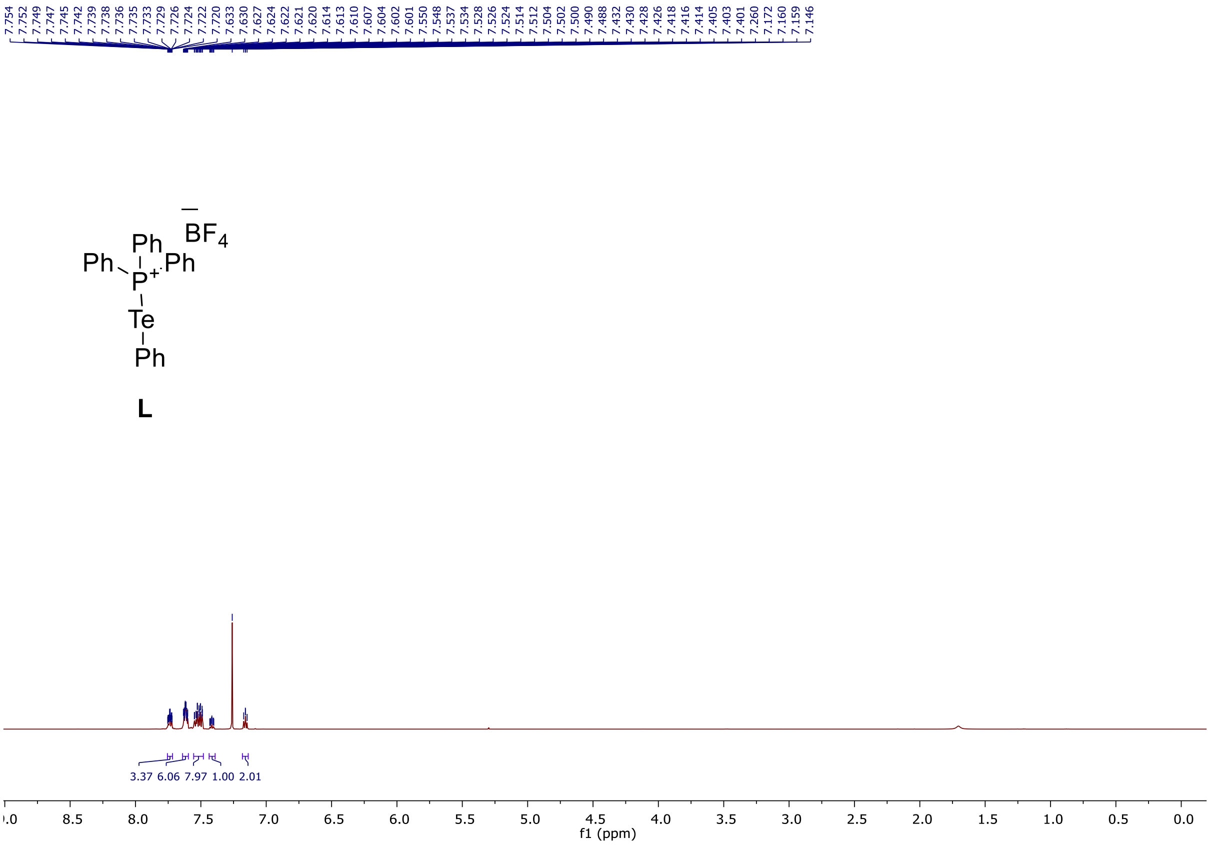

Supplementary Figure S80. ^{1}H NMR spectra for L

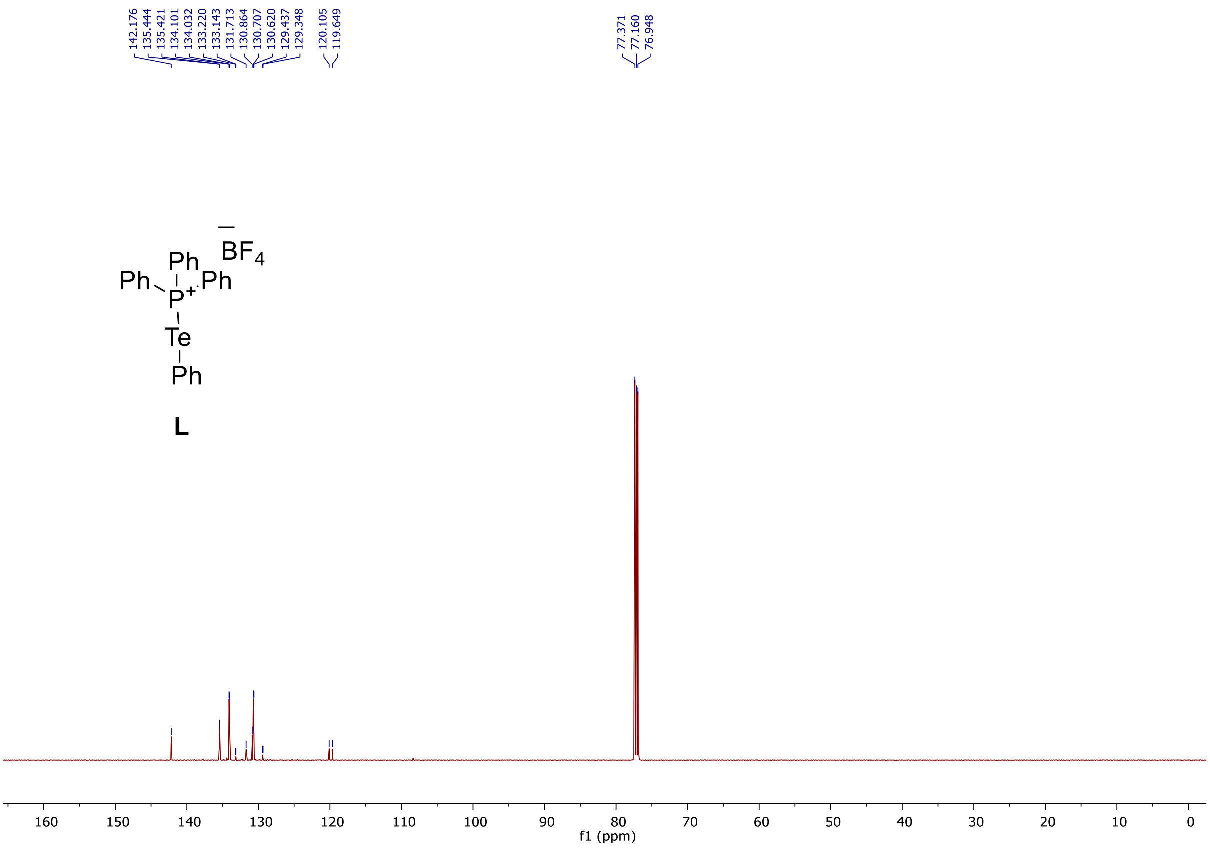

Supplementary Figure S81. ^{13}C NMR spectra for L

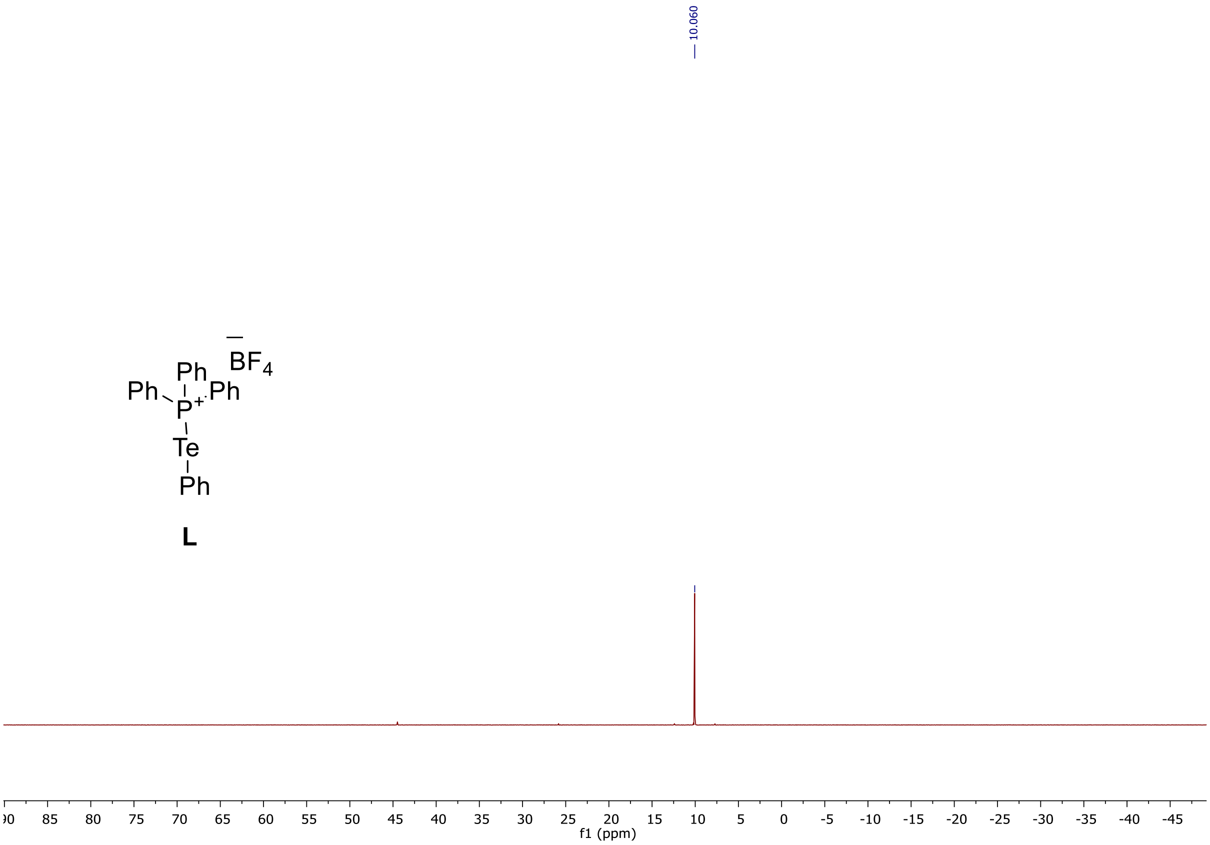

Supplementary Figure 82. ^{31}P NMR spectra for L

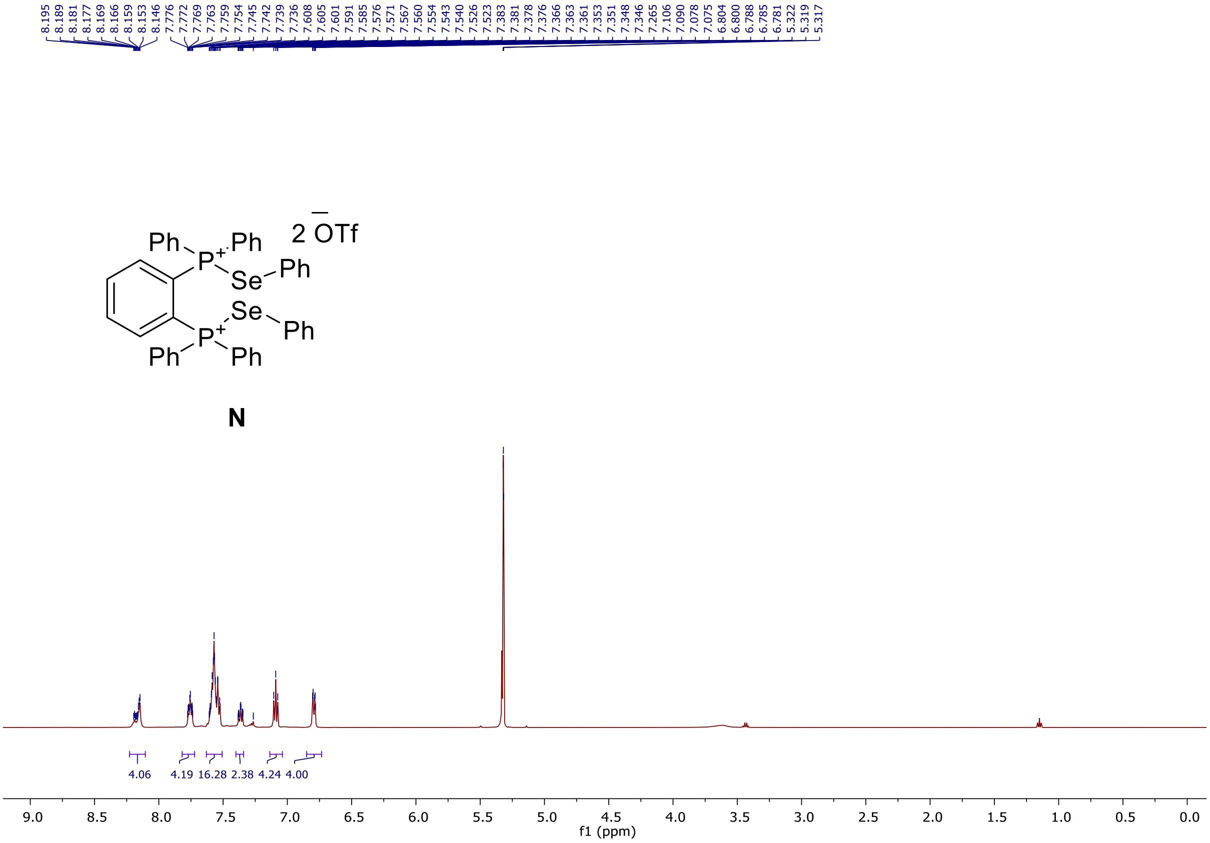

Supplementary Figure S83. ^{1}H **NMR spectra for N**

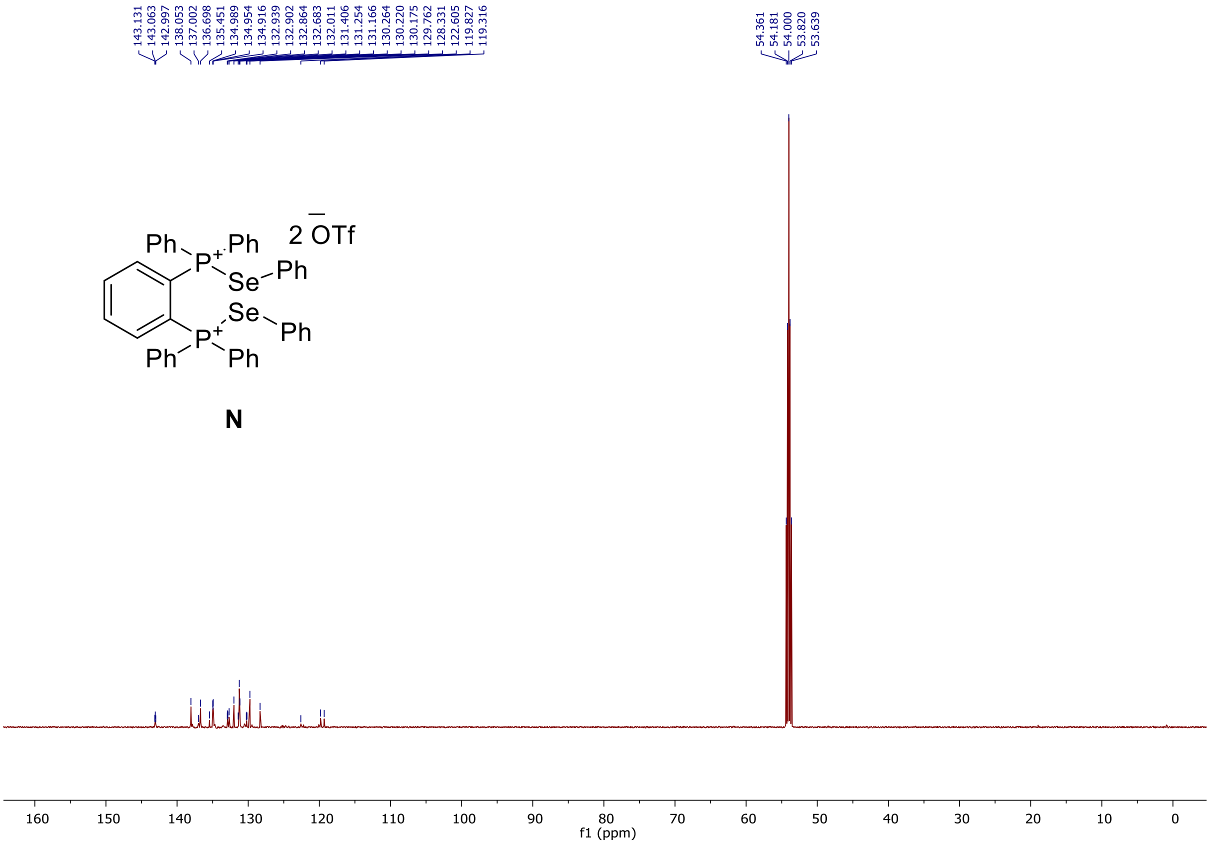

Supplementary Figure S84. ^{13}C **NMR spectra for N**

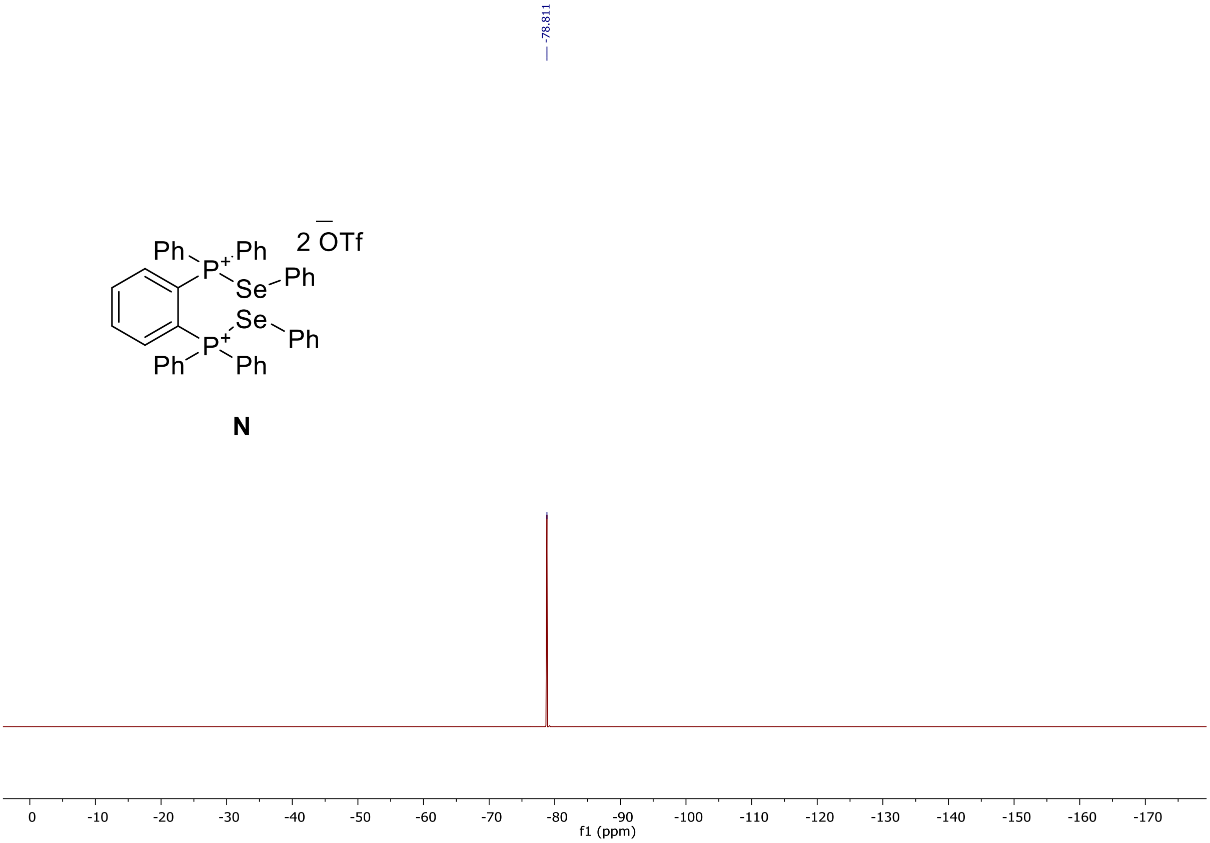

Supplementary Figure S85. ^{19}F NMR spectra for N

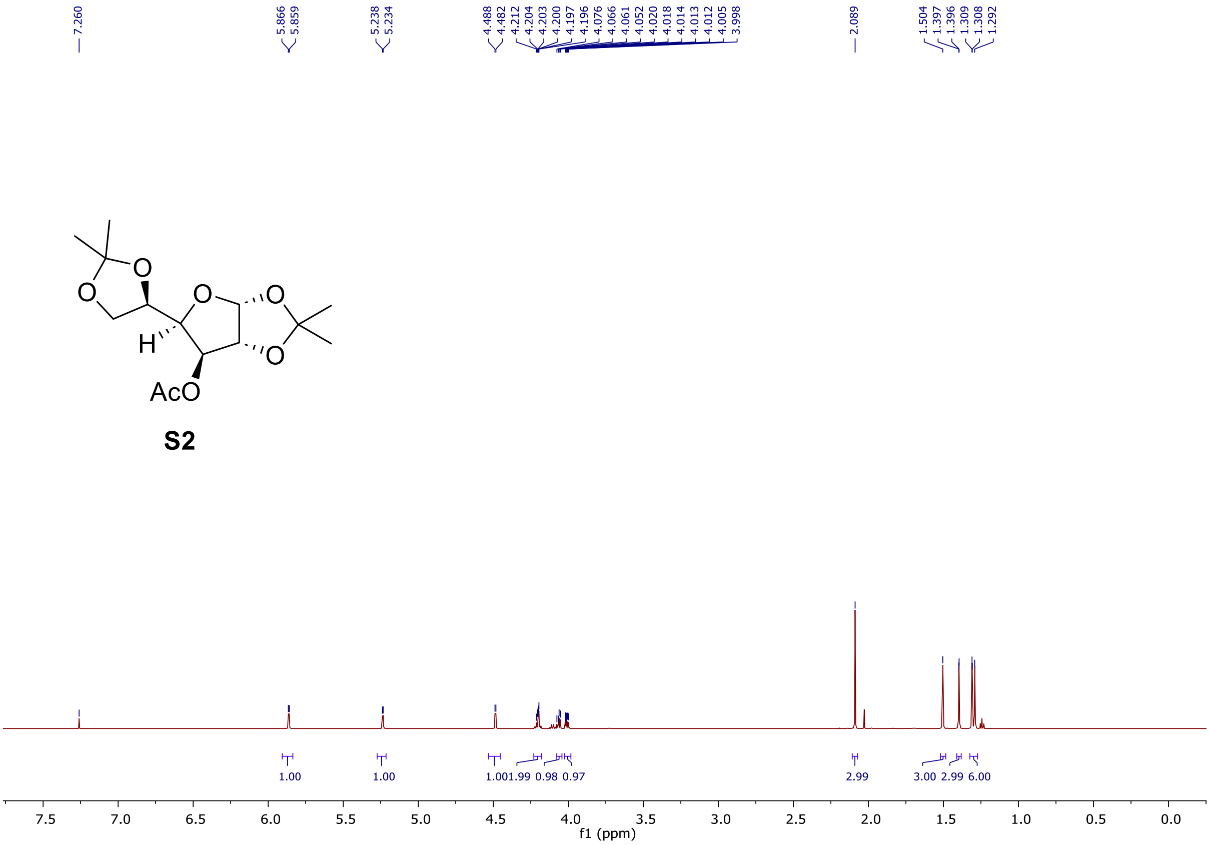

Supplementary Figure S86. ^{1}H NMR spectra for S2

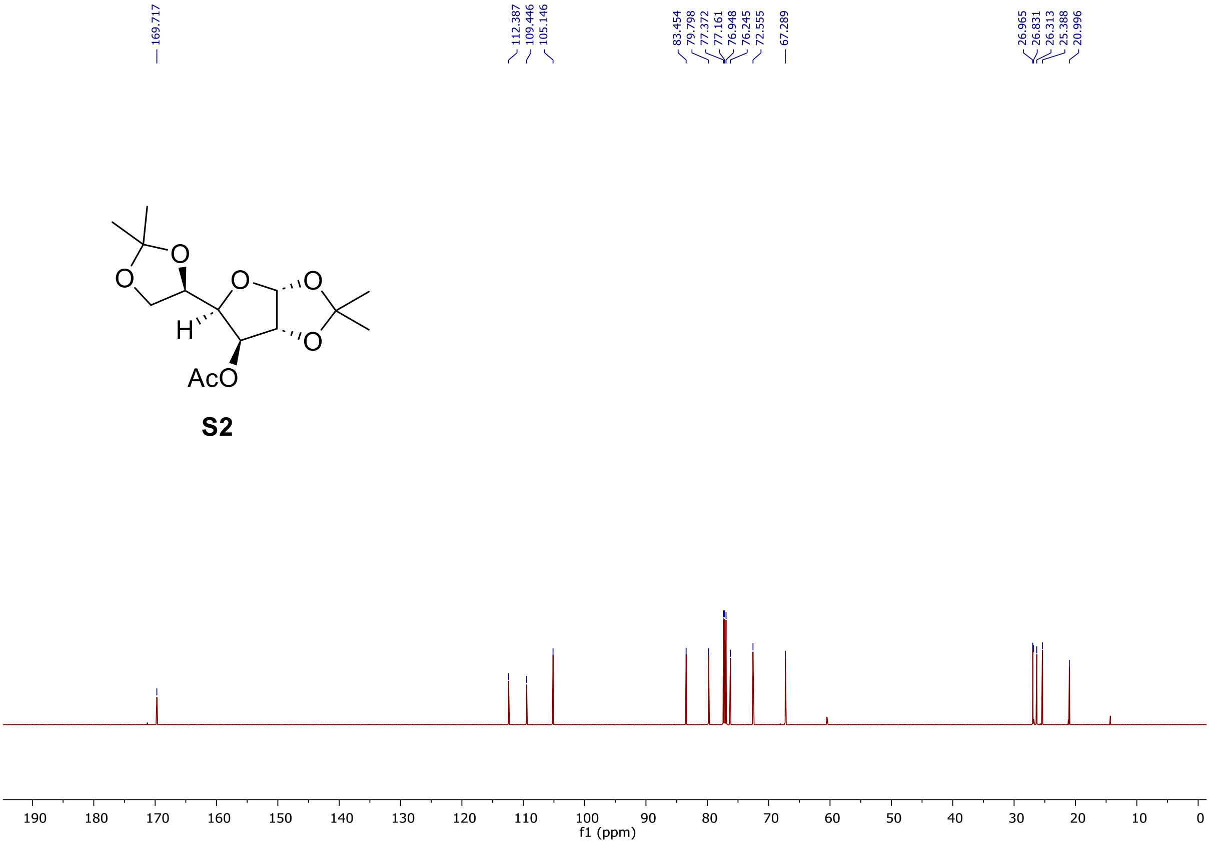

Supplementary Figure S87. ^{13}C NMR spectra for S2

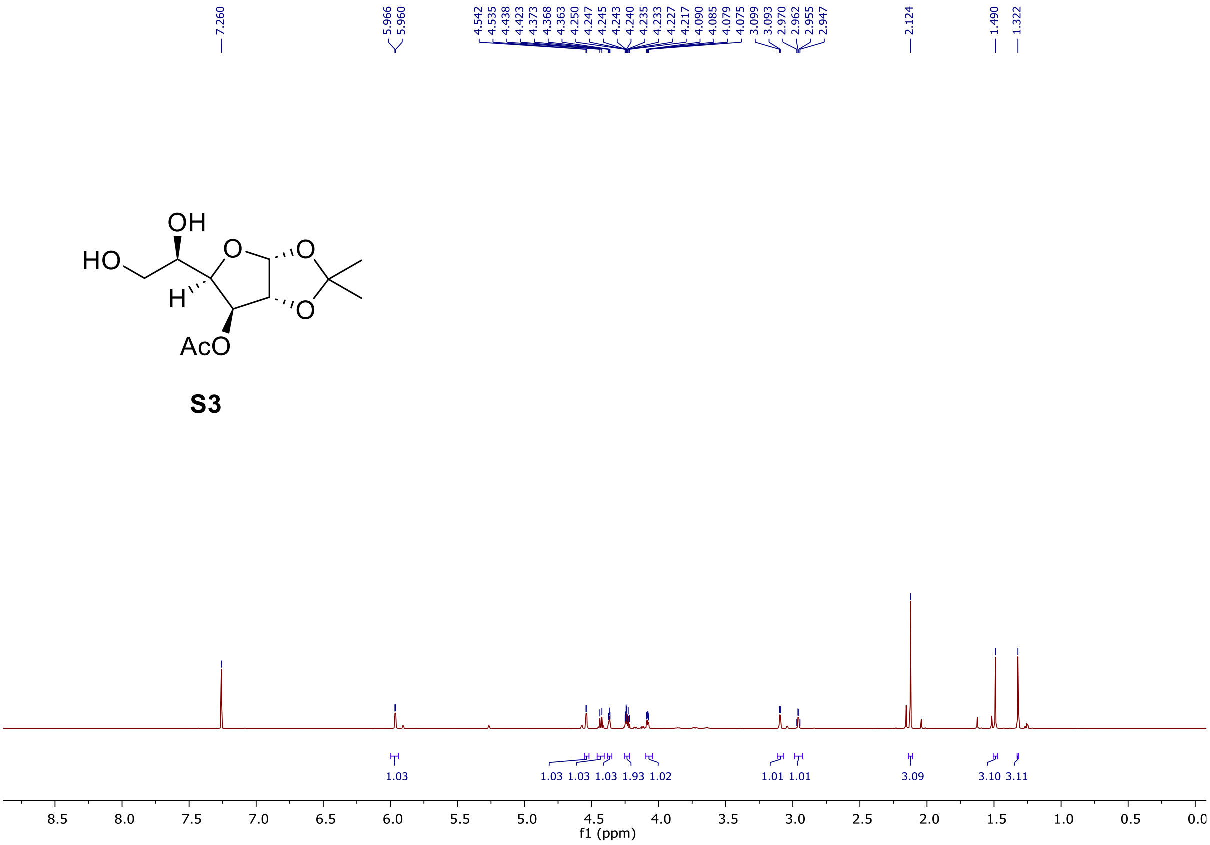

Supplementary Figure S88. ^{1}H NMR spectra for S3

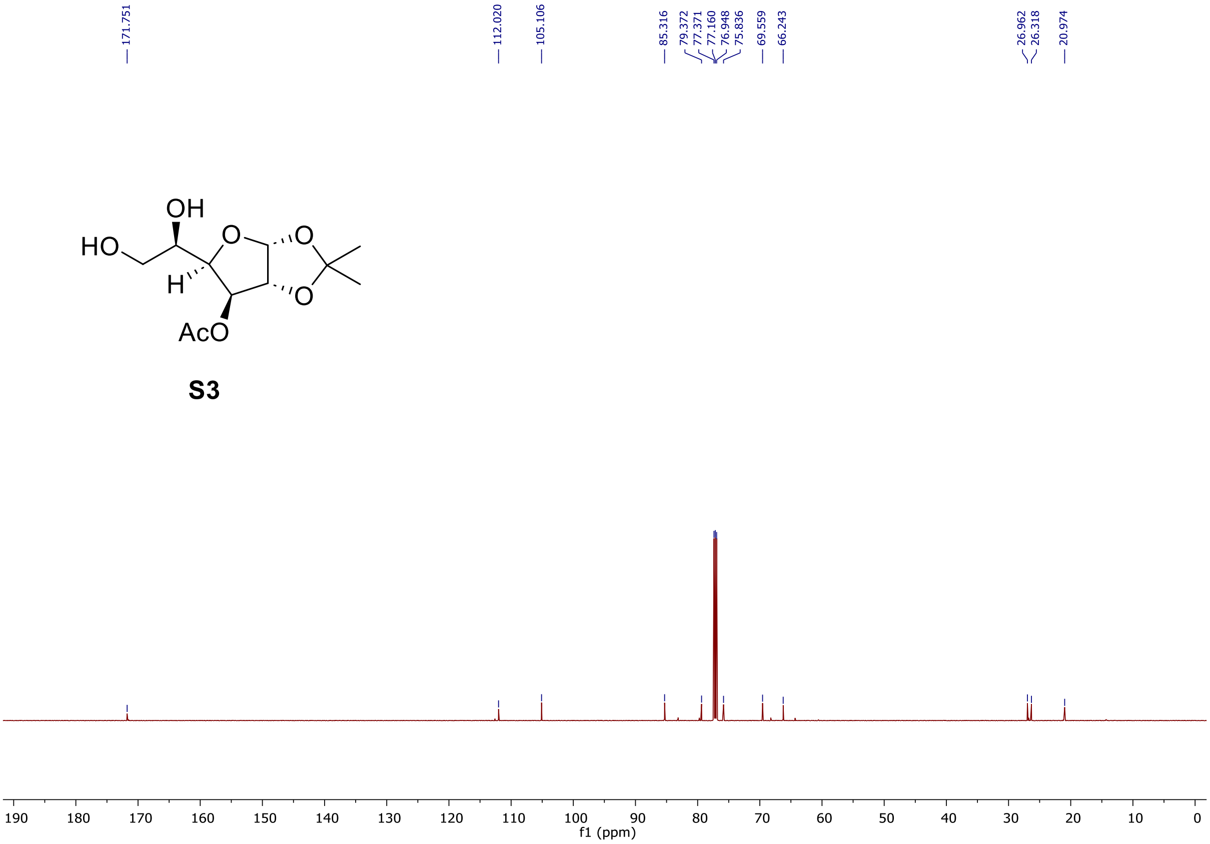

Supplementary Figure S89. ^{13}C NMR spectra for S3

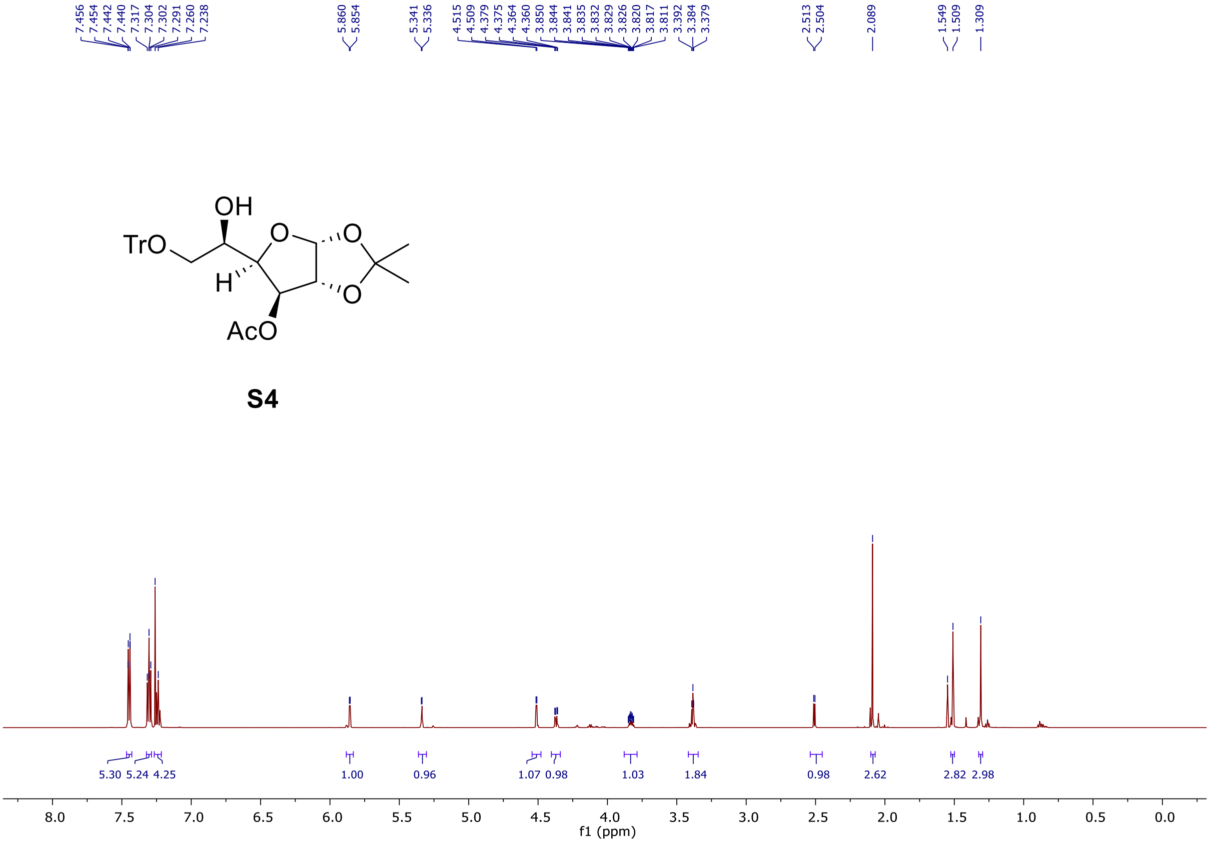

Supplementary Figure S90. ^{1}H NMR spectra for S4

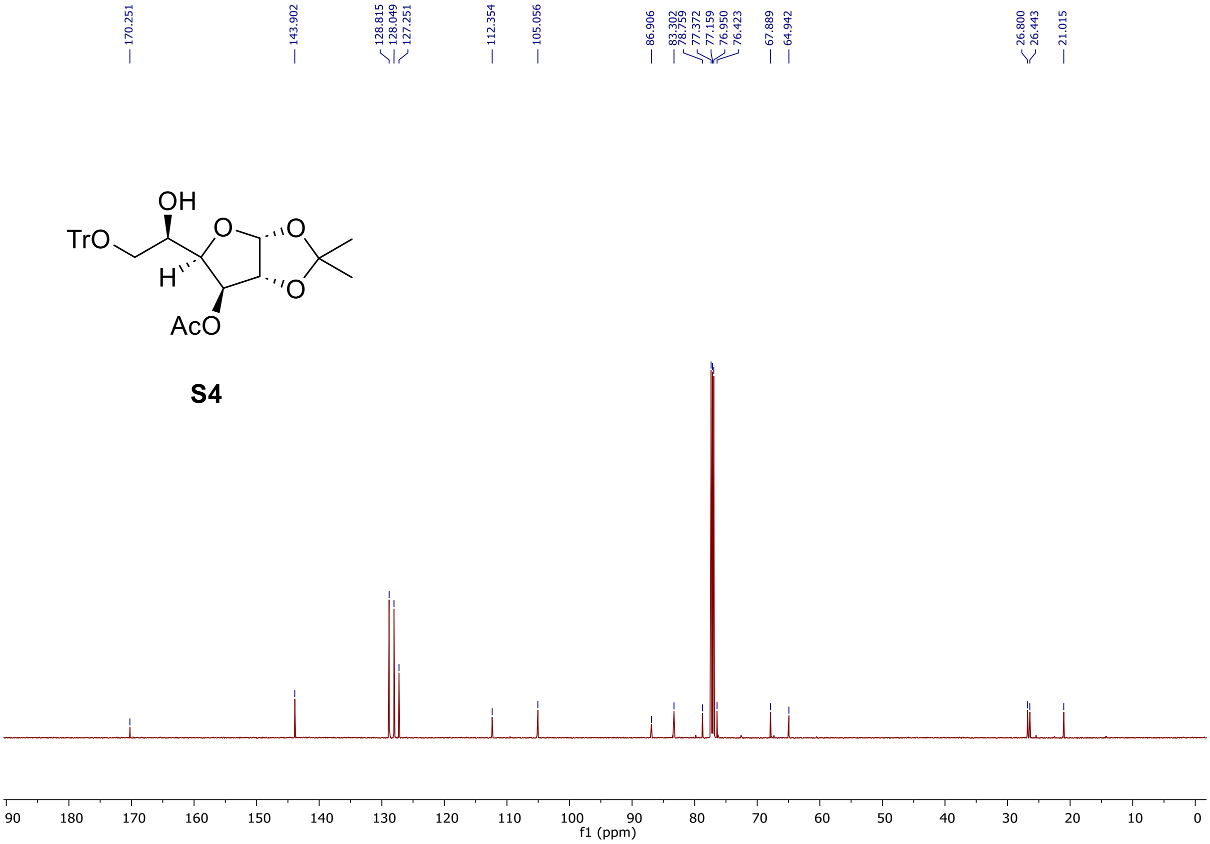

Supplementary Figure S91. ^{13}C NMR spectra for S4

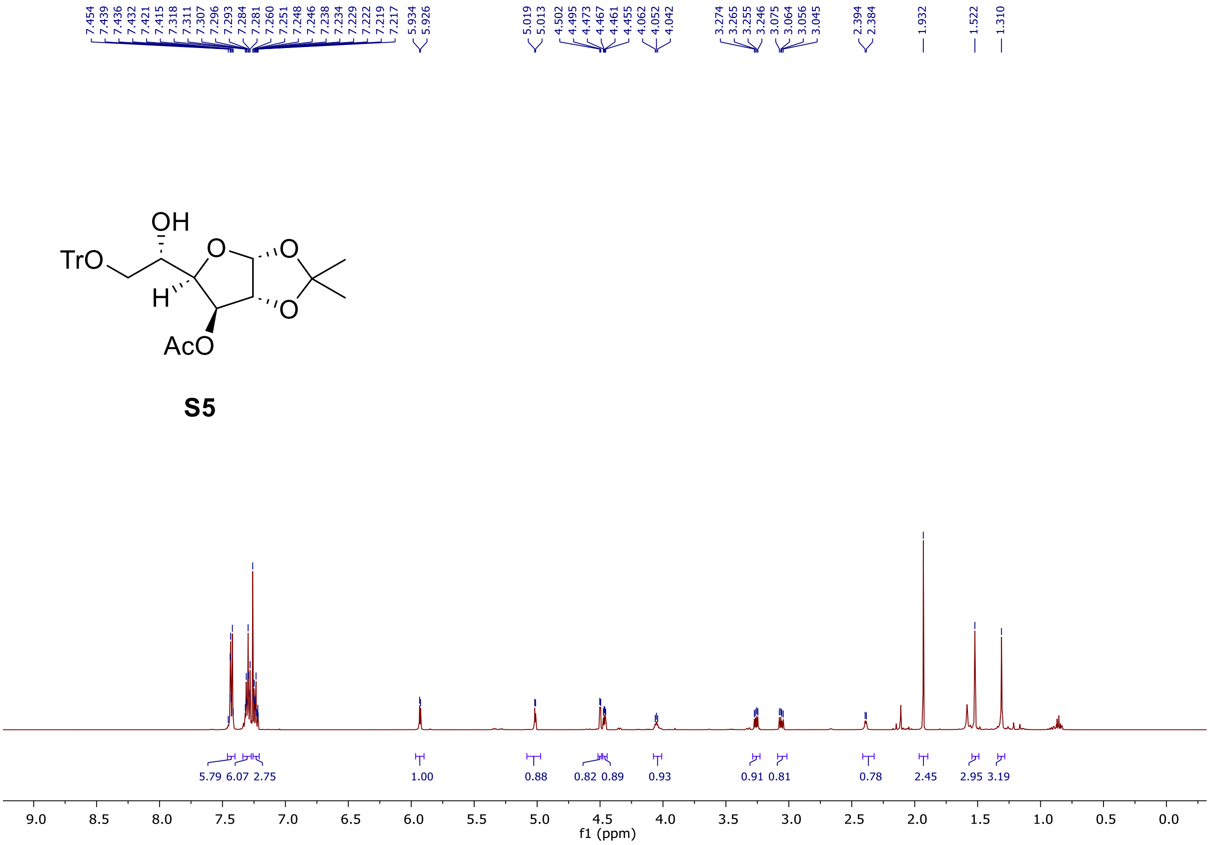

Supplementary Figure S92. ^{1}H NMR spectra for S5

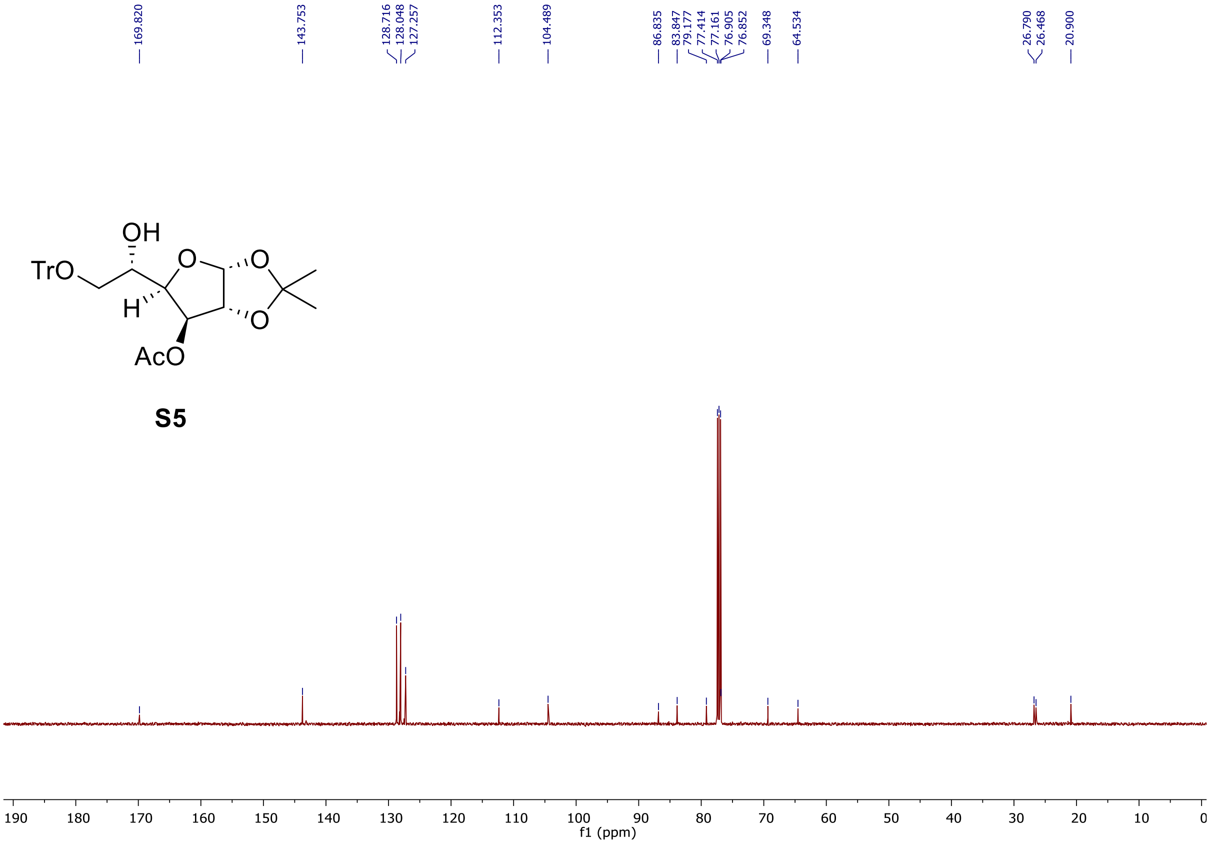

Supplementary Figure S93. ^{13}C NMR spectra for S5

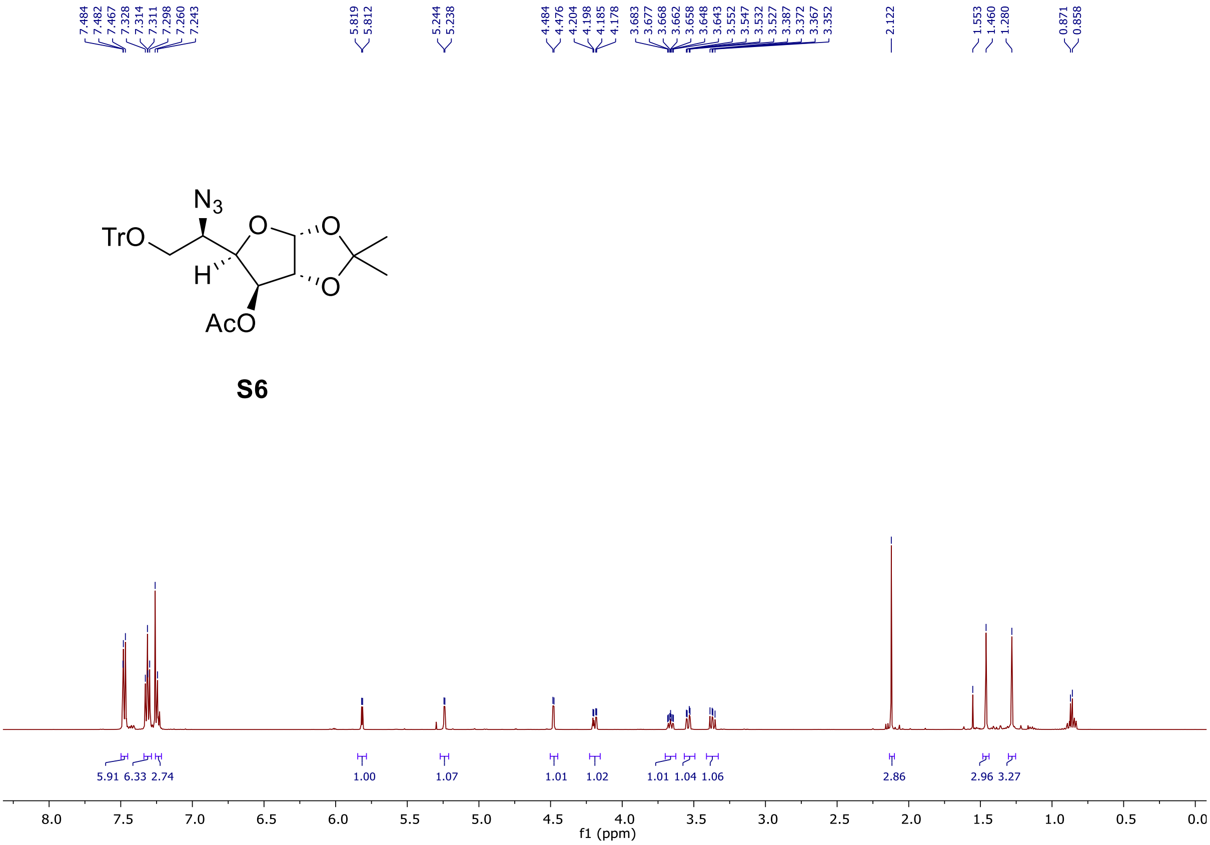

Supplementary Figure S94. ^{1}H NMR spectra for S6

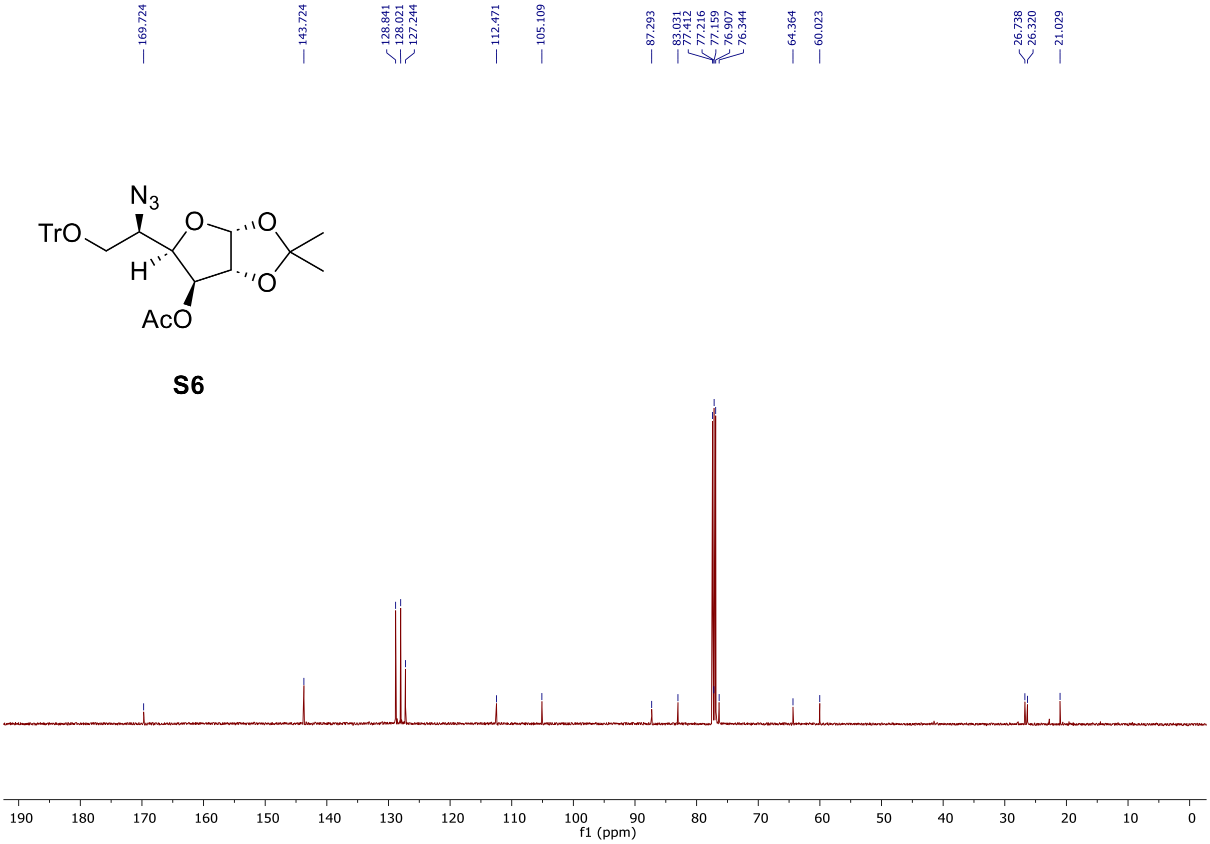

Supplementary Figure S95. ^{13}C NMR spectra for S6

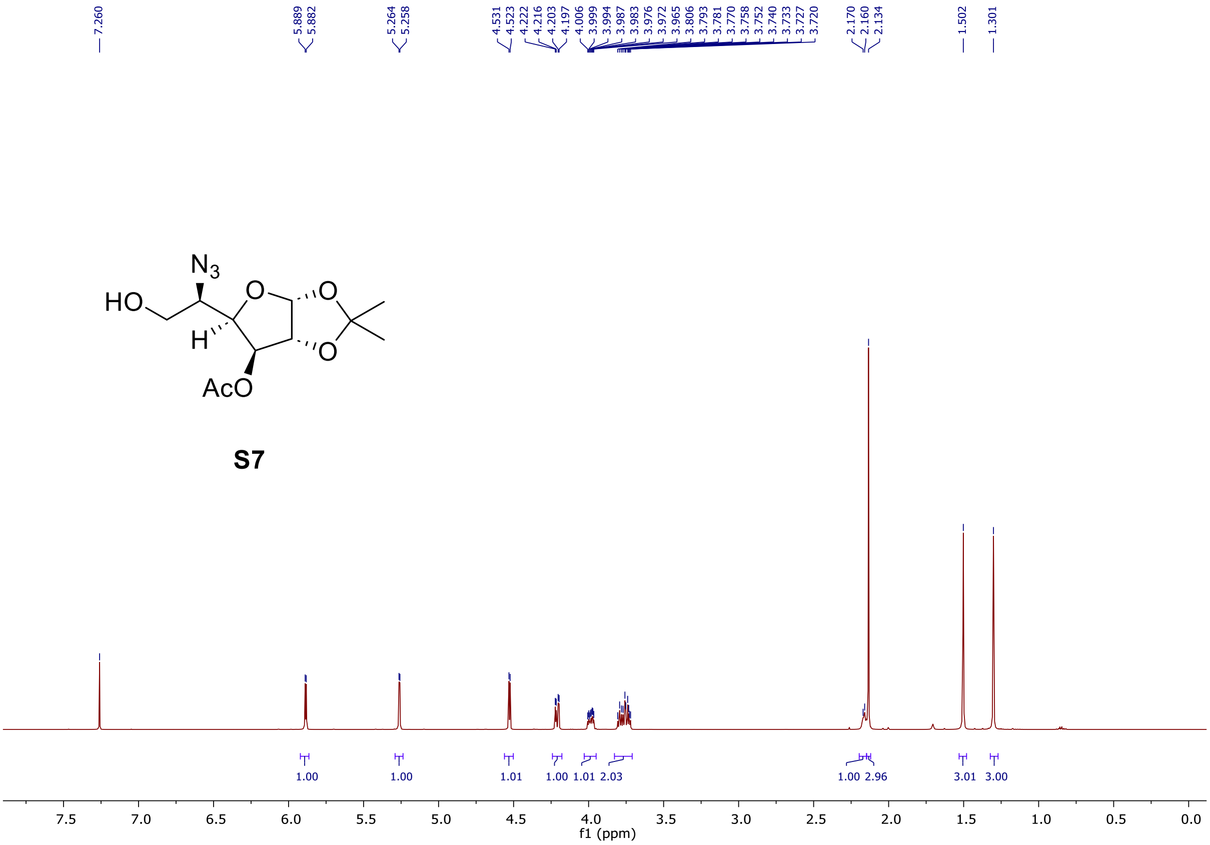

Supplementary Figure S96. ^{1}H NMR spectra for S7

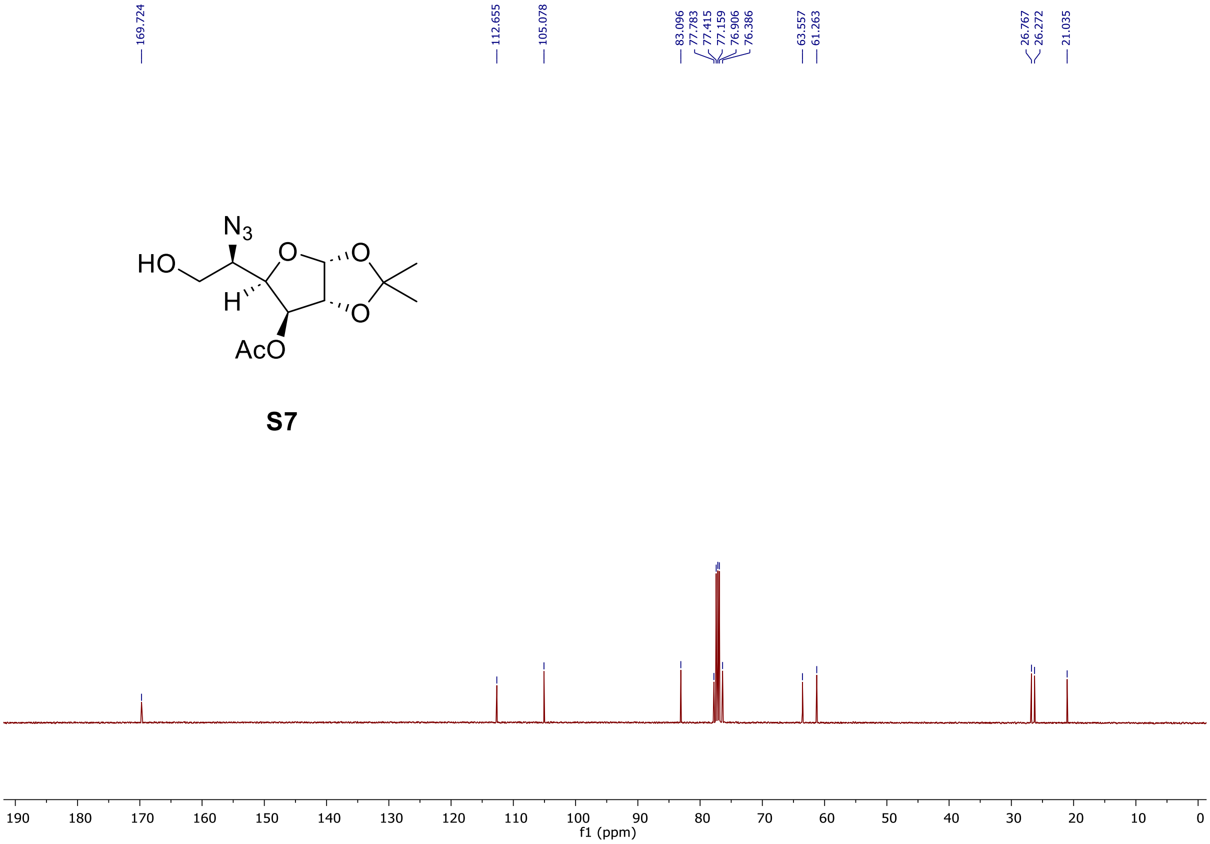

Supplementary Figure S97. ^{13}C NMR spectra for S7

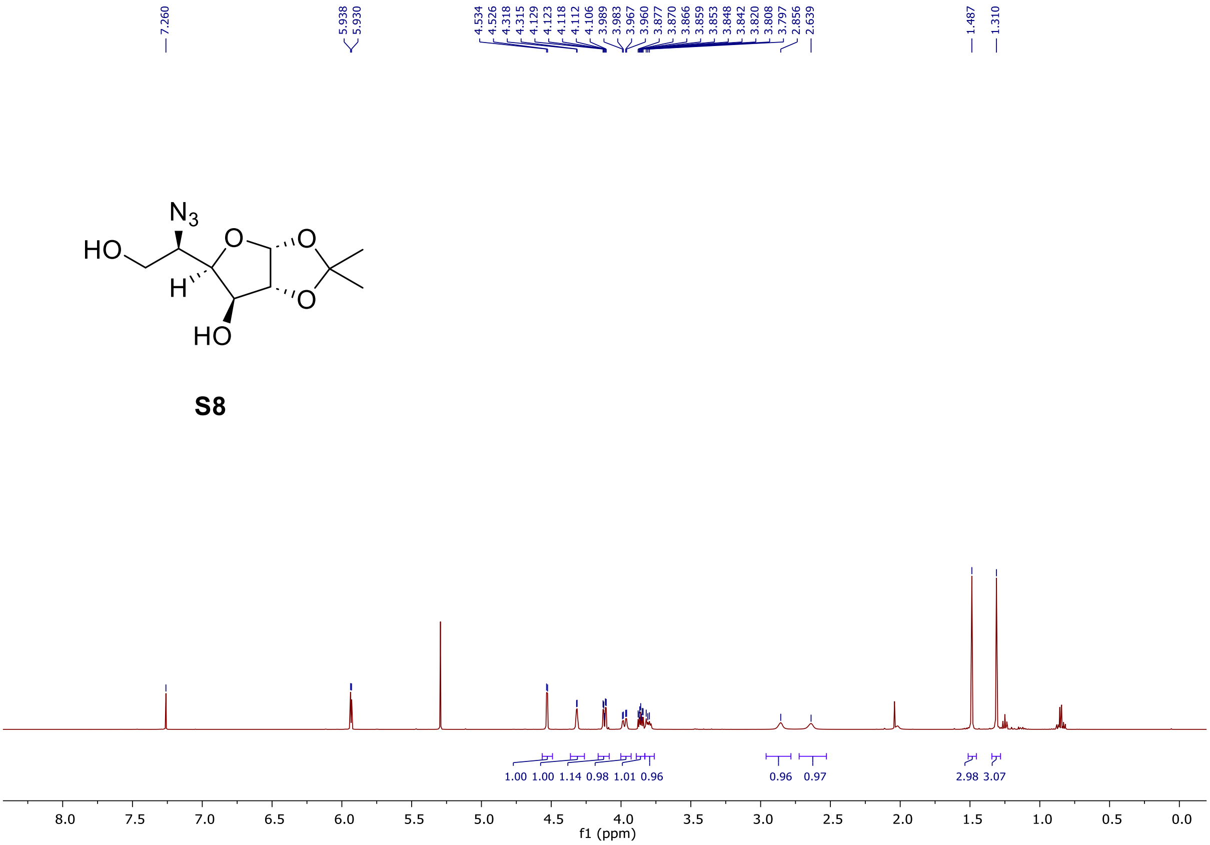

Supplementary Figure S98. ^{1}H NMR spectra for S8

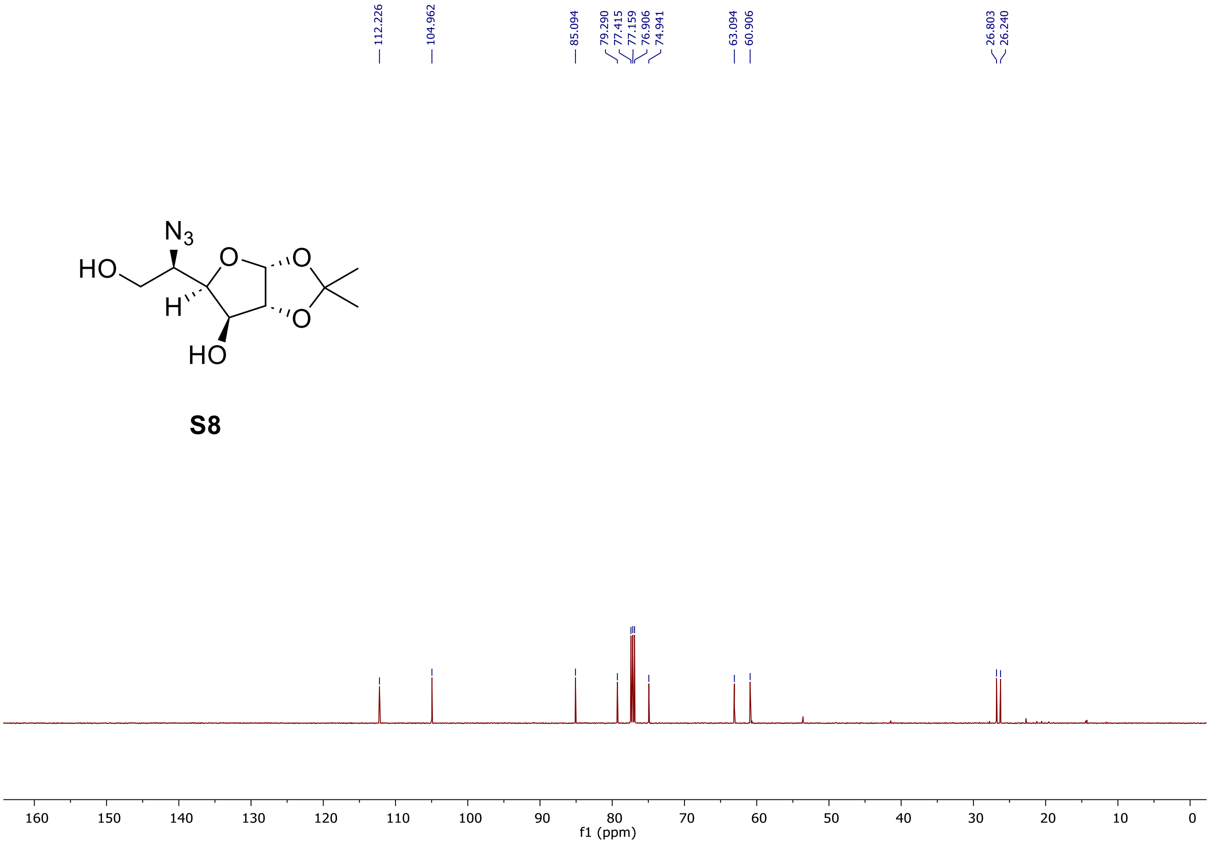

Supplementary Figure S99. ^{13}C NMR spectra for S8

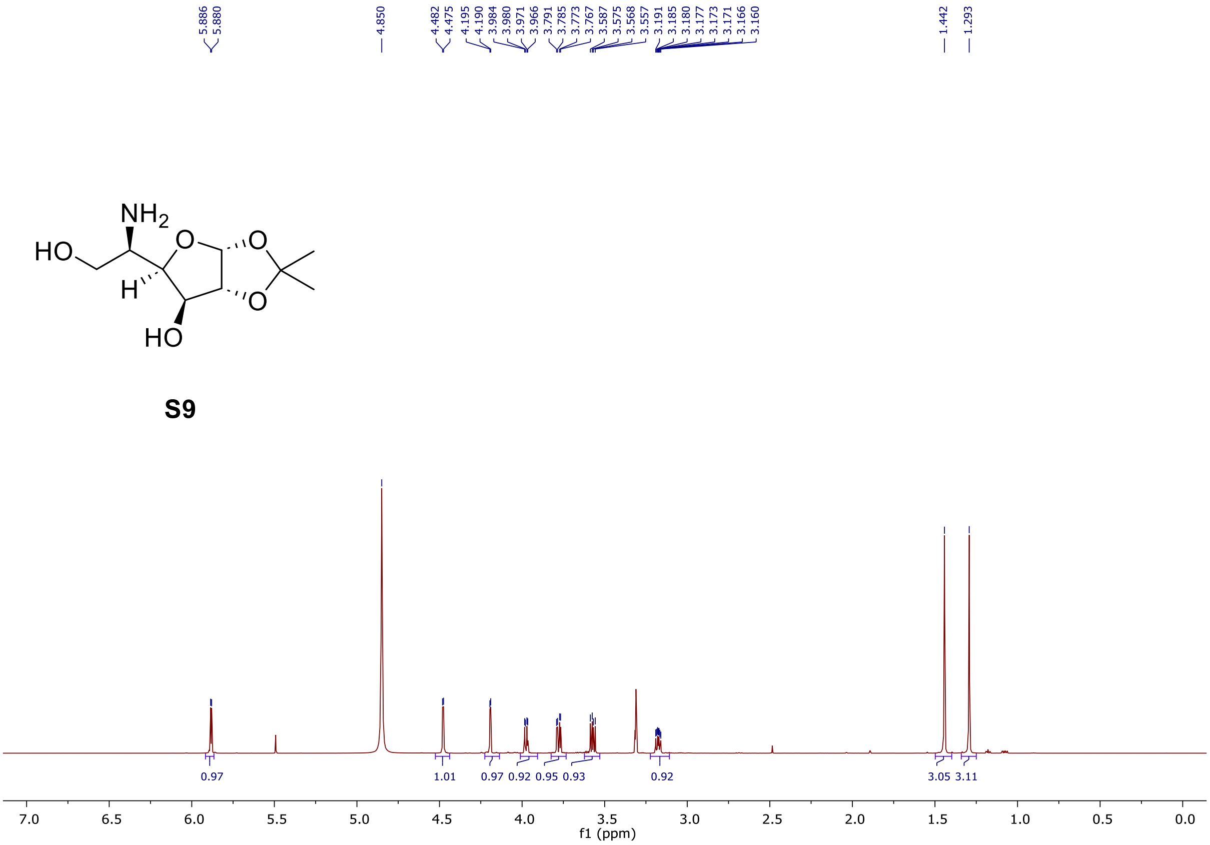

Supplementary Figure S100. ^{1}H NMR spectra for S9

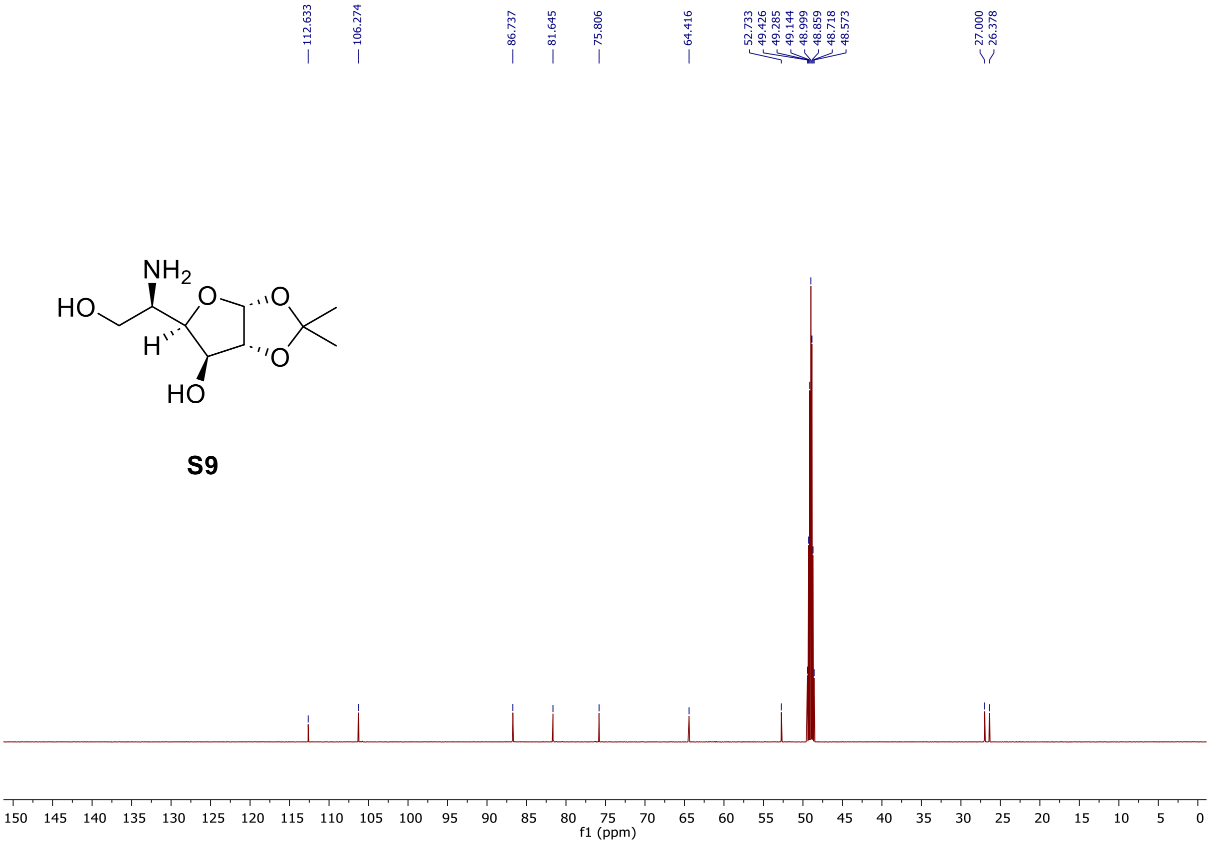

Supplementary Figure S101. ^{13}C NMR spectra for S9

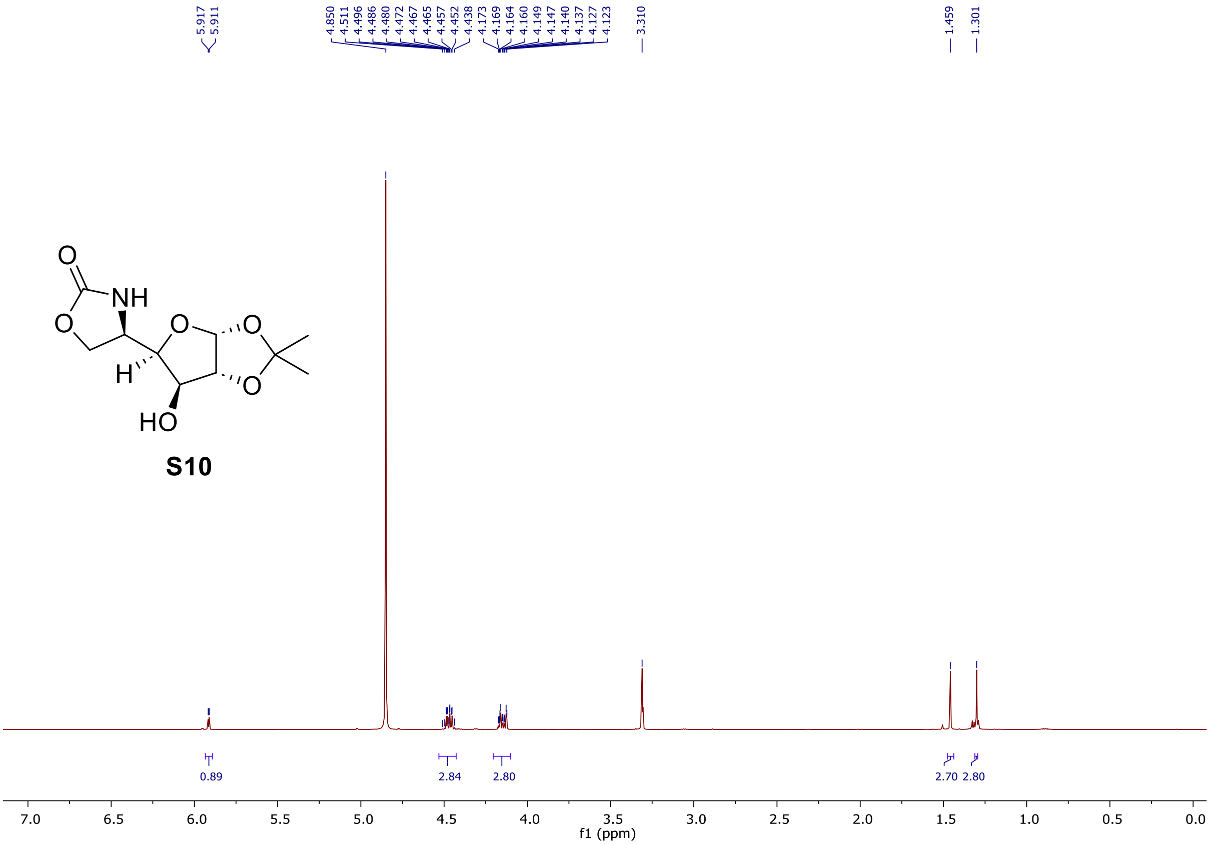

Supplementary Figure S102. ^{1}H NMR spectra for S10

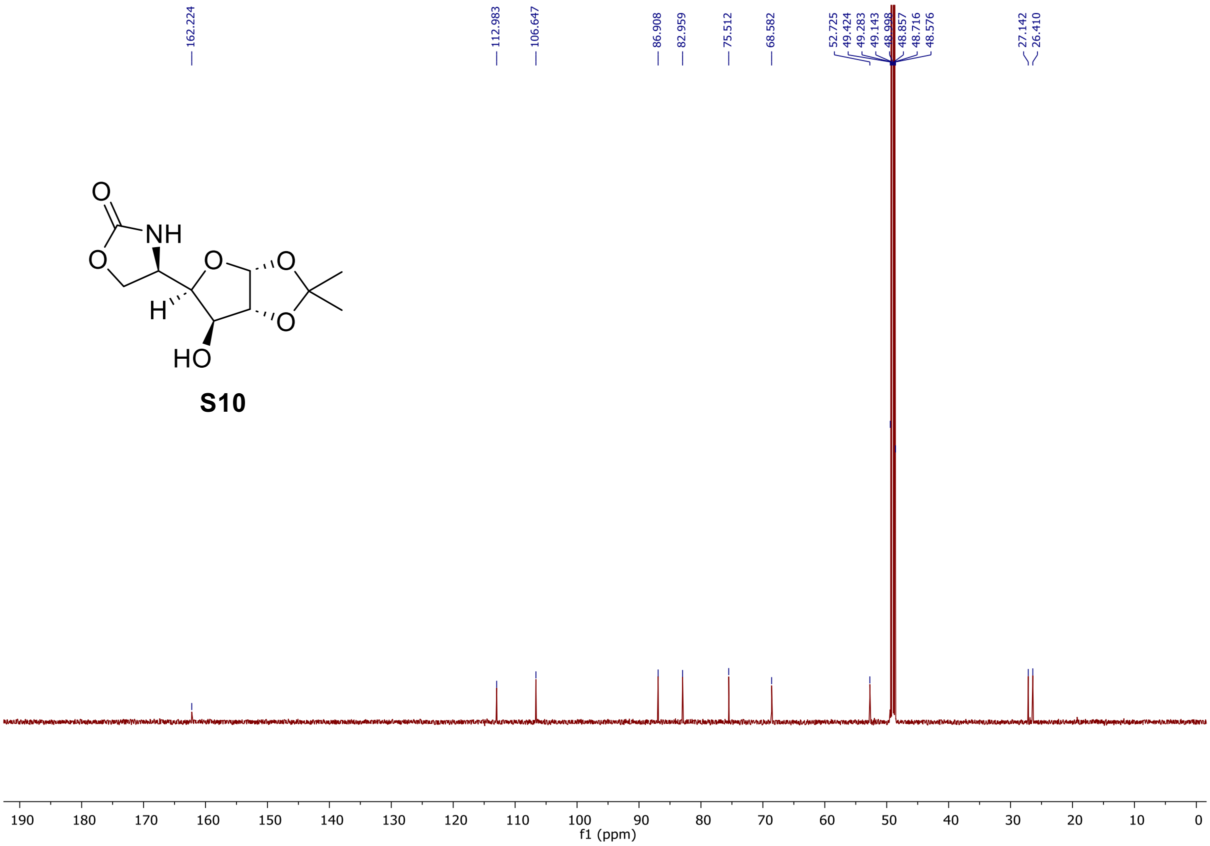

Supplementary Figure S103. ^{13}C NMR spectra for S10

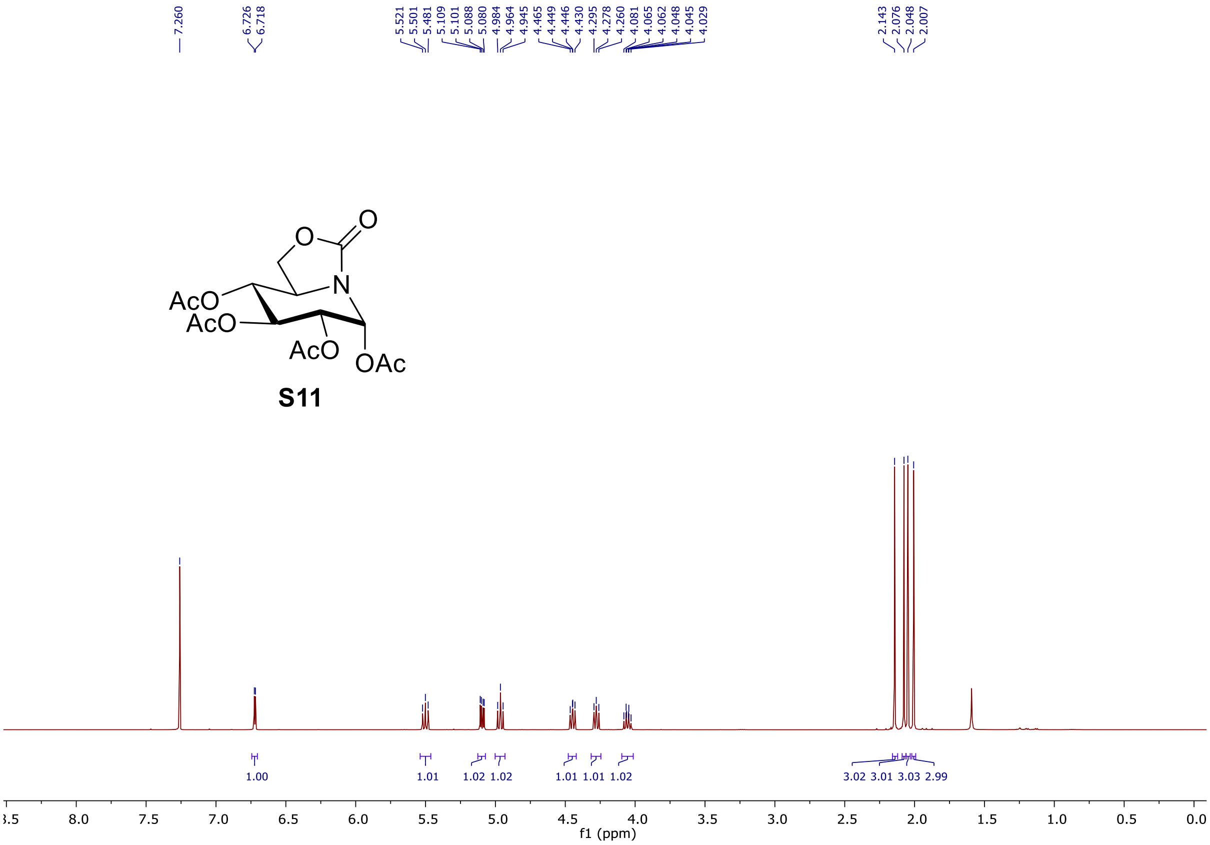

Supplementary Figure S104. ^{1}H NMR spectra for S11

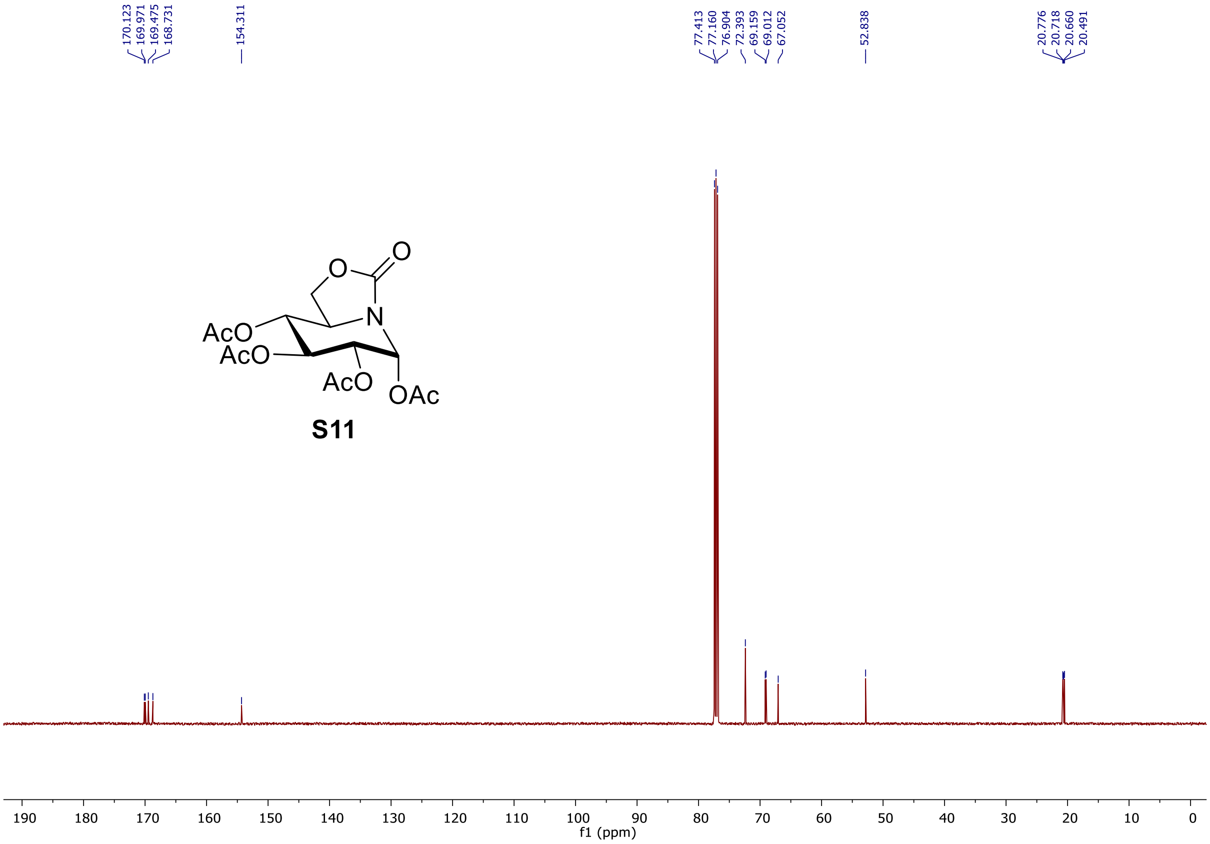

Supplementary Figure S105. ^{13}C NMR spectra for S11

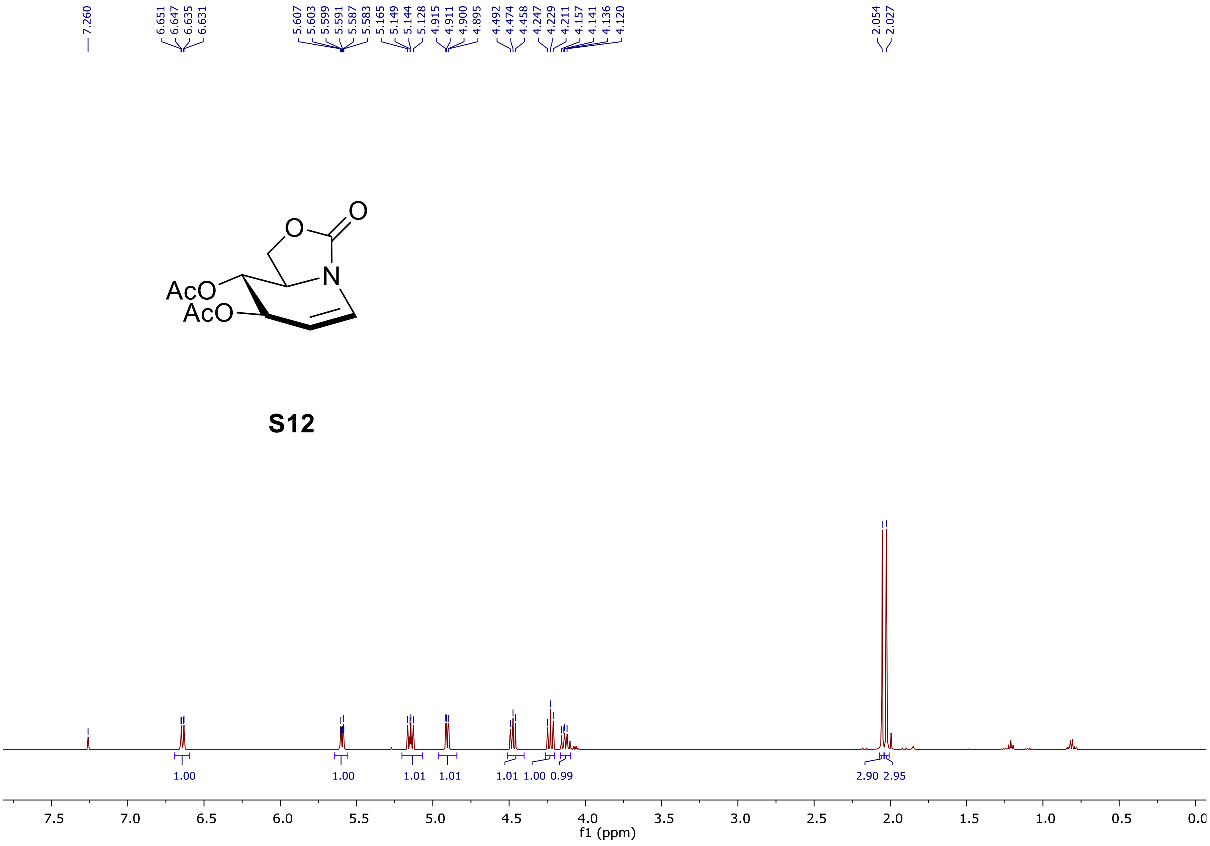

Supplementary Figure S106. ^{1}H NMR spectra for S12

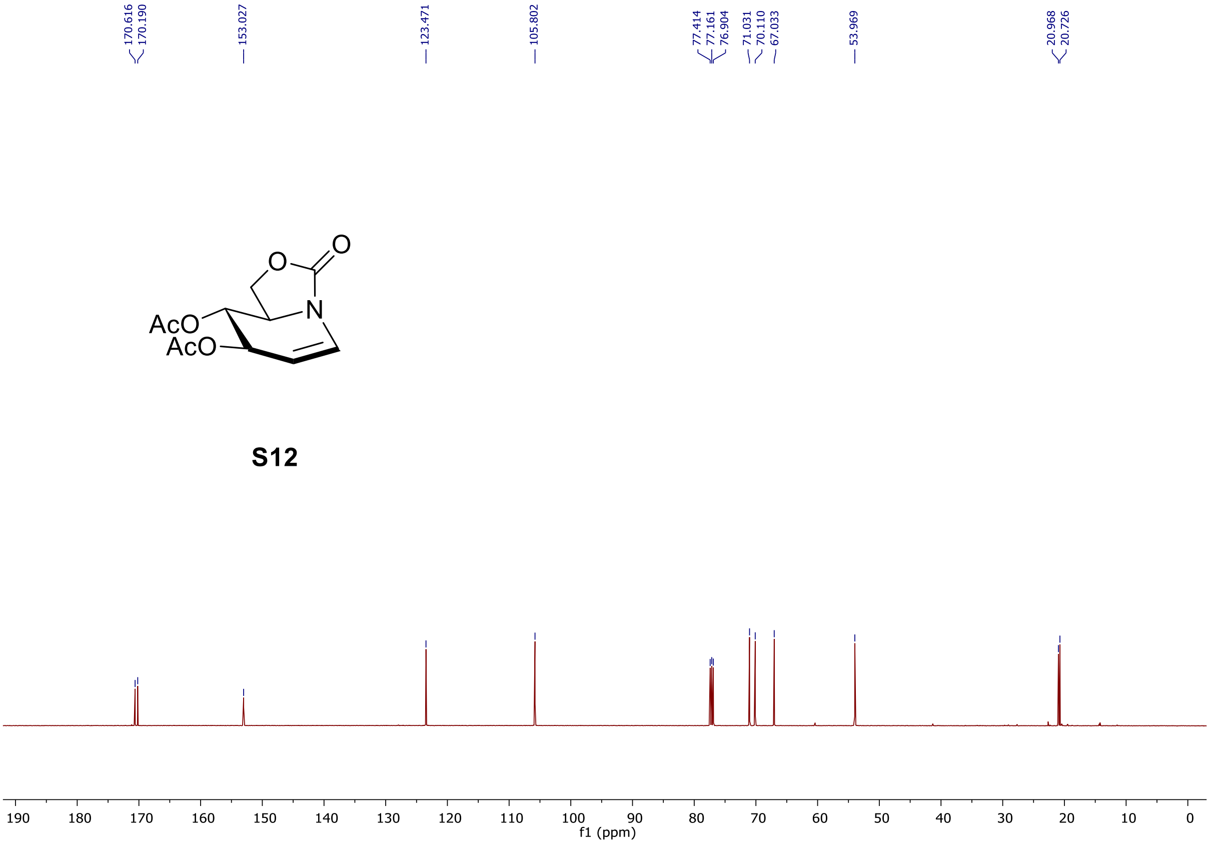

Supplementary Figure S107. ^{13}C NMR spectra for S12

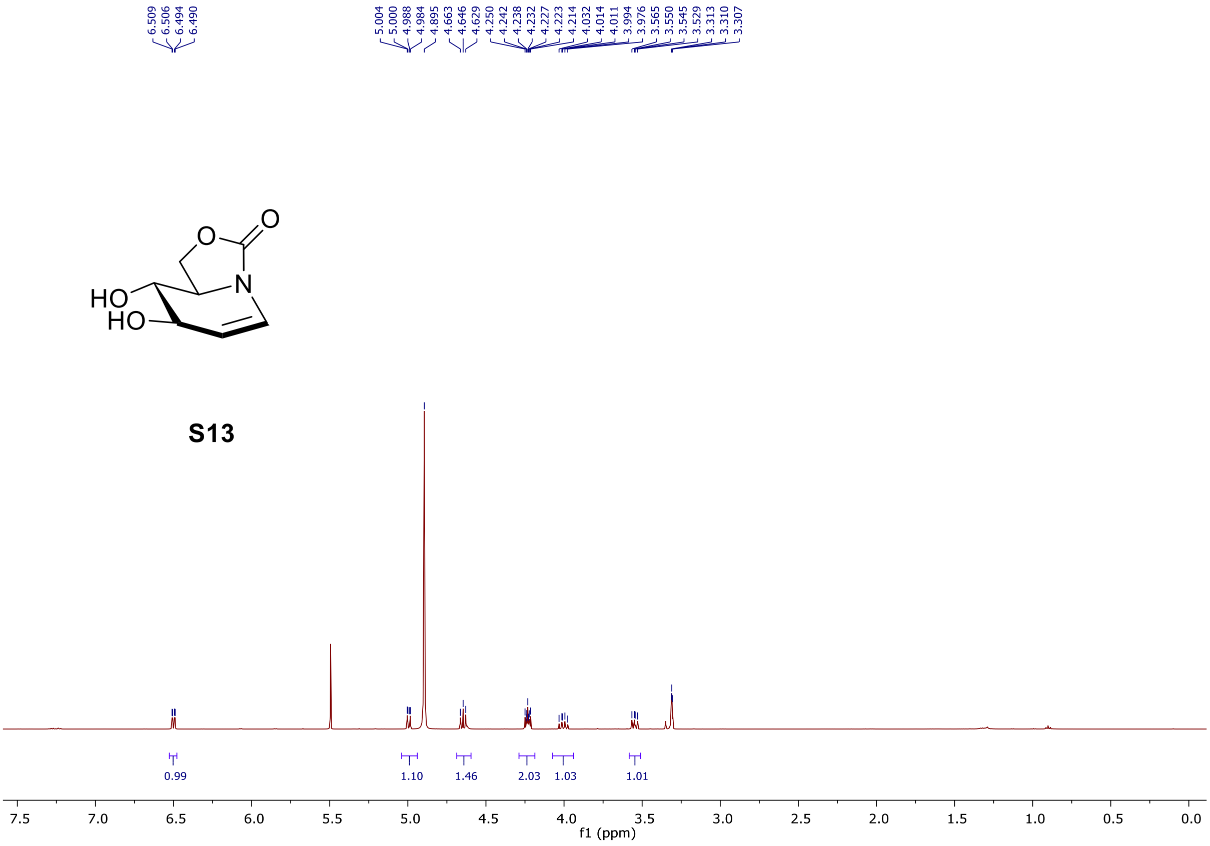

Supplementary Figure S108. ^{1}H NMR spectra for S13

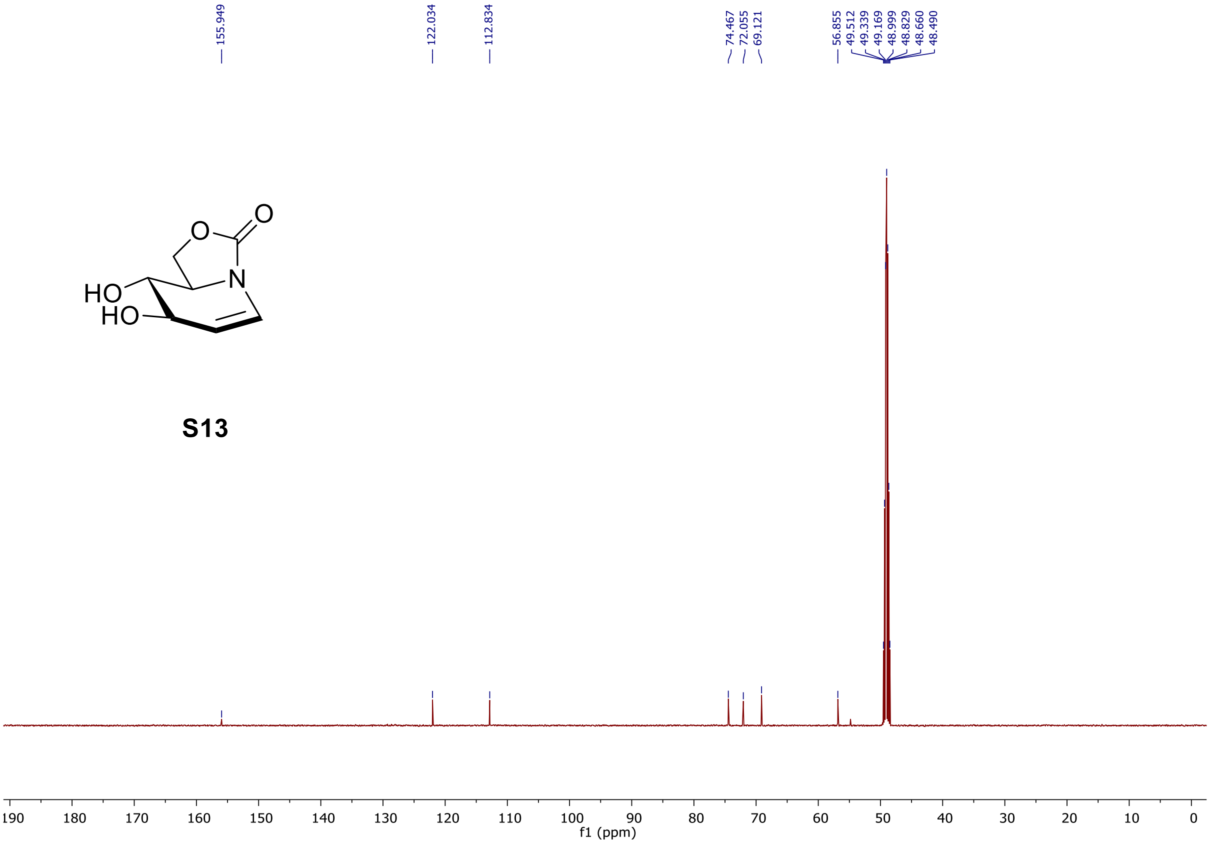

Supplementary Figure S109. ^{13}C NMR spectra for S13

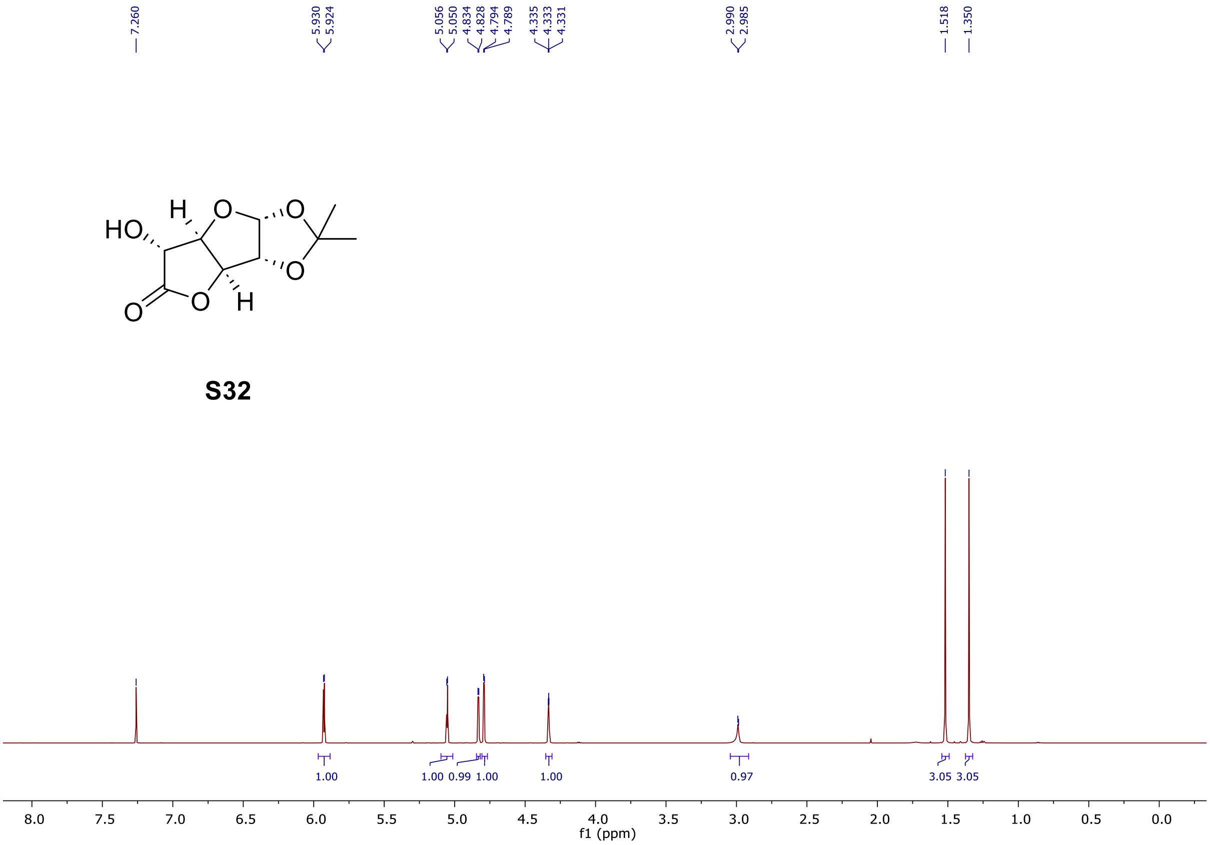

Supplementary Figure S110. ^{1}H NMR spectra for S32

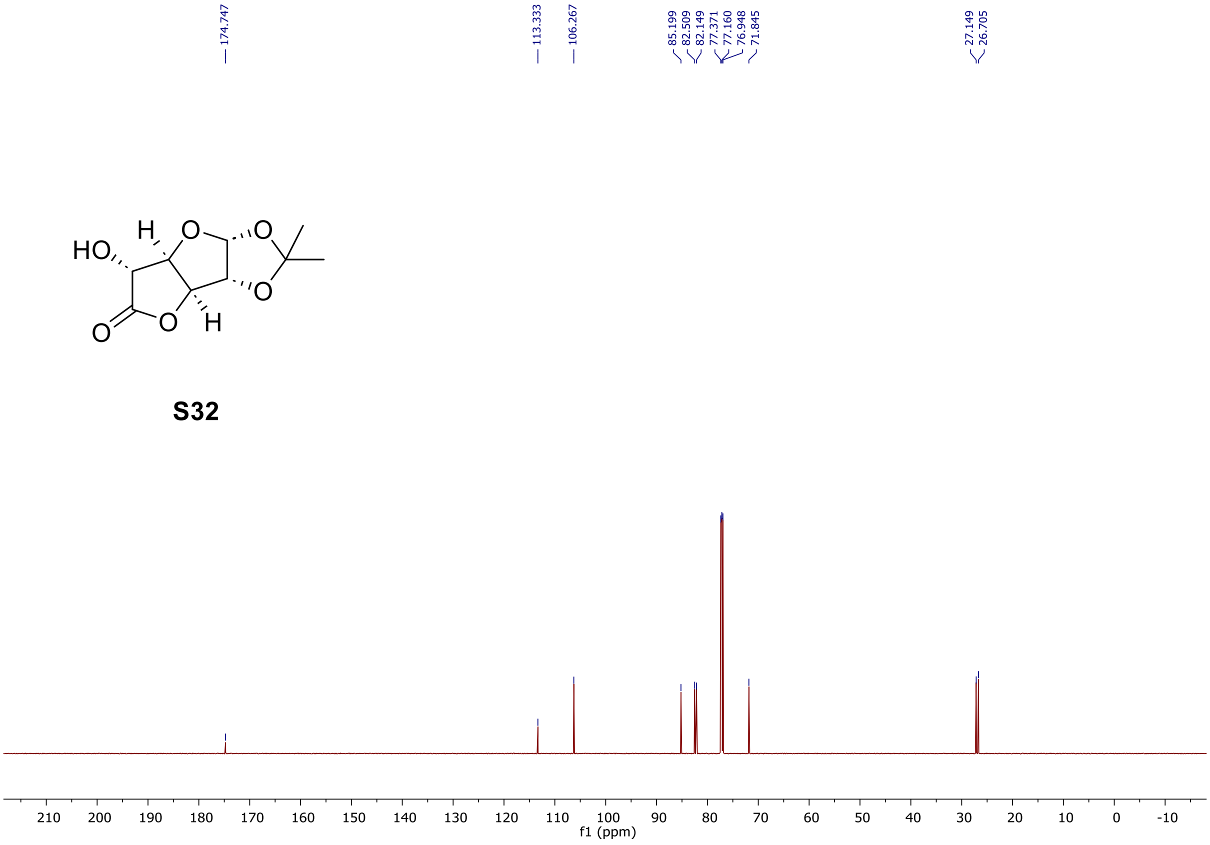

Supplementary Figure S111. ^{13}C NMR spectra for S32

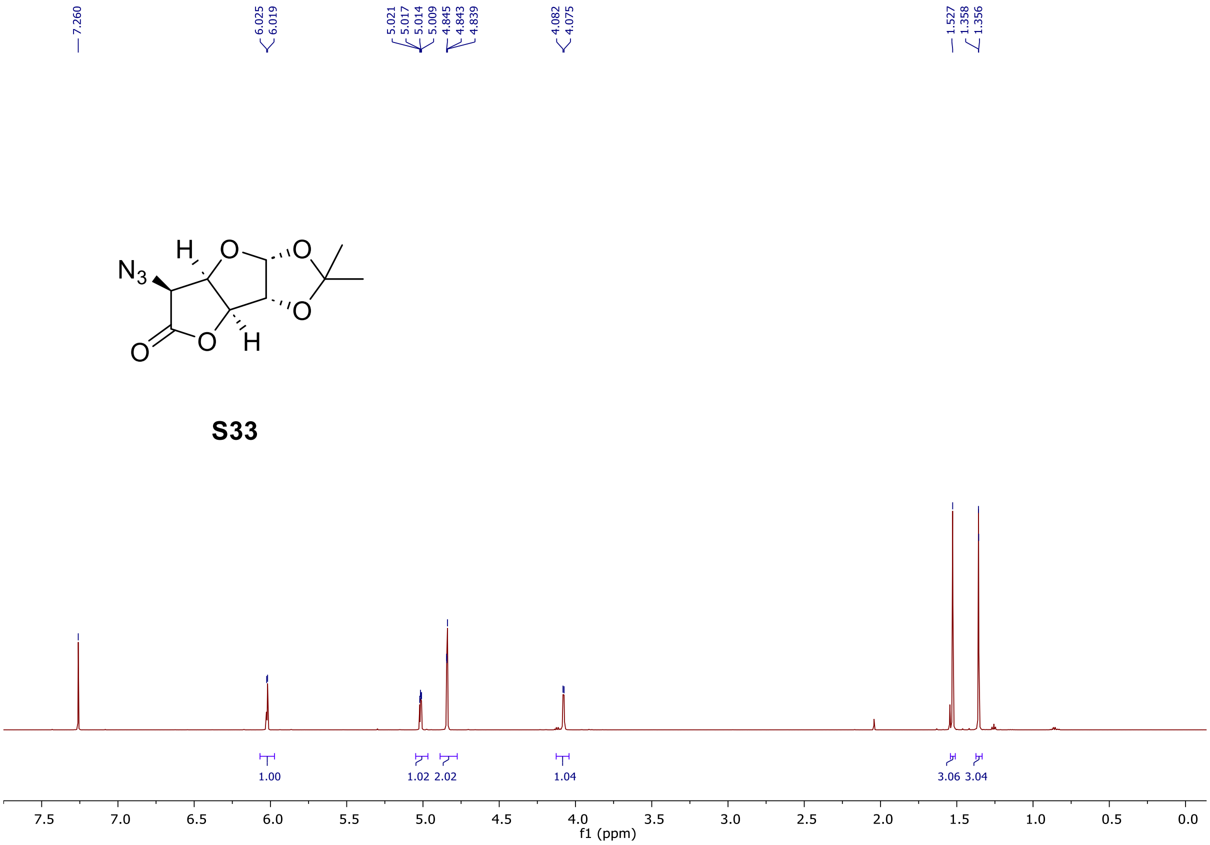

Supplementary Figure S112. ^{1}H NMR spectra for S33

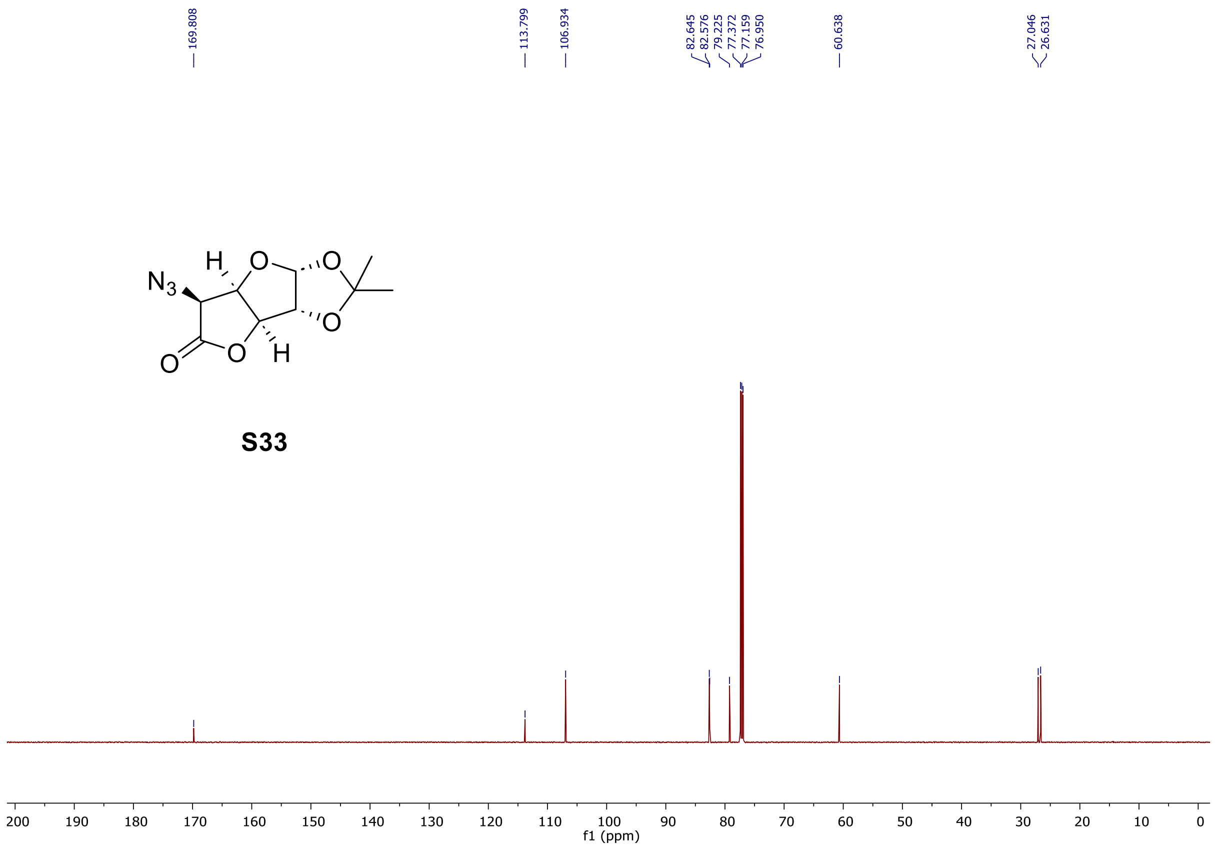

Supplementary Figure S113. ^{13}C NMR spectra for S33

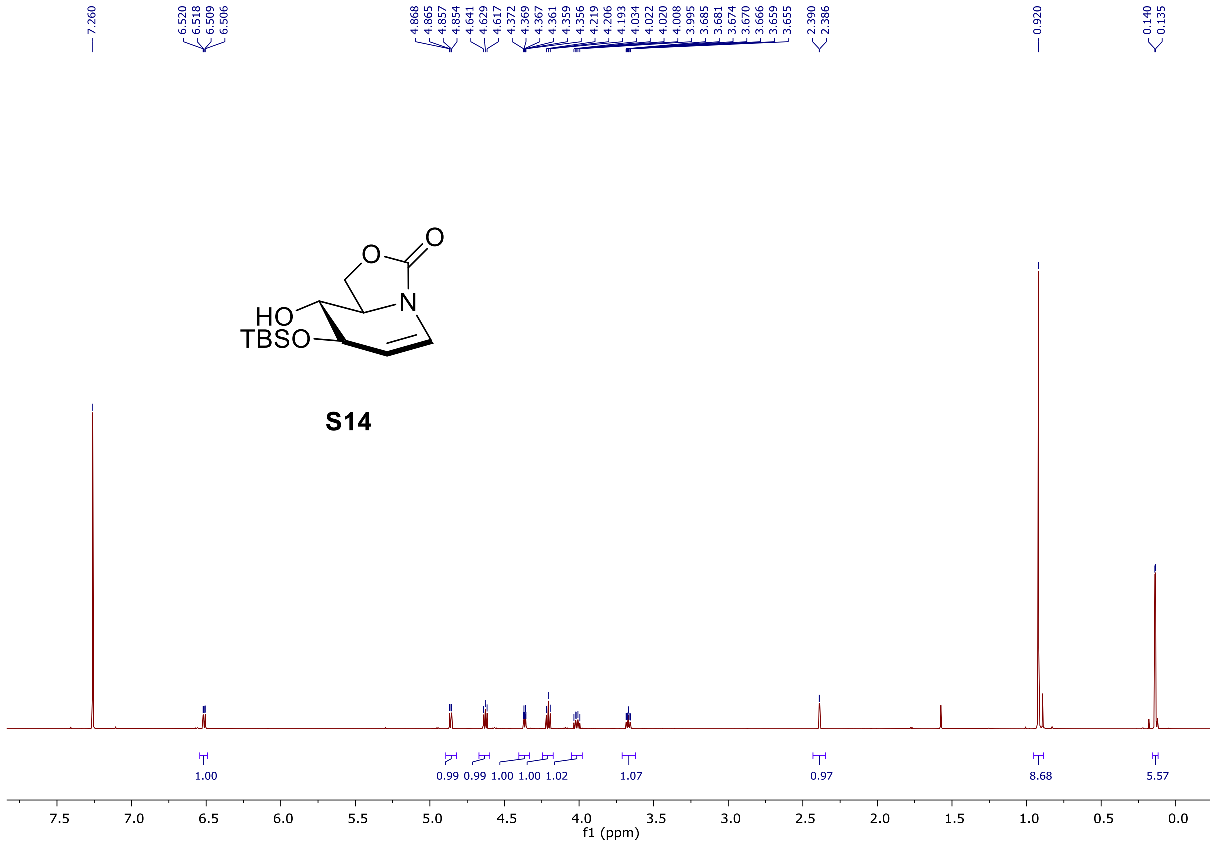

Supplementary Figure S114. ^{1}H NMR spectra for S14

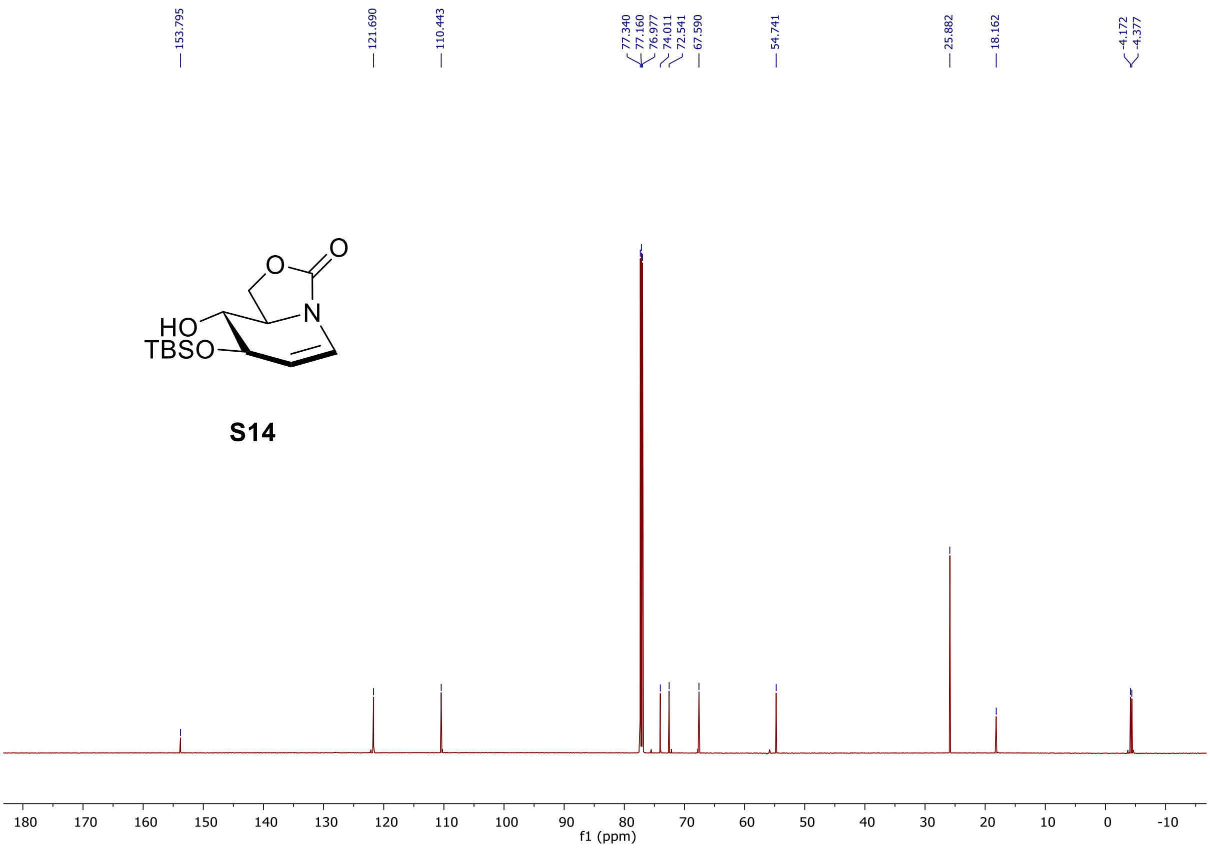

Supplementary Figure S115. ^{13}C NMR spectra for S14

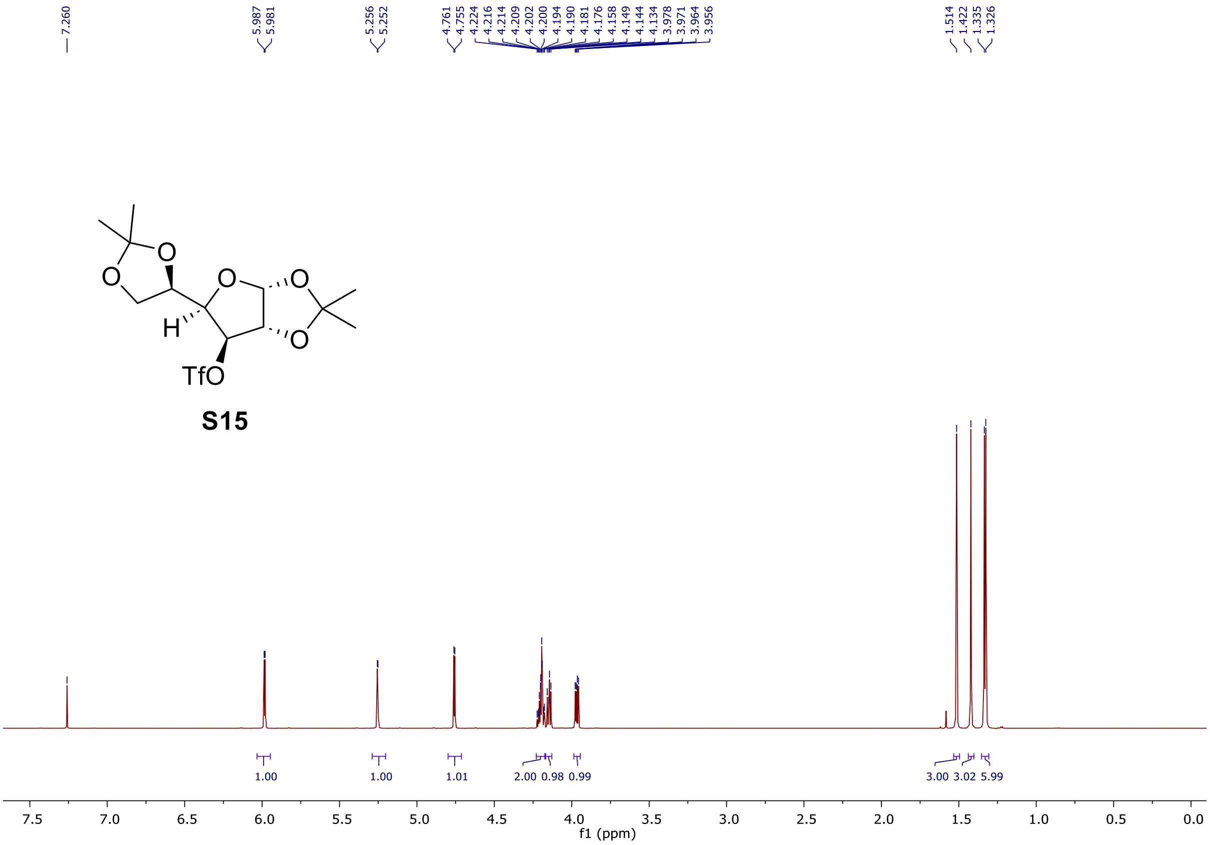

Supplementary Figure S116. ^{1}H NMR spectra for S15

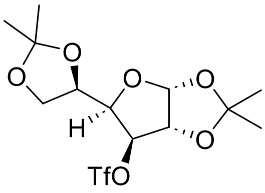

S15

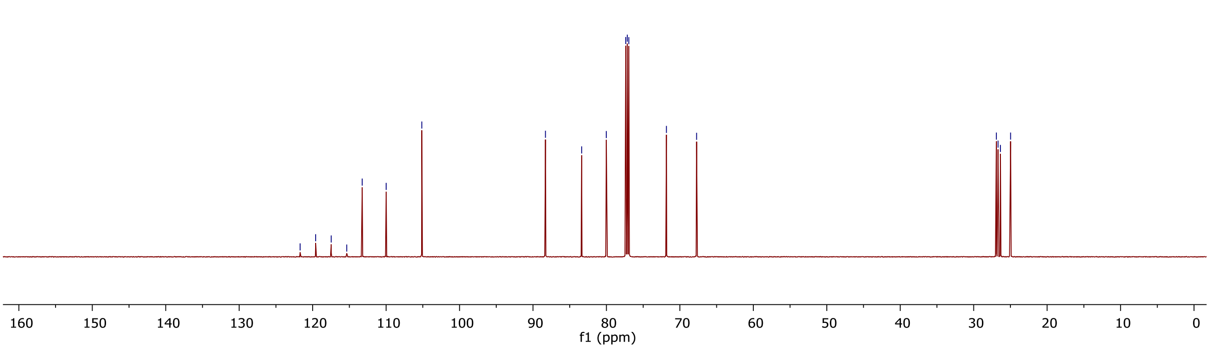

Supplementary Figure S117. ^{13}C NMR spectra for S15

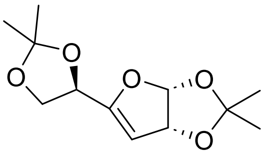

S16

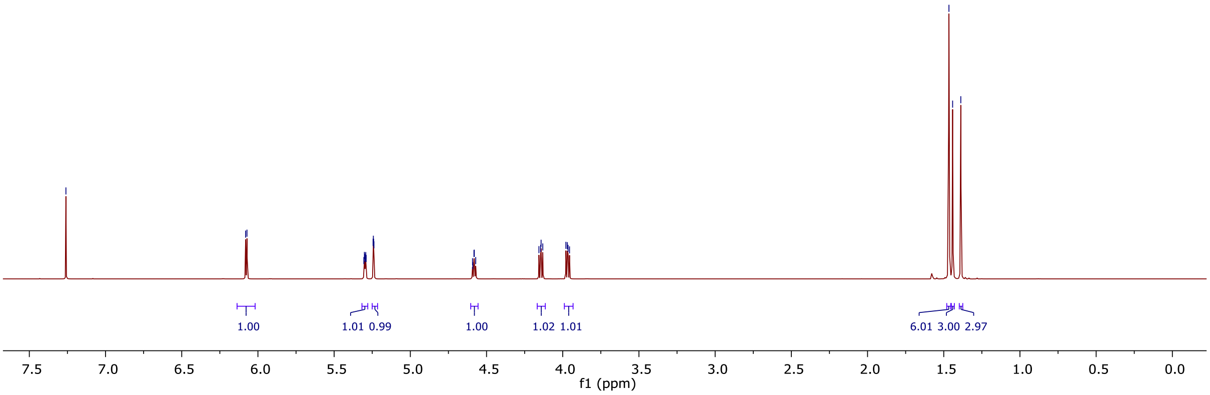

Supplementary Figure S118. ^{1}H NMR spectra for S16

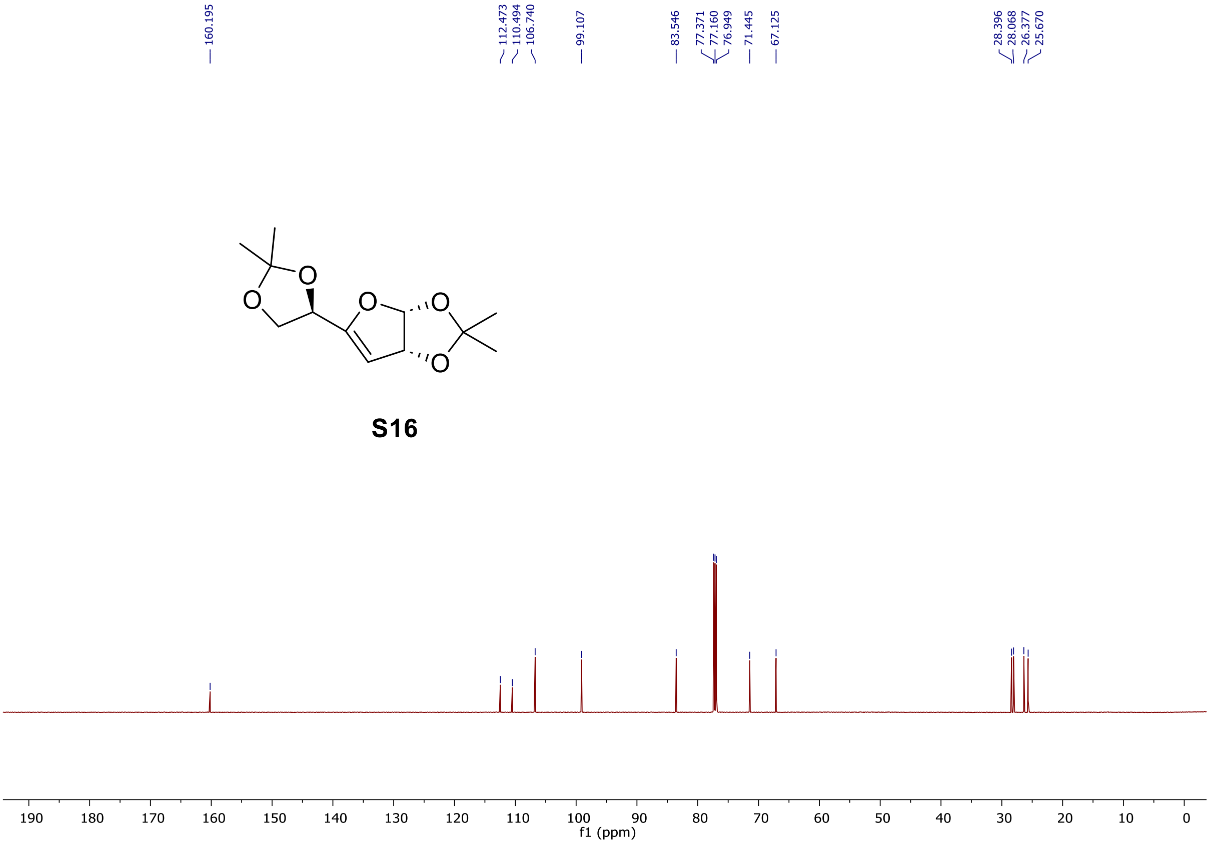

Supplementary Figure S119. ^{13}C NMR spectra for S16

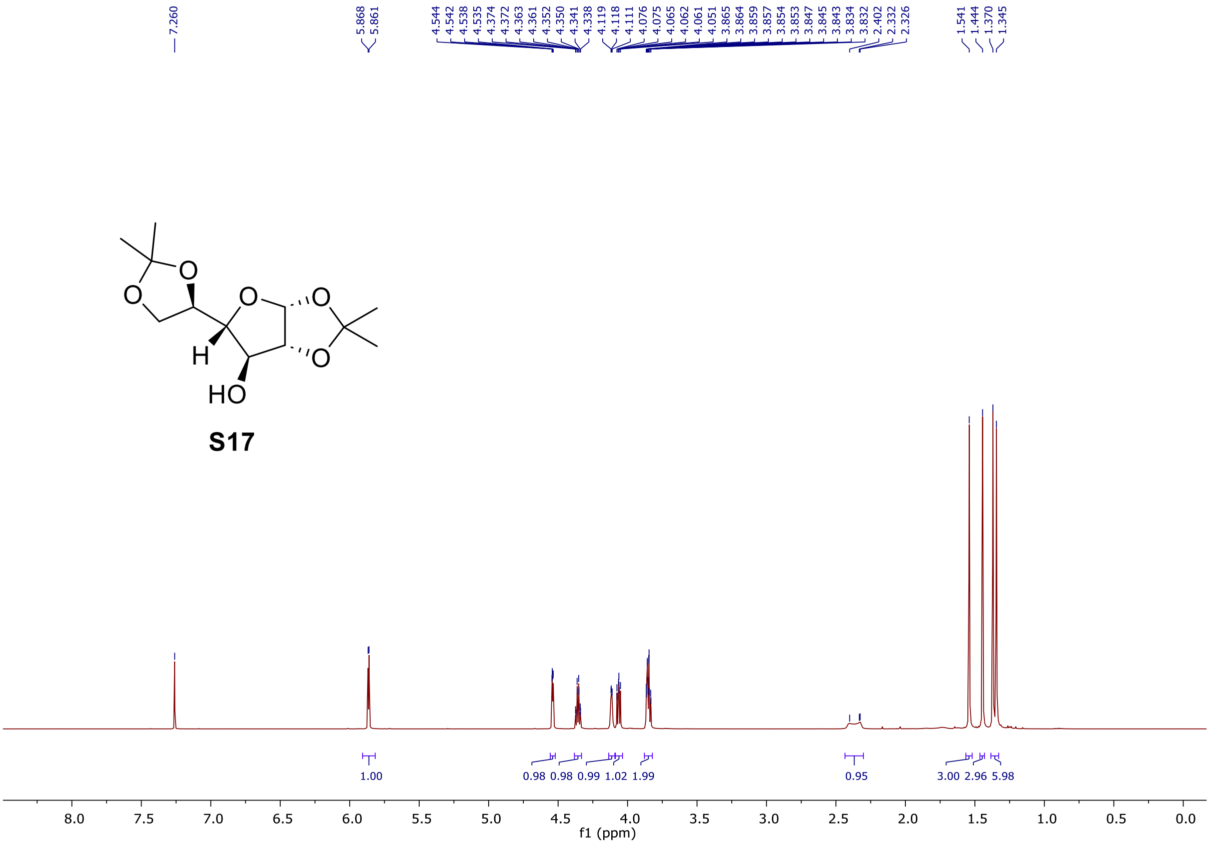

Supplementary Figure S120. ^{1}H NMR spectra for S17

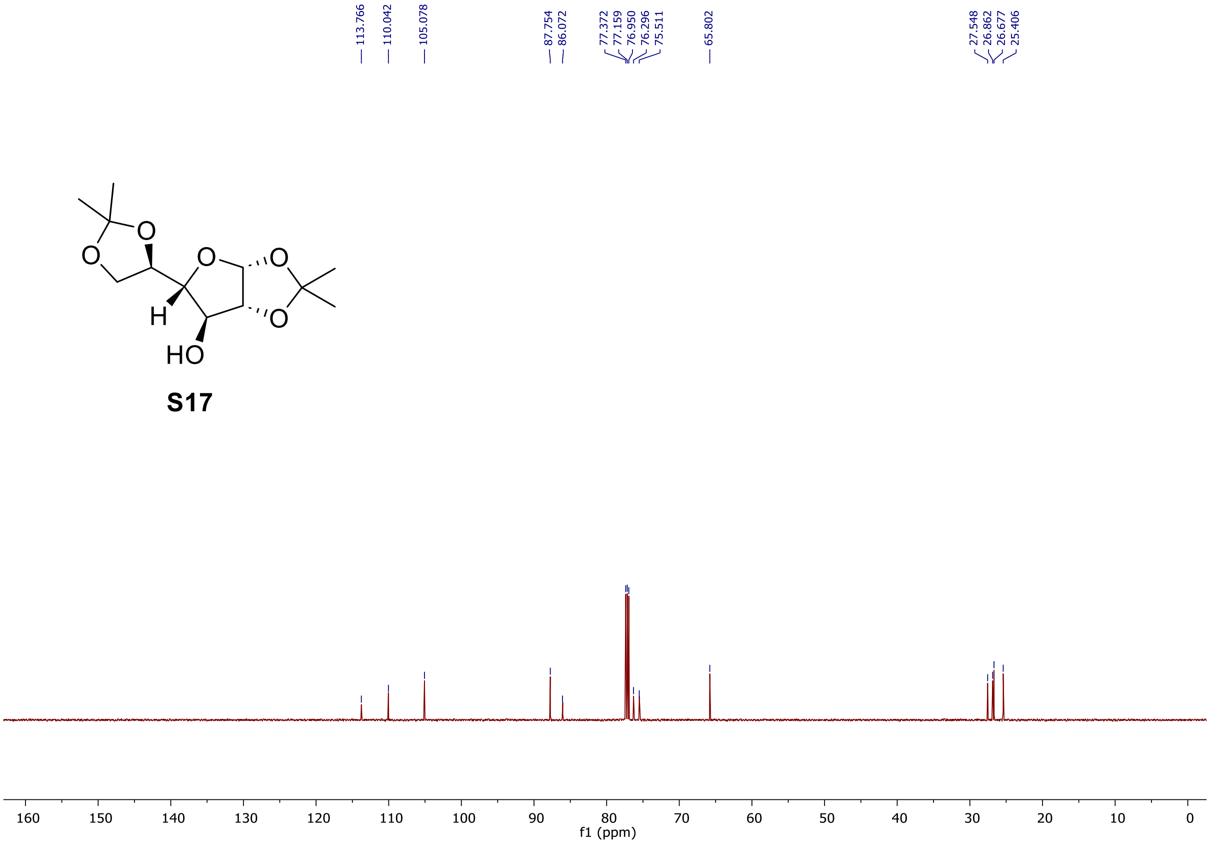

Supplementary Figure S121. ^{13}C NMR spectra for S17

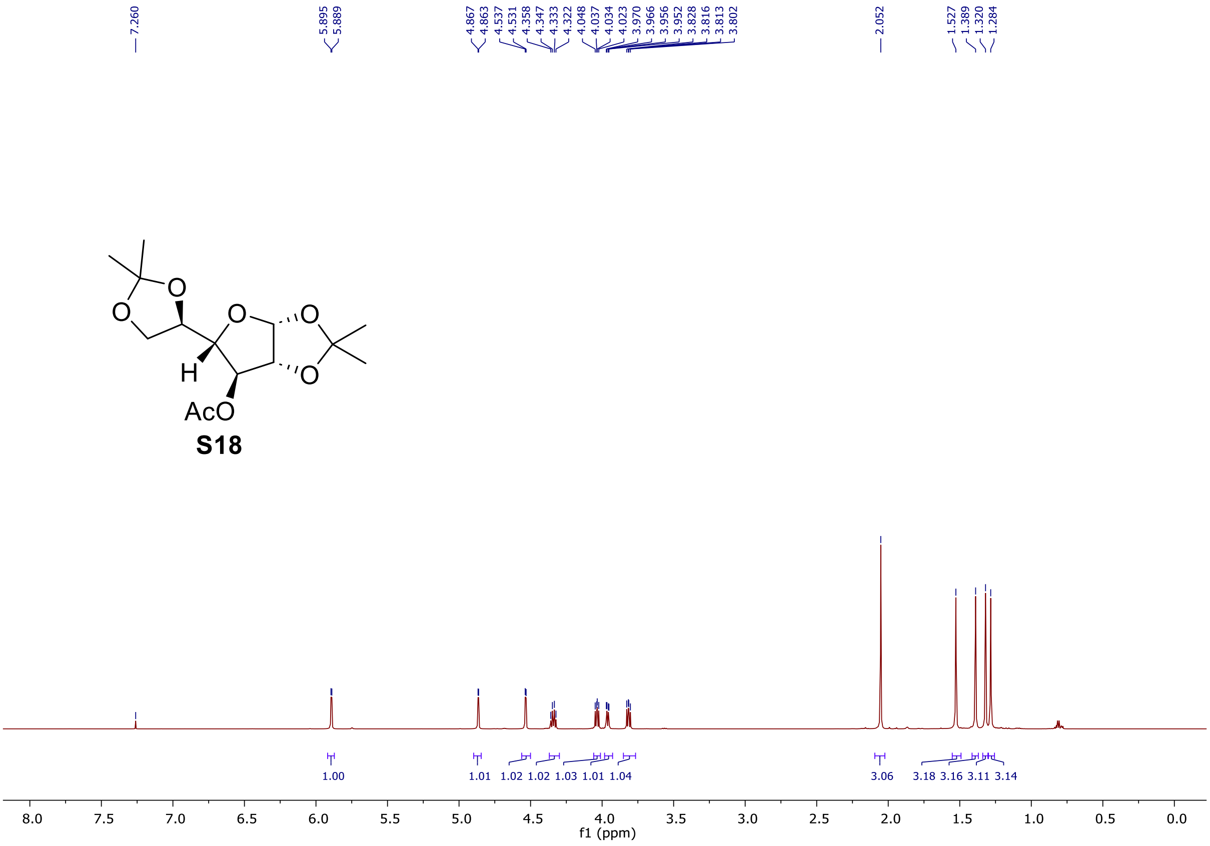

Supplementary Figure S122. ^{1}H NMR spectra for S18

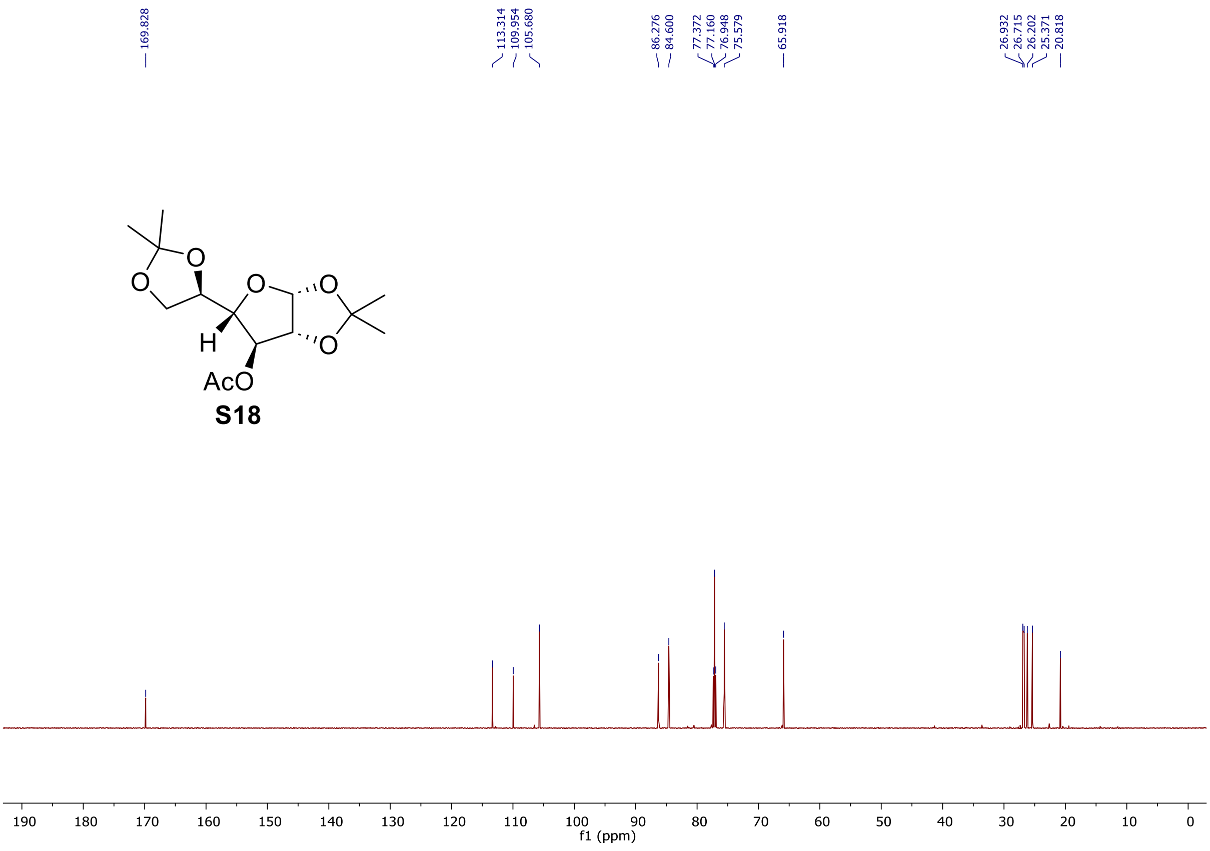

Supplementary Figure S123. ^{13}C NMR spectra for S18

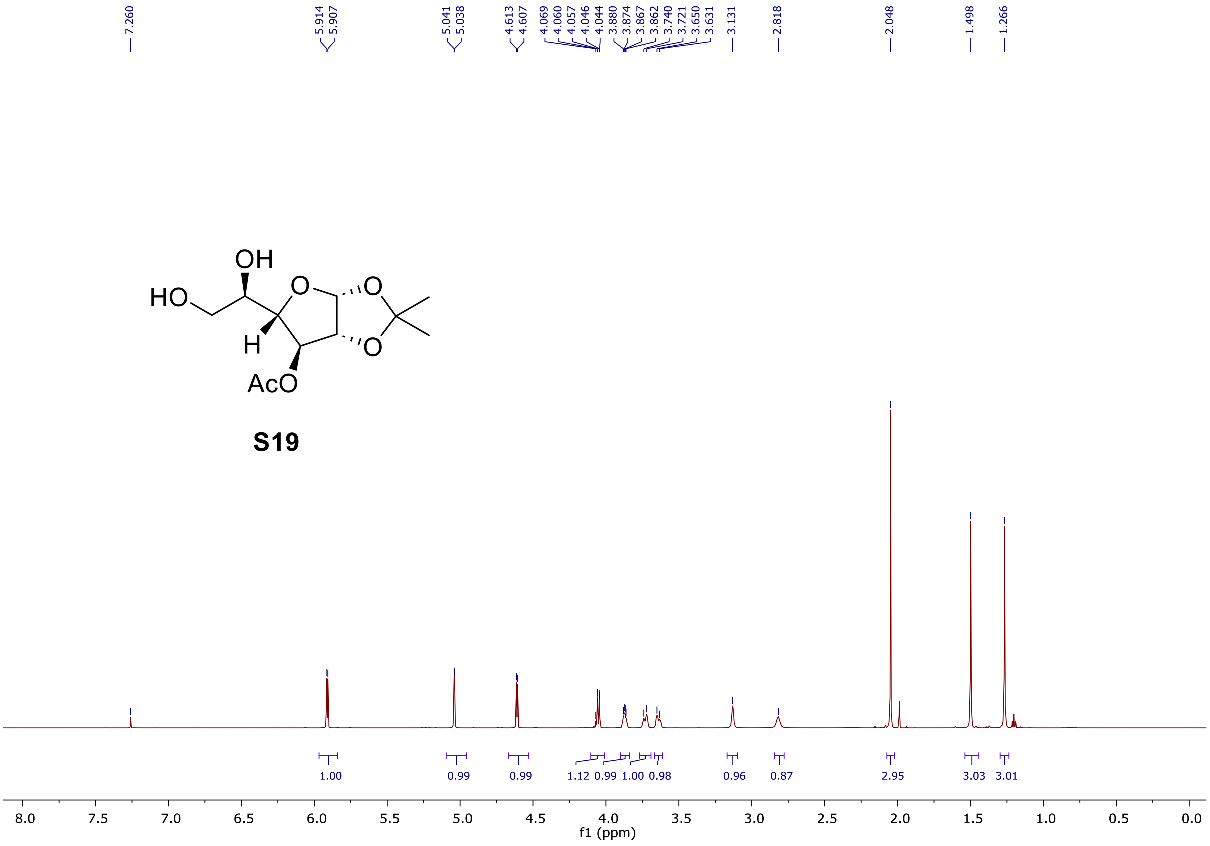

Supplementary Figure S124. ^{1}H NMR spectra for S19

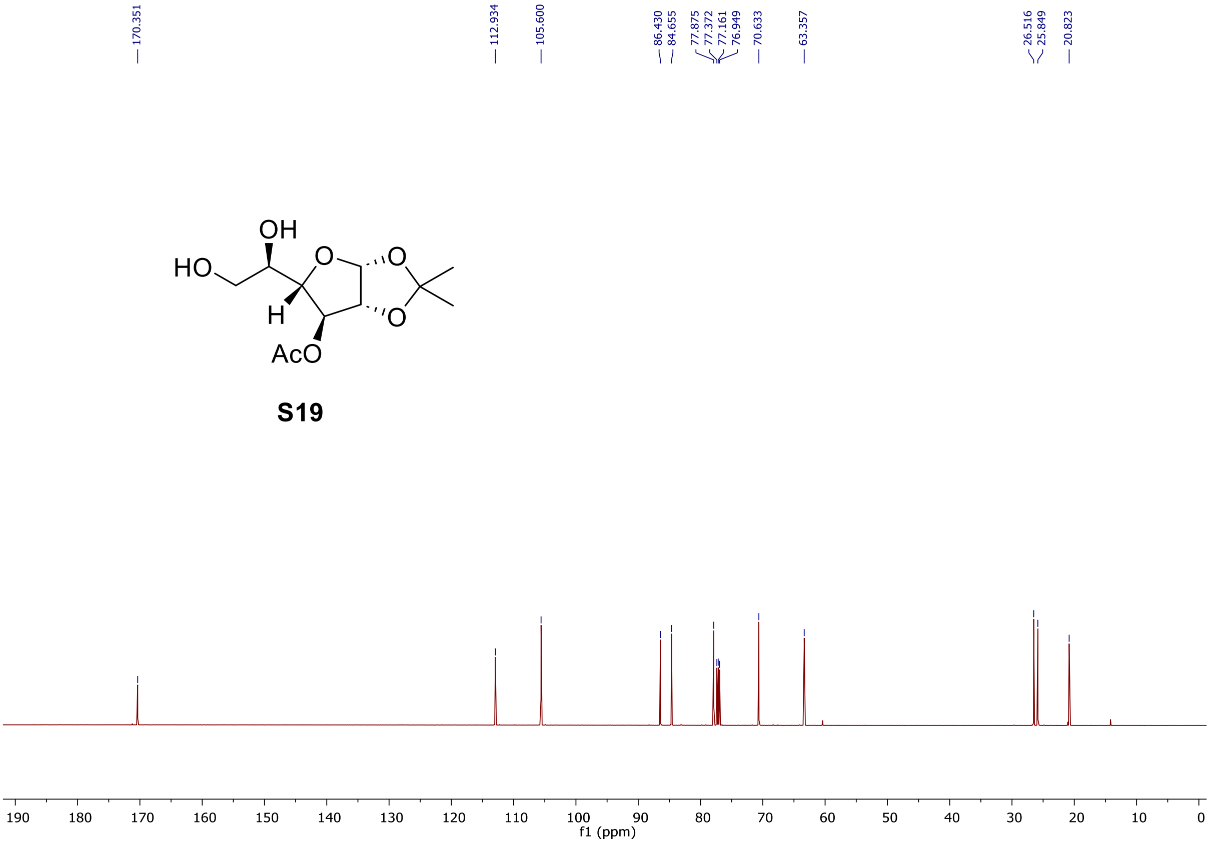

Supplementary Figure S125. ^{13}C NMR spectra for S19

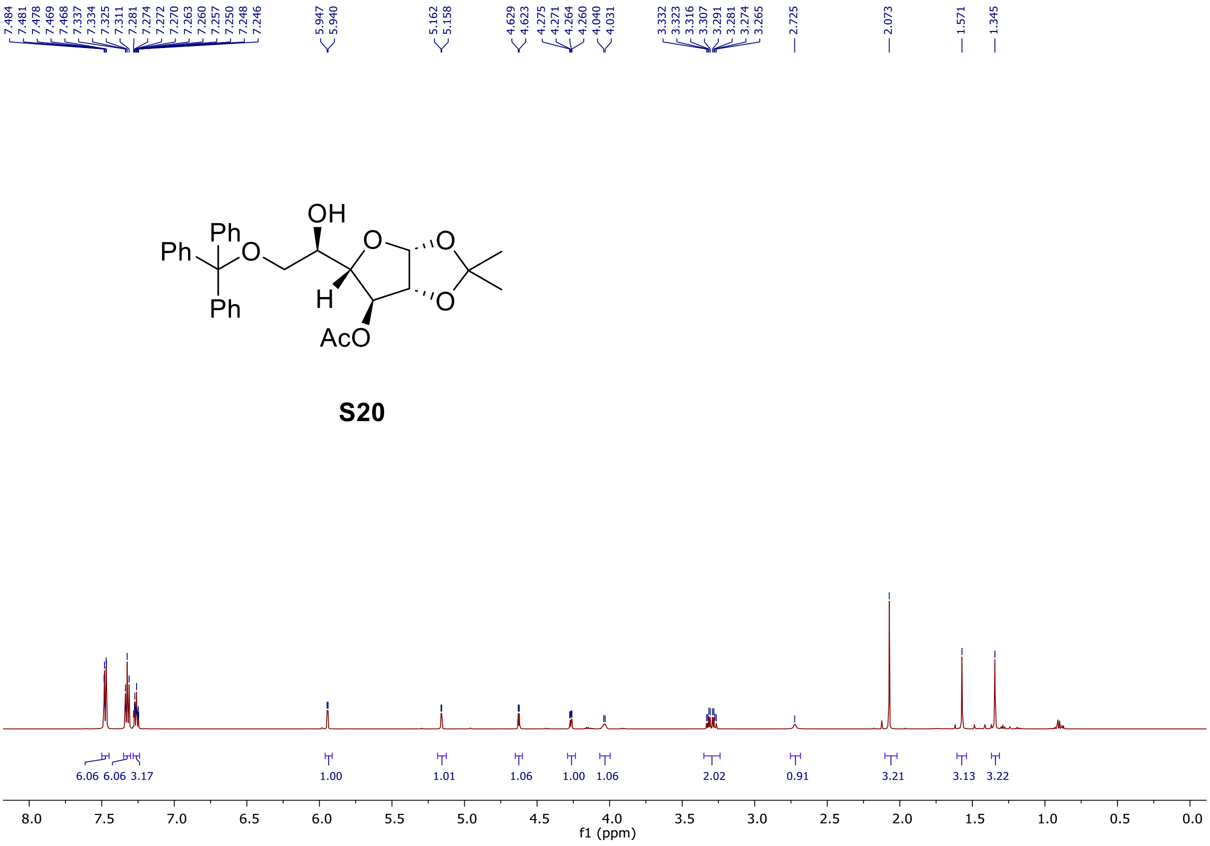

Supplementary Figure S126. ^{1}H NMR spectra for S20

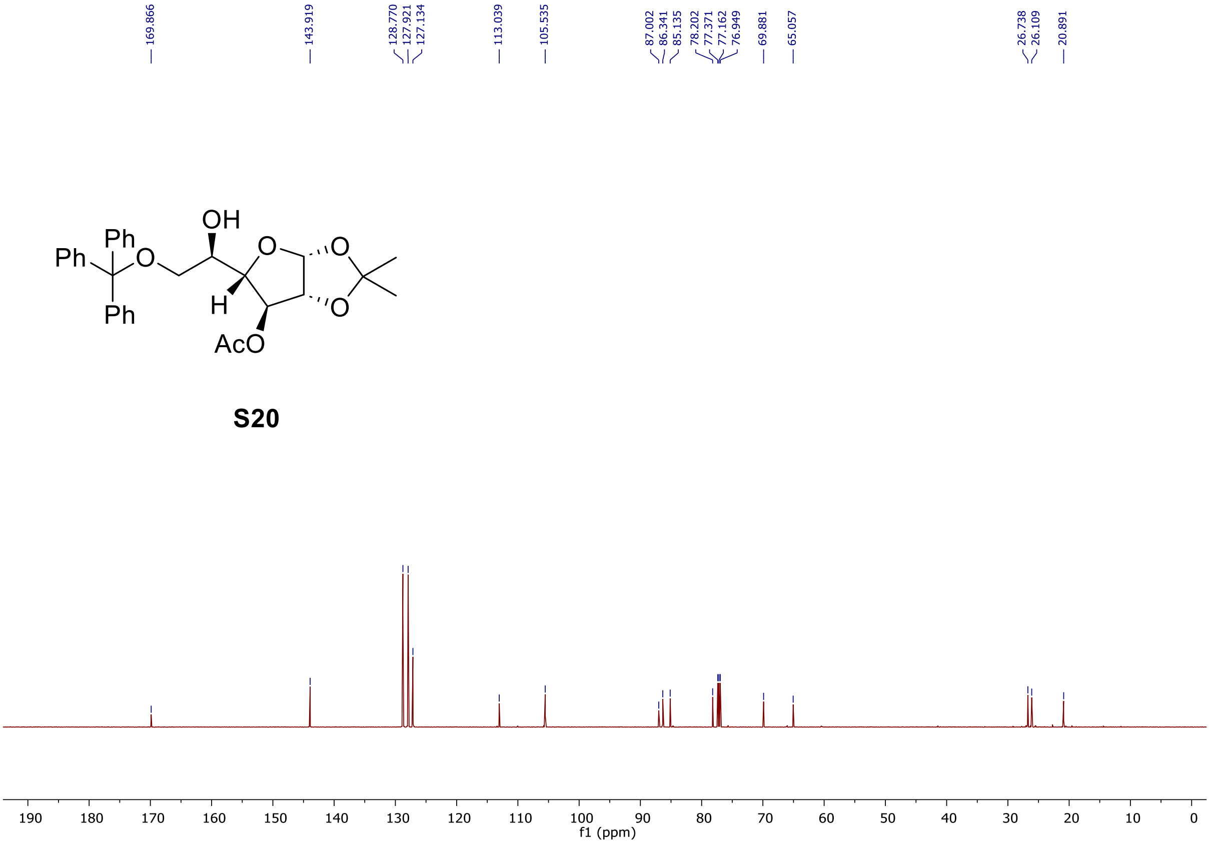

Supplementary Figure S127. ^{13}C NMR spectra for S20

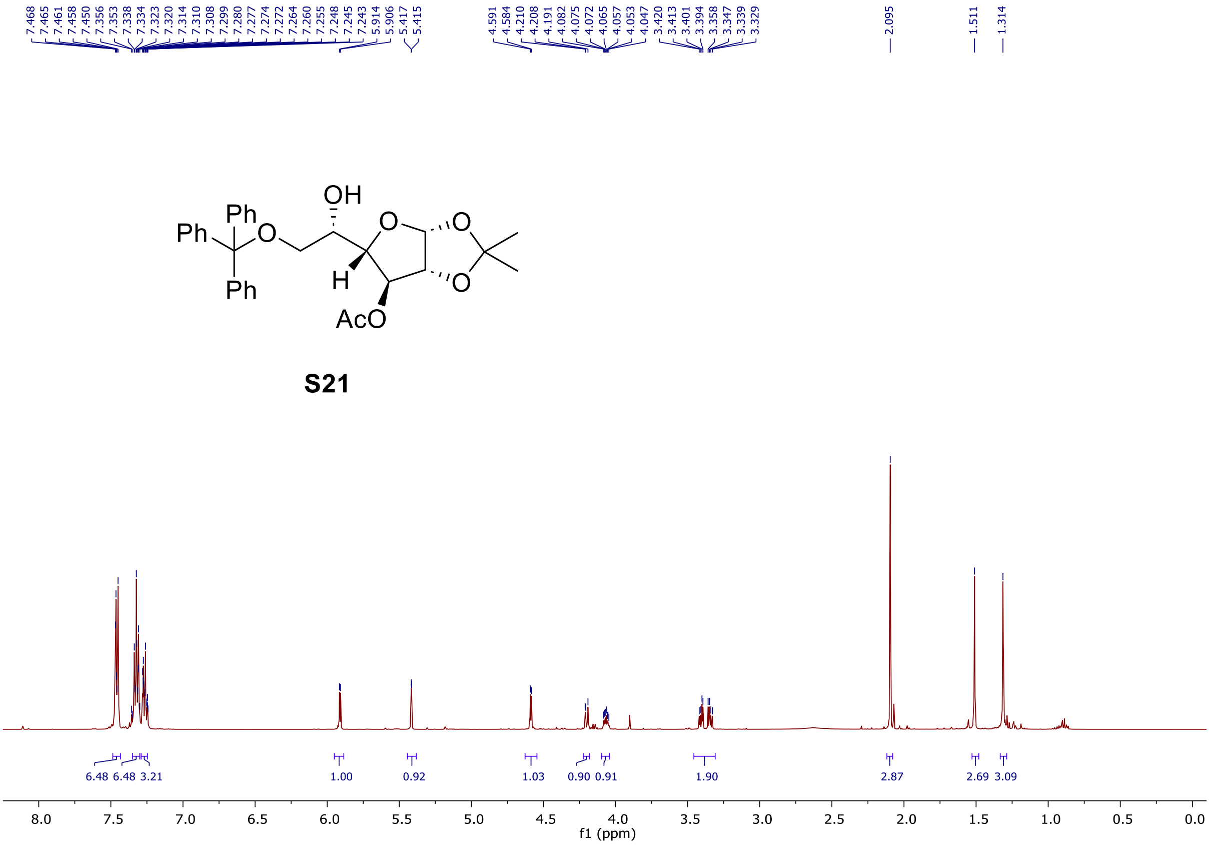

Supplementary Figure S128. ^{1}H NMR spectra for S21

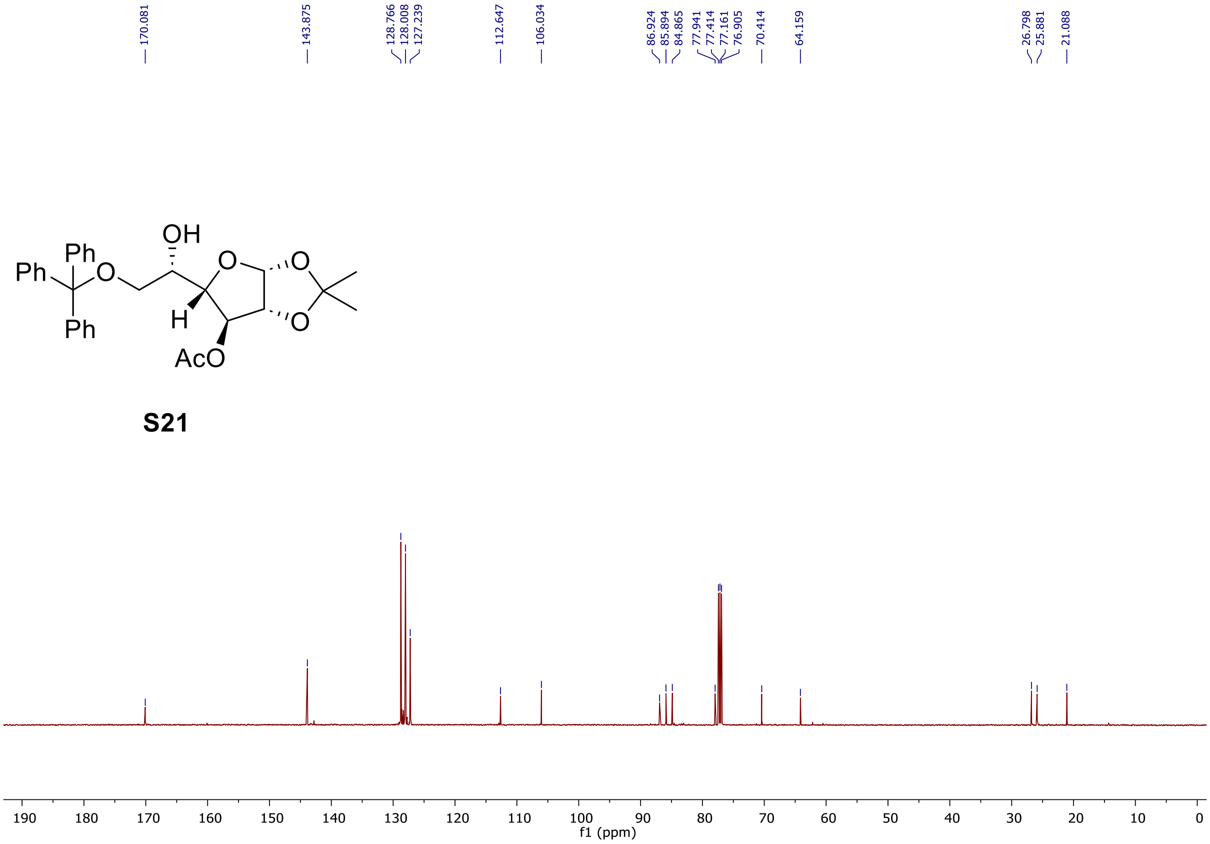

Supplementary Figure S129. ^{13}C NMR spectra for S21

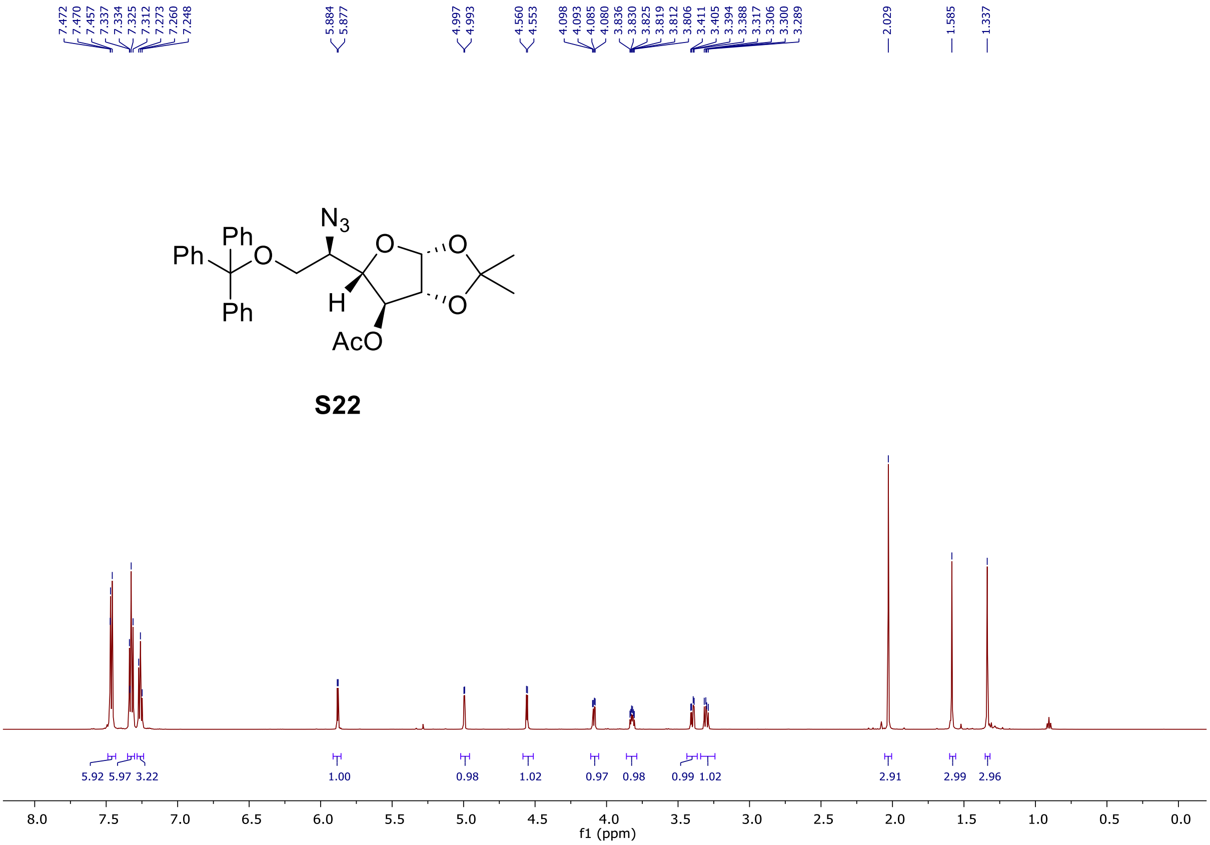

Supplementary Figure S130. ^{1}H NMR spectra for S22

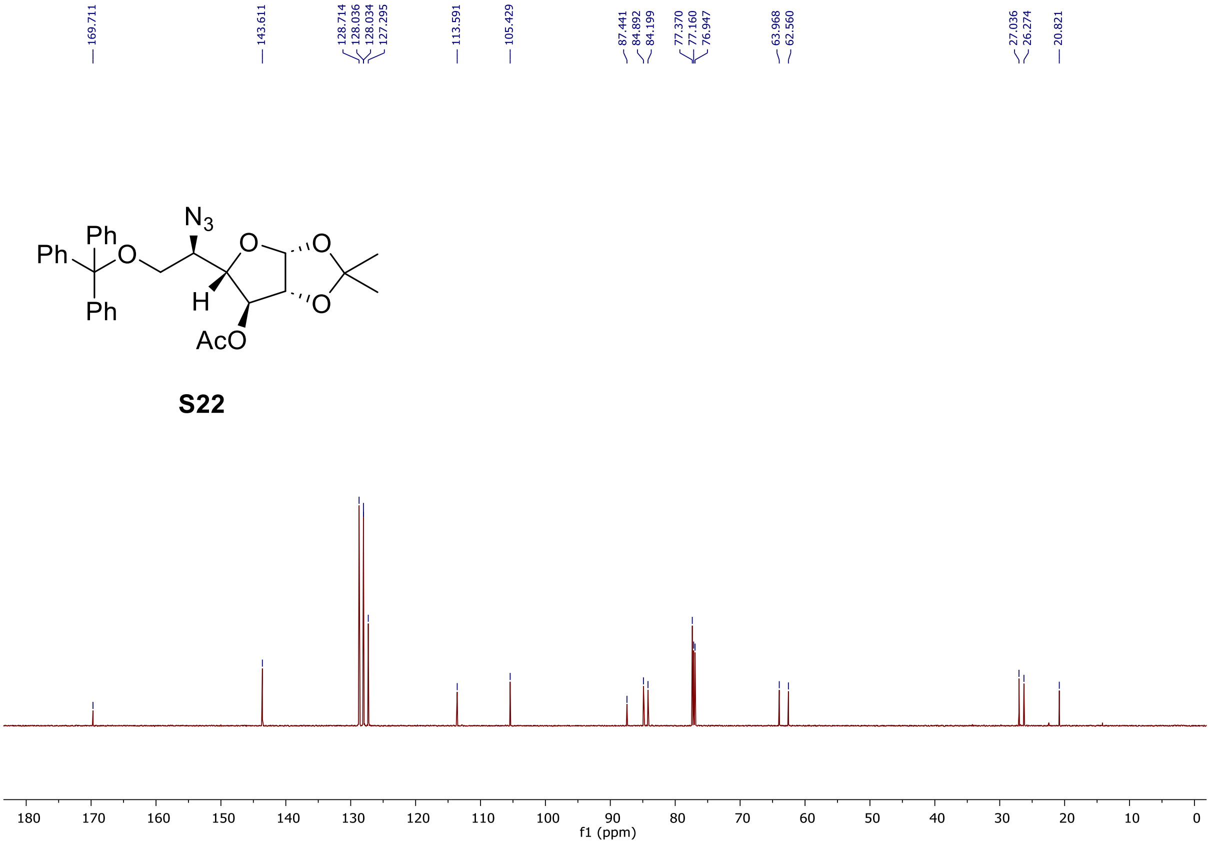

Supplementary Figure S131. ^{13}C NMR spectra for S22

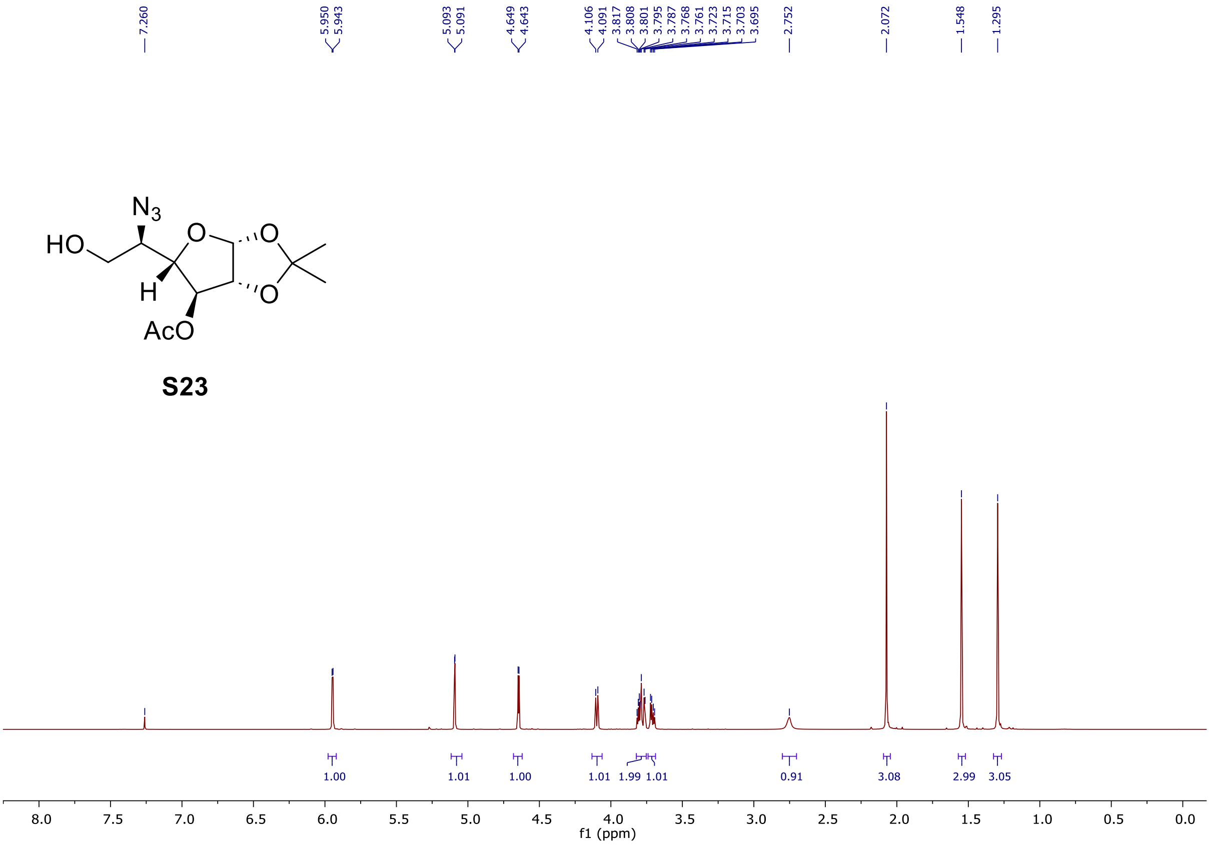

Supplementary Figure S132. ^{1}H NMR spectra for S23

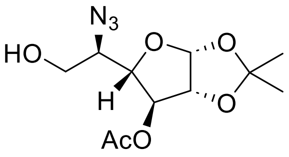

S23

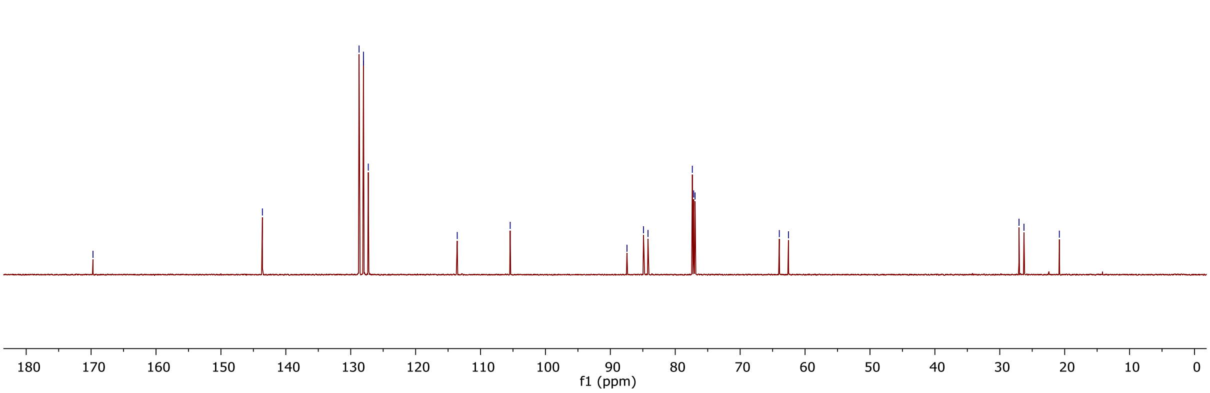

Supplementary Figure S133. ^{13}C NMR spectra for S23

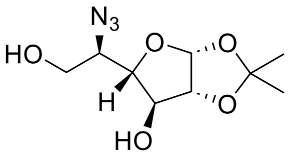

S24

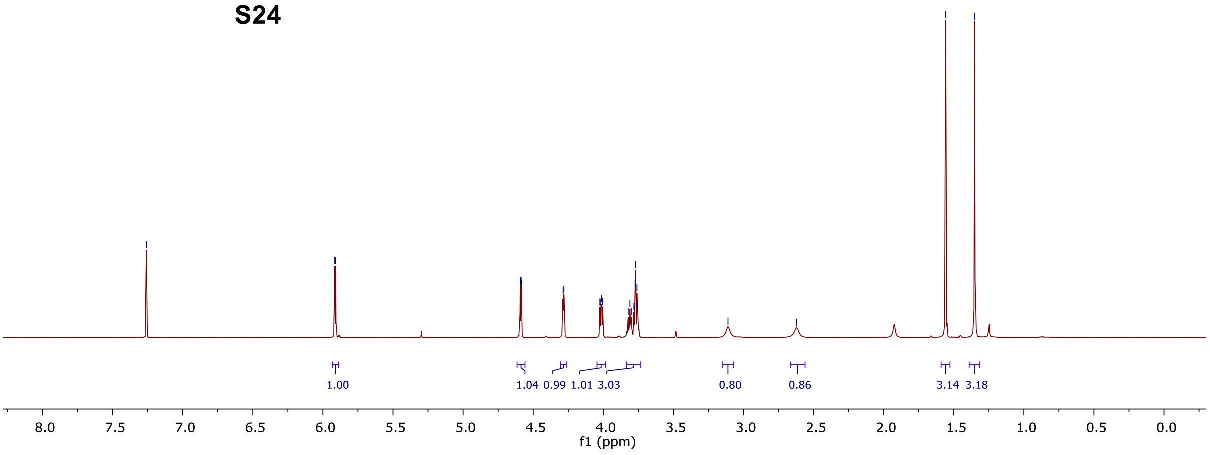

Supplementary Figure S134. ^{1}H NMR spectra for S24

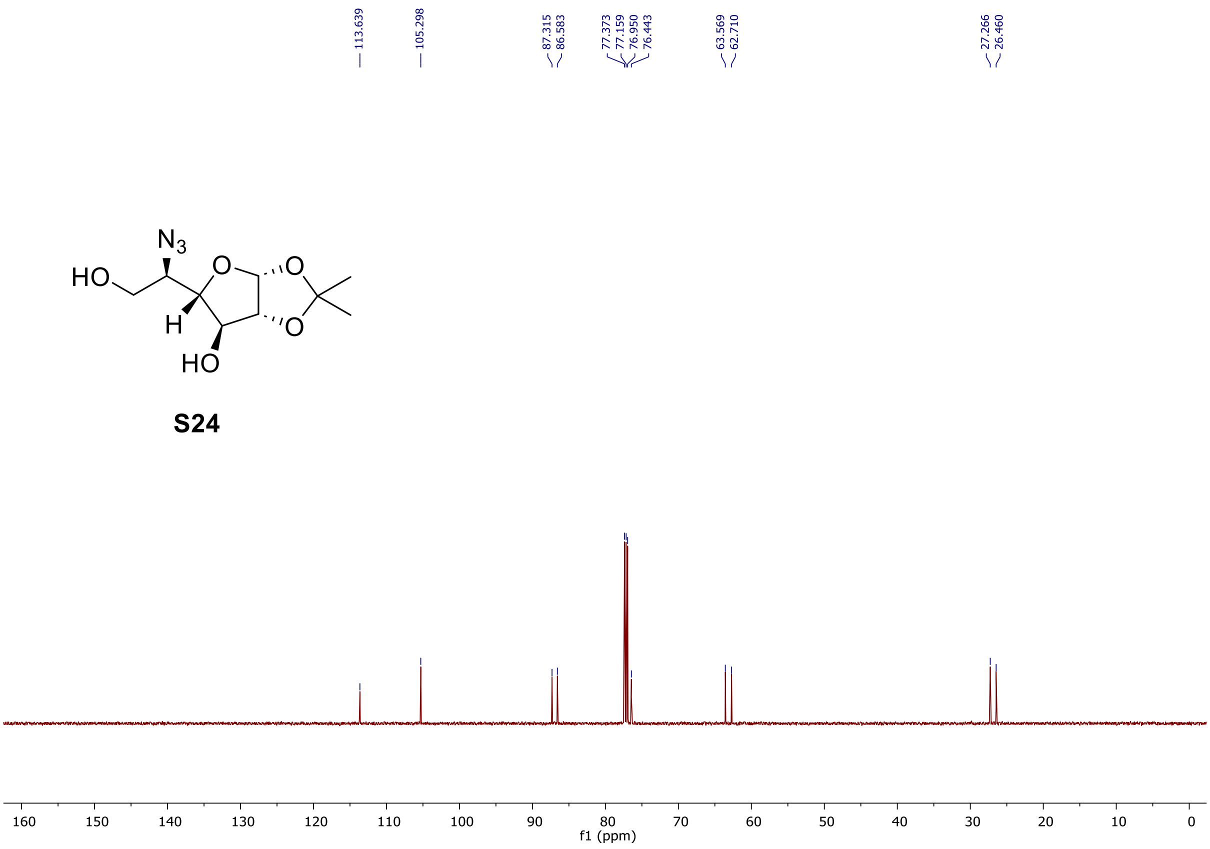

Supplementary Figure S135. ^{13}C NMR spectra for S24

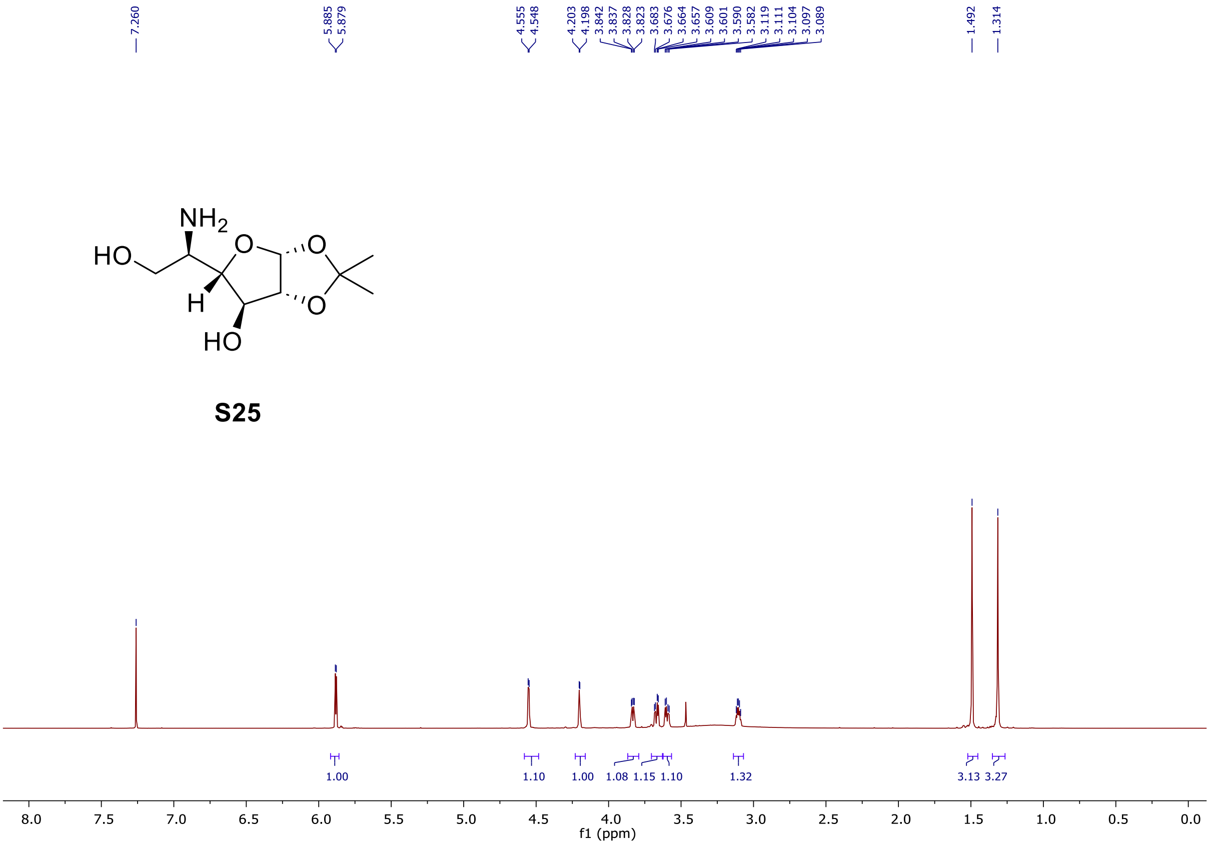

Supplementary Figure S136. ^{1}H NMR spectra for S25

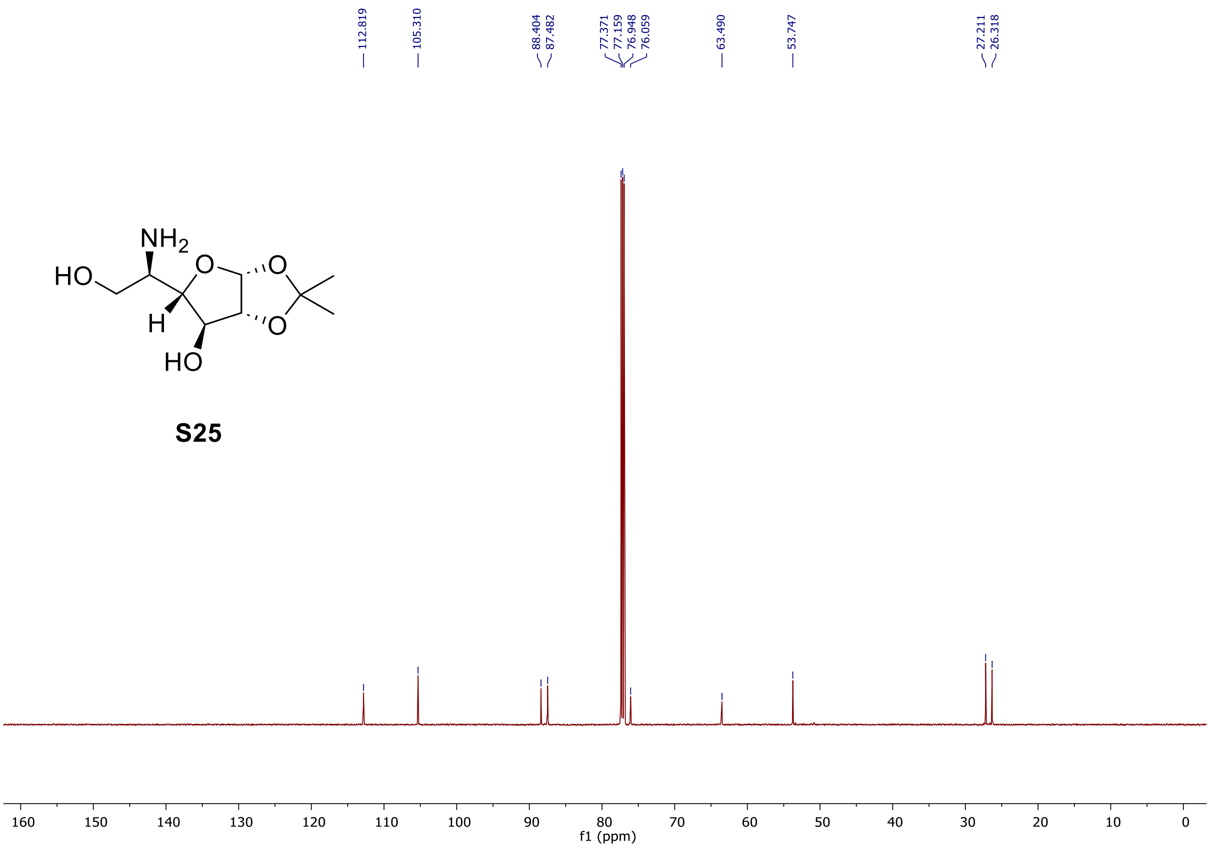

Supplementary Figure S137. ^{13}C NMR spectra for S25

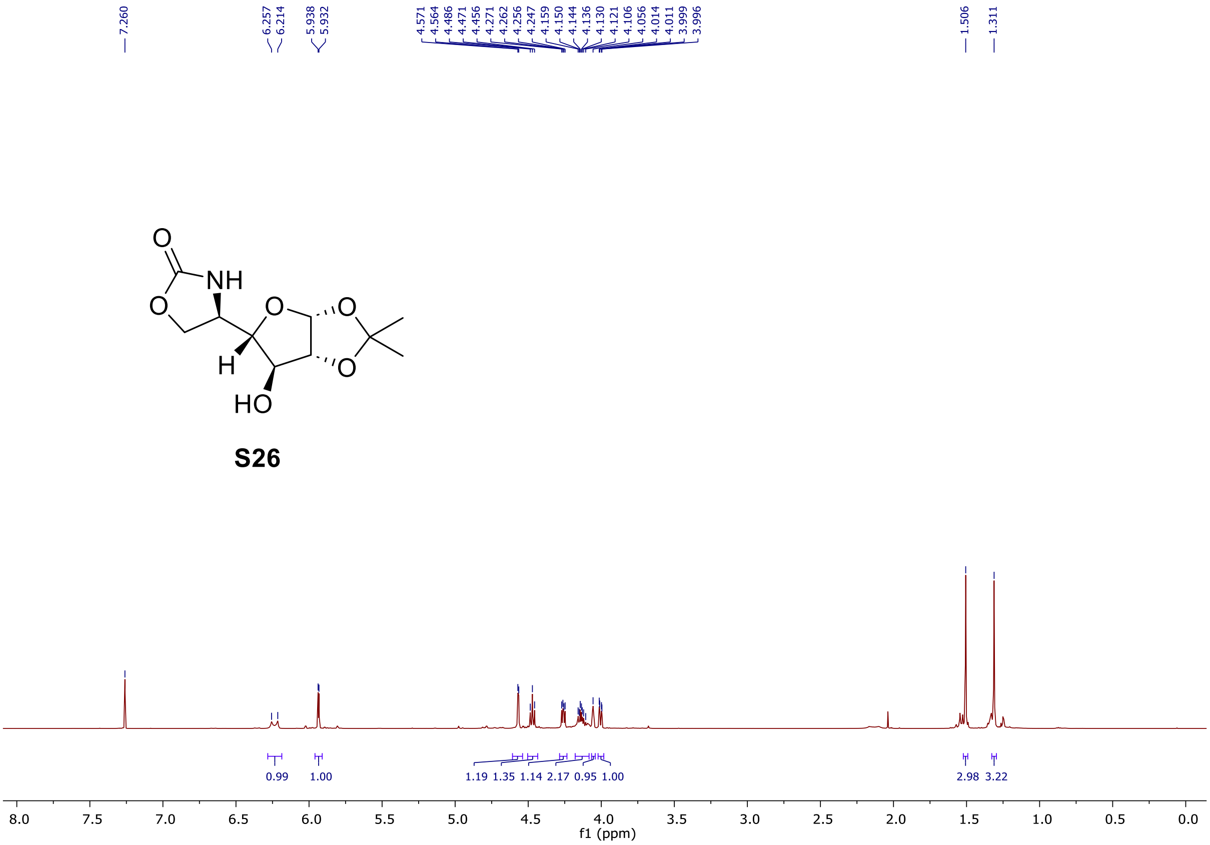

Supplementary Figure S138. ^{1}H NMR spectra for S26

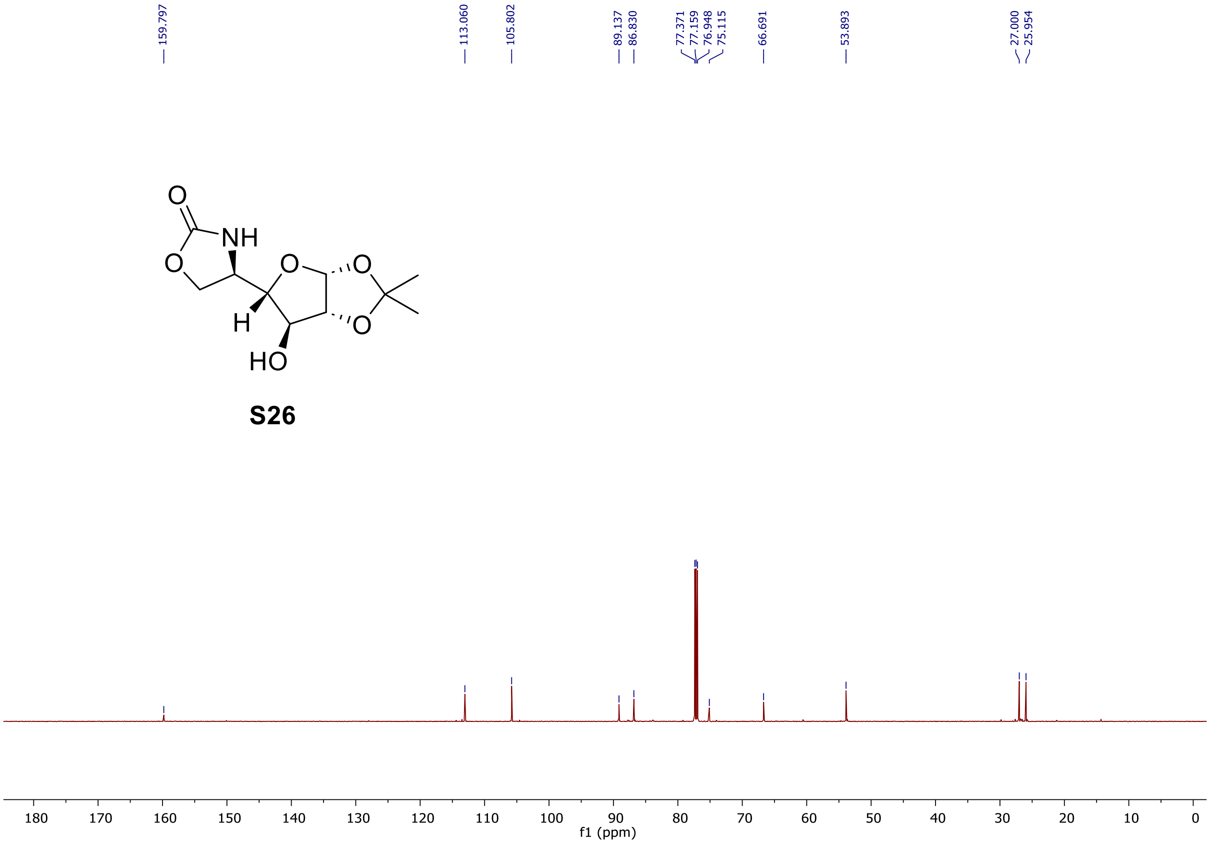

Supplementary Figure S139. ^{13}C NMR spectra for S26

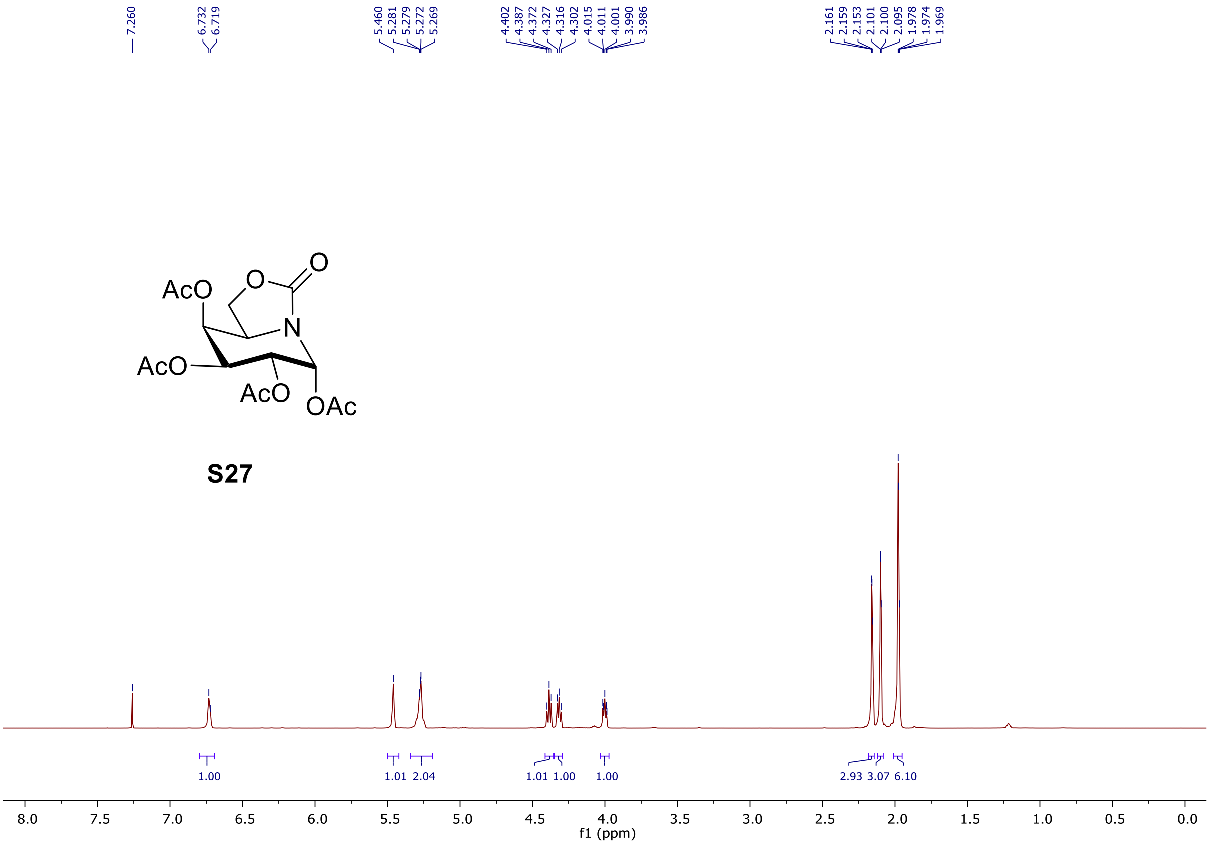

Supplementary Figure S140. ^{1}H NMR spectra for S27

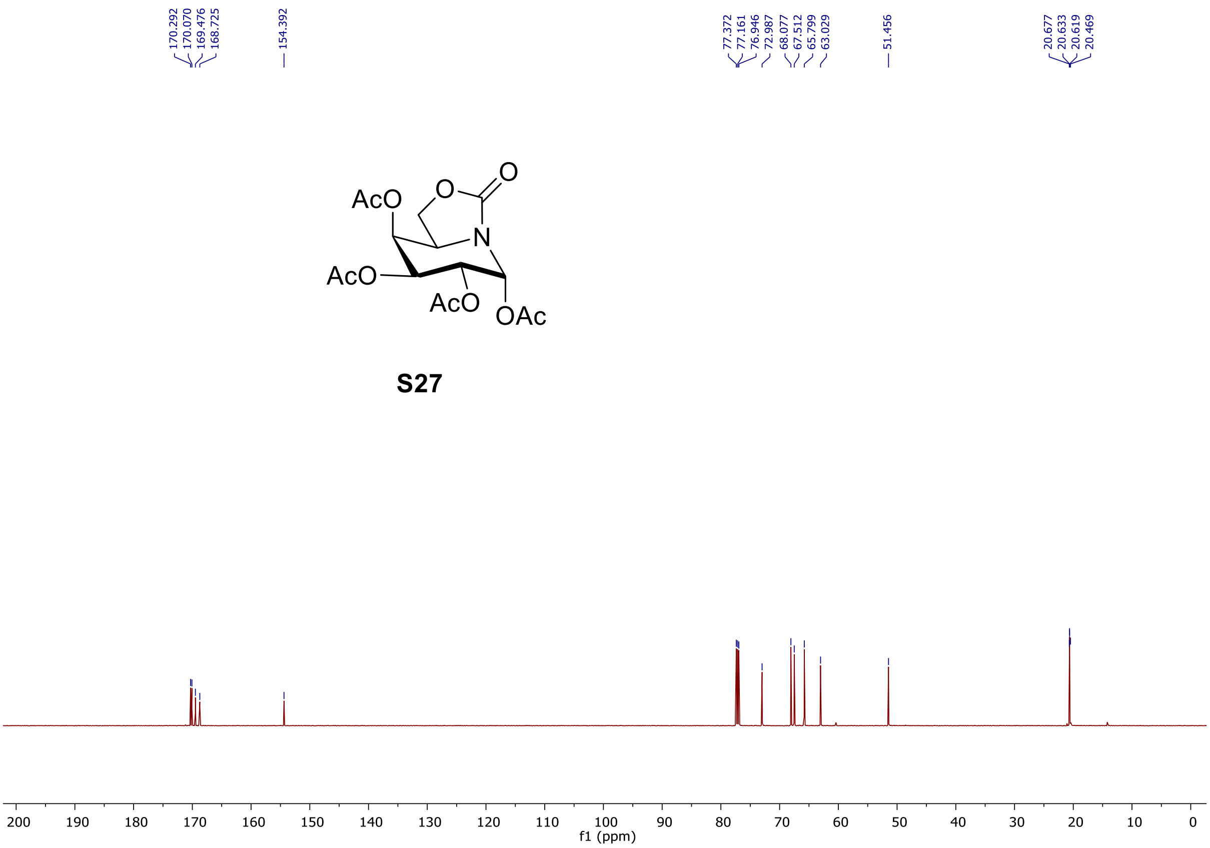

Supplementary Figure S141. ^{13}C NMR spectra for S27

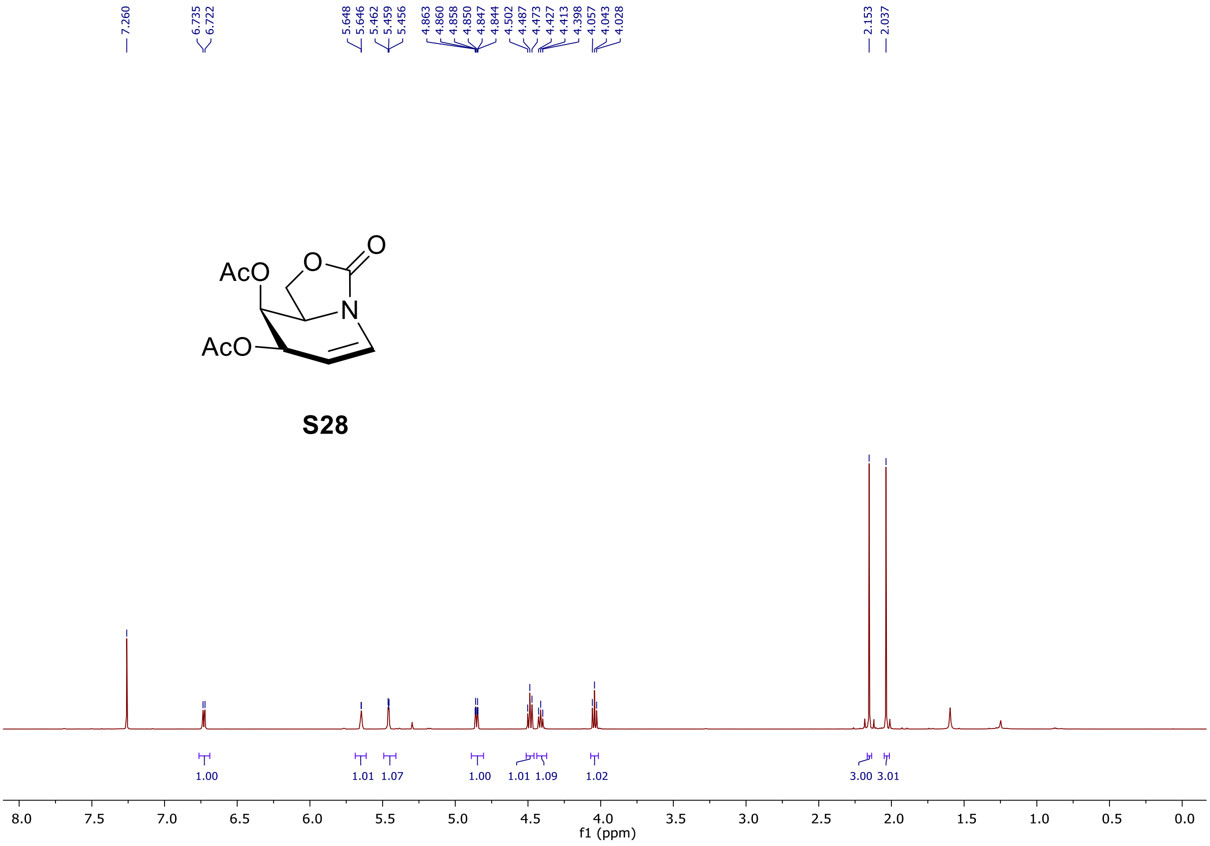

Supplementary Figure S142. ^{1}H NMR spectra for S28

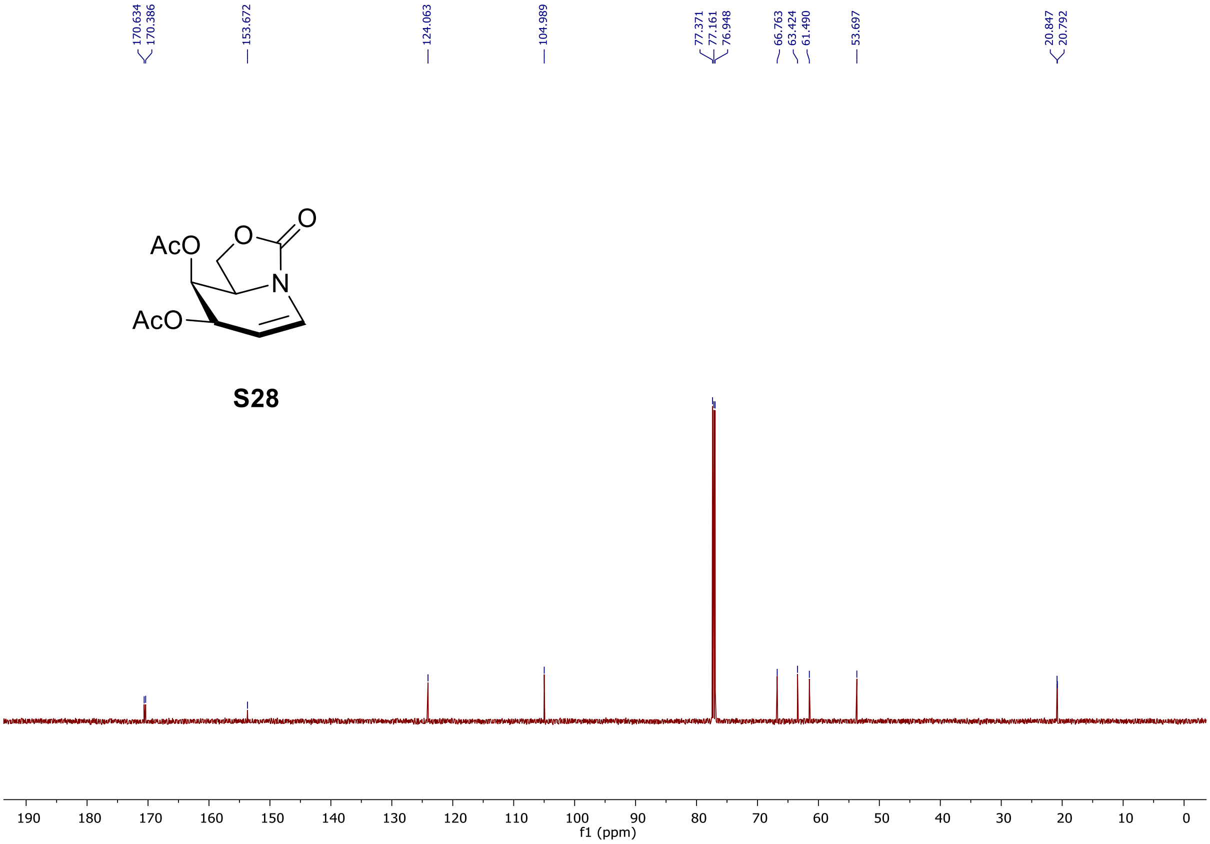

Supplementary Figure S143. ^{13}C NMR spectra for S28

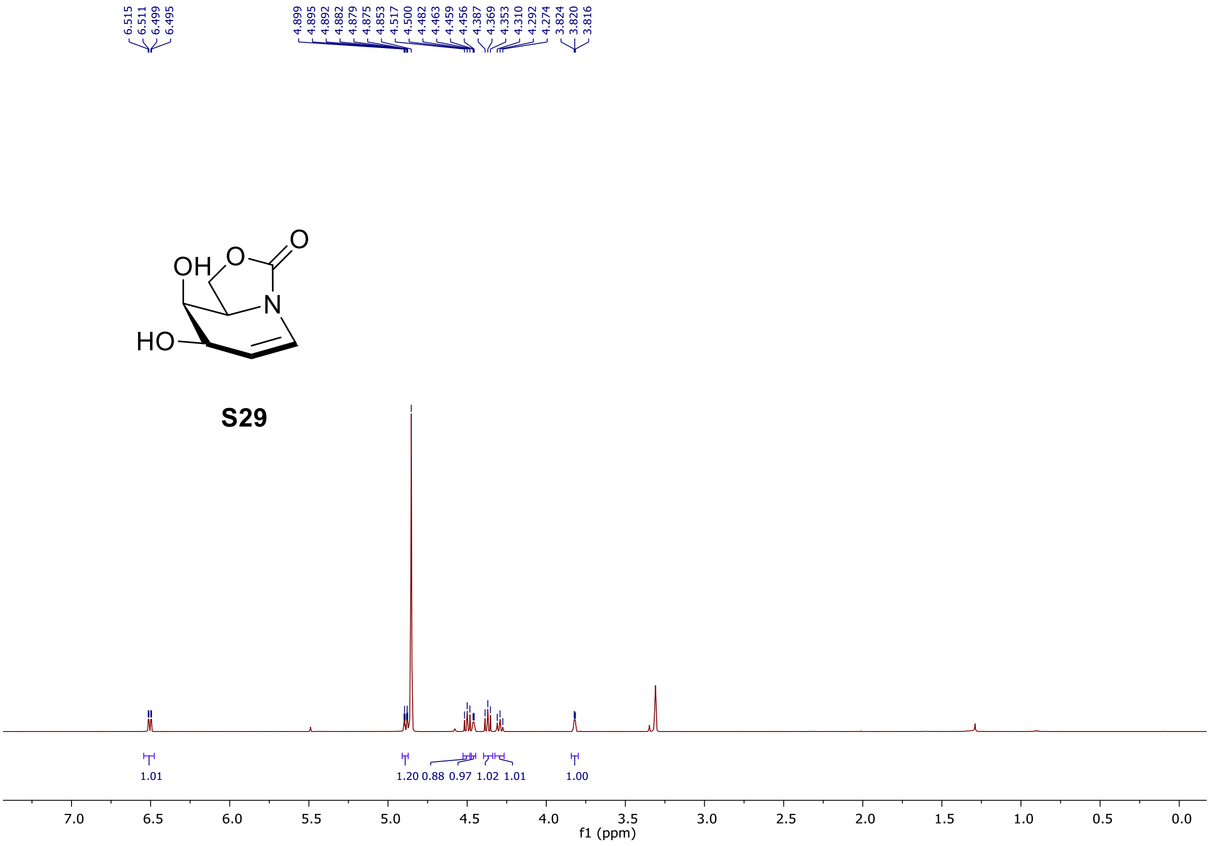

Supplementary Figure S144. ^{1}H NMR spectra for S29

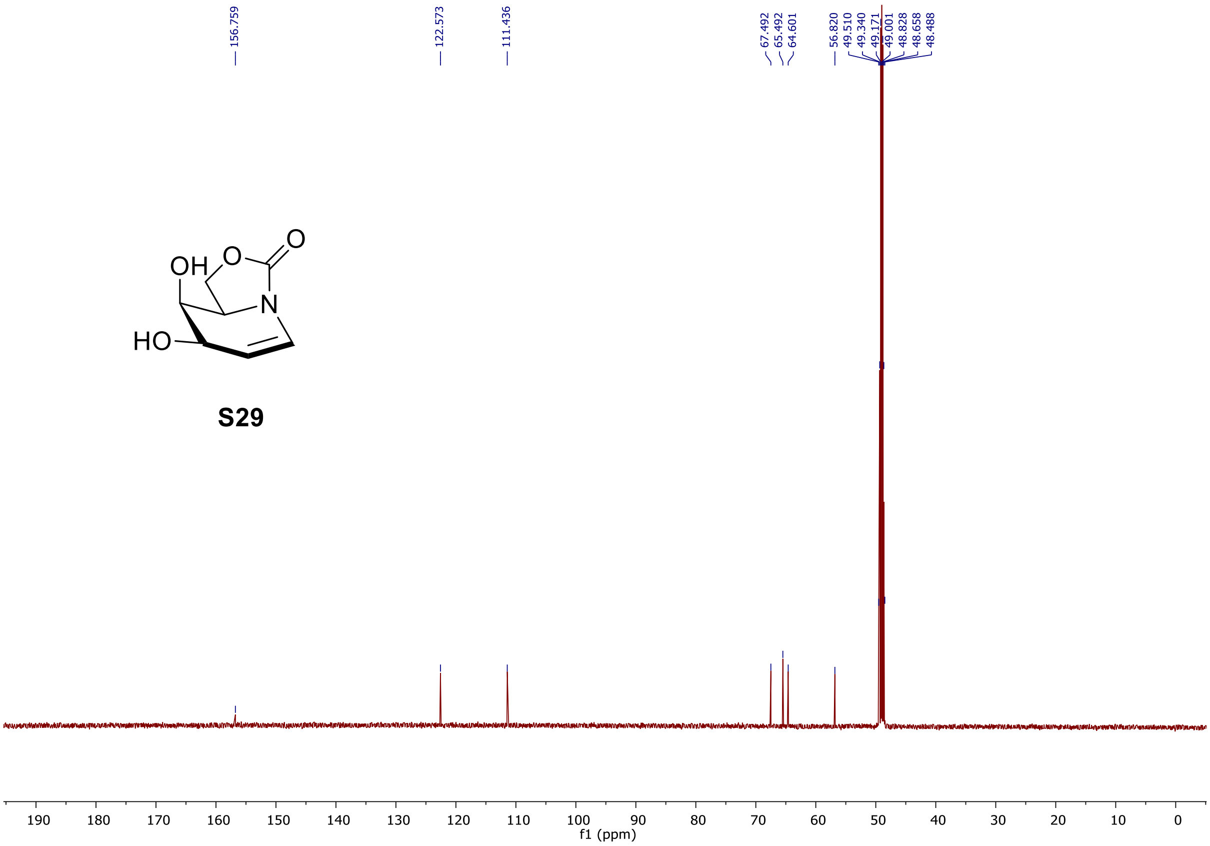

Supplementary Figure S145. ^{13}C NMR spectra for S29

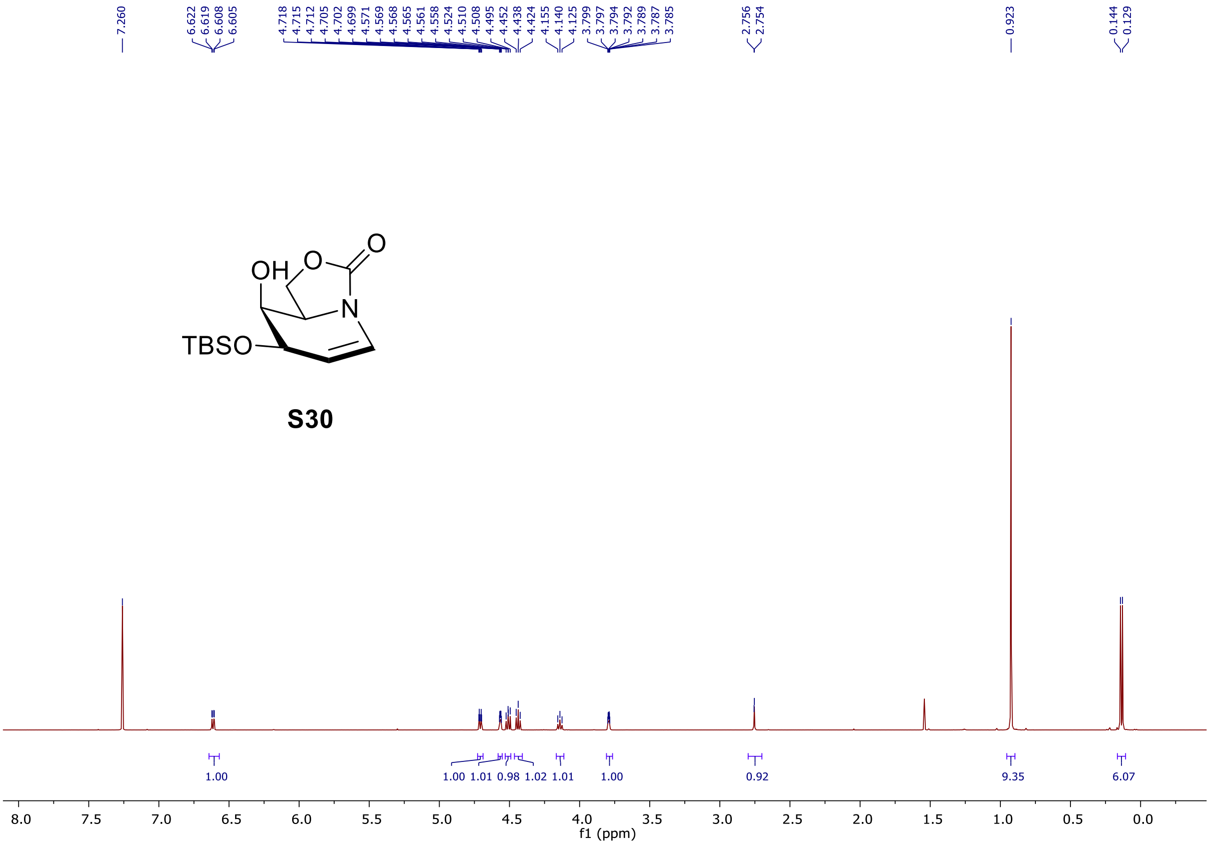

Supplementary Figure S146. ^{1}H NMR spectra for S30

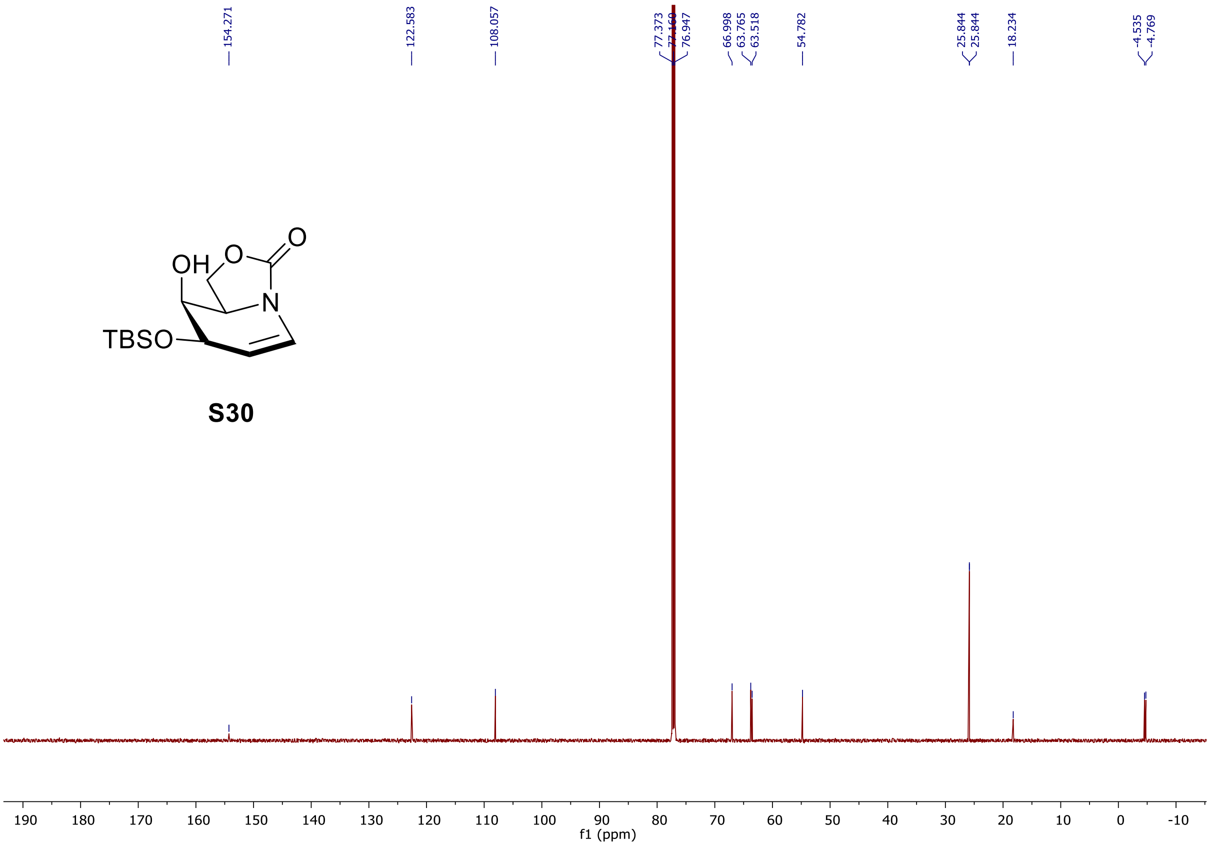

Supplementary Figure S147. ^{13}C NMR spectra for S30

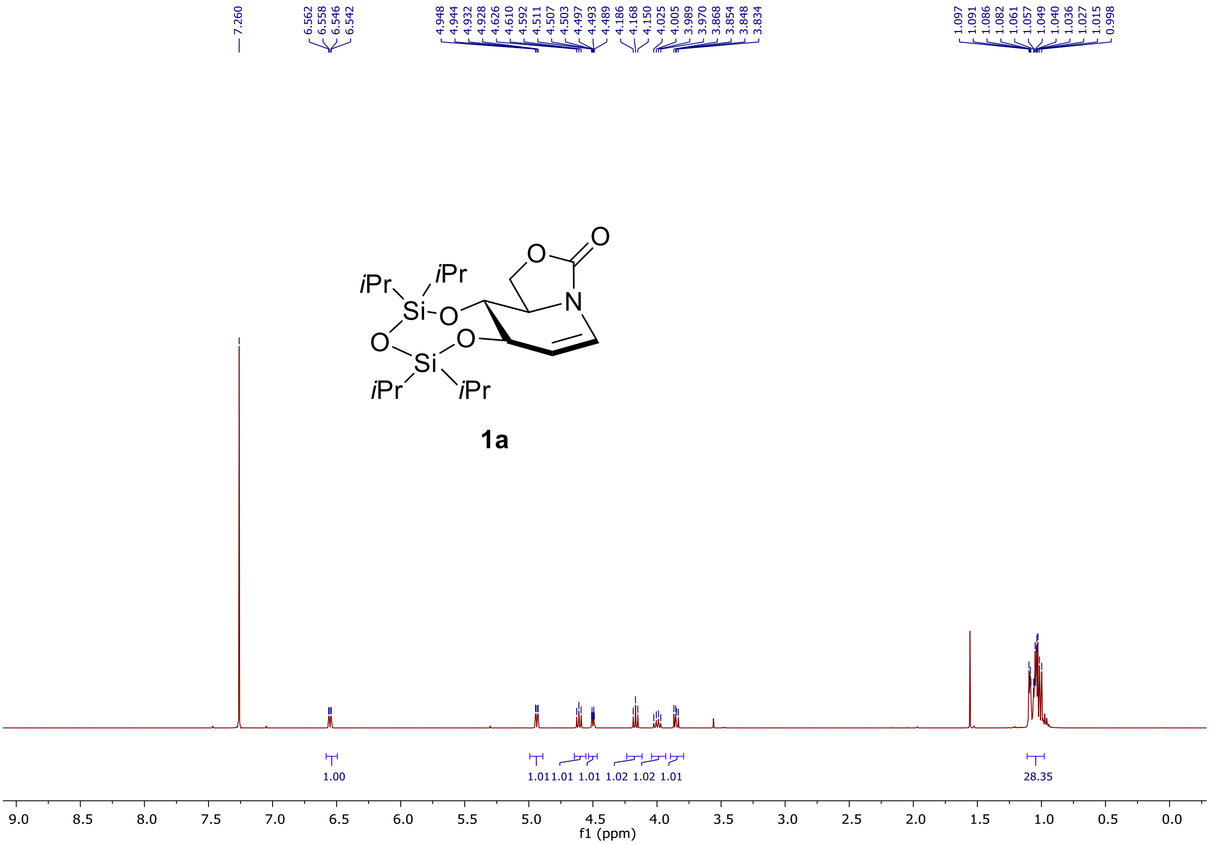

Supplementary Figure S148. ^{1}H NMR spectra for 1a

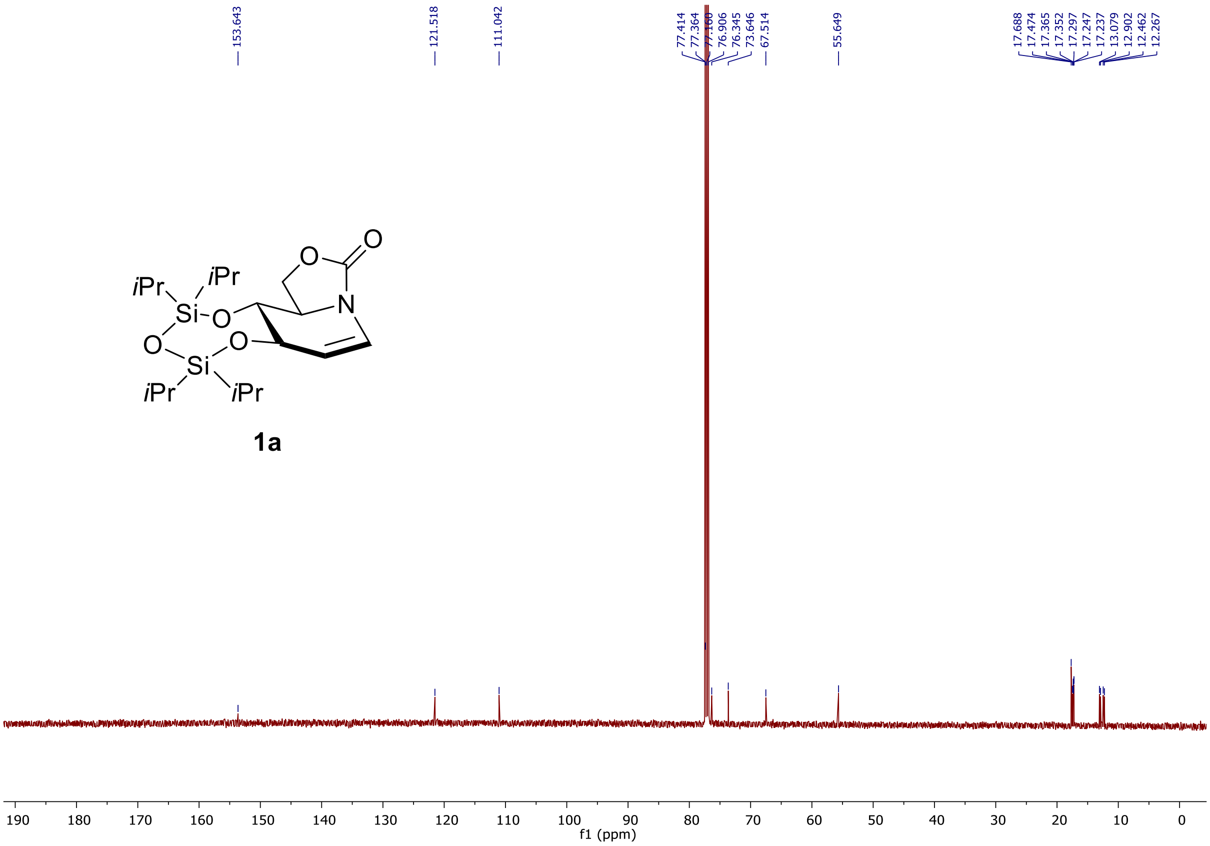

Supplementary Figure S149. ^{13}C **NMR spectra for 1a**

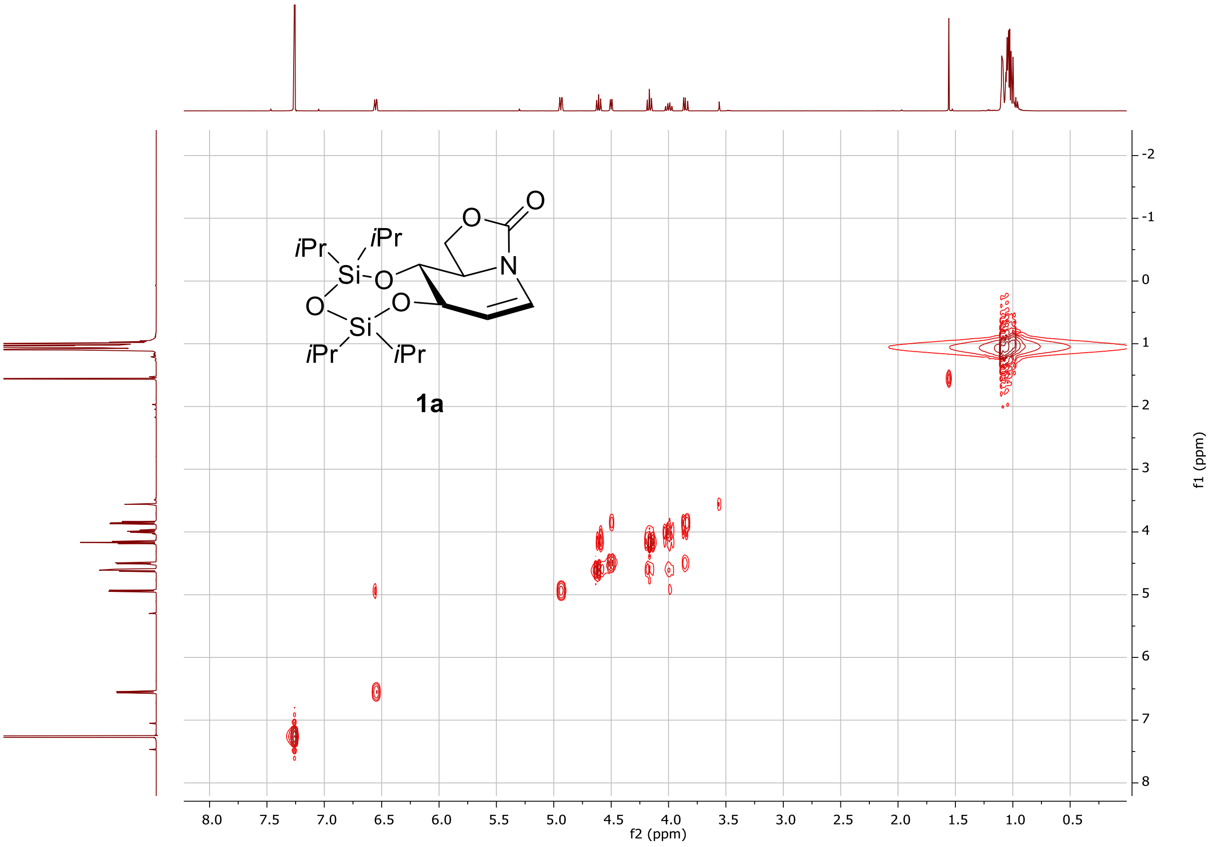

Supplementary Figure S150. COSY spectra for 1a

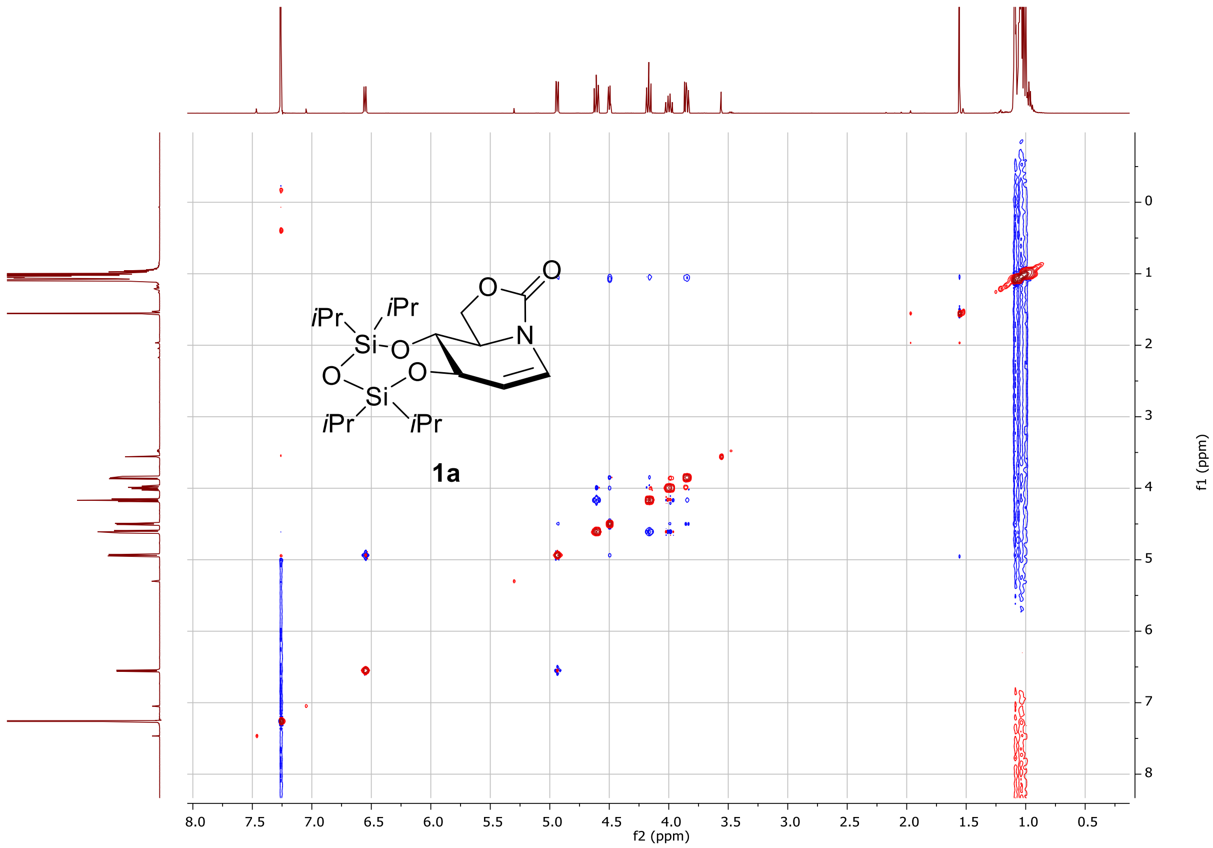

Supplementary Figure S151. NOESY spectra for 1a

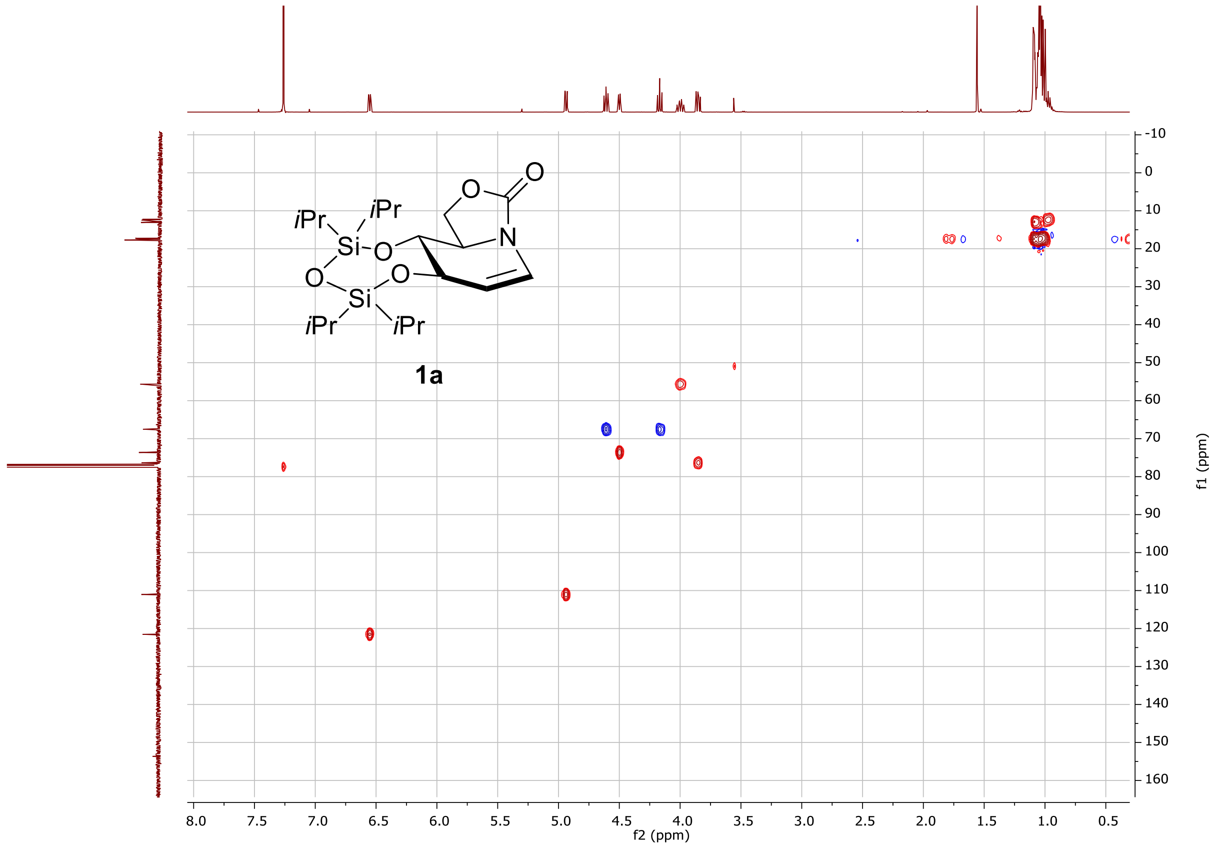

Supplementary Figure S152. HSQC spectra for 1a

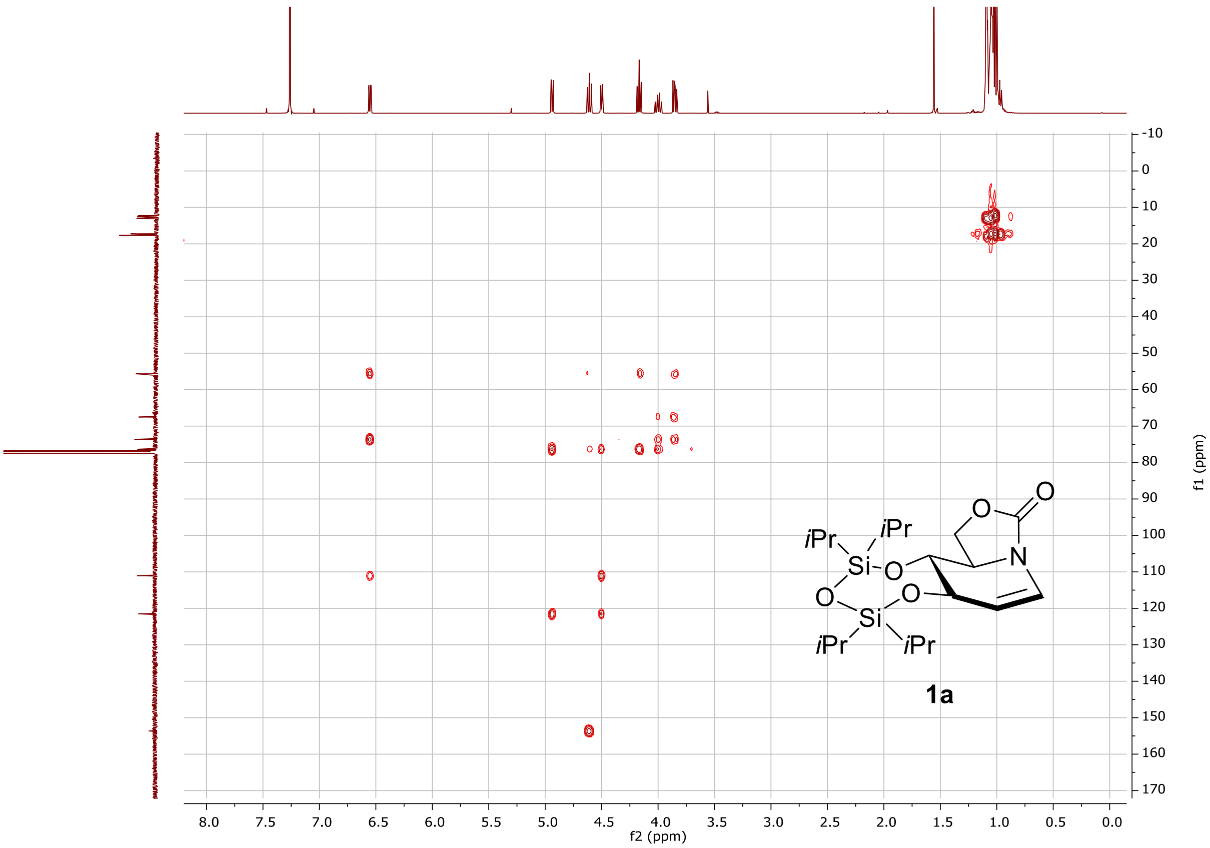

Supplementary Figure S153. HMBC spectra for 1a

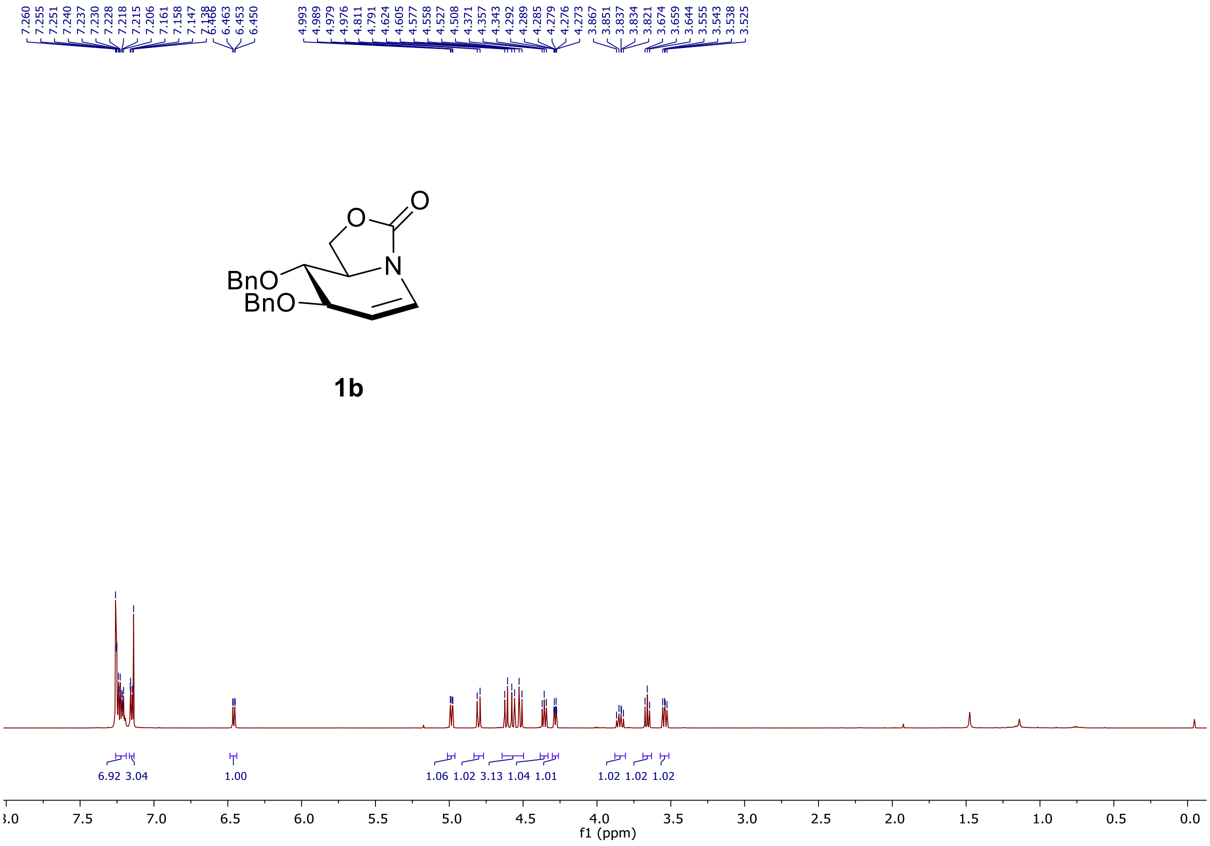

Supplementary Figure S154. ^{1}H NMR spectra for 1b

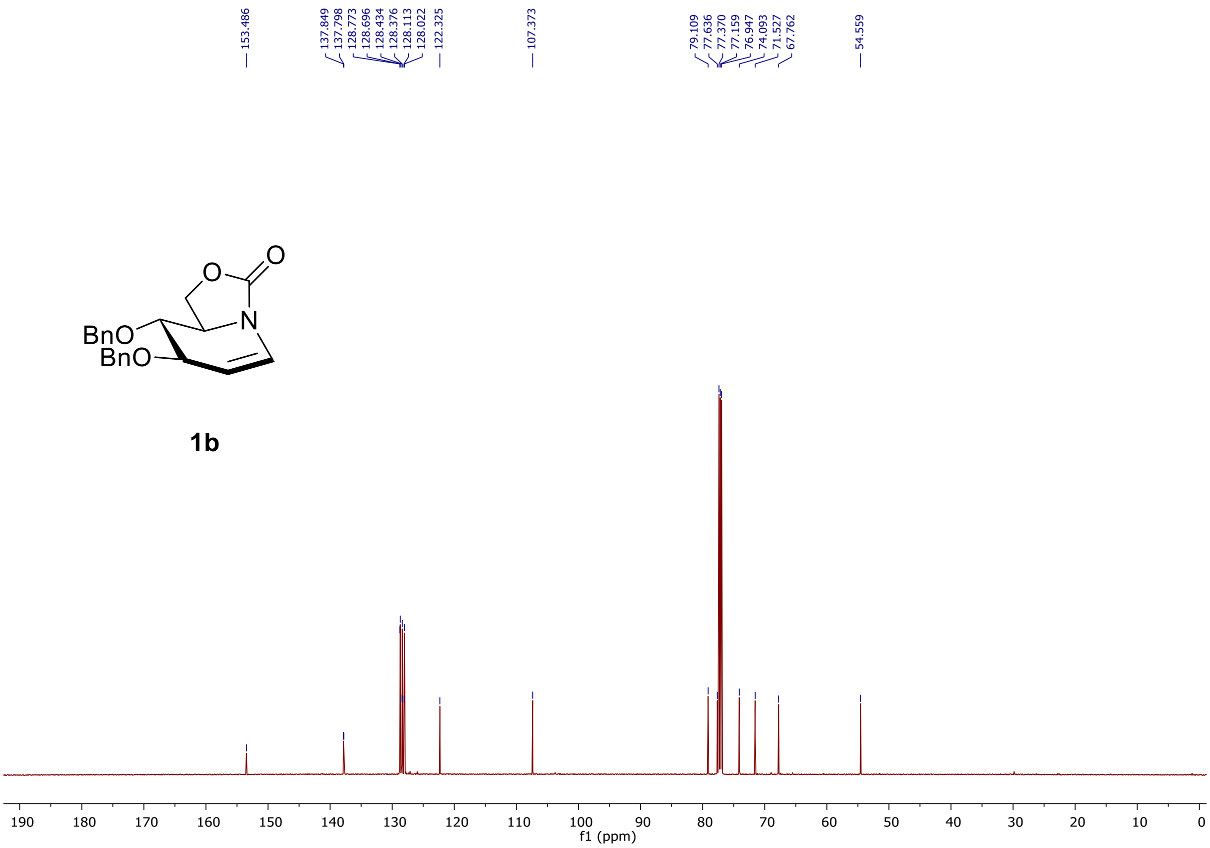

Supplementary Figure S155. ^{13}C NMR spectra for 1b

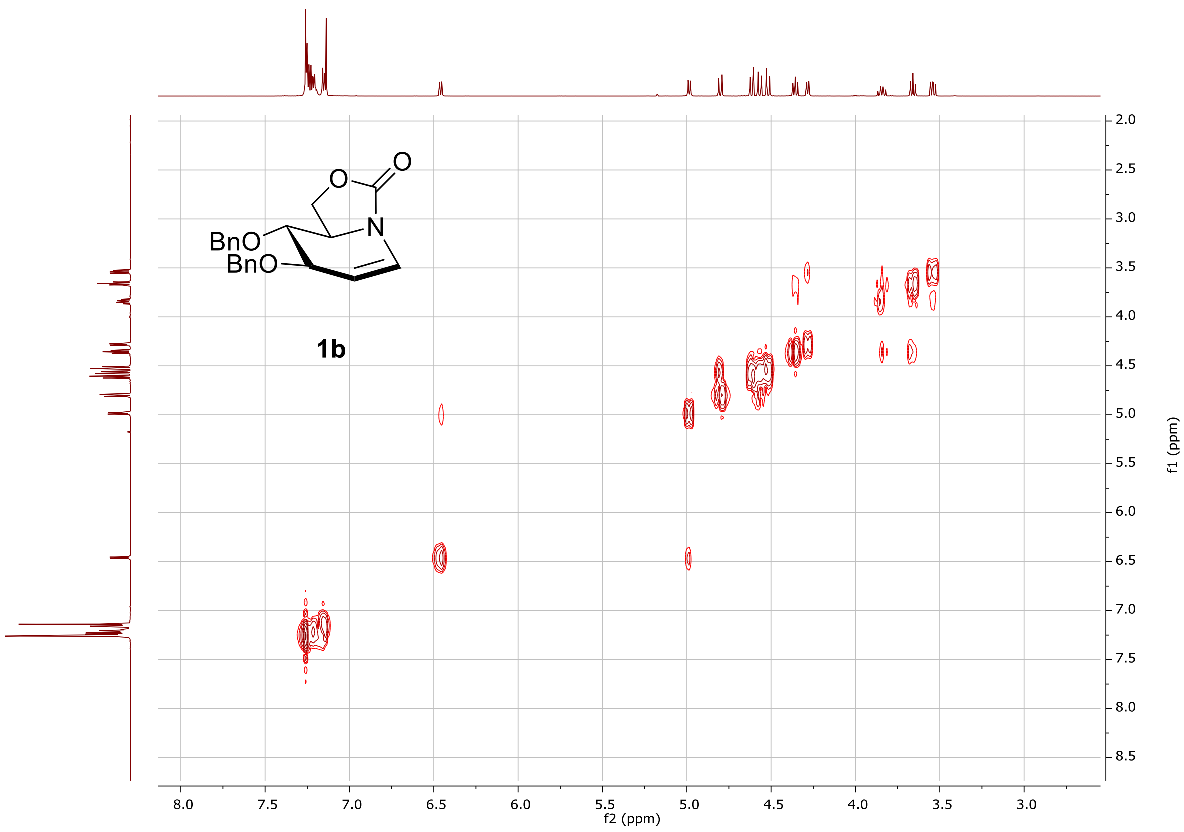

Supplementary Figure S156. COSY spectra for 1b

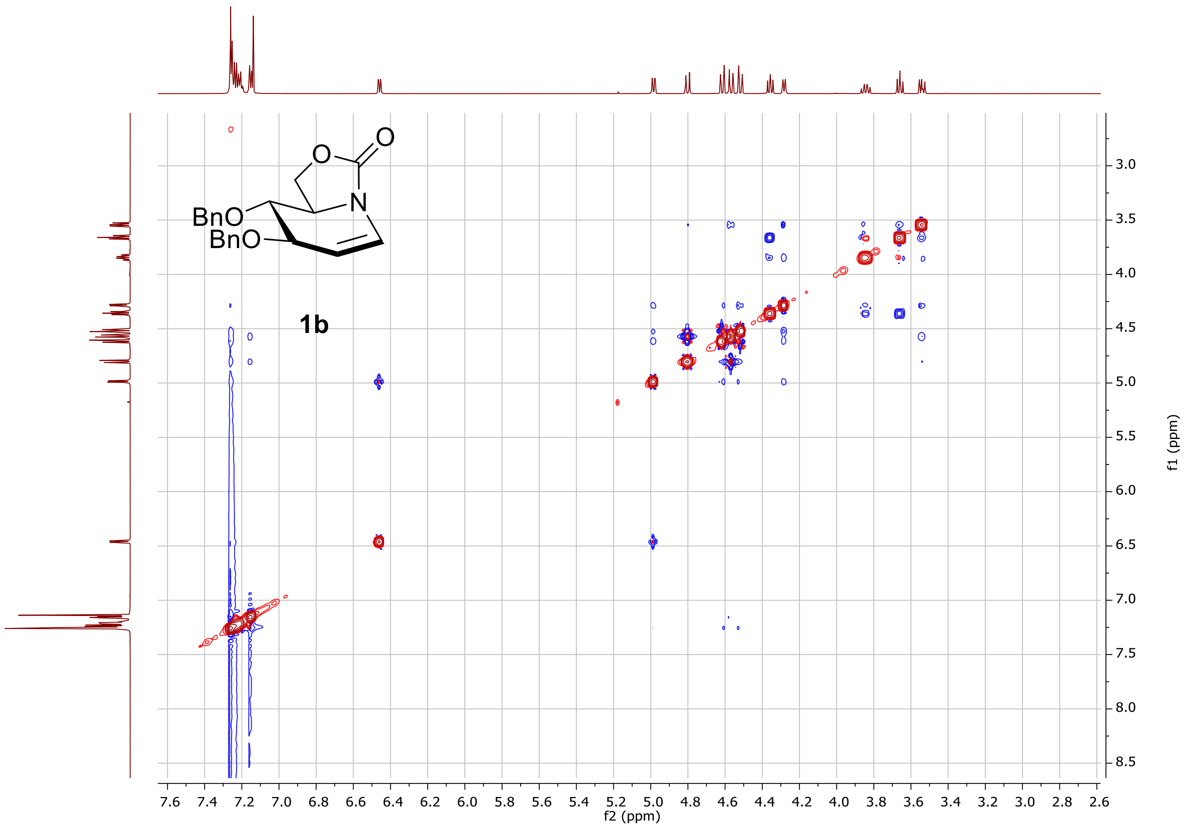

Supplementary Figure S157. NOESY spectra for 1b

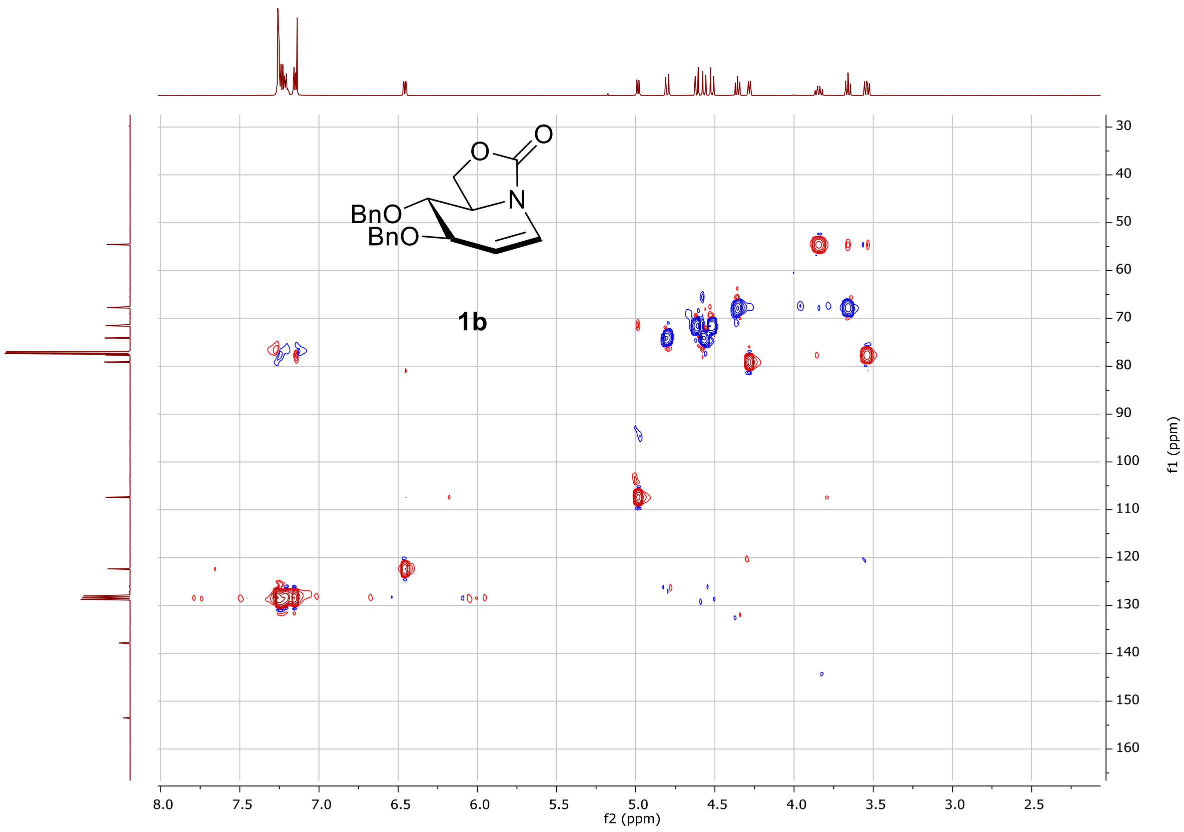

Supplementary Figure S158. HSQC spectra for 1b

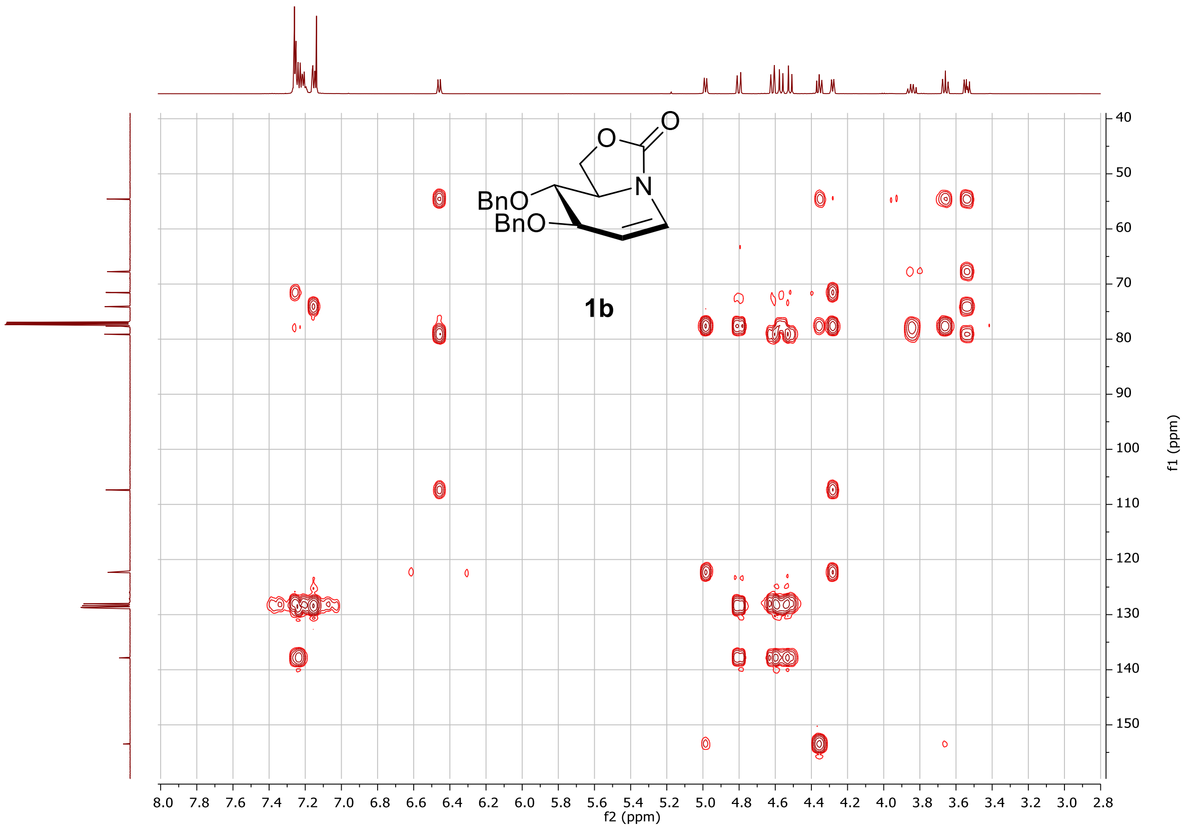

Supplementary Figure S159. HMBC spectra for 1b

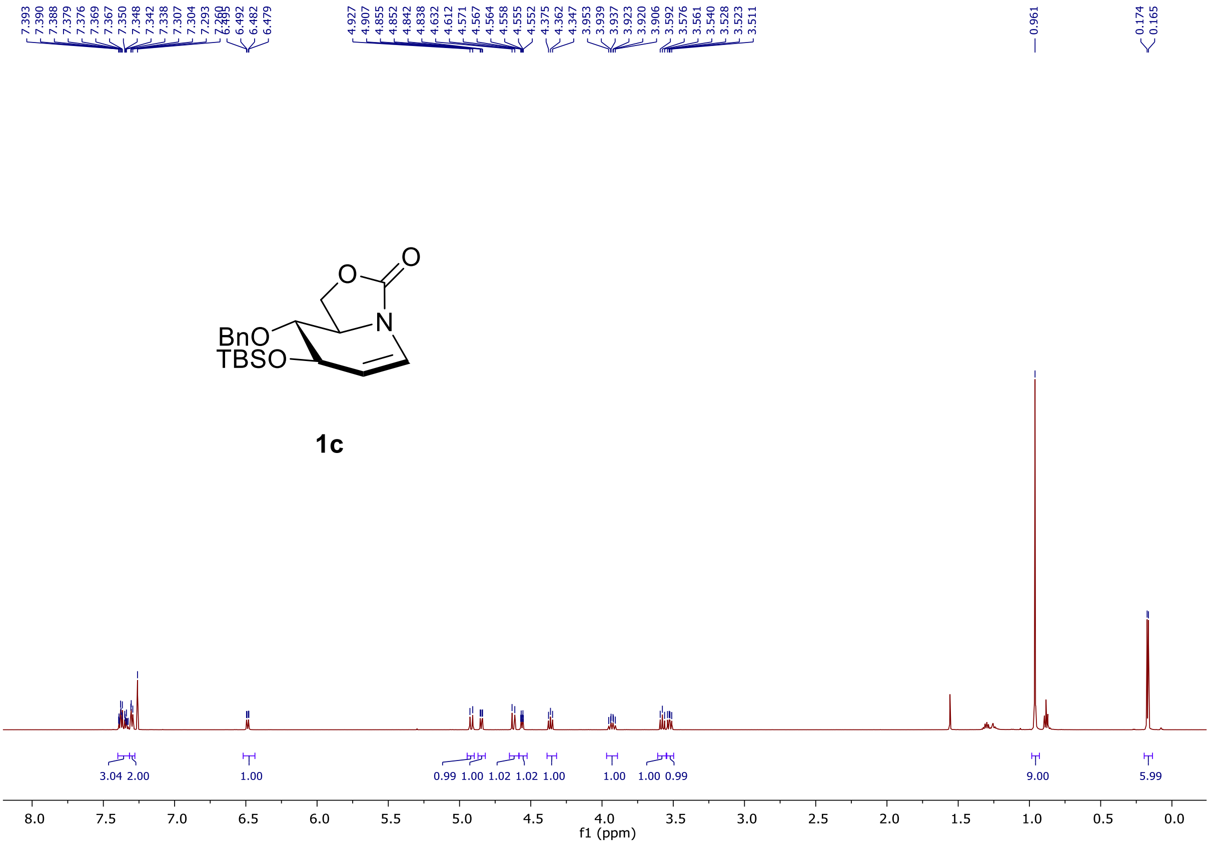

Supplementary Figure S160. ^{1}H NMR spectra for 1c

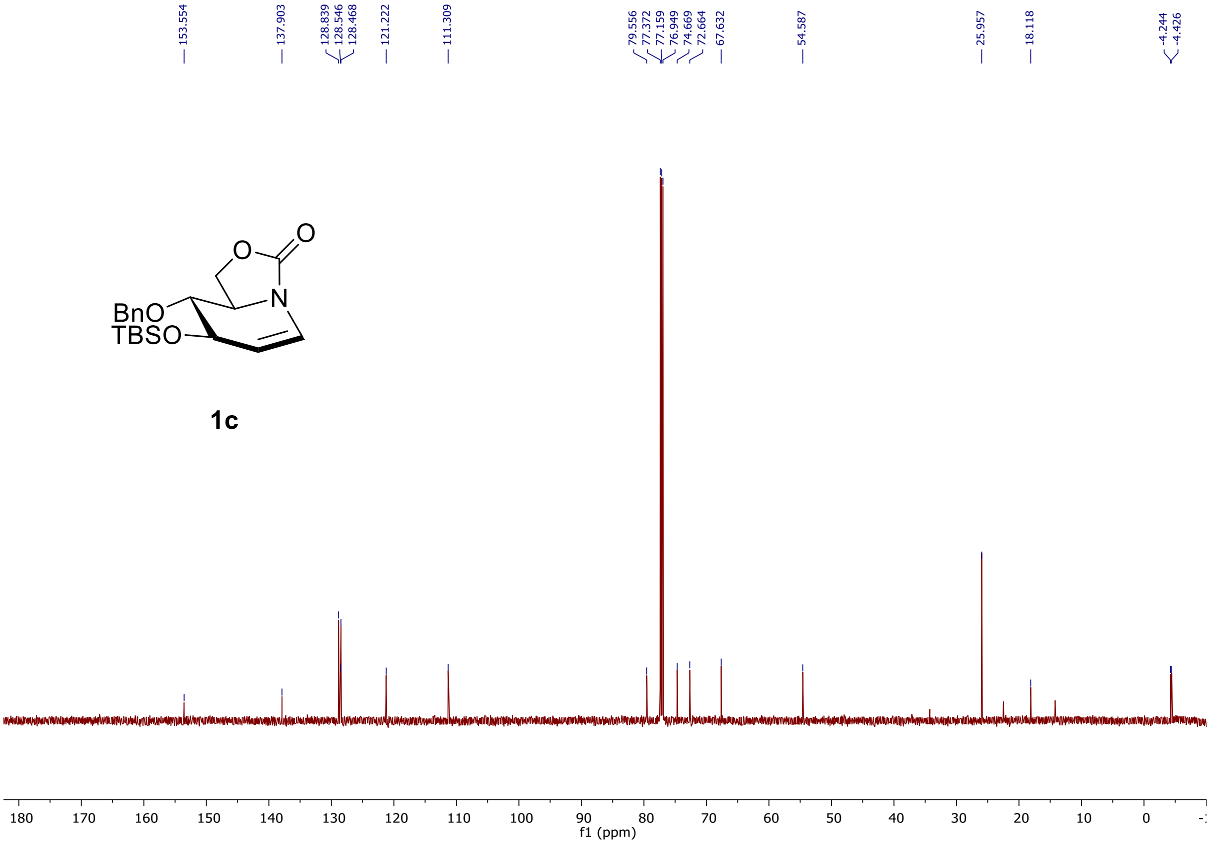

Supplementary Figure S161. ^{13}C **NMR spectra for 1c**

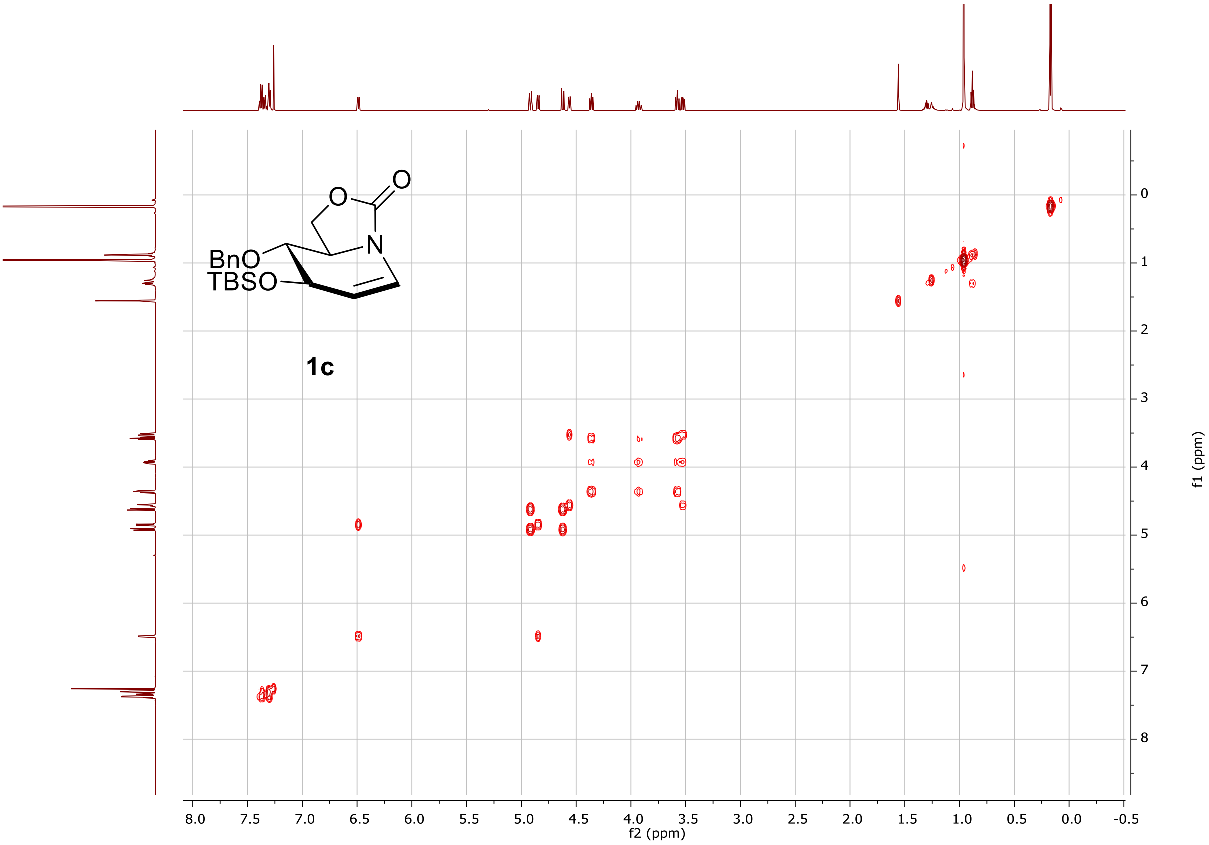

Supplementary Figure S162. COSY spectra for 1c

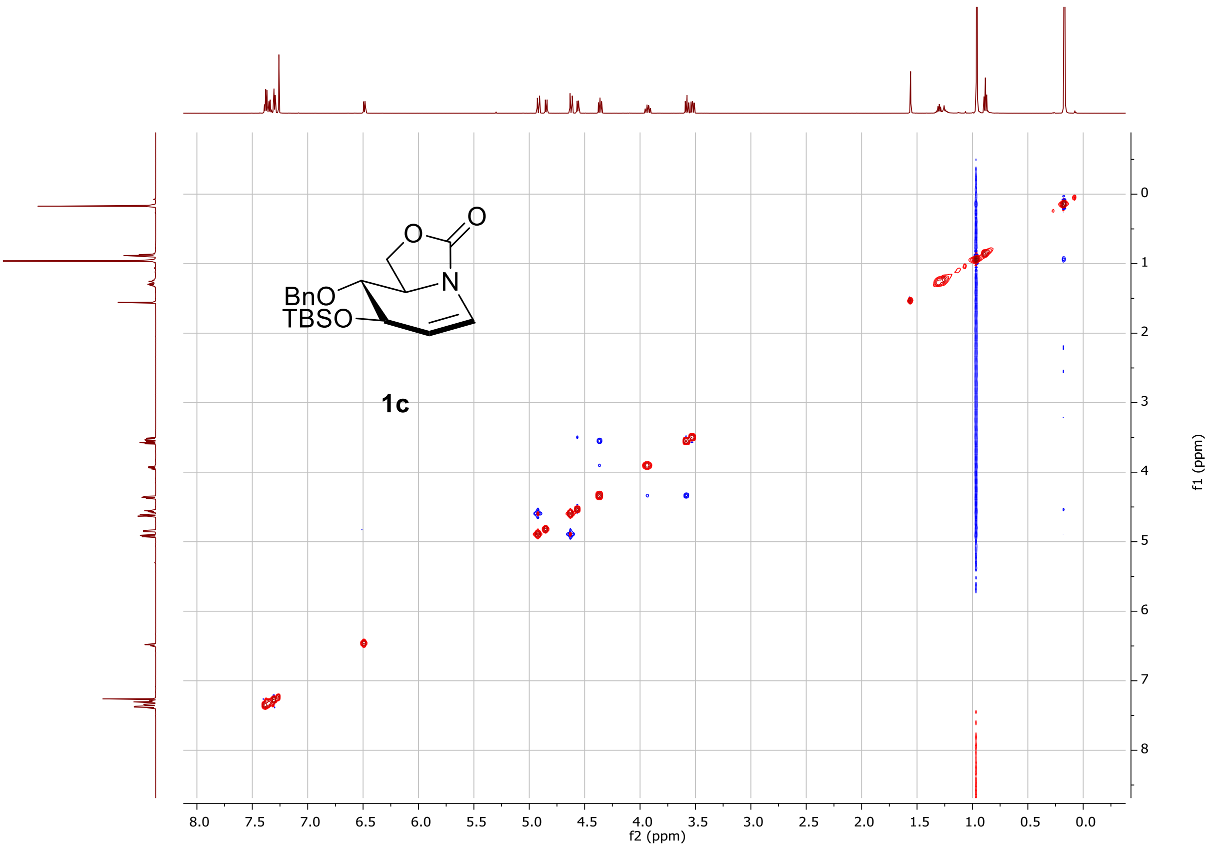

Supplementary Figure S163. NOESY spectra for 1c

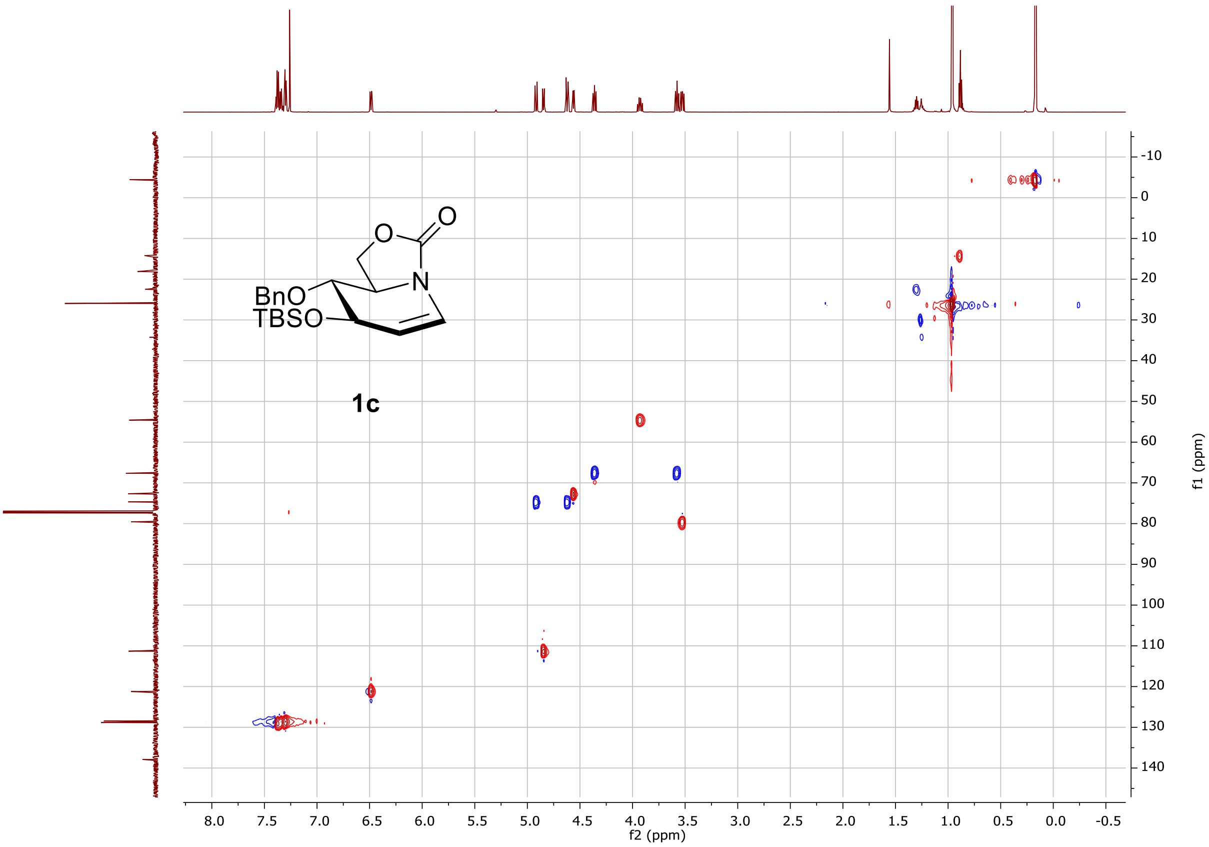

Supplementary Figure S164. HSQC spectra for 1c

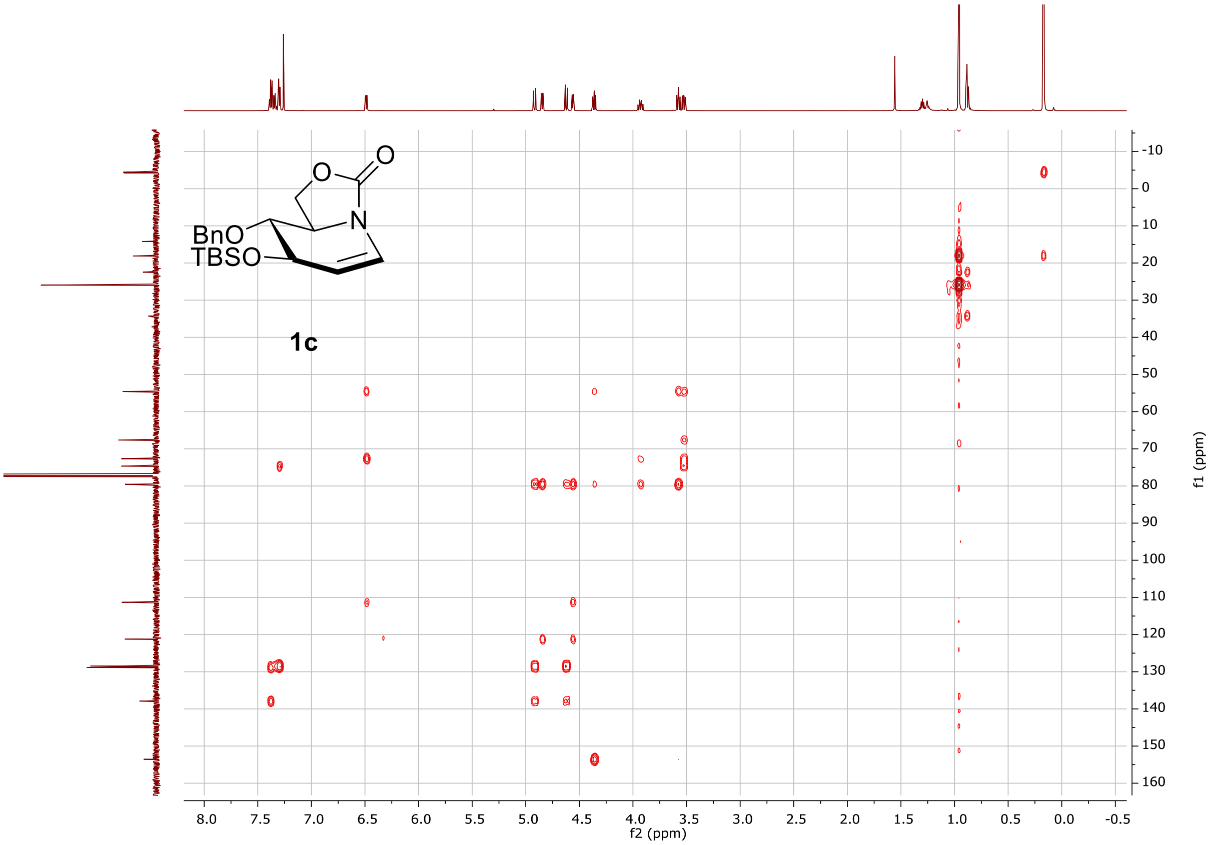

Supplementary Figure S165. HMBC spectra for 1c

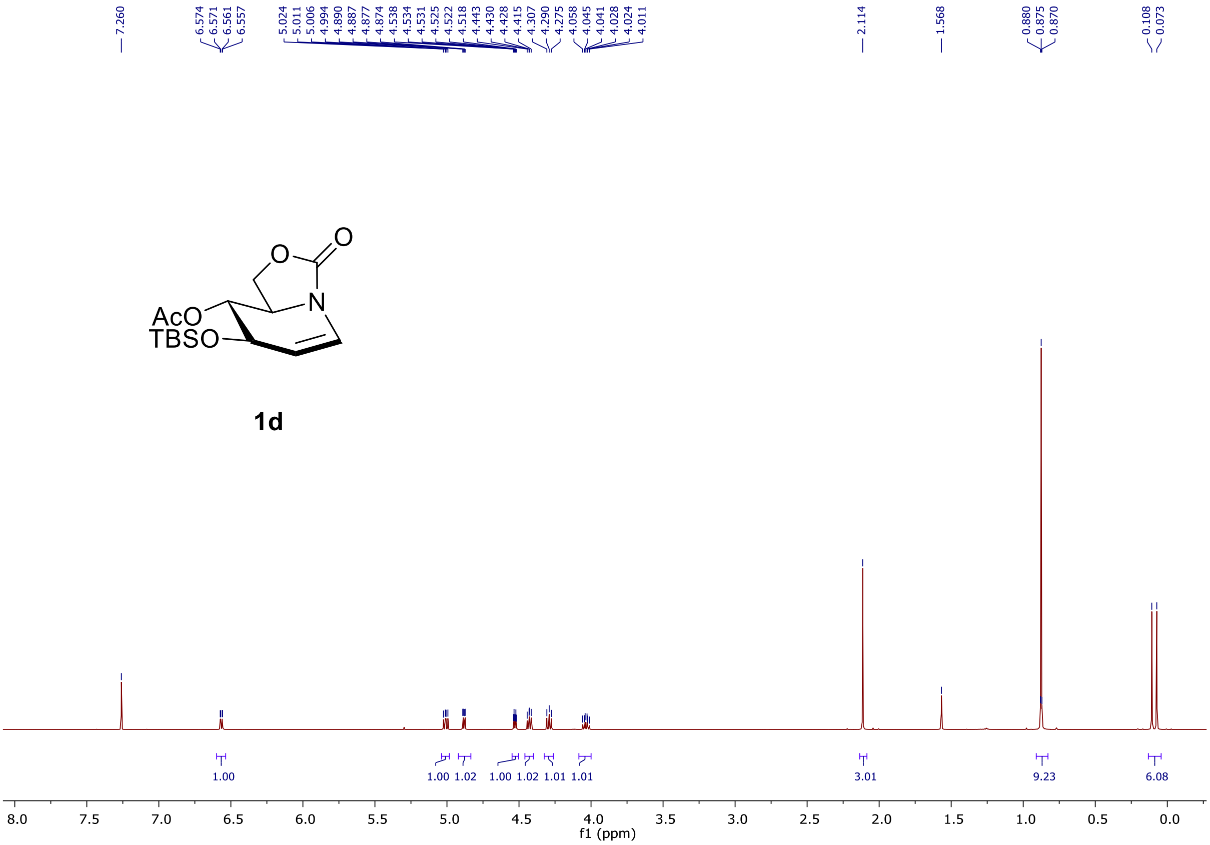

Supplementary Figure S166. ^{1}H NMR spectra for 1d

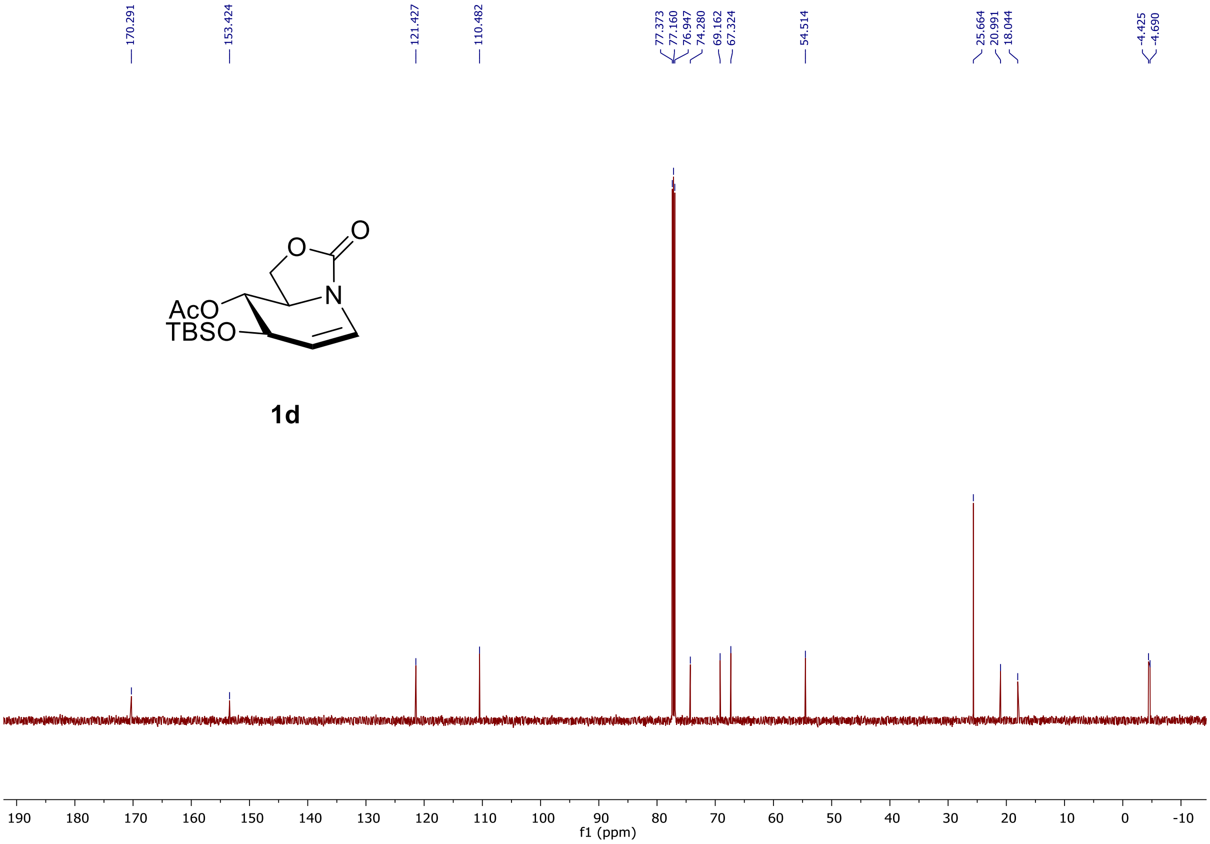

Supplementary Figure S167. ^{13}C NMR spectra for 1d

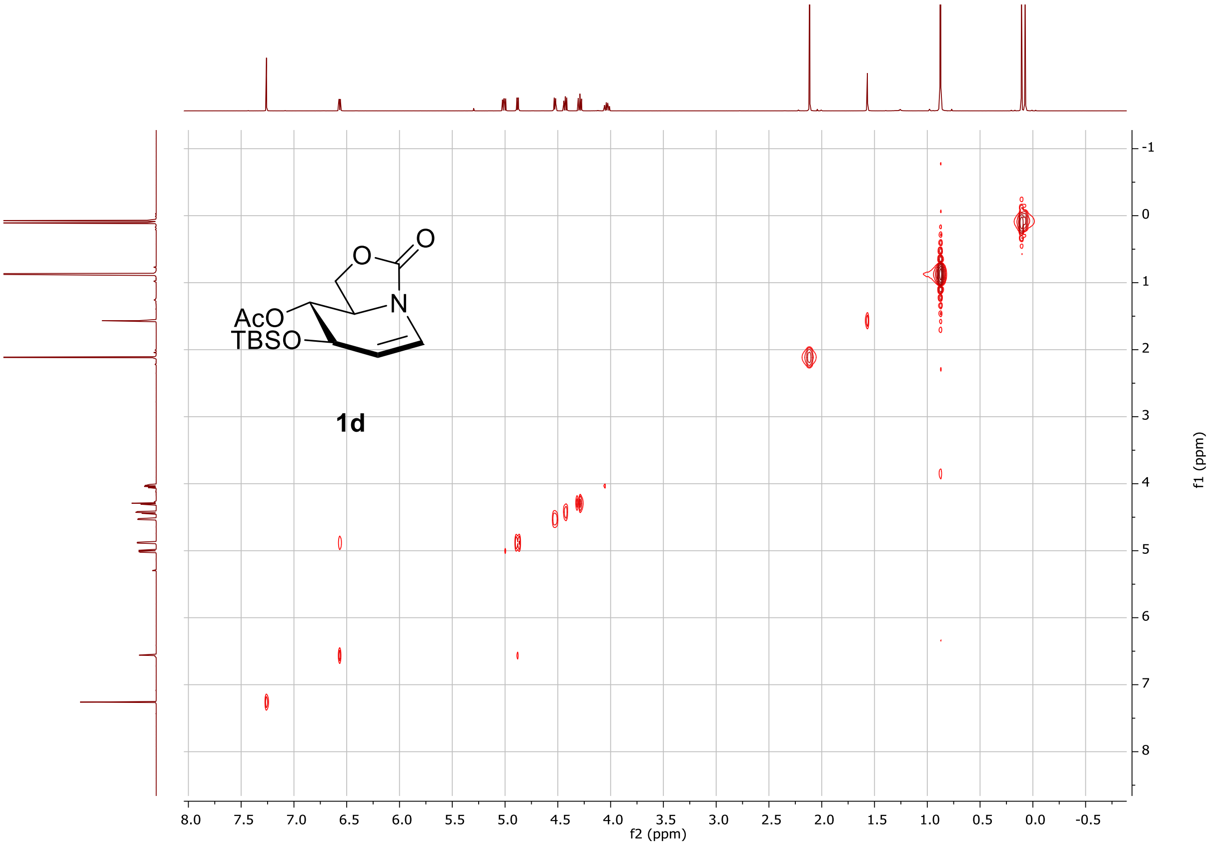

Supplementary Figure S168. COSY spectra for 1d

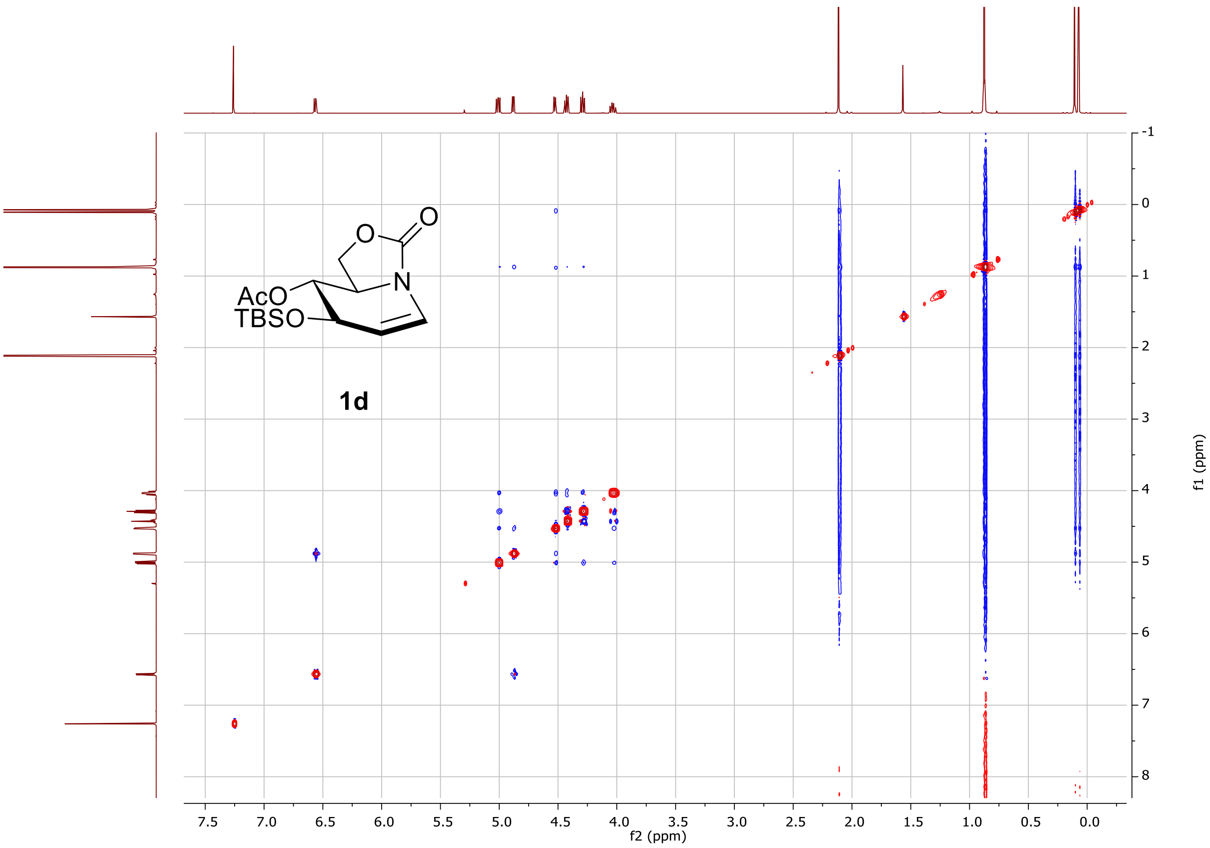

Supplementary Figure S169. NOESY spectra for 1d

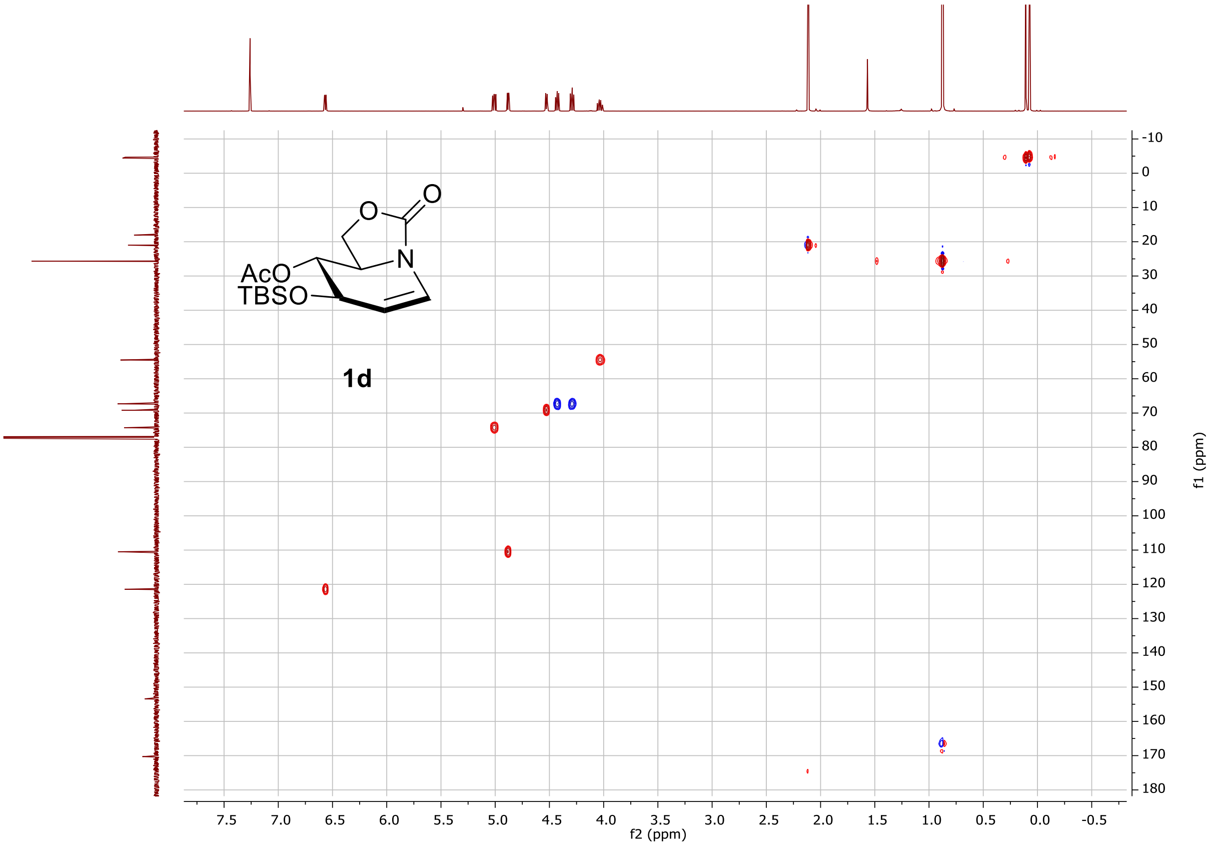

Supplementary Figure S170. HSQC spectra for 1d

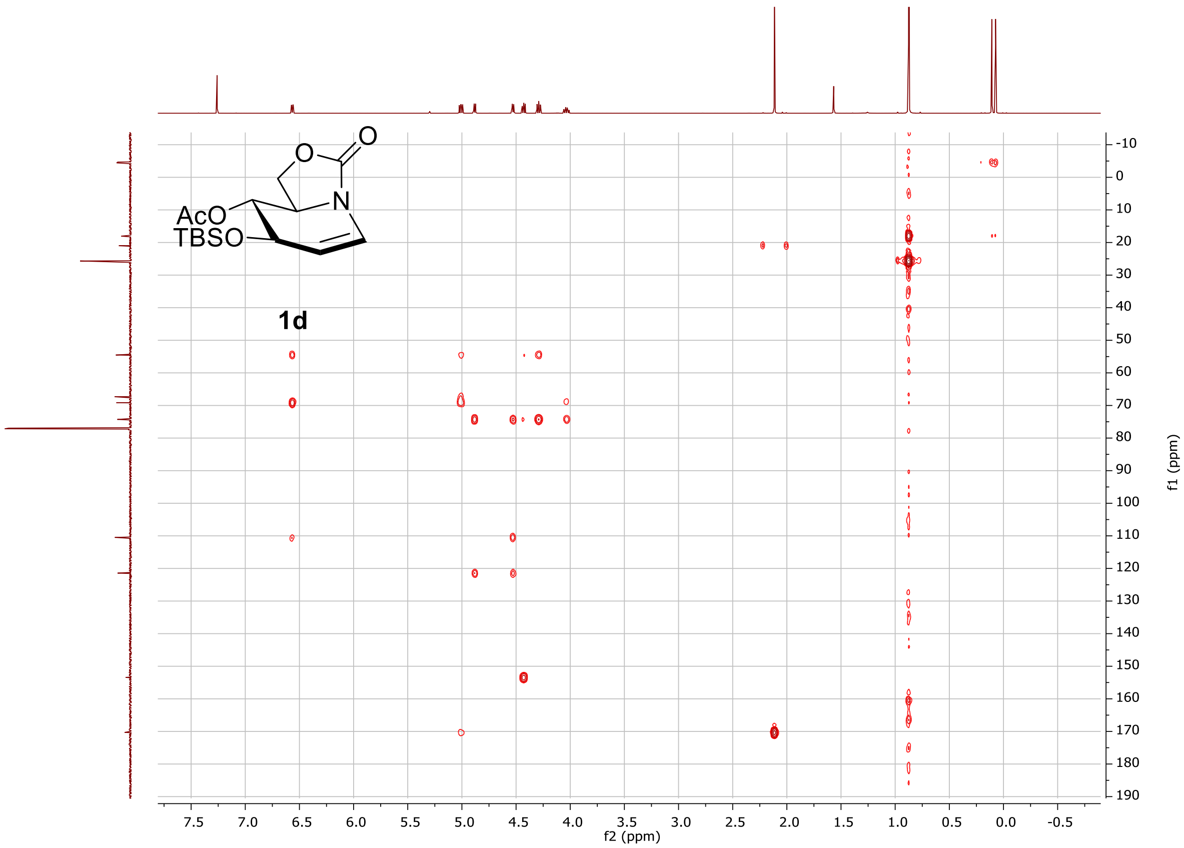

Supplementary Figure S171. HMBC spectra for 1d

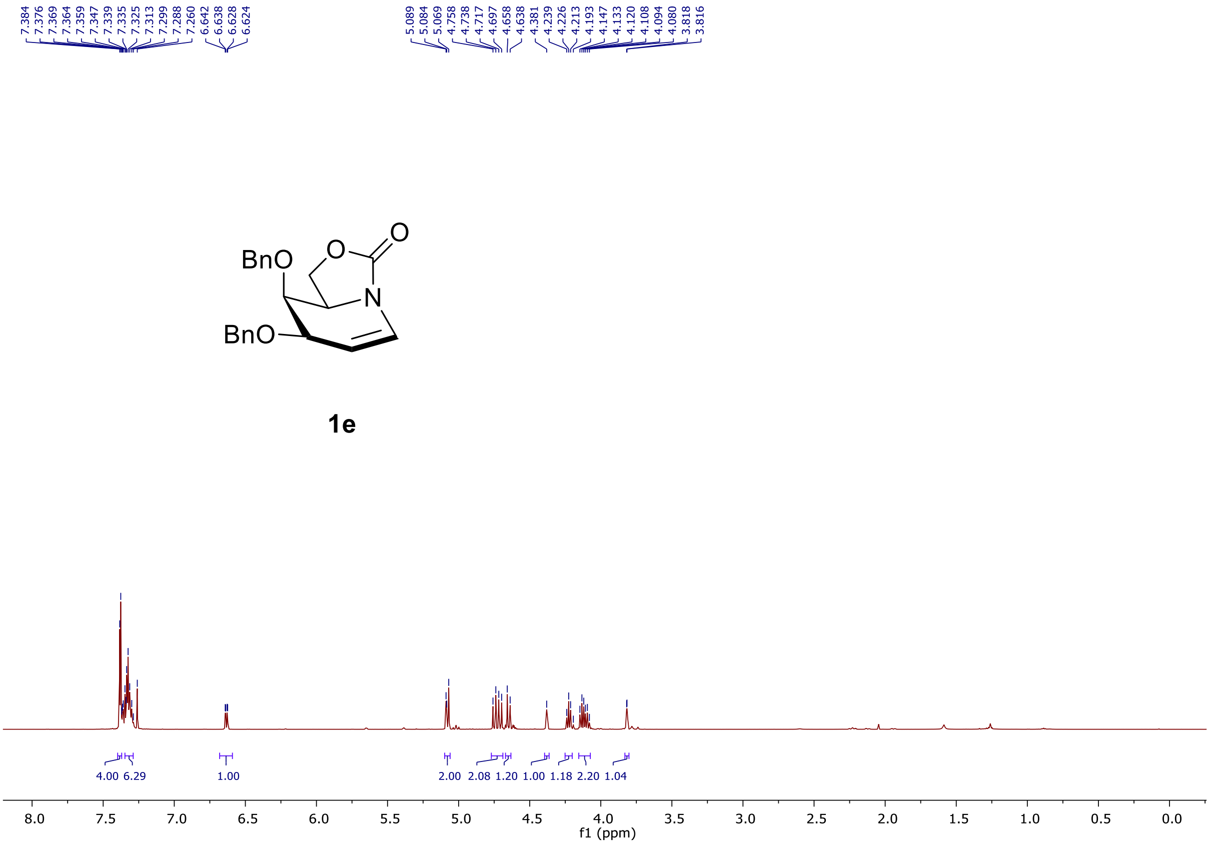

Supplementary Figure S172. ^{1}H NMR spectra for 1e

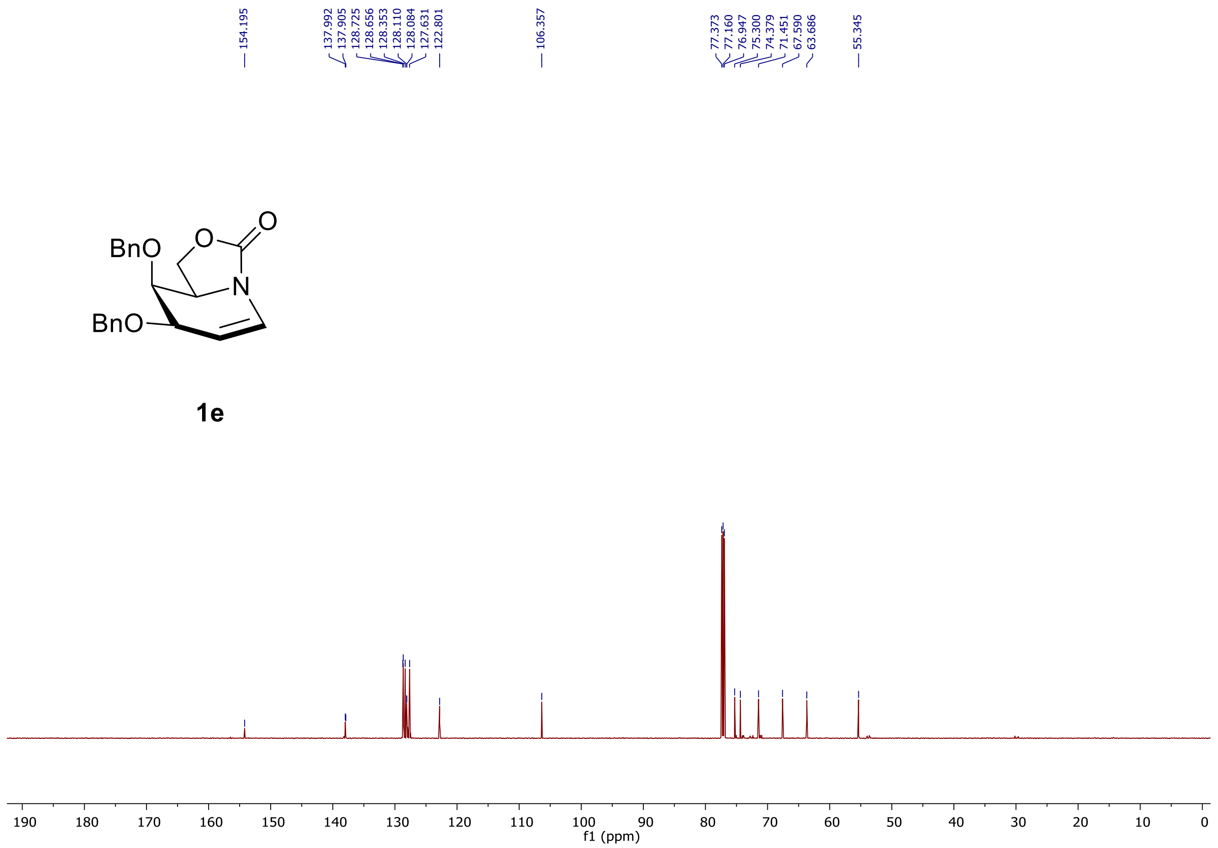

Supplementary Figure S173. ^{13}C NMR spectra for 1e

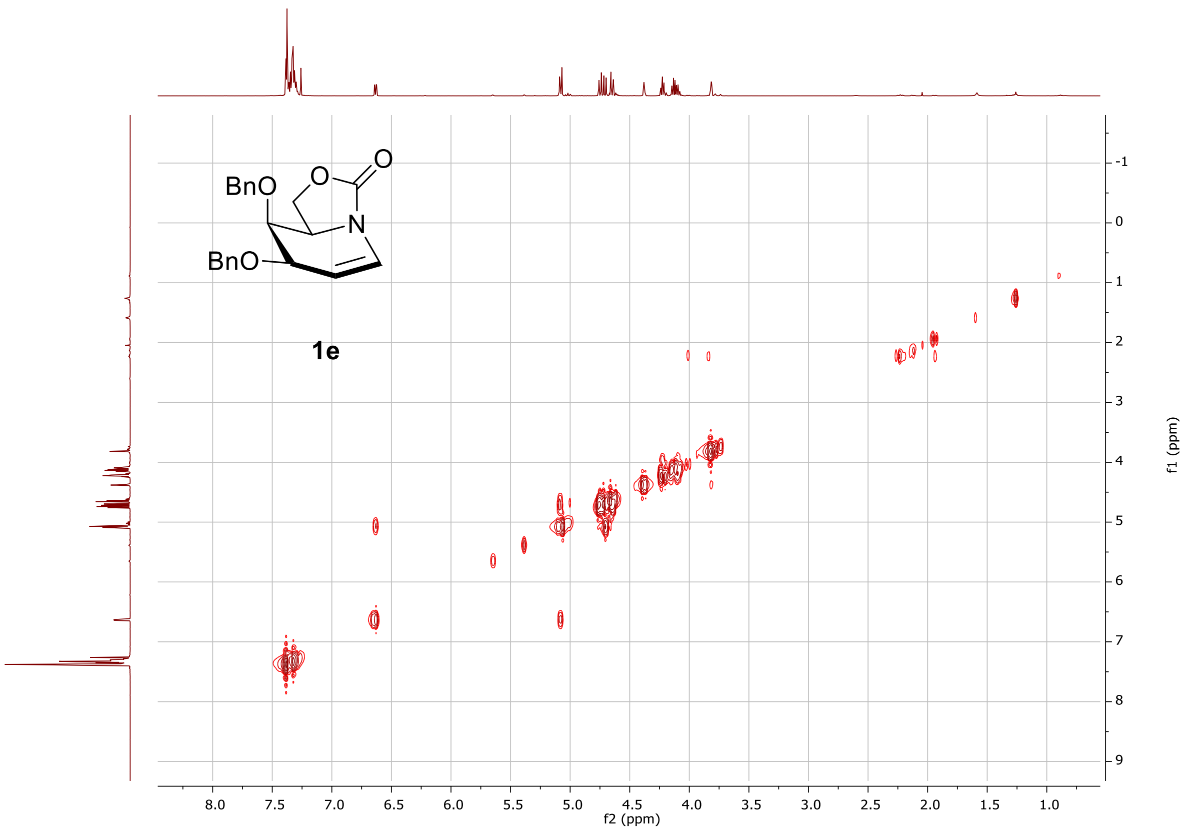

Supplementary Figure S174. COSY spectra for 1e

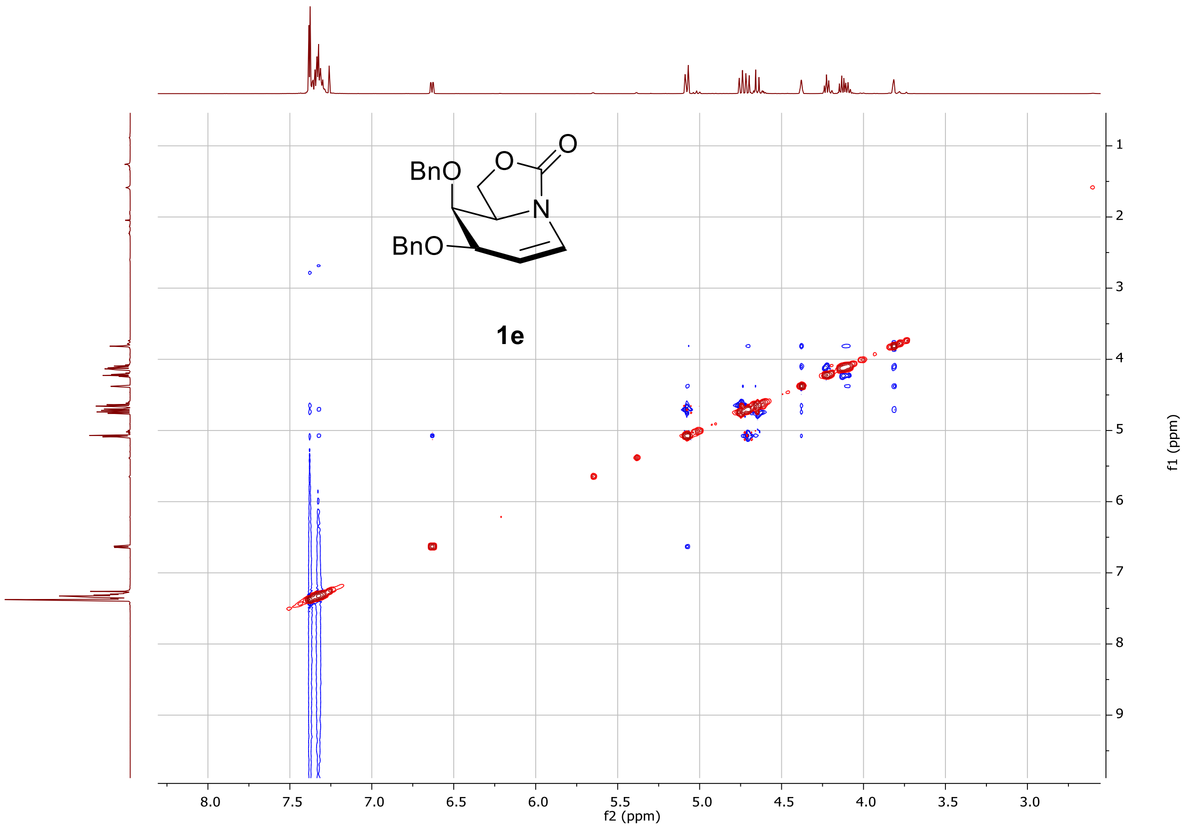

Supplementary Figure S175. NOESY spectra for 1e

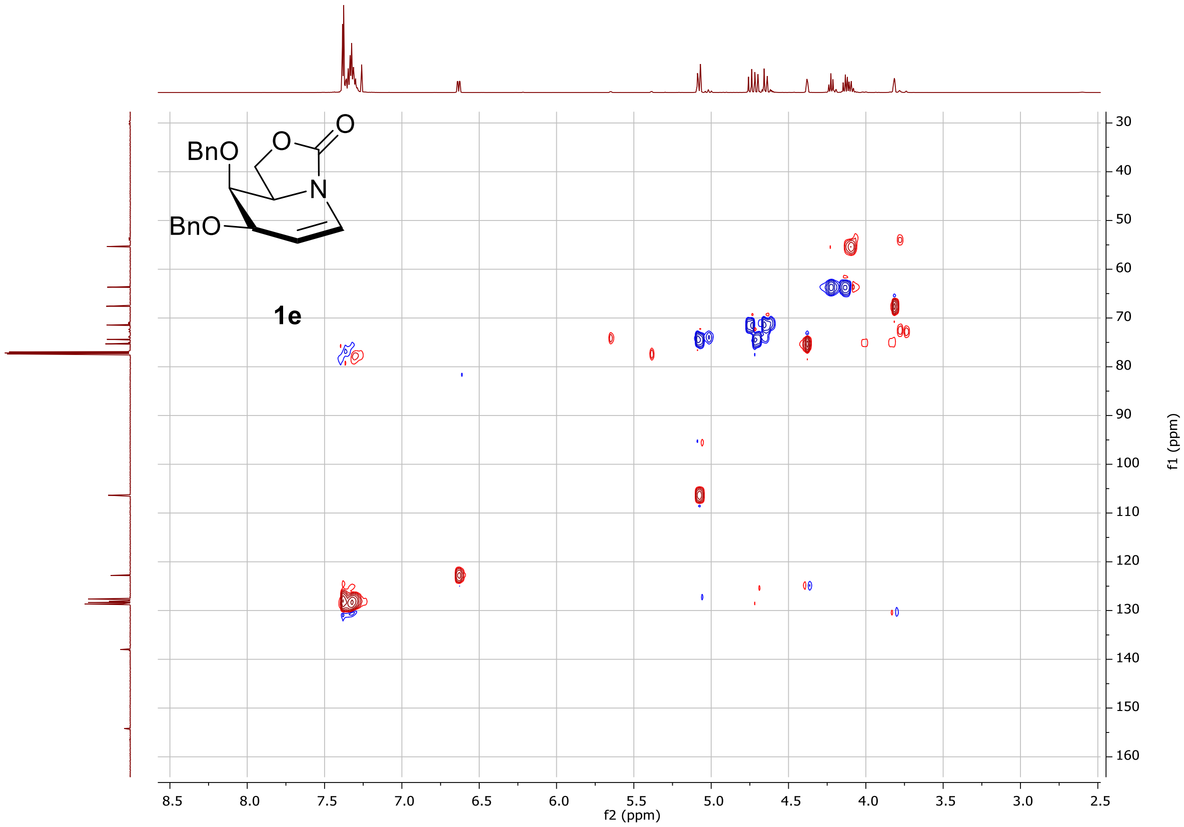

Supplementary Figure S176. HSQC spectra for 1e

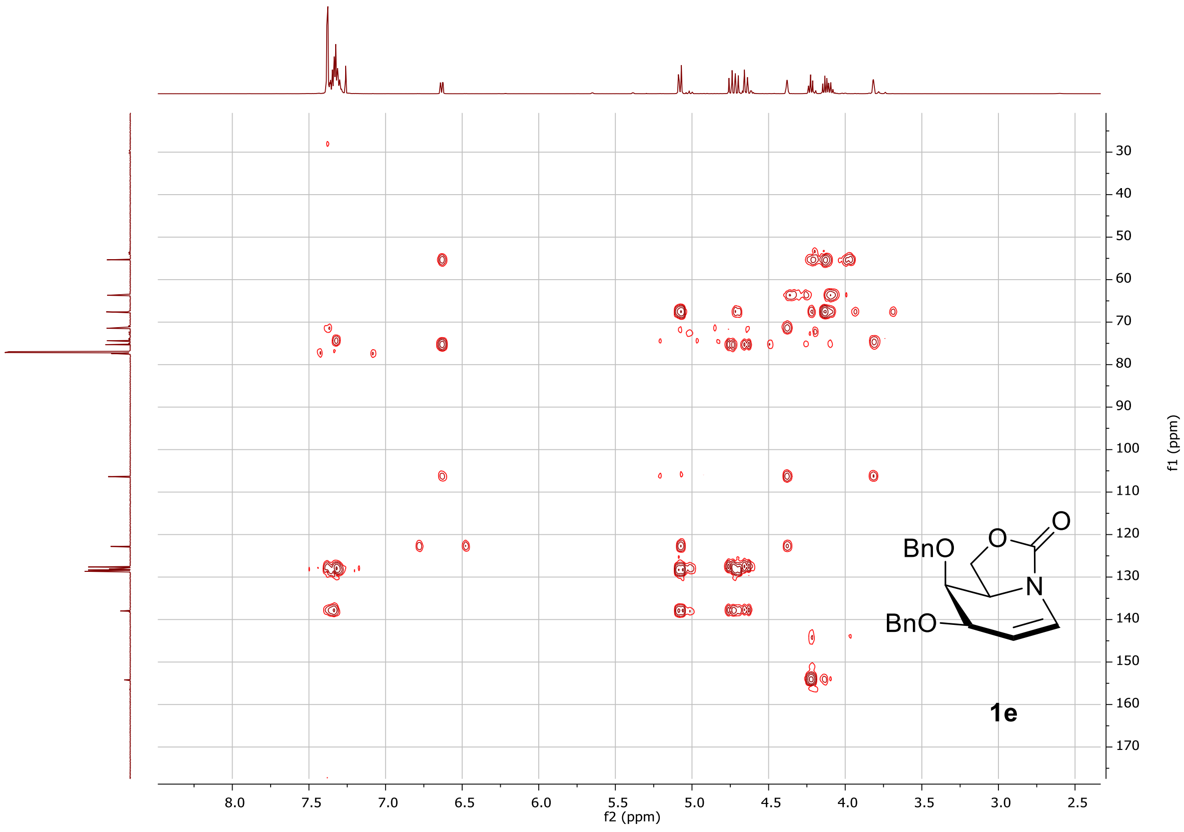

Supplementary Figure S177. HMBC spectra for 1e

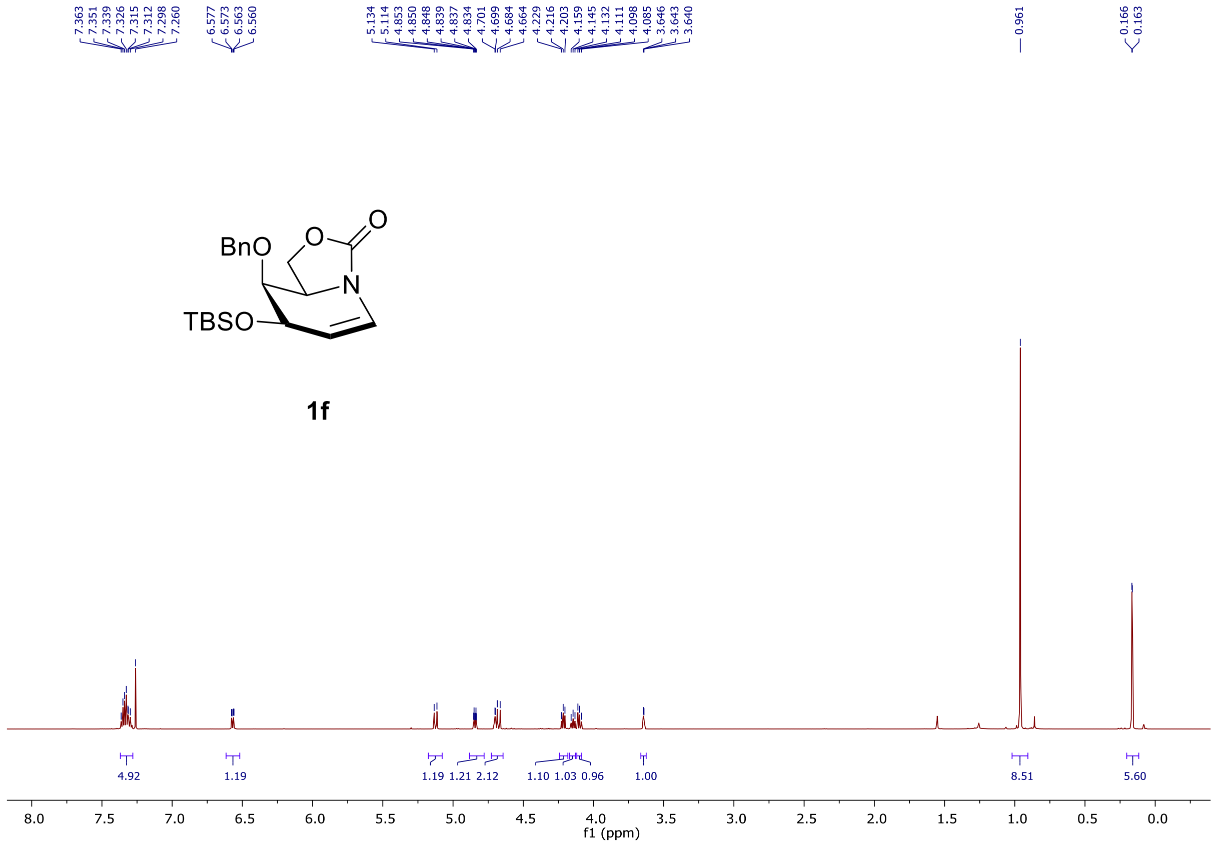

Supplementary Figure S178. ^{1}H NMR spectra for 1f

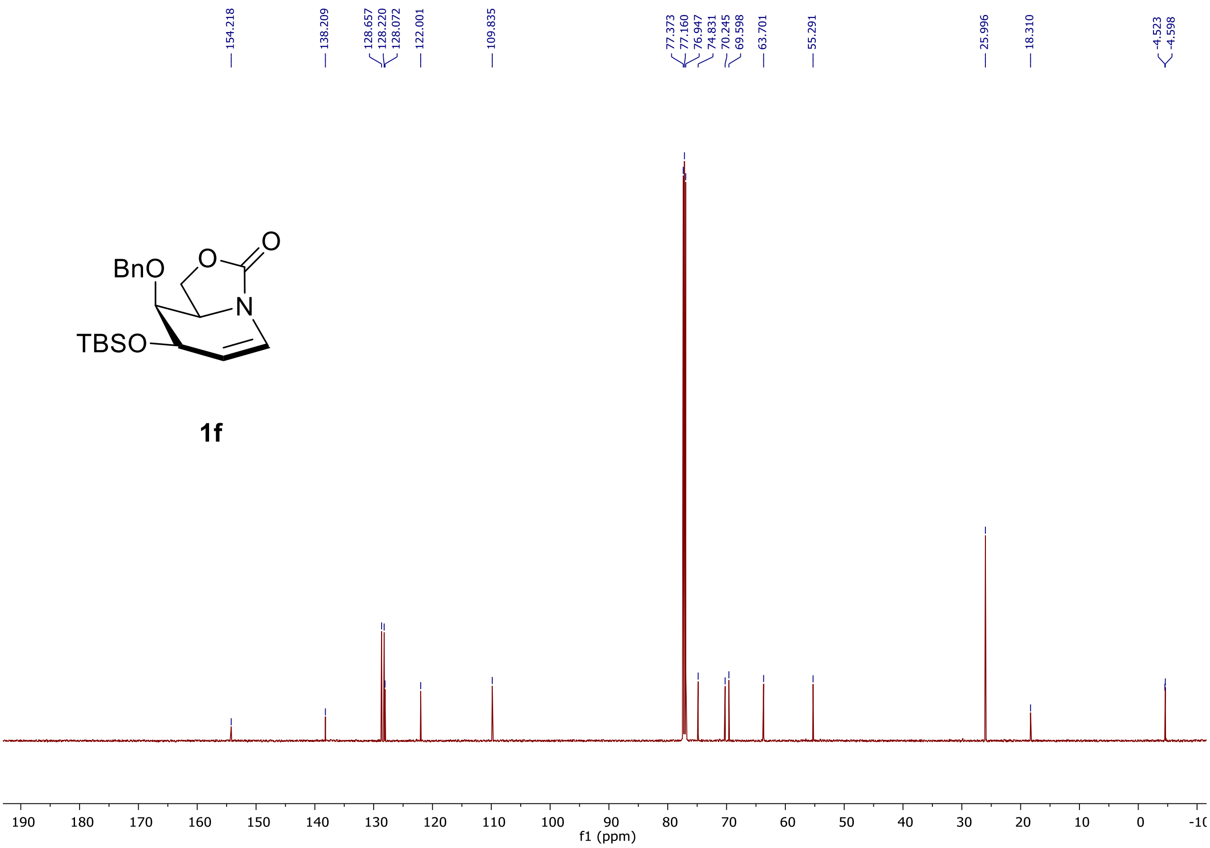

Supplementary Figure S179. ^{13}**C NMR spectra for 1f**

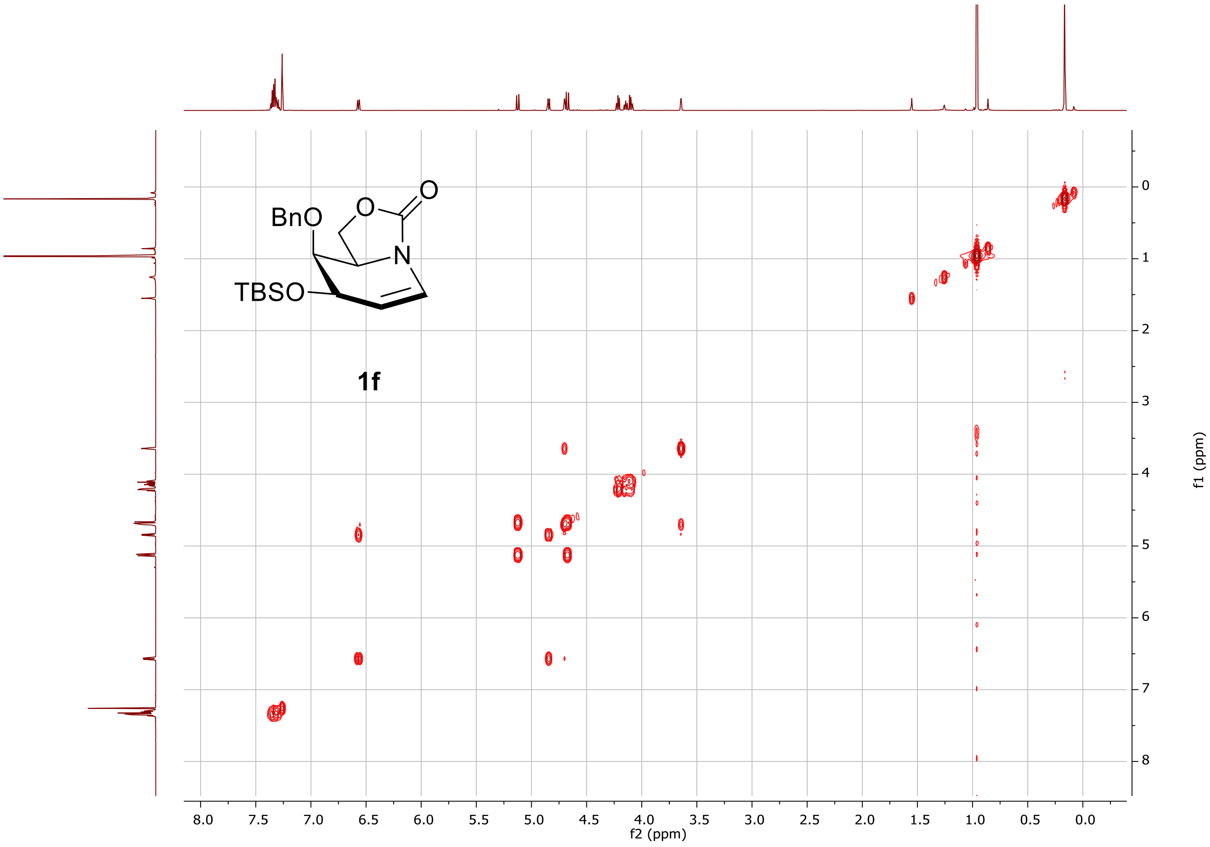

Supplementary Figure S180. COSY spectra for 1f

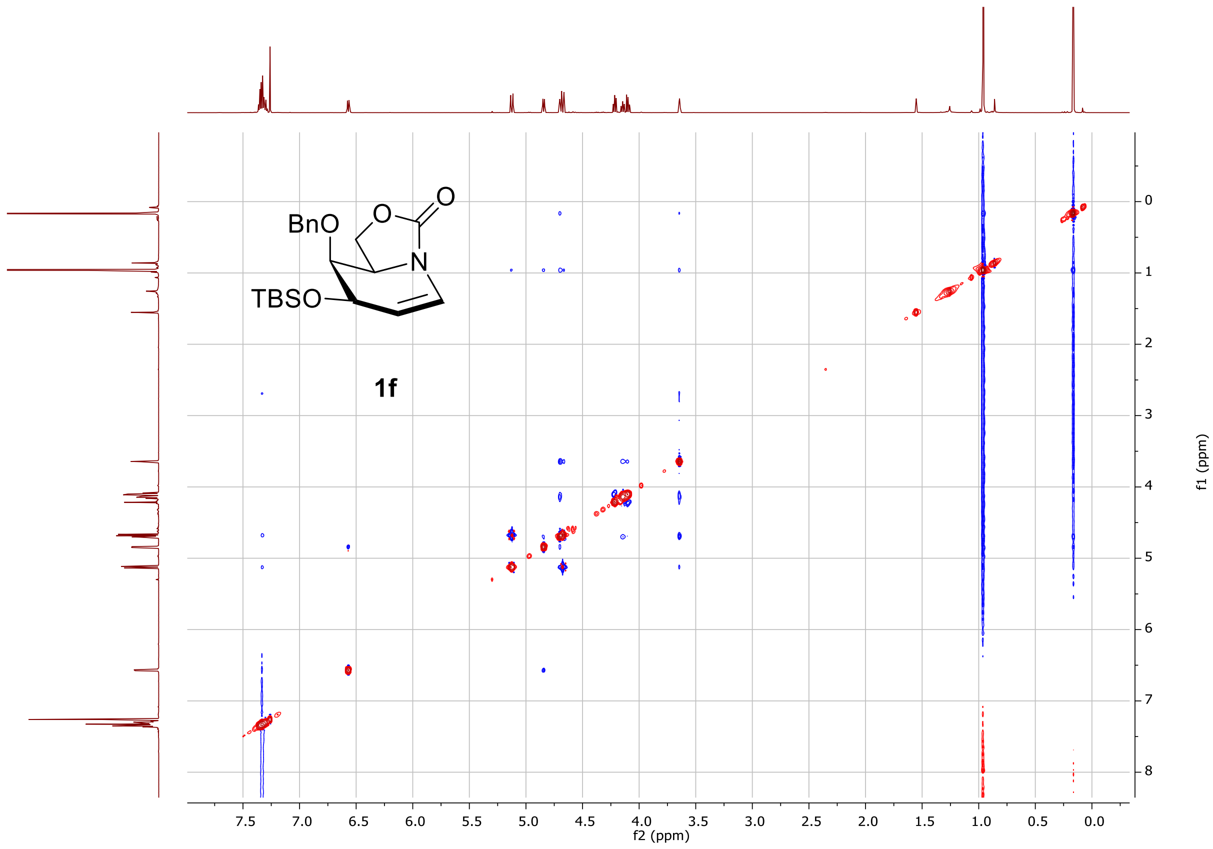

Supplementary Figure S181. NOESY spectra for 1f

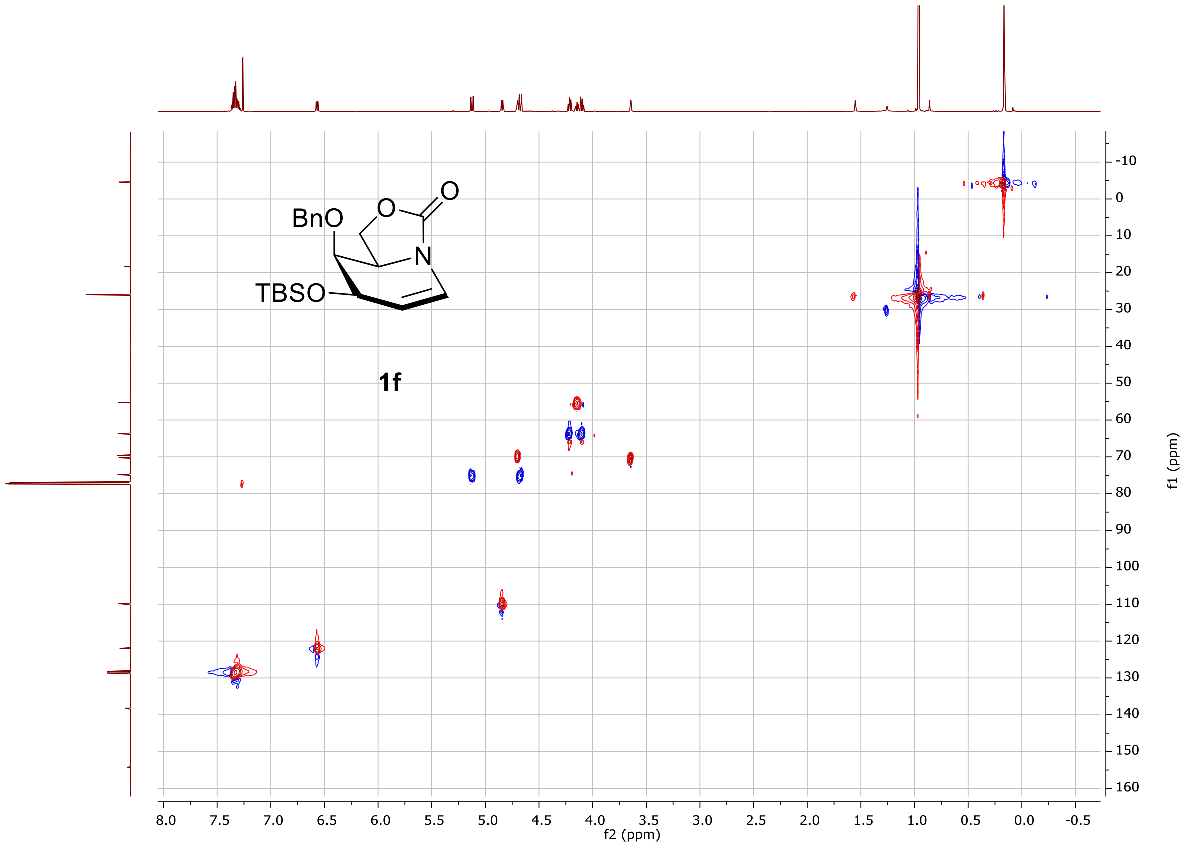

Supplementary Figure S182. HSQC spectra for 1f

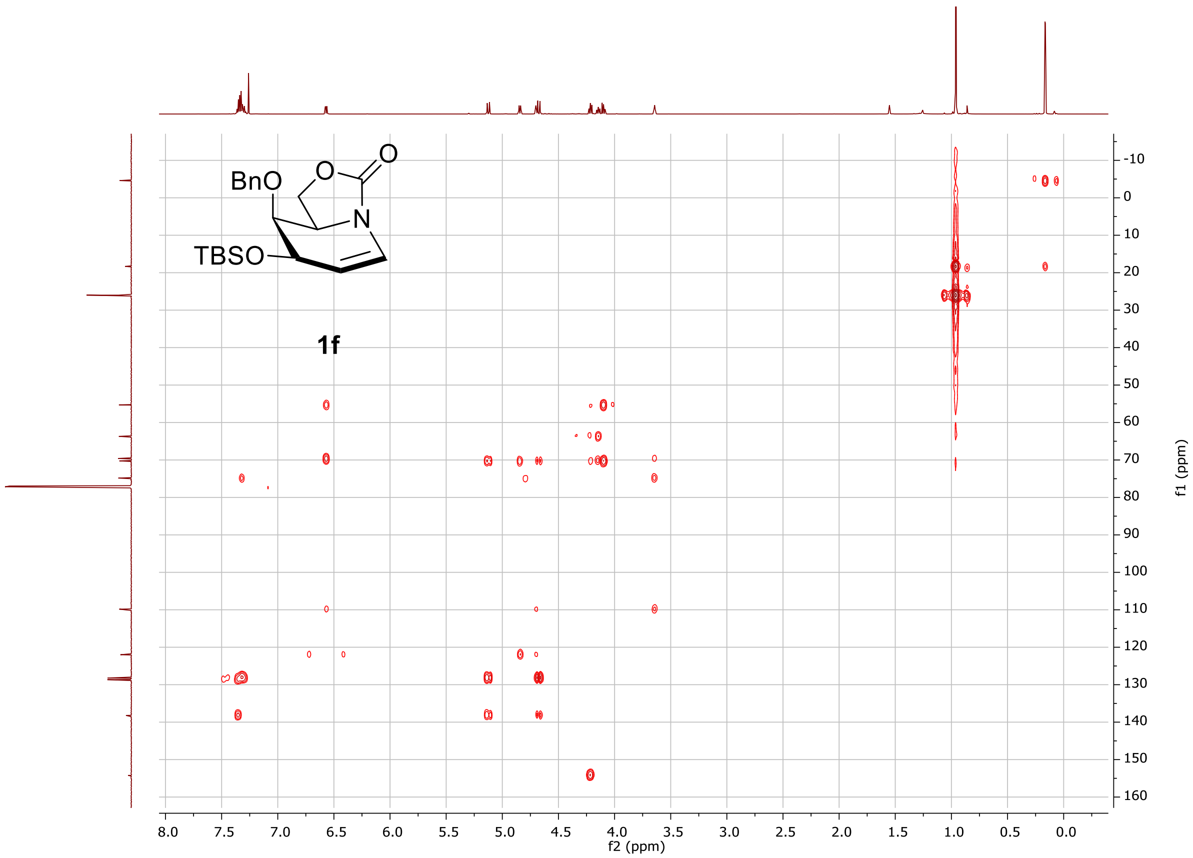

Supplementary Figure S183. HMBC spectra for 1f

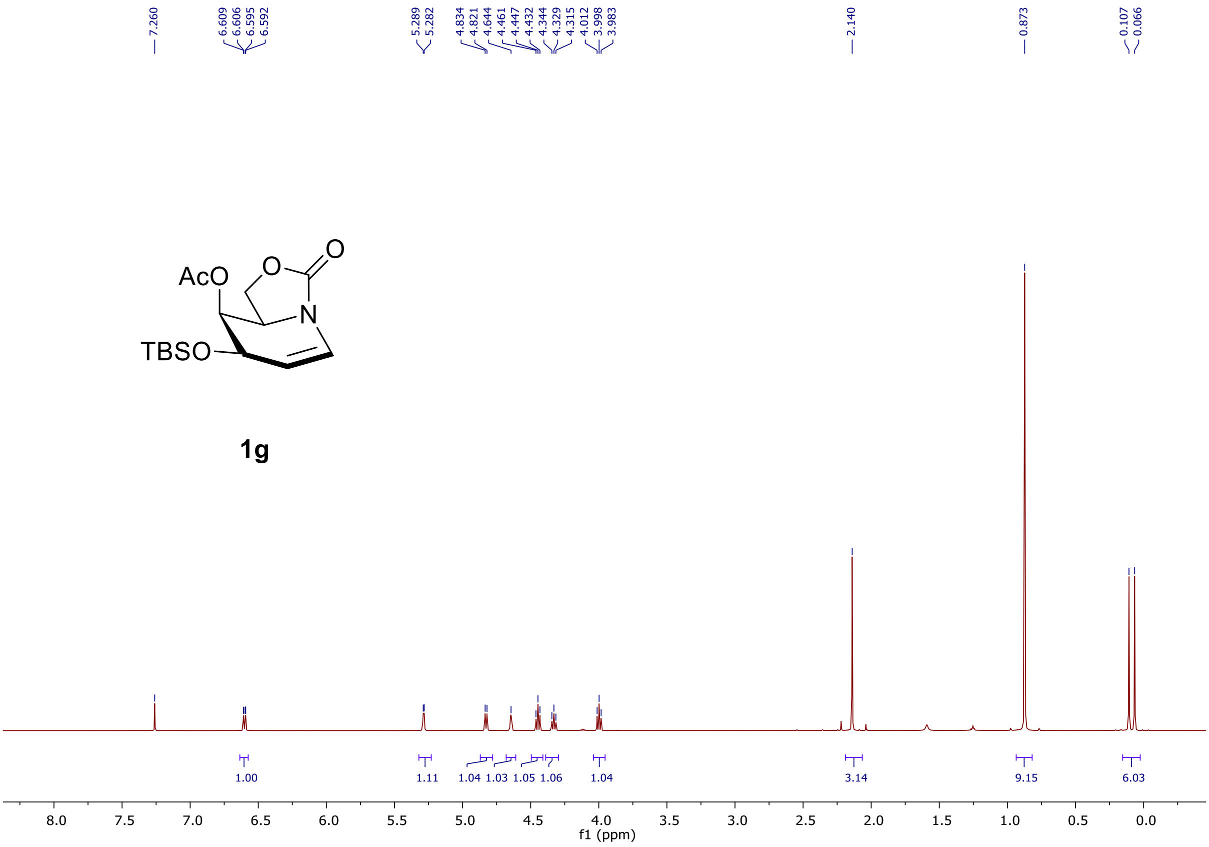

Supplementary Figure S184. ^{1}H NMR spectra for 1g

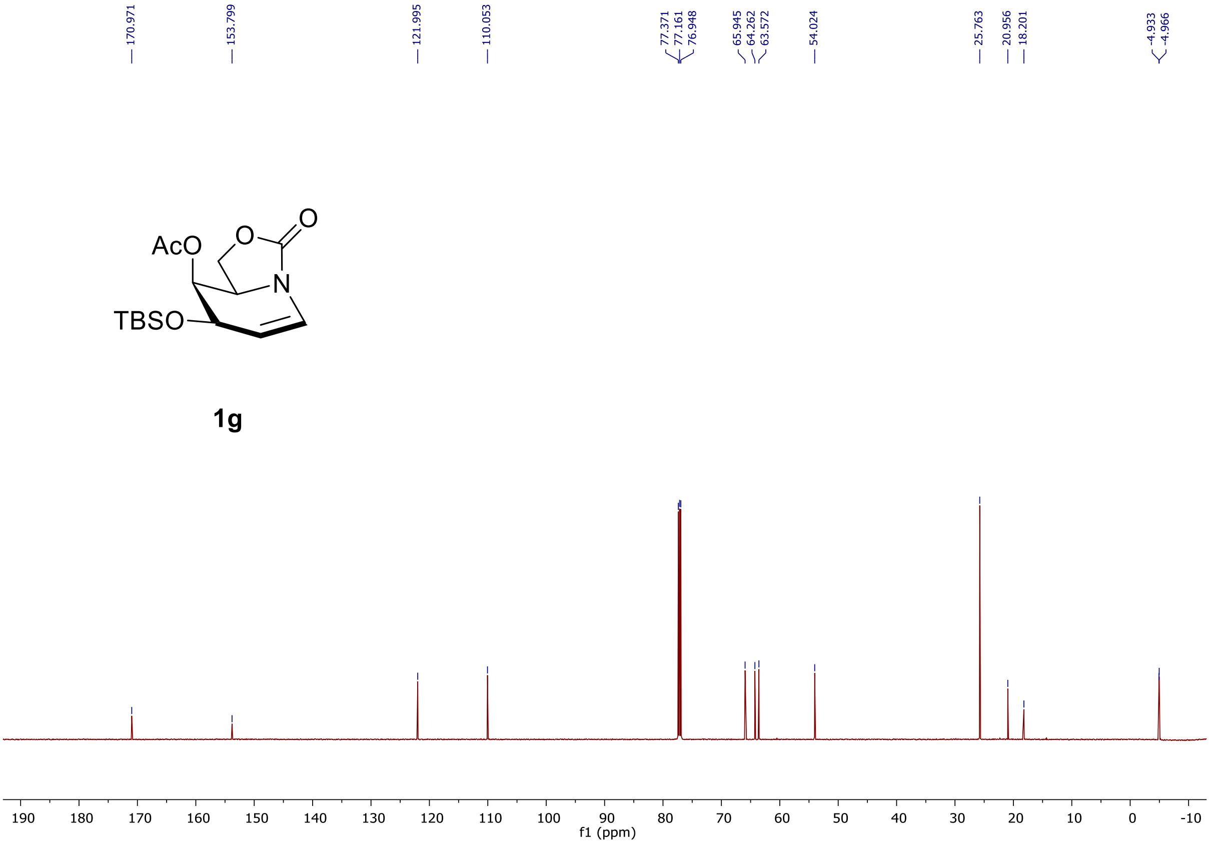

Supplementary Figure S185. ^{13}C NMR spectra for 1g

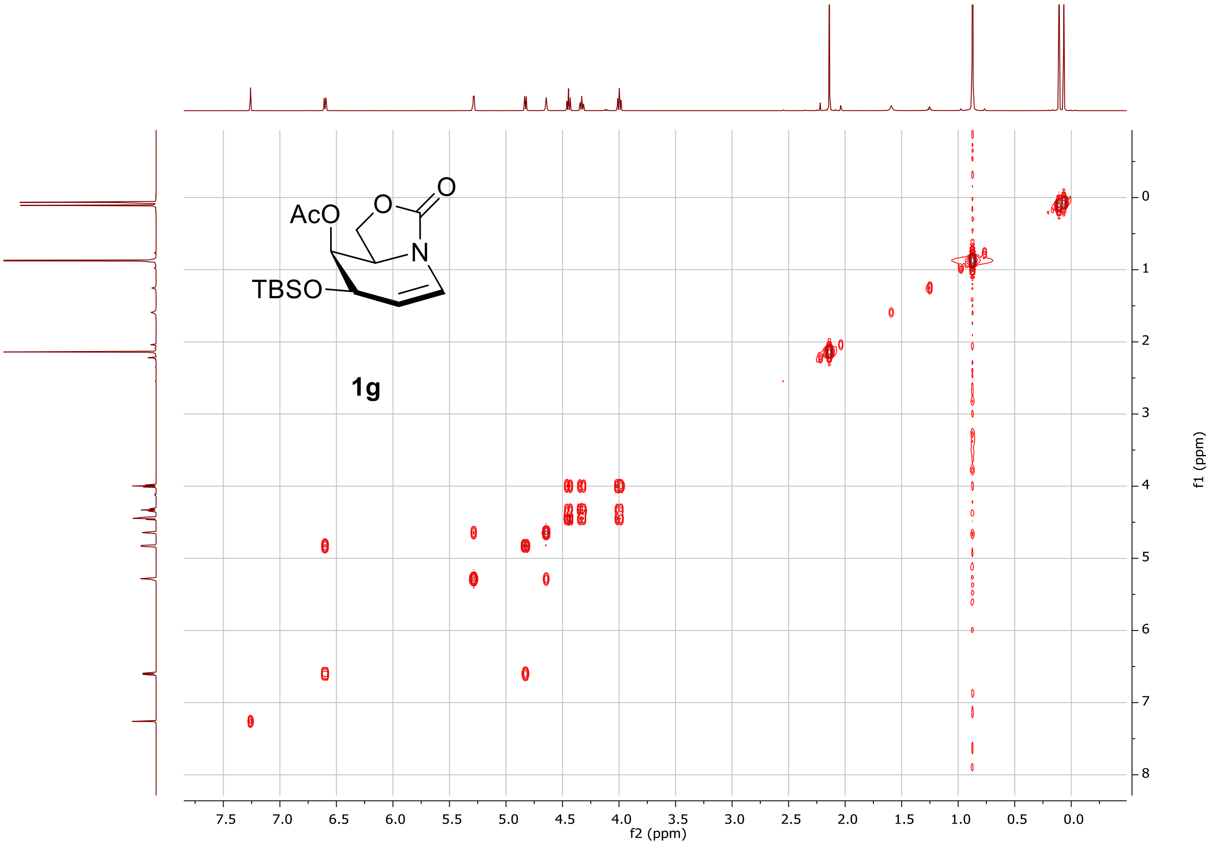

Supplementary Figure S186. COSY spectra for 1g

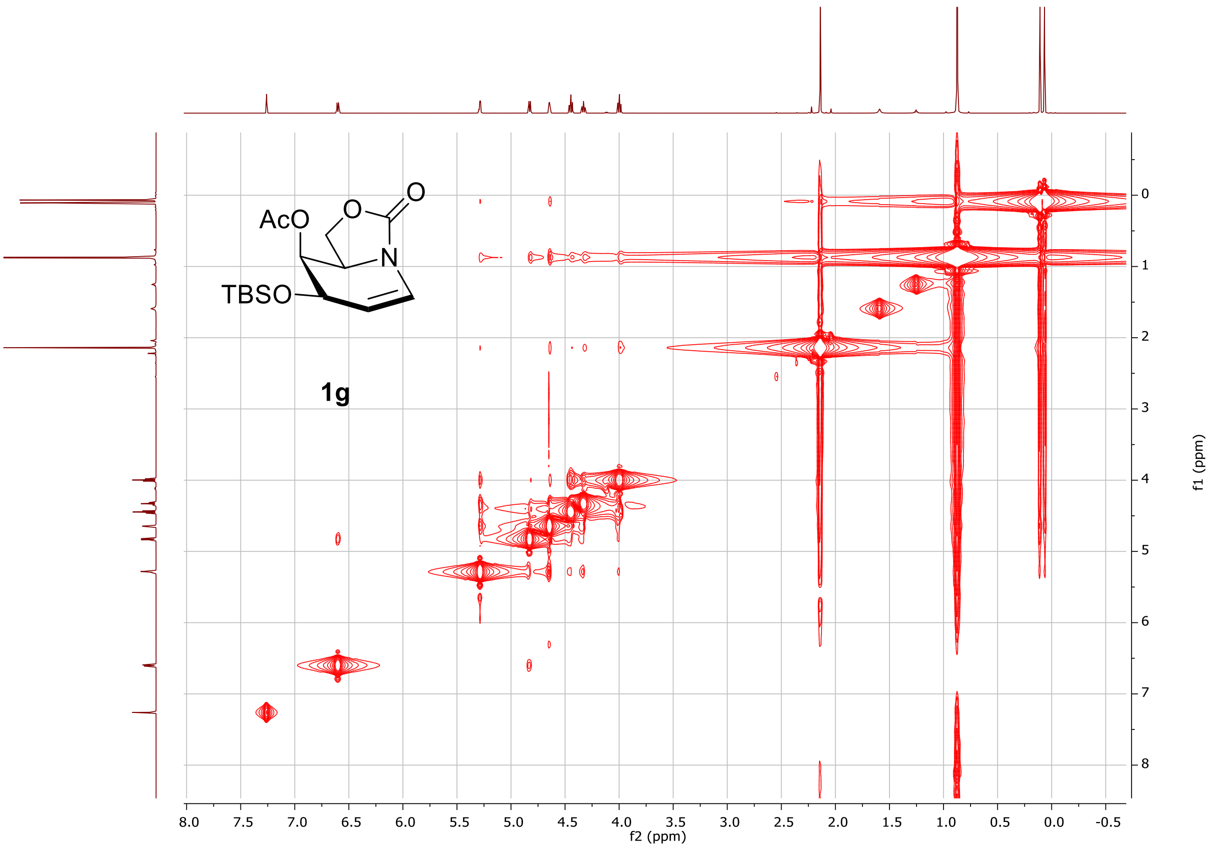

Supplementary Figure S187. NOESY spectra for 1g

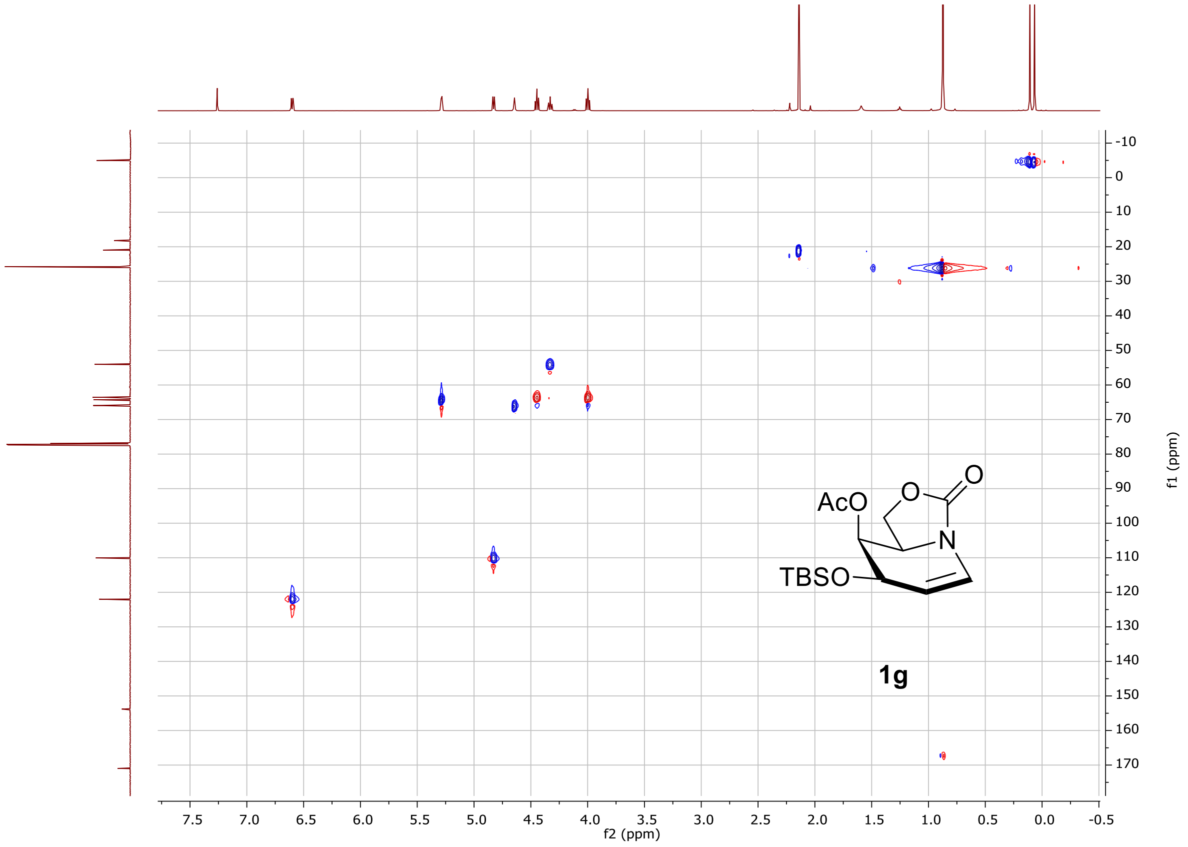

Supplementary Figure S188. HSQC spectra for 1g

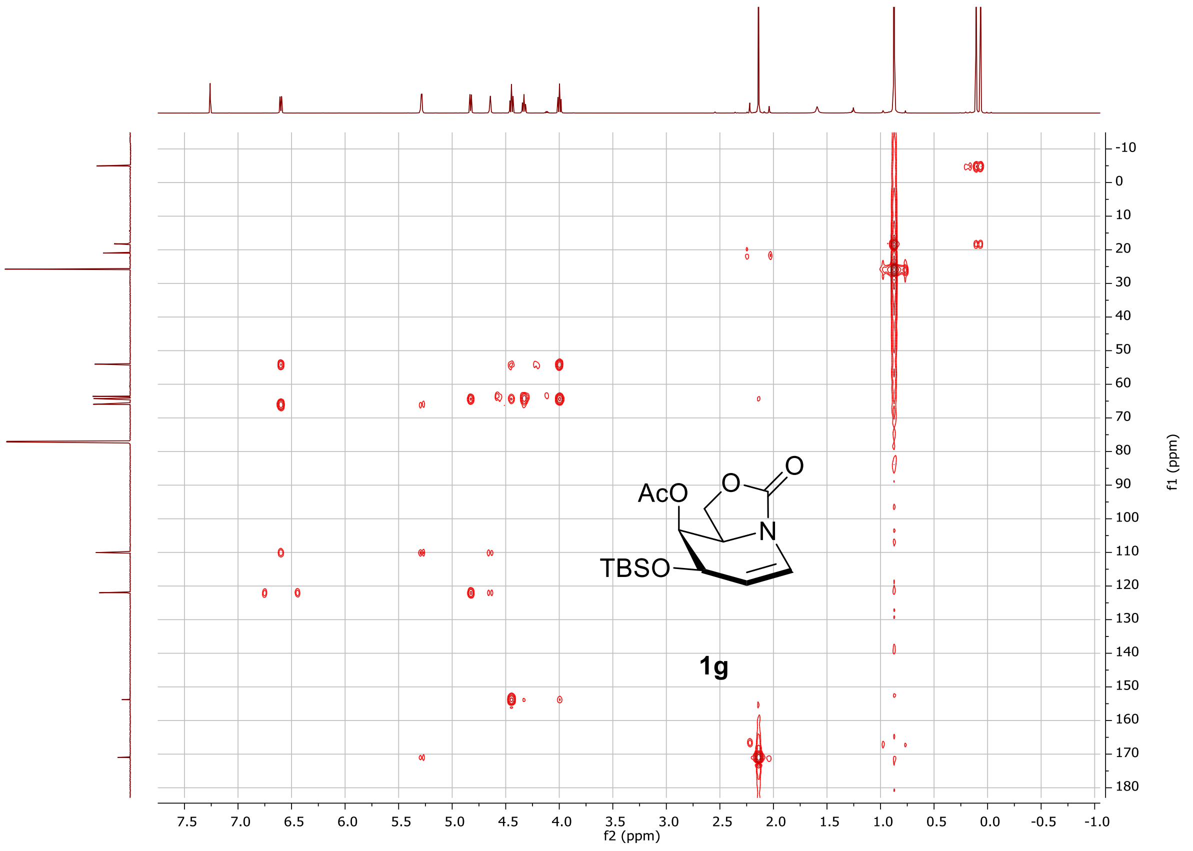

Supplementary Figure S189. HMBC spectra for 1g

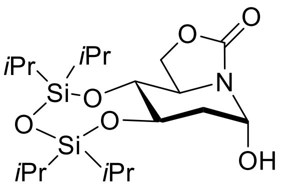

5

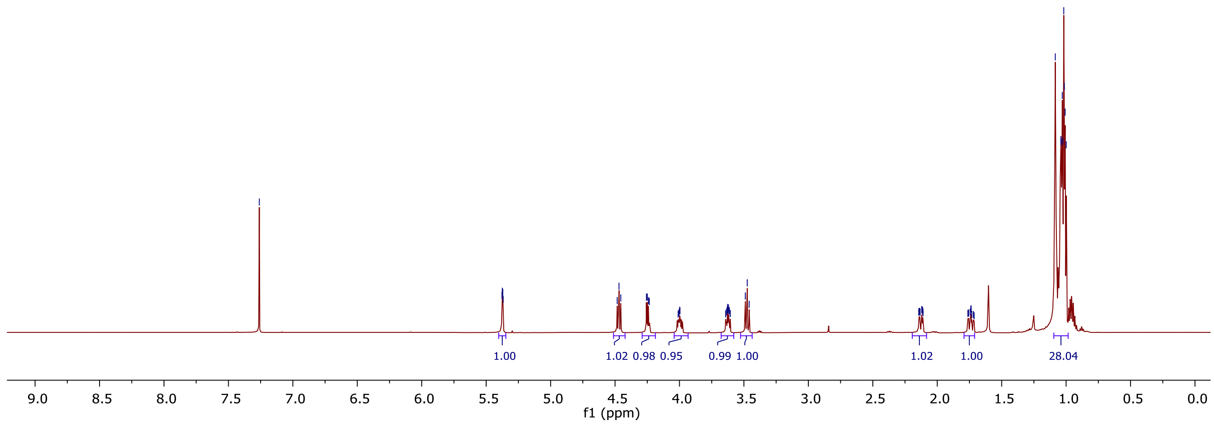

Supplementary Figure S190. ^{1}H NMR spectra for 5

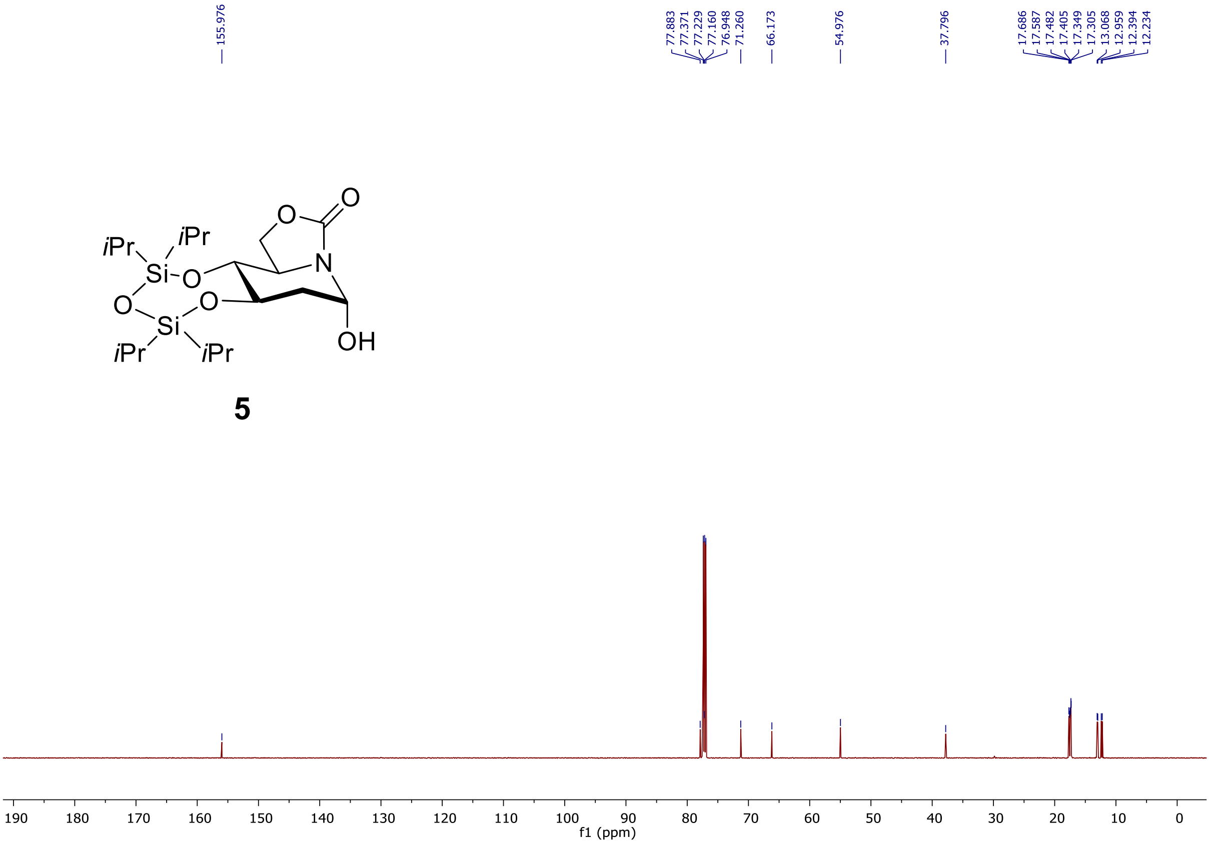

Supplementary Figure S191. ^{13}C NMR spectra for 5

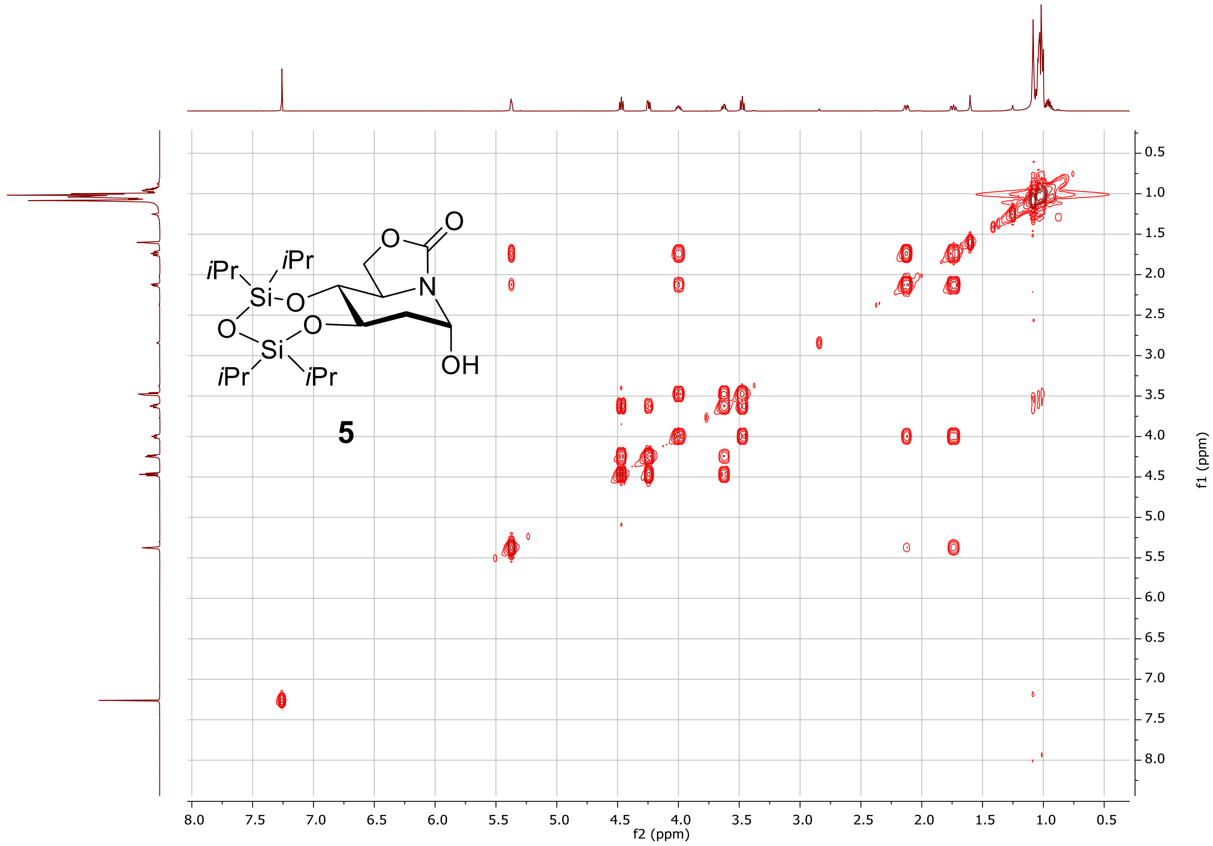

Supplementary Figure S192. COSY spectra for 5

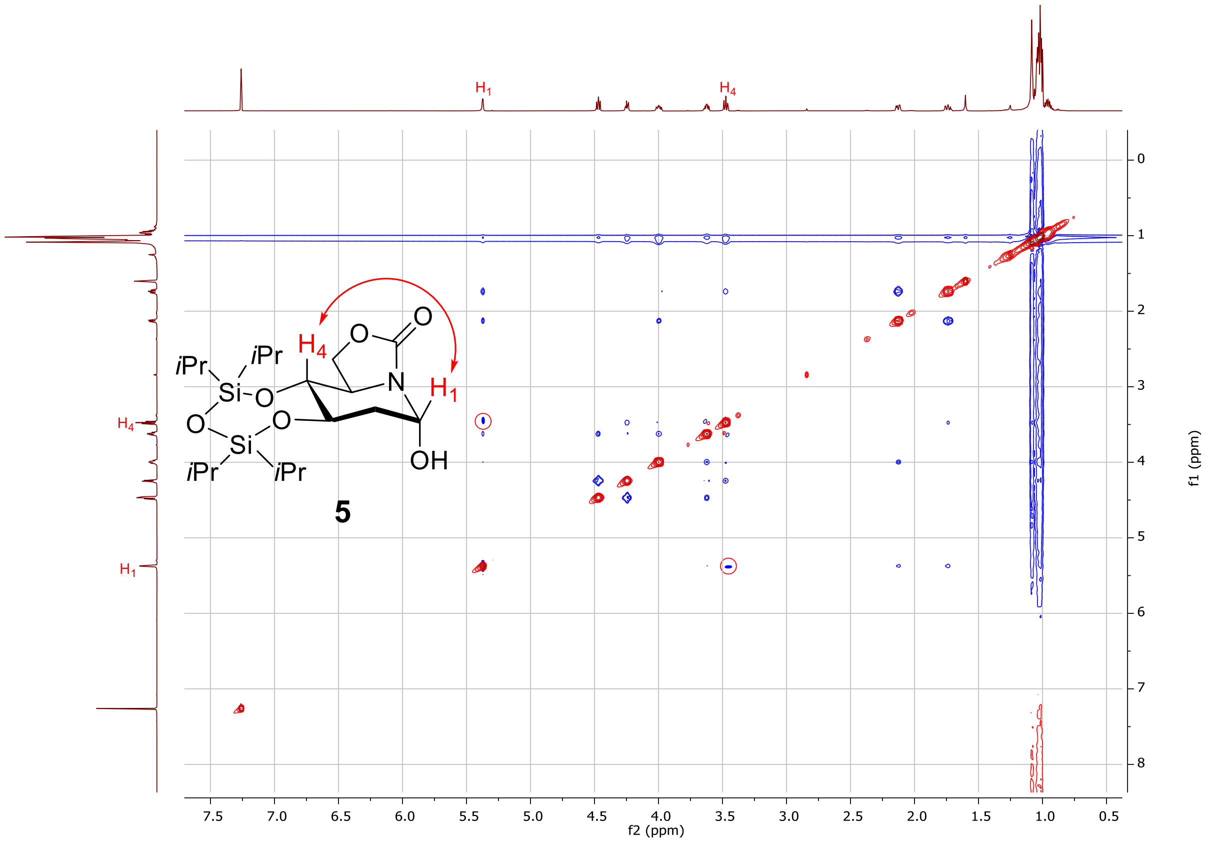

Supplementary Figure S193. NOESY spectra for 5

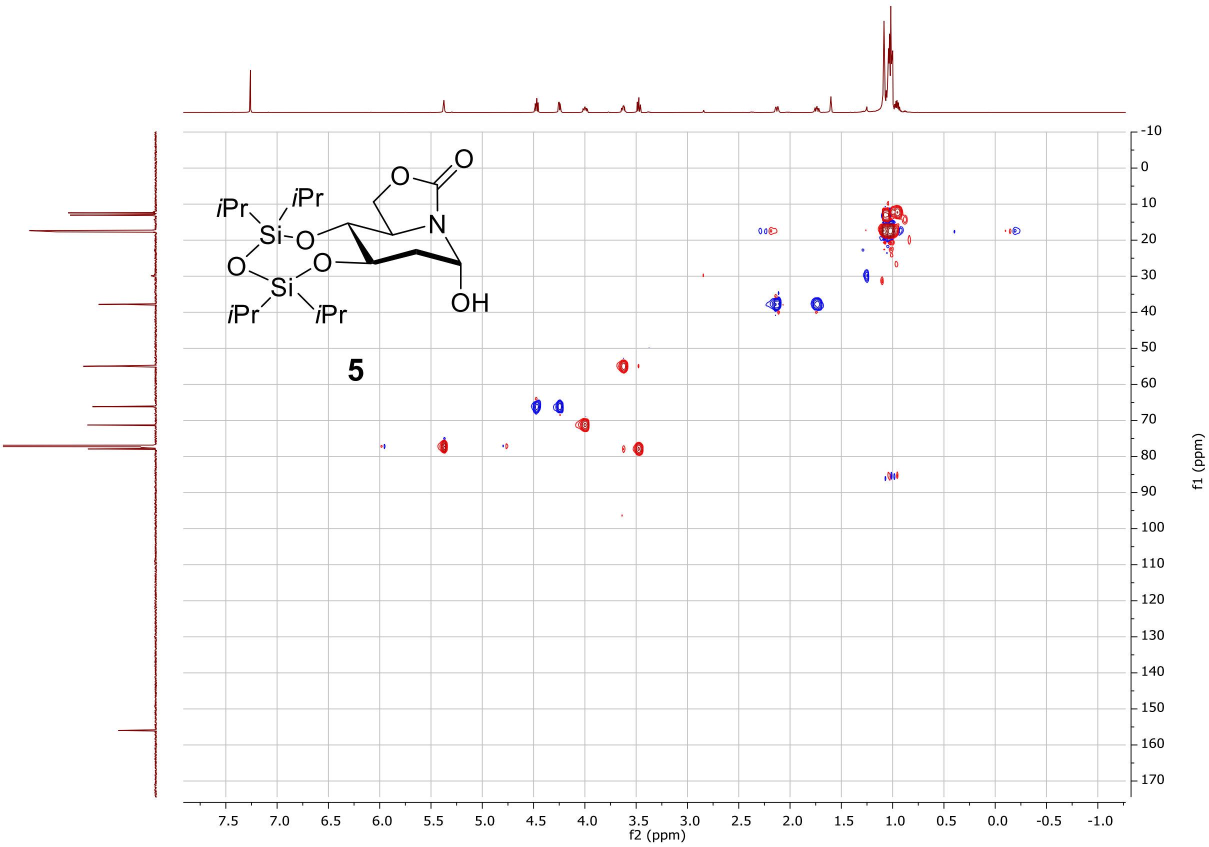

Supplementary Figure S194. HSQC spectra for 5

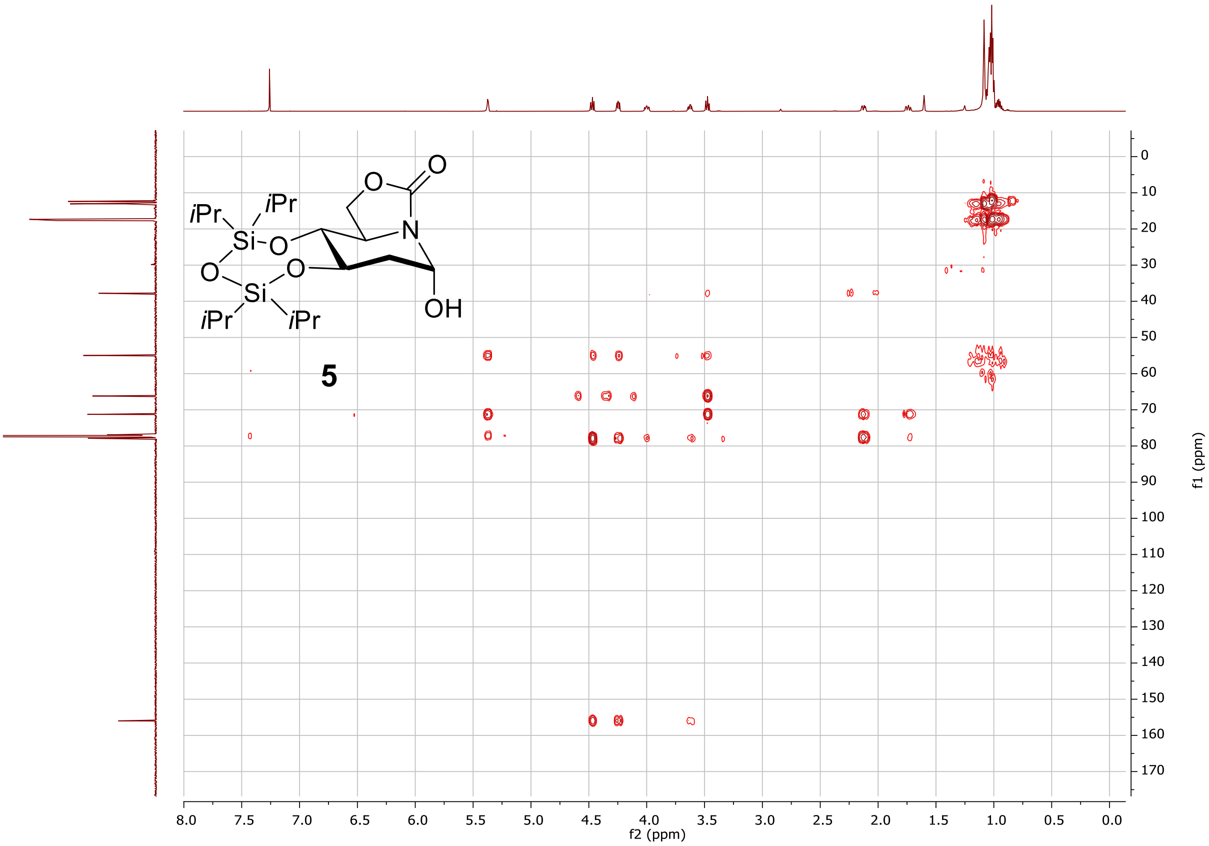

Supplementary Figure S195. HMBC spectra for 5

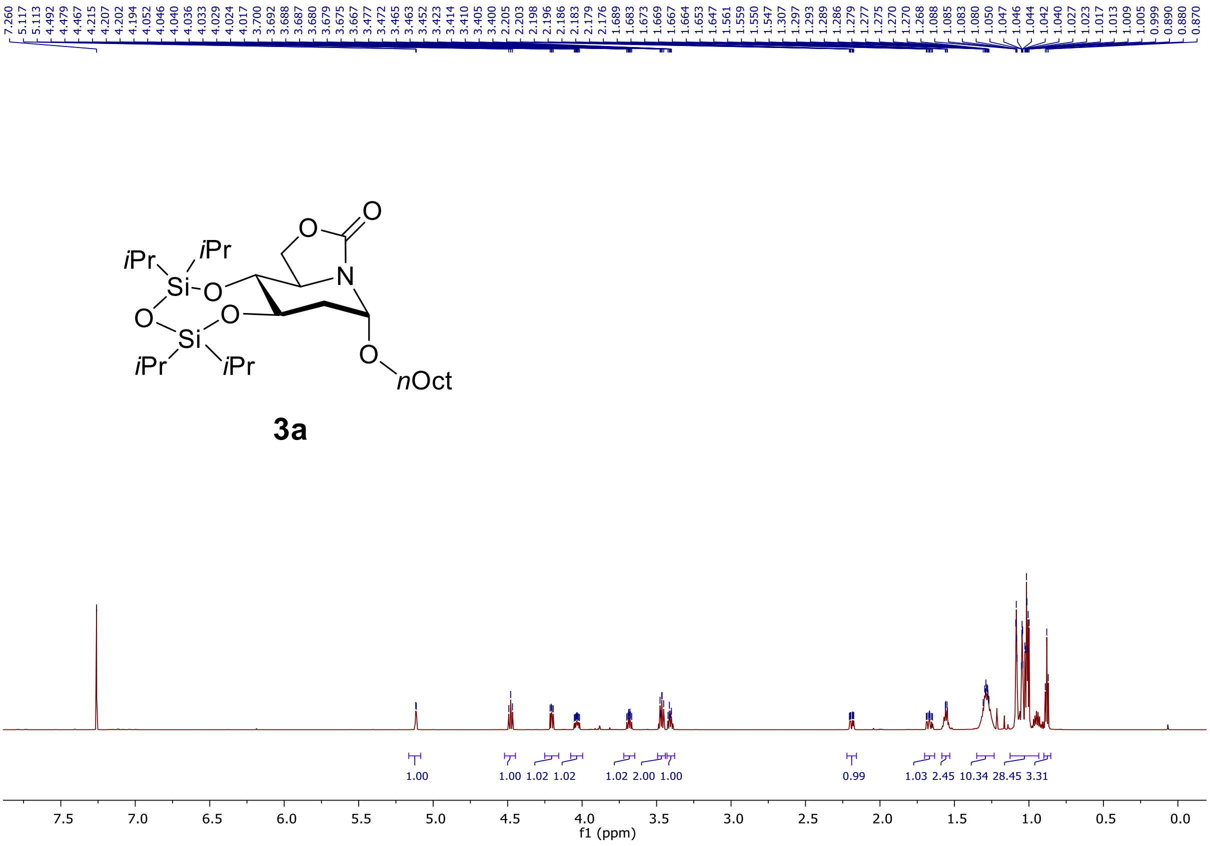

Supplementary Figure S196. ^{1}H NMR spectra for 3a

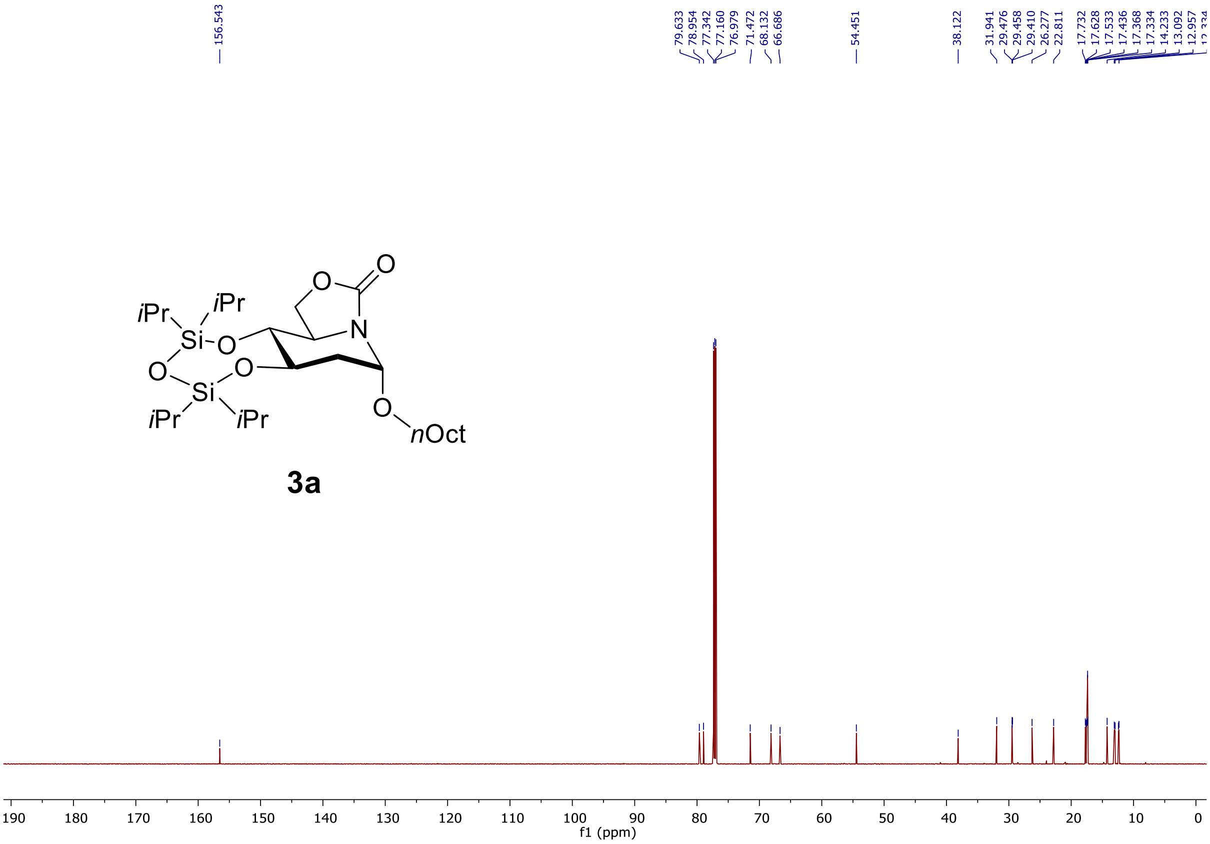

Supplementary Figure S197. ^{13}C NMR spectra for 3a

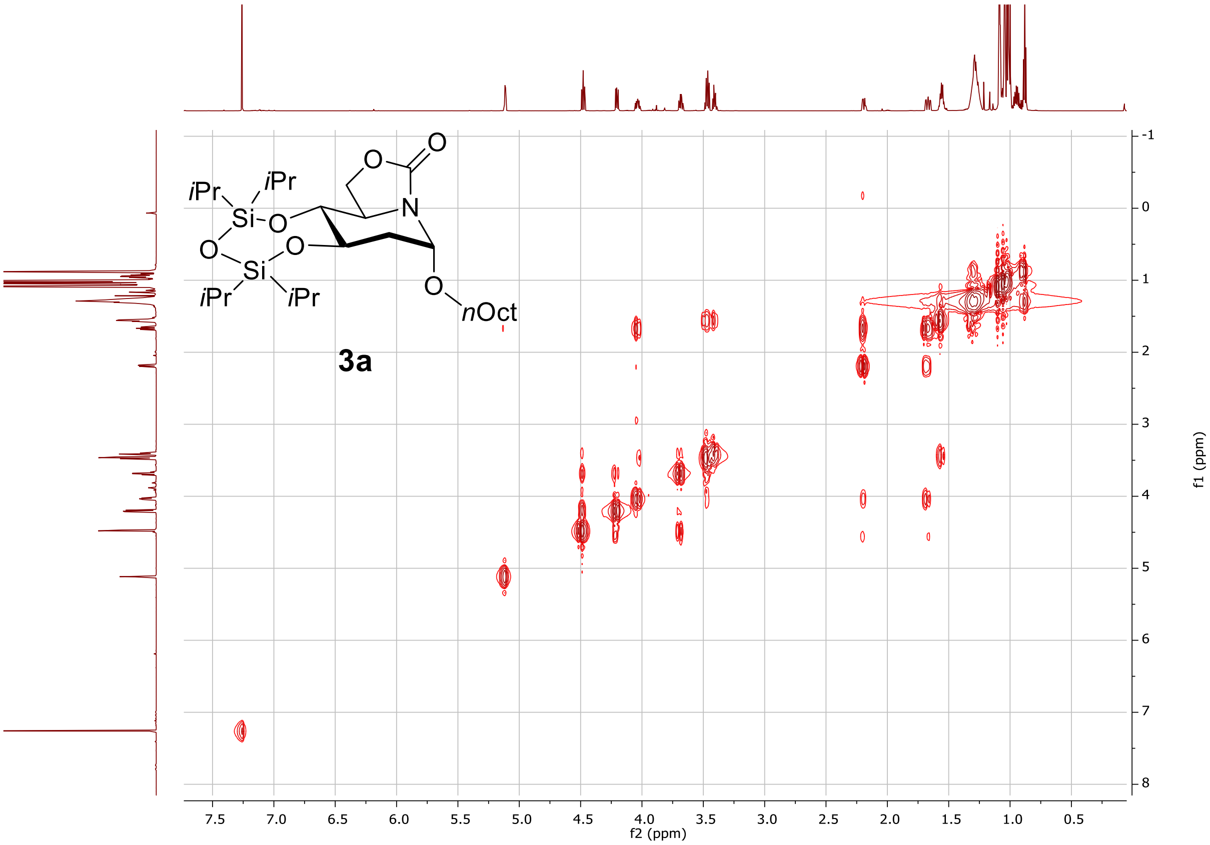

Supplementary Figure S198. COSY spectra for 3a

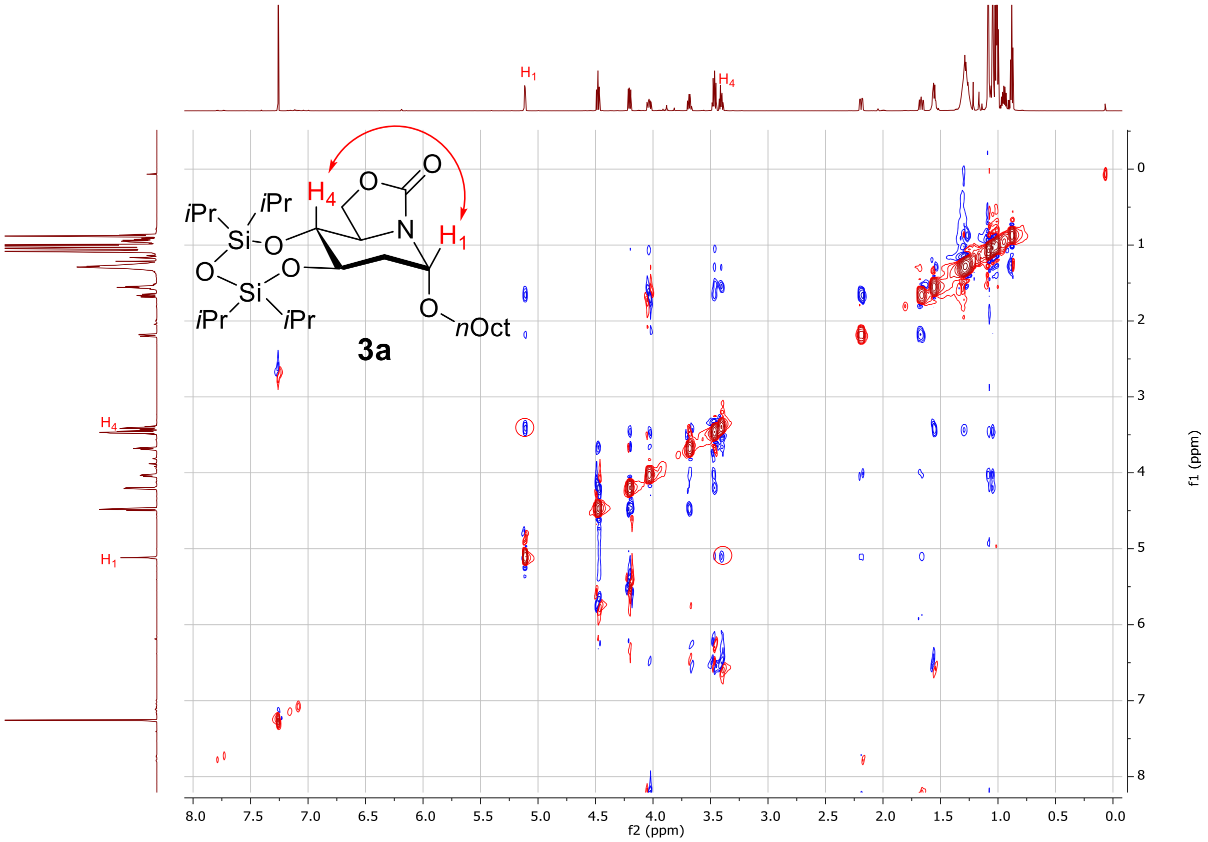

Supplementary Figure S199. NOESY spectra for 3a

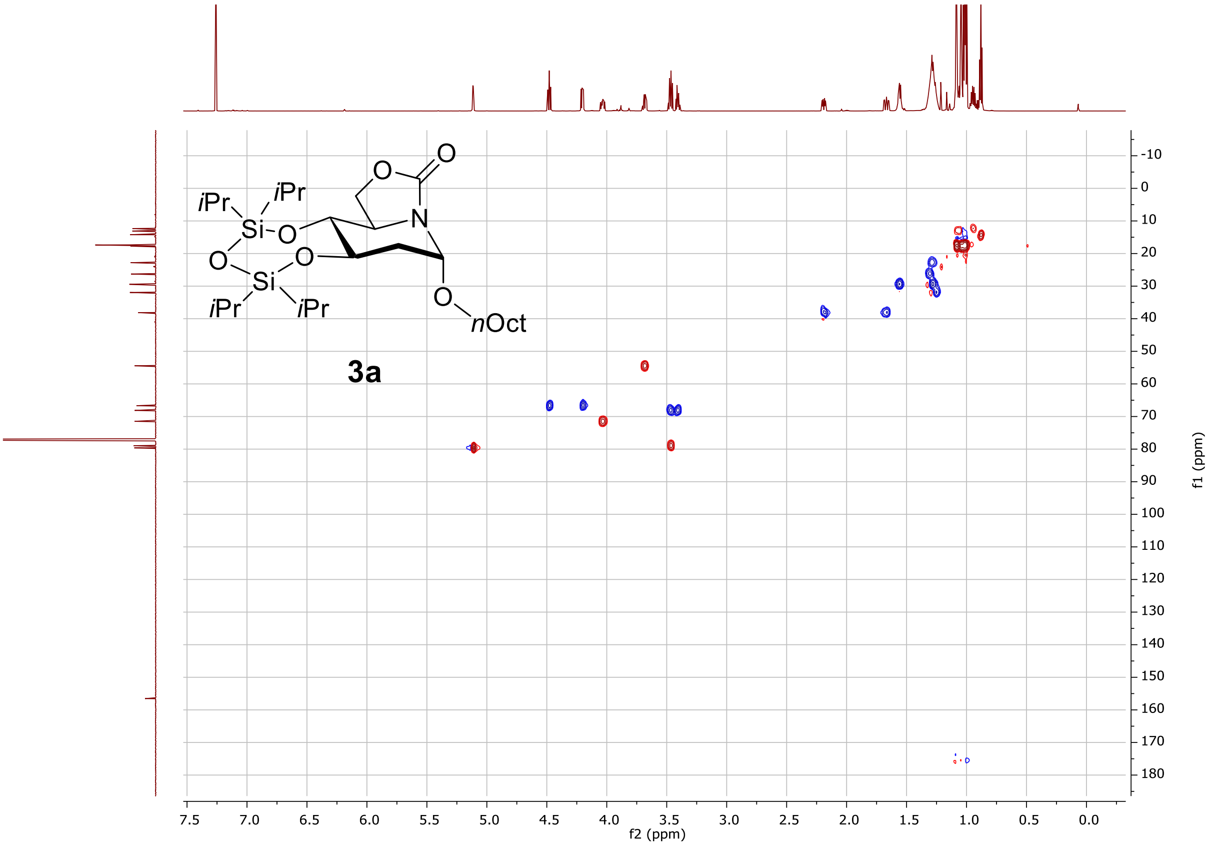

Supplementary Figure S200. HSQC spectra for 3a

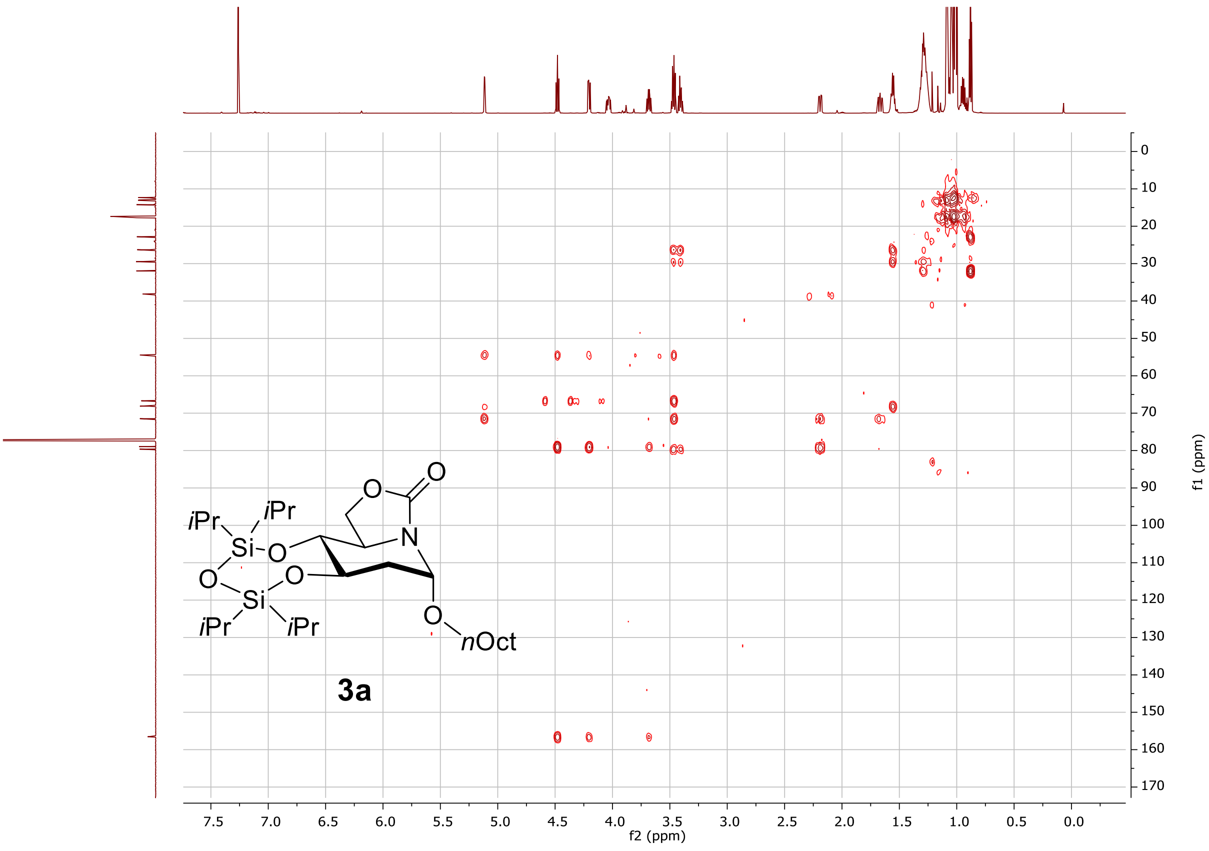

Supplementary Figure S201. HMBC spectra for 3a

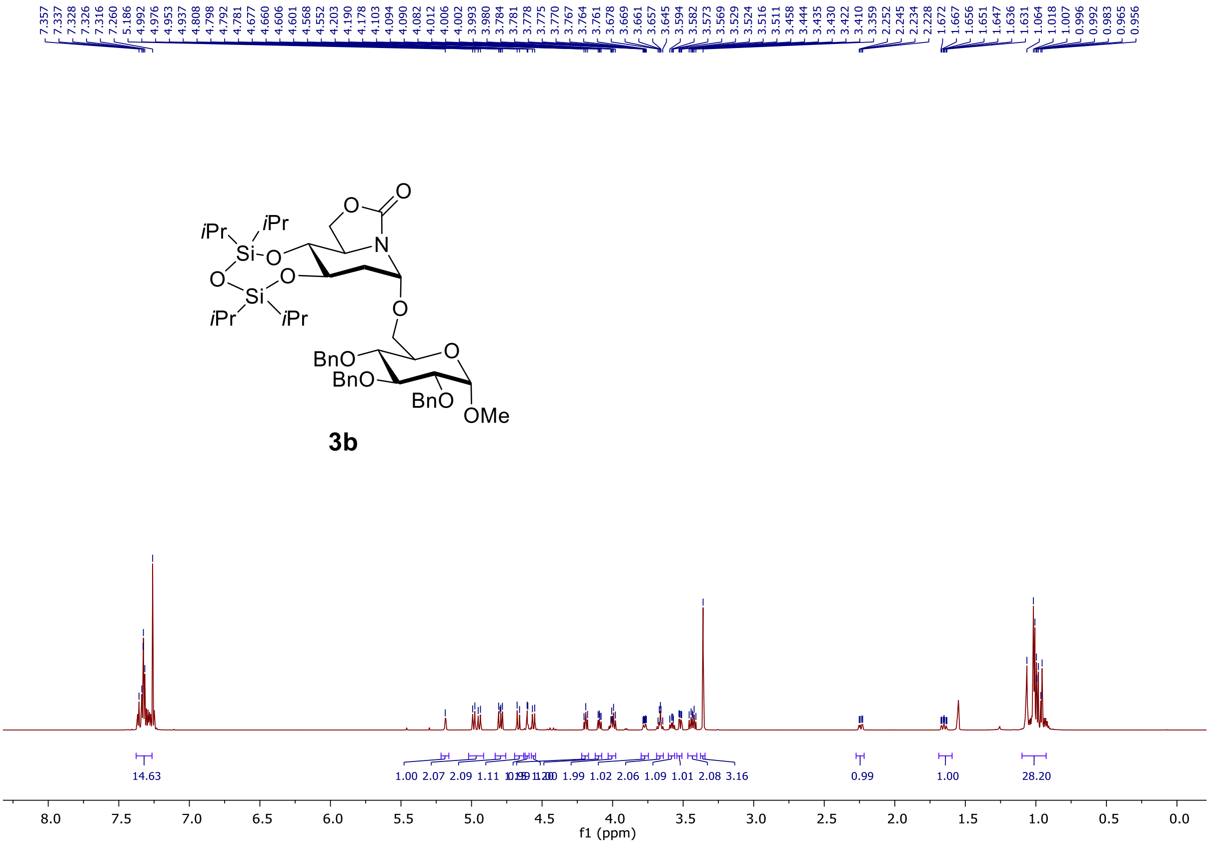

Supplementary Figure S202. ^{1}H NMR spectra for 3b

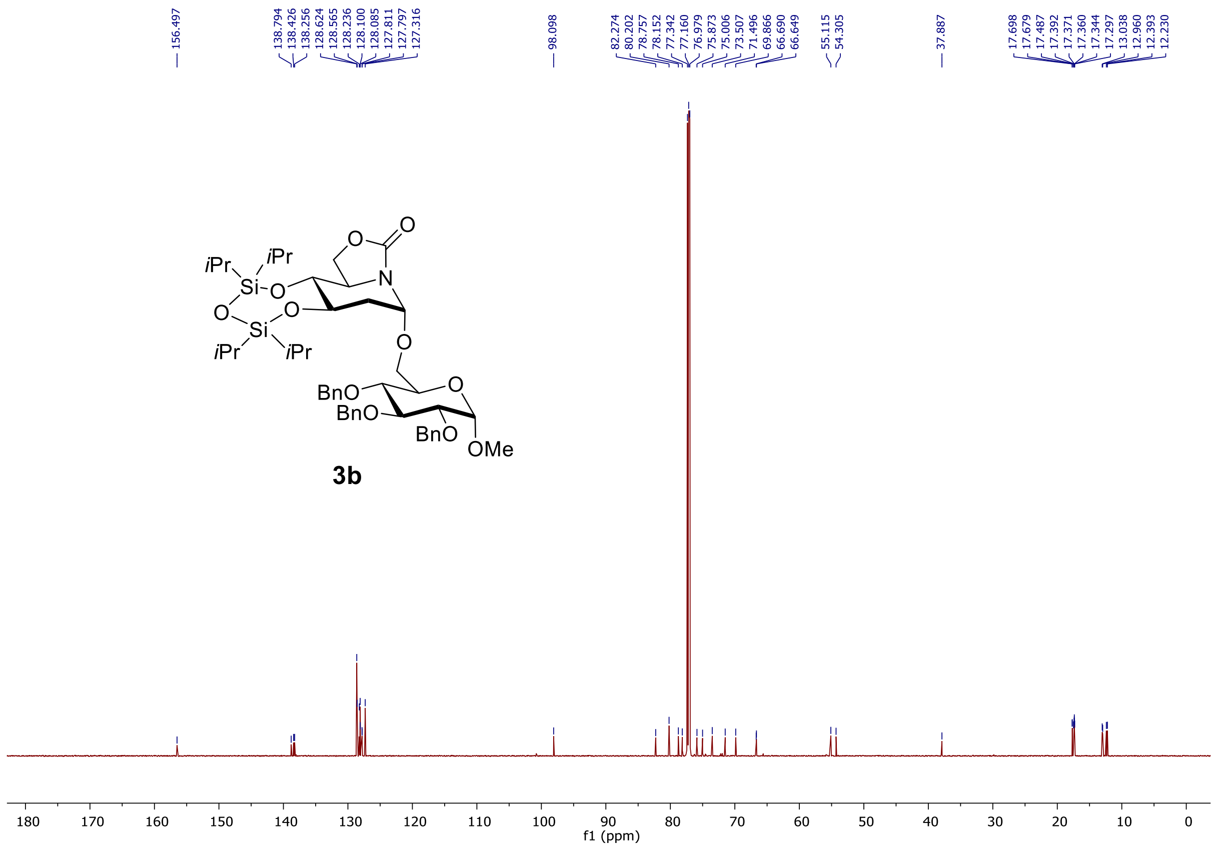

Supplementary Figure S203. ^{13}C NMR spectra for 3b

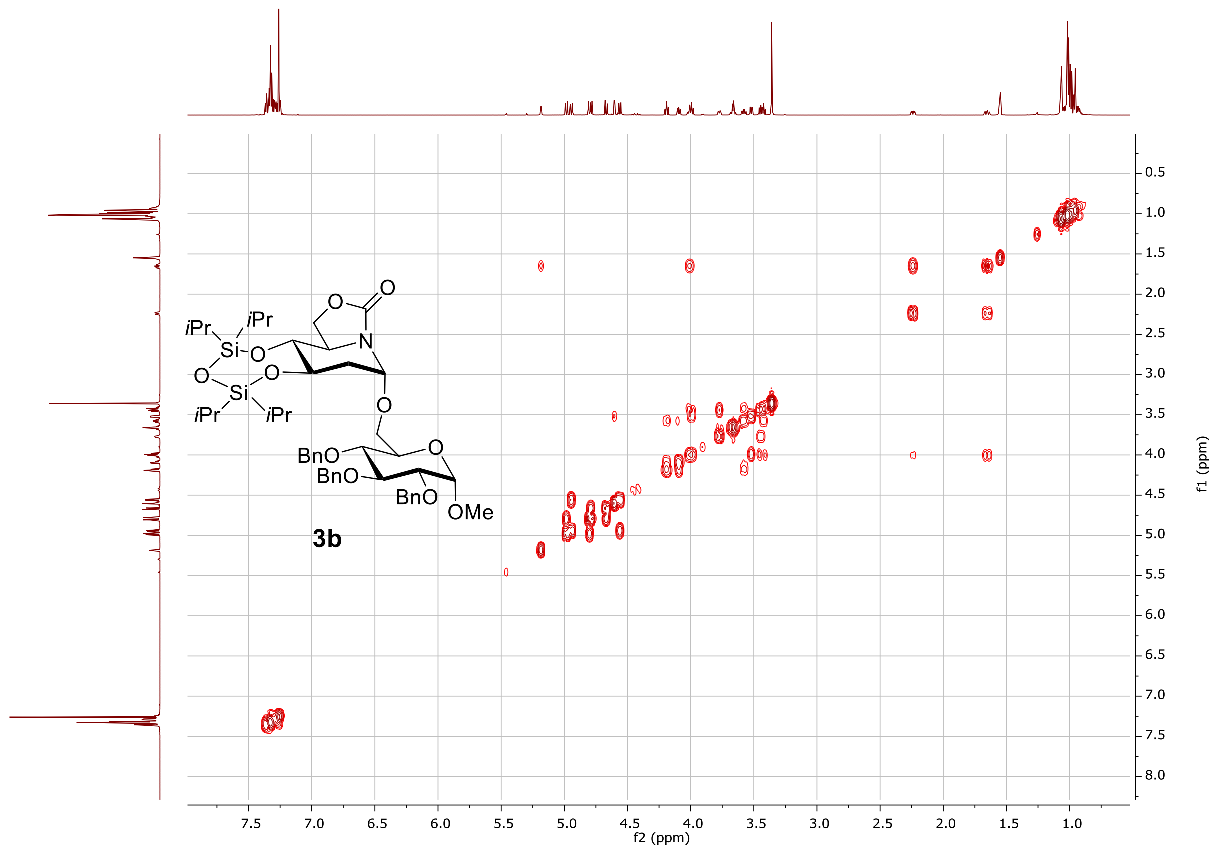

Supplementary Figure S204. COSY spectra for 3b

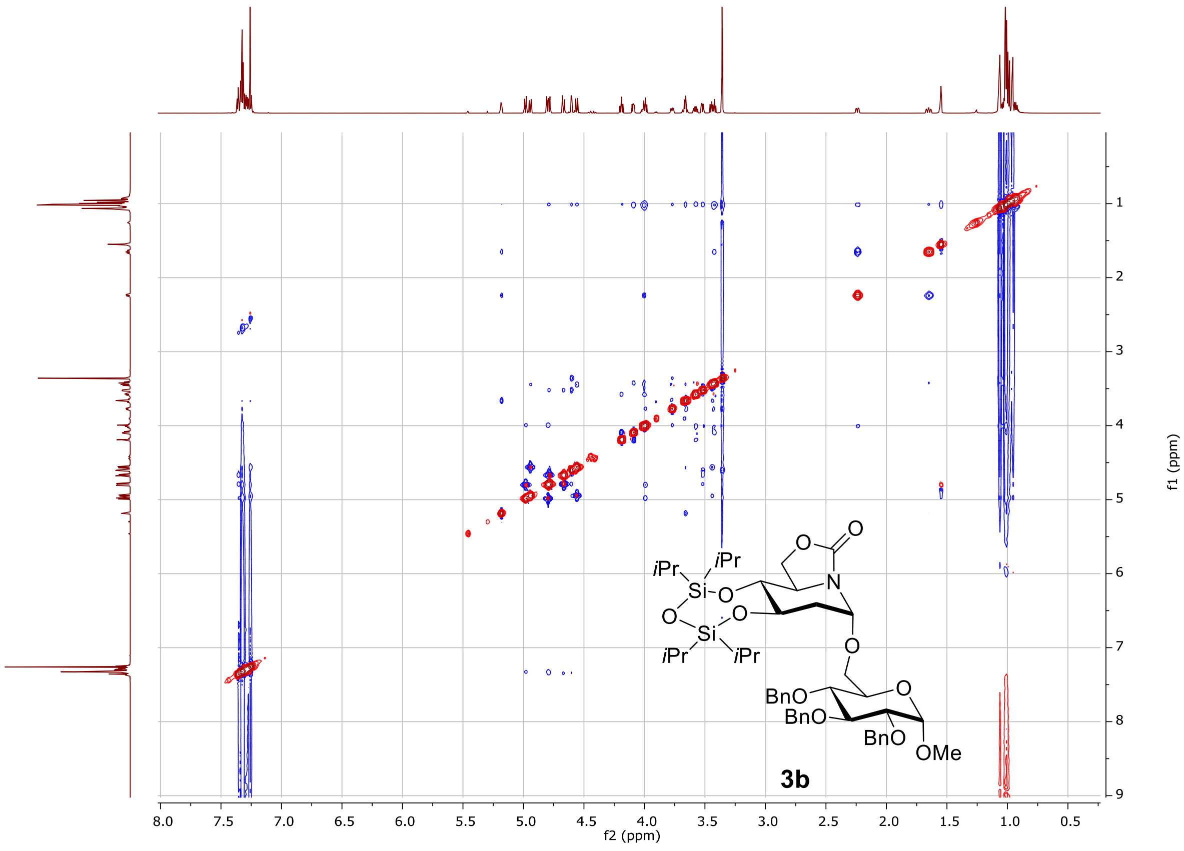

Supplementary Figure S205. NOESY spectra for 3b

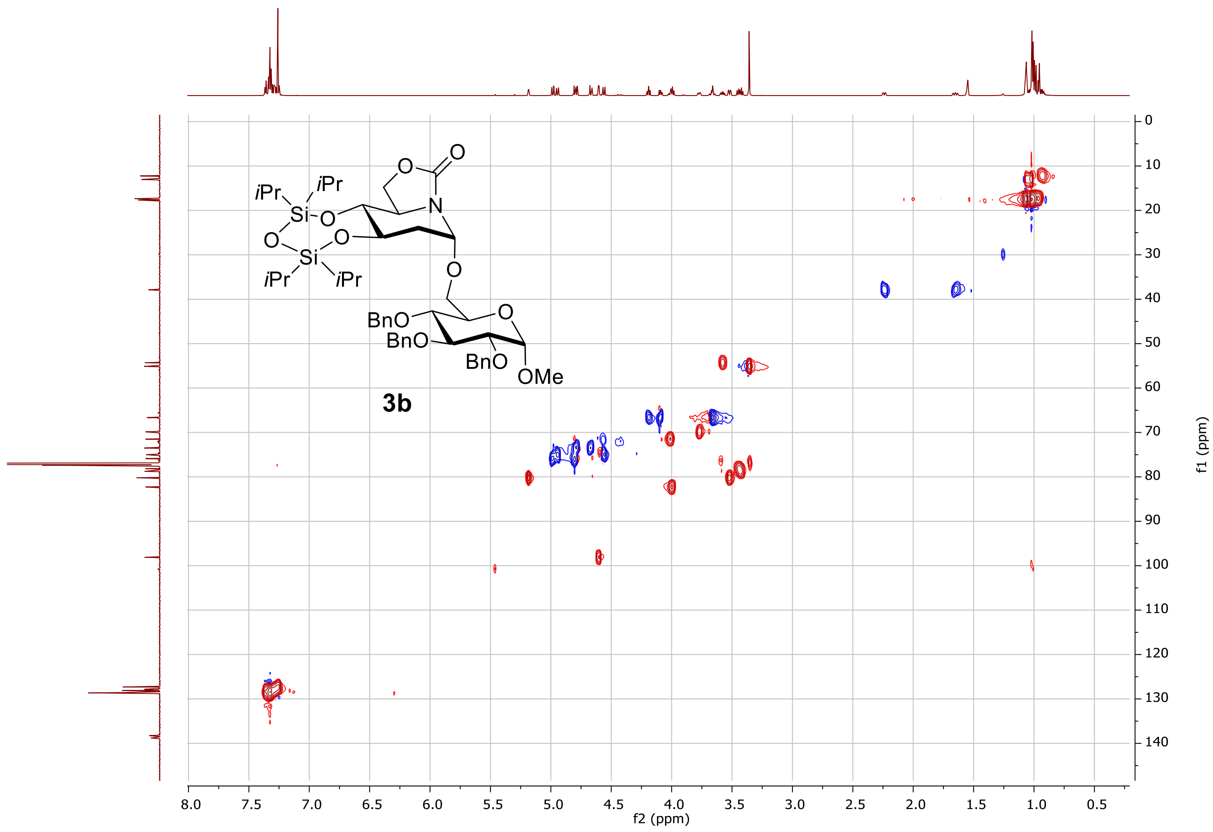

Supplementary Figure S206. HSQC spectra for 3b

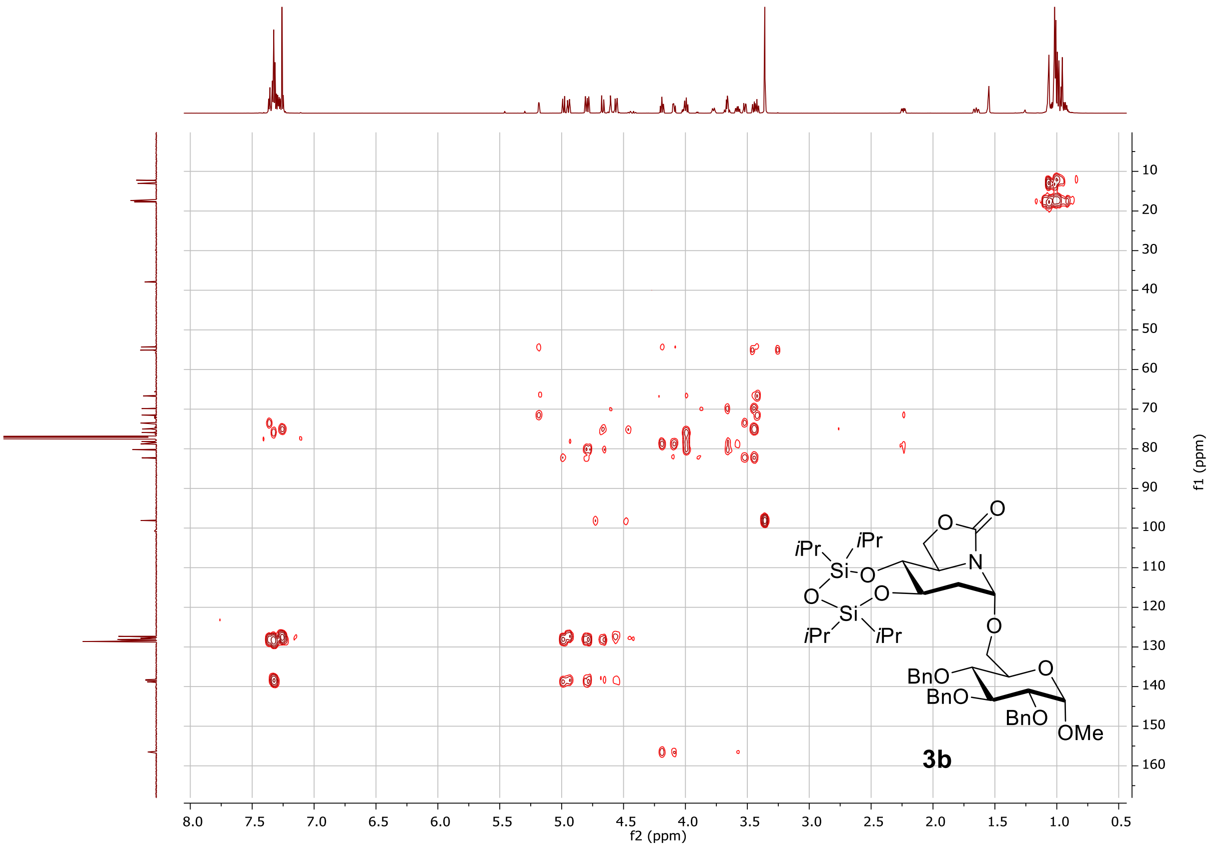

Supplementary Figure S207. HMBC spectra for 3b

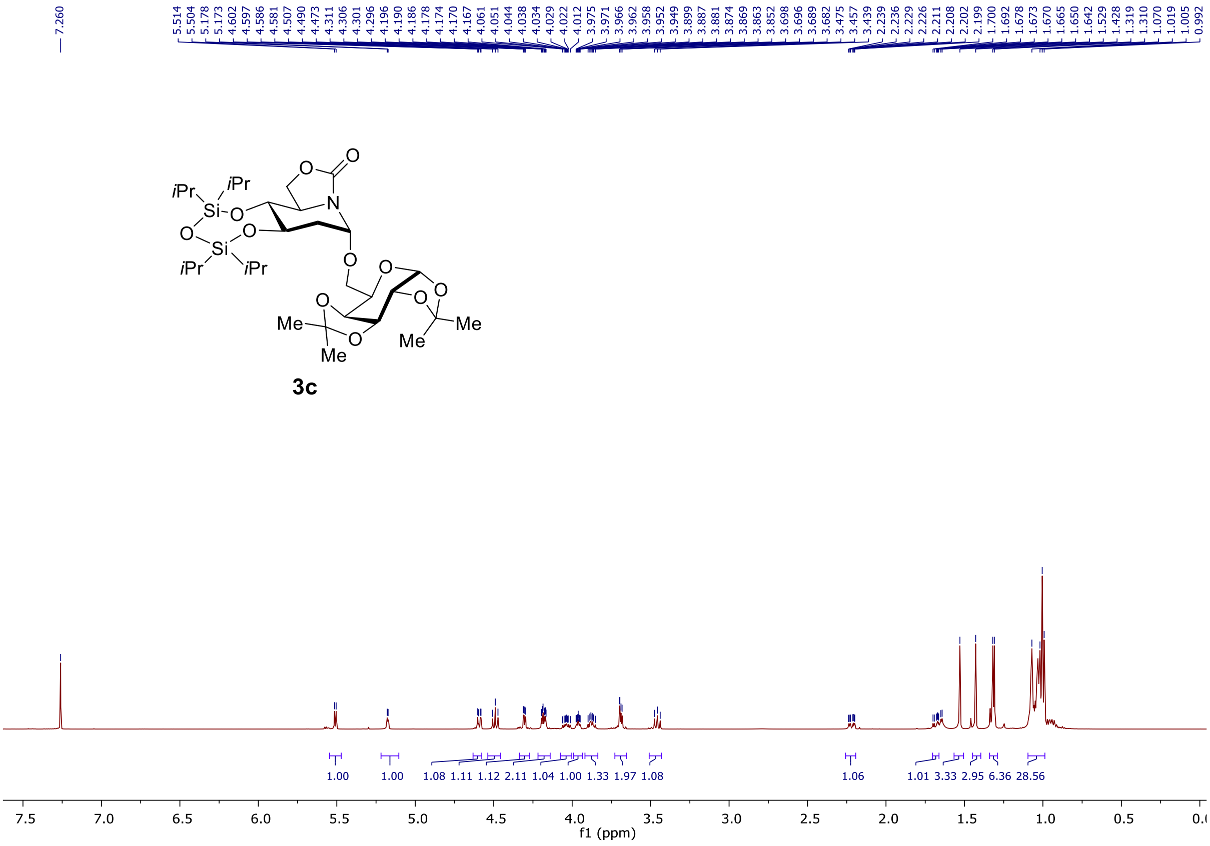

Supplementary Figure S208. ^{1}H NMR spectra for 3c

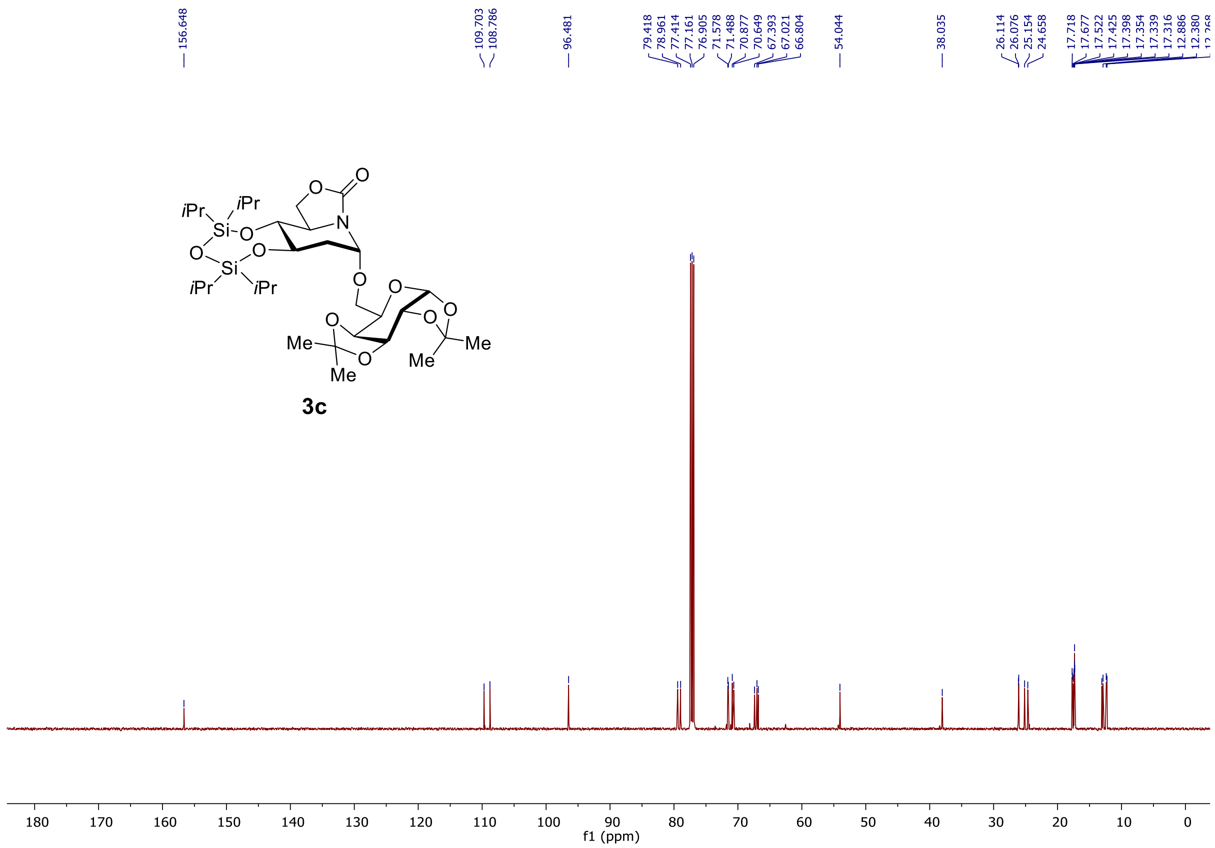

Supplementary Figure S209. ^{13}C NMR spectra for 3c

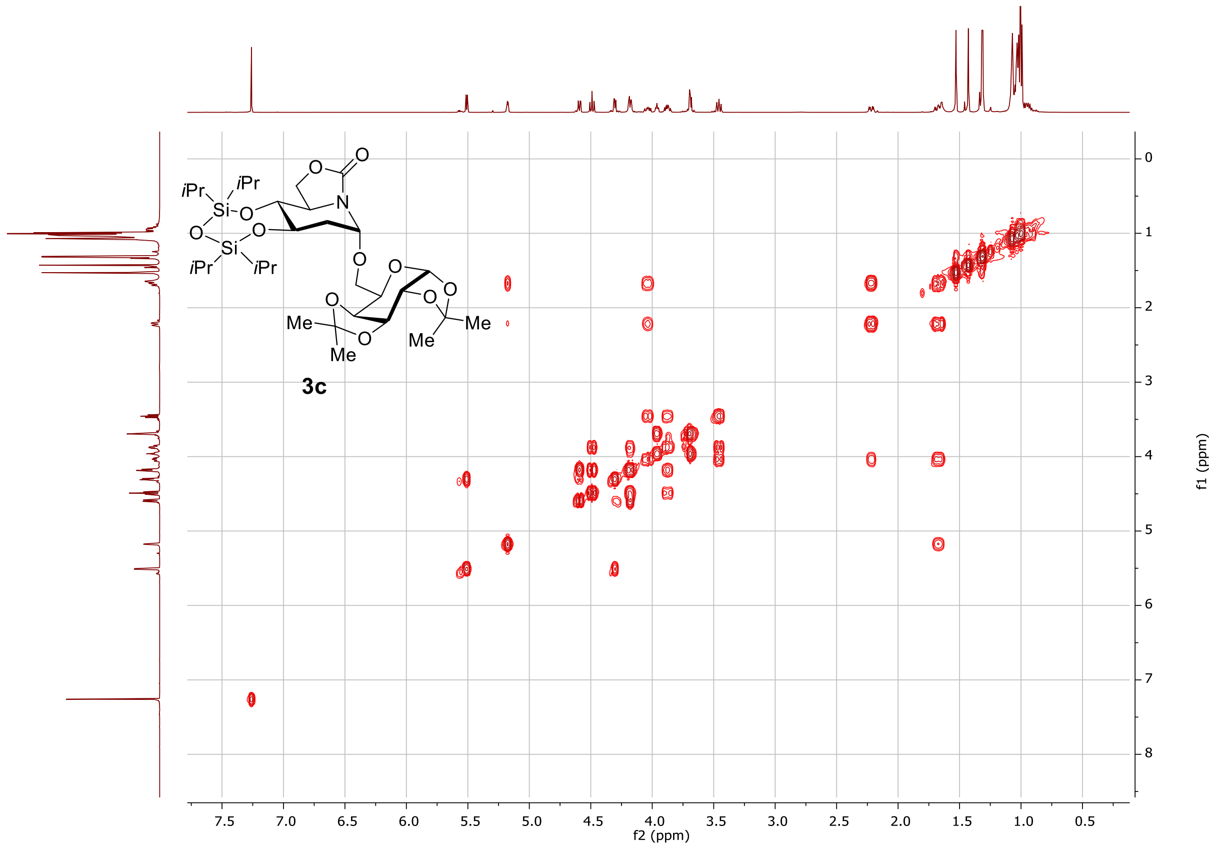

Supplementary Figure S210. COSY spectra for 3c

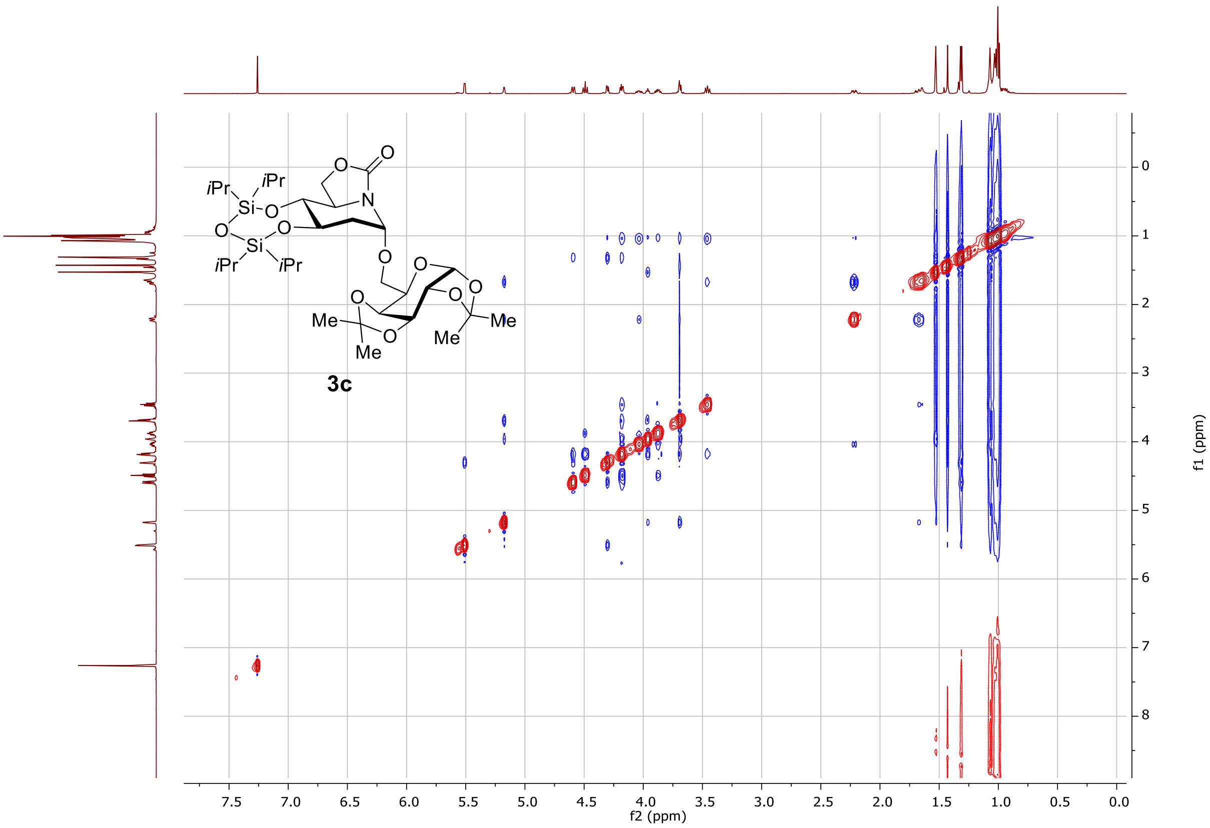

Supplementary Figure S211. NOESY spectra for 3c

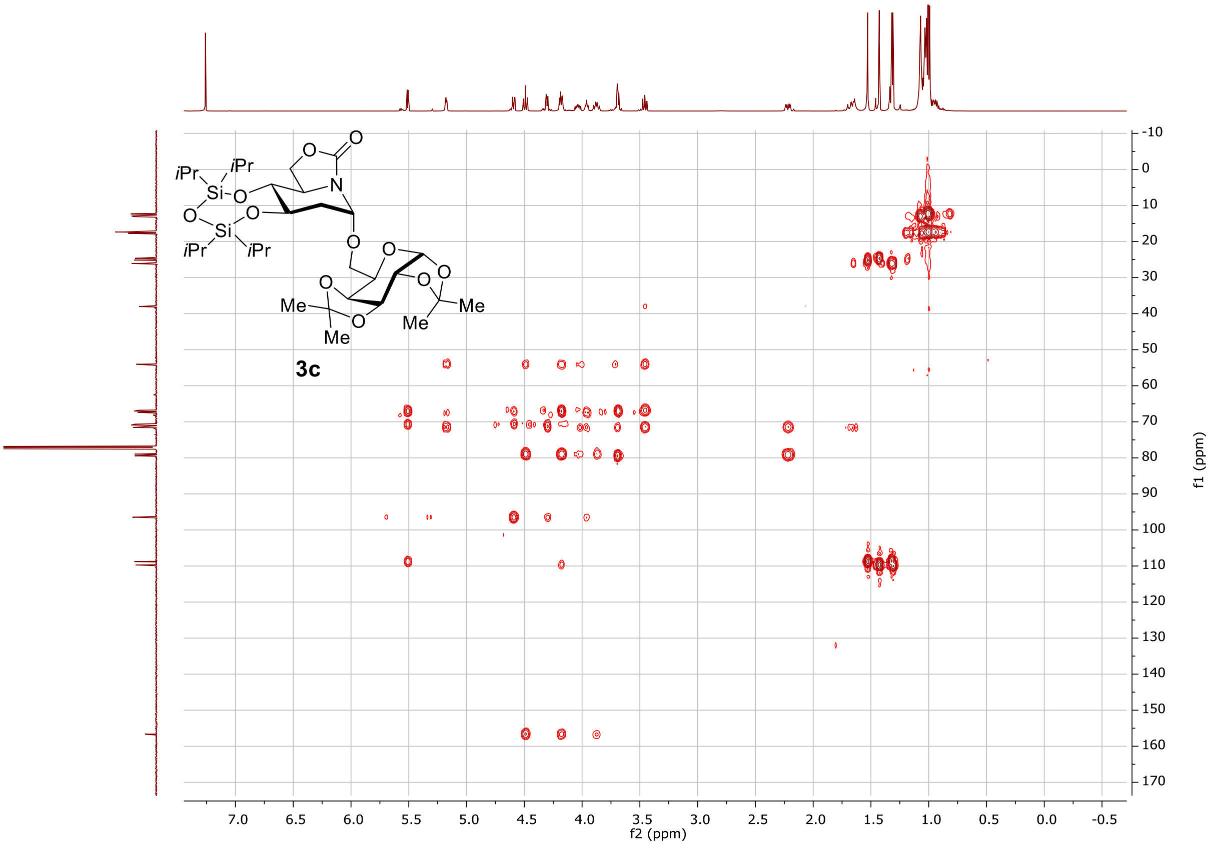

Supplementary Figure S212. HMBC spectra for 3c

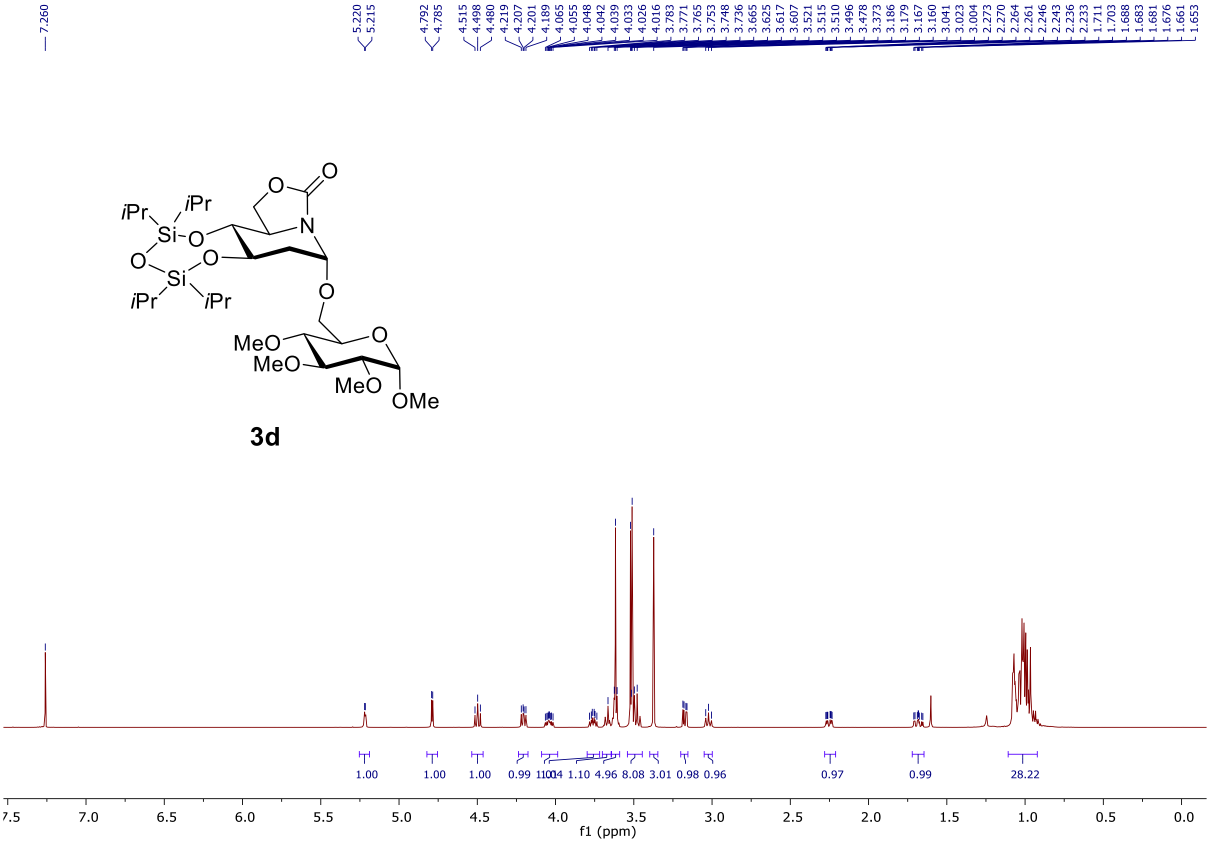

Supplementary Figure S213. ^{1}H NMR spectra for 3d

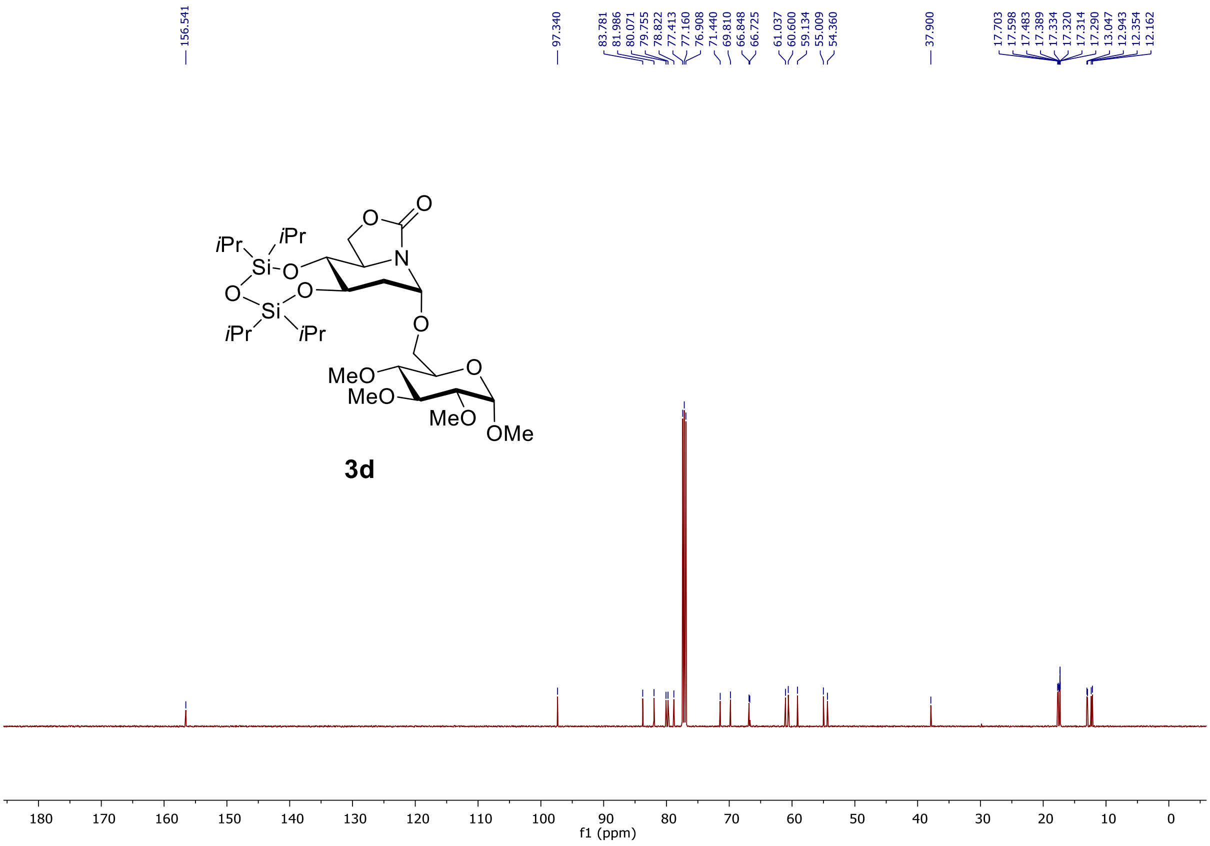

Supplementary Figure S214. ^{13}C NMR spectra for 3d

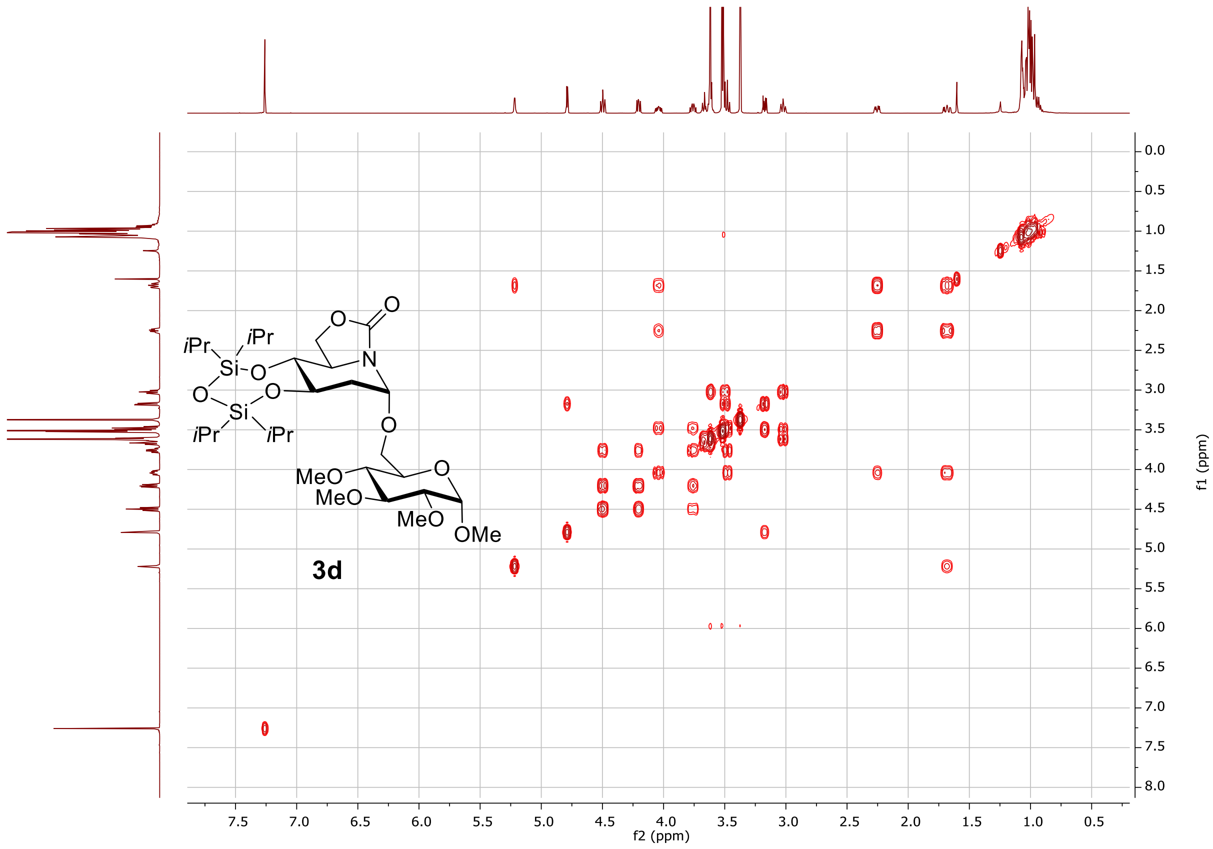

Supplementary Figure S215. COSY spectra for 3d

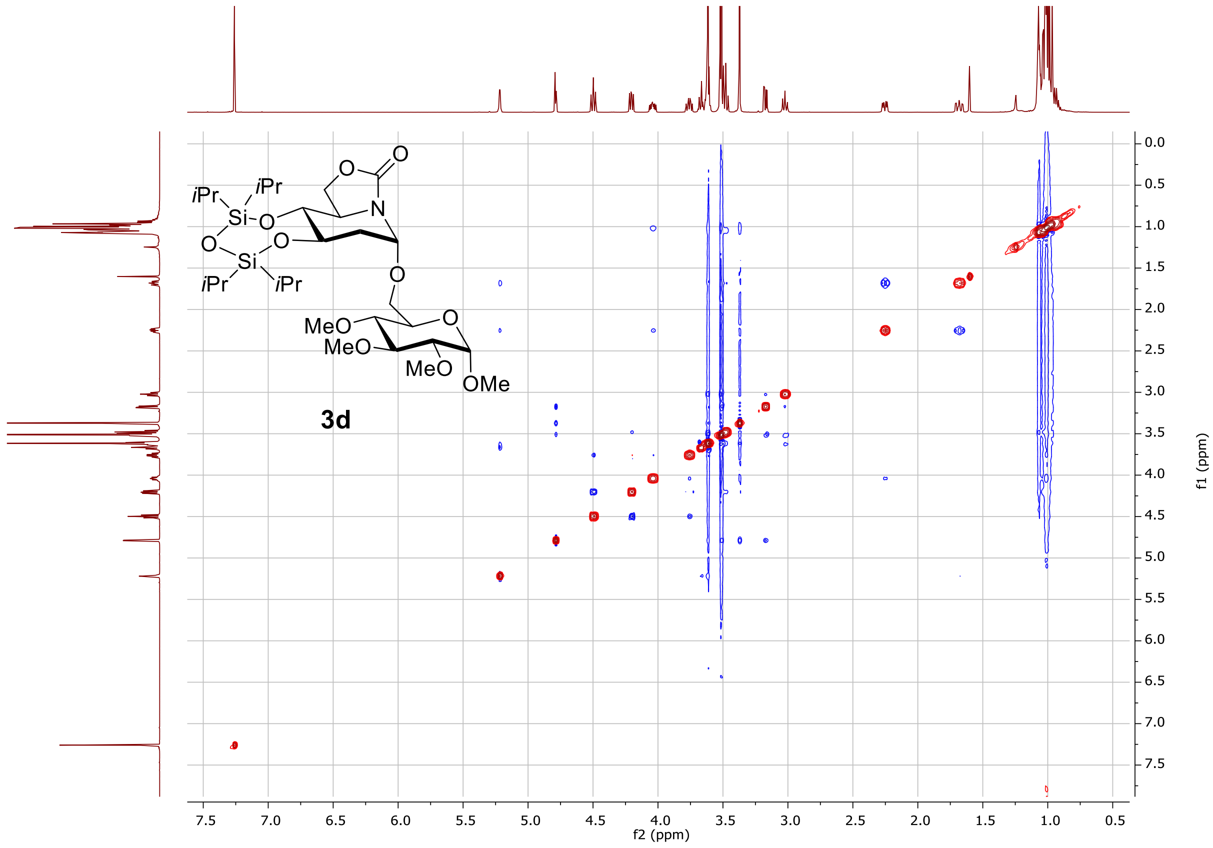

Supplementary Figure S216. NOESY spectra for 3d

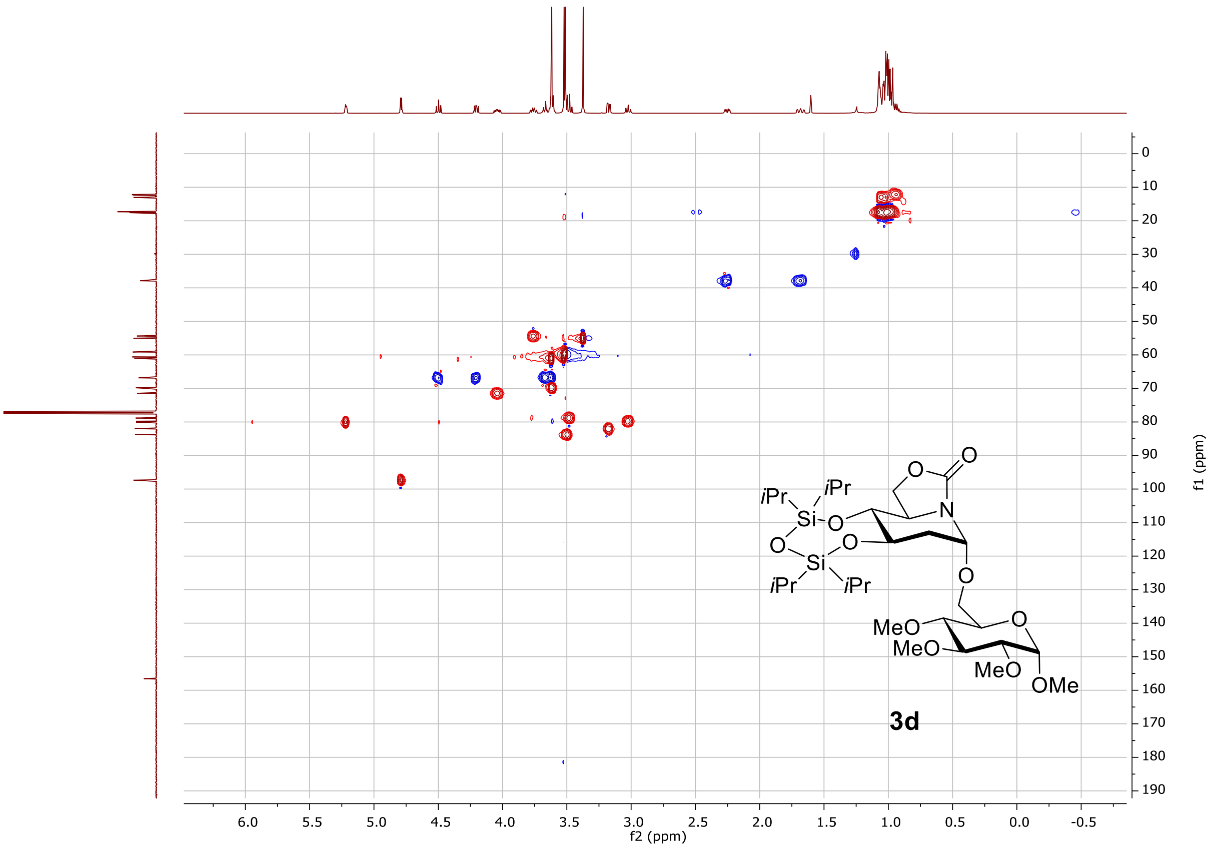

Supplementary Figure S217. HSQC spectra for 3d

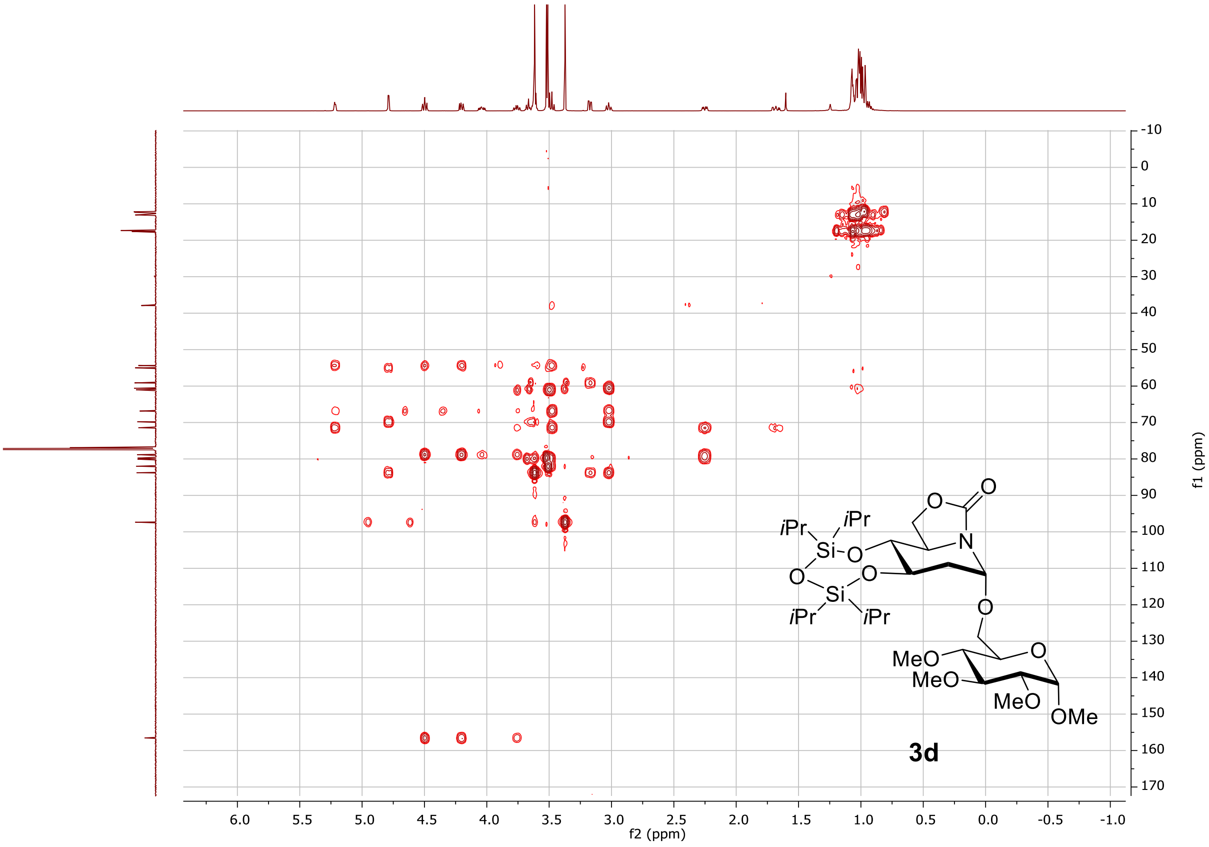

Supplementary Figure S218. HMBC spectra for 3d

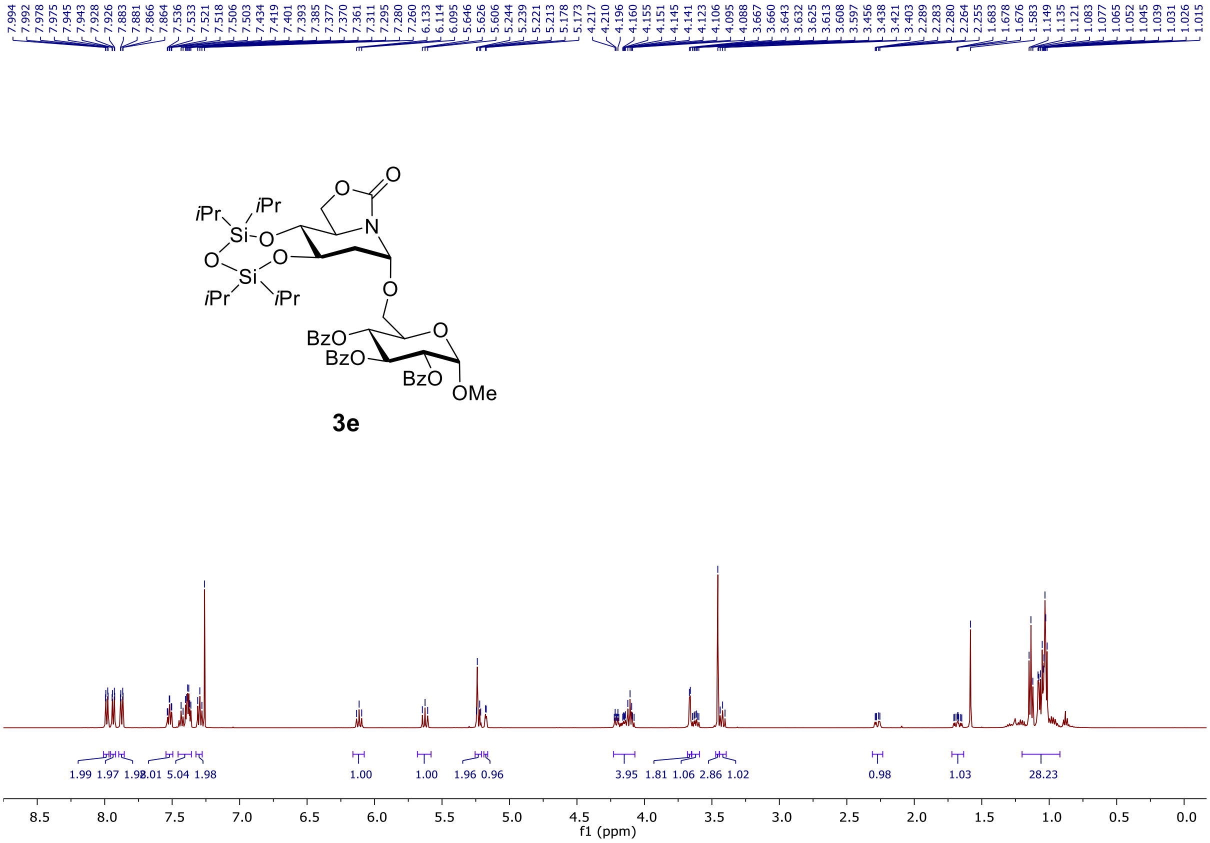

Supplementary Figure S219. ^{1}H NMR spectra for 3e

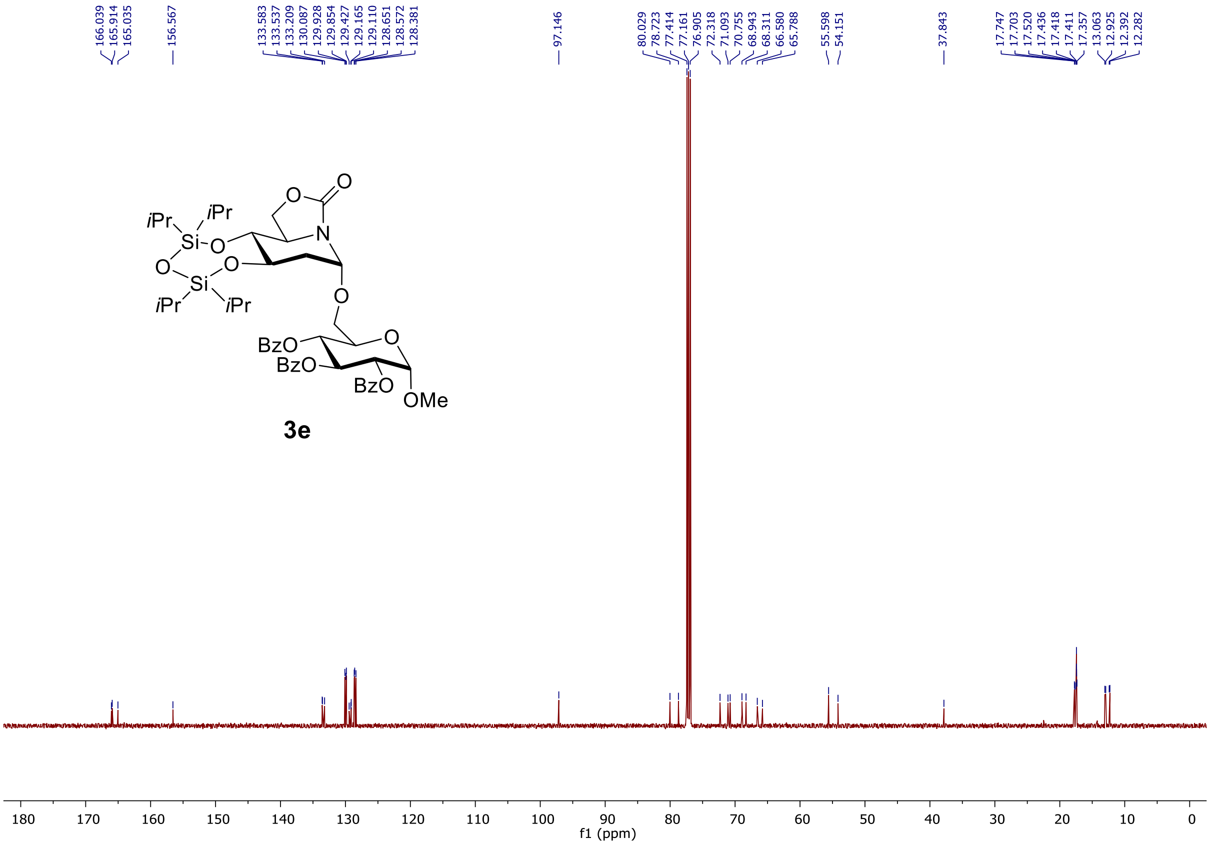

Supplementary Figure S220. ^{13}C NMR spectra for 3e

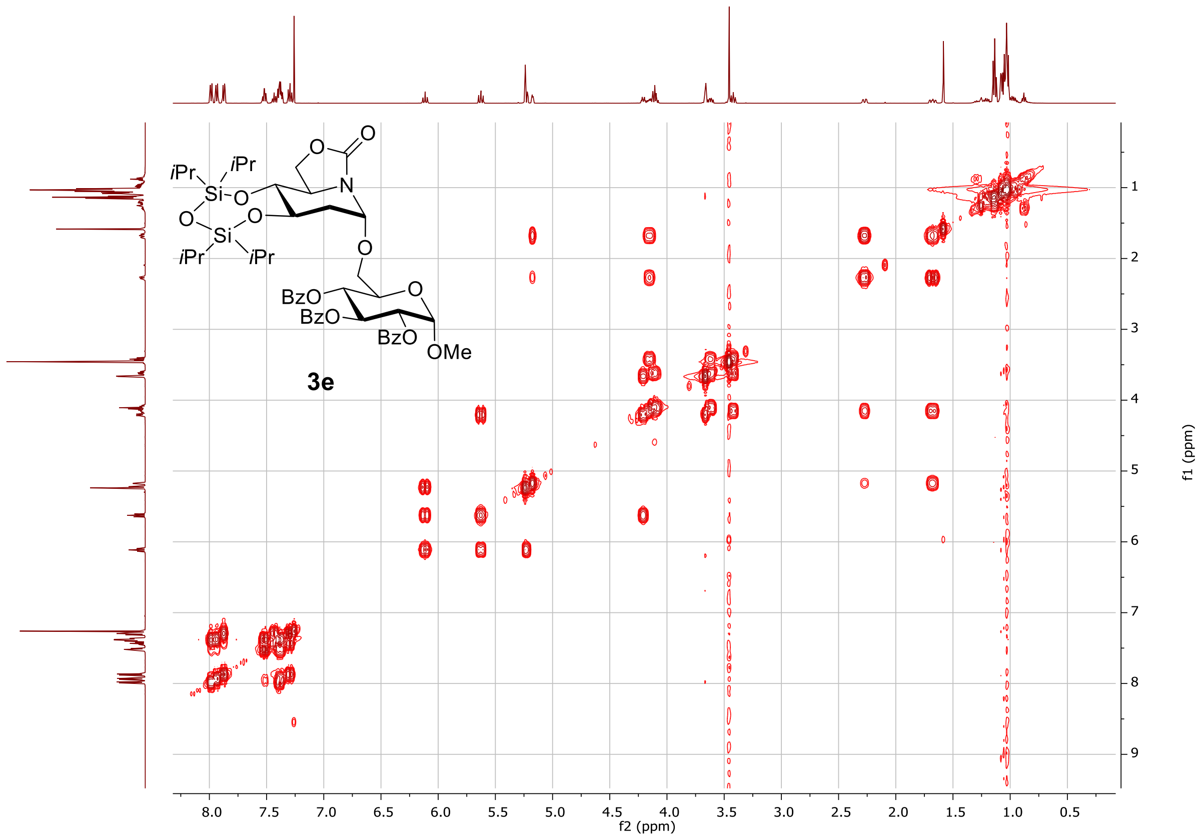

Supplementary Figure S221. COSY spectra for 3e

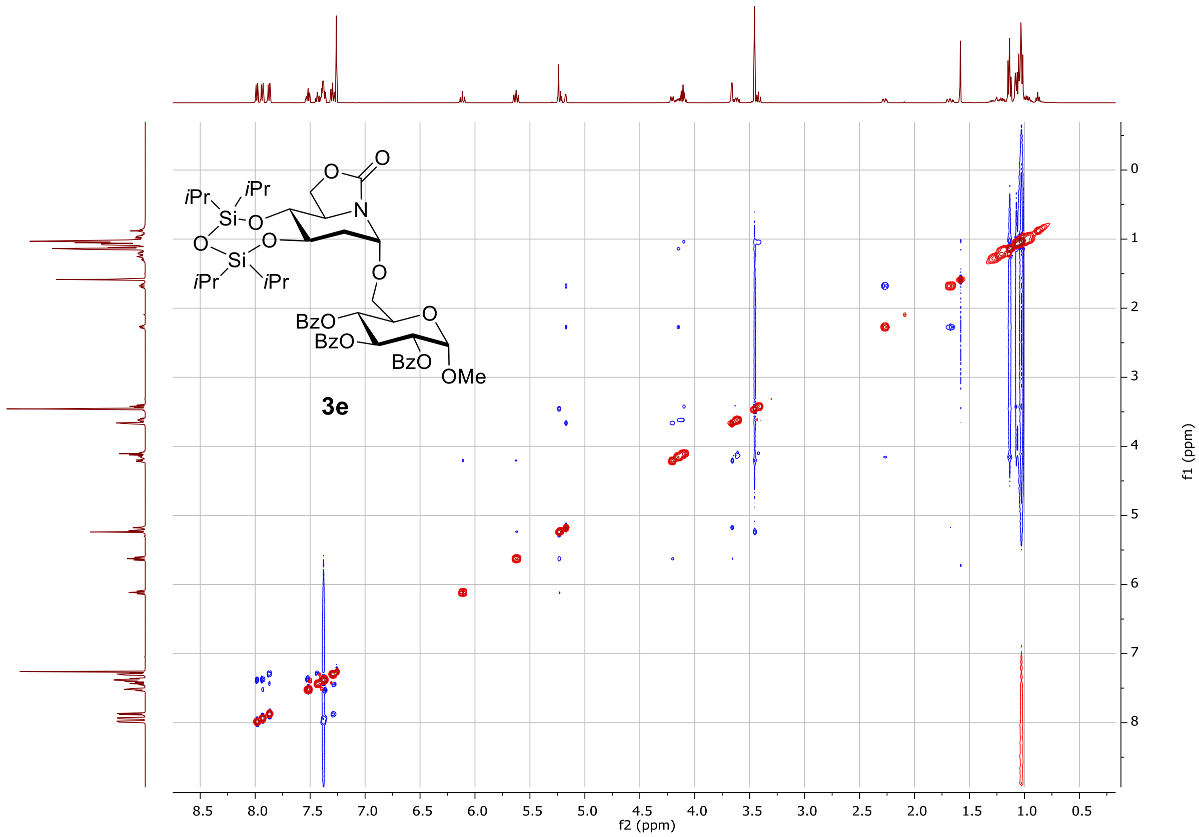

Supplementary Figure S222. NOESY spectra for 3e

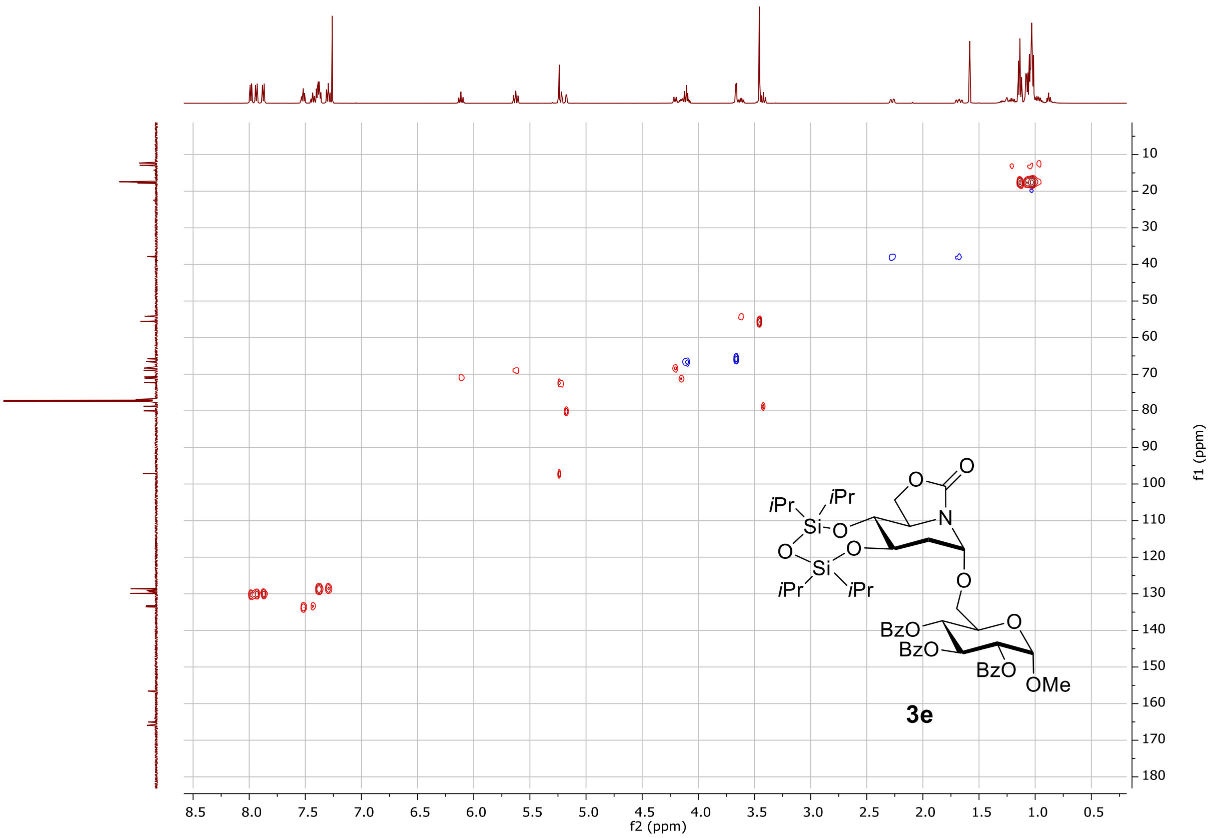

Supplementary Figure S223. HSQC spectra for 3e

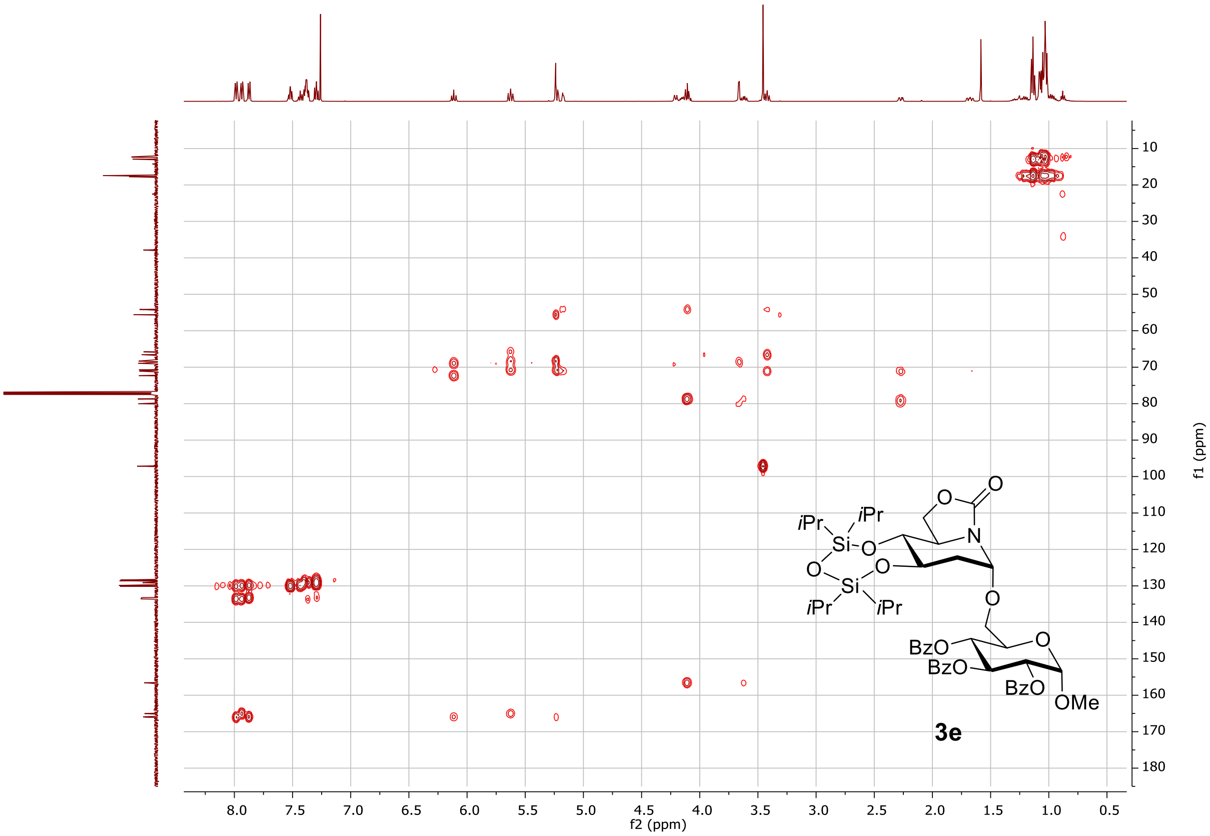

Supplementary Figure S224. HMBC spectra for 3e

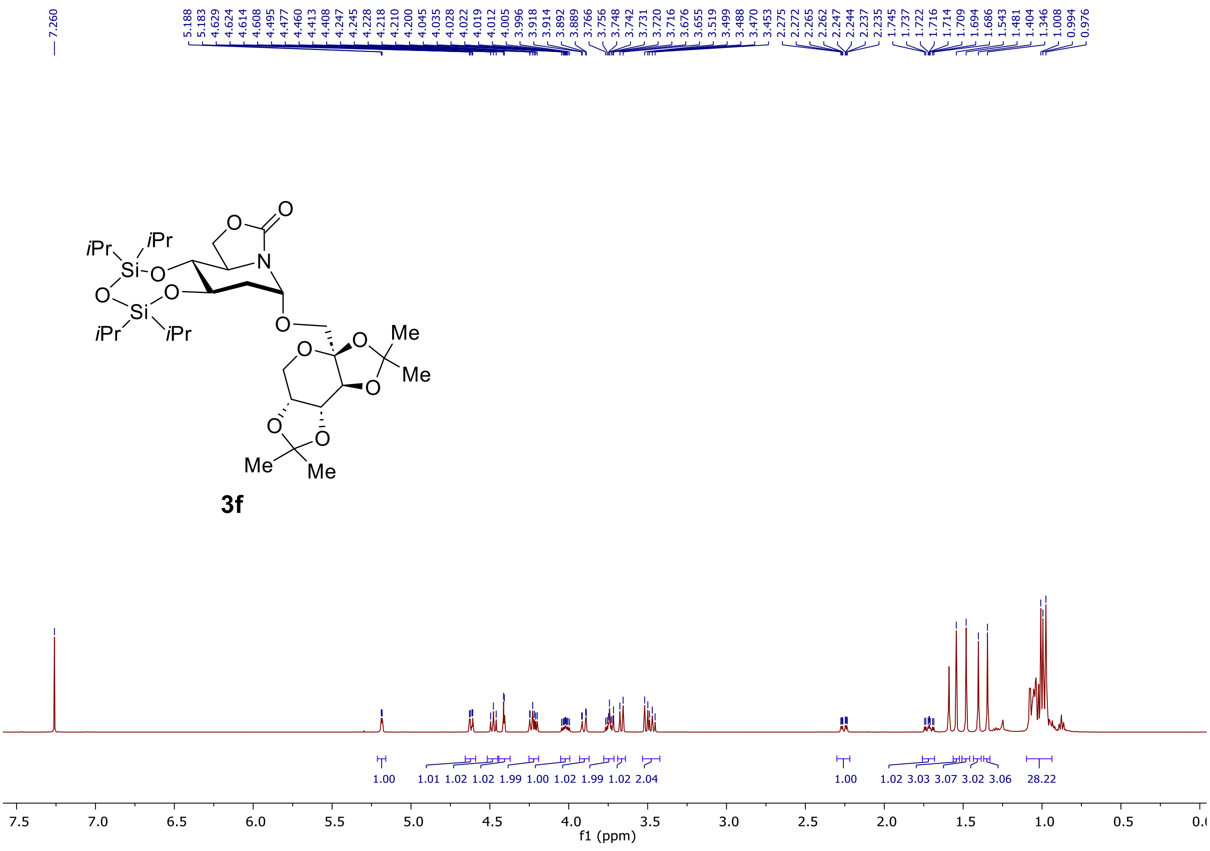

Supplementary Figure S225. ^{1}H NMR spectra for 3f

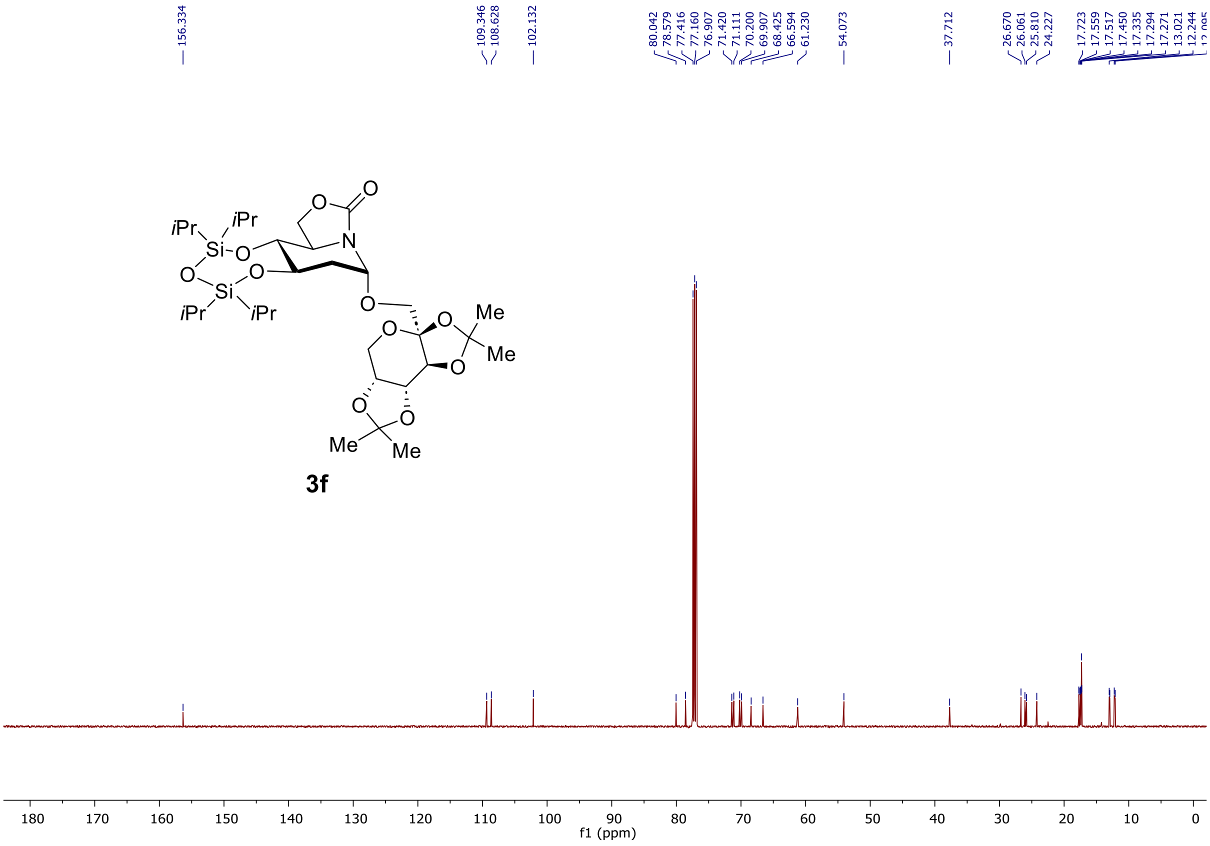

Supplementary Figure S226. ^{13}C NMR spectra for 3f

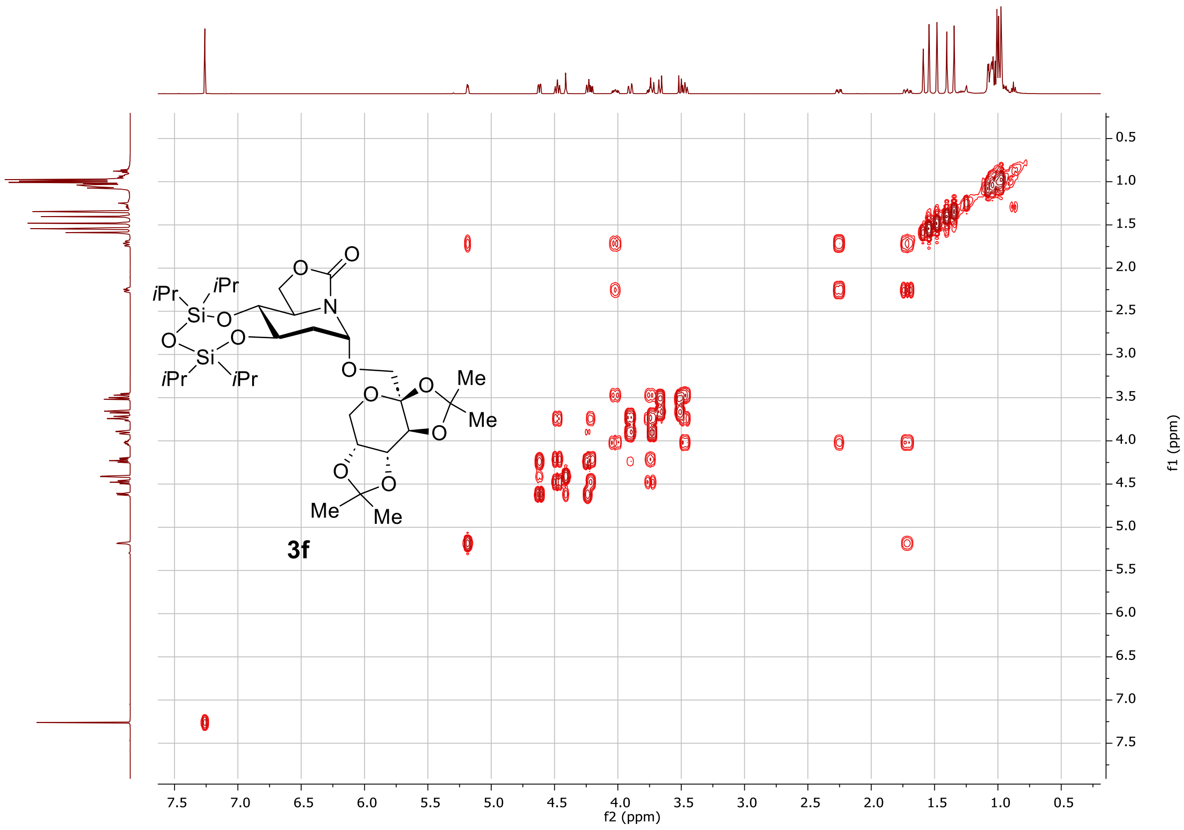

Supplementary Figure S227. COSY spectra for 3f

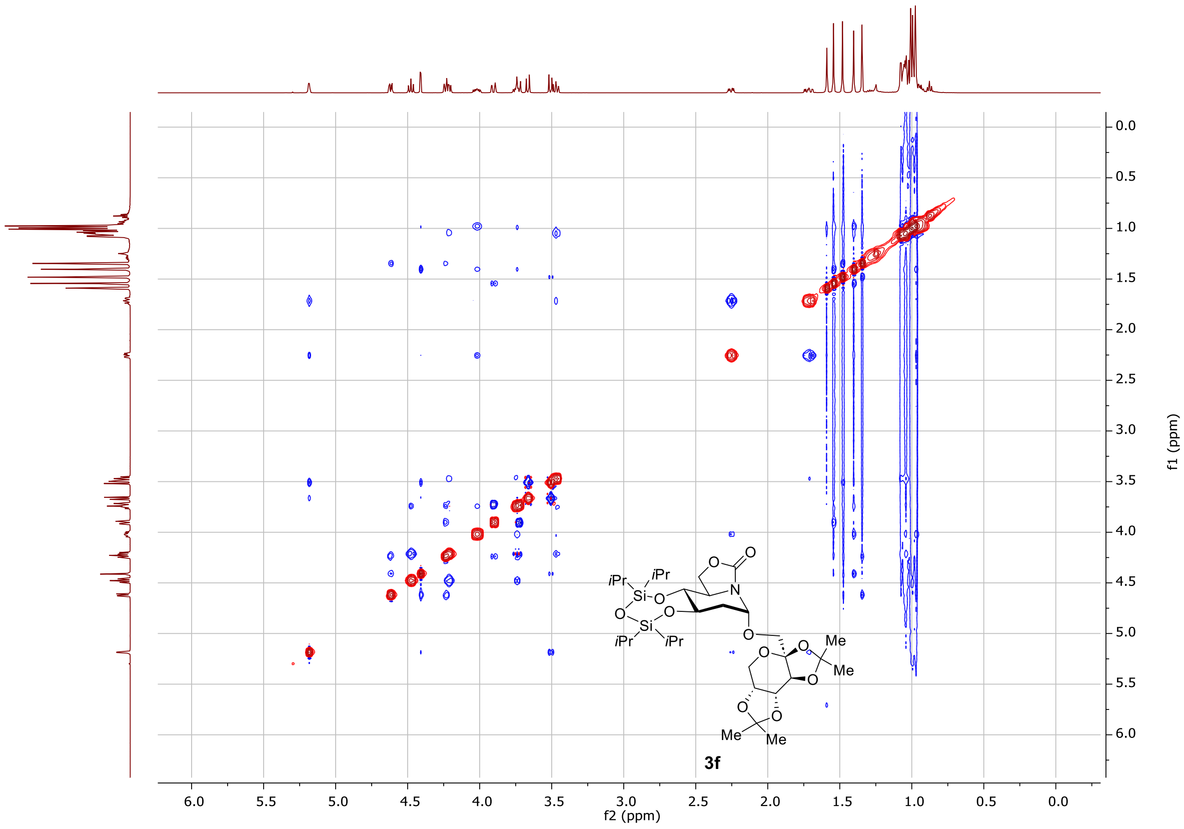

Supplementary Figure S228. NOESY spectra for 3f

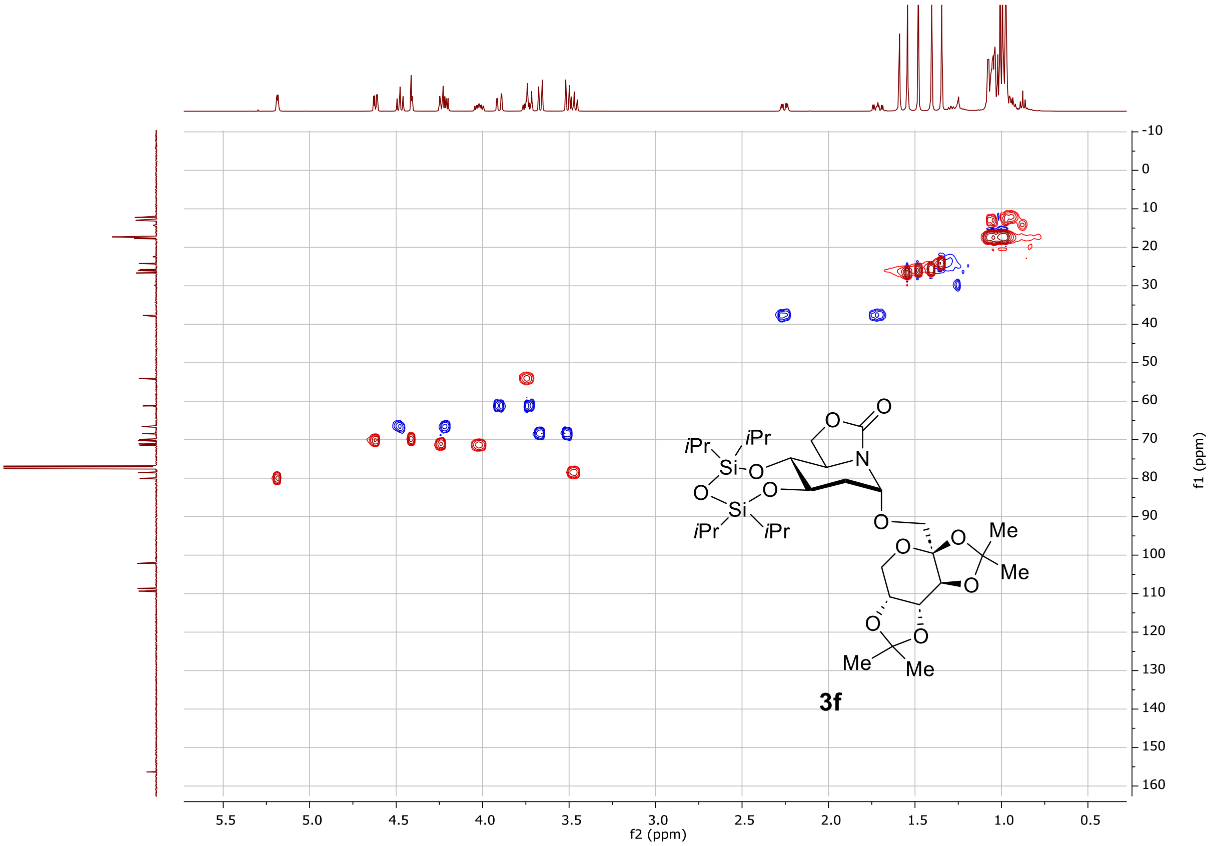

Supplementary Figure S229. HSQC spectra for 3f

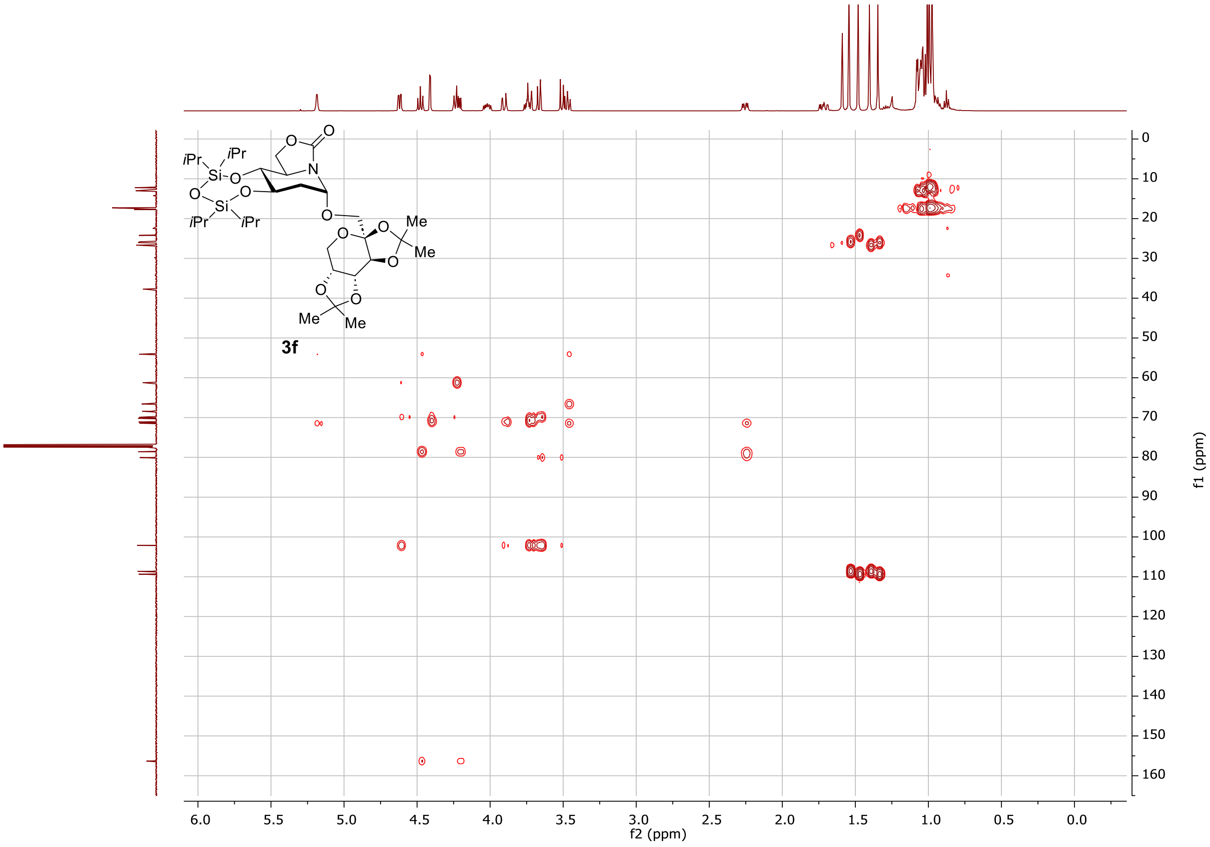

Supplementary Figure S230. HMBC spectra for 3f

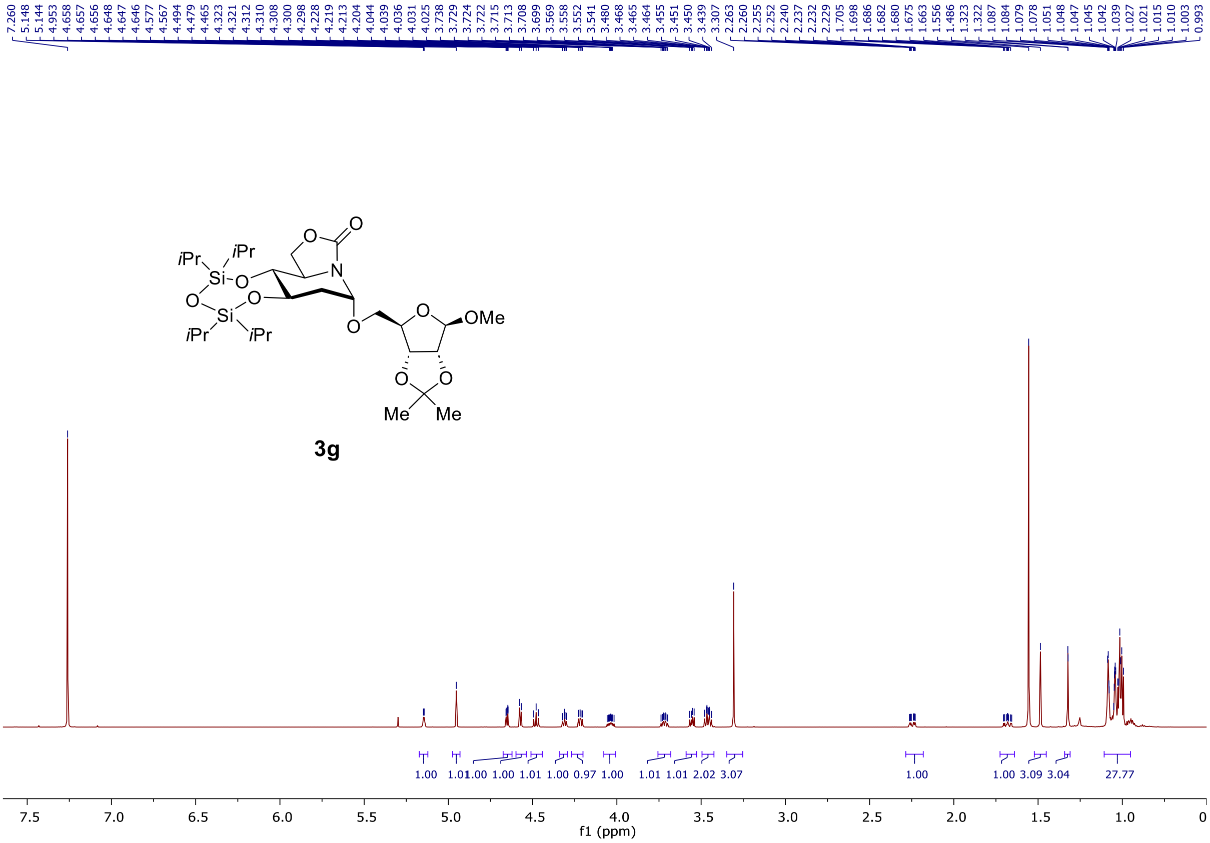

Supplementary Figure S231. ^{1}H NMR spectra for 3g

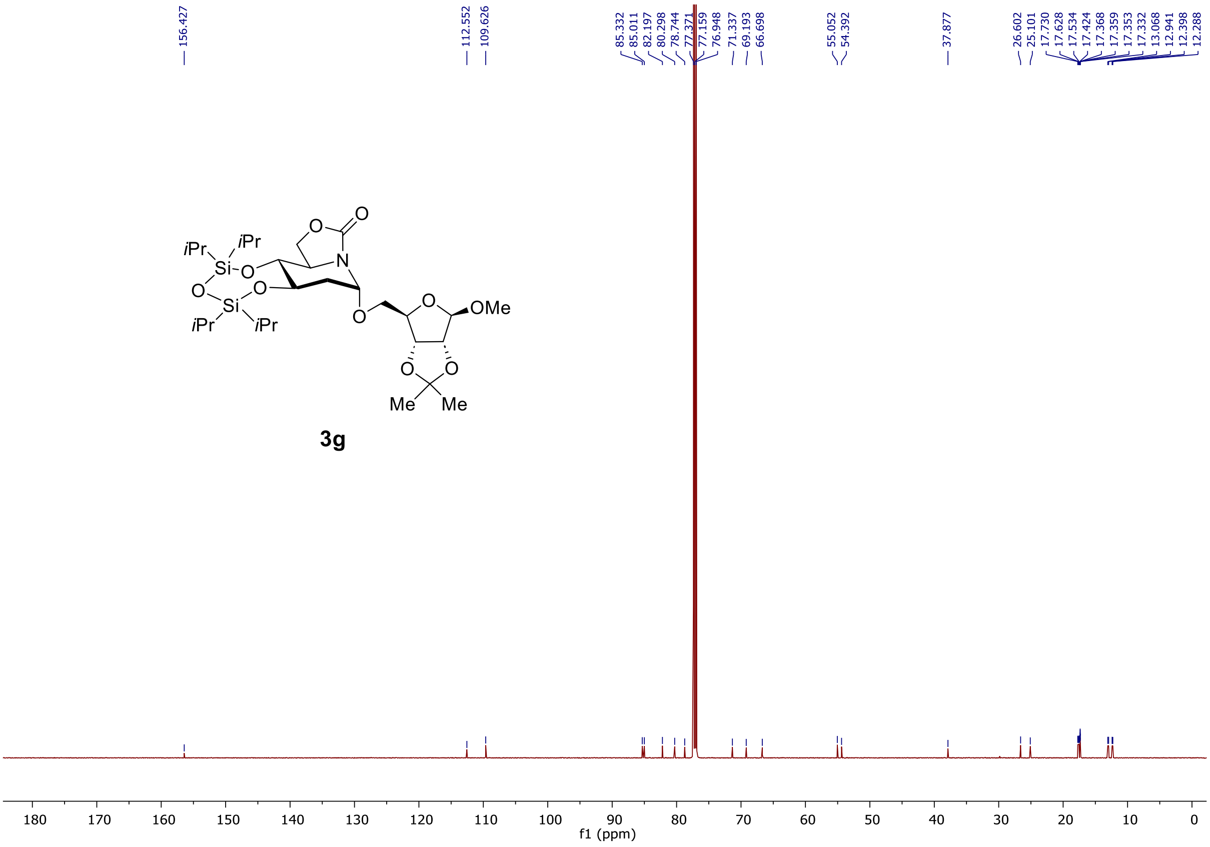

Supplementary Figure S232. ^{13}C NMR spectra for 3g

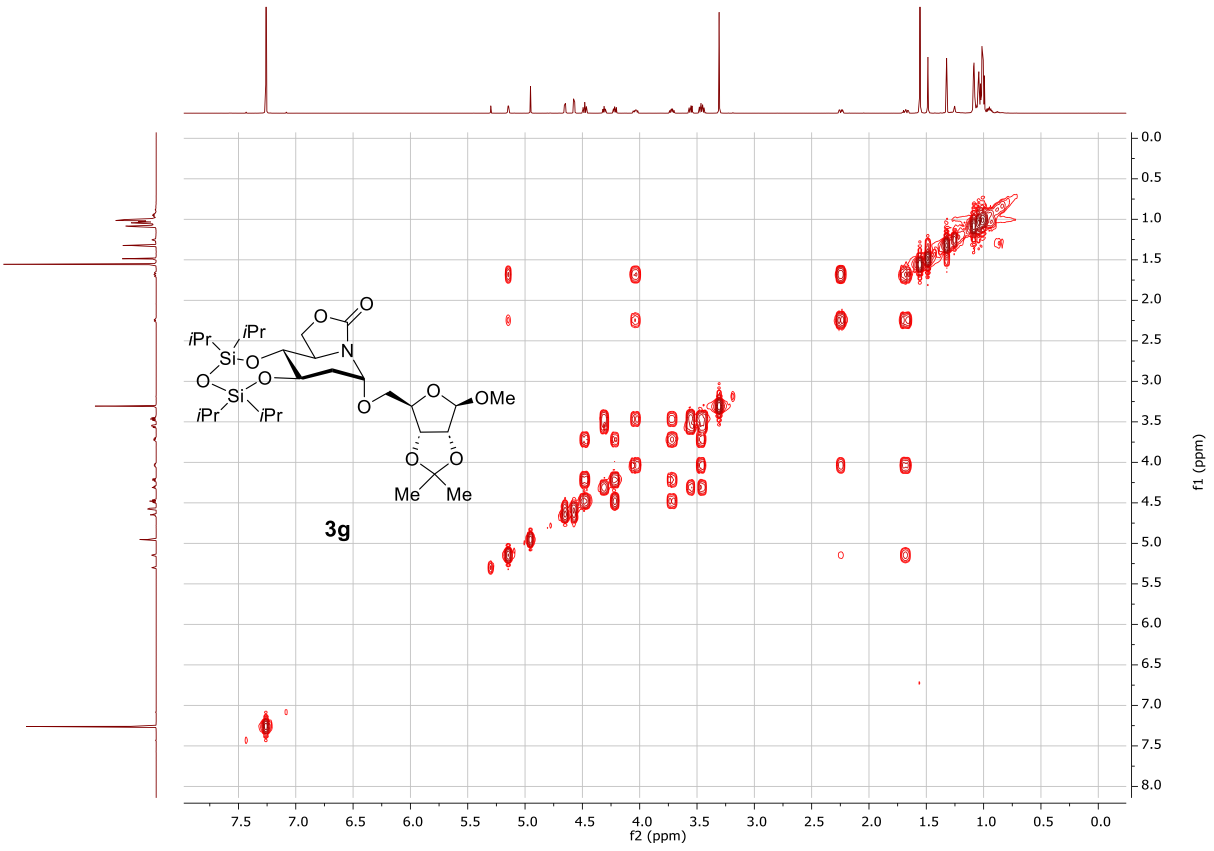

Supplementary Figure S233. COSY spectra for 3g

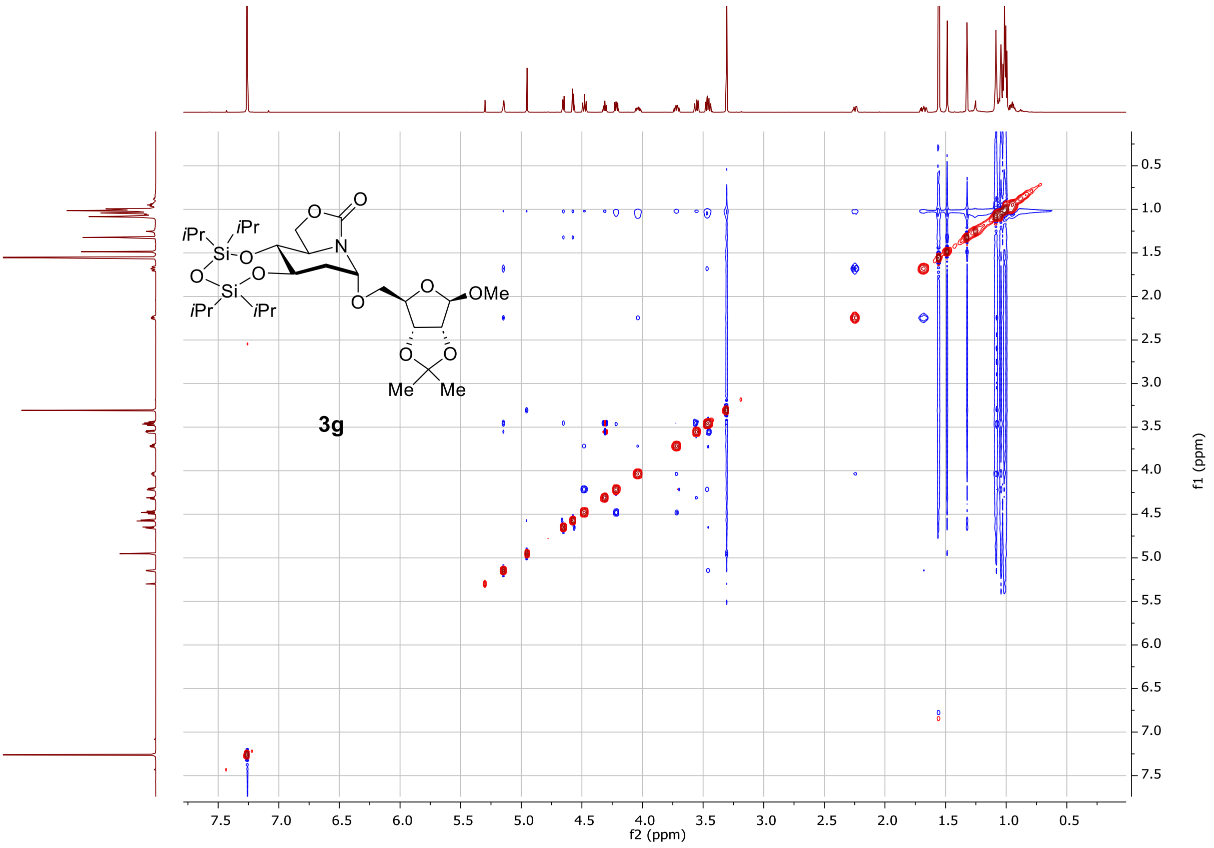

Supplementary Figure S234. NOESY spectra for 3g

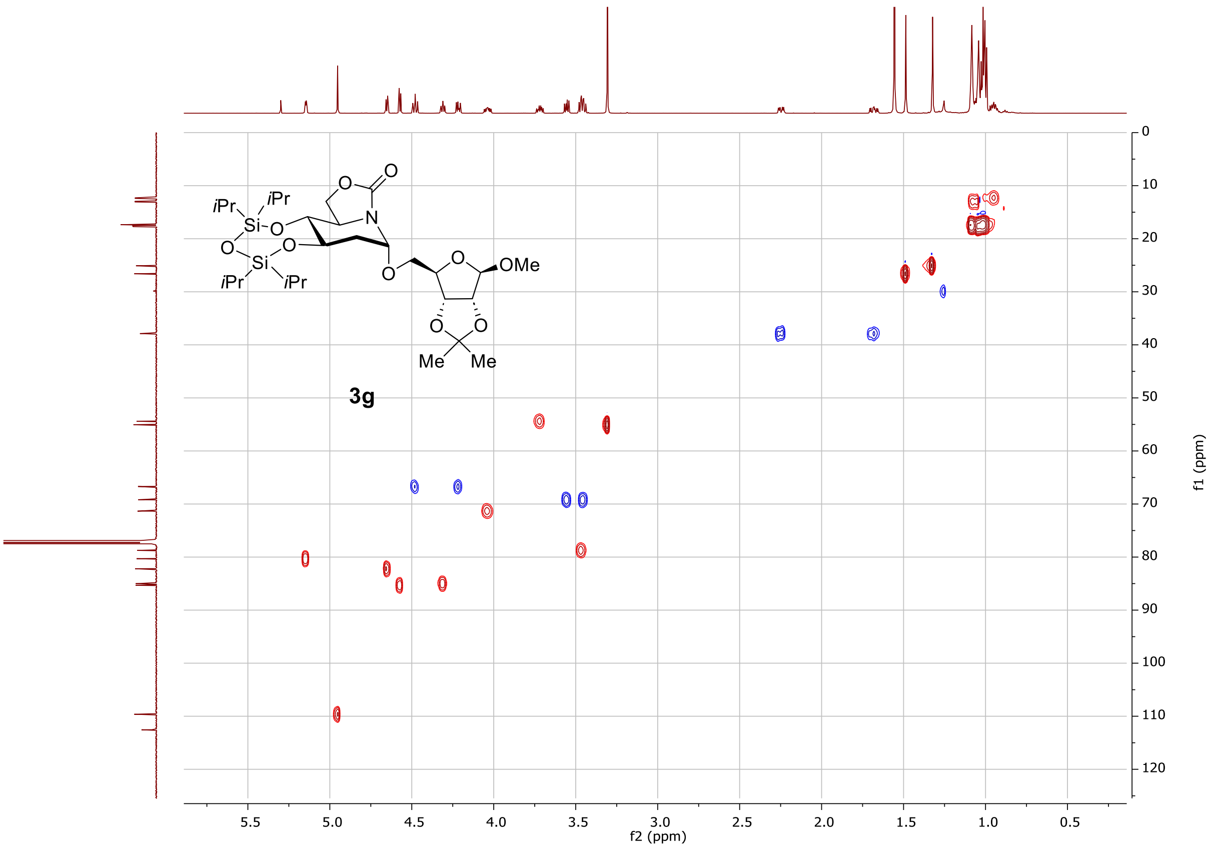

Supplementary Figure S235. HSQC spectra for 3g

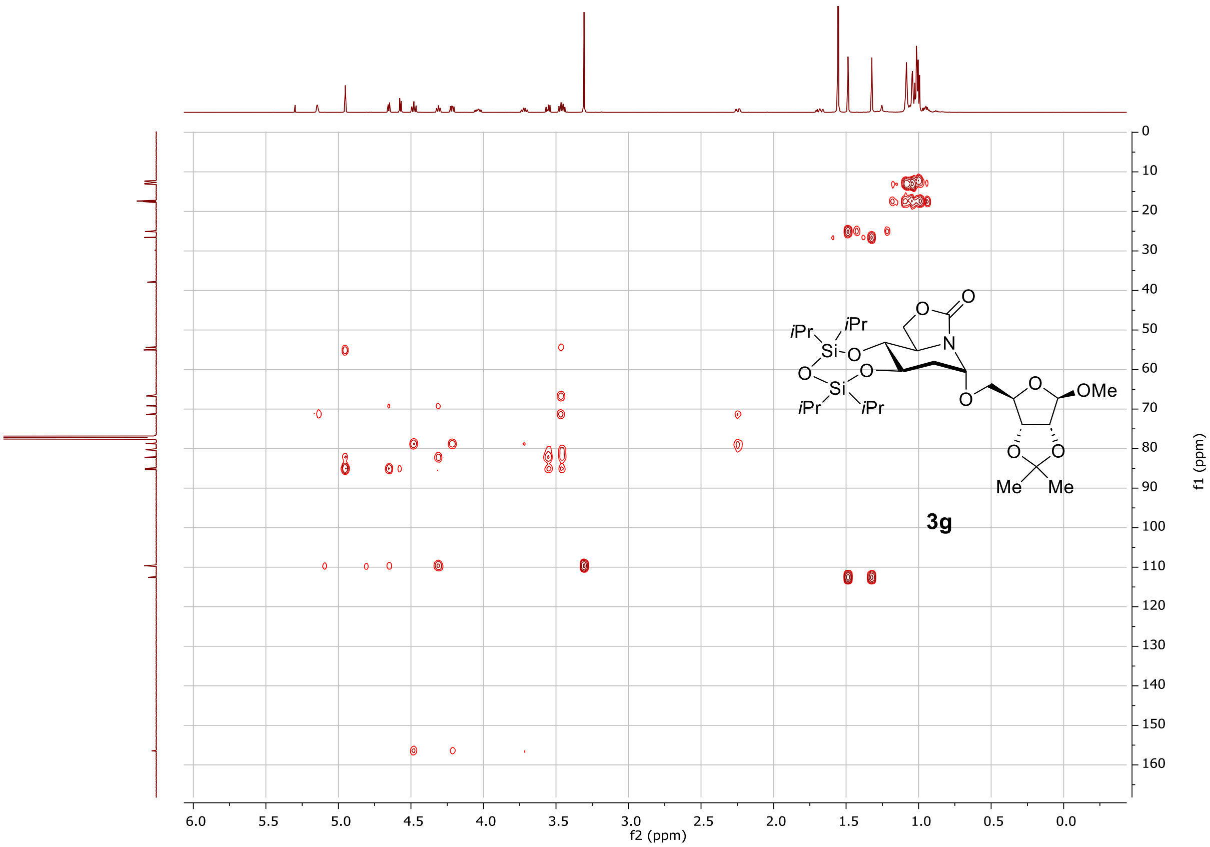

Supplementary Figure S236. HMBC spectra for 3g

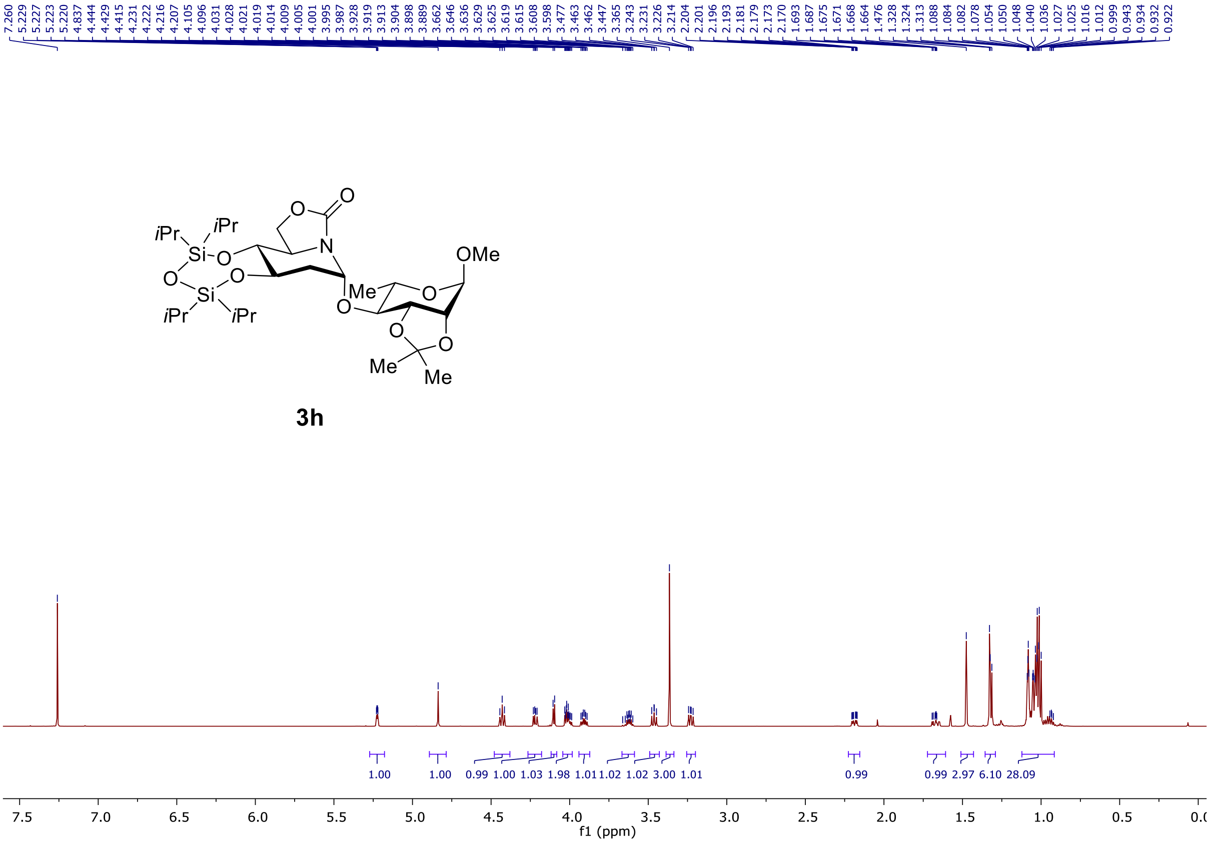

Supplementary Figure S237. ^{1}H NMR spectra for 3h

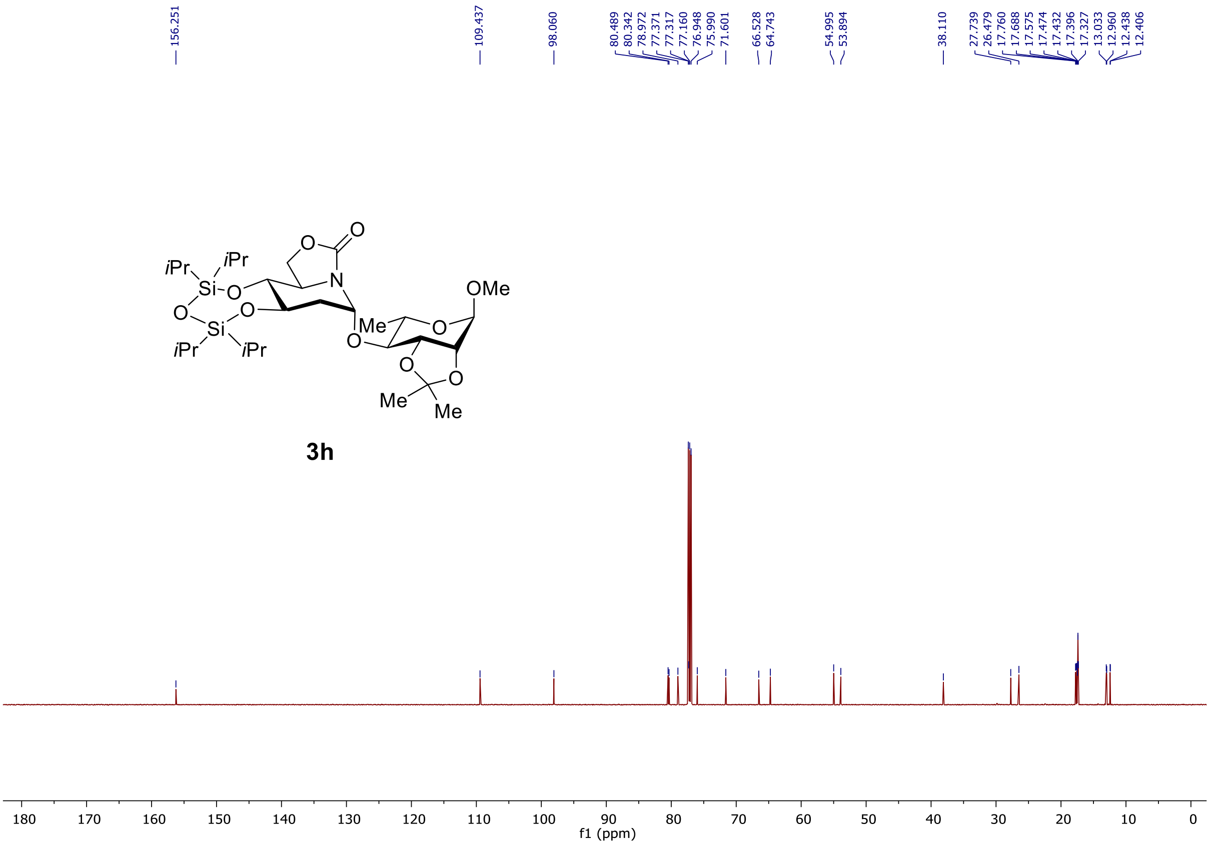

Supplementary Figure S238. ^{13}C NMR spectra for 3h

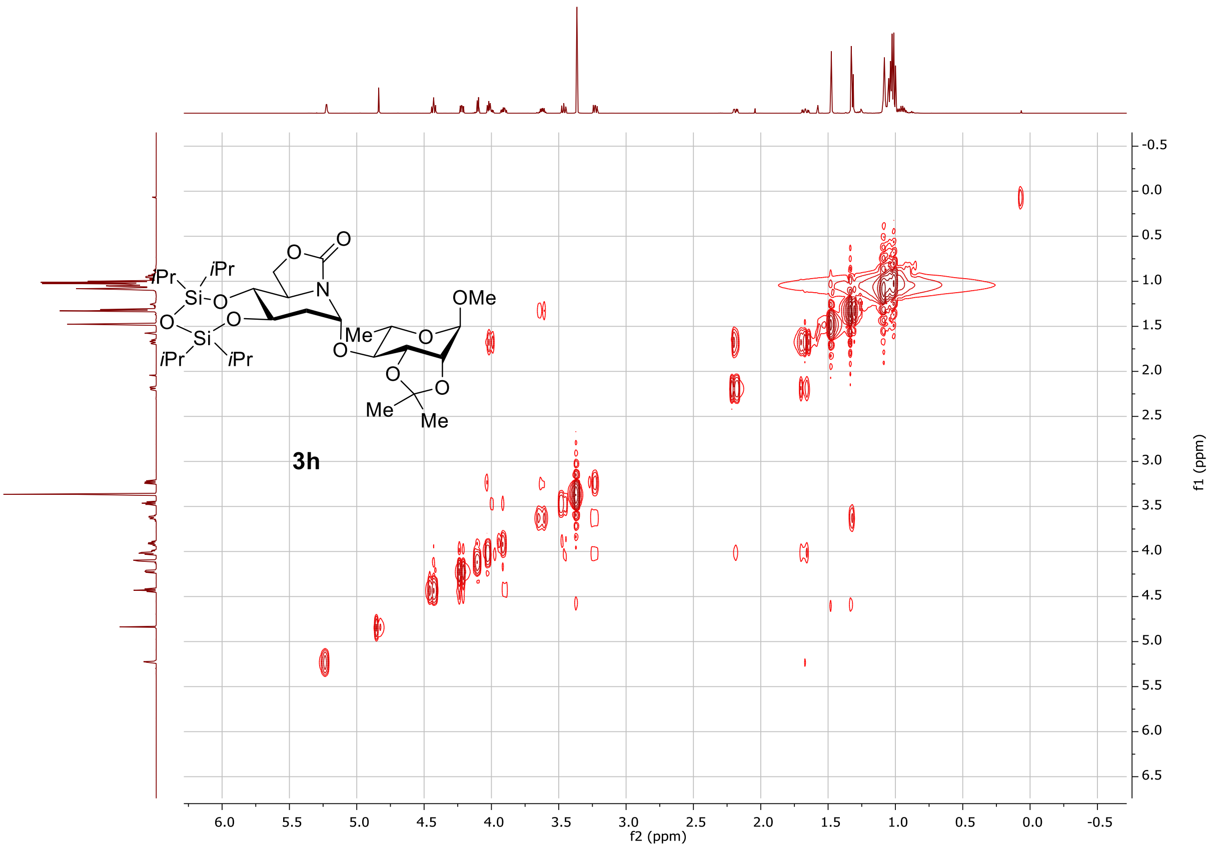

Supplementary Figure S239. COSY spectra for 3h

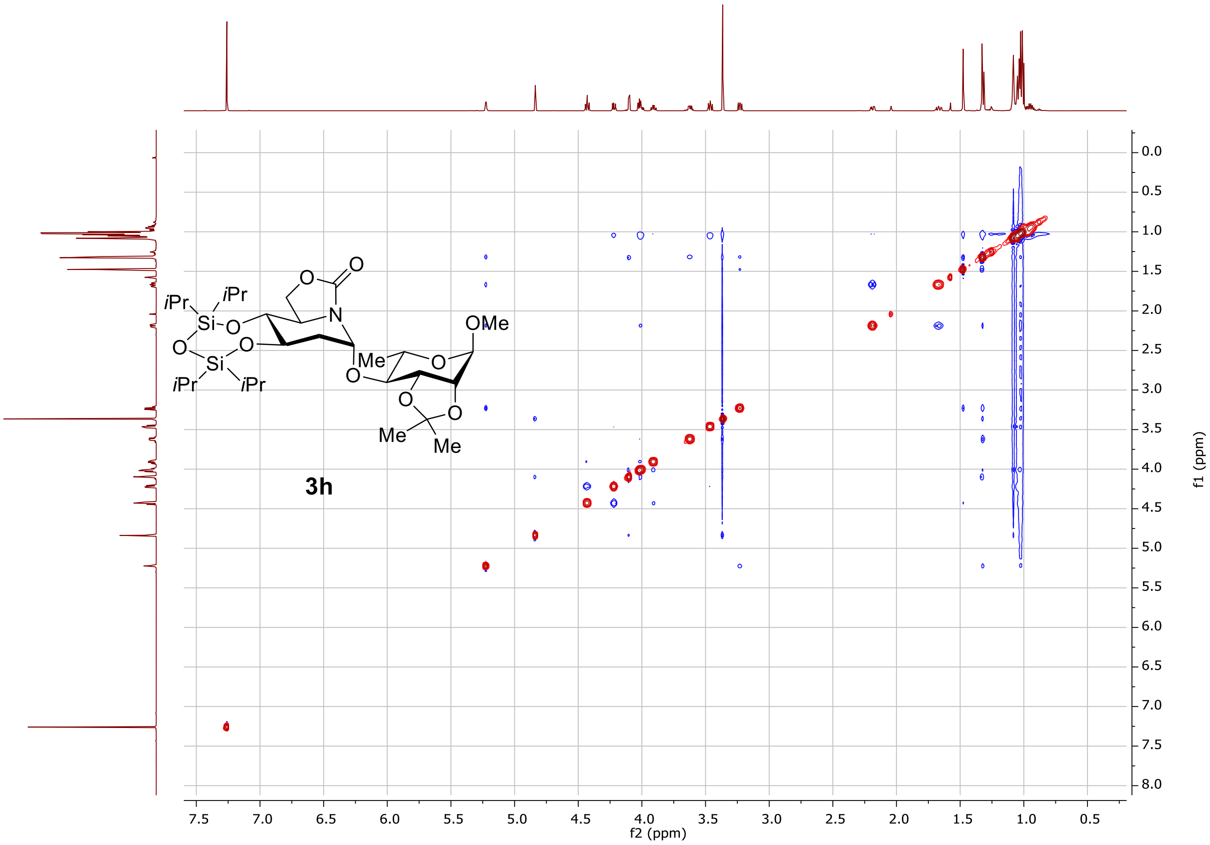

Supplementary Figure S240. NOESY spectra for 3g

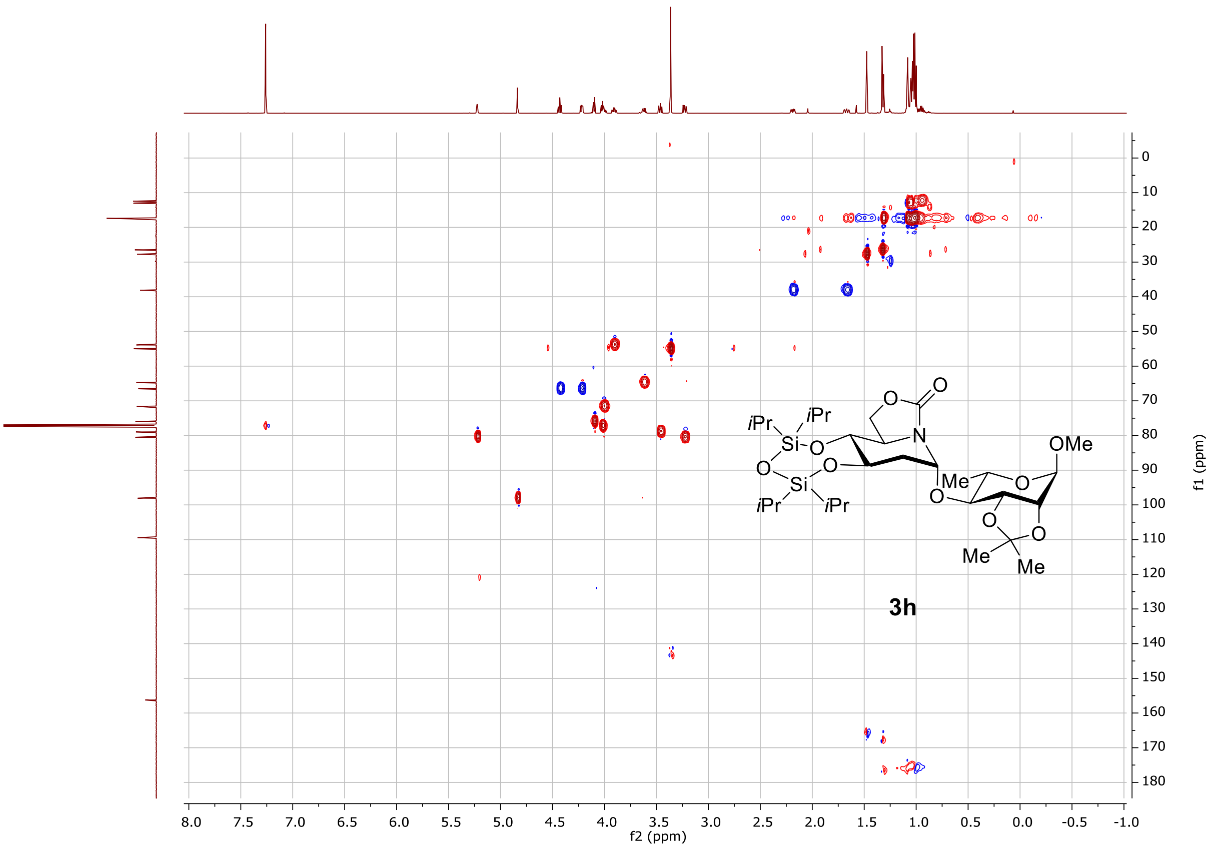

Supplementary Figure S241. HSQC spectra for 3h

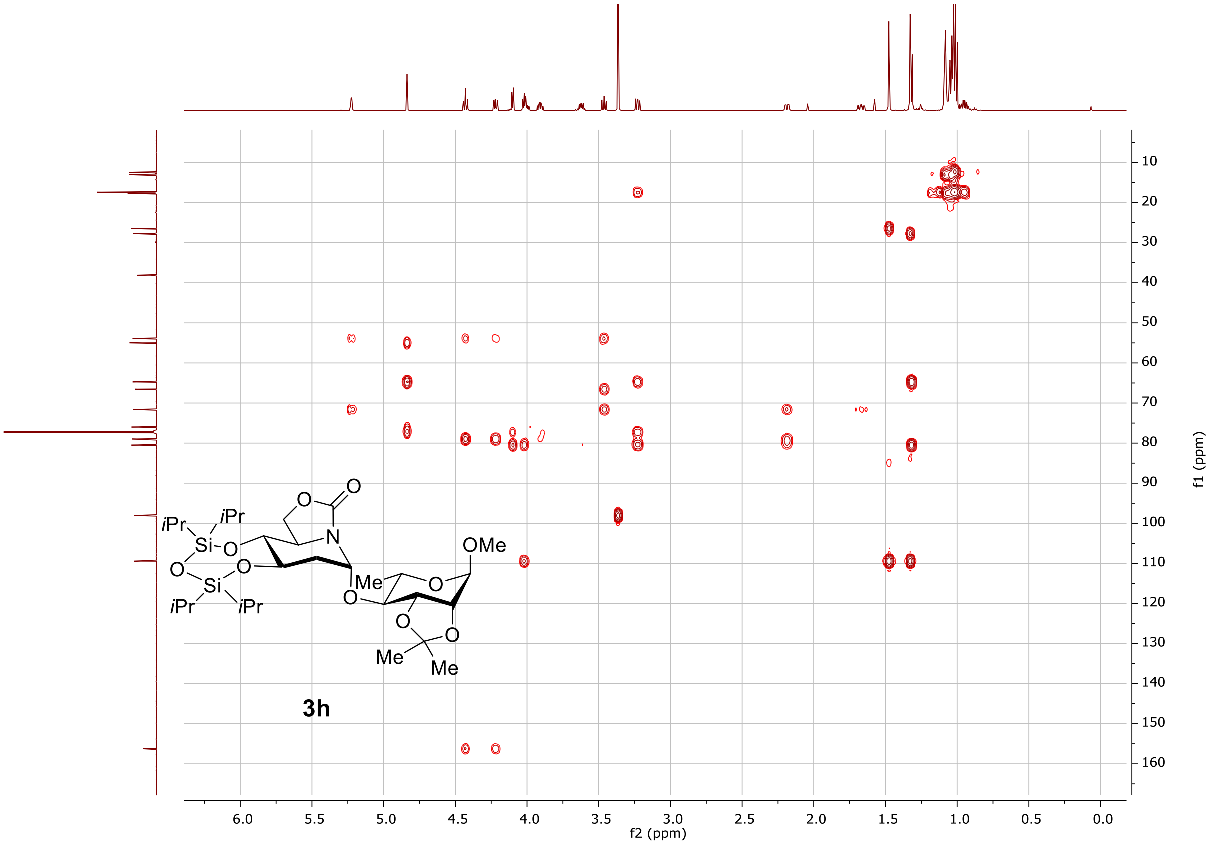

Supplementary Figure S242. HMBC spectra for 3h

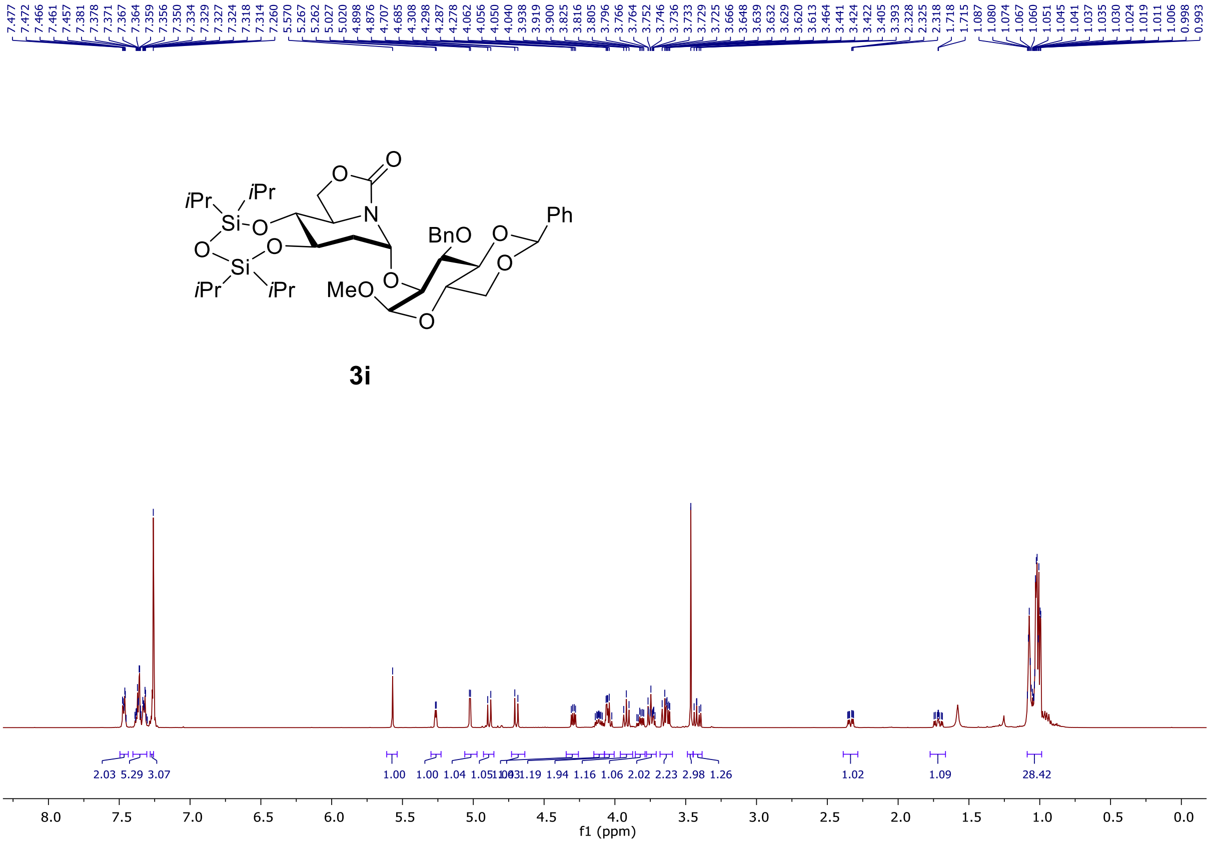

Supplementary Figure S243. ^{1}H NMR spectra for 3i

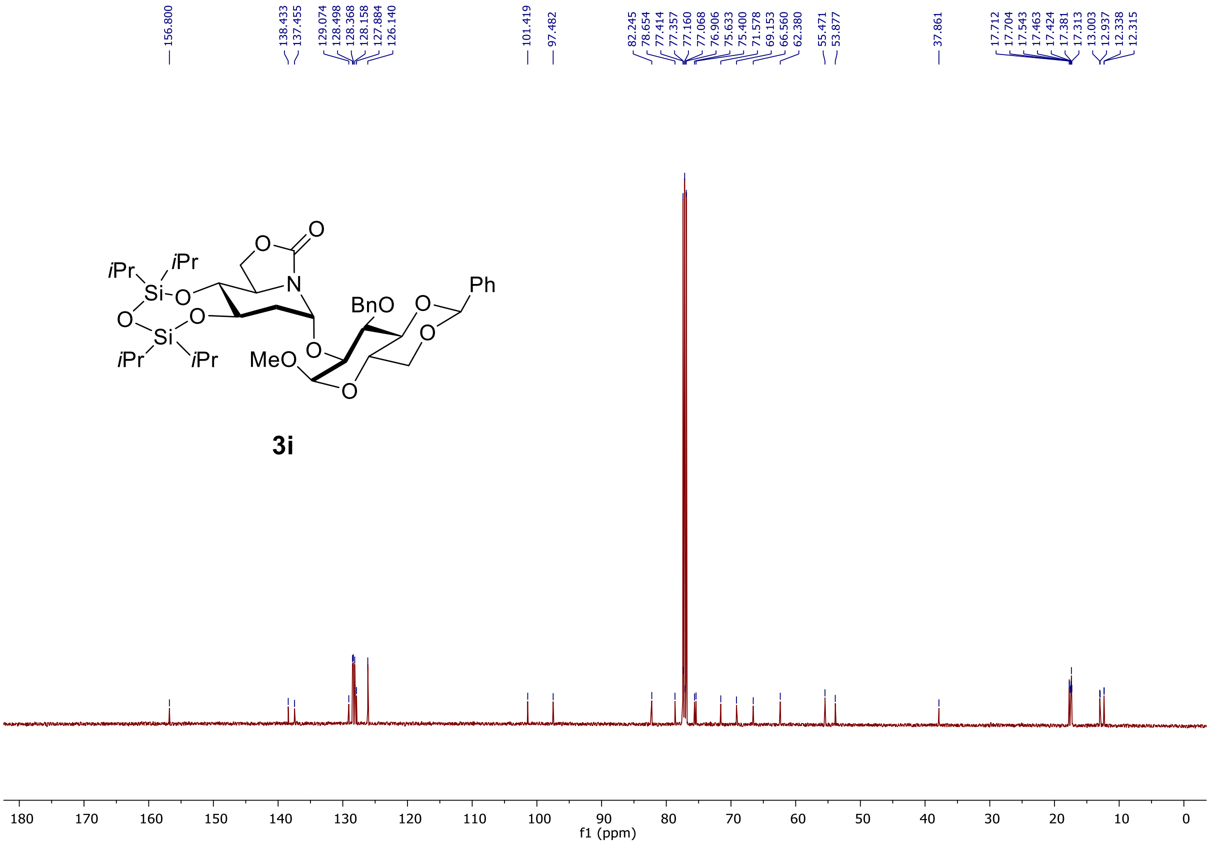

Supplementary Figure S244. ^{13}C NMR spectra for 3i

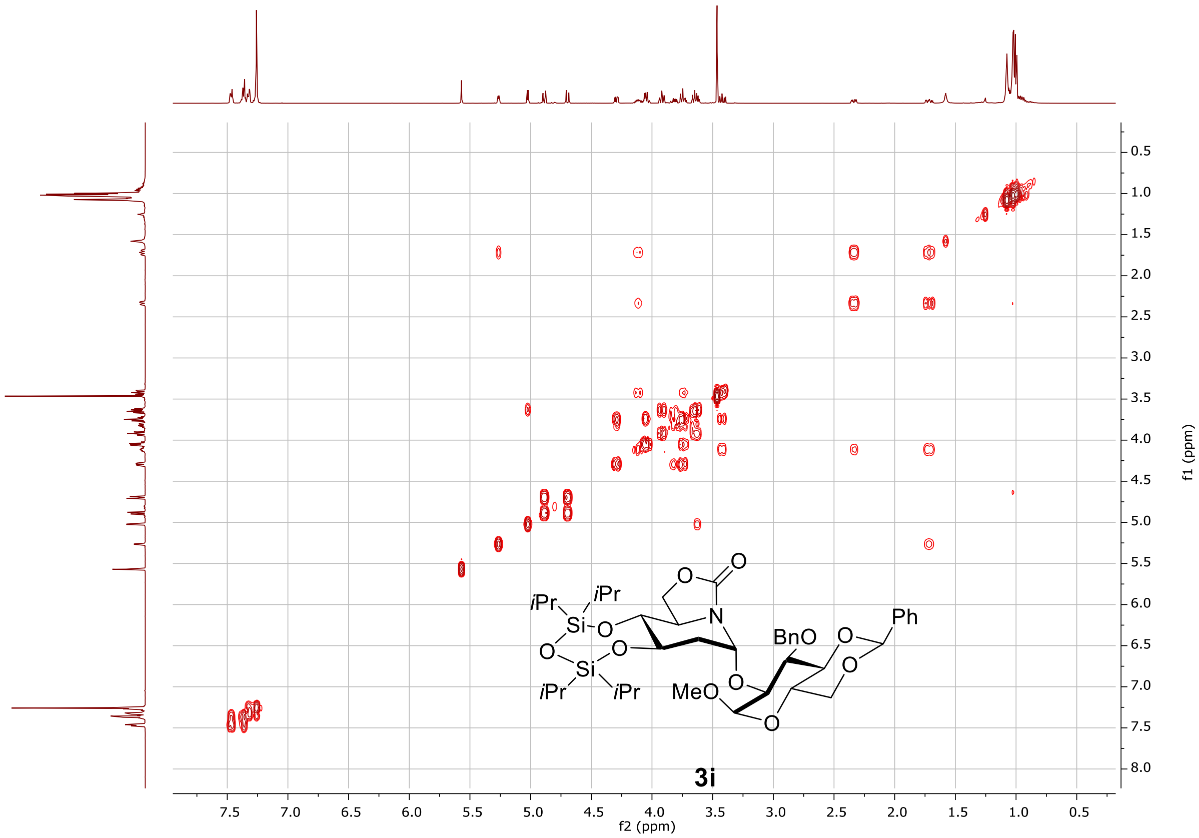

Supplementary Figure S245. COSY spectra for 3i

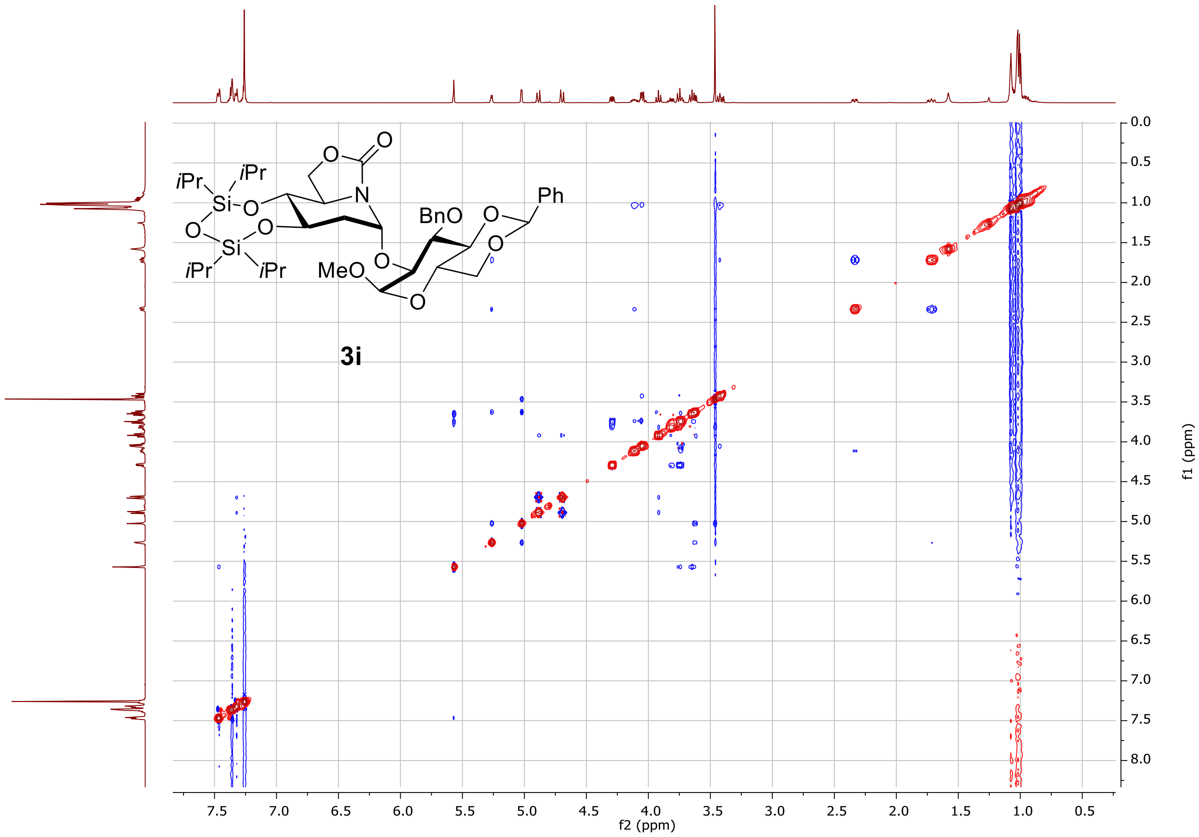

Supplementary Figure S246. NOESY spectra for 3i

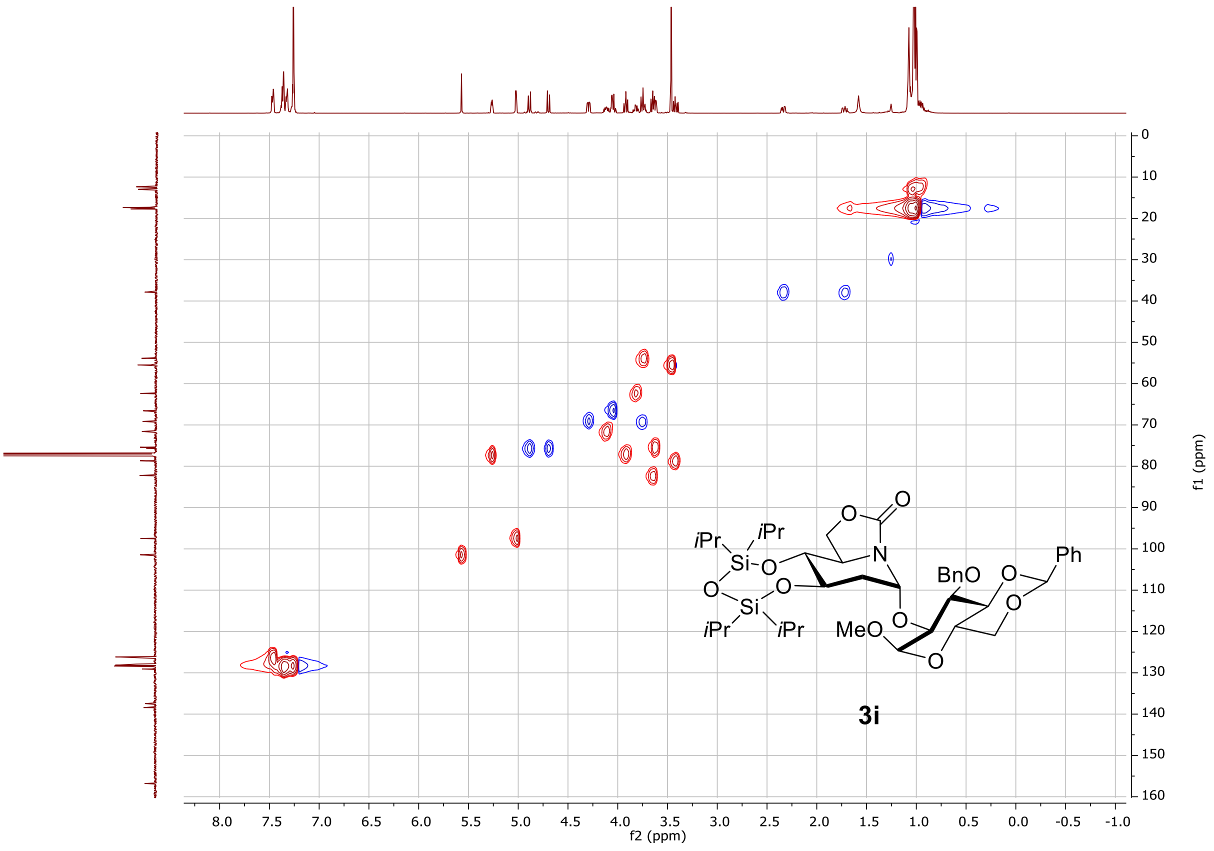

Supplementary Figure S247. HSQC spectra for 3i

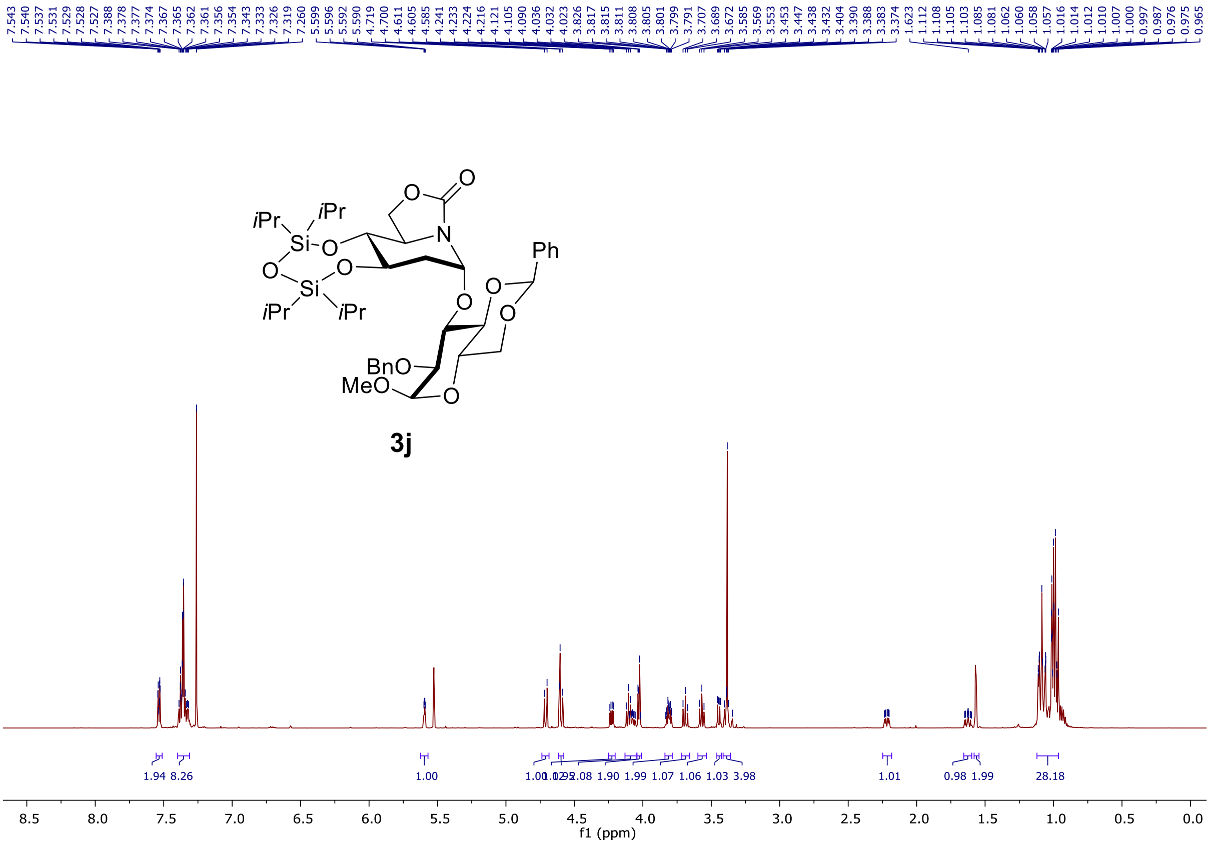

Supplementary Figure S248. ^{1}H NMR spectra for 3j

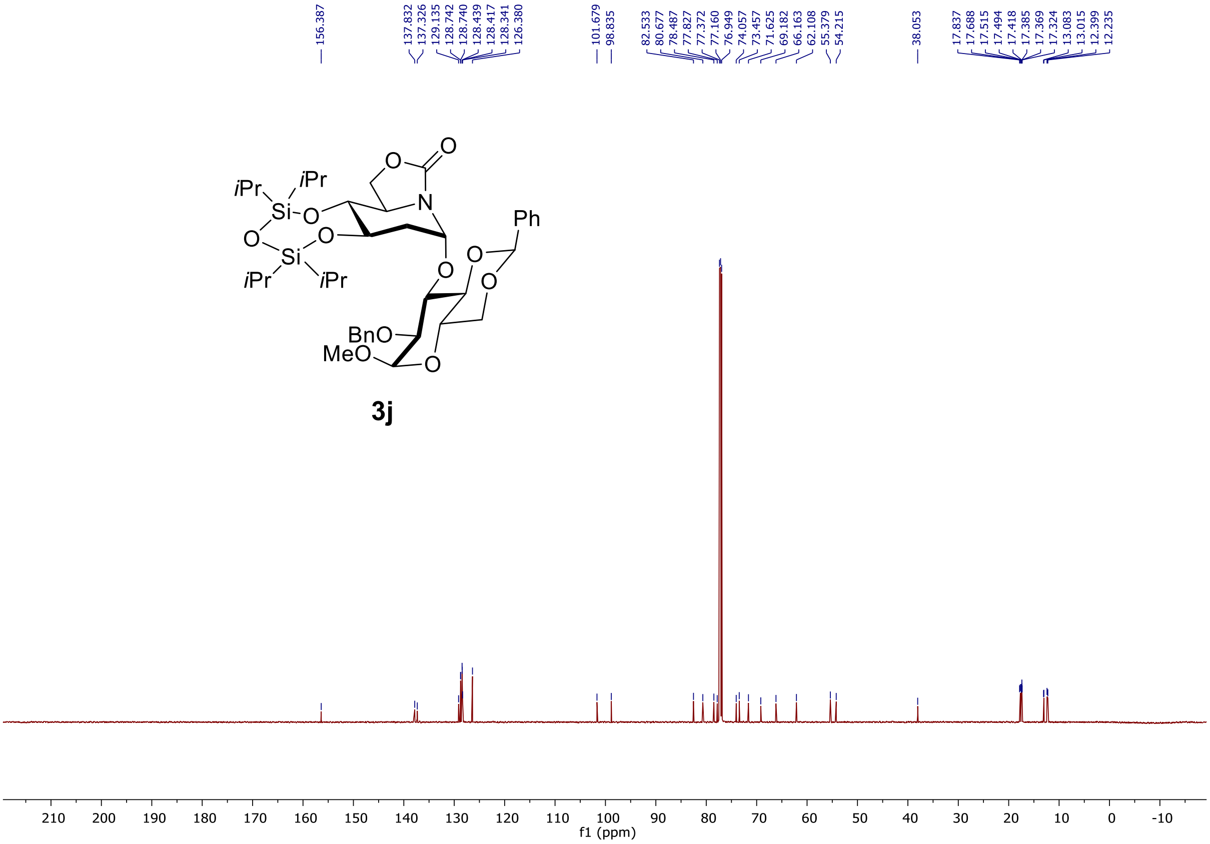

Supplementary Figure S249. ^{13}C NMR spectra for 3j

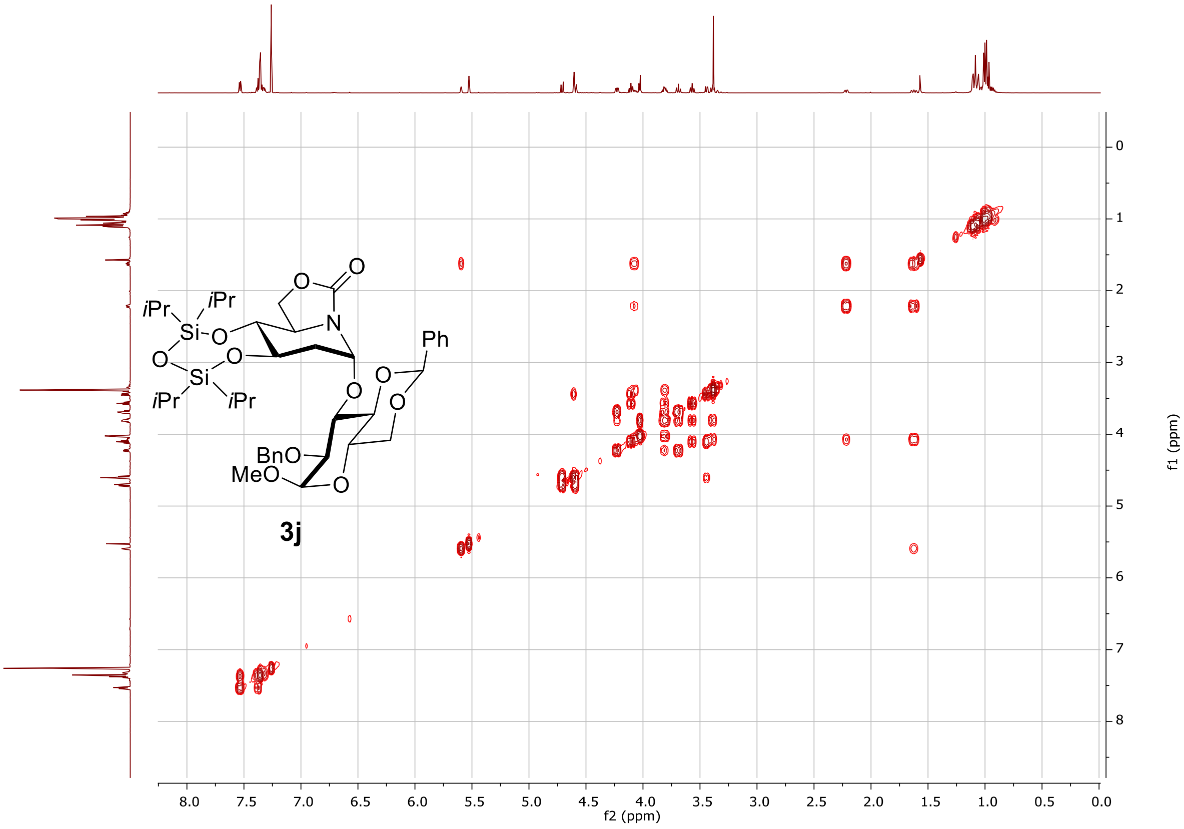

Supplementary Figure S250. COSY spectra for 3j

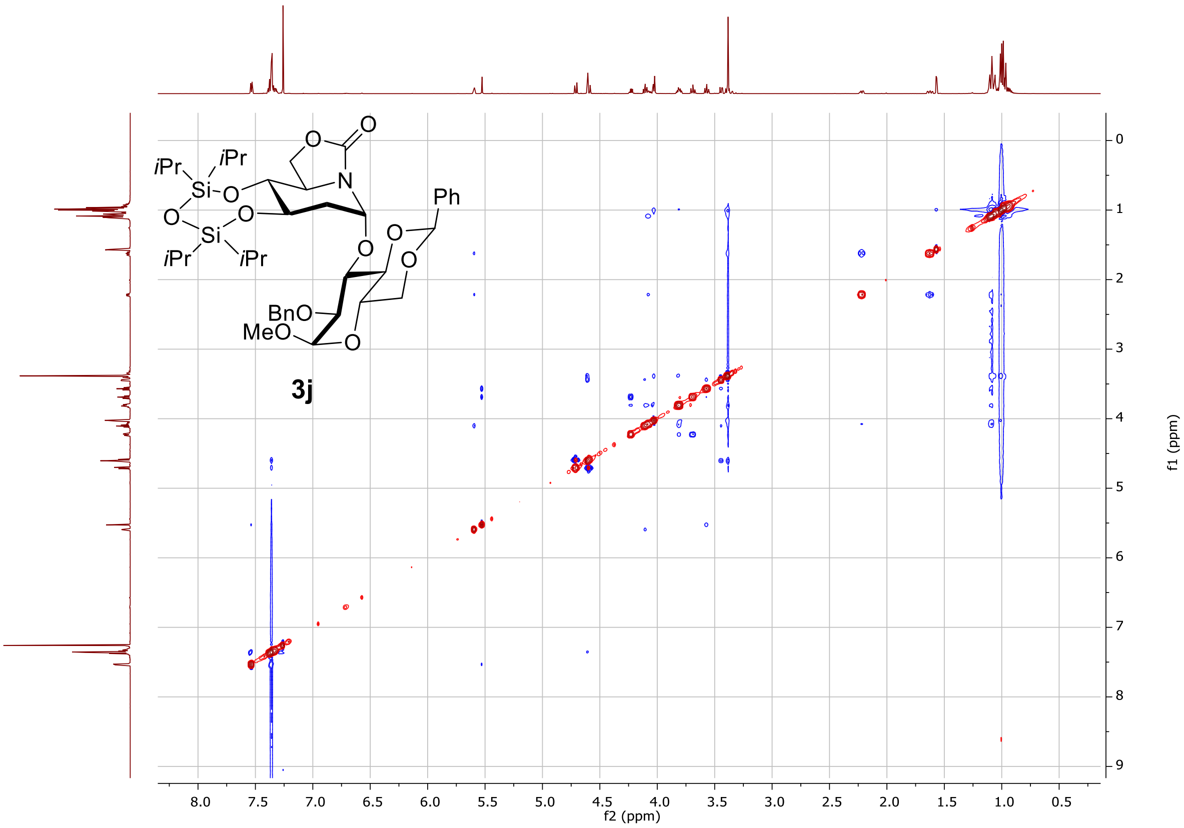

Supplementary Figure S251. NOESY spectra for 3j

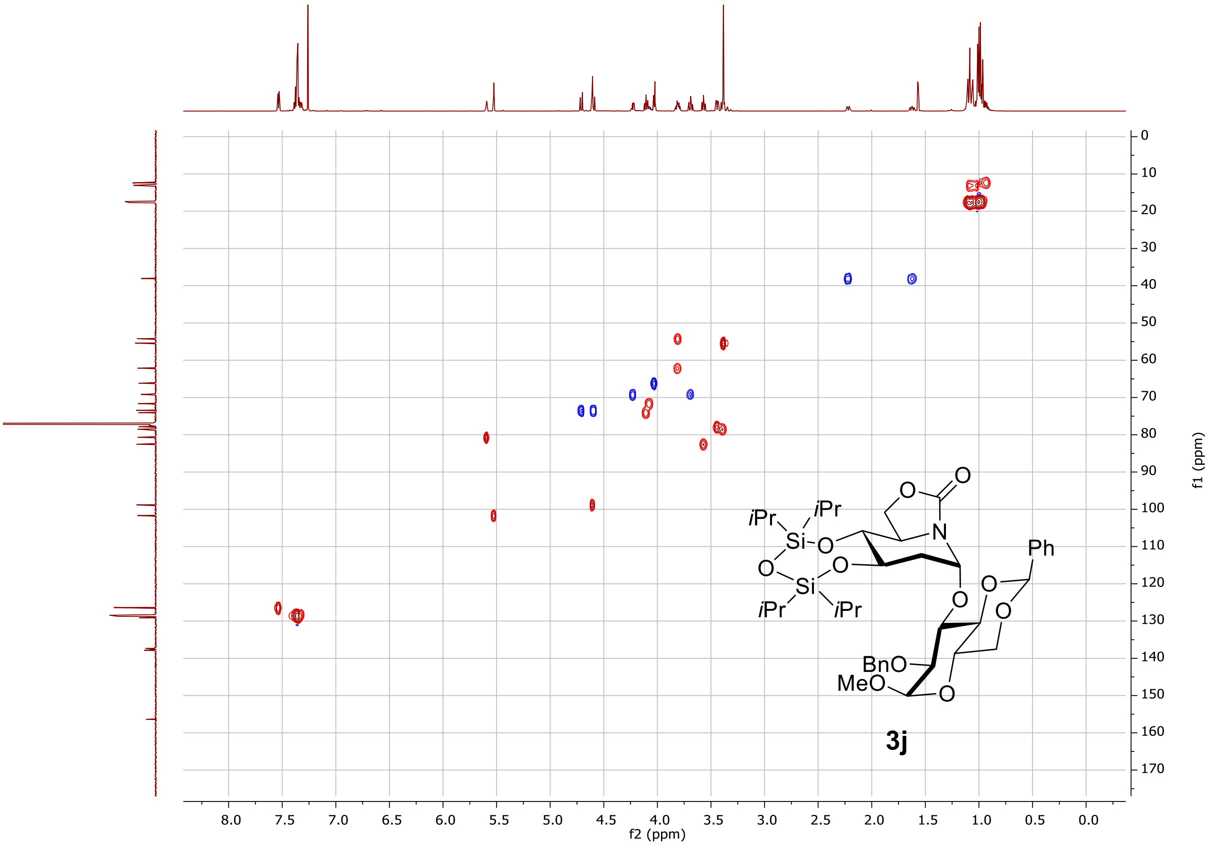

Supplementary Figure S252. HSQC spectra for 3j

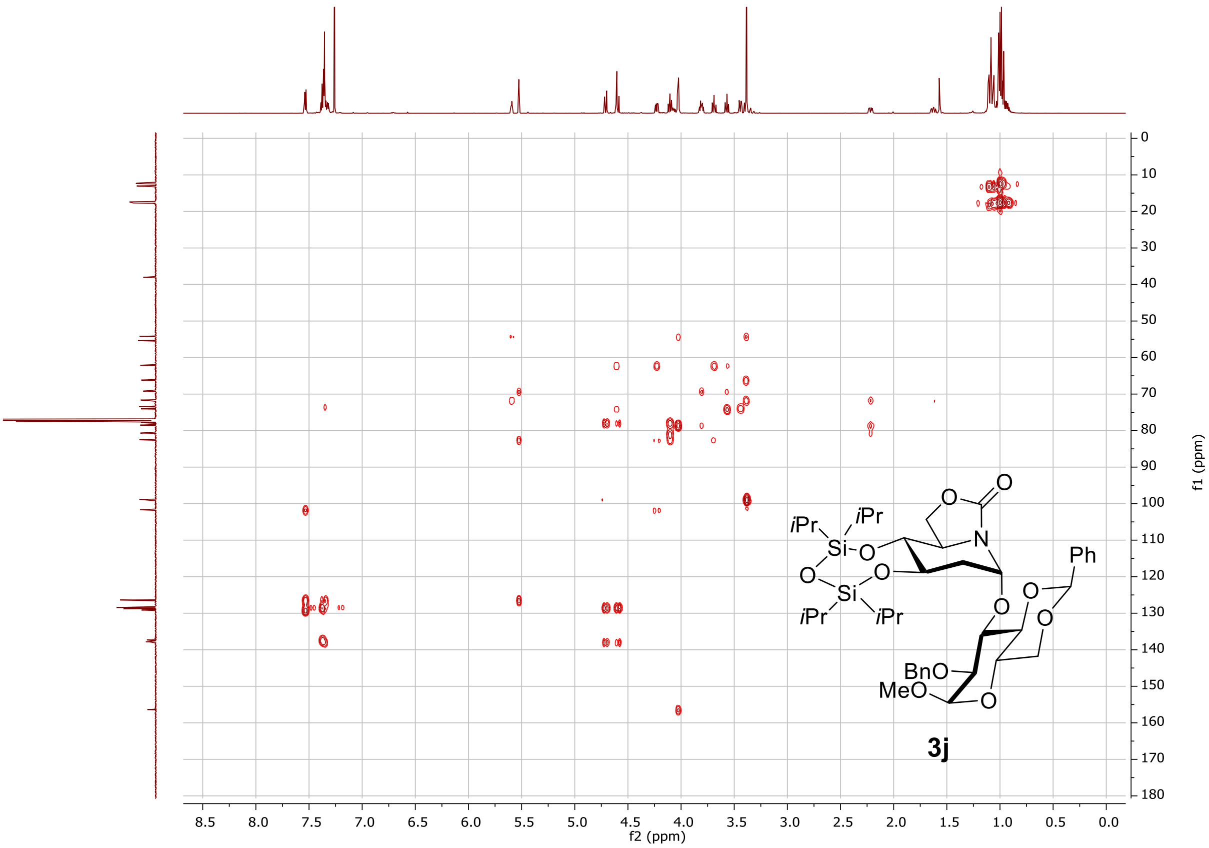

Supplementary Figure S253. HMBC spectra for 3j

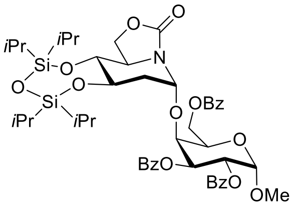

3k

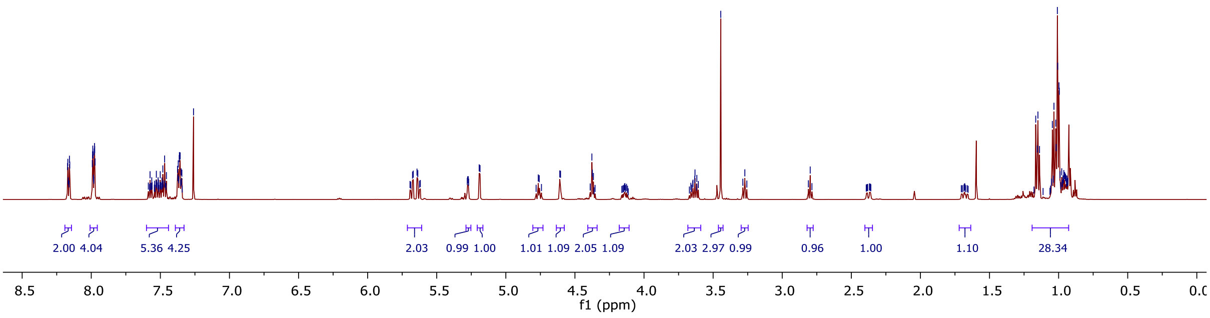

Supplementary Figure S254. ^{1}H NMR spectra for 3k

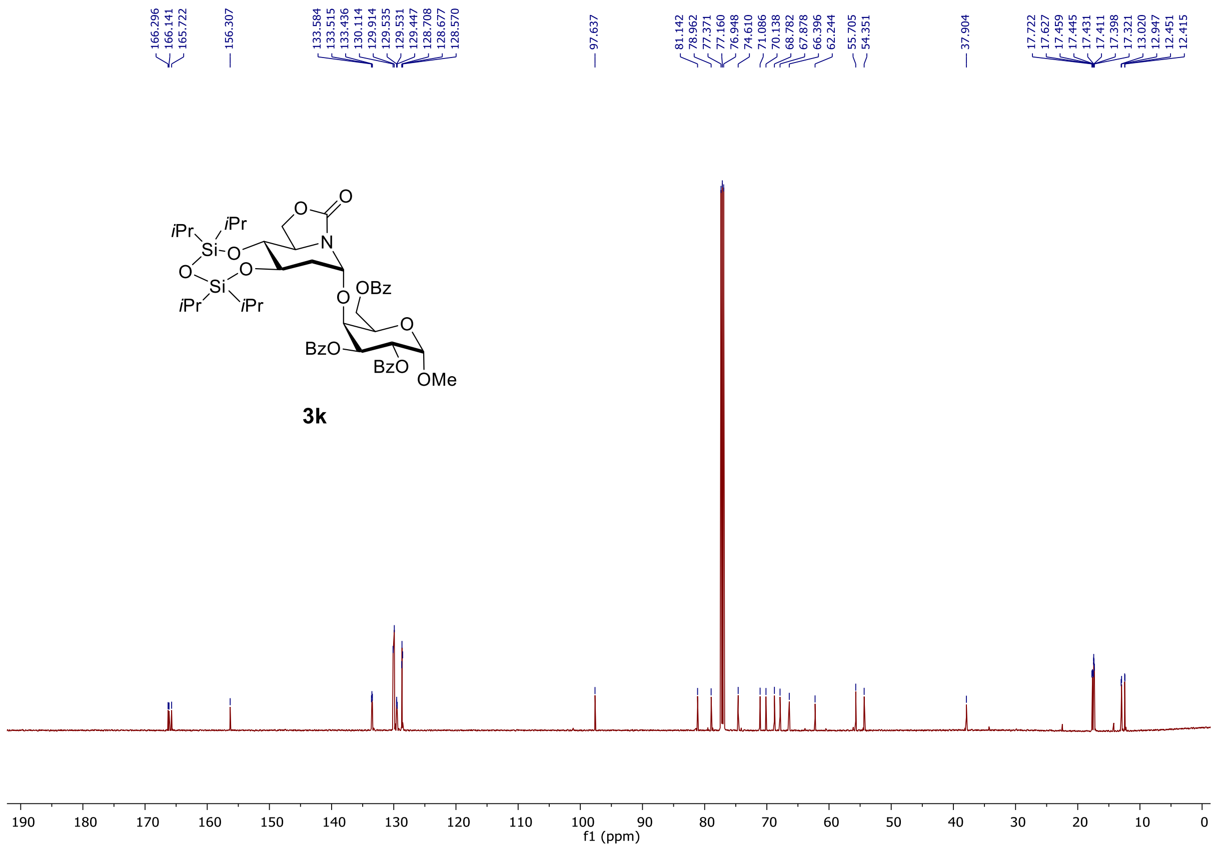

Supplementary Figure S255. ^{13}C NMR spectra for 3k

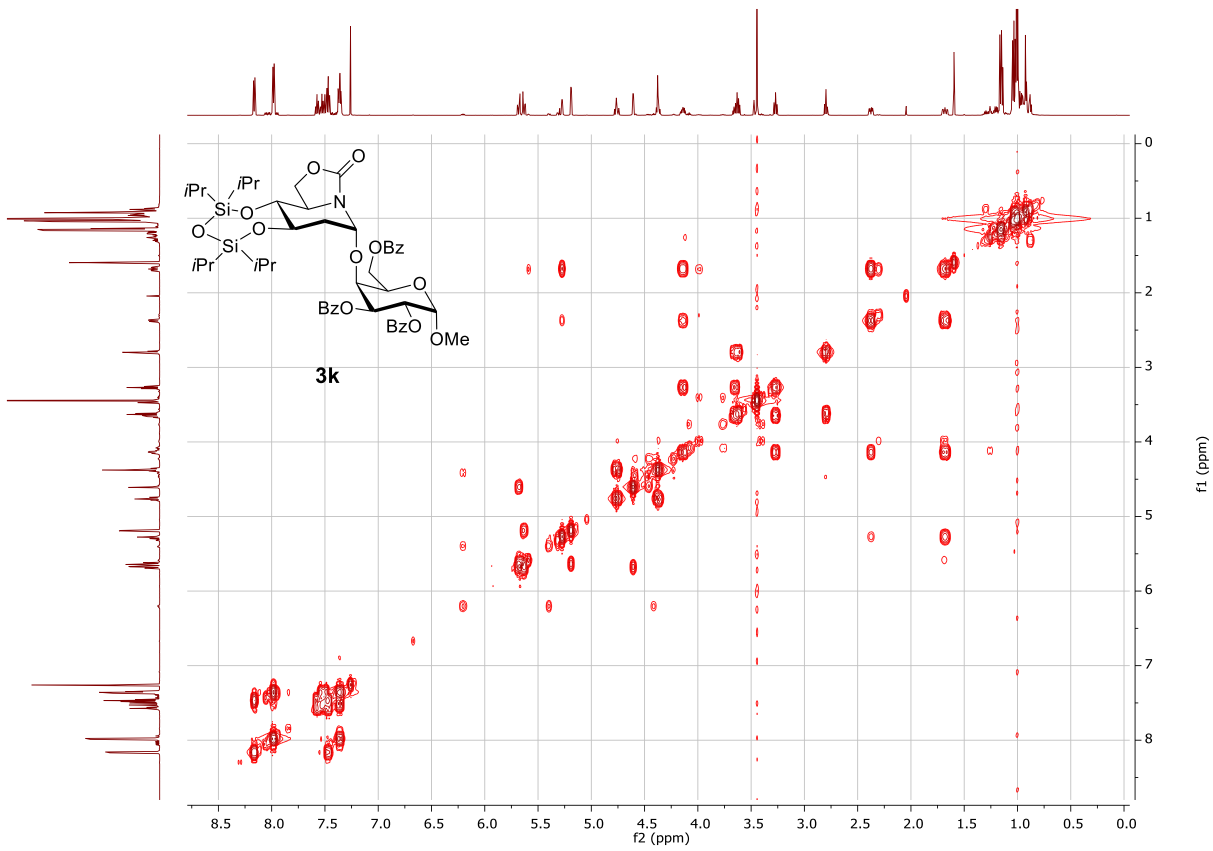

Supplementary Figure S256. COSY spectra for 3k

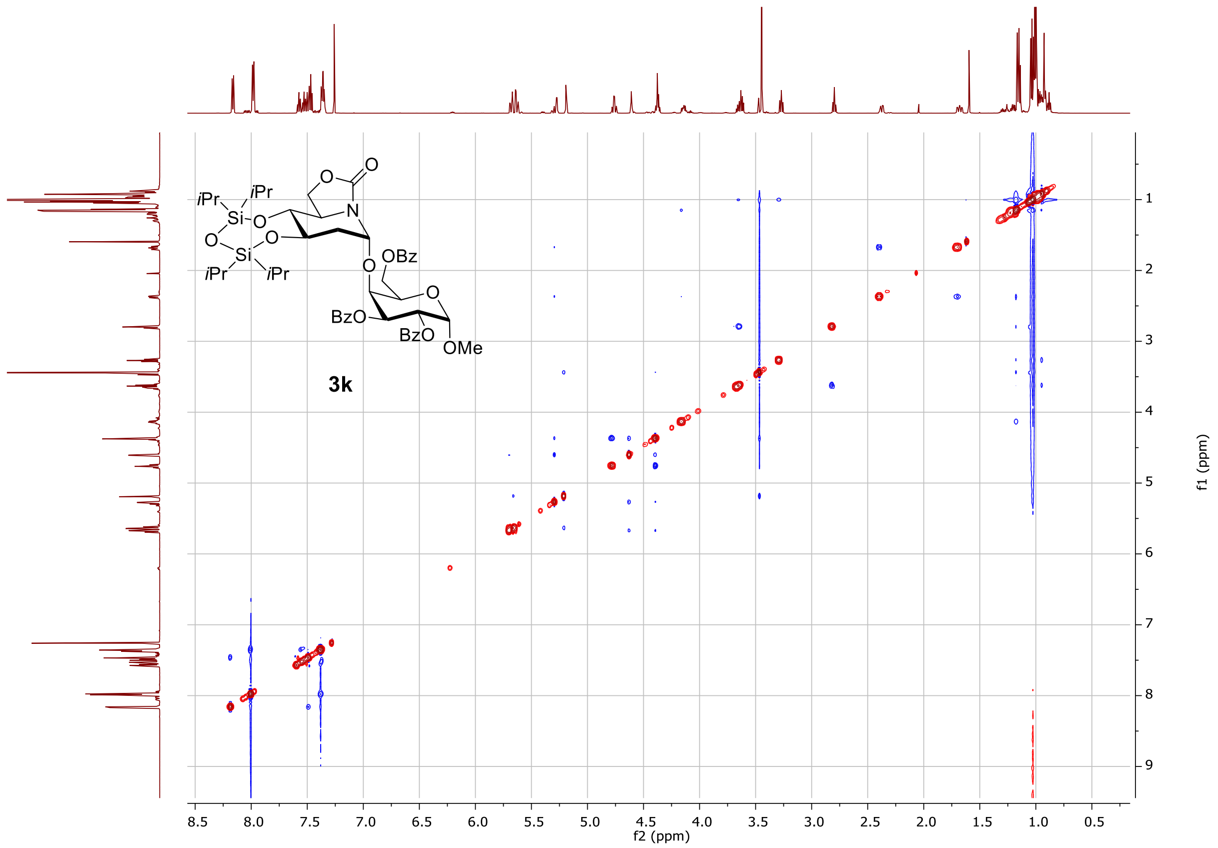

Supplementary Figure S257. NOESY spectra for 3k

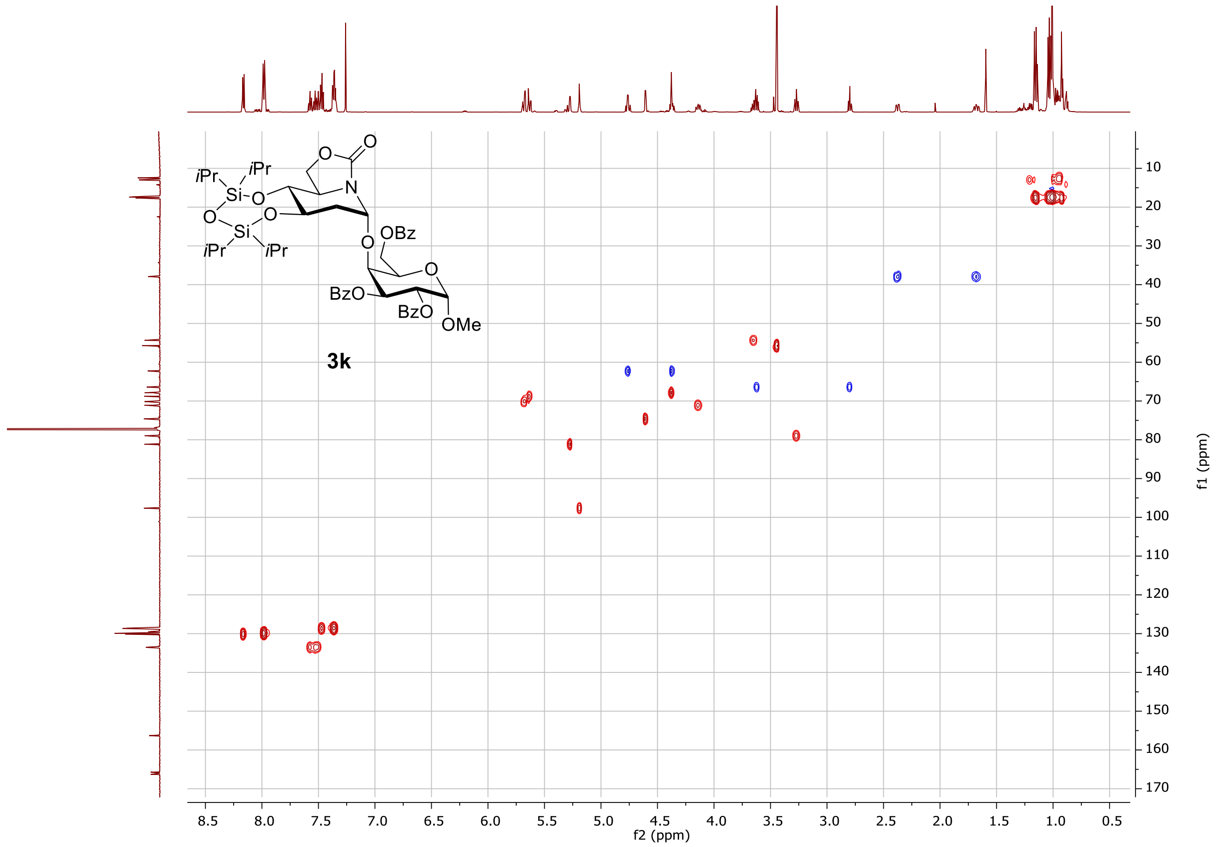

Supplementary Figure S258. HSQC spectra for 3k

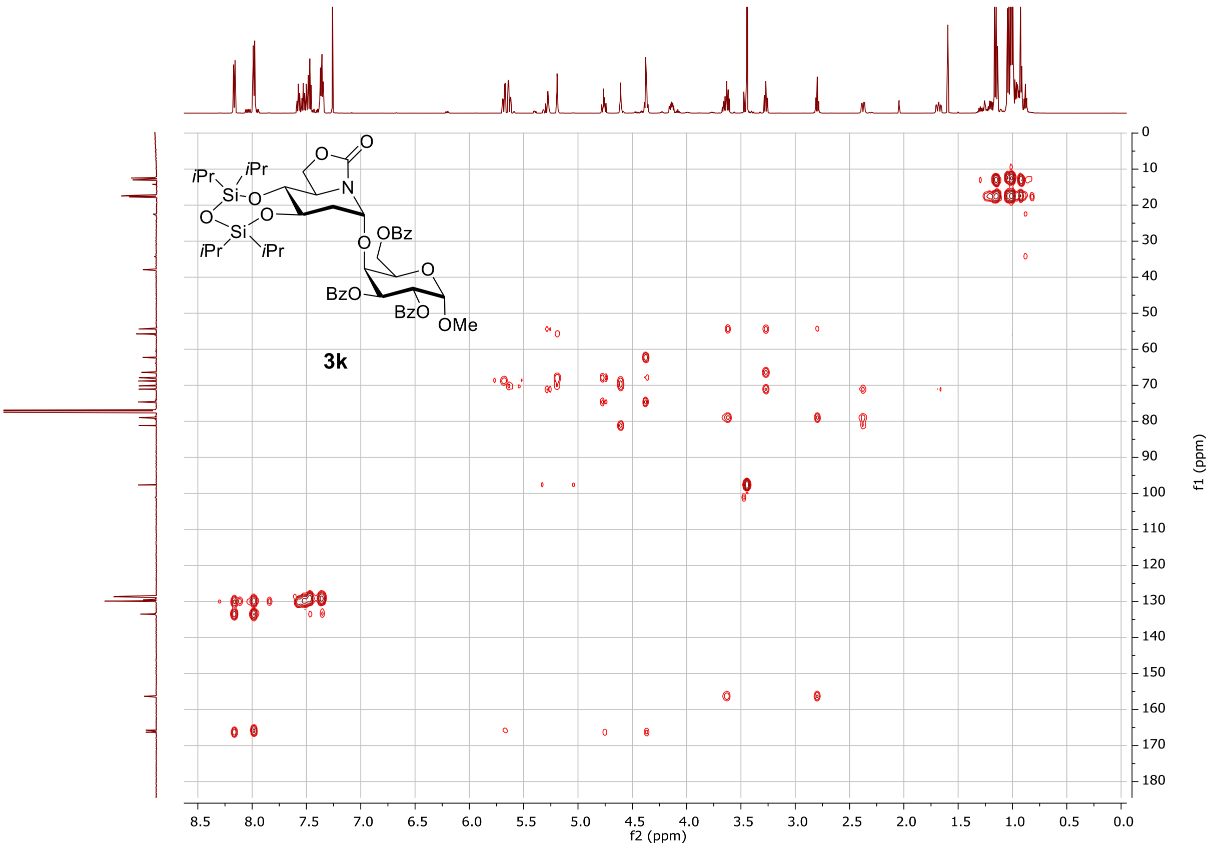

Supplementary Figure S259. HMBC spectra for 3k

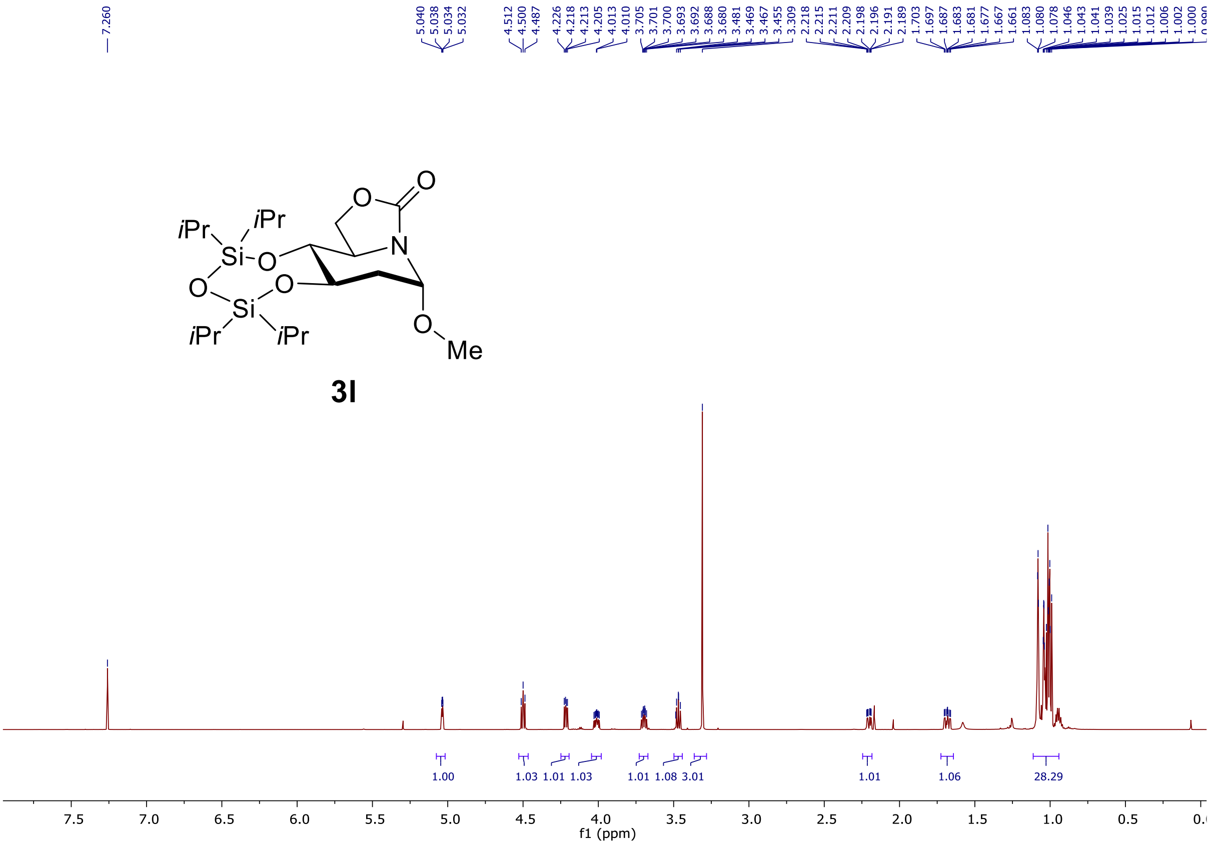

Supplementary Figure S260. ¹H NMR spectra for 3l

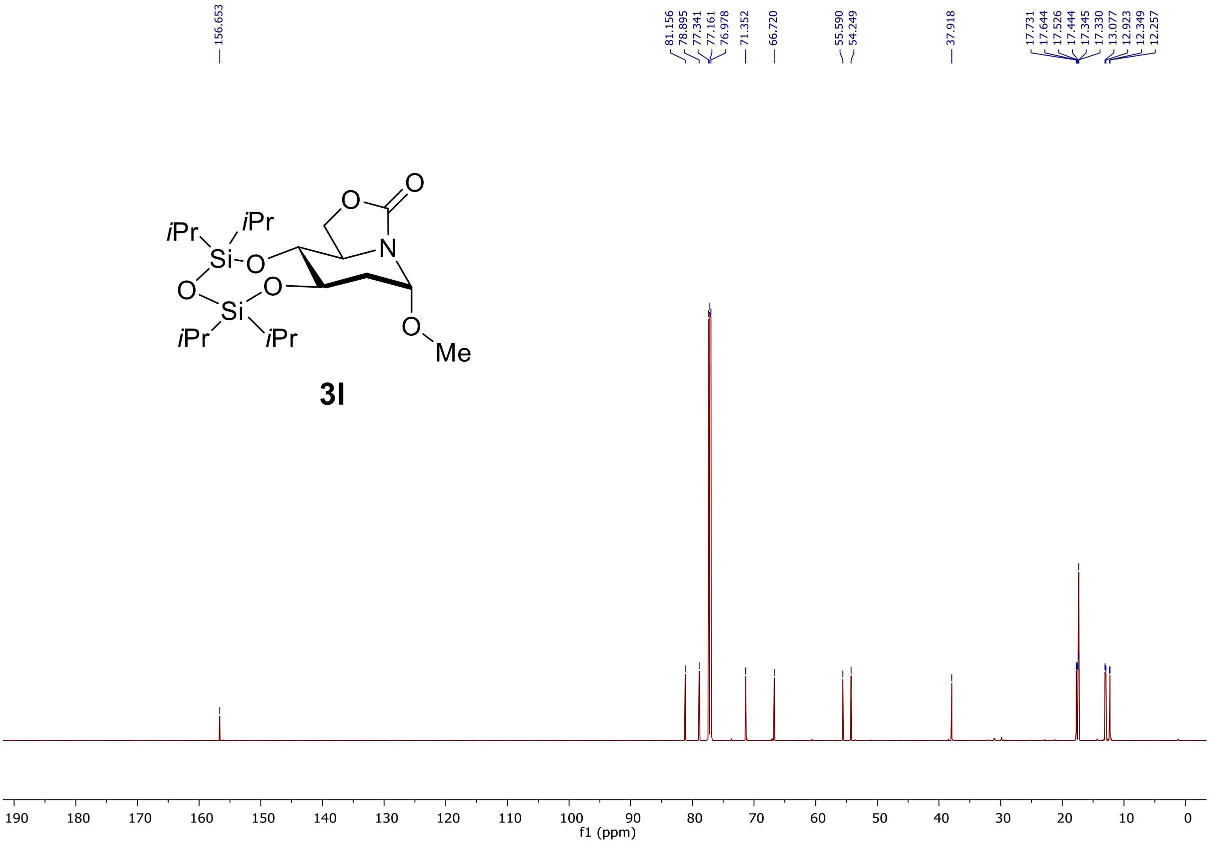

Supplementary Figure S261. ^{13}C NMR spectra for 3l

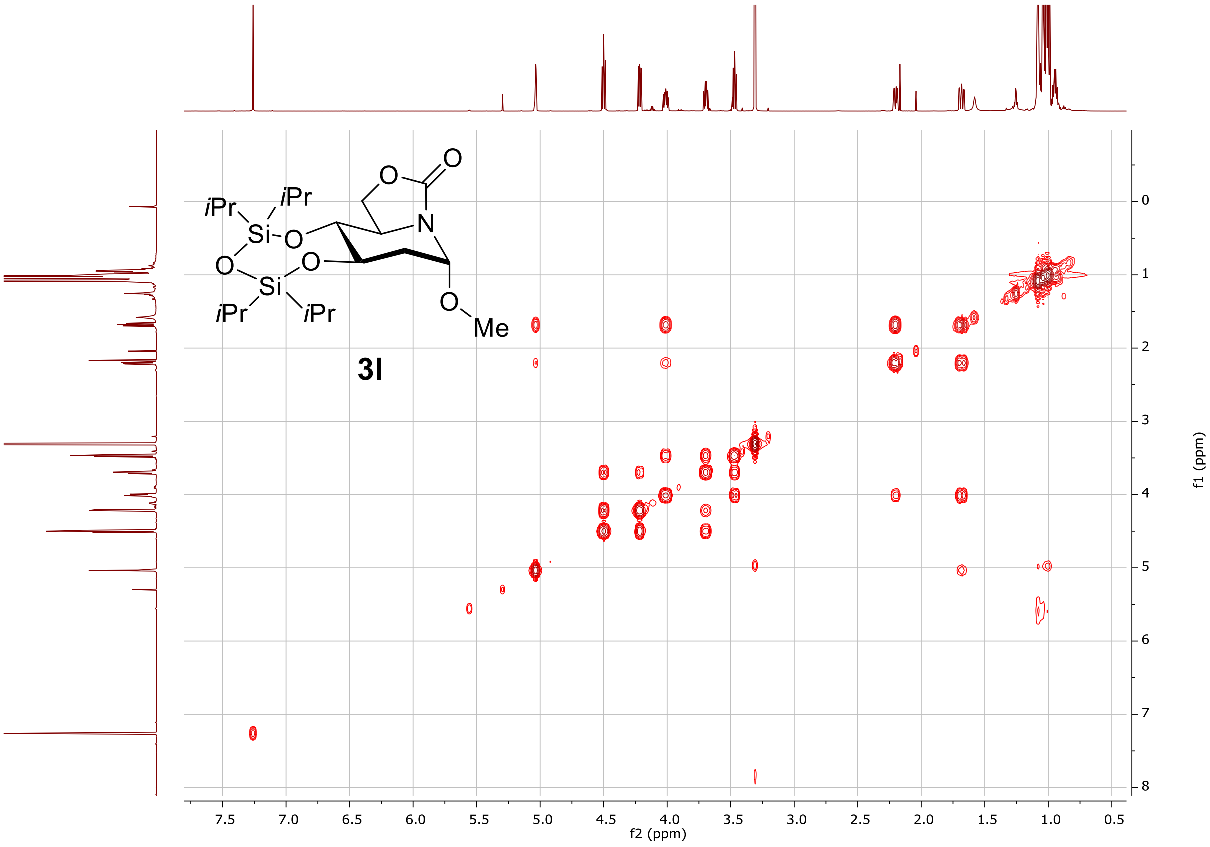

Supplementary Figure S262. COSY spectra for 3l

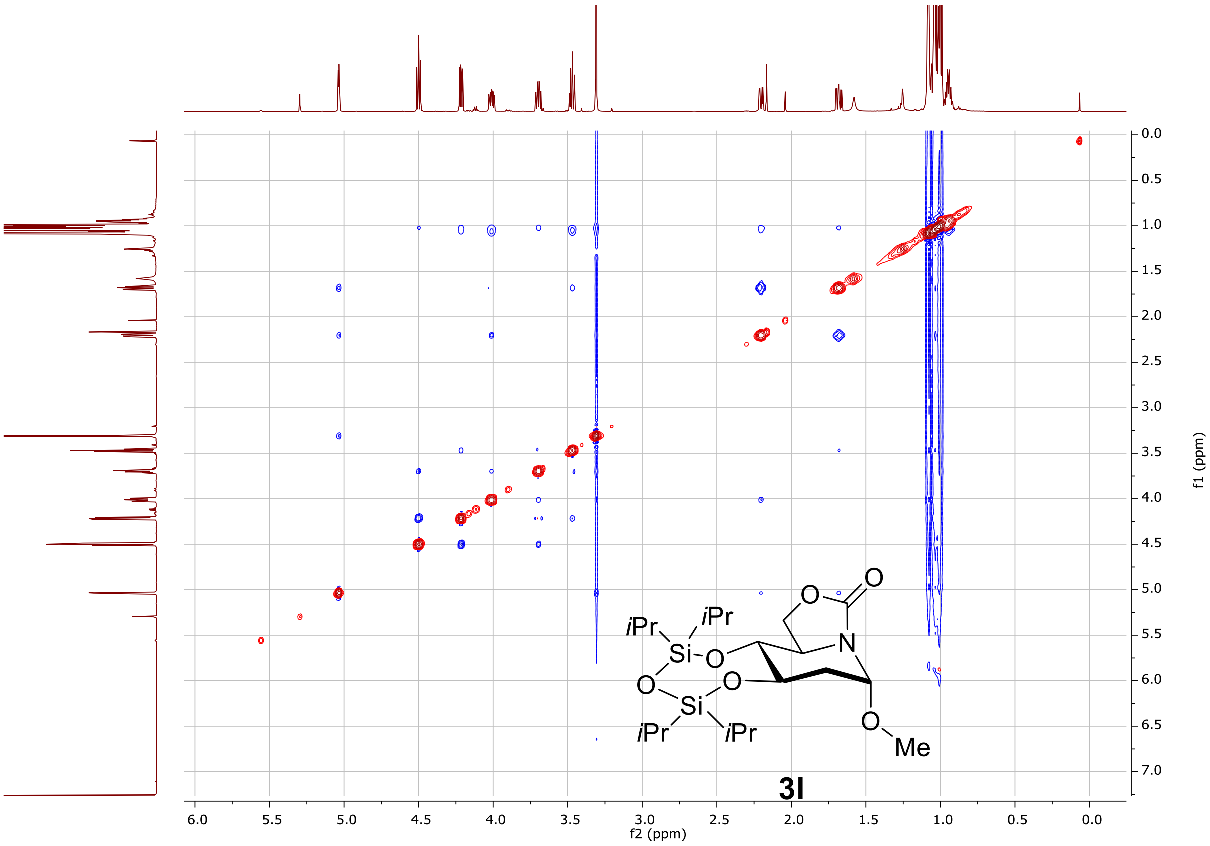

Supplementary Figure S263. NOESY spectra for 3l

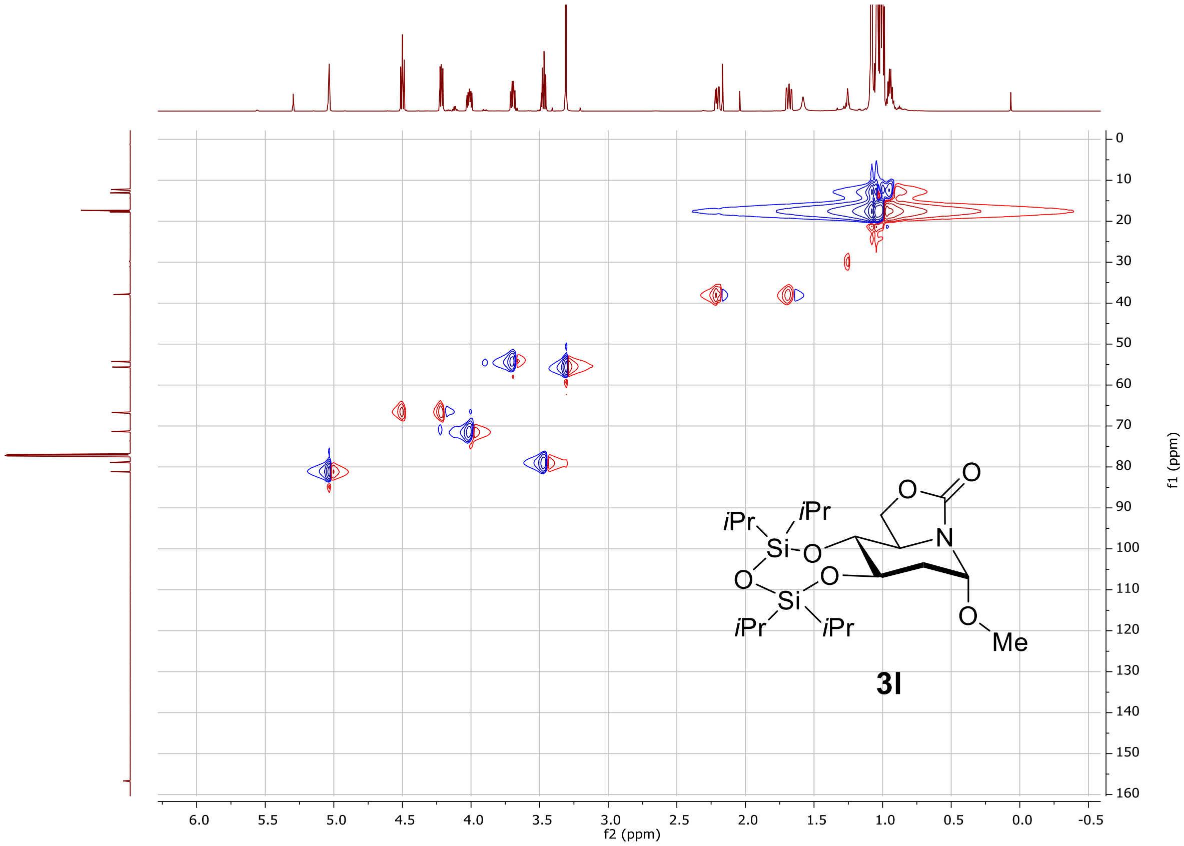

Supplementary Figure S264. HSQC spectra for 3l

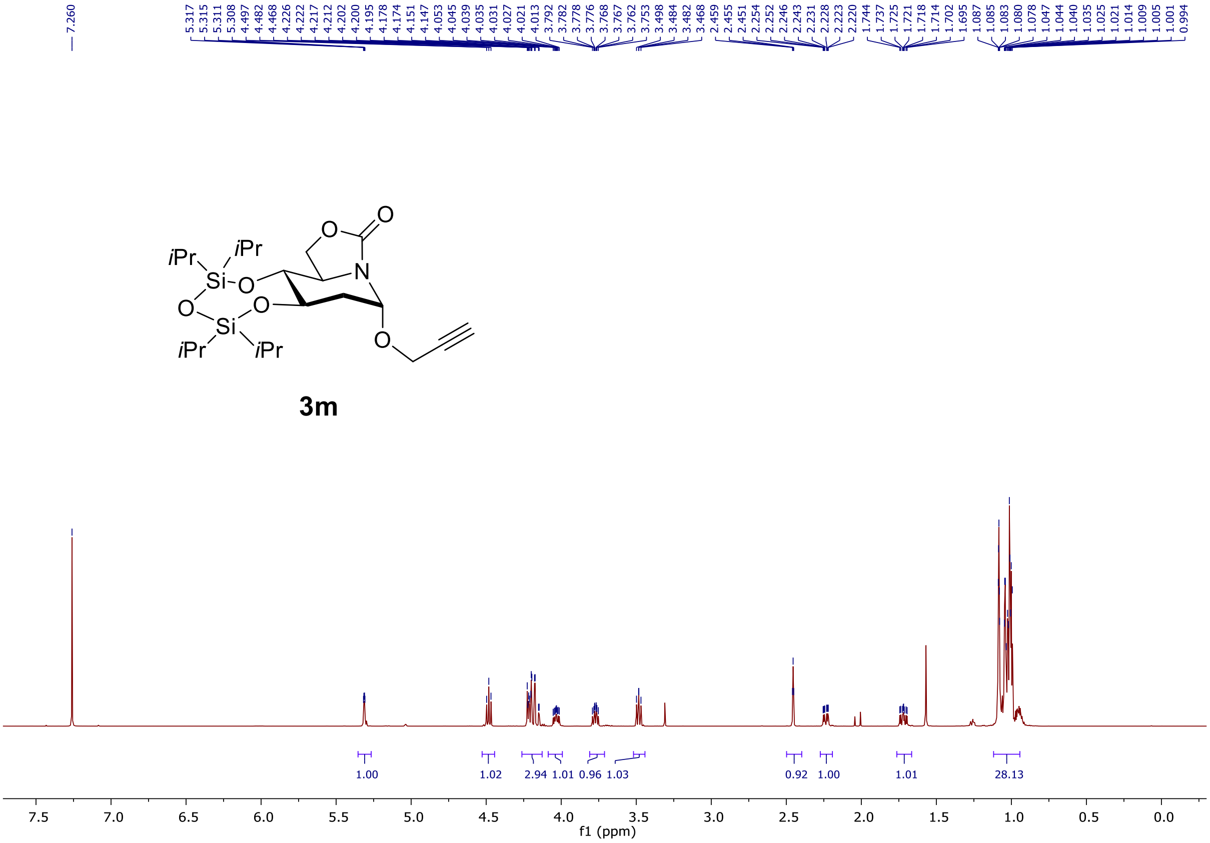

Supplementary Figure S265. ^{1}H NMR spectra for 3m

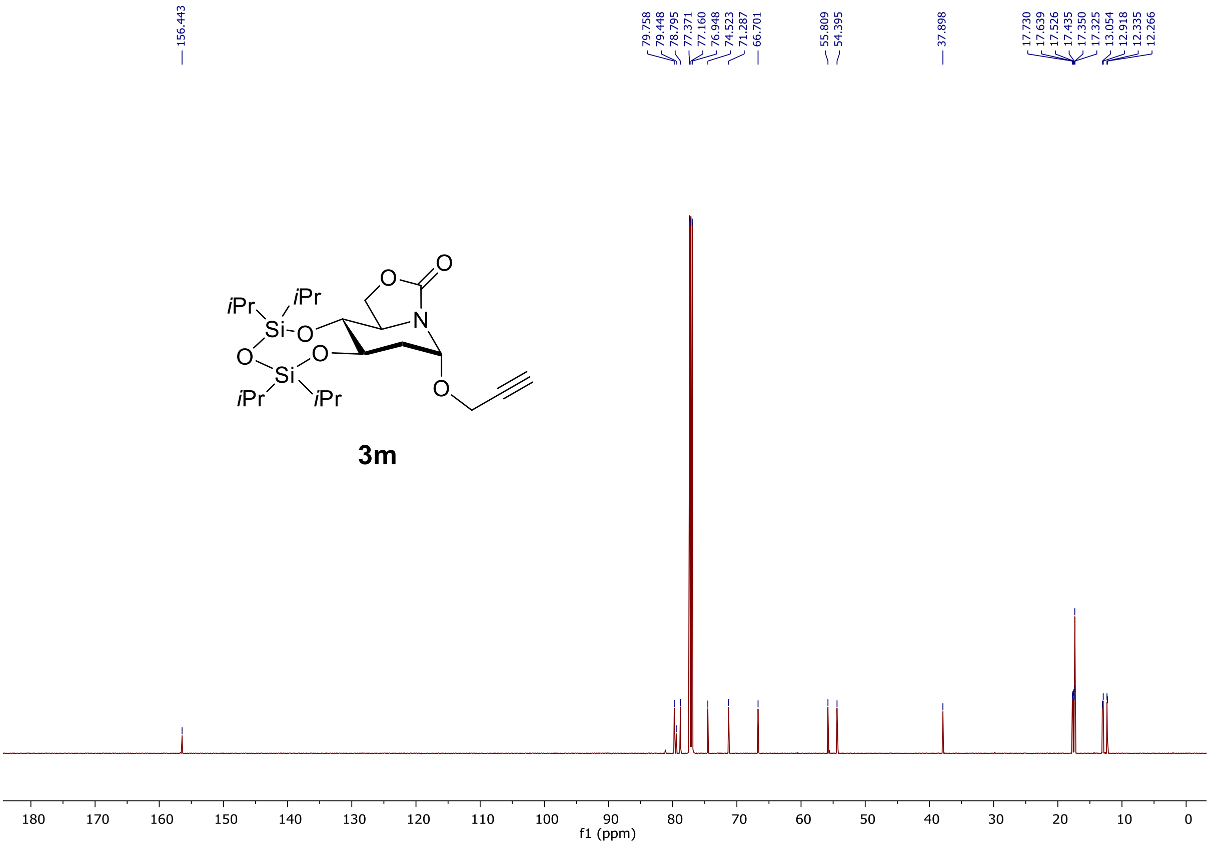

Supplementary Figure S266. ^{13}C NMR spectra for 3m

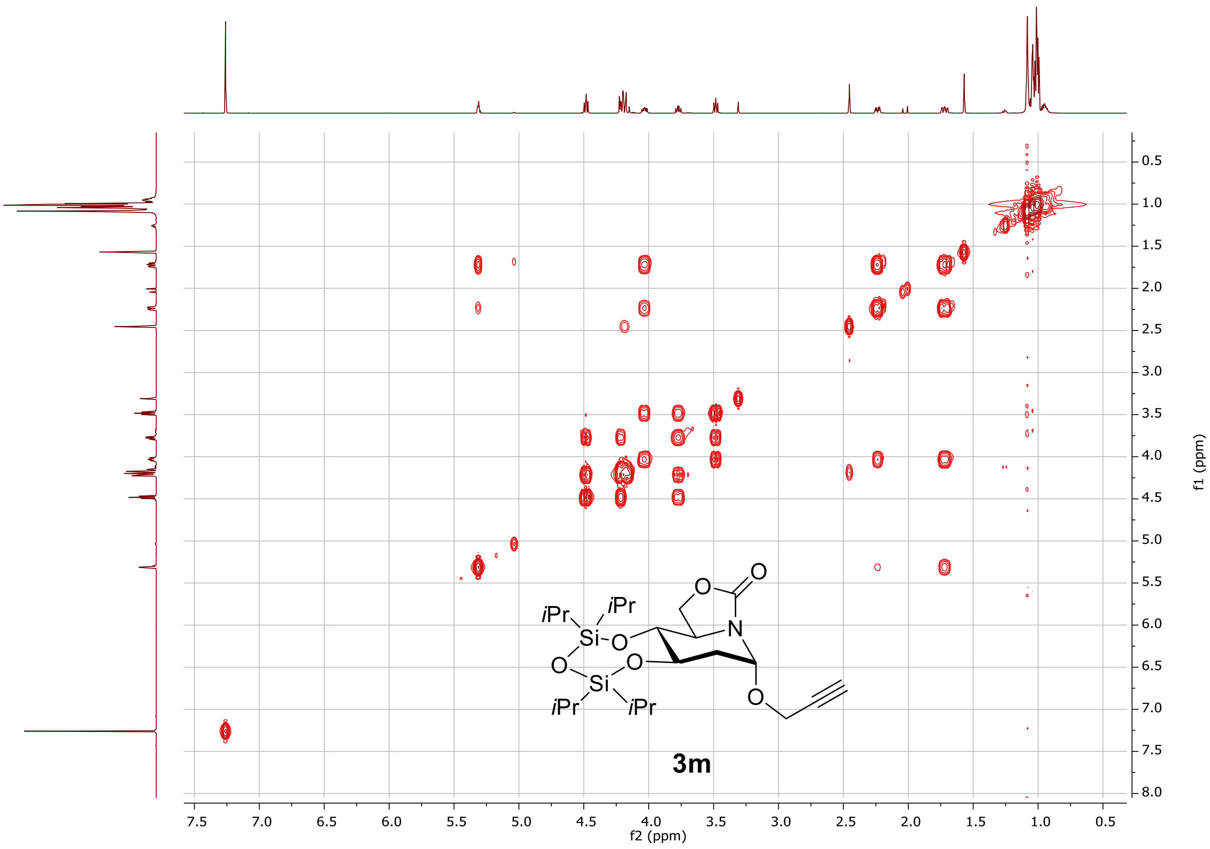

Supplementary Figure S267. COSY spectra for 3m

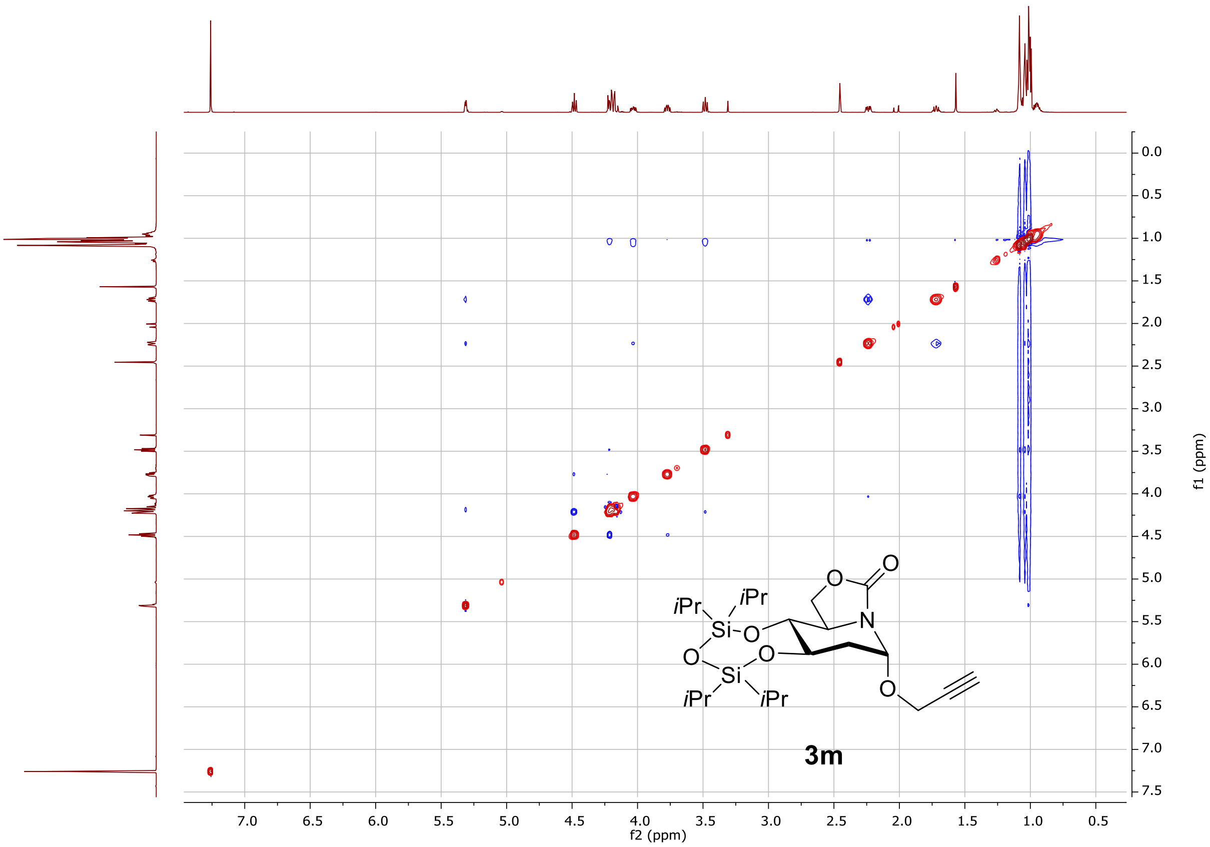

Supplementary Figure S268. NOESY spectra for 3m

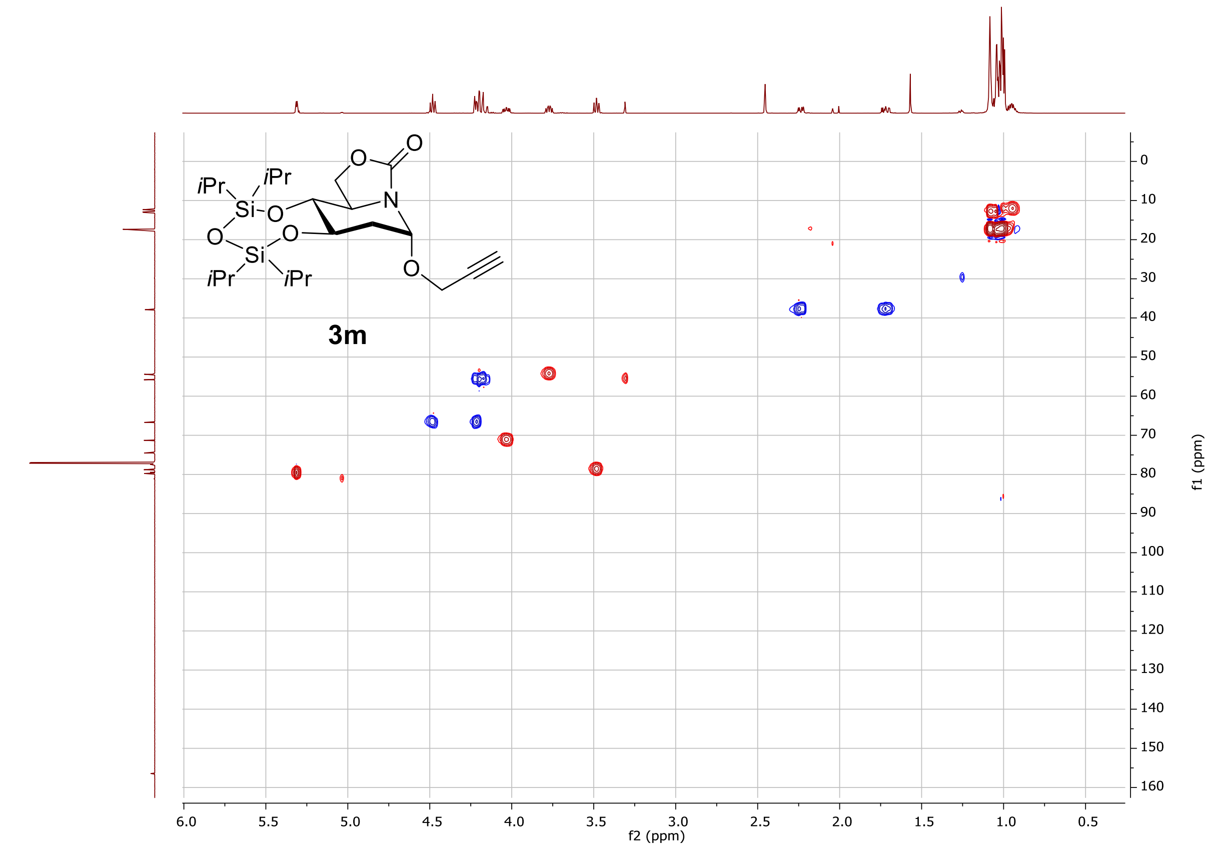

Supplementary Figure S269. HSQC spectra for 3m

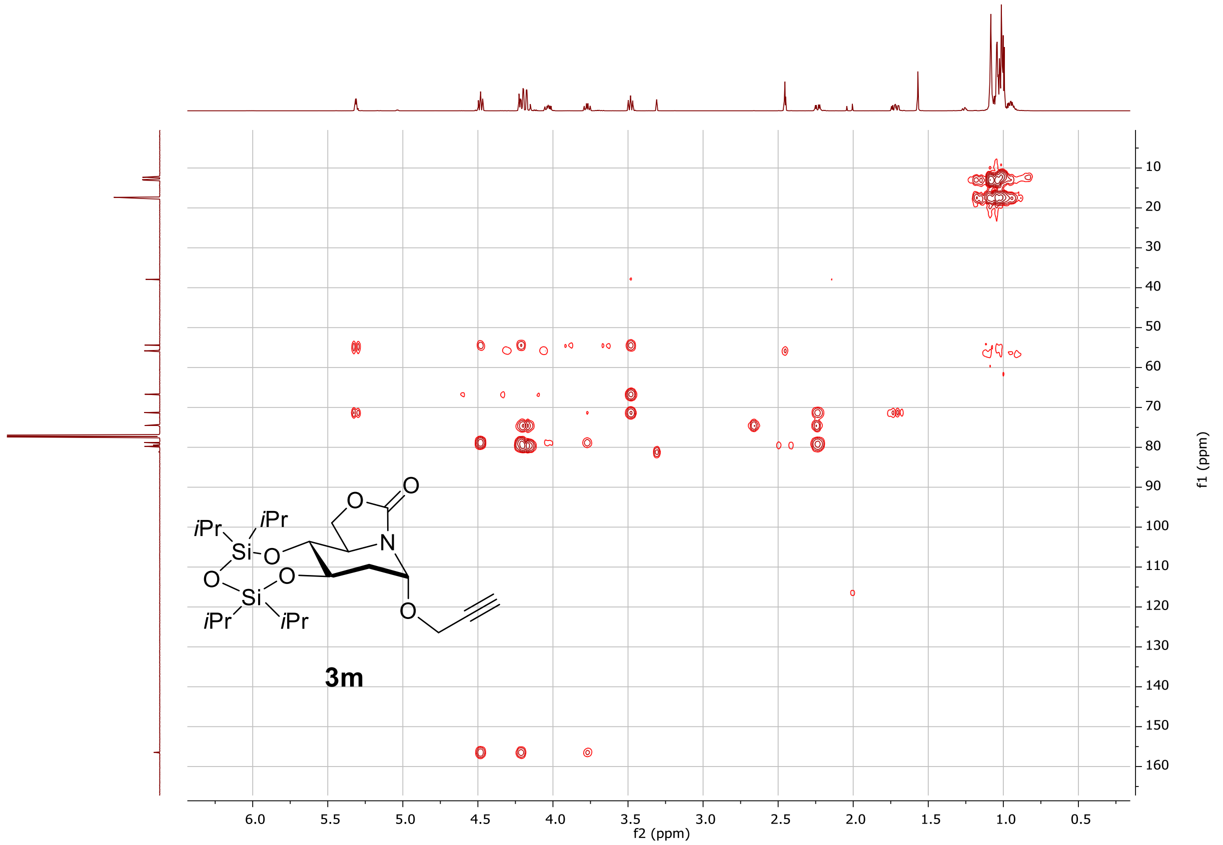

Supplementary Figure S270. HMBC spectra for 3m

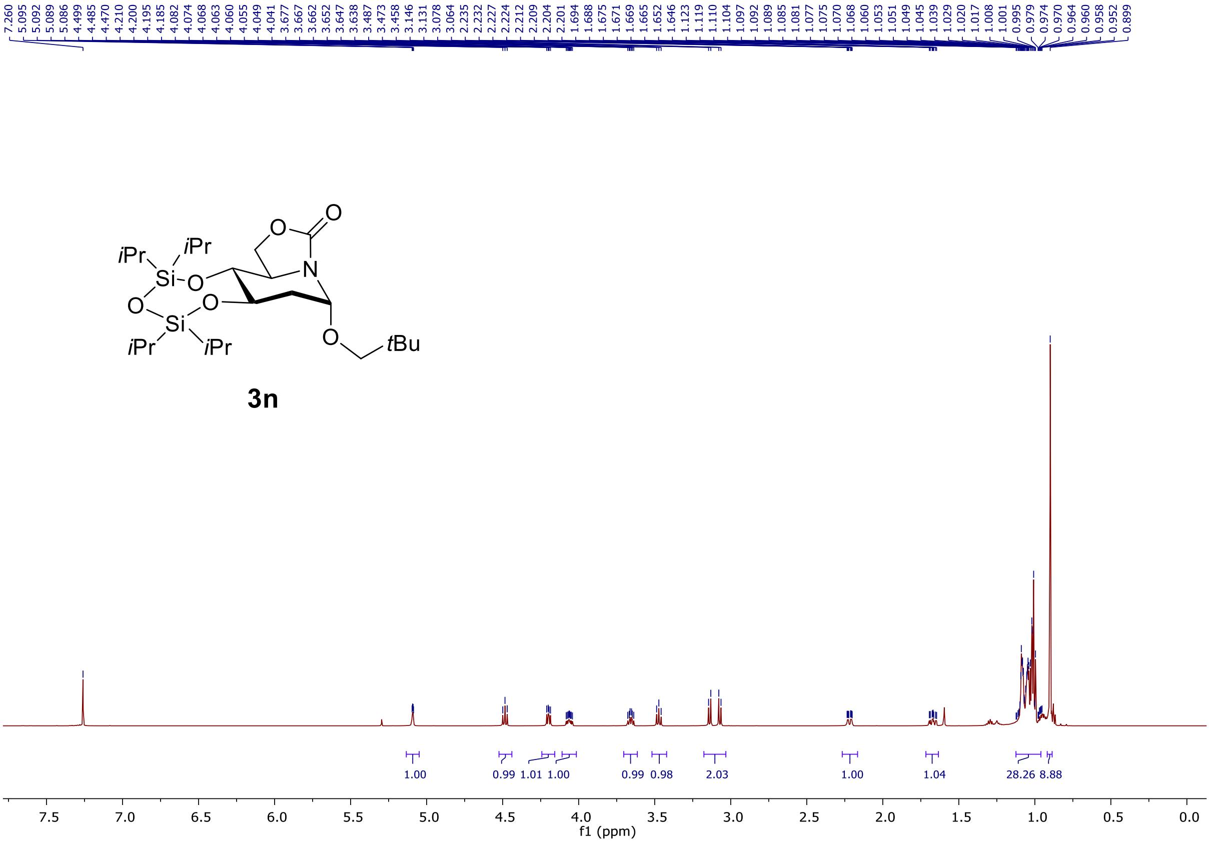

Supplementary Figure S271. ^{1}H NMR spectra for 3n

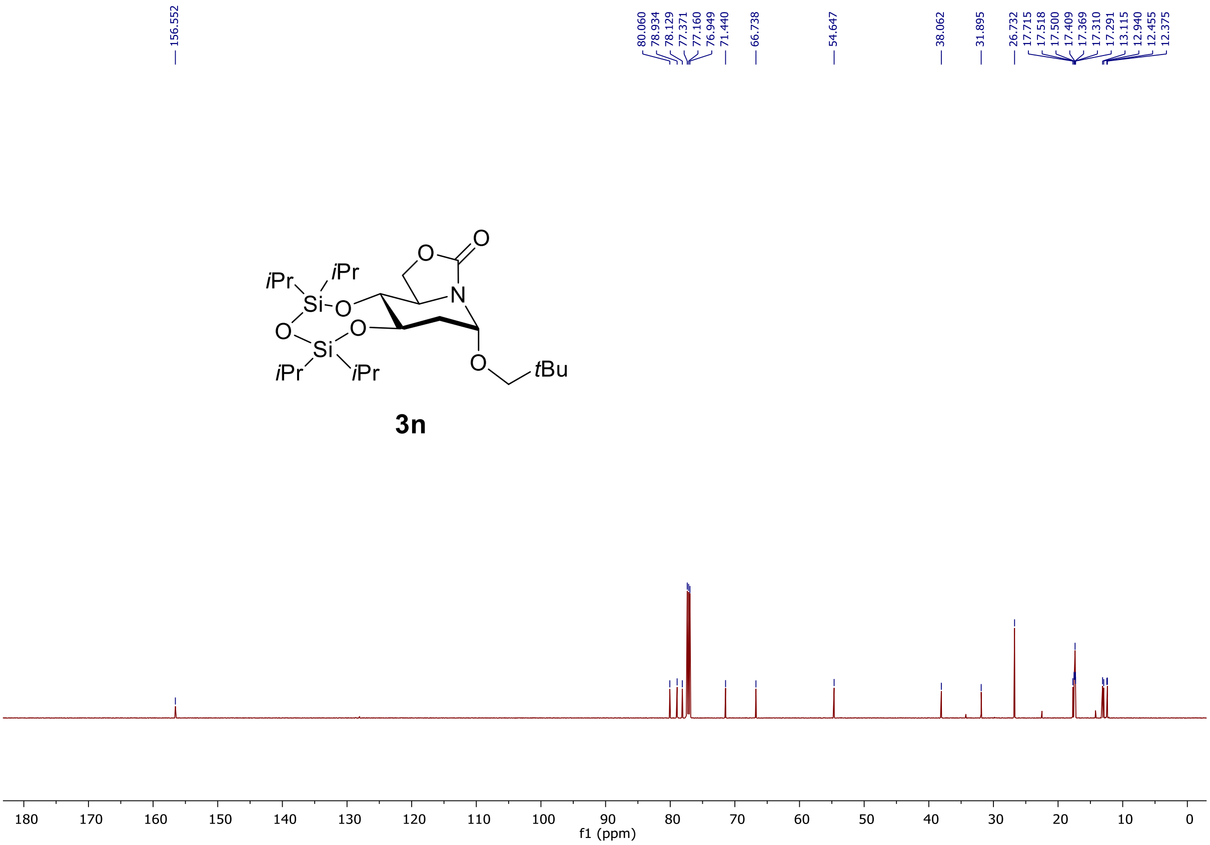

Supplementary Figure S272. ^{13}C NMR spectra for 3n

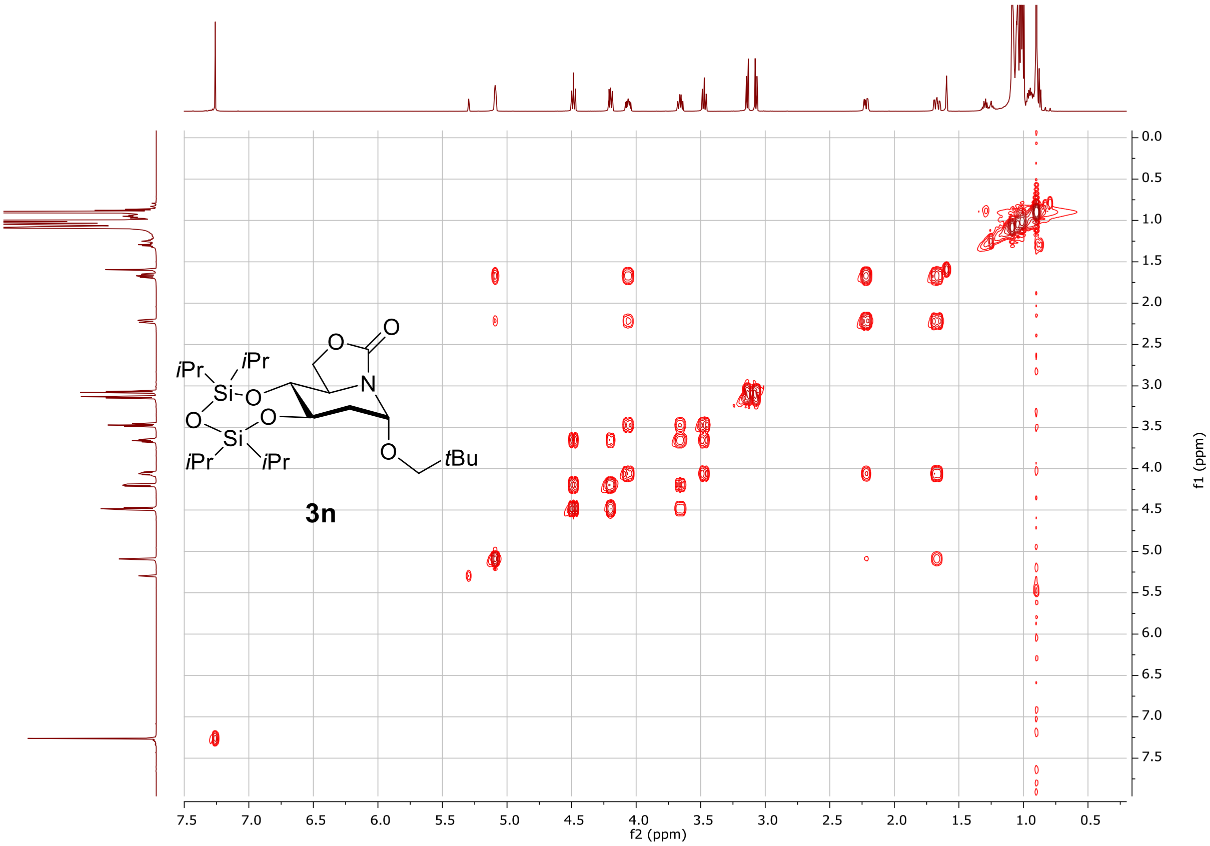

Supplementary Figure S273. COSY spectra for 3n

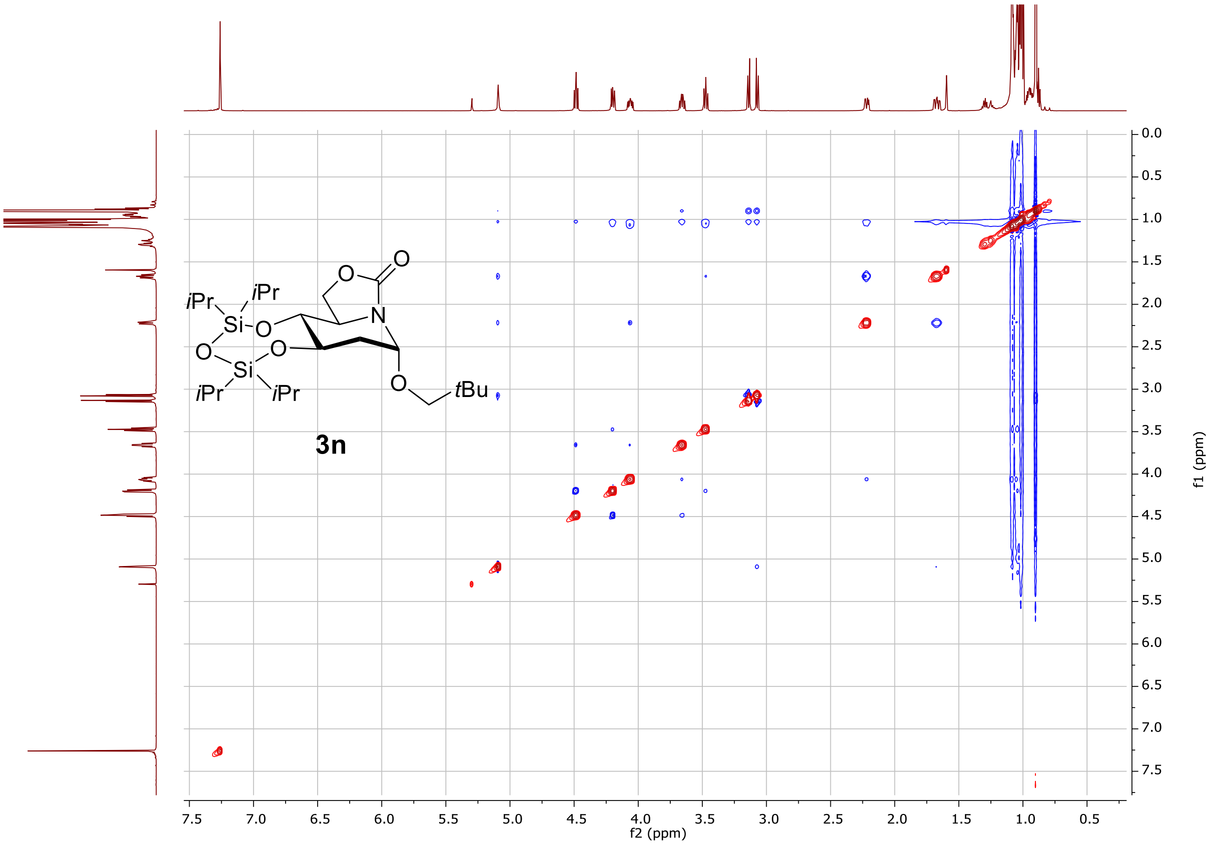

Supplementary Figure S274. NOESY spectra for 3n

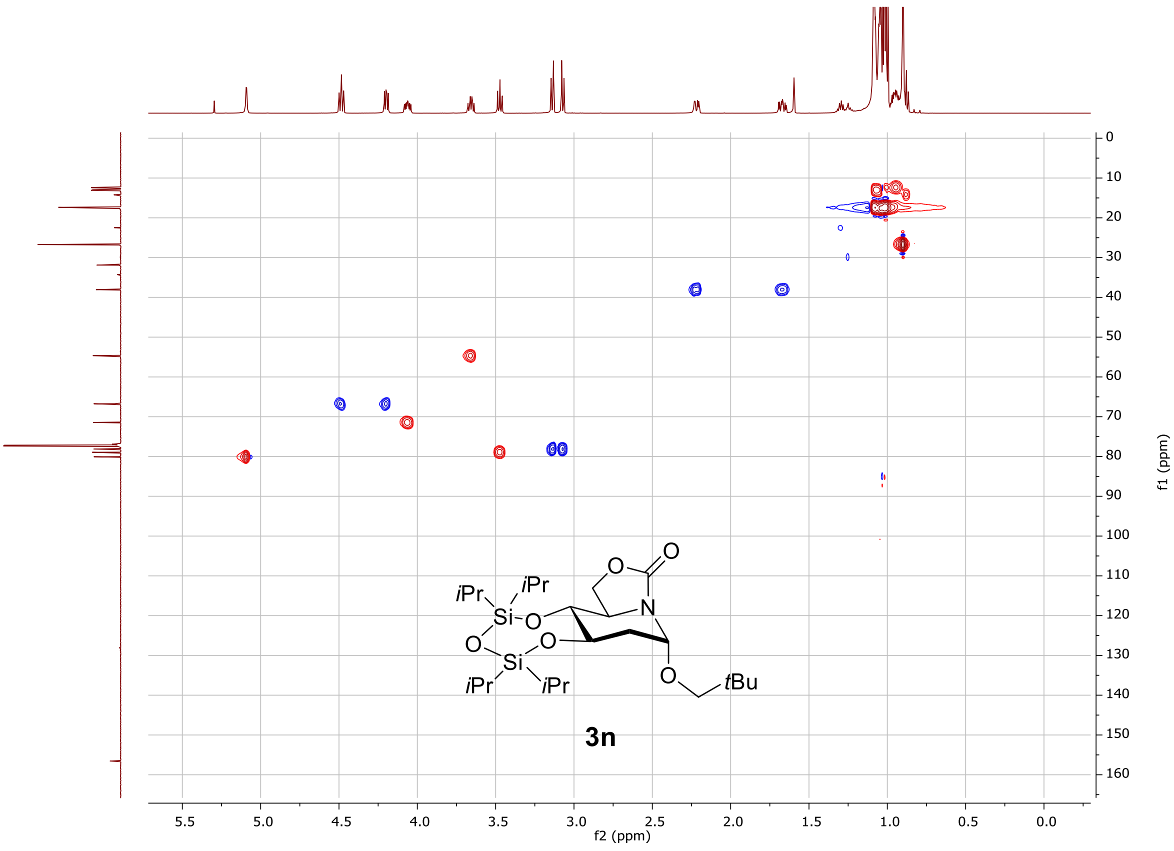

Supplementary Figure S275. HSQC spectra for 3n

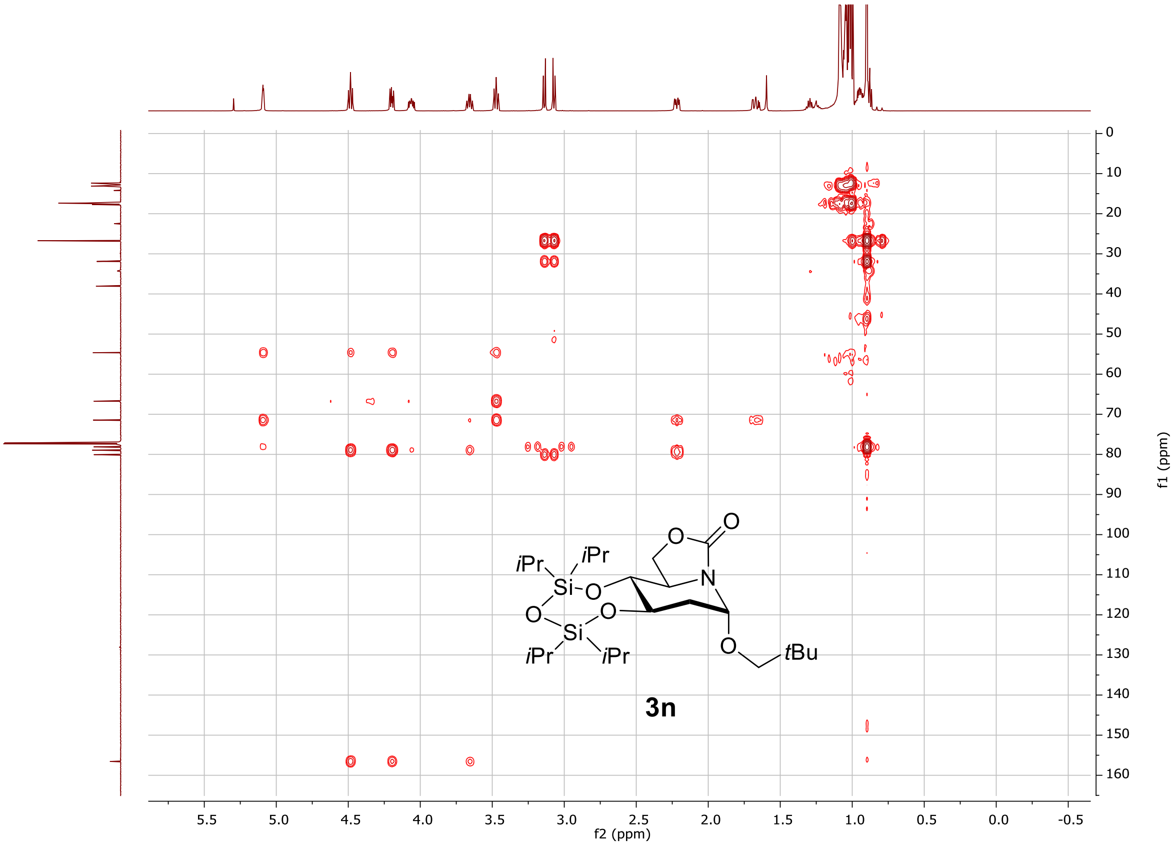

Supplementary Figure S276. HMBC spectra for 3n

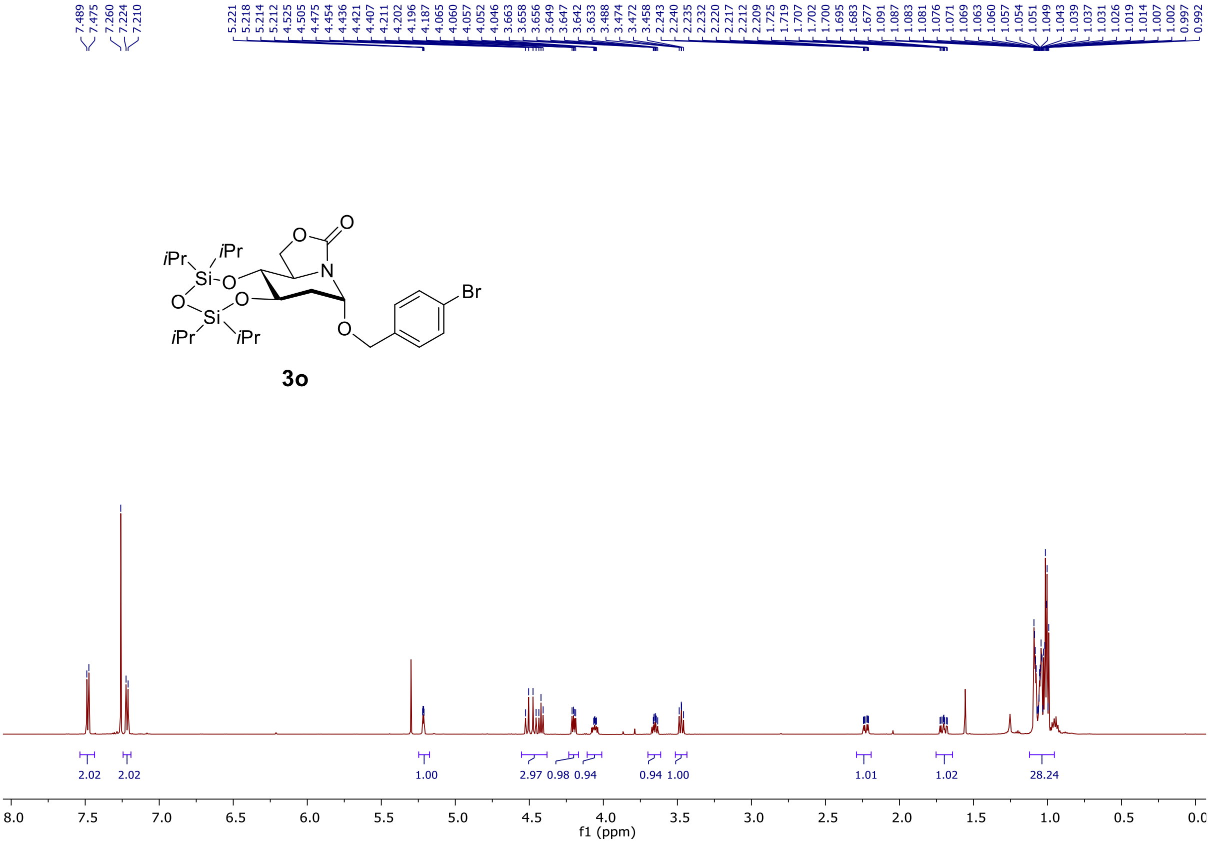

Supplementary Figure S277. ^{1}H NMR spectra for 3o

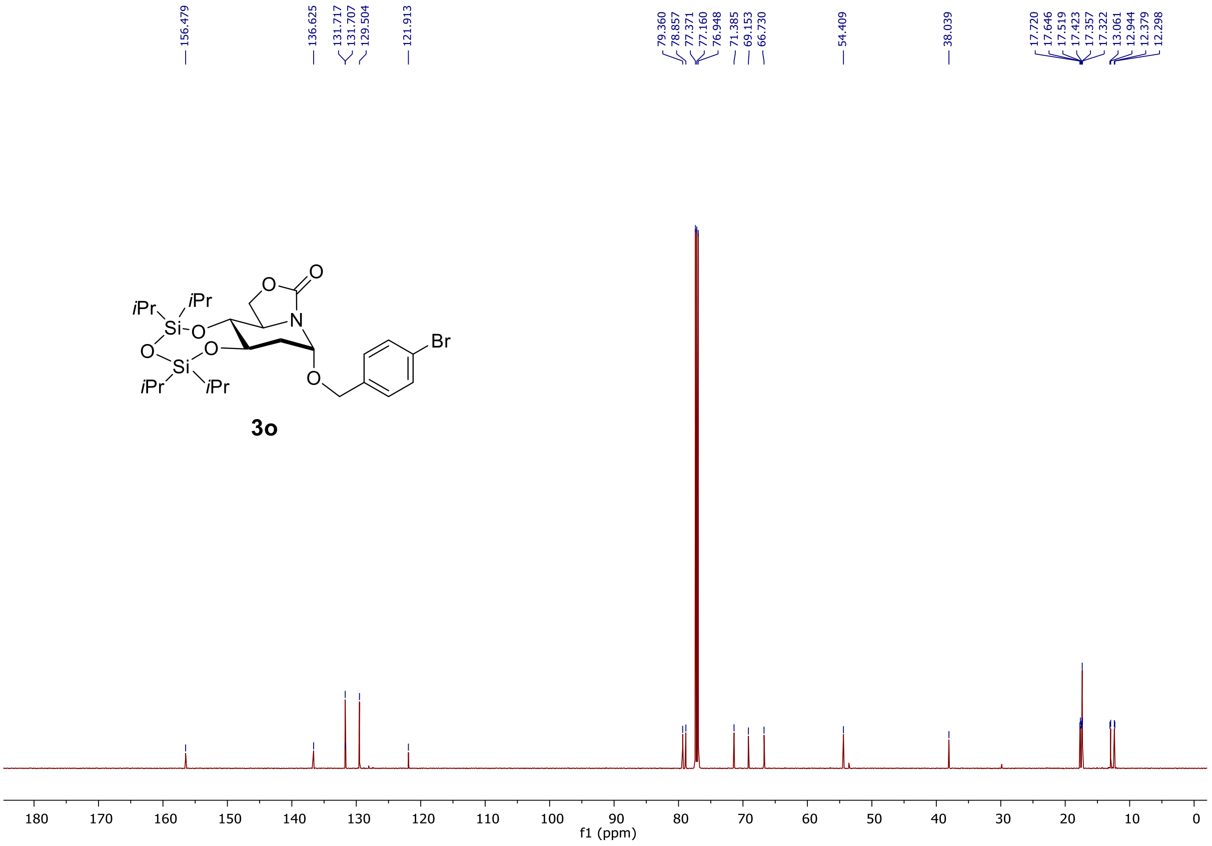

Supplementary Figure S278. ^{13}C NMR spectra for 3o

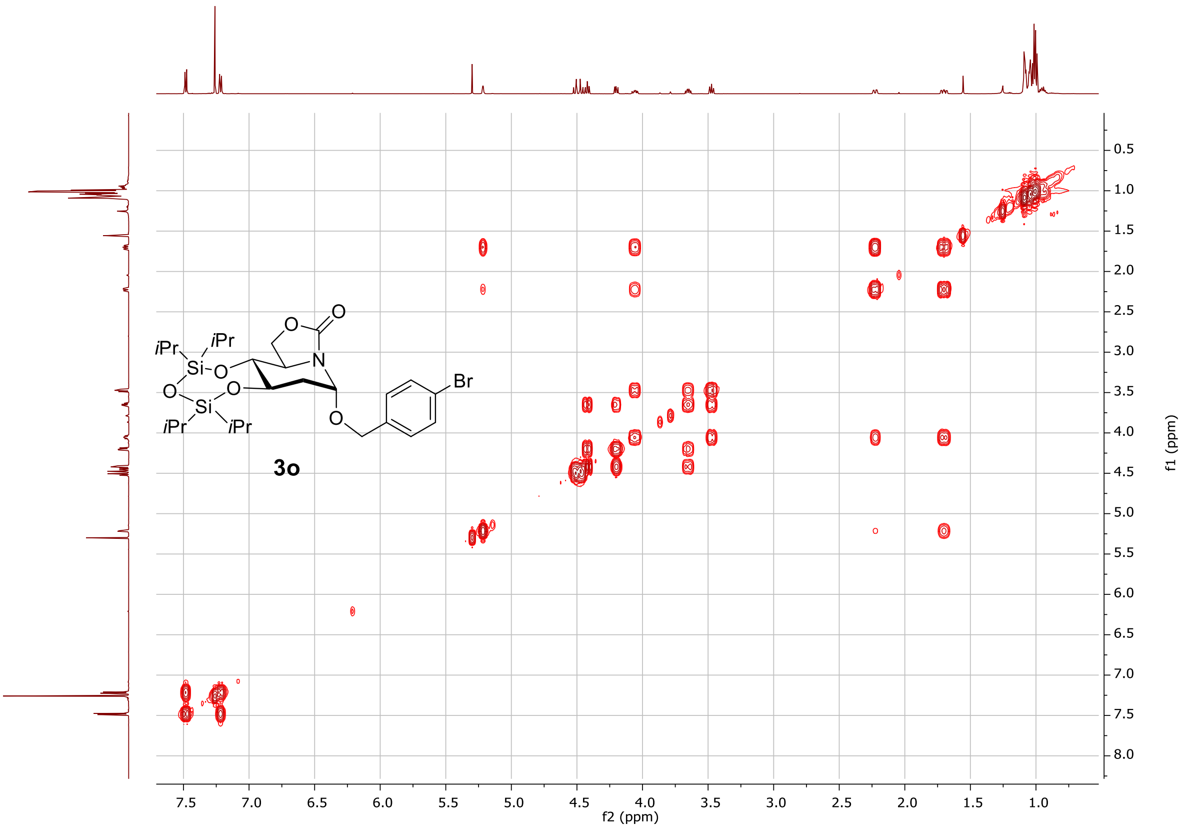

Supplementary Figure S279. COSY spectra for 3o

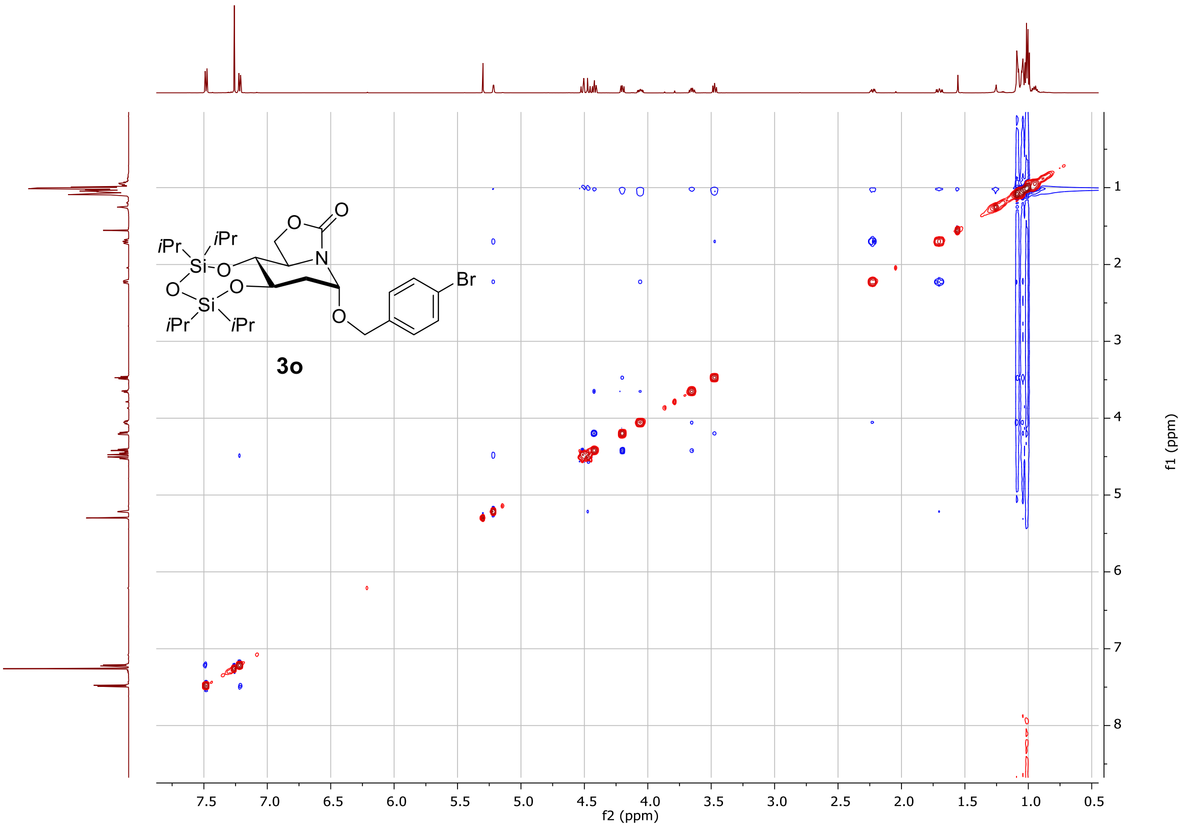

Supplementary Figure S280. NOESY spectra for 3o

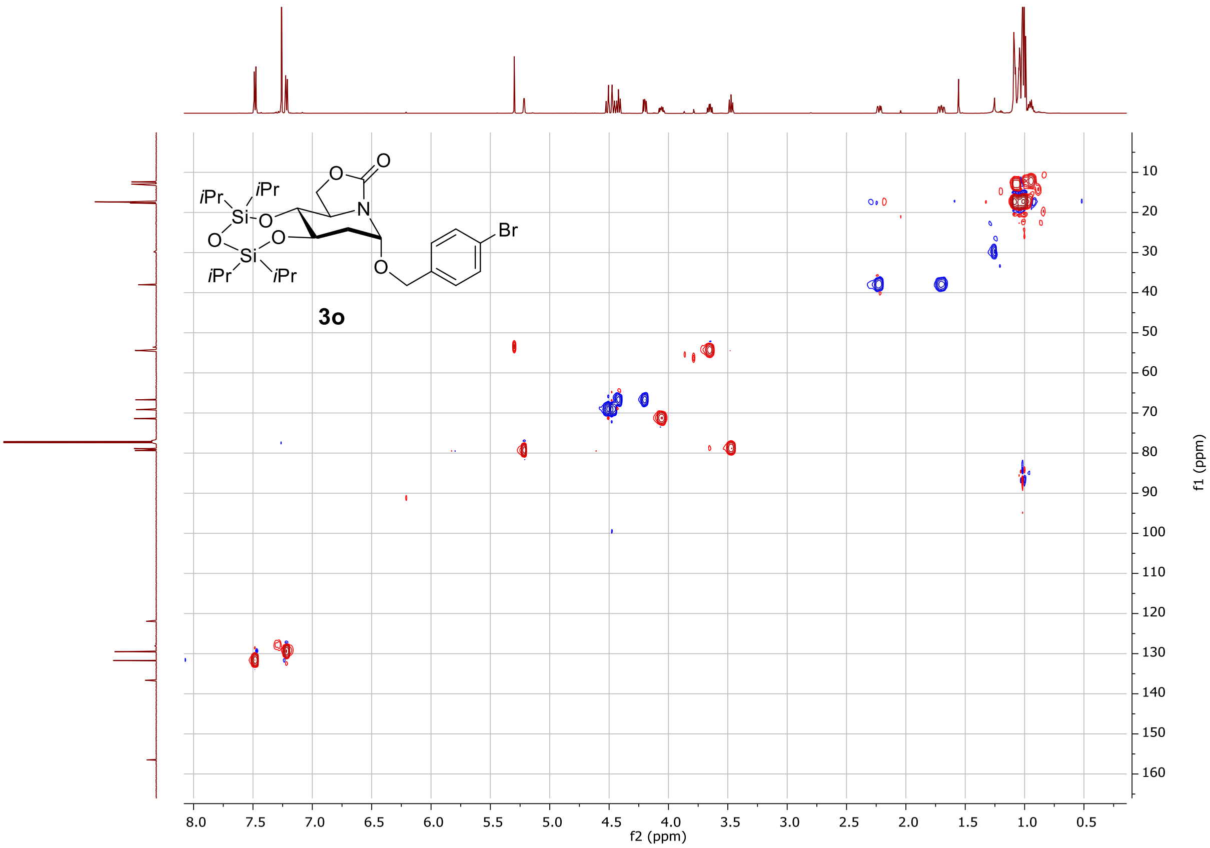

Supplementary Figure S281. HSQC spectra for 3o

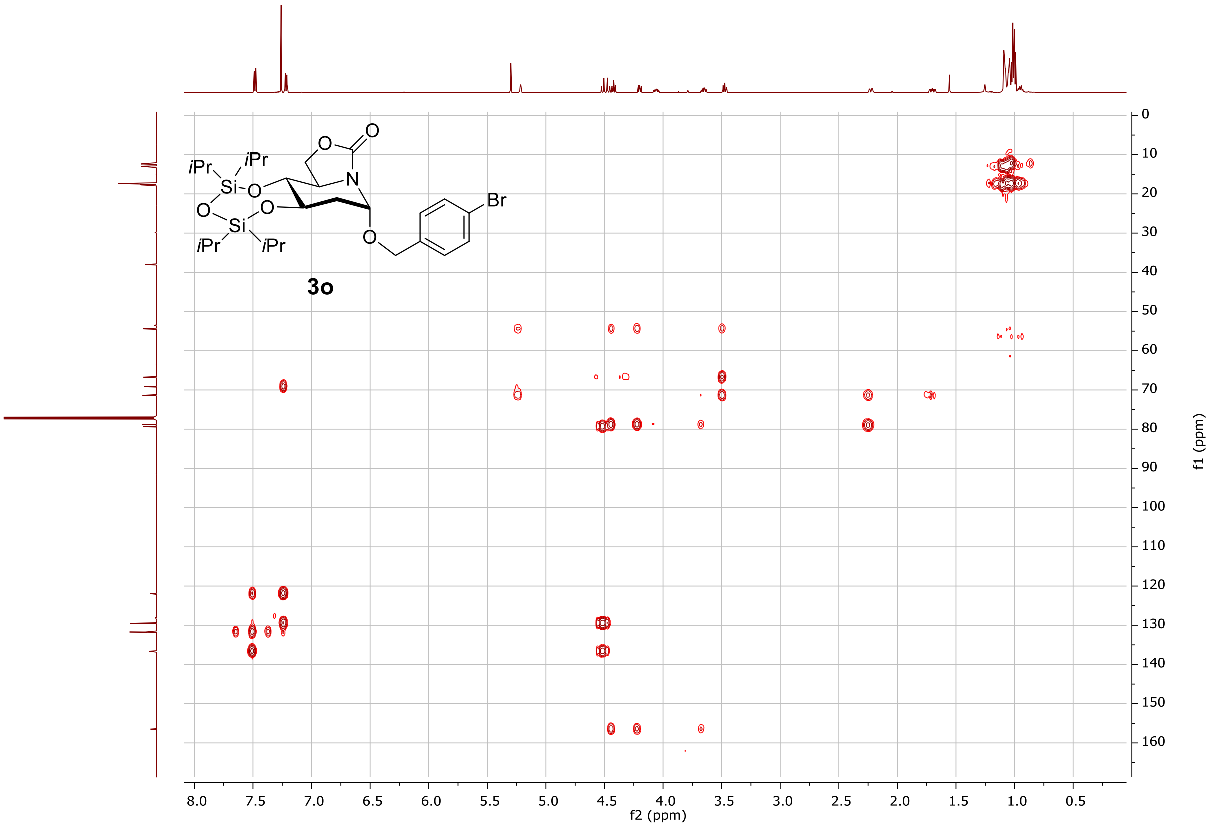

Supplementary Figure S282. HMBC spectra for 3o

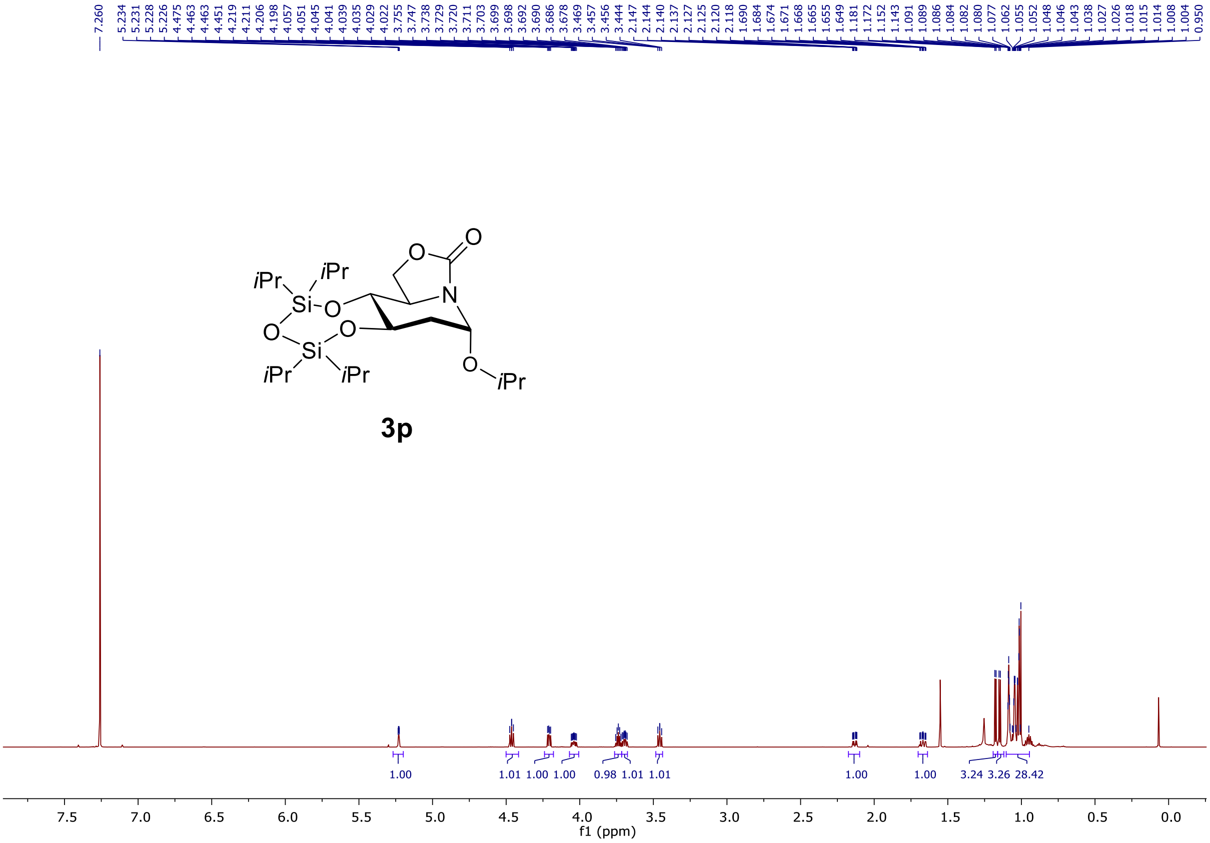

Supplementary Figure S283. ^{1}H NMR spectra for 3p

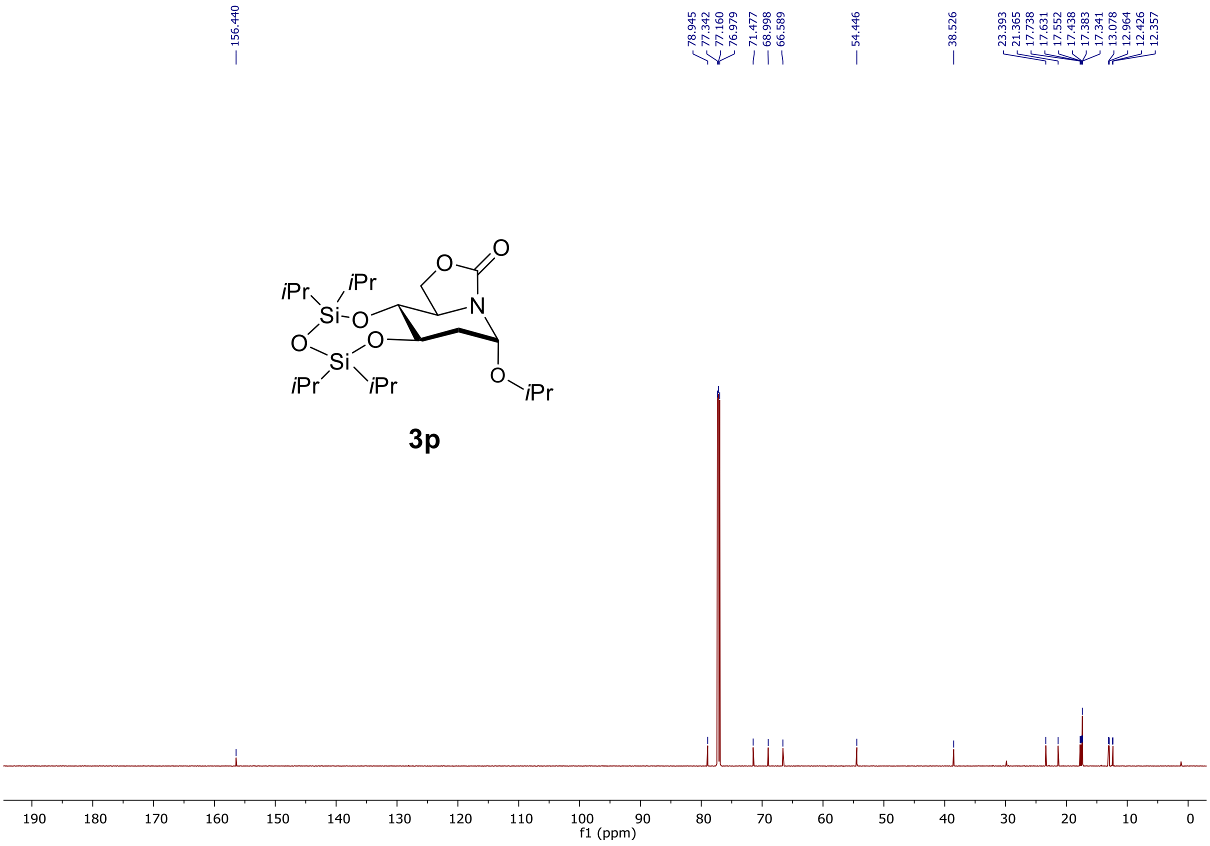

Supplementary Figure S284. ^{13}C NMR spectra for 3p

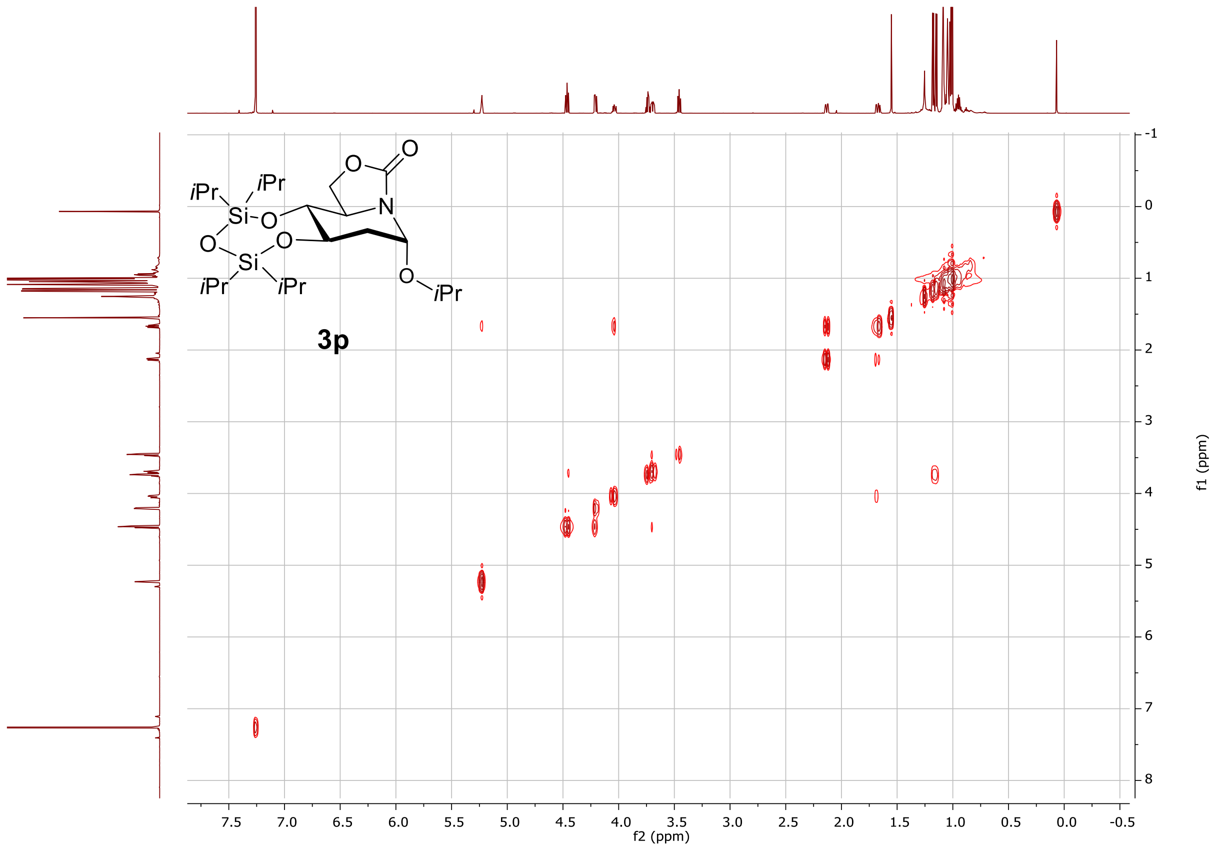

Supplementary Figure S285. COSY spectra for 3p

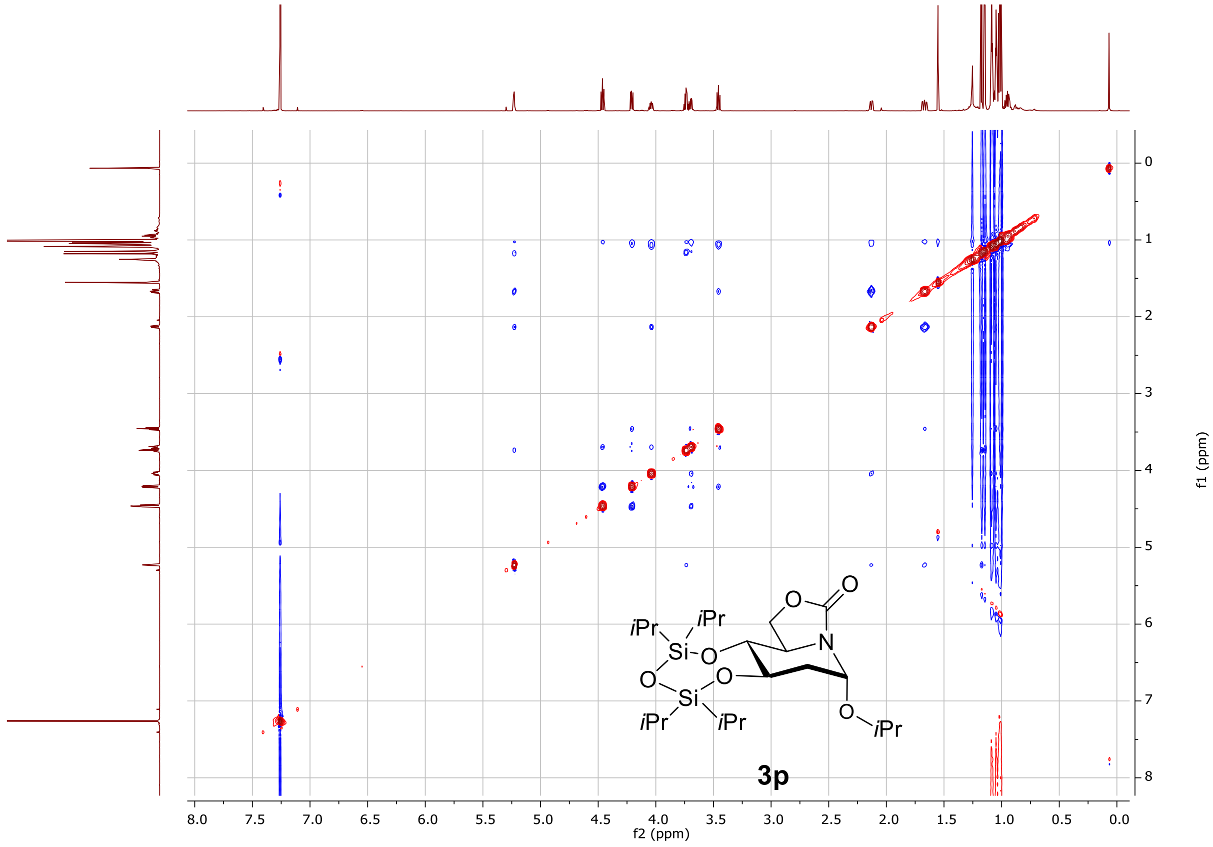

Supplementary Figure S286. NOESY spectra for 3p

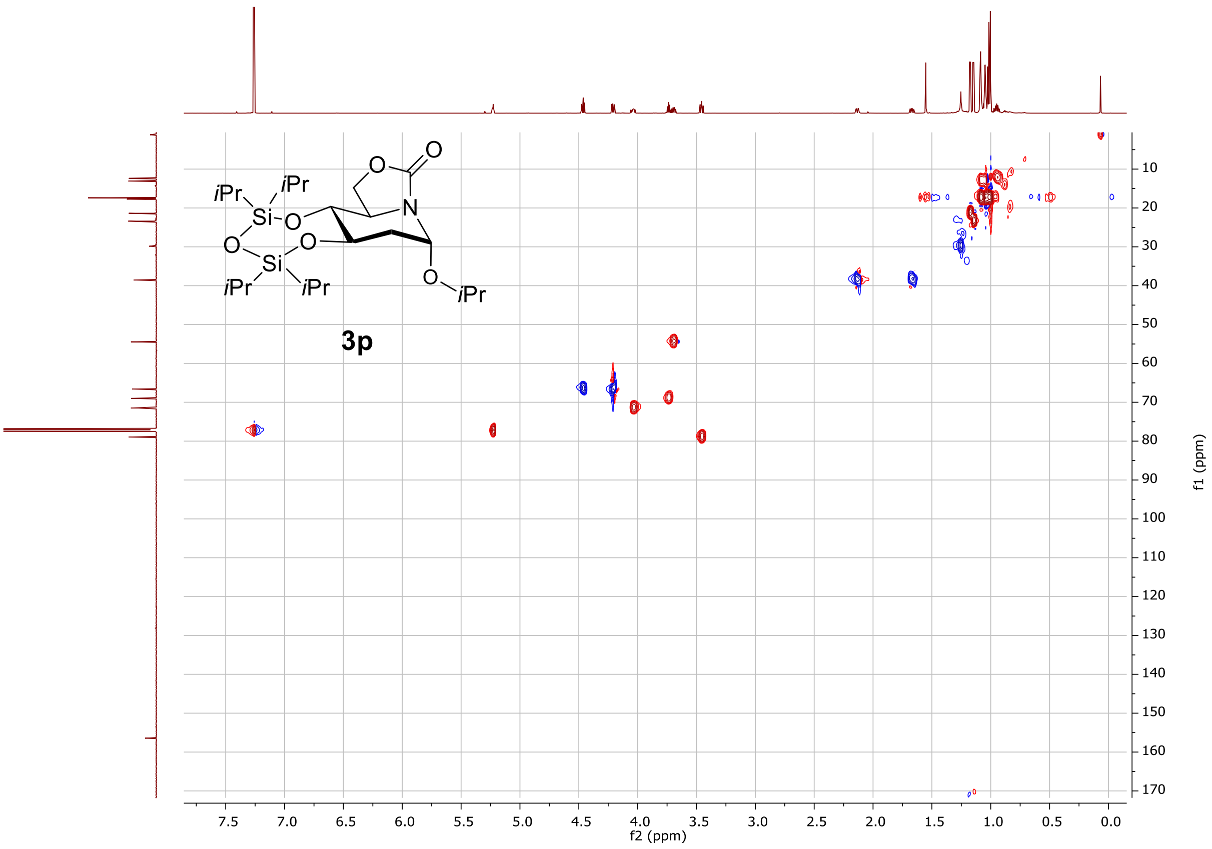

Supplementary Figure S287. HSQC spectra for 3p

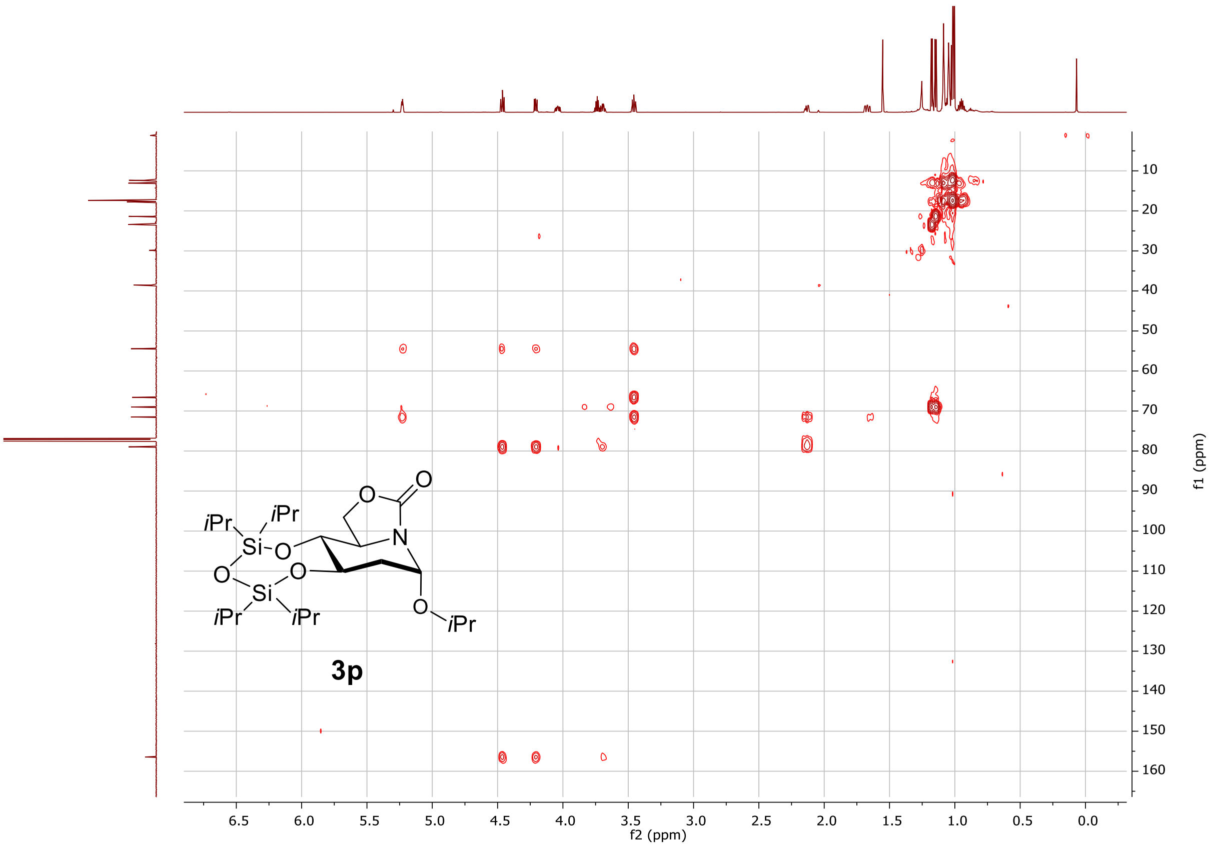

Supplementary Figure S288. HMBC spectra for 3p

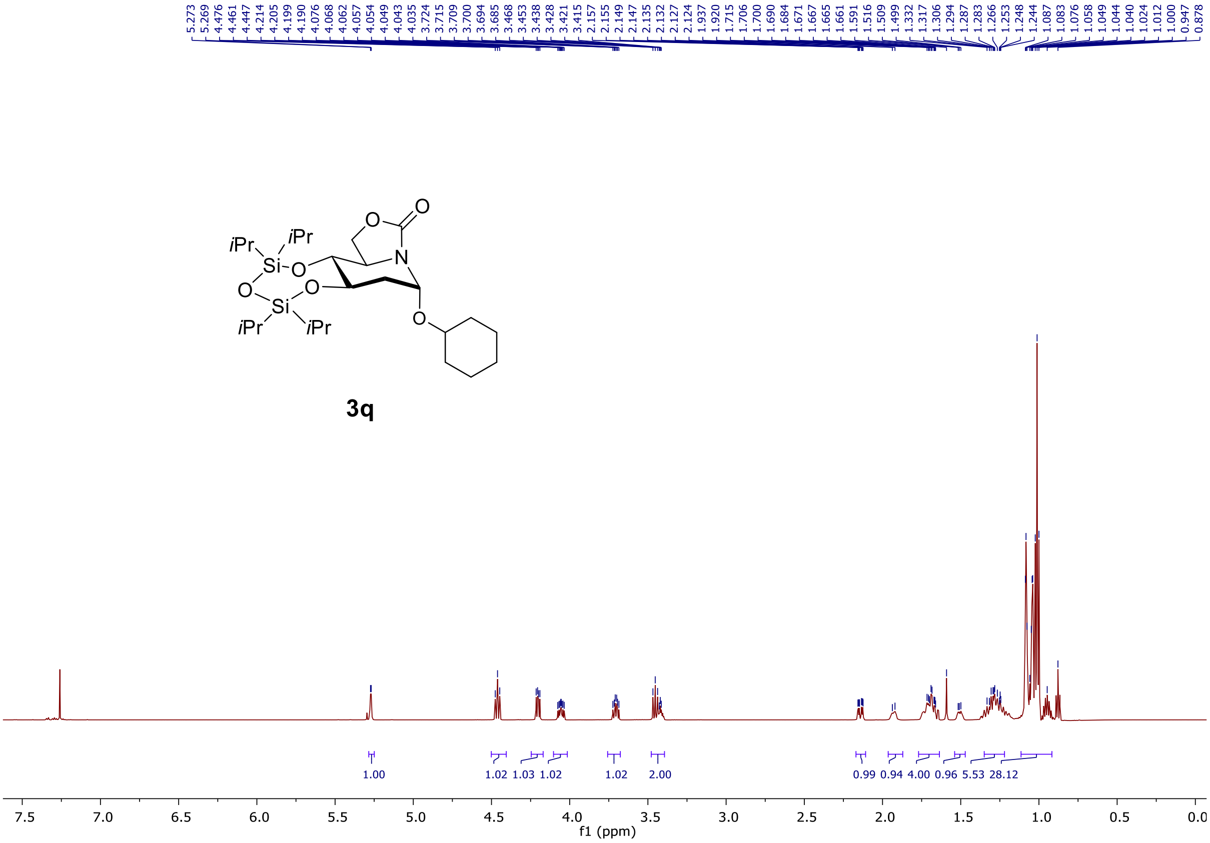

Supplementary Figure S289. ^{1}**H NMR spectra for 3q**

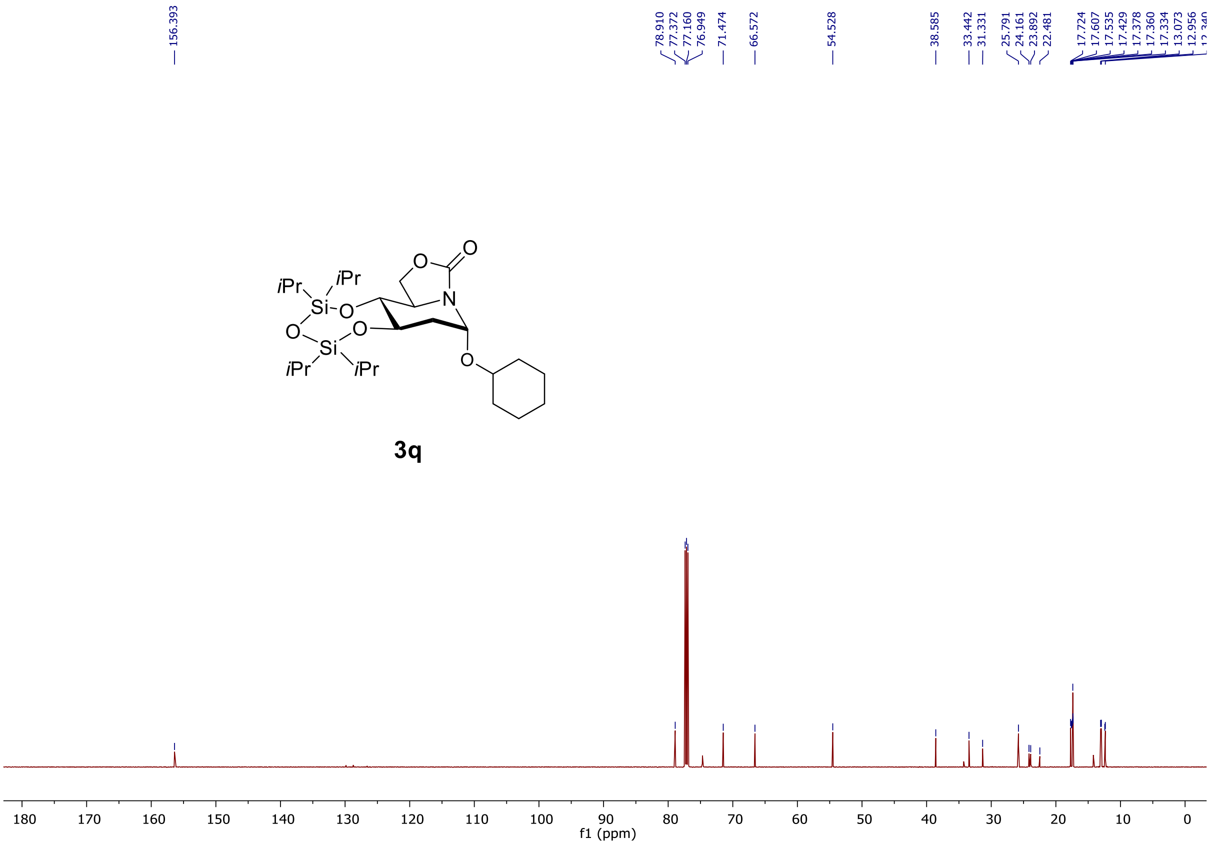

Supplementary Figure S290. ^{13}**C NMR spectra for 3q**

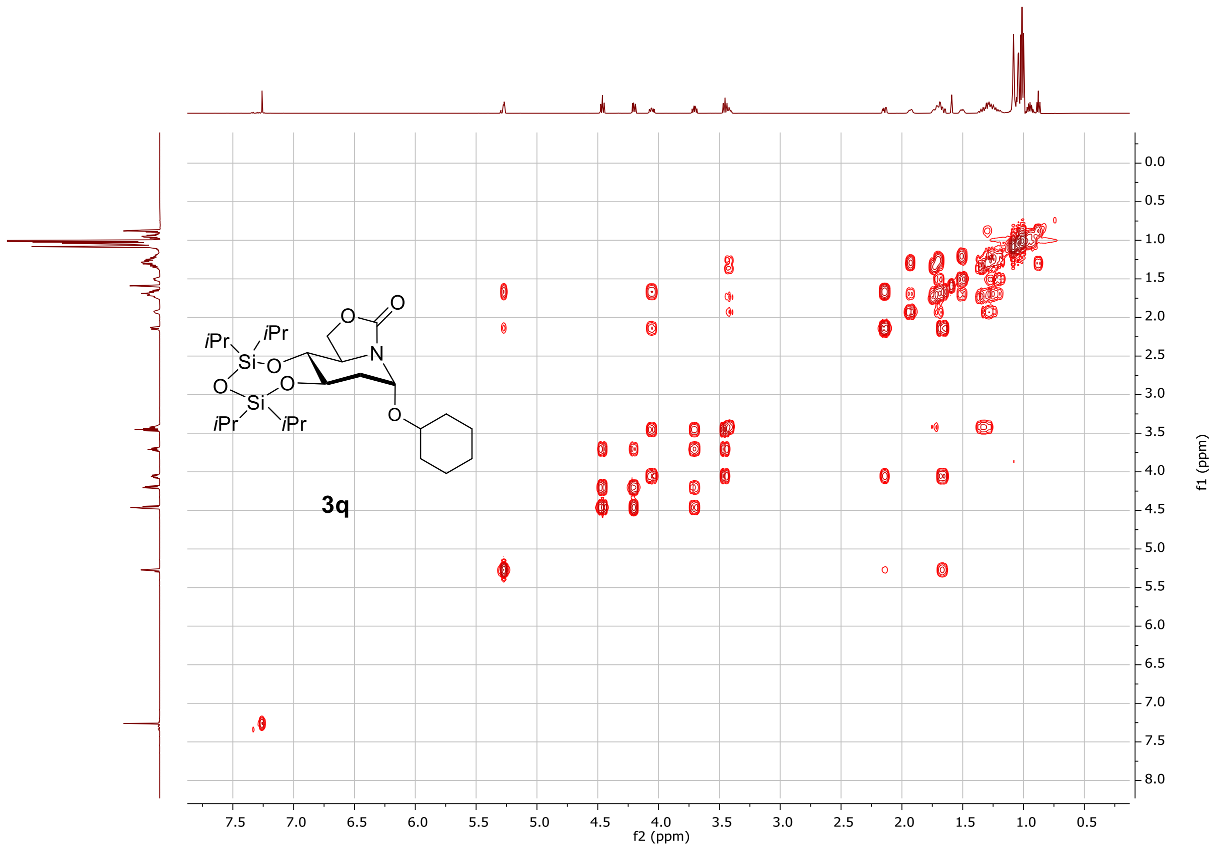

Supplementary Figure S291. COSY spectra for 3q

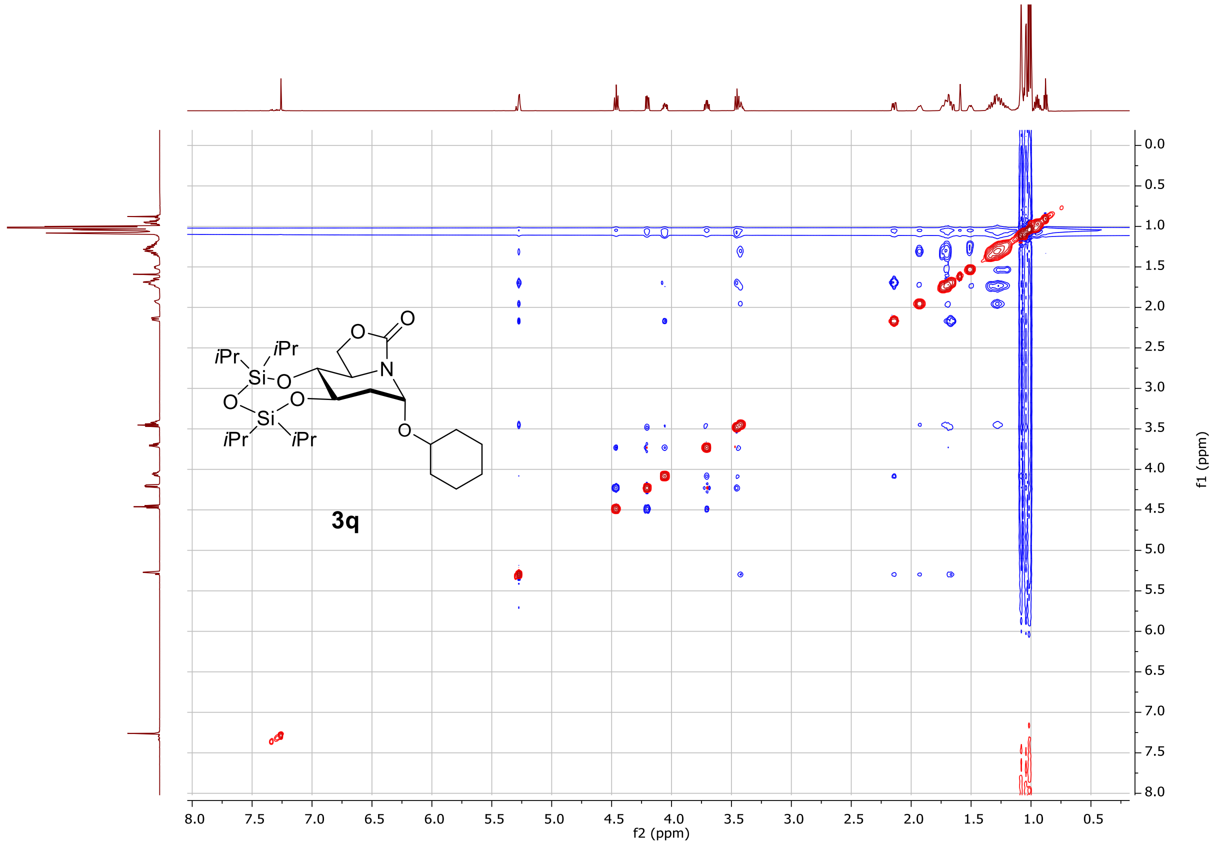

Supplementary Figure S292. NOESY spectra for 3q

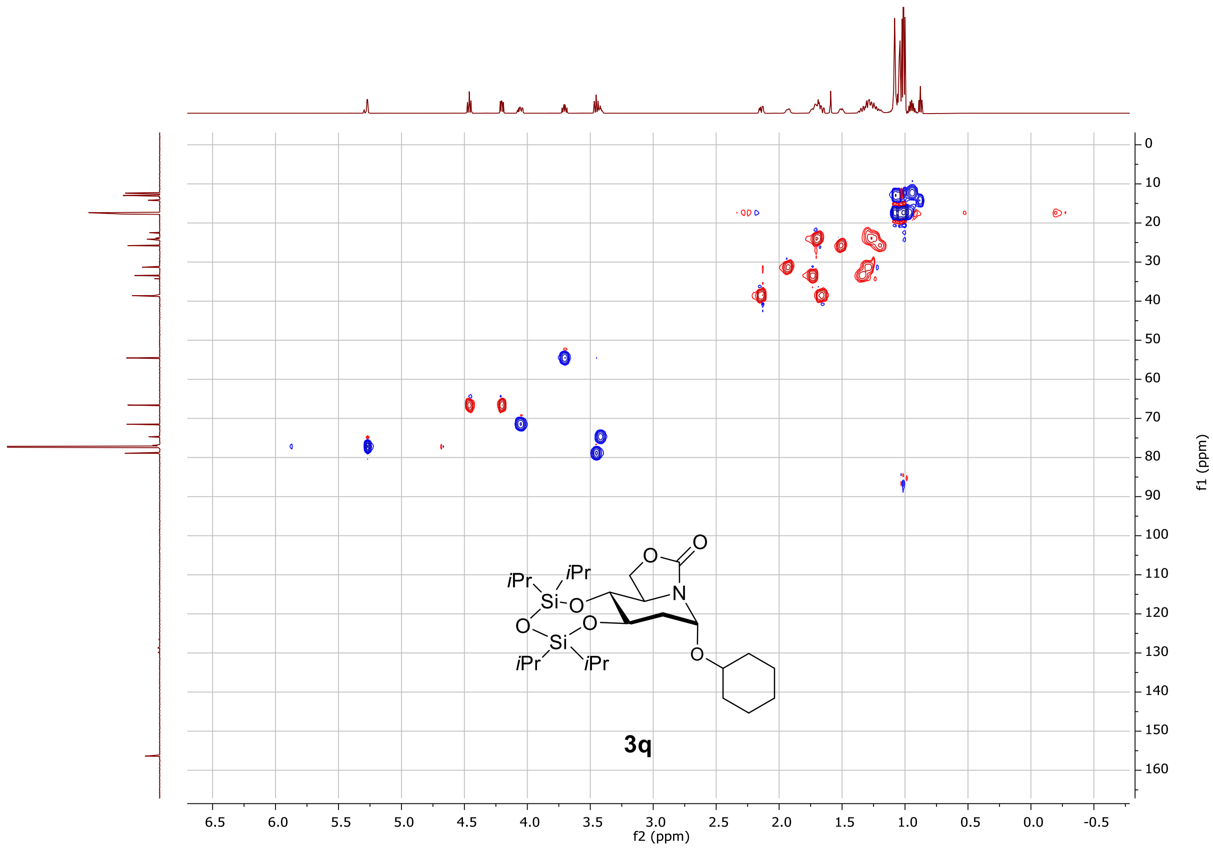

Supplementary Figure S293. HSQC spectra for 3q

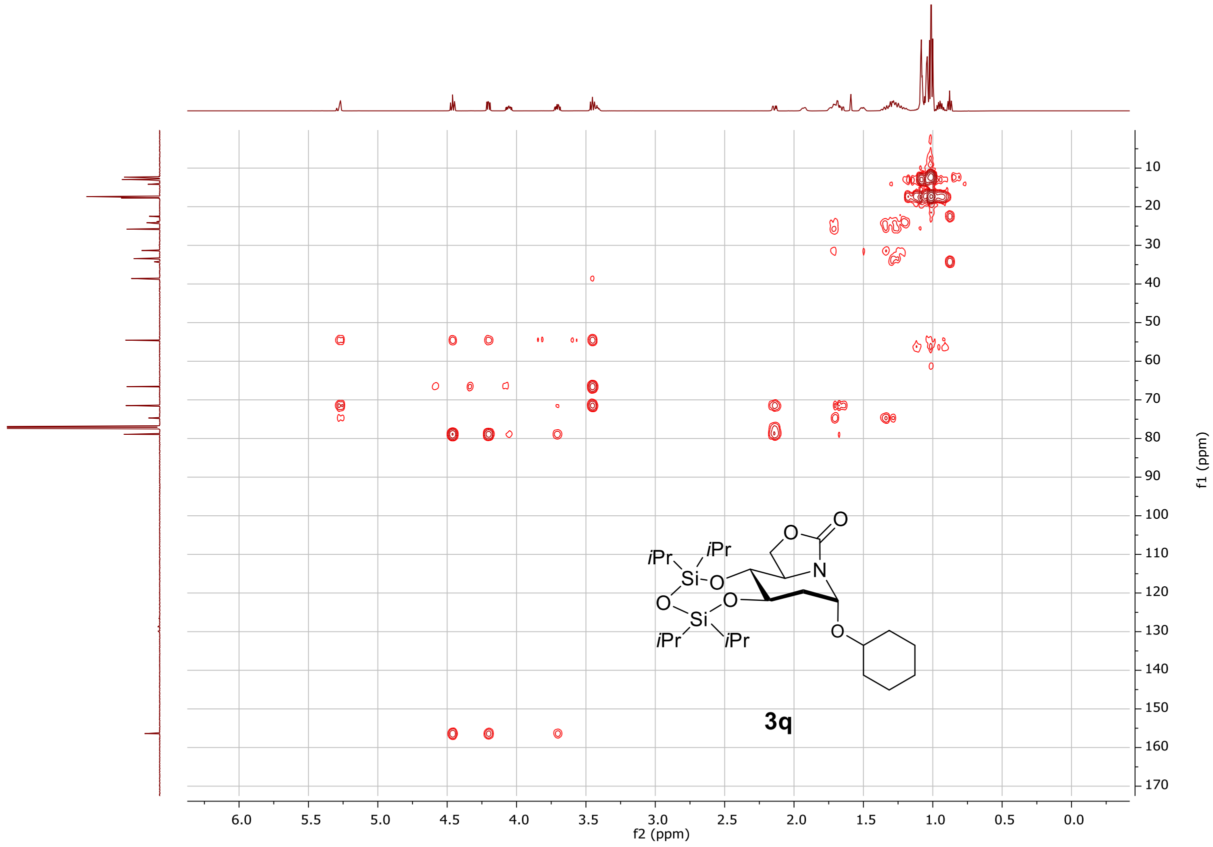

Supplementary Figure S294. HMBC spectra for 3q

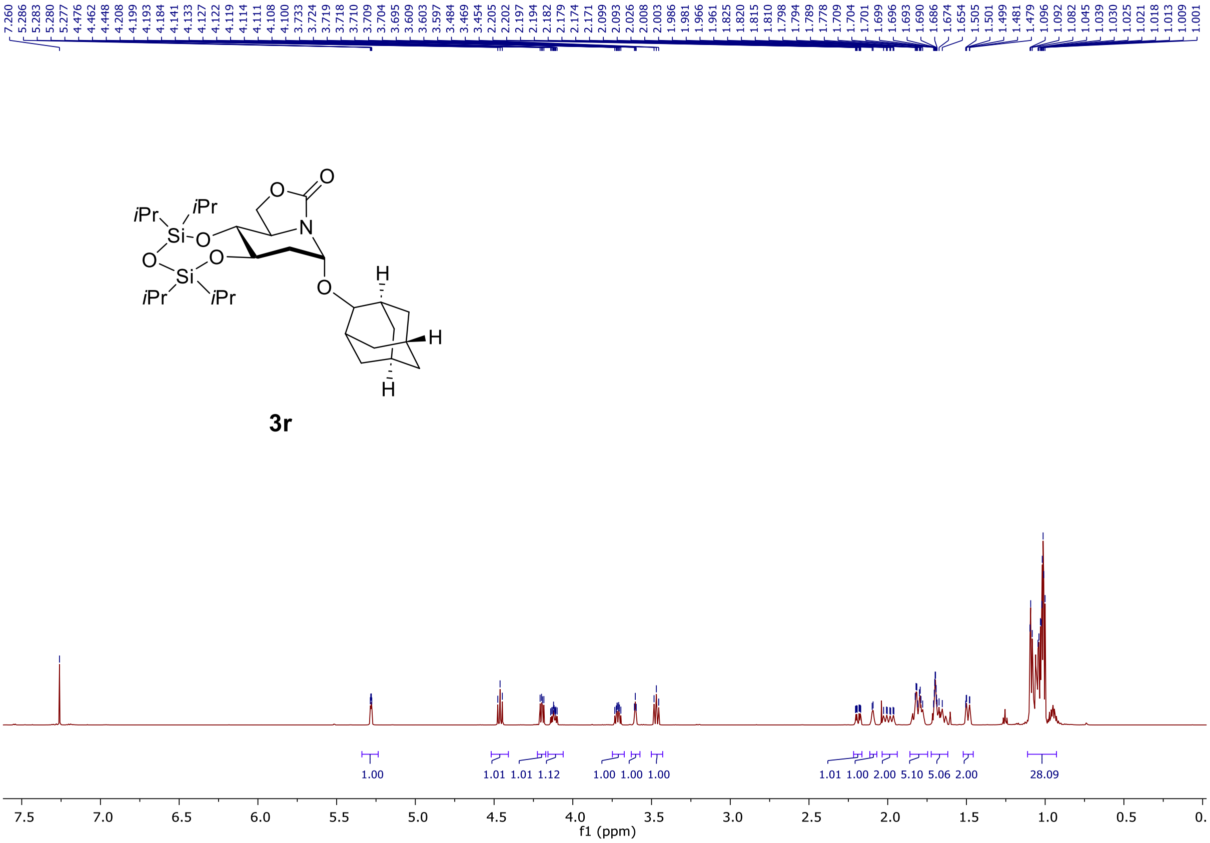

Supplementary Figure S295. ^{1}H NMR spectra for 3r

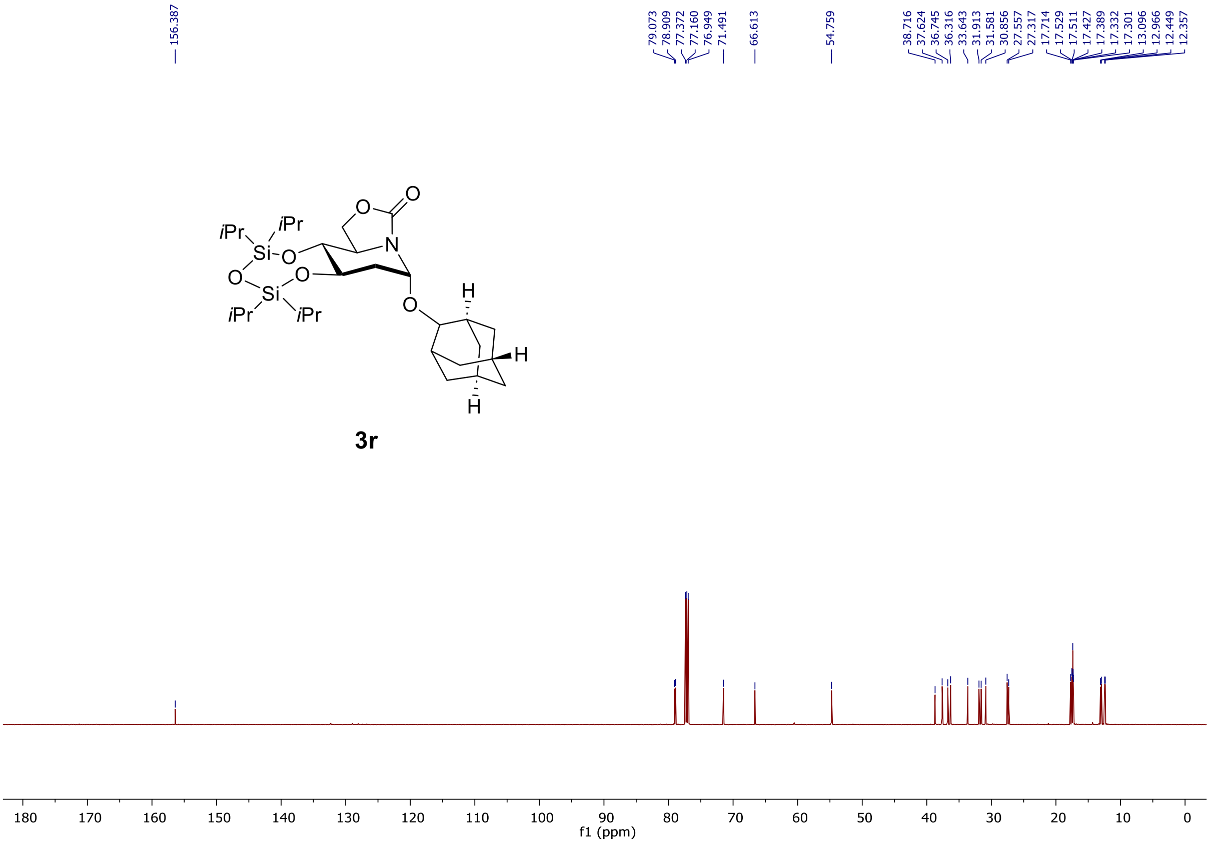

Supplementary Figure S296. ^{13}C NMR spectra for 3r

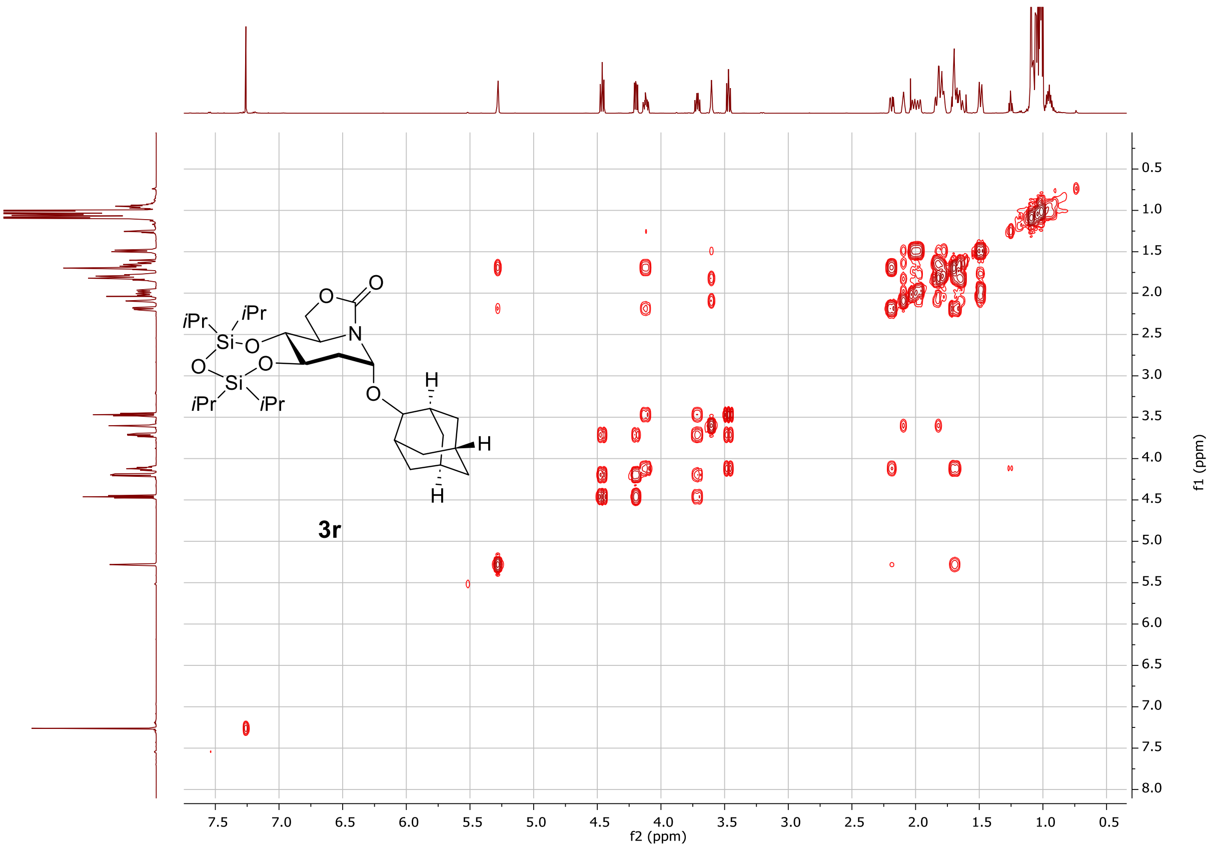

Supplementary Figure S297. COSY spectra for 3r

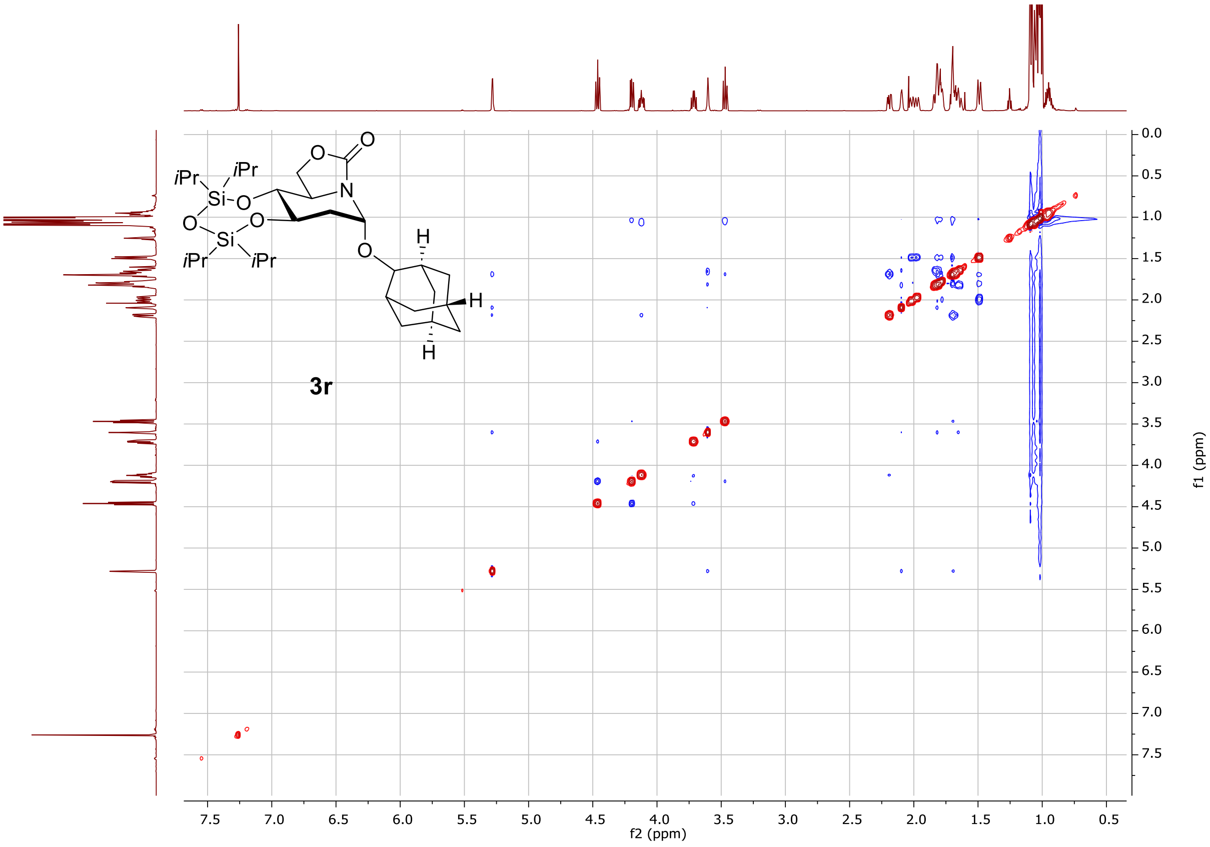

Supplementary Figure S298. NOESY spectra for 3r

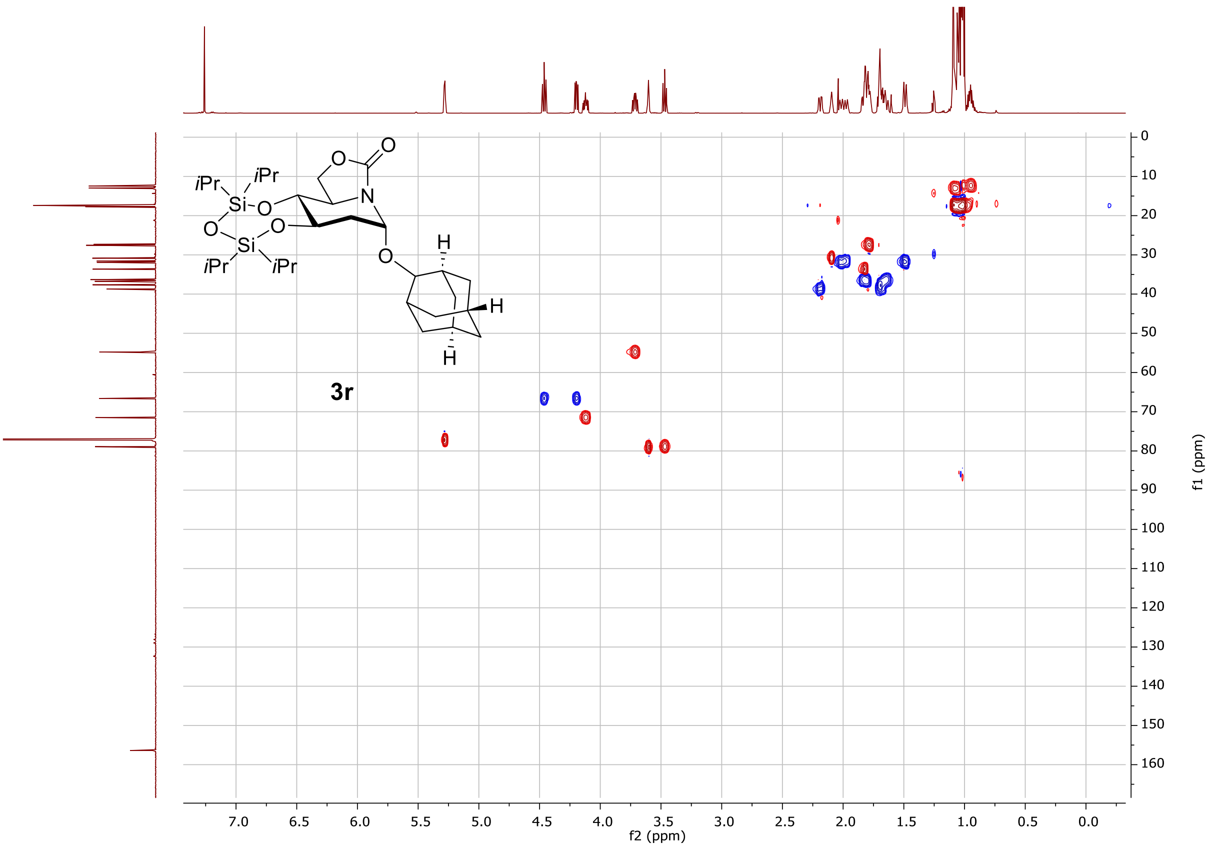

Supplementary Figure S299. HSQC spectra for 3r

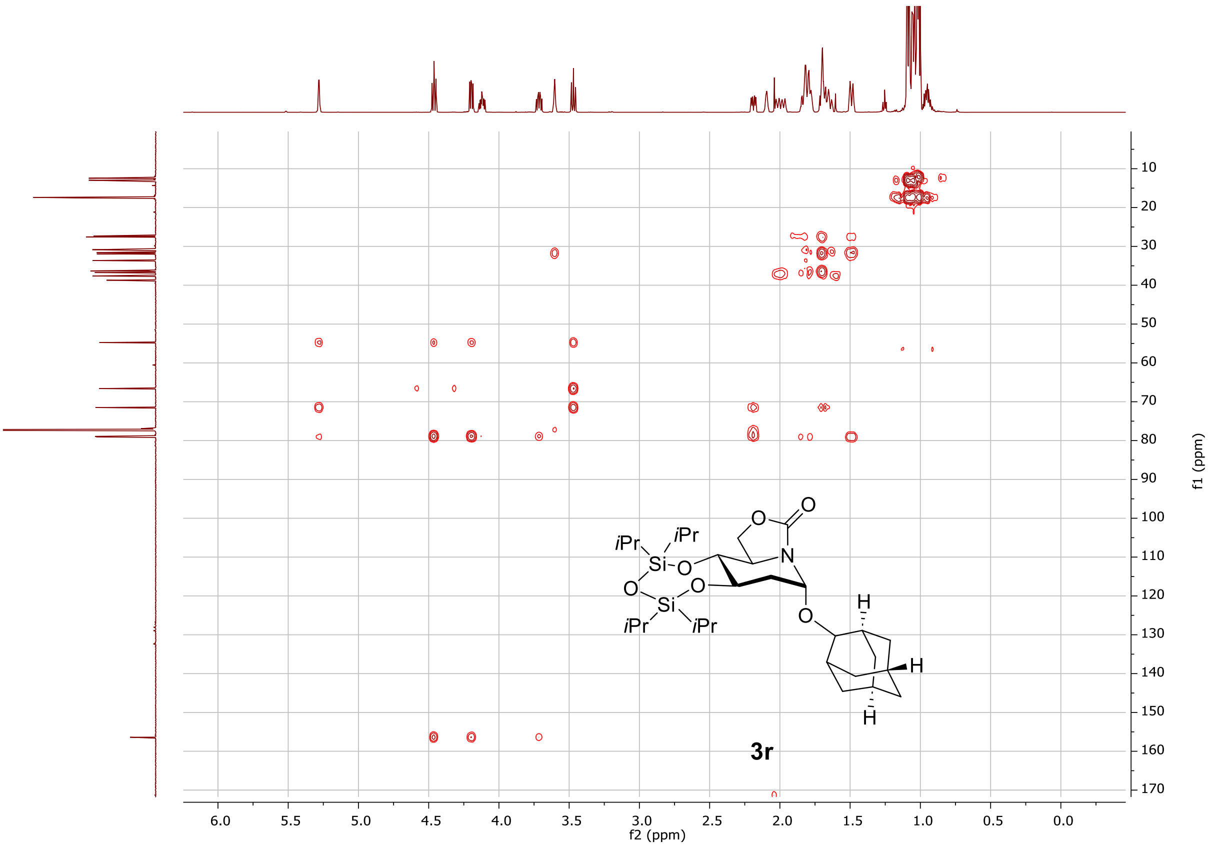

Supplementary Figure S300. HMBC spectra for 3r

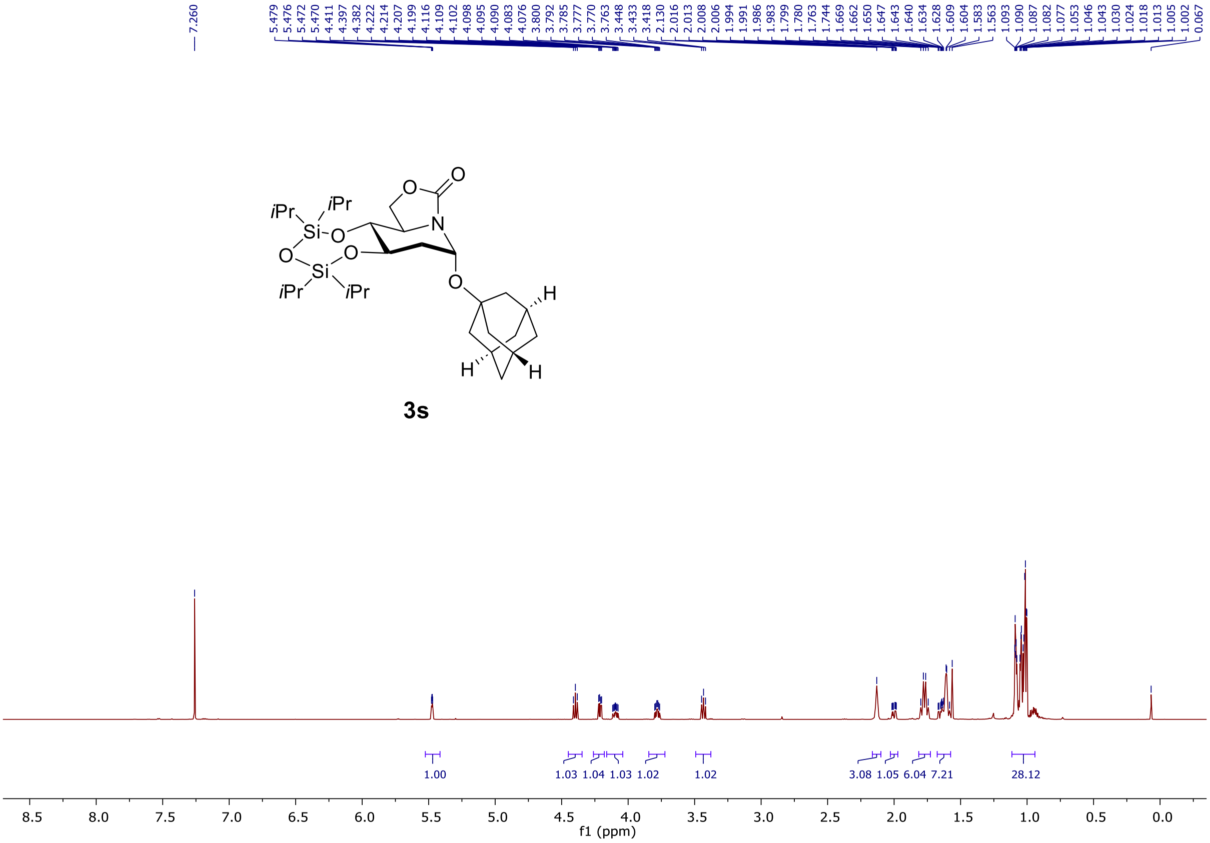

Supplementary Figure S301. ^{1}H NMR spectra for 3s

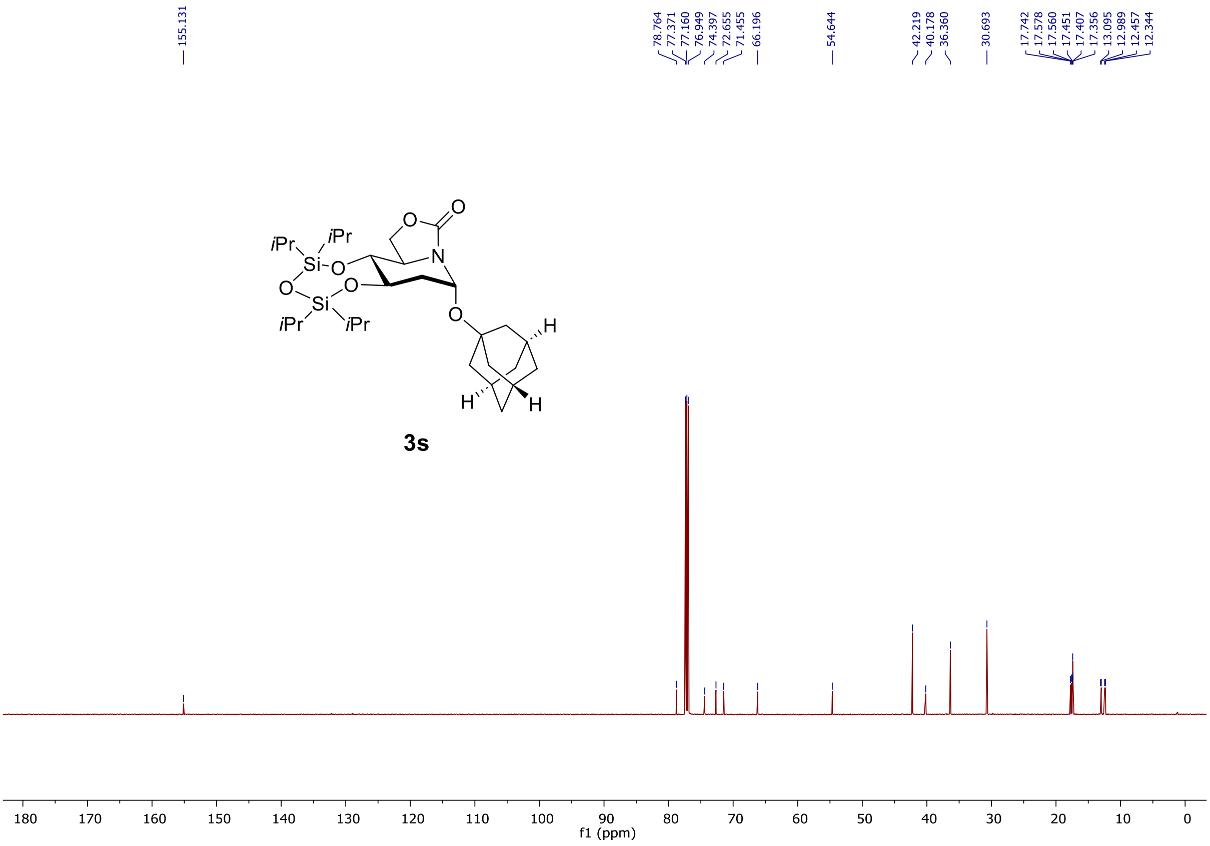

Supplementary Figure S302. ^{13}C NMR spectra for 3s

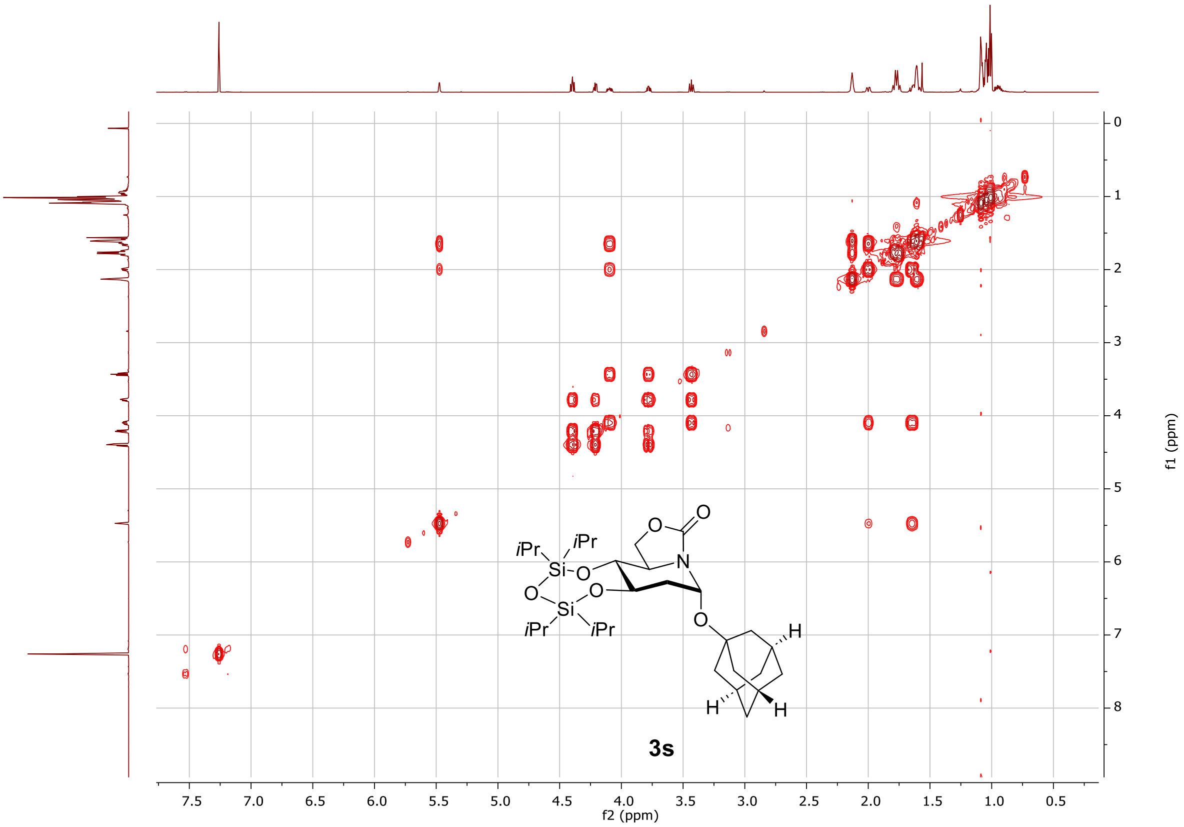

Supplementary Figure S303. COSY spectra for 3s

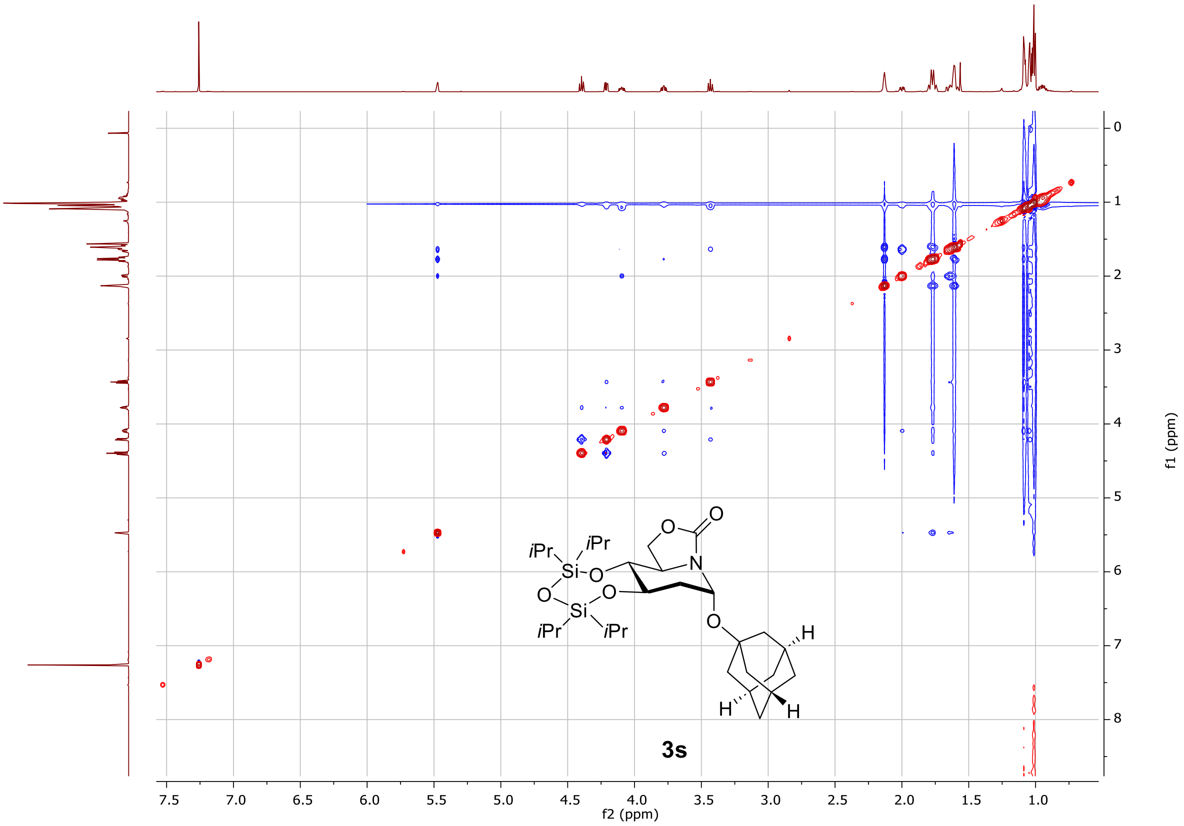

Supplementary Figure S304. NOESY spectra for 3s

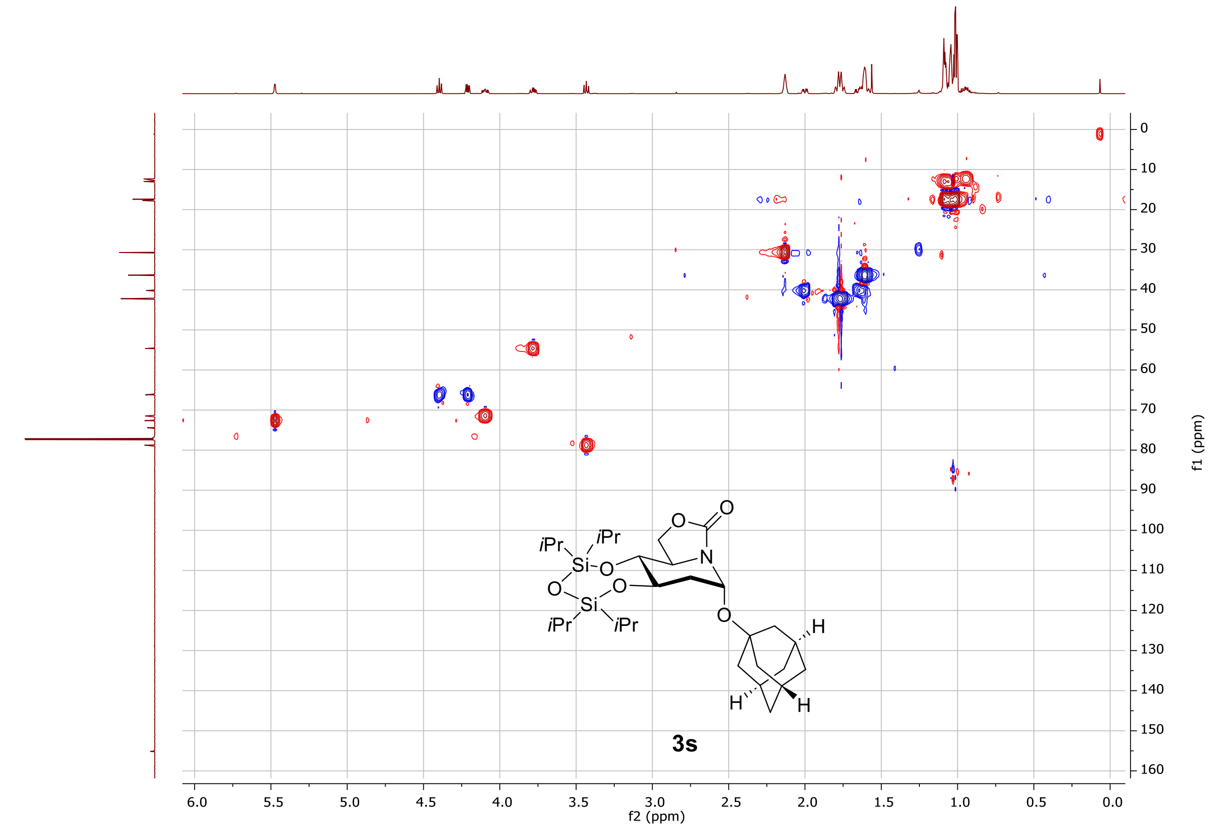

Supplementary Figure S305. HSQC spectra for 3s

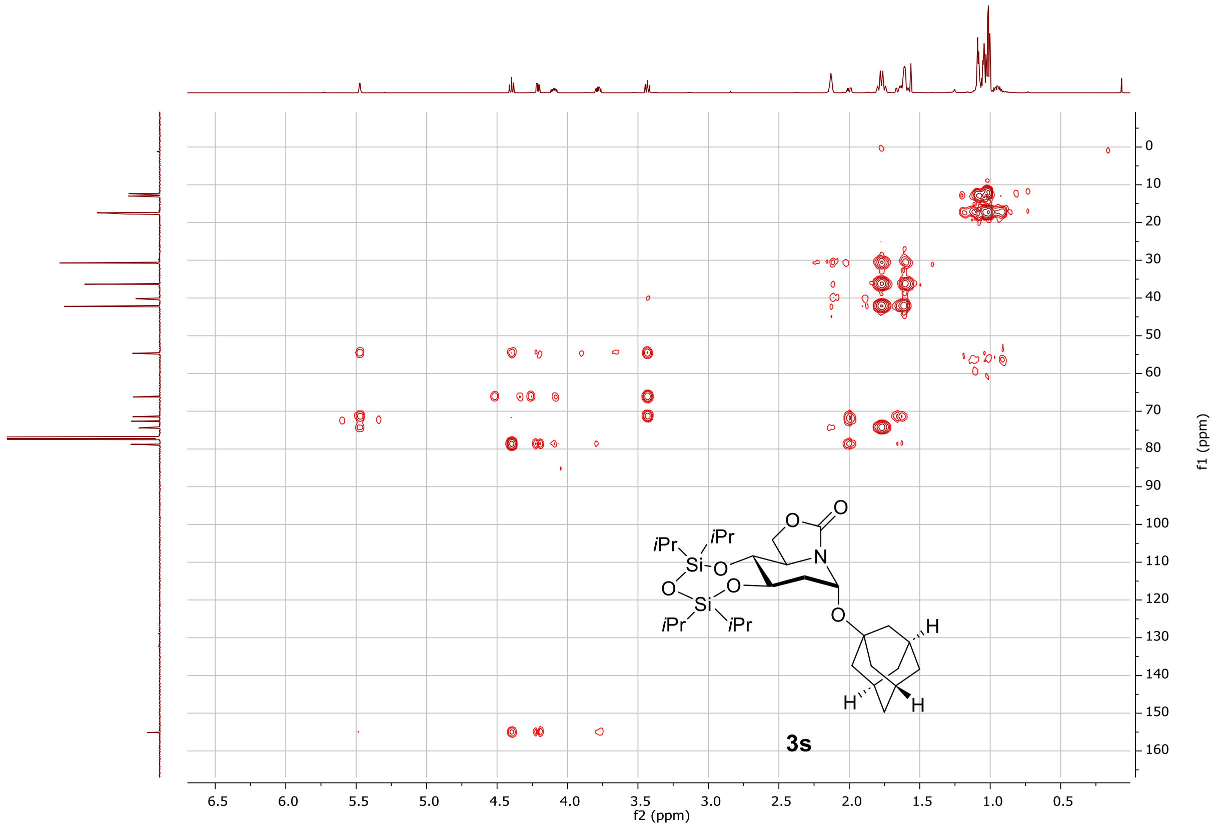

Supplementary Figure S306. HMBC spectra for 3s

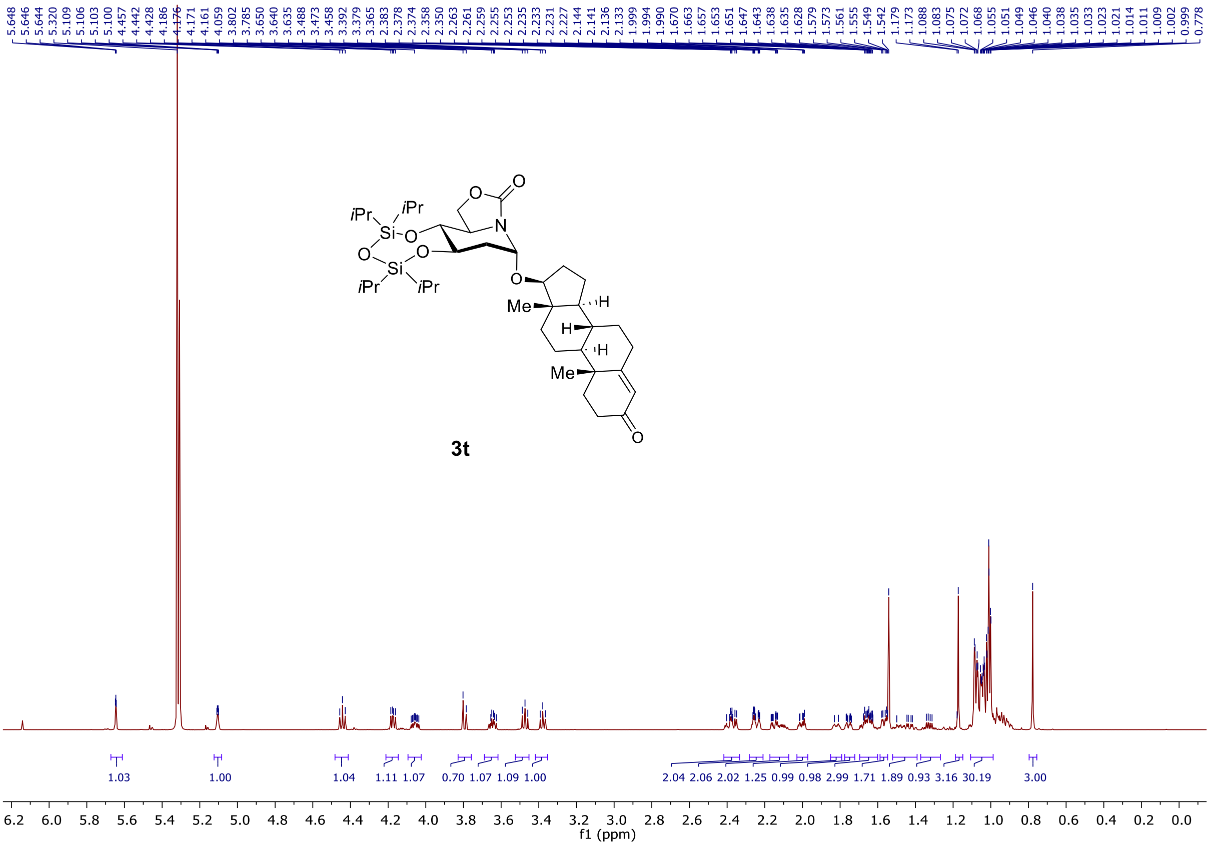

Supplementary Figure S307. ^{1}H **NMR spectra for 3t**

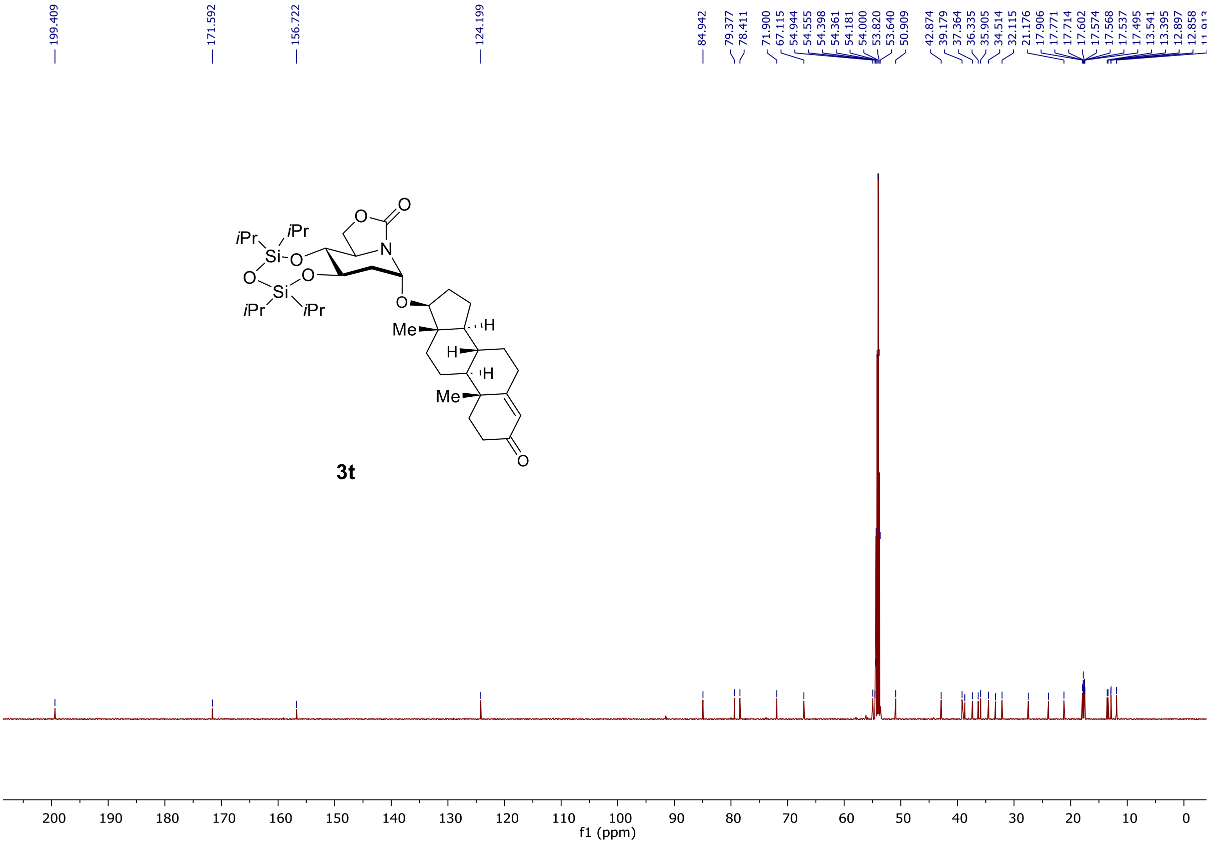

Supplementary Figure S308. ^{13}C **NMR spectra for 3t**

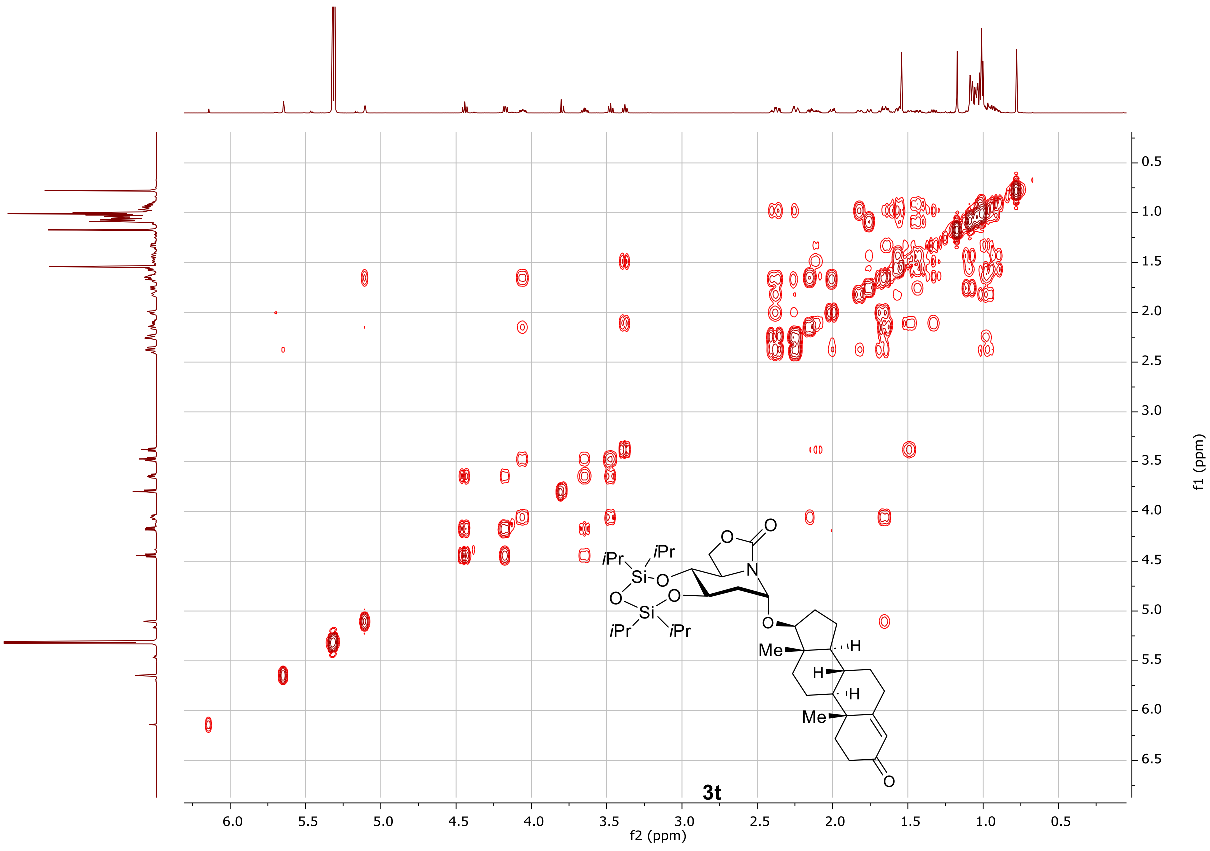

Supplementary Figure S309. COSY spectra for 3t

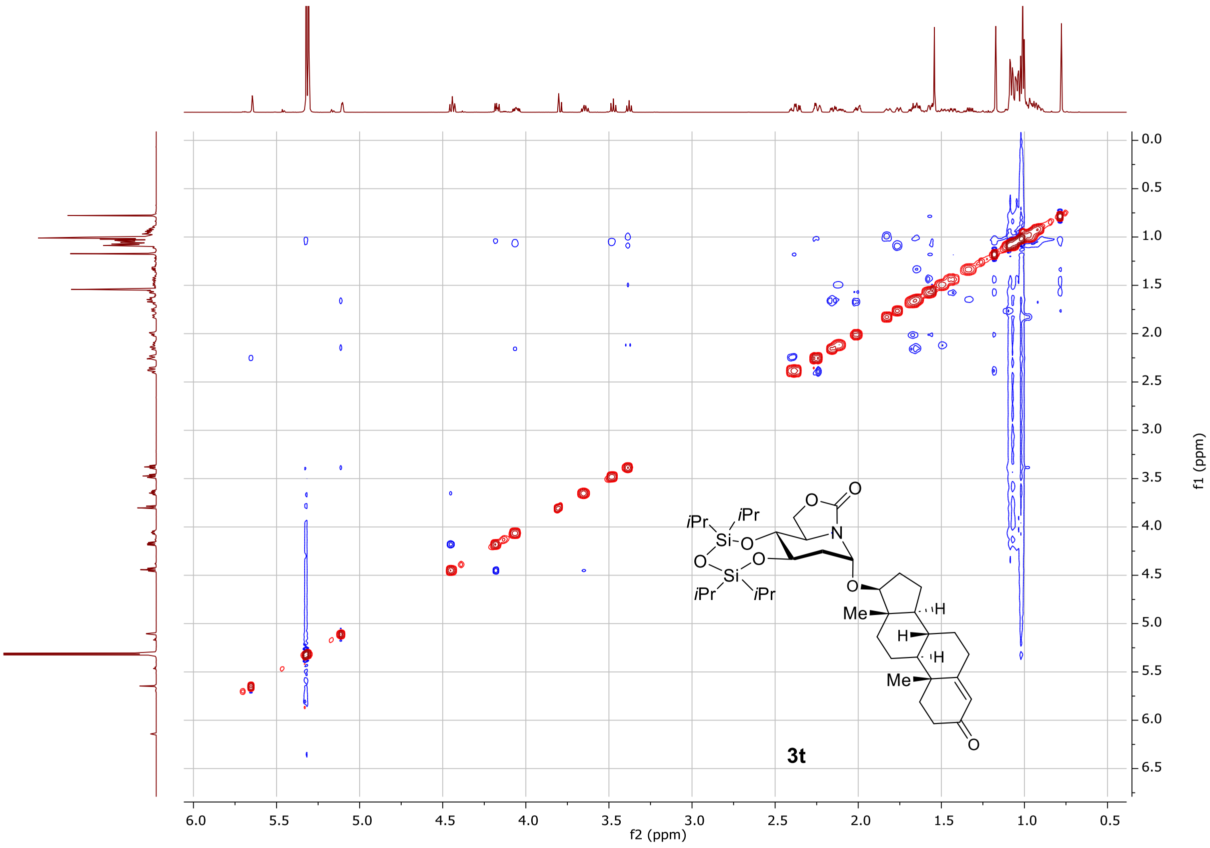

Supplementary Figure S310. NOESY spectra for 3t

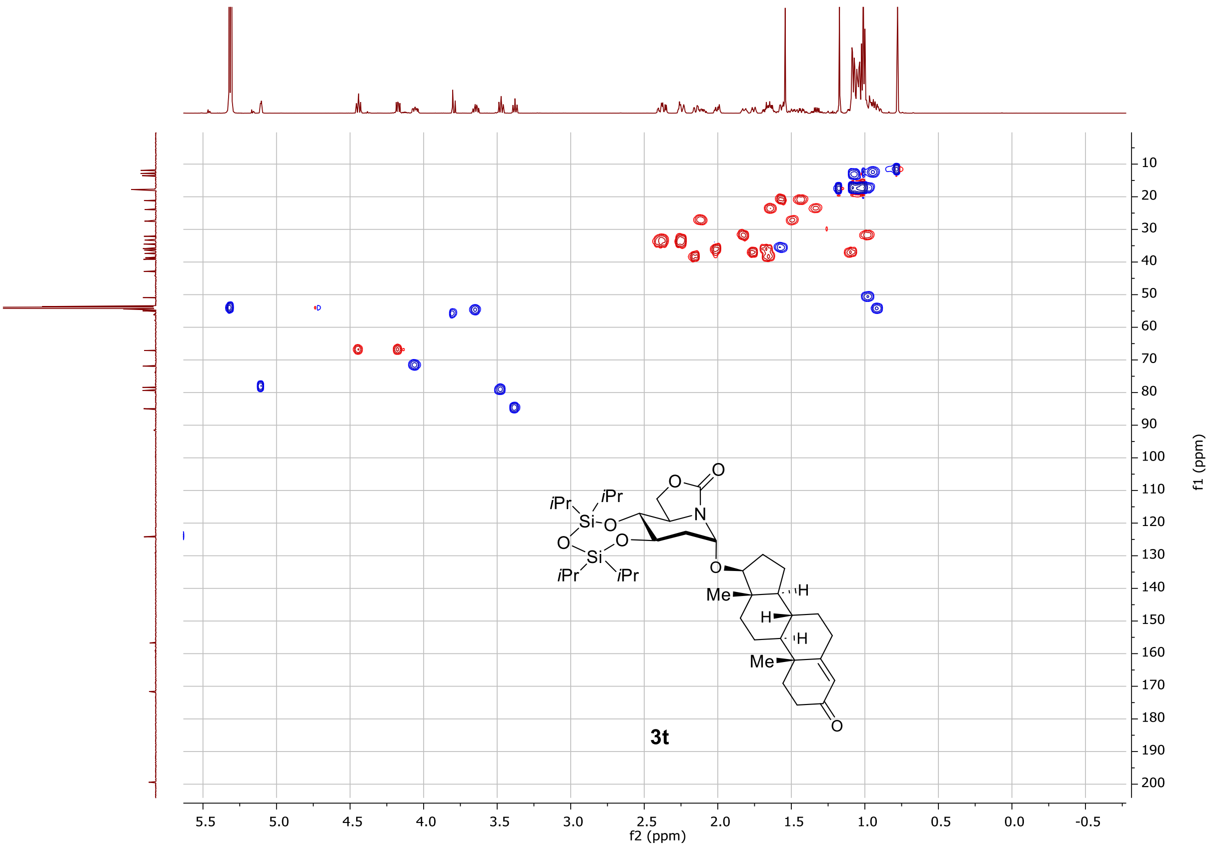

Supplementary Figure S311. HSQC spectra for 3t

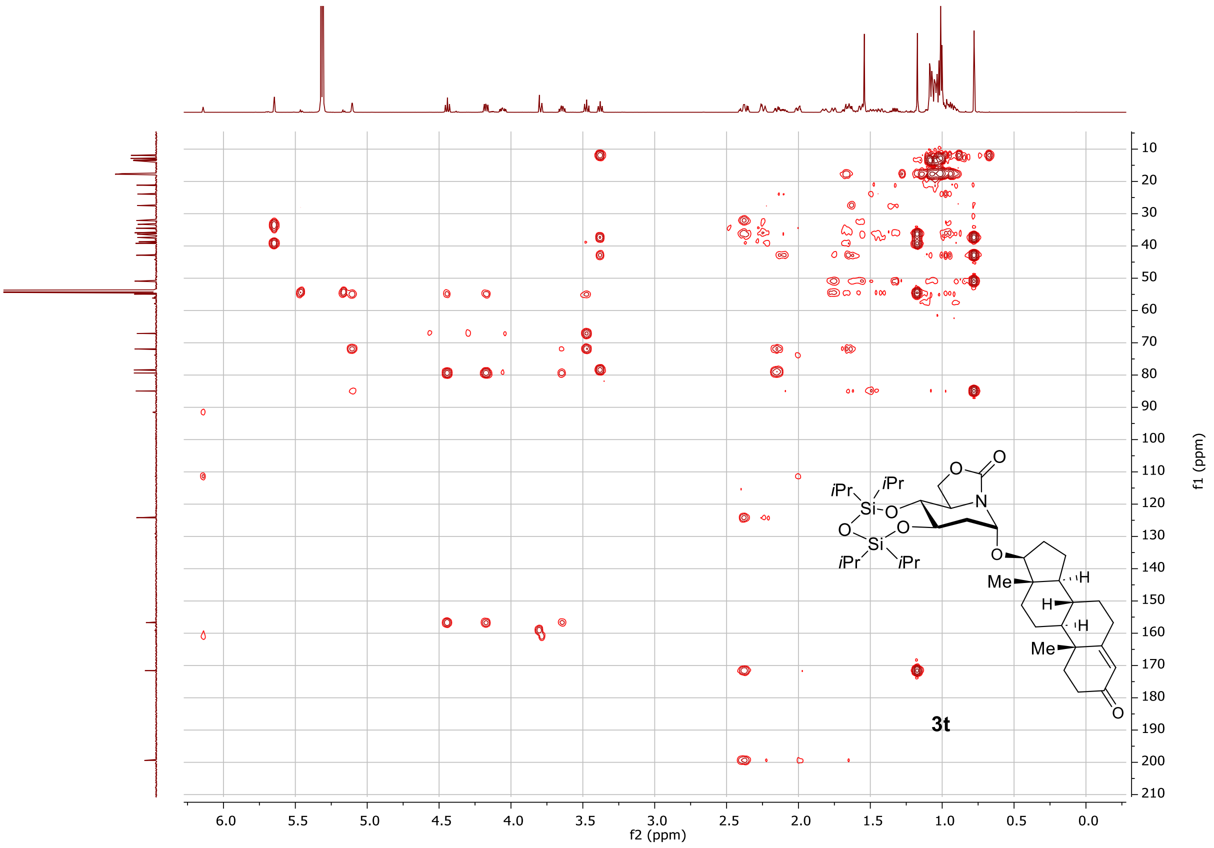

Supplementary Figure S312. HMBC spectra for 3t

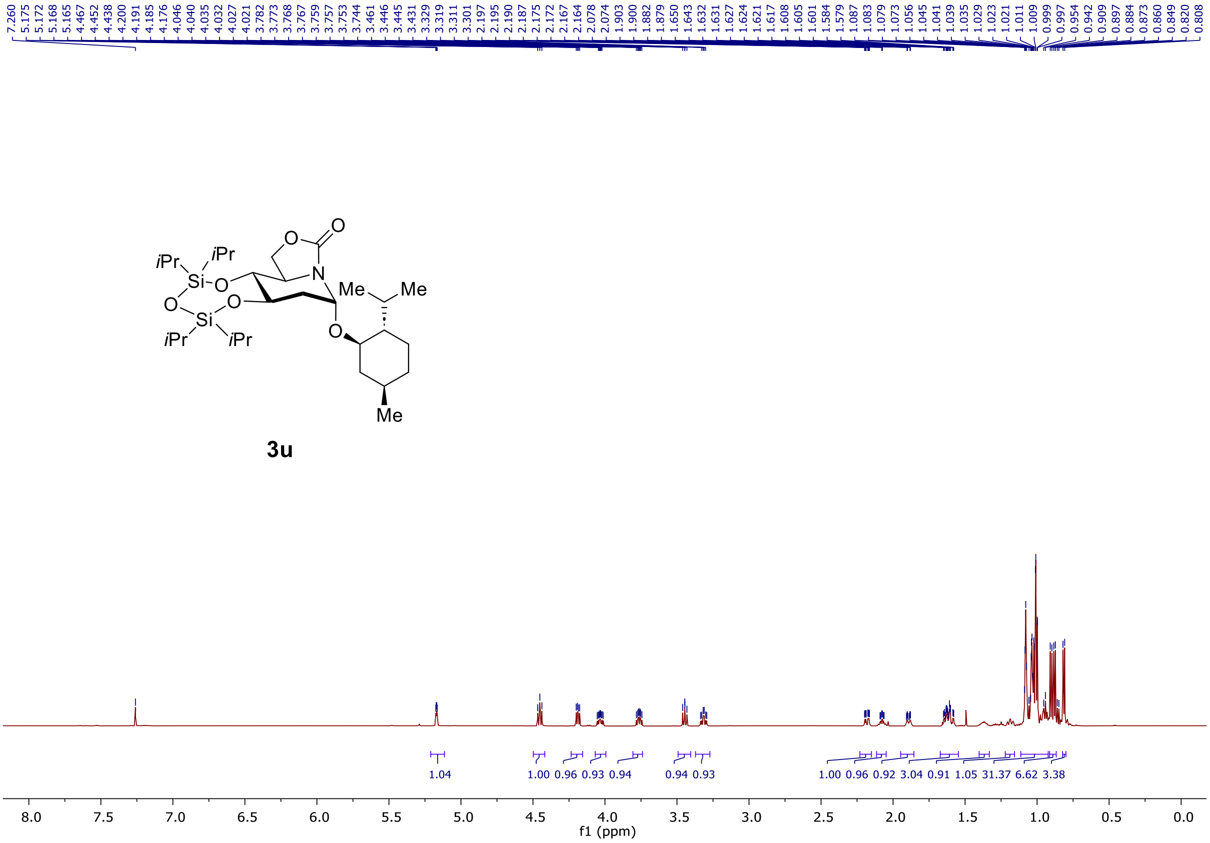

Supplementary Figure S313. ^{1}H NMR spectra for 3u

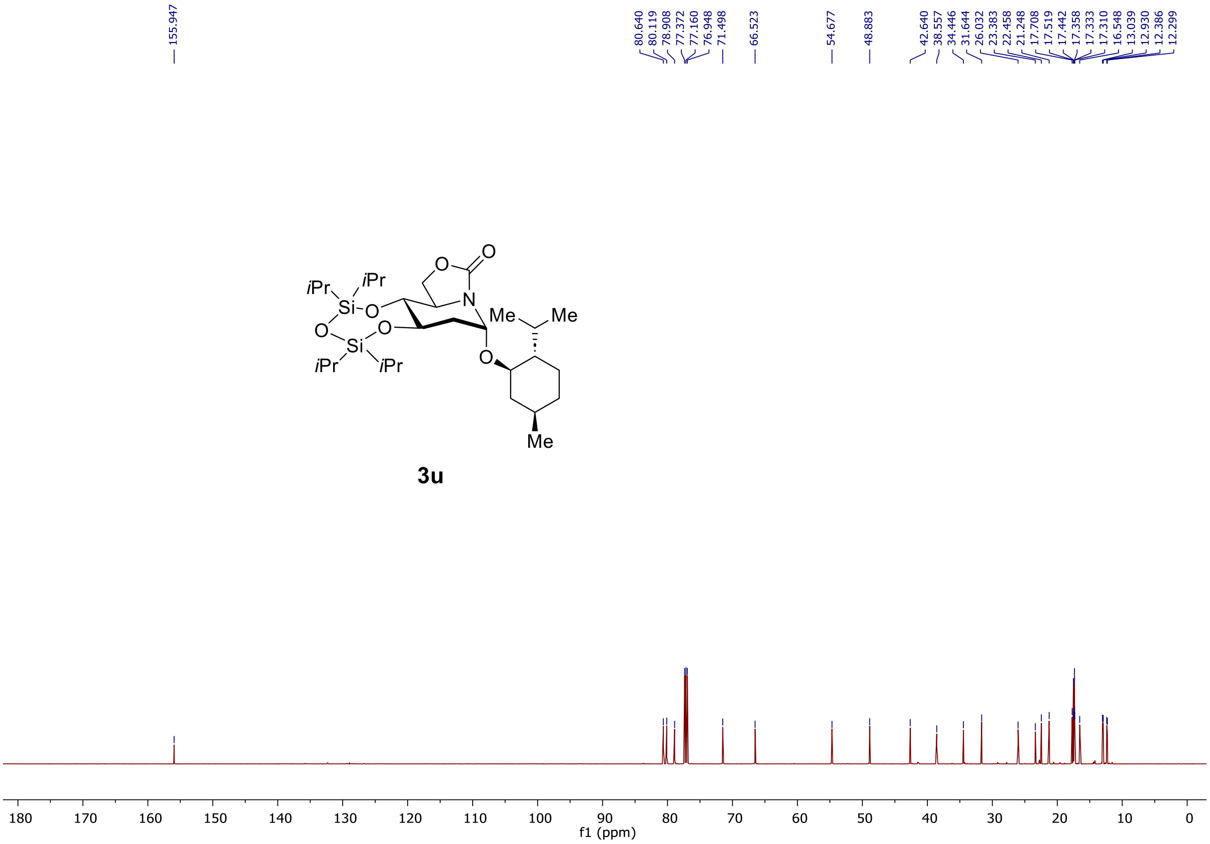

Supplementary Figure S314. ^{13}C NMR spectra for 3u

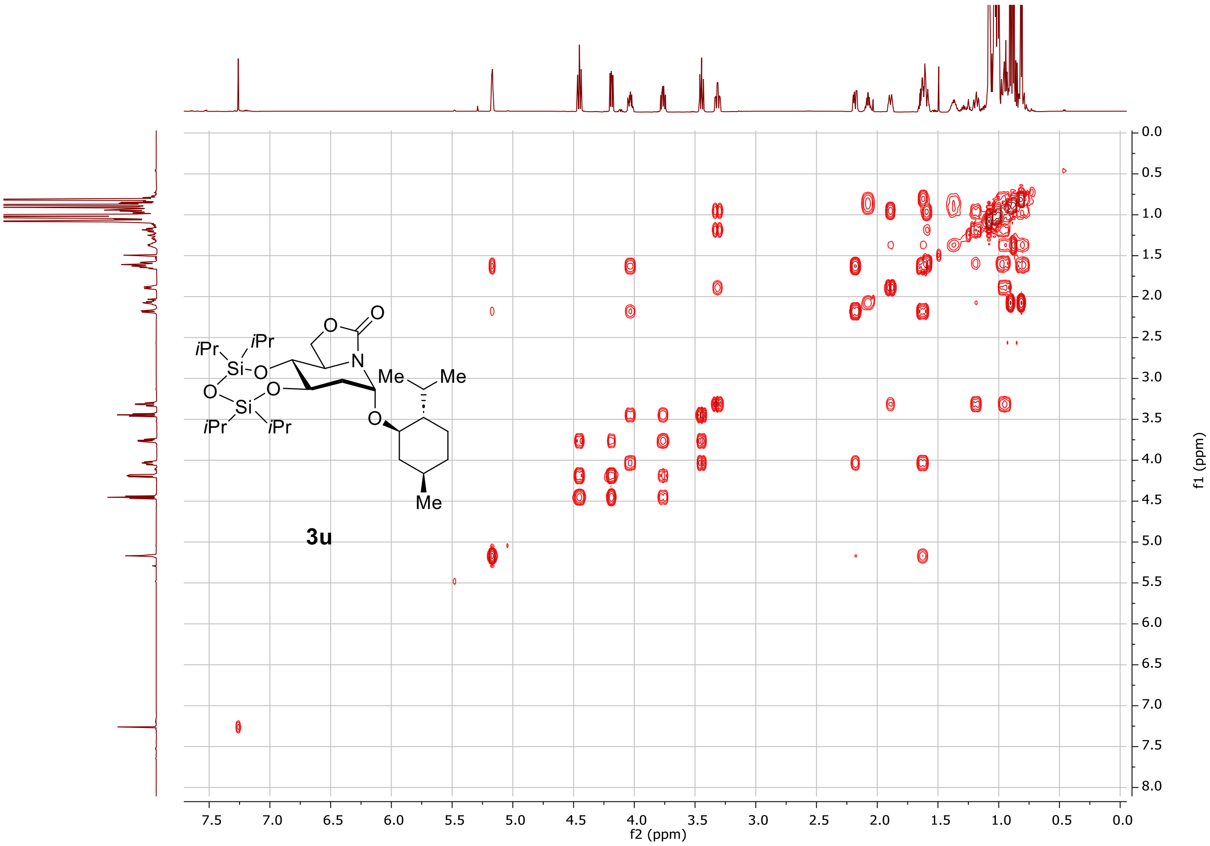

Supplementary Figure S315. COSY spectra for 3u

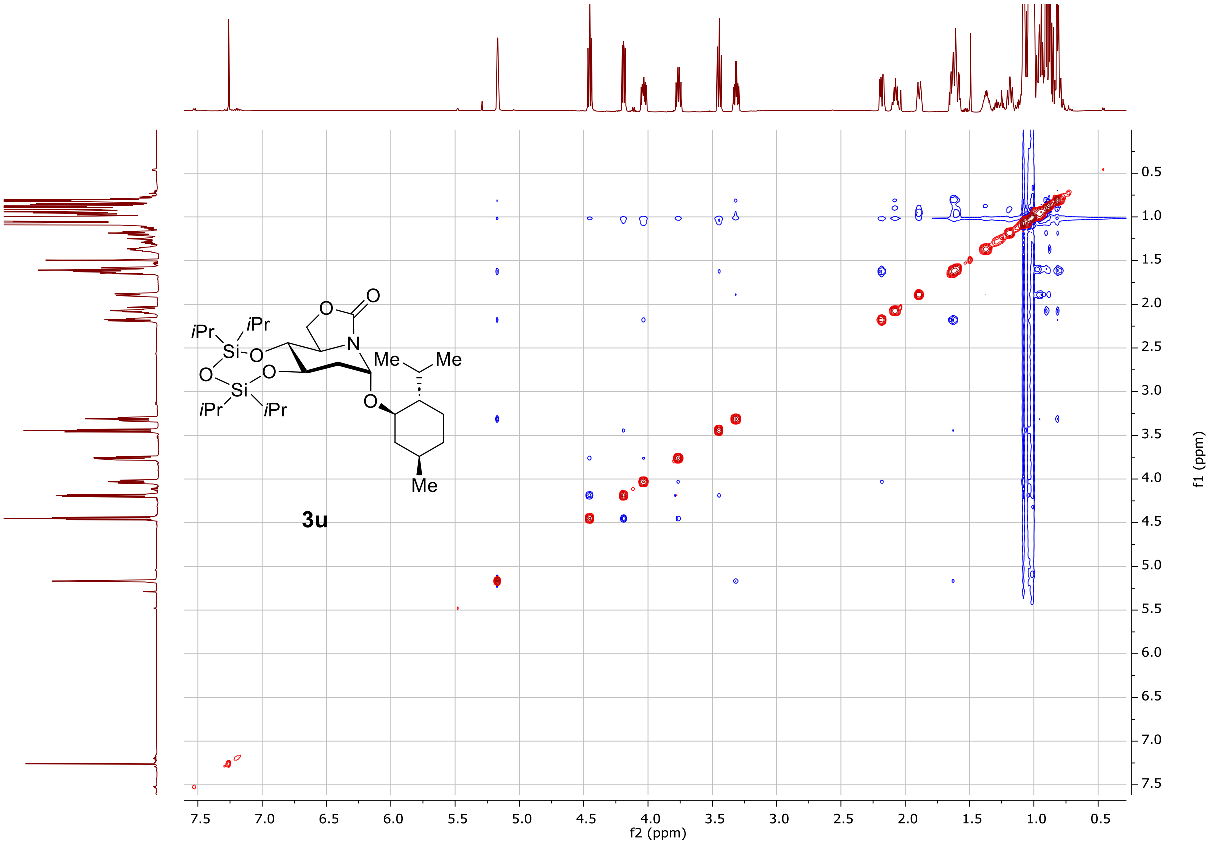

Supplementary Figure S316. NOESY spectra for 3u

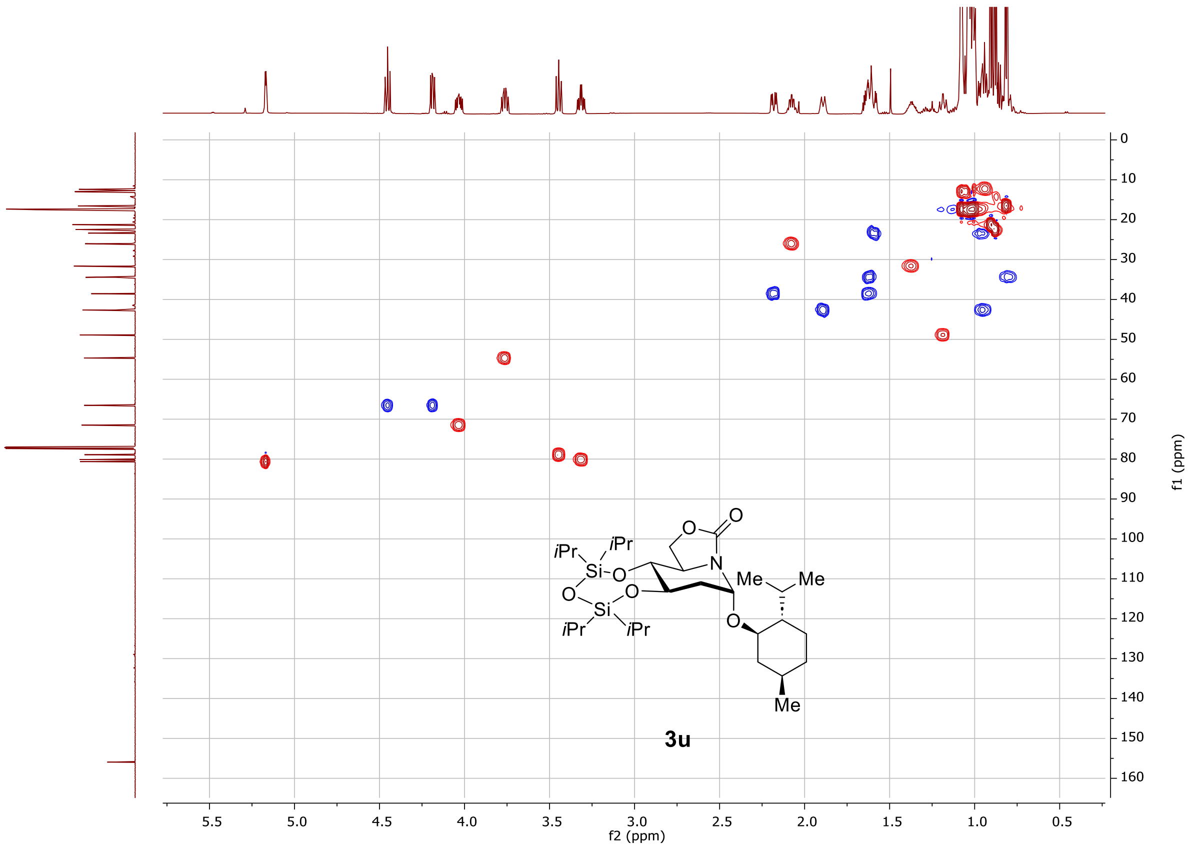

Supplementary Figure S317. HSQC spectra for 3u

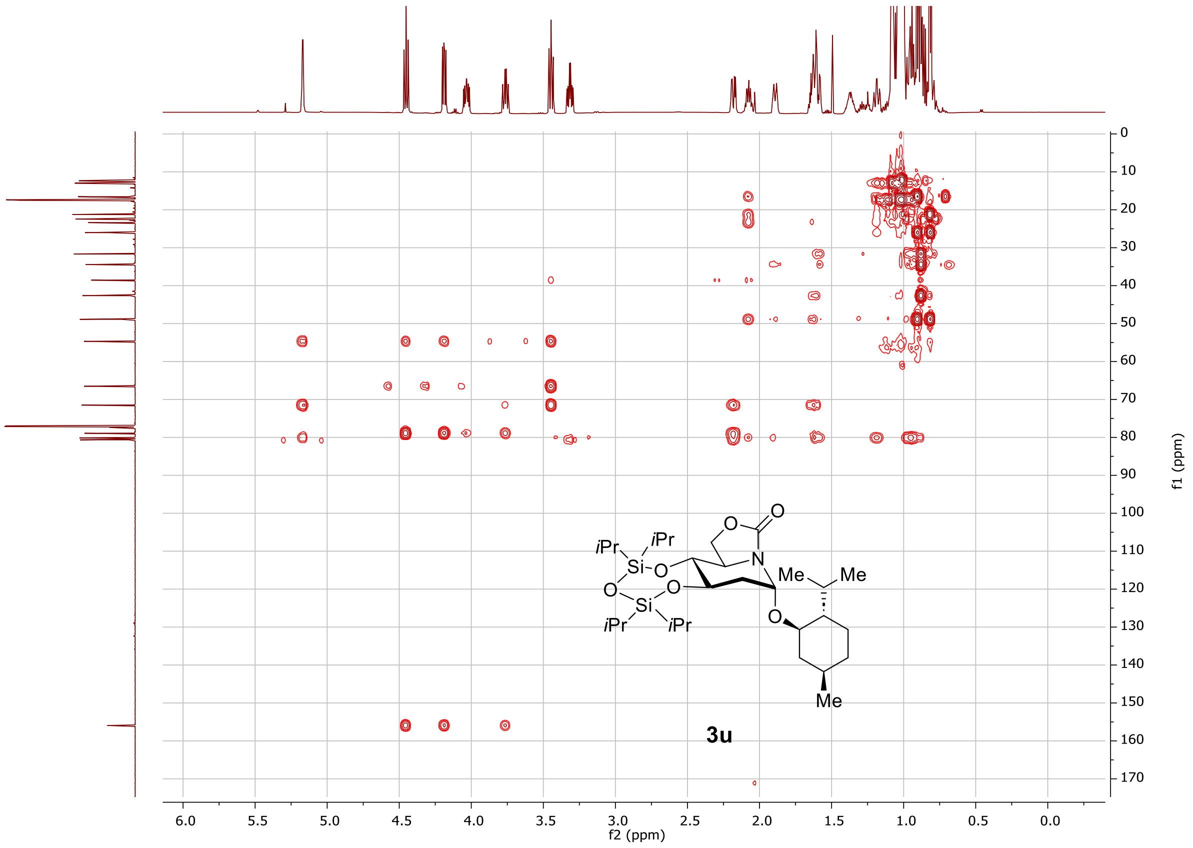

Supplementary Figure S318. HMBC spectra for 3u

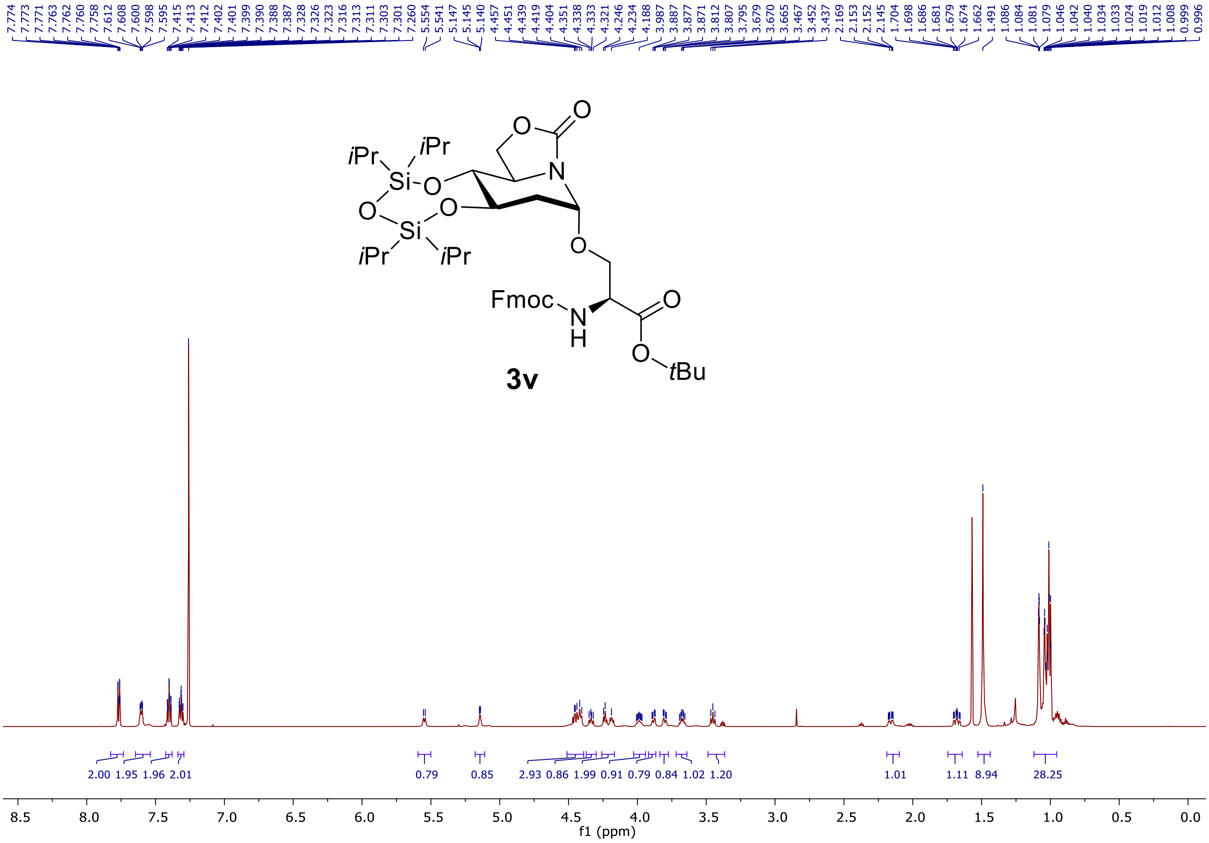

Supplementary Figure S319. ^{1}H NMR spectra for 3v

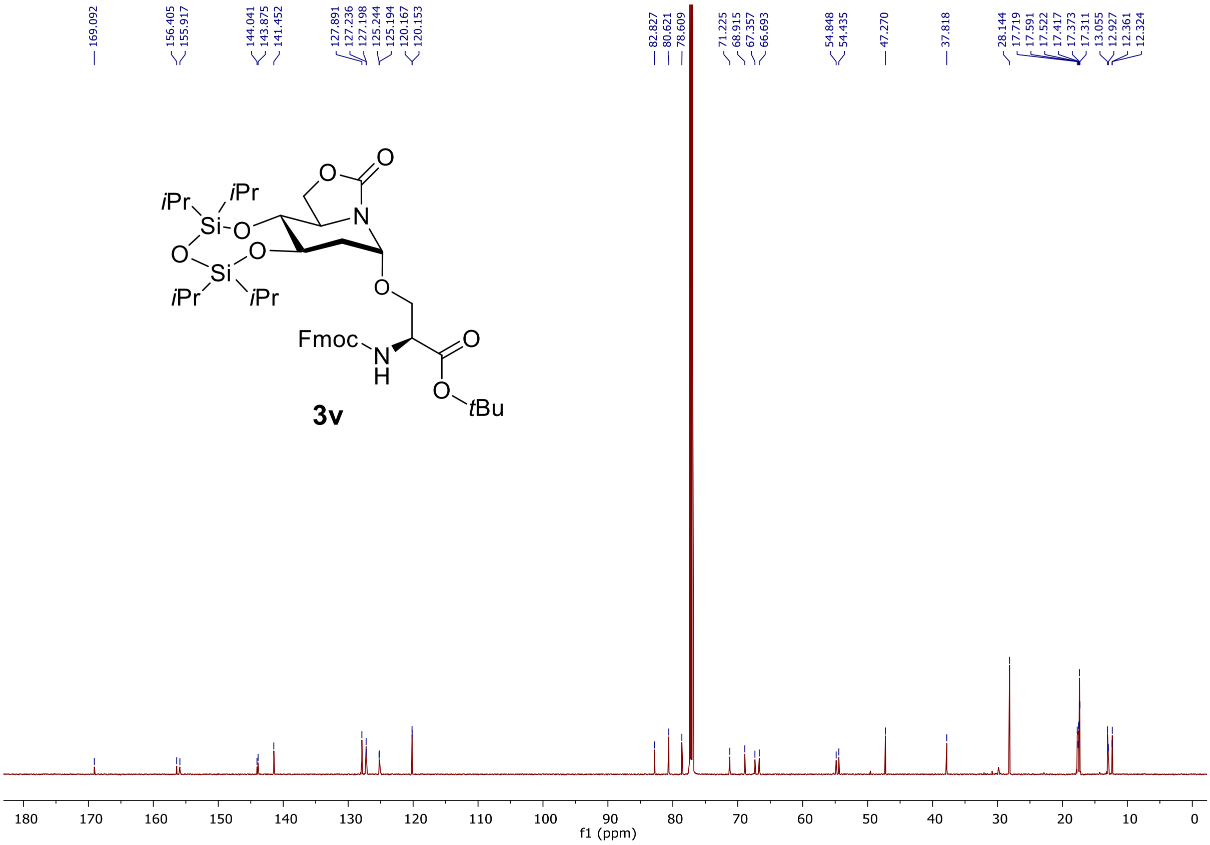

Supplementary Figure S320. ^{13}C NMR spectra for 3v

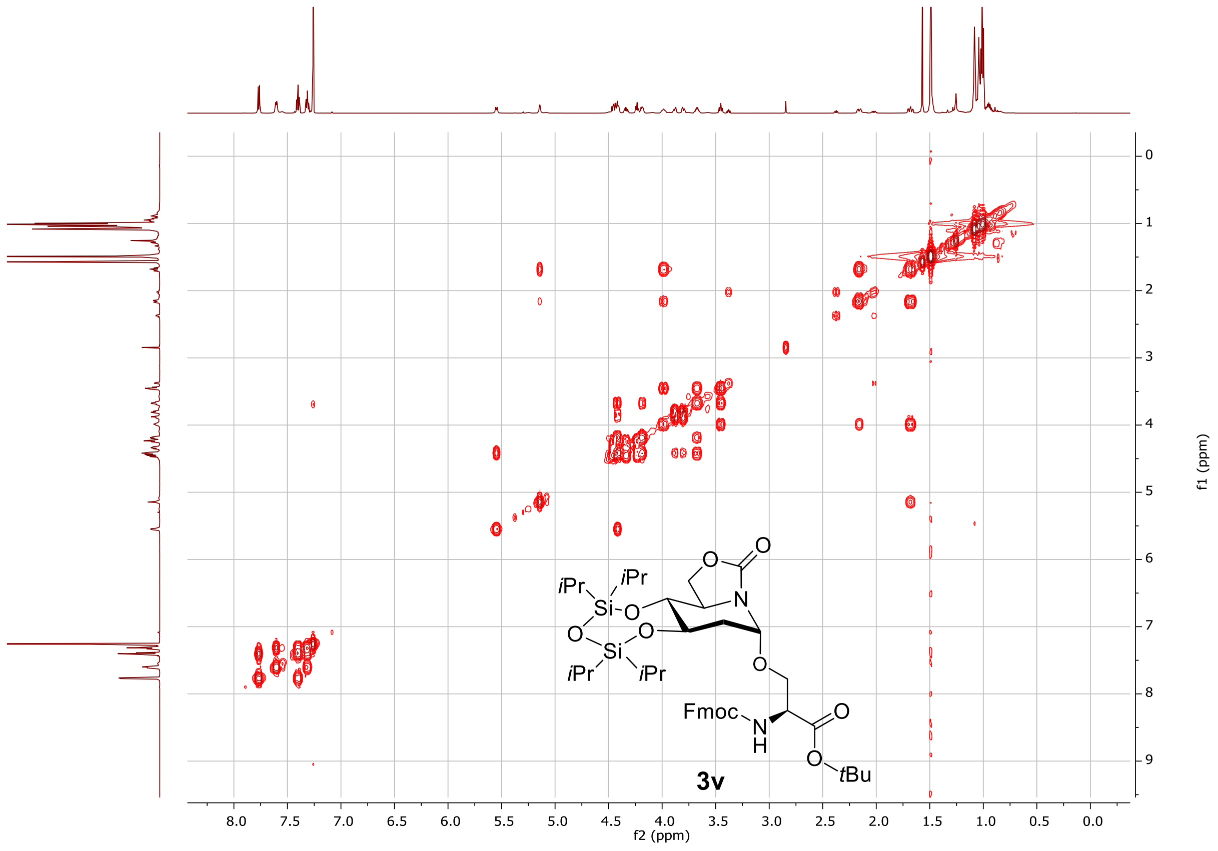

Supplementary Figure S321. COSY spectra for 3v

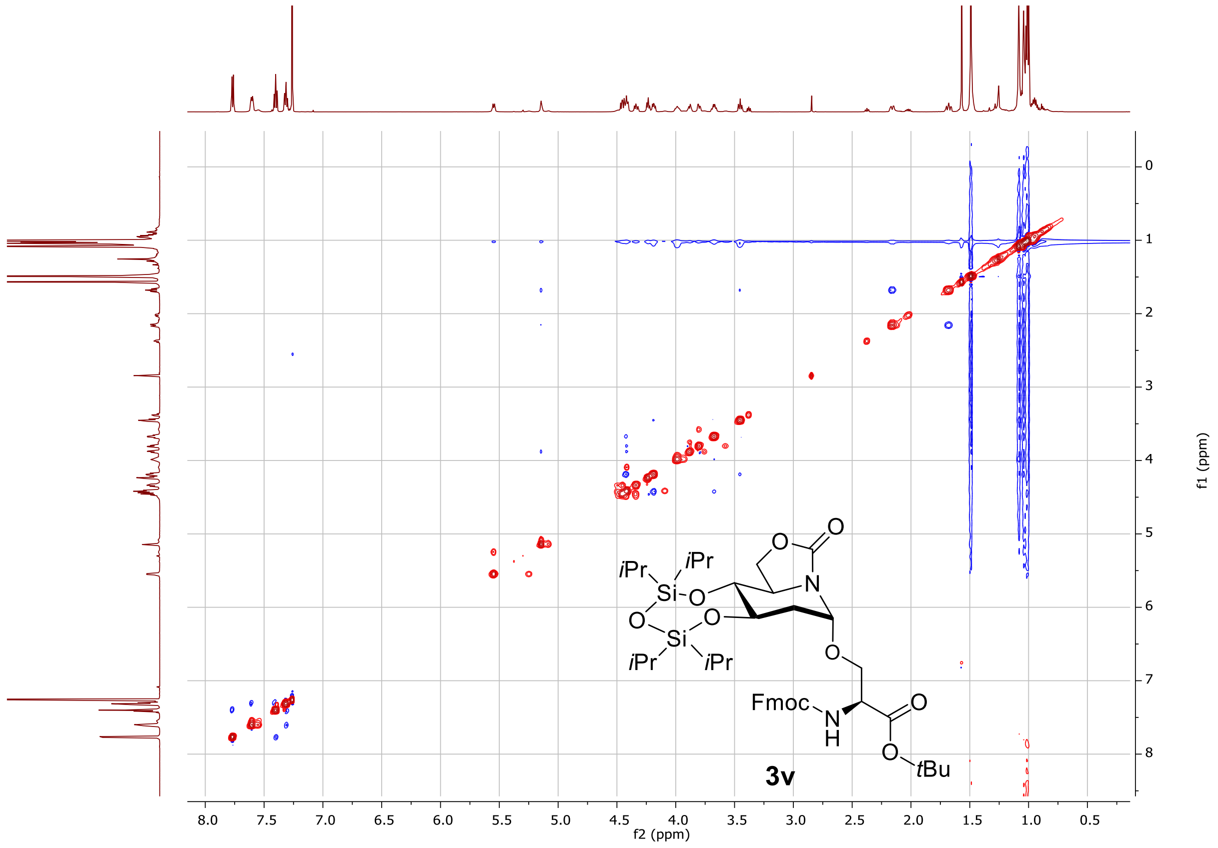

Supplementary Figure S322. NOESY spectra for 3v

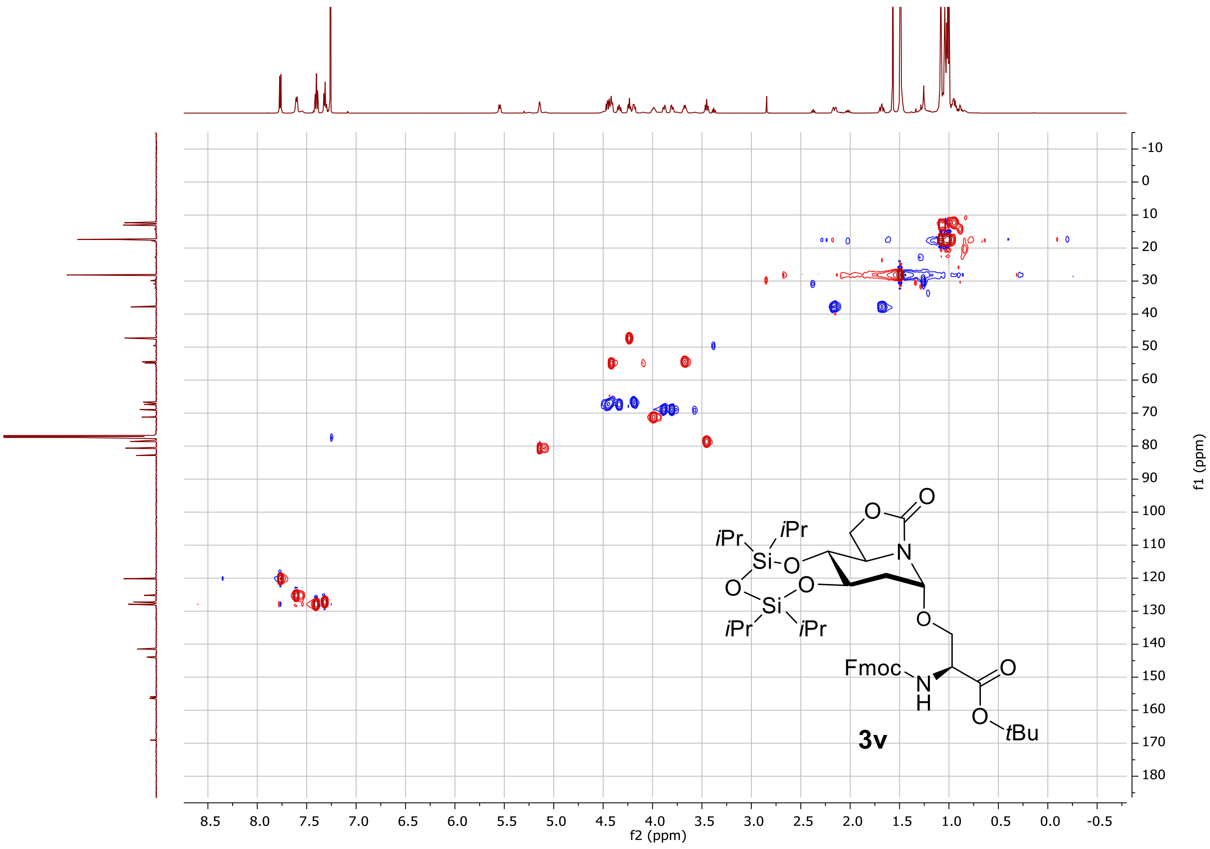

Supplementary Figure S323. HSQC spectra for 3v

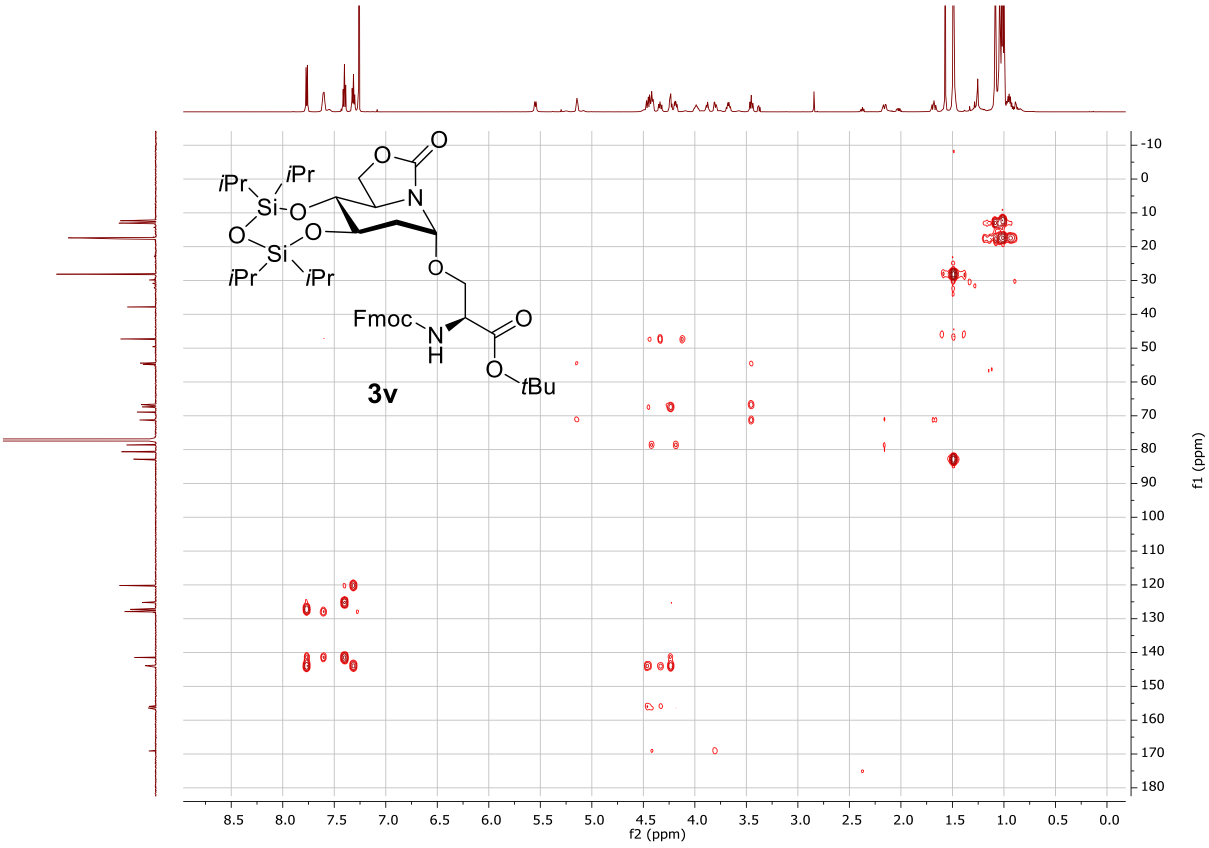

Supplementary Figure S324. HMBC spectra for 3v

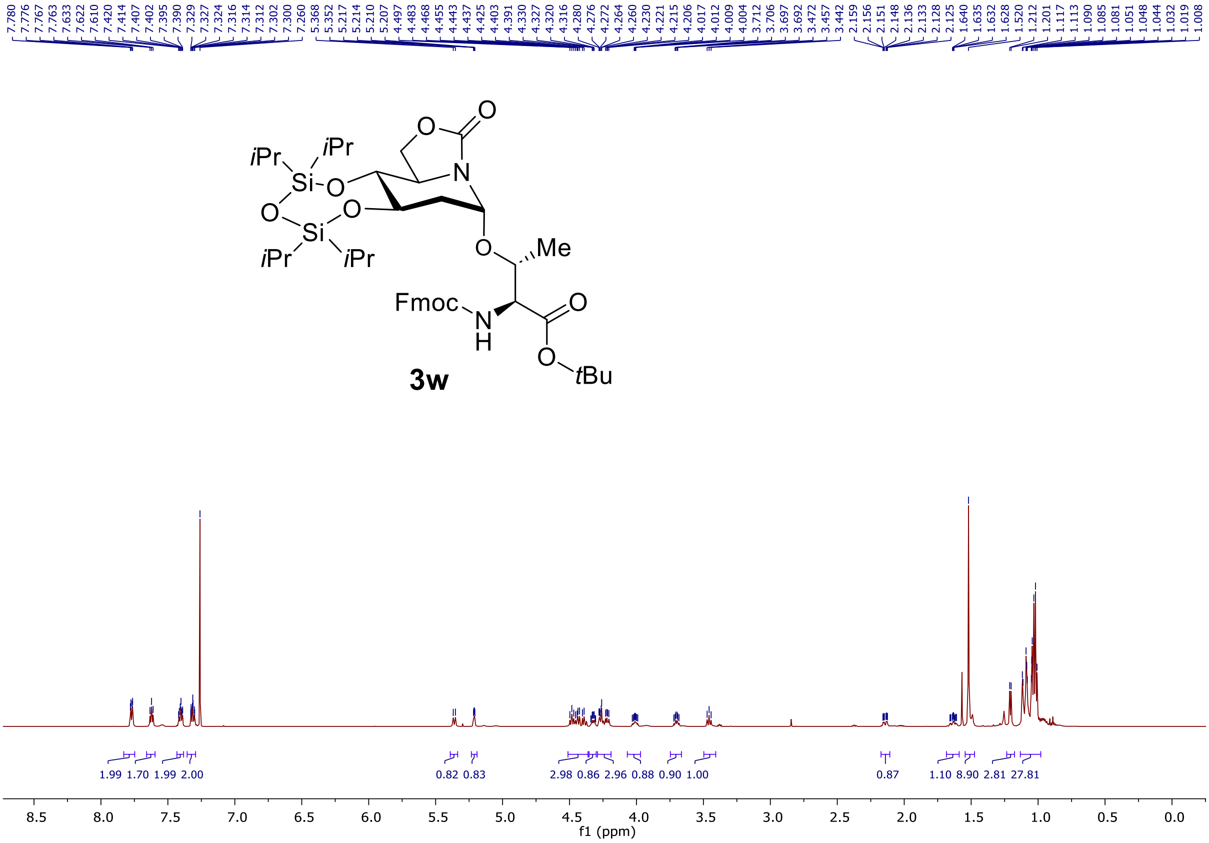

Supplementary Figure S325. ^{1}H NMR spectra for 3w

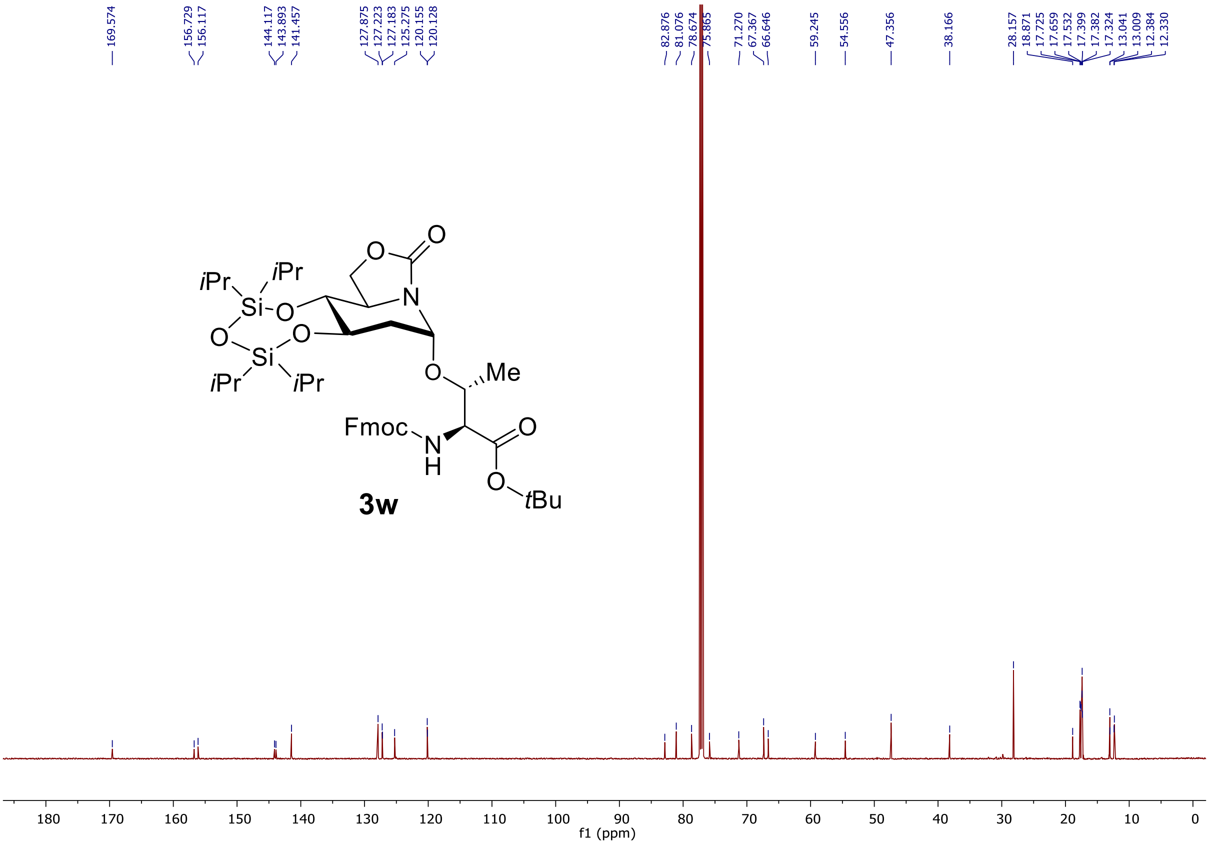

Supplementary Figure S326. ^{13}C NMR spectra for 3w

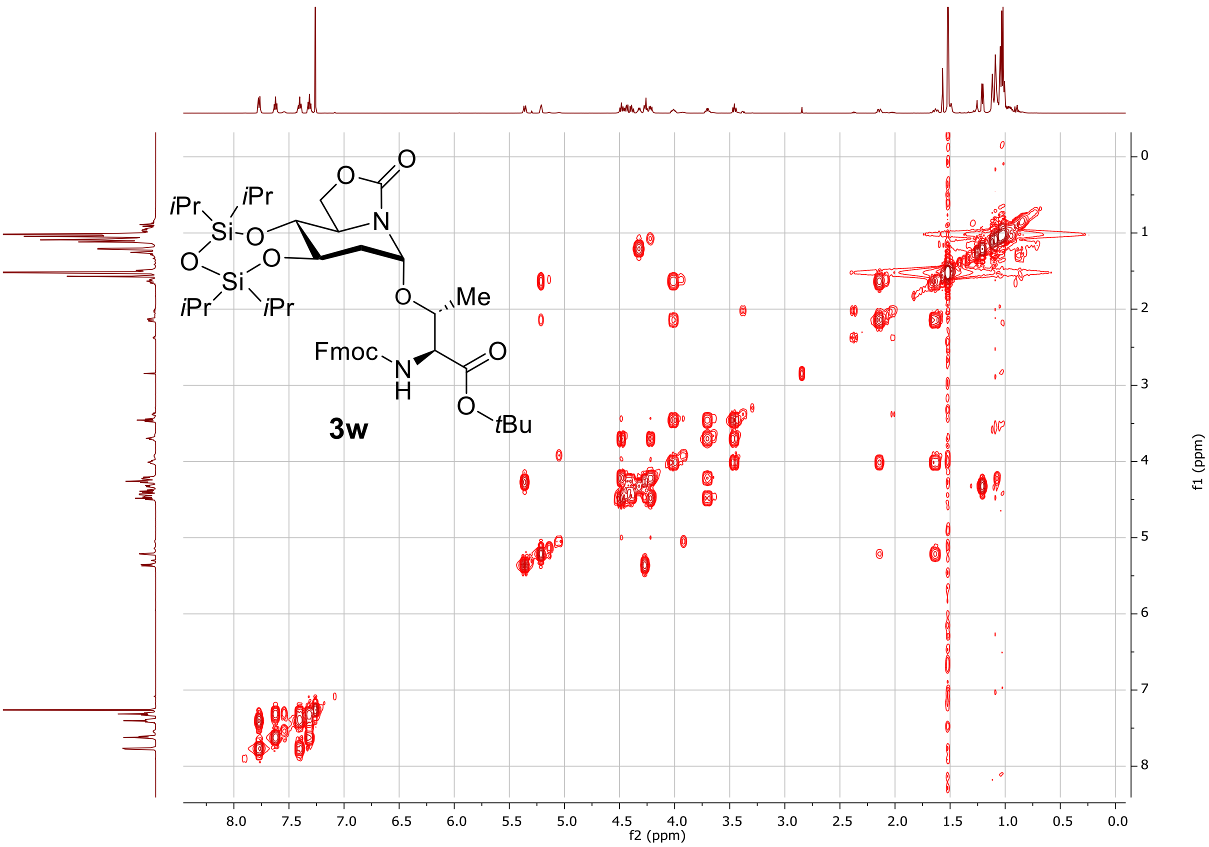

Supplementary Figure S327. COSY spectra for 3w

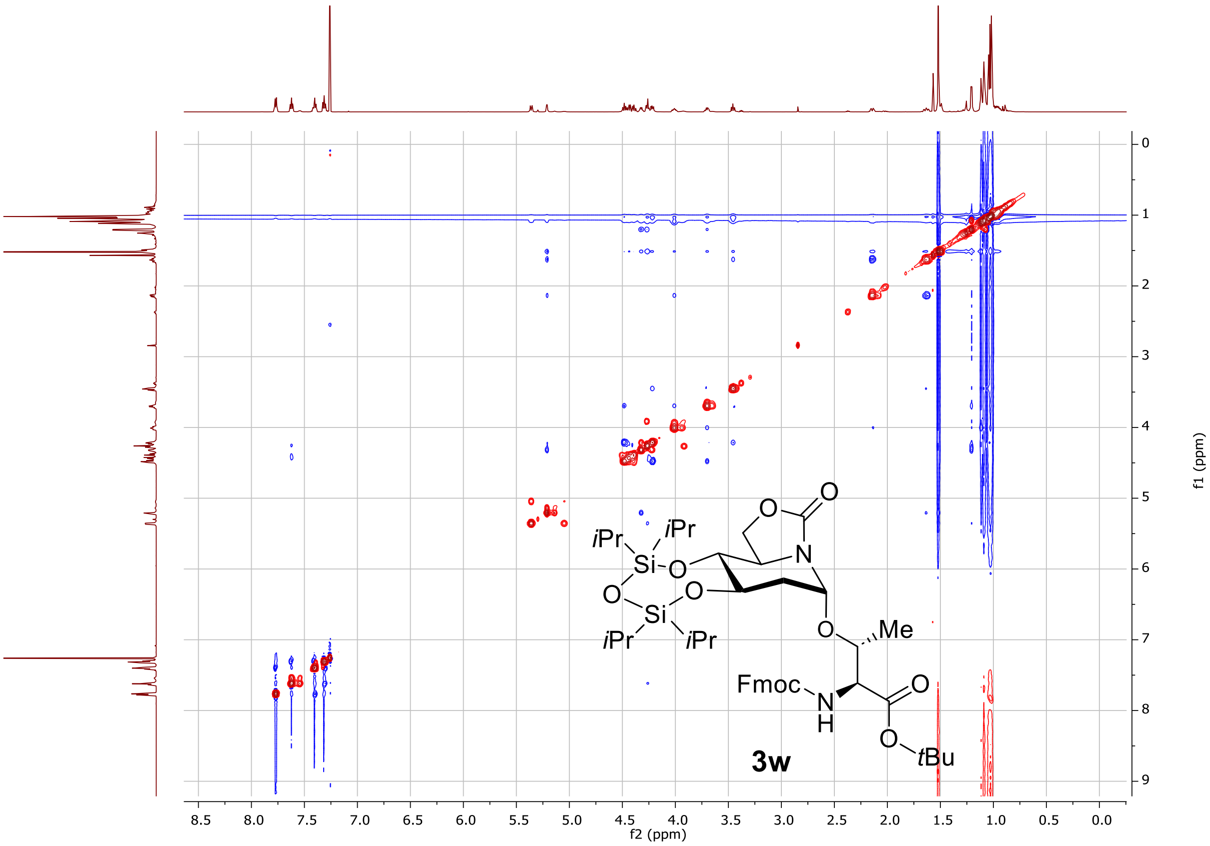

Supplementary Figure S328. NOESY spectra for 3w

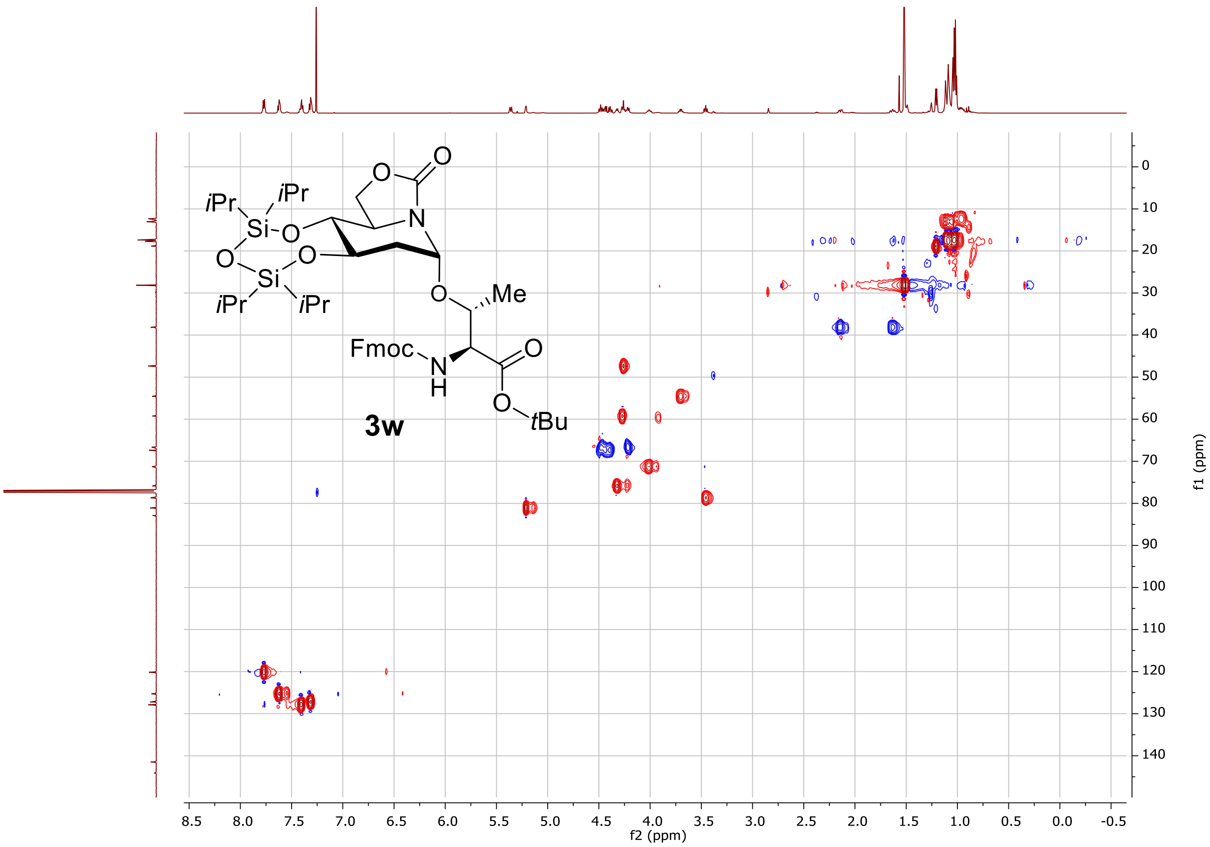

Supplementary Figure S329. HSQC spectra for 3w

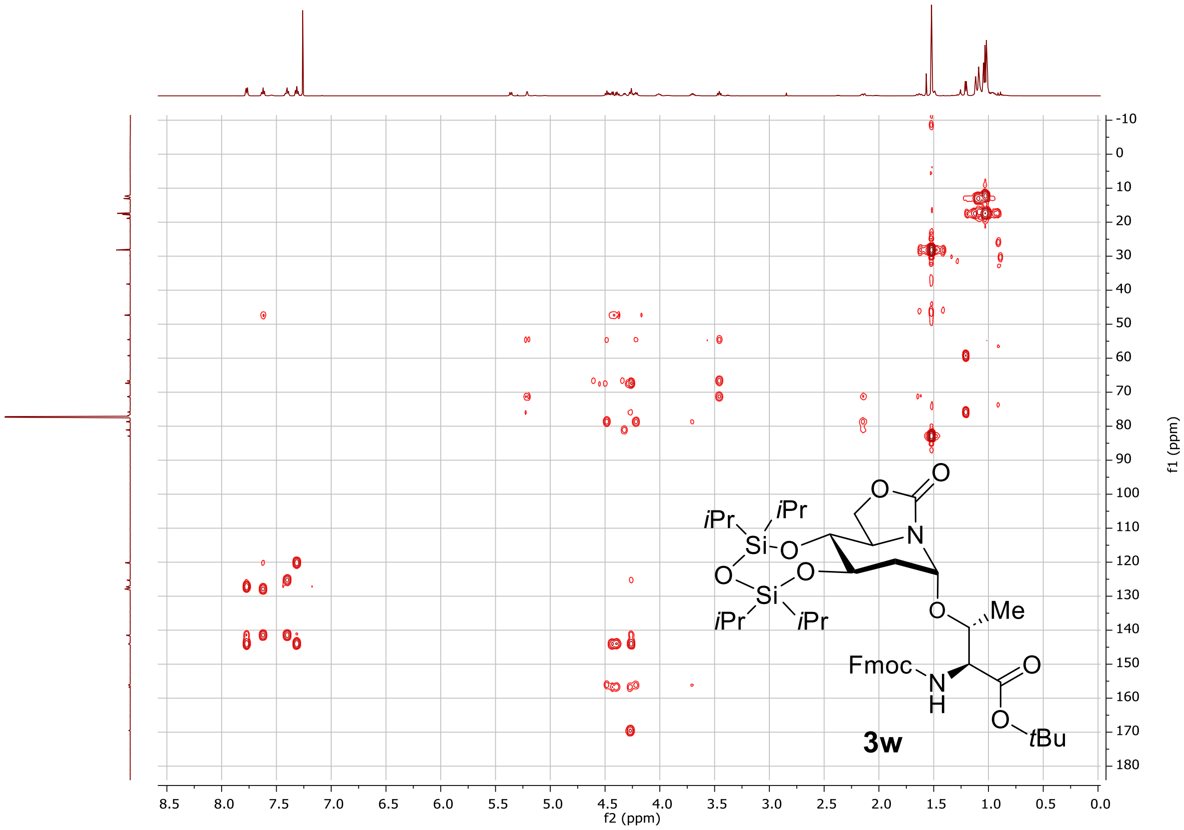

Supplementary Figure S330. HMBC spectra for 3w

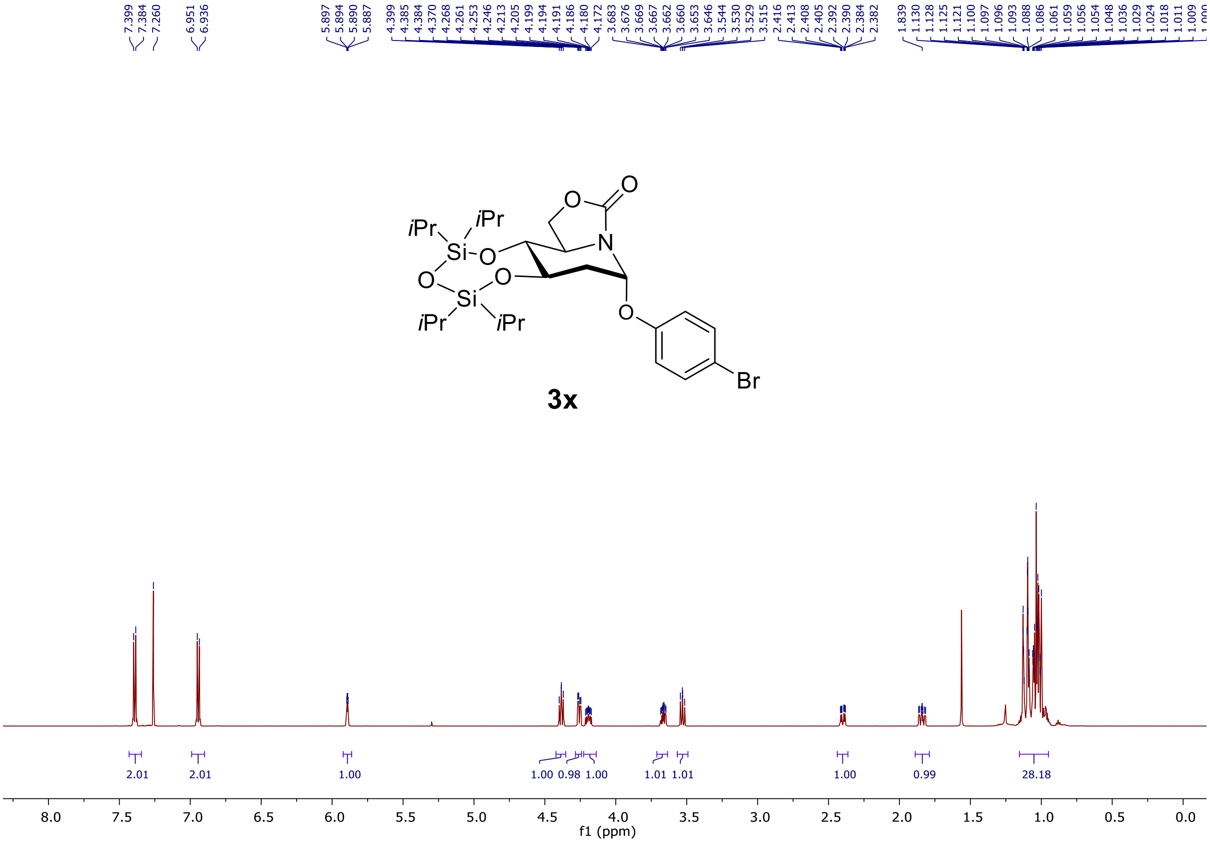

Supplementary Figure S331. ^{1}H NMR spectra for 3x

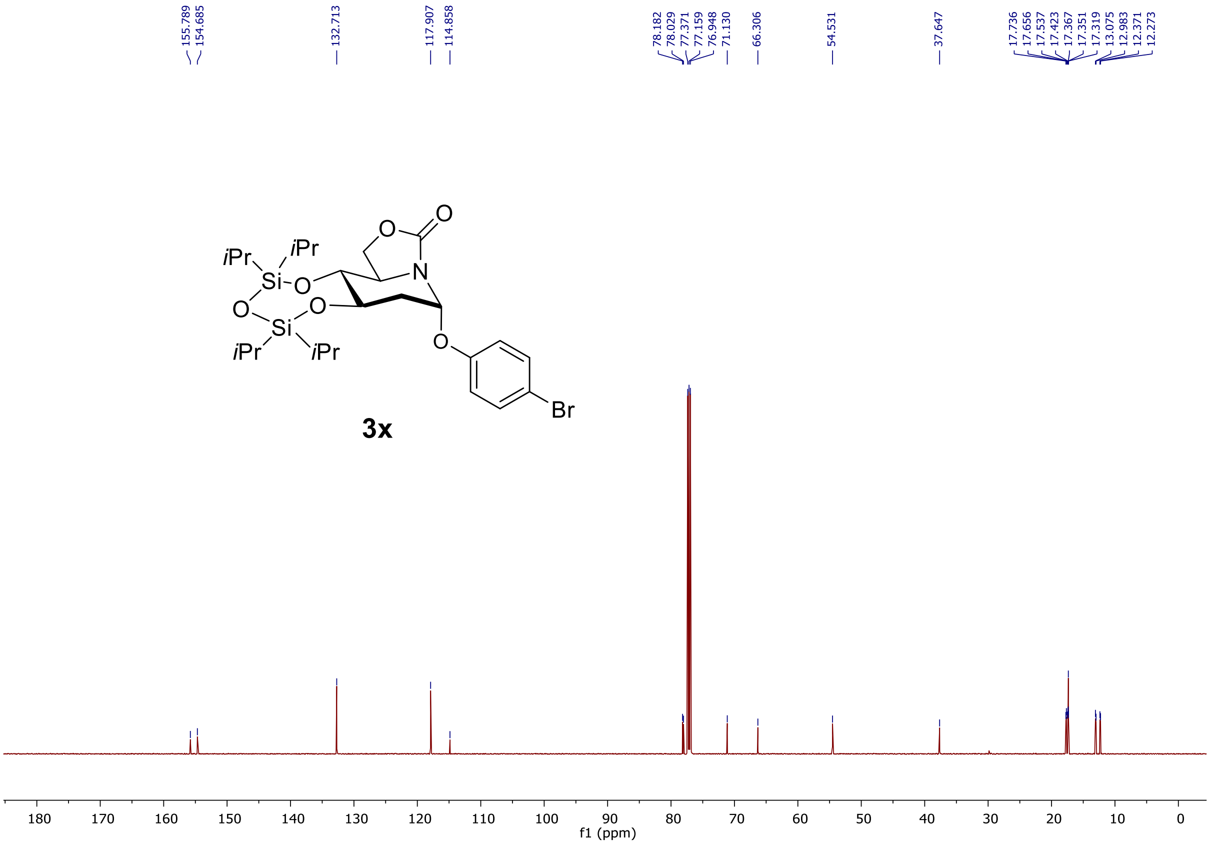

Supplementary Figure S332. ^{13}C NMR spectra for 3x

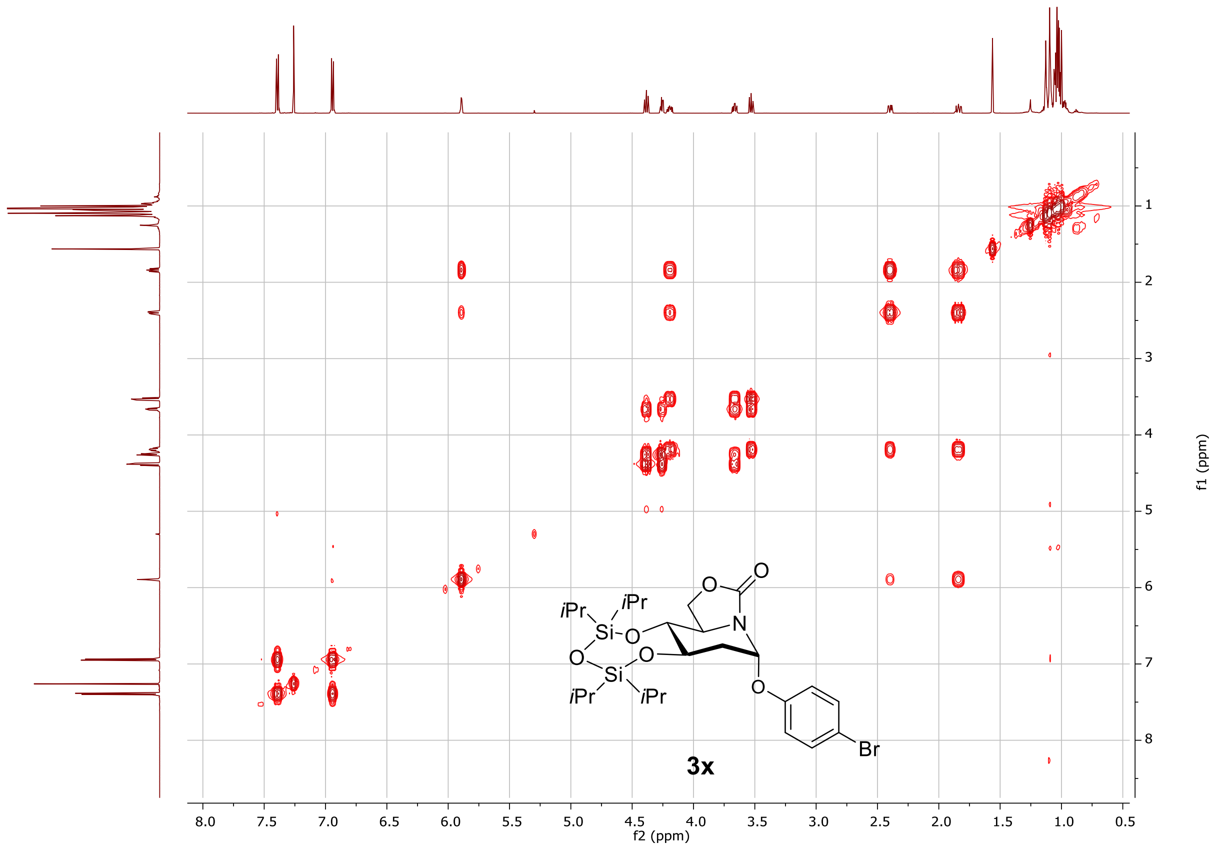

Supplementary Figure S333. COSY spectra for 3x

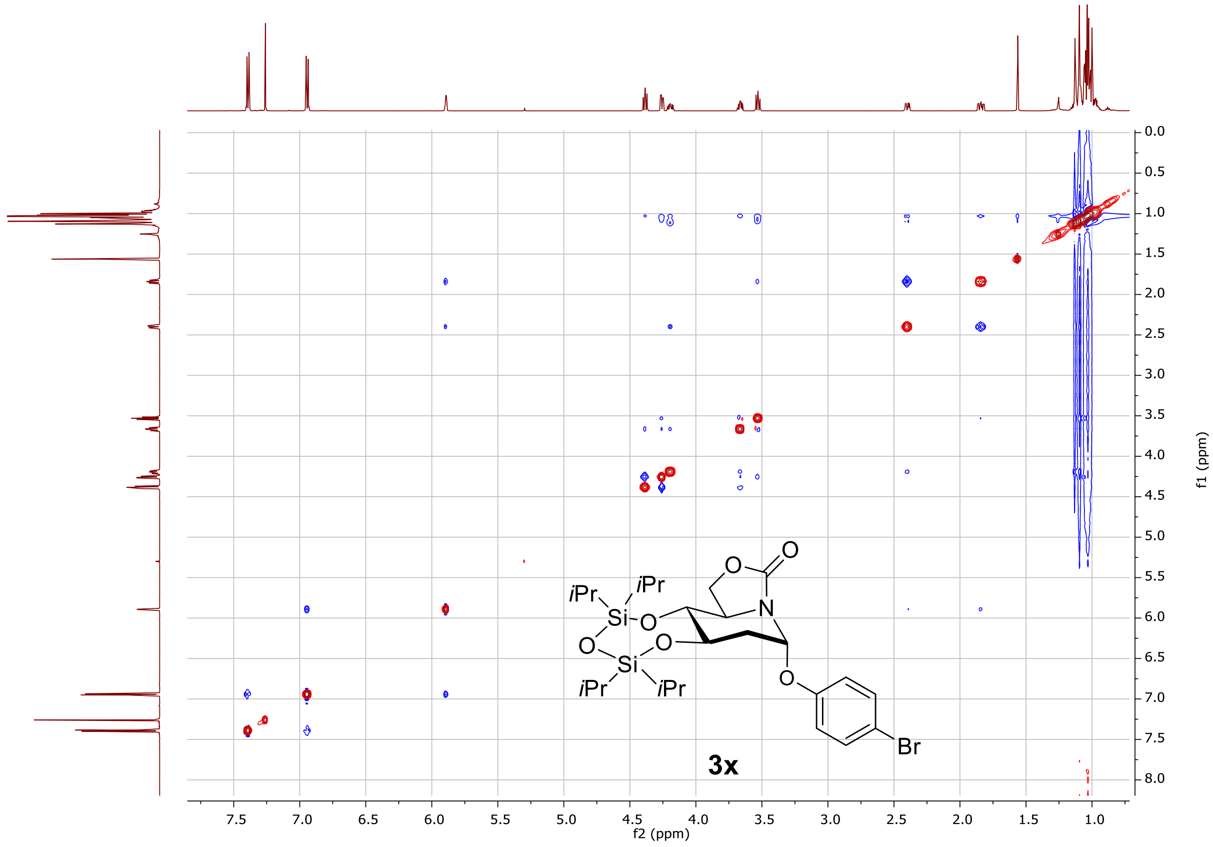

Supplementary Figure S334. NOESY spectra for 3x

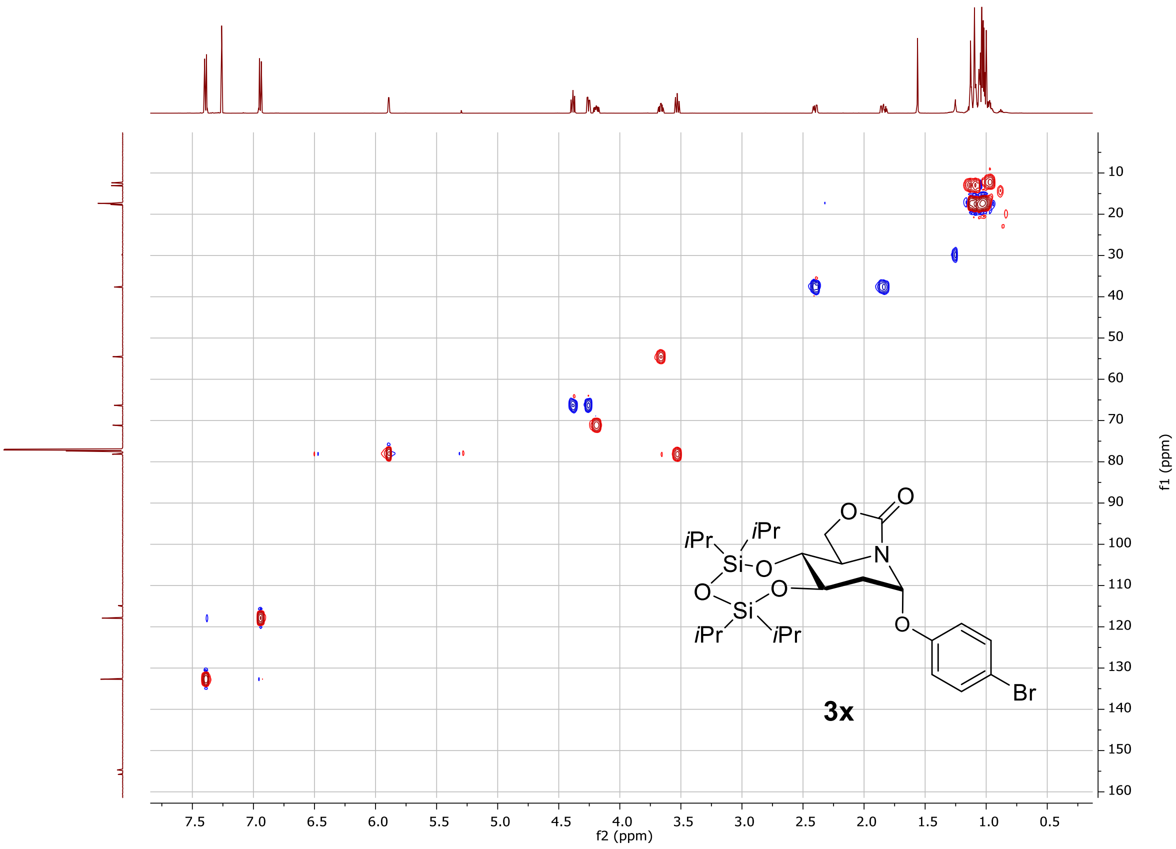

Supplementary Figure S335. HSQC spectra for 3x

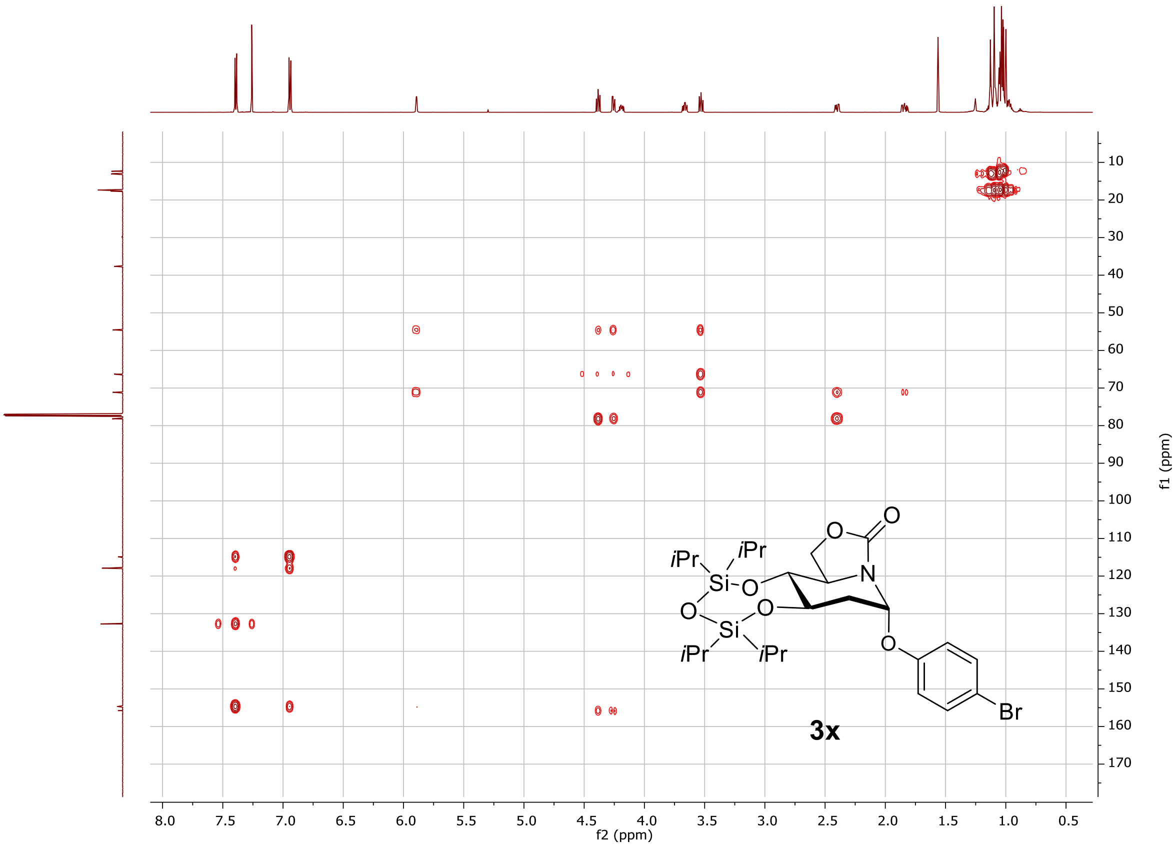

Supplementary Figure S336. HMBC spectra for 3x

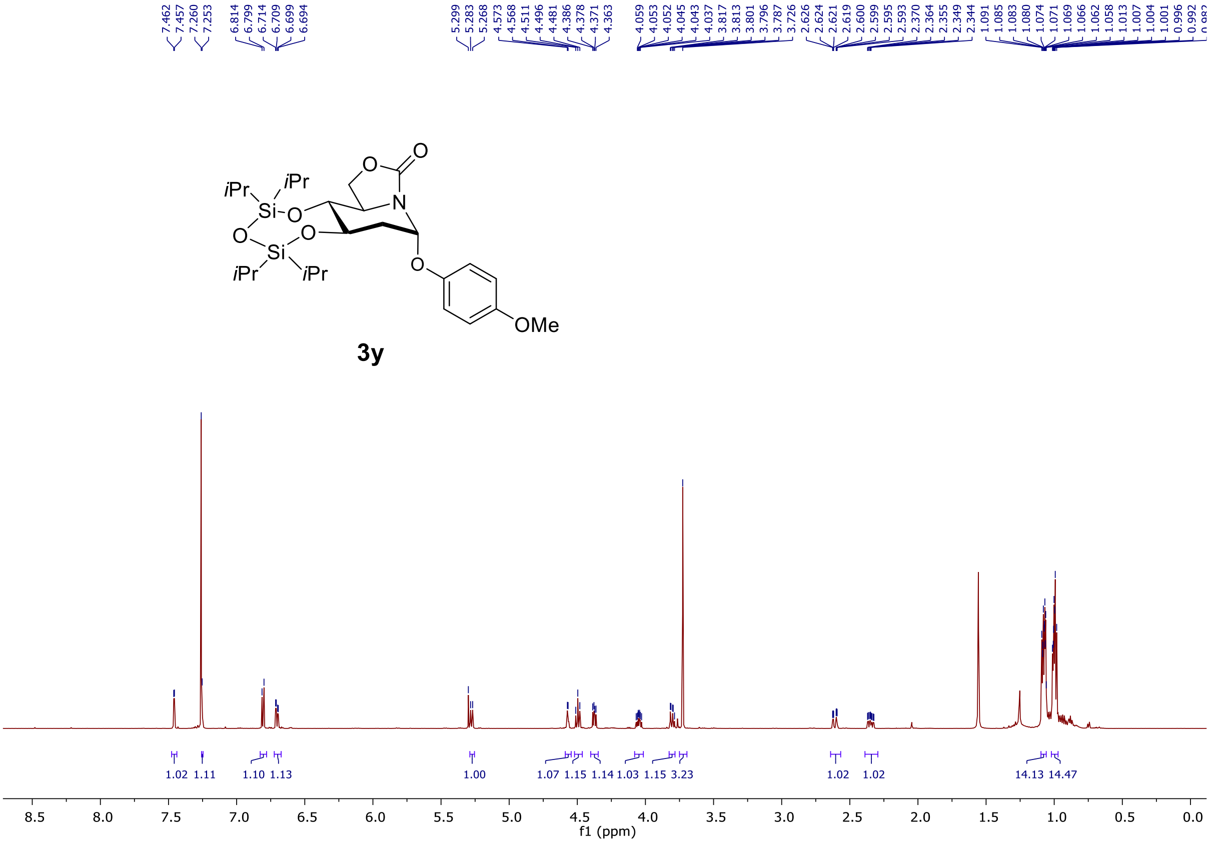

Supplementary Figure S337. ^{1}H NMR spectra for 3y

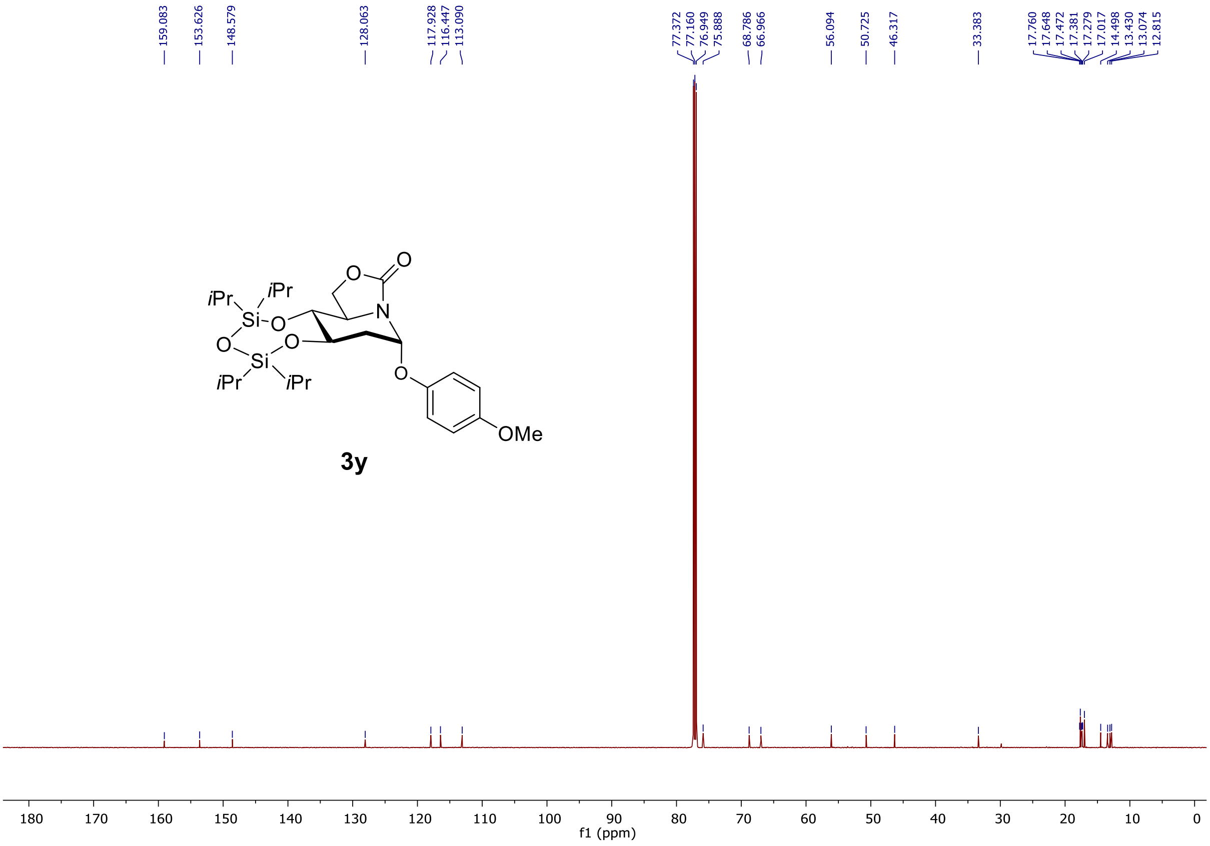

Supplementary Figure S338. ^{13}C NMR spectra for 3y

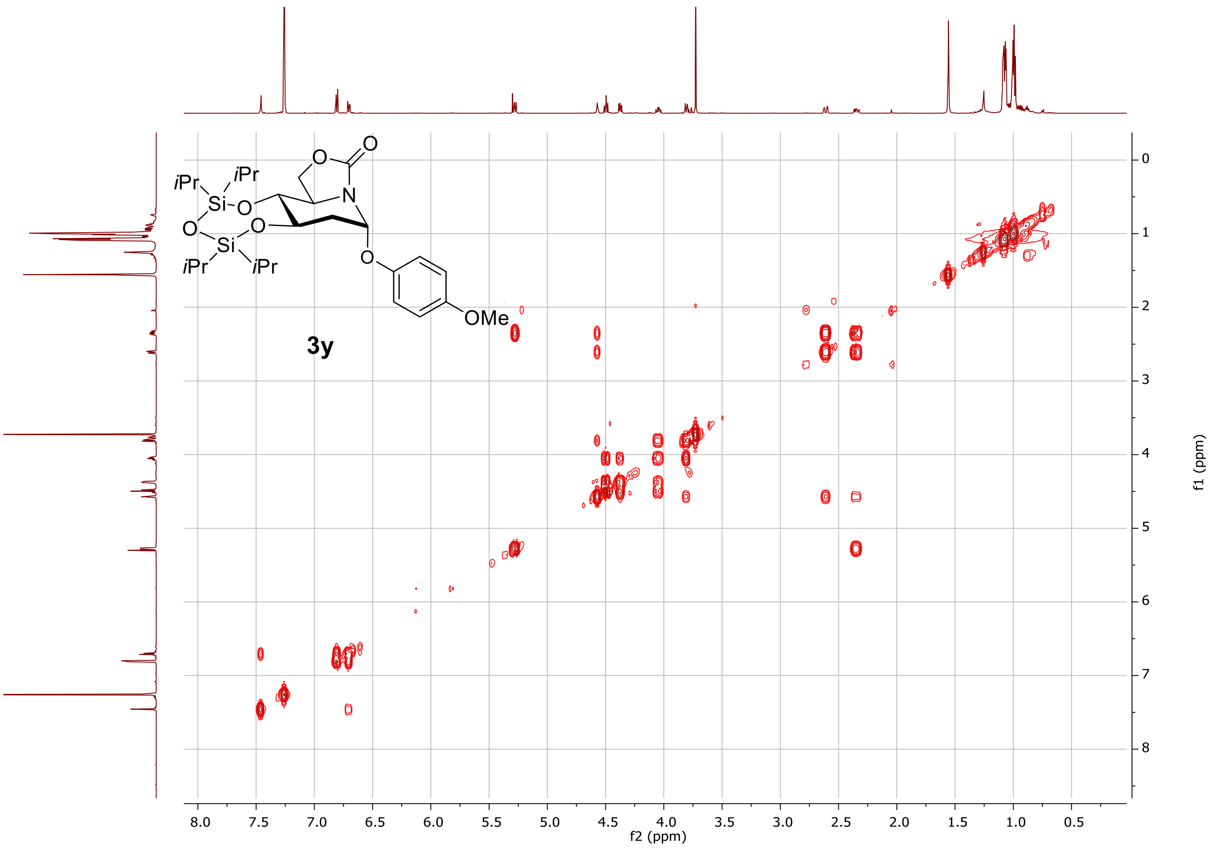

Supplementary Figure S339. COSY spectra for 3y

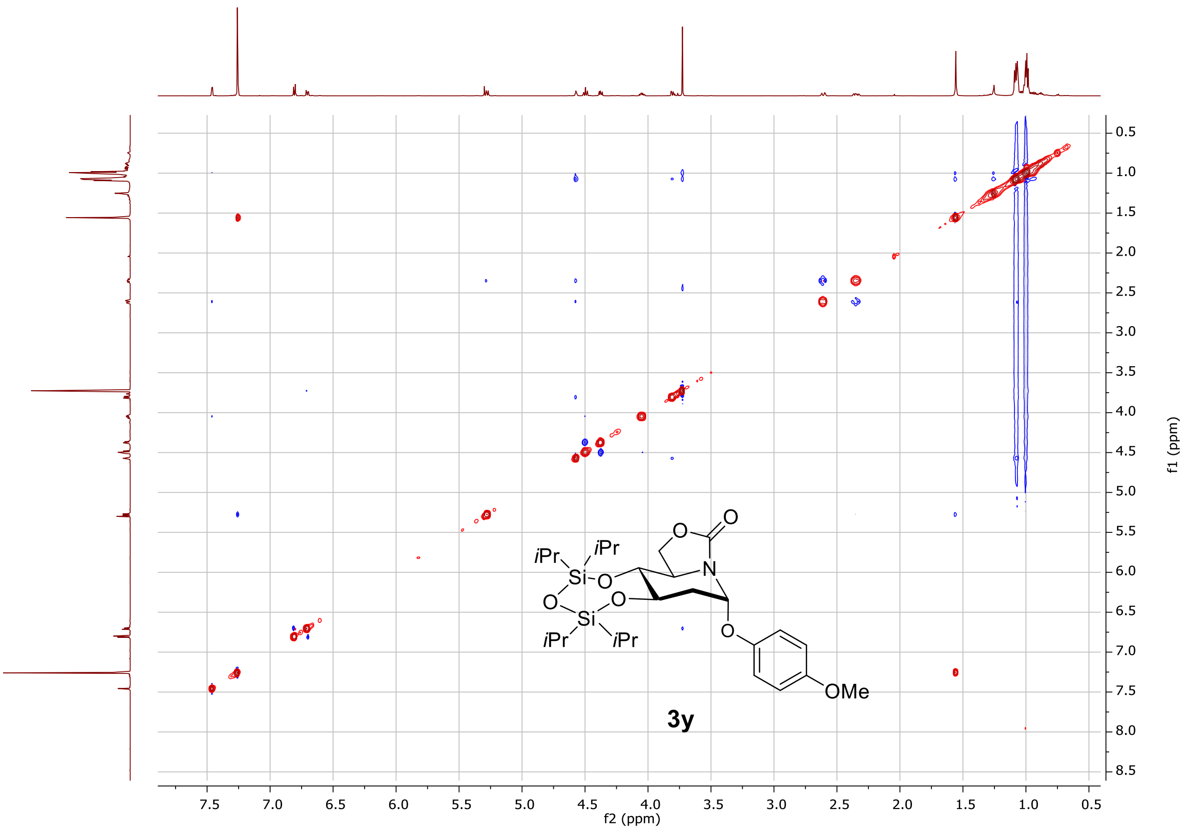

Supplementary Figure S340. NOESY spectra for 3y

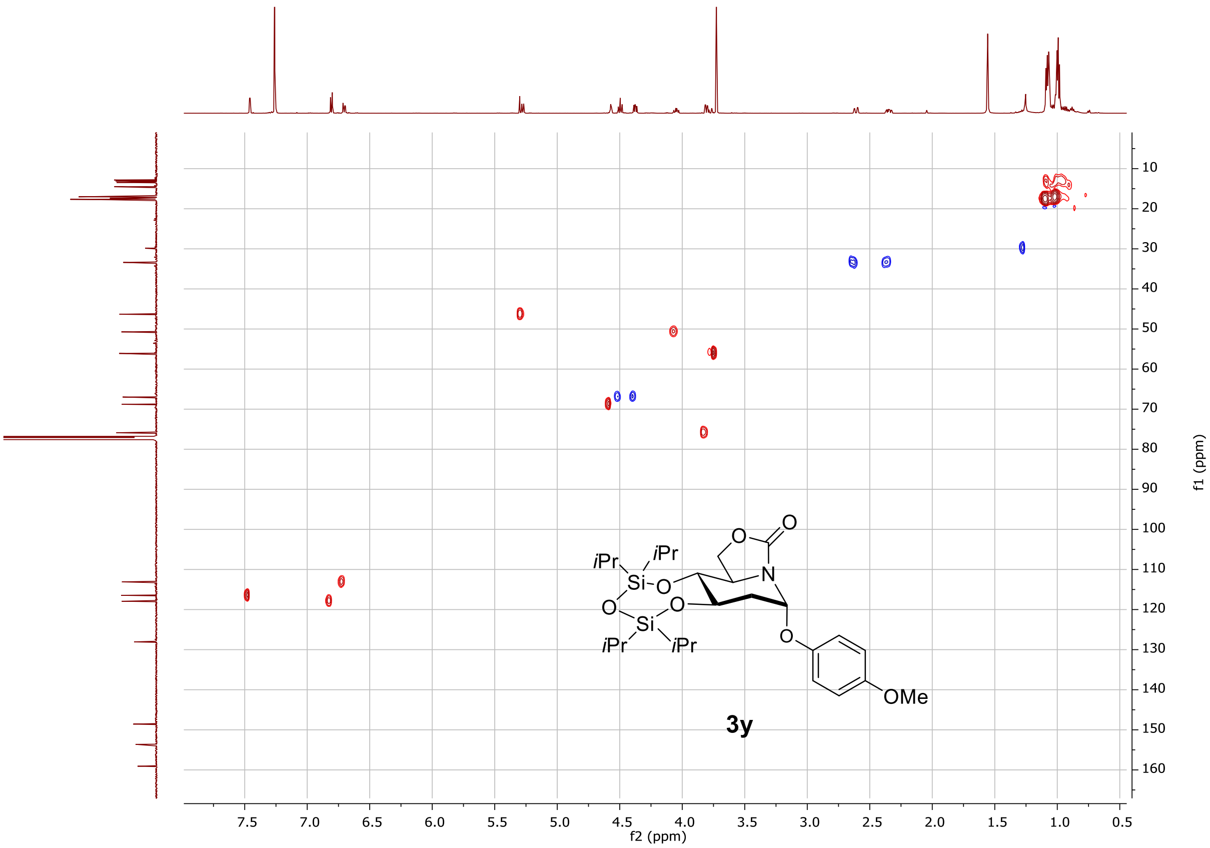

Supplementary Figure S341. HSQC spectra for 3y

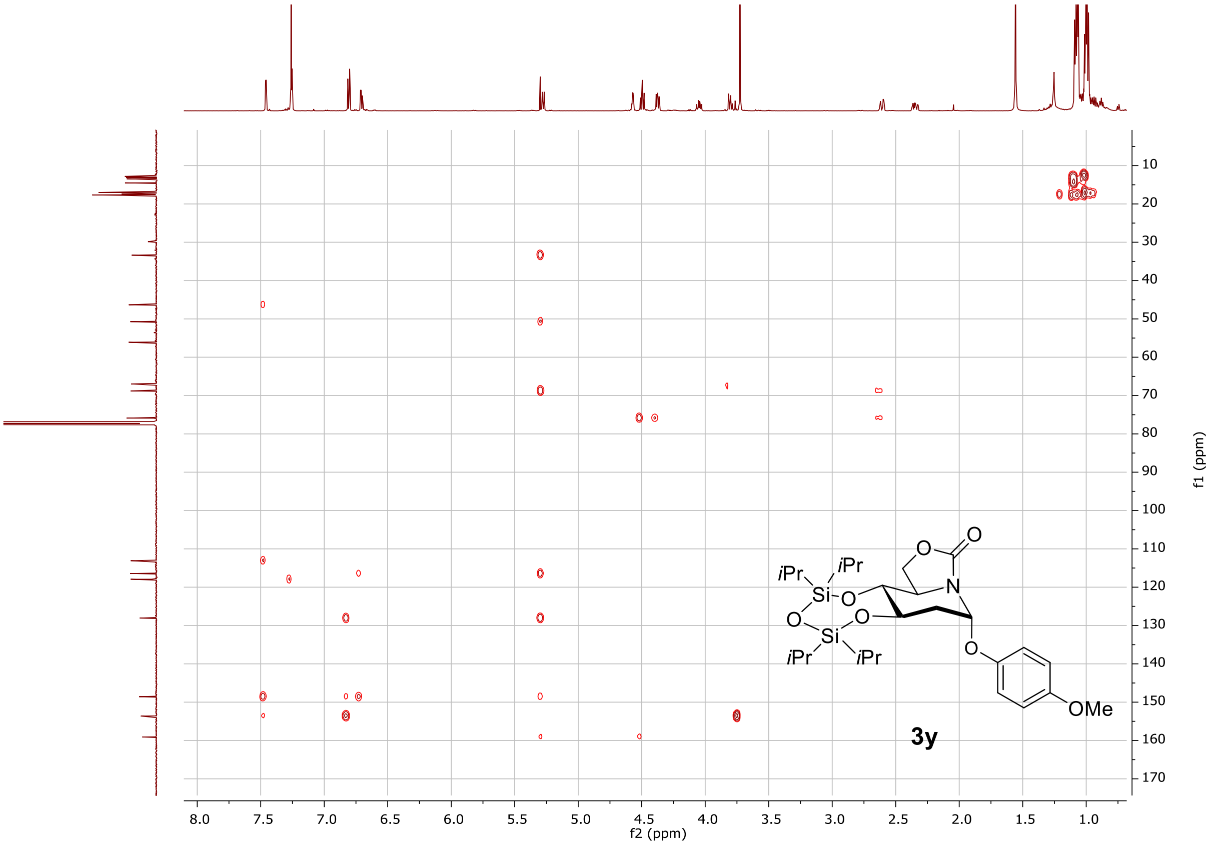

Supplementary Figure S342. HMBC spectra for 3y

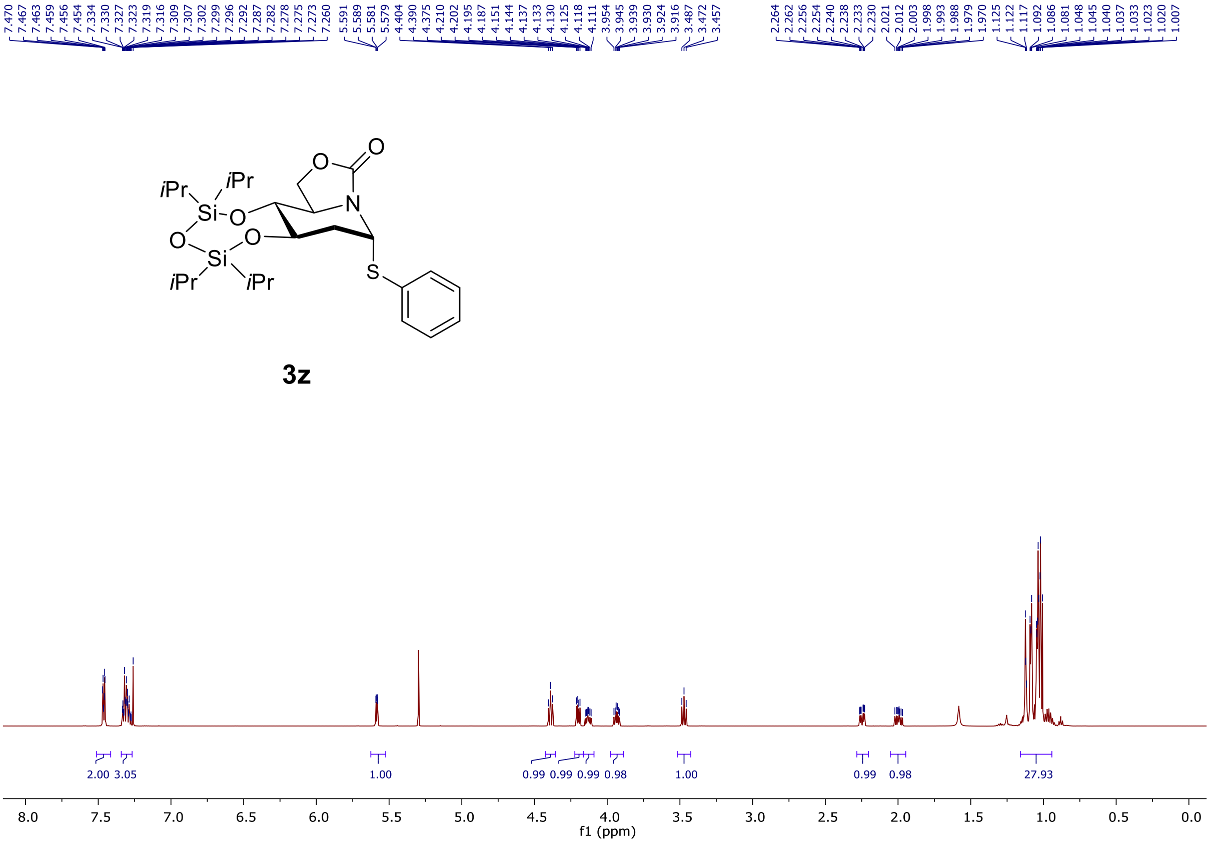

Supplementary Figure S343. ^{1}H NMR spectra for 3z

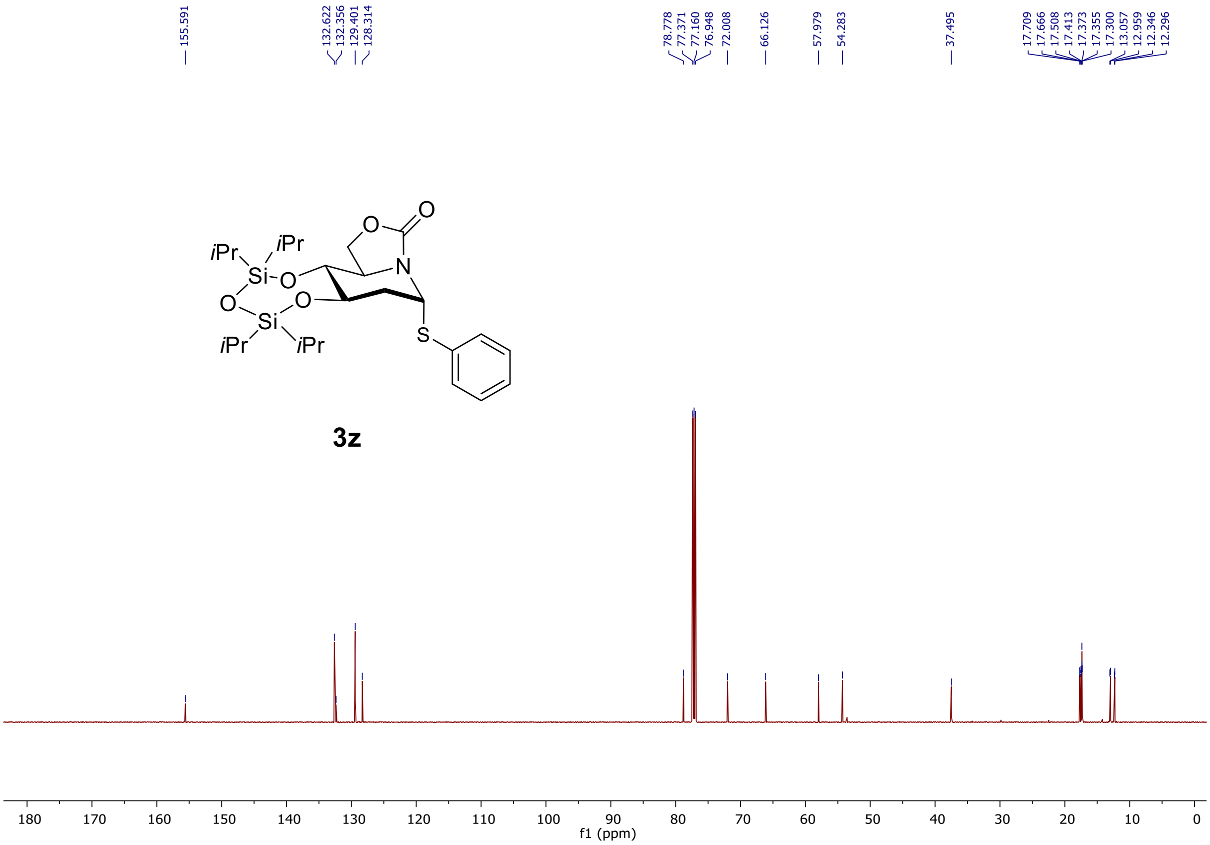

Supplementary Figure S344. ^{13}C NMR spectra for 3z

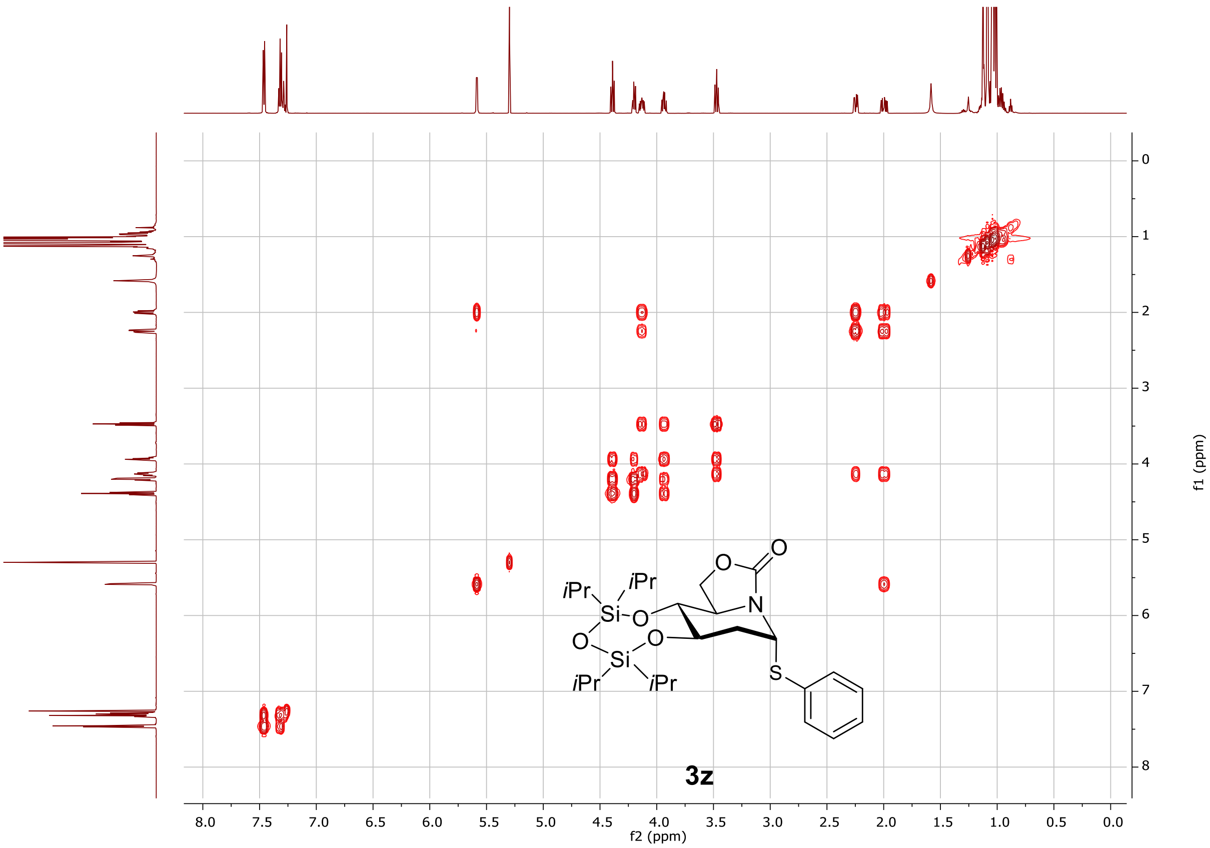

Supplementary Figure S345. COSY spectra for 3z

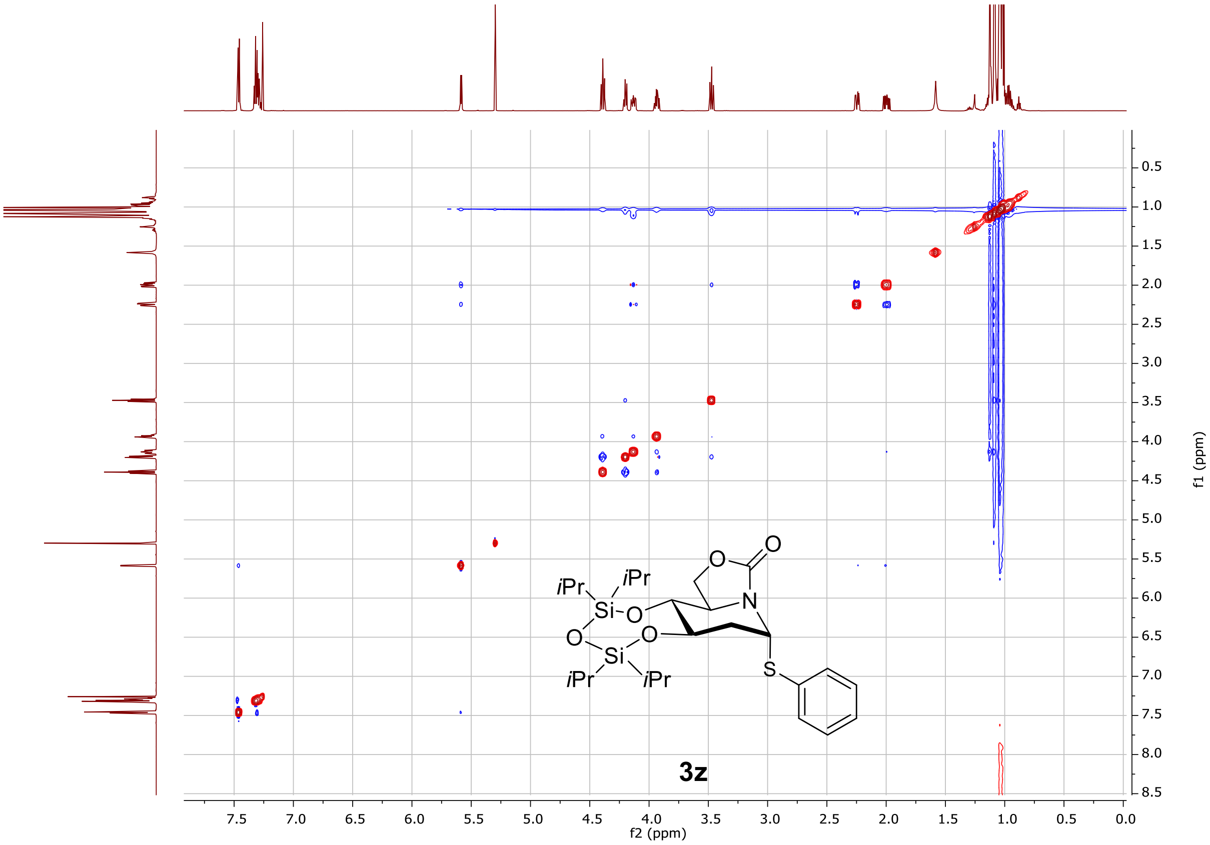

Supplementary Figure S346. NOESY spectra for 3z

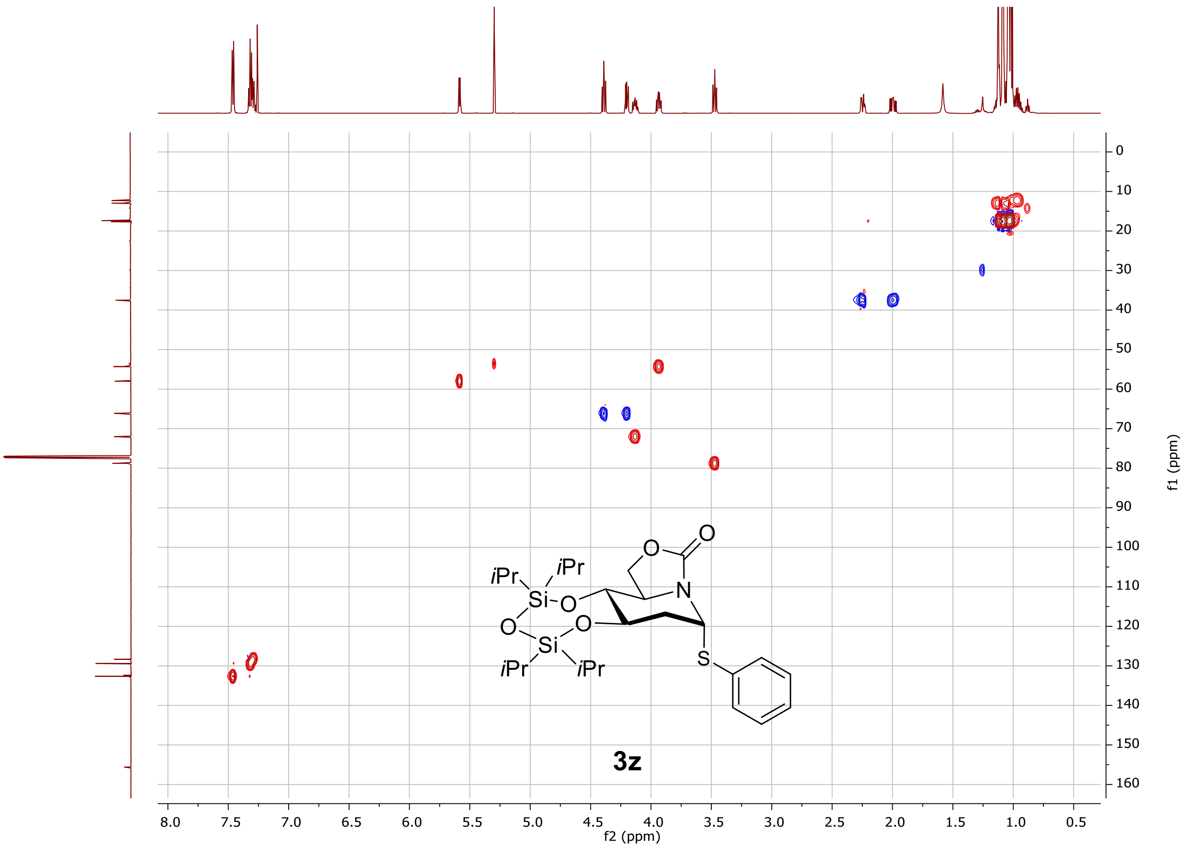

Supplementary Figure S347. HSQC spectra for 3z

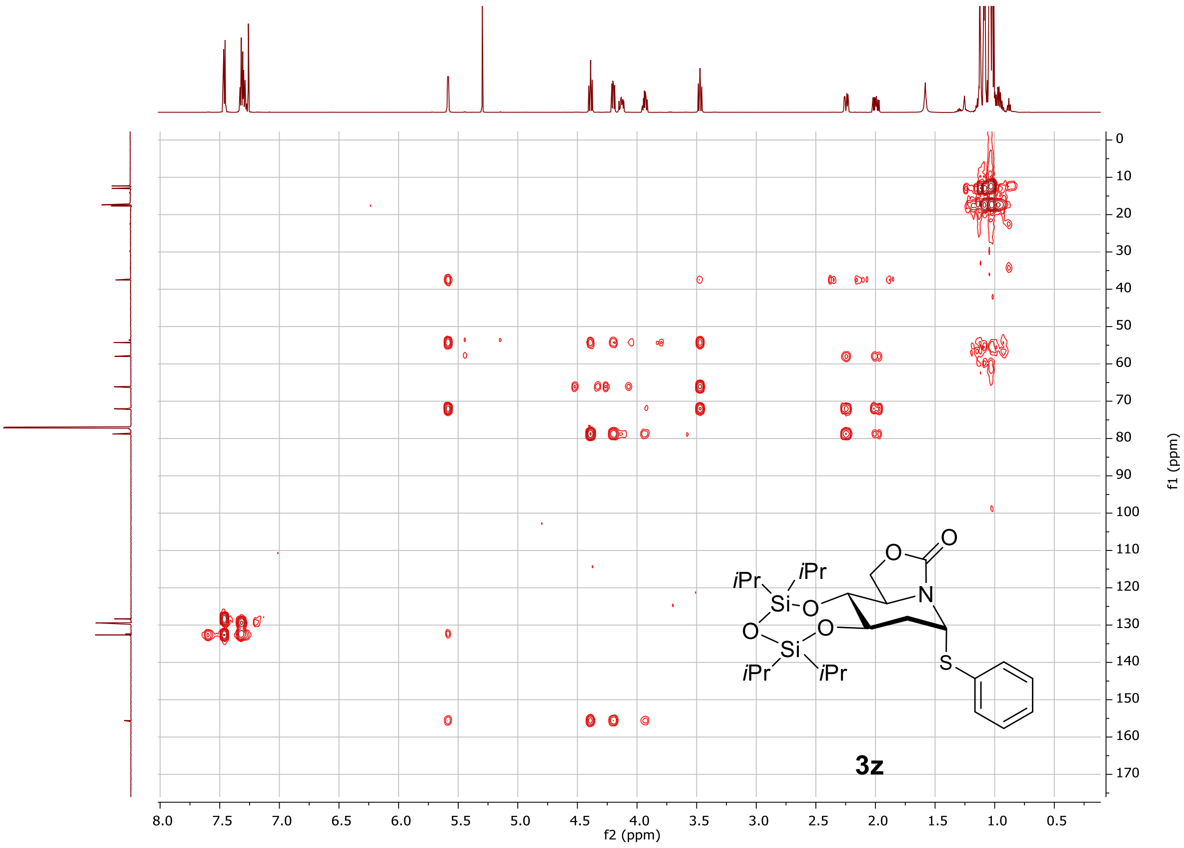

Supplementary Figure S348. HMBC spectra for 3z

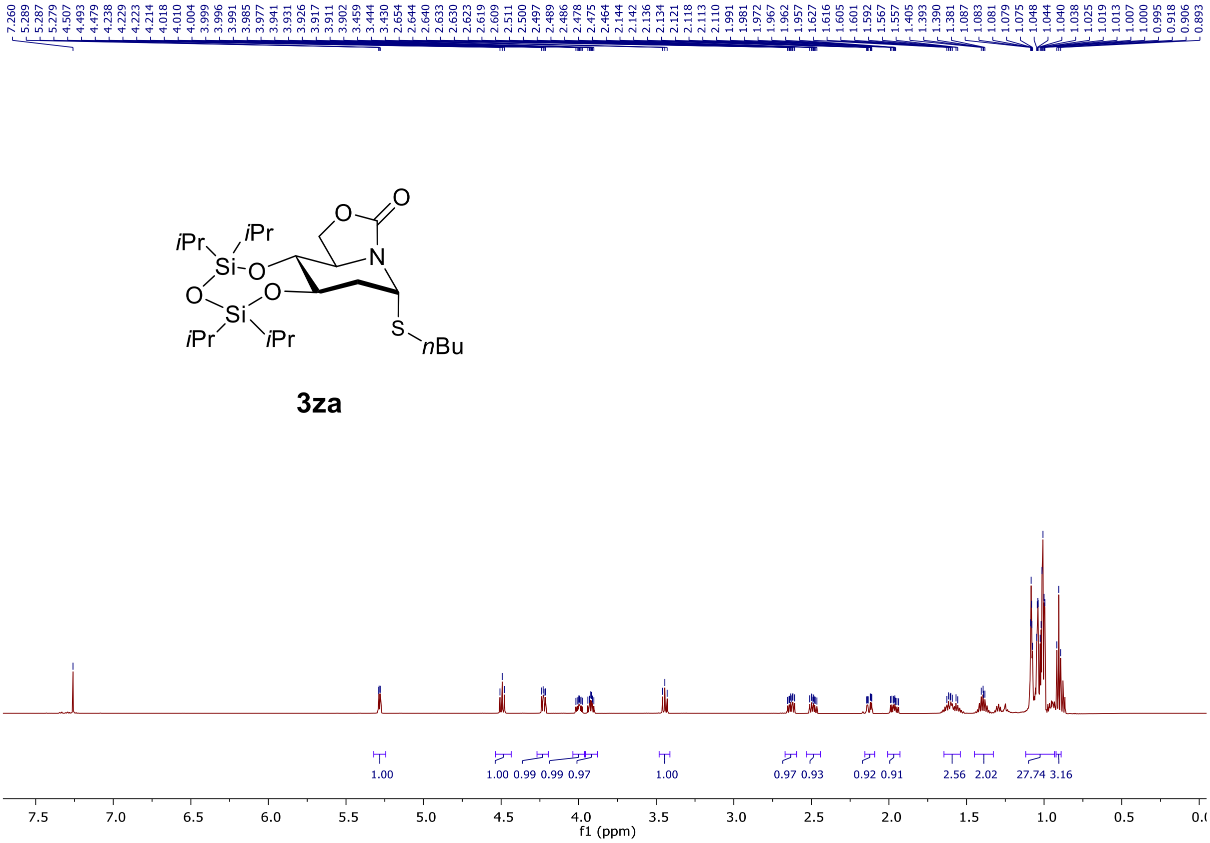

Supplementary Figure S349. ^{1}H NMR spectra for 3za

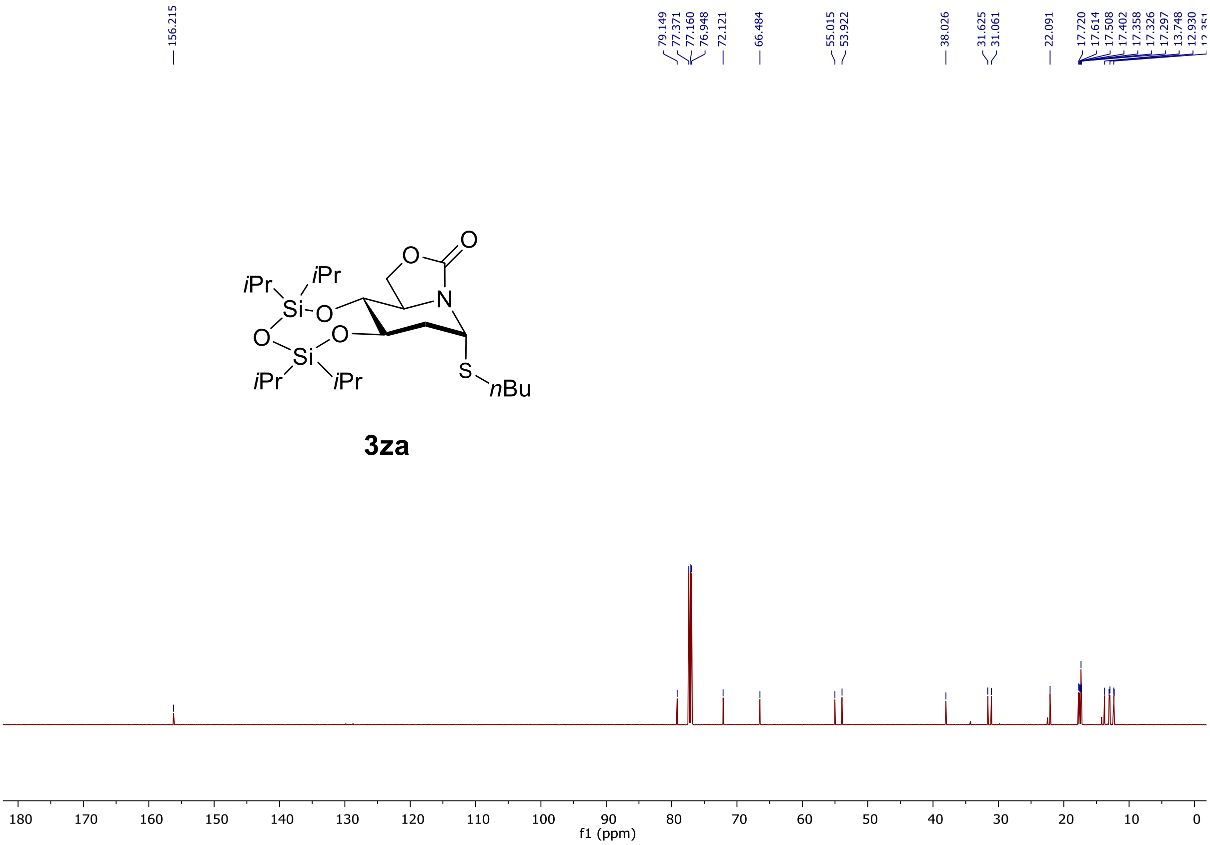

Supplementary Figure S350. ^{13}C NMR spectra for 3za

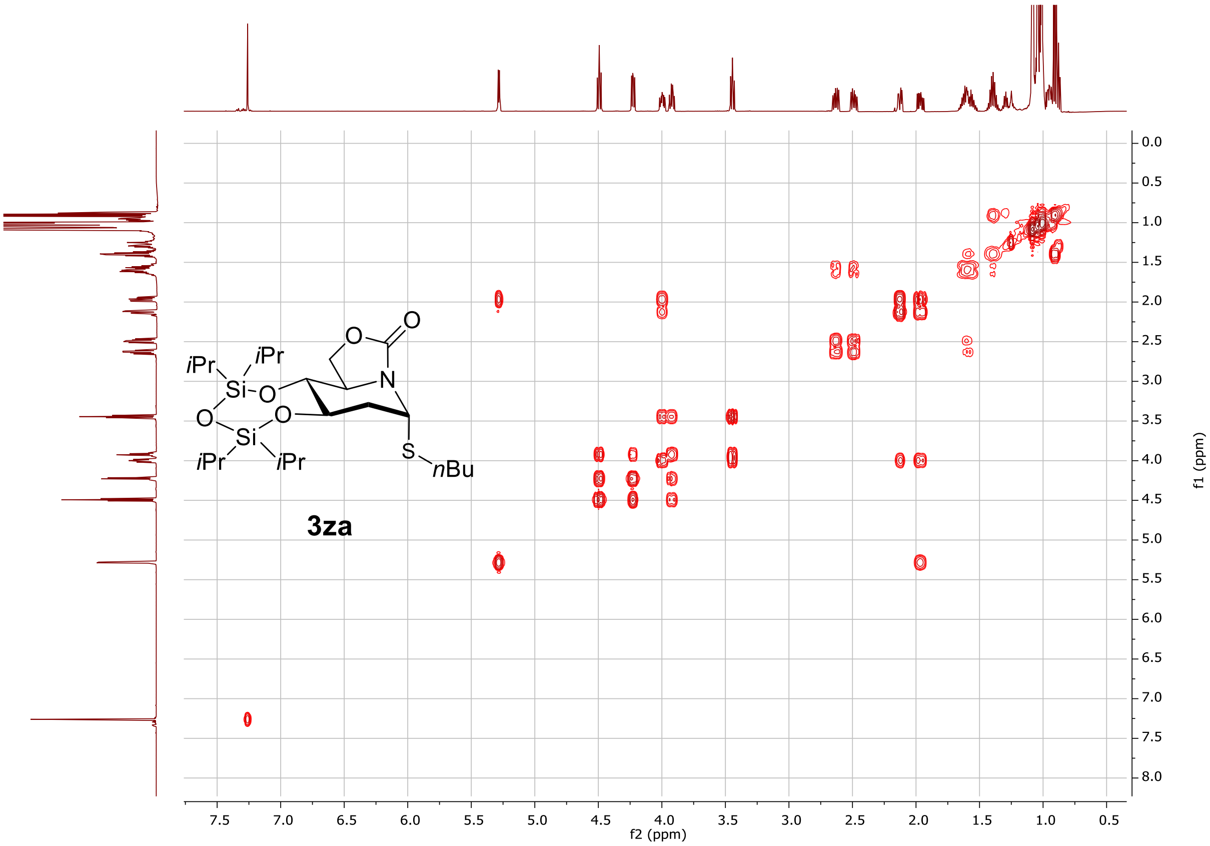

Supplementary Figure S351. COSY spectra for 3za

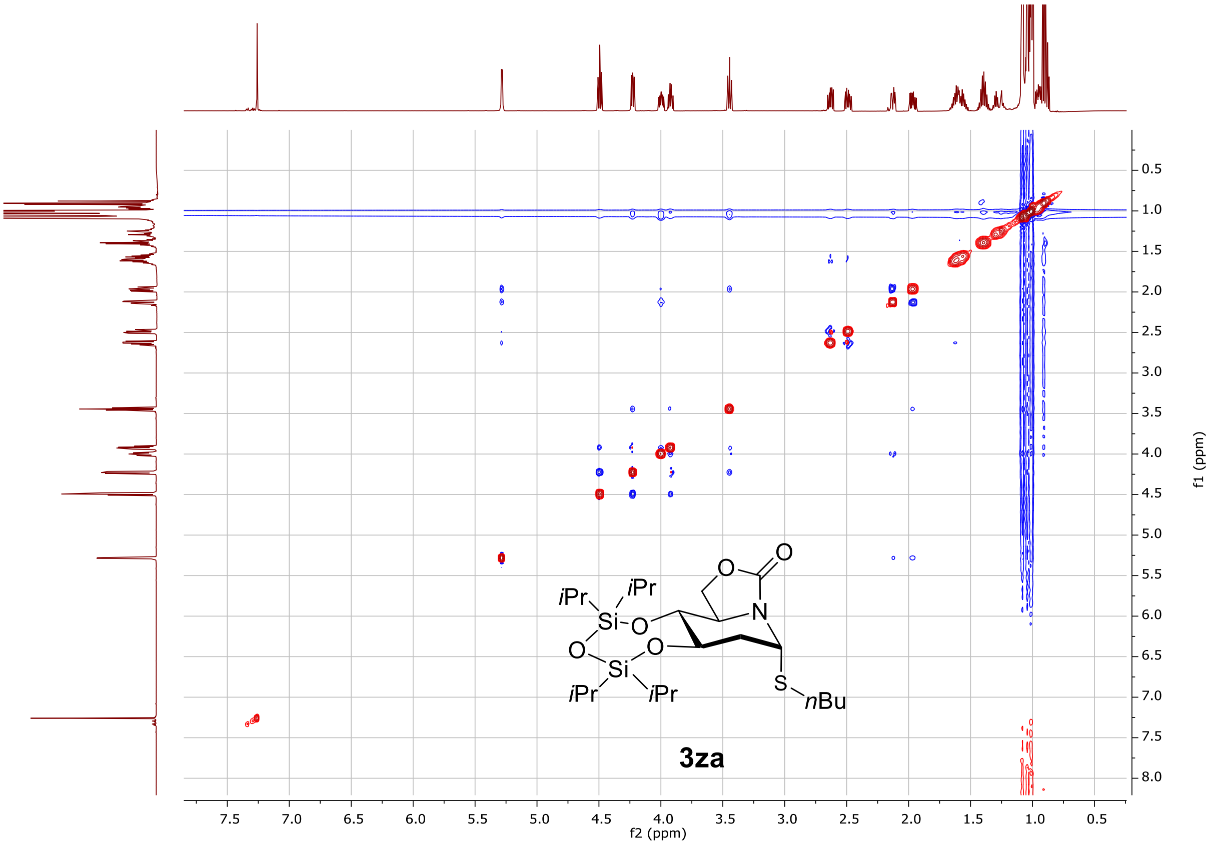

Supplementary Figure S352. NOESY spectra for 3za

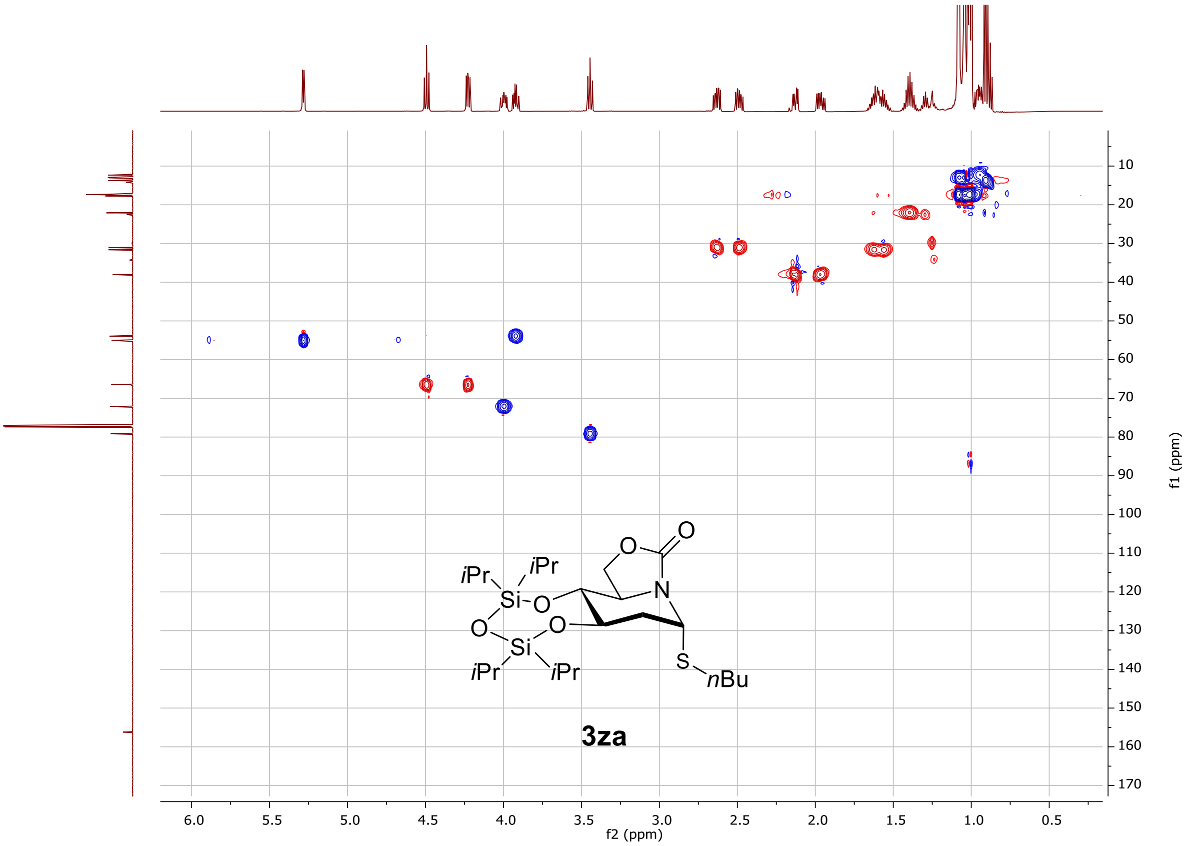

Supplementary Figure S353. HSQC spectra for 3za

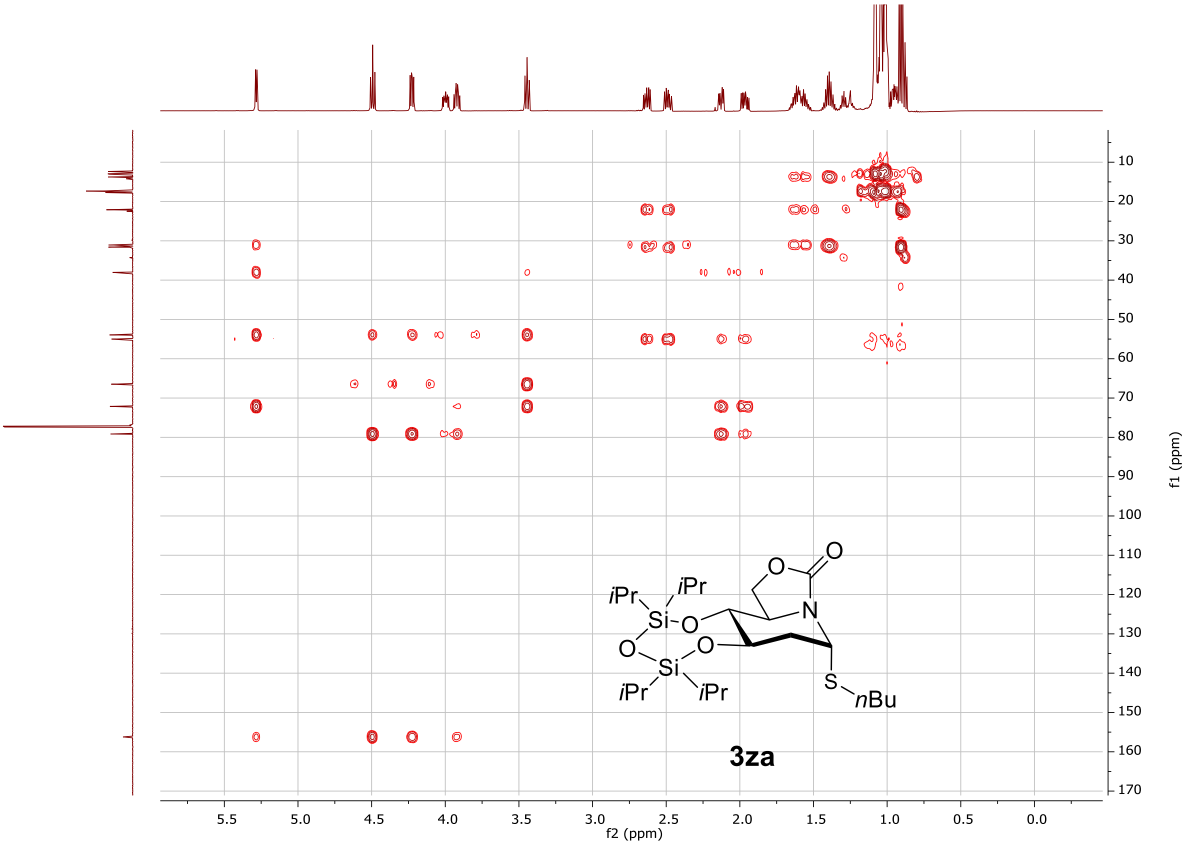

Supplementary Figure S354. HMBC spectra for 3za

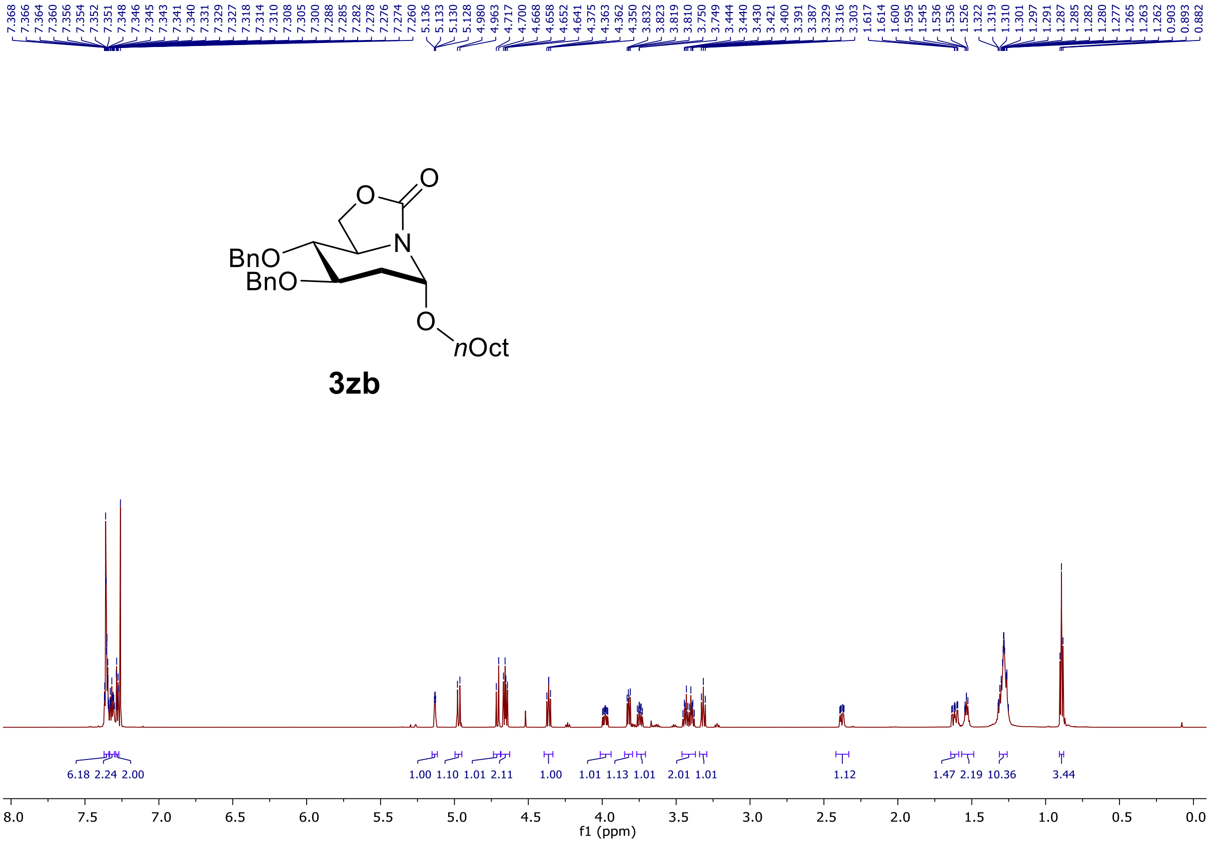

Supplementary Figure S355. ^{1}H NMR spectra for 3zb

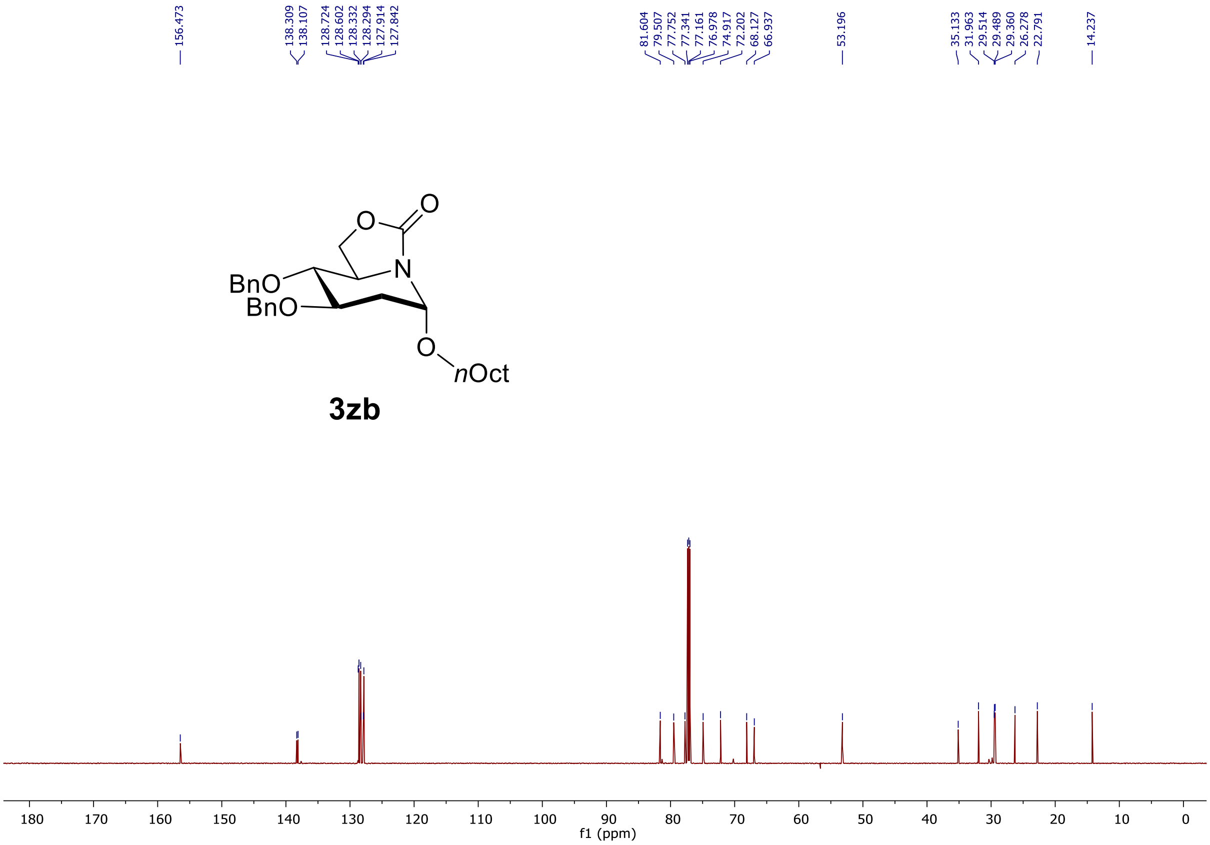

Supplementary Figure S356. ^{13}C NMR spectra for 3zb

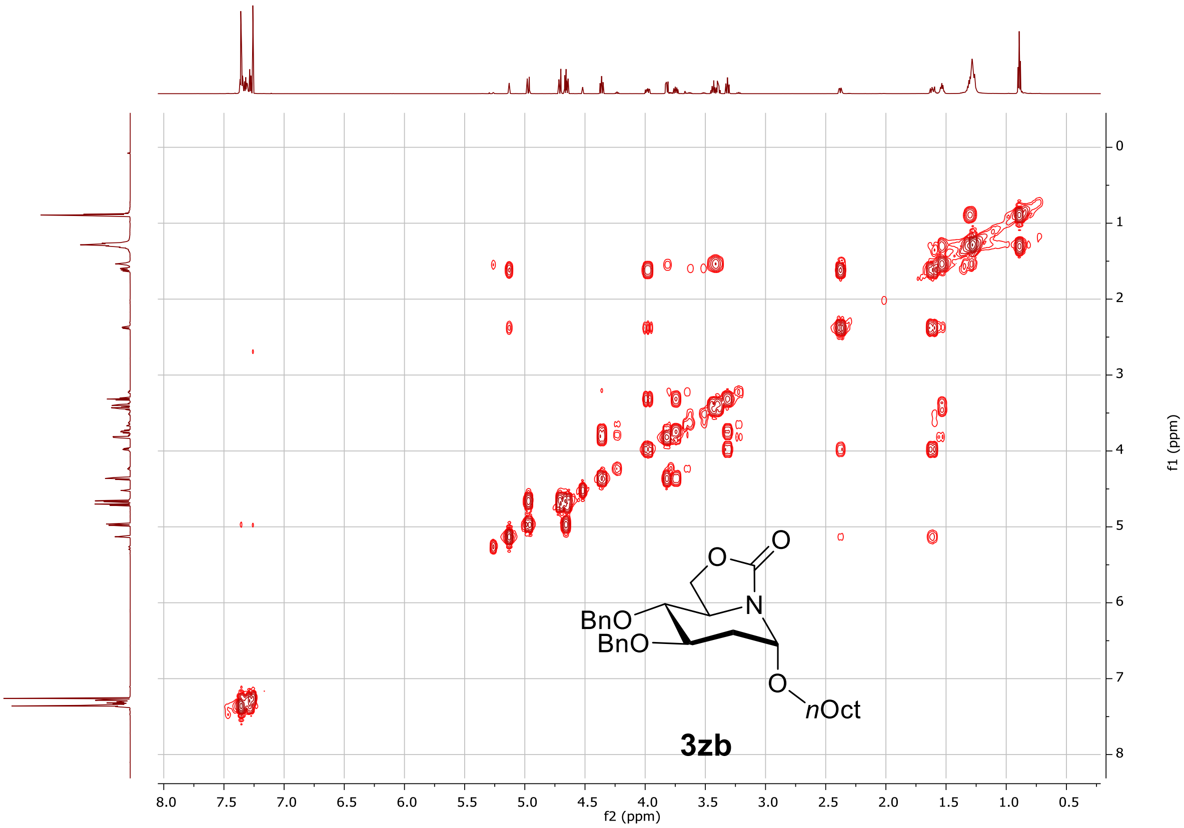

Supplementary Figure S357. COSY spectra for 3zb

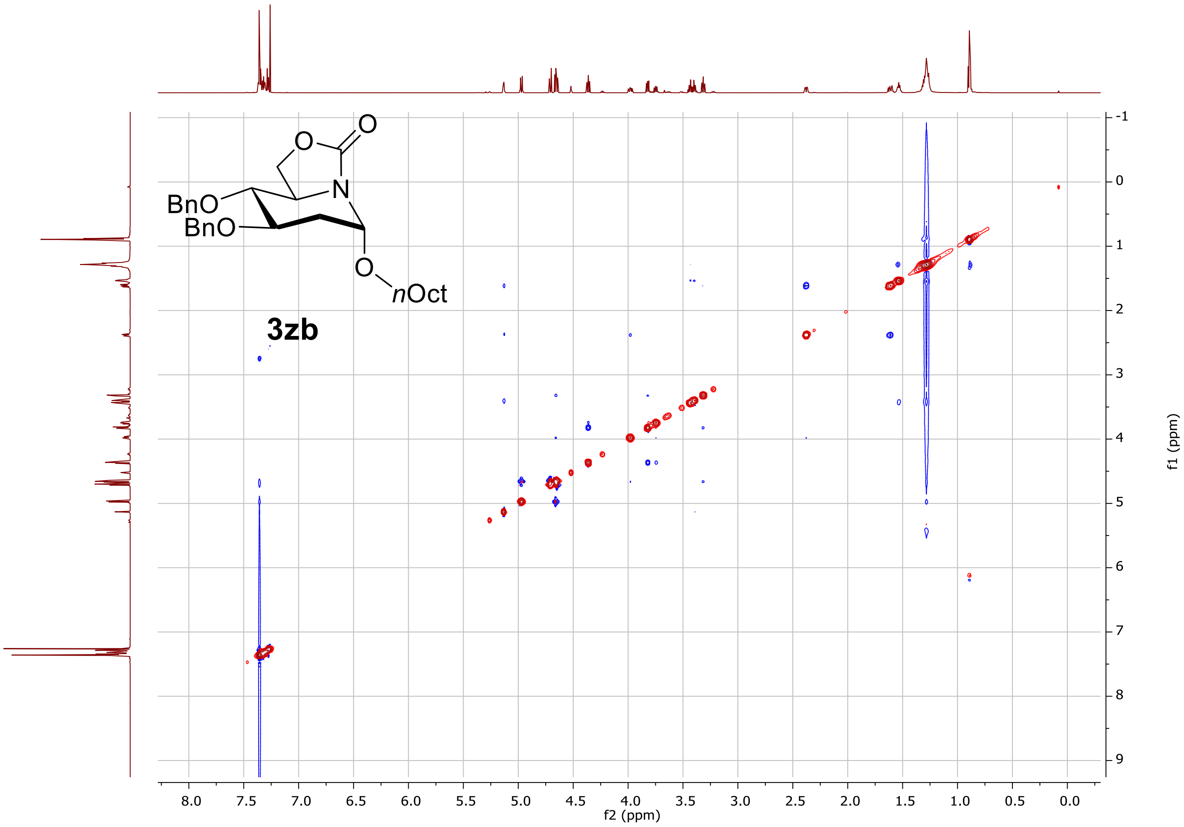

Supplementary Figure S358. NOESY spectra for 3zb

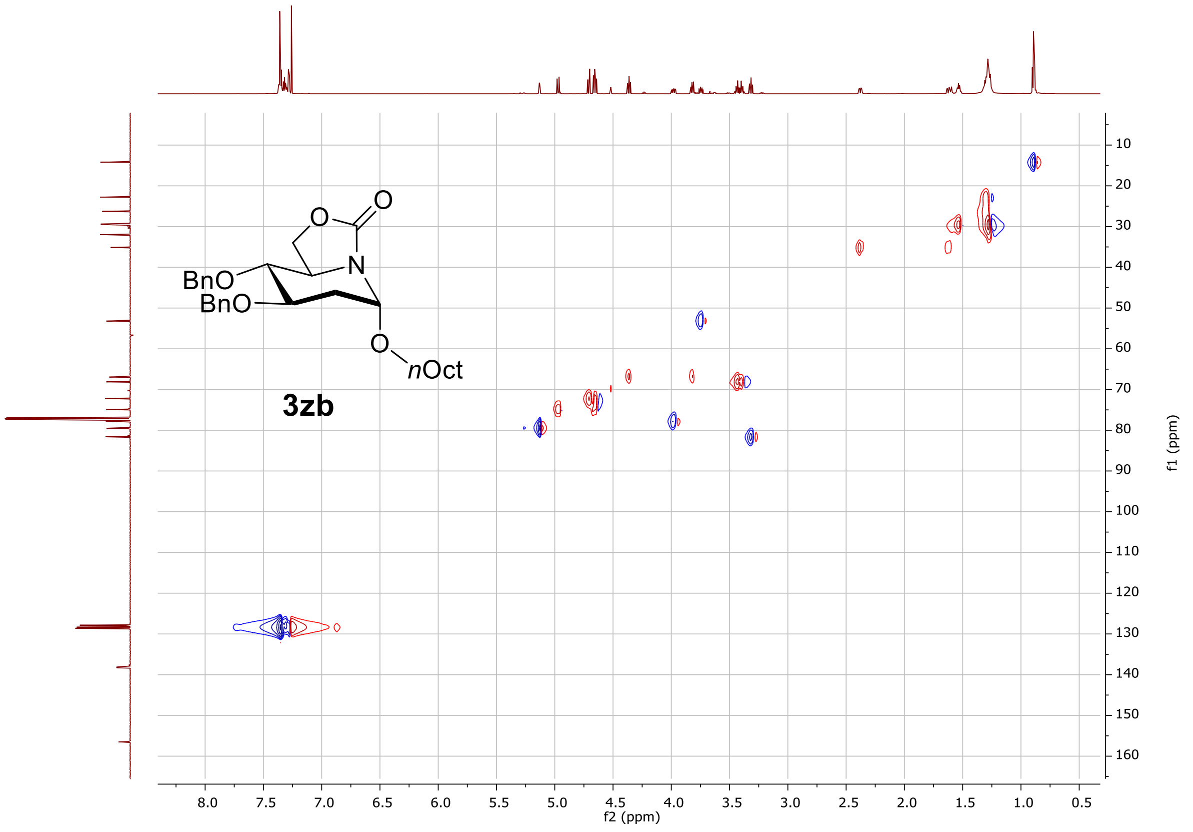

Supplementary Figure S359. HSQC spectra for 3zb

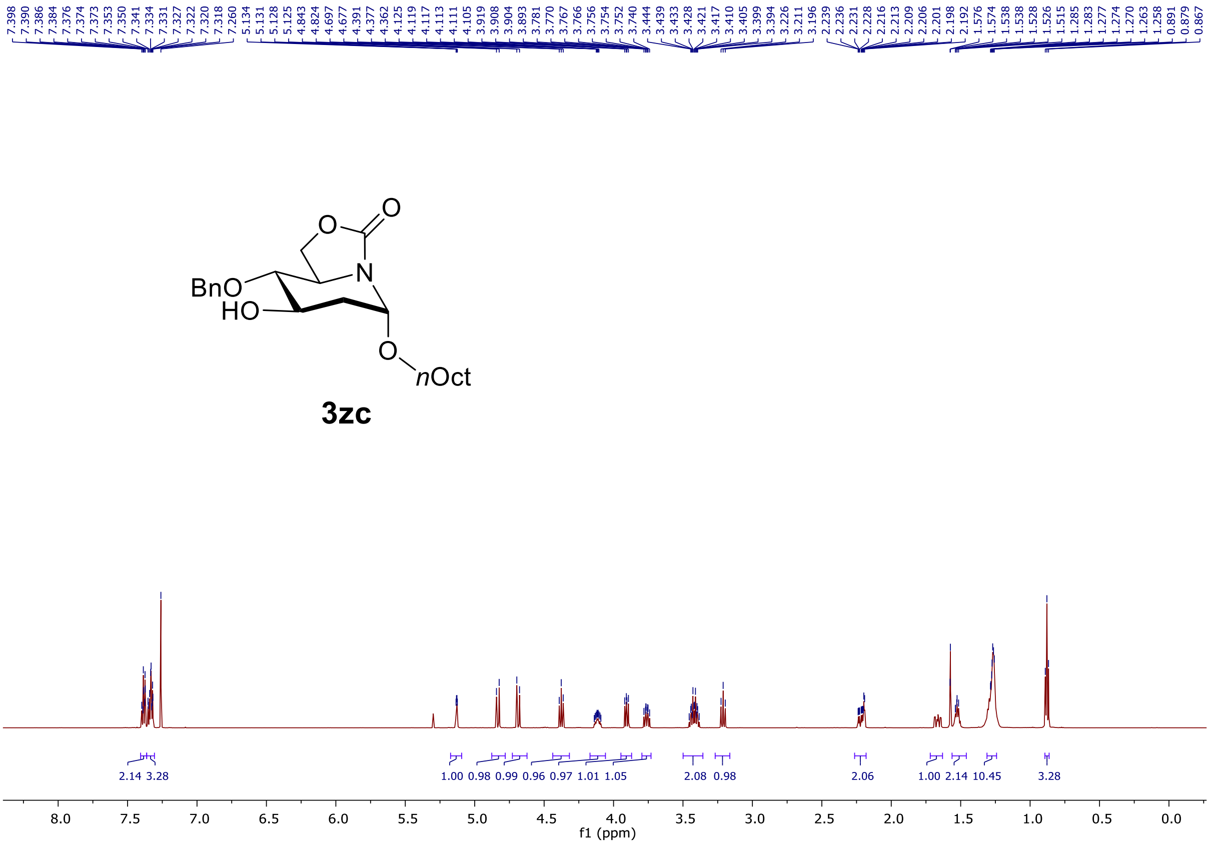

Supplementary Figure S360. ¹H NMR spectra for 3zc

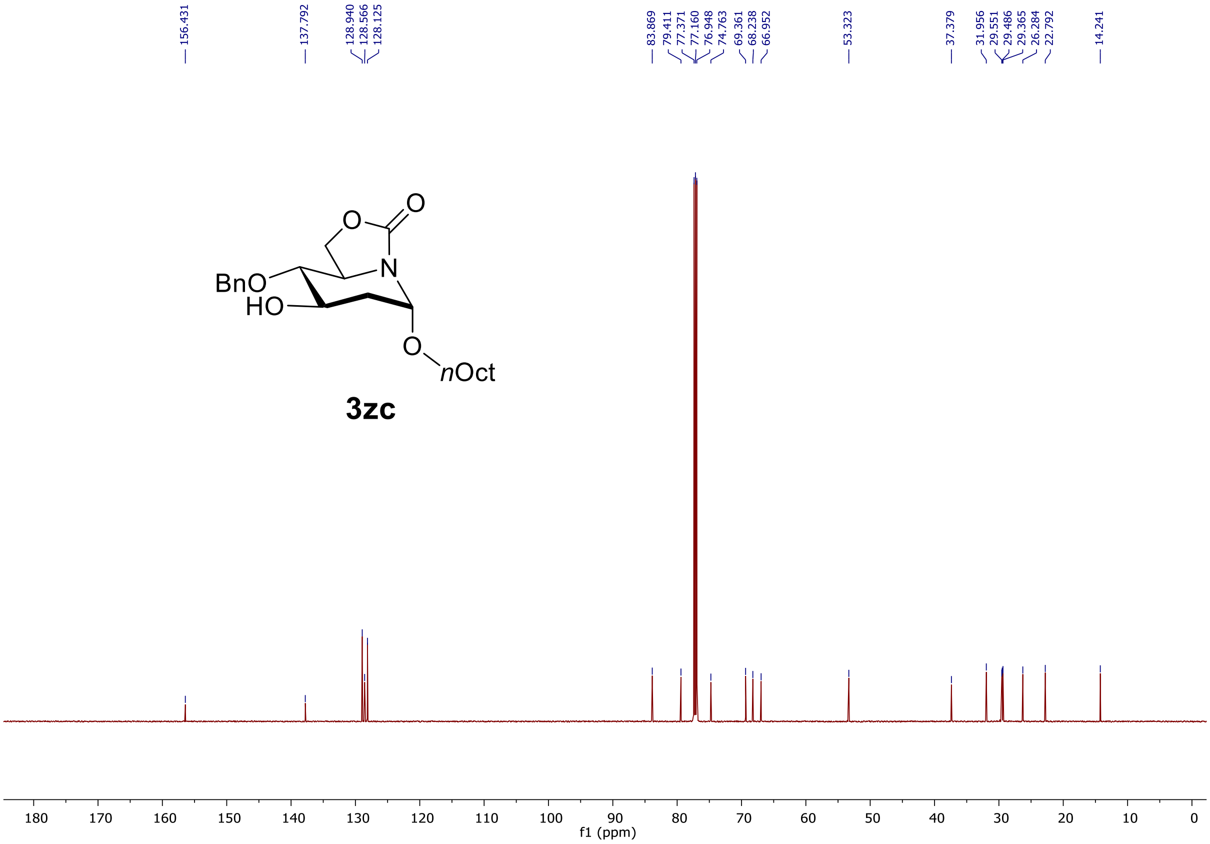

Supplementary Figure S361. ^{13}C NMR spectra for 3zc

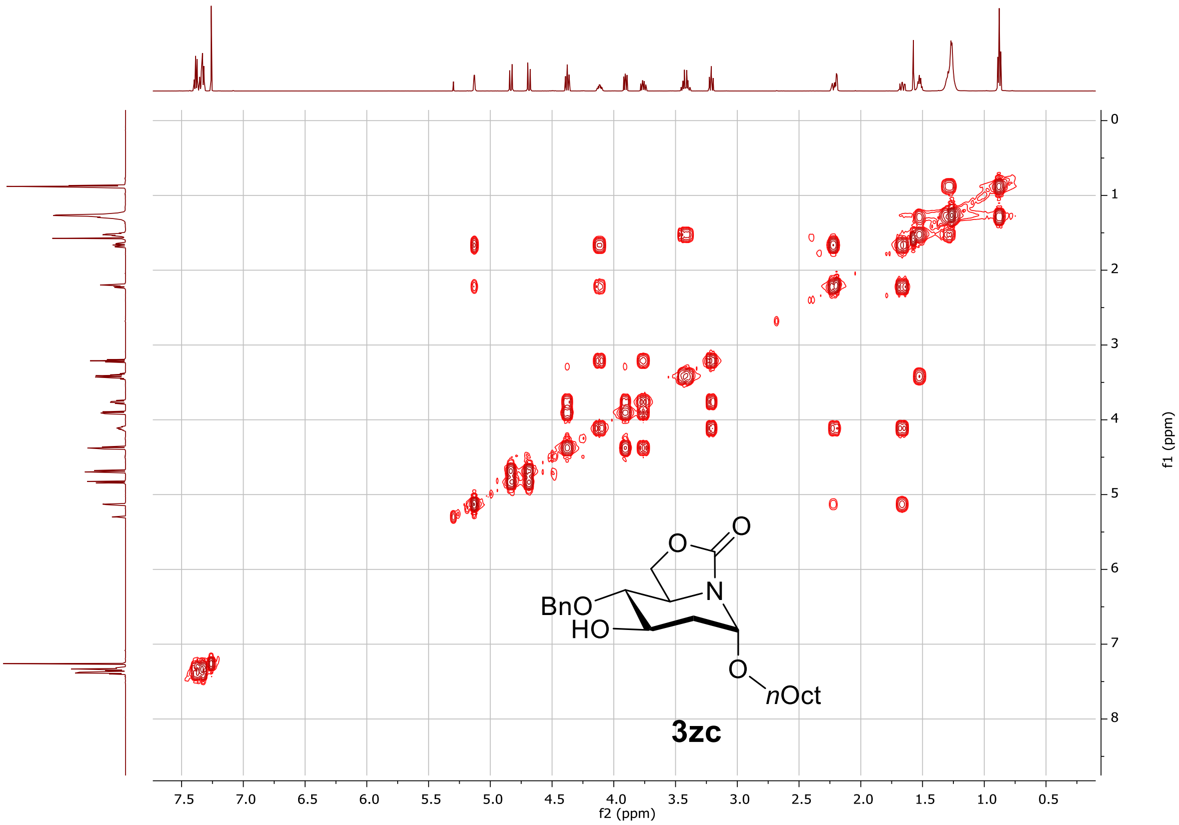

Supplementary Figure S362. COSY spectra for 3zc

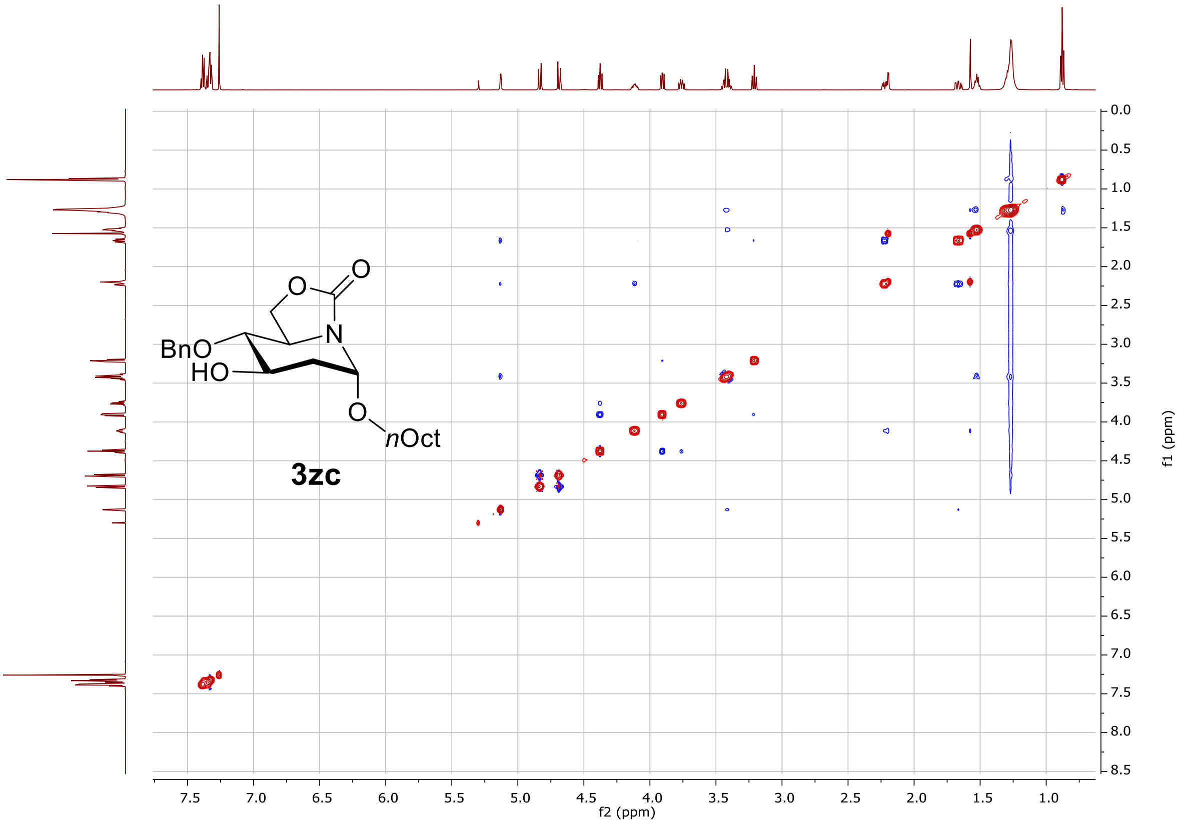

Supplementary Figure S363. NOESY spectra for 3zc

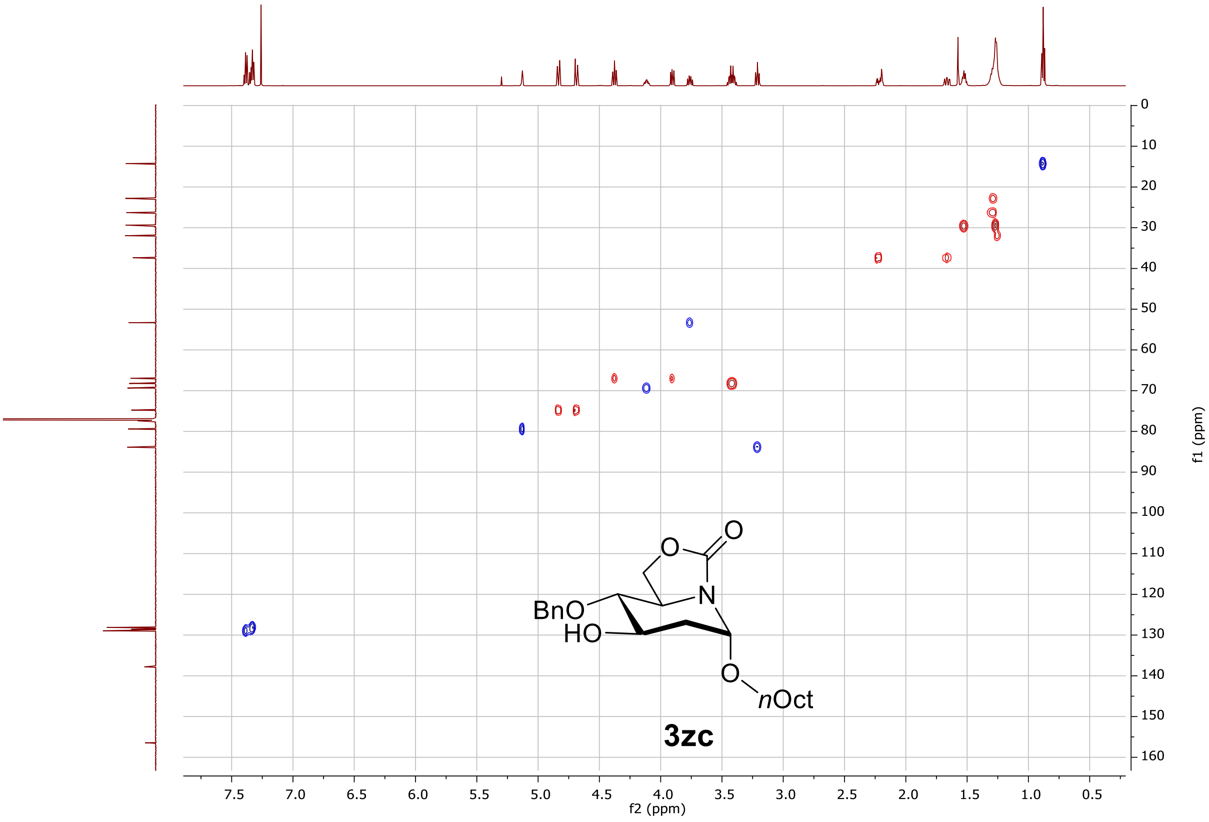

Supplementary Figure S364. HSQC spectra for 3zc

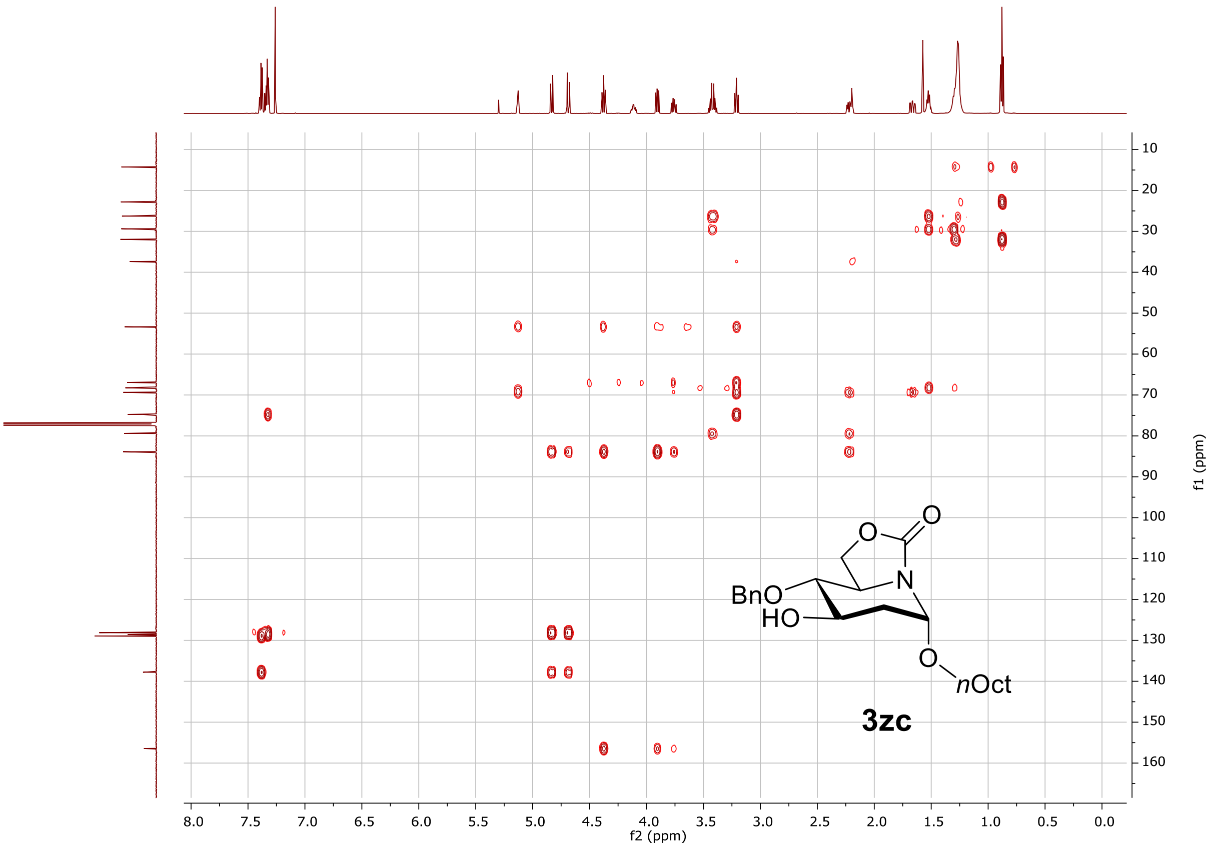

Supplementary Figure S365. HMBC spectra for 3zc

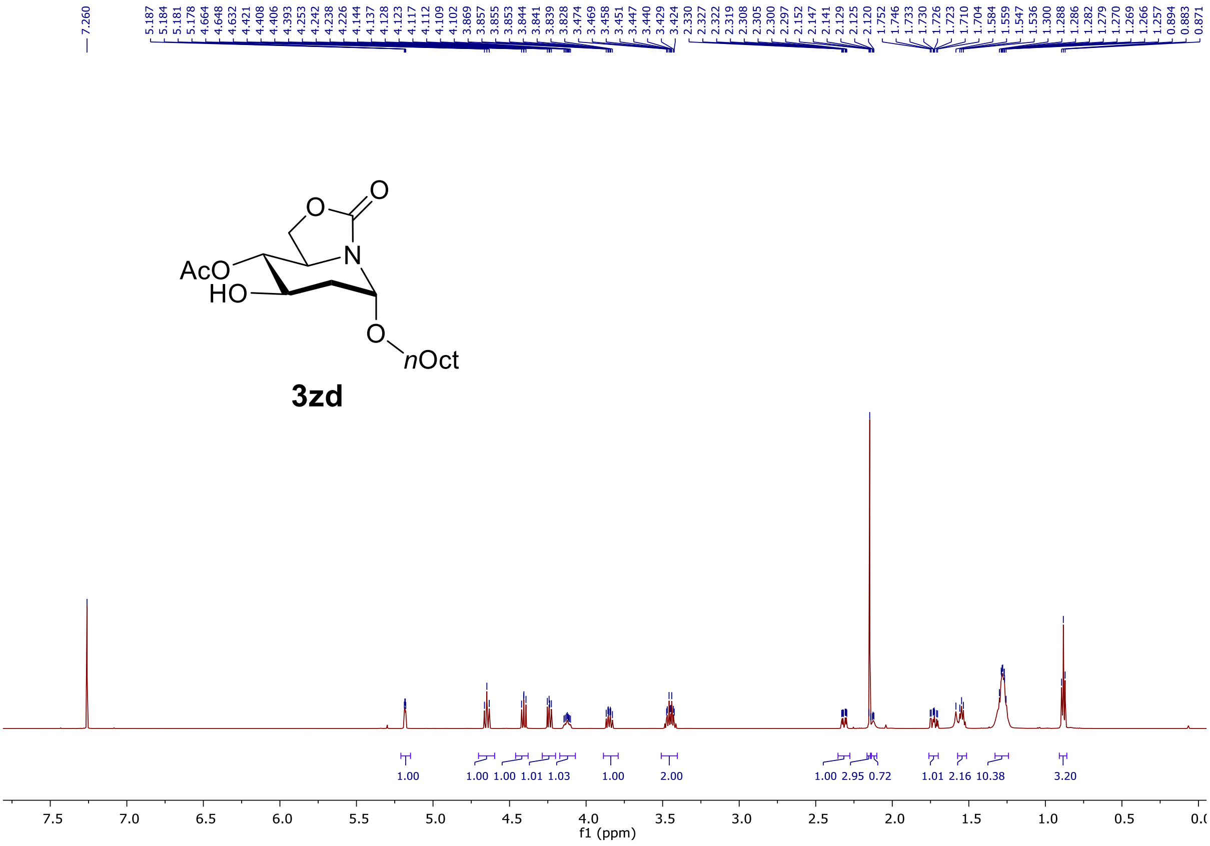

Supplementary Figure S366. ^{1}H NMR spectra for 3zd

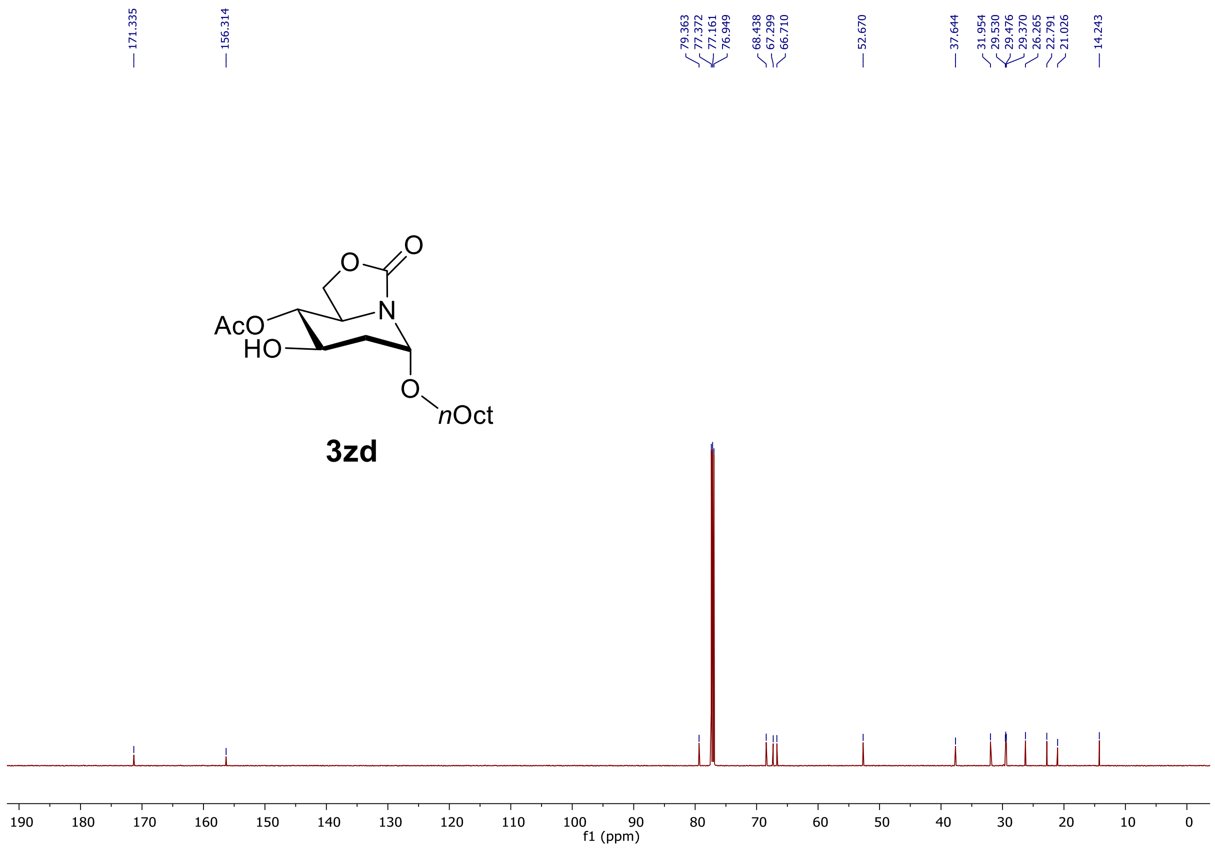

Supplementary Figure S367. ^{13}**C NMR spectra for 3zd**

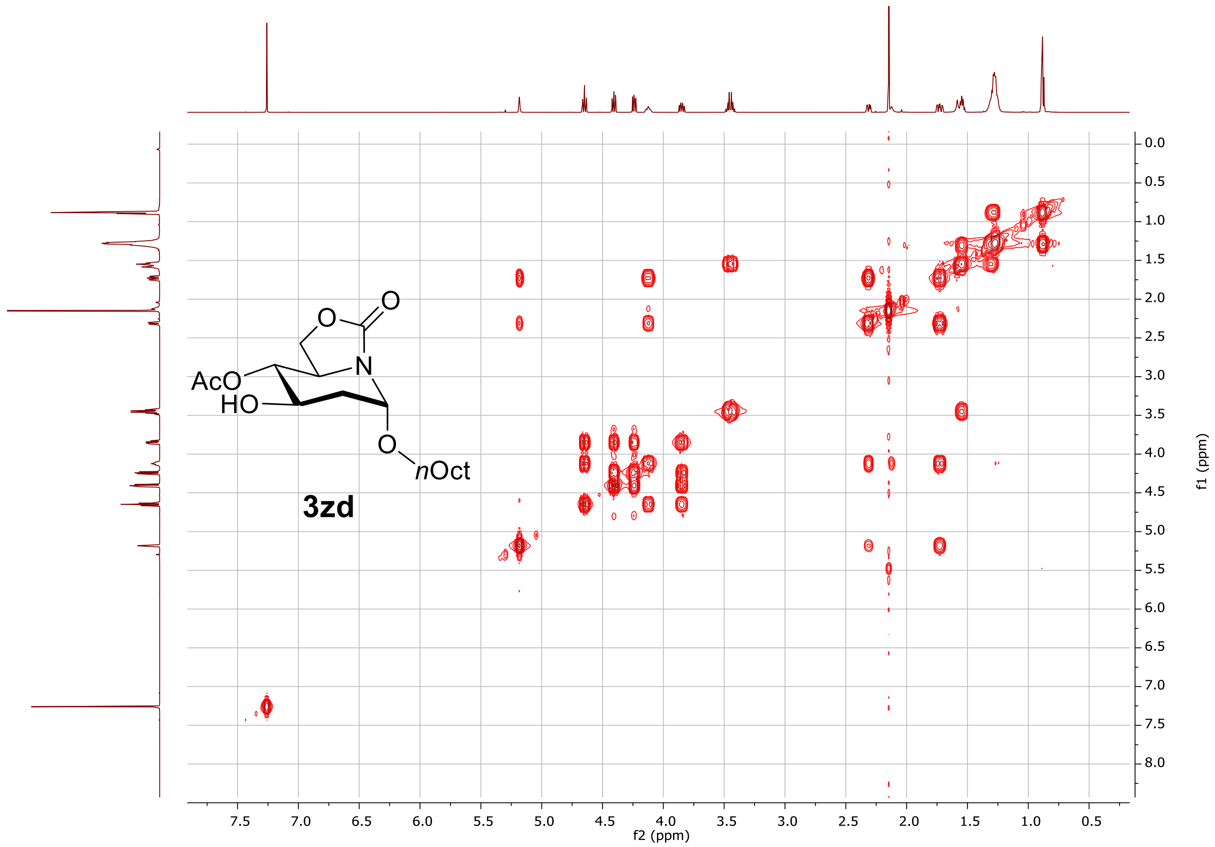

Supplementary Figure S368. COSY spectra for 3zd

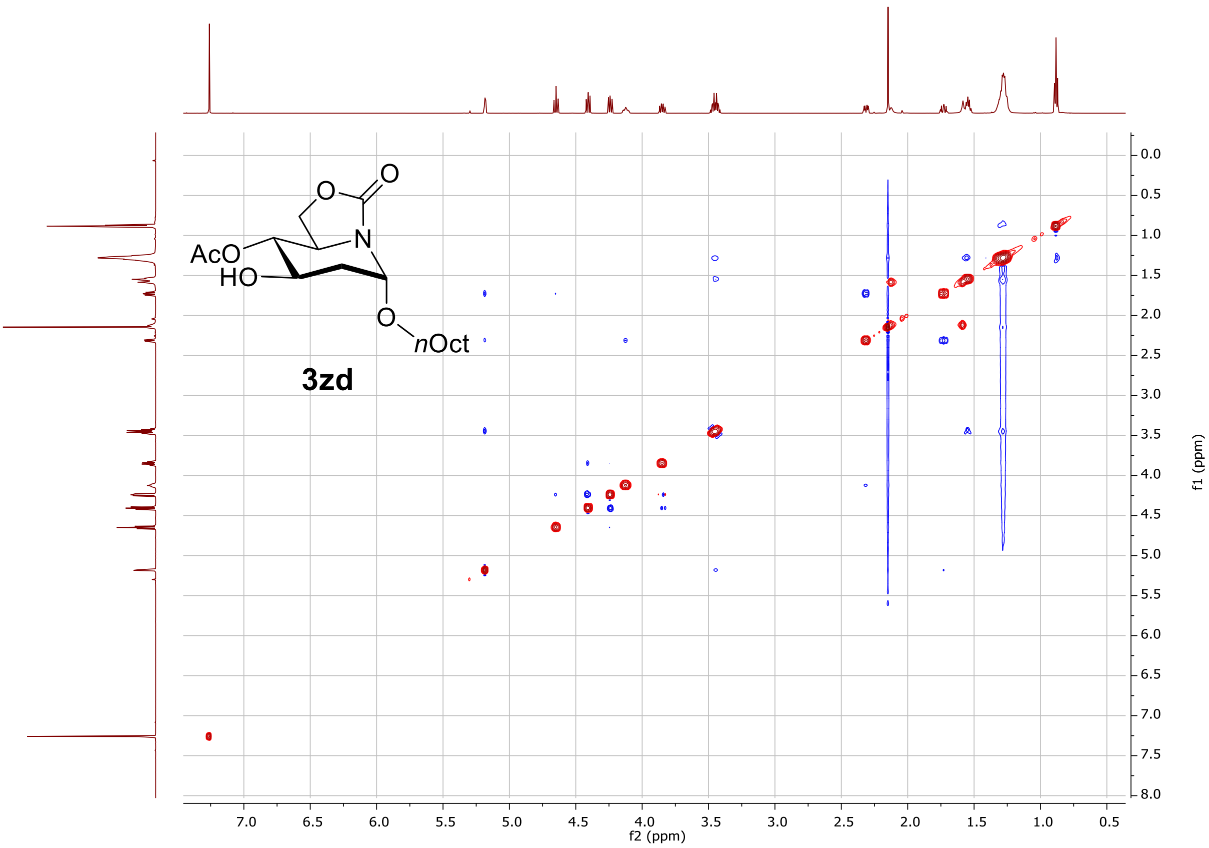

Supplementary Figure S369. NOESY spectra for 3zd

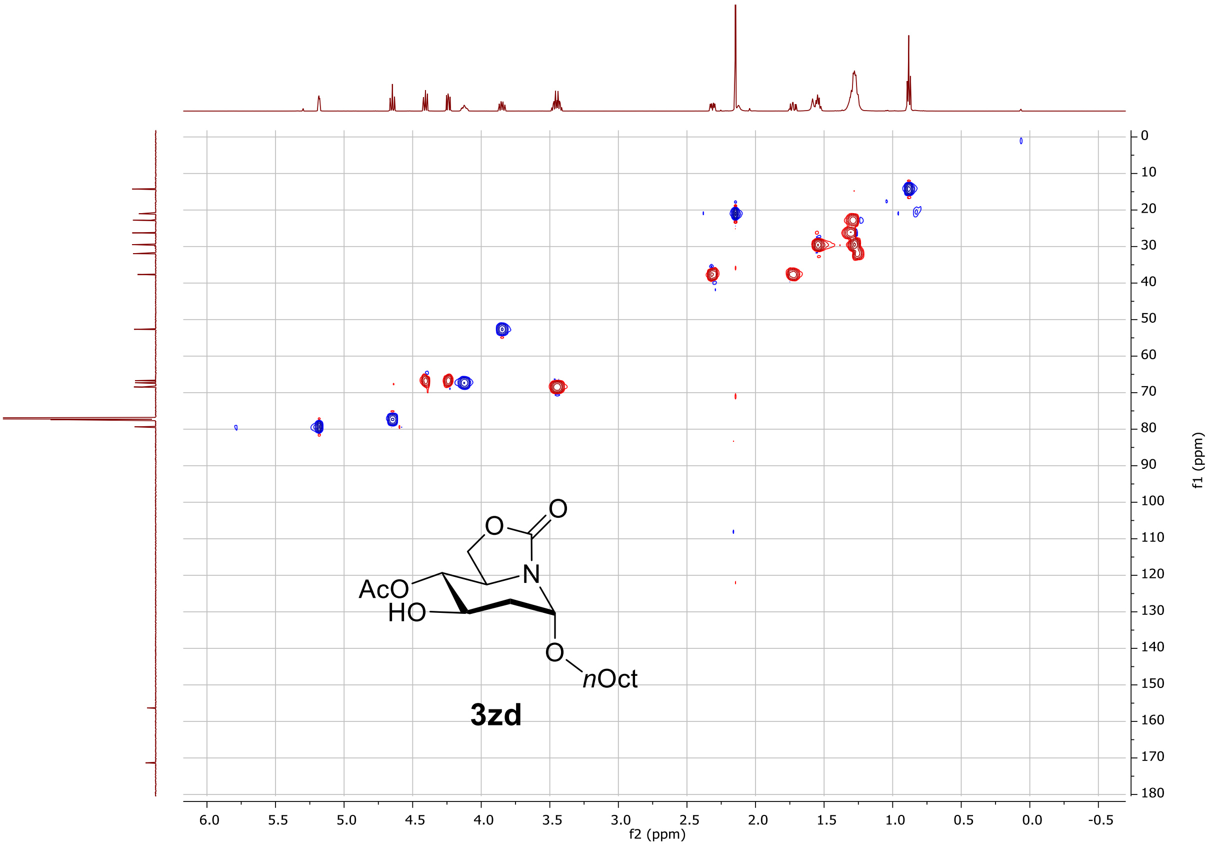

Supplementary Figure S370. HSQC spectra for 3zd

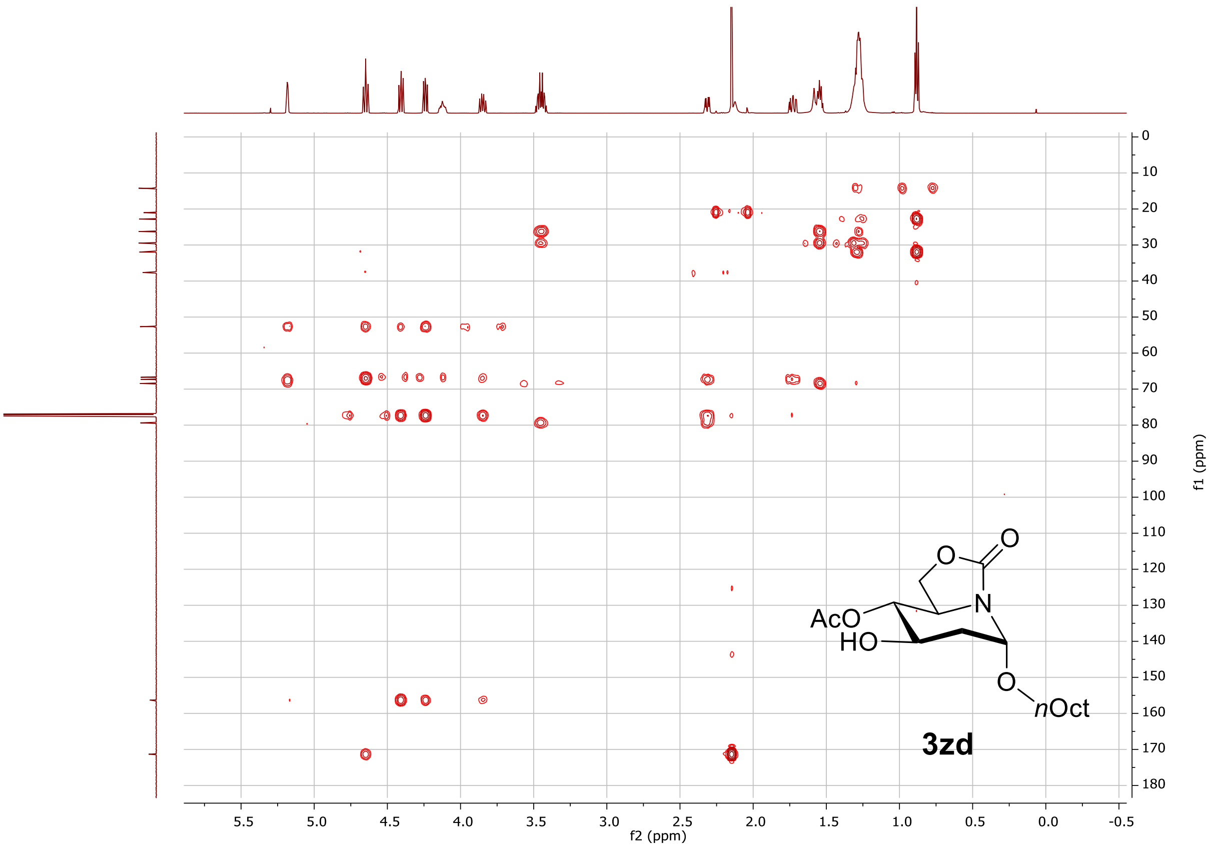

Supplementary Figure S371. HMBC spectra for 3zd

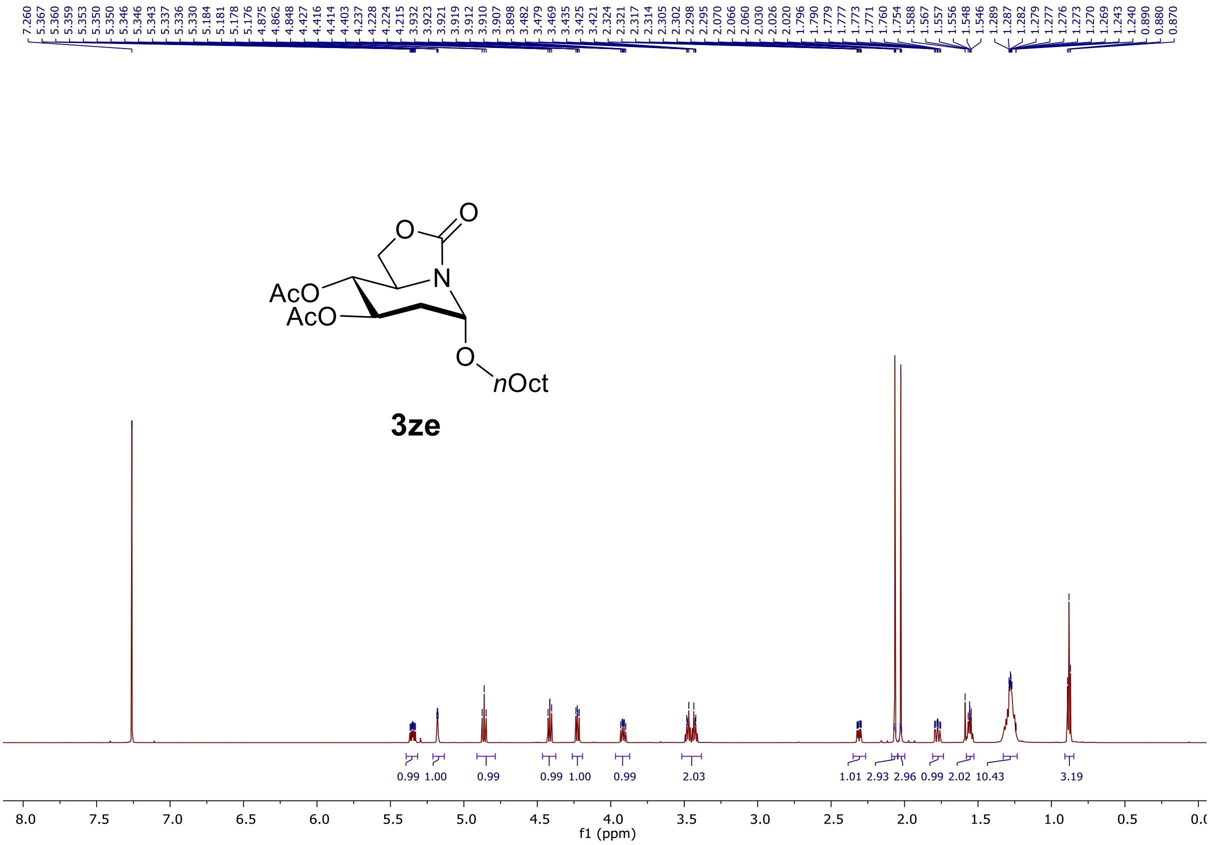

Supplementary Figure S372. ^{1}H NMR spectra for 3ze

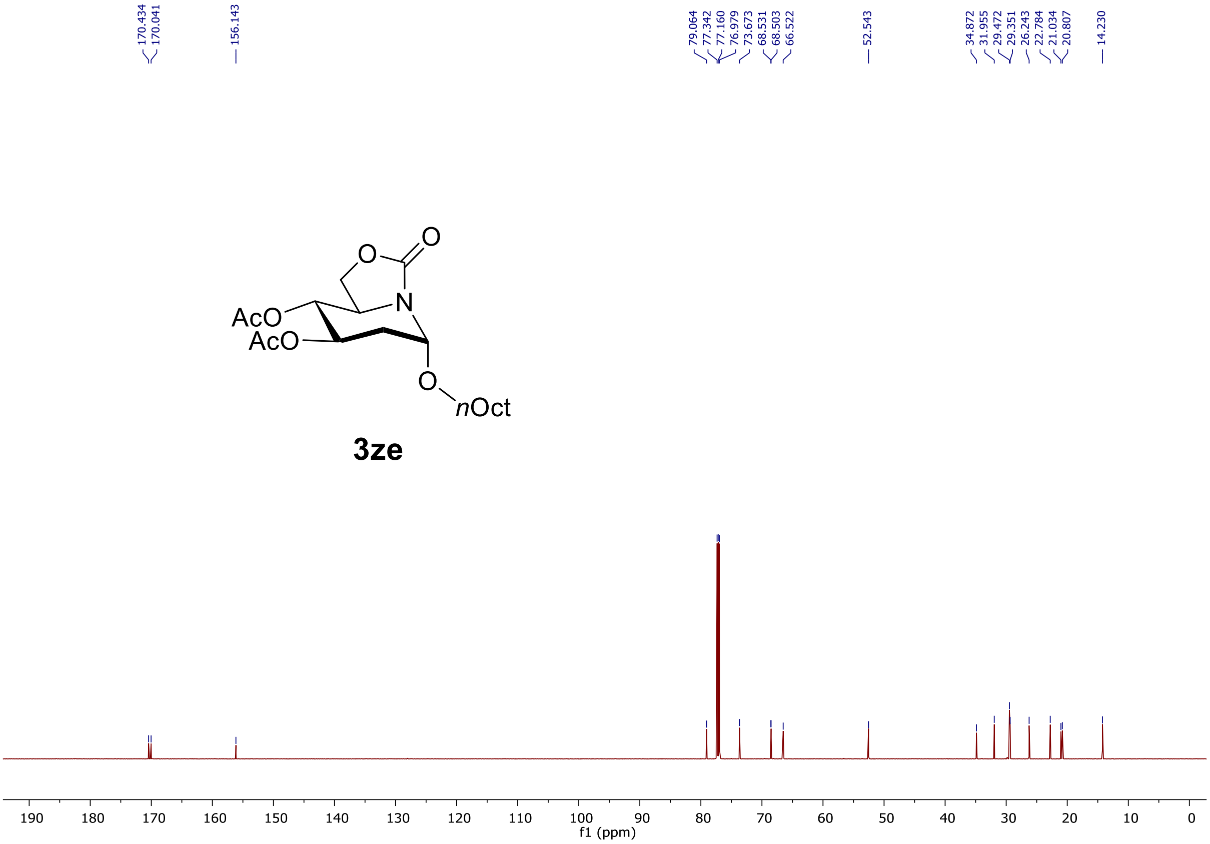

Supplementary Figure S373. ^{13}C NMR spectra for 3ze

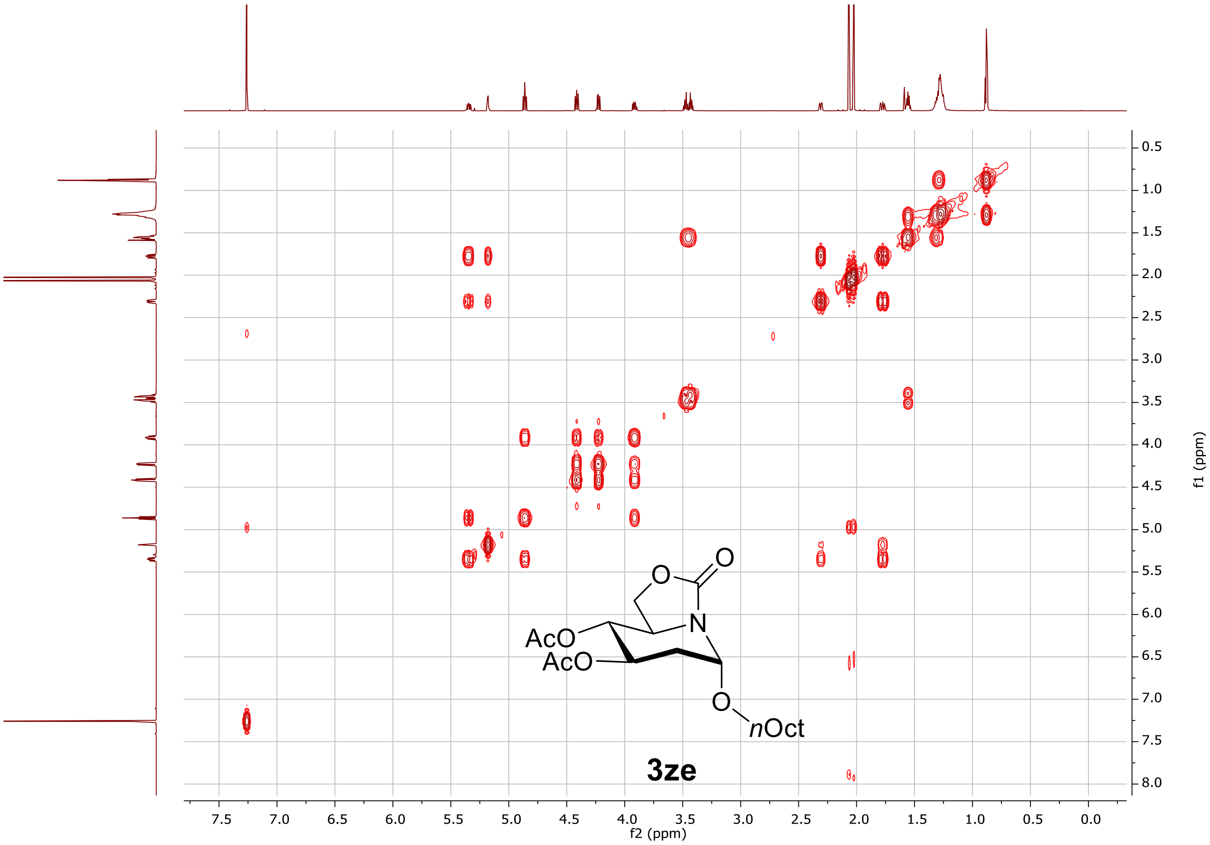

Supplementary Figure S374. COSY spectra for 3ze

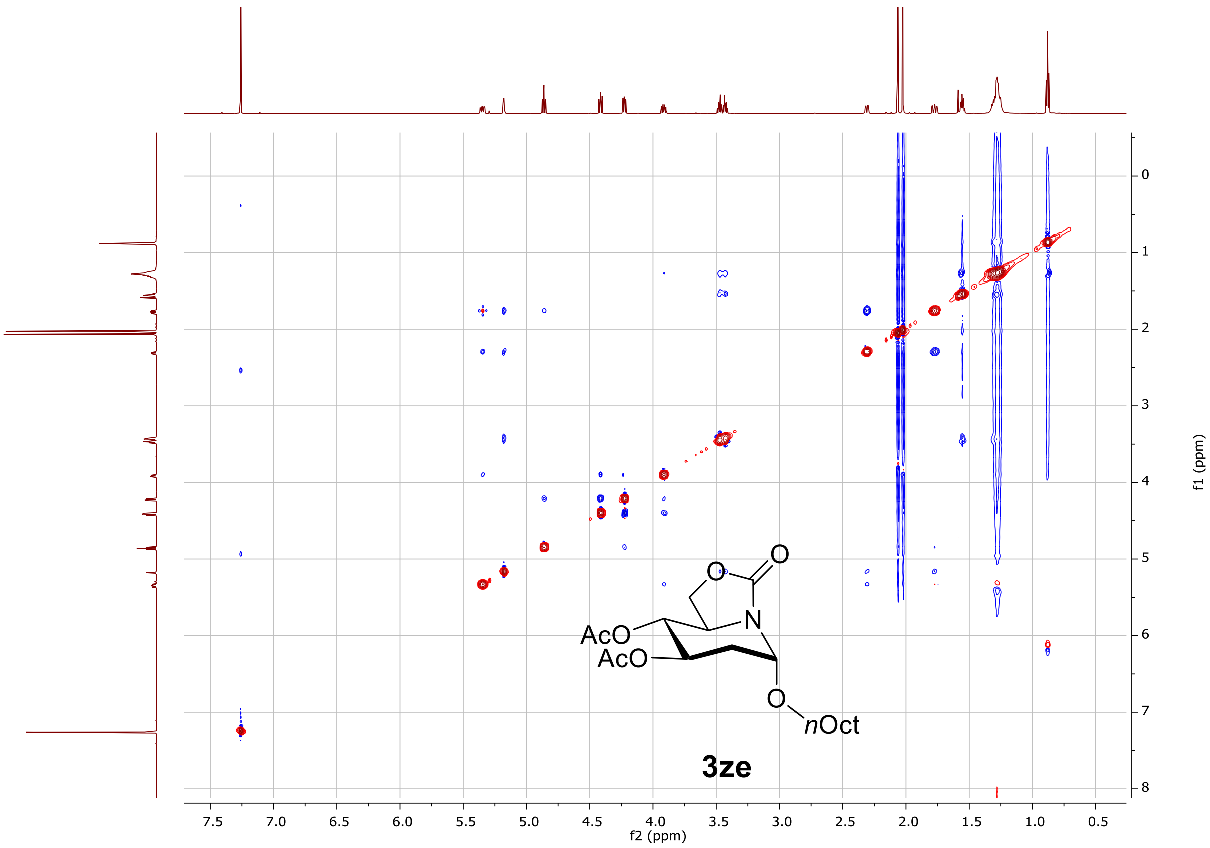

Supplementary Figure S375. NOESY spectra for 3ze

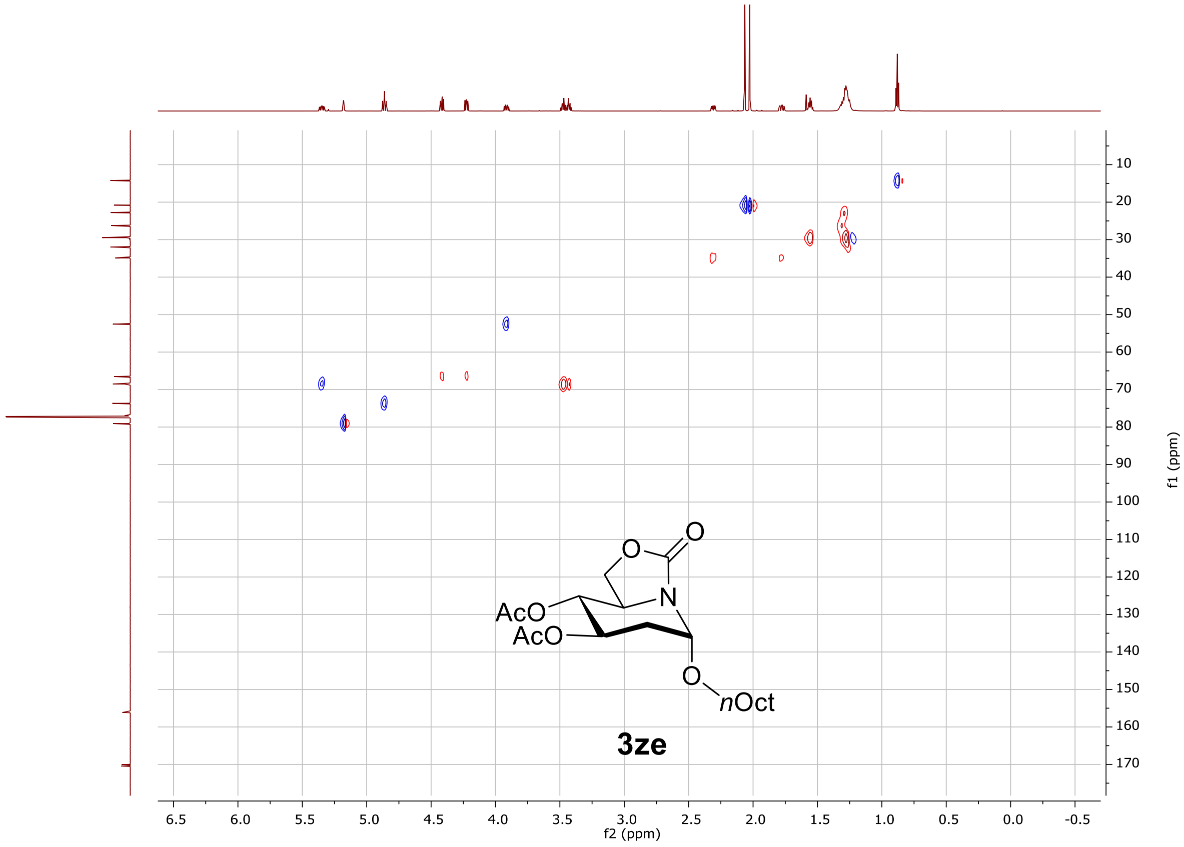

Supplementary Figure S376. HSQC spectra for 3ze

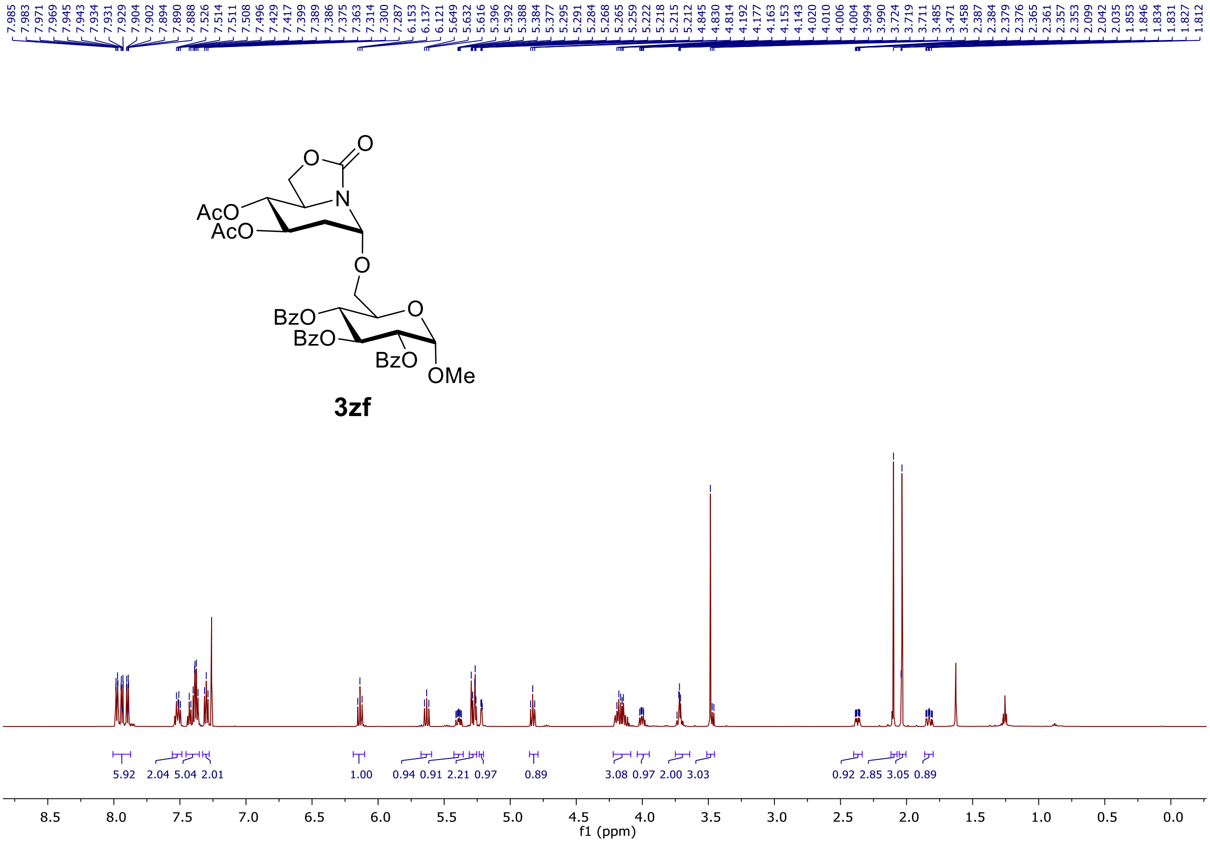

Supplementary Figure S377. ^{1}H NMR spectra for 3zf

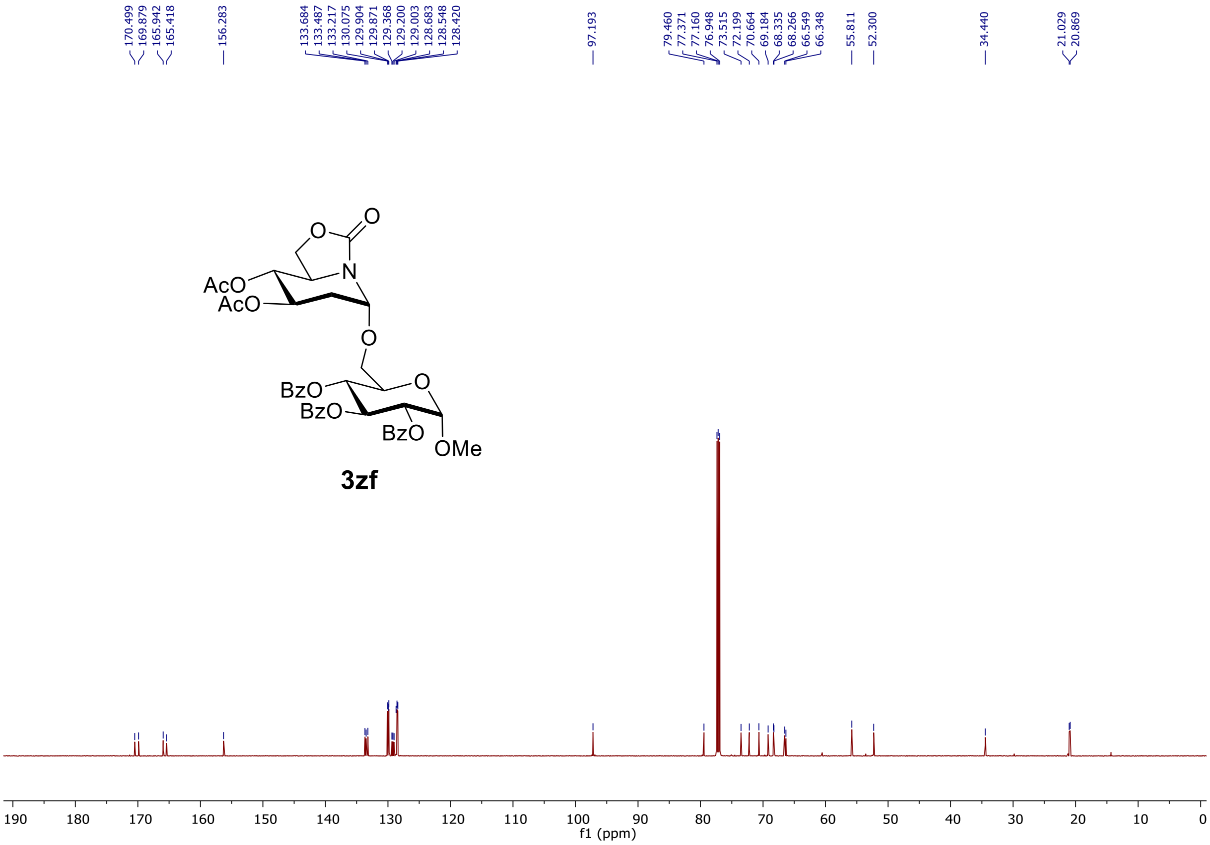

Supplementary Figure S378. ^{13}C NMR spectra for 3zf

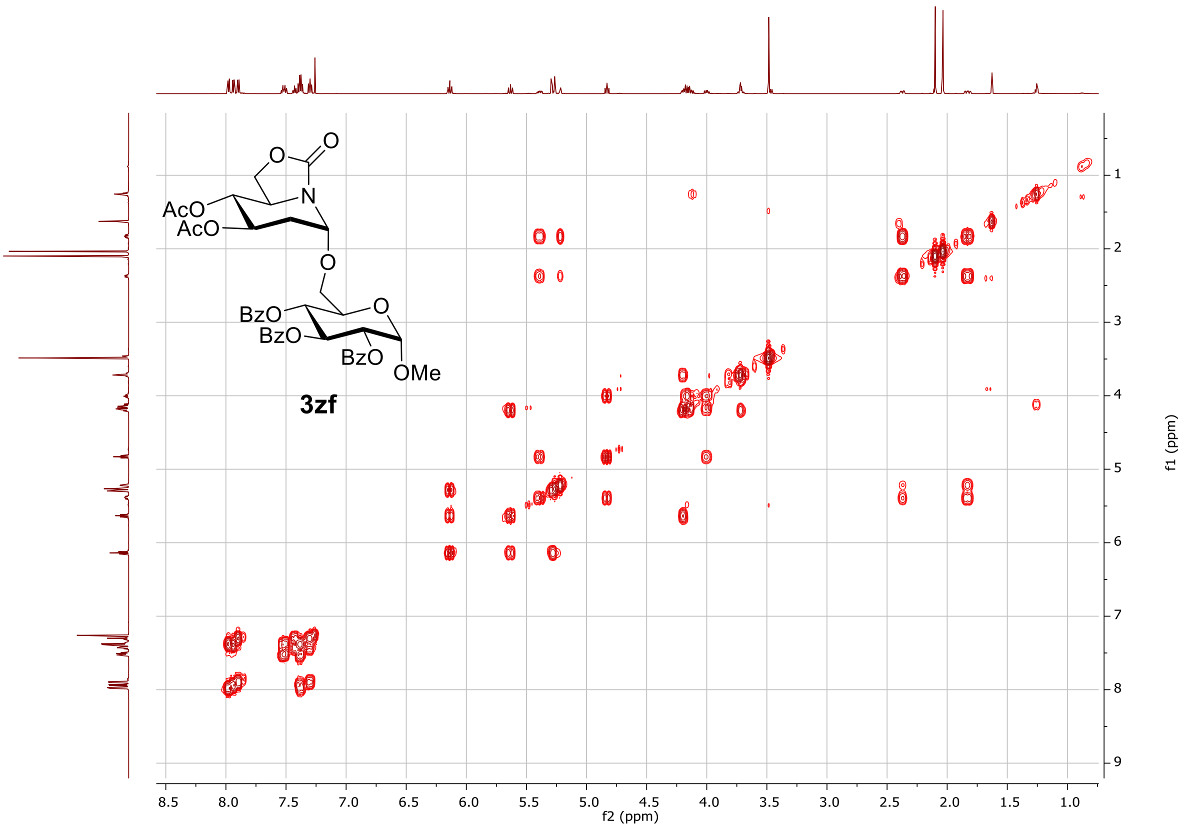

Supplementary Figure S379. COSY spectra for 3zf

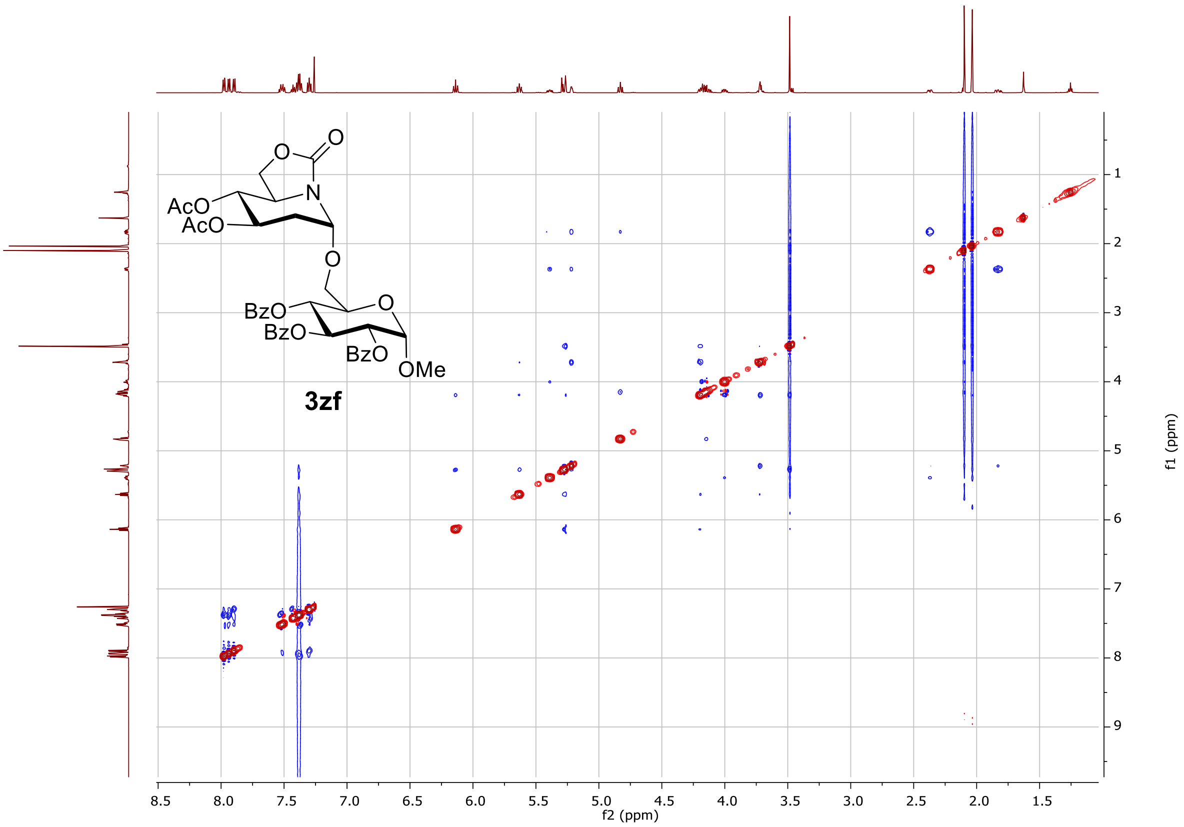

Supplementary Figure S380. NOESY spectra for 3zf

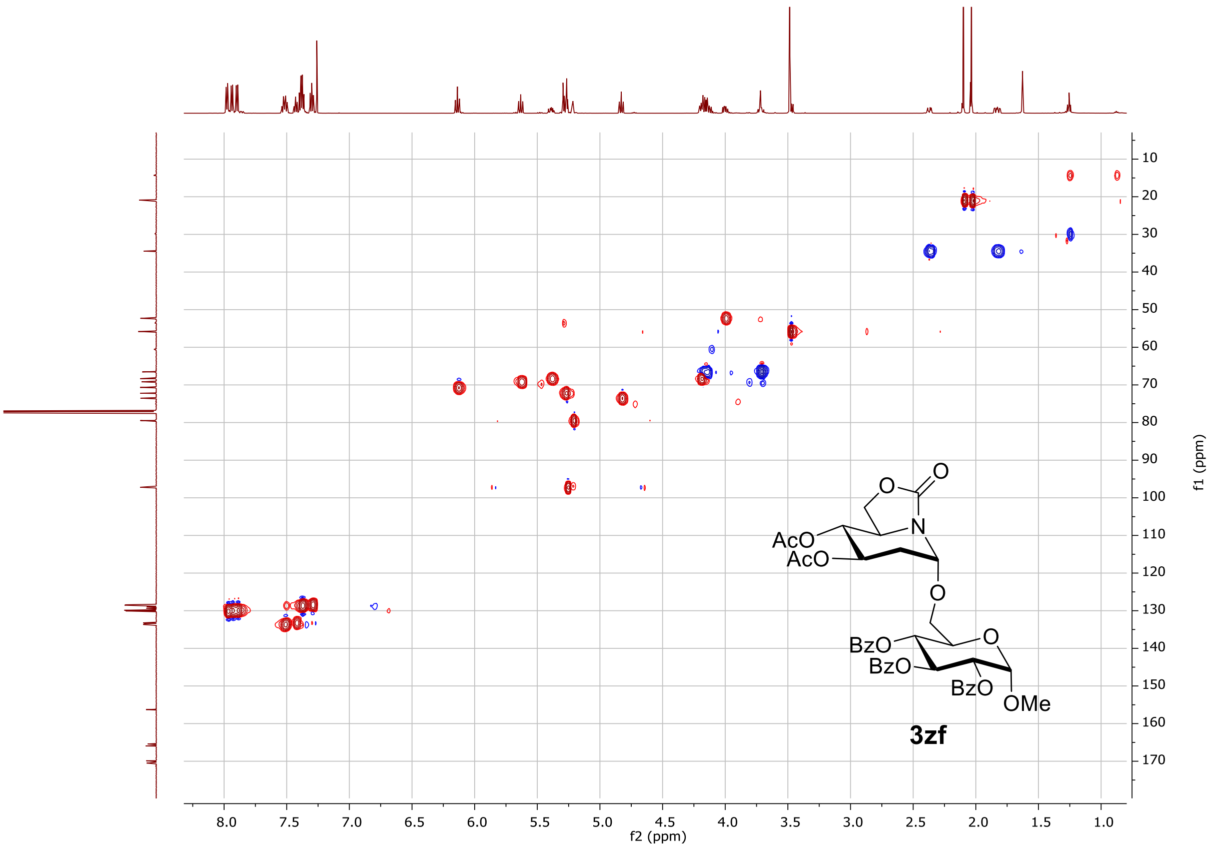

Supplementary Figure S381. HSQC spectra for 3zf

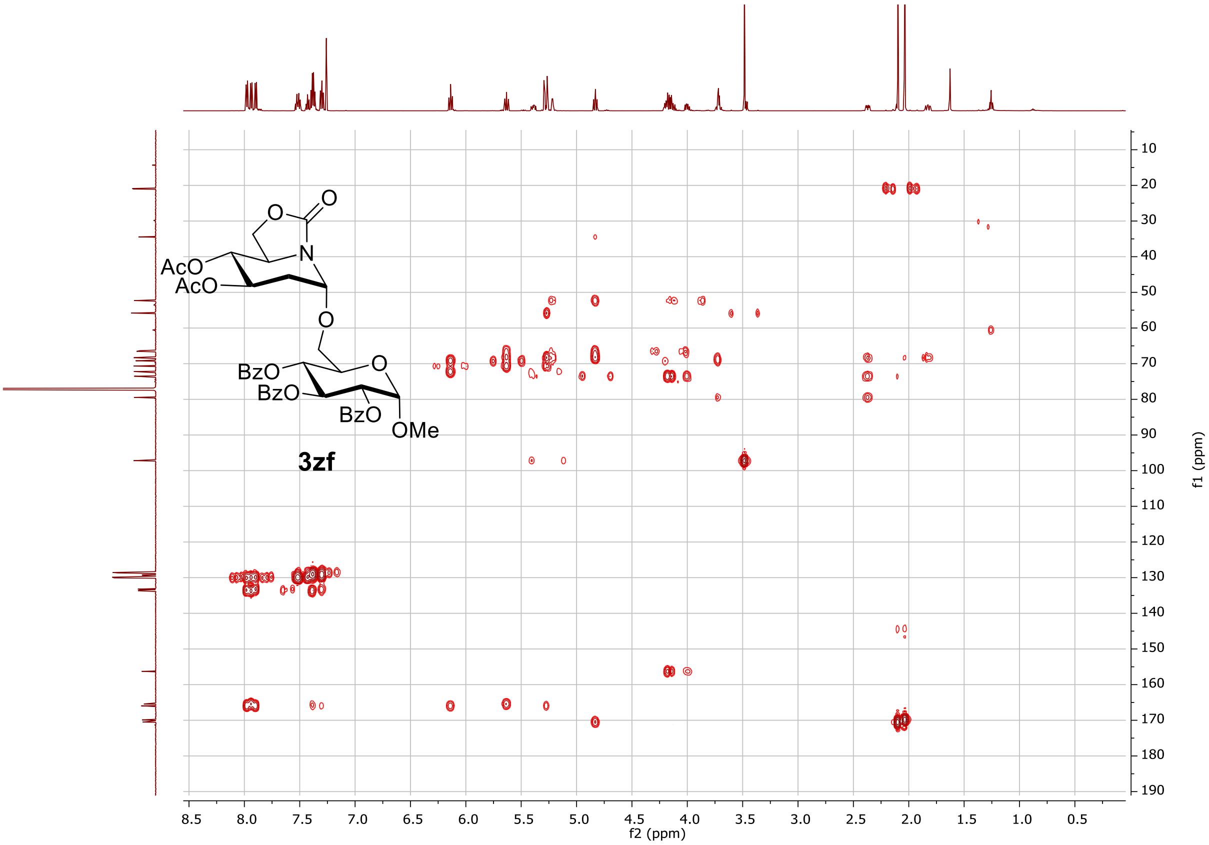

Supplementary Figure S382. HMBC spectra for 3zf

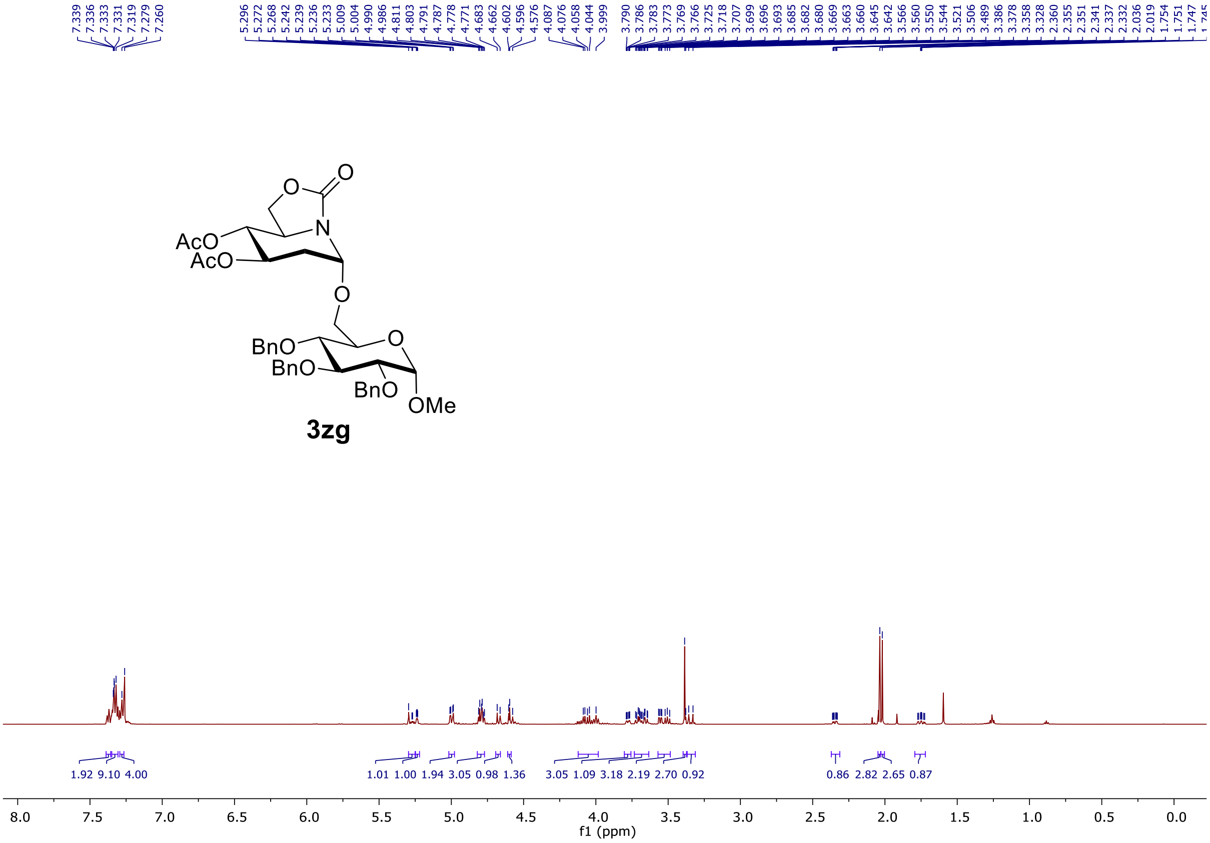

Supplementary Figure S383. ^{1}**H NMR spectra for 3zg**

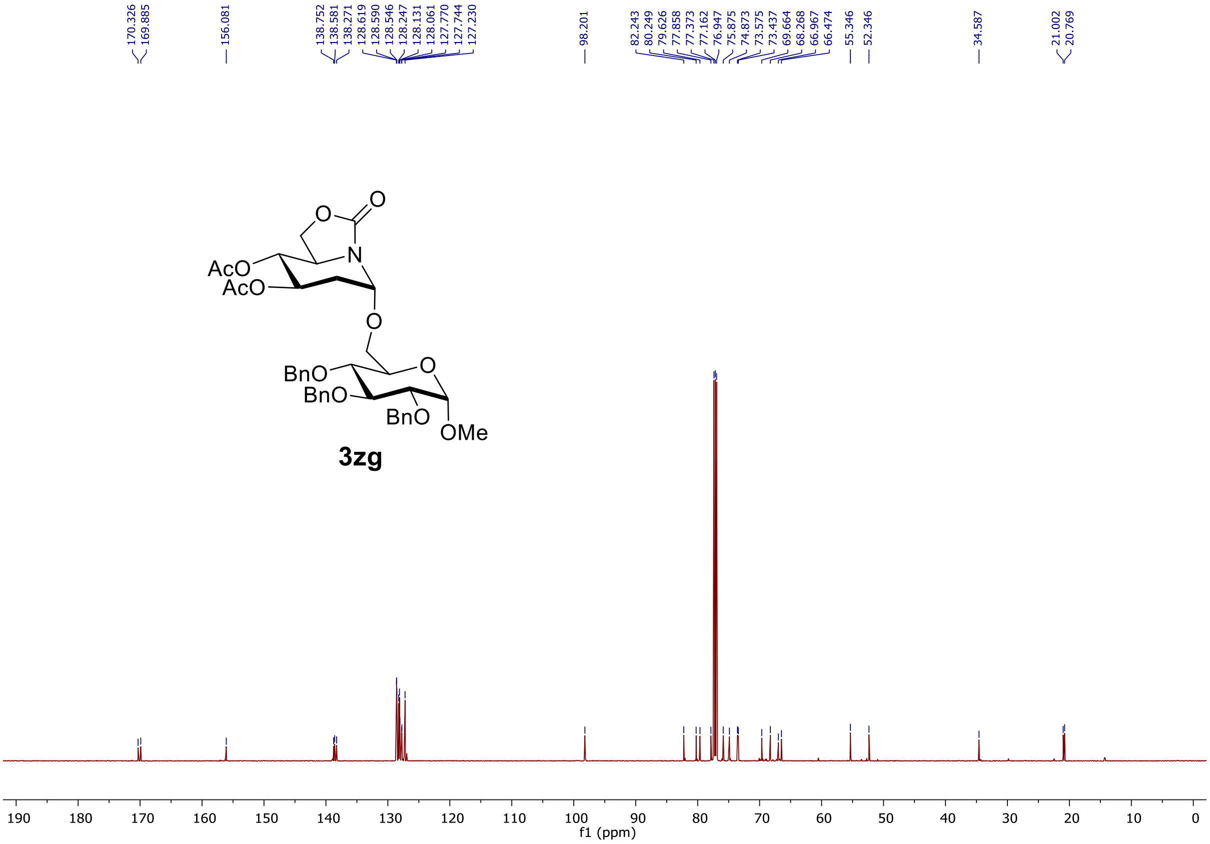

Supplementary Figure S384. ^{13}**C NMR spectra for 3zg**

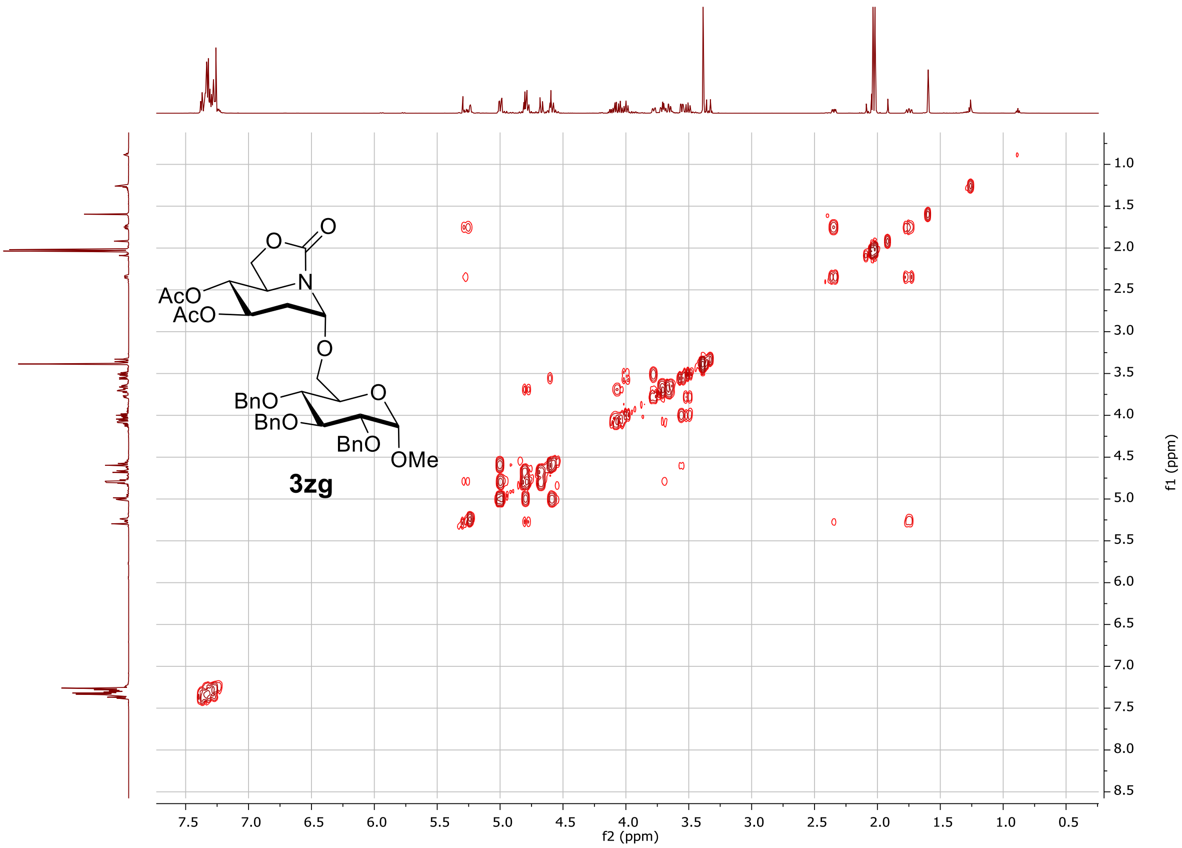

Supplementary Figure S385. COSY spectra for 3zg

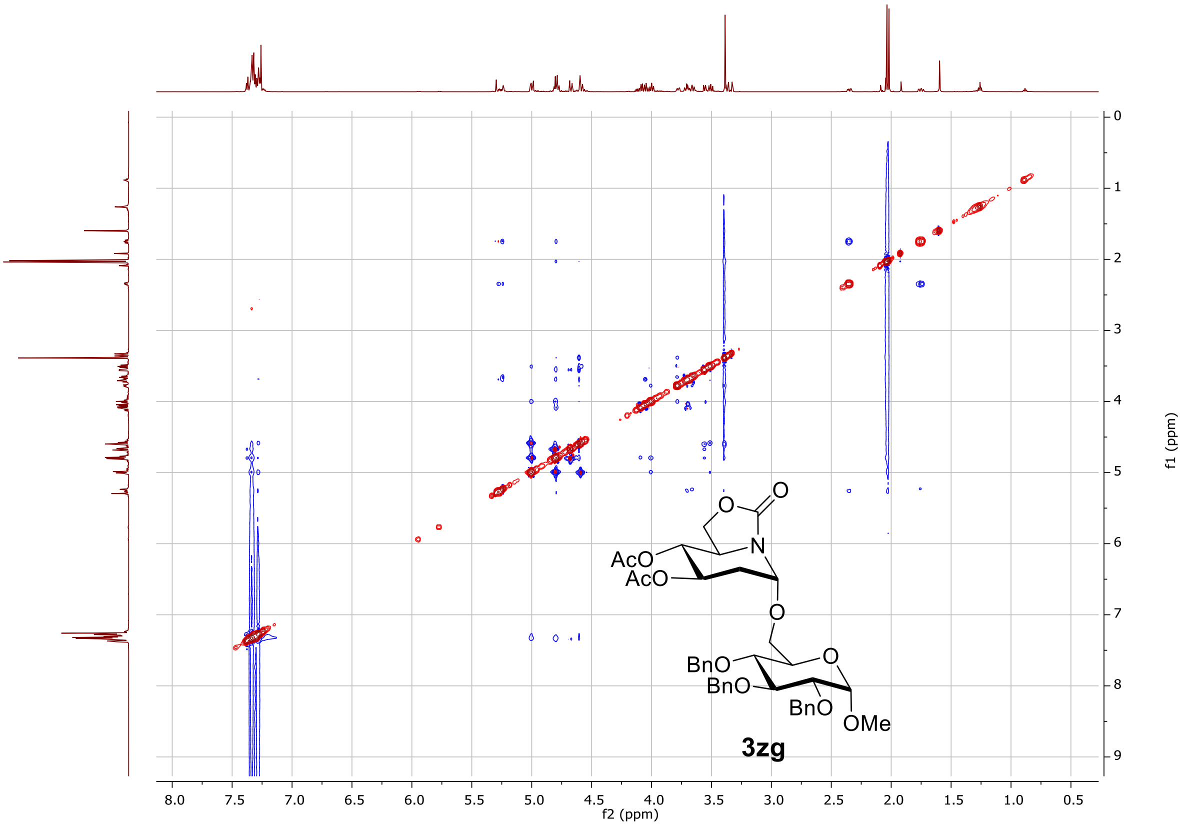

Supplementary Figure S386. NOESY spectra for 3zg

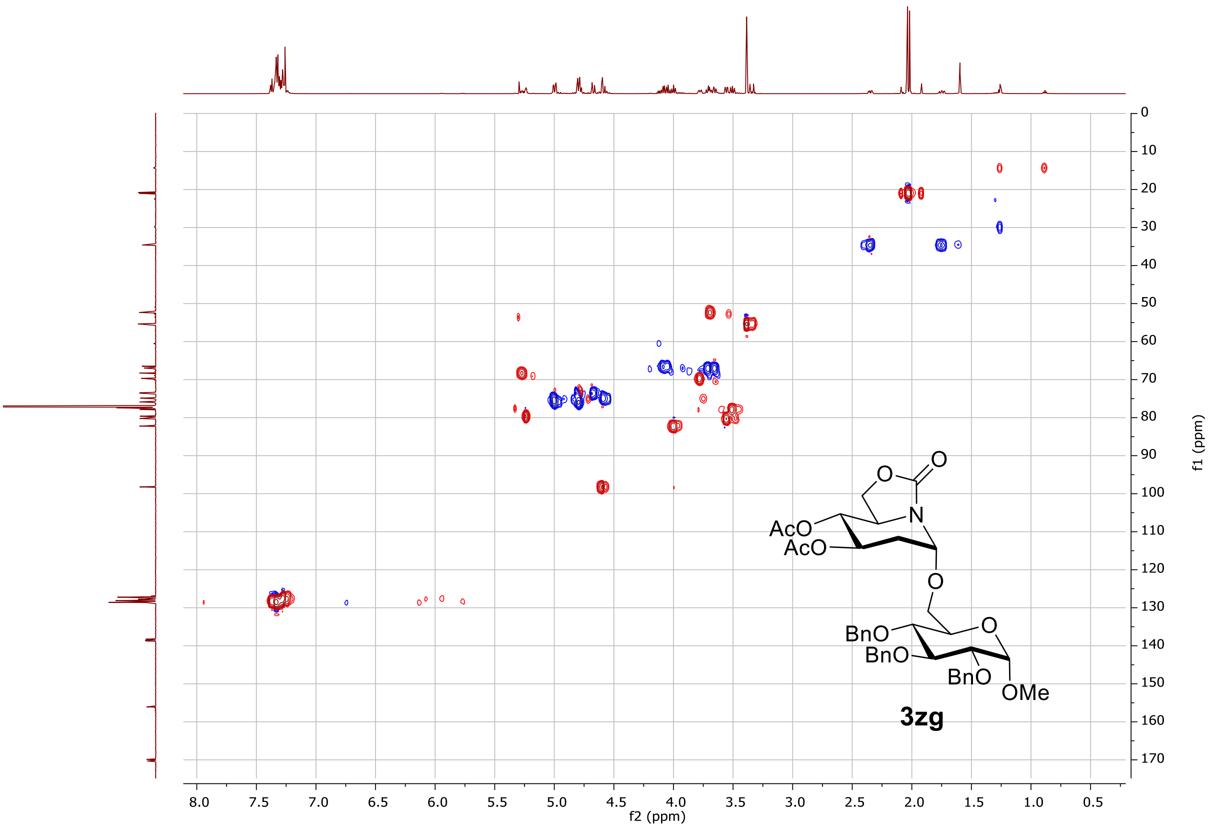

Supplementary Figure S387. HSQC spectra for 3zg

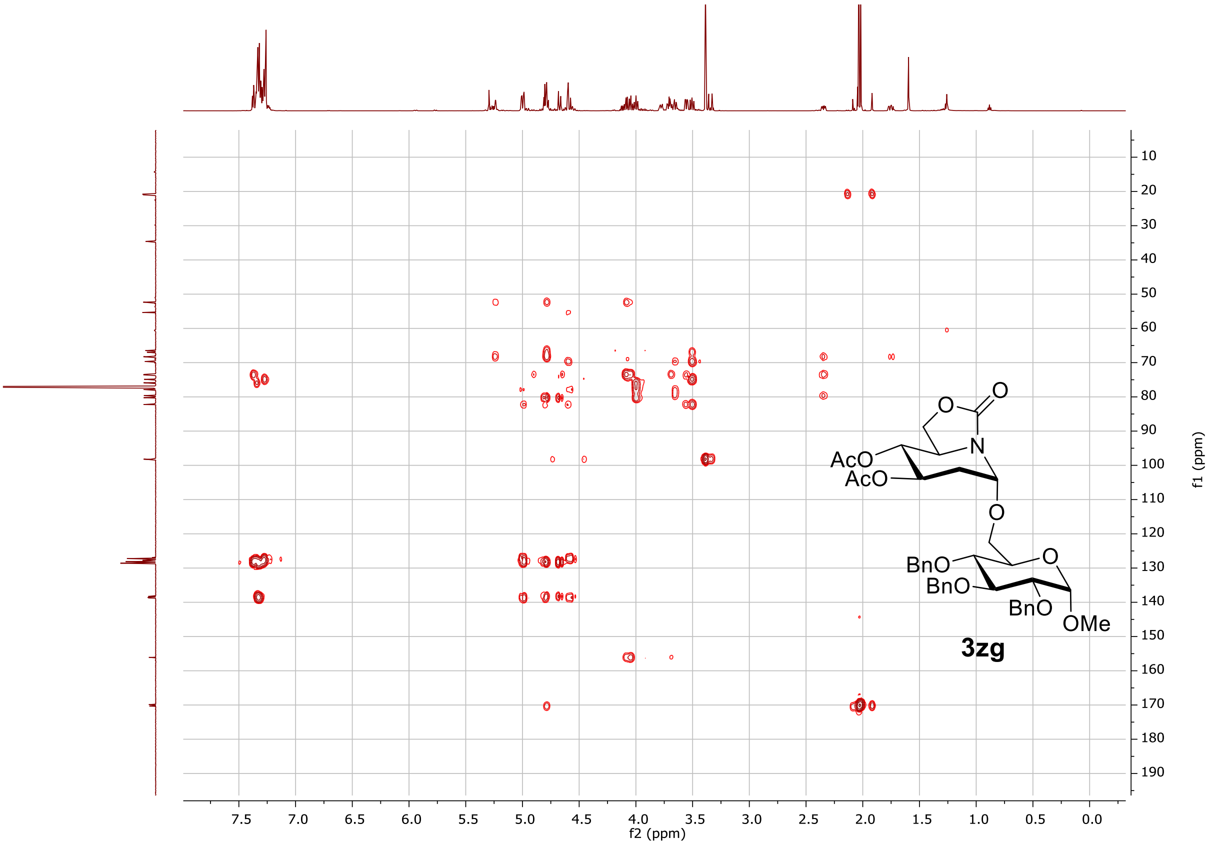

Supplementary Figure S388. HMBC spectra for 3zg

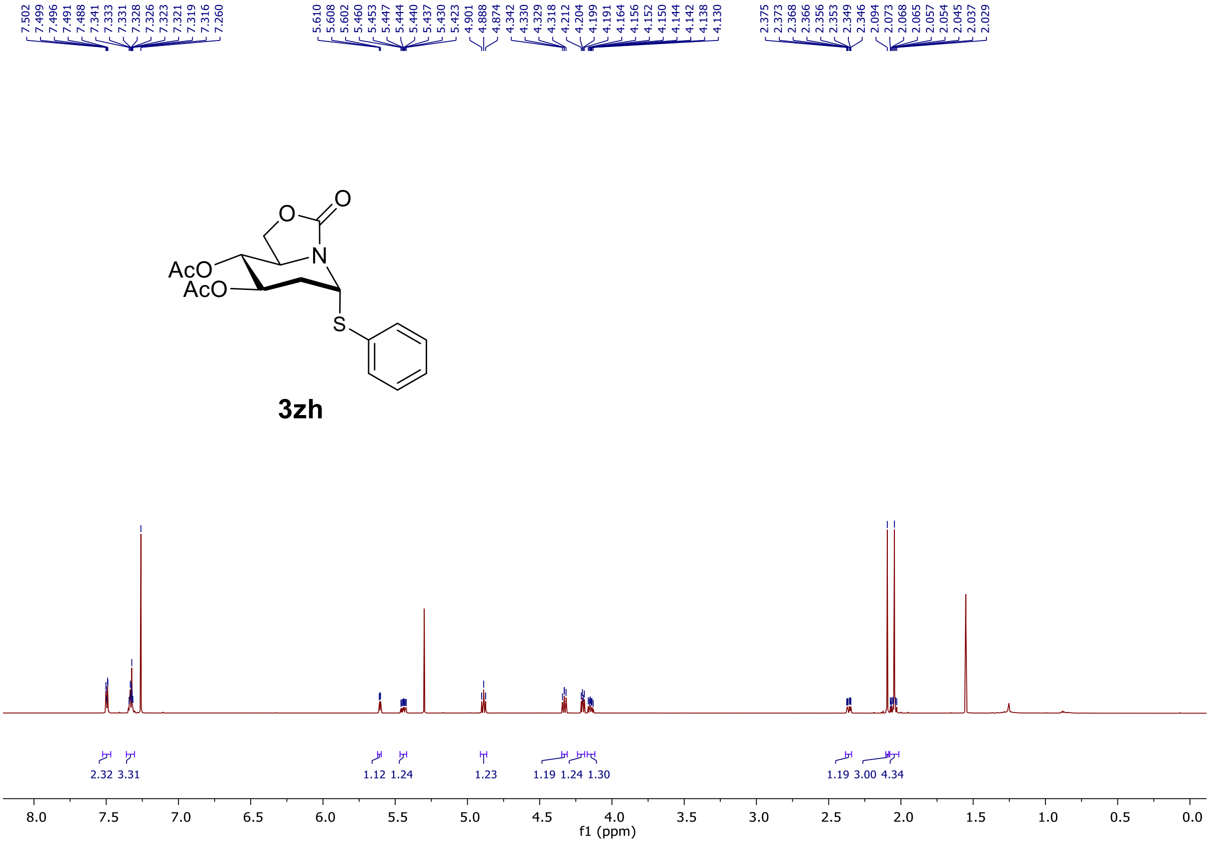

Supplementary Figure S389. ^{1}H NMR spectra for 3zh

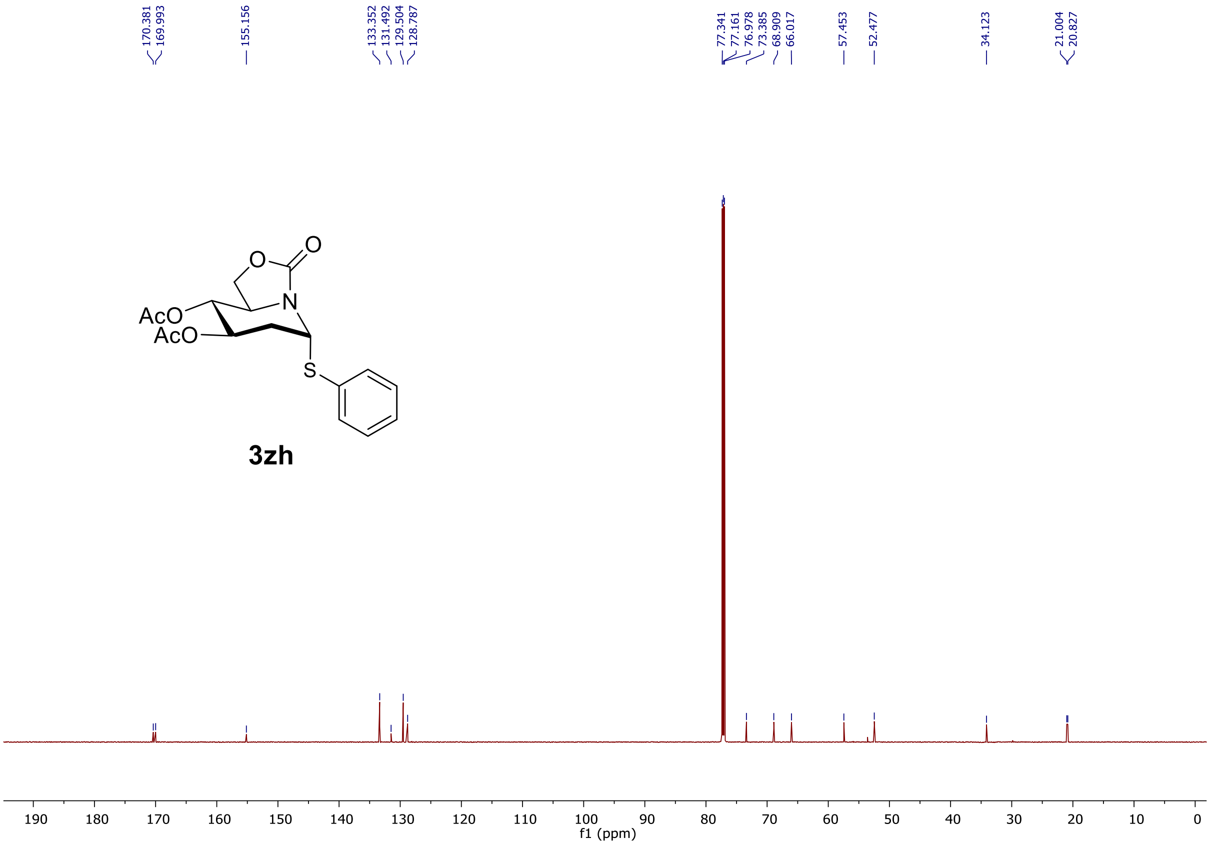

Supplementary Figure S390. ^{13}C NMR spectra for 3zh

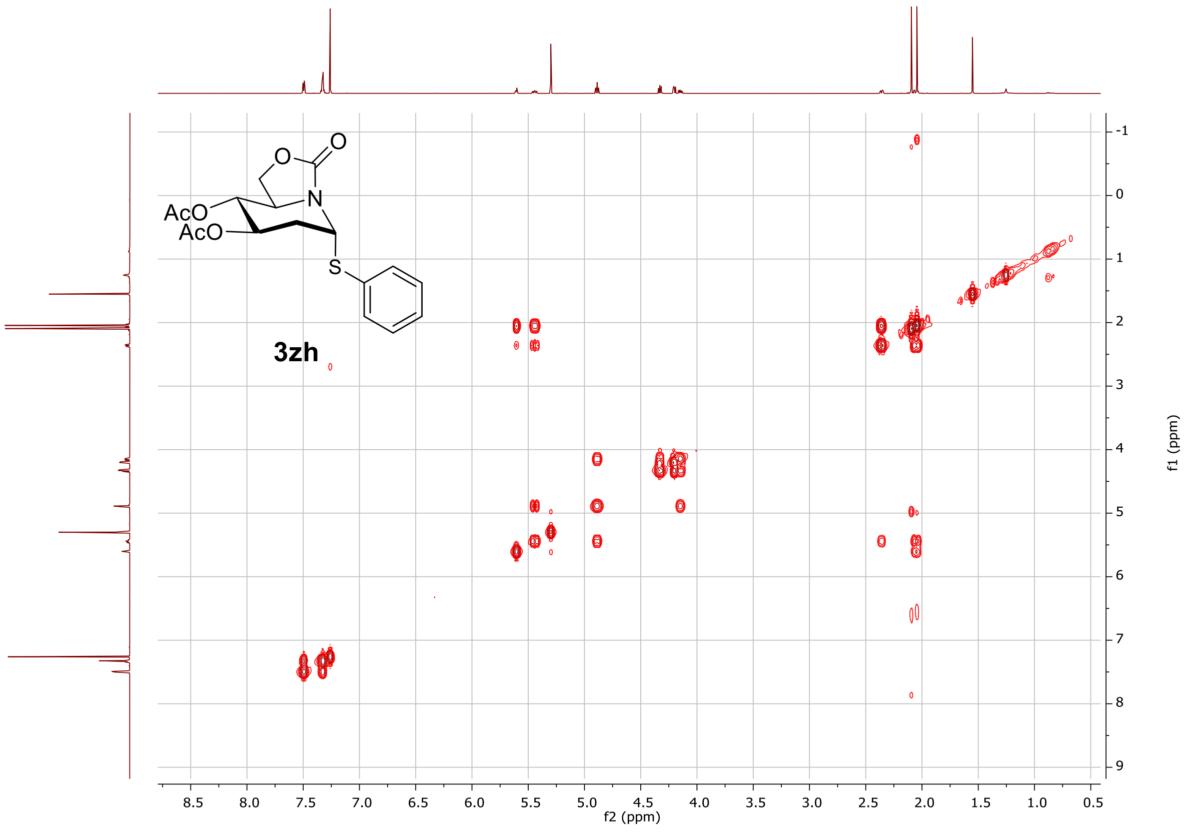

Supplementary Figure S391. COSY spectra for 3zh

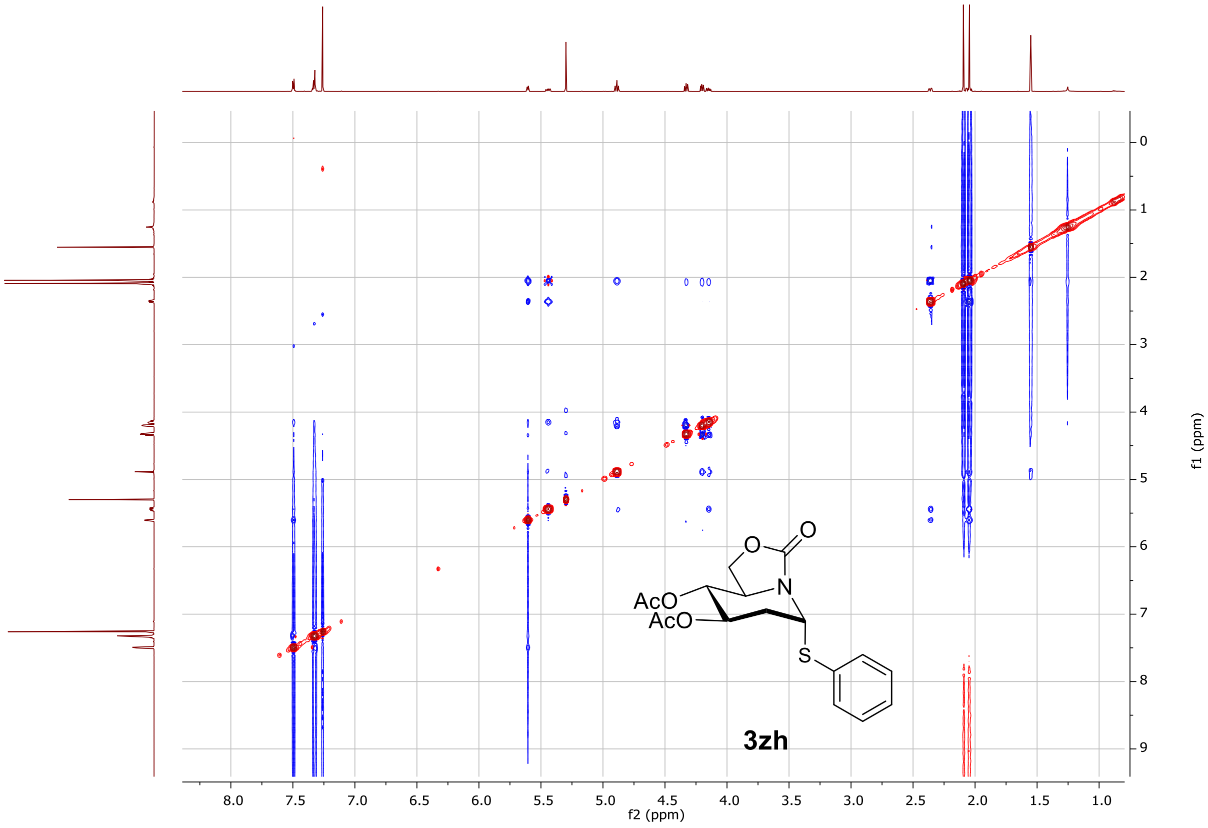

Supplementary Figure S392. NOESY spectra for 3zh

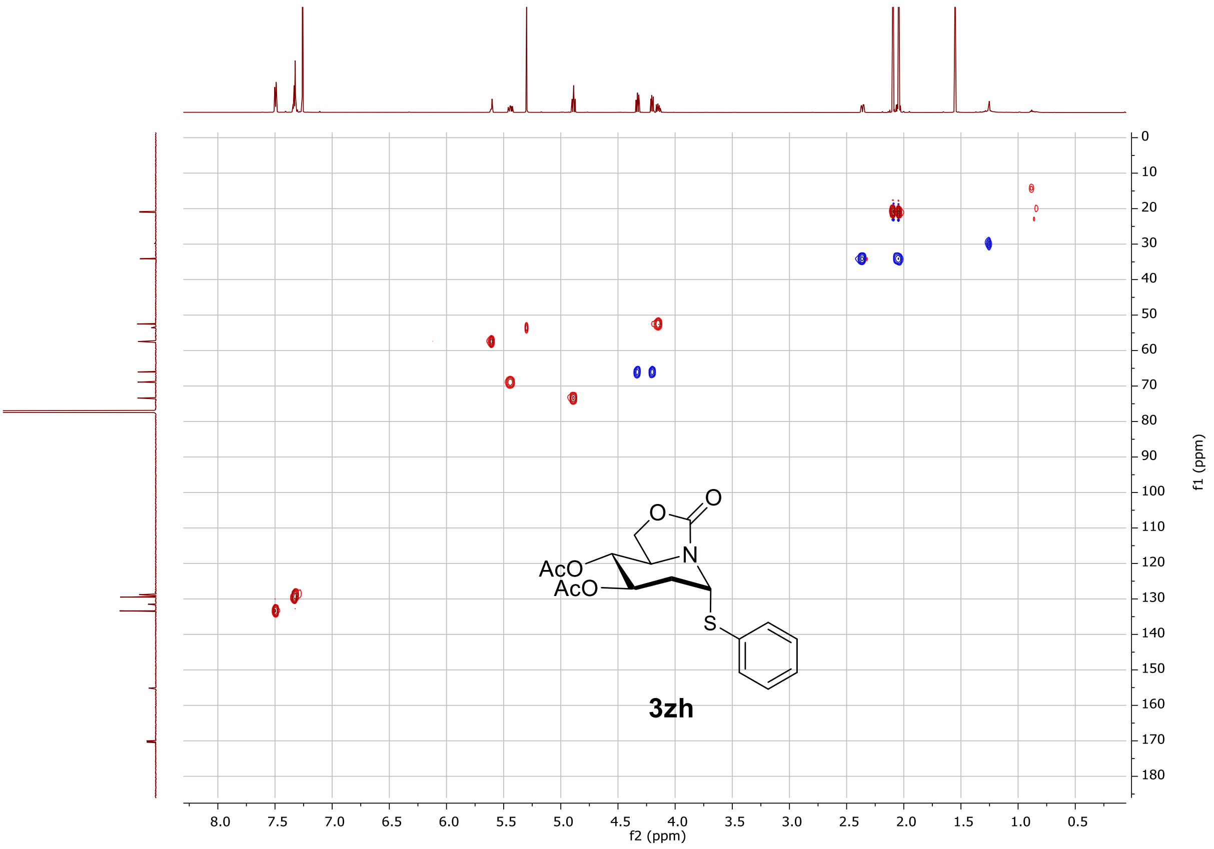

Supplementary Figure S393. HSQC spectra for 3zh

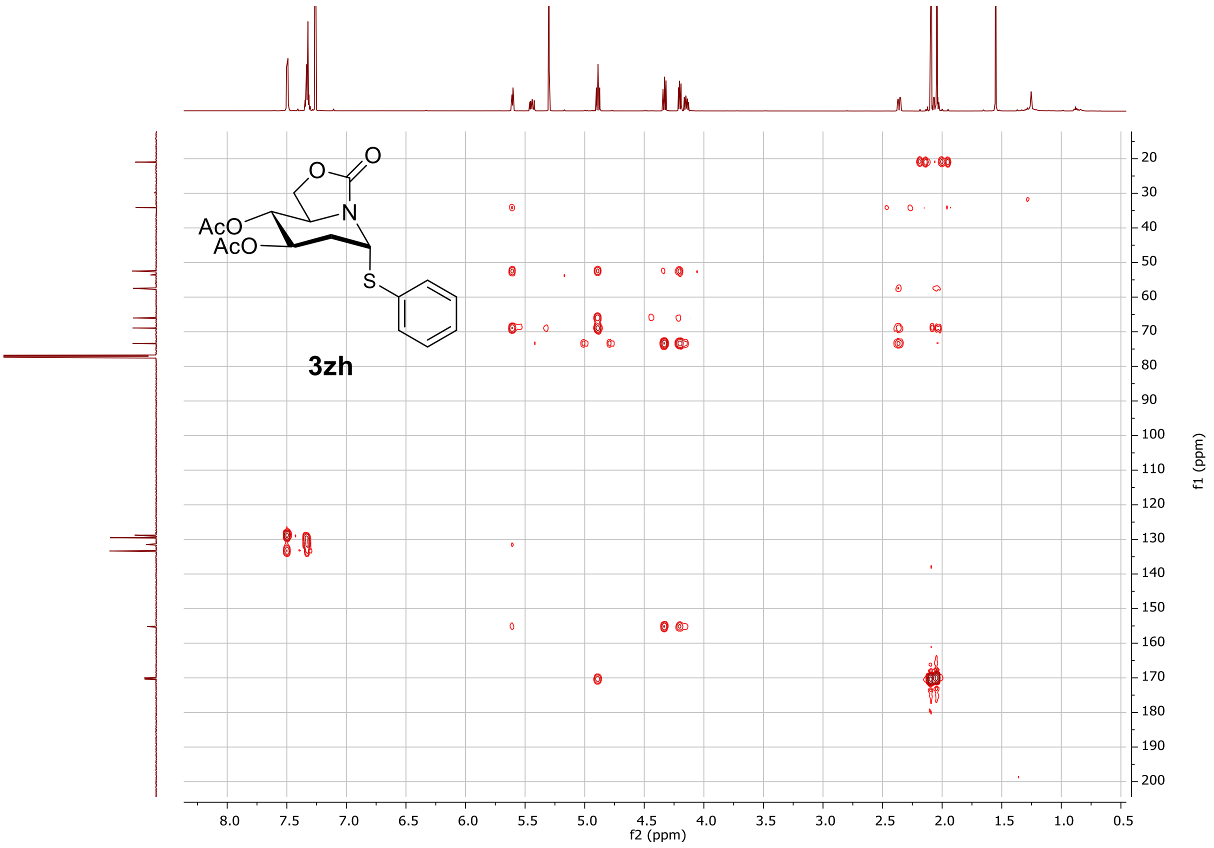

Supplementary Figure S394. HMBC spectra for 3zh

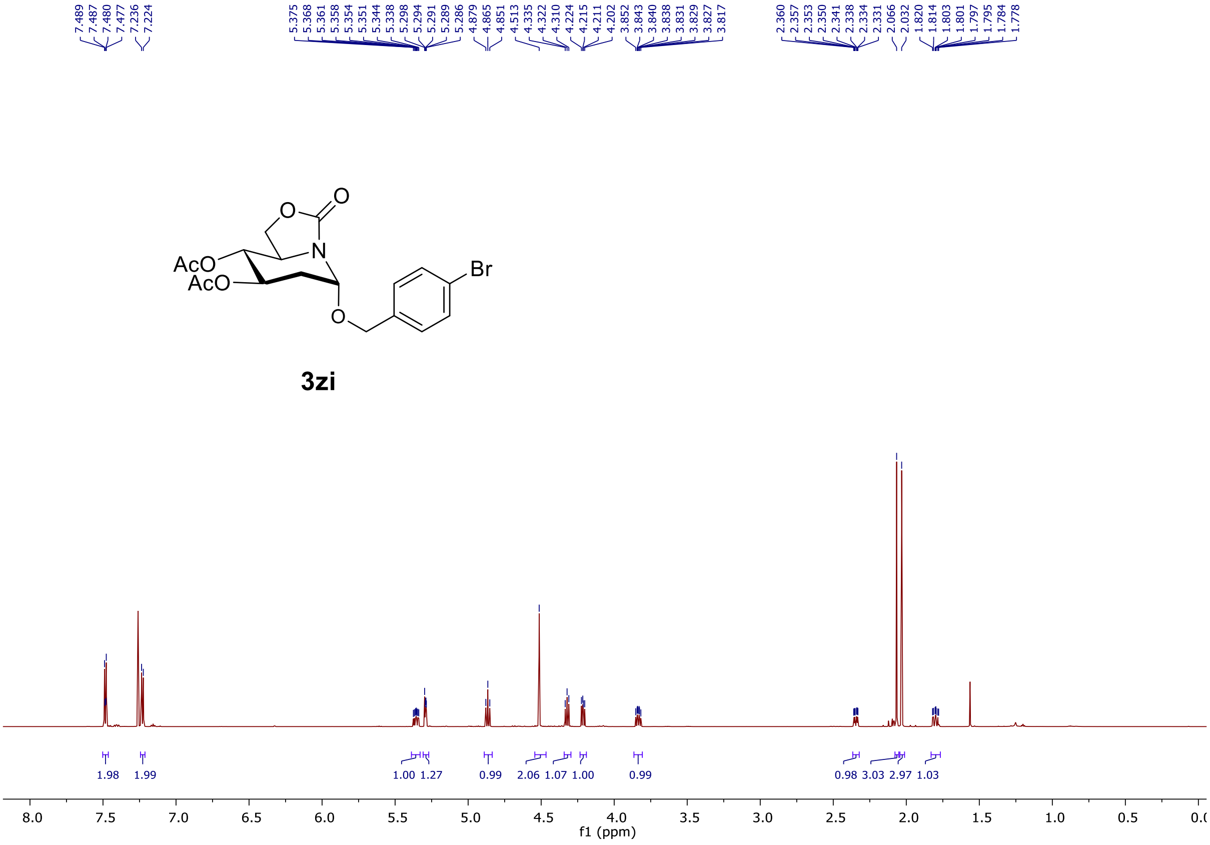

Supplementary Figure S395. ^{1}H NMR spectra for 3zi

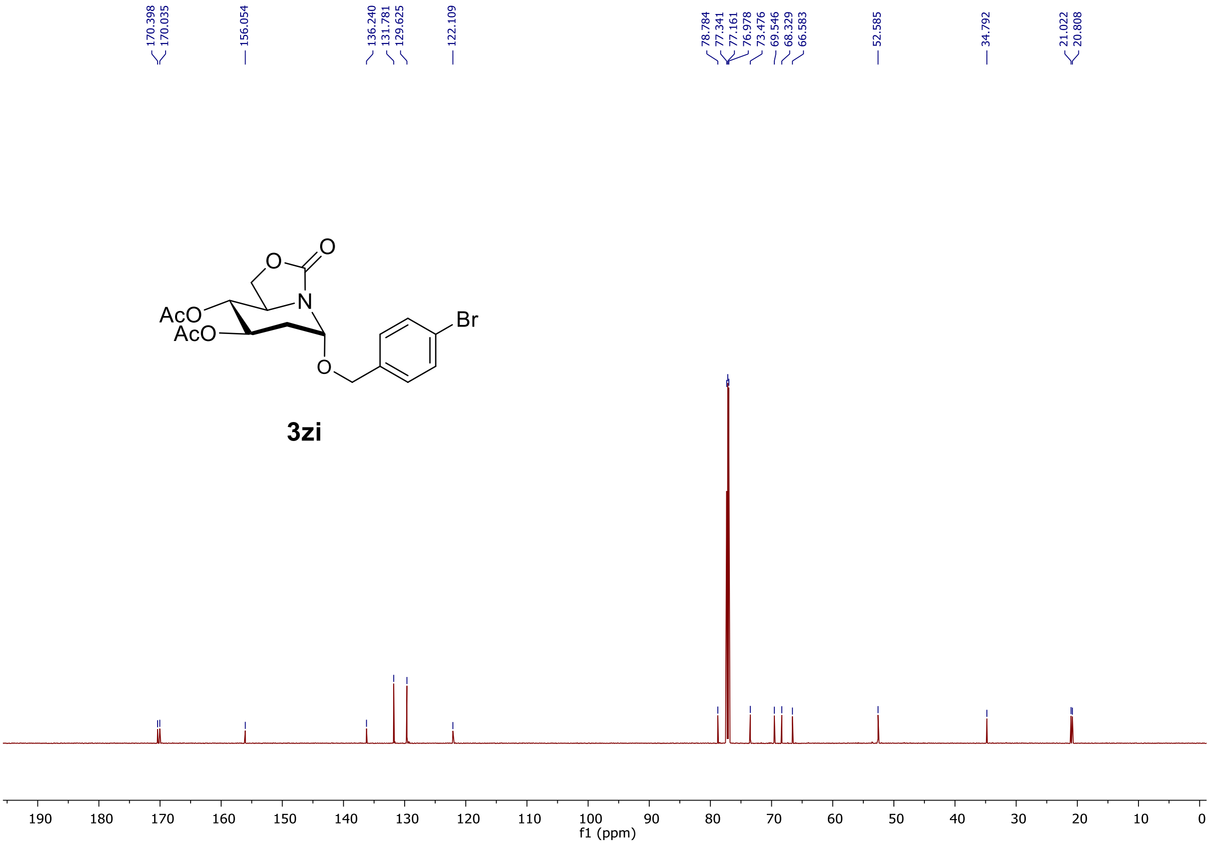

Supplementary Figure S396. ^{13}C NMR spectra for 3zi

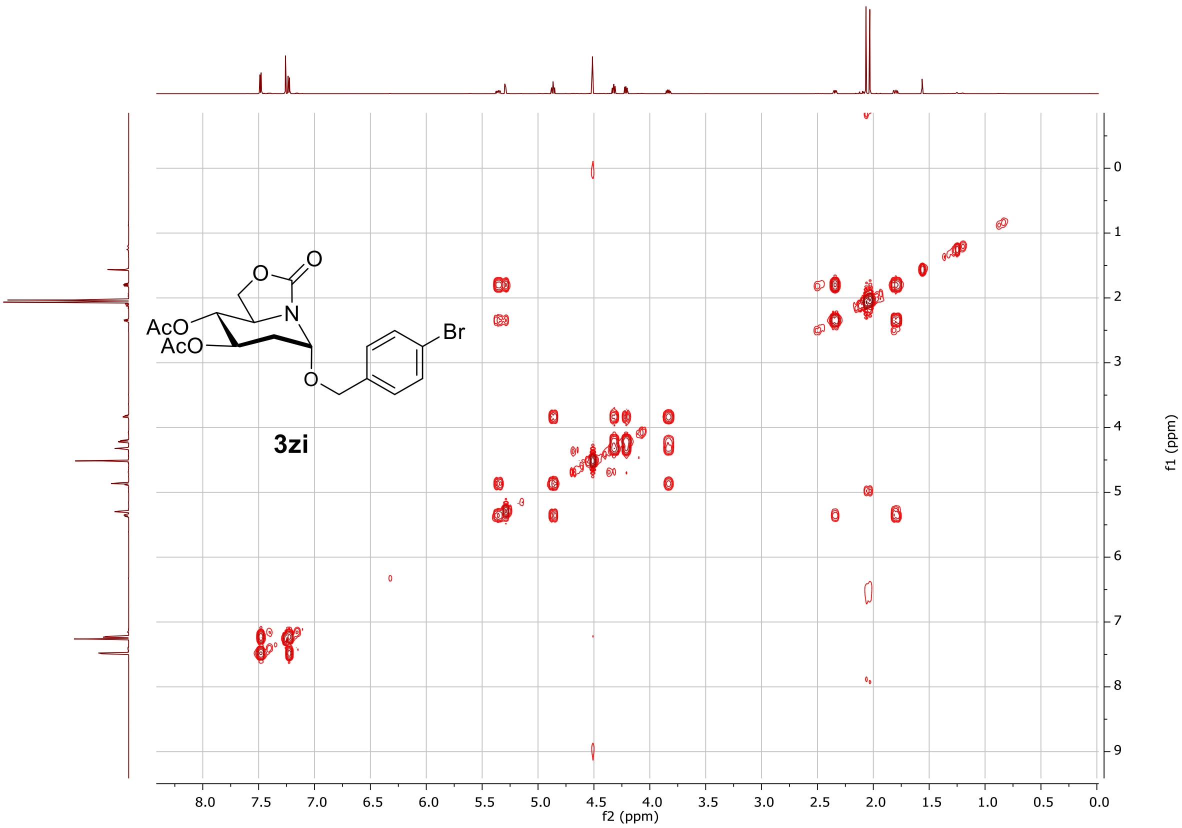

Supplementary Figure S397. COSY spectra for 3zi

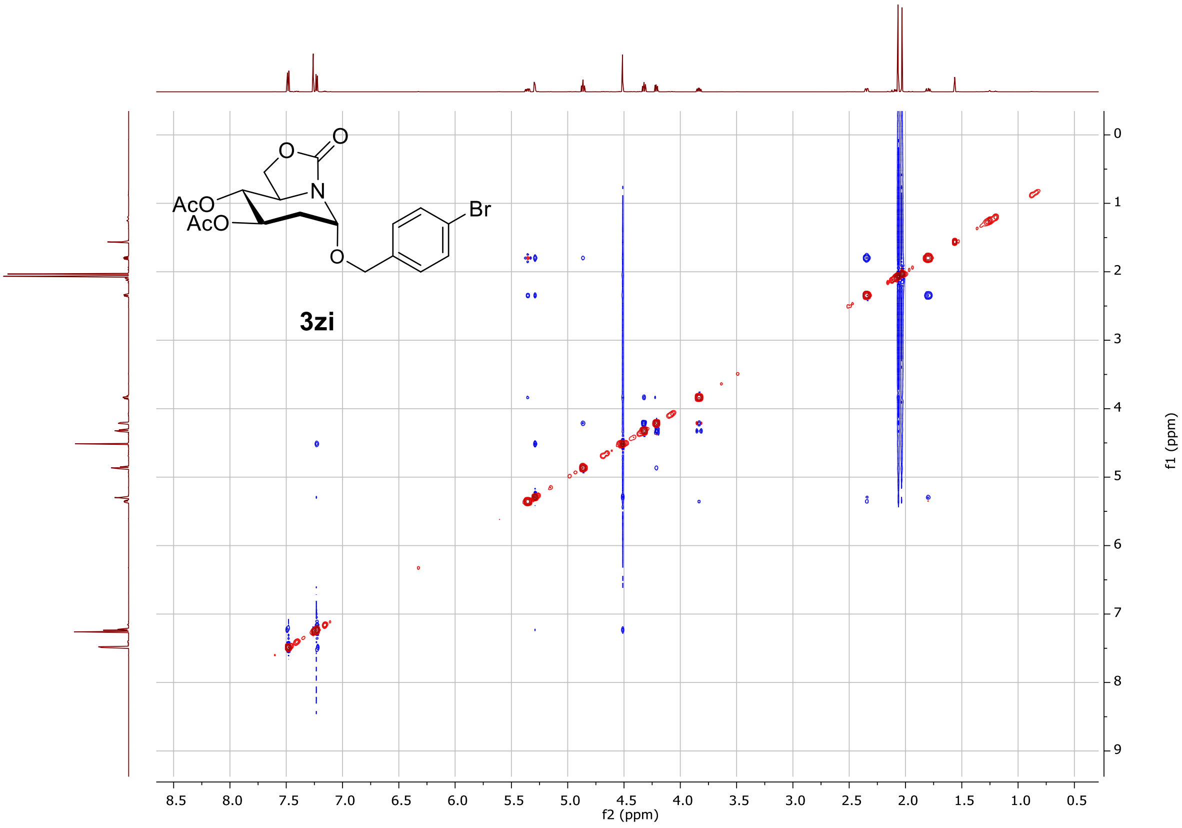

Supplementary Figure S398. NOESY spectra for 3zi

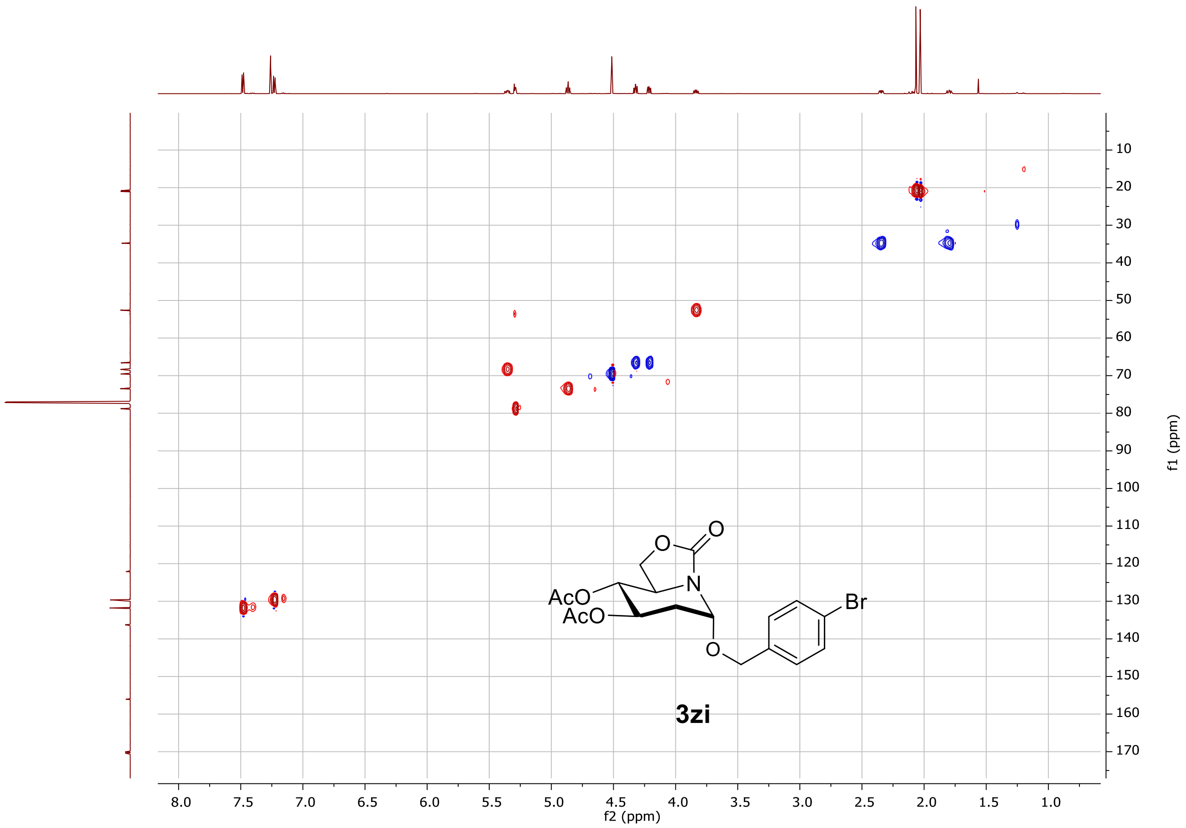

Supplementary Figure S399. HSQC spectra for 3zi

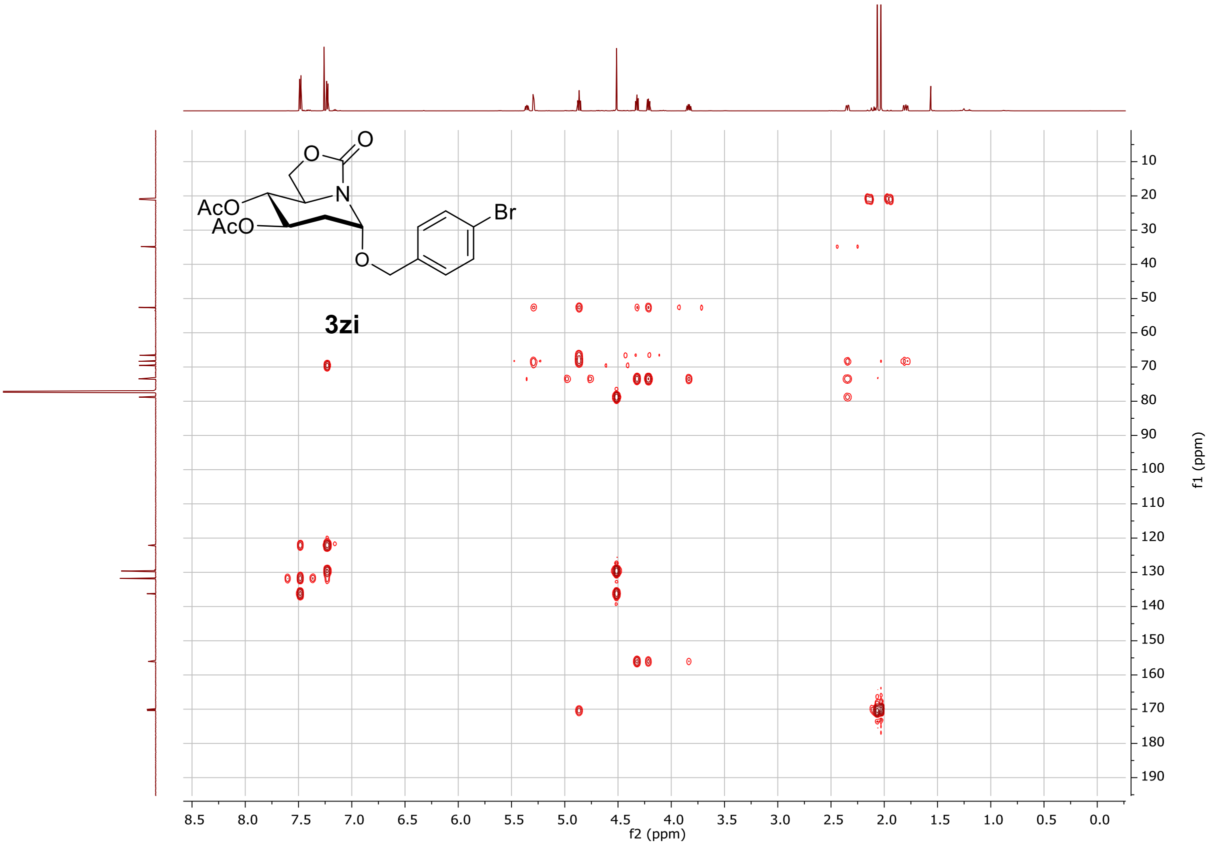

Supplementary Figure S400. HMBC spectra for 3zi

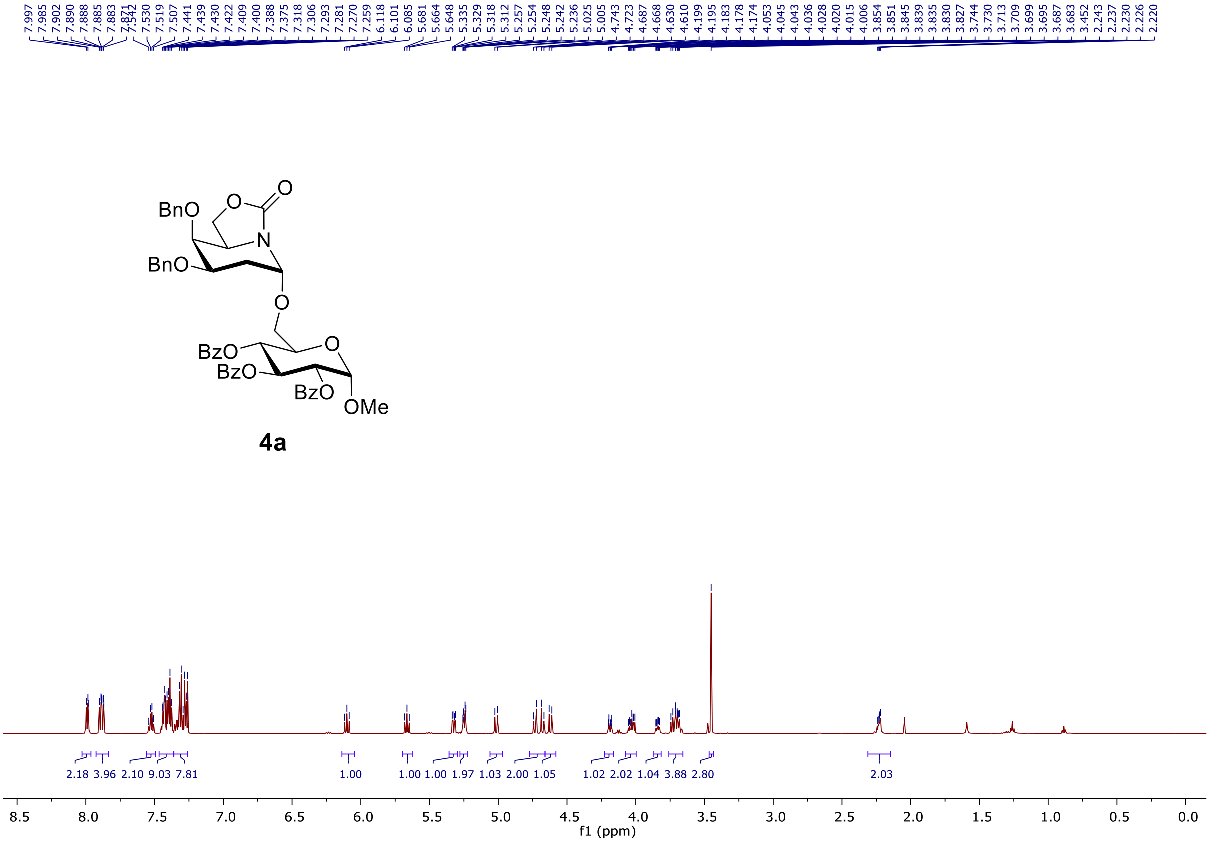

Supplementary Figure S401. ^{1}H NMR spectra for 4a

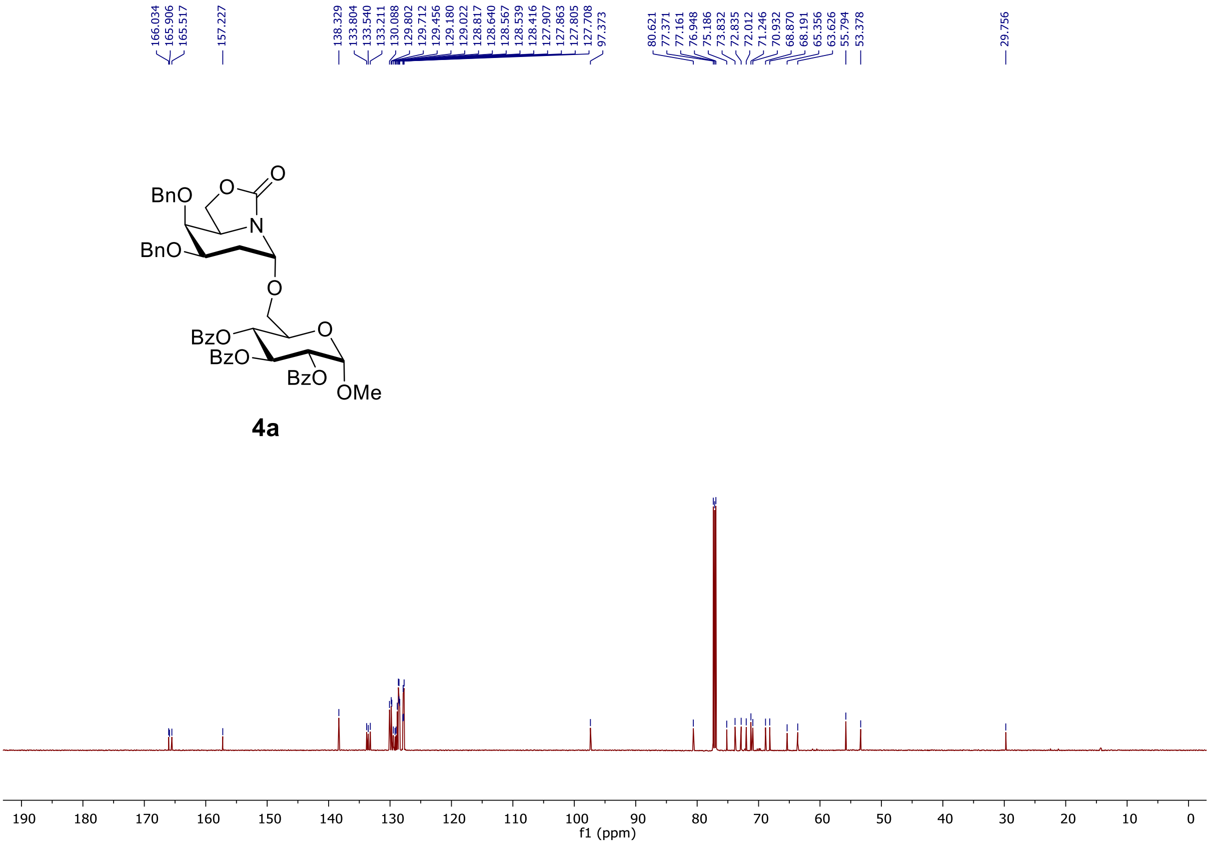

Supplementary Figure S402. ^{13}C NMR spectra for 4a

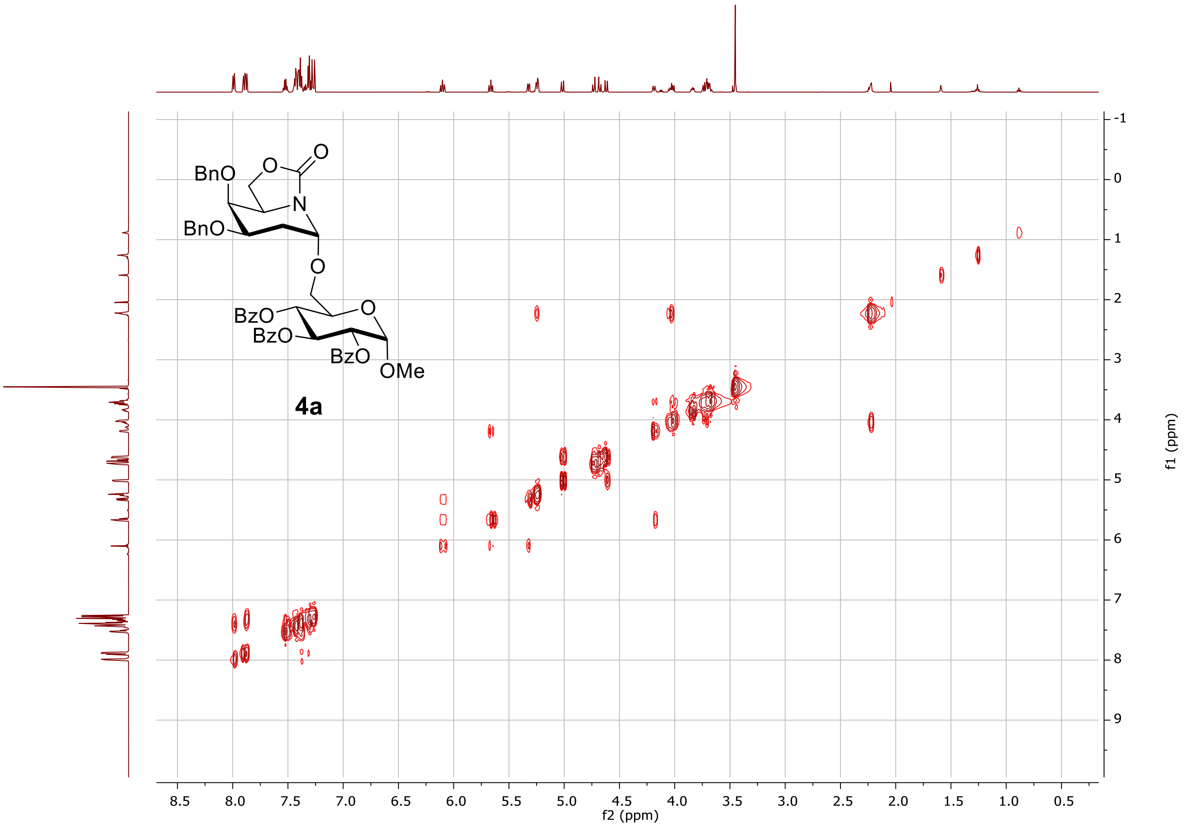

Supplementary Figure S403. COSY spectra for 4a

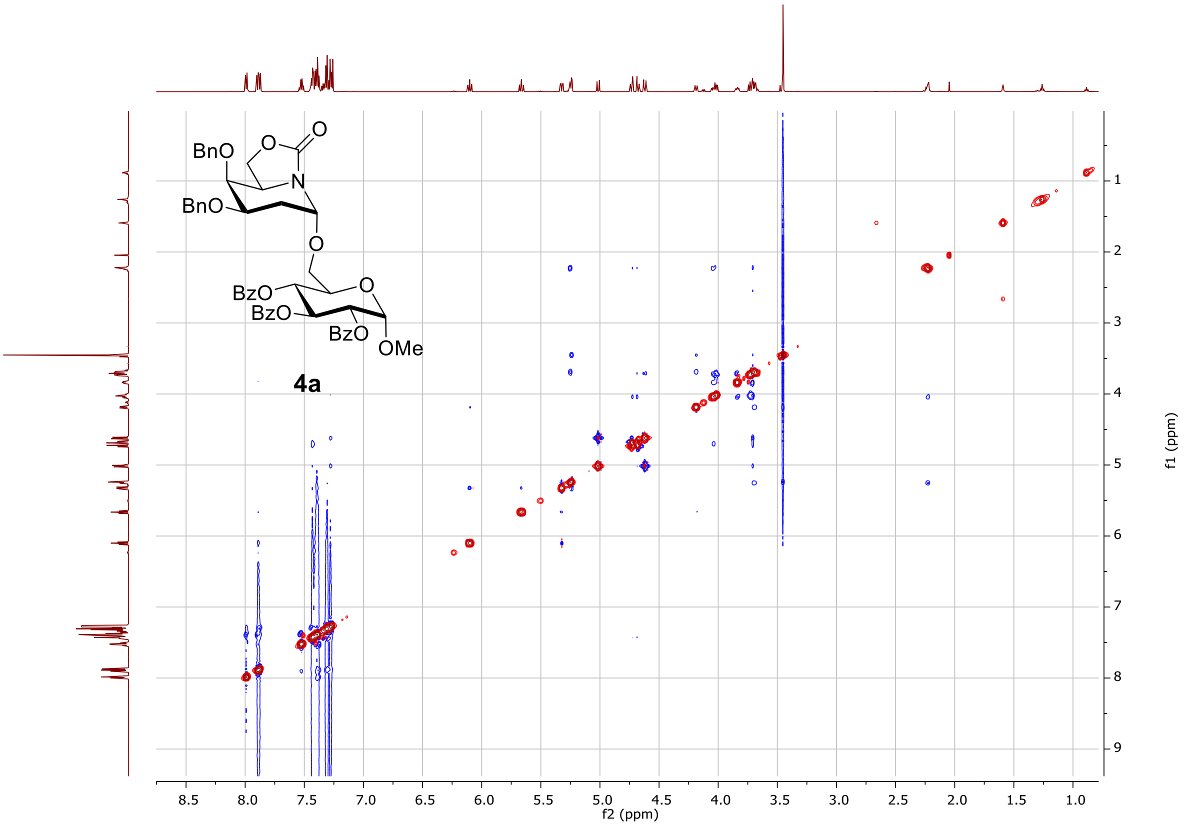

Supplementary Figure S404. NOESY spectra for 4a

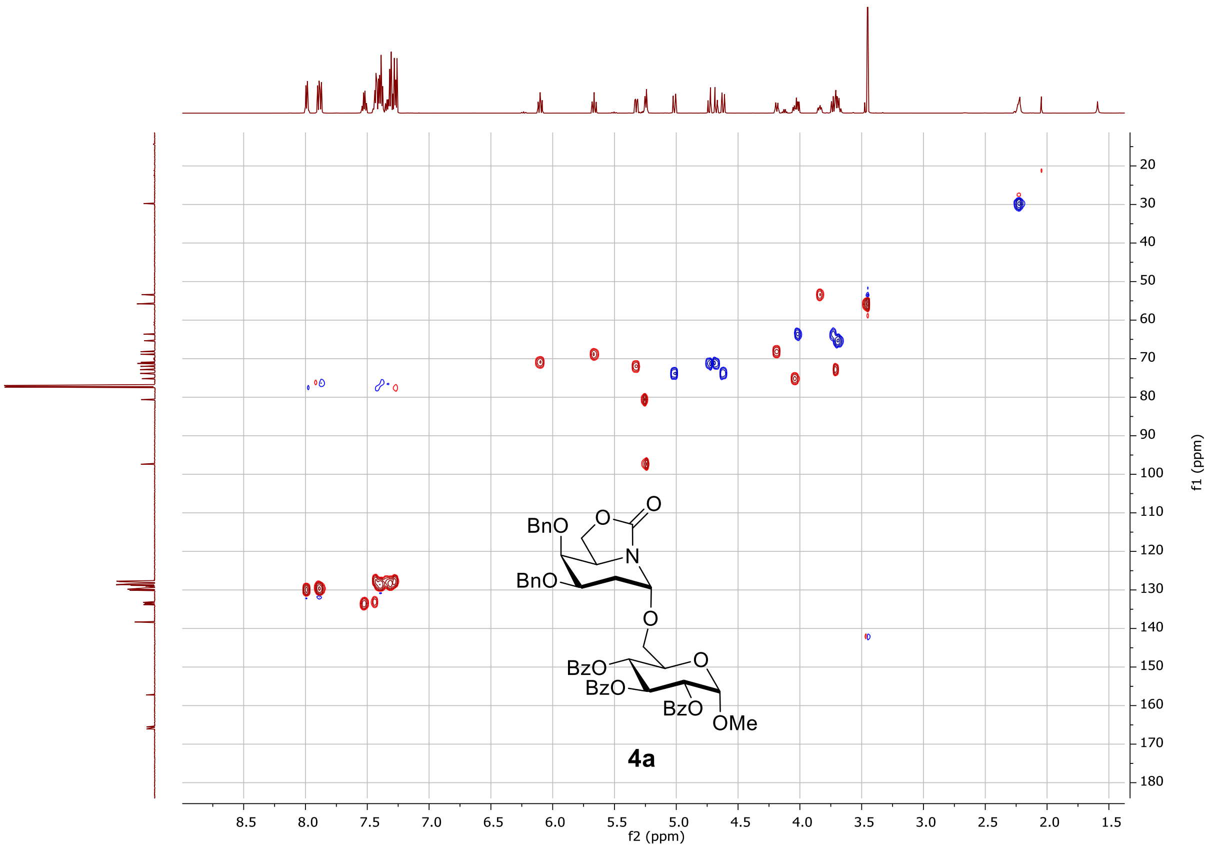

Supplementary Figure S405. HSQC spectra for 4a

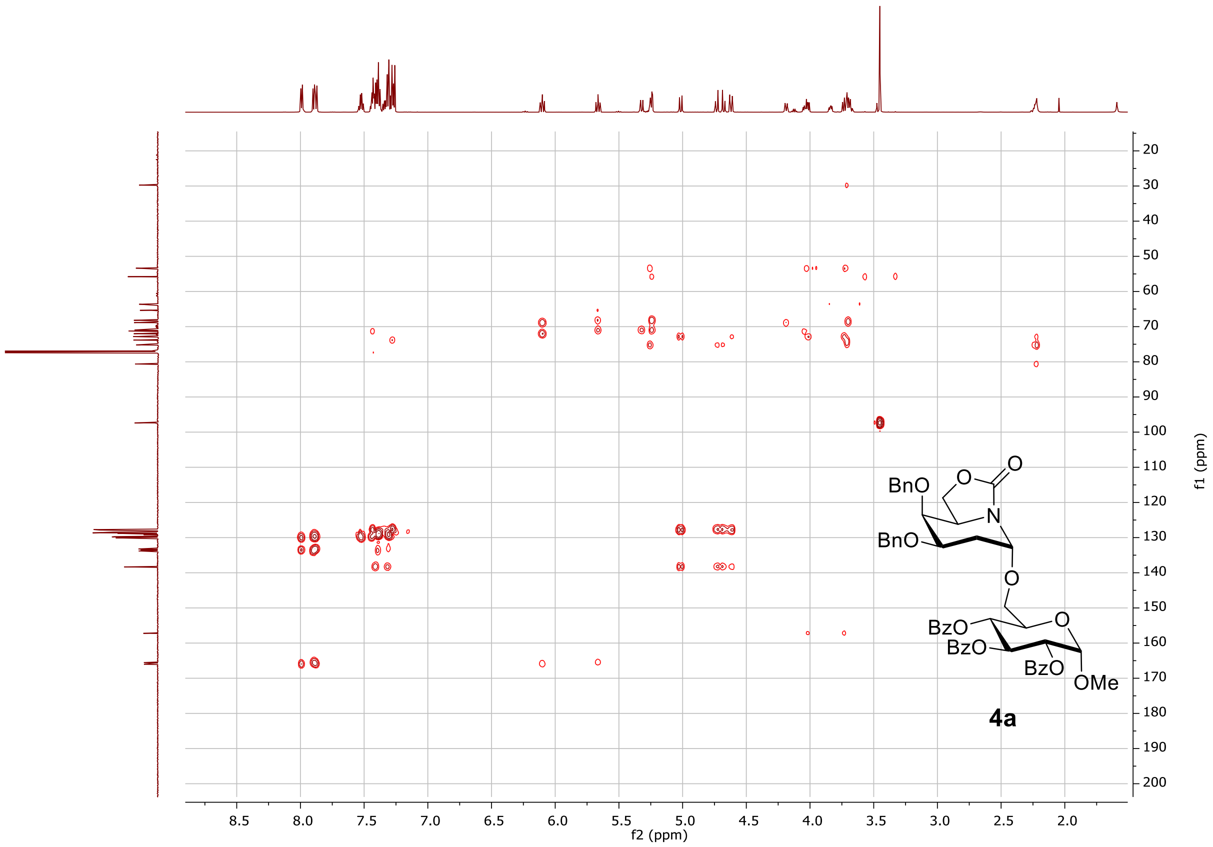

Supplementary Figure S406. HMBC spectra for 4a

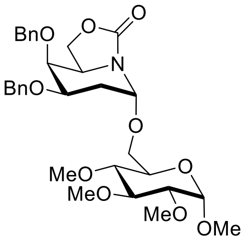

4b

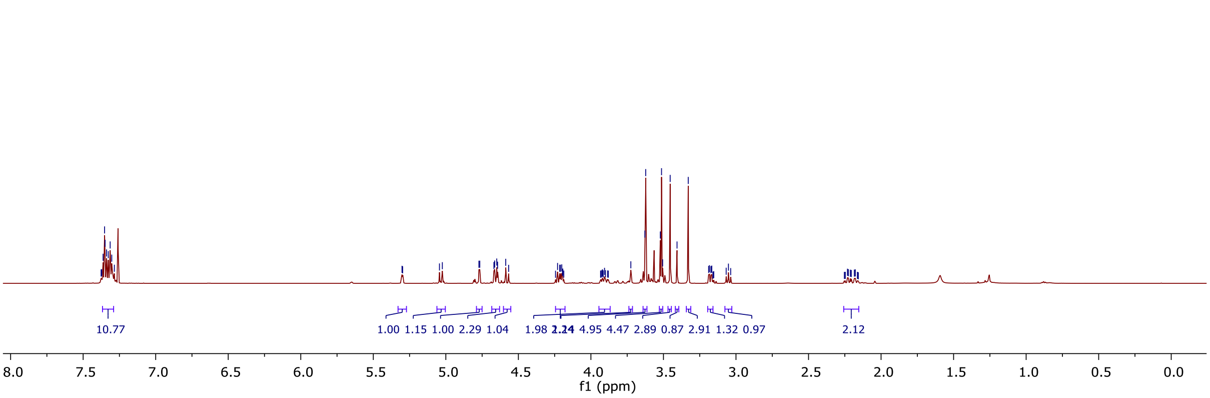

Supplementary Figure S407. ^{1}H NMR spectra for 4b

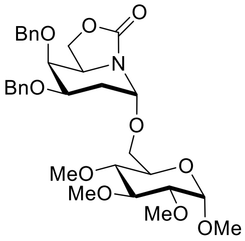

4b

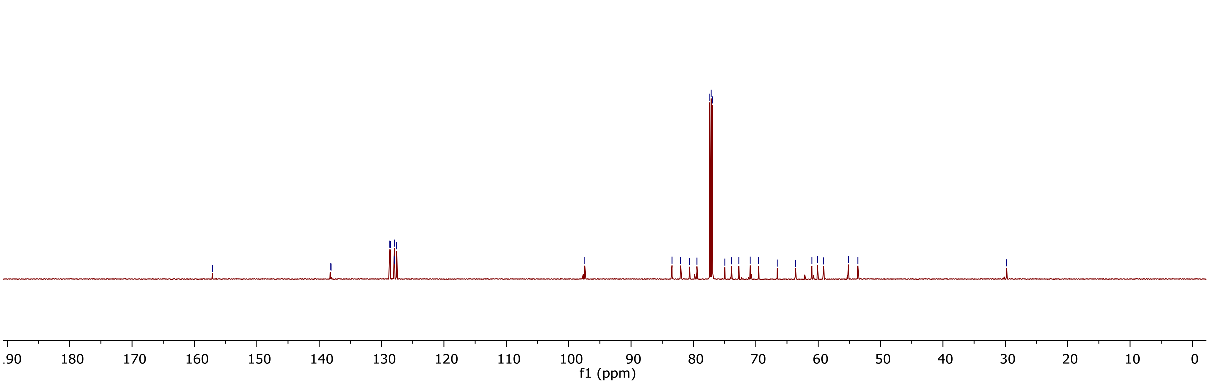

Supplementary Figure S408. ^{13}C NMR spectra for 4b

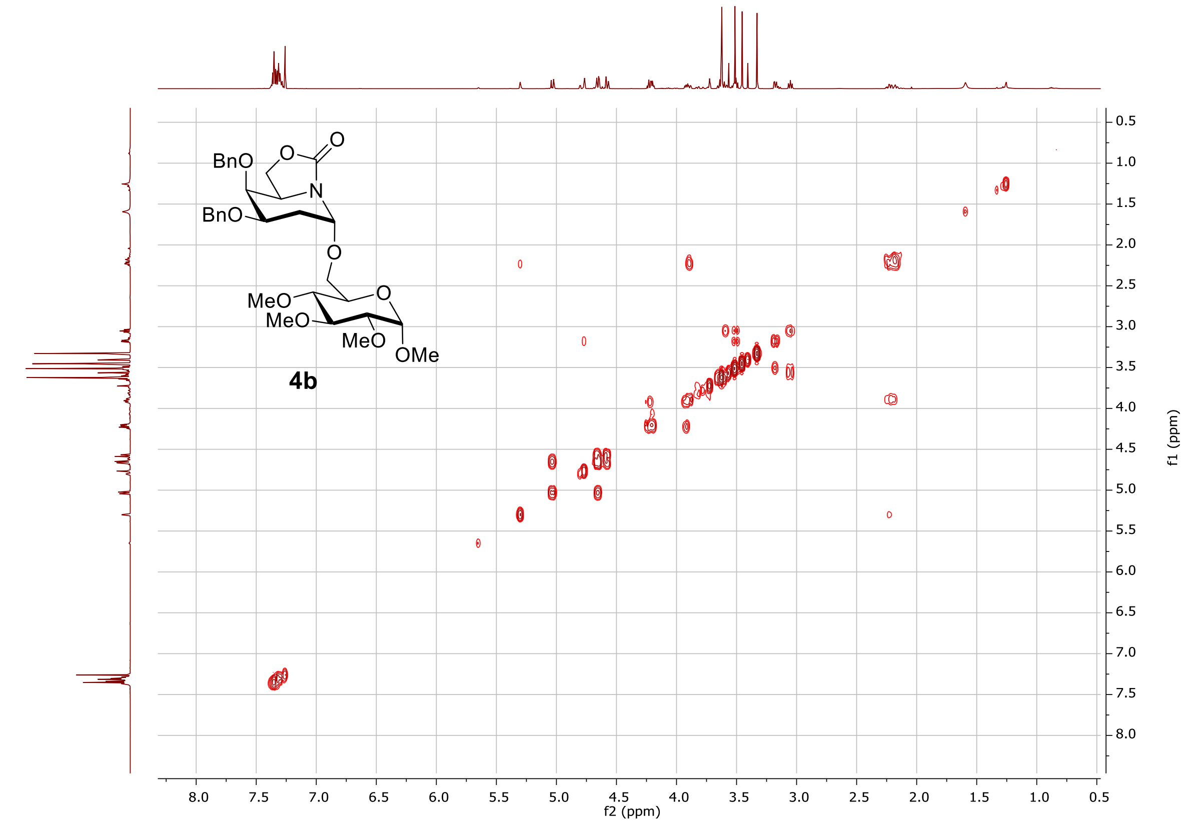

Supplementary Figure S409. COSY spectra for 4b

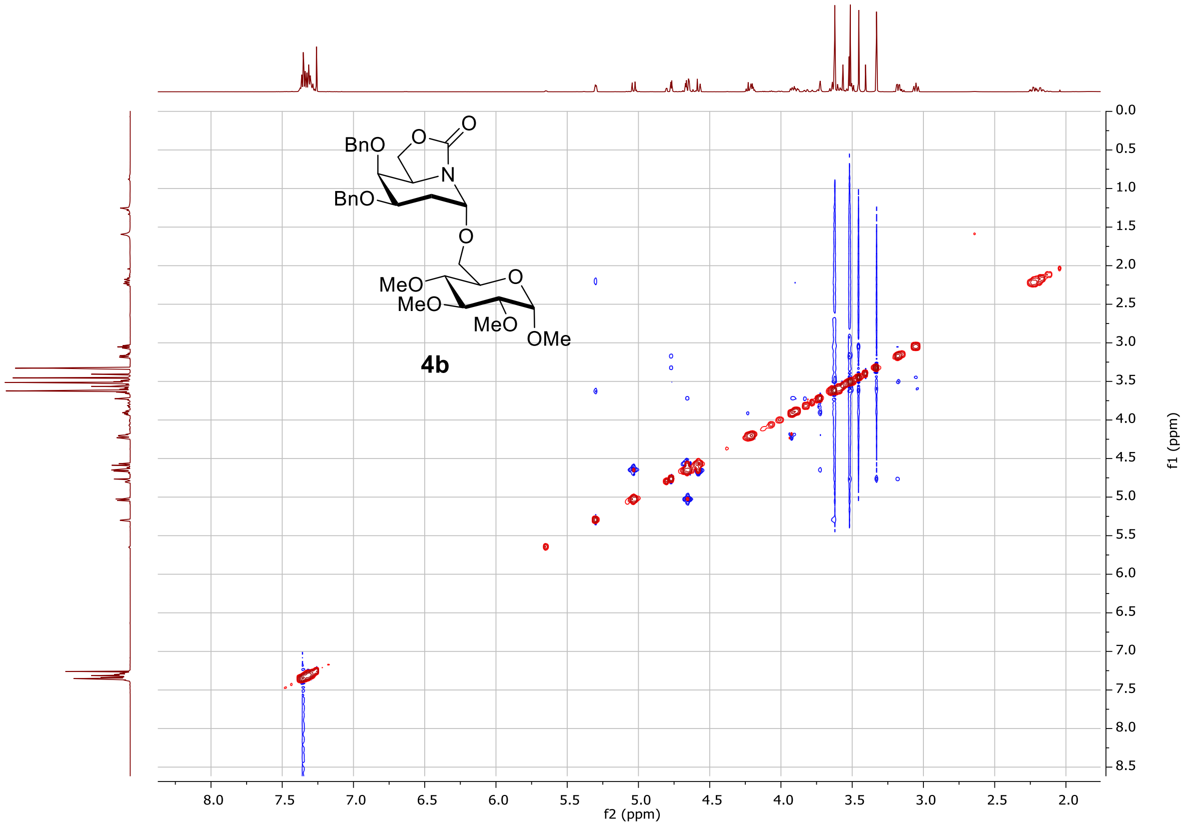

Supplementary Figure S410. NOESY spectra for 4b

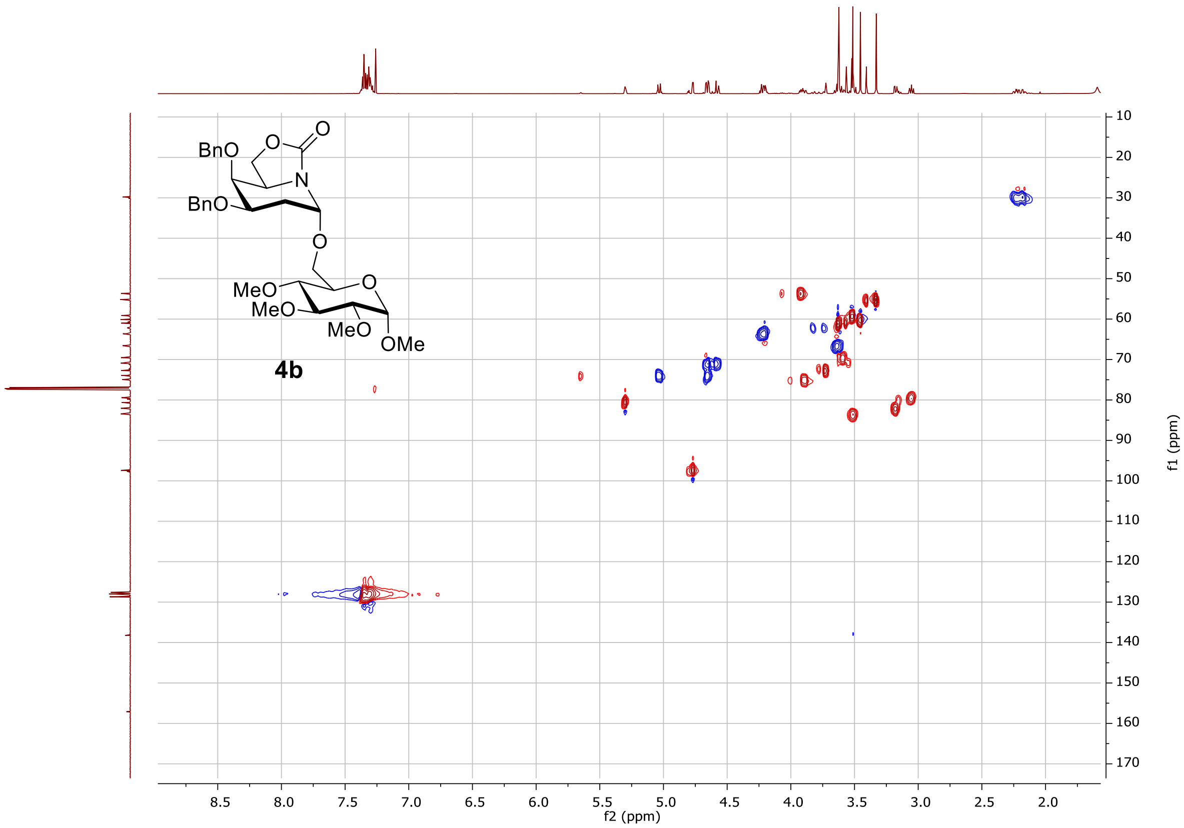

Supplementary Figure S411. HSQC spectra for 4b

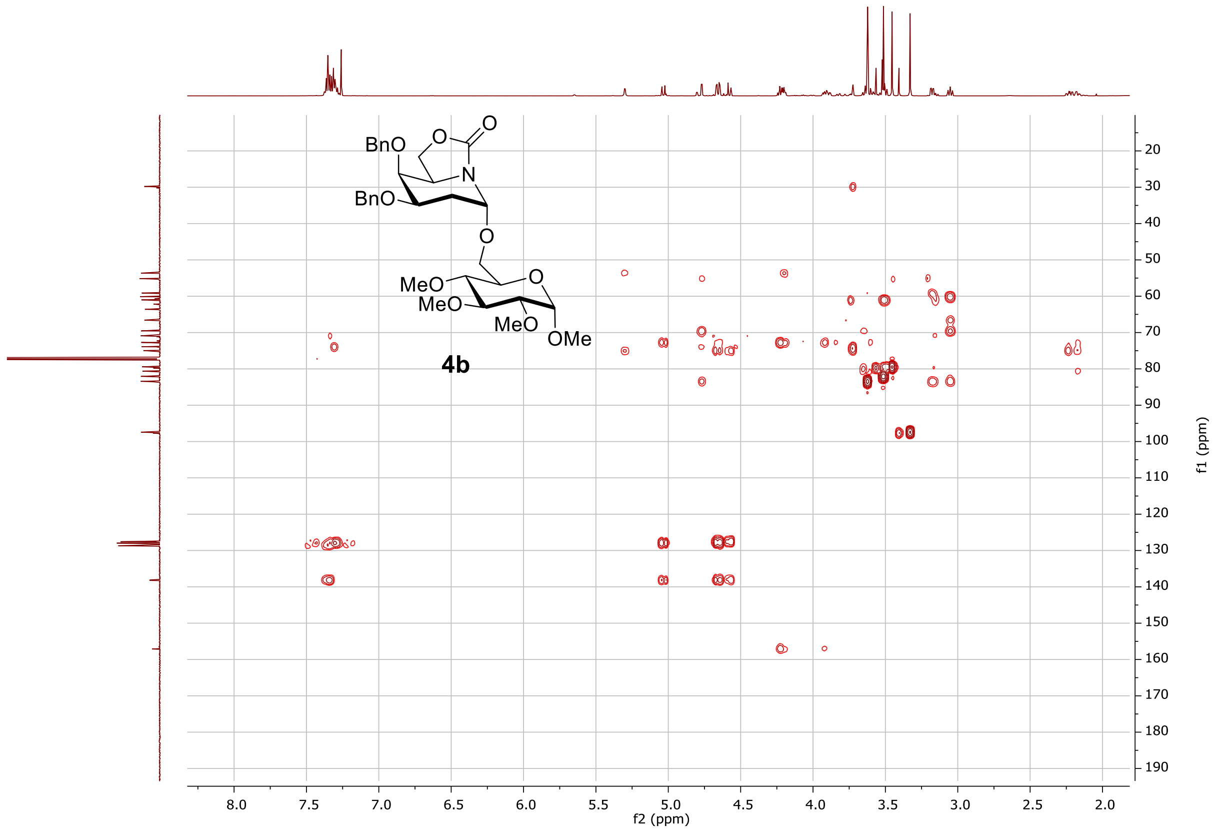

Supplementary Figure S412. HMBC spectra for 4b

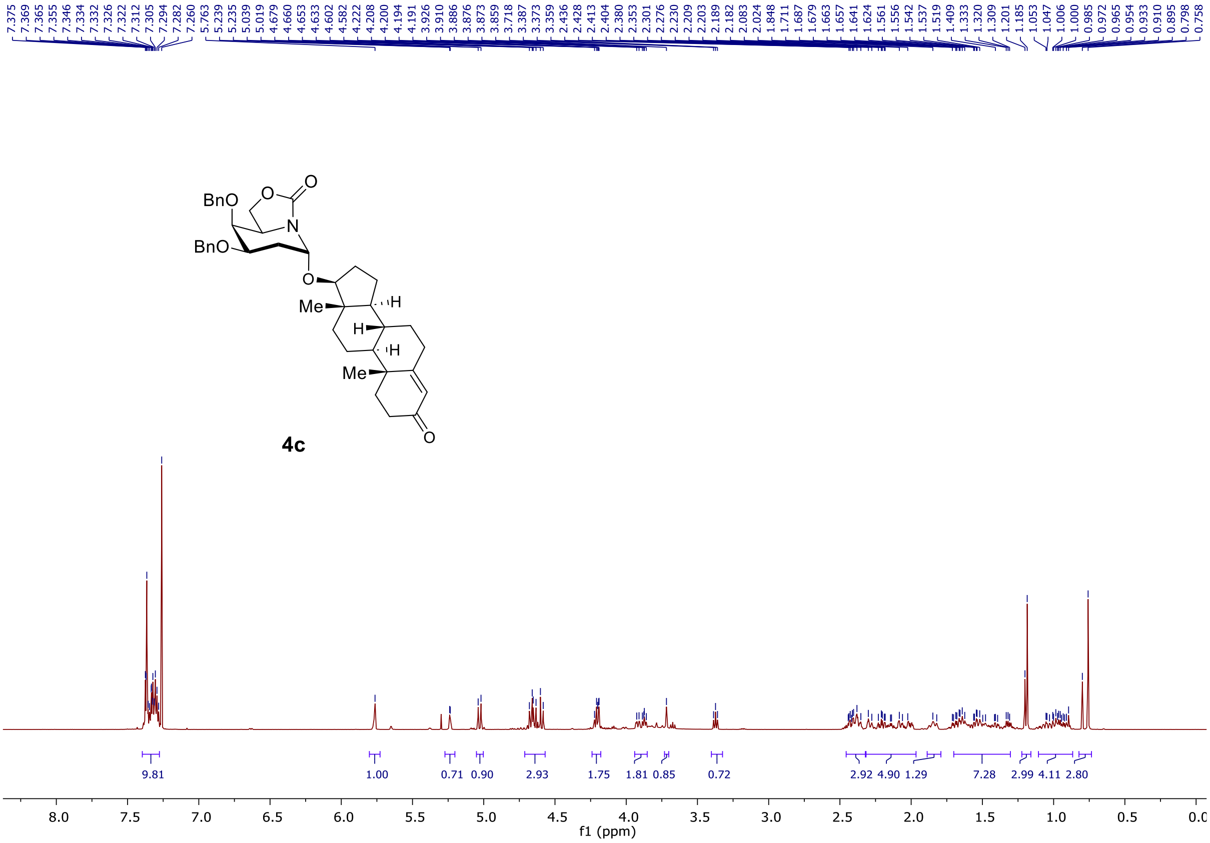

Supplementary Figure S413. ^{1}H NMR spectra for 4c

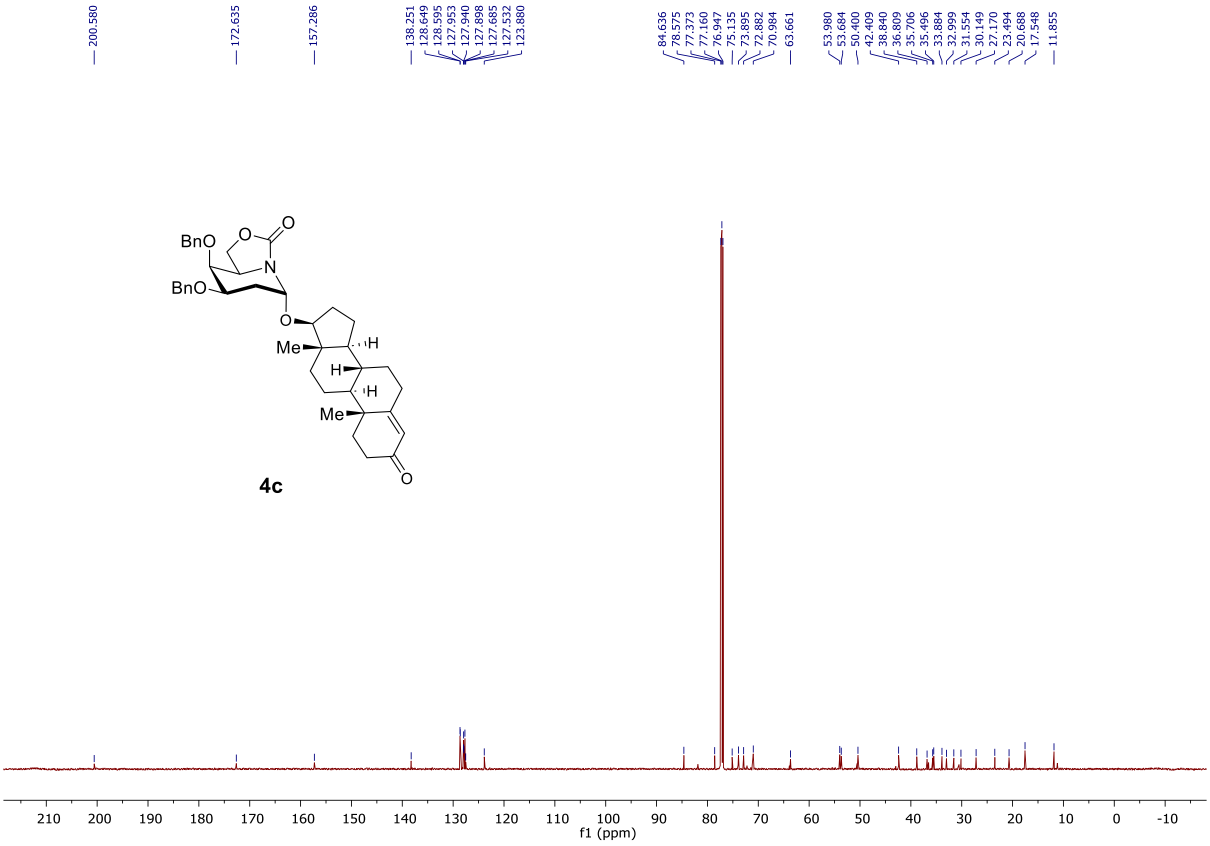

Supplementary Figure S414. ^{13}C NMR spectra for 4c

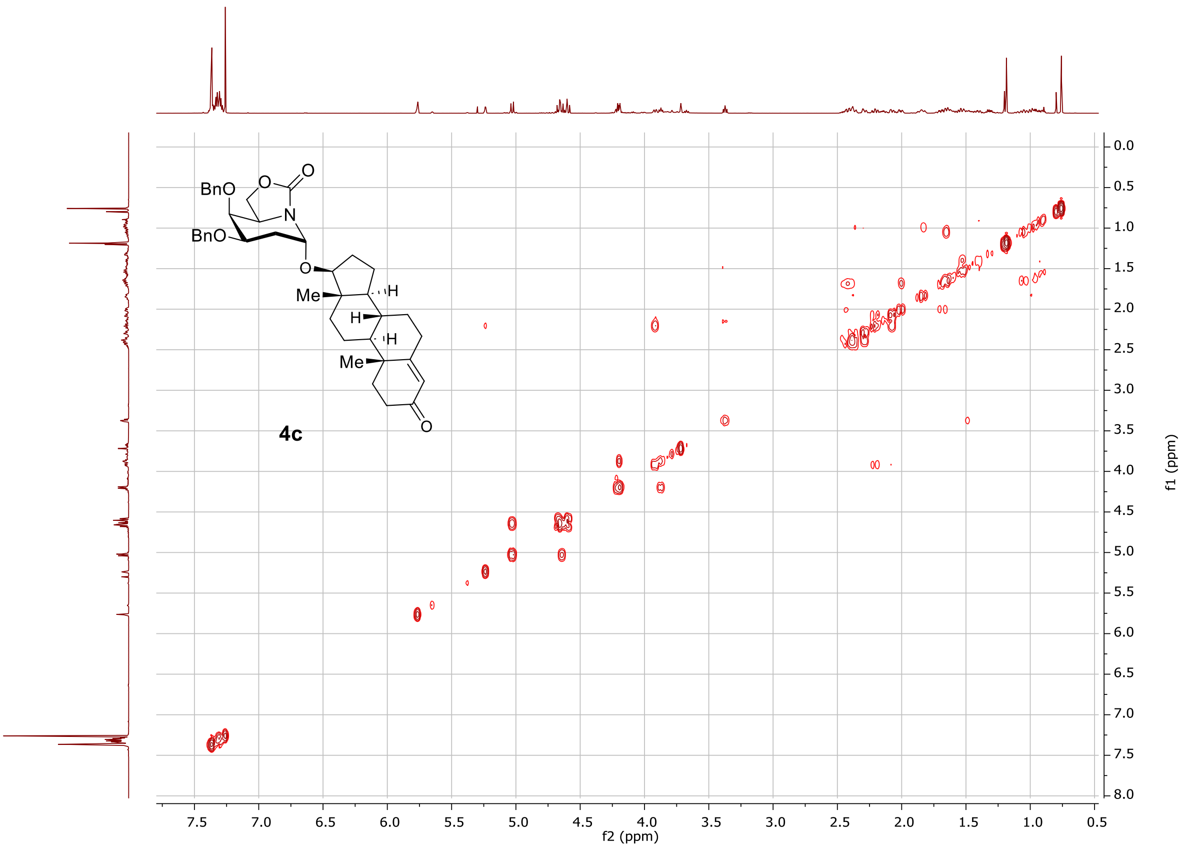

Supplementary Figure S415. COSY spectra for 4c

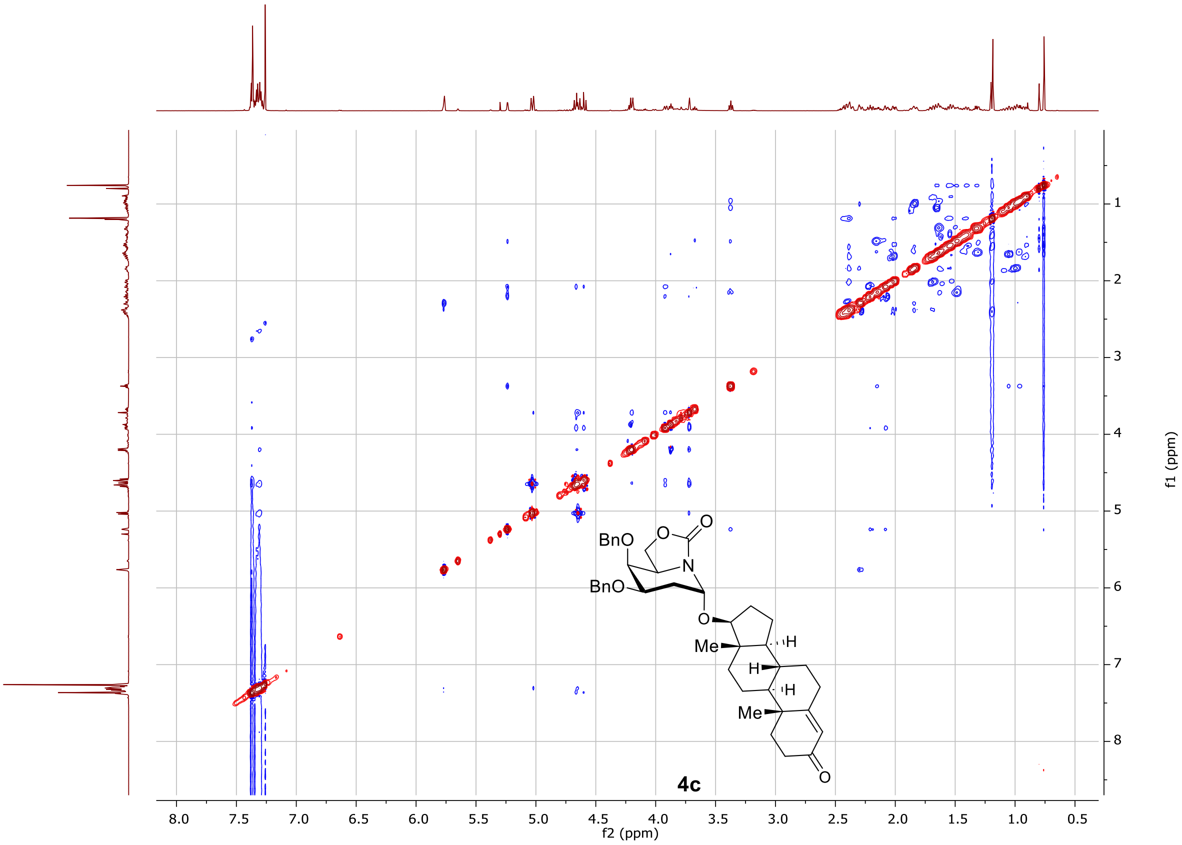

Supplementary Figure S416. NOESY spectra for 4c

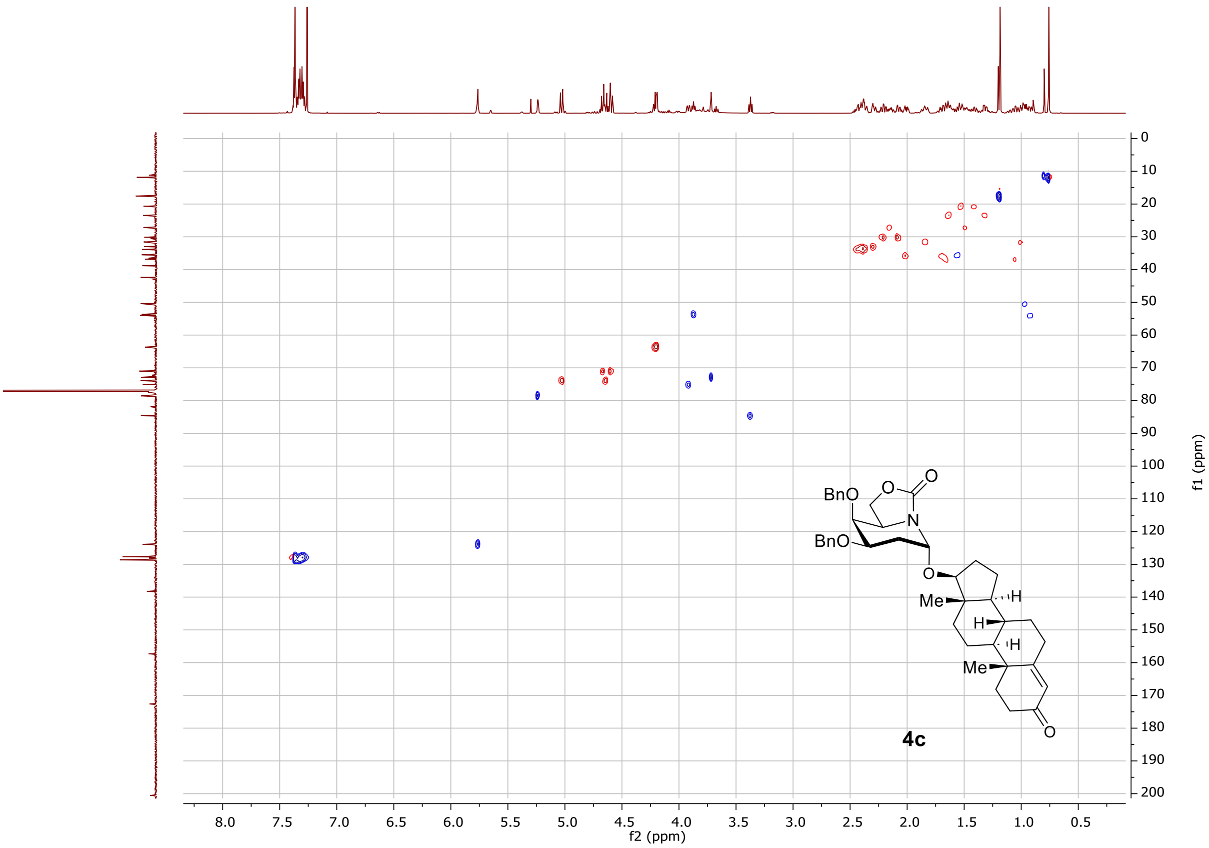

Supplementary Figure S417. HSQC spectra for 4c

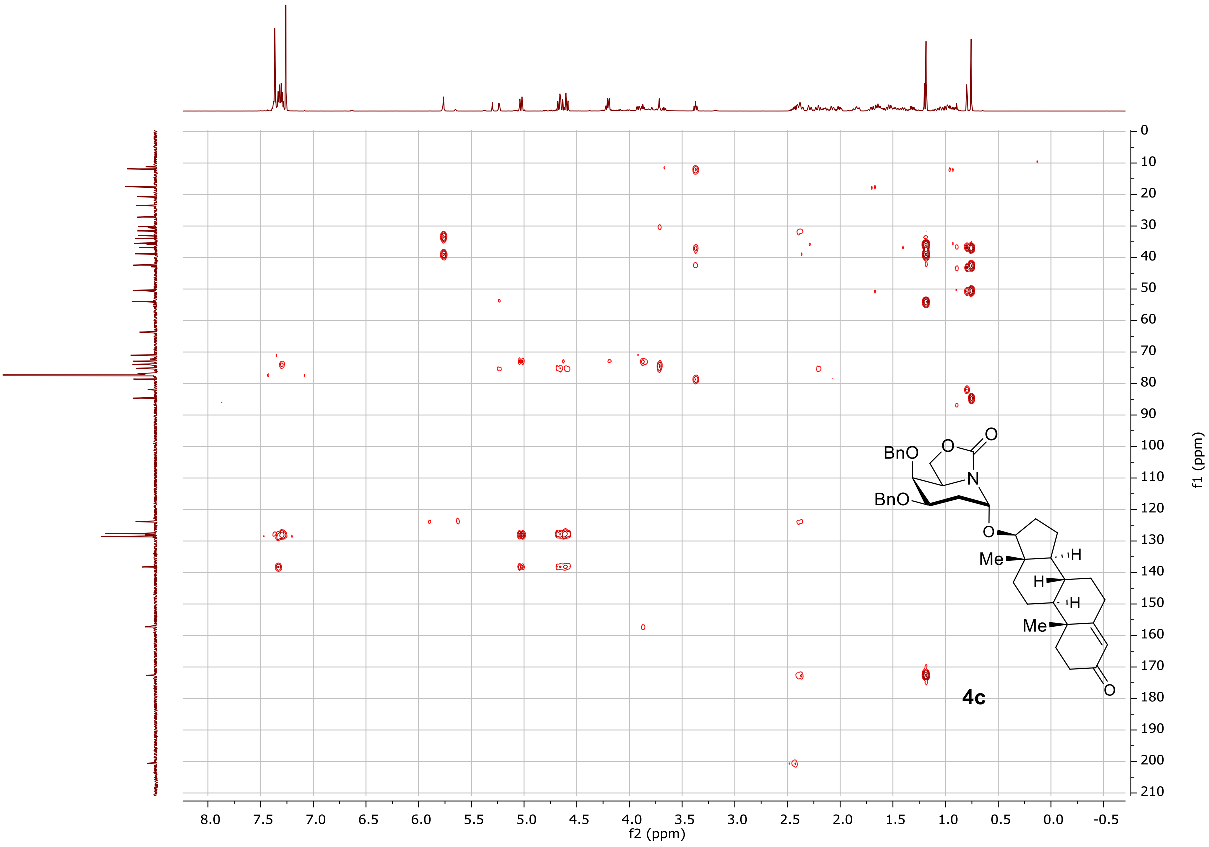

Supplementary Figure S418. HMBC spectra for 4c

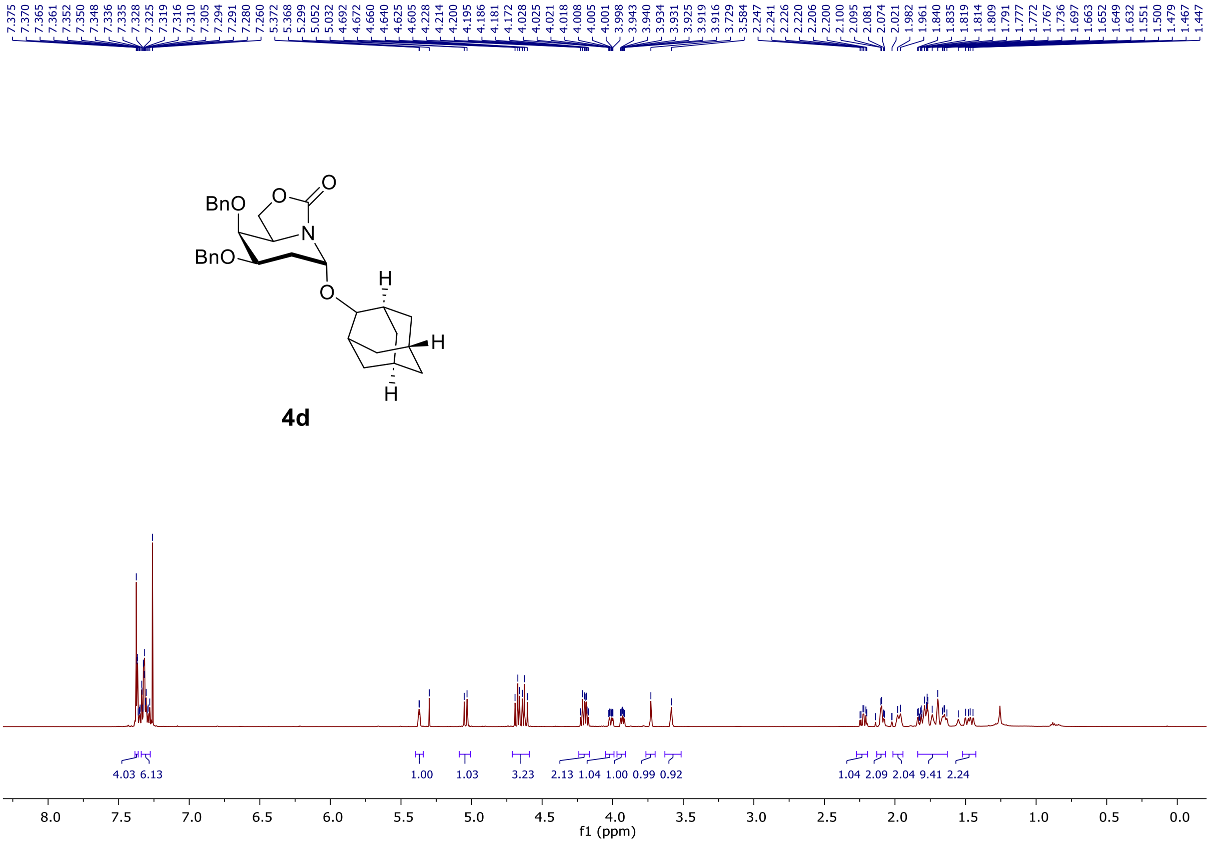

Supplementary Figure S419. ^{1}H NMR spectra for 4d

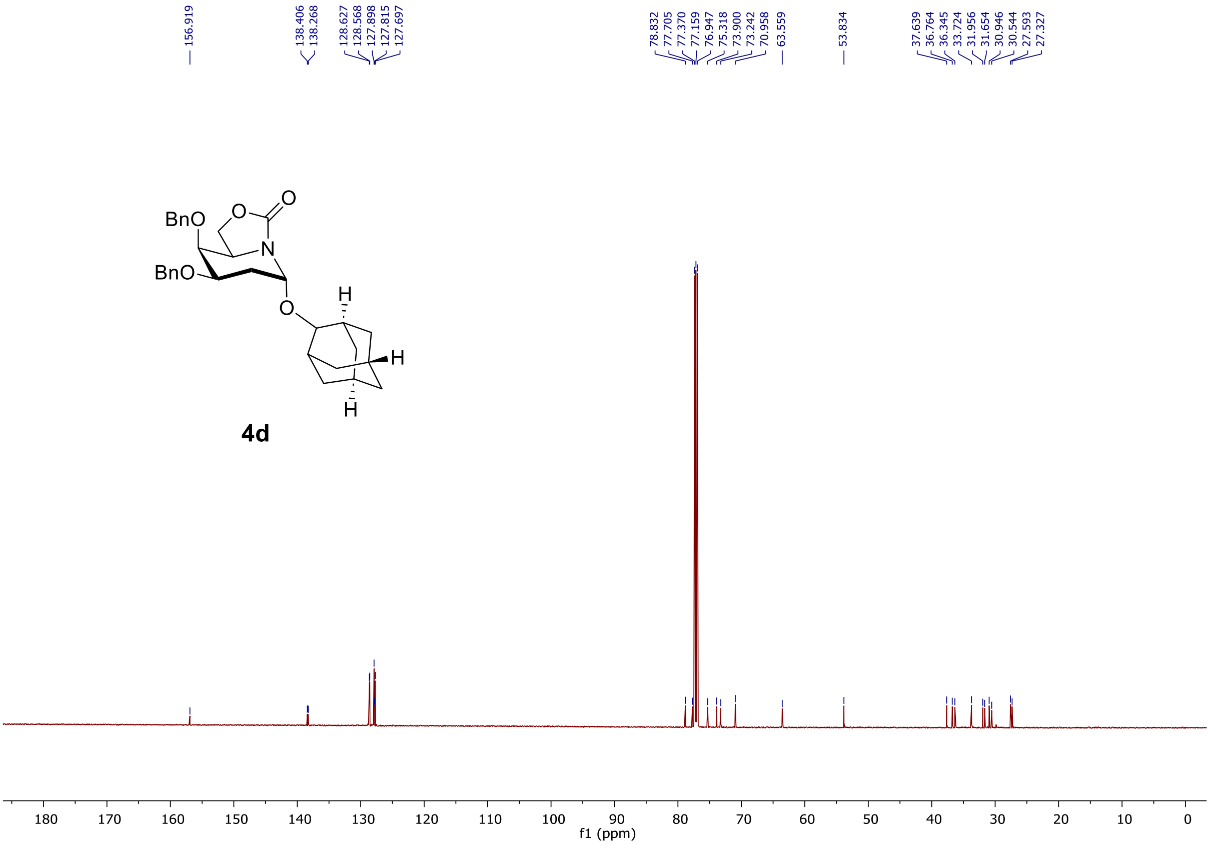

Supplementary Figure S420. ^{13}C NMR spectra for 4d

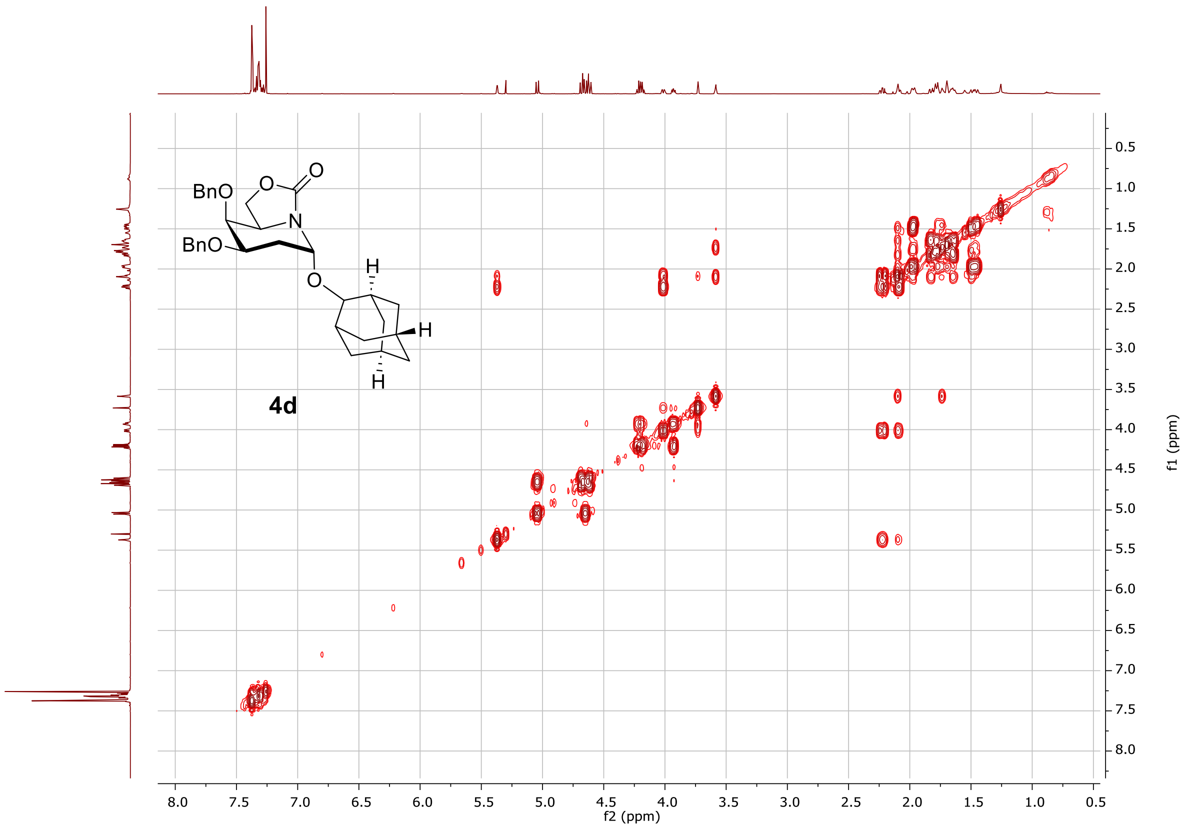

Supplementary Figure S421. COSY spectra for 4d

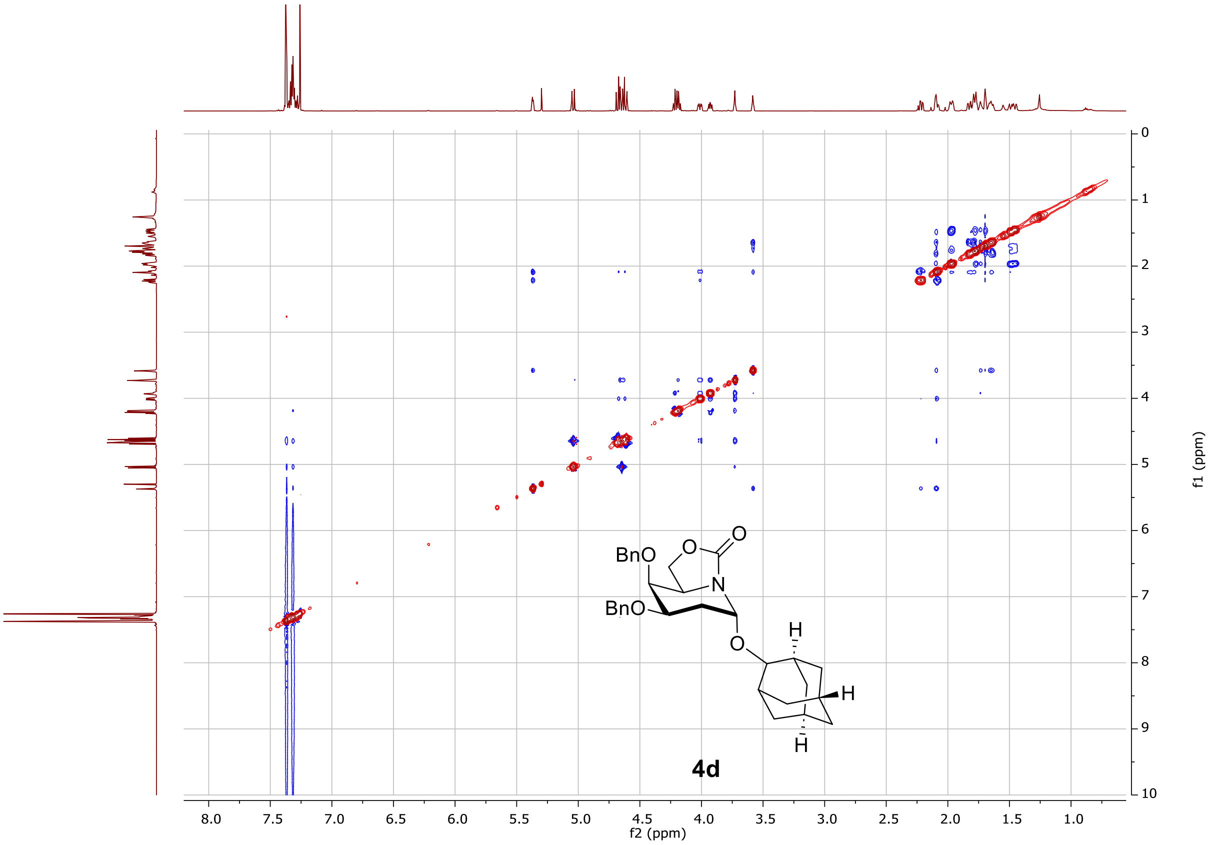

Supplementary Figure S422. NOESY spectra for 4d

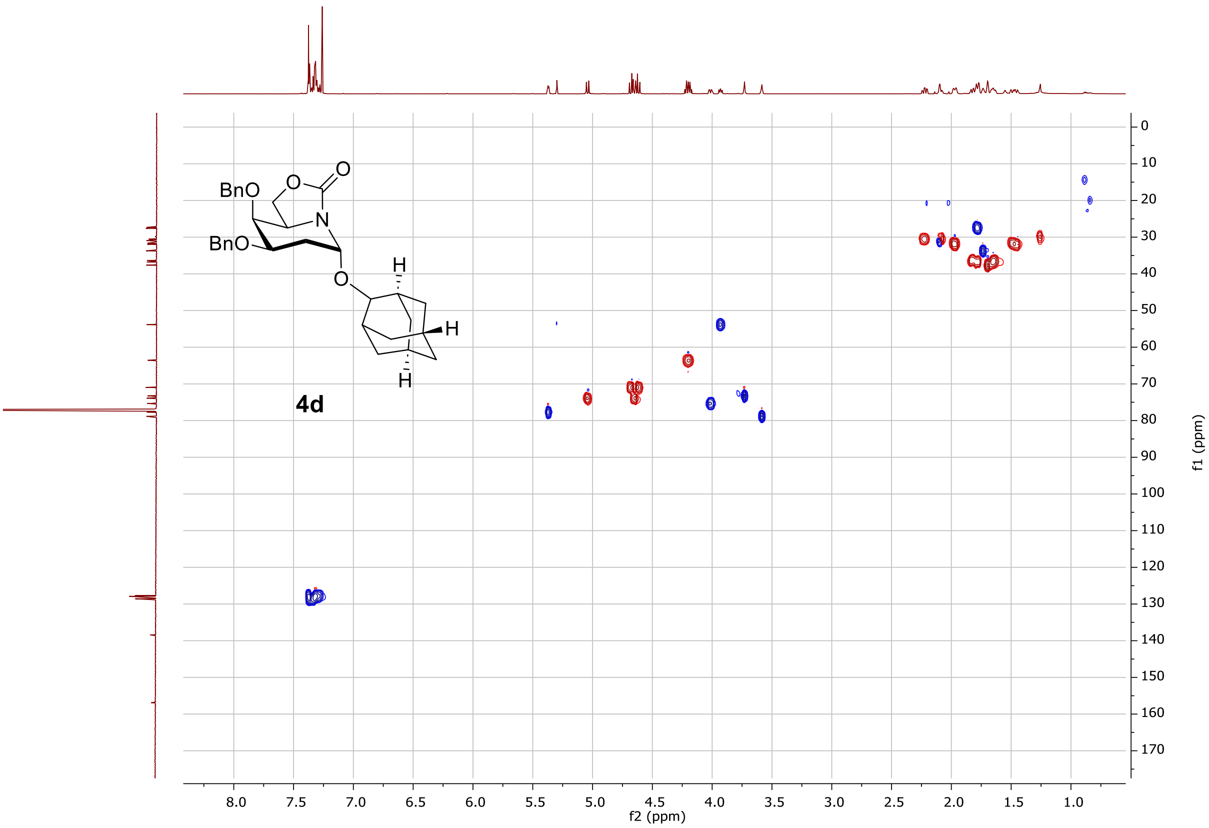

Supplementary Figure S423. HSQC spectra for 4d

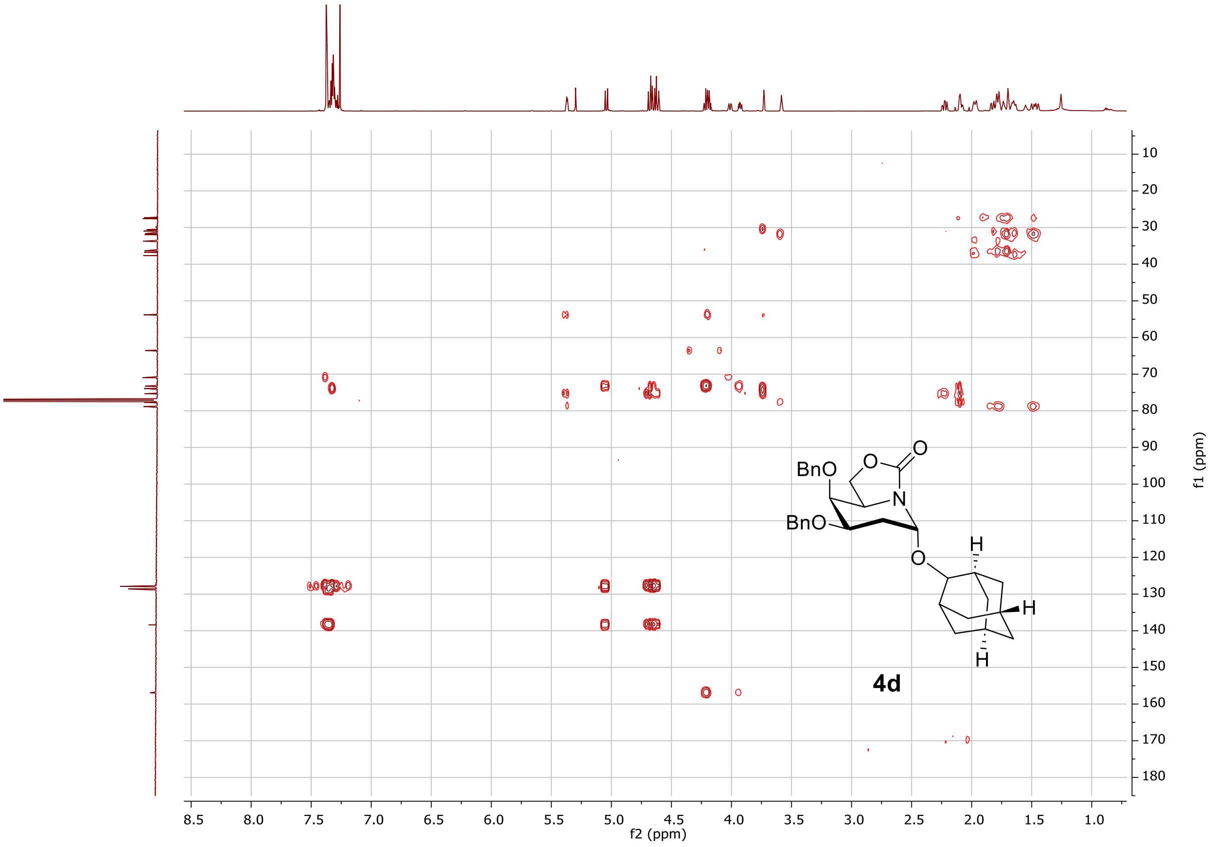

Supplementary Figure S424. HMBC spectra for 4d

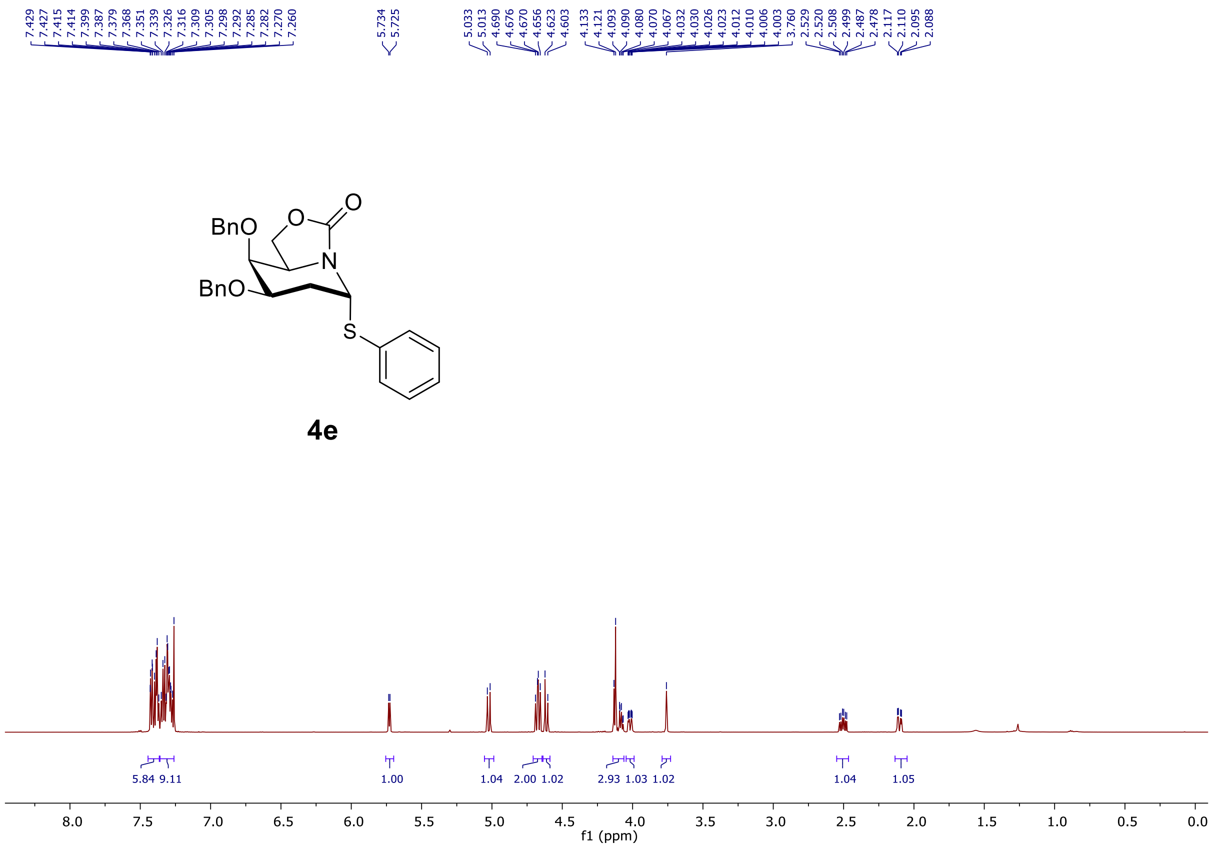

Supplementary Figure S425. ^{1}H NMR spectra for 4e

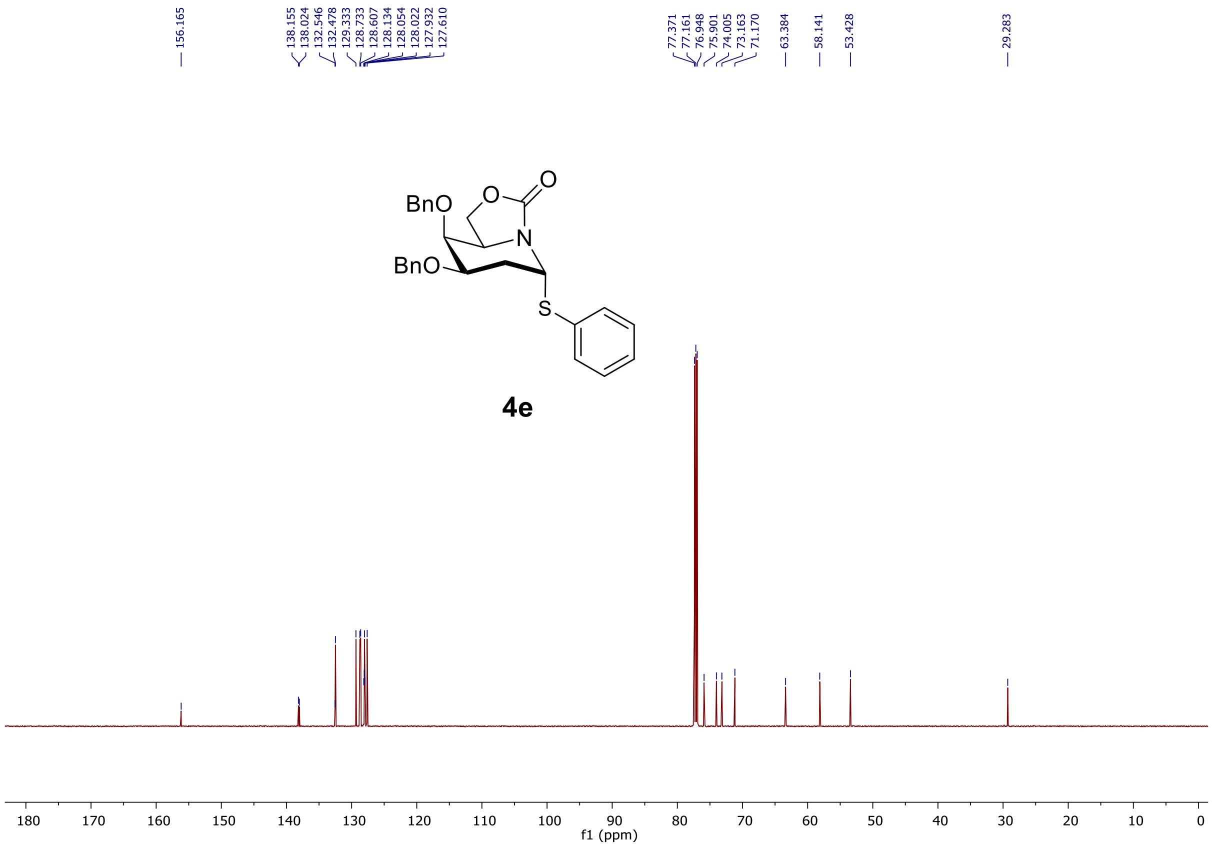

Supplementary Figure S426. ^{13}C NMR spectra for 4e

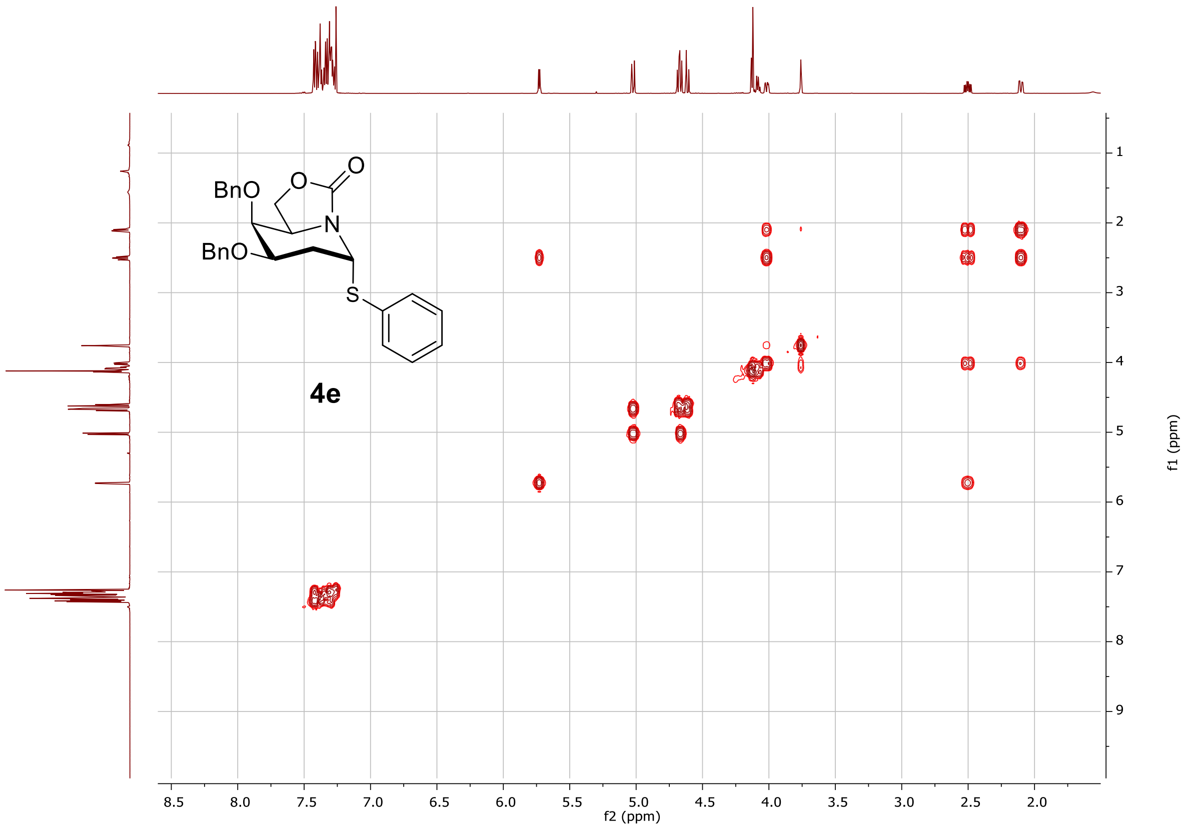

Supplementary Figure S427. COSY spectra for 4e

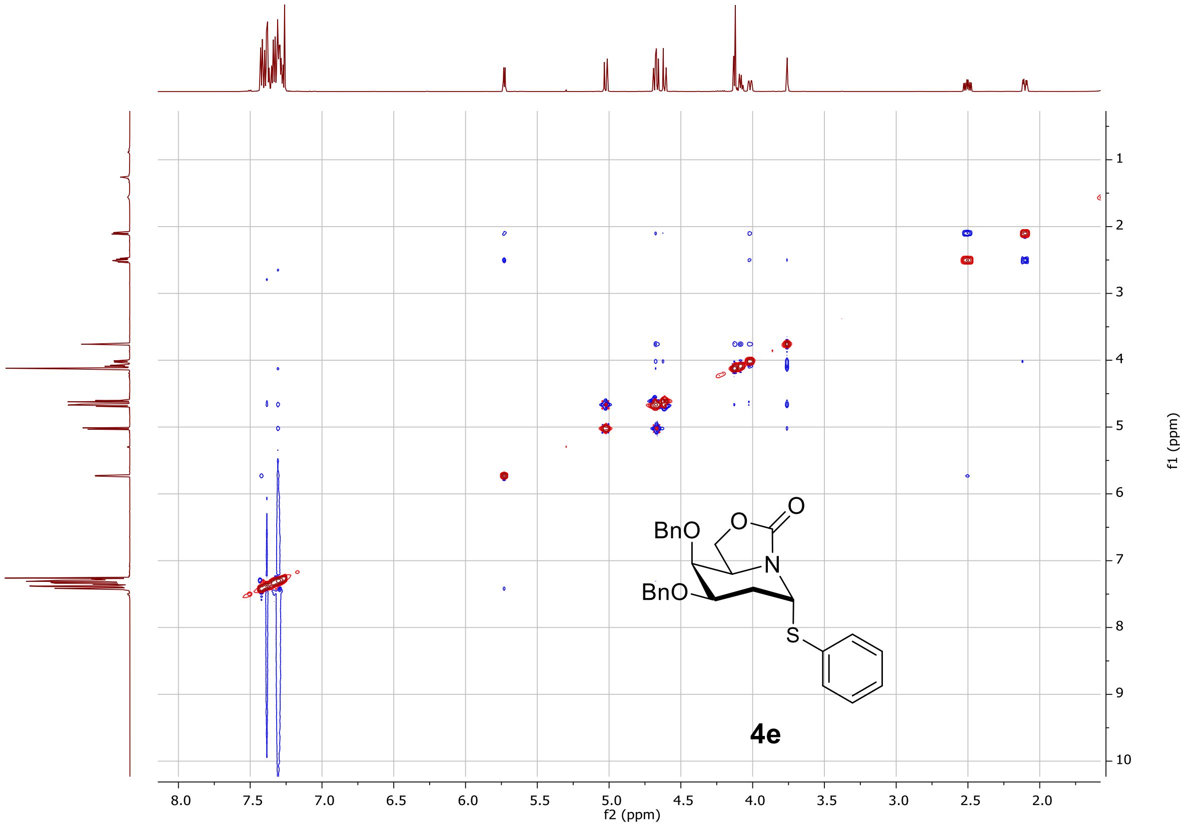

Supplementary Figure S428. NOESY spectra for 4e

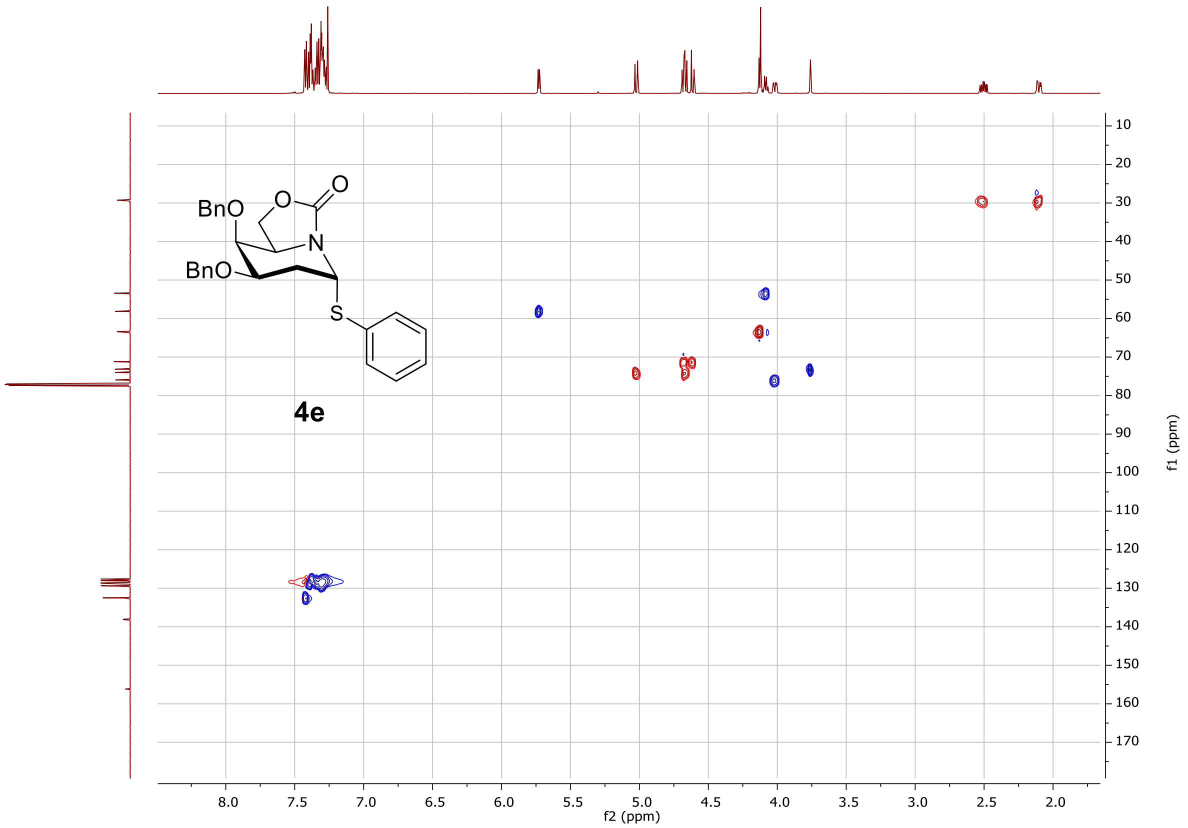

Supplementary Figure S429. HSQC spectra for 4e

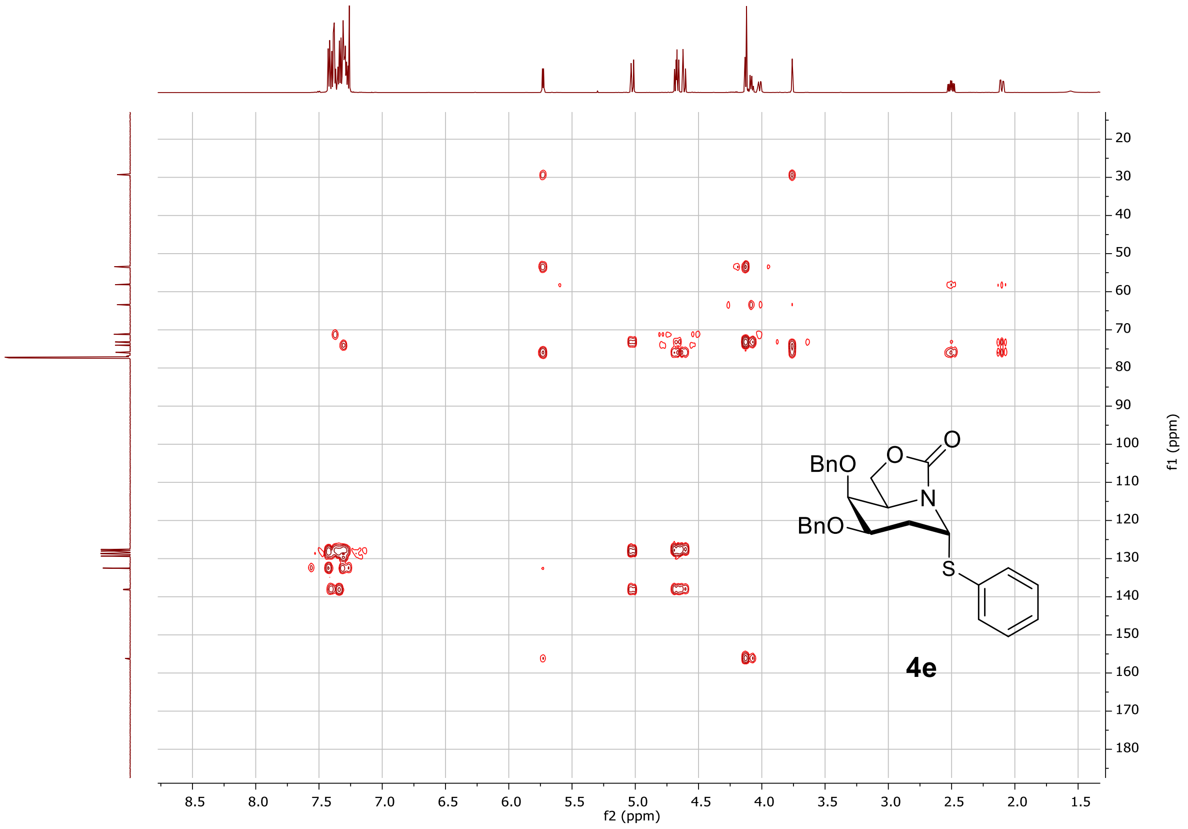

Supplementary Figure S430. HMBC spectra for 4e

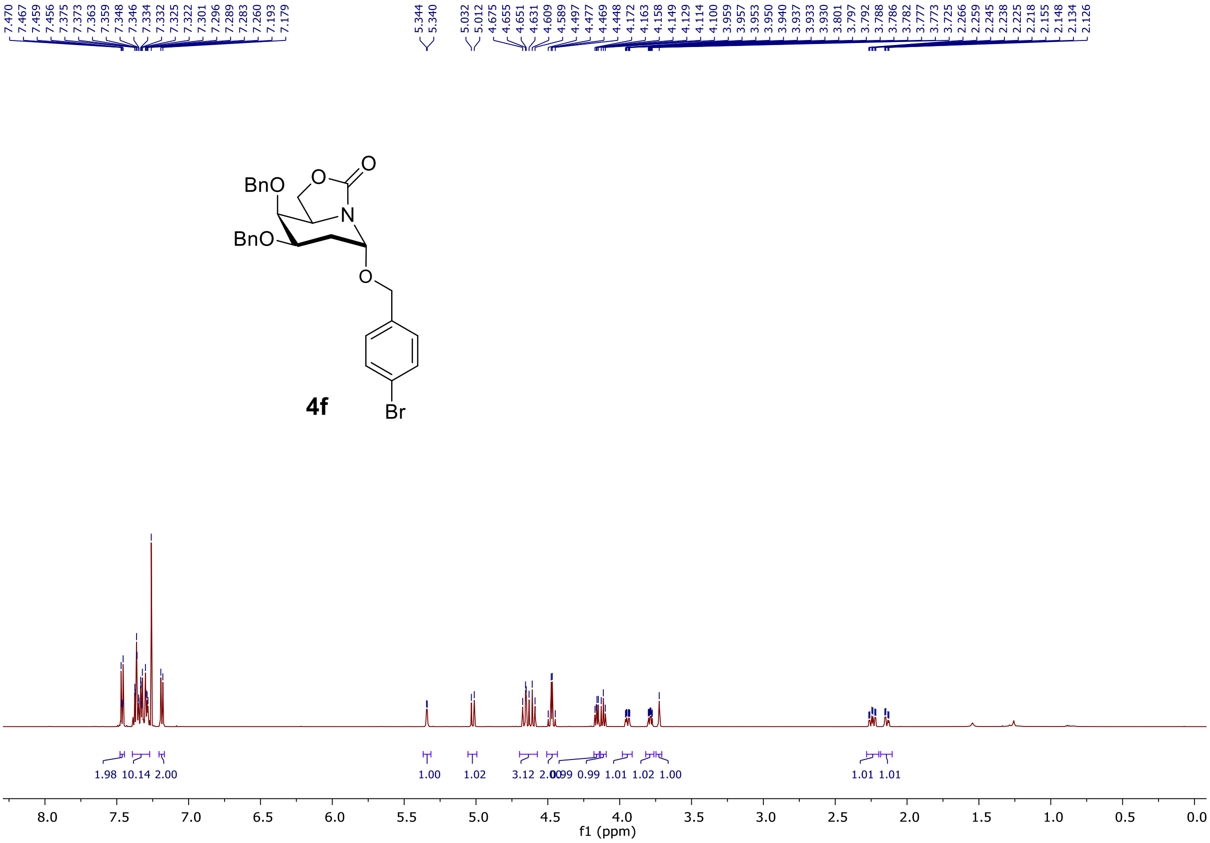

Supplementary Figure S431. ^{1}H NMR spectra for 4f

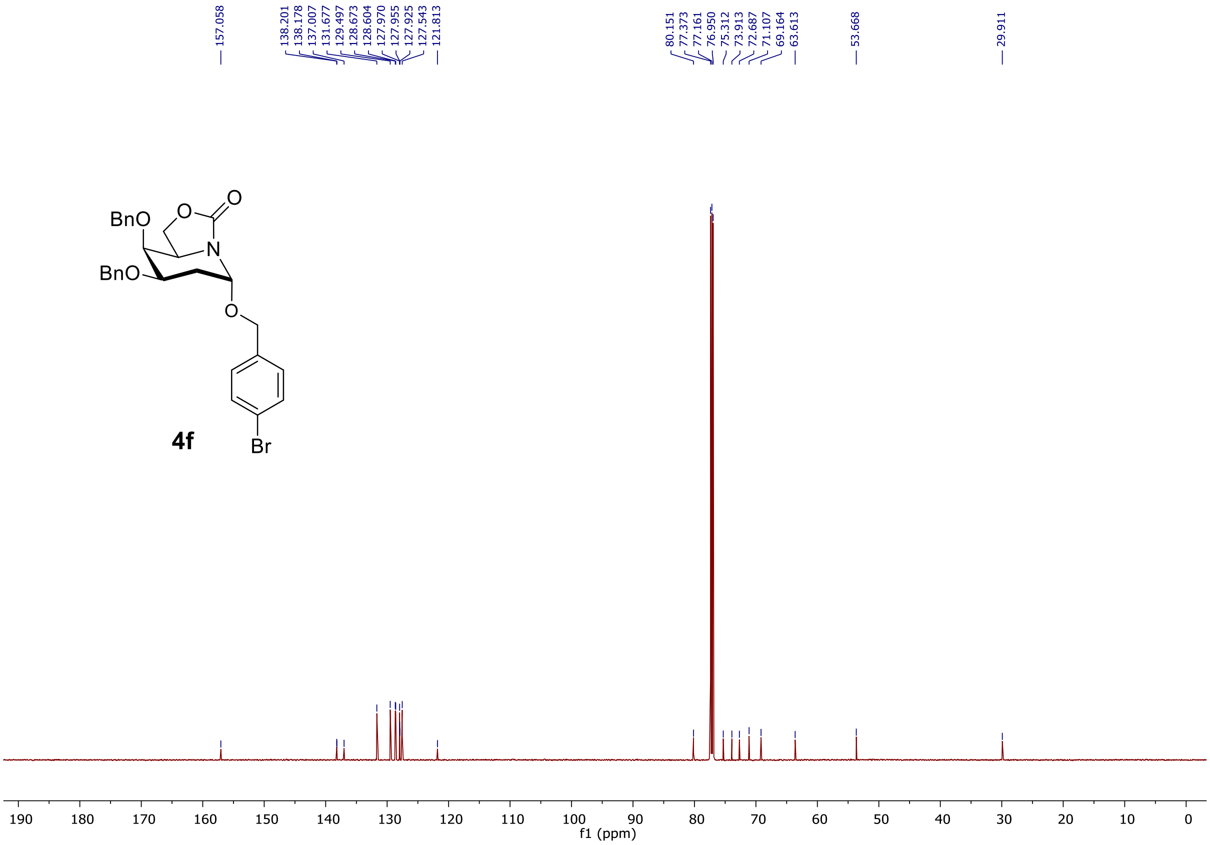

Supplementary Figure S432. ^{13}C NMR spectra for 4f

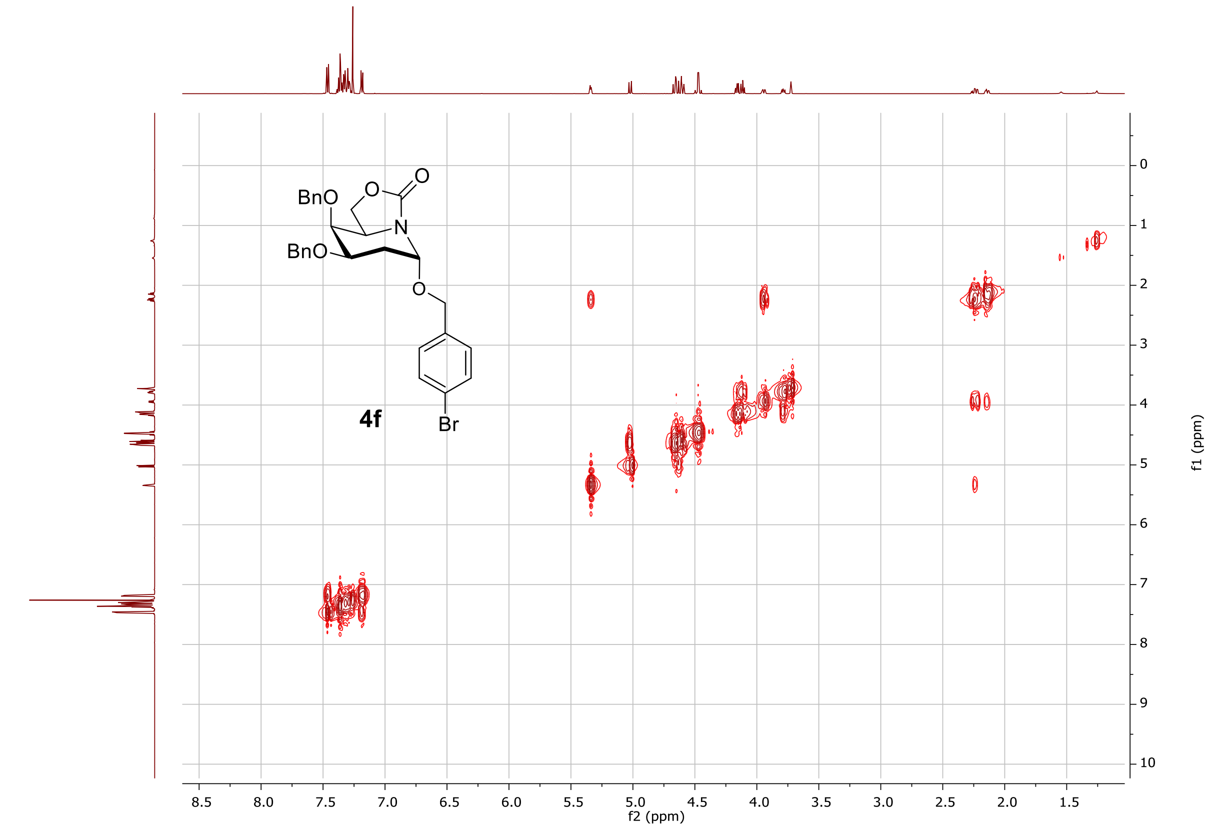

Supplementary Figure S433. COSY spectra for 4f

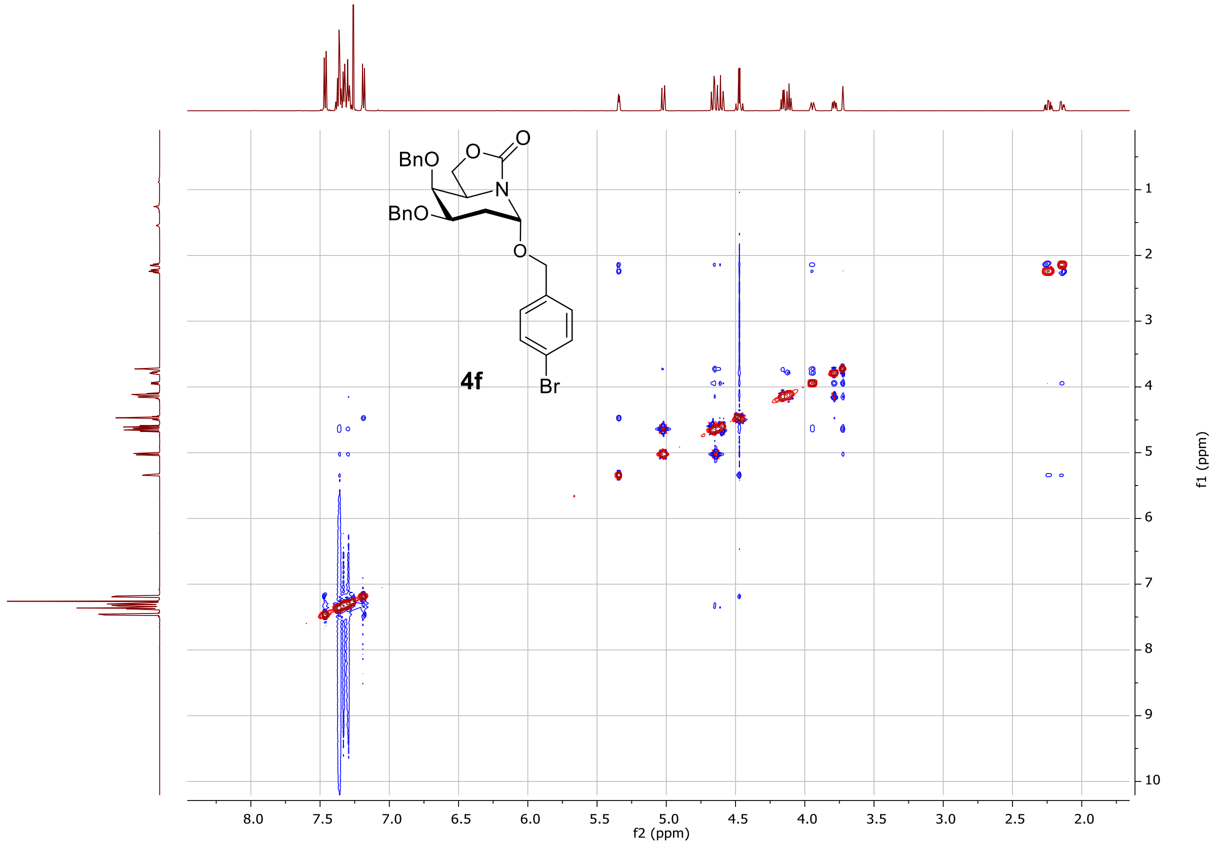

Supplementary Figure S434. NOESY spectra for 4f

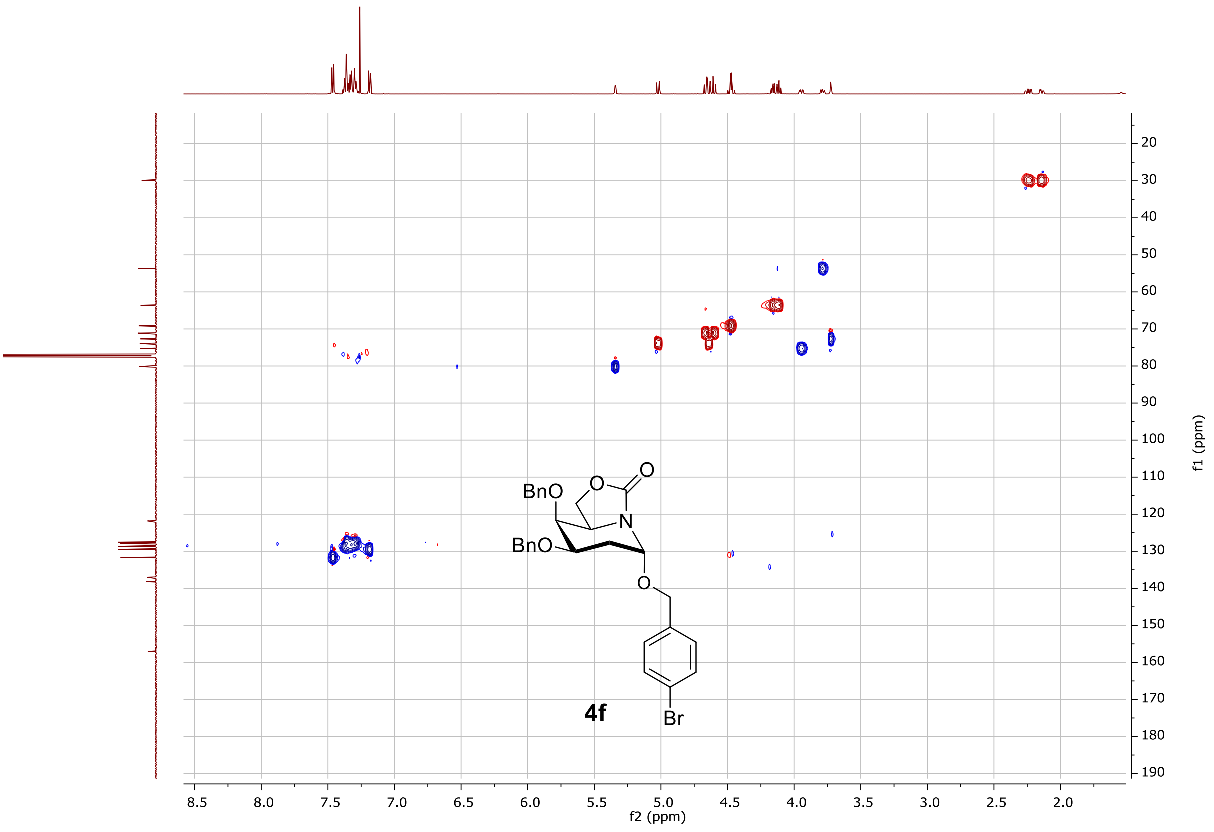

Supplementary Figure S435. HSQC spectra for 4f

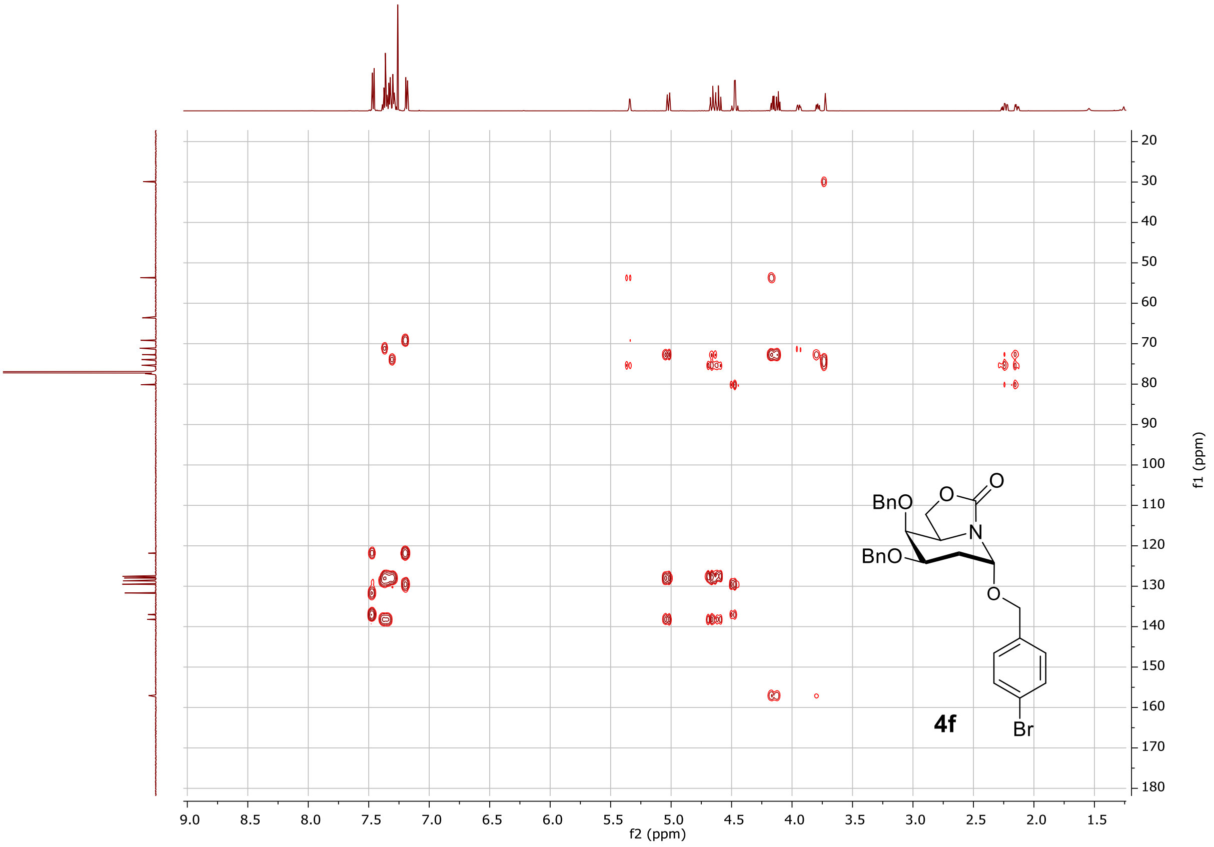

Supplementary Figure S436. HMBC spectra for 4f

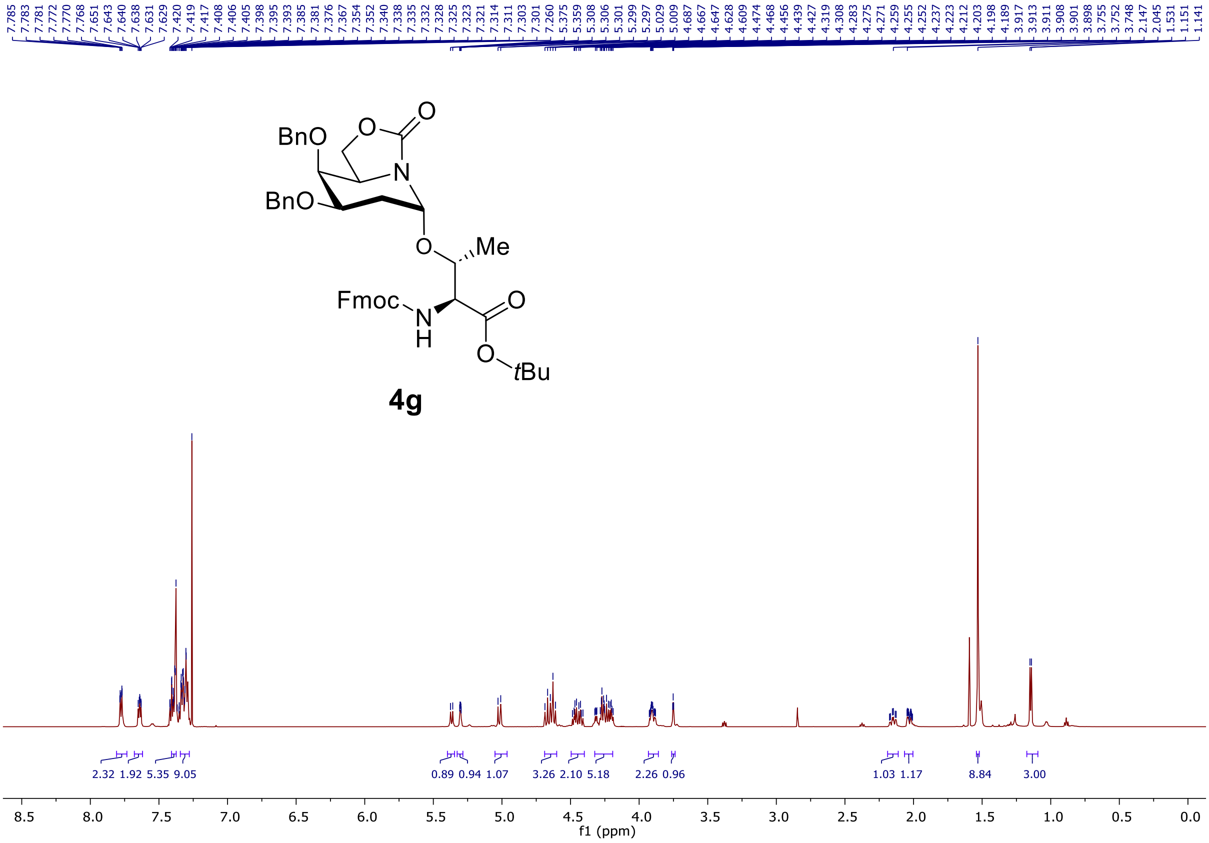

Supplementary Figure S437. ¹H NMR spectra for 4g

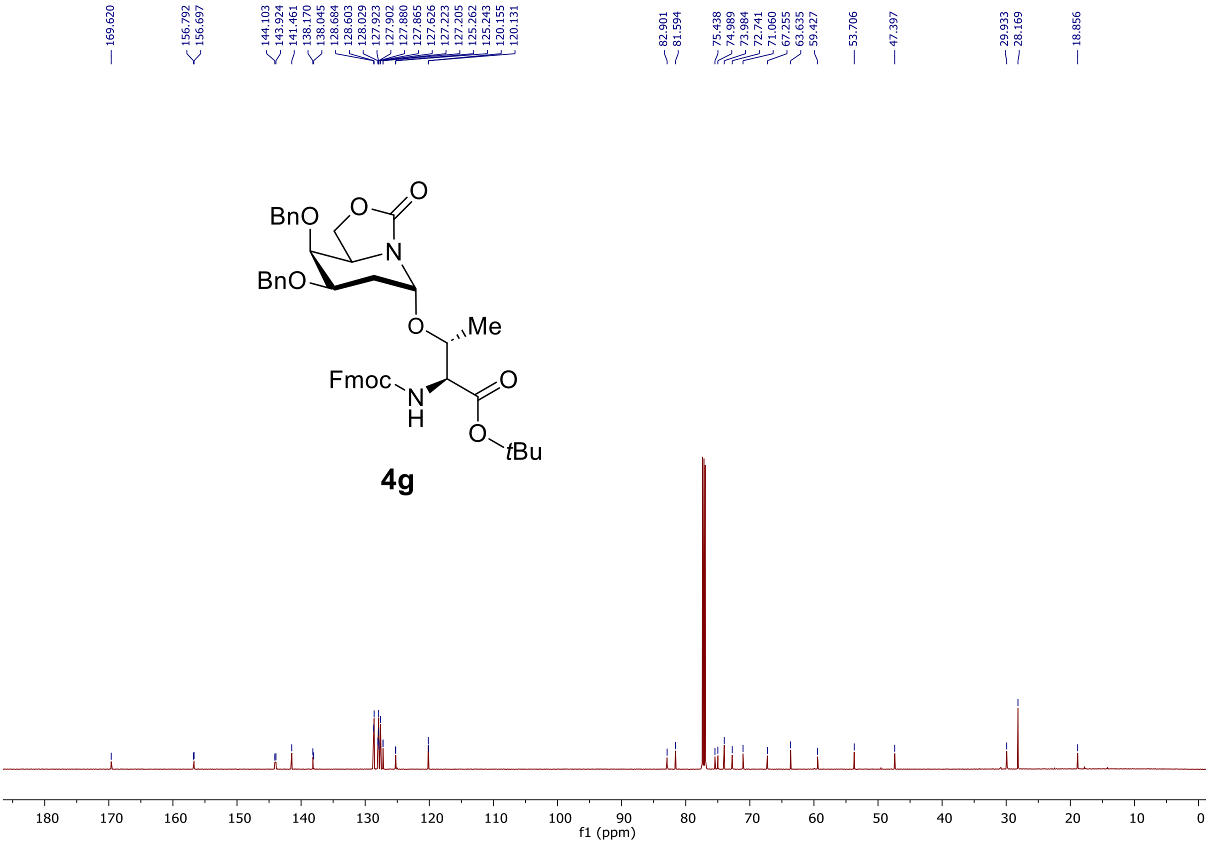

Supplementary Figure S438. ¹³C NMR spectra for 4g

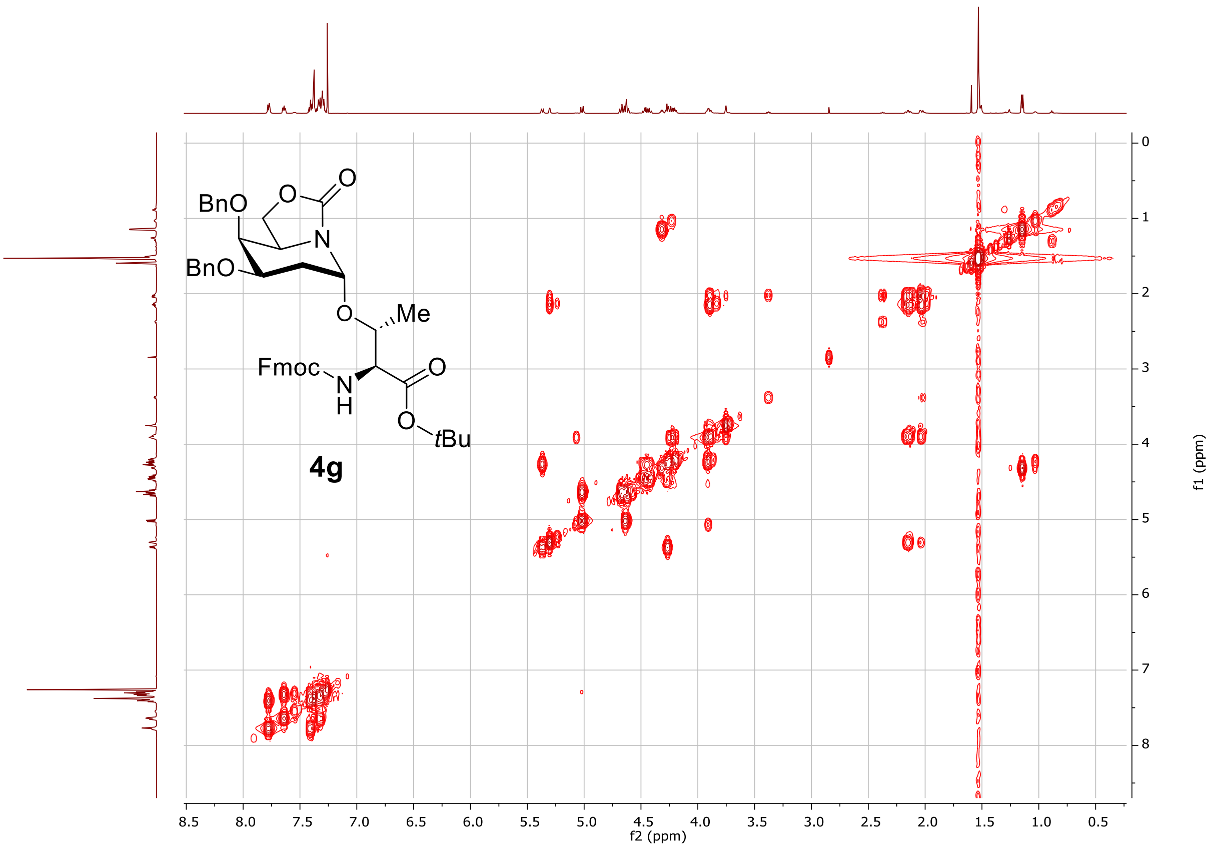

Supplementary Figure S439. COSY spectra for 4g

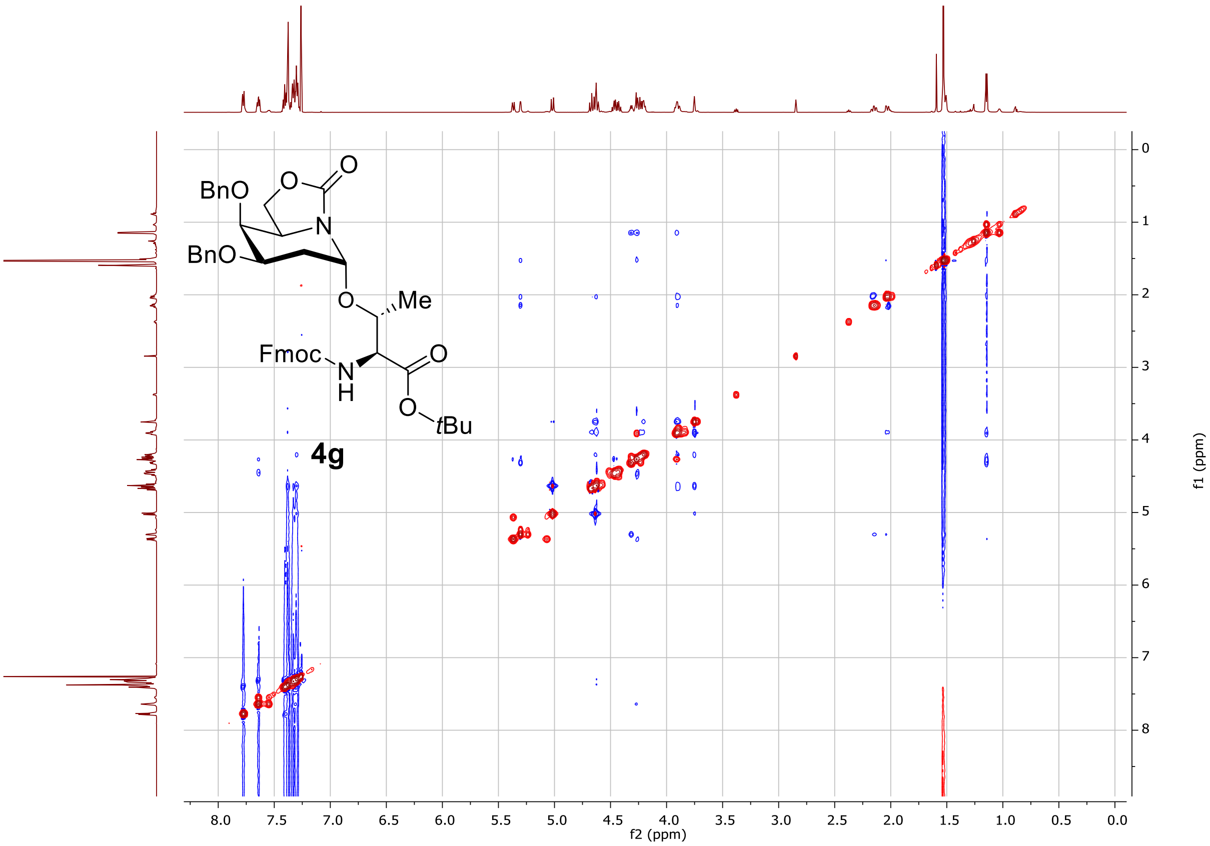

Supplementary Figure S440. NOESY spectra for 4g

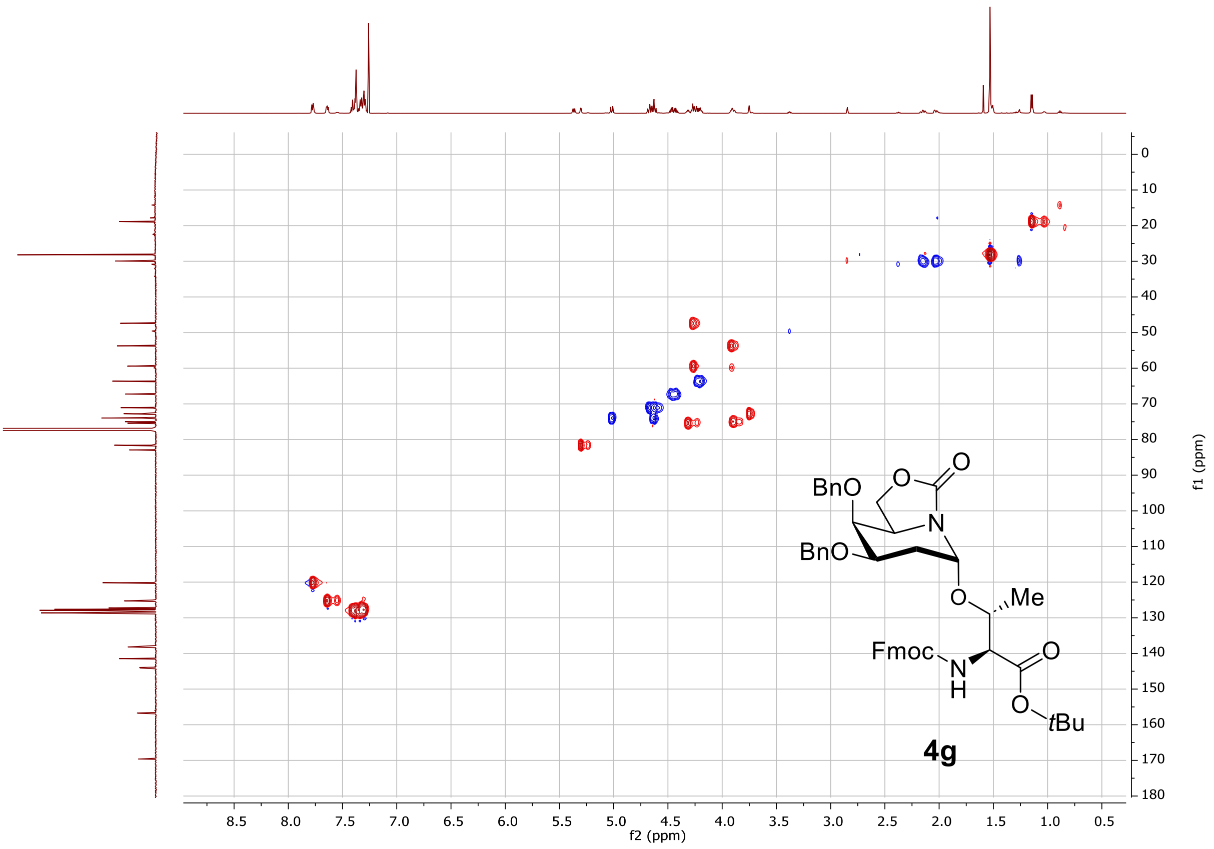

Supplementary Figure S441. HSQC spectra for 4g

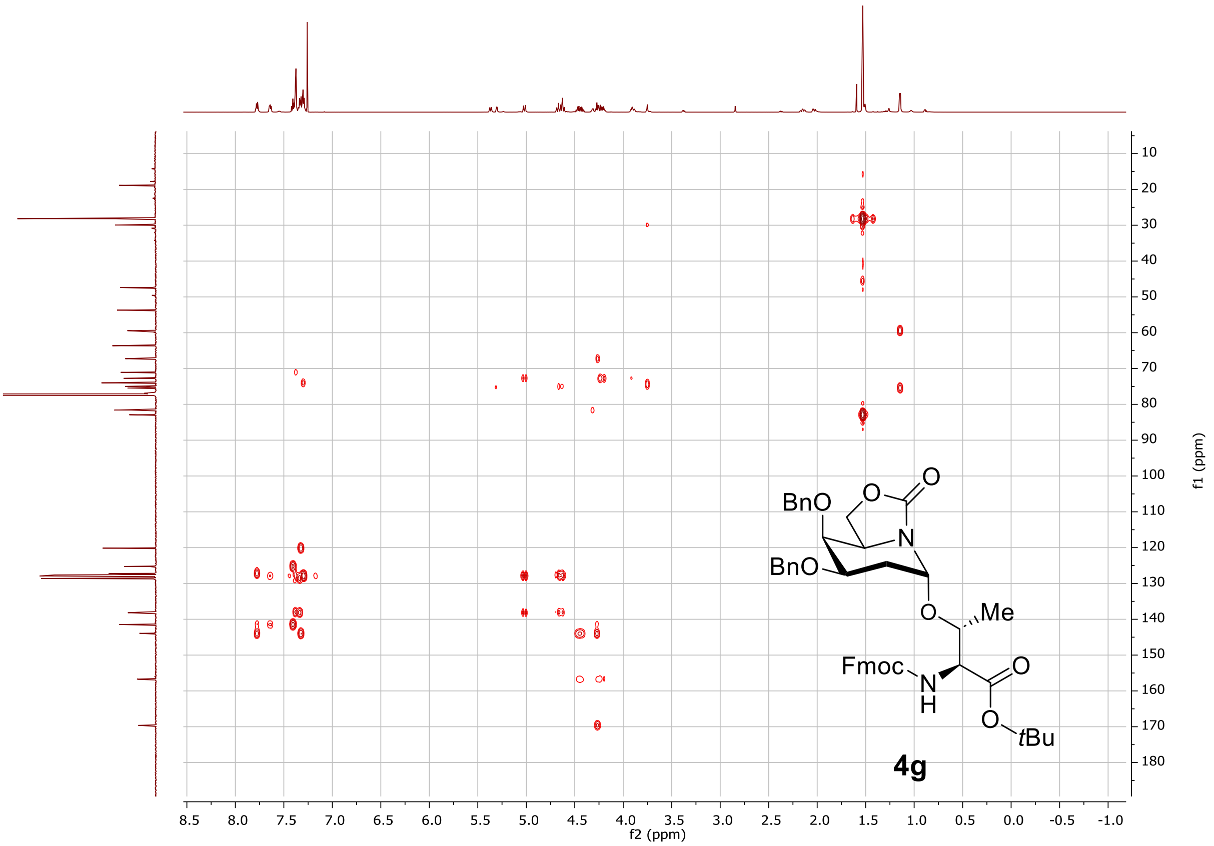

Supplementary Figure S442. HMBC spectra for 4g

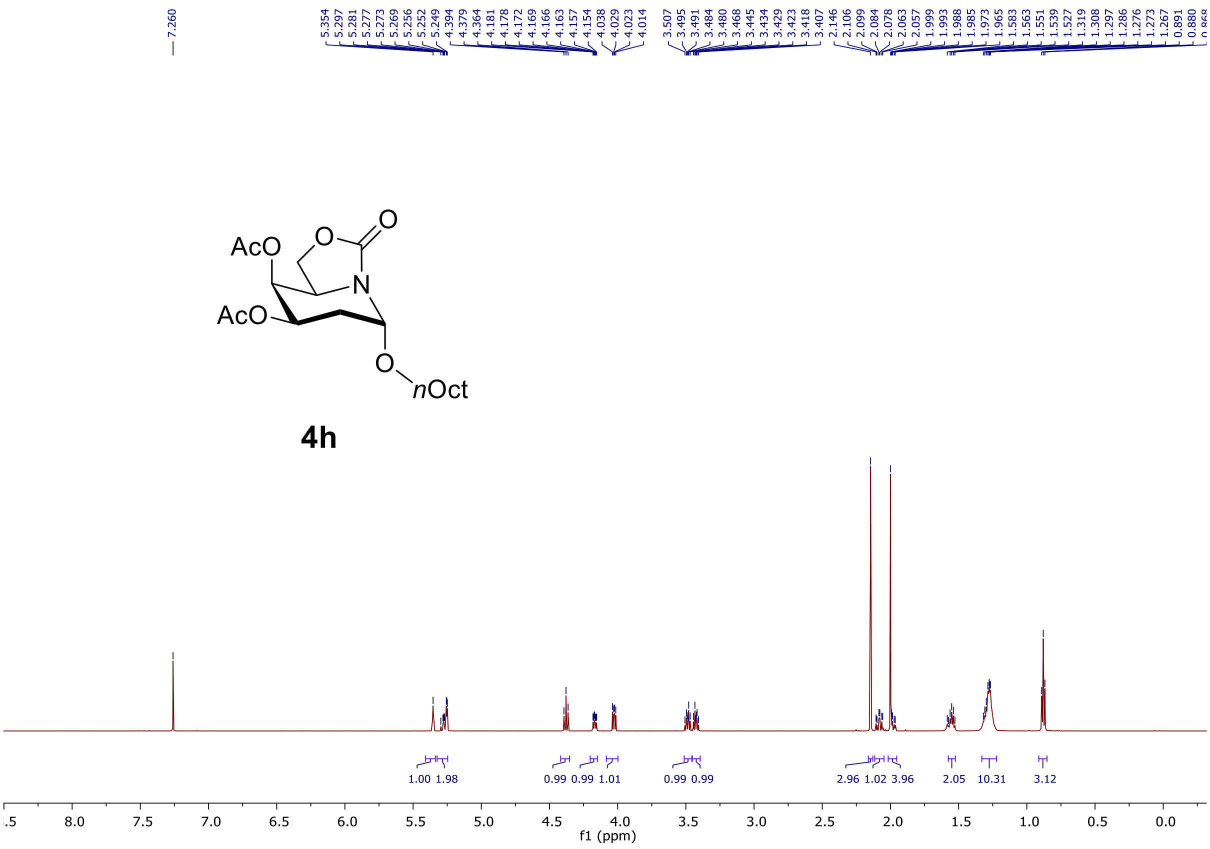

Supplementary Figure S443. ^{1}H NMR spectra for 4h

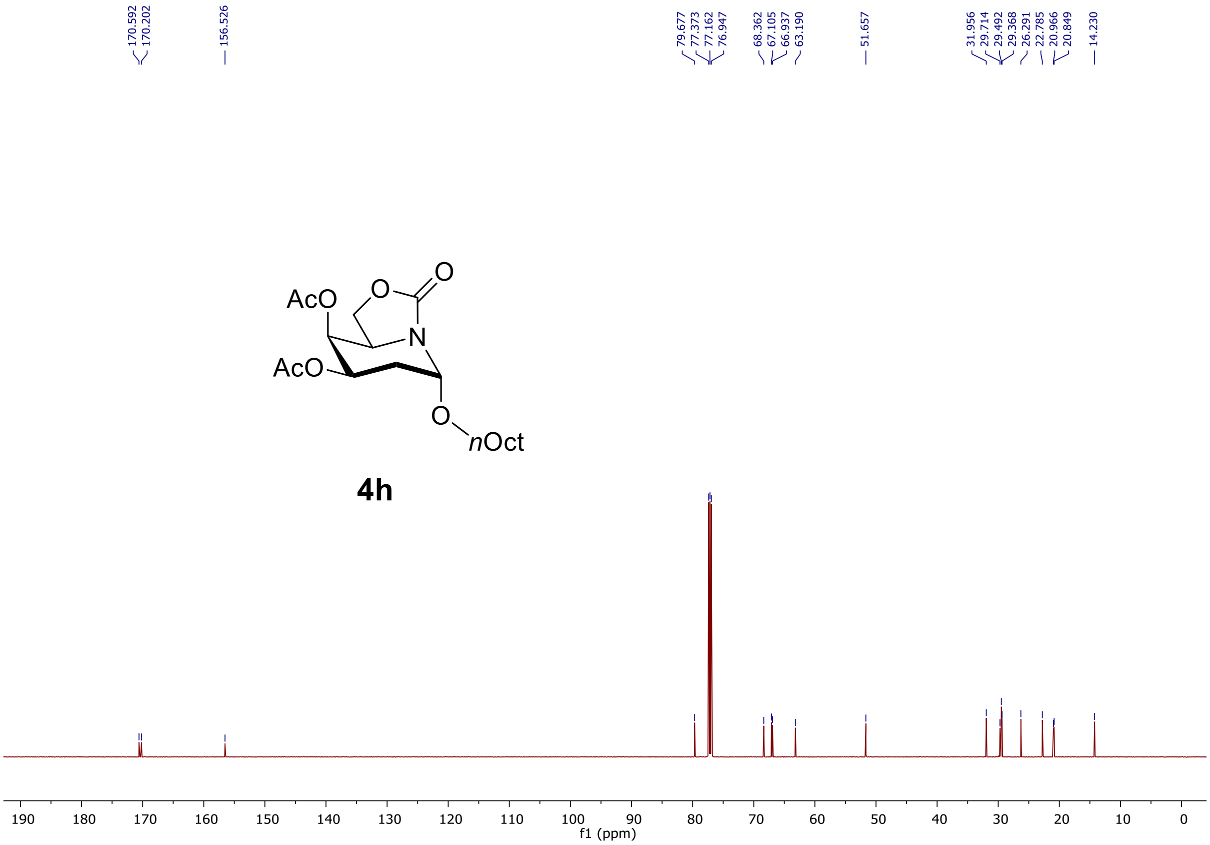

Supplementary Figure S444. ^{13}C NMR spectra for 4h

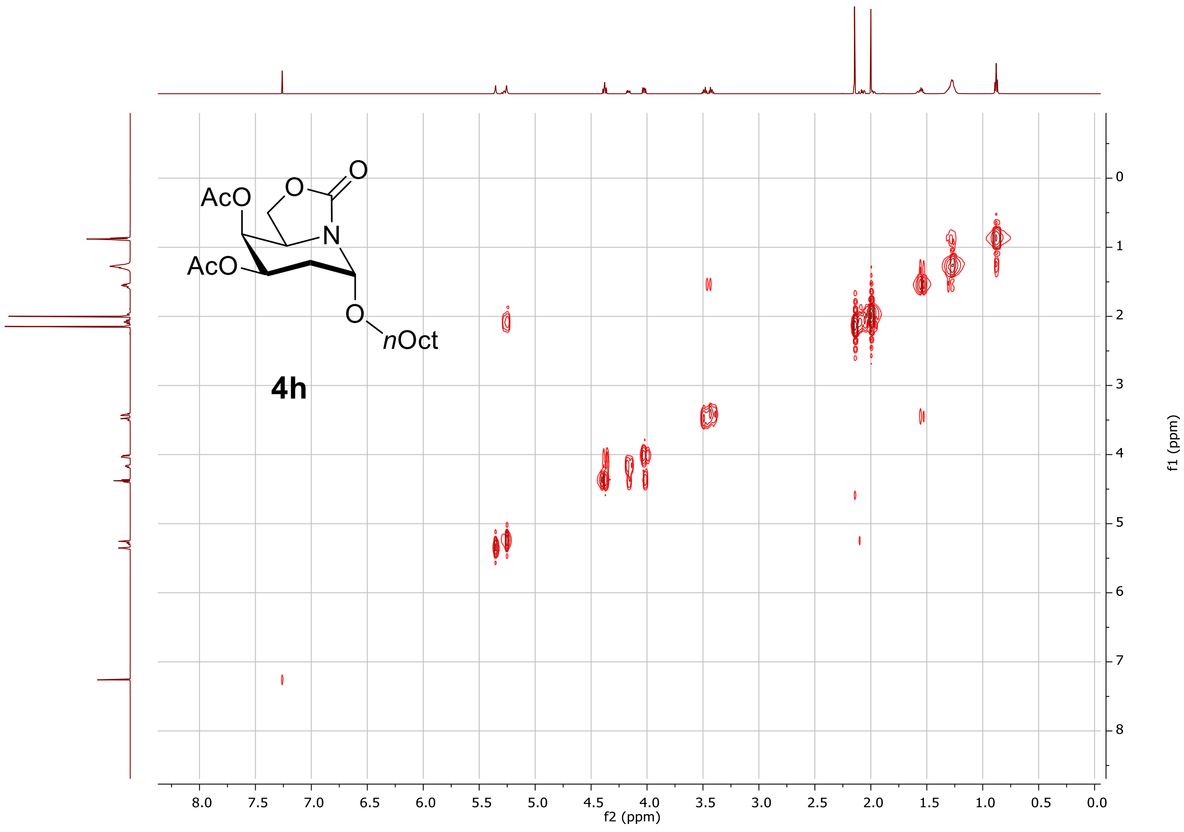

Supplementary Figure S445. COSY spectra for 4h

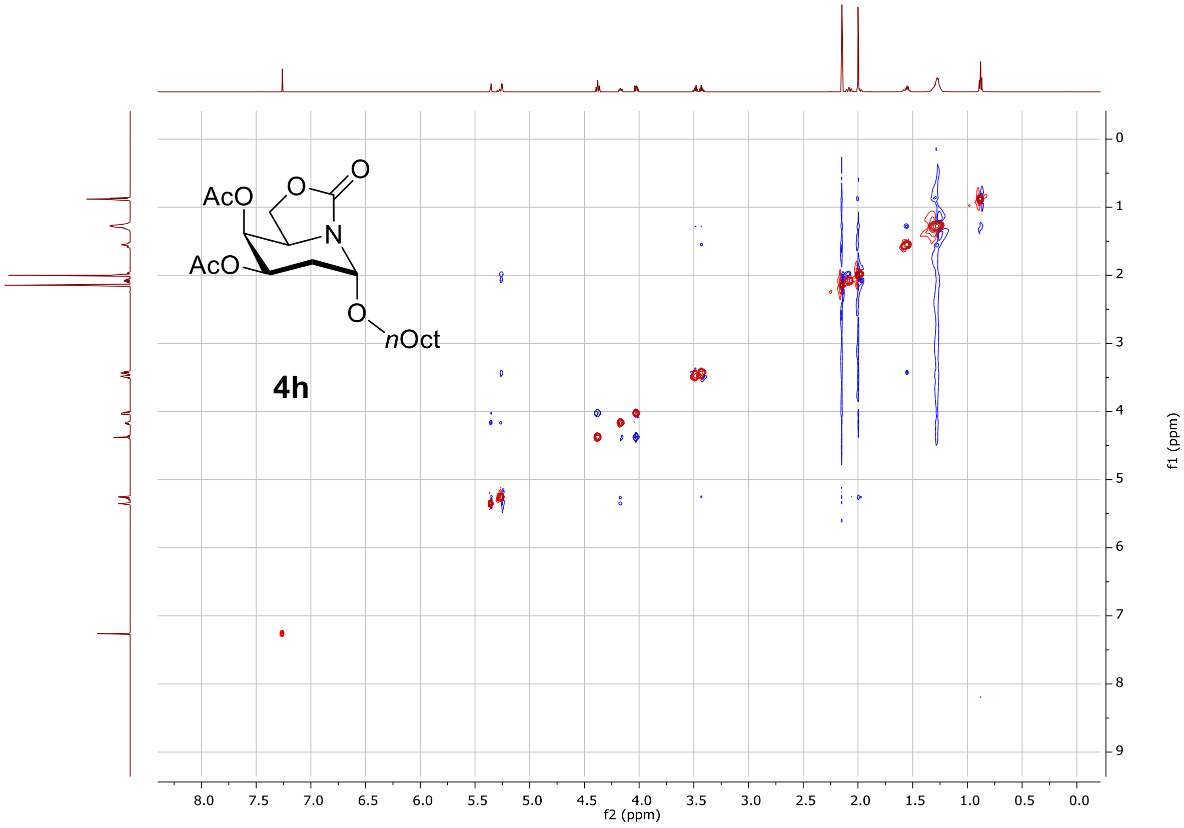

Supplementary Figure S446. NOESY spectra for 4h

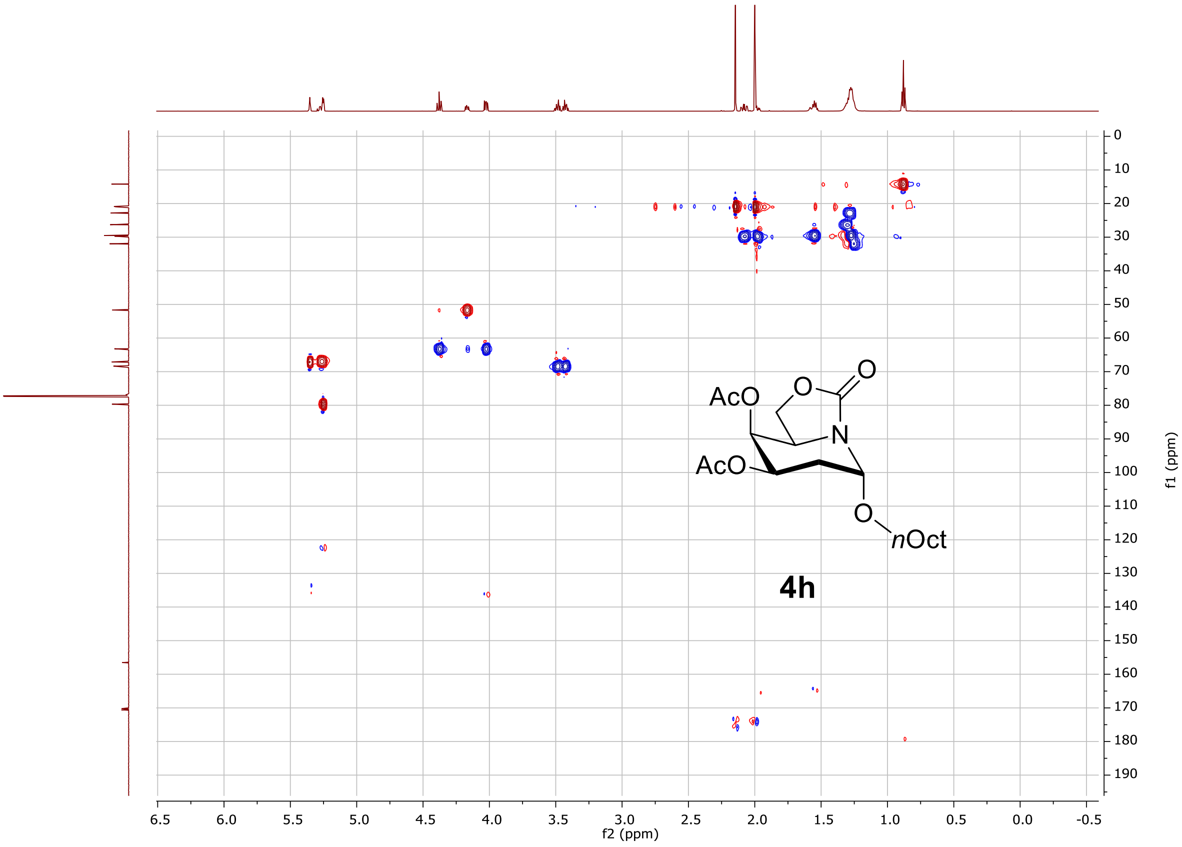

Supplementary Figure S447. HSQC spectra for 4h

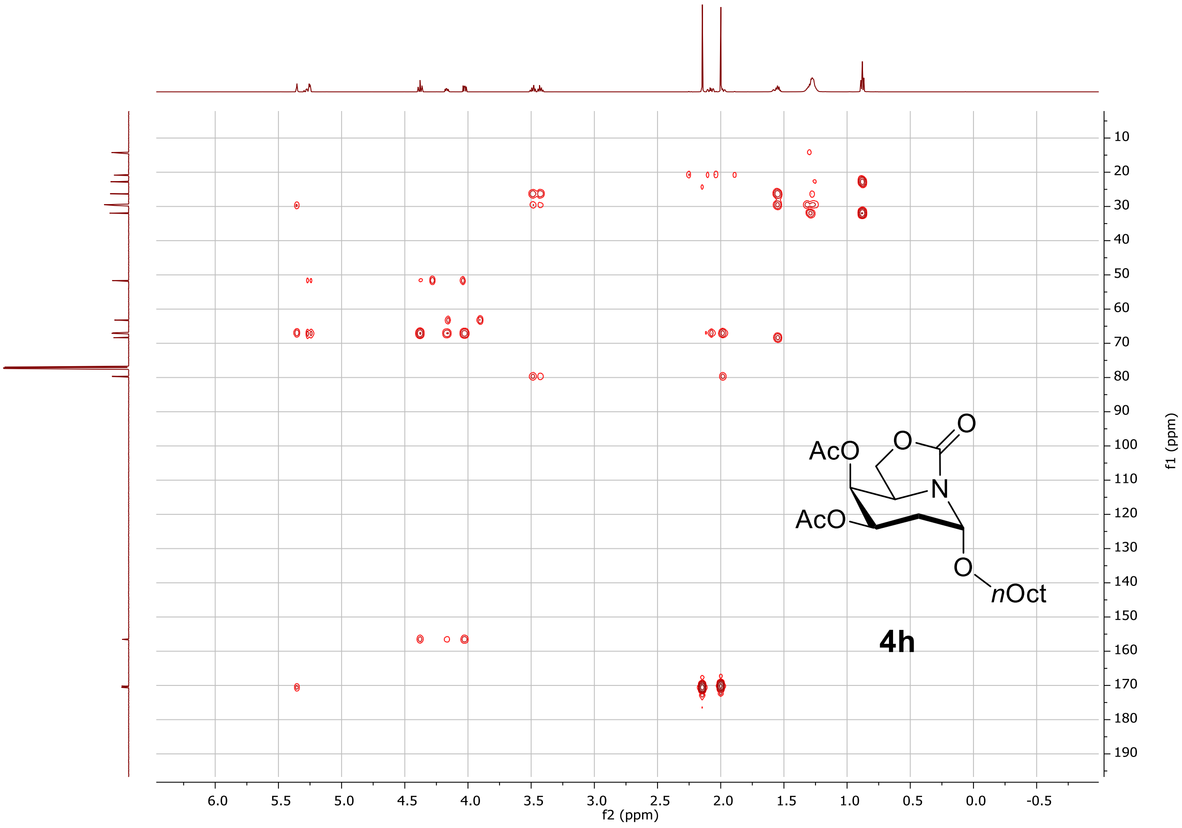

Supplementary Figure S448. HMBC spectra for 4h

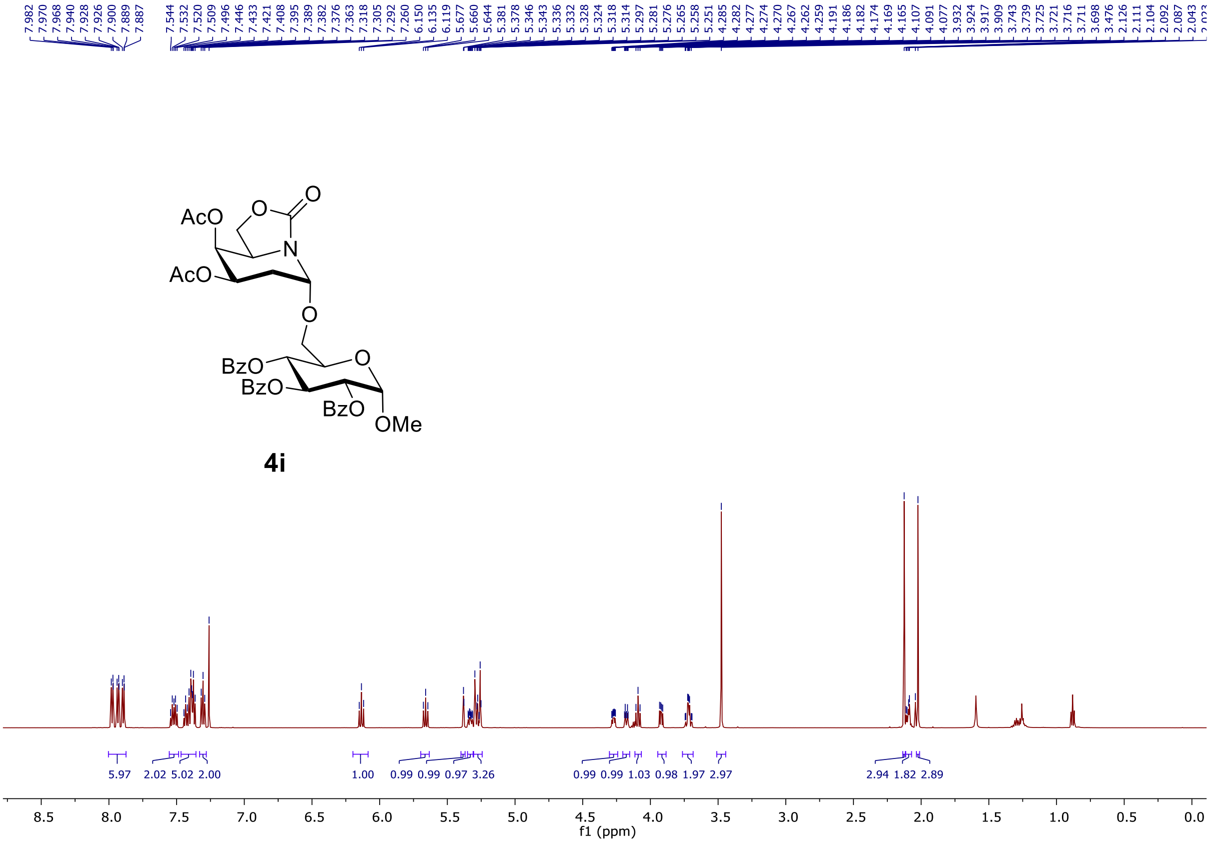

Supplementary Figure S449. ^{1}H NMR spectra for 4i

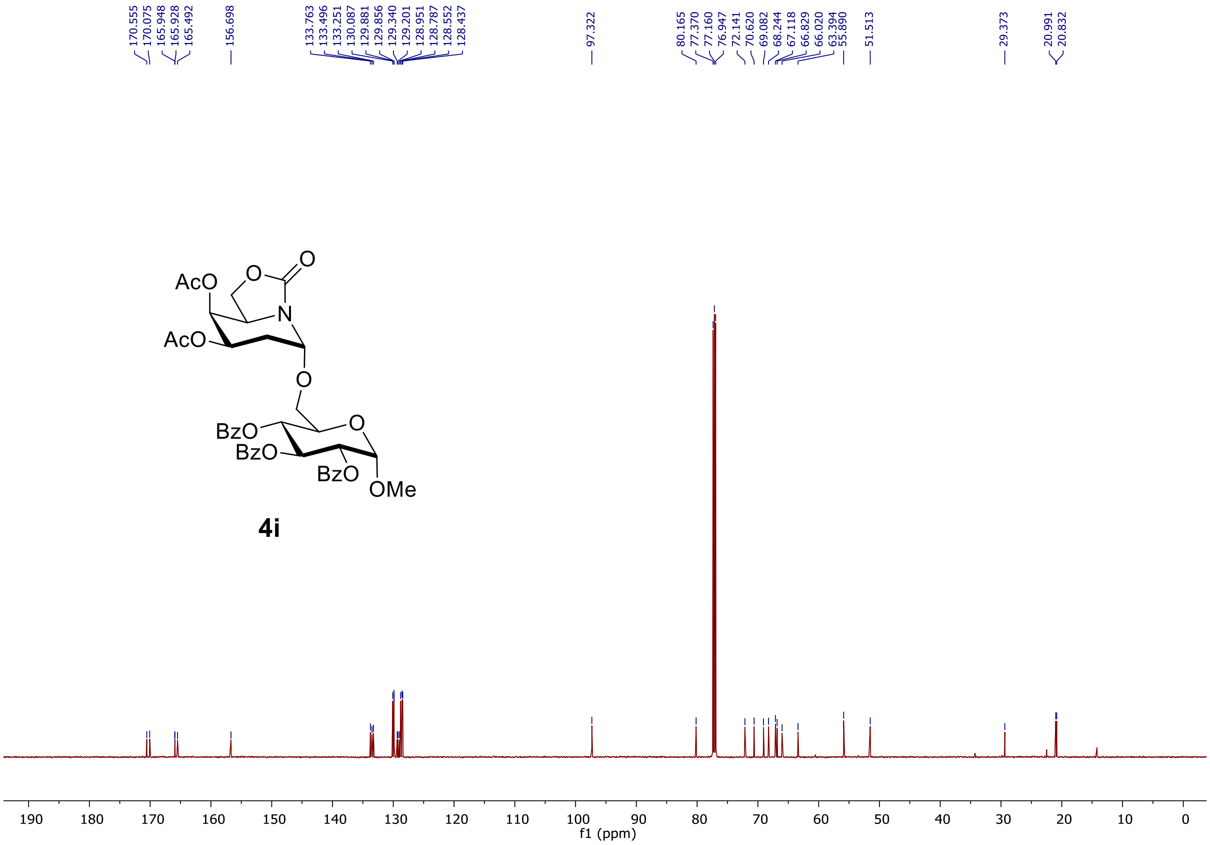

Supplementary Figure S450. ^{13}C NMR spectra for 4i

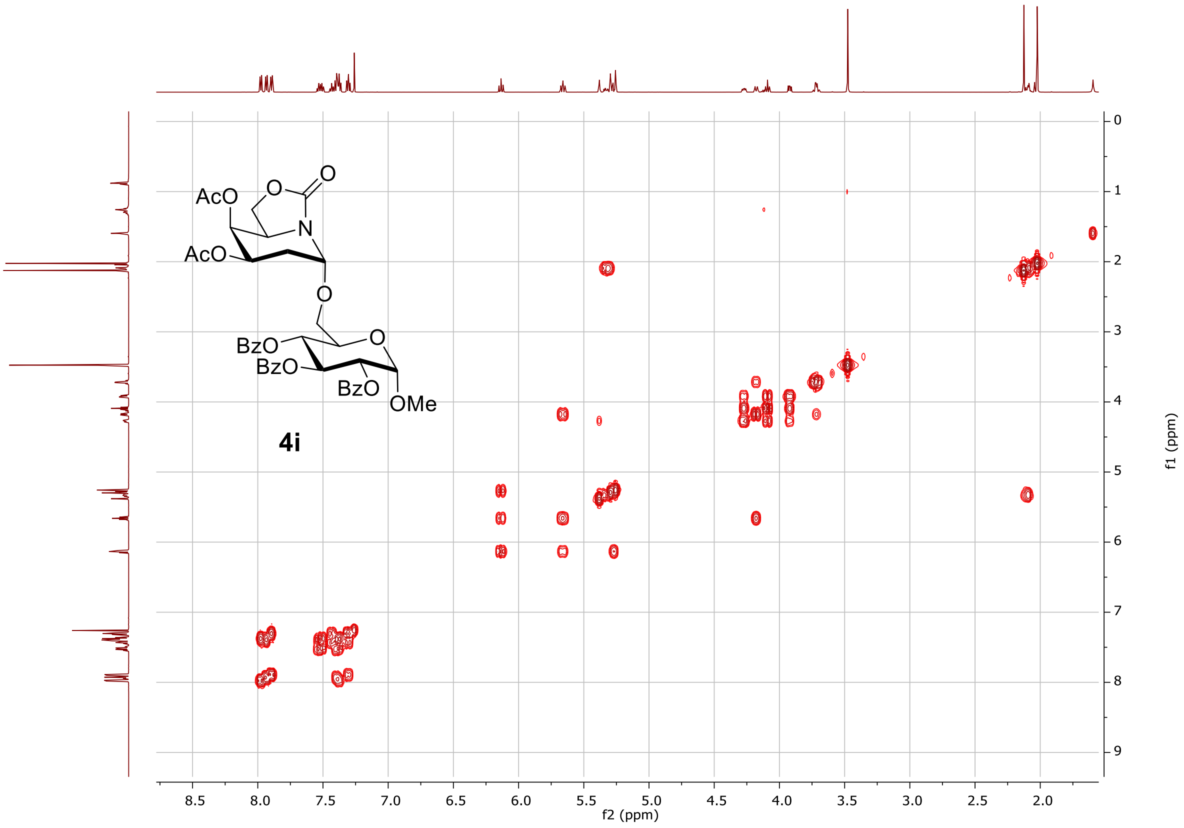

Supplementary Figure S451. COSY spectra for 4i

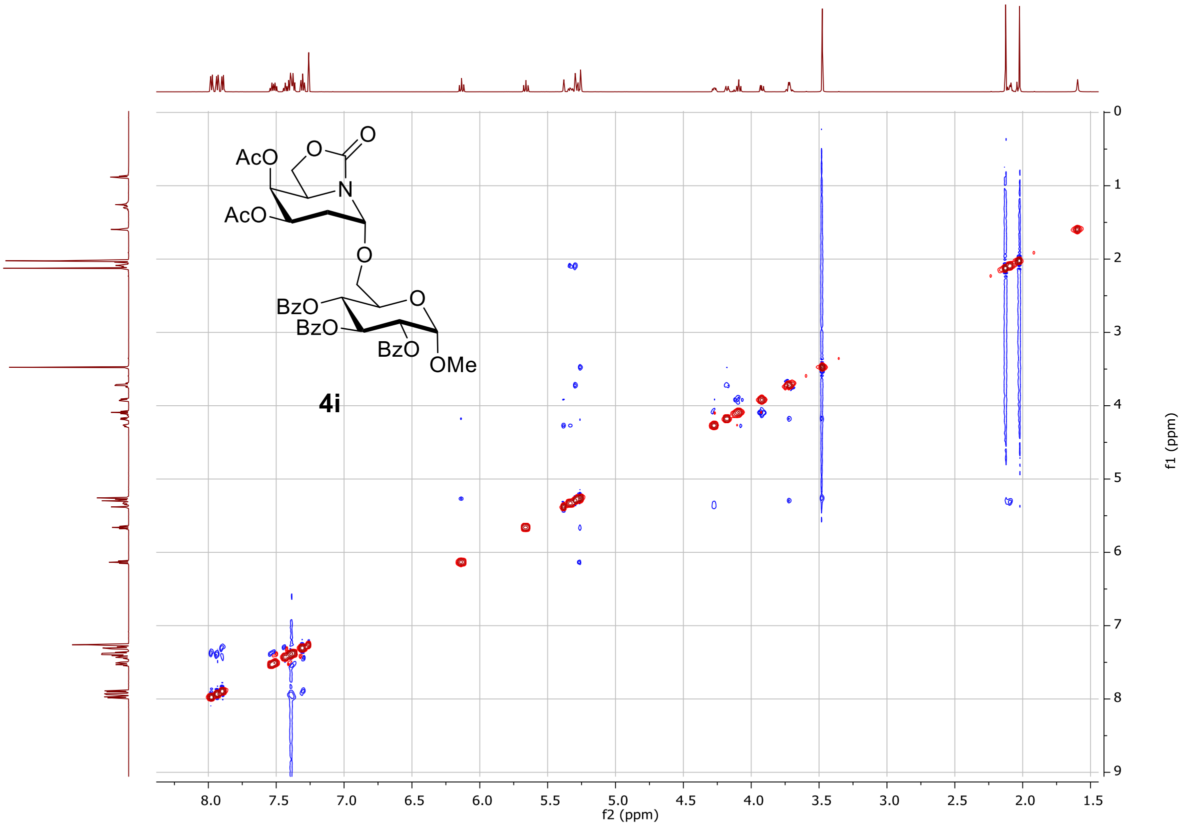

Supplementary Figure S452. NOESY spectra for 4i

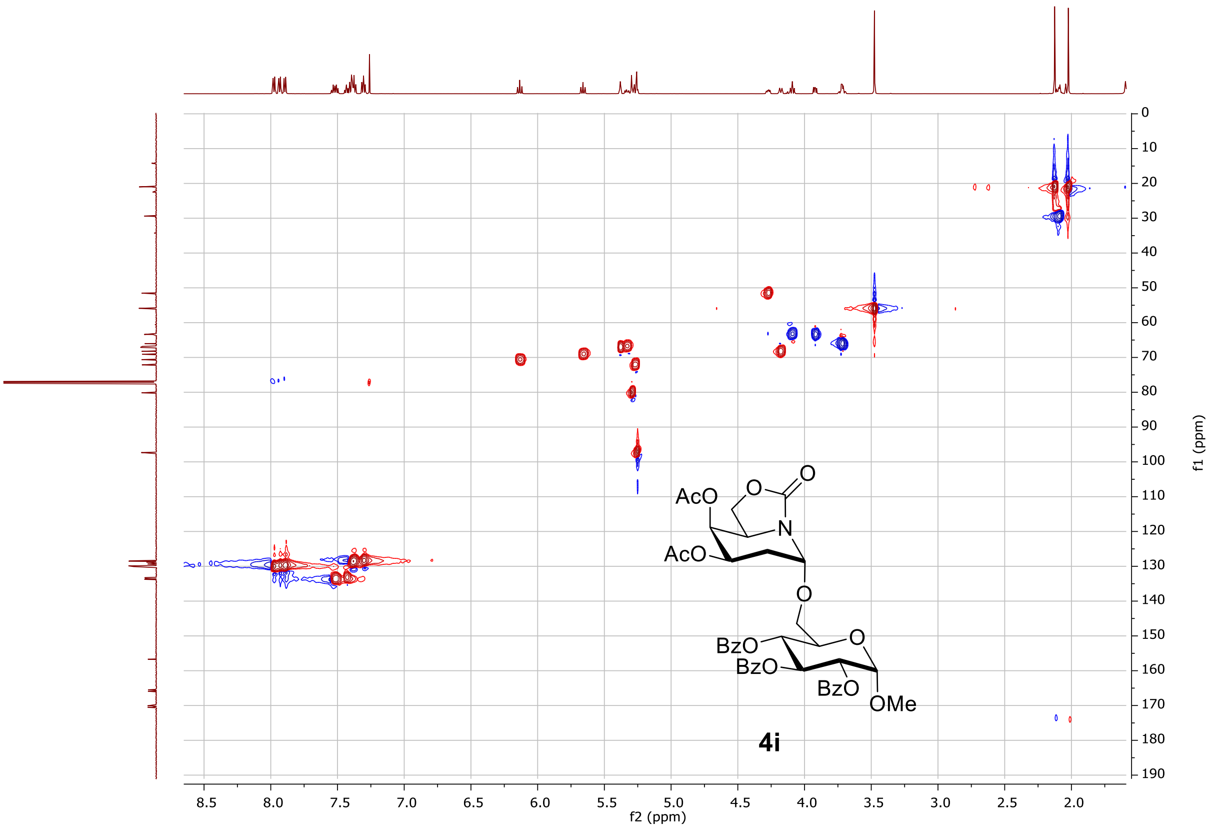

Supplementary Figure S453. HSQC spectra for 4i

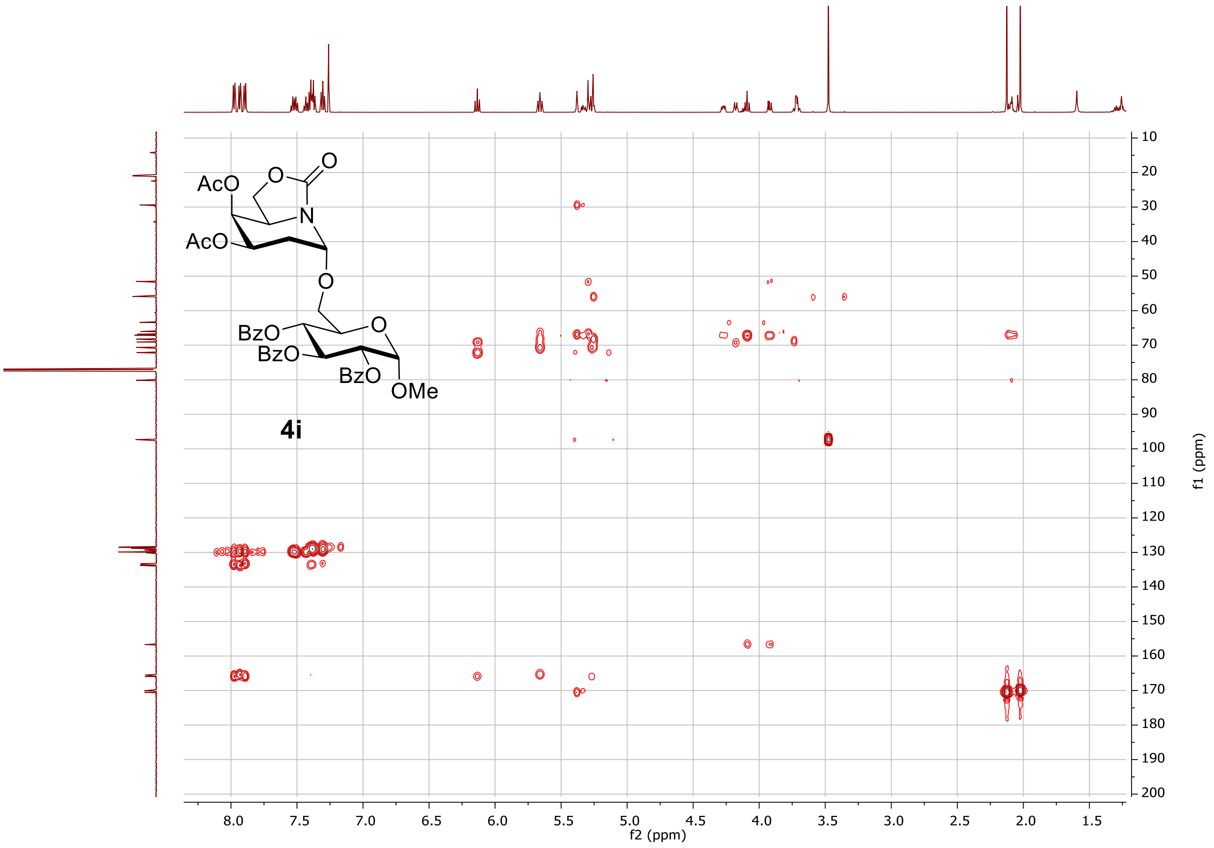

Supplementary Figure S454. HMBC spectra for 4i

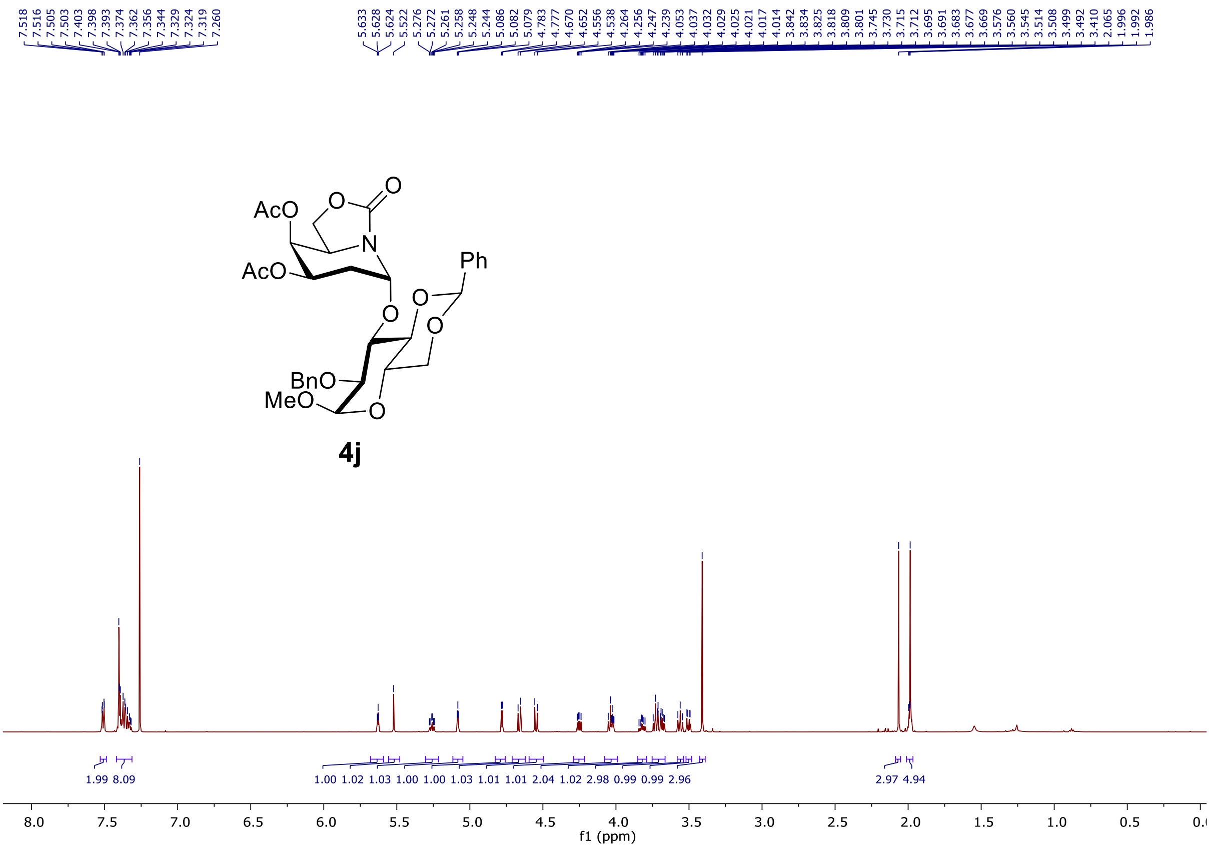

Supplementary Figure S455. ^{1}H NMR spectra for 4j

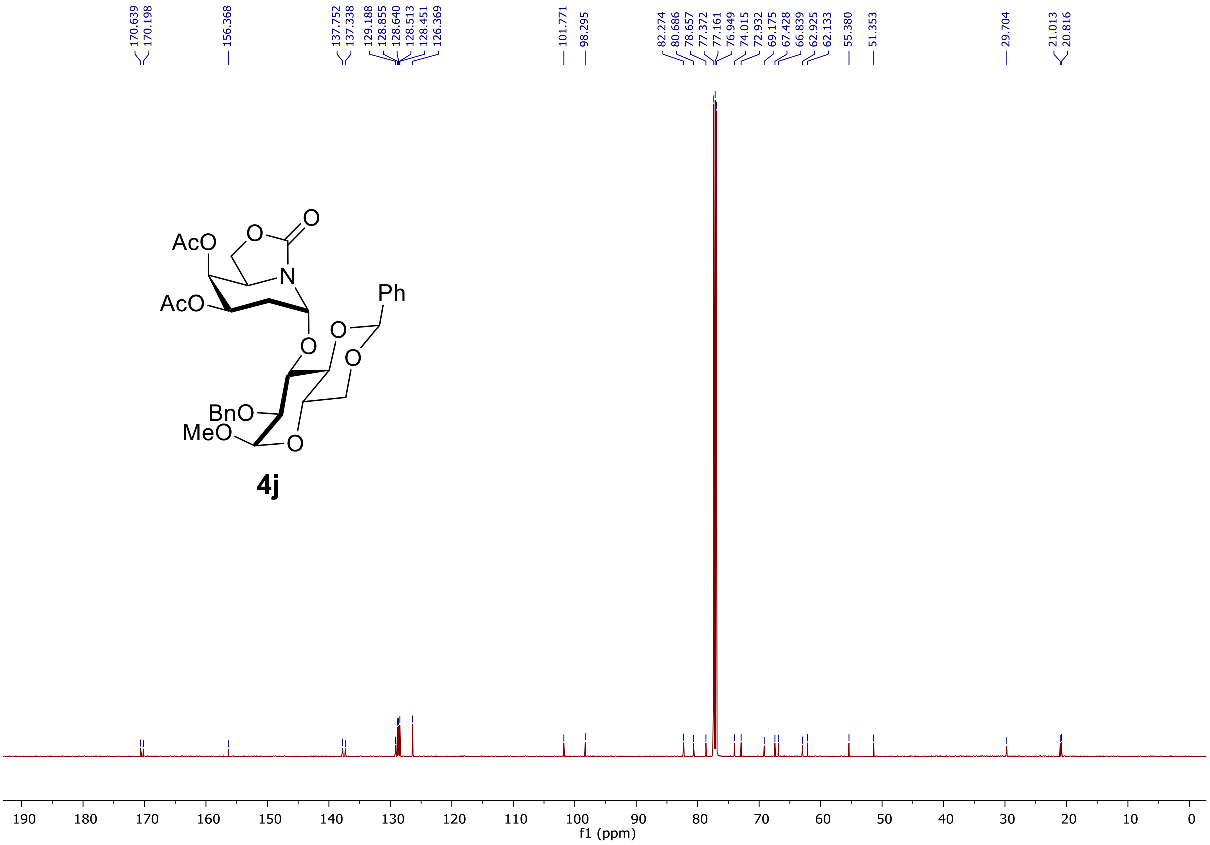

Supplementary Figure S456. ^{13}C NMR spectra for 4j

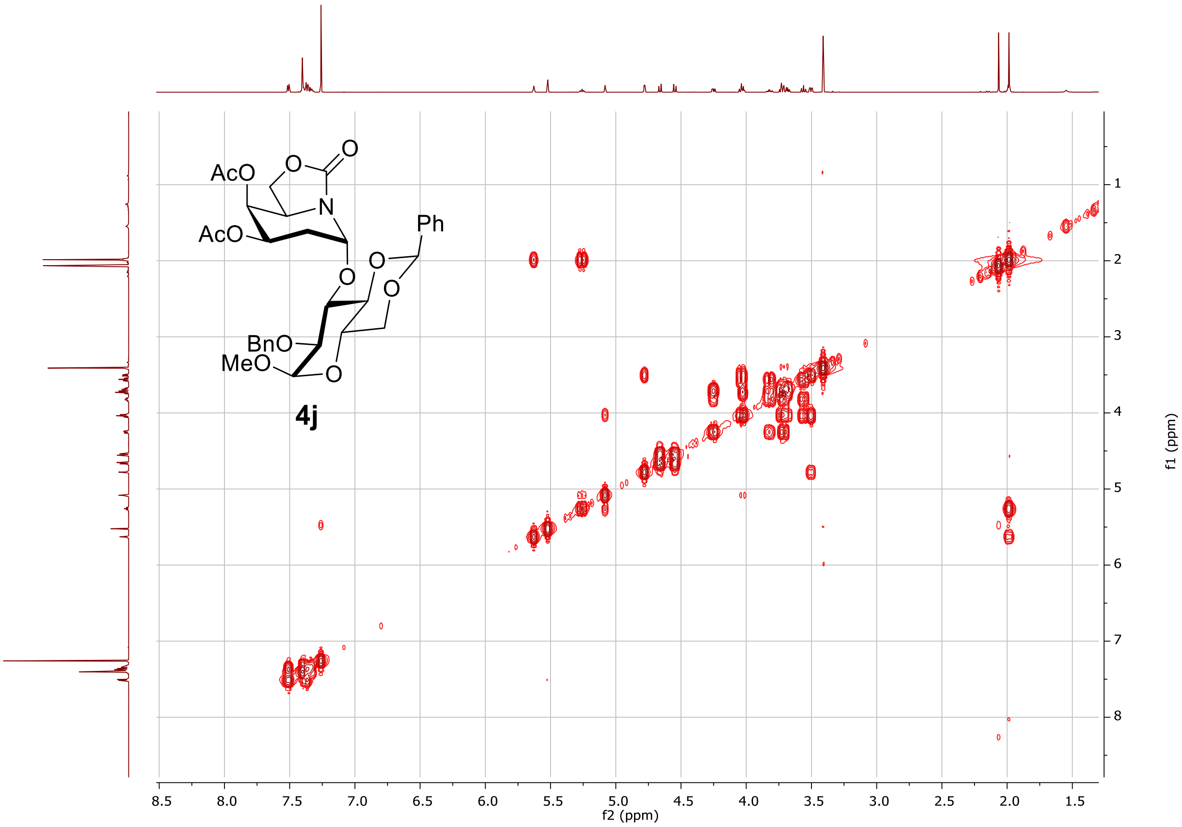

Supplementary Figure S457. COSY spectra for 4j

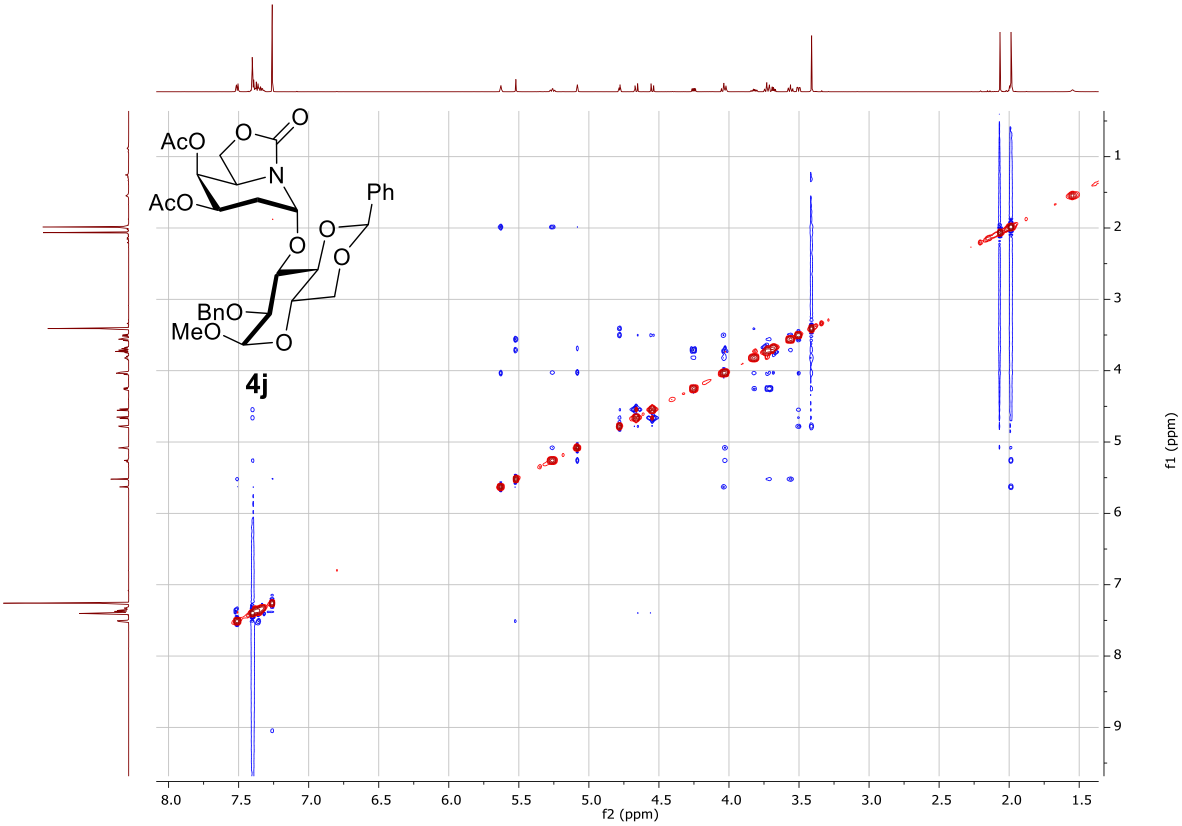

Supplementary Figure S458. NOESY spectra for 4j

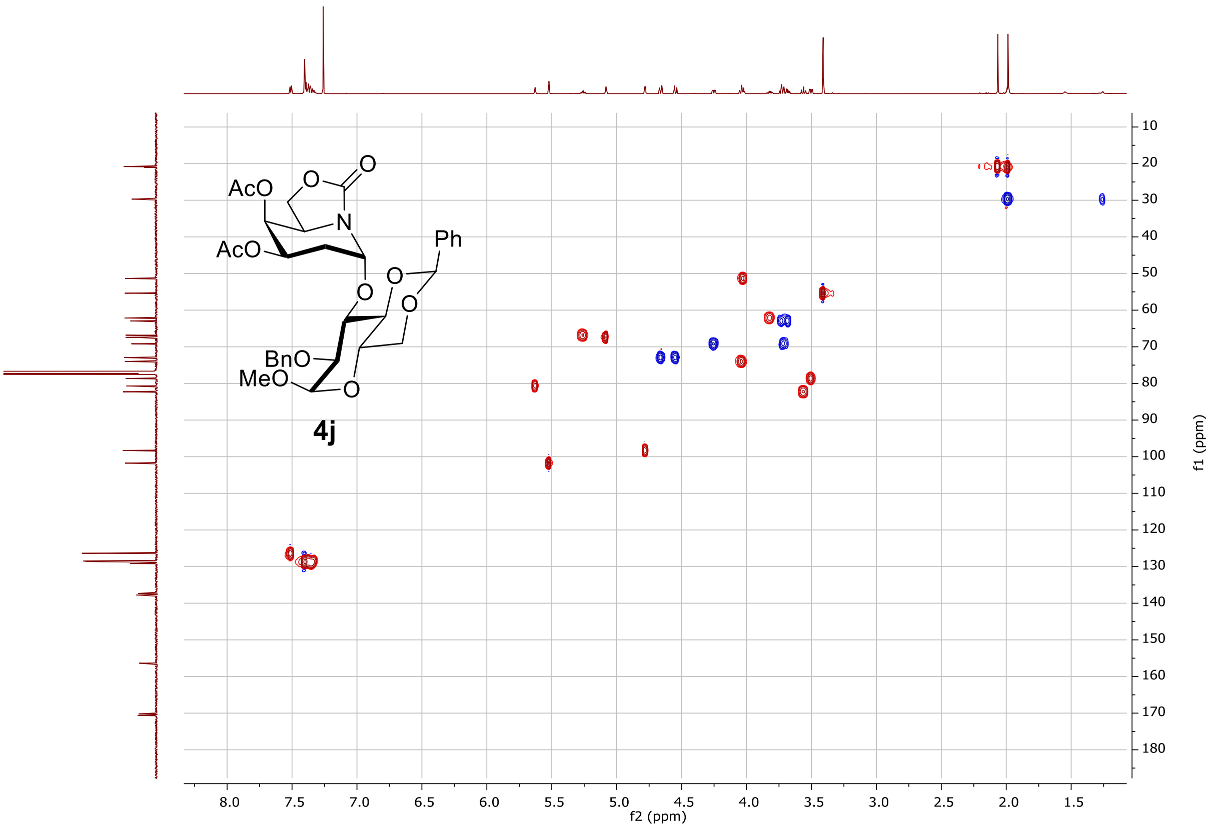

Supplementary Figure S459. HSQC spectra for 4j

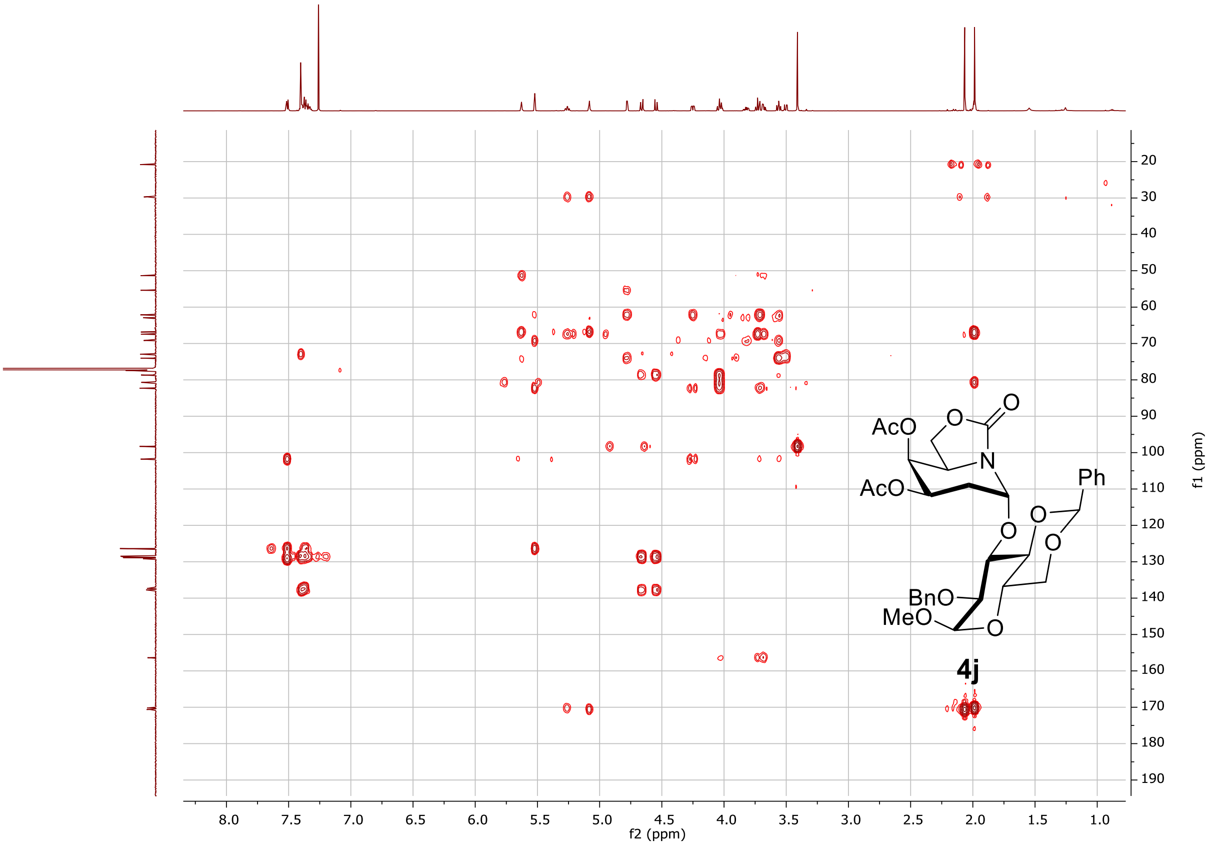

Supplementary Figure S460. HMBC spectra for 4j

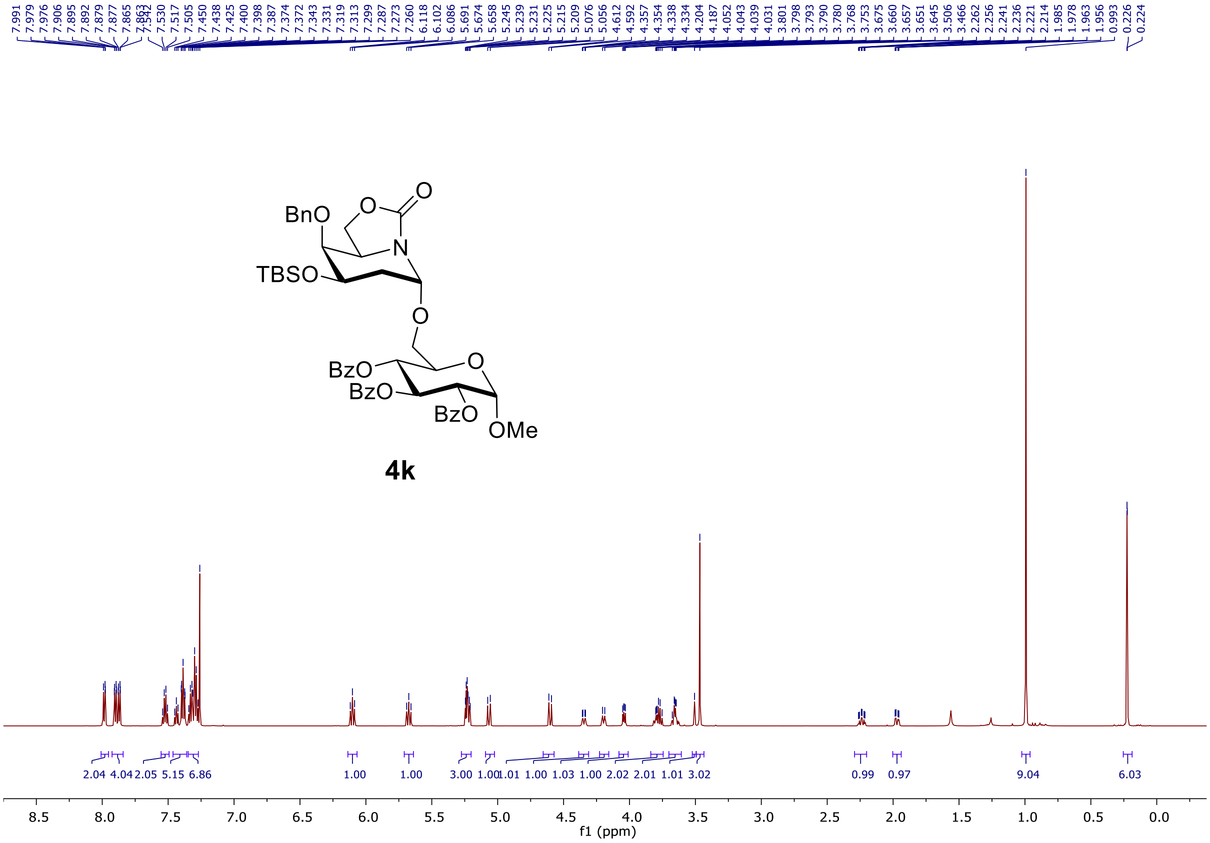

Supplementary Figure S461. ^{1}H NMR spectra for 4k

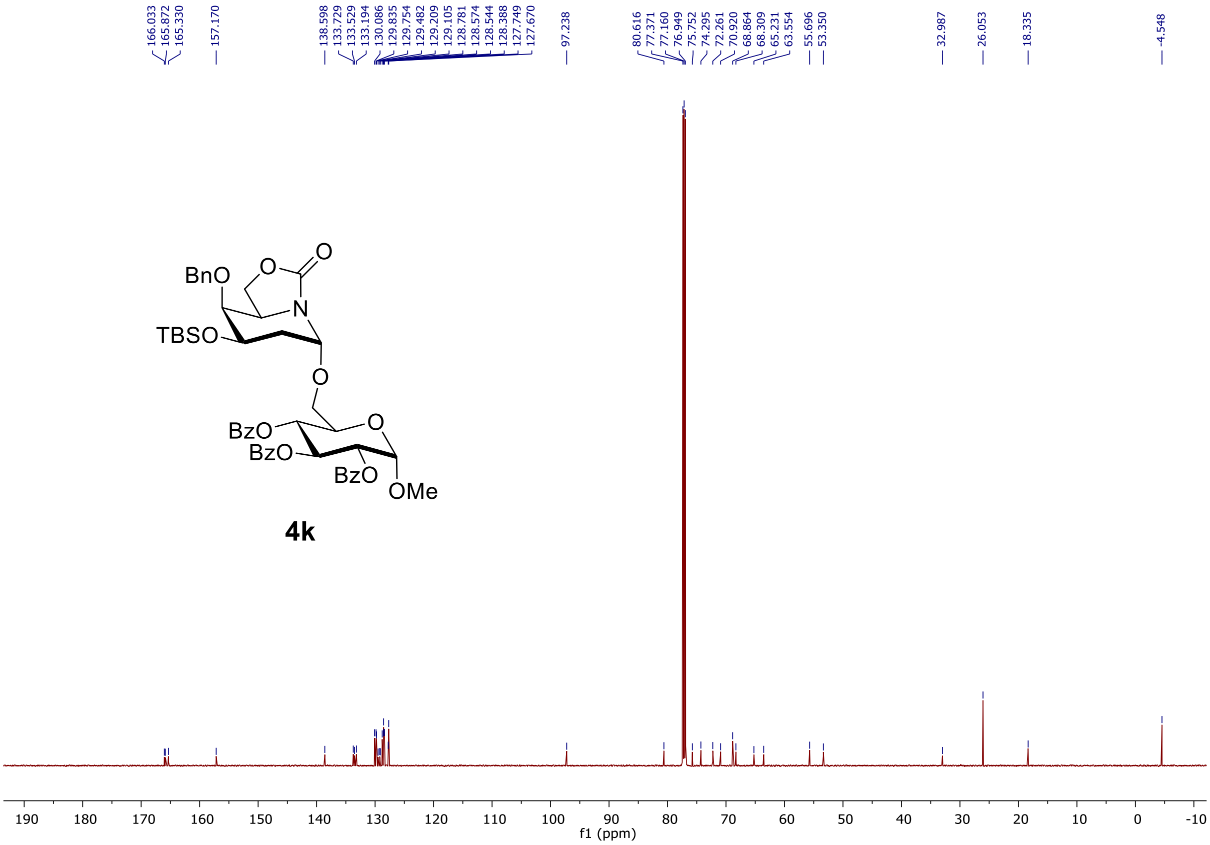

Supplementary Figure S462. ^{13}C NMR spectra for 4k

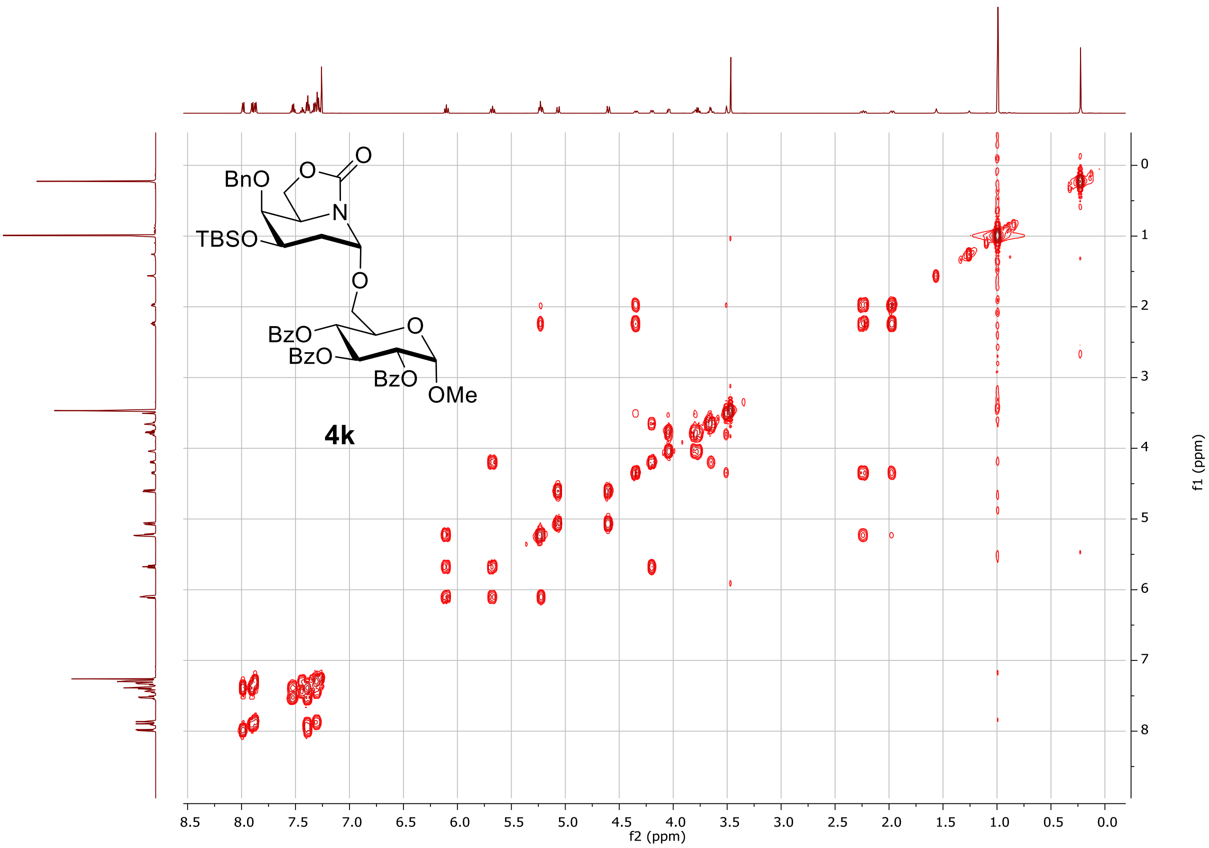

Supplementary Figure S463. COSY spectra for 4k

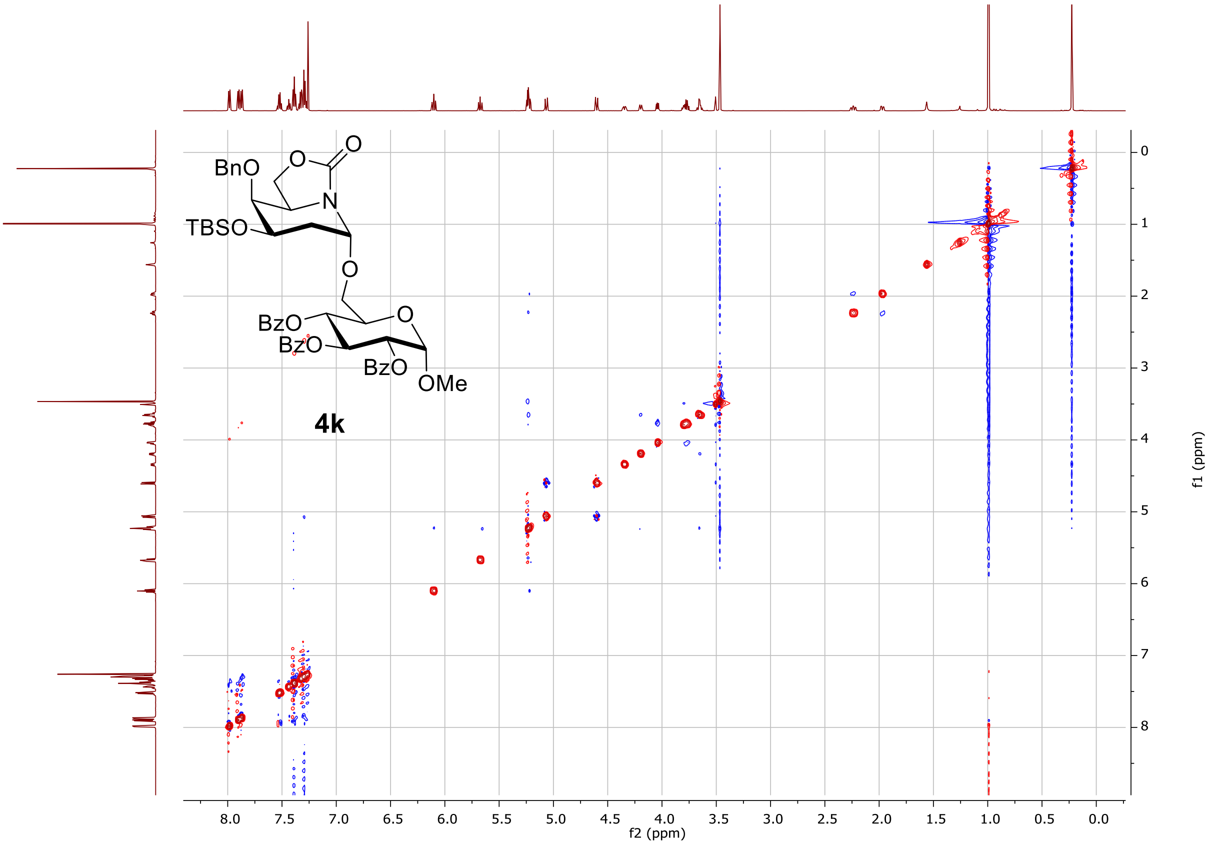

Supplementary Figure S464. NOESY spectra for 4k

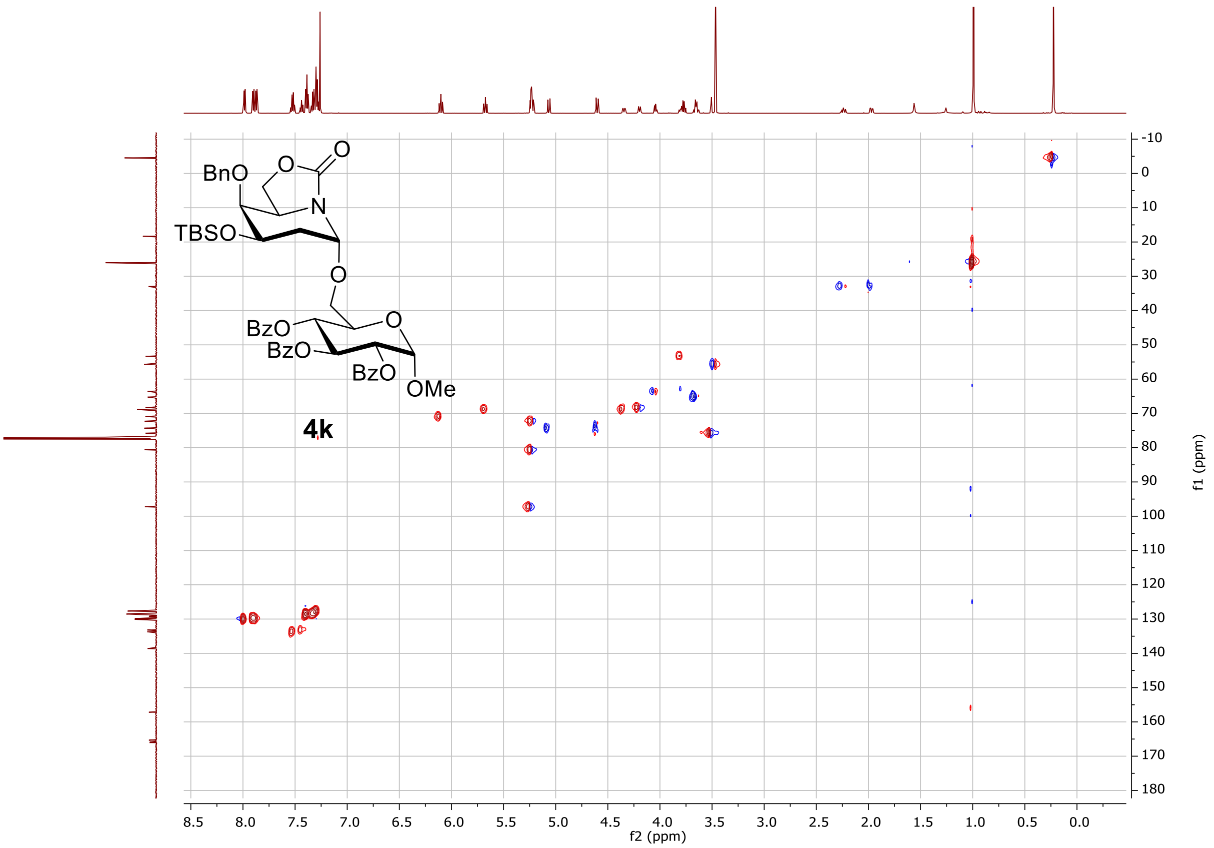

Supplementary Figure S465. HSQC spectra for 4k

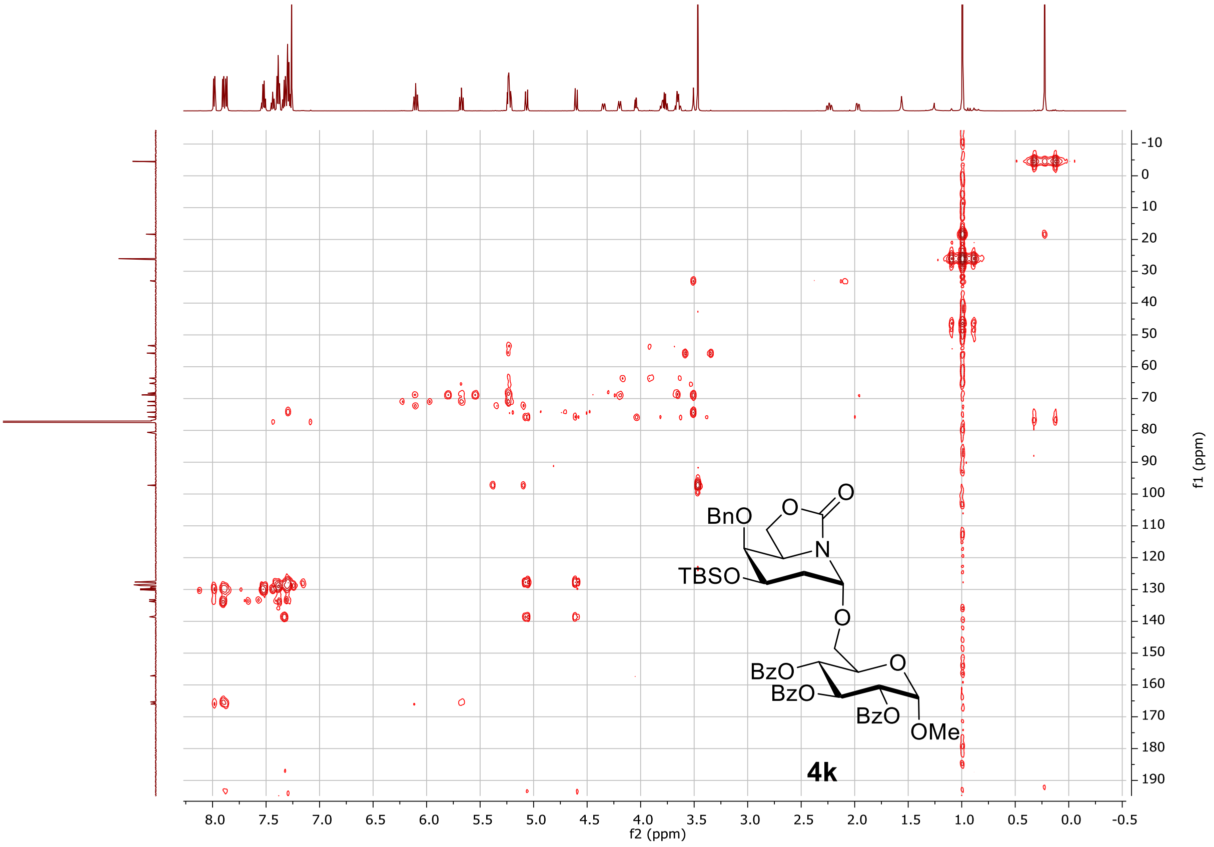

Supplementary Figure S466. HMBC spectra for 4k

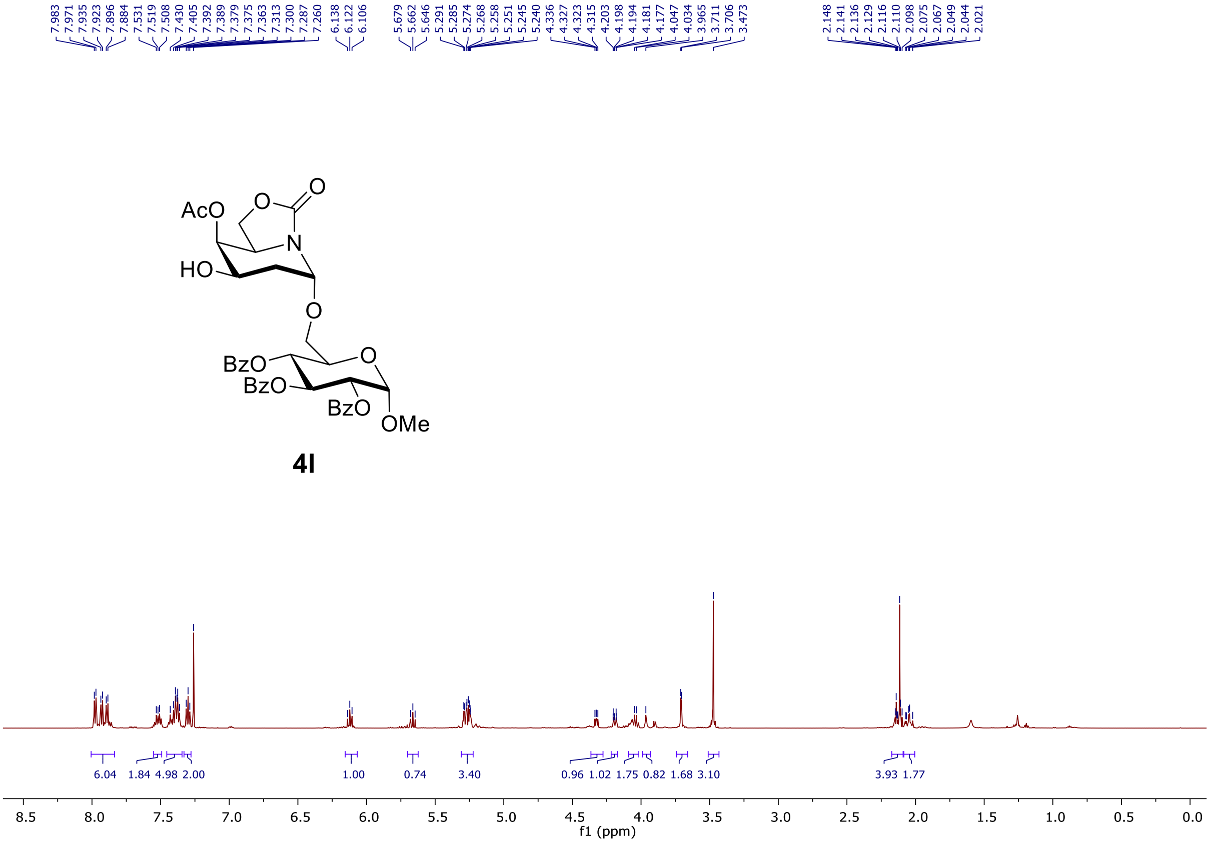

Supplementary Figure S467. ^{1}H NMR spectra for 4l

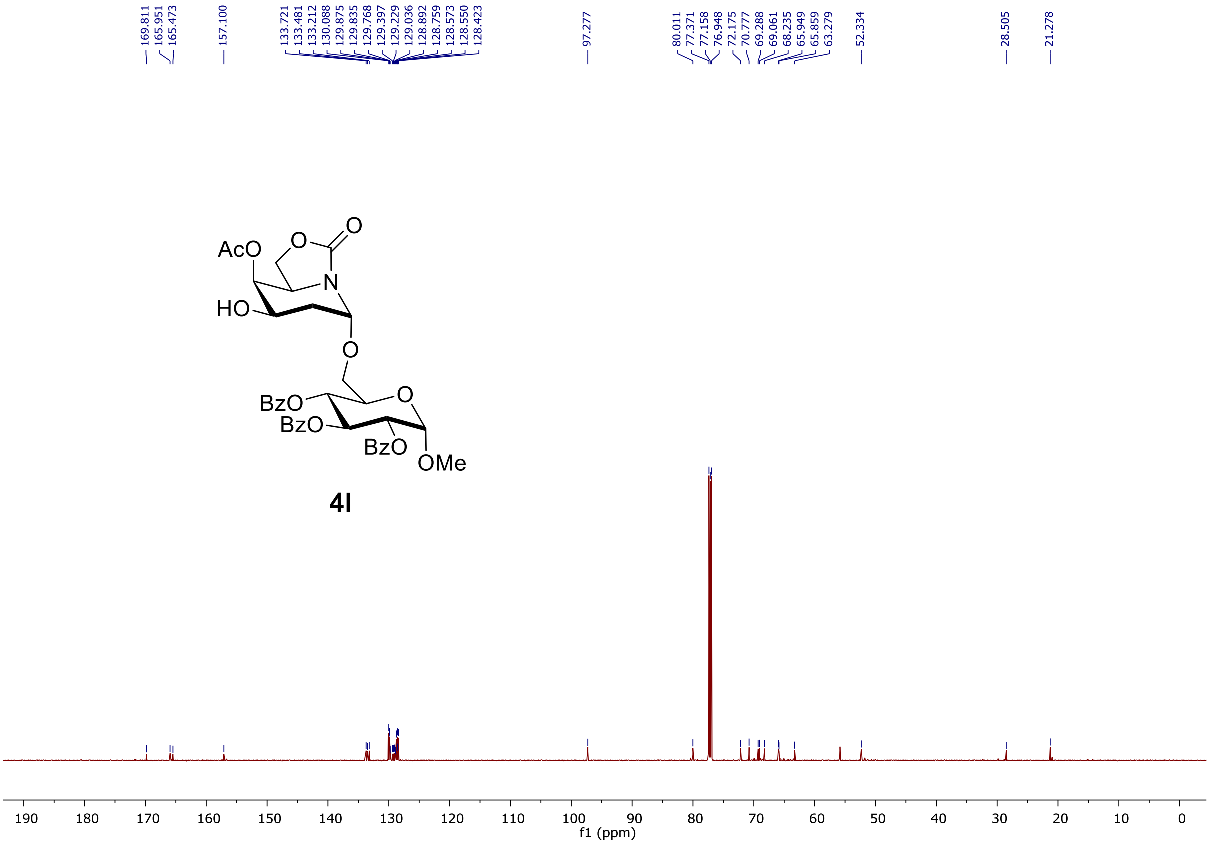

Supplementary Figure S468. ^{13}C NMR spectra for 4l

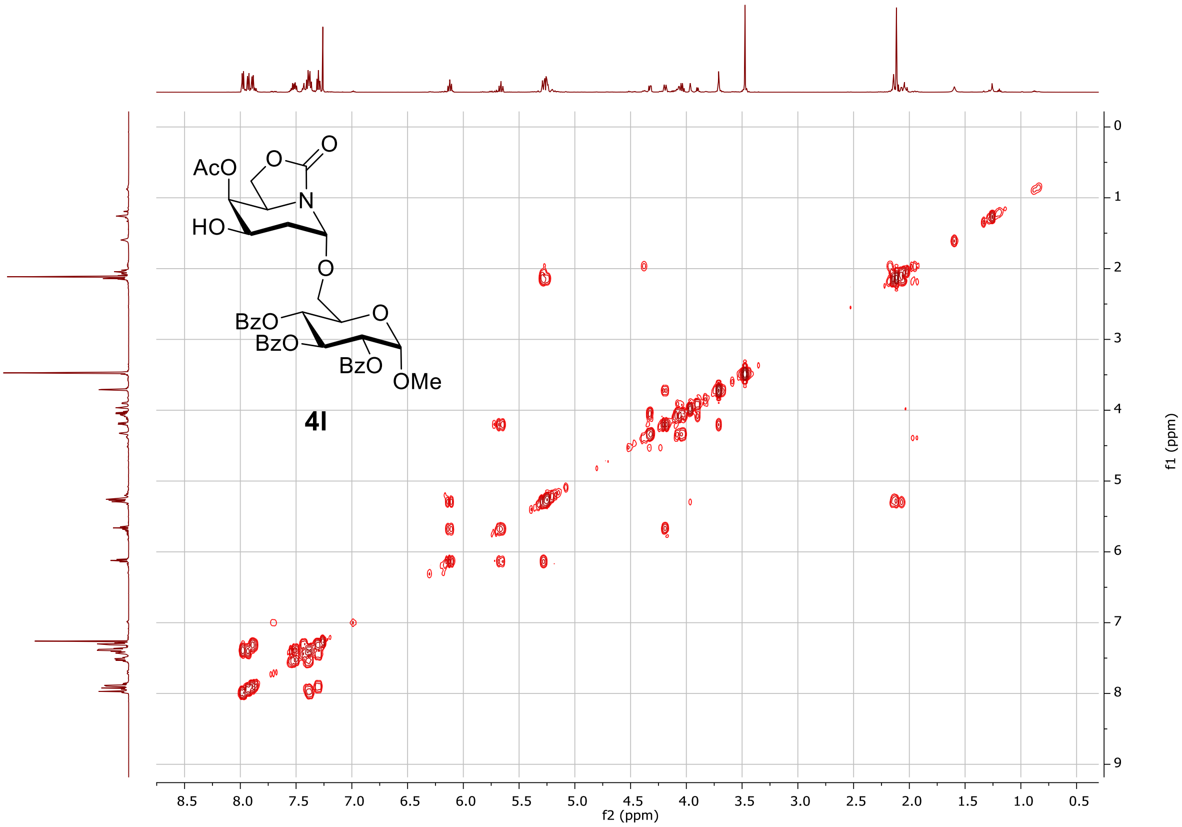

Supplementary Figure S469. COSY spectra for 4l

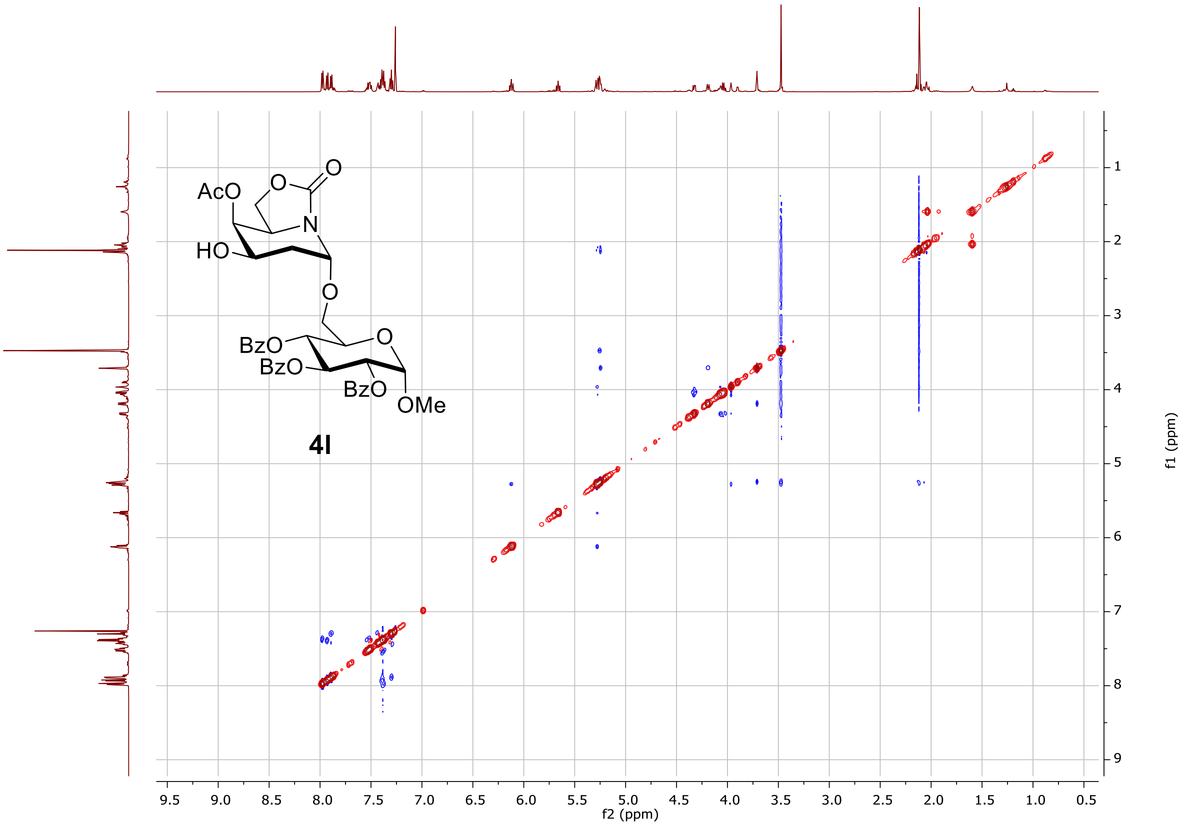

Supplementary Figure S470. NOESY spectra for 4l

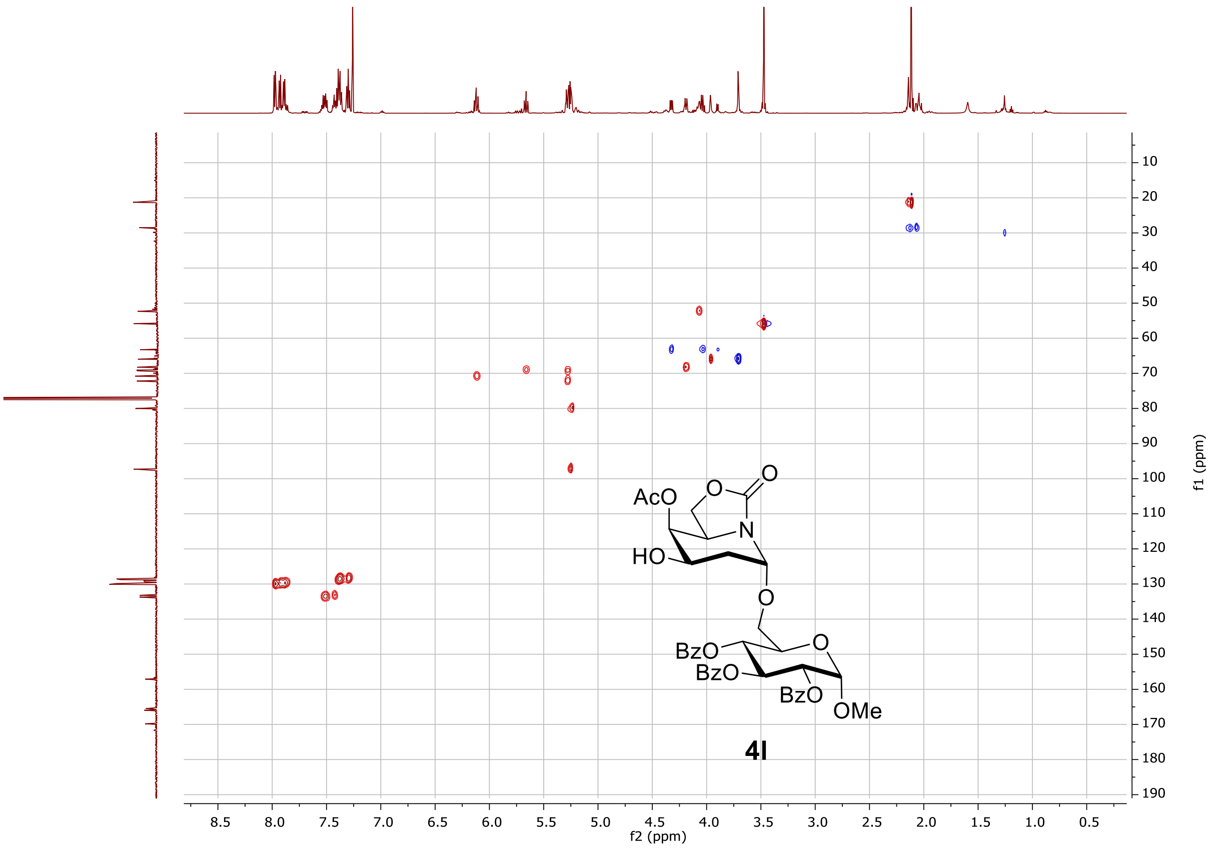

Supplementary Figure S471. HSQC spectra for 4l

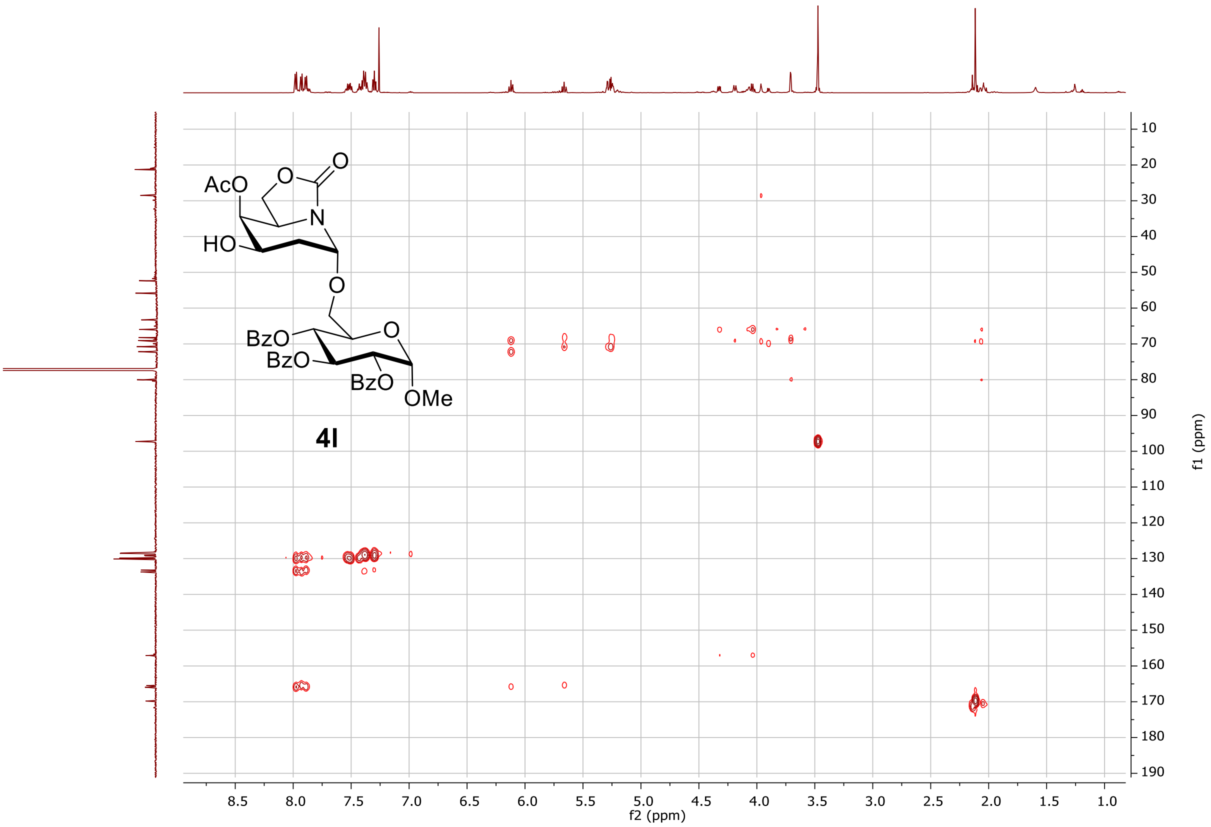

Supplementary Figure S472. HMBC spectra for 4l
